# Dissecting genetic architecture of rare dystonia: genetic, molecular and clinical insights

**DOI:** 10.1136/jmg-2022-109099

**Published:** 2024-03-08

**Authors:** Burcu Atasu, Javier Simón-Sánchez, Hasmet Hanagasi, Basar Bilgic, Ann-Kathrin Hauser, Gamze Guven, Peter Heutink, Thomas Gasser, Ebba Lohmann

**Affiliations:** 1 Eberhard Karls Universität Tübingen Hertie Institut für klinische Hirnforschung Allgemeine Neurologie, Tubingen, Germany; 2 Department of Neurology, Istanbul University Istanbul Faculty of Medicine, Istanbul, Turkey; 3 Genetics Department, Aziz Sancar Institute of Experimental Medicine, Istanbul, Turkey; 4 DZNE Tübingen, Tübingen, Germany

**Keywords:** nervous system diseases, genetics, medical, gene expression profiling, Gene Ontology, neurodegenerative diseases

## Abstract

**Background:**

Dystonia is one of the most common movement disorders. To date, the genetic causes of dystonia in populations of European descent have been extensively studied. However, other populations, particularly those from the Middle East, have not been adequately studied. The purpose of this study is to discover the genetic basis of dystonia in a clinically and genetically well-characterised dystonia cohort from Turkey, which harbours poorly studied populations.

**Methods:**

Exome sequencing analysis was performed in 42 Turkish dystonia families. Using co-expression network (CEN) analysis, identified candidate genes were interrogated for the networks including known dystonia-associated genes and genes further associated with the protein-protein interaction, animal model-based characteristics and clinical findings.

**Results:**

We identified potentially disease-causing variants in the established dystonia genes (*PRKRA, SGCE, KMT2B, SLC2A1, GCH1, THAP1, HPCA, TSPOAP1, AOPEP*; n=11 families (26%)), in the uncommon forms of dystonia-associated genes (*PCCB, CACNA1A, ALDH5A1, PRKN*; n=4 families (10%)) and in the candidate genes prioritised based on the pathogenicity of the variants and CEN-based analyses (n=11 families (21%)). The diagnostic yield was found to be 36%. Several pathways and gene ontologies implicated in immune system, transcription, metabolic pathways, endosomal-lysosomal and neurodevelopmental mechanisms were over-represented in our CEN analysis.

**Conclusions:**

Here, using a structured approach, we have characterised a clinically and genetically well-defined dystonia cohort from Turkey, where dystonia has not been widely studied, and provided an uncovered genetic basis, which will facilitate diagnostic dystonia research.

**WHAT IS ALREADY KNOWN ON THIS TOPIC**

- The genetic causes of dystonia have been extensively studied in European populations and different diagnostic yields ranging from 11.7% to 39.1% have been reported.

**WHAT THIS STUDY ADDS**

- This is the first study that covers dystonia families from Turkey, the population of which is known to be a mixture of populations from the Middle East, the Caucasus, the Balkans, Central Asia and Europe.
- By identifying the genetic causes of dystonia in a well-defined cohort of 42 families, we provide a distinct overview of the genetic makeup of dystonia in Turkey, and identify candidate genes and related mechanisms.

**HOW THIS STUDY MIGHT AFFECT RESEARCH, PRACTICE OR POLICY**

- This study will (1) aid in clinical-diagnostic settings in Turkey and some other related populations, such as Balkan, Caucasus and Middle Eastern populations and (2) contribute to dystonia research in terms of identifying candidate disease mechanisms.

## Introduction

Dystonia is one of the most common movement disorders and is characterised by sustained or intermittent muscle contractions that cause abnormal and often repetitive movements, postures or both.^1^

Hereditary dystonias are a clinically and genetically heterogeneous group of disorders. To date, based on the nomenclature of Genetic Movement Disorders^2^ and the recent publications,^3 4^ ~60 inherited forms of dystonia have been reported. A small proportion of those belong to an isolated dystonia group (40%) that has been associated with the autosomal recessive (AR) form of dystonia, suggesting the scarcity, genetic complexity and heterogeneity of the disease as well as the limitations of existing approaches, which have focused on specific populations.

So far, dystonia has been widely studied in European populations,^5–8^ and it has been scarcely investigated in Middle Eastern, Asian^9 10^ and African populations. Turkey lies at the crossroads between Europe and Asia. Due to Turkey’s geographical location, its population is a genetic mix of populations from the Balkans, Caucasus, Middle East, Central Asia and Europe.^11^ Studies on Turkish cohorts are also of great importance in dystonia research in view of the high degree of consanguinity observed in Turkey.^11^

In this study, our major purpose was to dissect the complex architecture of dystonia through the identification of rare genetic causes in a well-characterised study cohort from Turkey and to provide an uncovered genetic background of dystonia to aid in clinical-diagnostic settings. For this purpose, we have conducted a comprehensive study using a wide-scale genetic analysis approach. Through identifying known and new causes of the disease, we have shown that the genetic background of dystonia is overly complex and needs to be investigated systematically in different populations.

## Materials and methods

### Patient’s characteristics and clinical approach

A total of 42 dystonia families, which are compatible with the AR mode of inheritance, including 46 affected and 47 non-affected individuals, were recruited in Turkey for the study at the Movement Disorders Unit, Department of Neurology, Istanbul University, between 2012 and 2015. Fifteen of those families were included in this study as trios and three as quartets. The families were included in the study based on the consensus definition of dystonia.^1^ All the affected members and available unaffected family members underwent a detailed clinical examination. Radiological and laboratory findings were obtained from all the recruited individuals when needed. Consanguinity was reported in 16 families (38%). The mean age at onset of the affected members was 21.3±18 years (range 1–68 years).

In terms of clinical characteristics, the majority of our cohort were categorised into childhood onset (n=18; 42.8%), generalised (n=21; 49.9%) and isolated (n=28; 66.6%) dystonia based on the age at onset, body distribution and other associated features. The clinical characteristics are summarised in table 1, the details of which are provided in online supplemental materials S1–S4.

10.1136/jmg-2022-109099.supp1 Supplementary data

**Table 1:** Summary of the clinical characteristics of the dystonia cohort

	Families with variants in the dystonia-associated genes (n=15)	Total families (n=42)
Clinical characteristics		
Age at onset		
Infancy (birth to 2 years)	3 (7.1 %)	5 (11.9%)
Childhood (3–12 years)	7 (16.7%)	18 (42.8%)
Adolescence (13–20 years)	0	4 (9.5%)
Early adulthood (21–40 years)	2 (4.8%)	9 (21.5%)
Late adulthood (>40 years)	3 (7.1%)	6 (14.3%)
Body distribution		
Focal	1 (2.4%)	2 (4.8%)
Segmental	7 (16.7%)	18 (42.9%)
Multifocal	0	1 (2.4%)
Generalised	7 (16.7%)	21 (49.9%)
Associated features		
Isolated dystonia	8 (19%)	28 (66.6%)
Combined dystonia	7 (16.7%)	14 (33.4%)
Additional clinical characteristics		
Ataxia	2 (4.7%)	3 (7%)
Choreoathetosis	2 (4.7%)	4 (9.4%)
Developmental delay	1 (2.3%)	2 (4.6%)
Rigidity	2 (4.7%)	3 (7%)
Epilepsy and seizures	2 (4.7%)	3 (7%)
Bradykinesia	1 (2.3%)	3 (7%)
Cognitive impairment	2 (4.7%)	2 (4.7%)
MRI findings		
T2 hyperintensity in BG	1 (2.3%)	2 (4.7%)
Cerebellar atrophy	0	1 (2.3%)

BG, basal ganglia.

### Genetic analyses

A systematic genetic screening strategy was conducted to analyse 93 exome sequencing (ES) samples from 42 families (see online supplemental material S5 for the workflow).

### Exome sequencing analysis

ES data were processed as described in the online supplemental materials S5 and S6. The variants in the vcf files were first filtered for the dystonia-associated genes based on the dystonia-associated gene list, which was developed through a systematic search on OMIM and SCAIView neuro^12^ (for the details of the genes, see online supplemental material S18), and the clinical significance of the retrieved variants were interpreted using Intervar.^13^ The pathogenic or likely pathogenic variants based on the American College of Medical Genetics and Genomics (ACMG)/Association for Molecular Pathology (AMP) guidelines or variant of uncertain significance (VUS) variants in the dystonia-associated genes were kept when (1) the clinical presentation of the family is highly specific for the identified gene variant, (2) the variant is determined as deleterious based on at least three pathogenicity scores obtained from dbNSFP^14^ using SIFT,^15^ CADD (Combined Annotation Dependent Depletion)(Phred score ≥15),^16^ PolyPhen2,^17^ MutationTaster^18^ and the Mendelian Clinically Applicable Pathogenicity score^19^ or Splicing Clinically Applicable Pathogenicity^20^ score (for the splicing variants) and LIST (Local Identity and Shared Taxa)^21^ (and (3) the variant is not present as a homozygous state in the control datasets including GnomAD^22^ and Turkish (TR) Variome.^11^

10.1136/jmg-2022-109099.supp2 Supplementary data

To identify the most plausible variants in the candidate genes, the remaining variants were prioritised based on (1) at least two prediction scores determining pathogenic and (2) minor allele frequency (MAF) in the control datasets (no homozygotes). The candidate genes were further interrogated for the enrichment with the established dystonia genes in the dystonia-associated CEN modules,^23^ (2) involvement in an interaction pattern in a GeneMANIA network generated using the genes in the dystonia-associated WGCNA modules and (3) possible dystonia-associated findings (reported phenotypes in mouse models with established gene mutations) in the animal models (AM) using ‘The Human-Mouse: Disease Connection’^24^ and ‘The International Mouse Phenotyping Consortium’.^25^ The candidate genes in the modules that are significantly enriched with the dystonia-associated genes were prioritised as the most plausible candidate genes (online supplemental material S5).

All the retrieved variants were confirmed and co-segregation analysis was performed by Sanger sequencing using the Big Dye Terminator Cycle Sequencing Ready Reaction kit on an ABI 3730 automated sequencer.

The statistical analyses were performed as described in the online supplemental material S7.

### CEN analysis

Dystonia is defined as a network disorder with involvement of a basal ganglia (BG)-cerebello-thalamo-cortical circuit.^26^ Therefore, using CEN analysis, we interrogated the BG and cerebellum (CRBL) regions (discovery data) of the brain obtained from the the Genotype-Tissue Expression Consortium (GTEx Consortium, 2016; V.7)^23^ database and the putamen region (replication data) of the brain obtained from the United Kingdom Brain Expression Consortium (UKBEC)^27^ database. We could analyse the caudate, nucleus accumbens and putamen regions of the BG as the discovery dataset, but only the putamen region as the replication dataset due to the unavailability of the datasets. In the CEN analysis, the candidate genes were interrogated for the modules, which are statistically enriched for the known dystonia-associated genes and involve our candidate genes as described in the online supplemental material S8. Fisher’s exact test was used to determine the modules that are statistically enriched for the dystonia-associated genes.

The gene set enrichment and over-representation (GSEO) and protein-protein interaction (PPI) analyses are described in the online supplemental material S8.

## Results

### Overview of the genetic findings

**Figure 1:**
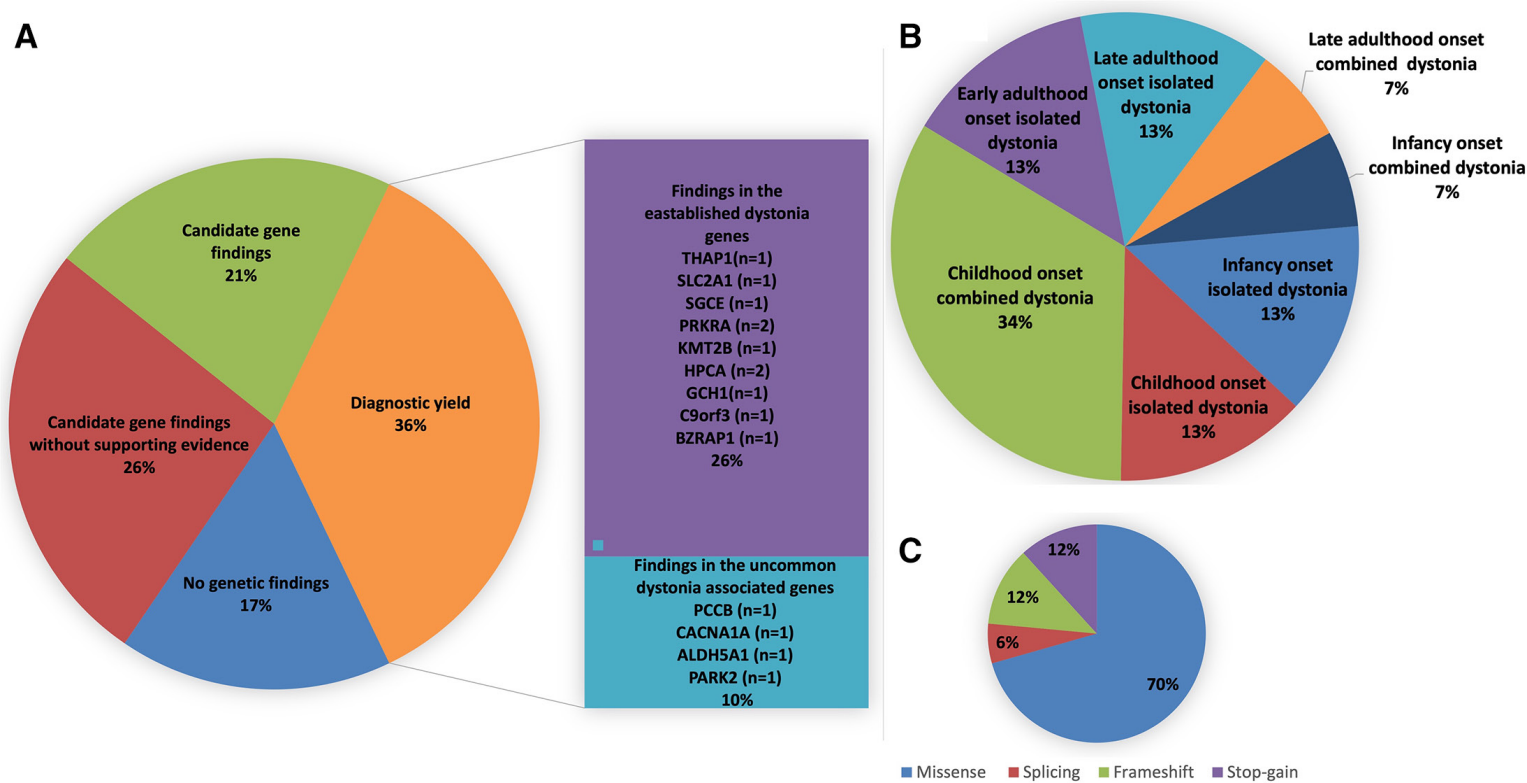
The classification and the characterisation of the 42 Turkish dystonia families based on the genetic findings. The chart indicates (A) the classification of the families based on the genetic findings, (B) the clinical distribution of the families in the diagnostic group and (C) the variant distribution in the diagnostic group.

Our ES analysis in 42 dystonia families allowed us to identify (1) pathogenic, likely pathogenic and VUS variants in the established and uncommonly dystonia-associated genes (n=15 (n=3 published elsewhere^28 29^); 36%) and (2) variants in the candidate genes prioritised based on the CEN-based evidence (n=9; 21%) (figure 1A). There is no statistical association between the mutation status and either age at onset or associated features.

All the pedigrees corresponding to the reported families are illustrated in the online supplemental materials S9, S10.

### Genetic findings in the dystonia-associated genes

Our ES approach revealed pathogenic, likely pathogenic and VUS variants in the established dystonia genes (*PRKRA*, *SGCE, KMT2B, SLC2A1, GCH1, THAP1, HPCA, TSPOAP1 (BZRAP1), AOPEP (C9orf3)*) as well as in the uncommonly dystonia-associated genes (*PCCB, CACNA1A, ALDH5A1, PRKN*) (table 2). Two variants were classified as VUS in the *PRKRA* (n=1 family) and *SGCE* (n=1 family) genes. However, because additional analysis (eg, structural variant analysis) did not reveal any other plausible variants and because the pathogenicity scores and the MAF-based findings supported the high possibility of pathogenicity, those variants were also listed in this study.

**Table 2:** The characteristics of the identified variants in the dystonia-associated genes

Gene symbol	Variant	GnomAD MAF* wt/het/hom	CADD†, SIFT, PolyPhen, mutation t@sting, M-CAP/S-CAP‡, LIST§ predictions	Zygosity	Disease involvement¶	Metabolic findings**	ACMG/AMP 2015 guideline-based pathogenicity††	Family (n=12)‡‡
Findings in the established dystonia genes								
PRKRA	NM_003690:c.704G>C:p.S235T	0.00004158 5/120264/0	22.8, T, B, DC, PP, 0.735297	HM	Generalised dystonia (MIM 612067)	–	VUS	DYS-66
PRKRA	NM_003690:c.665C>T:p.P222L	0.00009147 11/120256/0	21.8, T, B, DC, PP, 0.403158	CH	Generalised dystonia (MIM 612067)	–	Pathogenic	DYS-86
PRKRA	NM_003690:c.202T>C:p.F68L	0	29.8, D, PD, DC, PP, 0.922009	CH	Generalised dystonia (MIM 612067)	–	Likely pathogenic	
SGCE	NM_001099400:c.410G>A:p.R137H	0.0003517 42/119410/0	26, T, PD, DC, PP, 0.681176	H	Myoclonus-dystonia (MIM 159900)	–	VUS	DYS-72
KMT2B	NM_014727:c.2780T>G:p.V927G	0	22.6, D, B, DC, PP, 0.323893	H (de novo)	Childhood-onset generalised dystonia, developmental delay, microcephaly, intellectual disability, facial dysmorphism (MIM 617284)	–	Likely pathogenic	DYS-96
AOPEP (C9orf3)	NM_001193329:c.2055_2059delTGGAG:pG686EfsTer17	0	33, NA, NA, DC, NA, NA	HM	Generalised dystonia, dystonia-parkinsonism (MIM 619565)	–	Likely pathogenic	DYS-98
SLC2A1	NM_006516:c.259G>A:p.V87I	0	22.6, T, PD, DC, PP, 0.755548	H	Infantile-onset seizures, neurodevelopmental delay, microcephaly and complex movement disorders (MIM 601042)	CSF glucose concentration <60 mg/dL	Likely pathogenic	DYS-125
GCH1	NM_000161:c.520A>G:p.I174V	0	22.6, T, B, DC, PP, 0.953369	H (de novo)	Dystonia, DOPA-responsive, with or without hyperphenylalaninaemia (MIM 128230)	–	Likely pathogenic	DYS-134
THAP1	NM_018105:c.29G>T:p.C10F	0	32, D, PD, DC, PP, 0.883486	H	Adolescent-onset dystonia (MIM 602629)	–	Likely pathogenic	DYS-148
Findings in the uncommon dystonia genes								
PCCB	NM_000532:c.815G>A:p.R272Q	0.001480 178/120272/0	35, D, D, DC, PP, 0.883486	HM	Propionic acidemia (MIM 606054)	Decreased three hydroxypropionate	Likely pathogenic	DYS-68
CACNA1A	NM_023035:c.2137G>A:p.A713T	0	29.3, D, D, DC, PP, 0.980644	H (de novo)	Episodic ataxia, type 2 (MIM 108500)	–	Pathogenic	DYS-69
ALDH5A1	NM_170740:c.1441+1G>T	0	34, NA, NA, DC, NA, NA	CH	Succinic semialdehyde dehydrogenase deficiency (MIM 271980)	Decreased urine 4-hydroxybutyric acid (0 mmol/mol)	Pathogenic	DYS-71
ALDH5A1	NM_170740:c.1604A>G:p.D535G	0.00005484 6/109400/0	32, D, D, DC, PP, 0.971452	CH	Succinic semialdehyde dehydrogenase deficiency (MIM 271980)		Likely pathogenic	
PRKN	NM_004562:c.931C>T:p.Q311X	0	58, NA, NA, DC, PP, NA	HM	Parkinson’s disease, juvenile, type 2 (MIM 600116)	–	Pathogenic	DYS-147

*GnomAD-based MAF and allele counts.

†CADD score of >15 indicates deleteriousness for the variant.

‡M-CAP is a pathogenicity classifier for rare missense variants and S-CAP for the splicing variants.

§LIST score ranges between 0 and 1 where higher score indicates more deleterious effect.

¶OMIM-based information indicating the involvement of the genes in the diseases.

**Metabolic findings suggesting the abnormal levels of associated metabolites.

††ACMG/AMP-based pathogenicity classification.

‡‡Three families were published previously elsewhere.^28 29^

ACMG, American College of Medical Genetics and Genomics; AMP, Association for Molecular Pathology; B, benign; CH, compound heterozygous; CSF, cerebrospinal fluid; D, deleterious; DC, disease-causing; H, heterozygous; het, heterozygous; HM, homozygous; hom, homozygous alternate; LB, likely benign; MAF, minor allele frequency; M-CAP, Mendelian Clinically Applicable Pathogenicity; NA, not available; P, polymorphism; PD, possibly deleterious; PP, possibly pathogenic; S-CAP, Splicing Clinically Applicable Pathogenicity; T, tolerated; VUS, variant of uncertain significance; wt, homozygous reference.

The most frequently identified variants in the established dystonia genes in the diagnostic group were missense (n=12/17; 70%), followed by two stop-gain, two frameshift and one splicing variants (table 2, figure 1C).

In three families (DYS-96, DYS-134, DYS-69), de novo pathogenic variants in known dystonia-associated genes were identified (table 2).

Our analysis also uncovered pathogenic or likely pathogenic variants in uncommonly dystonia-associated genes (*PCCB, CACNA1A, ALDH5A1, PRKN*) in four families where dystonia has been rarely observed in the spectrum of the clinical characteristics so far.

We also identified a homozygous truncating variant in the *AOPEP (C9orf3*) gene, which has been recently identified in four independent families with varying clinical presentations including isolated and combined dystonia ranging from childhood onset to adulthood onset.^3^ The two patients of our DYS-98 family (IV.3, IV.6) were cousins presenting with early adulthood-onset isolated dystonia.

In the diagnostic group, the mostly presented phenotype was the childhood-onset combined dystonia (34%), yet the late-adulthood-onset combined dystonia (7%) and the infancy-onset combined dystonia (7%) (figure 1B) were also observed. Ataxia (4.7%), choreoathetosis (4.7%), developmental delay (2.3%), rigidity (4.7%), epilepsy or seizures (4.7%), bradykinesia (2.3%) and cognitive impairment (4.7%) were the additional clinical characteristics observed in the diagnostic group (table 1). Only one family (DYS-71) with *ALDH5A1* biallelic pathogenic variants was found to have T2 hyperintensity in BG.

### Genetic findings of the candidate genes in the Turkish dystonia cohort: a reference catalogue for the gene replication studies

Using our variant prioritising strategy, we have prioritised 12 variants in 10 plausible genes with supporting evidence for their possible role in the pathogenesis of dystonia, such as being involved in the CEN modules with the known dystonia genes (table 3). GSEO analysis further implicated those genes in several dystonia-associated pathways and gene ontologies (GOs) (online supplemental material S11).

**Table 3:** The characteristics of the prioritised variants in the candidate genes with CEN-based, PPI-based and AM-based evidence

Gene symbol	Variant/ZG	GnomAD MAF* hom1/het/hom2	CADD†, SIFT, PolyPhen, mutation t@sting, M-CAP‡, LIST§ predictions	Interaction partner	Reported disease¶/Neurological impairment in mouse models**	Module	Family	PLI††
Candidate genes								
TBC1D32	NM_152730:c.1184G>C:p.C395S/CH	0	22.3, T, PD, DC, LB, 0.503808	–	–	Turquoise	DYS-53	0
TBC1D32	NM_152730:c.254G>T:p.G85V/CH	0	6.2, T, P, DC, LB, 0.657924					
DZIP3	NM_014648:c.2589G>C:p.E863D/HM	0.000009183 1/108902/0	35, D, D, DC, PP, 0.720594	TH	–	Turquoise	DYS-54	0
CCNT1	NM_001240:c.1972C>G: p.Q658E/HM	0	23.3, D, B, DC, LB, 0.801523	TAF1	–	Turquoise	DYS-80	0.05
TBC1D8	NM_001102426:c.1532G>A:p.R511Q/HM	0	29, D, D, DC, PP, 0.992384	–	–	Turquoise	DYS-11	0
ANGEL1	NM_015305:c.82C>T:p.R28X/HM	0.001284154/119956/0	38, NA, NA, DC, NA	–	–	Turquoise	DYS-37	0
PDF	NM_022341:c.439C>T:p.P147S/HM	0	15, T, D, P, LB, 0.84258	–	–	Turquoise	DYS-111	0.05
Suggested phenotypic expansion genes								
PNP	NM_000270:c.141del:p.F48LfsTer26/CH	0	29, NA, NA, DC, NA	–	Immunodeficiency due to purine nucleoside phosphorylase deficiency (MIM 613179)/-	Black	DYS-4	0.07
PNP	NM_000270:c.637G>A:p.G213R/CH	0	32, D, D, DC, PP, 0.992812					
PRDM15	NM_022115:c.712del:p.F238LfsTer23/HM	0.0002287 25/109332/0	20.2, NA, NA, DC, NA	–	Galloway-Mowat syndrome/-	Turquoise	DYS-26	0.75
MCM4	NM_005914:c.1980G>C:p.R660S/HM	0	24.7, D, PD, DC, PP, 0.957833	CDC7	Immunodeficiency 54 (MIM 609981)/Increased grip strength (Mcm4tm1b(KOMP)Mbp/Mcm4+)/(Prkra^em1(IMPC)H^, Hpca^em1(IMPC)H^)‡‡	Turquoise	DYS-55	0
CEP120	NM_153223:c.482G>A:p.G161E/HM	0.0001657 18/108624/0	22.6, T, B, DC, LB, 0.624987	–	Joubert syndrome 31 (MIM 617761) Short-rib thoracic dysplasia 13 with or without polydactyly (MIM616300)/-	Turquoise	DYS-54	0

*GnomAD-based MAF and allele counts.

†CADD score of >15 indicates deleteriousness for the variant.

‡M-CAP is a pathogenicity classifier for rare missense variants and S-CAP for the splicing variants.

§LIST score ranges between 0 and 1 where higher score indicates more deleterious effect; >0.6 is considered as deleterious effect.

¶OMIM and literature findings were indicated if the mode of inheritance of the associated disease is compatible.

**HMDC-based mouse model information.

††Based on the ExAC (Exome Aggregation Consortium) consortium computed data, pLI, probability that a gene is intolerant to a loss-of-function mutation (pLI ≥0.9 are extremely loss-of-function intolerant).

‡‡Reported phenotype in the IMPC.

AM, animal models; B, benign; CEN, co-expression network; CH, compound heterozygous; D, deleterious; DC, disease-causing; H, heterozygous; het, heterozygous; HM, homozygous; HMDC, The Human-Mouse: Disease Connection; hom, homozygous alternate; IMPC, International Mouse Phenotyping Consortium; LB, likely benign; MAF, minor allele frequency; M-CAP, Mendelian Clinically Applicable Pathogenicity; NA, not available; P, polymorphism; PD, possibly deleterious; PP, possibly pathogenic; PPI, protein-protein interaction; S-CAP, Splicing Clinically Applicable Pathogenicity; T, tolerated; wt, homozygous reference.

Through CEN analysis, we identified one (turquoise module; false discovery rate (FDR)-corrected Fisher’s exact p=3.62×10^−8^) out of 13 modules that is statistically enriched for the dystonia-associated genes and involves our candidate genes in the BG region of the brain. Also, the black module (Fisher’s exact p=0.04716) in the BG region was classified as a significant module, even though after multiple test correction it was not found to be statistically significant. Our CEN analysis also identified one significant module (turquoise module; FDR-corrected Fisher’s exact p=0.022383) in the CRBL region. However, no candidate gene was identified in this module. In the putamen region of the UKBEC data, no significant module was identified after FDR correction (online supplemental material S19).

10.1136/jmg-2022-109099.supp3 Supplementary data

### Candidate genes in the CEN modules

Novel compound heterozygous pathogenic variants (NM_000270;c.141del;p.F48LfsTer26) were identified in *PNP* gene. Structural modelling analysis (online supplemental material S12 revealed that the pathogenic variants have an impact on the ligand binding and enzyme active sites in two affected siblings presenting with paroxysmal movement disorder including ataxia, choreoathetosis and dystonia as well as recurrent severe infection in childhood (family DYS-4). We assessed the effect of the pathogenic variants on the enzyme activity and observed that due to the possible enzyme deficiency, the level of inosine (DYS4-III.1; 4.3, DYS4-III.2; 1.4 mmol/moK) was elevated in blood.

**Figure 2:**
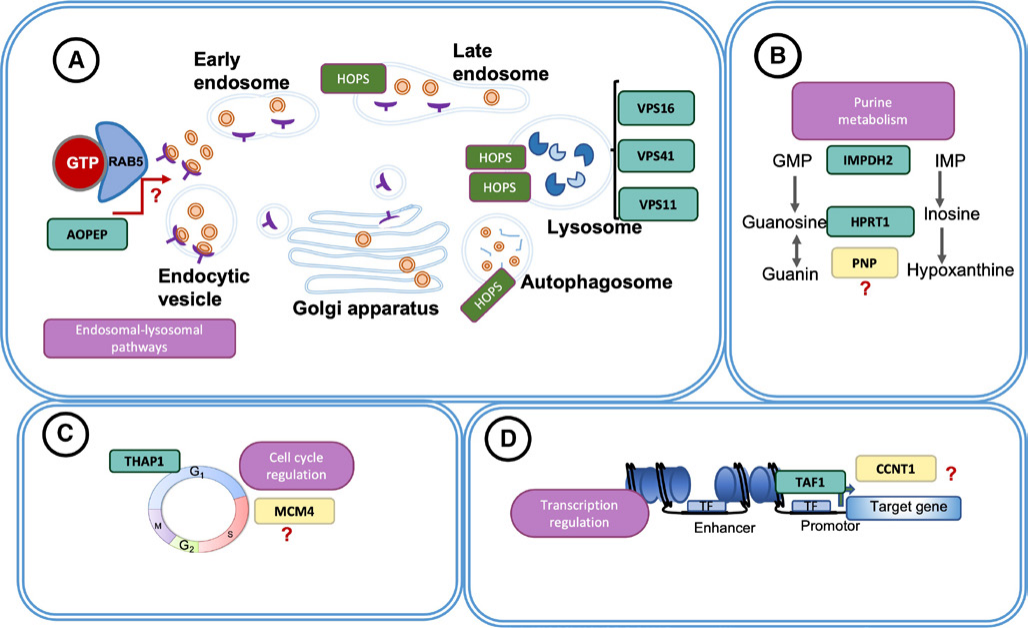
The prioritised pathways associated with dystonia. This figure illustrates the prioritised pathways associated with dystonia based on the findings of our study as well as the findings from previous studies. Those pathways include (A) the endosomal-lysosomal pathway involving *AOPEP, VPS16, VPS41* and *VPS11* genes, (B) the purine pathway involving I*MPDH2, HPRT1* and *PNP* genes, (C) the cell-cycle pathway including *THAP1* and *MCM4* genes, (D) the transcriptional regulation pathway including *TAF1* and *CCNT1* genes. In green rectangles the known dystonia-associated genes, in yellow rectangles candidate genes identified in our study have been shown. HOPS, the homotypic fusion and protein sorting complex; TF, transcription binding factor.

A novel homozygous pathogenic variant (NM_005914;c.1980G>C;p.R660S) in the *MCM4* gene, which was previously associated with immunodeficiency 54 (MIM:609981) (online supplemental material S13), has been found to co-segregate with the disease in a large family (DYS-55) including five unaffected and two affected individuals presenting with adolescence-onset isolated dystonia. Mouse model studies revealed neurological, cardiovascular, embryological and growth impairments in heterozygous knockout mouse models, as well as lethal impairments in homozygous knockout mouse models, implying that homozygous mutations may cause more severe molecular perturbations. *MCM4* is a component of the MCM2-7 complex involved in the initiation of eukaryotic genome replication and has been reported to be implicated in neurogenesis^30^ (figure 2C).

A novel homozygous variant (NM_001240;c.1972C>G;p.Q658E) of another compelling gene, *CCNT1*, was identified in one patient (DYS-80; II.2) presenting with childhood-onset myoclonus dystonia. The *CCNT1* gene encodes a subunit of the positive transcription elongation factor B (P-TEFb), or the so-called CDK9/cyclin-T1, which facilitates the elongation of the transcription (figure 2D).

Our analysis also revealed other compelling candidate genes (*PRDM15, TBC1D32, DZIP3, TBC1D8, ANGEL1, PDF, CEP120*), of which the details are provided in table 3.

## Discussion

This is the first study in the literature that has uncovered the genetic basis or associated clinical characteristics of dystonia families from Turkey using a combination of genetic screening strategies.

Our analyses showed that dystonia is a genetically and clinically highly heterogeneous group of disorders. Even though our study cohort consisted of only recessive forms of the disease from a particular geographical region where recessive forms are common due to the high rate of consanguinity, our analysis revealed a wide spectrum of genetic underpinnings, including known common and uncommon forms of the disease with various modes of inheritance (figure 1).

The highest rates of the variants have been found in the *PRKRA* and *HPCA* genes in our dystonia cohort. Findings for *HPCA* have been discussed before.^28^ The vast majority of *PRKRA* pathogenic variant carriers published so far have shown generalised dystonia^31^ as our patients do. Based on the ACMG/AMP guidelines, one (p.S235T) of the *PRKRA* gene variants was not categorised as a pathogenic variant in the group of dystonia-associated genes due to the incompatible co-segregation in the family (DYS-66), where an asymptomatic family member was also found to be the carrier of the variant. However, the onset of symptoms related to PRKRA pathogenic variants reported so far varies from 1 to 53 years. Therefore, the asymptomatic family member (33 (DYS66; IV.2) year) could also develop symptoms later.

The p.S235T variant has been previously reported to be associated with intellectual disability in infancy.^32^ Our patient (DYS-66, IV.5) started to suffer from dystonia in the childhood. A neuropsychological examination at the age of 20s revealed mild disinhibition and attentional problems as well as mild verbal memory deficits, but no intellectual disability was reported either for him or for his asymptomatic sister carrying the same variant (p.S235T). Our analysis did not reveal any other plausible variants in the family DYS-66, therefore; the variant was listed in our study.

The p.P222L variant identified together with the p.F68L variant in a compound heterozygous state in the family DYS-86 is the most frequently identified pathogenic variant in *PRKRA*. Clinical symptoms of this patient progressed quickly, leading to GPi-DBS at age of 20s only 3 years after the onset.

The p.R137H variant in the *SGCE* gene was not categorised as pathogenic based on the ACMG/AMP guidelines. Due to the clinical compatibility, the variant was also listed in our study.

Our ES approach also identified variants in the autosomal dominant (AD) genes, which include *SGCE*, *KMT2B, GCH1, SLC2A1* and *THAP1* (n=5/15; 33.3%). Despite the fact that our cohort consisted of families with an AR mode of inheritance, our study revealed a relatively high proportion of pathogenic or likely pathogenic variants in the AD genes due to (1) the presence of de novo variants, (2) maternal imprinting and (3) the genes’ reduced penetrance.

In addition, we identified a homozygous truncating variant in *AOPEP (C9orf3*) gene in a multigeneration family (DYS-98) with two affected individuals presenting with early adulthood-onset isolated dystonia. *AOPEP* gene has recently been linked to DYT31 causing multifocal or generalised dystonia.^3^ The pathogenic variant (p.G686EfsTer17) identified in our study is localised in the C-terminal outside of the putative active domains and leads to the early adulthood-onset dystonia, suggesting that the position of the pathogenic variants in the A*OPEP* gene does not change the impact on the dystonia pathogenesis. Additionally, structural modelling analysis showed that the mutant protein lacked one of the essential ligand binding sites (online supplemental material S14) and had a deficiency in some of the biological functions, such as leukotriene-A4 hydrolase activity, epoxide hydrolase activity and interleukin-1 receptor binding. Our CEN and GSEO analyses implicated *AOPEP* with some GOs including endocytosis (online supplemental material S11c). Furthermore, PPI analysis showed an interaction (online supplemental material S15b) of AP-O with *SUN2*, which has been shown as an activator of *RAB5* in the endocytic vesicles. Endocytic pathways have been suggested to have a role in differentiation and migration of neuronal cells during development and in adulthood.^33^ Additionally, a recent study suggested the role of the homotypic fusion and protein sorting complex-mediated endosomal-lysosomal pathways in the dystonia pathogenesis.^34^ Therefore, it might be speculated that *AOPEP* could be implicated in the dystonia pathogenesis through being involved in the endosomal-lysosomal pathways (figure 2A).

Pathogenic or likely pathogenic variants were found in four families in genes that are not commonly associated with dystonia (*PCCB, CACNA1A, ALDH5A1* and *PRKN*). Among those genes, *PCCB* and *ALDH5A1* have been associated with metabolic disturbances. In line with our findings, we identified downstream metabolic imbalances (table 2), indicating that those pathogenic variants played a role in disease pathogenesis.

In one family (DYS-69), our analysis revealed a de novo pathogenic variant (NM_023035:c.2137G>A:p.A713T) in the *CACNA1A* gene, which is mainly associated with episodic ataxia and rarely dystonia. It has been proven by AM studies as well as systems-biology-based analysis that dystonia and ataxia share some pathological mechanisms,^35^ suggesting the role of convergent disease mechanisms.

Another interesting finding was the identification of homozygous *PRKN* stop-gain pathogenic variant (NM_004562;c.931C>T;p.Q311X), which was previously reported in a Turkish family presenting with adolescence-onset parkinsonism without dystonia,^36^ in a patient with childhood-onset generalised dystonia without parkinsonism. Lower limb dystonia, which sometimes remains an isolated finding for years, is frequently associated with the Parkin-phenotype. However, isolated generalised dystonia is very uncommon in *PRKN* pathogenic variant carriers, suggesting shared and sometimes convergent mechanisms in different movement disorders, particularly ataxia, dystonia and parkinsonism.

So far, several studies have been performed in different dystonia cohorts^5–10^ from mainly European populations and different diagnostic yields ranging from 11.7% to 39.1% have been reported. In those studies, some common genes (*ADCY5, ANO3, ATM, ATP1A3, ATP7B, CACNA1A, FA2H, GCH1, GLB1, GNAL, GNAO1, KMT2B, NKX2-1, NPC1, PANK2, PLA2G6, PRKN, PRKRA, PRRT2, SGCE, SLC2A1, TH, THAP1, TOR1A*) have been reported (online supplemental material S16). Even though the *CACNA1A, GCH1, KMT2B, PRKN, PRKRA, SGCE, SLC2A1* and *THAP1* genes have been replicated, our study also differently identified the *HPCA, ALDH5A1* and *PCCB* genes in our cohort. The largest cohort of those studies has been reported by Zech *et al*,^37^ which consisted of cases with predominantly European (n=663/708; n=94%) and scarcely other ethnicities, including only 9 (1%) Turkish, and achieved a diagnostic yield of 19% (n=135/728 families). In the study by Zech *et al*, no pathogenic variants were reported in *PRKRA, HPCA, GCH1, ALDH5A1* and *PCCB* genes, suggesting differences in the composition of our cohort and theirs.

With the advent of NGS techniques, a substantial number of dominantly inherited genes have been associated with dystonia. However, a limited number of recessively inherited genes could be discovered and replicated by independent studies due to the rarity as well as the clinical heterogeneity of AR dystonia. Hitherto, of those genes, only *HPCA* and recently *AOPEP*, could be confirmed by independent studies.^4 28 38^ Therefore, we generated a list of plausible candidate genes with systems-biology-based evidence identified in a clinically well-characterised dystonia cohort to aid in future gene discovery approaches.

Dystonia is defined as a network disorder affecting the BG and the CRBL regions of the brain. Therefore, we generated CENs based on the BG and the CRBL regions (discovery data) from the GTEx database and putamen region (replication data) from the UKBEC database using WGCNA and interrogated the dystonia-associated modules including our candidate genes (n=30). In the CEN analysis, we identified one statistically dystonia-associated module (turquoise) including our candidate genes in the BG region. However, no candidate genes were identified in the significant module (turquoise) of the CRBL region. In the putamen data, no statistically dystonia-associated module was identified. The reason might be that while we could analyse three subregions (caudate, nucleus accumbens and putamen) from the BG region in the GTEx data, we could only analyse putamen subregion in the UKBEC data. Therefore, only the over-represented pathways, GOs and particular PPIs in the networks for the dystonia-associated modules based on the BG region were determined. Lastly, animal model research-based and literature-based information with all the evidence supporting the role of those genes in the dystonia pathogenesis were associated with the clinical and genetic findings (table 3).

Based on our strategy, 10 genes (*PNP*, *MCM4*, *PRDM15*, *TBC1D32*, *DZIP3*, *CCNT1, TBC1D8, ANGEL1, PDF, CEP120*) have been prioritised. Of those genes, *PNP* encodes purine nucleoside phosphorylase and is involved in the purine degradation and salvage pathway (figure 2B). *PNP* deficiency has been shown to cause a wide range of clinical symptoms, including neurological abnormalities, such as ataxia, tremor, hypertonia and dystonia (in only one case). As has been shown in the case of adenosine deaminase (ADA) deficiency, which is another enzyme involved in the purine degradation-salvage pathway, as well as in the knockout mouse models, purine metabolism could be implicated in neurological disturbances.^39^

Furthermore, our CEN analysis showed that *PNP* and co-expressed dystonia genes were enriched for immune-system-associated pathways in particular. Of the associated over-represented pathways, cytokine-cytokine receptor interaction, Jak-STAT signalling pathway and MAPK signalling pathways (online supplemental file S11b) have been shown to be dysregulated in the mutant (THAP1;c.71+9C>A) lymphoblastoid cells.^40^ In addition, in an independent study, it has been suggested that knockdown of the hypoxanthine guanine phosphoribosyltransferase—a purine biosynthesis gene—led to the constitutive activation of the MAPkinases phosphor-ERK1/2.^41^

Furthermore, a recently identified new dystonia-associated gene, *IMPDH2*, supports the role of the purine metabolism in the dystonia pathogenesis.^37 42^ Based on the recent findings, the association between the purinergic pathways and the neurodevelopmental disorders has been proposed, however, the contribution of those mechanisms to the disease could not be yet elucidated. The possible involvement of the immune systems in the pathogenesis of the purine-metabolism-associated diseases has been reported by different studies,^43^ suggesting a presumptive indication of a link with dystonia pathogenesis.

The genes in the CEN modules identified in our study were enriched also for the transcription processes, ubiquitination-related mechanisms and synaptic transmission, which are the molecular mechanisms disturbed in dystonia^44 45^ (online supplemental material S11a).

Of those genes, *MCM4* has been shown to be involved in DNA replication complex with a particular role in neurogenesis, and furthermore, CEN and PPI analysis showed that *MCM4* and Cdc7 (online supplemental material S15a) might act as a complex in cell-cycle regulation, suggesting a possible role of the *MCM4* gene in the cell-cycle-related dystonia pathogenesis (figure 2C). Another compelling gene, *CCNT1* is involved in the elongation of the transcription and reported to be an interactor^46^ of TAF1 (dystonia-parkinsonism; MIM 314250) (figure 2D).

We also identified variants in the genes (n=20) without CEN-based evidence due to their low or different expression pattern in different brain regions and their involvement in different pathogenic mechanisms, where the known dystonia-associated genes are not involved (online supplemental material S17). Some of those genes have been reported to be involved in neurological disturbances in AM studies. Therefore, we did not rule out those genes as possible genetic causes in the affected families in our cohort.

Our study failed to retrieve variants in seven families (17%) (for the detailed clinical information, see online supplemental material S4), suggesting either the limitations of the ES-based screening approach or some inconsistency in the clinical information obtained from the families.

In conclusion, in this study, we have uncovered the genetic basis in dystonia families with well-defined clinical characteristics from Turkey. Using comprehensive genetic screening strategies, we genetically and clinically characterised the families with pathogenic variants in the known dystonia-associated genes, and provided a list of candidate genes with CEN-based, animal research-based, PPI-based or literature-based evidence for the pathogenicity of dystonia. Our study revealed several insights into the pathogenesis of dystonia, including: (1) the substantial difference in the genetic composition of dystonia in different populations; (2) the importance of broad genetic screening approaches in diagnostic settings rather than focusing only on known dystonia genes; (3) the considerable involvement of convergent disease mechanisms in the pathogenesis of dystonia, ataxia, and Parkinson’s disease and (4) the significant contribution of novel and known pathways, such as immune system, transcription, neurodevelopmental, endosomal-lysosomal and metabolic pathways.

10.1136/jmg-2022-109099.supp4 Supplementary data

10.1136/jmg-2022-109099.supp5 Supplementary data

## Data Availability

Data are available on reasonable request.
