# Supplementary material for: Dissecting genetic architecture of rare dystonia: genetic, molecular and clinical insights
Source: J Med Genet. 2024 Mar 8;61(5):443–51. doi: 10.1136/jmg-2022-109099 (PMC11041572; doi:10.1136/jmg-2022-109099)
Supplement: Supplementary data [file jmg-2022-109099supp001.pdf]

Supplementary Material

S1 THE CLINICAL CHARACTERISTICS OF THE FAMILIES WITH KNOWN GENETIC FORMS OF DYSTONIA 2

S2 THE DETAILED CLINICAL CHARACTERISTICS OF THE FAMILIES WITH PRIORITIZED VARIANTS IN THE GENES WITH CEN BASED EVIDENCE 5

S3 THE DETAILED CLINICAL CHARACTERISTICS OF THE FAMILIES WITH VARIANTS IN THE GENES WITHOUT SUPPORTING EVIDENCE 7

S4 THE DETAILED CLINICAL CHARACTERISTICS OF THE FAMILIES WITH NO GENETIC CAUSE IDENTIFIED IN THE STUDY 8

S5 GENETIC SCREENING STRATEGY 10

S6 ES DATA PROCESSING 10

S7 STATISTICAL ANALYSES 11

S8 DATA PROCESSING OF THE CEN DATA 12

S9 PEDIGREES OF THE FAMILIES WITH FINDINGS IN THE DYSTONIA ASSOCIATED GENES 13

S10 PEDIGREES OF THE FAMILIES WITH PRIORITIZED VARIANTS IN THE CANDIDATE GENES WITH CEN-BASED EVIDENCE 15

S11 THE ASSOCIATED PATHWAYS IDENTIFIED IN FOUR MODULES BASED ON THE GSEO ANALYSIS 16

S12 STRUCTURAL MODELING OF THE PATHOGENIC VARIANTS IDENTIFIED IN THE FAMILY DYS-4 17

S13 *MCM4* ASSOCIATED REPORTED PHENOTYPE 17

S14 STRUCTURAL MODELING OF THE PATHOGENIC VARIANTS IDENTIFIED IN THE FAMILY DYS-98 18

S15 GENEMANIA NETWORKS OF THE GENES IN PARTICULAR MODULES 19

S16 COMMON GENES REPORTED IN STUDIES FROM DIFFERENT POPULATIONS 20

S17 THE CHARACTERISTICS OF THE VARIANTS WITHOUT CEN- BASED EVIDENCE 22

REFERENCES 25

Atasu B, *et al. J Med Genet* 2024; 61:443–451. doi: 10.1136/jmg-2022-109099

S1 The clinical characteristics of the families with known genetic forms of dystonia

Family	Clinical information	Gene
**Families with DYT classified Mendelian forms of dystonia**		
DYS-5	TR, M, CS<br>AO infancy: involuntary head movements, 20s: left side dystonia, gait abnormalities, facial grimacing, jerking of the trunk and limbs, 20s: generalized dystonia, ACE-R 54/91, MMSE: 22/28 right pallidotomy<br>AE 30s: generalized dystonia, dysphagia, dysarthria<br>cMRI: normal	HPCA
DYS-56	TR<br>Patient II.1, M<br>AO: 60s AE 70s: dystonic hand tremor, MCI<br>Patient II.3, M<br>AO: 60s AE 70s: CD, MCI<br>Patient II.4, F<br>AO: 50s AE 60s: CD, MCI	TSPOAP1
DYS-66	TR, M, CS<br>AO: Childhood AE: 20s, generalized dystonia	PRKRA
DYS-72	TR M<br>AO: birth, febrile seizure, permanent tremor of the UL<br>AE; 30s, myoclonus dystonia, cMRI: mega cisterna magna	SGCE

Atasu B, *et al. J Med Genet* 2024; 61:443–451. doi: 10.1136/jmg-2022-109099

DYS-86	TR, M<br><br>AO; 20s, generalized dystonia, DBS, AE 20s: CD	PRKRA
DYS-96	TR, M<br><br>AO; childhood, left dystonic hand tremor, articulation difficulties, low speech volume<br><br>AE: 20s, choreatic movements predominating in the UL, cervico-truncal dystonic postures, right sided rigidity, slow horizontal eye saccades<br><br>cMRI: normal	KMT2B
DYS-98	TR, CS<br><br>Patient IV.3, M<br><br>AO: 20s, AE 30s : left arm dystonia, CD, cMRI: normal<br><br>Patient IV.6 M<br><br>AO : 30s: BSP, CD AE 30s , segmental dystonia, cMRI : normal	AOPEP
DYS-125	TR/BG<br><br>Patient III.5, F<br><br>AO: childhood, action tremor of the UL, AO: 50s, BSP, MCI (MMSE 21/30)<br><br>Patient IV.1, F<br><br>AO: childhood, involuntary paroxysmal non-epileptic movements including ataxia, choreoathetosis and dystonic postures, learning disabilities, IQ51 (normal>85)<br><br>cMRI: normal, CSF/blood glucose ratio: 0,56 mg/dL, CSF lactate concentration: low-normal (1,28 mmol/L)<br><br>AE 10-20 years: in average two episodes of paroxysmal, mainly exercise-induced dyskinesia per month lasting from several up to 20 minutes. ACE-R: deficits in fluency (6/14), but nearly normal cognition (attention 18/18, memory 19/26, language 22/26, visuospatial 14/16, total 79/100, cut-off 83/100)	SLC2A1
DYS-134	TR, M<br><br>AO: 40s, writer's cramp, tremor of the right UL, symptoms fully disappear after alcohol consumption<br><br>AE: 40s: action and postural tremor of the right hand, rapid fatiguing of effort with repetitive motor tasks resulting in dystonic postures, particularly during writing	GCH1

DYS-139	TR, M, CS<br><br>AO: childhood, paroxysmal movement disorder, LL dystonia<br><br>AE: 20s, generalized dystonia<br><br>cMRI: normal	HPCA
DYS-148	TR<br><br>Patient III.2, M<br><br>AO: childhood , AE 30s: right hand-forearm dystonia, mainly manifest as WC<br><br>Patient III.3, F<br><br>AO: 20s, seizures (2x) without evident cause, AE: 30s, normal<br><br>Patient III.4, M<br><br>AO: childhood , AE: 20s: spasmodic dysphonia, mild CD	THAP1
**Families with uncommon forms of dystonia**		
DYS-68	TR, F, CS<br><br>AO 50s: BSP, AE 50s: generalized dystonia, loss of weight<br><br>Testing of plasma amino acids revealed elevated glycine.	PCCB
DYS-69	TR, F<br><br>AO: birth AE: childhood, dystonic truncal posture, dyskinesic permanent movements of the UL, walking difficulties	CACNA1A
DYS-71	TR<br><br>Patient III.1, M<br><br>AO: childhood, involuntarily, repetitive twisting movements of the back, severe retrocollis, mild dysarthria, hyporeflexia, 21s: cervico-laryngeal cramps and permanent feeling of retching. restless, aggressive behaviour, incoherent speech, hallucinations, diagnosis of psychosis<br><br>AE: 20s: truncal dystonia, irregular retrocollis, ataxia, choreoathetotic movements of the UL, ACE-R at 42/100 (cut-off 83/100), cMRI: small, non-specific right parietal T_2-hyperintensity in the white matter<br><br>Patient III.2, M	ALDH5A1

	AO: 10-20 years, AE 20s: dysarthria, CD<br>4-hydroxybutyric aciduria present in both patients.	
DYS-147	Patient V.3 F<br>TR, F, CS<br>AO: childhood , right-sided hemi-dystonia associated with involuntary movements, levodopa-responsive for several years, 33y: left pallidotomy<br>AE: 30s, DBS, dysarthria, LL dystonia and hyperreflexia, right predominant bradykinesia and rigidity, anxiety, MMSE 15/30	PARK2

AO: age at onset AE: age at examination, y: years, CS: consanguinity of the parents, M: male, F: female, TR: Turkey, BG: Bulgaria, ACE-R: Addenbrooke Cognitive Examination revised, MMSE: Mini Mental State Examination, cMRI: cerebral Magnetic Resonance Imaging, Sd: syndrome, WC: writer's cramp, DBS: deep brain stimulation, LL: lower limbs, UL: upper limbs, CD: cervical dystonia, MCI: mild cognitive impairment, BSP: blepharospasm.

S2 The detailed clinical characteristics of the families with Prioritized Variants in the genes with CEN based evidence

Family number	Clinical information	Candidate genes
DYS-4	TR<br>Patient III.1<br>F, AO: childhood , AE: 20s<br>Patient III.2<br>F, AO: birth, AE: 10-20 years<br>paroxysmal movement disorder including intermittent ataxia, choreoathetosis, dystonia	PNP
DYS-11	TR , CS, F	TBC1D8

	neonatal jaundice, motor development delay<br><br>AO: infancy, foot twisting, childhood: dystonic posture LL, walking distance 500m, 10y: involuntary spasms occurring in the foot, legs, trunk or also in the oromandibular region, brisk tendon reflexes, striatal toe, no cognitive impairment<br><br>AE 20s: generalized dystonia, important improvement with levodopa<br><br>cMRI: normal	
DYS-26	TR, CS, M<br><br>lingual titubation (AO: childhood)<br><br>alopecia (AO: 20s)<br><br>AO 20s: WC<br><br>AE 30s: WC, rest tremor, panic attacks	PRDM15
DYS-37	TR, CS, F<br>AO: childhood, right hand dystonia<br>AE: 10-20 years, hallucination, anxiety, neck, truncal and arm dystonia<br>cMRI: T2 bilateral hypotensities in globus pallidus internus<br>ACE-R: 71/100; MMSE 26/30	*ANGEL1*<br>*ABTB2*<br>*NPC1L1*<br>*DLST*
DYS-53	TR, M<br><br>AO: childhood , AE childhood , right foot dystonia, walking difficulties, improvement with levodopa	TBC1D32
DYS-54	TR, CS, M<br><br>AO: childhood, right arm dystonia<br><br>generalized dystonia, 20s: DBS<br><br>AE: 20s, generalized dystonia, dysphonia	DZIP3<br><br>CEP120
DYS-55	TR<br><br><u>Patient V.5</u><br><br>AO: juvenile, CD, AE: 30s, generalized dystonia, hand tremor	MCM4

	cMRI: normal<br>Patient V.6<br>AO: 10-15 years, CD, AE: 30s, segmental dystonia<br>cMRI: normal	
DYS-80	TR, M<br>AO: childhood, right hand dystonia<br>AE: 10s: WC, myoclonic dystonia	CCNT1<br>SH3TC2*
DYS-110	TR, F<br>AO: 40s, CD, head tremor<br>AE: 60s, CD, head and hand tremor	*TUBAL3*<br>*DENND3*<br>*DSG4*<br>*ADGRD1*

*: Corresponding variant has been previously reported.

S3 The detailed clinical characteristics of the families with variants in the genes without supporting evidence

Family	Clinical information	Candidate genes
DYS-13	TR, F<br>AO 30s: CD<br>AE 40s: CD, depression<br>cMRI: normal	*SHKBP1*
DYS-18	TR, M<br>AO: juvenile, hand tremor<br>AE: 20s, WC, exhaustible bilateral nystagmus, action tremor, rigidity<br>cMRI (21s): mild cerebellar atrophy	TBC1D2B
DYS-26	TR, CS, M<br>AO: childhood<br>AE 30s: WC, rest tremor, panic attacks	*UMODL1*<br>*ZNF79*
DYS-41	TR/BG, F<br>AO 20s: left leg tremor, muscle cramps<br>AE 20s: left leg dystonia, generalized bradykinesia, amimia, tremor<br>cMRI: normal	*SCNN1D*
DYS-70	TR, CS, M<br>AO: childhood AE: juvenile	*ARID1B*<br>*HUWE1*

	exercice induced mouvements<br>cMRT: normal	
DYS-74	TR, F<br>AO 30s: CD<br>AE 30s: generalized dystonia, dysarthria<br>cMRI: normal	*SLFN14*
DYS-82	TR, CS, M<br>AO: childhood , right hand tremor/dystonia juvenile onset: DBS<br>AE: juvenile, generalized dystonia	*HEATR5B*
DYS-91	TR, CS, F<br>AO: 20s, CD<br>20s: left thalamotomy<br>cCT: normal<br>AE: 40s, tremor generalized dystonia	*DNAH11*
DYS-97	TR, CS, M<br>AO: infantile, generalized choreatic movements<br>Febrile seizure<br>AE: juvenile, generalized dystonia<br>cMRI: normal	*FBXO10*<br>*ICAM3*
DYS-111	TR, F<br>AO 40s: dystonic tremor UL<br>AE 40s: mild rigidity/bradykinesia, generalized dystonic tremor<br>cMRI: normal	*PDF*<br>*STAB1*<br>*PLEKHG4*
DYS-146	TR, CS, M<br>AO: childhood, left hand tremor, epilepsy<br>AE 40s: left hemidystonia with tremor<br>cMRI : normal	*CABIN1*<br>*KCNJ8*<br>*EPHB4*

AO; age at onset, AE; age at examination, y; years, CS; consanguinity of the parents, M; male, F; female, TR; Turkey, BG; Bulgaria, cMRI; cerebral Magnetic Resonance Imaging, Sd; syndrome, DBS; deep brain stimulation, CD; cervical dystonia

S4 The detailed clinical characteristics of the families with no genetic cause identified in the study

Family	Origin	Sex	CS	Clinical presentation
DYS-3	TR	F	yes	AO: juvenile, rapid onset dystonia<br>AE 20s: severe generalized dystonia<br>cMRI: normal
DYS-28	TR	F, F	no	<u>Patient III.3:</u><br>AO : juvenile, cervical dystonia<br>AE 60s: segmental dystonia<br><u>Patient III.6:</u><br>AO : childhood, cervical dystonia

				AE 40s, segmental dystonia
DYS-62	TR/ TKM	M	no	AO: childhood: cervical dystonia, several myoclonia/tremor<br>AE 30s: myoclonus dystonia, suicide attempt
DYS-95	TR	F	no	AO: infantile, dystonic postures, micrognathism neonatal jaundice, motor development delay<br>AE: childhood, generalized dystonia<br>cMRI: normal
DYS-126	TR	F	no	AO 40s: cervical dystonia<br>AE 50s : cervical dystonia, mild head tremor
DYS-143	TR	M	no	AO childhood , dystonic and ballistic movements left arm<br>AE juvenile, segmental dystonia<br>cMRI: normal
DYS-150	TR	M	no	AO 20s: rapid onset dystonia<br>AE 20s: generalized dystonia<br>cMRI: normal

AO; age at onset, AE; age at examination, y; years, CS; consanguinity of the parents, M; male, F; female, TR; Turkey, TKM; Turkmenistan, cMRI; cerebral Magnetic Resonance Imaging

Atasu B, *et al. J Med Genet* 2024; 61:443–451. doi: 10.1136/jmg-2022-109099

S5 Genetic screening strategy

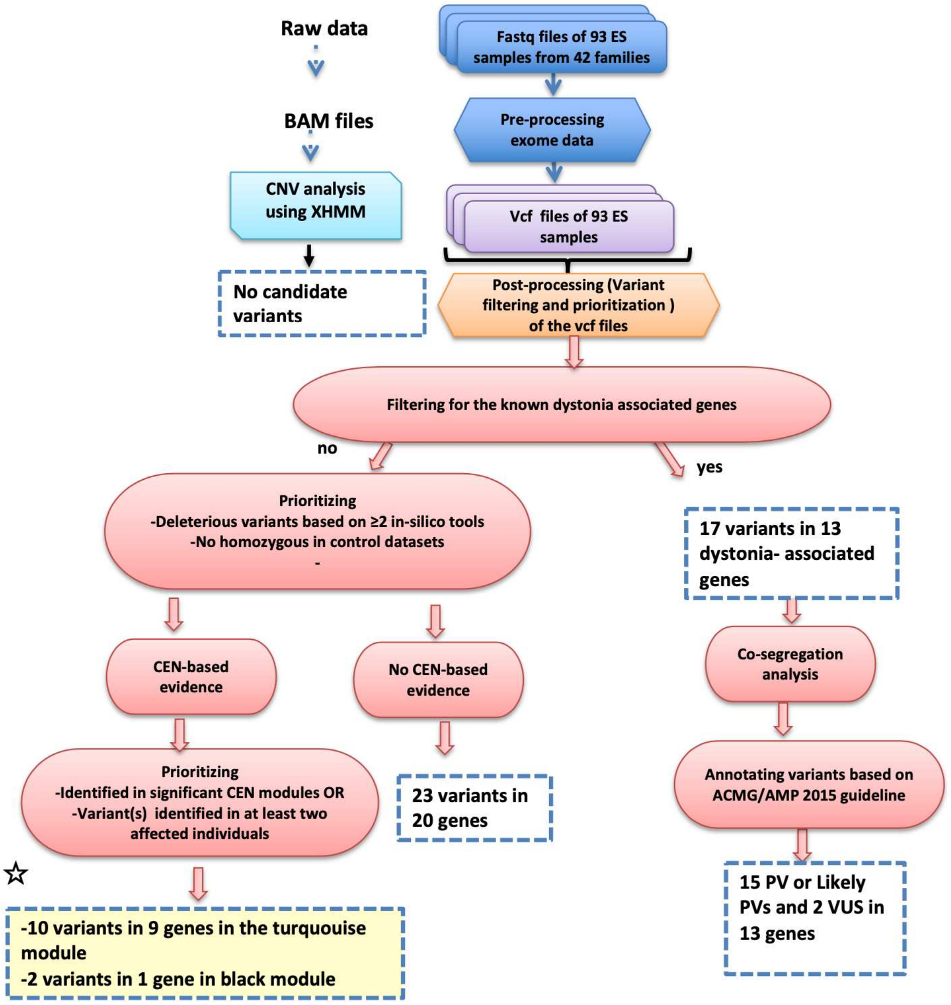

S5.1 Systematic illustration of the workflow. The figure illustrates the systematic screening approach conducted to analyze the 42 dystonia families from Turkey.

S6 ES Data Processing

Briefly, ES was performed using in-solution technology from Agilent (SureSelectXT Human All Exon V5) as per manufacturers' protocols at Cegat[1]. High-throughput sequencing was

carried out by the HiSeq 2500 Illumina platform with 2×100 bp. Sequence alignment to the human reference genome (UCSC GRCh37/hg19) was performed using the Burrows-Wheeler Alignment Tool[2]. Picard tools[3] were used for marking duplicates, and GATK (GATKtools-3.8) was employed for local realignment, base quality recalibration, and variant calling approaches based on the GATK best practices[4]. Runs of homozygosity were identified using H3M2[5].

After generating callsets, we proceeded with genotype refinement and variant prioritization approaches by using KGGSeq[6].

After confirming the mode of inheritance of the affected families based on the inbreeding coefficient values, the variants were filtered for (1) the confirmed mode of inheritance, (2) minor allele frequency (MAF ≤ 0.01) based on 1kg201305, dbsnp138, dbsnp141, ESP5400, ESP6500EA, ESP6500AA, GnomAD and in-house databases.

Structural modeling analysis was performed using I-TASSER server[7]. In essence, XHMM uses principal component analysis (PCA) for normalization and a hidden Markov model (HMM) to detect and to genotype copy number variations (CNVs) from normalized read-depth data from targeted sequencing experiments.

Our pipeline uses the BAM files from the preprocessing step of the ES data as input files and subsequently runs GATK to calculate raw depth-of-coverage values across the exome, followed by the first filter step, PCA, the second filter step, and lastly, calls the CNVs by using the standard HMM Viterbi algorithm, which provides the most likely copy-number state given all the sample's read-depth data and fixed HMM parameters.

S7 Statistical analyses

The statistical analyses were performed using R version 4.1.1. Statistical association between the groups and the disease-associated variables were calculated using Fisher's exact

Atasu B, *et al. J Med Genet* 2024; 61:443–451. doi: 10.1136/jmg-2022-109099

test and comparisons between the groups on continuous data was done using the Kruskal Wallis test. The statistical analysis of the overlap between the dystonia associated genes and the genes identified in the modules was performed using Fisher's exact test. The Benjamini Hochberg and Bonferroni methods were used to correct the p values.

S8 Data Processing of the CEN Data

Correlation networks were constructed using Transcript Per Million values (GTEx_Analysis_2016-01-15_v7_RNASeQCv1.1.8_gene_tpm.gct.gz) corresponding to the BG region and CRBL regions from GTEX, and log2 converted Reads Per Kilobase of transcript per Million (RPKM) values (resids.PUTM.8.rds) corresponding to the putamen region from UKBEC. UKBEC data corresponding to the putamen region of the brain was pre-processed as described in the study by Guelfi et al[8].Modules were identified using the hierarchical clustering method as per the instructions of the weighted correlation network analysis (WGCNA) R package of R (version 3.5.1). After clustering of the samples, no outlier was detected in the BG and the CRBL regions, however, one outlier (A653_1302) detected in the putamen region. After extracting the outliers and filtering the lowly expressed genes we proceeded with 17394 genes from 432 samples in the BG data, 18253 genes from 175 samples in the CRBL data, and 20886 genes from 104 samples in the putamen data. To construct the networks based on approximate scale-free topology, we chose the soft thresholding power β as 12, 10, 7 and the minimum module size as 30, 60 and 30 for the BG, CRBL and putamen datasets, respectively. For each module the module membership value cut off was +0.75 or -0.75 (Supplementary Material S19 and S20).

Based on the overlap between the dystonia-associated genes and the genes in the modules the significant modules were identified. In the BG region of the Gtex dataset only turquoise module was found to be statistically enriched for the dystonia-associated genes and to be involving our

candidate genes. In the CRBL region of the Gtex data no candidate gene was identified in the significant module and in the putamen region of the UKBEC data no significant module was detected.

Gene Set Enrichment and Over-representation Analysis

We performed GSEO analysis using the HTSanalyzeR[9] package (version 2.32.0) of R. We set the minimum gene-set size to 10 and used the default values of the remaining parameters.

Protein-Protein Interaction Analysis

Protein-protein interaction (PPI) and co-localization of the proteins of interest were further investigated using Cytoscape 3.8.0[10] based on the GeneMANIA[11] network.

S9 Pedigrees of the Families with Findings in the Dystonia Associated Genes

DYS-66

PRKRA homozygous variant : NM_003690:c.704G>C: p.S235T

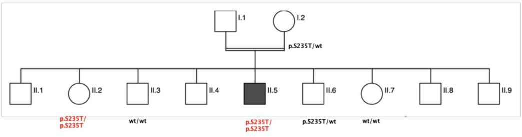

DYS-72

SGCE heterozygous variant : NM_001099400: c.410G>A: p.R137H

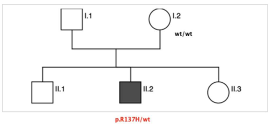

DYS-86

PRKRA compound heterozygous variant : NM_003690:c.665C>T:p.P222L;
NM_003690:c.202T>C:p.F68LP

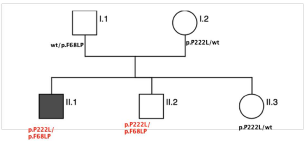

DYS-96

KMT2B heterozygous variant : NM_014727:c.2780T>G:p.V927G

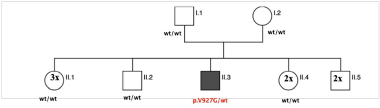

DYS-98

AOPEP (C9orf3) homozygous variant: NM_001193329:c.2055_2059delTGGAG:pG686EfsTer17

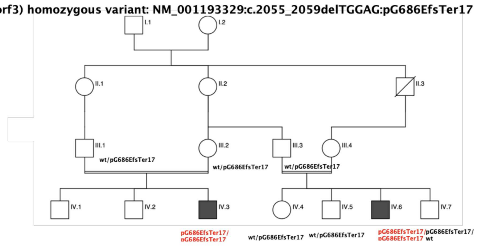

DYS-125

SLC2A1 heterozygous variant : NM_006516:c.259G>A:p.V87I

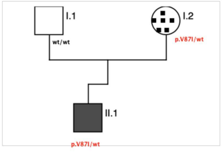

DYS-134

GCH1 heterozygous variant : NM_000161:c.520A>G:p.I174V

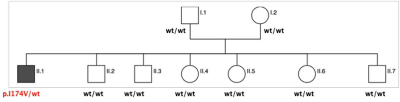

DYS-148

THAP1 heterozygous variant : NM_018105:c.29G>T:p.C10F

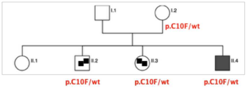

DYS-68

PCCB homozygous variant : NM_000532:c.815G>A:p.R272Q

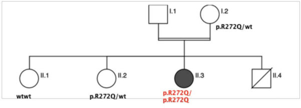

DYS-69

CACNA1A heterozygous variant :NM_023035:c.2137G>A:p.A713T

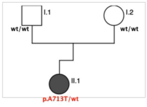

DYS-71

ALDH5A1 compund heterozygous variants : NM_170740:c.1441+1G>T; NM_170740:c.1604A>G:p.D535G

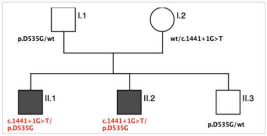

DYS-147

PARK2 homozygous variant :NM_004562:c.931C>T:p.Q311*

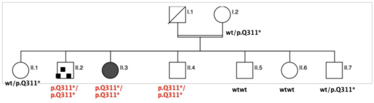

S10 Pedigrees of the families with Prioritized Variants in the Candidate Genes with CEN-based evidence

DYS-4

PNP compound heterozygous variant: NM_000270:c.141del:p.F48LfsTer26 / NM_000270:c.637G>A:p.G213R/

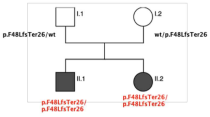

DYS-11

TBC1D8 homozygous variant: NM_001102426:c.1532G>A:p.R511Q

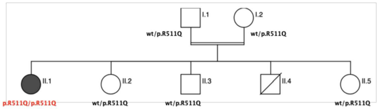

DYS-26

PRDM15 homozygous variant: NM_022115:c.712del:p.F238LfsTer23

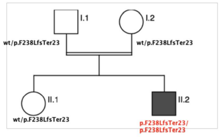

DYS-37

ANGEL1 homozygous variant: NM_015305:c.82C>T:p.R28X

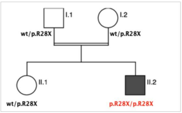

DYS-53

TBC1D32 compound heterozygous variants: NM_152730:c.1184G>C:p.C395S/ NM_152730:c.254G>T:p.G85V

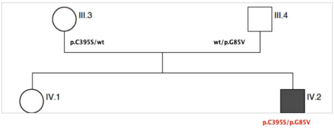

DYS-54

DZIP3 homozygous variant: NM_014648:c.2589G>C:p.E863D
CEP120 homozygous variant: NM_153223:c.482G>A:p.G161E

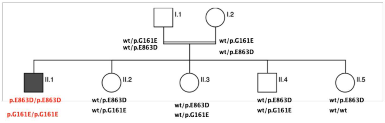

DYS-55

MCM4 homozygous variant: NM_005914:c.1980G>C:p.R660S

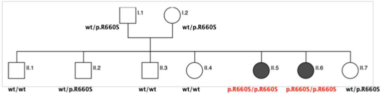

DYS-80

CCNT1 homozygous variant: NM_001240: c.1972C>G: p.Q658E
SH3TC2 compound heterozygous variants: NM_024577:c.2990G>A:p.R997Q/ NM_024577:c.1436A>G:p.Y479C

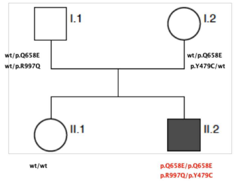

DYS-111

PDF homozygous variant: NM_022341:c.439C>T:p.P147S

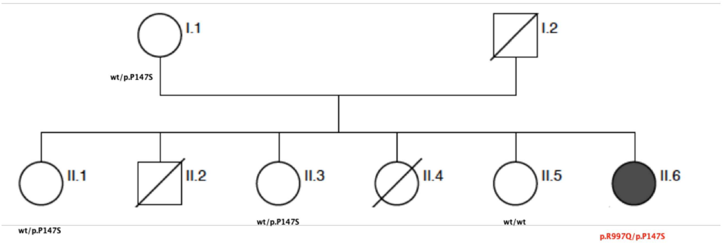

Atasu B, *et al. J Med Genet* 2024; 61:443–451. doi: 10.1136/jmg-2022-109099

S11 The associated pathways identified in four modules based on the GSEO analysis

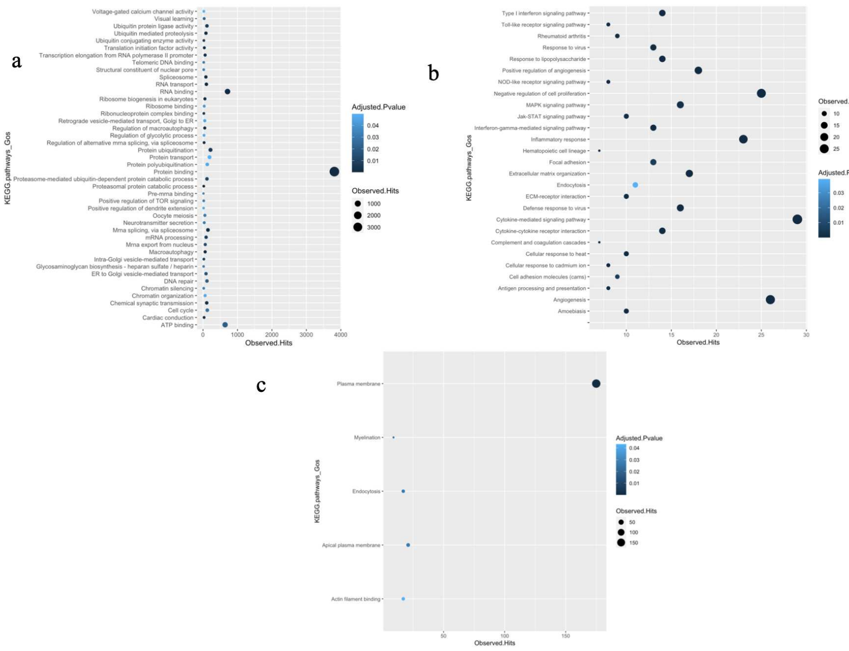

The dotplots represent the overrepresented pathways based on the enriched genes in the (a) turquoise module and (b) black module involving the *PNP* gene, and (c) brown module involving the *AOPEP* gene.

Atasu B, *et al. J Med Genet* 2024; 61:443–451. doi: 10.1136/jmg-2022-109099

S12 Structural modeling of the pathogenic variants identified in the family DYS-4

Ligand binding sites

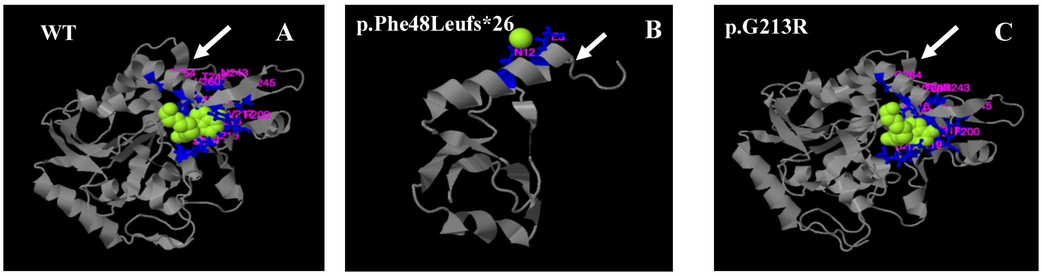

Enzyme active sites

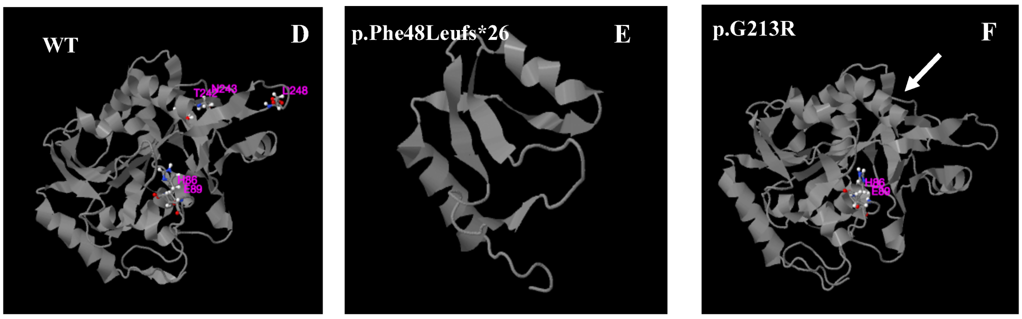

Structural modeling showed that the mutated (p.Phe48Leufs*26, p.G123R) PNP lack of the essential ligand binding sites (B,C) as well as the enzyme active sites (E,F), compared to the wild type protein (A,D).White arrow indicates the affected sites of the protein models.

S13 *MCM4* associated reported phenotype

MCM4 gene has previously been associated with immunodeficiency 54 (MIM 609981) that was described in independent families with the same PV from Irish traveller community presenting with recurrent viral infections, decreased number of natural killer (NK) cells and glucocorticoid deficiency, without neurological disturbances except for delayed cognitive development in only one family[12,13]. In this study, none of the symptoms associated with NK

Atasu B, *et al. J Med Genet* 2024; 61:443–451. doi: 10.1136/jmg-2022-109099

cells and glucocorticoid deficiency except for isolated dystonia in the affected cases of the family DYS-55 with MCM4 PVs was observed.

S14 Structural modeling of the pathogenic variants identified in the family DYS-98

Ligand binding sites

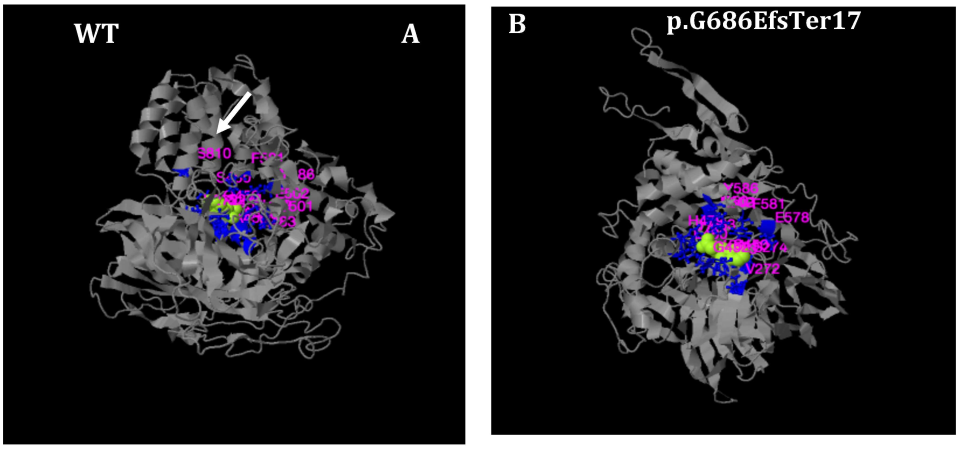

Enzyme active sites

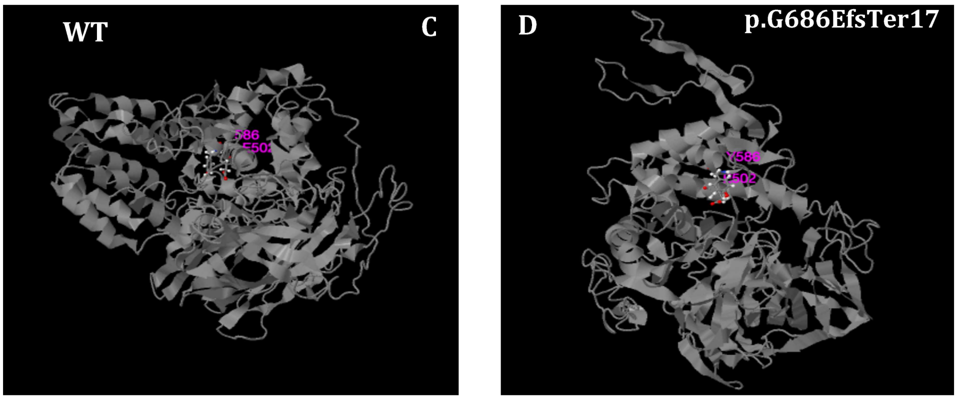

Atasu B, *et al. J Med Genet* 2024; 61:443–451. doi: 10.1136/jmg-2022-109099

Structural modeling showed that the mutated (pG686EfsTer17) AP-O lack of one of the essential ligand binding sites (indicated by an arrow) (A, B). No major difference was detected in the enzyme active sites (C, D), compared to the wild type protein.

S15 GeneMANIA networks of the genes in particular modules

a) b)

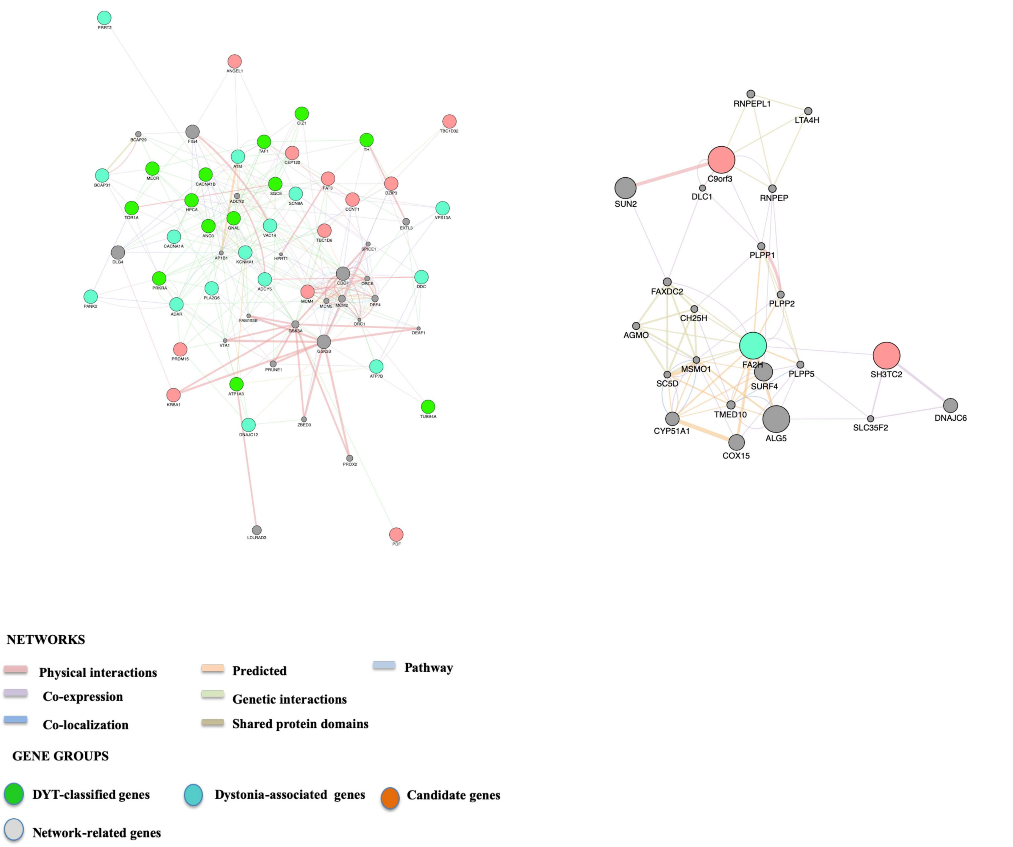

The representation of some of the interesting protein-protein interactions in the GeneMania modules. The PPI network generated based on the genes form the turquoise module identified in the BG region of Gtex dataset. b) The PPI network generated based on the genes from the brown module identified in the BG region of Gtex dataset. This network involves the *AOPEP(C9orf3)* gene. In the figure green nodes represent the DYT categorized dystonia associated genes, blue nodes represent dystonia associated genes, orange nodes represent our candidate genes and grey nodes represent the network associated genes.

S16 Common Genes Reported in Studies from Different Populations

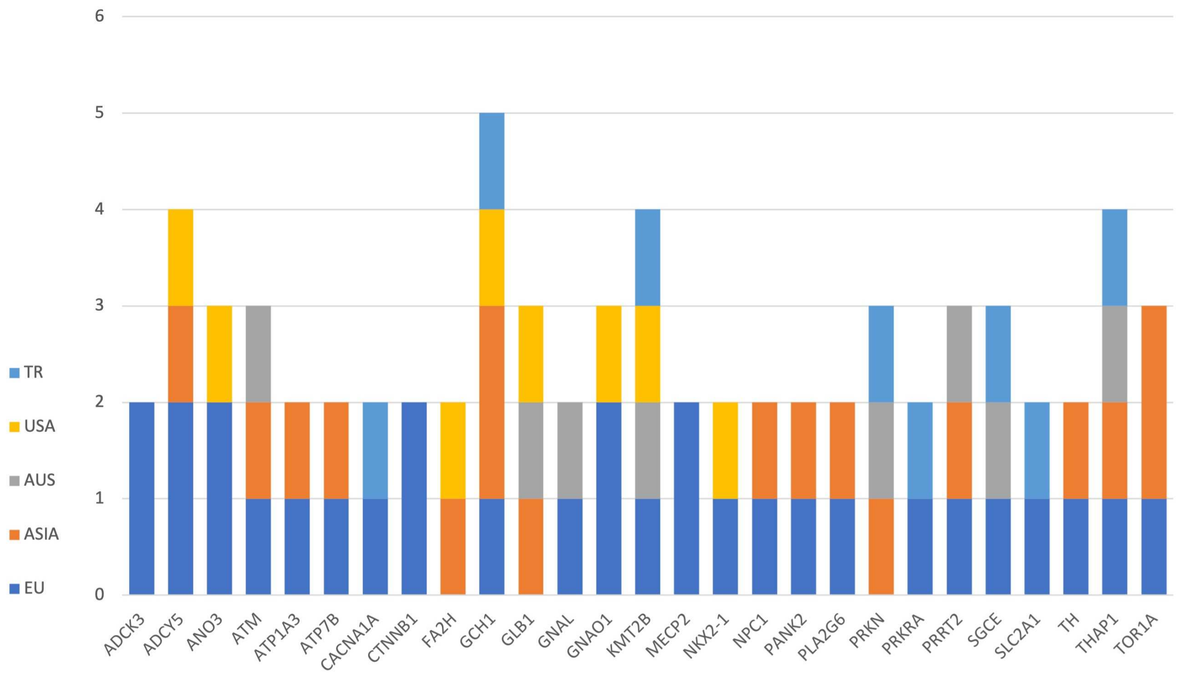

Representation of common genes reported in so far reported studies from different populations. Reported genes have been indicated as per study: EU; including mainly European populations[14,15,16,17], ASIA; including Asian populations[18,19,20], AUS; including Australian

population[20], USA; including American population[21], TR; including Turkish population reported in this study.

S17 The characteristics of the variants without CEN- based evidence

Gene symbol	Variant	Zygosity	GnomAD MAF[a] hom1/het/hom2	CADD[b], SIFT, PolyPhen, mutation t@sting, M-CAP/S-CAP[c]	Interaction partner	Reported Disease[d]	Neurological impairment in mouse models[e]	Family	PLI[f]	Expression in brain[g]
GLDC	NM_000170:c.2402G>T:p.S801I*[22]	HT (De-novo)	0	24.9, D, PD, DC, PP	-	-	-	DYS-19	0	L
SCNN1D	NM_001130413:c.1657A>G:p.R553G	HT (De-novo)	0	11.6, T, NA, P, DC	-<br>-	-<br>-	-<br>-	DYS-41	0	M
SLFN14	NM_001129820:c.1513A>T:p.K505X	HM	0	38, NA, NA, DC, NA				DYS-74		
HEATR5B	NM_019024:c.1475A>G:p.N492S	HT (De-novo)	0.00003657 4/109392/0	21.4, T, B, DC, LB	-	-	-	DYS-82	0	M
ADGRD1	NM_198827:c.278G>T:p.C93F	HT (De-novo)	0	23.7, D, B, DC, PP	-	-	-	DYS-110	0	L
NPC1L1	NM_013389:c.2293C>T: P765S	HM	0	17, T, NA, P, PP	-	-	-	DYS-37	0	L
DLST	NM_001933:c.442+25A>G	HM	0.000009144 1/109366/0	19, NA, NA, DC, PP	-	-	-		0.48	M
ABTB2	NM_145804:c.2966G>A: p.R989Q	HM	0.0002899 34/117268/0	23, D, B, DC, PP	-	-	Increased grip strength<br>$Abtb2^{tm1a(KOMP)Wtsi}/Abtb2^{tm1a(KOMP)Wtsi}$		0	L
FBXO10	NM_012166:c.1954A>G:p.M652V	CH	0	11.79, T, B, DC, PP	-	-	-	DYS-97	0	L
FBXO10	NM_012166:c.1609G>A:p.V537I	CH	0.0001270 15/118152/0	25.2, T, PD, DC, PP						
TUBAL3	NM_024803:c.178T>C:p.F60L	HT (De-novo)	0.00001828 2/109408/0	24.4, D, PD, DC, PP	-	-	-	DYS-110	0	L

PLEKHG4	NM_001129728:c.2966A>G:p.N989S	HM	0.00007523 29/119634/0	21.8, T, B, P, LB	-	-	-	DYS-111	0	L
STAB1	NM_015136:c.526C>T:p.R176C	CH	0.00006653 8/120244/0	19, T, P, P, PP	-	-	-		0	L
STAB1	NM_015136:c.1796C>T:p.A599V	CH	0.00001676 2/119336/0	25.3, D, D, DC, PP						
EPHB4	NM_004444:c.1692G>C:p.R564S	HM	0.000009140 1/109404/0	27, T, PD, DC, NA	-	-	-	DYS-146	0	L
KCNJ8	NM_004982:c.263C>G:p.A88G	HM	0.0001828 20/109406/0	21.3, T, B, DC, PP	-	-	-		0.29	L
CABIN1	NM_012295:c.5245G>A:p.D1749N	HM	0.0001957 23/117550/0	19.1, T, B, DC, PP	-	-	-		0	M

Suggested Phenotypic Expansion Genes

ITGA7	NM_001144996:c.3274C>T:p.R1092X	CH	0.00003327 4/120234/0	44, NA, NA, DC, NA	-	Muscular dystrophy, congenital, due to ITGA7 deficiency (MIM 613204)	-	DYS-41	0	M
ITGA7	NM_001144996:c.772C>T:p.L258F	CH	0.00004666 5/107162/0	25.2, D, PP, DC, PP	-	-	-			
HUWE1	NM_031407:c.10400A>T:p.N3467I:	HeM	0	38, D, PD, DC, PP	-	Mental retardation, X-linked syndromic, Turner type (MIM 300706)	-	DYS-70	1	M
DNAH11	NM_001277115:c.10967G>A:p.R3656H	HM	0.00001847 2/108294/0	22.4, NA, NA, DC, PP	-	Ciliary dyskinesia, primary, 7, with or without situs inversus (MIM 611884)	-	DYS-91	0	L
DSG4	NM_177986:c.3047C>T:p.T1016I	HT (De-novo)	0	16.5, NA, NA, DC, LB	-	Hypotrichosis 6 (MIM 607903)	-	DYS-110	0	No expression

TBC1D2B	NM_144572:c.1978C>T:p.R660C	HM	0	28.1, D, D, DC, PP, 0.96381	-	Neurodevelopmental disorder with seizures and gingival overgrowth (MIM 619323)	-	DYS-18	0	M

The table indicates the identified genes without CEN based supporting evidence. Some of the identified genes in this group have been shown to be causing variety of impairments in the mouse model studies. [a]: GnomAD based MAF and allele counts, wt: Homozygous reference, het: Heterozygous, hom: Homozygous alternate, MAF: Minor allele frequency. [b]: CADD score of >15 indicates deleteriousness for the variant. [c]: M-CAP is a pathogenicity classifier for rare missense variants and S-CAP for the splicing variants. [d]:OMIM and literature findings were indicated if the mode of inheritance of the associated disease is compatible. * HGMD report of the variant. [e]: HMDC based mouse model information. [f]: Based on the ExAc consortium computed data, pLI, probability that a gene is intolerant to a loss-of-function mutation (pLI>=0.9 are extremely loss-of-function intolerant). [g]: Expression levels based on the GTEx portal. H: Highly-expressed, M: Moderately-expressed, N: Not-expressed. B; benign, PD; possibly deleterious, D; deleterious, T; Tolerated, LB; likely-benign, PP; possibly pathogenic, NA; not available, DC; disease-causing, P; polymorphism, HM; homozygous, CH; compound heterozygous, H; heterozygous

References

[1] CeGaT, Genetic diagnostics and NGS services, Tubingen. http://www.cegat.de/en/.

[2] Li H, Durbin R. Fast and accurate short read alignment with Burrows-Wheeler transform. Bioinformatics. 2009 Jul 15;25(14):1754-60.

[3] "Picard Toolkit." 2019. Broad Institute, GitHub Repository. https://broadinstitute.github.io/picard/; Broad Institute.

[4] DePristo MA, Banks E, Poplin R, Garimella KV, Maguire JR, Hartl C, Philippakis AA, del Angel G, Rivas MA, Hanna M, McKenna A, Fennell TJ, Kernytsky AM, Sivachenko AY, Cibulskis K, Gabriel SB, Altshuler D, Daly MJ. A framework for variation discovery and genotyping using next-generation DNA sequencing data. Nat Genet. 2011 May;43(5):491-8.

[5] Pippucci T, Magi A, Gialluisi A, Romeo G. Detection of runs of homozygosity from whole exome sequencing data: state of the art and perspectives for clinical, population and epidemiological studies. Hum Hered. 2014;77(1-4):63-72.

[6] Li MX, Gui HS, Kwan JS, Bao SY, Sham PC. A comprehensive framework for prioritizing variants in exome sequencing studies of Mendelian diseases. Nucleic Acids Res. 2012 Apr;40(7):e53.

[7] Roy A, Kucukural A, Zhang Y. I-TASSER: a unified platform for automated protein structure and function prediction. Nature protocols. 2010; 5(4):725–38.

[8] Guelfi S, D'Sa K, Botía JA, Vandrovcova J, Reynolds RH, Zhang D, Trabzuni D, Collado-Torres L, Thomason A, Quijada Leyton P, Gagliano Taliun SA, Nalls MA; International Parkinson's Disease Genomics Consortium (IPDGC); UK Brain Expression Consortium (UKBEC); Small KS, Smith C, Ramasamy A, Hardy J, Weale

ME, Ryten M. Regulatory sites for splicing in human basal ganglia are enriched for disease-relevant information. Nat Commun. 2020 Feb 25;11(1):1041.

[9] Wang X, Terfve C, Rose JC, Markowetz F. HTSanalyzeR: an R/Bioconductor package for integrated network analysis of high-throughput screens. Bioinformatics. 2011 Mar 15;27(6):879-80.

[10] Shannon P, Markiel A, Ozier O, Baliga NS, Wang JT, Ramage D, Amin N, Schwikowski B, Ideker T. Cytoscape: a software environment for integrated models of biomolecular interaction networks. Genome Res. 2003 Nov;13(11):2498-504.

[11] Warde-Farley D, Donaldson SL, Comes O, Zuberi K, Badrawi R, Chao P, Franz M, Grouios C, Kazi F, Lopes CT, Maitland A, Mostafavi S, Montojo J, Shao Q, Wright G, Bader GD, Morris Q. The GeneMANIA prediction server: biological network integration for gene prioritization and predicting gene function. Nucleic Acids Res. 2010 Jul;38(Web Server issue):W214-20.

[12] Gineau, L., Cognet, C., Kara, N., Lach, F. P., Dunne, J., Veturi, U., Picard, C., Trouillet, C., Eidenschenk, C., Aoufouchi, S., Alcais, A., Smith, O., Geissmann, F., Feighery, C., Abel, L., Smogorzewska, A., Stillman, B., Vivier, E., Casanova, J.-L., Jouanguy, E. Partial MCM4 deficiency in patients with growth retardation, adrenal insufficiency, and natural killer cell deficiency. J. Clin. Invest. 122: 821-832, 2012.

[13] Hughes, C. R., Guasti, L., Meimaridou, E., Chuang, C.-H., Schimenti, J. C., King, P. J., Costigan, C., Clark, A. J. L., Metherell, L. A. MCM4 mutation causes adrenal failure, short stature, and natural killer cell deficiency in humans. J. Clin. Invest. 122: 814-820, 2012.

[14] van Egmond ME, Lugtenberg CHA, Brouwer OF, Contarino MF, Fung VSC, Heiner-Fokkema MR, van Hilten JJ, van der Hout AH, Peall KJ, Sinke RJ, Roze E, Verschuuren-Bemelmans CC, Willemsen MA, Wolf NI, Tijssen MA, de Koning TJ. A

post hoc study on gene panel analysis for the diagnosis of dystonia. Mov Disord. 2017 Apr;32(4):569-575. doi: 10.1002/mds.26937.

[15] Zech M, Boesch S, Jochim A, Weber S, Meindl T, Schormair B, Wieland T, Lunetta C, Sansone V, Messner M, Mueller J, Ceballos-Baumann A, Strom TM, Colombo R, Poewe W, Haslinger B, Winkelmann J. Clinical exome sequencing in early-onset generalized dystonia and large-scale resequencing follow-up. Mov Disord. 2017 Apr;32(4):549-559.

[16] Wirth T, Tranchant C, Drouot N, Keren B, Mignot C, Cif L, Lefaucheur R, Lion-François L, Méneret A, Gras D, Roze E, Laroche C, Burbaud P, Bannier S, Lagha-Boukbiza O, Spitz MA, Laugel V, Bereau M, Ollivier E, Nitschke P, Doummar D, Rudolf G, Anheim M, Chelly J. Increased diagnostic yield in complex dystonia through exome sequencing. Parkinsonism Relat Disord. 2020 May;74:50-56. doi: 10.1016/j.parkreldis.2020.04.003.

[17] Zech M et al. Monogenic variants in dystonia: an exome-wide sequencing study. Lancet Neurol. 2020 Nov;19(11):908-918. doi: 10.1016/S1474-4422(20)30312-4

[18] Ma J, Wang L, Yang YM, Wan XH. Targeted gene capture sequencing in diagnosis of dystonia patients. J Neurol Sci. 2018 Jul 15;390:36-41. doi: 10.1016/j.jns.2018.04.005.

[19] Holla VV, Neeraja K, Stezin A, Prasad S, Surisetti BK, Netravathi M, Kamble N, Yadav R, Pal PK. Utility of Clinical Exome Sequencing in Dystonia: A Single-Center Study From India. J Mov Disord. 2022 May;15(2):156-161. doi: 10.14802/jmd.21146.

[20] Kumar KR, Davis RL, Tchan MC, Wali GM, Mahant N, Ng K, Kotschet K, Siow SF, Gu J, Walls Z, Kang C, Wali G, Levy S, Phua CS, Yiannikas C, Darveniza P, Chang FCF, Morales-Briceño H, Rowe DB, Drew A, Gayevskiy V, Cowley MJ, Minoche AE, Tisch S, Hayes M, Kummerfeld S, Fung VSC, Sue CM. Whole genome sequencing for

the genetic diagnosis of heterogenous dystonia phenotypes. Parkinsonism Relat Disord. 2019 Dec;69:111-118.

[21] Powis Z, Towne MC, Hagman KDF, Blanco K, Palmaer E, Castro A, Sajan SA, Radtke K, Feyma TJ, Juliette K, Tang S, Sidiropoulos C. Clinical diagnostic exome sequencing in dystonia: Genetic testing challenges for complex conditions. Clin Genet. 2020 Feb;97(2):305-311. doi: 10.1111/cge.13657.

[22] Yoshikawa A, Nishimura F, Inai A, Eriguchi Y, Nishioka M, Takaya A, Tochigi M, Kawamura Y, Umekage T, Kato K, Sasaki T, Ohashi Y, Iwamoto K, Kasai K, Kakiuchi C. Mutations of the glycine cleavage system genes possibly affect the negative symptoms of schizophrenia through metabolomic profile changes. Psychiatry Clin Neurosci. 2018 Mar;72(3):168-179.
