# Supplementary material for: Dissecting genetic architecture of rare dystonia: genetic, molecular and clinical insights
Source: J Med Genet. 2024 Mar 8;61(5):443–51. doi: 10.1136/jmg-2022-109099 (PMC11041572; doi:10.1136/jmg-2022-109099)
Supplement: Supplementary data [file jmg-2022-109099supp002.pdf]

| Gene Name | MIM Number | Synonyms/ Locus | Phenotype | Source |
|---|---|---|---|---|
| *AARS* | 601065 | *AARS, CMT2N, EIEE29* | Epileptic encephalopathy, early infantile, MIM-616339, Autosomal recessive | OMIM |
| *AARS2* | 612035 | *AARS2, KIAA1270, MTALARS, COXPD8, LKENP* | Leukoencephalopathy, progressive, with ovarian failure, MIM-615889, Autosomal recessive | OMIM |
| *ACOX1* | 609751 | *ACOX1, ACOX, SCOX* | Peroxisomal acyl-CoA oxidase deficiency, MIM-264470, Autosomal recessive | OMIM |
| *ADAR* | 146920 | *ADAR, DRADA, DSH, DSRAD, IFI4, G1P1, AGS6* | Aicardi-Goutieres syndrome 6, MIM-615010, Autosomal recessive; Dyschromatosis symmetrica hereditaria, MIM-127400, Autosomal dominant | OMIM |
| *ADCY5* | 600293 | *ADCY5, FDFM* | Dyskinesia, familial, with facial myokymia, MIM-606703, Autosomal dominant | OMIM |
| *AFG3L2* | 604581 | *AFG3L2, SCA28, SPAX5* | Spastic ataxia 5, autosomal recessive, MIM-614487, Autosomal recessive; Spinocerebellar ataxia 28, MIM-610246, Autosomal dominant | OMIM |
| *AIMP1* | 603605 | *AIMP1, SCYE1, EMAP2, EMAPII, HLD3* | Leukodystrophy, hypomyelinating, 3, MIM-260600, Autosomal recessive | OMIM |
| *ALDH18A1* | 138250 | *ALDH18A1, PYCS, GSAS, ARCL3A, SPG9A, SPG9B, ADCL3* | Cutis laxa, autosomal dominant 3, MIM-616603, Autosomal dominant; Cutis laxa, autosomal recessive, type IIIA, MIM-219150, Autosomal recessive, Isolated cases; Spastic paraplegia 9A, autosomal dominant, 601162, Autosomal dominant; Spastic paraplegia 9B, autosomal recessive, MIM-616586 , Autosomal recessive | OMIM |
| *ALDH5A1* | 610045 | *ALDH5A1, SSADH* | Succinic semialdehyde dehydrogenase deficiency, MIM-271980, Autosomal recessive | OMIM |
| *ALS2* | 606352 | *ALS2, ALSJ, PLSJ, IAHSP* | Amyotrophic lateral sclerosis 2, juvenile, MIM-205100 Autosomal recessive; Primary lateral sclerosis, juvenile, MIM-606353, Autosomal recessive; Spastic paralysis, infantile onset ascending, MIM-607225, Autosomal recessive | OMIM and SCAIView neuro |
| *AMPD2* | 102771 | *AMPD2, SPG63, PCH9* | Pontocerebellar hypoplasia, type 9, MIM-615809, Autosomal recessive; Spastic paraplegia 63, MIM-615686, Autosomal recessive | OMIM |
| *ANO3* | 610110 | *ANO3, TMEM16C, C11orf25, DYT24* | Dystonia 24, MIM-615034, Autosomal dominant | OMIM and SCAIView neuro |
| *AP4B1* | 607245 | *AP4B1, SPG47, CPSQ5* | Spastic paraplegia 47, MIM-614066, Autosomal recessive | OMIM |
| *APTX* | 606350 | *APTX, AOA, AOA1* | Ataxia, early-onset, with oculomotor apraxia and hypoalbuminemia, MIM-208920, Autosomal recessive | OMIM |
| *ARSA* | 607574 | *ARSA* | Metachromatic leukodystrophy, MIM-250100, Autosomal recessive | OMIM |

| | | | | |
|---|---|---|---|---|
| *ARX* | 300382 | *ARX, ISSX, PRTS, MRXS1, MRX36, MRX54, MRX43, MRX87, MRX29, MRX32* | Epileptic encephalopathy, early infantile, 1, MIM-308350, X-linked recessive; Hydranencephaly with abnormal genitalia, MIM-300215, X-linked; Lissencephaly, X-linked 2, 300215, X-linked; Mental retardation, X-linked 29 and others, 300419, X-linked recessive; Partington syndrome, MIM-309510, X-linked recessive; Proud syndrome, MIM-300004 , X-linked | OMIM |
| *ATM* | 607585 | *ATM, ATA, AT1* | Ataxia-telangiectasia, MIM-208900, Autosomal recessive;  Lymphoma, B-cell non-Hodgkin, somatic; Lymphoma, mantle cell, somatic; T-cell prolymphocytic leukemia, somatic | OMIM |
| *ATP13A2* | 610513 | *ATP13A2, PARK9, KRPPD, SPG78* | Kufor-Rakeb syndrome, MIM-606693, Autosomal recessive; Spastic paraplegia 78, autosomal recessive, MIM-617225, Autosomal recessive | OMIM |
| *ATP1A3* | 182350 | *ATP1A3, DYT12, RDP, AHC2, CAPOS* | Dystonia-12, MIM-128235, Autosomal dominant | OMIM and SCAIView neuro |
| *ATP7B* | 606882 | *ATP7B, WND* | Wilson disease, MIM-277900, Autosomal recessive | OMIM and SCAIView neuro |
| *ATXN2* | 601517 | *ATXN2, ATX2, SCA2, ASL13* | Isolated cases, Multifactorial; Spinocerebellar ataxia 2, MIM-183090, Autosomal dominant | OMIM |
| *AUH* | 617887 | *AGMAT, AUH* | 3-methylglutaconic aciduria, type I, MIM-250950, Autosomal recessive | OMIM |
| *B4GALNT1* | 601873 | *B4GALNT1, GALGT, GALNACT, SPG26* | Spastic paraplegia 26, autosomal recessive, MIM-609195, Autosomal recessive | OMIM |
| *BCAP31* | 300398 | *BCAP31, BAP31, DXS1357E, DDCH* | Deafness, dystonia, and cerebral hypomyelination, MIM-300475, X-linked recessive | OMIM |
| *BSCL2* | 606158 | *BSCL2, SPG17, HMN5, PELD* | Encephalopathy, progressive, with or without lipodystrophy, MIM-615924, Autosomal recessive; Lipodystrophy, congenital generalized, type 2, MIM-69700, Autosomal recessive; Neuropathy, distal hereditary motor, type VA, MIM-600794, Autosomal dominant; Silver spastic paraplegia syndrome, MIM-270685, Autosomal dominant | OMIM |
| *C19orf12* | 614297 | *C19orf12, NBIA4, SPG43* | Neurodegeneration with brain iron accumulation 4, MIM-614298, Autosomal recessive; Spastic paraplegia 43, autosomal recessive, MIM-615043 , Autosomal recessive | OMIM |

| | | | | |
|---|---|---|---|---|
| *CACNA1A* | 601011 | *CACNA1A, CACNL1A4, SCA6, EIEE42* | Epileptic encephalopathy, early infantile, 42, MIM-617106, Autosomal dominant; Episodic ataxia, type 2, MIM-108500, Autosomal dominant; Migraine, familial hemiplegic, 1, MIM-141500, Autosomal dominant; Migraine, familial hemiplegic, 1, with progressive cerebellar ataxia, MIM-141500 , Autosomal dominant; MIM-Spinocerebellar ataxia 6, MIM-183086 , Autosomal dominant | OMIM and SCAIView neuro |
| *CACNA1B* | 601012 | *CACNA1B, CACNL1A5, DYT23* | Dystonia 23, MIM-614860, Autosomal dominant | OMIM and SCAIView neuro |
| *CHMP2B* | 609512 | *CHMP2B, DMT1, VPS2B, ALS17* | Dementia, familial, nonspecific, MIM-600795, Autosomal dominant | OMIM |
| *CIZ1* | 611420 | *CIZ1, ZNF356, LSFR1, NP94* | | OMIM and SCAIView neuro |
| *CLN3* | 607042 | *CLN3, BTS* | Ceroid lipofuscinosis, neuronal, 3, MIM-204200, Autosomal recessive | OMIM |
| *CLN6* | 606725 | *CLN6, CLN4A* | Ceroid lipofuscinosis, neuronal, 6, MIM-601780, Autosomal recessive; Ceroid lipofuscinosis, neuronal, Kufs type, adult onset, MIM-204300, Autosomal recessive | OMIM |
| *COASY* | 609855 | *COASY, NBIA6* | Neurodegeneration with brain iron accumulation 6, MIM-615643, Autosomal recessive | OMIM |
| *COL4A1* | 120130 | *COL4A1, POREN1, HANAC, ICH, BSVD, RATOR* | Porencephaly 1, MIM-175780, Autosomal dominant; | OMIM |
| *COL6A3* | 120250 | *COL6A3, DYT27, BTHLM1, UCMD1* | Bethlem myopathy 1, MIM-158810, Autosomal recessive, Autosomal dominant; Dystonia 27, MIM-616411 , Autosomal recessive; Ullrich congenital muscular dystrophy 1, MIM-254090 , Autosomal recessive, Autosomal dominant | OMIM and SCAIView neuro |
| *COX10* | 602125 | *COX10* | Leigh syndrome due to mitochondrial COX4 deficiency, MIM-256000, Autosomal recessive, Mitochondrial; Mitochondrial complex IV deficiency, MIM-220110, Autosomal recessive, Mitochondrial | OMIM |
| *COX15* | 603646 | *COX15, CEMCOX2* | Cardioencephalomyopathy, fatal infantile, due to cytochrome c oxidase deficiency 2, MIM-615119, Autosomal recessive; Leigh syndrome due to cytochrome c oxidase deficiency, MIM-256000, Autosomal recessive, Mitochondrial | OMIM |
| *COX20* | 614698 | *COX20, FAM36A* | Mitochondrial complex IV deficiency, MIM-220110, Autosomal recessive, Mitochondrial | OMIM |
| *CTC1* | 613129 | *CTC1, CRMCC, C17orf68, AAF132* | Cerebroretinal microangiopathy with calcifications and cysts, MIM-612199, Autosomal recessive | OMIM |
| *DCAF17* | 612515 | *DCAF17, C20orf37* | Woodhouse-Sakati syndrome, MIM-241080, Autosomal recessive | OMIM |

| | | | | |
|---|---|---|---|---|
| *DDC* | 107930 | *DDC* | Aromatic L-amino acid decarboxylase deficiency, MIM-608643, Autosomal recessive | OMIM |
| *DMXL2* | 612186 | *DMXL2, RC3, KIAA0856, PEPNS, DFNA71* | Polyendocrine-polyneuropathy syndrome, MIM-616113, Autosomal recessive | OMIM |
| *DNAJC12* | 606060 | *DNAJC12, JDP1, HPANBH4* | Hyperphenylalaninemia, mild, non-BH4-deficient, MIM-617384, Autosomal recessive | OMIM |
| *DNAJC6* | 608375 | *DNAJC6, DJC6, KIAA0473, PARK19* | Parkinson disease 19a, juvenile-onset, MIM-615528, Autosomal recessive; Parkinson disease 19b, early-onset, MIM-615528, Autosomal recessive | OMIM |
| *EARS2* | 612799 | *EARS2, KIAA1970, COXPD12* | Combined oxidative phosphorylation deficiency 12, MIM-614924, Autosomal recessive | OMIM |
| *FA2H* | 611026 | *FA2H, FAAH, FAXDC1, FAH1, SCS7, SPG35* | Spastic paraplegia 35, autosomal recessive, MIM-612319, Autosomal recessive | OMIM |
| *FARS2* | 611592 | *FARS2, FARS1, COXPD14, SPG77* | Combined oxidative phosphorylation deficiency 14, MIM-614946, Autosomal recessive; Spastic paraplegia 77, autosomal recessive, MIM-617046, Autosomal recessive | OMIM |
| *FBXO7* | 605648 | *FBXO7, FBX7, FBX, PKPS, PARK15* | Parkinson disease 15, autosomal recessive, MIM-260300, Autosomal recessive | OMIM |
| *FOXRED1* | 613622 | *FOXRED1* | Leigh syndrome due to mitochondrial complex I deficiency, MIM-256000, Autosomal recessive, Mitochondrial; Mitochondrial complex I deficiency, MIM-252010, Autosomal recessive, X-linked dominant, Mitochondrial | OMIM |
| *FTL* | 134790 | *FTL, NBIA3, LFTD* | Hyperferritinemia-cataract syndrome, MIM-600886, Autosomal dominant; L-ferritin deficiency, dominant and recessive, MIM-615604, Autosomal recessive, Autosomal dominant; Neurodegeneration with brain iron accumulation 3, MIM-606159, Autosomal dominant | OMIM |
| *FUCA1* | 612280 | *FUCA1* | Fucosidosis, MIM-230000, Autosomal recessive | OMIM |
| *GCDH* | 608801 | *GCDH* | Glutaricaciduria, type I, MIM-231670, Autosomal recessive | OMIM |
| *GCH1* | 600225 | *GCH1, DYT5, HPABH4B* | Dystonia, DOPA-responsive, with or without hyperphenylalaninemia, MIM-128230, Autosomal recessive, Autosomal dominant; Hyperphenylalaninemia, BH4-deficient, B, 233910, Autosomal recessive | OMIM and SCAIView neuro |
| *GJC2* | 608803 | *GJC2, GJA12, CX47, PMLDAR, HLD2, SPG44, LMPH1C* | Leukodystrophy, hypomyelinating, 2, MIM-608804, Autosomal recessive; Lymphedema, hereditary, IC, MIM-613480, Autosomal dominant; Spastic paraplegia 44, autosomal recessive, MIM-613206, Autosomal recessive | OMIM and SCAIView neuro |
| *GLB1* | 611458 | *GLB1, MPS4B* | GM1-gangliosidosis, type III, MIM-230650, Autosomal recessive | OMIM and SCAIView neuro |

| | | | | |
|---|---|---|---|---|
| *GNAL* | 139312 | *GNAL, DYT25* | Dystonia 25, MIM-615073, Autosomal dominant | OMIM and SCAIView neuro |
| *GNAO1* | 139311 | *GNAO1, EIEE17, NEDIM* | | OMIM |
| *GOSR2* | 604027 | *GOSR2, GS27, EPM6* | Epilepsy, progressive myoclonic 6, MIM-614018, Autosomal recessive | OMIM |
| *HEXA* | 606869 | *HEXA, TSD* | GM2-gangliosidosis, several forms, MIM-272800, Autosomal recessive; [Hex A pseudodeficiency], MIM-272800, Autosomal recessive; Tay-Sachs disease, MIM-272800, Autosomal recessive | OMIM |
| *HPCA* | 142622 | *HPCA, DYT2* | Dystonia 2, torsion, autosomal recessive, MIM-224500, Autosomal recessive | OMIM and SCAIView neuro |
| *HPRT1* | 308000 | *HPRT1, HPRT* | Lesch-Nyhan syndrome, MIM-300322, X-linked recessive | OMIM |
| *HTT* | 182138 | *SLC6A4, HTT, OCD1* | Huntington disease, MIM-143100, Autosomal dominant; Lopes-Maciel-Rodan syndrome, MIM-617435, Autosomal recessive | OMIM and SCAIView neuro |
| *IFIH1* | 606951 | *IFIH1, MDA5, AGS7, SGMRT1* | Aicardi-Goutieres syndrome 7, MIM-615846, Autosomal dominant; Singleton-Merten syndrome 1, MIM-182250, Autosomal dominant | OMIM |
| *JPH3* | 605268 | *JPH3, JP3, HDL2* | Huntington disease-like 2, MIM-606438, Autosomal dominant | OMIM |
| *KCNMA1* | 600150 | *KCNMA1, SLO, PNKD3, CADEDS* | Cerebellar atrophy, developmental delay, and seizures, MIM-617643, Autosomal recessive; Paroxysmal nonkinesigenic dyskinesia, 3, with or without generalized epilepsy, MIM-609446 Autosomal dominant | OMIM |
| *KIF1C* | 603060 | *KIF1C, LTXS1, KIAA0706, SPAX2* | Spastic ataxia 2, autosomal recessive, MIM-611302, Autosomal recessive | OMIM |
| *KMT2B* | 606834 | *KMT2B, MLL4, KIAA0304, DYT28* | Dystonia 28, childhood-onset, MIM-617284, Autosomal dominant | OMIM |
| *L2HGDH* | 609584 | *L2HGDH, C14orf160, L2HGA* | L-2-hydroxyglutaric aciduria, MIM-236792, Autosomal recessive | OMIM |
| *LRRK2* | 609007 | *LRRK2, PARK8* | Parkinson disease 8, MIM-607060, Autosomal dominant | SCAIView neuro |
| *LYRM7* | 615831 | *LYRM7, MZM1L, MC3DN8* | Mitochondrial complex III deficiency, nuclear type 8, MIM-615838, Autosomal recessive | OMIM |
| *MAPT* | 157140 | *MAPT, MTBT1, DDPAC, MSTD* | Dementia, frontotemporal, with or without parkinsonism, MIM-600274, Autosomal dominant; Parkinson disease, susceptibility to}, MIM-168600, Isolated cases, Multifactorial; Pick disease, MIM-172700, Autosomal dominant, Isolated cases; Supranuclear palsy, progressive, MIM-601104, Autosomal dominant; Supranuclear palsy, progressive atypical, 260540, Autosomal recessive | OMIM and SCAIView neuro |
| *MARS2* | 609728 | *MARS2, SPAX3, COXPD25* | Spastic ataxia 3, autosomal recessive, MIM-611390, Autosomal recessive | OMIM |

| | | | | |
|---|---|---|---|---|
| *MCCC1* | 609010 | *MCCC1, MCCA* | 3-Methylcrotonyl-CoA carboxylase 1 deficiency, MIM-210200, Autosomal recessive | OMIM |
| *MCOLN1* | 605248 | *MCOLN1, ML4* | Mucolipidosis IV, MIM-252650, Autosomal recessive | OMIM |
| *MECP2* | 300005 | *MECP2, RTT, PPMX, MRX16, MRX79, AUTSX3, MRXSL, MRXS13* | Rett syndrome, X linked autosomal recessive, MIM-312750 | OMIM |
| *MECR* | 608205 | *MECR, NRBF1, DYTOABG* | Dystonia, childhood-onset, with optic atrophy and basal ganglia abnormalities, MIM-617282, Autosomal recessive | OMIM |
| *MED20* | 612915 | *MED20, TRFP* | Infantile-onset basal ganglia degeneration and brain atrophy | OMIM |
| *MPV17* | 616133 | *MPV17L2* | | OMIM |
| *MRE11A* | 600814 | *MRE11A, MRE11, ATLD* | Ataxia-telangiectasia-like disorder 1, MIM-604391, Autosomal recessive | OMIM |
| *NDUFA11* | 612638 | *NDUFA11* | Mitochondrial complex I deficiency, MIM-252010, Autosomal recessive, X-linked dominant, Mitochondrial | OMIM |
| *NDUFA9* | 603834 | *NDUFA9* | Leigh syndrome due to mitochondrial complex I deficiency, MIM-256000, Autosomal recessive, Mitochondrial | OMIM |
| *NDUFAF1* | 606934 | *NDUFAF1, CIA30, CGI65* | Mitochondrial complex I deficiency, MIM-252010, Autosomal recessive, X-linked dominant, Mitochondrial | OMIM |
| *NDUFAF5* | 612360 | *NDUFAF5, C20orf7* | Mitochondrial complex 1 deficiency, MIM-252010, Autosomal recessive, X-linked dominant, Mitochondrial | OMIM |
| *NDUFS1* | 157655 | *NDUFS1* | Mitochondrial complex I deficiency, MIM-252010, Autosomal recessive, X-linked dominant, Mitochondrial | OMIM |
| *NDUFS4* | 602694 | *NDUFS4, AQDQ* | Leigh syndrome, MIM-256000, Autosomal recessive, Mitochondrial; Mitochondrial complex I deficiency, MIM-252010, Autosomal recessive, X-linked dominant, Mitochondrial | OMIM |
| *NDUFS8* | 602141 | *NDUFS8* | Leigh syndrome due to mitochondrial complex I deficiency, MIM-256000, Autosomal recessive, Mitochondrial | OMIM |
| *NDUFV1* | 161015 | *NDUFV1, UQOR1* | Mitochondrial complex I deficiency, MIM-252010, Autosomal recessive, X-linked dominant, Mitochondrial | OMIM |
| *NKX2-1* | 600635 | *NKX2-1, TITF1, NKX2A, TTF1, NMTC1* | Choreoathetosis, hypothyroidism, and neonatal respiratory distress | OMIM and SCAIView neuro |
| *NKX6-2* | 605955 | *NKX6-2, NKX6B, NKX6.2, SPAX8* | Spastic ataxia 8, autosomal recessive, with hypomyelinating leukodystrophy, MIM-617560, Autosomal recessive | OMIM |
| *NPC1* | 607623 | *NPC1, NPC* | Niemann-Pick disease, type C1, MIM-257220, Autosomal recessive; Niemann-Pick disease, type D, MIM-257220, Autosomal recessive | OMIM |
| *NPC2* | 601015 | *NPC2, HE1* | Niemann-pick disease, type C2, MIM-607625, Autosomal recessive | OMIM |
| *NUBPL* | 613621 | *NUBPL, IND1* | Mitochondrial complex I deficiency, MIM-252010, Autosomal recessive, X-linked dominant | OMIM |

| | | | | |
|---|---|---|---|---|
| *NUP62* | 605815 | *NUP62, SNDI, IBSN* | Striatonigral degeneration, infantile, MIM-271930, Autosomal recessive | OMIM |
| *OBFC1* | 613128 | *STN1, OBFC1, AAF44* | Cerebroretinal microangiopathy with calcifications and cysts 2, MIM-617341, Autosomal recessive | OMIM |
| *PANK2* | 606157 | *PANK2, NBIA1, PKAN, HARP* | HARP syndrome, MIM-607236, Autosomal recessive; Neurodegeneration with brain iron accumulation 1, MIM-234200, Autosomal recessive | OMIM and SCAIView neuro |
| *PARK2* | 602544 | *PRKN, PARK2, PDJ, LPRS2* | Adenocarcinoma of lung, somatic, MIM-211980; Adenocarcinoma, ovarian, somatic, MIM-167000; MIM-607572; Parkinson disease, juvenile, type 2, MIM-600116, Autosomal recessive | OMIM and SCAIView neuro |
| *PDGFB* | 190040 | *PDGFB, SIS, IBGC5* | Basal ganglia calcification, idiopathic, 5, MIM-615483, Autosomal dominant; Dermatofibrosarcoma protuberans, MIM-607907; Meningioma, SIS-related, MIM-607174, Autosomal dominant | OMIM |
| *PDHX* | 608769 | *PDHX, PDX1, E3BP, PDHXD* | Lacticacidemia due to PDX1 deficiency, MIM-245349, Autosomal recessive | OMIM |
| *PINK1* | 608309 | *PINK1, PARK6* | Parkinson disease 6, early onset, MIM-605909 Autosomal recessive | OMIM |
| *PLA2G6* | 603604 | *PLA2G6, IPLA2, INAD1, NBIA2B, NBIA2A, PARK14* | Infantile neuroaxonal dystrophy 1, MIM-256600, Autosomal recessive; Neurodegeneration with brain iron accumulation 2B, MIM-610217, Autosomal recessive; Parkinson disease 14, autosomal recessive, MIM-612953, Autosomal recessive | OMIM |
| *PLEKHG2* | 611893 | *PLEKHG2, CLG, LDAMD* | Leukodystrophy and acquired microcephaly with or without dystonia, MIM-616763, Autosomal recessive | OMIM |
| *PLP1* | 300401 | *PLP1, PMD, HLD1, SPG2* | Pelizaeus-Merzbacher disease, MIM-312080, X-linked recessive; Spastic paraplegia 2, X-linked, MIM-312920, X-linked recessive | OMIM |
| *PNKP* | 605610 | *PNKP, PNK, MCSZ, EIEE10, AOA4* | Ataxia-oculomotor apraxia 4, MIM-616267, Autosomal recessive; Microcephaly, seizures, and developmental delay, MIM-613402, Autosomal recessive | OMIM |
| *POLR3A* | 614258 | *POLR3A, RPC1, RPC155, ADDH, HLD7* | Leukodystrophy, hypomyelinating, 7, with or without oligodontia | OMIM |
| *POLR3B* | 614366 | *POLR3B, RPC2, C128, HLD8* | Leukodystrophy, hypomyelinating, 8, with or without oligodontia | OMIM |
| *PPP2R2B* | 604325 | *PPP2R2B* | Spinocerebellar ataxia 12, MIM-604326, Autosomal dominant | OMIM |
| *PRKCG* | 176980 | *PRKCG, PKCC, PKCG, SCA14* | Spinocerebellar ataxia 14, MIM-605361, Autosomal dominant | OMIM |
| *PRKRA* | 603424 | *PRKRA, PACT, RAX, DYT16* | Dystonia 16, MIM-612067, Autosomal recessive | OMIM and SCAIView neuro |

| | | | | |
|---|---|---|---|---|
| *PRNP* | | *PRNP, PRIP, KURU, CJD* | Cerebral amyloid angiopathy, PRNP-related, MIM-137440, Autosomal dominant; Creutzfeldt-Jakob disease, MIM-123400, Autosomal dominant; Gerstmann-Straussler disease, MIM-137440 , Autosomal dominant; Huntington disease-like 1, MIM-603218 , Autosomal dominant; Insomnia, fatal familial, MIM-600072 , Autosomal dominant; Kuru, susceptibility to}, MIM-245300 ; Prion disease with protracted course, MIM-606688 , Autosomal dominant | SCAIView neuro |
| *PRRT2* | 614386 | *PRRT2, PKC, DYT10, EKD1, BFIS2, BFIC2, ICCA* | Convulsions, familial infantile, with paroxysmal choreoathetosis, MIM-602066, Autosomal dominant; Episodic kinesigenic dyskinesia 1, MIM-128200, Autosomal dominant; Seizures, benign familial infantile, 2, MIM-605751, Autosomal dominant | OMIM and SCAIView neuro |
| *PSEN1* | 104311 | *PSEN1, AD3, ACNINV3* | Acne inversa, familial, 3, MIM-613737, Autosomal dominant; Alzheimer disease, type 3, MIM-607822, Autosomal dominant; Alzheimer disease, type 3, with spastic paraparesis and apraxia, MIM-607822, Autosomal dominant; Alzheimer disease, type 3, with spastic paraparesis and unusual plaques, MIM-607822, Autosomal dominant; Cardiomyopathy, dilated, 1U, MIM-613694, Autosomal dominant; Dementia, frontotemporal, MIM-600274 , Autosomal dominant; Pick disease, MIM-172700 , Autosomal dominant, Isolated cases | OMIM |
| *QDPR* | 612676 | *QDPR, DHPR* | Hyperphenylalaninemia, BH4-deficient, C, MIM-261630, Autosomal recessive | OMIM |
| *RAB39B* | 300774 | *RAB39B, MRX72, WSMN* | Mental retardation, X-linked 72, 300271, X-linked recessive; Waisman syndrome, MIM-311510, X-linked recessive | OMIM |
| *RELN* | 600514 | *RELN, RL, LIS2, ETL7* | Epilepsy, familial temporal lobe, 7, MIM-616436, Autosomal dominant; Lissencephaly 2 (Norman-Roberts type), MIM-257320, Autosomal recessive | SCAIView neuro |
| *REPS1* | 614825 | *REPS1, NBIA7* | Neurodegeneration with brain iron accumulation 7, MIM-617916, Autosomal recessive | OMIM |
| *RNASEH2B* | 610326 | *RNASEH2B, DLEU8, FLJ11712, AGS2* | Aicardi-Goutieres syndrome 2, MIM-610181, Autosomal recessive | OMIM |
| *RNASEH2C* | 610330 | *RNASEH2C, AYP1, FLJ20974, AGS3* | Aicardi-Goutieres syndrome 3, MIM-610329, Autosomal recessive | OMIM |
| *RNASET2* | 612944 | *RNASET2, RNASE6PL* | Leukoencephalopathy, cystic, without megalencephaly, MIM-612951, Autosomal recessive | OMIM |
| *SCP2* | 604105 | *SYCP2, SCP2* | Leukoencephalopathy with dystonia and motor neuropathy, MIM-613724, Autosomal recessive | OMIM |

| | | | | |
|---|---|---|---|---|
| *SDHA* | 600857 | *SDHA, SDH1, SDHF, CMD1GG, PGL5* | Cardiomyopathy, dilated, 1GG, 613642; Leigh syndrome, MIM-256000, Autosomal recessive, Mitochondrial; Mitochondrial respiratory chain complex II deficiency, MIM-252011, Autosomal recessive; Paragangliomas 5, MIM-614165, Autosomal dominant | OMIM |
| *SDHAF1* | 612848 | *SDHAF1* | Mitochondrial complex II deficiency, MIM-252011, Autosomal recessive | OMIM |
| *SETX* | 608465 | *SETX, SCAR1, AOA2, ALS4* | Spinocerebellar ataxia, autosomal recessive 1, MIM-606002, Autosomal recessive | OMIM |
| *SGCE* | 604149 | *SGCE, DYT11* | Dystonia-11, myoclonic, MIM-15990, Autosomal dominant | OMIM and SCAIView neuro |
| *SLC16A2* | 300095 | *SLC16A2, DXS128, XPCT* | Allan-Herndon-Dudley syndrome, MIM-300523, X-linked | OMIM |
| *SLC19A3* | 606152 | *SLC19A3, THMD2, BBGD* | Thiamine metabolism dysfunction syndrome 2 (biotin- or thiamine-responsive encephalopathy type 2), MIM-607483, Autosomal recessive | OMIM |
| *SLC20A2* | 158378 | *SLC20A2, MLVAR, GLVR2, IBGC1* | Basal ganglia calcification, idiopathic, 1, MIM-213600, Autosomal dominant | OMIM and SCAIView neuro |
| *SLC2A1* | 138140 | *SLC2A1, GLUT1, HTLVR, DYT18, PED, GLUT1DS, EIG12, DYT9, SDCHCN* | Dystonia 9, MIM-601042 Autosomal dominant; Epilepsy, idiopathic generalized, MIM-614847, Autosomal dominant; GLUT1 deficiency syndrome 1, infantile onset, severe, MIM-606777, Autosomal recessive, Autosomal dominant; GLUT1 deficiency syndrome 2, childhood onset, MIM-612126, Autosomal dominant; Stomatin-deficient cryohydrocytosis with neurologic defects, MIM-608885, Autosomal dominant | OMIM and SCAIView neuro |
| *SLC30A10* | 611146 | *SLC30A10, ZNT10, HMNDYT1* | Hypermanganesemia with dystonia 1, MIM-613280, Autosomal recessive | OMIM |
| *SLC39A14* | 608736 | *SLC39A14, ZIP14, KIAA0062, HMNDYT2, HCIN* | Hypermanganesemia with dystonia 2, MIM-617013, Autosomal recessive; Hyperostosis cranalis interna, MIM-144755 Autosomal dominant | OMIM |
| *SLC6A3* | 126455 | *SLC6A3, DAT1, PKDYS1* | Parkinsonism-dystonia, infantile, 1, MIM-613135, Autosomal recessive | OMIM and SCAIView neuro |
| *SNCA* | 163890 | *SNCA, NACP, PARK1, PARK4* | Parkinson disease 1, MIM-168601, Autosomal dominant; Parkinson disease 4, MIM-605543, Autosomal dominant | OMIM and SCAIView neuro |
| *SNORD118* | 616663 | *SNORD118, LCC* | Leukoencephalopathy, brain calcifications, and cysts, MIM-614561, Autosomal recessive | OMIM |
| *SPR* | 182125 | *SPR* | Dystonia, dopa-responsive, due to sepiapterin reductase deficiency, MIM-612716, Autosomal dominant, Autosomal recessive | OMIM |
| *SQSTM1* | 601530 | *SQSTM1, P62, PDB3, FTDALS3, NADGP, DMRV* | Neurodegeneration with ataxia, dystonia, and gaze palsy, childhood-onset, MIM-617145 | OMIM |

| | | | | |
|---|---|---|---|---|
| *SUCLG1* | 611224 | *SUCLG1, SUCLA1, MTDPS9* | Mitochondrial DNA depletion syndrome 9 (encephalomyopathic type with methylmalonic aciduria), MIM-245400, Autosomal recessive | OMIM |
| *SUOX* | 606887 | *SUOX* | Sulfite oxidase deficiency, MIM-272300, Autosomal recessive | OMIM |
| *SURF1* | 185620 | *SURF1, CMT4K* | Charcot-Marie-Tooth disease, type 4K, MIM-616684, Autosomal recessive; Leigh syndrome, due to COX IV deficiency, MIM-256000, Autosomal recessive, Mitochondrial | OMIM |
| *SYNJ1* | 604297 | *SYNJ1, PARK20, EIEE53* | Epileptic encephalopathy, early infantile, 53, MIM-617389, Autosomal recessive; Parkinson disease 20, early-onset, MIM-615530, Autosomal recessive | OMIM |
| *TAF1* | 313650 | *TAF1, TAF2A, CCG1, BA2R, DYT3, MRXS33* | Dystonia-Parkinsonism, X-linked, MIM-314250, X-linked recessive; Mental retardation, X-linked, syndromic 33, MIM-300966 X-linked recessive | OMIM and SCAIView neuro |
| *TBP* | 600075 | *TBP, SCA17, HDL4* | Parkinson disease, susceptibility, MIM-168600, Isolated cases, Multifactorial; Spinocerebellar ataxia 17, MIM-607136, Autosomal dominant | OMIM |
| *TH* | 191290 | *TH, TYH* | Segawa syndrome, recessive, MIM-605407, Autosomal recessive | OMIM and SCAIView neuro |
| *THAP1* | 609520 | *THAP1, DYT6* | Dystonia 6, torsion, MIM-602629, Autosomal dominant | OMIM and SCAIView neuro |
| *TOR1A* | 605204 | *DYT1, TOR1A* | Dystonia-1; Dystonia-1, torsion, MIM-128100, Autosomal dominant | OMIM and SCAIView neuro |
| *TRAPPC11* | 614138 | *TRAPPC11, C4orf41, LGMD2S* | Muscular dystrophy, limb-girdle, type 2S, MIM-615356, Autosomal recessive | OMIM |
| *TREX1* | 606609 | *TREX1, AGS1, CRV, HERNS* | Aicardi-Goutieres syndrome 1, dominant and recessive, MIM-225750, Autosomal recessive, Autosomal dominant; Chilblain lupus, MIM-610448, Autosomal dominant, 152700, Autosomal dominant; Vasculopathy, retinal, with cerebral leukodystrophy, MIM-192315, Autosomal dominant | OMIM |
| *TRPM7* | 605692 | *TRPM7, LTRPC7, CHAK, ALSPDC* | Amyotrophic lateral sclerosis-parkinsonism | OMIM |
| *TSEN2* | 608753 | *TSEN2, SEN2, PCH2B* | Pontocerebellar hypoplasia type 2B, MIM-612389, Autosomal recessive | OMIM |
| *TSEN54* | 608755 | *TSEN54, SEN54, PCH2A, PCH4, PCH5* | Pontocerebellar hypoplasia type 2A, MIM-277470, Autosomal recessive; Pontocerebellar hypoplasia type 4, MIM-225753, Autosomal recessive; Pontocerebellar hypoplasia type 5, MIM-610204, Autosomal recessive | OMIM |
| *TSFM* | 604723 | *TSFM, COXPD3* | Combined oxidative phosphorylation deficiency 3, MIM-610505, Autosomal recessive | OMIM |
| *TTC19* | 613814 | *TTC19, MC3DN2* | Mitochondrial complex III deficiency, nuclear type 2, MIM-615157, Autosomal recessive | OMIM |

| | | | | |
|---|---|---|---|---|
| *TTPA* | 600415 | *TTPA, TTP1, AVED* | Ataxia with isolated vitamin E deficiency, MIM-277460, Autosomal recessive | OMIM |
| *TUBB4A* | 602662 | *TUBB4A, DYT4, HLD6* | Dystonia 4, torsion, autosomal dominant, MIM-128101, Autosomal dominant; Leukodystrophy, hypomyelinating, 6, MIM-612438, Autosomal dominant | OMIM and SCAIView neuro |
| *VAC14* | 604632 | *VAC14, TAX1BP2, TRX, SNDC* | Striatonigral degeneration, childhood-onset, MIM-617054, Autosomal recessive | OMIM |
| *VAMP1* | 185880 | *VAMP1, SYB1, SPAX1* | Spastic ataxia 1, autosomal dominant, MIM-108600, Autosomal dominant | OMIM |
| *VPS13A* | 605978 | *VPS13A, CHAC* | Choreoacanthocytosis, MIM-200150, Autosomal recessive | OMIM |
| *VPS13C* | 608879 | *VPS13C, KIAA1421, PARK23* | Parkinson disease 23, autosomal recessive, early onset, MIM-616840, Autosomal recessive | OMIM |
| *VPS35* | 601501 | *VPS35, MEM3, PARK17* | Parkinson disease 17}, MIM-614203, Autosomal dominant | OMIM |
| *VPS37A* | 609927 | *VPS37A, HCRP1, SPG53* | Spastic paraplegia 53, autosomal recessive, MIM-614898, Autosomal recessive | OMIM |
| *WDR45* | 300526 | *WDR45, WIPI4, NBIA5* | Neurodegeneration with brain iron accumulation 5, MIM-300894, X-linked dominant | OMIM |
| *XPR1* | 605237 | *XPR1, SYG1, IBGC6* | Basal ganglia calcification, idiopathic, 6, MIM-616413, Autosomal dominant | OMIM |
