# Supplementary material for: Dissecting genetic architecture of rare dystonia: genetic, molecular and clinical insights
Source: J Med Genet. 2024 Mar 8;61(5):443–51. doi: 10.1136/jmg-2022-109099 (PMC11041572; doi:10.1136/jmg-2022-109099)
Supplement: Supplementary data [file jmg-2022-109099supp003.pdf]

WGCNA Statistical Results								
**Type**	**Database**	**Brain Region**	**Module**	**Fisher´s Exact test p value**	**FDR**	**Bonferroni**	**Size of the module**	**Candidate Genes**
**Discovery**	**Gtex V7**	**Basal ganglia**	Black	0.04716	0.80	0.71	213	PNP
			Blue	1	1	1	1199	
			Brown	0.07587	1	1	604	
			Cyan	0.2482	1	1	104	
			Green	0.7731	1	1	335	
			Greenyellow	0.5656	1	1	90	
			Grey	0.1841	1	1	22	
			Magenta	0.7265	1	1	221	
			Midnightblue	0.5007	1	1	75	
			Pink	0.531	1	1	297	
			Red	1	1	1	372	
			Turquoise	2.13E-09	3.62E-08	3.62E-08	8883	ANGEL1 CCNT1 CEP120 DZIP3 MCM4 PDF PRDM15 TBC1D32 TBC1D8
			Yellow	0.1823	1	1	584	
**Discovery**	**Gtex V7**	**Cerrebellum**	Black	0.1281	1	1	339	
			Blue	0.03991	1	0.99775	1069	
			Brown	0.4105	1	1	1115	
			Cyan	0.02728	0.73656	0.70928	129	
			Darkgreen	0.272	1	1	116	
			Darkgrey	0.6547	1	1	161	
			Darkred	1	1	1	121	
			Green	0.7793	1	1	389	
			Greenyellow	1	1	1	91	
			Grey60	0.3497	1	1	140	
			Lightgreen	1	1	1	120	
			Lightyellow	1	1	1	72	
			Magenta	0.4579	1	1	229	
			Orange	1	1	1	148	
			Pink	1	1	1	655	
			Purple	0.4247	1	1	421	
			Red	1	1	1	351	
			Royalblue	1	1	1	194	
			Tan	0.3687	1	1	146	
			Turquoise	0.000829	0.022383	0.022383	942	
			Yellow	0.1783	1	1	832	
**Replication**	**10UKBEC**	**Putamen**	Bisque4	0.09845	1	1	14	
			Blue	0.136	0.29568	0.27456	173	
			Brown	0.3754	1	1	178	
			Cyan	0.359	1	1	60	
			Darkorange	0.09175	1	1	13	
			Darkred	0.0782	1	1	11	
			Green	4.36E-07	0.237496	0.229014	93	
			Magenta	0.002348	1	1	36	
			Pink	0.06625	1	1	57	
			Salmon	0.1566	1	1	23	
			Turquoise	1	1	1	192	
