# Supplementary material for: Dissecting genetic architecture of rare dystonia: genetic, molecular and clinical insights
Source: J Med Genet. 2024 Mar 8;61(5):443–51. doi: 10.1136/jmg-2022-109099 (PMC11041572; doi:10.1136/jmg-2022-109099)
Supplement: Supplementary data [file jmg-2022-109099supp004.pdf]

CEN Genes in the BG region with Module Membership Values

Ensembl Gene id	Gene Symbol	Module	Module Membership Value
ENSG00000121410.7	A1BG	turquoise	0.773975391
ENSG00000268895.1	A1BG-AS1	turquoise	0.872910208
ENSG00000175899.10	A2M	black	0.604913015
ENSG00000245105.1	A2M-AS1	blue	0.699725377
ENSG00000166535.15	A2ML1	green	0.742881637
ENSG00000128274.11	A4GALT	black	0.831728998
ENSG00000094914.8	AAAS	yellow	0.891337637
ENSG00000081760.12	AACS	turquoise	0.950177677
ENSG00000109576.9	AADAT	blue	0.813374694
ENSG00000158122.7	AAED1	turquoise	0.869470196
ENSG00000103591.8	AAGAB	turquoise	0.966345568
ENSG00000115977.14	AAK1	turquoise	0.943982305
ENSG00000087884.10	AAMDC	brown	0.767538598
ENSG00000127837.5	AAMP	turquoise	0.952596835
ENSG00000131043.7	AAR2	turquoise	0.919738689
ENSG00000205002.3	AARD	turquoise	0.792574304
ENSG00000090861.11	AARS	turquoise	0.962519563
ENSG00000124608.4	AARS2	turquoise	0.931426185
ENSG00000266967.2	AARSD1	turquoise	0.952050045
ENSG00000157426.9	AASDH	blue	0.907266224
ENSG00000149313.6	AASDHPPT	turquoise	0.945637559
ENSG00000008311.10	AASS	blue	0.828842846
ENSG00000108270.6	AATF	turquoise	0.824020892
ENSG00000181409.7	AATK	brown	0.919575544
ENSG00000183044.7	ABAT	turquoise	0.925163004
ENSG00000226210.3	ABC7-42389800N1	turquoise	0.520931474
ENSG00000165029.11	ABCA1	green	0.803707093
ENSG00000251595.3	ABCA11P	turquoise	0.837554191
ENSG00000238098.4	ABCA17P	turquoise	0.804270535
ENSG00000107331.12	ABCA2	brown	0.934501136
ENSG00000167972.9	ABCA3	turquoise	0.94693487
ENSG00000154265.11	ABCA5	turquoise	0.914990269
ENSG00000064687.8	ABCA7	yellow	0.875886498
ENSG00000141338.9	ABCA8	brown	0.852825603
ENSG00000154258.12	ABCA9	brown	0.649379376
ENSG00000085563.10	ABCB1	black	0.578569734
ENSG00000135776.4	ABCB10	turquoise	0.925551425
ENSG00000115657.8	ABCB6	turquoise	0.898837762
ENSG00000131269.11	ABCB7	turquoise	0.875107825
ENSG00000197150.8	ABCB8	turquoise	0.953555417
ENSG00000150967.13	ABCB9	turquoise	0.906820899
ENSG00000103222.14	ABCC1	pink	0.880030506
ENSG00000124574.10	ABCC10	yellow	0.964740975
ENSG00000125257.9	ABCC4	magenta	0.819037928
ENSG00000114770.12	ABCC5	turquoise	0.933184302

ENSG00000006071.7	ABCC8	turquoise	0.850615098
ENSG00000069431.6	ABCC9	green	0.68052737
ENSG00000101986.8	ABCD1	blue	0.734761515
ENSG00000173208.3	ABCD2	green	0.700977349
ENSG00000117528.7	ABCD3	blue	0.894665017
ENSG00000119688.16	ABCD4	turquoise	0.874680665
ENSG00000164163.6	ABCE1	turquoise	0.940840369
ENSG00000204574.8	ABCF1	turquoise	0.933569814
ENSG00000033050.3	ABCF2	turquoise	0.958096706
ENSG00000161204.7	ABCF3	turquoise	0.971383934
ENSG00000160179.14	ABCG1	turquoise	0.815896372
ENSG00000118777.6	ABCG2	brown	0.467020233
ENSG00000172350.5	ABCG4	turquoise	0.905342549
ENSG00000143994.9	ABHD1	green	0.671312427
ENSG00000144827.4	ABHD10	turquoise	0.942744563
ENSG00000106077.14	ABHD11	turquoise	0.818203731
ENSG00000100997.14	ABHD12	turquoise	0.936888015
ENSG00000131969.10	ABHD12B	midnightblue	0.883425716
ENSG00000139826.5	ABHD13	turquoise	0.932311215
ENSG00000248487.4	ABHD14A	turquoise	0.898391051
ENSG00000114779.15	ABHD14B	turquoise	0.713066878
ENSG00000168792.4	ABHD15	blue	0.776784613
ENSG00000204427.7	ABHD16A	turquoise	0.95962576
ENSG00000183260.5	ABHD16B	turquoise	0.776012309
ENSG00000129968.11	ABHD17A	turquoise	0.786070631
ENSG00000107362.9	ABHD17B	brown	0.927933123
ENSG00000136379.7	ABHD17C	turquoise	0.904082859
ENSG00000158201.5	ABHD3	cyan	0.82799738
ENSG00000100439.6	ABHD4	blue	0.723652914
ENSG00000011198.3	ABHD5	green	0.784516612
ENSG00000163686.9	ABHD6	brown	0.886589802
ENSG00000127220.5	ABHD8	turquoise	0.869932305
ENSG00000136754.12	ABI1	blue	0.891553392
ENSG00000138443.11	ABI2	turquoise	0.957220146
ENSG00000108798.4	ABI3	magenta	0.922469925
ENSG00000097007.13	ABL1	brown	0.791944039
ENSG00000143322.15	ABL2	turquoise	0.969204582
ENSG00000099204.14	ABLIM1	blue	0.837578771
ENSG00000163995.14	ABLIM2	turquoise	0.901453489
ENSG00000173210.15	ABLIM3	turquoise	0.844724458
ENSG00000159842.10	ABR	turquoise	0.922362614
ENSG00000146386.7	ABRACL	turquoise	0.921281331
ENSG00000146109.3	ABT1	turquoise	0.957228492
ENSG00000114626.13	ABTB1	turquoise	0.900606384
ENSG00000166016.4	ABTB2	blue	0.763168728
ENSG00000273300.1	AC000068.9	yellow	0.81131722
ENSG00000224138.1	AC000123.4	blue	0.652949963
ENSG00000184319.11	AC002055.4	turquoise	0.819357325

ENSG00000273216.1	AC002059.10	turquoise	0.431934568
ENSG00000267439.1	AC002398.11	red	0.706175807
ENSG00000267049.1	AC002398.13	yellow	0.755402178
ENSG00000236197.2	AC002429.5	green	0.603837734
ENSG00000237819.1	AC002454.1	turquoise	0.653616661
ENSG00000223969.1	AC002456.2	grey	0.374021358
ENSG00000241764.3	AC002467.7	turquoise	0.772414739
ENSG00000267750.1	AC003102.3	turquoise	0.866906916
ENSG00000234494.3	AC003665.1	turquoise	0.883855627
ENSG00000268948.1	AC004017.1	turquoise	0.769754296
ENSG00000236754.1	AC004019.13	green	0.700806144
ENSG00000251259.1	AC004069.2	turquoise	0.632637801
ENSG00000260664.1	AC004158.3	brown	0.660655424
ENSG00000267778.1	AC004221.2	turquoise	0.819095253
ENSG00000270143.1	AC004257.3	turquoise	0.741077844
ENSG00000005189.15	AC004381.6	turquoise	0.884037028
ENSG00000226107.1	AC004383.3	turquoise	0.656313369
ENSG00000269131.1	AC004447.2	yellow	0.943708561
ENSG00000227078.1	AC004448.2	grey	0.64278574
ENSG00000261204.1	AC004449.6	turquoise	0.552222736
ENSG00000260924.2	AC004463.6	turquoise	0.698629475
ENSG00000268069.1	AC004466.1	yellow	0.844224663
ENSG00000225792.1	AC004540.4	grey	0.178859857
ENSG00000214870.4	AC004540.5	turquoise	0.794434159
ENSG00000225877.1	AC004603.4	brown	0.501186477
ENSG00000175873.3	AC004840.9	turquoise	0.874535599
ENSG00000242687.2	AC004893.11	turquoise	0.801028483
ENSG00000239556.2	AC004951.5	turquoise	0.900776323
ENSG00000243554.1	AC004967.7	blue	0.697088428
ENSG00000205485.9	AC004980.7	turquoise	0.843392207
ENSG00000241057.1	AC004985.12	turquoise	0.891954849
ENSG00000233225.2	AC004987.9	turquoise	0.857111628
ENSG00000213888.2	AC005003.1	turquoise	0.851102899
ENSG00000228421.2	AC005013.5	turquoise	0.516044164
ENSG00000224046.1	AC005076.5	turquoise	0.786537926
ENSG00000226816.2	AC005082.12	turquoise	0.819584597
ENSG00000223374.1	AC005104.3	blue	0.681615801
ENSG00000234899.5	AC005152.2	green	0.814307954
ENSG00000196295.7	AC005154.6	yellow	0.917698526
ENSG00000244480.1	AC005154.7	yellow	0.901894944
ENSG00000269694.1	AC005197.2	green	0.715041272
ENSG00000236352.1	AC005220.3	grey	0.239639421
ENSG00000231595.1	AC005224.2	turquoise	0.759232527
ENSG00000268030.1	AC005253.2	yellow	0.876223211
ENSG00000267283.1	AC005306.3	grey	0.782147613
ENSG00000233002.2	AC005324.6	turquoise	0.677554667
ENSG00000248015.2	AC005329.7	yellow	0.850746923
ENSG00000267372.1	AC005330.2	turquoise	0.898558291

ENSG00000267056.2	AC005336.4	green	0.77215461
ENSG00000262686.1	AC005356.1	turquoise	0.778470161
ENSG00000255513.1	AC005363.9	blue	0.653780969
ENSG00000268938.2	AC005387.3	turquoise	0.524704934
ENSG00000267421.2	AC005498.3	red	0.738003915
ENSG00000267224.1	AC005498.4	grey	0.510332525
ENSG00000223878.1	AC005517.3	yellow	0.780176956
ENSG00000258559.2	AC005519.4	yellow	0.935424603
ENSG00000272070.1	AC005618.6	turquoise	0.804621649
ENSG00000226040.2	AC005740.3	turquoise	0.730210159
ENSG00000272108.1	AC005754.8	turquoise	0.807081385
ENSG00000266933.1	AC005775.2	red	0.465583711
ENSG00000268189.2	AC005785.2	grey	0.770586789
ENSG00000267473.1	AC005789.11	turquoise	0.718770174
ENSG00000267161.1	AC005943.5	turquoise	0.681734571
ENSG00000227719.1	AC006042.6	turquoise	0.793807396
ENSG00000269793.1	AC006115.3	turquoise	0.885192245
ENSG00000267776.1	AC006116.24	turquoise	0.622215613
ENSG00000270164.1	AC006129.4	magenta	0.816316166
ENSG00000268460.1	AC006262.6	turquoise	0.793460471
ENSG00000269483.1	AC006272.1	turquoise	0.649621981
ENSG00000253392.2	AC006277.2	turquoise	0.610070731
ENSG00000261342.1	AC006538.1	turquoise	0.758277114
ENSG00000236540.3	AC006547.13	turquoise	0.848883794
ENSG00000234409.4	AC006547.14	turquoise	0.742911709
ENSG00000269194.1	AC006942.4	yellow	0.736065438
ENSG00000235478.1	AC006946.15	turquoise	0.774204493
ENSG00000273203.1	AC006946.16	turquoise	0.883064358
ENSG00000273442.1	AC006946.17	turquoise	0.698122323
ENSG00000235859.4	AC006978.6	blue	0.812515399
ENSG00000237815.1	AC007000.11	turquoise	0.513095759
ENSG00000244239.1	AC007009.1	turquoise	0.643578708
ENSG00000233723.3	AC007092.1	turquoise	0.894051446
ENSG00000237166.1	AC007163.3	brown	0.773424476
ENSG00000269124.1	AC007193.10	green	0.609776474
ENSG00000268810.1	AC007193.9	yellow	0.882285272
ENSG00000269696.1	AC007228.11	turquoise	0.85857651
ENSG00000268568.1	AC007228.9	turquoise	0.534411994
ENSG00000231312.2	AC007246.3	turquoise	0.931720356
ENSG00000227855.3	AC007276.5	turquoise	0.853355644
ENSG00000267980.1	AC007292.6	grey	0.715657854
ENSG00000271275.1	AC007326.9	turquoise	0.795902446
ENSG00000180672.4	AC007362.1	blue	0.478993882
ENSG00000268688.1	AC007382.1	turquoise	0.747337249
ENSG00000227946.1	AC007383.3	turquoise	0.780129666
ENSG00000218739.5	AC007390.5	turquoise	0.966986523
ENSG00000239467.1	AC007405.6	turquoise	0.907461846
ENSG00000235934.1	AC007405.8	turquoise	0.775339506

ENSG00000267893.1	AC007461.1	green	0.582788575
ENSG00000228878.3	AC007551.3	brown	0.706543284
ENSG00000244055.1	AC007566.10	brown	0.871724561
ENSG00000242539.2	AC007620.3	blue	0.750769364
ENSG00000233251.3	AC007743.1	turquoise	0.598779206
ENSG00000267213.3	AC007773.2	turquoise	0.531852505
ENSG00000256439.1	AC008731.1	yellow	0.786058772
ENSG00000267838.1	AC008746.12	grey	0.660662709
ENSG00000235681.1	AC008746.5	turquoise	0.6281612
ENSG00000271824.1	AC009014.3	midnightblue	0.87662045
ENSG00000203401.4	AC009061.1	grey	0.559925868
ENSG00000239763.2	AC009120.3	turquoise	0.771265958
ENSG00000260884.1	AC009120.5	turquoise	0.760736028
ENSG00000259972.1	AC009120.6	turquoise	0.838065307
ENSG00000261690.1	AC009133.12	yellow	0.910091201
ENSG00000238045.5	AC009133.14	pink	0.848336393
ENSG00000242628.1	AC009228.1	turquoise	0.555892385
ENSG00000236431.1	AC009237.11	turquoise	0.705951131
ENSG00000229689.2	AC009237.8	turquoise	0.78155848
ENSG00000232320.5	AC009299.5	turquoise	0.707634063
ENSG00000216895.4	AC009403.2	turquoise	0.904396369
ENSG00000233143.1	AC009492.1	turquoise	0.778986369
ENSG00000231609.1	AC009501.4	blue	0.684768553
ENSG00000224152.1	AC009506.1	turquoise	0.832242936
ENSG00000223960.2	AC009948.5	turquoise	0.812912733
ENSG00000238082.1	AC009948.7	turquoise	0.778638117
ENSG00000249249.1	AC010226.4	brown	0.496024675
ENSG00000269808.1	AC010327.2	turquoise	0.897186017
ENSG00000267219.1	AC010504.2	turquoise	0.764295814
ENSG00000226180.2	AC010536.1	turquoise	0.70530112
ENSG00000235118.3	AC010731.4	turquoise	0.778483456
ENSG00000264577.1	AC010761.8	turquoise	0.643506728
ENSG00000265474.1	AC010761.9	blue	0.605575752
ENSG00000234936.1	AC010883.5	pink	0.558623574
ENSG00000224509.1	AC010884.1	turquoise	0.81800561
ENSG00000226853.2	AC010894.3	red	0.697625832
ENSG00000237732.5	AC010980.2	turquoise	0.86839281
ENSG00000223658.5	AC011242.6	green	0.651560374
ENSG00000236015.1	AC011290.5	yellow	0.858405934
ENSG00000233487.6	AC011322.1	turquoise	0.760295676
ENSG00000268038.1	AC011516.2	grey	0.191017092
ENSG00000267107.2	AC011526.1	black	0.710629263
ENSG00000235092.1	AC011747.7	green	0.593436061
ENSG00000229233.1	AC011891.5	turquoise	0.694288372
ENSG00000237720.1	AC011995.1	turquoise	0.706859197
ENSG00000179061.1	AC012074.1	turquoise	0.737897376
ENSG00000235072.1	AC012074.2	turquoise	0.716081489
ENSG00000234327.3	AC012146.7	turquoise	0.826044592

ENSG00000204685.5	AC012307.3	turquoise	0.841248439
ENSG00000226686.3	AC012309.5	turquoise	0.498829479
ENSG00000224543.3	AC012318.3	turquoise	0.800076476
ENSG00000225156.2	AC012354.6	turquoise	0.794959748
ENSG00000227799.1	AC012358.4	turquoise	0.860508815
ENSG00000243389.1	AC012442.5	turquoise	0.530845067
ENSG00000232520.1	AC012507.3	turquoise	0.88944664
ENSG00000204588.5	AC013268.5	turquoise	0.802193782
ENSG00000221625.1	AC013283.1	turquoise	0.643106756
ENSG00000272888.1	AC013394.2	brown	0.686598403
ENSG00000235597.1	AC013402.2	brown	0.770234042
ENSG00000229727.2	AC013460.1	turquoise	0.69690147
ENSG00000230286.1	AC013472.4	turquoise	0.664157983
ENSG00000267025.1	AC015849.19	yellow	0.720048468
ENSG00000237805.1	AC015849.2	grey	0.561683615
ENSG00000267858.1	AC016629.8	pink	0.900947495
ENSG00000189223.9	AC016683.6	turquoise	0.551982275
ENSG00000231327.1	AC016700.5	turquoise	0.715105586
ENSG00000224731.1	AC016716.2	grey	0.016081552
ENSG00000239332.1	AC016722.2	turquoise	0.759758279
ENSG00000224043.3	AC016725.4	brown	0.653439088
ENSG00000223947.1	AC016738.4	turquoise	0.766665026
ENSG00000212978.5	AC016747.3	blue	0.841445326
ENSG00000186235.6	AC016757.3	brown	0.70655861
ENSG00000218422.2	AC016773.1	green	0.549244579
ENSG00000163364.5	AC017048.3	turquoise	0.673435695
ENSG00000225964.1	AC017076.5	turquoise	0.736782256
ENSG00000228486.5	AC017099.3	yellow	0.823122752
ENSG00000224376.1	AC017104.6	grey	0.60637336
ENSG00000231453.1	AC018470.4	turquoise	0.681754498
ENSG00000229413.1	AC018638.1	yellow	0.644321818
ENSG00000227544.4	AC018647.3	brown	0.786060936
ENSG00000230104.1	AC018712.2	turquoise	0.761726994
ENSG00000233639.1	AC018730.1	red	0.661581151
ENSG00000236859.2	AC018737.1	brown	0.618845313
ENSG00000237039.1	AC018738.2	grey	0.156390958
ENSG00000167765.3	AC018755.1	turquoise	0.787348197
ENSG00000269388.1	AC018755.16	red	0.70797214
ENSG00000267896.1	AC018766.4	yellow	0.722492135
ENSG00000269352.1	AC018766.5	yellow	0.937902677
ENSG00000234423.1	AC019118.2	turquoise	0.813147412
ENSG00000269210.1	AC019171.1	turquoise	0.833405341
ENSG00000268328.1	AC019206.1	turquoise	0.686304926
ENSG00000237133.1	AC020594.5	grey	0.509126899
ENSG00000203403.2	AC020907.2	turquoise	0.617821792
ENSG00000256383.1	AC020910.2	turquoise	0.864657117
ENSG00000206567.5	AC022007.5	turquoise	0.741317188
ENSG00000267623.2	AC022153.1	turquoise	0.744698401

ENSG00000206176.5	AC023490.1	turquoise	0.509733399
ENSG00000214135.4	AC024560.3	turquoise	0.923417869
ENSG00000244063.1	AC024704.2	turquoise	0.776150414
ENSG00000231443.2	AC024937.6	yellow	0.753346
ENSG00000224713.3	AC025165.8	yellow	0.775349775
ENSG00000179859.7	AC025335.1	pink	0.903894207
ENSG00000270947.1	AC025811.3	grey	0.110425351
ENSG00000174977.7	AC026271.5	turquoise	0.616533144
ENSG00000268885.1	AC026740.1	turquoise	0.73301378
ENSG00000268913.1	AC026806.2	magenta	0.69746757
ENSG00000268798.1	AC027307.3	turquoise	0.826635909
ENSG00000143429.5	AC027612.6	turquoise	0.849060745
ENSG00000215067.5	AC027763.2	blue	0.909805104
ENSG00000180385.4	AC034193.5	turquoise	0.8627058
ENSG00000233006.2	AC034220.3	turquoise	0.874934516
ENSG00000223548.1	AC034228.3	grey	0.309966658
ENSG00000229334.1	AC046143.3	turquoise	0.735768137
ENSG00000222020.2	AC062017.1	grey	0.478686173
ENSG00000226939.1	AC062021.1	grey	0.132360745
ENSG00000235026.1	AC066593.1	greenyellow	0.829258062
ENSG00000268055.1	AC067969.2	grey	0.12279223
ENSG00000228389.1	AC068039.4	yellow	0.678076926
ENSG00000228528.1	AC068057.1	turquoise	0.579537038
ENSG00000251485.1	AC068134.10	turquoise	0.790655641
ENSG00000225049.1	AC068279.2	turquoise	0.597805262
ENSG00000236682.1	AC068282.3	turquoise	0.52690291
ENSG00000235027.1	AC068580.6	blue	0.701479861
ENSG00000224441.1	AC068831.3	turquoise	0.630201254
ENSG00000224769.1	AC069213.1	turquoise	0.40116394
ENSG00000237418.1	AC069257.6	turquoise	0.674271433
ENSG00000267242.1	AC069278.4	turquoise	0.731179264
ENSG00000225507.1	AC069282.6	turquoise	0.854811101
ENSG00000229178.1	AC069513.4	grey	0.416650081
ENSG00000269165.1	AC069547.1	grey	0.306669305
ENSG00000231359.3	AC072052.7	midnightblue	0.773040881
ENSG00000231840.1	AC073342.12	brown	0.588487804
ENSG00000198580.6	AC073343.1	turquoise	0.84653749
ENSG00000228010.1	AC073343.13	turquoise	0.837610585
ENSG00000232502.1	AC073464.7	turquoise	0.721748849
ENSG00000232044.4	AC073479.1	brown	0.854421858
ENSG00000234072.1	AC074117.10	yellow	0.886368648
ENSG00000225975.2	AC074138.3	turquoise	0.891940646
ENSG00000225889.3	AC074289.1	turquoise	0.692097783
ENSG00000182329.6	AC079354.1	pink	0.85237198
ENSG00000214100.4	AC079776.2	brown	0.62656143
ENSG00000225449.2	AC079776.7	brown	0.66601285
ENSG00000233230.1	AC079807.2	turquoise	0.802124715
ENSG00000237753.1	AC079922.3	turquoise	0.836142751

ENSG00000203644.2	AC083799.1	blue	0.582049285
ENSG00000259820.1	AC083843.1	blue	0.677025914
ENSG00000212694.4	AC084018.1	yellow	0.81370322
ENSG00000186019.10	AC084219.4	turquoise	0.912746428
ENSG00000236377.1	AC084809.3	turquoise	0.605102914
ENSG00000236819.1	AC087393.1	turquoise	0.801321909
ENSG00000250404.1	AC090587.2	yellow	0.819791544
ENSG00000229368.1	AC090587.4	turquoise	0.800099649
ENSG00000228661.1	AC090587.5	turquoise	0.726707274
ENSG00000230532.1	AC091133.1	yellow	0.874467793
ENSG00000229043.2	AC091729.9	turquoise	0.667546392
ENSG00000230733.2	AC092171.4	turquoise	0.886161939
ENSG00000233527.4	AC092295.7	turquoise	0.738673743
ENSG00000237887.1	AC092839.1	grey	0.111706387
ENSG00000238018.1	AC093110.3	blue	0.74884003
ENSG00000269175.1	AC093157.1	yellow	0.809962806
ENSG00000228541.1	AC093159.1	grey	0.44240214
ENSG00000170846.11	AC093323.3	turquoise	0.938842134
ENSG00000233654.1	AC093388.3	red	0.564884574
ENSG00000235257.4	AC093415.2	turquoise	0.900171319
ENSG00000241269.1	AC093620.5	grey	0.361180036
ENSG00000240859.1	AC093627.10	turquoise	0.870677365
ENSG00000237940.3	AC093642.3	turquoise	0.658268816
ENSG00000256604.1	AC093668.2	grey	0.415949532
ENSG00000232533.1	AC093673.5	red	0.667186811
ENSG00000152117.13	AC093838.4	yellow	0.909757331
ENSG00000269690.1	AC096677.1	blue	0.668589249
ENSG00000244567.1	AC096772.6	turquoise	0.942969226
ENSG00000235024.1	AC097468.7	turquoise	0.822102221
ENSG00000226833.1	AC097724.3	green	0.558364835
ENSG00000213846.3	AC098614.2	turquoise	0.622595033
ENSG00000227745.1	AC098826.5	turquoise	0.744767521
ENSG00000225386.1	AC099754.1	grey	0.225319213
ENSG00000224738.1	AC099850.1	turquoise	0.724843057
ENSG00000265967.1	AC100830.4	yellow	0.75420724
ENSG00000233850.1	AC103563.8	brown	0.516568791
ENSG00000231062.1	AC103563.9	brown	0.751517674
ENSG00000229203.1	AC103564.7	turquoise	0.656740884
ENSG00000225151.6	AC103965.1	turquoise	0.526169852
ENSG00000267571.1	AC104532.4	blue	0.587023844
ENSG00000228857.2	AC104653.1	turquoise	0.760248667
ENSG00000232504.4	AC105053.4	brown	0.759493467
ENSG00000234171.1	AC108488.3	red	0.59573177
ENSG00000242282.2	AC108488.4	yellow	0.794531299
ENSG00000269302.1	AC110771.1	turquoise	0.670617106
ENSG00000175772.10	AC112229.7	turquoise	0.841163279
ENSG00000233223.2	AC113189.5	red	0.845100214
ENSG00000235351.1	AC114730.11	yellow	0.818096329

ENSG00000235151.1	AC114730.2	turquoise	0.55653059
ENSG00000224272.2	AC114730.3	turquoise	0.438923726
ENSG00000268601.1	AC115522.3	turquoise	0.764575071
ENSG00000233585.1	AC115617.2	yellow	0.728189057
ENSG00000268175.1	AC117395.1	pink	0.834853938
ENSG00000211553.1	AC118278.1	turquoise	0.428614568
ENSG00000229782.1	AC118754.4	turquoise	0.734810376
ENSG00000197815.3	AC122129.1	blue	0.764666733
ENSG00000262728.1	AC123768.4	turquoise	0.74946372
ENSG00000251695.2	AC124890.1	yellow	0.821543607
ENSG00000226155.1	AC124944.3	grey	0.610881553
ENSG00000231259.3	AC125232.1	turquoise	0.752599562
ENSG00000237167.1	AC128709.2	turquoise	0.710842094
ENSG00000235126.1	AC128709.3	turquoise	0.798376398
ENSG00000214999.3	AC129492.6	turquoise	0.820265966
ENSG00000216921.3	AC131097.4	grey	0.139954869
ENSG00000213519.2	AC132008.1	turquoise	0.649063842
ENSG00000268403.1	AC132192.1	grey	0.432632524
ENSG00000228989.1	AC133528.2	turquoise	0.821541445
ENSG00000198150.2	AC135178.1	yellow	0.915118777
ENSG00000235296.1	AC137723.5	turquoise	0.858448683
ENSG00000268218.1	AC137932.1	turquoise	0.819973933
ENSG00000260279.1	AC137932.5	turquoise	0.630561335
ENSG00000183889.8	AC138969.4	grey	0.509606156
ENSG00000267270.1	AC139100.2	cyan	0.768356844
ENSG00000267251.1	AC139100.3	turquoise	0.813919525
ENSG00000178162.4	AC140481.1	blue	0.761754883
ENSG00000240253.1	AC140481.7	turquoise	0.746912347
ENSG00000232503.1	AC140481.9	turquoise	0.656041641
ENSG00000261325.1	AC140542.2	midnightblue	0.695372892
ENSG00000261227.2	AC140912.1	turquoise	0.818762858
ENSG00000215154.2	AC141586.5	turquoise	0.831569994
ENSG00000250986.1	AC141928.1	turquoise	0.818928257
ENSG00000231969.1	AC144449.1	grey	0.152286069
ENSG00000273117.1	AC144652.1	turquoise	0.837764222
ENSG00000223855.1	AC147651.3	turquoise	0.568352552
ENSG00000230606.6	AC159540.1	turquoise	0.739819324
ENSG00000255748.1	AC226150.4	turquoise	0.702231222
ENSG00000251775.1	ACA59	turquoise	0.534340793
ENSG00000060971.13	ACAA1	blue	0.905054798
ENSG00000167315.13	ACAA2	green	0.892333302
ENSG00000132142.15	ACACA	turquoise	0.971371183
ENSG00000076555.11	ACACB	green	0.805839875
ENSG00000111271.10	ACAD10	turquoise	0.928177708
ENSG00000151498.7	ACAD8	turquoise	0.965329684
ENSG00000177646.13	ACAD9	turquoise	0.947713524
ENSG00000117054.9	ACADM	blue	0.848745878
ENSG00000122971.4	ACADS	green	0.72824987

ENSG00000196177.8	ACADSB	turquoise	0.920184047
ENSG00000072778.15	ACADVL	blue	0.832868966
ENSG00000114331.8	ACAP2	turquoise	0.85822762
ENSG00000229325.1	ACAP2-IT1	turquoise	0.83067911
ENSG00000131584.14	ACAP3	turquoise	0.840841877
ENSG00000075239.9	ACAT1	blue	0.892277646
ENSG00000120437.7	ACAT2	turquoise	0.917206188
ENSG00000182827.8	ACBD3	turquoise	0.861384868
ENSG00000181513.10	ACBD4	turquoise	0.861463928
ENSG00000107897.14	ACBD5	blue	0.853068128
ENSG00000135847.5	ACBD6	turquoise	0.94497461
ENSG00000176244.6	ACBD7	green	0.851490748
ENSG00000110455.9	ACCS	yellow	0.760676311
ENSG00000102977.9	ACD	turquoise	0.931656606
ENSG00000159640.10	ACE	turquoise	0.811216486
ENSG00000177076.4	ACER2	black	0.673714631
ENSG00000078124.7	ACER3	brown	0.874979032
ENSG00000087085.9	ACHE	turquoise	0.874852308
ENSG00000100813.10	ACIN1	turquoise	0.892604724
ENSG00000144476.5	ACKR3	green	0.781797189
ENSG00000131473.12	ACLY	brown	0.789880897
ENSG00000196636.7	ACN9	turquoise	0.882065052
ENSG00000122729.14	ACO1	turquoise	0.880080641
ENSG00000100412.11	ACO2	turquoise	0.923873202
ENSG00000184227.3	ACOT1	blue	0.514740834
ENSG00000162390.13	ACOT11	green	0.790798595
ENSG00000112304.6	ACOT13	blue	0.861397076
ENSG00000119673.10	ACOT2	turquoise	0.778663225
ENSG00000177465.4	ACOT4	turquoise	0.844704082
ENSG00000097021.15	ACOT7	turquoise	0.932625574
ENSG00000101473.12	ACOT8	turquoise	0.937641127
ENSG00000123130.12	ACOT9	blue	0.915937209
ENSG00000161533.7	ACOX1	blue	0.895502478
ENSG00000168306.8	ACOX2	green	0.838697153
ENSG00000087008.11	ACOX3	turquoise	0.799427425
ENSG00000143727.11	ACP1	turquoise	0.968519503
ENSG00000134575.5	ACP2	turquoise	0.927824436
ENSG00000162836.7	ACP6	blue	0.749221591
ENSG00000155893.7	ACPL2	turquoise	0.952365809
ENSG00000100312.6	ACR	turquoise	0.77830949
ENSG00000147174.7	ACRC	grey	0.704933937
ENSG00000134940.9	ACRV1	turquoise	0.831047668
ENSG00000103740.5	ACSBG1	green	0.83184513
ENSG00000167107.8	ACSF2	green	0.856353365
ENSG00000176715.11	ACSF3	turquoise	0.917264095
ENSG00000151726.9	ACSL1	turquoise	0.800619054
ENSG00000123983.9	ACSL3	turquoise	0.908557008
ENSG00000068366.15	ACSL4	turquoise	0.939396222

ENSG00000197142.6	ACSL5	black	0.866876409
ENSG00000164398.8	ACSL6	turquoise	0.813698011
ENSG00000183549.6	ACSM5	blue	0.563882971
ENSG00000154930.10	ACSS1	green	0.766057551
ENSG00000131069.15	ACSS2	brown	0.880410439
ENSG00000111058.3	ACSS3	green	0.827831216
ENSG00000143632.10	ACTA1	grey	0.17759464
ENSG00000107796.8	ACTA2	grey	0.486782969
ENSG00000075624.9	ACTB	turquoise	0.927251632
ENSG00000159251.6	ACTC1	grey	0.368245894
ENSG00000184009.5	ACTG1	turquoise	0.932667879
ENSG00000163017.9	ACTG2	grey	0.233977706
ENSG00000136518.12	ACTL6A	blue	0.862283221
ENSG00000077080.5	ACTL6B	turquoise	0.902087062
ENSG00000072110.9	ACTN1	turquoise	0.920716938
ENSG00000077522.8	ACTN2	turquoise	0.916698634
ENSG00000130402.7	ACTN4	blue	0.857078424
ENSG00000131966.9	ACTR10	turquoise	0.969283866
ENSG00000138107.7	ACTR1A	turquoise	0.956877518
ENSG00000115073.6	ACTR1B	turquoise	0.950399137
ENSG00000138071.9	ACTR2	turquoise	0.954276098
ENSG00000115091.7	ACTR3	turquoise	0.94126002
ENSG00000133627.13	ACTR3B	turquoise	0.922726436
ENSG00000101442.8	ACTR5	turquoise	0.935692499
ENSG00000075089.5	ACTR6	turquoise	0.924682364
ENSG00000113812.9	ACTR8	turquoise	0.919497887
ENSG00000184378.2	ACTRT3	turquoise	0.44333386
ENSG00000115170.9	ACVR1	turquoise	0.804207119
ENSG00000135503.8	ACVR1B	turquoise	0.960968237
ENSG00000123612.11	ACVR1C	turquoise	0.888347642
ENSG00000121989.10	ACVR2A	turquoise	0.844627196
ENSG00000114739.9	ACVR2B	turquoise	0.858380369
ENSG00000229589.1	ACVR2B-AS1	turquoise	0.835764895
ENSG00000139567.8	ACVRL1	black	0.775191595
ENSG00000243989.3	ACY1	blue	0.733292932
ENSG00000132744.3	ACY3	grey	0.289128914
ENSG00000119640.4	ACYP1	turquoise	0.919328311
ENSG00000170634.8	ACYP2	turquoise	0.879290895
ENSG00000236144.2	AD000090.2	yellow	0.919682585
ENSG00000240616.1	AD000092.3	blue	0.692384143
ENSG00000268947.1	AD000684.2	yellow	0.773143799
ENSG00000196839.8	ADA	brown	0.742177056
ENSG00000140955.6	ADAD2	turquoise	0.417848074
ENSG00000168803.10	ADAL	turquoise	0.925332059
ENSG00000137845.10	ADAM10	turquoise	0.87675837
ENSG00000073670.9	ADAM11	turquoise	0.875378974
ENSG00000148848.10	ADAM12	turquoise	0.76587397
ENSG00000143537.9	ADAM15	yellow	0.850622566

ENSG00000151694.8	ADAM17	turquoise	0.9055747
ENSG00000135074.11	ADAM19	brown	0.759179185
ENSG00000229186.3	ADAM1A	turquoise	0.881361959
ENSG00000008277.10	ADAM22	turquoise	0.950546186
ENSG00000114948.8	ADAM23	turquoise	0.934600941
ENSG00000042980.8	ADAM28	magenta	0.869996209
ENSG00000149451.13	ADAM33	grey	0.589168232
ENSG00000151651.11	ADAM8	yellow	0.886270824
ENSG00000168615.7	ADAM9	blue	0.912242617
ENSG00000154734.10	ADAMTS1	black	0.74774706
ENSG00000142303.9	ADAMTS10	yellow	0.755949093
ENSG00000160323.14	ADAMTS13	yellow	0.911450113
ENSG00000138316.6	ADAMTS14	brown	0.804216447
ENSG00000140470.9	ADAMTS17	green	0.71261485
ENSG00000145808.4	ADAMTS19	turquoise	0.778283735
ENSG00000156140.4	ADAMTS3	turquoise	0.824617448
ENSG00000158859.9	ADAMTS4	brown	0.724850123
ENSG00000136378.10	ADAMTS7	blue	0.552863797
ENSG00000134917.9	ADAMTS8	turquoise	0.810119106
ENSG00000163638.9	ADAMTS9	black	0.81591373
ENSG00000197859.5	ADAMTSL2	turquoise	0.839534661
ENSG00000143382.9	ADAMTSL4	black	0.771393489
ENSG00000185761.6	ADAMTSL5	grey	0.325907879
ENSG00000105963.9	ADAP1	turquoise	0.7025632
ENSG00000184060.6	ADAP2	magenta	0.887922609
ENSG00000160710.11	ADAR	turquoise	0.979985512
ENSG00000197381.11	ADARB1	turquoise	0.939445031
ENSG00000185736.11	ADARB2	brown	0.711325557
ENSG00000205696.4	ADARB2-AS1	brown	0.563370311
ENSG00000065457.6	ADAT1	turquoise	0.881666909
ENSG00000189007.11	ADAT2	turquoise	0.887428825
ENSG00000213638.4	ADAT3	blue	0.564373954
ENSG00000142920.12	ADC	brown	0.807889052
ENSG00000063761.11	ADCK1	turquoise	0.869695906
ENSG00000133597.5	ADCK2	turquoise	0.94753556
ENSG00000163050.12	ADCK3	turquoise	0.955622794
ENSG00000123815.7	ADCK4	pink	0.84519491
ENSG00000173137.7	ADCK5	turquoise	0.843910946
ENSG00000164742.10	ADCY1	turquoise	0.730494557
ENSG00000161912.13	ADCY10P1	brown	0.75054623
ENSG00000078295.11	ADCY2	green	0.875808424
ENSG00000138031.10	ADCY3	turquoise	0.928925042
ENSG00000129467.9	ADCY4	black	0.667687364
ENSG00000173175.10	ADCY5	turquoise	0.896032593
ENSG00000174233.7	ADCY6	blue	0.798535898
ENSG00000121281.8	ADCY7	magenta	0.830108847
ENSG00000155897.5	ADCY8	green	0.805385991
ENSG00000162104.5	ADCY9	turquoise	0.940243194

ENSG00000141433.8	ADCYAP1	grey	0.372097388
ENSG00000078549.10	ADCYAP1R1	green	0.834643908
ENSG00000087274.12	ADD1	turquoise	0.914109532
ENSG00000075340.18	ADD2	turquoise	0.923064491
ENSG00000148700.9	ADD3	green	0.87087577
ENSG00000196616.8	ADH1B	grey	0.258878983
ENSG00000197894.6	ADH5	blue	0.896450997
ENSG00000147576.11	ADHFE1	green	0.789274246
ENSG00000182551.9	ADI1	blue	0.763112514
ENSG00000159346.8	ADIPOR1	turquoise	0.962141831
ENSG00000006831.9	ADIPOR2	brown	0.874552745
ENSG00000272734.1	ADIRF-AS1	blue	0.589697759
ENSG00000156110.9	ADK	turquoise	0.946366424
ENSG00000148926.5	ADM	black	0.689016069
ENSG00000224420.3	ADM5	blue	0.571379467
ENSG00000101126.11	ADNP	turquoise	0.942914761
ENSG00000101544.4	ADNP2	turquoise	0.881896701
ENSG00000181915.3	ADO	turquoise	0.912833067
ENSG00000163485.11	ADORA1	turquoise	0.700474991
ENSG00000128271.15	ADORA2A	turquoise	0.731091869
ENSG00000170425.3	ADORA2B	green	0.64270793
ENSG00000121933.13	ADORA3	magenta	0.924969793
ENSG00000159322.13	ADPGK	turquoise	0.915915751
ENSG00000144843.7	ADPRH	black	0.855848191
ENSG00000153531.8	ADPRHL1	turquoise	0.866627803
ENSG00000116863.10	ADPRHL2	pink	0.862735117
ENSG00000170222.11	ADPRM	turquoise	0.906146607
ENSG00000170214.3	ADRA1B	turquoise	0.461157729
ENSG00000171873.6	ADRA1D	midnightblue	0.842724332
ENSG00000222040.3	ADRA2B	turquoise	0.744590254
ENSG00000184160.6	ADRA2C	turquoise	0.674111958
ENSG00000043591.4	ADRB1	turquoise	0.745086035
ENSG00000169252.4	ADRB2	magenta	0.677358472
ENSG00000173020.6	ADRBK1	turquoise	0.916857238
ENSG00000100077.10	ADRBK2	turquoise	0.888502727
ENSG00000130706.8	ADRM1	turquoise	0.886449664
ENSG00000239900.7	ADSL	turquoise	0.957308057
ENSG00000035687.9	ADSS	turquoise	0.943208962
ENSG00000185100.6	ADSSL1	red	0.74437825
ENSG00000139154.10	AEBP2	turquoise	0.909622212
ENSG00000181026.13	AEN	cyan	0.800406277
ENSG00000104964.10	AES	turquoise	0.893906128
ENSG00000263335.1	AF001548.5	brown	0.669681925
ENSG00000238039.1	AF011889.2	turquoise	0.603231542
ENSG00000267388.1	AF038458.5	grey	0.609945001
ENSG00000237721.1	AF064858.11	turquoise	0.681660906
ENSG00000232837.1	AF064858.7	turquoise	0.548187969
ENSG00000235888.1	AF064858.8	turquoise	0.61066641

ENSG00000255310.2	AF131215.2	turquoise	0.811116373
ENSG00000270076.1	AF131215.8	turquoise	0.625605471
ENSG00000269918.1	AF131215.9	turquoise	0.766734628
ENSG00000255020.1	AF131216.5	turquoise	0.67366425
ENSG00000255085.4	AF186192.5	blue	0.707450451
ENSG00000196526.6	AFAP1	turquoise	0.929379894
ENSG00000157510.9	AFAP1L1	black	0.649515527
ENSG00000169129.10	AFAP1L2	brown	0.641799051
ENSG00000172493.16	AFF1	blue	0.842000362
ENSG00000144218.14	AFF3	turquoise	0.855249956
ENSG00000072364.8	AFF4	turquoise	0.949427469
ENSG00000223959.4	AFG3L1P	yellow	0.962247138
ENSG00000141385.5	AFG3L2	turquoise	0.965934935
ENSG00000183077.11	AFMID	brown	0.917707686
ENSG00000119844.10	AFTPH	turquoise	0.964201264
ENSG00000038002.4	AGA	blue	0.860522393
ENSG00000157985.13	AGAP1	brown	0.826043604
ENSG00000135439.7	AGAP2	turquoise	0.875676871
ENSG00000255737.2	AGAP2-AS1	yellow	0.799895623
ENSG00000133612.14	AGAP3	turquoise	0.917769642
ENSG00000188234.9	AGAP4	turquoise	0.456876924
ENSG00000172650.8	AGAP5	turquoise	0.8520566
ENSG00000204149.5	AGAP6	yellow	0.956550014
ENSG00000174194.11	AGAP8	turquoise	0.588463489
ENSG00000186094.12	AGBL4	turquoise	0.88404217
ENSG00000084693.11	AGBL5	turquoise	0.941715858
ENSG00000229122.1	AGBL5-IT1	turquoise	0.7166574
ENSG00000204305.9	AGER	yellow	0.737563515
ENSG00000173744.13	AGFG1	turquoise	0.935111804
ENSG00000106351.8	AGFG2	turquoise	0.90610979
ENSG00000164252.8	AGGF1	turquoise	0.942597939
ENSG00000006530.11	AGK	turquoise	0.968176034
ENSG00000162688.11	AGL	turquoise	0.839299862
ENSG00000092847.6	AGO1	turquoise	0.905674325
ENSG00000126070.14	AGO3	turquoise	0.898155926
ENSG00000134698.10	AGO4	yellow	0.921403645
ENSG00000204310.6	AGPAT1	turquoise	0.946525975
ENSG00000169692.8	AGPAT2	red	0.737318888
ENSG00000160216.14	AGPAT3	blue	0.895046408
ENSG00000026652.9	AGPAT4	brown	0.814413155
ENSG00000155189.7	AGPAT5	blue	0.88213303
ENSG00000158669.7	AGPAT6	turquoise	0.946417361
ENSG00000018510.8	AGPS	blue	0.919272764
ENSG00000188157.9	AGRN	midnightblue	0.817338978
ENSG00000135744.7	AGT	green	0.752178962
ENSG00000135049.11	AGTPBP1	brown	0.887690469
ENSG00000177674.11	AGTRAP	black	0.773630057
ENSG00000153207.10	AHCTF1	turquoise	0.91360762

ENSG00000101444.8	AHCY	turquoise	0.966697313
ENSG00000168710.13	AHCYL1	green	0.887373055
ENSG00000158467.12	AHCYL2	green	0.898291824
ENSG00000126705.9	AHDC1	turquoise	0.910185585
ENSG00000135541.16	AHI1	turquoise	0.9100218
ENSG00000124942.9	AHNAK	green	0.801754172
ENSG00000185567.6	AHNAK2	pink	0.757065834
ENSG00000106546.8	AHR	turquoise	0.802535123
ENSG00000100591.3	AHSA1	cyan	0.950357867
ENSG00000173209.18	AHSA2	yellow	0.820503931
ENSG00000186063.8	AIDA	blue	0.884411975
ENSG00000204472.8	AIF1	magenta	0.929928192
ENSG00000126878.8	AIF1L	brown	0.670823254
ENSG00000156709.9	AIFM1	turquoise	0.952739842
ENSG00000042286.10	AIFM2	turquoise	0.85864763
ENSG00000183773.11	AIFM3	green	0.690213652
ENSG00000146416.12	AIG1	blue	0.907446628
ENSG00000164022.12	AIMP1	turquoise	0.891647209
ENSG00000106305.5	AIMP2	turquoise	0.868010959
ENSG00000110711.5	AIP	red	0.954549533
ENSG00000160224.12	AIRE	turquoise	0.614246879
ENSG00000262668.1	AJ003147.9	turquoise	0.733131109
ENSG00000196581.6	AJAP1	turquoise	0.927805379
ENSG00000004455.12	AK2	blue	0.93617218
ENSG00000147853.12	AK3	blue	0.87129471
ENSG00000162433.10	AK4	blue	0.767721069
ENSG00000154027.14	AK5	turquoise	0.873447744
ENSG00000140057.4	AK7	pink	0.932972722
ENSG00000165695.5	AK8	pink	0.883989519
ENSG00000155085.11	AK9	turquoise	0.793020934
ENSG00000121057.8	AKAP1	turquoise	0.94671013
ENSG00000108599.10	AKAP10	blue	0.90656066
ENSG00000023516.7	AKAP11	turquoise	0.940612208
ENSG00000131016.12	AKAP12	turquoise	0.833229294
ENSG00000170776.15	AKAP13	turquoise	0.874983358
ENSG00000197976.6	AKAP17A	yellow	0.893921548
ENSG00000241978.5	AKAP2	blue	0.773564801
ENSG00000111254.3	AKAP3	blue	0.681859765
ENSG00000179841.8	AKAP5	turquoise	0.869221081
ENSG00000151320.6	AKAP6	turquoise	0.931623014
ENSG00000118507.11	AKAP7	turquoise	0.776486242
ENSG00000105127.4	AKAP8	turquoise	0.923115907
ENSG00000011243.13	AKAP8L	turquoise	0.877326419
ENSG00000127914.12	AKAP9	turquoise	0.93442063
ENSG00000166452.7	AKIP1	turquoise	0.94078258
ENSG00000174574.11	AKIRIN1	turquoise	0.943412788
ENSG00000135334.8	AKIRIN2	turquoise	0.961463337
ENSG00000106948.12	AKNA	pink	0.912781701

ENSG00000117448.9	AKR1A1	turquoise	0.943835982
ENSG00000085662.9	AKR1B1	turquoise	0.96563419
ENSG00000187134.8	AKR1C1	grey	0.355769223
ENSG00000151632.12	AKR1C2	brown	0.574296396
ENSG00000196139.7	AKR1C3	green	0.569526856
ENSG00000053371.8	AKR7A2	blue	0.681544264
ENSG00000162482.4	AKR7A3	green	0.529524368
ENSG00000211454.9	AKR7L	yellow	0.789344138
ENSG00000142208.11	AKT1	turquoise	0.930883444
ENSG00000204673.6	AKT1S1	red	0.922368006
ENSG00000105221.12	AKT2	yellow	0.969221658
ENSG00000117020.12	AKT3	turquoise	0.92832571
ENSG00000166971.12	AKTIP	blue	0.888058346
ENSG00000213062.3	AL021068.1	turquoise	0.78855133
ENSG00000216901.1	AL022393.7	grey	0.393151675
ENSG00000224997.1	AL049840.1	yellow	0.642193706
ENSG00000225683.1	AL078585.1	brown	0.619767632
ENSG00000225746.4	AL132709.5	turquoise	0.798549944
ENSG00000197146.2	AL133458.1	turquoise	0.7136138
ENSG00000203602.1	AL137059.1	yellow	0.813225276
ENSG00000258604.1	AL161668.5	grey	0.206171342
ENSG00000212670.1	AL161915.1	brown	0.604791504
ENSG00000227921.1	AL353791.1	turquoise	0.684135826
ENSG00000237611.1	AL353898.3	grey	0.268130387
ENSG00000268578.1	AL354718.1	turquoise	0.633472964
ENSG00000248835.2	AL357673.1	turquoise	0.831700299
ENSG00000203815.2	AL358813.2	turquoise	0.498431992
ENSG00000227821.1	AL392086.1	green	0.595965596
ENSG00000234614.1	AL450992.2	brown	0.654474552
ENSG00000263391.1	AL512428.1	turquoise	0.429987074
ENSG00000203614.3	AL512652.1	grey	0.680438497
ENSG00000269753.1	AL589739.1	pink	0.664864936
ENSG00000269554.1	AL590822.2	turquoise	0.792061262
ENSG00000269337.1	AL591479.1	turquoise	0.800508556
ENSG00000227082.1	AL592494.5	turquoise	0.833923714
ENSG00000237683.5	AL627309.1	magenta	0.79163011
ENSG00000215014.3	AL645728.1	turquoise	0.803265899
ENSG00000268996.1	AL807752.1	turquoise	0.676644425
ENSG00000148218.11	ALAD	blue	0.834080106
ENSG00000023330.10	ALAS1	turquoise	0.938930418
ENSG00000163631.12	ALB	turquoise	0.637218957
ENSG00000170017.8	ALCAM	turquoise	0.935701553
ENSG00000161618.5	ALDH16A1	blue	0.78059449
ENSG00000059573.4	ALDH18A1	turquoise	0.910493117
ENSG00000184254.12	ALDH1A3	greenyellow	0.627584553
ENSG00000137124.6	ALDH1B1	turquoise	0.670006944
ENSG00000144908.9	ALDH1L1	green	0.786443706
ENSG00000246022.2	ALDH1L1-AS2	green	0.648883438

ENSG00000136010.9	ALDH1L2	turquoise	0.757780149
ENSG00000111275.8	ALDH2	green	0.888886771
ENSG00000072210.14	ALDH3A2	turquoise	0.927669522
ENSG00000159423.12	ALDH4A1	green	0.699214976
ENSG00000112294.8	ALDH5A1	blue	0.805856821
ENSG00000119711.8	ALDH6A1	green	0.913826653
ENSG00000164904.11	ALDH7A1	green	0.841782923
ENSG00000118514.9	ALDH8A1	yellow	0.771995515
ENSG00000143149.8	ALDH9A1	green	0.925989018
ENSG00000149925.12	ALDOA	turquoise	0.930762329
ENSG00000109107.9	ALDOC	green	0.810913086
ENSG00000033011.7	ALG1	turquoise	0.927294191
ENSG00000182858.9	ALG12	turquoise	0.854353557
ENSG00000101901.6	ALG13	turquoise	0.951025928
ENSG00000172339.9	ALG14	turquoise	0.930516068
ENSG00000189366.5	ALG1L	turquoise	0.387987022
ENSG00000250794.2	ALG1L12P	turquoise	0.585884665
ENSG00000254978.2	ALG1L9P	turquoise	0.577850363
ENSG00000119523.8	ALG2	turquoise	0.879299342
ENSG00000214160.5	ALG3	turquoise	0.928308953
ENSG00000120697.4	ALG5	turquoise	0.845431261
ENSG00000088035.11	ALG6	turquoise	0.860961026
ENSG00000159063.8	ALG8	turquoise	0.950394832
ENSG00000086848.10	ALG9	turquoise	0.95716931
ENSG00000171094.11	ALK	turquoise	0.823560609
ENSG00000100601.5	ALKBH1	turquoise	0.937181861
ENSG00000189046.6	ALKBH2	turquoise	0.830088208
ENSG00000166199.8	ALKBH3	turquoise	0.886566453
ENSG00000160993.3	ALKBH4	turquoise	0.950450847
ENSG00000091542.8	ALKBH5	turquoise	0.953483891
ENSG00000239382.6	ALKBH6	turquoise	0.896809263
ENSG00000125652.3	ALKBH7	red	0.940729533
ENSG00000137760.10	ALKBH8	turquoise	0.933264261
ENSG00000116127.13	ALMS1	turquoise	0.828998901
ENSG00000179477.5	ALOX12B	turquoise	0.75010967
ENSG00000161905.8	ALOX15	turquoise	0.75420032
ENSG00000012779.6	ALOX5	magenta	0.948758882
ENSG00000132965.5	ALOX5AP	magenta	0.944607476
ENSG00000073331.13	ALPK1	green	0.642069052
ENSG00000136383.6	ALPK3	blue	0.658346982
ENSG00000162551.9	ALPL	grey	0.432649767
ENSG00000003393.10	ALS2	turquoise	0.963006173
ENSG00000178038.12	ALS2CL	brown	0.601407058
ENSG00000155749.8	ALS2CR12	pink	0.861435083
ENSG00000183684.6	ALYREF	turquoise	0.845264831
ENSG00000242110.3	AMACR	turquoise	0.908535932
ENSG00000178522.10	AMBN	turquoise	0.616287246
ENSG00000110497.10	AMBRA1	turquoise	0.959388824

ENSG00000123505.10	AMD1	turquoise	0.91798975
ENSG00000139344.3	AMDHD1	turquoise	0.876597529
ENSG00000162066.10	AMDHD2	turquoise	0.89432115
ENSG00000184675.5	AMER1	turquoise	0.913344737
ENSG00000165566.11	AMER2	brown	0.909378263
ENSG00000234056.1	AMER2-AS1	brown	0.605726334
ENSG00000178171.6	AMER3	turquoise	0.895764644
ENSG00000159461.10	AMFR	turquoise	0.963665912
ENSG00000104899.4	AMH	grey	0.685909806
ENSG00000181754.6	AMIGO1	turquoise	0.955880837
ENSG00000139211.5	AMIGO2	turquoise	0.671962213
ENSG00000101935.5	AMMECR1	turquoise	0.836653126
ENSG00000144233.5	AMMECR1L	blue	0.932502591
ENSG00000151743.6	AMN1	turquoise	0.939722466
ENSG00000126016.9	AMOT	green	0.913541682
ENSG00000166025.13	AMOTL1	blue	0.838437106
ENSG00000114019.10	AMOTL2	brown	0.9036819
ENSG00000116337.11	AMPD2	turquoise	0.923134722
ENSG00000133805.11	AMPD3	brown	0.888005599
ENSG00000078053.12	AMPH	turquoise	0.922373761
ENSG00000145020.10	AMT	turquoise	0.867569777
ENSG00000243480.3	AMY2A	turquoise	0.733159376
ENSG00000240038.2	AMY2B	yellow	0.91937639
ENSG00000174945.9	AMZ1	turquoise	0.82308109
ENSG00000196704.7	AMZ2	turquoise	0.911699842
ENSG00000214174.4	AMZ2P1	turquoise	0.937896285
ENSG00000153107.7	ANAPC1	turquoise	0.953020636
ENSG00000164162.8	ANAPC10	turquoise	0.884145898
ENSG00000141552.13	ANAPC11	red	0.967215842
ENSG00000129055.8	ANAPC13	turquoise	0.929263561
ENSG00000110200.4	ANAPC15	turquoise	0.898743993
ENSG00000166295.4	ANAPC16	turquoise	0.899910814
ENSG00000176248.7	ANAPC2	turquoise	0.920095808
ENSG00000053900.6	ANAPC4	turquoise	0.845437045
ENSG00000089053.8	ANAPC5	brown	0.837381302
ENSG00000196510.8	ANAPC7	turquoise	0.967078714
ENSG00000013523.5	ANGEL1	turquoise	0.930481424
ENSG00000174606.8	ANGEL2	turquoise	0.946730928
ENSG00000091879.9	ANGPT2	black	0.732413942
ENSG00000116194.8	ANGPTL1	grey	0.279126312
ENSG00000136859.5	ANGPTL2	brown	0.826901219
ENSG00000167772.7	ANGPTL4	black	0.694923074
ENSG00000029534.15	ANK1	greenyellow	0.848158597
ENSG00000145362.12	ANK2	turquoise	0.963124337
ENSG00000151150.16	ANK3	turquoise	0.883428667
ENSG00000166839.12	ANKDD1A	turquoise	0.802826923
ENSG00000189045.9	ANKDD1B	pink	0.901632178
ENSG00000185722.12	ANKFY1	turquoise	0.905867126

ENSG00000154122.8	ANKH	turquoise	0.950395744
ENSG00000131503.16	ANKHD1	blue	0.948051608
ENSG00000001629.5	ANKIB1	blue	0.882458612
ENSG00000170209.4	ANKK1	pink	0.754850368
ENSG00000160117.10	ANKLE1	yellow	0.876570926
ENSG00000176915.10	ANKLE2	turquoise	0.955262215
ENSG00000144504.11	ANKMY1	pink	0.91211764
ENSG00000106524.4	ANKMY2	turquoise	0.954973159
ENSG00000164331.5	ANKRA2	turquoise	0.940829222
ENSG00000088448.10	ANKRD10	yellow	0.951424159
ENSG00000229152.1	ANKRD10-IT1	blue	0.770681357
ENSG00000167522.10	ANKRD11	turquoise	0.95625296
ENSG00000101745.11	ANKRD12	turquoise	0.862635449
ENSG00000076513.12	ANKRD13A	turquoise	0.901300855
ENSG00000198720.8	ANKRD13B	turquoise	0.871442006
ENSG00000118454.8	ANKRD13C	turquoise	0.966535963
ENSG00000172932.10	ANKRD13D	turquoise	0.914659023
ENSG00000134461.11	ANKRD16	turquoise	0.958771579
ENSG00000132466.13	ANKRD17	turquoise	0.959551129
ENSG00000180071.14	ANKRD18A	turquoise	0.816980018
ENSG00000159712.10	ANKRD18CP	turquoise	0.794006104
ENSG00000217165.1	ANKRD18EP	turquoise	0.796729264
ENSG00000187984.8	ANKRD19P	turquoise	0.813012228
ENSG00000165887.7	ANKRD2	turquoise	0.782012808
ENSG00000163126.10	ANKRD23	brown	0.54624355
ENSG00000089847.8	ANKRD24	turquoise	0.804618779
ENSG00000107890.12	ANKRD26	turquoise	0.913032691
ENSG00000105186.10	ANKRD27	turquoise	0.912014258
ENSG00000206560.6	ANKRD28	turquoise	0.946893346
ENSG00000154065.12	ANKRD29	blue	0.763132195
ENSG00000163046.11	ANKRD30BL	turquoise	0.774879655
ENSG00000133302.8	ANKRD32	turquoise	0.898634392
ENSG00000181039.7	ANKRD34A	turquoise	0.889846121
ENSG00000189127.3	ANKRD34B	turquoise	0.885607713
ENSG00000198483.8	ANKRD35	green	0.707204913
ENSG00000135976.12	ANKRD36	turquoise	0.870099843
ENSG00000230006.3	ANKRD36BP2	turquoise	0.823596965
ENSG00000174501.10	ANKRD36C	turquoise	0.893510491
ENSG00000186352.4	ANKRD37	cyan	0.673478561
ENSG00000213337.4	ANKRD39	turquoise	0.956045847
ENSG00000154945.6	ANKRD40	brown	0.691880714
ENSG00000137494.9	ANKRD42	turquoise	0.929015449
ENSG00000065413.12	ANKRD44	blue	0.776512014
ENSG00000183831.6	ANKRD45	pink	0.872301213
ENSG00000186106.7	ANKRD46	turquoise	0.95290329
ENSG00000168876.4	ANKRD49	turquoise	0.928368674
ENSG00000151458.7	ANKRD50	turquoise	0.883170999
ENSG00000139645.9	ANKRD52	turquoise	0.926501737

ENSG00000100124.8	ANKRD54	pink	0.875198893
ENSG00000135299.12	ANKRD6	turquoise	0.810718733
ENSG00000230778.1	ANKRD63	turquoise	0.805106931
ENSG00000235098.4	ANKRD65	red	0.757059305
ENSG00000156381.4	ANKRD9	grey	0.358676145
ENSG00000064999.12	ANKS1A	turquoise	0.901562258
ENSG00000185046.14	ANKS1B	turquoise	0.941683848
ENSG00000168096.10	ANKS3	turquoise	0.843549871
ENSG00000165138.12	ANKS6	brown	0.922384137
ENSG00000163516.9	ANKZF1	yellow	0.967047486
ENSG00000011426.6	ANLN	brown	0.803988657
ENSG00000160746.8	ANO10	turquoise	0.910503631
ENSG00000047617.10	ANO2	turquoise	0.75225313
ENSG00000134343.8	ANO3	turquoise	0.74769977
ENSG00000151572.12	ANO4	brown	0.920968413
ENSG00000171714.10	ANO5	turquoise	0.90984933
ENSG00000177119.11	ANO6	green	0.750274361
ENSG00000146205.9	ANO7	yellow	0.681018456
ENSG00000074855.6	ANO8	green	0.762809744
ENSG00000140350.11	ANP32A	turquoise	0.966594484
ENSG00000136938.8	ANP32B	brown	0.700488575
ENSG00000143401.10	ANP32E	blue	0.828606068
ENSG00000166825.9	ANPEP	grey	0.380549291
ENSG00000169604.15	ANTXR1	green	0.752982083
ENSG00000163297.12	ANTXR2	green	0.621183783
ENSG00000122359.13	ANXA11	turquoise	0.935166599
ENSG00000182718.12	ANXA2	black	0.824616652
ENSG00000138772.8	ANXA3	black	0.536646412
ENSG00000196975.10	ANXA4	blue	0.781468978
ENSG00000164111.10	ANXA5	blue	0.801339575
ENSG00000197043.9	ANXA6	turquoise	0.912086364
ENSG00000138279.11	ANXA7	turquoise	0.95201951
ENSG00000143412.5	ANXA9	brown	0.902506224
ENSG00000136250.7	AOAH	turquoise	0.804437397
ENSG00000131480.4	AOC2	turquoise	0.831555527
ENSG00000131471.2	AOC3	grey	0.624665361
ENSG00000273492.1	AP000230.1	turquoise	0.734124538
ENSG00000273271.1	AP000254.8	blue	0.683538589
ENSG00000273091.1	AP000255.6	turquoise	0.415713221
ENSG00000222018.1	AP000322.54	turquoise	0.870162323
ENSG00000272578.1	AP000347.2	yellow	0.972006384
ENSG00000234353.2	AP000347.4	brown	0.52992555
ENSG00000273295.1	AP000350.5	grey	0.461767249
ENSG00000235231.3	AP000351.10	grey	0.367413741
ENSG00000240906.2	AP000356.1	turquoise	0.553757248
ENSG00000255980.1	AP000439.1	magenta	0.537343616
ENSG00000270093.1	AP000473.8	blue	0.685372009
ENSG00000254604.1	AP000487.6	grey	0.294506435

ENSG00000233393.1	AP000688.29	brown	0.744644289
ENSG00000228107.1	AP000692.9	blue	0.645633504
ENSG00000224790.1	AP000704.5	turquoise	0.586803122
ENSG00000173727.7	AP000769.1	turquoise	0.742765596
ENSG00000203306.3	AP001007.1	magenta	0.793407167
ENSG00000225331.1	AP001055.6	brown	0.778813532
ENSG00000184441.4	AP001062.7	yellow	0.954783527
ENSG00000236304.1	AP001189.4	black	0.642901871
ENSG00000245571.2	AP001258.4	blue	0.719773257
ENSG00000254837.1	AP001372.2	turquoise	0.874508657
ENSG00000272948.1	AP001412.1	grey	0.502607587
ENSG00000242553.1	AP001432.14	turquoise	0.65720553
ENSG00000273210.1	AP001437.1	grey	0.374077705
ENSG00000269038.1	AP001462.6	turquoise	0.812683602
ENSG00000235878.4	AP001468.1	yellow	0.864007677
ENSG00000239415.1	AP001469.9	turquoise	0.808981644
ENSG00000254531.1	AP001816.1	turquoise	0.876989269
ENSG00000256885.1	AP001877.1	blue	0.631324429
ENSG00000237612.1	AP002856.7	grey	0.246150404
ENSG00000255176.1	AP002954.3	brown	0.795901106
ENSG00000224795.1	AP003039.3	brown	0.743617296
ENSG00000254614.1	AP003068.23	turquoise	0.692229596
ENSG00000269089.1	AP003733.1	turquoise	0.667021025
ENSG00000181908.4	AP003774.4	turquoise	0.729095704
ENSG00000215527.3	AP005482.1	grey	0.398608203
ENSG00000263305.1	AP005530.2	grey	0.298604561
ENSG00000226645.1	AP006216.10	turquoise	0.772114433
ENSG00000231611.1	AP006216.11	turquoise	0.868726239
ENSG00000235910.1	AP006216.12	blue	0.654092744
ENSG00000177236.3	AP006621.1	turquoise	0.722215421
ENSG00000255284.1	AP006621.5	turquoise	0.782848367
ENSG00000255142.1	AP006621.6	yellow	0.775757863
ENSG00000138660.7	AP1AR	turquoise	0.933566408
ENSG00000100280.12	AP1B1	turquoise	0.923411778
ENSG00000166747.8	AP1G1	turquoise	0.961427218
ENSG00000213983.7	AP1G2	yellow	0.893729566
ENSG00000072958.4	AP1M1	turquoise	0.944633987
ENSG00000106367.9	AP1S1	turquoise	0.906936059
ENSG00000182287.9	AP1S2	turquoise	0.954344407
ENSG00000152056.12	AP1S3	turquoise	0.863135329
ENSG00000196961.8	AP2A1	turquoise	0.953338876
ENSG00000183020.9	AP2A2	turquoise	0.962134071
ENSG00000006125.12	AP2B1	turquoise	0.977100536
ENSG00000161203.9	AP2M1	turquoise	0.978633389
ENSG00000042753.7	AP2S1	turquoise	0.926536231
ENSG00000132842.9	AP3B1	blue	0.884329122
ENSG00000103723.8	AP3B2	turquoise	0.934605035
ENSG00000065000.11	AP3D1	turquoise	0.967626954

ENSG00000185009.8	AP3M1	blue	0.947074647
ENSG00000070718.7	AP3M2	turquoise	0.961189107
ENSG00000177879.10	AP3S1	turquoise	0.946060134
ENSG00000157823.12	AP3S2	turquoise	0.964850961
ENSG00000134262.8	AP4B1	turquoise	0.91933977
ENSG00000226167.1	AP4B1-AS1	yellow	0.79003164
ENSG00000081014.6	AP4E1	turquoise	0.838636745
ENSG00000221838.5	AP4M1	turquoise	0.860287109
ENSG00000100478.10	AP4S1	turquoise	0.936237125
ENSG00000254470.2	AP5B1	turquoise	0.894116364
ENSG00000053770.7	AP5M1	turquoise	0.935655709
ENSG00000125843.6	AP5S1	turquoise	0.911157679
ENSG00000242802.2	AP5Z1	turquoise	0.880359278
ENSG00000120868.9	APAF1	turquoise	0.9517485
ENSG00000107282.5	APBA1	turquoise	0.945696301
ENSG00000034053.10	APBA2	turquoise	0.930410139
ENSG00000011132.7	APBA3	blue	0.848843646
ENSG00000166313.14	APBB1	turquoise	0.944577261
ENSG00000077420.11	APBB1IP	magenta	0.940252782
ENSG00000163697.12	APBB2	brown	0.859880751
ENSG00000113108.13	APBB3	turquoise	0.909768561
ENSG00000134982.12	APC	turquoise	0.844341967
ENSG00000115266.7	APC2	turquoise	0.837011857
ENSG00000154856.8	APCDD1	green	0.767956821
ENSG00000164062.8	APEH	turquoise	0.929688531
ENSG00000100823.7	APEX1	turquoise	0.96271024
ENSG00000169188.4	APEX2	turquoise	0.924938011
ENSG00000117362.8	APH1A	blue	0.896899781
ENSG00000138613.9	APH1B	turquoise	0.8854608
ENSG00000166181.8	API5	turquoise	0.964989979
ENSG00000149089.8	APIP	blue	0.803469116
ENSG00000175279.17	APITD1	turquoise	0.93322058
ENSG00000171388.9	APLN	brown	0.568781205
ENSG00000134817.9	APLNR	magenta	0.65189272
ENSG00000105290.7	APLP1	brown	0.7646714
ENSG00000084234.12	APLP2	turquoise	0.95605805
ENSG00000101474.7	APMAP	turquoise	0.95750753
ENSG00000118137.5	APOA1	red	0.49786941
ENSG00000163382.7	APOA1BP	red	0.931453504
ENSG00000244509.3	APOBEC3C	magenta	0.782557211
ENSG00000239713.3	APOBEC3G	magenta	0.70824723
ENSG00000184730.6	APOBR	magenta	0.825103784
ENSG00000130208.5	APOC1	grey	0.147435162
ENSG00000214855.5	APOC1P1	grey	0.070549883
ENSG00000234906.4	APOC2	magenta	0.692633272
ENSG00000189058.4	APOD	brown	0.800426046
ENSG00000130203.5	APOE	green	0.648618916
ENSG00000100342.16	APOL1	black	0.853004255

ENSG00000128335.9	APOL2	black	0.805667372
ENSG00000128284.15	APOL3	black	0.786888703
ENSG00000100336.13	APOL4	black	0.756657106
ENSG00000221963.5	APOL6	black	0.809607699
ENSG00000178878.8	APOLD1	black	0.572425761
ENSG00000204444.6	APOM	brown	0.511349535
ENSG00000184831.9	APOO	turquoise	0.924311793
ENSG00000256053.3	APOPT1	turquoise	0.904933035
ENSG00000142192.16	APP	turquoise	0.966153245
ENSG00000062725.5	APPBP2	turquoise	0.948618871
ENSG00000157500.6	APPL1	turquoise	0.903710619
ENSG00000136044.7	APPL2	green	0.929022877
ENSG00000198931.6	APRT	turquoise	0.878618914
ENSG00000137074.14	APTX	turquoise	0.954141672
ENSG00000240583.6	AQP1	black	0.506792517
ENSG00000178301.3	AQP11	turquoise	0.896789611
ENSG00000171885.9	AQP4	green	0.926413054
ENSG00000161798.6	AQP5	grey	0.367787482
ENSG00000086159.8	AQP6	brown	0.500712208
ENSG00000103375.6	AQP8	turquoise	0.560146003
ENSG00000103569.5	AQP9	turquoise	0.504455313
ENSG00000021776.6	AQR	turquoise	0.848277383
ENSG00000078061.8	ARAF	blue	0.935934736
ENSG00000186635.10	ARAP1	brown	0.842458948
ENSG00000047365.7	ARAP2	turquoise	0.843234958
ENSG00000120318.11	ARAP3	yellow	0.813376448
ENSG00000198576.2	ARC	turquoise	0.378071913
ENSG00000095139.9	ARCN1	turquoise	0.950948828
ENSG00000119682.12	AREL1	turquoise	0.964809057
ENSG00000143761.9	ARF1	turquoise	0.968955375
ENSG00000134287.5	ARF3	turquoise	0.953860864
ENSG00000168374.6	ARF4	turquoise	0.950116012
ENSG00000004059.6	ARF5	turquoise	0.938544017
ENSG00000165527.5	ARF6	blue	0.892425036
ENSG00000101199.8	ARFGAP1	turquoise	0.888222523
ENSG00000149182.10	ARFGAP2	turquoise	0.942313666
ENSG00000242247.6	ARFGAP3	blue	0.924191305
ENSG00000066777.4	ARFGEF1	turquoise	0.949592233
ENSG00000124198.8	ARFGEF2	turquoise	0.91697734
ENSG00000164144.10	ARFIP1	blue	0.936597649
ENSG00000132254.8	ARFIP2	turquoise	0.96243649
ENSG00000101246.15	ARFRP1	turquoise	0.916871622
ENSG00000081181.3	ARG2	turquoise	0.905715878
ENSG00000134884.9	ARGLU1	turquoise	0.938509238
ENSG00000175220.7	ARHGAP1	brown	0.846156234
ENSG00000071205.7	ARHGAP10	brown	0.672004274
ENSG00000165322.13	ARHGAP12	blue	0.899702979
ENSG00000140750.12	ARHGAP17	blue	0.89510866

ENSG00000146376.6	ARHGAP18	pink	0.826247921
ENSG00000213390.6	ARHGAP19	blue	0.838250476
ENSG00000137727.8	ARHGAP20	turquoise	0.8876923
ENSG00000107863.12	ARHGAP21	brown	0.902694913
ENSG00000128805.10	ARHGAP22	brown	0.820173599
ENSG00000225485.3	ARHGAP23	brown	0.915945642
ENSG00000138639.13	ARHGAP24	turquoise	0.773888414
ENSG00000163219.7	ARHGAP25	magenta	0.873503404
ENSG00000145819.11	ARHGAP26	turquoise	0.924229953
ENSG00000159314.7	ARHGAP27	turquoise	0.880709454
ENSG00000137962.8	ARHGAP29	black	0.702792924
ENSG00000186517.9	ARHGAP30	magenta	0.950397484
ENSG00000031081.6	ARHGAP31	green	0.825687543
ENSG00000134909.14	ARHGAP32	turquoise	0.939188585
ENSG00000004777.14	ARHGAP33	turquoise	0.910254364
ENSG00000160007.13	ARHGAP35	turquoise	0.875160423
ENSG00000147799.7	ARHGAP39	pink	0.867798259
ENSG00000089820.11	ARHGAP4	yellow	0.747852988
ENSG00000165895.13	ARHGAP42	green	0.72630214
ENSG00000006740.12	ARHGAP44	turquoise	0.944760261
ENSG00000100852.8	ARHGAP5	green	0.804168818
ENSG00000258655.1	ARHGAP5-AS1	green	0.724291262
ENSG00000123329.13	ARHGAP9	magenta	0.896230471
ENSG00000141522.7	ARHGDIA	turquoise	0.941632384
ENSG00000111348.4	ARHGDIB	magenta	0.901552804
ENSG00000242173.4	ARHGDIG	turquoise	0.835521116
ENSG00000076928.13	ARHGEF1	yellow	0.836615115
ENSG00000104728.11	ARHGEF10	blue	0.807957123
ENSG00000074964.12	ARHGEF10L	turquoise	0.846428231
ENSG00000132694.14	ARHGEF11	turquoise	0.961634947
ENSG00000196914.4	ARHGEF12	blue	0.878815589
ENSG00000198844.6	ARHGEF15	black	0.606876411
ENSG00000130762.10	ARHGEF16	blue	0.483064245
ENSG00000110237.3	ARHGEF17	turquoise	0.946493473
ENSG00000104880.13	ARHGEF18	turquoise	0.876758239
ENSG00000142632.12	ARHGEF19	blue	0.524627634
ENSG00000116584.13	ARHGEF2	brown	0.81064057
ENSG00000240771.2	ARHGEF25	turquoise	0.895378896
ENSG00000114790.8	ARHGEF26	green	0.882744621
ENSG00000243069.3	ARHGEF26-AS1	green	0.755443803
ENSG00000163947.7	ARHGEF3	turquoise	0.920208805
ENSG00000214694.6	ARHGEF33	turquoise	0.905292014
ENSG00000183111.7	ARHGEF37	brown	0.838632387
ENSG00000136002.12	ARHGEF4	turquoise	0.929779092
ENSG00000165801.5	ARHGEF40	blue	0.84409454
ENSG00000129675.11	ARHGEF6	green	0.874895056
ENSG00000102606.13	ARHGEF7	turquoise	0.967738015
ENSG00000235875.3	ARHGEF7-AS2	turquoise	0.732333778

ENSG00000131089.9	ARHGEF9	turquoise	0.943201972
ENSG00000117713.13	ARID1A	turquoise	0.934057864
ENSG00000049618.17	ARID1B	turquoise	0.969154658
ENSG00000189079.11	ARID2	turquoise	0.804056316
ENSG00000116017.6	ARID3A	blue	0.741585793
ENSG00000179361.13	ARID3B	yellow	0.906545616
ENSG00000032219.14	ARID4A	turquoise	0.944117162
ENSG00000054267.16	ARID4B	turquoise	0.917574749
ENSG00000196843.11	ARID5A	black	0.791123167
ENSG00000150347.10	ARID5B	turquoise	0.776754947
ENSG00000166233.8	ARIH1	turquoise	0.961456343
ENSG00000177479.15	ARIH2	turquoise	0.931731737
ENSG00000221883.2	ARIH2OS	blue	0.674052263
ENSG00000120805.9	ARL1	turquoise	0.974575016
ENSG00000175414.6	ARL10	turquoise	0.920211332
ENSG00000169379.11	ARL13B	blue	0.888686433
ENSG00000152219.4	ARL14EP	turquoise	0.958068464
ENSG00000185305.6	ARL15	turquoise	0.878561895
ENSG00000214087.4	ARL16	turquoise	0.910942614
ENSG00000213465.3	ARL2	turquoise	0.902023691
ENSG00000102931.3	ARL2BP	turquoise	0.967769829
ENSG00000138175.8	ARL3	turquoise	0.949932781
ENSG00000122644.8	ARL4A	turquoise	0.891189539
ENSG00000188042.5	ARL4C	blue	0.723493348
ENSG00000175906.4	ARL4D	turquoise	0.779332948
ENSG00000162980.12	ARL5A	turquoise	0.928199884
ENSG00000165997.4	ARL5B	turquoise	0.807146901
ENSG00000113966.5	ARL6	turquoise	0.959099364
ENSG00000170540.10	ARL6IP1	turquoise	0.929691149
ENSG00000182196.9	ARL6IP4	red	0.964870142
ENSG00000144746.6	ARL6IP5	turquoise	0.944747194
ENSG00000177917.6	ARL6IP6	green	0.831732638
ENSG00000143862.3	ARL8A	turquoise	0.931466073
ENSG00000134108.8	ARL8B	turquoise	0.963634426
ENSG00000196503.2	ARL9	turquoise	0.85760625
ENSG00000104442.5	ARMC1	turquoise	0.926531043
ENSG00000170632.9	ARMC10	turquoise	0.946831051
ENSG00000157343.4	ARMC12	pink	0.768592304
ENSG00000118690.8	ARMC2	pink	0.931488721
ENSG00000140691.12	ARMC5	turquoise	0.893287236
ENSG00000105676.9	ARMC6	turquoise	0.938492994
ENSG00000125449.2	ARMC7	turquoise	0.918520731
ENSG00000114098.13	ARMC8	turquoise	0.976063116
ENSG00000135931.13	ARMC9	turquoise	0.938809299
ENSG00000126947.7	ARMCX1	turquoise	0.97443409
ENSG00000184867.9	ARMCX2	turquoise	0.930508694
ENSG00000102401.15	ARMCX3	turquoise	0.954118476
ENSG00000228275.1	ARMCX3-AS1	grey	0.377164926

ENSG00000196440.7	ARMCX4	turquoise	0.926866351
ENSG00000125962.10	ARMCX5	turquoise	0.896404676
ENSG00000198960.6	ARMCX6	blue	0.911221448
ENSG00000143437.16	ARNT	blue	0.948061262
ENSG00000172379.14	ARNT2	turquoise	0.946467053
ENSG00000133794.13	ARNTL	turquoise	0.874464607
ENSG00000029153.10	ARNTL2	turquoise	0.782021695
ENSG00000241685.4	ARPC1A	turquoise	0.960194693
ENSG00000130429.8	ARPC1B	magenta	0.882792491
ENSG00000163466.11	ARPC2	turquoise	0.962662553
ENSG00000111229.11	ARPC3	turquoise	0.950926227
ENSG00000241553.8	ARPC4	turquoise	0.960363328
ENSG00000162704.11	ARPC5	turquoise	0.925635547
ENSG00000136950.9	ARPC5L	turquoise	0.941731833
ENSG00000128989.6	ARPP19	turquoise	0.887996343
ENSG00000172995.12	ARPP21	turquoise	0.856060802
ENSG00000137486.12	ARRB1	turquoise	0.794444356
ENSG00000141480.13	ARRB2	turquoise	0.876097785
ENSG00000197070.9	ARRDC1	yellow	0.933359478
ENSG00000105643.5	ARRDC2	brown	0.668407012
ENSG00000113369.4	ARRDC3	black	0.743310272
ENSG00000140450.7	ARRDC4	blue	0.704006688
ENSG00000100299.13	ARSA	yellow	0.930374075
ENSG00000113273.11	ARSB	turquoise	0.907263588
ENSG00000006756.11	ARSD	blue	0.806396721
ENSG00000157399.10	ARSE	green	0.531171449
ENSG00000062096.10	ARSF	green	0.428476308
ENSG00000232226.2	ARSFP1	grey	0.312000664
ENSG00000141337.8	ARSG	turquoise	0.790360439
ENSG00000164291.12	ARSK	turquoise	0.886000509
ENSG00000156219.12	ART3	turquoise	0.757638141
ENSG00000173409.9	ARV1	turquoise	0.95693863
ENSG00000099889.9	ARVCF	turquoise	0.78648212
ENSG00000004848.6	ARX	turquoise	0.868606839
ENSG00000214435.3	AS3MT	blue	0.862799718
ENSG00000104763.13	ASAH1	blue	0.934916488
ENSG00000204147.5	ASAH2B	turquoise	0.905725537
ENSG00000153317.10	ASAP1	turquoise	0.883167629
ENSG00000088280.14	ASAP3	blue	0.722118628
ENSG00000065802.7	ASB1	turquoise	0.844211302
ENSG00000196372.8	ASB13	turquoise	0.913564159
ENSG00000161664.2	ASB16	turquoise	0.695544128
ENSG00000267080.1	ASB16-AS1	yellow	0.779519722
ENSG00000005981.8	ASB4	green	0.496639848
ENSG00000148331.7	ASB6	turquoise	0.947319358
ENSG00000183475.8	ASB7	turquoise	0.964099166
ENSG00000177981.6	ASB8	turquoise	0.950225432
ENSG00000138303.13	ASCC1	turquoise	0.829456613

ENSG00000100325.10	ASCC2	blue	0.875668555
ENSG00000112249.9	ASCC3	turquoise	0.904389894
ENSG00000139352.3	ASCL1	grey	0.228816437
ENSG00000111875.7	ASF1A	blue	0.854891226
ENSG00000141505.7	ASGR1	turquoise	0.78769585
ENSG00000116539.6	ASH1L	turquoise	0.942007924
ENSG00000235919.3	ASH1L-AS1	turquoise	0.865619219
ENSG00000129691.11	ASH2L	turquoise	0.975074197
ENSG00000110881.7	ASIC1	turquoise	0.810915721
ENSG00000108684.10	ASIC2	turquoise	0.855907479
ENSG00000213199.3	ASIC3	yellow	0.904754467
ENSG00000072182.8	ASIC4	greenyellow	0.749152929
ENSG00000126522.12	ASL	blue	0.857113262
ENSG00000244723.3	ASLP1	yellow	0.742832432
ENSG00000169093.10	ASMTL	turquoise	0.925471186
ENSG00000236017.3	ASMTL-AS1	yellow	0.828224812
ENSG00000198356.7	ASNA1	turquoise	0.956812621
ENSG00000070669.12	ASNS	turquoise	0.91679131
ENSG00000138381.5	ASNSD1	turquoise	0.922066529
ENSG00000108381.6	ASPA	brown	0.836709666
ENSG00000204653.5	ASPDH	turquoise	0.687752792
ENSG00000198363.11	ASPH	blue	0.883250658
ENSG00000174939.6	ASPHD1	turquoise	0.694656883
ENSG00000128203.6	ASPHD2	turquoise	0.905412306
ENSG00000106819.7	ASPN	brown	0.416419528
ENSG00000244617.1	ASPRV1	blue	0.759793639
ENSG00000169696.11	ASPSCR1	red	0.918052462
ENSG00000162174.8	ASRGL1	green	0.588896813
ENSG00000130707.13	ASS1	turquoise	0.823648302
ENSG00000034533.7	ASTE1	turquoise	0.873922249
ENSG00000152092.11	ASTN1	turquoise	0.907597916
ENSG00000148219.12	ASTN2	blue	0.808737519
ENSG00000064102.10	ASUN	turquoise	0.815165367
ENSG00000171456.12	ASXL1	turquoise	0.891893259
ENSG00000143970.12	ASXL2	turquoise	0.872363899
ENSG00000138138.9	ATAD1	turquoise	0.964000867
ENSG00000156802.8	ATAD2	turquoise	0.885689448
ENSG00000119778.10	ATAD2B	turquoise	0.795222091
ENSG00000197785.9	ATAD3A	turquoise	0.888796564
ENSG00000160072.15	ATAD3B	yellow	0.907904084
ENSG00000215915.5	ATAD3C	blue	0.522486548
ENSG00000137343.13	ATAT1	turquoise	0.958365859
ENSG00000167654.13	ATCAY	turquoise	0.915721723
ENSG00000107669.13	ATE1	turquoise	0.9681698
ENSG00000123268.4	ATF1	blue	0.840367826
ENSG00000115966.12	ATF2	turquoise	0.955501161
ENSG00000162772.12	ATF3	black	0.618508216
ENSG00000128272.10	ATF4	cyan	0.878980149

ENSG00000169136.4	ATF5	turquoise	0.907453469
ENSG00000118217.5	ATF6	turquoise	0.917555096
ENSG00000213676.6	ATF6B	turquoise	0.927089737
ENSG00000170653.14	ATF7	blue	0.930148442
ENSG00000166669.9	ATF7IP2	turquoise	0.847361434
ENSG00000152348.11	ATG10	turquoise	0.905716156
ENSG00000145782.8	ATG12	turquoise	0.959894817
ENSG00000175224.12	ATG13	turquoise	0.941731163
ENSG00000126775.8	ATG14	turquoise	0.927353627
ENSG00000085978.17	ATG16L1	turquoise	0.956423823
ENSG00000168010.6	ATG16L2	yellow	0.889424914
ENSG00000110046.8	ATG2A	turquoise	0.918769588
ENSG00000066739.7	ATG2B	turquoise	0.966970784
ENSG00000144848.6	ATG3	turquoise	0.805989466
ENSG00000101844.13	ATG4A	turquoise	0.896340328
ENSG00000168397.12	ATG4B	turquoise	0.931334789
ENSG00000125703.10	ATG4C	brown	0.900804968
ENSG00000130734.5	ATG4D	turquoise	0.911049523
ENSG00000057663.8	ATG5	turquoise	0.943897234
ENSG00000197548.8	ATG7	turquoise	0.904858243
ENSG00000198925.6	ATG9A	turquoise	0.968152206
ENSG00000181652.14	ATG9B	turquoise	0.685839904
ENSG00000142102.11	ATHL1	blue	0.565090899
ENSG00000138363.10	ATIC	turquoise	0.932254017
ENSG00000198513.7	ATL1	turquoise	0.961798279
ENSG00000119787.9	ATL2	turquoise	0.928079112
ENSG00000184743.8	ATL3	blue	0.92223492
ENSG00000149311.13	ATM	turquoise	0.8555406
ENSG00000166454.5	ATMIN	turquoise	0.948698311
ENSG00000111676.10	ATN1	turquoise	0.869767698
ENSG00000179774.7	ATOH7	turquoise	0.823306099
ENSG00000168874.8	ATOH8	black	0.748734005
ENSG00000177556.7	ATOX1	red	0.9487371
ENSG00000206190.7	ATP10A	black	0.507740518
ENSG00000118322.8	ATP10B	brown	0.87856723
ENSG00000145246.9	ATP10D	turquoise	0.781251486
ENSG00000068650.14	ATP11A	turquoise	0.835360368
ENSG00000058063.11	ATP11B	turquoise	0.825388356
ENSG00000101974.10	ATP11C	turquoise	0.913093175
ENSG00000105726.12	ATP13A1	turquoise	0.912888286
ENSG00000159363.13	ATP13A2	turquoise	0.930425504
ENSG00000133657.10	ATP13A3	turquoise	0.907881348
ENSG00000127249.10	ATP13A4	green	0.901260557
ENSG00000187527.6	ATP13A5	green	0.582969005
ENSG00000163399.11	ATP1A1	turquoise	0.954705491
ENSG00000203865.5	ATP1A1OS	blue	0.771642828
ENSG00000018625.10	ATP1A2	green	0.860683711
ENSG00000105409.11	ATP1A3	turquoise	0.903912577

ENSG00000143153.8	ATP1B1	turquoise	0.933507454
ENSG00000129244.4	ATP1B2	green	0.830419855
ENSG00000069849.6	ATP1B3	turquoise	0.906297922
ENSG00000244124.1	ATP1B3-AS1	turquoise	0.770743047
ENSG00000174437.12	ATP2A2	turquoise	0.948527309
ENSG00000074370.13	ATP2A3	brown	0.495755291
ENSG00000070961.10	ATP2B1	turquoise	0.880040228
ENSG00000157087.12	ATP2B2	turquoise	0.899095209
ENSG00000067842.13	ATP2B3	turquoise	0.928045155
ENSG00000058668.10	ATP2B4	turquoise	0.78722499
ENSG00000017260.15	ATP2C1	turquoise	0.910059398
ENSG00000064270.8	ATP2C2	grey	0.342182366
ENSG00000152234.11	ATP5A1	turquoise	0.946966741
ENSG00000110955.4	ATP5B	turquoise	0.950472036
ENSG00000165629.15	ATP5C1	turquoise	0.942138482
ENSG00000099624.3	ATP5D	red	0.888344154
ENSG00000124172.5	ATP5E	red	0.961764908
ENSG00000116459.6	ATP5F1	turquoise	0.924923494
ENSG00000254944.1	ATP5F1P5	turquoise	0.779140482
ENSG00000159199.9	ATP5G1	turquoise	0.916781884
ENSG00000135390.13	ATP5G2	turquoise	0.897732223
ENSG00000154518.5	ATP5G3	turquoise	0.948402213
ENSG00000167863.7	ATP5H	turquoise	0.926712035
ENSG00000169020.5	ATP5I	red	0.901092745
ENSG00000154723.8	ATP5J	turquoise	0.896972144
ENSG00000241468.3	ATP5J2	red	0.947067487
ENSG00000167283.3	ATP5L	turquoise	0.936691834
ENSG00000241837.2	ATP5O	turquoise	0.912524349
ENSG00000125375.10	ATP5S	blue	0.866574683
ENSG00000105341.14	ATP5SL	turquoise	0.923881129
ENSG00000071553.12	ATP6AP1	turquoise	0.969514537
ENSG00000205464.7	ATP6AP1L	yellow	0.787703336
ENSG00000182220.9	ATP6AP2	turquoise	0.974222819
ENSG00000033627.10	ATP6V0A1	turquoise	0.970942368
ENSG00000185344.9	ATP6V0A2	turquoise	0.918001766
ENSG00000117410.9	ATP6V0B	turquoise	0.940459653
ENSG00000185883.6	ATP6V0C	turquoise	0.934394267
ENSG00000159720.7	ATP6V0D1	turquoise	0.951458136
ENSG00000113732.4	ATP6V0E1	blue	0.767576422
ENSG00000171130.13	ATP6V0E2	turquoise	0.820644154
ENSG00000204934.6	ATP6V0E2-AS1	green	0.644958777
ENSG00000114573.5	ATP6V1A	turquoise	0.967278768
ENSG00000147416.6	ATP6V1B2	turquoise	0.96825629
ENSG00000155097.7	ATP6V1C1	turquoise	0.977278351
ENSG00000143882.5	ATP6V1C2	turquoise	0.658180588
ENSG00000100554.7	ATP6V1D	turquoise	0.967265061
ENSG00000131100.8	ATP6V1E1	turquoise	0.965782487
ENSG00000250565.2	ATP6V1E2	turquoise	0.907628328

ENSG00000128524.4	ATP6V1F	turquoise	0.92392696
ENSG00000136888.6	ATP6V1G1	turquoise	0.903240345
ENSG00000213760.6	ATP6V1G2	turquoise	0.92936862
ENSG00000047249.12	ATP6V1H	turquoise	0.958576684
ENSG00000165240.13	ATP7A	turquoise	0.864033742
ENSG00000123191.9	ATP7B	turquoise	0.852483271
ENSG00000124406.12	ATP8A1	turquoise	0.9345157
ENSG00000132932.12	ATP8A2	turquoise	0.929020534
ENSG00000143515.12	ATP8B2	turquoise	0.91092048
ENSG00000054793.9	ATP9A	turquoise	0.972184547
ENSG00000166377.15	ATP9B	turquoise	0.921059176
ENSG00000123472.8	ATPAF1	blue	0.836261523
ENSG00000171953.11	ATPAF2	blue	0.849976825
ENSG00000130770.13	ATPIF1	turquoise	0.945243387
ENSG00000175054.10	ATR	turquoise	0.956756878
ENSG00000138085.12	ATRAID	turquoise	0.865594637
ENSG00000164053.13	ATRIP	turquoise	0.933088905
ENSG00000088812.13	ATRN	turquoise	0.965857796
ENSG00000107518.12	ATRNL1	turquoise	0.909755271
ENSG00000085224.16	ATRX	turquoise	0.939815345
ENSG00000124788.13	ATXN1	turquoise	0.943034579
ENSG00000130638.11	ATXN10	turquoise	0.968913055
ENSG00000224470.3	ATXN1L	brown	0.820088903
ENSG00000204842.10	ATXN2	turquoise	0.931199922
ENSG00000168488.14	ATXN2L	turquoise	0.904846694
ENSG00000066427.17	ATXN3	turquoise	0.934115339
ENSG00000163635.13	ATXN7	turquoise	0.936113867
ENSG00000146776.10	ATXN7L1	turquoise	0.918156019
ENSG00000162650.11	ATXN7L2	yellow	0.897475335
ENSG00000087152.11	ATXN7L3	turquoise	0.954646166
ENSG00000253719.2	ATXN7L3B	turquoise	0.978739352
ENSG00000148090.7	AUH	turquoise	0.963803361
ENSG00000115307.12	AUP1	blue	0.837689929
ENSG00000087586.13	AURKA	turquoise	0.766730192
ENSG00000175756.9	AURKAIP1	red	0.959554809
ENSG00000105146.8	AURKC	blue	0.6204778
ENSG00000158321.11	AUTS2	turquoise	0.863802975
ENSG00000169857.3	AVEN	red	0.735264365
ENSG00000135407.6	AVIL	green	0.537648283
ENSG00000105778.13	AVL9	turquoise	0.966986907
ENSG00000119986.6	AVPI1	turquoise	0.926878433
ENSG00000103126.10	AXIN1	turquoise	0.844476634
ENSG00000168646.8	AXIN2	blue	0.844166098
ENSG00000167601.7	AXL	green	0.851006315
ENSG00000160862.8	AZGP1	brown	0.566617584
ENSG00000141577.9	AZI1	yellow	0.877156077
ENSG00000163512.9	AZI2	turquoise	0.939621501
ENSG00000155096.9	AZIN1	turquoise	0.859893802

ENSG00000172232.5	AZU1	turquoise	0.400896492
ENSG00000166710.13	B2M	black	0.791932492
ENSG00000169255.9	B3GALNT1	turquoise	0.949511195
ENSG00000172318.4	B3GALT1	turquoise	0.896531109
ENSG00000162630.5	B3GALT2	turquoise	0.836870591
ENSG00000235863.2	B3GALT4	turquoise	0.856231889
ENSG00000183778.13	B3GALT5	turquoise	0.86753293
ENSG00000176022.3	B3GALT6	turquoise	0.905085024
ENSG00000187676.7	B3GALTL	turquoise	0.896256535
ENSG00000109956.8	B3GAT1	turquoise	0.862380498
ENSG00000112309.6	B3GAT2	turquoise	0.784675363
ENSG00000149541.5	B3GAT3	turquoise	0.896952747
ENSG00000174684.6	B3GNT1	turquoise	0.95788545
ENSG00000170340.10	B3GNT2	turquoise	0.886608092
ENSG00000176383.8	B3GNT4	turquoise	0.897348298
ENSG00000156966.6	B3GNT7	brown	0.552922682
ENSG00000237172.3	B3GNT9	blue	0.758935458
ENSG00000175711.4	B3GNTL1	turquoise	0.91074352
ENSG00000135454.9	B4GALNT1	turquoise	0.927616858
ENSG00000139044.6	B4GALNT3	turquoise	0.770928128
ENSG00000182272.7	B4GALNT4	turquoise	0.834373086
ENSG00000086062.8	B4GALT1	black	0.893759882
ENSG00000117411.12	B4GALT2	turquoise	0.84707571
ENSG00000158850.10	B4GALT3	turquoise	0.935494521
ENSG00000121578.8	B4GALT4	blue	0.908313619
ENSG00000158470.5	B4GALT5	turquoise	0.942884421
ENSG00000118276.7	B4GALT6	turquoise	0.907048738
ENSG00000027847.9	B4GALT7	turquoise	0.879865298
ENSG00000108641.10	B9D1	turquoise	0.918350573
ENSG00000123810.3	B9D2	turquoise	0.863544308
ENSG00000164929.12	BAALC	green	0.674840576
ENSG00000105393.11	BABAM1	turquoise	0.949680211
ENSG00000186318.12	BACE1	brown	0.864643124
ENSG00000182240.11	BACE2	black	0.817759545
ENSG00000156273.11	BACH1	blue	0.750795243
ENSG00000112182.10	BACH2	turquoise	0.8492526
ENSG00000002330.9	BAD	red	0.910444074
ENSG00000107262.12	BAG1	turquoise	0.845354913
ENSG00000112208.10	BAG2	turquoise	0.808854562
ENSG00000151929.5	BAG3	black	0.65025484
ENSG00000156735.6	BAG4	turquoise	0.960059365
ENSG00000166170.9	BAG5	turquoise	0.969938473
ENSG00000204463.8	BAG6	turquoise	0.95953551
ENSG00000140320.7	BAHD1	turquoise	0.94666737
ENSG00000181790.6	BAI1	turquoise	0.785929875
ENSG00000121753.8	BAI2	turquoise	0.894086213
ENSG00000135298.9	BAI3	turquoise	0.933289961
ENSG00000175866.11	BAIAP2	turquoise	0.917499703

ENSG00000226137.3	BAIAP2-AS1	turquoise	0.909306972
ENSG00000006453.9	BAIAP2L1	black	0.686375007
ENSG00000128298.12	BAIAP2L2	yellow	0.886490003
ENSG00000030110.8	BAK1	turquoise	0.831969677
ENSG00000175334.3	BANF1	turquoise	0.855602687
ENSG00000172530.15	BANP	cyan	0.879579624
ENSG00000163930.5	BAP1	turquoise	0.919654299
ENSG00000138376.6	BARD1	blue	0.733533984
ENSG00000176788.7	BASP1	turquoise	0.919621287
ENSG00000156127.6	BATF	midnightblue	0.707707001
ENSG00000168062.5	BATF2	black	0.674401462
ENSG00000123685.4	BATF3	turquoise	0.848067468
ENSG00000087088.15	BAX	turquoise	0.817033117
ENSG00000198604.6	BAZ1A	blue	0.770524574
ENSG00000009954.6	BAZ1B	turquoise	0.888217817
ENSG00000076108.7	BAZ2A	blue	0.906421762
ENSG00000123636.13	BAZ2B	turquoise	0.747844929
ENSG00000105327.11	BBC3	red	0.602913814
ENSG00000214413.3	BBIP1	turquoise	0.928345295
ENSG00000129151.4	BBOX1	green	0.798056893
ENSG00000174483.15	BBS1	turquoise	0.943491542
ENSG00000179941.6	BBS10	turquoise	0.878514297
ENSG00000181004.5	BBS12	turquoise	0.917149001
ENSG00000125124.7	BBS2	blue	0.882563044
ENSG00000140463.9	BBS4	blue	0.911784888
ENSG00000163093.7	BBS5	pink	0.910970996
ENSG00000138686.5	BBS7	turquoise	0.943609009
ENSG00000122507.16	BBS9	blue	0.904462156
ENSG00000114439.14	BBX	blue	0.91097899
ENSG00000187244.6	BCAM	green	0.564256565
ENSG00000132692.14	BCAN	green	0.752304008
ENSG00000075790.6	BCAP29	turquoise	0.938105536
ENSG00000185825.11	BCAP31	turquoise	0.90145159
ENSG00000050820.12	BCAR1	blue	0.835561721
ENSG00000137936.12	BCAR3	green	0.842972402
ENSG00000064787.8	BCAS1	brown	0.49269584
ENSG00000116752.5	BCAS2	turquoise	0.929972677
ENSG00000141376.16	BCAS3	turquoise	0.955438044
ENSG00000124243.13	BCAS4	turquoise	0.888729586
ENSG00000060982.10	BCAT1	turquoise	0.846005113
ENSG00000105552.10	BCAT2	yellow	0.830462009
ENSG00000107949.12	BCCIP	turquoise	0.875790289
ENSG00000186666.4	BCDIN3D	blue	0.819569568
ENSG00000258057.1	BCDIN3D-AS1	turquoise	0.923892769
ENSG00000114200.5	BCHE	brown	0.702077206
ENSG00000248098.6	BCKDHA	blue	0.8011125
ENSG00000083123.10	BCKDHB	blue	0.829267889
ENSG00000103507.9	BCKDK	turquoise	0.906017965

ENSG00000142867.8	BCL10	cyan	0.901424638
ENSG00000119866.16	BCL11A	turquoise	0.869552296
ENSG00000127152.13	BCL11B	turquoise	0.852617924
ENSG00000171791.10	BCL2	green	0.886089255
ENSG00000140379.7	BCL2A1	magenta	0.661986071
ENSG00000171552.8	BCL2L1	turquoise	0.914875349
ENSG00000137875.4	BCL2L10	turquoise	0.851125512
ENSG00000153094.17	BCL2L11	blue	0.800026368
ENSG00000126453.5	BCL2L12	blue	0.674097433
ENSG00000099968.13	BCL2L13	turquoise	0.889008387
ENSG00000129473.5	BCL2L2	brown	0.798376654
ENSG00000258643.1	BCL2L2-PABPN1	blue	0.533751269
ENSG00000069399.8	BCL3	black	0.825750845
ENSG00000113916.13	BCL6	black	0.78616978
ENSG00000161940.6	BCL6B	black	0.688303661
ENSG00000110987.4	BCL7A	turquoise	0.963231221
ENSG00000106635.3	BCL7B	turquoise	0.947207805
ENSG00000099385.7	BCL7C	red	0.847930105
ENSG00000116128.5	BCL9	blue	0.896665068
ENSG00000186174.8	BCL9L	turquoise	0.844674945
ENSG00000029363.11	BCLAF1	turquoise	0.964026163
ENSG00000197580.7	BCO2	pink	0.891122822
ENSG00000183337.12	BCOR	blue	0.837146065
ENSG00000085185.11	BCORL1	turquoise	0.873785732
ENSG00000186716.15	BCR	turquoise	0.894339187
ENSG00000215481.4	BCRP3	turquoise	0.709610899
ENSG00000074582.8	BCS1L	turquoise	0.934804003
ENSG00000236824.1	BCYRN1	grey	0.01838389
ENSG00000161267.7	BDH1	turquoise	0.918423182
ENSG00000164039.10	BDH2	green	0.80891434
ENSG00000245573.3	BDNF-AS	brown	0.754069849
ENSG00000145734.14	BDP1	turquoise	0.938721825
ENSG00000126581.8	BECN1	turquoise	0.931779475
ENSG00000183092.11	BEGAIN	turquoise	0.841048277
ENSG00000162373.8	BEND5	turquoise	0.888899778
ENSG00000151917.13	BEND6	turquoise	0.907863238
ENSG00000165626.12	BEND7	turquoise	0.821789175
ENSG00000167995.11	BEST1	brown	0.835516514
ENSG00000142959.4	BEST4	pink	0.908894335
ENSG00000105829.7	BET1	turquoise	0.909869195
ENSG00000177951.13	BET1L	turquoise	0.8931476
ENSG00000133169.5	BEX1	turquoise	0.931148653
ENSG00000133134.7	BEX2	turquoise	0.923026212
ENSG00000102409.9	BEX4	turquoise	0.959393583
ENSG00000184515.6	BEX5	turquoise	0.905773643
ENSG00000103429.6	BFAR	turquoise	0.951231634
ENSG00000125864.7	BFSP1	turquoise	0.880643547
ENSG00000242252.1	BGLAP	red	0.69522879

ENSG00000182492.11	BGN	black	0.626842363
ENSG00000198908.7	BHLHB9	turquoise	0.965284388
ENSG00000180828.1	BHLHE22	grey	0.244081129
ENSG00000134107.4	BHLHE40	turquoise	0.743624958
ENSG00000235831.2	BHLHE40-AS1	turquoise	0.862300516
ENSG00000123095.5	BHLHE41	turquoise	0.874634766
ENSG00000132840.5	BHMT2	green	0.841672873
ENSG00000122870.7	BICC1	green	0.644791546
ENSG00000151746.9	BICD1	turquoise	0.886832638
ENSG00000185963.9	BICD2	turquoise	0.946263692
ENSG00000015475.14	BID	turquoise	0.893508281
ENSG00000136717.10	BIN1	blue	0.717575006
ENSG00000110934.6	BIN2	magenta	0.885586777
ENSG00000147439.7	BIN3	turquoise	0.921117955
ENSG00000110330.4	BIRC2	turquoise	0.861598633
ENSG00000023445.9	BIRC3	cyan	0.788715648
ENSG00000115760.9	BIRC6	turquoise	0.88746283
ENSG00000134897.9	BIVM	turquoise	0.951599963
ENSG00000166619.8	BLCAP	turquoise	0.909744971
ENSG00000197299.6	BLM	greenyellow	0.666151833
ENSG00000108578.10	BLMH	turquoise	0.953733813
ENSG00000095585.12	BLNK	magenta	0.773756195
ENSG00000135441.3	BLOC1S1	red	0.936268561
ENSG00000196072.7	BLOC1S2	turquoise	0.907427612
ENSG00000189114.6	BLOC1S3	turquoise	0.936098606
ENSG00000186222.3	BLOC1S4	turquoise	0.946769108
ENSG00000188428.12	BLOC1S5	blue	0.892745149
ENSG00000104164.6	BLOC1S6	turquoise	0.964479229
ENSG00000106605.6	BLVRA	turquoise	0.868153004
ENSG00000090013.5	BLVRB	turquoise	0.841846669
ENSG00000117475.9	BLZF1	turquoise	0.895372579
ENSG00000168283.9	BMI1	turquoise	0.841314534
ENSG00000168487.13	BMP1	yellow	0.839555459
ENSG00000125845.6	BMP2	blue	0.671412144
ENSG00000138756.13	BMP2K	turquoise	0.780453779
ENSG00000153162.8	BMP6	turquoise	0.694178921
ENSG00000101144.8	BMP7	green	0.861151942
ENSG00000183682.7	BMP8A	turquoise	0.847555557
ENSG00000116985.6	BMP8B	turquoise	0.785064073
ENSG00000164619.4	BMPER	turquoise	0.806405571
ENSG00000107779.7	BMPR1A	blue	0.88543669
ENSG00000138696.6	BMPR1B	green	0.8914134
ENSG00000204217.8	BMPR2	turquoise	0.944420723
ENSG00000165733.7	BMS1	turquoise	0.965017471
ENSG00000237238.2	BMS1P10	turquoise	0.797514069
ENSG00000240089.2	BMS1P3	blue	0.518538239
ENSG00000113734.13	BNIP1	turquoise	0.902766796
ENSG00000140299.7	BNIP2	blue	0.934771172

ENSG00000176171.7	BNIP3	blue	0.874455428
ENSG00000104765.10	BNIP3L	turquoise	0.859150276
ENSG00000144857.10	BOC	green	0.668702487
ENSG00000145919.6	BOD1	turquoise	0.948482304
ENSG00000038219.8	BOD1L1	turquoise	0.945627052
ENSG00000176720.3	BOK	brown	0.843929055
ENSG00000178096.8	BOLA1	red	0.924057133
ENSG00000183336.7	BOLA2	turquoise	0.690063649
ENSG00000169627.7	BOLA2B	red	0.653352869
ENSG00000163170.7	BOLA3	turquoise	0.89459841
ENSG00000225439.2	BOLA3-AS1	turquoise	0.942871579
ENSG00000170727.7	BOP1	turquoise	0.878901656
ENSG00000136122.11	BORA	turquoise	0.834193585
ENSG00000172331.7	BPGM	blue	0.86437911
ENSG00000137274.8	BPHL	turquoise	0.897148505
ENSG00000162813.13	BPNT1	turquoise	0.97085718
ENSG00000171634.12	BPTF	turquoise	0.91052001
ENSG00000157764.8	BRAF	turquoise	0.974824119
ENSG00000089234.11	BRAP	turquoise	0.954782454
ENSG00000106009.11	BRAT1	turquoise	0.87478398
ENSG00000012048.15	BRCA1	brown	0.855432571
ENSG00000185515.10	BRCC3	turquoise	0.964784007
ENSG00000100425.14	BRD1	turquoise	0.941398313
ENSG00000204256.8	BRD2	turquoise	0.951160056
ENSG00000169925.12	BRD3	blue	0.846475084
ENSG00000141867.13	BRD4	blue	0.852158673
ENSG00000166164.11	BRD7	turquoise	0.868032409
ENSG00000112983.13	BRD8	turquoise	0.954474873
ENSG00000028310.13	BRD9	turquoise	0.889400055
ENSG00000158019.16	BRE	turquoise	0.95620672
ENSG00000185024.11	BRF1	turquoise	0.927440642
ENSG00000104221.8	BRF2	turquoise	0.916237667
ENSG00000164713.5	BRI3	red	0.929560953
ENSG00000184992.10	BRI3BP	turquoise	0.946192722
ENSG00000182685.3	BRICD5	yellow	0.926875167
ENSG00000078725.8	BRINP1	turquoise	0.925450724
ENSG00000198797.6	BRINP2	turquoise	0.677916878
ENSG00000162670.8	BRINP3	blue	0.660243632
ENSG00000113460.8	BRIX1	turquoise	0.912067761
ENSG00000254999.2	BRK1	turquoise	0.942884904
ENSG00000174744.9	BRMS1	turquoise	0.829833984
ENSG00000100916.9	BRMS1L	turquoise	0.933408747
ENSG00000162819.7	BROX	turquoise	0.90872691
ENSG00000156983.11	BRPF1	turquoise	0.903475488
ENSG00000096070.15	BRPF3	turquoise	0.853969487
ENSG00000160469.12	BRSK1	turquoise	0.9178617
ENSG00000174672.11	BRSK2	turquoise	0.898041986
ENSG00000185658.9	BRWD1	turquoise	0.965597806

ENSG00000255568.2	BRWD1-IT2	turquoise	0.624207903
ENSG00000165288.10	BRWD3	turquoise	0.871470061
ENSG00000168000.10	BSCL2	turquoise	0.931812149
ENSG00000160058.14	BSDC1	turquoise	0.97642639
ENSG00000172270.14	BSG	blue	0.704515227
ENSG00000164061.4	BSN	turquoise	0.917710461
ENSG00000119411.10	BSPRY	turquoise	0.820244884
ENSG00000109743.6	BST1	blue	0.637909947
ENSG00000130303.8	BST2	black	0.694031493
ENSG00000095564.9	BTAF1	turquoise	0.829492408
ENSG00000064726.5	BTBD1	turquoise	0.916216097
ENSG00000148925.6	BTBD10	turquoise	0.950701401
ENSG00000151136.10	BTBD11	turquoise	0.765269812
ENSG00000138152.7	BTBD16	brown	0.787340625
ENSG00000204347.3	BTBD17	grey	0.146258112
ENSG00000222009.4	BTBD19	blue	0.657793431
ENSG00000133243.4	BTBD2	turquoise	0.839843327
ENSG00000132640.10	BTBD3	brown	0.894785806
ENSG00000184887.9	BTBD6	turquoise	0.920637591
ENSG00000011114.10	BTBD7	turquoise	0.849015805
ENSG00000183826.12	BTBD9	turquoise	0.924548081
ENSG00000169814.8	BTD	blue	0.857301552
ENSG00000145741.11	BTF3	turquoise	0.926664235
ENSG00000134717.13	BTF3L4	turquoise	0.970337596
ENSG00000133639.3	BTG1	turquoise	0.778091216
ENSG00000159388.5	BTG2	black	0.58150029
ENSG00000154640.10	BTG3	turquoise	0.880059479
ENSG00000010671.11	BTK	magenta	0.945760658
ENSG00000112763.11	BTN2A1	turquoise	0.946053454
ENSG00000124508.12	BTN2A2	turquoise	0.75573465
ENSG00000026950.12	BTN3A1	blue	0.801430558
ENSG00000186470.9	BTN3A2	blue	0.558946851
ENSG00000111801.11	BTN3A3	turquoise	0.738058323
ENSG00000165810.12	BTNL9	black	0.503403326
ENSG00000166167.13	BTRC	turquoise	0.973586693
ENSG00000154473.13	BUB3	turquoise	0.906828366
ENSG00000137656.7	BUD13	turquoise	0.886323678
ENSG00000106245.5	BUD31	turquoise	0.908426464
ENSG00000112276.9	BVES	blue	0.762715656
ENSG00000215447.3	BX322557.10	turquoise	0.828185248
ENSG00000235510.1	BX842568.1	turquoise	0.751466912
ENSG00000197180.1	BX936347.1	red	0.805776275
ENSG00000112578.5	BYSL	turquoise	0.912541791
ENSG00000005379.11	BZRAP1	turquoise	0.90911238
ENSG00000265148.1	BZRAP1-AS1	turquoise	0.797093128
ENSG00000082153.13	BZW1	turquoise	0.845093503
ENSG00000136261.10	BZW2	turquoise	0.93139352
ENSG00000165507.8	C10orf10	black	0.803570413

ENSG00000183346.6	C10orf107	pink	0.822816495
ENSG00000148655.10	C10orf11	turquoise	0.673843305
ENSG00000176236.4	C10orf111	pink	0.861882164
ENSG00000165813.12	C10orf118	turquoise	0.863372363
ENSG00000204161.9	C10orf128	brown	0.800606976
ENSG00000107938.13	C10orf137	turquoise	0.945332898
ENSG00000107815.3	C10orf2	turquoise	0.8486069
ENSG00000165511.4	C10orf25	turquoise	0.763528186
ENSG00000166275.11	C10orf32	turquoise	0.906210001
ENSG00000171224.7	C10orf35	turquoise	0.922960221
ENSG00000107738.15	C10orf54	green	0.556847941
ENSG00000120029.8	C10orf76	turquoise	0.967930418
ENSG00000177234.5	C10orf85	black	0.532397215
ENSG00000119965.8	C10orf88	turquoise	0.955966046
ENSG00000154493.13	C10orf90	brown	0.911174374
ENSG00000137720.3	C11orf1	turquoise	0.91503149
ENSG00000171067.6	C11orf24	midnightblue	0.895342235
ENSG00000158636.12	C11orf30	turquoise	0.858050452
ENSG00000211450.5	C11orf31	turquoise	0.912239556
ENSG00000185522.4	C11orf35	turquoise	0.742180748
ENSG00000174370.5	C11orf45	turquoise	0.796898029
ENSG00000162194.8	C11orf48	red	0.957914576
ENSG00000149179.9	C11orf49	turquoise	0.932567298
ENSG00000182919.10	C11orf54	blue	0.915254487
ENSG00000150776.13	C11orf57	turquoise	0.955200524
ENSG00000110696.5	C11orf58	turquoise	0.957273837
ENSG00000109944.6	C11orf63	pink	0.934519997
ENSG00000175573.6	C11orf68	turquoise	0.945579278
ENSG00000149196.11	C11orf73	turquoise	0.913141499
ENSG00000166352.11	C11orf74	turquoise	0.866367885
ENSG00000173715.11	C11orf80	turquoise	0.865623915
ENSG00000204922.4	C11orf83	red	0.933470451
ENSG00000168005.4	C11orf84	cyan	0.90117585
ENSG00000185742.6	C11orf87	turquoise	0.88795374
ENSG00000188070.8	C11orf95	turquoise	0.878104751
ENSG00000187479.4	C11orf96	black	0.635989853
ENSG00000139637.9	C12orf10	turquoise	0.927519152
ENSG00000151135.5	C12orf23	turquoise	0.941614213
ENSG00000133641.13	C12orf29	turquoise	0.906629618
ENSG00000134548.5	C12orf39	green	0.575875287
ENSG00000047621.7	C12orf4	turquoise	0.930565789
ENSG00000157895.7	C12orf43	turquoise	0.954660885
ENSG00000123395.10	C12orf44	turquoise	0.897419528
ENSG00000151131.5	C12orf45	turquoise	0.860832513
ENSG00000078237.4	C12orf5	turquoise	0.765099483
ENSG00000139405.11	C12orf52	turquoise	0.944283328
ENSG00000111678.6	C12orf57	red	0.936252417
ENSG00000182993.3	C12orf60	blue	0.768373385

ENSG00000130921.3	C12orf65	turquoise	0.945773677
ENSG00000174206.8	C12orf66	turquoise	0.956695006
ENSG00000177875.3	C12orf68	turquoise	0.874822104
ENSG00000204954.5	C12orf73	turquoise	0.951607119
ENSG00000235162.4	C12orf75	turquoise	0.720440874
ENSG00000174456.9	C12orf76	brown	0.848911878
ENSG00000133935.6	C14orf1	turquoise	0.937321466
ENSG00000179933.4	C14orf119	turquoise	0.895050743
ENSG00000227051.4	C14orf132	turquoise	0.85916045
ENSG00000170270.4	C14orf142	pink	0.876036406
ENSG00000133943.16	C14orf159	blue	0.860332564
ENSG00000087302.4	C14orf166	turquoise	0.91229701
ENSG00000156411.5	C14orf2	turquoise	0.916527486
ENSG00000186960.6	C14orf23	turquoise	0.861770406
ENSG00000179476.3	C14orf28	turquoise	0.836573746
ENSG00000139971.11	C14orf37	green	0.815007319
ENSG00000140104.9	C14orf79	pink	0.913719816
ENSG00000185347.13	C14orf80	turquoise	0.832296317
ENSG00000100802.10	C14orf93	blue	0.79307245
ENSG00000169758.8	C15orf27	turquoise	0.81026081
ENSG00000259642.1	C15orf37	turquoise	0.672792667
ENSG00000167173.14	C15orf39	turquoise	0.650737016
ENSG00000169609.9	C15orf40	turquoise	0.911794547
ENSG00000186073.7	C15orf41	turquoise	0.925478509
ENSG00000188549.8	C15orf52	brown	0.835599435
ENSG00000128891.11	C15orf57	turquoise	0.95735246
ENSG00000205363.4	C15orf59	turquoise	0.810411276
ENSG00000189227.4	C15orf61	turquoise	0.913386345
ENSG00000188277.8	C15orf62	yellow	0.891433097
ENSG00000261652.2	C15orf65	turquoise	0.781815739
ENSG00000130731.11	C16orf13	turquoise	0.852330565
ENSG00000166780.6	C16orf45	turquoise	0.947217375
ENSG00000166455.9	C16orf46	blue	0.872037839
ENSG00000185716.7	C16orf52	turquoise	0.954060394
ENSG00000140688.12	C16orf58	turquoise	0.969234416
ENSG00000162062.10	C16orf59	turquoise	0.872187148
ENSG00000103544.10	C16orf62	turquoise	0.94415612
ENSG00000125149.7	C16orf70	turquoise	0.963676898
ENSG00000182831.7	C16orf72	turquoise	0.889249881
ENSG00000154102.6	C16orf74	turquoise	0.505894967
ENSG00000070761.3	C16orf80	turquoise	0.965023038
ENSG00000159761.10	C16orf86	turquoise	0.880376018
ENSG00000155330.5	C16orf87	turquoise	0.909703217
ENSG00000153446.11	C16orf89	green	0.715794182
ENSG00000174109.3	C16orf91	turquoise	0.929945943
ENSG00000196118.7	C16orf93	pink	0.910471117
ENSG00000260456.2	C16orf95	red	0.817604667
ENSG00000212734.4	C17orf100	turquoise	0.770595545

ENSG00000154035.6	C17orf103	turquoise	0.809864161
ENSG00000205710.3	C17orf107	turquoise	0.853040916
ENSG00000258315.1	C17orf49	red	0.913695753
ENSG00000154768.4	C17orf50	turquoise	0.679129342
ENSG00000212719.6	C17orf51	turquoise	0.942494423
ENSG00000186665.8	C17orf58	turquoise	0.88248854
ENSG00000196544.6	C17orf59	red	0.902650534
ENSG00000178927.12	C17orf62	blue	0.83247579
ENSG00000214226.4	C17orf67	cyan	0.790813141
ENSG00000185504.12	C17orf70	turquoise	0.844854322
ENSG00000224383.3	C17orf72	pink	0.884526588
ENSG00000108666.5	C17orf75	turquoise	0.955564851
ENSG00000175061.13	C17orf76-AS1	red	0.926865169
ENSG00000141219.11	C17orf80	turquoise	0.916870877
ENSG00000074356.12	C17orf85	brown	0.862200283
ENSG00000224877.3	C17orf89	red	0.88493552
ENSG00000179294.5	C17orf96	turquoise	0.763497739
ENSG00000187624.7	C17orf97	turquoise	0.888615866
ENSG00000141428.12	C18orf21	turquoise	0.921859281
ENSG00000177576.6	C18orf32	blue	0.799865795
ENSG00000166845.9	C18orf54	brown	0.827816108
ENSG00000176912.3	C18orf56	brown	0.771118511
ENSG00000141452.5	C18orf8	turquoise	0.923220915
ENSG00000074842.3	C19orf10	turquoise	0.853503715
ENSG00000131943.13	C19orf12	turquoise	0.973969483
ENSG00000177025.3	C19orf18	yellow	0.541368676
ENSG00000228300.9	C19orf24	red	0.909870795
ENSG00000119559.11	C19orf25	red	0.890702108
ENSG00000099625.8	C19orf26	turquoise	0.835762041
ENSG00000167644.7	C19orf33	grey	0.175663188
ENSG00000214212.4	C19orf38	magenta	0.87988751
ENSG00000123144.6	C19orf43	red	0.948338507
ENSG00000105072.4	C19orf44	pink	0.874106802
ENSG00000160392.9	C19orf47	brown	0.82645906
ENSG00000167747.9	C19orf48	red	0.782433118
ENSG00000142444.6	C19orf52	turquoise	0.939836893
ENSG00000104979.4	C19orf53	red	0.963424552
ENSG00000188493.10	C19orf54	blue	0.814449604
ENSG00000167595.10	C19orf55	pink	0.905683475
ENSG00000132016.7	C19orf57	yellow	0.794529996
ENSG00000006015.13	C19orf60	turquoise	0.802890587
ENSG00000130813.13	C19orf66	turquoise	0.919114725
ENSG00000185453.8	C19orf68	turquoise	0.778536726
ENSG00000174917.4	C19orf70	red	0.964642941
ENSG00000183397.5	C19orf71	yellow	0.906702115
ENSG00000221916.2	C19orf73	turquoise	0.772090354
ENSG00000095932.5	C19orf77	turquoise	0.548869444
ENSG00000235034.2	C19orf81	red	0.674985936

ENSG00000267106.1	C19orf82	turquoise	0.841640386
ENSG00000197223.7	C1D	turquoise	0.926251318
ENSG00000106392.6	C1GALT1	turquoise	0.773736493
ENSG00000171155.7	C1GALT1C1	blue	0.898787936
ENSG00000116922.10	C1orf109	turquoise	0.956179015
ENSG00000162817.6	C1orf115	turquoise	0.89828606
ENSG00000197982.12	C1orf122	brown	0.582310539
ENSG00000162384.9	C1orf123	turquoise	0.929496192
ENSG00000143633.8	C1orf131	turquoise	0.911863798
ENSG00000203709.5	C1orf132	grey	0.759554186
ENSG00000204377.3	C1orf134	turquoise	0.698637795
ENSG00000162913.9	C1orf145	turquoise	0.791267401
ENSG00000131591.13	C1orf159	turquoise	0.822487009
ENSG00000143110.7	C1orf162	magenta	0.866197556
ENSG00000178965.9	C1orf173	turquoise	0.914266273
ENSG00000198912.6	C1orf174	turquoise	0.931292445
ENSG00000162398.7	C1orf177	turquoise	0.727221276
ENSG00000188931.3	C1orf192	pink	0.854725975
ENSG00000179902.8	C1orf194	pink	0.861167923
ENSG00000119280.12	C1orf198	blue	0.811793085
ENSG00000188004.5	C1orf204	pink	0.89712208
ENSG00000116667.8	C1orf21	blue	0.782440129
ENSG00000249087.3	C1orf213	yellow	0.884524546
ENSG00000142686.7	C1orf216	turquoise	0.947237676
ENSG00000142609.13	C1orf222	pink	0.905482221
ENSG00000198520.6	C1orf228	yellow	0.792709759
ENSG00000228594.1	C1orf233	turquoise	0.72969316
ENSG00000157181.10	C1orf27	blue	0.911752679
ENSG00000143793.8	C1orf35	turquoise	0.898965118
ENSG00000143612.14	C1orf43	turquoise	0.944264055
ENSG00000164008.9	C1orf50	turquoise	0.894011122
ENSG00000159208.11	C1orf51	turquoise	0.810561433
ENSG00000162642.9	C1orf52	turquoise	0.962845197
ENSG00000203724.6	C1orf53	turquoise	0.654070173
ENSG00000118292.4	C1orf54	green	0.670953868
ENSG00000143443.9	C1orf56	blue	0.879090224
ENSG00000125462.12	C1orf61	green	0.640718405
ENSG00000117616.13	C1orf63	yellow	0.919350401
ENSG00000183888.4	C1orf64	black	0.719633263
ENSG00000162757.3	C1orf74	turquoise	0.889968455
ENSG00000198715.7	C1orf85	turquoise	0.851332053
ENSG00000162585.12	C1orf86	red	0.782062012
ENSG00000203685.5	C1orf95	turquoise	0.823864567
ENSG00000173372.12	C1QA	magenta	0.916374184
ENSG00000173369.11	C1QB	magenta	0.911017567
ENSG00000108561.4	C1QBP	turquoise	0.926143766
ENSG00000159189.7	C1QC	magenta	0.921102879
ENSG00000131094.3	C1QL1	grey	0.375852748

ENSG00000173918.10	C1QTNF1	brown	0.525855136
ENSG00000082196.16	C1QTNF3	green	0.802652594
ENSG00000172247.3	C1QTNF4	turquoise	0.743097403
ENSG00000223953.3	C1QTNF5	brown	0.494284533
ENSG00000133466.9	C1QTNF6	blue	0.687165469
ENSG00000159403.11	C1R	black	0.843522223
ENSG00000139178.6	C1RL	blue	0.760506342
ENSG00000182326.10	C1S	black	0.836235645
ENSG00000166278.10	C2	turquoise	0.721172729
ENSG00000197183.8	C20orf112	brown	0.809570029
ENSG00000088854.11	C20orf194	turquoise	0.917728125
ENSG00000171984.10	C20orf196	turquoise	0.863072728
ENSG00000171695.6	C20orf201	turquoise	0.583873404
ENSG00000198547.4	C20orf203	turquoise	0.785037806
ENSG00000101084.12	C20orf24	turquoise	0.909994986
ENSG00000089101.13	C20orf26	pink	0.915581206
ENSG00000101220.13	C20orf27	turquoise	0.815615996
ENSG00000196476.7	C20orf96	pink	0.94038532
ENSG00000256073.2	C21orf119	turquoise	0.834266826
ENSG00000160226.11	C21orf2	turquoise	0.894983605
ENSG00000160221.12	C21orf33	turquoise	0.917548758
ENSG00000232560.2	C21orf37	brown	0.508206867
ENSG00000160298.13	C21orf58	pink	0.917365266
ENSG00000159079.14	C21orf59	pink	0.892512578
ENSG00000183250.7	C21orf67	turquoise	0.851027553
ENSG00000184809.8	C21orf88	turquoise	0.885506356
ENSG00000154642.6	C21orf91	brown	0.899999026
ENSG00000128346.6	C22orf23	pink	0.85209473
ENSG00000215012.4	C22orf29	turquoise	0.955233166
ENSG00000100249.4	C22orf31	grey	0.37245219
ENSG00000242259.4	C22orf39	turquoise	0.9414856
ENSG00000184208.10	C22orf46	brown	0.714452135
ENSG00000157617.12	C2CD2	turquoise	0.867608856
ENSG00000172375.8	C2CD2L	turquoise	0.936613595
ENSG00000168014.12	C2CD3	turquoise	0.928054825
ENSG00000205502.3	C2CD4B	grey	0.100207119
ENSG00000183186.6	C2CD4C	greenyellow	0.692624259
ENSG00000111731.8	C2CD5	turquoise	0.960821102
ENSG00000273045.1	C2ORF15	turquoise	0.870385806
ENSG00000197927.8	C2orf27A	turquoise	0.806140764
ENSG00000119147.5	C2orf40	pink	0.80682828
ENSG00000115998.3	C2orf42	turquoise	0.917291271
ENSG00000118961.10	C2orf43	turquoise	0.93965323
ENSG00000163026.7	C2orf44	turquoise	0.885942382
ENSG00000162972.6	C2orf47	turquoise	0.917306901
ENSG00000135974.5	C2orf49	blue	0.928913433
ENSG00000150873.7	C2orf50	pink	0.873870285
ENSG00000168887.6	C2orf68	blue	0.890208049

ENSG00000178074.5	C2orf69	turquoise	0.939131454
ENSG00000204128.5	C2orf72	green	0.68837584
ENSG00000177994.11	C2orf73	pink	0.859239196
ENSG00000186132.10	C2orf76	blue	0.855651852
ENSG00000188674.6	C2orf80	turquoise	0.898708635
ENSG00000159239.7	C2orf81	pink	0.932382794
ENSG00000182600.5	C2orf82	grey	0.25195072
ENSG00000187699.6	C2orf88	blue	0.819077114
ENSG00000125730.12	C3	magenta	0.924783523
ENSG00000171860.4	C3AR1	magenta	0.953791818
ENSG00000114405.6	C3orf14	turquoise	0.945273247
ENSG00000163608.10	C3orf17	turquoise	0.963354712
ENSG00000088543.10	C3orf18	turquoise	0.928112098
ENSG00000174928.11	C3orf33	turquoise	0.92119009
ENSG00000179021.5	C3orf38	turquoise	0.892237087
ENSG00000181744.4	C3orf58	blue	0.802957792
ENSG00000188315.3	C3orf62	turquoise	0.879330424
ENSG00000187068.2	C3orf70	blue	0.791234776
ENSG00000225526.4	C3orf83	turquoise	0.789503381
ENSG00000244731.3	C4A	black	0.679974725
ENSG00000224389.4	C4B	black	0.673630589
ENSG00000154274.10	C4orf19	blue	0.498764174
ENSG00000138658.11	C4orf21	brown	0.786650303
ENSG00000056050.6	C4orf27	turquoise	0.921538359
ENSG00000164074.10	C4orf29	turquoise	0.937439238
ENSG00000164096.10	C4orf3	turquoise	0.773281235
ENSG00000151470.8	C4orf33	turquoise	0.899250561
ENSG00000163633.6	C4orf36	blue	0.866451463
ENSG00000205208.4	C4orf46	turquoise	0.707074419
ENSG00000205129.4	C4orf47	pink	0.897665195
ENSG00000243449.2	C4orf48	red	0.648740047
ENSG00000181215.8	C4orf50	turquoise	0.811547044
ENSG00000106804.6	C5	turquoise	0.806572975
ENSG00000197405.3	C5AR1	magenta	0.740734718
ENSG00000113583.6	C5orf15	turquoise	0.880477198
ENSG00000082213.13	C5orf22	turquoise	0.974253293
ENSG00000181904.8	C5orf24	turquoise	0.949871507
ENSG00000236882.3	C5orf27	grey	0.33881409
ENSG00000151881.10	C5orf28	turquoise	0.798924177
ENSG00000181751.5	C5orf30	turquoise	0.90928255
ENSG00000172244.4	C5orf34	turquoise	0.876788379
ENSG00000197603.9	C5orf42	turquoise	0.956646801
ENSG00000161010.10	C5orf45	turquoise	0.903046612
ENSG00000215217.2	C5orf49	pink	0.810546171
ENSG00000205765.4	C5orf51	turquoise	0.903196459
ENSG00000221886.3	C5orf54	turquoise	0.867625594
ENSG00000221990.2	C5orf55	turquoise	0.833433933
ENSG00000197536.6	C5orf56	black	0.680935349

ENSG00000178722.8	C5orf64	brown	0.708037687
ENSG00000186577.7	C6orf1	black	0.597013877
ENSG00000196821.5	C6orf106	turquoise	0.926092087
ENSG00000185127.5	C6orf120	turquoise	0.943477644
ENSG00000204564.7	C6orf136	turquoise	0.948560921
ENSG00000197261.7	C6orf141	turquoise	0.824718135
ENSG00000130349.5	C6orf203	turquoise	0.893191082
ENSG00000146476.6	C6orf211	turquoise	0.938257698
ENSG00000221821.2	C6orf226	turquoise	0.826377821
ENSG00000255389.1	C6orf3	turquoise	0.622020496
ENSG00000204439.3	C6orf47	turquoise	0.863091798
ENSG00000204387.8	C6orf48	red	0.916675317
ENSG00000145965.5	C6ORF50	yellow	0.783478981
ENSG00000154079.5	C6orf57	turquoise	0.943883442
ENSG00000112308.8	C6orf62	turquoise	0.826745874
ENSG00000198663.12	C6orf89	turquoise	0.966688324
ENSG00000112936.14	C7	magenta	0.598429339
ENSG00000175600.11	C7orf10	green	0.808945803
ENSG00000244291.1	C7orf13	turquoise	0.893706872
ENSG00000136197.8	C7orf25	turquoise	0.90256307
ENSG00000146576.8	C7orf26	turquoise	0.920552375
ENSG00000153790.7	C7orf31	turquoise	0.830607486
ENSG00000180354.11	C7orf41	brown	0.503111391
ENSG00000146826.10	C7orf43	turquoise	0.9240492
ENSG00000122783.12	C7orf49	blue	0.881111392
ENSG00000146540.10	C7orf50	red	0.820258595
ENSG00000164898.8	C7orf55	red	0.922537999
ENSG00000146963.13	C7orf55-LUC7L2	turquoise	0.922543358
ENSG00000164603.7	C7orf60	turquoise	0.887734377
ENSG00000185955.4	C7orf61	black	0.532151554
ENSG00000105792.15	C7orf63	pink	0.95859182
ENSG00000243317.3	C7orf73	blue	0.864731944
ENSG00000182307.8	C8orf33	blue	0.879631772
ENSG00000165084.11	C8orf34	pink	0.879381769
ENSG00000156172.5	C8orf37	blue	0.845016864
ENSG00000176907.3	C8orf4	black	0.553855566
ENSG00000213865.3	C8orf44	turquoise	0.945300345
ENSG00000169085.7	C8orf46	turquoise	0.841130735
ENSG00000177459.6	C8orf47	turquoise	0.898969264
ENSG00000164743.4	C8orf48	turquoise	0.843027184
ENSG00000236939.2	C8orf56	red	0.622185276
ENSG00000241852.5	C8orf58	blue	0.733036226
ENSG00000176731.7	C8orf59	turquoise	0.873080588
ENSG00000189376.7	C8orf76	turquoise	0.870832568
ENSG00000213563.2	C8orf82	turquoise	0.787379764
ENSG00000198917.7	C9orf114	turquoise	0.900048821
ENSG00000160345.8	C9orf116	pink	0.921236672
ENSG00000204352.2	C9orf129	turquoise	0.813656113

ENSG00000148362.6	C9orf142	red	0.85228775
ENSG00000136932.9	C9orf156	turquoise	0.868800399
ENSG00000171159.4	C9orf16	turquoise	0.79082541
ENSG00000197191.3	C9orf169	pink	0.811570325
ENSG00000232434.1	C9orf172	turquoise	0.854091867
ENSG00000164972.8	C9orf24	pink	0.830237294
ENSG00000148120.10	C9orf3	brown	0.84244346
ENSG00000203993.3	C9orf37	red	0.897136393
ENSG00000135045.6	C9orf40	turquoise	0.916039898
ENSG00000156017.8	C9orf41	turquoise	0.870838637
ENSG00000165118.10	C9orf64	turquoise	0.83769339
ENSG00000238227.3	C9orf69	turquoise	0.909020743
ENSG00000147894.10	C9orf72	turquoise	0.863442983
ENSG00000136819.10	C9orf78	turquoise	0.959836412
ENSG00000155621.10	C9orf85	blue	0.863840564
ENSG00000165233.13	C9orf89	blue	0.819288108
ENSG00000165698.11	C9orf9	pink	0.933035222
ENSG00000157693.10	C9orf91	turquoise	0.959425103
ENSG00000198870.6	C9orf96	turquoise	0.849142889
ENSG00000154975.9	CA10	greenyellow	0.711982687
ENSG00000063180.4	CA11	turquoise	0.808872339
ENSG00000074410.9	CA12	turquoise	0.825888408
ENSG00000118298.6	CA14	brown	0.798587562
ENSG00000104267.5	CA2	brown	0.812873778
ENSG00000167434.5	CA4	grey	0.235359716
ENSG00000169239.8	CA5B	blue	0.76501437
ENSG00000186312.6	CA5BP1	blue	0.695268019
ENSG00000168748.9	CA7	turquoise	0.748664142
ENSG00000178538.5	CA8	green	0.759213639
ENSG00000120159.7	CAAP1	turquoise	0.885940353
ENSG00000135932.6	CAB39	turquoise	0.948581817
ENSG00000102547.14	CAB39L	blue	0.821402565
ENSG00000099991.12	CABIN1	turquoise	0.933681723
ENSG00000134508.8	CABLES1	green	0.743193723
ENSG00000149679.7	CABLES2	turquoise	0.910147172
ENSG00000157782.5	CABP1	turquoise	0.812080247
ENSG00000100314.3	CABP7	midnightblue	0.777006877
ENSG00000154040.16	CABYR	turquoise	0.835604248
ENSG00000160325.10	CACFD1	blue	0.779894027
ENSG00000158966.9	CACHD1	turquoise	0.882959418
ENSG00000141837.14	CACNA1A	turquoise	0.881594155
ENSG00000148408.8	CACNA1B	turquoise	0.922857174
ENSG00000151067.16	CACNA1C	turquoise	0.908928963
ENSG00000256271.1	CACNA1C-AS2	yellow	0.602005618
ENSG00000157388.9	CACNA1D	turquoise	0.902562398
ENSG00000198216.6	CACNA1E	turquoise	0.882752886
ENSG00000006283.13	CACNA1G	greenyellow	0.809355337
ENSG00000196557.6	CACNA1H	turquoise	0.846346912

ENSG00000100346.13	CACNA1I	turquoise	0.826969535
ENSG00000153956.11	CACNA2D1	turquoise	0.940497092
ENSG00000007402.7	CACNA2D2	turquoise	0.90043281
ENSG00000157445.10	CACNA2D3	turquoise	0.913900558
ENSG00000067191.11	CACNB1	turquoise	0.912458401
ENSG00000165995.14	CACNB2	turquoise	0.932899942
ENSG00000167535.3	CACNB3	turquoise	0.878726547
ENSG00000182389.14	CACNB4	turquoise	0.907891698
ENSG00000108878.3	CACNG1	turquoise	0.735089819
ENSG00000166862.6	CACNG2	turquoise	0.817456071
ENSG00000006116.3	CACNG3	turquoise	0.872708044
ENSG00000075461.5	CACNG4	turquoise	0.841359248
ENSG00000105605.3	CACNG7	turquoise	0.856094447
ENSG00000142408.2	CACNG8	turquoise	0.874120212
ENSG00000105298.9	CACTIN	turquoise	0.902029457
ENSG00000151893.10	CACUL1	turquoise	0.974502949
ENSG00000116161.13	CACYBP	cyan	0.922874185
ENSG00000177855.8	CACYBPP2	cyan	0.752398088
ENSG00000084774.9	CAD	turquoise	0.898276078
ENSG00000182985.12	CADM1	blue	0.782713646
ENSG00000175161.9	CADM2	turquoise	0.852519129
ENSG00000162706.8	CADM3	turquoise	0.910347669
ENSG00000105767.2	CADM4	brown	0.836906274
ENSG00000163618.13	CADPS	turquoise	0.923791407
ENSG00000081803.11	CADPS2	green	0.682182132
ENSG00000270419.1	CAHM	brown	0.771941039
ENSG00000104327.3	CALB1	turquoise	0.863073076
ENSG00000172137.14	CALB2	greenyellow	0.885000778
ENSG00000012822.11	CALCOCO1	turquoise	0.966554429
ENSG00000136436.10	CALCOCO2	blue	0.822357888
ENSG00000064989.8	CALCRL	blue	0.578904545
ENSG00000122786.15	CALD1	blue	0.657753773
ENSG00000138172.6	CALHM2	blue	0.716555063
ENSG00000198668.6	CALM1	turquoise	0.952355571
ENSG00000143933.12	CALM2	turquoise	0.945822381
ENSG00000229097.1	CALM2P2	turquoise	0.71388242
ENSG00000160014.12	CALM3	turquoise	0.935306304
ENSG00000129007.10	CALML4	turquoise	0.865316164
ENSG00000169885.5	CALML6	grey	0.734339046
ENSG00000183166.6	CALN1	turquoise	0.916628277
ENSG00000179218.9	CALR	cyan	0.921349824
ENSG00000128595.12	CALU	turquoise	0.847428445
ENSG00000130643.4	CALY	turquoise	0.856585031
ENSG00000134072.6	CAMK1	turquoise	0.905162344
ENSG00000183049.8	CAMK1D	turquoise	0.906822281
ENSG00000008118.5	CAMK1G	turquoise	0.861722497
ENSG00000070808.11	CAMK2A	turquoise	0.83045232
ENSG00000058404.15	CAMK2B	turquoise	0.839722098

ENSG00000145349.12	CAMK2D	greenyellow	0.85643405
ENSG00000148660.16	CAMK2G	blue	0.740194927
ENSG00000162545.5	CAMK2N1	turquoise	0.828493357
ENSG00000163888.3	CAMK2N2	greenyellow	0.848194321
ENSG00000152495.6	CAMK4	turquoise	0.894207406
ENSG00000004660.10	CAMKK1	turquoise	0.900995399
ENSG00000110931.14	CAMKK2	turquoise	0.844133622
ENSG00000143919.10	CAMKMT	turquoise	0.891562344
ENSG00000164076.12	CAMKV	turquoise	0.867616039
ENSG00000164615.3	CAMLG	turquoise	0.938714684
ENSG00000130559.14	CAMSAP1	turquoise	0.977494723
ENSG00000118200.10	CAMSAP2	turquoise	0.948071048
ENSG00000076826.5	CAMSAP3	turquoise	0.909903992
ENSG00000171735.14	CAMTA1	turquoise	0.935523062
ENSG00000108509.16	CAMTA2	turquoise	0.938819859
ENSG00000111530.8	CAND1	turquoise	0.971221889
ENSG00000144712.7	CAND2	turquoise	0.954381126
ENSG00000171302.12	CANT1	turquoise	0.914954979
ENSG00000127022.10	CANX	turquoise	0.866797511
ENSG00000131236.12	CAP1	turquoise	0.905953494
ENSG00000112186.7	CAP2	turquoise	0.92534008
ENSG00000042493.11	CAPG	magenta	0.682427156
ENSG00000014216.11	CAPN1	turquoise	0.92290309
ENSG00000142330.15	CAPN10	turquoise	0.911989786
ENSG00000260942.1	CAPN10-AS1	yellow	0.826992972
ENSG00000103326.6	CAPN15	turquoise	0.838196123
ENSG00000162909.13	CAPN2	green	0.904134515
ENSG00000092529.18	CAPN3	brown	0.907370728
ENSG00000149260.10	CAPN5	blue	0.842339162
ENSG00000131375.5	CAPN7	turquoise	0.919116158
ENSG00000126247.6	CAPNS1	turquoise	0.938658679
ENSG00000135387.15	CAPRIN1	turquoise	0.947003475
ENSG00000110888.13	CAPRIN2	turquoise	0.951072223
ENSG00000180881.15	CAPS2	pink	0.867373403
ENSG00000116489.8	CAPZA1	turquoise	0.866676459
ENSG00000198898.8	CAPZA2	turquoise	0.893734383
ENSG00000077549.13	CAPZB	turquoise	0.950151247
ENSG00000100065.10	CARD10	black	0.620380893
ENSG00000198286.5	CARD11	magenta	0.804894743
ENSG00000204397.3	CARD16	magenta	0.670818431
ENSG00000132357.9	CARD6	black	0.793505932
ENSG00000105483.12	CARD8	blue	0.762630932
ENSG00000187796.9	CARD9	yellow	0.780375597
ENSG00000138380.13	CARF	turquoise	0.827878382
ENSG00000213995.7	CARKD	turquoise	0.955047784
ENSG00000142453.7	CARM1	turquoise	0.946836492
ENSG00000172508.6	CARNS1	brown	0.900436948
ENSG00000110619.12	CARS	turquoise	0.863416816

ENSG00000134905.12	CARS2	blue	0.822330633
ENSG00000118307.14	CASC1	pink	0.894071985
ENSG00000204682.5	CASC10	green	0.704734562
ENSG00000272168.1	CASC15	turquoise	0.813778499
ENSG00000177640.11	CASC2	pink	0.907412704
ENSG00000108349.10	CASC3	brown	0.793595202
ENSG00000166734.14	CASC4	blue	0.90381628
ENSG00000259758.1	CASC7	turquoise	0.907389043
ENSG00000127995.12	CASD1	turquoise	0.95458873
ENSG00000147044.16	CASK	turquoise	0.951361171
ENSG00000167971.14	CASKIN1	turquoise	0.860083328
ENSG00000177303.5	CASKIN2	blue	0.726782148
ENSG00000137752.18	CASP1	magenta	0.823177264
ENSG00000003400.10	CASP10	black	0.709867523
ENSG00000106144.15	CASP2	turquoise	0.901320925
ENSG00000164305.13	CASP3	blue	0.871487448
ENSG00000196954.8	CASP4	black	0.879804985
ENSG00000138794.5	CASP6	blue	0.81519763
ENSG00000165806.15	CASP7	blue	0.588458481
ENSG00000118412.8	CASP8AP2	turquoise	0.836741954
ENSG00000132906.13	CASP9	turquoise	0.909262122
ENSG00000143318.8	CASQ1	turquoise	0.7438051
ENSG00000118729.10	CASQ2	grey	0.311692297
ENSG00000153113.19	CAST	blue	0.910712246
ENSG00000121691.4	CAT	green	0.858124155
ENSG00000166762.12	CATSPER2	yellow	0.889837688
ENSG00000152705.6	CATSPER3	turquoise	0.597437595
ENSG00000105974.7	CAV1	brown	0.621230011
ENSG00000105971.10	CAV2	brown	0.699396638
ENSG00000078699.17	CBFA2T2	turquoise	0.950673297
ENSG00000129993.10	CBFA2T3	turquoise	0.887060001
ENSG00000067955.9	CBFB	blue	0.74788027
ENSG00000110395.4	CBL	turquoise	0.918060583
ENSG00000114423.14	CBLB	blue	0.888226762
ENSG00000105879.7	CBLL1	turquoise	0.948729341
ENSG00000102924.7	CBLN1	greenyellow	0.750941433
ENSG00000139899.6	CBLN3	grey	0.397395007
ENSG00000159228.8	CBR1	brown	0.830916652
ENSG00000159231.5	CBR3	turquoise	0.774064829
ENSG00000236830.2	CBR3-AS1	turquoise	0.850580893
ENSG00000145439.7	CBR4	turquoise	0.922920418
ENSG00000160200.13	CBS	blue	0.733877439
ENSG00000172785.14	CBWD1	turquoise	0.813218561
ENSG00000136682.10	CBWD2	turquoise	0.935513147
ENSG00000147996.12	CBWD5	turquoise	0.852846056
ENSG00000204790.8	CBWD6	turquoise	0.825469985
ENSG00000108468.10	CBX1	turquoise	0.951718628
ENSG00000122565.14	CBX3	turquoise	0.945819071

ENSG00000266405.2	CBX3P2	turquoise	0.715585276
ENSG00000141582.10	CBX4	turquoise	0.929874303
ENSG00000094916.9	CBX5	turquoise	0.960613276
ENSG00000183741.7	CBX6	turquoise	0.931595165
ENSG00000100307.8	CBX7	turquoise	0.94833342
ENSG00000141570.6	CBX8	turquoise	0.779973905
ENSG00000100211.6	CBY1	pink	0.845622192
ENSG00000204659.4	CBY3	turquoise	0.539410612
ENSG00000132024.13	CC2D1A	yellow	0.939751542
ENSG00000154222.10	CC2D1B	brown	0.885547366
ENSG00000048342.11	CC2D2A	blue	0.788156177
ENSG00000060339.9	CCAR1	turquoise	0.895303179
ENSG00000158941.12	CCAR2	turquoise	0.971767824
ENSG00000171097.9	CCBL1	turquoise	0.90845995
ENSG00000137944.12	CCBL2	turquoise	0.81667728
ENSG00000176476.4	CCDC101	blue	0.770763658
ENSG00000150636.11	CCDC102B	blue	0.761499954
ENSG00000163001.7	CCDC104	turquoise	0.958158291
ENSG00000173581.3	CCDC106	turquoise	0.877692224
ENSG00000159884.7	CCDC107	red	0.872108362
ENSG00000005059.11	CCDC109B	blue	0.705520683
ENSG00000168491.5	CCDC110	turquoise	0.839560867
ENSG00000164221.8	CCDC112	turquoise	0.935790493
ENSG00000103021.5	CCDC113	pink	0.944060582
ENSG00000136710.5	CCDC115	turquoise	0.943707609
ENSG00000159873.5	CCDC117	cyan	0.931348128
ENSG00000160799.7	CCDC12	red	0.940986624
ENSG00000147144.8	CCDC120	turquoise	0.788884941
ENSG00000176714.9	CCDC121	green	0.768842947
ENSG00000007080.6	CCDC124	red	0.94778232
ENSG00000183323.8	CCDC125	blue	0.838816501
ENSG00000169193.7	CCDC126	turquoise	0.908812648
ENSG00000164366.3	CCDC127	turquoise	0.929859527
ENSG00000244607.1	CCDC13	pink	0.952243844
ENSG00000173811.6	CCDC13-AS1	pink	0.819780535
ENSG00000104957.9	CCDC130	yellow	0.938518893
ENSG00000004766.11	CCDC132	turquoise	0.970991205
ENSG00000100147.9	CCDC134	blue	0.775914972
ENSG00000128596.12	CCDC136	turquoise	0.892616034
ENSG00000185298.8	CCDC137	turquoise	0.882780894
ENSG00000163006.7	CCDC138	pink	0.837026298
ENSG00000175455.10	CCDC14	yellow	0.920322682
ENSG00000135637.9	CCDC142	turquoise	0.865333098
ENSG00000135205.10	CCDC146	pink	0.947434752
ENSG00000120051.10	CCDC147	pink	0.875912065
ENSG00000231233.1	CCDC147-AS1	turquoise	0.826592823
ENSG00000153237.13	CCDC148	turquoise	0.813519053
ENSG00000181982.13	CCDC149	turquoise	0.921822534

ENSG00000198003.7	CCDC151	pink	0.82284021
ENSG00000198865.5	CCDC152	blue	0.826365578
ENSG00000197599.8	CCDC154	yellow	0.78638594
ENSG00000187860.6	CCDC157	pink	0.947838608
ENSG00000183401.7	CCDC159	brown	0.887779467
ENSG00000203952.5	CCDC160	blue	0.623189644
ENSG00000236624.4	CCDC163P	blue	0.637857426
ENSG00000198937.8	CCDC167	turquoise	0.549476465
ENSG00000154781.11	CCDC174	turquoise	0.854955909
ENSG00000151838.7	CCDC175	pink	0.887857485
ENSG00000119636.11	CCDC176	pink	0.944408524
ENSG00000255994.1	CCDC177	turquoise	0.708723908
ENSG00000122483.13	CCDC18	turquoise	0.774300171
ENSG00000197816.9	CCDC180	pink	0.936397473
ENSG00000117477.8	CCDC181	turquoise	0.891402909
ENSG00000101997.8	CCDC22	turquoise	0.942694034
ENSG00000177868.7	CCDC23	turquoise	0.861401037
ENSG00000159214.8	CCDC24	pink	0.835683322
ENSG00000147419.12	CCDC25	turquoise	0.911756748
ENSG00000024862.12	CCDC28A	turquoise	0.942370381
ENSG00000160050.10	CCDC28B	turquoise	0.857738141
ENSG00000151468.9	CCDC3	midnightblue	0.847456935
ENSG00000186409.10	CCDC30	pink	0.967913968
ENSG00000109881.12	CCDC34	turquoise	0.858818575
ENSG00000145075.7	CCDC39	pink	0.905970235
ENSG00000141519.10	CCDC40	pink	0.878757798
ENSG00000173588.10	CCDC41	pink	0.93155595
ENSG00000180329.9	CCDC43	turquoise	0.932711129
ENSG00000108588.9	CCDC47	turquoise	0.953028848
ENSG00000152492.9	CCDC50	blue	0.836544127
ENSG00000164051.9	CCDC51	turquoise	0.911647073
ENSG00000120860.6	CCDC53	turquoise	0.921424996
ENSG00000176155.14	CCDC57	yellow	0.917708794
ENSG00000160124.5	CCDC58	turquoise	0.870918422
ENSG00000133773.7	CCDC59	turquoise	0.900839517
ENSG00000108091.10	CCDC6	turquoise	0.934625938
ENSG00000104983.4	CCDC61	blue	0.723200468
ENSG00000135127.7	CCDC64	turquoise	0.8782887
ENSG00000139537.6	CCDC65	pink	0.945455832
ENSG00000180376.12	CCDC66	yellow	0.917314785
ENSG00000166510.9	CCDC68	grey	0.285661918
ENSG00000177352.9	CCDC71	turquoise	0.805065891
ENSG00000253276.1	CCDC71L	turquoise	0.697739155
ENSG00000163040.10	CCDC74A	pink	0.902965397
ENSG00000152076.14	CCDC74B	pink	0.903766757
ENSG00000120647.5	CCDC77	turquoise	0.89629002
ENSG00000162004.12	CCDC78	midnightblue	0.704886751
ENSG00000091986.11	CCDC80	green	0.553837118

ENSG00000149231.7	CCDC82	turquoise	0.835319705
ENSG00000186166.4	CCDC84	yellow	0.839015427
ENSG00000055813.5	CCDC85A	turquoise	0.839551008
ENSG00000175602.2	CCDC85B	turquoise	0.616971767
ENSG00000205476.4	CCDC85C	turquoise	0.847795725
ENSG00000110104.7	CCDC86	turquoise	0.910888207
ENSG00000115355.11	CCDC88A	turquoise	0.80134856
ENSG00000168071.17	CCDC88B	yellow	0.849363853
ENSG00000015133.14	CCDC88C	turquoise	0.8712192
ENSG00000105321.8	CCDC9	blue	0.802636407
ENSG00000137500.5	CCDC90B	turquoise	0.892479908
ENSG00000123106.6	CCDC91	turquoise	0.924061239
ENSG00000119242.4	CCDC92	turquoise	0.938351652
ENSG00000125633.6	CCDC93	turquoise	0.905449981
ENSG00000105248.11	CCDC94	red	0.865860405
ENSG00000173013.4	CCDC96	turquoise	0.8458425
ENSG00000142039.3	CCDC97	blue	0.910416827
ENSG00000204536.9	CCHCR1	turquoise	0.886987185
ENSG00000110148.5	CCKBR	turquoise	0.816265557
ENSG00000172724.7	CCL19	grey	0.152558793
ENSG00000108691.5	CCL2	black	0.696948617
ENSG00000213927.3	CCL27	turquoise	0.665228687
ENSG00000006075.11	CCL3	grey	0.248873186
ENSG00000129277.9	CCL4	grey	0.208436257
ENSG00000161570.4	CCL5	grey	0.168209472
ENSG00000136280.11	CCM2	turquoise	0.85079476
ENSG00000101331.11	CCM2L	grey	0.482480223
ENSG00000133101.5	CCNA1	turquoise	0.871998273
ENSG00000145386.5	CCNA2	blue	0.635423823
ENSG00000134057.10	CCNB1	turquoise	0.952212671
ENSG00000100814.13	CCNB1IP1	turquoise	0.902628381
ENSG00000112237.8	CCNC	turquoise	0.96213393
ENSG00000110092.3	CCND1	brown	0.611712838
ENSG00000118971.3	CCND2	turquoise	0.872860796
ENSG00000112576.8	CCND3	turquoise	0.788205157
ENSG00000166946.9	CCNDBP1	turquoise	0.972429316
ENSG00000105173.9	CCNE1	turquoise	0.930050797
ENSG00000175305.12	CCNE2	brown	0.861720982
ENSG00000162063.8	CCNF	turquoise	0.88662819
ENSG00000113328.14	CCNG1	turquoise	0.766315442
ENSG00000138764.9	CCNG2	turquoise	0.850674687
ENSG00000134480.9	CCNH	turquoise	0.90517435
ENSG00000118816.5	CCNI	turquoise	0.823700918
ENSG00000205089.3	CCNI2	turquoise	0.86440461
ENSG00000107443.11	CCNJ	turquoise	0.886074387
ENSG00000135083.10	CCNJL	green	0.662347569
ENSG00000090061.13	CCNK	turquoise	0.90541244
ENSG00000163660.7	CCNL1	yellow	0.849674738

ENSG00000221978.7	CCNL2	yellow	0.969555608
ENSG00000152669.8	CCNO	turquoise	0.802901261
ENSG00000129315.5	CCNT1	turquoise	0.842778602
ENSG00000082258.8	CCNT2	turquoise	0.9361906
ENSG00000108100.13	CCNY	turquoise	0.959262831
ENSG00000163249.5	CCNYL1	turquoise	0.853243494
ENSG00000103540.12	CCP110	brown	0.92027572
ENSG00000260916.1	CCPG1	turquoise	0.959832182
ENSG00000163823.3	CCR1	magenta	0.893452111
ENSG00000184451.5	CCR10	turquoise	0.754453327
ENSG00000151014.4	CCRN4L	cyan	0.782693921
ENSG00000173992.4	CCS	turquoise	0.879284429
ENSG00000154429.6	CCSAP	turquoise	0.959951995
ENSG00000107771.11	CCSER2	turquoise	0.94117055
ENSG00000166226.8	CCT2	turquoise	0.935086654
ENSG00000163468.10	CCT3	turquoise	0.905553419
ENSG00000115484.10	CCT4	cyan	0.955445177
ENSG00000150753.7	CCT5	turquoise	0.91771745
ENSG00000146731.6	CCT6A	turquoise	0.937951818
ENSG00000132141.9	CCT6B	brown	0.526178487
ENSG00000228409.1	CCT6P1	turquoise	0.864812974
ENSG00000234585.2	CCT6P3	turquoise	0.765659749
ENSG00000135624.11	CCT7	turquoise	0.957805187
ENSG00000156261.8	CCT8	turquoise	0.957116795
ENSG00000122674.8	CCZ1	turquoise	0.7794954
ENSG00000146574.11	CCZ1B	turquoise	0.769227168
ENSG00000170458.9	CD14	magenta	0.902011985
ENSG00000177697.13	CD151	blue	0.709076227
ENSG00000177575.8	CD163	magenta	0.794851028
ENSG00000135535.10	CD164	blue	0.943553909
ENSG00000091972.14	CD200	turquoise	0.966452709
ENSG00000012124.10	CD22	brown	0.858242672
ENSG00000198821.6	CD247	turquoise	0.757311408
ENSG00000174807.3	CD248	grey	0.342028062
ENSG00000185275.6	CD24P4	pink	0.790367642
ENSG00000139193.3	CD27	turquoise	0.823337032
ENSG00000215039.2	CD27-AS1	turquoise	0.938773689
ENSG00000120217.9	CD274	blue	0.682719182
ENSG00000103855.13	CD276	turquoise	0.872493946
ENSG00000198087.7	CD2AP	turquoise	0.796747602
ENSG00000169217.4	CD2BP2	turquoise	0.933278816
ENSG00000167851.9	CD300A	magenta	0.92124495
ENSG00000241399.2	CD302	blue	0.823828954
ENSG00000167775.6	CD320	blue	0.645787766
ENSG00000105383.10	CD33	magenta	0.922947516
ENSG00000174059.12	CD34	grey	0.46901546
ENSG00000104894.7	CD37	magenta	0.933291897
ENSG00000004468.8	CD38	green	0.693526295

ENSG00000117877.6	CD3EAP	turquoise	0.905842878
ENSG00000010610.5	CD4	magenta	0.860197004
ENSG00000101017.9	CD40	blue	0.701577932
ENSG00000026508.12	CD44	black	0.767073944
ENSG00000117335.14	CD46	blue	0.840267311
ENSG00000196776.10	CD47	turquoise	0.969345614
ENSG00000169442.4	CD52	midnightblue	0.772217605
ENSG00000143119.8	CD53	magenta	0.965096633
ENSG00000196352.9	CD55	brown	0.798855266
ENSG00000116815.11	CD58	blue	0.80993888
ENSG00000085063.10	CD59	turquoise	0.738630857
ENSG00000135404.7	CD63	black	0.793061916
ENSG00000129226.9	CD68	magenta	0.945177193
ENSG00000110848.4	CD69	magenta	0.556009247
ENSG00000019582.10	CD74	magenta	0.940716633
ENSG00000110651.7	CD81	blue	0.822093344
ENSG00000085117.7	CD82	brown	0.828259347
ENSG00000112149.5	CD83	turquoise	0.773095218
ENSG00000114013.11	CD86	magenta	0.935214644
ENSG00000153563.11	CD8A	turquoise	0.883077194
ENSG00000254126.2	CD8BP	grey	0.191358389
ENSG00000010278.7	CD9	brown	0.83973069
ENSG00000125810.9	CD93	black	0.846001114
ENSG00000123146.15	CD97	brown	0.825600565
ENSG00000002586.13	CD99	black	0.574520285
ENSG00000102181.15	CD99L2	turquoise	0.958764808
ENSG00000158825.5	CDA	black	0.636614052
ENSG00000102543.10	CDADC1	blue	0.896294139
ENSG00000140326.8	CDAN1	turquoise	0.920679618
ENSG00000151465.9	CDC123	turquoise	0.972298221
ENSG00000079335.13	CDC14A	green	0.745885753
ENSG00000081377.12	CDC14B	blue	0.912038153
ENSG00000130177.10	CDC16	turquoise	0.898634569
ENSG00000231007.4	CDC20P1	turquoise	0.814431188
ENSG00000094880.6	CDC23	turquoise	0.961300535
ENSG00000101224.13	CDC25B	yellow	0.925511983
ENSG00000176386.4	CDC26	turquoise	0.82533345
ENSG00000004897.7	CDC27	turquoise	0.967894939
ENSG00000099804.4	CDC34	red	0.902832938
ENSG00000105401.2	CDC37	turquoise	0.929657131
ENSG00000106993.7	CDC37L1	turquoise	0.955184537
ENSG00000168438.10	CDC40	turquoise	0.958162815
ENSG00000070831.11	CDC42	turquoise	0.92900408
ENSG00000143776.14	CDC42BPA	turquoise	0.904355908
ENSG00000198752.5	CDC42BPB	turquoise	0.915759947
ENSG00000128283.6	CDC42EP1	brown	0.83937283
ENSG00000149798.3	CDC42EP2	brown	0.844119585
ENSG00000163171.6	CDC42EP3	turquoise	0.876669015

ENSG00000179604.8	CDC42EP4	green	0.850318242
ENSG00000167617.2	CDC42EP5	black	0.676615836
ENSG00000197622.8	CDC42SE1	blue	0.914861944
ENSG00000158985.9	CDC42SE2	turquoise	0.927304325
ENSG00000096401.7	CDC5L	turquoise	0.925032948
ENSG00000094804.5	CDC6	turquoise	0.782721361
ENSG00000097046.8	CDC7	turquoise	0.895096489
ENSG00000134371.9	CDC73	blue	0.924934073
ENSG00000111665.7	CDCA3	turquoise	0.823187088
ENSG00000170779.10	CDCA4	blue	0.783662799
ENSG00000144354.9	CDCA7	pink	0.735297368
ENSG00000164649.15	CDCA7L	turquoise	0.76115821
ENSG00000134690.6	CDCA8	turquoise	0.780540286
ENSG00000039068.14	CDH1	brown	0.747655647
ENSG00000040731.6	CDH10	turquoise	0.715485119
ENSG00000140937.9	CDH11	turquoise	0.907076132
ENSG00000154162.9	CDH12	turquoise	0.759412802
ENSG00000140945.11	CDH13	greenyellow	0.85284089
ENSG00000145526.7	CDH18	turquoise	0.850453178
ENSG00000071991.4	CDH19	brown	0.834997612
ENSG00000170558.4	CDH2	blue	0.894362972
ENSG00000101542.5	CDH20	green	0.875115372
ENSG00000149654.5	CDH22	turquoise	0.796336316
ENSG00000107736.15	CDH23	blue	0.643580953
ENSG00000139880.15	CDH24	grey	0.810462513
ENSG00000062038.9	CDH3	turquoise	0.831189522
ENSG00000179242.11	CDH4	turquoise	0.819583298
ENSG00000179776.13	CDH5	black	0.817713669
ENSG00000113361.8	CDH6	midnightblue	0.860265529
ENSG00000150394.9	CDH8	turquoise	0.906477919
ENSG00000113100.5	CDH9	turquoise	0.851259588
ENSG00000148600.10	CDHR1	turquoise	0.890531763
ENSG00000128536.11	CDHR3	pink	0.895779595
ENSG00000089486.12	CDIP1	turquoise	0.932023209
ENSG00000103502.9	CDIPT	turquoise	0.947064112
ENSG00000214725.3	CDIPT-AS1	turquoise	0.66212216
ENSG00000185324.17	CDK10	yellow	0.893504424
ENSG00000008128.18	CDK11A	turquoise	0.820022384
ENSG00000248333.3	CDK11B	brown	0.776461556
ENSG00000167258.9	CDK12	turquoise	0.906895075
ENSG00000065883.10	CDK13	turquoise	0.945945247
ENSG00000058091.12	CDK14	turquoise	0.927927273
ENSG00000102225.11	CDK16	turquoise	0.937778942
ENSG00000059758.3	CDK17	turquoise	0.951399395
ENSG00000117266.11	CDK18	brown	0.909099179
ENSG00000155111.10	CDK19	brown	0.910606541
ENSG00000123374.6	CDK2	black	0.909966823
ENSG00000156345.13	CDK20	turquoise	0.944745506

ENSG00000111328.2	CDK2AP1	green	0.752755432
ENSG00000167797.3	CDK2AP2	cyan	0.790428803
ENSG00000250506.2	CDK3	brown	0.654943388
ENSG00000135446.12	CDK4	turquoise	0.787295474
ENSG00000164885.8	CDK5	turquoise	0.912816523
ENSG00000176749.4	CDK5R1	turquoise	0.947573499
ENSG00000171450.4	CDK5R2	turquoise	0.853263326
ENSG00000101391.16	CDK5RAP1	turquoise	0.94880841
ENSG00000136861.13	CDK5RAP2	brown	0.921569232
ENSG00000108465.10	CDK5RAP3	yellow	0.949576861
ENSG00000105810.5	CDK6	brown	0.824185595
ENSG00000134058.6	CDK7	turquoise	0.960616748
ENSG00000132964.7	CDK8	turquoise	0.95068663
ENSG00000136807.7	CDK9	yellow	0.943263061
ENSG00000145996.7	CDKAL1	turquoise	0.835070352
ENSG00000100490.5	CDKL1	turquoise	0.863683115
ENSG00000138769.6	CDKL2	turquoise	0.913336106
ENSG00000006837.7	CDKL3	turquoise	0.872900382
ENSG00000124762.9	CDKN1A	black	0.751826538
ENSG00000111276.6	CDKN1B	blue	0.910702669
ENSG00000129757.8	CDKN1C	brown	0.839689777
ENSG00000168564.5	CDKN2AIP	turquoise	0.905304412
ENSG00000237190.3	CDKN2AIPNL	turquoise	0.947361735
ENSG00000147883.9	CDKN2B	blue	0.555143538
ENSG00000123080.6	CDKN2C	brown	0.634598765
ENSG00000129355.6	CDKN2D	turquoise	0.904649991
ENSG00000100526.15	CDKN3	turquoise	0.847752952
ENSG00000185267.5	CDNF	brown	0.568061018
ENSG00000129596.4	CDO1	turquoise	0.942736629
ENSG00000064309.10	CDON	turquoise	0.864433096
ENSG00000205643.6	CDPF1	blue	0.77610961
ENSG00000184258.5	CDR1	midnightblue	0.668281505
ENSG00000140743.3	CDR2	turquoise	0.947176647
ENSG00000109089.7	CDR2L	brown	0.899454054
ENSG00000239704.6	CDRT4	yellow	0.911976937
ENSG00000163624.5	CDS1	turquoise	0.923899495
ENSG00000101290.9	CDS2	turquoise	0.956870769
ENSG00000167513.4	CDT1	greenyellow	0.750739724
ENSG00000091527.11	CDV3	turquoise	0.824437267
ENSG00000153046.13	CDYL	turquoise	0.883410386
ENSG00000166446.10	CDYL2	turquoise	0.900412225
ENSG00000186567.8	CEACAM19	turquoise	0.670116449
ENSG00000245848.2	CEBPA	turquoise	0.572779737
ENSG00000267296.2	CEBPA-AS1	turquoise	0.793546353
ENSG00000172216.4	CEBPB	magenta	0.683546511
ENSG00000221869.4	CEBPD	black	0.758433539
ENSG00000153879.4	CEBPG	cyan	0.922467113
ENSG00000115816.9	CEBPZ	turquoise	0.879865515

ENSG00000093072.11	CECR1	blue	0.757946812
ENSG00000099954.14	CECR2	blue	0.793186208
ENSG00000069998.8	CECR5	turquoise	0.930066323
ENSG00000185837.3	CECR5-AS1	turquoise	0.602567227
ENSG00000183307.3	CECR6	turquoise	0.895326218
ENSG00000237438.2	CECR7	turquoise	0.665417556
ENSG00000170835.10	CEL	grey	0.536067326
ENSG00000142789.15	CELA3A	grey	0.053849982
ENSG00000149187.13	CELF1	turquoise	0.969679912
ENSG00000048740.13	CELF2	turquoise	0.952899983
ENSG00000159409.10	CELF3	turquoise	0.880821863
ENSG00000101489.14	CELF4	turquoise	0.900372847
ENSG00000161082.8	CELF5	turquoise	0.851648265
ENSG00000140488.10	CELF6	turquoise	0.844938443
ENSG00000143126.7	CELSR2	turquoise	0.925541515
ENSG00000008300.10	CELSR3	turquoise	0.91623772
ENSG00000184524.5	CEND1	turquoise	0.848120146
ENSG00000125817.7	CENPB	brown	0.674011583
ENSG00000177946.4	CENPBD1	turquoise	0.915423026
ENSG00000145241.6	CENPC	turquoise	0.92582846
ENSG00000153044.5	CENPH	turquoise	0.858377621
ENSG00000151849.10	CENPJ	turquoise	0.872220682
ENSG00000166451.9	CENPN	brown	0.815548424
ENSG00000138092.6	CENPO	turquoise	0.94938172
ENSG00000188312.9	CENPP	turquoise	0.904074788
ENSG00000031691.6	CENPQ	blue	0.83746166
ENSG00000102901.8	CENPT	turquoise	0.905529152
ENSG00000166582.5	CENPV	turquoise	0.91508513
ENSG00000203760.4	CENPW	turquoise	0.745073984
ENSG00000116198.8	CEP104	brown	0.867339215
ENSG00000168944.11	CEP120	turquoise	0.910317776
ENSG00000174799.6	CEP135	turquoise	0.731679539
ENSG00000110274.10	CEP164	yellow	0.970708576
ENSG00000143702.11	CEP170	turquoise	0.93074218
ENSG00000099814.11	CEP170B	turquoise	0.934270945
ENSG00000174007.7	CEP19	turquoise	0.915170052
ENSG00000101639.14	CEP192	turquoise	0.825157079
ENSG00000126001.11	CEP250	turquoise	0.946119874
ENSG00000198707.10	CEP290	turquoise	0.873074842
ENSG00000135837.11	CEP350	blue	0.862651615
ENSG00000106477.14	CEP41	turquoise	0.944675771
ENSG00000164118.8	CEP44	turquoise	0.829468068
ENSG00000166037.6	CEP57	turquoise	0.954700603
ENSG00000183137.10	CEP57L1	turquoise	0.848296333
ENSG00000182923.12	CEP63	turquoise	0.913702961
ENSG00000011523.9	CEP68	turquoise	0.959321021
ENSG00000114107.4	CEP70	turquoise	0.942620625
ENSG00000112877.6	CEP72	turquoise	0.949439257

ENSG00000101624.6	CEP76	turquoise	0.928340851
ENSG00000148019.8	CEP78	turquoise	0.875142116
ENSG00000130695.9	CEP85	turquoise	0.938663817
ENSG00000111860.9	CEP85L	blue	0.829820061
ENSG00000121289.13	CEP89	turquoise	0.929704133
ENSG00000258890.2	CEP95	yellow	0.914690634
ENSG00000182504.6	CEP97	turquoise	0.903309302
ENSG00000134255.9	CEPT1	turquoise	0.887621127
ENSG00000167123.14	CERCAM	brown	0.874639254
ENSG00000100422.9	CERK	turquoise	0.86319291
ENSG00000223802.3	CERS1	brown	0.455583277
ENSG00000143418.15	CERS2	brown	0.906241249
ENSG00000090661.7	CERS4	blue	0.850042716
ENSG00000139624.8	CERS5	turquoise	0.928053086
ENSG00000172292.10	CERS6	turquoise	0.917327656
ENSG00000198848.8	CES1	grey	0.342620941
ENSG00000172831.7	CES2	turquoise	0.909453591
ENSG00000172828.8	CES3	turquoise	0.848827499
ENSG00000172824.10	CES4A	yellow	0.93329896
ENSG00000147400.8	CETN2	pink	0.938862628
ENSG00000153140.4	CETN3	blue	0.859570213
ENSG00000224786.1	CETN4P	turquoise	0.746195859
ENSG00000243649.4	CFB	black	0.779701735
ENSG00000197766.3	CFD	red	0.614940717
ENSG00000153774.4	CFDP1	turquoise	0.928101543
ENSG00000000971.11	CFH	black	0.718067563
ENSG00000205403.8	CFI	black	0.751815322
ENSG00000172757.8	CFL1	turquoise	0.944406882
ENSG00000223820.5	CFL1P1	turquoise	0.865470335
ENSG00000165410.10	CFL2	brown	0.928806644
ENSG00000003402.15	CFLAR	blue	0.845629849
ENSG00000126759.8	CFP	yellow	0.755036892
ENSG00000163320.6	CGGBP1	turquoise	0.93765406
ENSG00000143375.10	CGN	brown	0.846749308
ENSG00000128849.9	CGNL1	green	0.78858458
ENSG00000138028.10	CGREF1	turquoise	0.908514473
ENSG00000100532.7	CGRRF1	blue	0.834985316
ENSG00000138135.5	CH25H	magenta	0.583453212
ENSG00000128965.7	CHAC1	turquoise	0.631268368
ENSG00000143942.4	CHAC2	blue	0.678314086
ENSG00000136457.5	CHAD	grey	0.666944165
ENSG00000100399.11	CHADL	brown	0.773044358
ENSG00000167670.11	CHAF1A	brown	0.750642803
ENSG00000159259.7	CHAF1B	turquoise	0.884176693
ENSG00000198824.4	CHAMP1	turquoise	0.937865422
ENSG00000070748.13	CHAT	midnightblue	0.803841859
ENSG00000172586.7	CHCHD1	turquoise	0.916847992
ENSG00000250479.4	CHCHD10	turquoise	0.796219822

ENSG00000106153.12	CHCHD2	turquoise	0.899396359
ENSG00000106554.7	CHCHD3	turquoise	0.939665346
ENSG00000163528.8	CHCHD4	turquoise	0.933415781
ENSG00000125611.11	CHCHD5	red	0.908945111
ENSG00000159685.6	CHCHD6	turquoise	0.920247289
ENSG00000170791.13	CHCHD7	turquoise	0.930118193
ENSG00000153922.6	CHD1	turquoise	0.804704552
ENSG00000131778.13	CHD1L	brown	0.879878819
ENSG00000173575.14	CHD2	blue	0.918302827
ENSG00000170004.12	CHD3	turquoise	0.887059643
ENSG00000111642.10	CHD4	turquoise	0.923858652
ENSG00000116254.13	CHD5	greenyellow	0.919075059
ENSG00000124177.10	CHD6	turquoise	0.903383483
ENSG00000171316.7	CHD7	brown	0.894269636
ENSG00000100888.8	CHD8	turquoise	0.966622441
ENSG00000177200.11	CHD9	turquoise	0.897701933
ENSG00000016391.6	CHDH	green	0.902314472
ENSG00000149554.8	CHEK1	turquoise	0.859887049
ENSG00000183765.16	CHEK2	blue	0.706543121
ENSG00000085872.10	CHERP	turquoise	0.945135013
ENSG00000072609.13	CHFR	turquoise	0.907068859
ENSG00000100604.8	CHGA	turquoise	0.852282586
ENSG00000089199.5	CHGB	turquoise	0.929665739
ENSG00000133048.8	CHI3L1	black	0.704527679
ENSG00000064886.9	CHI3L2	black	0.598154866
ENSG00000204116.7	CHIC1	turquoise	0.956856182
ENSG00000109220.6	CHIC2	cyan	0.896296885
ENSG00000177830.13	CHID1	turquoise	0.936712754
ENSG00000110721.7	CHKA	yellow	0.917702328
ENSG00000100288.15	CHKB	turquoise	0.905679569
ENSG00000205559.3	CHKB-AS1	turquoise	0.696854664
ENSG00000134121.5	CHL1	turquoise	0.920201453
ENSG00000224318.1	CHL1-AS2	red	0.71133421
ENSG00000188419.9	CHM	turquoise	0.951372413
ENSG00000203668.1	CHML	turquoise	0.85670263
ENSG00000131165.10	CHMP1A	turquoise	0.932912513
ENSG00000255112.2	CHMP1B	turquoise	0.917896991
ENSG00000130724.4	CHMP2A	red	0.961767751
ENSG00000083937.4	CHMP2B	turquoise	0.91669005
ENSG00000115561.10	CHMP3	turquoise	0.941126594
ENSG00000254505.4	CHMP4A	red	0.921850033
ENSG00000101421.3	CHMP4B	turquoise	0.936805507
ENSG00000258469.1	CHMP4BP1	black	0.579478366
ENSG00000086065.9	CHMP5	turquoise	0.947048844
ENSG00000176108.5	CHMP6	turquoise	0.841939724
ENSG00000147457.9	CHMP7	turquoise	0.97732949
ENSG00000128656.9	CHN1	turquoise	0.922068748
ENSG00000106069.16	CHN2	turquoise	0.88073477

ENSG00000154645.9	CHODL	turquoise	0.757390835
ENSG00000110172.7	CHORDC1	cyan	0.903026296
ENSG00000187446.7	CHP1	turquoise	0.913452825
ENSG00000166869.2	CHP2	turquoise	0.716716283
ENSG00000123989.9	CHPF	turquoise	0.881230502
ENSG00000033100.10	CHPF2	turquoise	0.881671202
ENSG00000111666.6	CHPT1	blue	0.797847824
ENSG00000104472.5	CHRAC1	brown	0.876103341
ENSG00000090539.11	CHRD	turquoise	0.672300879
ENSG00000101938.10	CHRDL1	green	0.705484892
ENSG00000168539.3	CHRM1	turquoise	0.815798136
ENSG00000133019.7	CHRM3	midnightblue	0.85609723
ENSG00000180720.6	CHRM4	turquoise	0.8656043
ENSG00000129749.3	CHRNA10	grey	0.676226717
ENSG00000080644.11	CHRNA3	turquoise	0.642694002
ENSG00000101204.11	CHRNA4	greenyellow	0.747866614
ENSG00000170175.6	CHRNB1	blue	0.832212309
ENSG00000160716.4	CHRNB2	turquoise	0.89763861
ENSG00000108556.7	CHRNE	blue	0.516130722
ENSG00000175264.3	CHST1	turquoise	0.783738848
ENSG00000115526.6	CHST10	turquoise	0.885284848
ENSG00000171310.6	CHST11	turquoise	0.918526244
ENSG00000136213.7	CHST12	turquoise	0.856152239
ENSG00000169105.6	CHST14	green	0.759515336
ENSG00000182022.13	CHST15	turquoise	0.924819405
ENSG00000175040.4	CHST2	turquoise	0.911707002
ENSG00000122863.5	CHST3	blue	0.739110083
ENSG00000135702.10	CHST5	turquoise	0.836478412
ENSG00000183196.4	CHST6	blue	0.705100311
ENSG00000147119.3	CHST7	green	0.705900777
ENSG00000131873.5	CHSY1	black	0.848933045
ENSG00000198108.3	CHSY3	turquoise	0.843920745
ENSG00000127586.12	CHTF18	yellow	0.913242825
ENSG00000168802.8	CHTF8	turquoise	0.929359104
ENSG00000160679.8	CHTOP	turquoise	0.955620344
ENSG00000213341.6	CHUK	turquoise	0.879188145
ENSG00000258289.3	CHURC1	blue	0.886950213
ENSG00000144021.2	CIAO1	turquoise	0.970095076
ENSG00000005194.10	CIAPIN1	turquoise	0.960232895
ENSG00000185043.6	CIB1	blue	0.687708618
ENSG00000136425.8	CIB2	red	0.732947651
ENSG00000079432.3	CIC	turquoise	0.933893839
ENSG00000271529.1	CICP14	yellow	0.876739376
ENSG00000176194.13	CIDEA	turquoise	0.665775728
ENSG00000136305.7	CIDEB	turquoise	0.586481635
ENSG00000186162.6	CIDECP	turquoise	0.88555487
ENSG00000179583.13	CIITA	magenta	0.858934744
ENSG00000100865.10	CINP	turquoise	0.924901186

ENSG00000138433.11	CIR1	turquoise	0.939935417
ENSG00000099622.9	CIRBP	turquoise	0.76077172
ENSG00000267493.2	CIRBP-AS1	turquoise	0.736101768
ENSG00000141076.13	CIRH1A	turquoise	0.95938659
ENSG00000122873.7	CISD1	turquoise	0.948568775
ENSG00000145354.5	CISD2	turquoise	0.911909704
ENSG00000230055.2	CISD3	red	0.909263257
ENSG00000114737.11	CISH	black	0.708942586
ENSG00000122966.9	CIT	turquoise	0.898435332
ENSG00000125931.6	CITED1	turquoise	0.644003283
ENSG00000164442.8	CITED2	turquoise	0.850125717
ENSG00000179862.5	CITED4	turquoise	0.718384969
ENSG00000273145.1	CITF22-92A6.1	turquoise	0.705100688
ENSG00000148337.15	CIZ1	turquoise	0.936437422
ENSG00000136108.10	CKAP2	turquoise	0.934156406
ENSG00000136026.9	CKAP4	blue	0.848522162
ENSG00000175216.10	CKAP5	turquoise	0.960441669
ENSG00000166165.8	CKB	turquoise	0.827631595
ENSG00000217555.8	CKLF	turquoise	0.810140597
ENSG00000104879.4	CKM	grey	0.054354364
ENSG00000223572.5	CKMT1A	turquoise	0.854260763
ENSG00000237289.5	CKMT1B	turquoise	0.838732798
ENSG00000131730.11	CKMT2	brown	0.537420954
ENSG00000247572.3	CKMT2-AS1	turquoise	0.758281434
ENSG00000173207.8	CKS1B	turquoise	0.835499614
ENSG00000123975.4	CKS2	turquoise	0.751666366
ENSG00000074054.13	CLASP1	turquoise	0.954326409
ENSG00000163539.11	CLASP2	turquoise	0.918071809
ENSG00000104859.10	CLASRP	yellow	0.891649001
ENSG00000016602.8	CLCA4	brown	0.7908309
ENSG00000121940.11	CLCC1	turquoise	0.937930697
ENSG00000114859.10	CLCN2	blue	0.8457871
ENSG00000109572.9	CLCN3	turquoise	0.923059019
ENSG00000073464.7	CLCN4	turquoise	0.939257557
ENSG00000171365.11	CLCN5	blue	0.882966597
ENSG00000011021.17	CLCN6	turquoise	0.955101298
ENSG00000103249.13	CLCN7	turquoise	0.930669536
ENSG00000163347.5	CLDN1	grey	0.33753748
ENSG00000134873.5	CLDN10	green	0.713822864
ENSG00000013297.6	CLDN11	brown	0.913176811
ENSG00000157224.11	CLDN12	turquoise	0.906873922
ENSG00000106404.9	CLDN15	blue	0.67985093
ENSG00000184113.8	CLDN5	black	0.723207527
ENSG00000184697.6	CLDN6	midnightblue	0.842931058
ENSG00000213937.3	CLDN9	midnightblue	0.695166592
ENSG00000080822.12	CLDND1	brown	0.881239761
ENSG00000160318.2	CLDND2	red	0.685834576
ENSG00000105472.8	CLEC11A	red	0.845816462

ENSG00000176435.6	CLEC14A	brown	0.558187997
ENSG00000038532.10	CLEC16A	turquoise	0.917447751
ENSG00000140839.7	CLEC18B	green	0.523798116
ENSG00000150048.6	CLEC1A	black	0.79192012
ENSG00000110852.4	CLEC2B	black	0.769669761
ENSG00000069493.10	CLEC2D	blue	0.627462047
ENSG00000236279.2	CLEC2L	turquoise	0.858309
ENSG00000163815.5	CLEC3B	grey	0.330192259
ENSG00000111729.8	CLEC4A	yellow	0.769384162
ENSG00000182566.8	CLEC4G	turquoise	0.739676392
ENSG00000268297.1	CLEC4GP1	turquoise	0.485019401
ENSG00000172243.13	CLEC7A	magenta	0.879768437
ENSG00000197992.2	CLEC9A	magenta	0.641370017
ENSG00000153132.8	CLGN	turquoise	0.85138699
ENSG00000213719.4	CLIC1	black	0.90110494
ENSG00000155962.8	CLIC2	cyan	0.788455202
ENSG00000169583.12	CLIC3	grey	0.236872612
ENSG00000169504.10	CLIC4	blue	0.713562303
ENSG00000113282.9	CLINT1	turquoise	0.954145769
ENSG00000130779.15	CLIP1	turquoise	0.89600318
ENSG00000106665.11	CLIP2	brown	0.83806862
ENSG00000105270.10	CLIP3	turquoise	0.961268762
ENSG00000115295.15	CLIP4	turquoise	0.943122322
ENSG00000013441.11	CLK1	yellow	0.806259452
ENSG00000176444.14	CLK2	yellow	0.969620846
ENSG00000179335.14	CLK3	yellow	0.915445637
ENSG00000113240.8	CLK4	turquoise	0.881219926
ENSG00000165959.7	CLMN	brown	0.900506152
ENSG00000188603.12	CLN3	blue	0.896959888
ENSG00000102805.9	CLN5	blue	0.920973748
ENSG00000128973.7	CLN6	turquoise	0.875787764
ENSG00000182372.6	CLN8	brown	0.859972482
ENSG00000074201.4	CLNS1A	blue	0.902737099
ENSG00000134852.10	CLOCK	turquoise	0.946687783
ENSG00000172409.5	CLP1	turquoise	0.901768205
ENSG00000162129.8	CLPB	turquoise	0.848668963
ENSG00000125656.4	CLPP	turquoise	0.882921245
ENSG00000137392.5	CLPS	grey	0.104827647
ENSG00000104853.11	CLPTM1	turquoise	0.902204857
ENSG00000049656.9	CLPTM1L	turquoise	0.961571062
ENSG00000166855.5	CLPX	turquoise	0.92481328
ENSG00000171603.12	CLSTN1	turquoise	0.954371097
ENSG00000158258.11	CLSTN2	midnightblue	0.725870584
ENSG00000139182.9	CLSTN3	turquoise	0.927453921
ENSG00000122705.12	CLTA	turquoise	0.929609331
ENSG00000175416.8	CLTB	turquoise	0.906870998
ENSG00000141367.7	CLTC	turquoise	0.979294973
ENSG00000070371.11	CLTCL1	yellow	0.861091943

ENSG00000120885.15	CLU	green	0.813363131
ENSG00000103351.8	CLUAP1	turquoise	0.935483052
ENSG00000132361.12	CLUH	turquoise	0.920609424
ENSG00000131797.8	CLUHP3	turquoise	0.904699698
ENSG00000177182.6	CLVS1	turquoise	0.899801353
ENSG00000146352.8	CLVS2	turquoise	0.897604327
ENSG00000125246.11	CLYBL	turquoise	0.850737601
ENSG00000168405.10	CMAHP	black	0.718778327
ENSG00000111726.8	CMAS	turquoise	0.962533555
ENSG00000164237.4	CMBL	blue	0.811102338
ENSG00000187118.8	CMC1	turquoise	0.925879839
ENSG00000103121.4	CMC2	turquoise	0.901510366
ENSG00000153815.12	CMIP	turquoise	0.90541577
ENSG00000174600.9	CMKLR1	magenta	0.87207723
ENSG00000272958.1	CMP21-97G8.2	grey	0.605914484
ENSG00000162368.9	CMPK1	turquoise	0.809189169
ENSG00000134326.7	CMPK2	turquoise	0.914891681
ENSG00000184220.6	CMSS1	turquoise	0.945553904
ENSG00000089505.13	CMTM1	turquoise	0.890624978
ENSG00000140932.5	CMTM2	turquoise	0.599474675
ENSG00000140931.15	CMTM3	magenta	0.765491731
ENSG00000183723.8	CMTM4	blue	0.873063624
ENSG00000166091.15	CMTM5	brown	0.831922249
ENSG00000091317.7	CMTM6	blue	0.79628918
ENSG00000153551.9	CMTM7	magenta	0.924165891
ENSG00000170293.4	CMTM8	black	0.737802573
ENSG00000137200.8	CMTR1	turquoise	0.968488971
ENSG00000180917.12	CMTR2	turquoise	0.840562171
ENSG00000164309.10	CMYA5	blue	0.743502816
ENSG00000169714.12	CNBP	turquoise	0.921534423
ENSG00000150656.10	CNDP1	brown	0.829820621
ENSG00000133313.10	CNDP2	turquoise	0.862217975
ENSG00000205423.7	CNEP1R1	turquoise	0.940064947
ENSG00000105427.5	CNFN	turquoise	0.673684308
ENSG00000100528.7	CNIH1	turquoise	0.901928606
ENSG00000174871.6	CNIH2	turquoise	0.820081636
ENSG00000143786.3	CNIH3	turquoise	0.926818432
ENSG00000143771.7	CNIH4	turquoise	0.949612028
ENSG00000142675.13	CNKSR1	yellow	0.805909313
ENSG00000149970.10	CNKSR2	turquoise	0.920920183
ENSG00000153721.13	CNKSR3	brown	0.825388557
ENSG00000130176.3	CNN1	grey	0.350044282
ENSG00000064666.10	CNN2	black	0.693824359
ENSG00000117519.11	CNN3	blue	0.757159721
ENSG00000119946.9	CNNM1	turquoise	0.897898151
ENSG00000148842.13	CNNM2	turquoise	0.961756479
ENSG00000168763.11	CNNM3	turquoise	0.916980043
ENSG00000158158.7	CNNM4	turquoise	0.944323107

ENSG00000125107.12	CNOT1	turquoise	0.920314137
ENSG00000182973.14	CNOT10	turquoise	0.952357164
ENSG00000158435.3	CNOT11	blue	0.935066434
ENSG00000111596.7	CNOT2	turquoise	0.94052961
ENSG00000088038.13	CNOT3	turquoise	0.944345965
ENSG00000080802.14	CNOT4	turquoise	0.946736914
ENSG00000113300.7	CNOT6	turquoise	0.932974785
ENSG00000138767.8	CNOT6L	turquoise	0.805154214
ENSG00000198791.7	CNOT7	turquoise	0.952974735
ENSG00000155508.9	CNOT8	turquoise	0.79789507
ENSG00000173786.12	CNP	brown	0.918492386
ENSG00000115649.11	CNPPD1	turquoise	0.879093521
ENSG00000257727.1	CNPY2	turquoise	0.940724906
ENSG00000137161.12	CNPY3	red	0.867425987
ENSG00000166997.3	CNPY4	blue	0.772527077
ENSG00000118432.11	CNR1	turquoise	0.778621044
ENSG00000119865.4	CNRIP1	turquoise	0.863296793
ENSG00000162852.9	CNST	turquoise	0.955330933
ENSG00000176563.5	CNTD1	turquoise	0.83977844
ENSG00000105219.4	CNTD2	turquoise	0.650537998
ENSG00000242689.1	CNTF	blue	0.5610262
ENSG00000122756.10	CNTFR	green	0.707589979
ENSG00000044459.10	CNTLN	blue	0.852975781
ENSG00000018236.10	CNTN1	turquoise	0.796720449
ENSG00000184144.5	CNTN2	brown	0.9482509
ENSG00000113805.8	CNTN3	pink	0.891494963
ENSG00000144619.10	CNTN4	turquoise	0.842573082
ENSG00000108797.7	CNTNAP1	turquoise	0.945473248
ENSG00000174469.13	CNTNAP2	turquoise	0.908211186
ENSG00000106714.13	CNTNAP3	turquoise	0.822969483
ENSG00000152910.14	CNTNAP4	brown	0.82635962
ENSG00000119397.12	CNTRL	pink	0.887619509
ENSG00000170037.9	CNTROB	turquoise	0.885369842
ENSG00000106603.13	COA1	turquoise	0.962480624
ENSG00000183978.7	COA3	red	0.961212742
ENSG00000181924.6	COA4	brown	0.771796214
ENSG00000183513.4	COA5	blue	0.876685094
ENSG00000168275.10	COA6	turquoise	0.79513321
ENSG00000068120.10	COASY	turquoise	0.921205169
ENSG00000106078.13	COBL	brown	0.812521493
ENSG00000082438.11	COBLL1	brown	0.54140238
ENSG00000100473.11	COCH	turquoise	0.816825334
ENSG00000166685.7	COG1	turquoise	0.969917582
ENSG00000135775.9	COG2	turquoise	0.842579375
ENSG00000136152.10	COG3	turquoise	0.943521515
ENSG00000103051.14	COG4	blue	0.911756173
ENSG00000164597.9	COG5	turquoise	0.90733229
ENSG00000133103.12	COG6	turquoise	0.940729306

ENSG00000168434.8	COG7	yellow	0.957561049
ENSG00000213380.9	COG8	turquoise	0.958798096
ENSG00000121058.4	COIL	turquoise	0.962421501
ENSG00000060718.14	COL11A1	turquoise	0.774993966
ENSG00000204248.6	COL11A2	yellow	0.861292529
ENSG00000111799.16	COL12A1	greenyellow	0.707087908
ENSG00000084636.13	COL16A1	yellow	0.831787472
ENSG00000065618.12	COL17A1	turquoise	0.739121269
ENSG00000182871.10	COL18A1	blue	0.614130305
ENSG00000108821.9	COL1A1	turquoise	0.6244884
ENSG00000164692.13	COL1A2	brown	0.529829875
ENSG00000101203.12	COL20A1	grey	0.390018759
ENSG00000124749.12	COL21A1	pink	0.769664062
ENSG00000171502.10	COL24A1	turquoise	0.714219616
ENSG00000188517.10	COL25A1	turquoise	0.711486864
ENSG00000160963.9	COL26A1	turquoise	0.814732928
ENSG00000196739.10	COL27A1	blue	0.639277115
ENSG00000215018.5	COL28A1	pink	0.768149828
ENSG00000168542.8	COL3A1	grey	0.324938402
ENSG00000187498.10	COL4A1	black	0.808430727
ENSG00000134871.13	COL4A2	black	0.817655417
ENSG00000169031.14	COL4A3	brown	0.572554658
ENSG00000113163.11	COL4A3BP	turquoise	0.969490619
ENSG00000081052.10	COL4A4	brown	0.605368545
ENSG00000188153.8	COL4A5	brown	0.909355688
ENSG00000204262.7	COL5A2	turquoise	0.724708912
ENSG00000080573.6	COL5A3	grey	0.463924984
ENSG00000142156.10	COL6A1	yellow	0.837281546
ENSG00000142173.10	COL6A2	black	0.607222743
ENSG00000228252.4	COL6A4P2	grey	0.545407943
ENSG00000114270.11	COL7A1	yellow	0.916919354
ENSG00000171812.6	COL8A2	magenta	0.808958222
ENSG00000112280.11	COL9A1	grey	0.393865855
ENSG00000049089.9	COL9A2	brown	0.849891005
ENSG00000092758.11	COL9A3	brown	0.802504166
ENSG00000196167.5	COLCA1	pink	0.848705697
ENSG00000214290.3	COLCA2	green	0.734052183
ENSG00000118004.13	COLEC11	grey	0.23692666
ENSG00000158270.10	COLEC12	brown	0.57442081
ENSG00000130309.6	COLGALT1	turquoise	0.788894176
ENSG00000198756.6	COLGALT2	brown	0.899735584
ENSG00000206561.8	COLQ	yellow	0.905454676
ENSG00000173163.6	COMMD1	turquoise	0.907674287
ENSG00000145781.4	COMMD10	turquoise	0.914074305
ENSG00000114744.4	COMMD2	turquoise	0.88788101
ENSG00000148444.11	COMMD3	turquoise	0.953854017
ENSG00000140365.11	COMMD4	red	0.899845864
ENSG00000170619.8	COMMD5	red	0.90248076

ENSG00000188243.8	COMMD6	red	0.920393407
ENSG00000149600.7	COMMD7	turquoise	0.918453495
ENSG00000169019.9	COMMD8	turquoise	0.893590239
ENSG00000110442.7	COMMD9	turquoise	0.941800097
ENSG00000093010.7	COMT	blue	0.821442856
ENSG00000165644.6	COMTD1	turquoise	0.688355246
ENSG00000122218.10	COPA	turquoise	0.968816379
ENSG00000129083.8	COPB1	turquoise	0.904801121
ENSG00000184432.5	COPB2	turquoise	0.877634708
ENSG00000105669.8	COPE	red	0.944784081
ENSG00000181789.10	COPG1	turquoise	0.963653229
ENSG00000158623.10	COPG2	turquoise	0.946893428
ENSG00000172301.6	COPRS	turquoise	0.923402089
ENSG00000166200.10	COPS2	turquoise	0.951431858
ENSG00000141030.8	COPS3	turquoise	0.969190054
ENSG00000138663.4	COPS4	turquoise	0.95429173
ENSG00000121022.9	COPS5	turquoise	0.931924799
ENSG00000168090.5	COPS6	turquoise	0.951365393
ENSG00000111652.5	COPS7A	turquoise	0.963868982
ENSG00000144524.13	COPS7B	turquoise	0.931066701
ENSG00000198612.6	COPS8	turquoise	0.953059951
ENSG00000111481.5	COPZ1	turquoise	0.964770344
ENSG00000005243.5	COPZ2	brown	0.563319833
ENSG00000135469.9	COQ10A	turquoise	0.851150676
ENSG00000115520.4	COQ10B	turquoise	0.9530718
ENSG00000173085.9	COQ2	turquoise	0.867034421
ENSG00000132423.6	COQ3	turquoise	0.938016038
ENSG00000167113.6	COQ4	turquoise	0.918834763
ENSG00000110871.10	COQ5	turquoise	0.943181666
ENSG00000119723.11	COQ6	turquoise	0.968112387
ENSG00000167186.6	COQ7	turquoise	0.943507282
ENSG00000088682.9	COQ9	blue	0.860254505
ENSG00000102879.11	CORO1A	turquoise	0.886391692
ENSG00000172725.9	CORO1B	blue	0.873562932
ENSG00000110880.6	CORO1C	brown	0.804479235
ENSG00000106789.8	CORO2A	turquoise	0.843610509
ENSG00000103647.8	CORO2B	turquoise	0.945104449
ENSG00000167549.14	CORO6	turquoise	0.775208509
ENSG00000262246.1	CORO7	turquoise	0.86285781
ENSG00000241563.2	CORT	midnightblue	0.894097714
ENSG00000103187.6	COTL1	blue	0.749941628
ENSG00000006695.6	COX10	turquoise	0.940598138
ENSG00000236088.5	COX10-AS1	turquoise	0.894411978
ENSG00000166260.6	COX11	turquoise	0.948750505
ENSG00000178449.4	COX14	red	0.943520255
ENSG00000014919.8	COX15	blue	0.890750263
ENSG00000133983.10	COX16	turquoise	0.912140416
ENSG00000138495.2	COX17	red	0.925051876

ENSG00000163626.12	COX18	turquoise	0.951812206
ENSG00000240230.1	COX19	turquoise	0.929142076
ENSG00000203667.5	COX20	blue	0.85605284
ENSG00000213025.2	COX20P1	grey	0.098582282
ENSG00000131143.4	COX4I1	red	0.950920302
ENSG00000131055.4	COX4I2	grey	0.284352107
ENSG00000178741.7	COX5A	turquoise	0.928057652
ENSG00000135940.2	COX5B	red	0.945458271
ENSG00000237082.1	COX5BP6	brown	0.72863518
ENSG00000111775.2	COX6A1	red	0.937839348
ENSG00000156885.4	COX6A2	red	0.481556818
ENSG00000126267.4	COX6B1	red	0.949630591
ENSG00000164919.6	COX6C	turquoise	0.899981663
ENSG00000161281.6	COX7A1	red	0.502120063
ENSG00000112695.7	COX7A2	turquoise	0.900586532
ENSG00000115944.10	COX7A2L	turquoise	0.947419763
ENSG00000131174.4	COX7B	turquoise	0.924984575
ENSG00000127184.6	COX7C	turquoise	0.895922399
ENSG00000176340.3	COX8A	red	0.947091985
ENSG00000047457.9	CP	black	0.731354883
ENSG00000091704.5	CPA1	grey	0.089011169
ENSG00000235903.3	CPB2-AS1	brown	0.626535911
ENSG00000108582.7	CPD	turquoise	0.838425233
ENSG00000109472.9	CPE	green	0.911829915
ENSG00000214575.5	CPEB1	turquoise	0.889799893
ENSG00000137449.11	CPEB2	turquoise	0.861607221
ENSG00000107864.10	CPEB3	turquoise	0.912980479
ENSG00000113742.8	CPEB4	turquoise	0.826390615
ENSG00000168993.10	CPLX1	turquoise	0.759695856
ENSG00000145920.10	CPLX2	turquoise	0.806045488
ENSG00000213578.4	CPLX3	midnightblue	0.833950514
ENSG00000135678.7	CPM	brown	0.806953277
ENSG00000214078.7	CPNE1	turquoise	0.871624147
ENSG00000140848.12	CPNE2	yellow	0.827491819
ENSG00000085719.7	CPNE3	green	0.884495286
ENSG00000196353.7	CPNE4	turquoise	0.802834809
ENSG00000124772.7	CPNE5	turquoise	0.851246402
ENSG00000100884.5	CPNE6	turquoise	0.885168285
ENSG00000178773.10	CPNE7	turquoise	0.701611152
ENSG00000139117.9	CPNE8	blue	0.715175411
ENSG00000144550.8	CPNE9	midnightblue	0.747736211
ENSG00000144410.4	CPO	turquoise	0.814934676
ENSG00000080819.2	CPOX	brown	0.867663117
ENSG00000103381.7	CPPED1	brown	0.931496148
ENSG00000104324.11	CPQ	blue	0.811264766
ENSG00000021826.10	CPS1	brown	0.736606166
ENSG00000071894.10	CPSF1	turquoise	0.859736054
ENSG00000165934.8	CPSF2	blue	0.952400227

ENSG00000119203.9	CPSF3	turquoise	0.921722979
ENSG00000127054.14	CPSF3L	turquoise	0.899324603
ENSG00000160917.10	CPSF4	turquoise	0.933913097
ENSG00000111605.12	CPSF6	turquoise	0.957811821
ENSG00000149532.11	CPSF7	turquoise	0.947829233
ENSG00000110090.8	CPT1A	green	0.891770729
ENSG00000205560.8	CPT1B	yellow	0.91742393
ENSG00000169169.10	CPT1C	turquoise	0.924350697
ENSG00000157184.5	CPT2	green	0.869959289
ENSG00000106066.9	CPVL	turquoise	0.844463119
ENSG00000121898.8	CPXM2	grey	0.320630436
ENSG00000166426.7	CRABP1	turquoise	0.80047744
ENSG00000143320.4	CRABP2	midnightblue	0.811589766
ENSG00000169372.8	CRADD	turquoise	0.906243078
ENSG00000007545.11	CRAMP1L	turquoise	0.911491572
ENSG00000095321.12	CRAT	blue	0.790279851
ENSG00000148204.7	CRB2	blue	0.716479951
ENSG00000113851.9	CRBN	turquoise	0.94028631
ENSG00000241258.2	CRCP	turquoise	0.971319252
ENSG00000118260.10	CREB1	turquoise	0.864304182
ENSG00000107175.6	CREB3	turquoise	0.918125029
ENSG00000157613.6	CREB3L1	grey	0.329057405
ENSG00000182158.10	CREB3L2	blue	0.896937967
ENSG00000143578.11	CREB3L4	blue	0.712568283
ENSG00000146592.12	CREB5	brown	0.901669188
ENSG00000005339.8	CREBBP	turquoise	0.909621641
ENSG00000111269.2	CREBL2	turquoise	0.937273799
ENSG00000164463.8	CREBRF	turquoise	0.863006239
ENSG00000137504.9	CREBZF	turquoise	0.874749386
ENSG00000143162.7	CREG1	turquoise	0.930449085
ENSG00000175874.5	CREG2	turquoise	0.76912099
ENSG00000163703.13	CRELD1	turquoise	0.907938347
ENSG00000184164.10	CRELD2	turquoise	0.81918424
ENSG00000095794.15	CREM	turquoise	0.880989884
ENSG00000147571.3	CRH	turquoise	0.638815338
ENSG00000145708.6	CRHBP	greenyellow	0.783263776
ENSG00000120088.10	CRHR1	turquoise	0.773013267
ENSG00000204650.9	CRHR1-IT1	turquoise	0.922589005
ENSG00000106113.14	CRHR2	turquoise	0.794148741
ENSG00000150938.5	CRIM1	turquoise	0.838990367
ENSG00000182809.6	CRIP2	turquoise	0.880374996
ENSG00000179979.7	CRIPAK	yellow	0.924000734
ENSG00000119878.5	CRIPT	turquoise	0.905762205
ENSG00000103196.7	CRISPLD2	black	0.723528381
ENSG00000167193.7	CRK	turquoise	0.855949954
ENSG00000099942.8	CRKL	turquoise	0.896207194
ENSG00000176390.10	CRLF3	turquoise	0.905319514
ENSG00000088766.7	CRLS1	turquoise	0.928076519

ENSG00000072832.10	CRMP1	turquoise	0.937157148
ENSG00000245694.4	CRNDE	pink	0.827629373
ENSG00000101343.10	CRNKL1	turquoise	0.940837288
ENSG00000058453.12	CROCC	pink	0.865373187
ENSG00000215908.5	CROCCP2	turquoise	0.830926179
ENSG00000080947.10	CROCCP3	yellow	0.895781427
ENSG00000005469.7	CROT	blue	0.868500208
ENSG00000095713.9	CRTAC1	greenyellow	0.783212464
ENSG00000170275.10	CRTAP	brown	0.780266241
ENSG00000105662.11	CRTC1	turquoise	0.86961768
ENSG00000160741.12	CRTC2	blue	0.866130245
ENSG00000140577.11	CRTC3	brown	0.830820102
ENSG00000008405.7	CRY1	turquoise	0.812116405
ENSG00000121671.7	CRY2	turquoise	0.904191115
ENSG00000109846.3	CRYAB	brown	0.600582642
ENSG00000163499.7	CRYBA2	midnightblue	0.701542617
ENSG00000244752.2	CRYBB2	turquoise	0.399883781
ENSG00000100058.8	CRYBB2P1	turquoise	0.663793729
ENSG00000118231.4	CRYGD	midnightblue	0.744808598
ENSG00000213139.3	CRYGS	grey	0.747620084
ENSG00000165475.9	CRYL1	blue	0.749658133
ENSG00000103316.6	CRYM	turquoise	0.766845613
ENSG00000116791.9	CRYZ	blue	0.747161788
ENSG00000205758.7	CRYZL1	turquoise	0.923954681
ENSG00000062485.14	CS	turquoise	0.973668557
ENSG00000139631.14	CSAD	yellow	0.969095614
ENSG00000198930.8	CSAG1	grey	0.245440373
ENSG00000172346.10	CSDC2	grey	0.298565188
ENSG00000009307.11	CSDE1	turquoise	0.871158197
ENSG00000124207.12	CSE1L	turquoise	0.965813959
ENSG00000184371.9	CSF1	black	0.850702143
ENSG00000182578.9	CSF1R	magenta	0.914756043
ENSG00000198223.10	CSF2RA	magenta	0.869313901
ENSG00000108342.8	CSF3	black	0.495720935
ENSG00000119535.13	CSF3R	magenta	0.853427469
ENSG00000147408.10	CSGALNACT1	green	0.845908683
ENSG00000169826.6	CSGALNACT2	turquoise	0.816595094
ENSG00000103653.12	CSK	blue	0.866486189
ENSG00000183117.13	CSMD1	turquoise	0.889063467
ENSG00000121904.13	CSMD2	turquoise	0.892929145
ENSG00000164796.13	CSMD3	turquoise	0.842182842
ENSG00000113712.12	CSNK1A1	turquoise	0.899917232
ENSG00000141551.10	CSNK1D	turquoise	0.941882928
ENSG00000213923.6	CSNK1E	turquoise	0.885292363
ENSG00000169118.11	CSNK1G1	turquoise	0.934750276
ENSG00000133275.11	CSNK1G2	turquoise	0.898071974
ENSG00000151292.13	CSNK1G3	turquoise	0.949140626
ENSG00000101266.12	CSNK2A1	turquoise	0.969837743

ENSG00000070770.4	CSNK2A2	turquoise	0.957749585
ENSG00000204435.9	CSNK2B	turquoise	0.933195965
ENSG00000173546.7	CSPG4	grey	0.55941376
ENSG00000114646.5	CSPG5	green	0.559616776
ENSG00000104218.9	CSPP1	pink	0.958381596
ENSG00000144655.10	CSRNP1	black	0.753016871
ENSG00000110925.2	CSRNP2	turquoise	0.969099699
ENSG00000178662.11	CSRNP3	turquoise	0.918120887
ENSG00000159176.9	CSRP1	brown	0.755108055
ENSG00000175183.5	CSRP2	black	0.60081965
ENSG00000149474.9	CSRP2BP	turquoise	0.949388087
ENSG00000101439.4	CST3	green	0.509293888
ENSG00000077984.4	CST7	grey	0.181902744
ENSG00000160213.5	CSTB	red	0.905097874
ENSG00000101138.7	CSTF1	turquoise	0.967996445
ENSG00000101811.9	CSTF2	turquoise	0.96094955
ENSG00000177613.7	CSTF2T	turquoise	0.936563093
ENSG00000176102.7	CSTF3	turquoise	0.971754957
ENSG00000225670.3	CTA-134P22.2	turquoise	0.76477177
ENSG00000261353.1	CTA-14H9.5	red	0.568972666
ENSG00000259891.1	CTA-204B4.2	brown	0.796243045
ENSG00000244625.1	CTA-211A9.5	turquoise	0.756016125
ENSG00000226328.2	CTA-217C2.1	turquoise	0.959166929
ENSG00000203280.3	CTA-221G9.11	turquoise	0.843438228
ENSG00000273424.1	CTA-223H9.9	grey	0.51835159
ENSG00000272720.1	CTA-228A9.3	yellow	0.923685909
ENSG00000272942.1	CTA-246H3.12	turquoise	0.571231198
ENSG00000260655.1	CTA-250D10.23	magenta	0.447532163
ENSG00000231084.2	CTA-253N17.1	green	0.647566337
ENSG00000273287.1	CTA-268H5.14	turquoise	0.597435027
ENSG00000271133.1	CTA-293F17.1	grey	0.338958809
ENSG00000260708.1	CTA-29F11.1	turquoise	0.824471468
ENSG00000272072.1	CTA-363E19.2	turquoise	0.61723496
ENSG00000272821.1	CTA-384D8.36	grey	0.789718159
ENSG00000272977.1	CTA-390C10.10	turquoise	0.623394583
ENSG00000261188.1	CTA-445C9.14	turquoise	0.927791547
ENSG00000260065.1	CTA-445C9.15	turquoise	0.518140036
ENSG00000227117.2	CTA-85E5.10	grey	0.209080085
ENSG00000230051.1	CTA-929C8.8	turquoise	0.780298365
ENSG00000250318.1	CTA-963H5.5	pink	0.802051696
ENSG00000237015.1	CTA-984G1.5	yellow	0.735241485
ENSG00000231405.1	CTA-992D9.7	turquoise	0.70706427
ENSG00000272568.1	CTB-113D17.1	turquoise	0.598703255
ENSG00000271737.1	CTB-113I20.2	red	0.663776941
ENSG00000272112.1	CTB-113P19.5	turquoise	0.671120382
ENSG00000273237.1	CTB-119C2.1	turquoise	0.802609986
ENSG00000248367.1	CTB-129O4.1	yellow	0.690569603
ENSG00000266469.1	CTB-131K11.1	turquoise	0.93617862

ENSG00000273055.1	CTB-13F3.1	turquoise	0.676774933
ENSG00000272918.1	CTB-152G17.6	turquoise	0.800256718
ENSG00000267934.1	CTB-176F20.3	grey	0.460226565
ENSG00000253978.1	CTB-178M22.2	red	0.491223933
ENSG00000267348.2	CTB-179K24.3	turquoise	0.742769532
ENSG00000263726.1	CTB-187M2.3	turquoise	0.738383432
ENSG00000250284.1	CTB-1I21.1	grey	0.171875258
ENSG00000267317.1	CTB-25B13.12	blue	0.613306391
ENSG00000267715.1	CTB-25B13.6	pink	0.765327642
ENSG00000261596.1	CTB-31N19.3	blue	0.54435645
ENSG00000261526.1	CTB-31O20.2	yellow	0.82905378
ENSG00000267244.1	CTB-31O20.4	yellow	0.890308032
ENSG00000267498.1	CTB-32O4.2	brown	0.529003997
ENSG00000250692.1	CTB-35F21.4	turquoise	0.620986221
ENSG00000260686.1	CTB-36H16.2	turquoise	0.889475022
ENSG00000244921.2	CTB-36O1.7	grey	-0.172989612
ENSG00000267344.1	CTB-39G8.3	turquoise	0.6604201
ENSG00000254211.1	CTB-43E15.4	grey	0.203947502
ENSG00000250244.1	CTB-49A3.4	turquoise	0.571877216
ENSG00000269374.1	CTB-50E14.5	turquoise	0.818784638
ENSG00000267030.1	CTB-50L17.7	yellow	0.82640707
ENSG00000267769.1	CTB-50L17.9	turquoise	0.701482456
ENSG00000267169.1	CTB-55O6.12	blue	0.703122223
ENSG00000261005.1	CTB-58E17.1	green	0.573352052
ENSG00000266040.1	CTB-58E17.3	turquoise	0.533129793
ENSG00000267067.1	CTB-75G16.3	grey	0.710759541
ENSG00000159692.11	CTBP1	turquoise	0.960605911
ENSG00000196810.4	CTBP1-AS2	turquoise	0.935703107
ENSG00000175029.12	CTBP2	green	0.845193188
ENSG00000117151.8	CTBS	blue	0.865079485
ENSG00000270021.1	CTC-203F4.2	brown	0.455141418
ENSG00000245937.3	CTC-228N24.3	turquoise	0.867905692
ENSG00000248127.1	CTC-235G5.3	turquoise	0.741212017
ENSG00000268001.1	CTC-241F20.3	magenta	0.72420706
ENSG00000245317.2	CTC-241N9.1	turquoise	0.860332554
ENSG00000269246.1	CTC-246B18.10	grey	0.437247774
ENSG00000268262.1	CTC-246B18.8	turquoise	0.772669215
ENSG00000269814.1	CTC-273B12.10	turquoise	0.855187449
ENSG00000269423.1	CTC-273B12.6	grey	0.086805025
ENSG00000261996.1	CTC-281F24.1	turquoise	0.834693825
ENSG00000263400.2	CTC-297N7.5	green	0.664436485
ENSG00000273290.1	CTC-297N7.8	turquoise	0.91532267
ENSG00000264791.1	CTC-304I17.2	grey	0.195048293
ENSG00000250167.1	CTC-321K16.1	green	0.524140702
ENSG00000267510.1	CTC-325H20.4	turquoise	0.720479486
ENSG00000249637.1	CTC-329D1.2	turquoise	0.724326095
ENSG00000271849.1	CTC-332L22.1	brown	0.562770921
ENSG00000271228.1	CTC-336P14.1	brown	0.568005469

ENSG00000233937.2	CTC-338M12.4	turquoise	0.845240205
ENSG00000250222.1	CTC-338M12.5	red	0.78332667
ENSG00000249335.1	CTC-340D7.1	turquoise	0.719103639
ENSG00000268401.1	CTC-344H19.4	turquoise	0.721041639
ENSG00000224186.4	CTC-349C3.1	yellow	0.891872384
ENSG00000242615.1	CTC-359D24.3	blue	0.620844842
ENSG00000262484.1	CTC-360G5.1	grey	0.747687004
ENSG00000269486.1	CTC-360G5.9	turquoise	0.715545307
ENSG00000272040.1	CTC-366B18.4	yellow	0.781888356
ENSG00000205041.1	CTC-425O23.2	yellow	0.81093987
ENSG00000248175.1	CTC-428G20.3	turquoise	0.757143694
ENSG00000272367.1	CTC-428H11.2	yellow	0.698294401
ENSG00000269427.1	CTC-429P9.1	turquoise	0.518214272
ENSG00000268087.1	CTC-429P9.2	turquoise	0.80428825
ENSG00000269044.1	CTC-429P9.3	turquoise	0.834236
ENSG00000267904.1	CTC-429P9.5	turquoise	0.782173725
ENSG00000268205.1	CTC-444N24.11	yellow	0.922175357
ENSG00000267871.1	CTC-444N24.6	turquoise	0.752621207
ENSG00000268713.1	CTC-444N24.8	yellow	0.817819414
ENSG00000266910.1	CTC-448F2.6	turquoise	0.8538186
ENSG00000267575.2	CTC-459F4.3	turquoise	0.890548528
ENSG00000267264.1	CTC-459F4.6	turquoise	0.801804098
ENSG00000251450.1	CTC-459I6.1	midnightblue	0.710549055
ENSG00000187534.5	CTC-471F3.4	grey	0.371911537
ENSG00000269069.1	CTC-471F3.5	turquoise	0.893059658
ENSG00000268166.1	CTC-471F3.6	turquoise	0.879052135
ENSG00000260160.1	CTC-471J1.2	grey	0.650031487
ENSG00000243806.1	CTC-484P3.1	grey	-0.021173976
ENSG00000270067.1	CTC-487M23.5	blue	0.682227074
ENSG00000272389.1	CTC-487M23.7	blue	0.540659525
ENSG00000250156.3	CTC-498M16.2	turquoise	0.738536547
ENSG00000271904.1	CTC-498M16.4	grey	0.32704752
ENSG00000267427.1	CTC-503J8.6	turquoise	0.886846932
ENSG00000267082.1	CTC-510F12.2	blue	0.5764449
ENSG00000263272.1	CTC-524C5.2	yellow	0.94853204
ENSG00000264515.1	CTC-525D6.1	turquoise	0.848864919
ENSG00000266248.1	CTC-525D6.2	turquoise	0.800762722
ENSG00000253251.2	CTC-534A2.2	turquoise	0.855827244
ENSG00000263990.1	CTC-542B22.2	yellow	0.886330796
ENSG00000251293.1	CTC-552D5.1	turquoise	0.76401436
ENSG00000267481.1	CTC-559E9.5	turquoise	0.855281351
ENSG00000267419.1	CTC-559E9.6	turquoise	0.808653369
ENSG00000267565.1	CTC-559E9.8	turquoise	0.811662592
ENSG00000178971.9	CTC1	turquoise	0.92099842
ENSG00000102974.10	CTCF	turquoise	0.930375685
ENSG00000258682.1	CTD-2002H8.2	turquoise	0.752344272
ENSG00000219665.4	CTD-2006C1.2	turquoise	0.700666095
ENSG00000271971.1	CTD-2006H14.2	yellow	0.844612694

ENSG00000248489.1	CTD-2007H13.3	turquoise	0.691971926
ENSG00000254595.1	CTD-2010I16.1	turquoise	0.846533129
ENSG00000261386.2	CTD-2012K14.6	grey	0.670612159
ENSG00000258847.1	CTD-2014B16.3	turquoise	0.767248492
ENSG00000249042.1	CTD-2015H6.3	turquoise	0.921088448
ENSG00000268362.1	CTD-2017D11.1	turquoise	0.676847605
ENSG00000267121.1	CTD-2020K17.1	turquoise	0.867914608
ENSG00000233175.2	CTD-2020K17.3	turquoise	0.83036134
ENSG00000264281.2	CTD-2031P19.4	red	0.628363325
ENSG00000269961.1	CTD-2033C11.1	red	0.490160486
ENSG00000245556.2	CTD-2037K23.2	turquoise	0.875855276
ENSG00000272520.1	CTD-2044J15.2	turquoise	0.906088358
ENSG00000263069.1	CTD-2047H16.4	turquoise	0.819432383
ENSG00000261329.1	CTD-2049O4.1	brown	0.834821309
ENSG00000258696.1	CTD-2058B24.3	brown	0.606794753
ENSG00000259031.1	CTD-2062F14.3	turquoise	0.718848243
ENSG00000272416.1	CTD-2081C10.7	grey	0.454988051
ENSG00000267014.1	CTD-2081K17.2	grey	0.144262367
ENSG00000248925.1	CTD-2083E4.6	green	0.579356429
ENSG00000261434.1	CTD-2083E4.7	turquoise	0.773796659
ENSG00000266930.2	CTD-2085J24.4	turquoise	0.633775121
ENSG00000267381.1	CTD-2086O20.3	turquoise	0.831374011
ENSG00000250360.1	CTD-2089N3.1	turquoise	0.735780358
ENSG00000270558.1	CTD-2124B8.2	turquoise	0.750861158
ENSG00000204666.3	CTD-2126E3.1	pink	0.900655198
ENSG00000269091.1	CTD-2126E3.3	pink	0.847656482
ENSG00000267890.1	CTD-2126E3.4	turquoise	0.617558108
ENSG00000267221.1	CTD-2132N18.2	blue	0.702281795
ENSG00000270010.1	CTD-2132N18.4	grey	0.574667083
ENSG00000272324.1	CTD-2154B17.4	grey	0.427331742
ENSG00000267260.1	CTD-2162K18.4	turquoise	0.876697829
ENSG00000267254.1	CTD-2162K18.5	turquoise	0.912761524
ENSG00000261360.1	CTD-2165H16.4	yellow	0.779155192
ENSG00000259327.1	CTD-2184D3.6	turquoise	0.872163891
ENSG00000272086.1	CTD-2186M15.3	red	0.754739536
ENSG00000268945.1	CTD-2192J16.26	grey	0.700763766
ENSG00000249577.1	CTD-2195M15.1	blue	0.630039976
ENSG00000259806.2	CTD-2196E14.4	turquoise	0.926287475
ENSG00000205106.2	CTD-2210P24.4	grey	0.166850145
ENSG00000270933.1	CTD-2227E11.1	turquoise	0.645467002
ENSG00000271892.1	CTD-2228A4.1	turquoise	0.53167484
ENSG00000188242.4	CTD-2228K2.5	turquoise	0.939204623
ENSG00000225138.3	CTD-2228K2.7	yellow	0.844752027
ENSG00000242198.1	CTD-2235C13.1	blue	0.661498681
ENSG00000271815.1	CTD-2235C13.3	turquoise	0.679339887
ENSG00000269288.1	CTD-2245F17.6	turquoise	0.620064731
ENSG00000248794.1	CTD-2248H3.1	turquoise	0.486666702
ENSG00000259802.1	CTD-2256P15.2	turquoise	0.809795181

ENSG00000247121.2	CTD-2260A17.2	turquoise	0.899891295
ENSG00000269815.1	CTD-2278I10.4	turquoise	0.771742359
ENSG00000254231.1	CTD-2284J15.1	turquoise	0.74892993
ENSG00000267655.1	CTD-2286N8.2	turquoise	0.55393147
ENSG00000271918.1	CTD-2287O16.5	turquoise	0.795102304
ENSG00000258768.2	CTD-2292M16.8	turquoise	0.871374494
ENSG00000253741.1	CTD-2292P10.4	turquoise	0.746802596
ENSG00000273018.1	CTD-2303H24.2	turquoise	0.61467988
ENSG00000272944.1	CTD-2308L22.1	turquoise	0.751817949
ENSG00000259685.1	CTD-2315E11.1	grey	0.263384589
ENSG00000267248.1	CTD-2319I12.4	turquoise	0.682877266
ENSG00000253596.1	CTD-2320G14.2	turquoise	0.749905745
ENSG00000272234.1	CTD-2325A15.5	blue	0.666642591
ENSG00000267939.1	CTD-2325M2.1	grey	0.387841492
ENSG00000259038.1	CTD-2325P2.4	grey	0.303386225
ENSG00000226958.1	CTD-2328D6.1	grey	-0.008525021
ENSG00000268225.1	CTD-2331H12.5	turquoise	0.865696297
ENSG00000253982.1	CTD-2336O2.1	red	0.782219337
ENSG00000268164.1	CTD-2337J16.1	turquoise	0.603593459
ENSG00000258733.1	CTD-2341M24.1	green	0.573417545
ENSG00000267277.1	CTD-2342J14.6	turquoise	0.663124604
ENSG00000265791.1	CTD-2349P21.10	yellow	0.773491022
ENSG00000266490.1	CTD-2349P21.9	turquoise	0.794592075
ENSG00000264098.1	CTD-2350C19.1	yellow	0.91523869
ENSG00000261731.2	CTD-2358C21.4	grey	0.58481659
ENSG00000247796.2	CTD-2366F13.1	turquoise	0.822412447
ENSG00000272123.1	CTD-2366F13.2	turquoise	0.781230882
ENSG00000176593.3	CTD-2368P22.1	yellow	0.911403611
ENSG00000175898.4	CTD-2369P2.2	grey	0.452803207
ENSG00000260871.1	CTD-2373J6.1	grey	0.313357993
ENSG00000271714.1	CTD-2377O17.1	turquoise	0.724548805
ENSG00000261195.1	CTD-2380F24.1	brown	0.63298686
ENSG00000204556.4	CTD-2514C3.1	green	0.636060949
ENSG00000246731.2	CTD-2514K5.2	brown	0.614803083
ENSG00000255182.1	CTD-2517M22.14	yellow	0.894661535
ENSG00000272323.1	CTD-2517O10.6	turquoise	0.778087559
ENSG00000269481.1	CTD-2521M24.6	blue	0.677527394
ENSG00000269640.1	CTD-2521M24.9	blue	0.512819281
ENSG00000271032.1	CTD-2527I21.14	blue	0.630753648
ENSG00000267874.1	CTD-2527I21.9	turquoise	0.731759595
ENSG00000267319.1	CTD-2528L19.3	turquoise	0.485206935
ENSG00000267152.1	CTD-2528L19.6	turquoise	0.813734596
ENSG00000255326.1	CTD-2530H12.4	turquoise	0.753191259
ENSG00000255434.1	CTD-2530H12.8	turquoise	0.691726648
ENSG00000254064.1	CTD-2530N21.4	turquoise	0.750462442
ENSG00000267523.1	CTD-2537I9.12	yellow	0.866794422
ENSG00000267475.1	CTD-2538C1.2	turquoise	0.824653595
ENSG00000267714.1	CTD-2540B15.6	grey	0.323445998

ENSG00000267130.1	CTD-2540B15.9	turquoise	0.61123585
ENSG00000260578.1	CTD-2541J13.1	turquoise	0.75345094
ENSG00000249898.3	CTD-2541M15.1	turquoise	0.844456643
ENSG00000271743.1	CTD-2541M15.3	turquoise	0.833703332
ENSG00000268518.1	CTD-2545M3.8	brown	0.711143179
ENSG00000245281.2	CTD-2547L16.1	turquoise	0.869015664
ENSG00000258875.1	CTD-2547L24.3	turquoise	0.760832006
ENSG00000260810.1	CTD-2547L24.4	grey	0.601205579
ENSG00000267640.1	CTD-2554C21.2	turquoise	0.849796304
ENSG00000229481.1	CTD-2554C21.3	turquoise	0.869227303
ENSG00000256982.1	CTD-2555A7.2	turquoise	0.797363766
ENSG00000258824.2	CTD-2555O16.2	blue	0.700782818
ENSG00000272828.1	CTD-2555O16.3	turquoise	0.747470513
ENSG00000272909.1	CTD-2555O16.4	brown	0.786556437
ENSG00000262098.1	CTD-2561B21.10	turquoise	0.774521227
ENSG00000262873.1	CTD-2561B21.11	turquoise	0.663097222
ENSG00000268119.1	CTD-2561J22.5	turquoise	0.881209335
ENSG00000255136.2	CTD-2562J17.4	turquoise	0.851372228
ENSG00000254429.1	CTD-2562J17.7	turquoise	0.7160601
ENSG00000180279.5	CTD-2568A17.5	brown	0.561440325
ENSG00000260114.1	CTD-2574D22.4	turquoise	0.854561161
ENSG00000268041.1	CTD-2575K13.6	yellow	0.700232911
ENSG00000270804.1	CTD-2583A14.11	yellow	0.829268937
ENSG00000269867.1	CTD-2583A14.8	yellow	0.911492017
ENSG00000268496.1	CTD-2587H19.2	green	0.67698765
ENSG00000269873.1	CTD-2587H19.3	grey	0.452904416
ENSG00000267577.1	CTD-2587H24.5	turquoise	0.791037457
ENSG00000249908.1	CTD-2589H19.4	blue	0.594047633
ENSG00000271781.1	CTD-2589H19.6	yellow	0.717620596
ENSG00000268593.1	CTD-2611O12.6	turquoise	0.748896926
ENSG00000269574.1	CTD-2611O12.7	grey	0.451631866
ENSG00000232098.2	CTD-2619J13.14	turquoise	0.903801624
ENSG00000268543.1	CTD-2619J13.16	turquoise	0.72196174
ENSG00000268912.1	CTD-2619J13.17	turquoise	0.910227811
ENSG00000269054.1	CTD-2619J13.3	pink	0.846868059
ENSG00000268049.1	CTD-2619J13.9	turquoise	0.912886112
ENSG00000268613.1	CTD-2620I22.7	grey	0.665435944
ENSG00000267370.1	CTD-2623N2.3	turquoise	0.771370054
ENSG00000267309.1	CTD-2630F21.1	turquoise	0.909608348
ENSG00000261604.1	CTD-2636A23.2	turquoise	0.772111922
ENSG00000267898.1	CTD-2639E6.9	turquoise	0.79308592
ENSG00000259366.1	CTD-2647L4.4	blue	0.783128007
ENSG00000259539.1	CTD-2651B20.1	turquoise	0.682850456
ENSG00000259520.1	CTD-2651B20.3	turquoise	0.495187992
ENSG00000234773.2	CTD-2666L21.1	turquoise	0.845109544
ENSG00000269834.1	CTD-3018O17.3	turquoise	0.740029937
ENSG00000254081.1	CTD-3025N20.2	brown	0.76807952
ENSG00000272010.1	CTD-3025N20.3	turquoise	0.675749223

ENSG00000266916.1	CTD-3064H18.1	brown	0.733928809
ENSG00000180458.2	CTD-3064H18.4	turquoise	0.831310303
ENSG00000271959.1	CTD-3064M3.7	grey	0.440969213
ENSG00000204791.4	CTD-3065J16.6	turquoise	0.713355732
ENSG00000255224.1	CTD-3065J16.9	turquoise	0.801962451
ENSG00000269990.1	CTD-3074O7.12	turquoise	0.937671613
ENSG00000255517.2	CTD-3074O7.5	brown	0.724405822
ENSG00000254744.3	CTD-3076O17.1	grey	0.141754173
ENSG00000270055.1	CTD-3092A11.2	yellow	0.818336143
ENSG00000268816.1	CTD-3093M3.1	turquoise	0.675710004
ENSG00000268970.1	CTD-3099C6.11	turquoise	0.741980196
ENSG00000269349.1	CTD-3099C6.5	turquoise	0.702535405
ENSG00000259715.1	CTD-3110H11.1	turquoise	0.695553519
ENSG00000267612.1	CTD-3116E22.7	grey	0.675509481
ENSG00000269439.1	CTD-3131K8.2	blue	0.752408295
ENSG00000268516.1	CTD-3138B18.5	turquoise	0.86916563
ENSG00000268201.1	CTD-3138B18.6	turquoise	0.745743611
ENSG00000265519.1	CTD-3157E16.1	green	0.733604009
ENSG00000267053.2	CTD-3162L10.1	yellow	0.843345389
ENSG00000232442.1	CTD-3184A7.4	yellow	0.788672924
ENSG00000261351.1	CTD-3185P2.1	turquoise	0.816865512
ENSG00000269999.1	CTD-3185P2.2	yellow	0.76418475
ENSG00000267765.1	CTD-3193K9.3	turquoise	0.71170788
ENSG00000269139.1	CTD-3193O13.8	turquoise	0.597408185
ENSG00000183248.7	CTD-3193O13.9	turquoise	0.83579909
ENSG00000262758.1	CTD-3195I5.1	brown	0.865413272
ENSG00000262692.1	CTD-3195I5.3	yellow	0.678835773
ENSG00000262194.1	CTD-3195I5.5	grey	0.715370288
ENSG00000267681.1	CTD-3199J23.6	turquoise	0.6052429
ENSG00000269399.1	CTD-3222D19.7	turquoise	0.75458613
ENSG00000266935.1	CTD-3234P18.2	turquoise	0.791213513
ENSG00000175826.7	CTDNEP1	turquoise	0.863351119
ENSG00000060069.12	CTDP1	turquoise	0.884732926
ENSG00000144579.3	CTDSP1	blue	0.695394678
ENSG00000175215.5	CTDSP2	blue	0.911555127
ENSG00000144677.10	CTDSPL	turquoise	0.836716622
ENSG00000137770.9	CTDSPL2	turquoise	0.933653384
ENSG00000150281.6	CTF1	turquoise	0.659059625
ENSG00000118523.5	CTGF	grey	0.319971846
ENSG00000179296.9	CTGLF12P	yellow	0.797344146
ENSG00000116761.7	CTH	blue	0.647048955
ENSG00000164932.8	CTHRC1	blue	0.487845495
ENSG00000134030.9	CTIF	turquoise	0.924446451
ENSG00000044115.16	CTNNA1	turquoise	0.837917165
ENSG00000066032.14	CTNNA2	blue	0.801013766
ENSG00000183230.12	CTNNA3	brown	0.541364092
ENSG00000119326.10	CTNNAL1	blue	0.793298059
ENSG00000168036.12	CTNNB1	turquoise	0.879571006

ENSG00000178585.10	CTNNBIP1	brown	0.73533583
ENSG00000132792.14	CTNNBL1	turquoise	0.922145501
ENSG00000198561.8	CTNND1	blue	0.946738489
ENSG00000169862.14	CTNND2	blue	0.793722933
ENSG00000040531.10	CTNS	blue	0.861091025
ENSG00000171793.9	CTPS1	turquoise	0.943708543
ENSG00000047230.10	CTPS2	turquoise	0.925157004
ENSG00000198730.3	CTR9	turquoise	0.947940418
ENSG00000168928.8	CTRB2	grey	0.074147591
ENSG00000141086.13	CTRL	turquoise	0.727512393
ENSG00000064601.12	CTSA	blue	0.880908062
ENSG00000164733.16	CTSB	turquoise	0.913436987
ENSG00000109861.11	CTSC	magenta	0.912246383
ENSG00000117984.8	CTSD	turquoise	0.79362602
ENSG00000174080.6	CTSF	turquoise	0.891013201
ENSG00000103811.11	CTSH	grey	0.528628415
ENSG00000143387.8	CTSK	blue	0.757585091
ENSG00000135047.10	CTSL	blue	0.870607211
ENSG00000256043.2	CTSO	blue	0.813542788
ENSG00000163131.6	CTSS	magenta	0.926229517
ENSG00000136943.6	CTSV	turquoise	0.675250793
ENSG00000101160.9	CTSZ	turquoise	0.773645441
ENSG00000085733.11	CTTN	turquoise	0.917160016
ENSG00000077063.6	CTTNBP2	turquoise	0.921014538
ENSG00000143079.10	CTTNBP2NL	blue	0.910442124
ENSG00000142544.6	CTU1	turquoise	0.73880794
ENSG00000174177.8	CTU2	turquoise	0.935443799
ENSG00000178531.4	CTXN1	turquoise	0.714142091
ENSG00000233932.3	CTXN2	turquoise	0.845266787
ENSG00000205279.4	CTXN3	greenyellow	0.733677284
ENSG00000107611.10	CUBN	pink	0.845389917
ENSG00000180891.8	CUEDC1	brown	0.873722483
ENSG00000107874.6	CUEDC2	turquoise	0.924603972
ENSG00000055130.11	CUL1	turquoise	0.986464766
ENSG00000108094.10	CUL2	turquoise	0.978570438
ENSG00000036257.8	CUL3	turquoise	0.948610973
ENSG00000139842.10	CUL4A	turquoise	0.902483649
ENSG00000158290.12	CUL4B	turquoise	0.956654553
ENSG00000166266.9	CUL5	turquoise	0.944474682
ENSG00000044090.4	CUL7	turquoise	0.948432777
ENSG00000112659.9	CUL9	turquoise	0.92995677
ENSG00000112514.11	CUTA	turquoise	0.937428746
ENSG00000119929.8	CUTC	turquoise	0.909725599
ENSG00000257923.5	CUX1	turquoise	0.946293824
ENSG00000111249.9	CUX2	greenyellow	0.809580942
ENSG00000138161.8	CUZD1	turquoise	0.497382612
ENSG00000150316.7	CWC15	turquoise	0.927430707
ENSG00000163510.9	CWC22	turquoise	0.903750306

ENSG00000108296.7	CWC25	turquoise	0.912698343
ENSG00000153015.11	CWC27	turquoise	0.933165867
ENSG00000095485.12	CWF19L1	turquoise	0.933731098
ENSG00000152404.11	CWF19L2	blue	0.733582724
ENSG00000006210.6	CX3CL1	turquoise	0.86140798
ENSG00000168329.9	CX3CR1	magenta	0.639236593
ENSG00000154639.14	CXADR	turquoise	0.896441769
ENSG00000163739.4	CXCL1	black	0.576960401
ENSG00000169245.4	CXCL10	black	0.497451963
ENSG00000107562.12	CXCL12	grey	0.413073053
ENSG00000145824.8	CXCL14	grey	0.211034837
ENSG00000161921.10	CXCL16	magenta	0.877523945
ENSG00000081041.8	CXCL2	black	0.553207639
ENSG00000163735.6	CXCL5	green	0.51378692
ENSG00000121966.6	CXCR4	grey	0.365405614
ENSG00000173681.12	CXorf23	turquoise	0.869522794
ENSG00000196741.3	CXorf24	turquoise	0.798395753
ENSG00000228459.2	CXorf28	grey	0.182756773
ENSG00000205081.4	CXorf30	pink	0.878700183
ENSG00000147113.12	CXorf36	black	0.75169004
ENSG00000185753.8	CXorf38	turquoise	0.79483111
ENSG00000197620.6	CXorf40A	turquoise	0.939491075
ENSG00000197021.4	CXorf40B	turquoise	0.920944698
ENSG00000018610.9	CXorf56	turquoise	0.947833258
ENSG00000147231.9	CXorf57	turquoise	0.822220396
ENSG00000154832.10	CXXC1	turquoise	0.89545756
ENSG00000188011.5	CXXC11	turquoise	0.812264244
ENSG00000168772.9	CXXC4	turquoise	0.865915923
ENSG00000171604.7	CXXC5	brown	0.839607154
ENSG00000008283.11	CYB561	turquoise	0.914119606
ENSG00000162144.5	CYB561A3	turquoise	0.851069725
ENSG00000174151.10	CYB561D1	turquoise	0.934560714
ENSG00000114395.6	CYB561D2	turquoise	0.811943894
ENSG00000166347.14	CYB5A	turquoise	0.704922276
ENSG00000103018.12	CYB5B	turquoise	0.855439506
ENSG00000182224.7	CYB5D1	pink	0.93736587
ENSG00000167740.5	CYB5D2	turquoise	0.919730214
ENSG00000159348.8	CYB5R1	blue	0.899093563
ENSG00000166394.10	CYB5R2	brown	0.835926914
ENSG00000100243.16	CYB5R3	blue	0.786552913
ENSG00000065615.9	CYB5R4	turquoise	0.931380377
ENSG00000051523.6	CYBA	magenta	0.825690711
ENSG00000165168.6	CYBB	magenta	0.924344082
ENSG00000071967.7	CYBRD1	green	0.898493554
ENSG00000179091.4	CYC1	turquoise	0.920444773
ENSG00000172115.4	CYCS	turquoise	0.902042273
ENSG00000176268.5	CYCSP34	turquoise	0.667452248
ENSG00000068793.13	CYFIP1	blue	0.897643356

ENSG00000055163.14	CYFIP2	turquoise	0.945615562
ENSG00000161544.5	CYGB	midnightblue	0.916947904
ENSG00000187954.8	CYHR1	turquoise	0.881636419
ENSG00000083799.13	CYLD	turquoise	0.961990853
ENSG00000140459.13	CYP11A1	green	0.687312349
ENSG00000148795.5	CYP17A1	turquoise	0.466800991
ENSG00000203886.4	CYP17A1-AS1	turquoise	0.504294569
ENSG00000138061.7	CYP1B1	black	0.6466622
ENSG00000119004.10	CYP20A1	blue	0.890593984
ENSG00000204338.4	CYP21A1P	turquoise	0.540580775
ENSG00000231852.2	CYP21A2	grey	0.413669905
ENSG00000003137.4	CYP26B1	turquoise	0.763815239
ENSG00000135929.4	CYP27A1	brown	0.837512722
ENSG00000256612.3	CYP2B7P1	turquoise	0.729034543
ENSG00000138115.9	CYP2C8	turquoise	0.775597633
ENSG00000100197.16	CYP2D6	yellow	0.776911756
ENSG00000205702.6	CYP2D7P1	yellow	0.888819856
ENSG00000226450.2	CYP2D8P1	yellow	0.836810451
ENSG00000130649.5	CYP2E1	turquoise	0.893146344
ENSG00000134716.5	CYP2J2	green	0.707766192
ENSG00000186104.6	CYP2R1	turquoise	0.888344666
ENSG00000233622.1	CYP2T2P	turquoise	0.705972665
ENSG00000155016.13	CYP2U1	blue	0.90609782
ENSG00000146233.3	CYP39A1	green	0.498447221
ENSG00000036530.4	CYP46A1	turquoise	0.842695691
ENSG00000171903.12	CYP4F11	green	0.835511232
ENSG00000267594.5	CYP4F24P	grey	0.227120355
ENSG00000228314.1	CYP4F29P	grey	0.463872313
ENSG00000265787.1	CYP4F35P	grey	0.659238139
ENSG00000145476.11	CYP4V2	green	0.850802934
ENSG00000186377.6	CYP4X1	turquoise	0.908500602
ENSG00000186160.4	CYP4Z1	turquoise	0.698473275
ENSG00000152207.3	CYSLTR2	grey	0.35949096
ENSG00000120306.5	CYSTM1	blue	0.741515527
ENSG00000108669.12	CYTH1	brown	0.838824931
ENSG00000105443.9	CYTH2	turquoise	0.954883422
ENSG00000008256.11	CYTH3	midnightblue	0.728178177
ENSG00000100055.16	CYTH4	magenta	0.91259985
ENSG00000170891.6	CYTL1	magenta	0.696636509
ENSG00000166265.7	CYYR1	black	0.553876814
ENSG00000180902.12	D2HGDH	turquoise	0.79380172
ENSG00000100592.11	DAAM1	blue	0.863305703
ENSG00000146122.12	DAAM2	brown	0.709419912
ENSG00000173406.11	DAB1	greenyellow	0.833373587
ENSG00000153071.10	DAB2	blue	0.746850446
ENSG00000136848.12	DAB2IP	turquoise	0.840265433
ENSG00000165659.12	DACH1	turquoise	0.8186762
ENSG00000126733.16	DACH2	turquoise	0.884724143

ENSG00000165617.10	DACT1	turquoise	0.795598518
ENSG00000164488.7	DACT2	turquoise	0.799547514
ENSG00000197380.6	DACT3	turquoise	0.879339799
ENSG00000129562.6	DAD1	blue	0.85872443
ENSG00000173402.7	DAG1	green	0.937273017
ENSG00000134780.5	DAGLA	turquoise	0.890524736
ENSG00000164535.10	DAGLB	turquoise	0.938986718
ENSG00000149476.10	DAK	turquoise	0.915773192
ENSG00000178149.12	DALRD3	pink	0.921974574
ENSG00000226950.2	DANCR	turquoise	0.871673796
ENSG00000112977.11	DAP	blue	0.866169485
ENSG00000132676.11	DAP3	turquoise	0.97065367
ENSG00000196730.8	DAPK1	turquoise	0.942158143
ENSG00000035664.7	DAPK2	brown	0.852772317
ENSG00000167657.7	DAPK3	turquoise	0.904383873
ENSG00000213088.5	DARC	turquoise	0.689463858
ENSG00000115866.6	DARS	blue	0.885664305
ENSG00000117593.8	DARS2	turquoise	0.860805981
ENSG00000123977.5	DAW1	turquoise	0.775691261
ENSG00000204209.6	DAXX	blue	0.872326471
ENSG00000071626.12	DAZAP1	turquoise	0.826300023
ENSG00000183283.11	DAZAP2	blue	0.921547988
ENSG00000006634.3	DBF4	turquoise	0.906922719
ENSG00000161692.13	DBF4B	turquoise	0.883947466
ENSG00000225756.1	DBH-AS1	yellow	0.609439604
ENSG00000155368.12	DBI	brown	0.646522365
ENSG00000231784.4	DBIL5P	turquoise	0.819230465
ENSG00000113758.9	DBN1	turquoise	0.924013082
ENSG00000003249.9	DBNDD1	turquoise	0.85491531
ENSG00000244274.3	DBNDD2	brown	0.916433769
ENSG00000136279.14	DBNL	turquoise	0.954223778
ENSG00000105516.6	DBP	turquoise	0.843040219
ENSG00000138231.8	DBR1	blue	0.93052353
ENSG00000137992.10	DBT	turquoise	0.922513643
ENSG00000185610.6	DBX2	grey	0.240474687
ENSG00000122741.11	DCAF10	turquoise	0.956508087
ENSG00000100897.13	DCAF11	turquoise	0.957707176
ENSG00000198876.8	DCAF12	blue	0.947296201
ENSG00000198354.4	DCAF12L2	turquoise	0.817312533
ENSG00000164934.9	DCAF13	turquoise	0.879708129
ENSG00000132017.6	DCAF15	turquoise	0.883004986
ENSG00000163257.6	DCAF16	turquoise	0.889918361
ENSG00000115827.9	DCAF17	turquoise	0.924623139
ENSG00000119599.12	DCAF4	turquoise	0.936036535
ENSG00000139990.13	DCAF5	turquoise	0.940816453
ENSG00000143164.11	DCAF6	turquoise	0.947072837
ENSG00000132716.14	DCAF8	turquoise	0.948753208
ENSG00000172992.7	DCAKD	blue	0.854692537

ENSG00000164465.14	DCBLD1	blue	0.626081569
ENSG00000057019.11	DCBLD2	turquoise	0.849410179
ENSG00000166341.6	DCHS1	turquoise	0.768228867
ENSG00000156136.5	DCK	turquoise	0.938933037
ENSG00000133083.10	DCLK1	turquoise	0.919250462
ENSG00000170390.10	DCLK2	turquoise	0.909895046
ENSG00000163673.6	DCLK3	turquoise	0.882057597
ENSG00000198924.3	DCLRE1A	turquoise	0.943374623
ENSG00000118655.4	DCLRE1B	turquoise	0.852074512
ENSG00000152457.13	DCLRE1C	turquoise	0.772330365
ENSG00000011465.12	DCN	brown	0.574702499
ENSG00000162290.12	DCP1A	turquoise	0.921589427
ENSG00000151065.9	DCP1B	turquoise	0.914926357
ENSG00000172795.11	DCP2	turquoise	0.942220939
ENSG00000110063.4	DCPS	turquoise	0.843274521
ENSG00000163354.10	DCST2	yellow	0.75839412
ENSG00000080166.11	DCT	turquoise	0.874463287
ENSG00000129187.10	DCTD	blue	0.884049019
ENSG00000204843.8	DCTN1	turquoise	0.950504982
ENSG00000237737.1	DCTN1-AS1	turquoise	0.900928572
ENSG00000175203.11	DCTN2	turquoise	0.965350863
ENSG00000137100.11	DCTN3	turquoise	0.958814922
ENSG00000132912.8	DCTN4	turquoise	0.942229978
ENSG00000166847.5	DCTN5	turquoise	0.91022466
ENSG00000104671.3	DCTN6	blue	0.840743924
ENSG00000179958.4	DCTPP1	turquoise	0.911492413
ENSG00000043093.9	DCUN1D1	turquoise	0.864141483
ENSG00000150401.10	DCUN1D2	turquoise	0.896895574
ENSG00000188215.5	DCUN1D3	cyan	0.896959706
ENSG00000109184.10	DCUN1D4	turquoise	0.95021716
ENSG00000137692.7	DCUN1D5	turquoise	0.884024003
ENSG00000077279.12	DCX	turquoise	0.888779073
ENSG00000169738.3	DCXR	red	0.813832016
ENSG00000130311.6	DDA1	turquoise	0.941290474
ENSG00000153904.14	DDAH1	green	0.906540274
ENSG00000213722.4	DDAH2	blue	0.705623802
ENSG00000167986.9	DDB1	turquoise	0.913404217
ENSG00000134574.7	DDB2	blue	0.774704301
ENSG00000132437.13	DDC	turquoise	0.828421134
ENSG00000100523.10	DDHD1	turquoise	0.87456312
ENSG00000085788.9	DDHD2	turquoise	0.920218172
ENSG00000197312.7	DDI2	turquoise	0.938386159
ENSG00000175197.6	DDIT3	cyan	0.868414419
ENSG00000168209.4	DDIT4	black	0.632025652
ENSG00000145358.2	DDIT4L	midnightblue	0.86315906
ENSG00000181418.7	DDN	turquoise	0.783910349
ENSG00000203797.5	DDO	turquoise	0.799941868
ENSG00000244038.5	DDOST	turquoise	0.946888192

ENSG00000204580.7	DDR1	blue	0.839199324
ENSG00000162733.12	DDR2	blue	0.682497668
ENSG00000198171.8	DDRGK1	turquoise	0.917026958
ENSG00000099977.9	DDT	red	0.898754388
ENSG00000099974.7	DDTL	turquoise	0.776712085
ENSG00000079785.10	DDX1	turquoise	0.964082044
ENSG00000178105.5	DDX10	turquoise	0.958415114
ENSG00000013573.12	DDX11	turquoise	0.53902823
ENSG00000236397.2	DDX11L2	grey	0.350317202
ENSG00000100201.14	DDX17	yellow	0.957136925
ENSG00000088205.8	DDX18	turquoise	0.9114235
ENSG00000168872.11	DDX19A	turquoise	0.96868447
ENSG00000157349.11	DDX19B	turquoise	0.922284232
ENSG00000064703.7	DDX20	turquoise	0.944945638
ENSG00000165732.8	DDX21	blue	0.867472822
ENSG00000174243.5	DDX23	turquoise	0.87829267
ENSG00000089737.11	DDX24	turquoise	0.978428271
ENSG00000109832.8	DDX25	turquoise	0.950900285
ENSG00000165359.10	DDX26B	yellow	0.898523911
ENSG00000124228.10	DDX27	turquoise	0.939441078
ENSG00000182810.5	DDX28	turquoise	0.937352756
ENSG00000125485.13	DDX31	blue	0.863372899
ENSG00000123136.10	DDX39A	blue	0.827072811
ENSG00000198563.9	DDX39B	turquoise	0.94110867
ENSG00000215301.5	DDX3X	turquoise	0.858790994
ENSG00000067048.12	DDX3Y	grey	0.218899167
ENSG00000183258.7	DDX41	turquoise	0.957148596
ENSG00000198231.8	DDX42	turquoise	0.956797384
ENSG00000145833.11	DDX46	turquoise	0.939303851
ENSG00000105671.7	DDX49	turquoise	0.916706037
ENSG00000108654.7	DDX5	turquoise	0.925378826
ENSG00000107625.8	DDX50	turquoise	0.956184567
ENSG00000185163.5	DDX51	turquoise	0.945954514
ENSG00000141141.10	DDX52	turquoise	0.884056549
ENSG00000123064.8	DDX54	turquoise	0.932928996
ENSG00000111364.11	DDX55	turquoise	0.941237628
ENSG00000136271.6	DDX56	turquoise	0.930292767
ENSG00000107201.5	DDX58	blue	0.803607426
ENSG00000118197.9	DDX59	turquoise	0.831662816
ENSG00000137628.12	DDX60	blue	0.724508451
ENSG00000181381.9	DDX60L	black	0.708237445
ENSG00000177030.12	DEAF1	turquoise	0.867725832
ENSG00000104325.2	DECR1	blue	0.792724083
ENSG00000242612.2	DECR2	turquoise	0.874026988
ENSG00000158796.12	DEDD	turquoise	0.927902403
ENSG00000160570.9	DEDD2	turquoise	0.819974028
ENSG00000023892.9	DEF6	yellow	0.775214722
ENSG00000140995.12	DEF8	turquoise	0.962152588

ENSG00000239839.1	DEFA3	grey	0.038691068
ENSG00000143753.8	DEGS1	turquoise	0.937906693
ENSG00000168350.6	DEGS2	turquoise	0.768800636
ENSG00000124795.10	DEK	blue	0.909586466
ENSG00000119522.11	DENND1A	turquoise	0.961039487
ENSG00000213047.7	DENND1B	turquoise	0.894080113
ENSG00000205744.5	DENND1C	turquoise	0.82899178
ENSG00000146966.8	DENND2A	brown	0.710661097
ENSG00000175984.10	DENND2C	turquoise	0.81103
ENSG00000105339.6	DENND3	blue	0.697940451
ENSG00000174485.10	DENND4A	turquoise	0.906263416
ENSG00000198837.5	DENND4B	turquoise	0.949200355
ENSG00000137145.16	DENND4C	turquoise	0.887634145
ENSG00000184014.3	DENND5A	brown	0.910464937
ENSG00000170456.10	DENND5B	turquoise	0.954821006
ENSG00000174839.8	DENND6A	turquoise	0.886919668
ENSG00000205593.7	DENND6B	turquoise	0.891580483
ENSG00000139726.6	DENR	turquoise	0.968420989
ENSG00000100150.12	DEPDC5	turquoise	0.948637866
ENSG00000121690.5	DEPDC7	brown	0.817756213
ENSG00000155792.5	DEPTOR	brown	0.88063967
ENSG00000023697.8	DERA	blue	0.850146134
ENSG00000136986.5	DERL1	turquoise	0.959764347
ENSG00000072849.6	DERL2	turquoise	0.850752477
ENSG00000175084.7	DES	grey	0.200319723
ENSG00000100418.7	DESI1	turquoise	0.953977508
ENSG00000121644.14	DESI2	turquoise	0.949668271
ENSG00000140543.9	DET1	turquoise	0.938035605
ENSG00000182108.5	DEXI	turquoise	0.593063063
ENSG00000160049.7	DFFA	blue	0.935848727
ENSG00000169598.11	DFFB	yellow	0.908964324
ENSG00000105928.9	DFNA5	turquoise	0.877817306
ENSG00000095397.9	DFNB31	blue	0.839874394
ENSG00000204311.7	DFNB59	yellow	0.821614899
ENSG00000185000.5	DGAT1	turquoise	0.909042679
ENSG00000062282.10	DGAT2	turquoise	0.881922933
ENSG00000273164.1	DGCR10	grey	0.509060226
ENSG00000100056.7	DGCR14	turquoise	0.922452052
ENSG00000070413.14	DGCR2	turquoise	0.937469157
ENSG00000237517.4	DGCR5	turquoise	0.882666099
ENSG00000183628.8	DGCR6	turquoise	0.826581004
ENSG00000128185.5	DGCR6L	red	0.923752329
ENSG00000128191.9	DGCR8	turquoise	0.90603525
ENSG00000273032.1	DGCR9	turquoise	0.882133509
ENSG00000065357.15	DGKA	turquoise	0.802170306
ENSG00000136267.9	DGKB	turquoise	0.863321036
ENSG00000077044.5	DGKD	turquoise	0.925317059
ENSG00000153933.5	DGKE	turquoise	0.935635334

ENSG00000058866.10	DGKG	turquoise	0.768714037
ENSG00000102780.12	DGKH	turquoise	0.894583317
ENSG00000157680.11	DGKI	turquoise	0.904401265
ENSG00000145214.9	DGKQ	turquoise	0.909099838
ENSG00000149091.11	DGKZ	turquoise	0.881780351
ENSG00000114956.15	DGUOK	turquoise	0.94193566
ENSG00000237883.1	DGUOK-AS1	yellow	0.835235945
ENSG00000116133.7	DHCR24	turquoise	0.935026832
ENSG00000172893.11	DHCR7	blue	0.811871228
ENSG00000117682.12	DHDDS	turquoise	0.9664598
ENSG00000104808.3	DHDH	turquoise	0.796855104
ENSG00000228716.2	DHFR	brown	0.748807503
ENSG00000178700.3	DHFRL1	turquoise	0.844976876
ENSG00000102967.7	DHODH	turquoise	0.871109817
ENSG00000095059.11	DHPS	turquoise	0.970609947
ENSG00000157379.9	DHRS1	turquoise	0.80933297
ENSG00000108272.9	DHRS11	turquoise	0.87302712
ENSG00000102796.6	DHRS12	turquoise	0.774182424
ENSG00000167536.9	DHRS13	turquoise	0.815314022
ENSG00000162496.4	DHRS3	green	0.617563195
ENSG00000157326.14	DHRS4	turquoise	0.80923852
ENSG00000215256.3	DHRS4-AS1	blue	0.837167823
ENSG00000187630.10	DHRS4L2	turquoise	0.708987203
ENSG00000100612.9	DHRS7	turquoise	0.817064702
ENSG00000109016.13	DHRS7B	turquoise	0.896924942
ENSG00000169084.8	DHRSX	blue	0.819002625
ENSG00000181192.7	DHTKD1	green	0.911745352
ENSG00000109606.8	DHX15	turquoise	0.92536759
ENSG00000204560.5	DHX16	turquoise	0.910350265
ENSG00000067248.5	DHX29	turquoise	0.947460009
ENSG00000132153.10	DHX30	turquoise	0.979137855
ENSG00000089876.7	DHX32	turquoise	0.901248309
ENSG00000005100.8	DHX33	turquoise	0.966641345
ENSG00000134815.14	DHX34	yellow	0.863644579
ENSG00000101452.10	DHX35	turquoise	0.944367111
ENSG00000174953.9	DHX36	turquoise	0.96397148
ENSG00000150990.3	DHX37	turquoise	0.870325841
ENSG00000140829.7	DHX38	turquoise	0.883257698
ENSG00000108406.5	DHX40	turquoise	0.895823808
ENSG00000163214.16	DHX57	turquoise	0.95468899
ENSG00000108771.8	DHX58	blue	0.797315789
ENSG00000067596.6	DHX8	turquoise	0.93592237
ENSG00000135829.12	DHX9	turquoise	0.964670836
ENSG00000184047.11	DIABLO	turquoise	0.901640229
ENSG00000131504.11	DIAPH1	turquoise	0.943798491
ENSG00000147202.13	DIAPH2	turquoise	0.919245444
ENSG00000139734.13	DIAPH3	green	0.59489668
ENSG00000100697.10	DICER1	turquoise	0.764623596

ENSG00000235706.3	DICER1-AS1	yellow	0.890618233
ENSG00000101191.12	DIDO1	turquoise	0.951562303
ENSG00000117597.13	DIEXF	turquoise	0.944322441
ENSG00000086189.5	DIMT1	turquoise	0.915339983
ENSG00000211448.7	DIO2	grey	-0.005862054
ENSG00000197406.6	DIO3	grey	0.279711565
ENSG00000258498.2	DIO3OS	grey	0.400561439
ENSG00000160305.13	DIP2A	turquoise	0.893793738
ENSG00000066084.8	DIP2B	brown	0.944923398
ENSG00000151240.11	DIP2C	turquoise	0.963819573
ENSG00000176490.4	DIRAS1	turquoise	0.900741395
ENSG00000165023.5	DIRAS2	turquoise	0.889491026
ENSG00000138463.8	DIRC2	green	0.904329485
ENSG00000083520.10	DIS3	turquoise	0.949614802
ENSG00000166938.8	DIS3L	turquoise	0.914249984
ENSG00000144535.15	DIS3L2	turquoise	0.923198098
ENSG00000162946.16	DISC1	brown	0.8321515
ENSG00000154309.7	DISP1	green	0.746300345
ENSG00000140323.4	DISP2	turquoise	0.882232057
ENSG00000150764.9	DIXDC1	brown	0.813814854
ENSG00000130826.11	DKC1	turquoise	0.946781834
ENSG00000105520.6	DKFZP761J1410	turquoise	0.903165399
ENSG00000050165.13	DKK3	turquoise	0.938146364
ENSG00000150768.11	DLAT	turquoise	0.945802439
ENSG00000164741.10	DLC1	turquoise	0.844633522
ENSG00000091140.8	DLD	turquoise	0.927347238
ENSG00000231607.4	DLEU2	brown	0.731666494
ENSG00000186047.9	DLEU7	turquoise	0.888018008
ENSG00000075711.16	DLG1	brown	0.812890229
ENSG00000150672.12	DLG2	turquoise	0.940220743
ENSG00000082458.7	DLG3	turquoise	0.937714572
ENSG00000132535.14	DLG4	turquoise	0.902872735
ENSG00000151208.12	DLG5	blue	0.872440541
ENSG00000170579.10	DLGAP1	turquoise	0.915210809
ENSG00000198010.7	DLGAP2	turquoise	0.907973874
ENSG00000116544.7	DLGAP3	turquoise	0.816834799
ENSG00000080845.13	DLGAP4	turquoise	0.919094057
ENSG00000171462.10	DLK2	turquoise	0.868508952
ENSG00000198719.7	DLL1	yellow	0.736814139
ENSG00000090932.6	DLL3	turquoise	0.757592451
ENSG00000128917.5	DLL4	blue	0.512530818
ENSG00000119689.10	DLST	turquoise	0.917939893
ENSG00000144355.10	DLX1	greenyellow	0.813162867
ENSG00000115844.6	DLX2	greenyellow	0.695816209
ENSG00000105880.4	DLX5	turquoise	0.850668168
ENSG00000006377.9	DLX6	turquoise	0.885490012
ENSG00000231764.4	DLX6-AS1	turquoise	0.819714392
ENSG00000178028.9	DMAP1	turquoise	0.952197917

ENSG00000198947.10	DMD	blue	0.798162371
ENSG00000132837.10	DMGDH	blue	0.705066037
ENSG00000161249.16	DMKN	turquoise	0.814080768
ENSG00000104936.13	DMPK	yellow	0.935500054
ENSG00000173253.10	DMRT2	brown	0.830490672
ENSG00000135164.14	DMTF1	turquoise	0.90286848
ENSG00000158856.13	DMTN	turquoise	0.902207207
ENSG00000185800.7	DMWD	turquoise	0.94538934
ENSG00000172869.10	DMXL1	turquoise	0.905451544
ENSG00000104093.9	DMXL2	turquoise	0.956124224
ENSG00000138346.10	DNA2	blue	0.755465487
ENSG00000165506.10	DNAAF2	turquoise	0.909326997
ENSG00000114841.13	DNAH1	pink	0.910832313
ENSG00000250091.2	DNAH10OS	turquoise	0.918912107
ENSG00000187775.12	DNAH17	brown	0.859979254
ENSG00000183914.10	DNAH2	pink	0.921237397
ENSG00000115423.14	DNAH6	pink	0.914053406
ENSG00000007174.13	DNAH9	pink	0.907927632
ENSG00000122735.11	DNAI1	pink	0.920948861
ENSG00000086061.11	DNAJA1	cyan	0.950279692
ENSG00000069345.7	DNAJA2	turquoise	0.980385577
ENSG00000103423.9	DNAJA3	turquoise	0.972277465
ENSG00000140403.8	DNAJA4	turquoise	0.824478467
ENSG00000132002.3	DNAJB1	black	0.571284507
ENSG00000090520.6	DNAJB11	cyan	0.921754887
ENSG00000148719.10	DNAJB12	turquoise	0.964473345
ENSG00000164031.12	DNAJB14	turquoise	0.946072193
ENSG00000135924.11	DNAJB2	blue	0.809195282
ENSG00000162616.8	DNAJB4	cyan	0.915948512
ENSG00000137094.10	DNAJB5	turquoise	0.715475557
ENSG00000105993.10	DNAJB6	cyan	0.949684603
ENSG00000128590.4	DNAJB9	turquoise	0.876262096
ENSG00000136770.6	DNAJC1	blue	0.828119632
ENSG00000077232.12	DNAJC10	turquoise	0.916236983
ENSG00000007923.11	DNAJC11	turquoise	0.969972394
ENSG00000108176.10	DNAJC12	turquoise	0.932877947
ENSG00000138246.11	DNAJC13	turquoise	0.924850485
ENSG00000135392.11	DNAJC14	turquoise	0.891580555
ENSG00000120675.4	DNAJC15	blue	0.751200308
ENSG00000116138.8	DNAJC16	turquoise	0.970834348
ENSG00000104129.5	DNAJC17	turquoise	0.910571621
ENSG00000170464.5	DNAJC18	turquoise	0.971516957
ENSG00000205981.2	DNAJC19	turquoise	0.947091829
ENSG00000105821.10	DNAJC2	turquoise	0.946297742
ENSG00000168724.10	DNAJC21	turquoise	0.896426431
ENSG00000170946.10	DNAJC24	turquoise	0.92241537
ENSG00000059769.15	DNAJC25	turquoise	0.914977597
ENSG00000115137.7	DNAJC27	turquoise	0.936601286

ENSG00000224165.1	DNAJC27-AS1	turquoise	0.767710091
ENSG00000177692.7	DNAJC28	turquoise	0.888239318
ENSG00000102580.10	DNAJC3	turquoise	0.789546252
ENSG00000247400.3	DNAJC3-AS1	turquoise	0.845946596
ENSG00000176410.7	DNAJC30	turquoise	0.922033024
ENSG00000110011.9	DNAJC4	red	0.905957367
ENSG00000101152.6	DNAJC5	turquoise	0.964493744
ENSG00000116675.11	DNAJC6	turquoise	0.930622072
ENSG00000168259.10	DNAJC7	turquoise	0.964388712
ENSG00000126698.6	DNAJC8	turquoise	0.94564662
ENSG00000213551.4	DNAJC9	turquoise	0.950284137
ENSG00000119661.10	DNAL1	pink	0.914355192
ENSG00000100246.8	DNAL4	turquoise	0.935128121
ENSG00000213918.6	DNASE1	yellow	0.886445289
ENSG00000013563.9	DNASE1L1	turquoise	0.92352013
ENSG00000167968.8	DNASE1L2	grey	0.809322185
ENSG00000105612.4	DNASE2	blue	0.803064275
ENSG00000264070.1	DND1P1	grey	0.416219989
ENSG00000187957.7	DNER	brown	0.864943359
ENSG00000179532.8	DNHD1	yellow	0.86440601
ENSG00000213221.4	DNLZ	red	0.807110787
ENSG00000106976.14	DNM1	turquoise	0.914594784
ENSG00000087470.13	DNM1L	turquoise	0.976797971
ENSG00000079805.12	DNM2	brown	0.873090165
ENSG00000197959.9	DNM3	turquoise	0.916746269
ENSG00000107554.11	DNMBP	blue	0.844317518
ENSG00000130816.10	DNMT1	turquoise	0.91193717
ENSG00000119772.12	DNMT3A	turquoise	0.912884956
ENSG00000123992.14	DNPEP	turquoise	0.920952011
ENSG00000112667.8	DNPH1	turquoise	0.840294298
ENSG00000101457.8	DNTTIP1	turquoise	0.912744708
ENSG00000067334.9	DNTTIP2	turquoise	0.854633041
ENSG00000149927.13	DOC2A	pink	0.919299173
ENSG00000272636.1	DOC2B	turquoise	0.88070289
ENSG00000231793.4	DOC2GP	midnightblue	0.762265271
ENSG00000150760.8	DOCK1	brown	0.852219944
ENSG00000135905.14	DOCK10	brown	0.882341789
ENSG00000147251.11	DOCK11	turquoise	0.901093827
ENSG00000134516.11	DOCK2	magenta	0.95337873
ENSG00000088538.12	DOCK3	turquoise	0.969843609
ENSG00000128512.15	DOCK4	turquoise	0.926401973
ENSG00000147459.13	DOCK5	brown	0.892011389
ENSG00000130158.9	DOCK6	black	0.790805136
ENSG00000116641.11	DOCK7	blue	0.880395039
ENSG00000107099.11	DOCK8	magenta	0.877358219
ENSG00000088387.13	DOCK9	blue	0.814796458
ENSG00000260992.1	DOCK9-AS2	turquoise	0.776967487
ENSG00000129932.3	DOHH	red	0.792711051

ENSG00000115325.9	DOK1	magenta	0.786165098
ENSG00000146094.9	DOK3	turquoise	0.694420329
ENSG00000125170.6	DOK4	turquoise	0.901513907
ENSG00000101134.7	DOK5	turquoise	0.633546701
ENSG00000206052.6	DOK6	turquoise	0.884188371
ENSG00000175920.11	DOK7	turquoise	0.700577922
ENSG00000175283.7	DOLK	blue	0.838456503
ENSG00000167130.13	DOLPP1	turquoise	0.860783272
ENSG00000159147.13	DONSON	yellow	0.904952185
ENSG00000083097.10	DOPEY1	turquoise	0.929449356
ENSG00000142197.8	DOPEY2	turquoise	0.946957622
ENSG00000104885.13	DOT1L	turquoise	0.816282411
ENSG00000172269.12	DPAGT1	turquoise	0.931271507
ENSG00000166171.8	DPCD	turquoise	0.911149747
ENSG00000011332.15	DPF1	turquoise	0.887814771
ENSG00000133884.5	DPF2	turquoise	0.929612271
ENSG00000205683.7	DPF3	brown	0.777417483
ENSG00000108963.13	DPH1	turquoise	0.940580034
ENSG00000132768.9	DPH2	turquoise	0.961890934
ENSG00000154813.5	DPH3	blue	0.87025921
ENSG00000117543.15	DPH5	turquoise	0.941574749
ENSG00000134146.7	DPH6	blue	0.834982631
ENSG00000248079.2	DPH6-AS1	turquoise	0.804191871
ENSG00000148399.7	DPH7	yellow	0.954997737
ENSG00000000419.8	DPM1	turquoise	0.934857849
ENSG00000136908.13	DPM2	turquoise	0.872028997
ENSG00000179085.7	DPM3	red	0.927981947
ENSG00000175497.12	DPP10	turquoise	0.697092373
ENSG00000254986.3	DPP3	blue	0.882935467
ENSG00000130226.12	DPP6	turquoise	0.929312227
ENSG00000176978.9	DPP7	turquoise	0.871678628
ENSG00000074603.14	DPP8	turquoise	0.926045817
ENSG00000142002.12	DPP9	blue	0.896282156
ENSG00000173852.9	DPY19L1	blue	0.823786179
ENSG00000229358.3	DPY19L1P1	yellow	0.901043824
ENSG00000177990.7	DPY19L2	turquoise	0.866803867
ENSG00000189212.8	DPY19L2P1	turquoise	0.877310553
ENSG00000170629.10	DPY19L2P2	pink	0.874332248
ENSG00000235436.6	DPY19L2P4	turquoise	0.834603875
ENSG00000178904.14	DPY19L3	blue	0.86725075
ENSG00000156162.12	DPY19L4	blue	0.848043911
ENSG00000162961.9	DPY30	turquoise	0.894032274
ENSG00000188641.8	DPYD	blue	0.777626617
ENSG00000092964.12	DPYSL2	turquoise	0.808877516
ENSG00000113657.8	DPYSL3	green	0.849469642
ENSG00000151640.8	DPYSL4	turquoise	0.920238921
ENSG00000157851.12	DPYSL5	turquoise	0.90099191
ENSG00000117505.8	DR1	turquoise	0.936096878

ENSG00000136048.9	DRAM1	blue	0.722684823
ENSG00000156171.10	DRAM2	blue	0.896602477
ENSG00000175550.3	DRAP1	red	0.955133669
ENSG00000162490.6	DRAXIN	turquoise	0.736013477
ENSG00000184845.3	DRD1	turquoise	0.844538793
ENSG00000149295.9	DRD2	turquoise	0.771230363
ENSG00000151577.8	DRD3	turquoise	0.739226304
ENSG00000069696.6	DRD4	turquoise	0.520009513
ENSG00000169676.4	DRD5	midnightblue	0.855670765
ENSG00000185721.7	DRG1	turquoise	0.950084307
ENSG00000108591.5	DRG2	turquoise	0.93824057
ENSG00000113360.12	DROSHA	turquoise	0.984060234
ENSG00000102385.8	DRP2	turquoise	0.906989627
ENSG00000134755.10	DSC2	blue	0.756605146
ENSG00000171587.10	DSCAM	greenyellow	0.842038864
ENSG00000177103.9	DSCAML1	turquoise	0.884690422
ENSG00000136982.5	DSCC1	blue	0.635504126
ENSG00000157538.9	DSCR3	turquoise	0.918420031
ENSG00000111817.12	DSE	black	0.801707239
ENSG00000171451.13	DSEL	turquoise	0.876506951
ENSG00000149636.11	DSN1	turquoise	0.933640596
ENSG00000096696.9	DSP	turquoise	0.58380753
ENSG00000151914.13	DST	turquoise	0.830692702
ENSG00000125868.11	DSTN	turquoise	0.950573611
ENSG00000230982.1	DSTNP1	turquoise	0.678134422
ENSG00000248593.3	DSTNP2	blue	0.74703972
ENSG00000133059.12	DSTYK	turquoise	0.969584744
ENSG00000125821.7	DTD1	turquoise	0.939160481
ENSG00000129480.8	DTD2	blue	0.832182526
ENSG00000134769.17	DTNA	blue	0.767689693
ENSG00000138101.14	DTNB	turquoise	0.932607593
ENSG00000047579.15	DTNBP1	turquoise	0.888519582
ENSG00000104047.10	DTWD1	turquoise	0.946248073
ENSG00000169570.5	DTWD2	turquoise	0.879765869
ENSG00000135144.3	DTX1	turquoise	0.866782638
ENSG00000091073.15	DTX2	blue	0.785559409
ENSG00000186704.8	DTX2P1	yellow	0.812472751
ENSG00000178498.11	DTX3	turquoise	0.953107974
ENSG00000163840.5	DTX3L	black	0.864678952
ENSG00000110042.3	DTX4	turquoise	0.754879935
ENSG00000168393.8	DTYMK	blue	0.716054972
ENSG00000137857.13	DUOX1	yellow	0.796673785
ENSG00000169718.13	DUS1L	turquoise	0.898243957
ENSG00000167264.13	DUS2	turquoise	0.903970831
ENSG00000141994.11	DUS3L	turquoise	0.896060401
ENSG00000105865.6	DUS4L	turquoise	0.888272398
ENSG00000120129.5	DUSP1	grey	0.352666753
ENSG00000143507.13	DUSP10	brown	0.81027628

ENSG00000144048.6	DUSP11	turquoise	0.950684954
ENSG00000081721.7	DUSP12	turquoise	0.920964762
ENSG00000161326.8	DUSP14	turquoise	0.896315807
ENSG00000149599.11	DUSP15	red	0.803493929
ENSG00000111266.4	DUSP16	blue	0.830599239
ENSG00000167065.9	DUSP18	pink	0.895284517
ENSG00000162999.8	DUSP19	turquoise	0.889970408
ENSG00000158050.4	DUSP2	turquoise	0.55527891
ENSG00000112679.10	DUSP22	turquoise	0.906962596
ENSG00000158716.4	DUSP23	turquoise	0.784892516
ENSG00000133878.4	DUSP26	turquoise	0.909649443
ENSG00000188542.5	DUSP28	turquoise	0.957193398
ENSG00000108861.4	DUSP3	turquoise	0.950542448
ENSG00000120875.4	DUSP4	turquoise	0.664606356
ENSG00000138166.5	DUSP5	black	0.457577543
ENSG00000139318.7	DUSP6	turquoise	0.570441208
ENSG00000164086.8	DUSP7	brown	0.865975216
ENSG00000184545.6	DUSP8	turquoise	0.862150718
ENSG00000235316.1	DUSP8P5	yellow	0.865609692
ENSG00000130829.13	DUSP9	turquoise	0.768658003
ENSG00000128951.9	DUT	turquoise	0.940449711
ENSG00000225171.2	DUTP6	grey	0.367430852
ENSG00000107404.13	DVL1	turquoise	0.908246991
ENSG00000004975.7	DVL2	turquoise	0.900980564
ENSG00000161202.13	DVL3	turquoise	0.945986439
ENSG00000204348.5	DXO	turquoise	0.927778491
ENSG00000133665.8	DYDC2	pink	0.851870035
ENSG00000141627.9	DYM	turquoise	0.941818402
ENSG00000197102.6	DYNC1H1	turquoise	0.957996778
ENSG00000158560.10	DYNC1I1	turquoise	0.942498505
ENSG00000077380.11	DYNC1I2	turquoise	0.874836295
ENSG00000144635.4	DYNC1LI1	turquoise	0.968083527
ENSG00000135720.8	DYNC1LI2	brown	0.741738714
ENSG00000187240.9	DYNC2H1	pink	0.939350289
ENSG00000138036.14	DYNC2LI1	turquoise	0.934440263
ENSG00000088986.6	DYNLL1	turquoise	0.900316158
ENSG00000248008.2	DYNLL1-AS1	pink	0.93819608
ENSG00000264364.2	DYNLL2	turquoise	0.922303611
ENSG00000125971.12	DYNLRB1	turquoise	0.932491277
ENSG00000146425.6	DYNLT1	blue	0.852516471
ENSG00000165169.6	DYNLT3	turquoise	0.944324353
ENSG00000157540.15	DYRK1A	turquoise	0.981138813
ENSG00000105204.9	DYRK1B	turquoise	0.894551156
ENSG00000127334.10	DYRK2	turquoise	0.804055704
ENSG00000010219.9	DYRK4	turquoise	0.870809753
ENSG00000135636.9	DYSF	brown	0.835755805
ENSG00000256061.3	DYX1C1	pink	0.887976688
ENSG00000089091.12	DZANK1	pink	0.954059743

ENSG00000134874.13	DZIP1	turquoise	0.910273944
ENSG00000198919.8	DZIP3	turquoise	0.951744064
ENSG00000101412.9	E2F1	turquoise	0.749906365
ENSG00000112242.10	E2F3	turquoise	0.931167516
ENSG00000205250.4	E2F4	turquoise	0.948643429
ENSG00000133740.6	E2F5	pink	0.869475273
ENSG00000169016.12	E2F6	turquoise	0.905294578
ENSG00000167967.11	E4F1	turquoise	0.924093857
ENSG00000144597.9	EAF1	turquoise	0.964441745
ENSG00000145088.4	EAF2	blue	0.703988779
ENSG00000129518.4	EAPP	turquoise	0.947664094
ENSG00000103356.11	EARS2	turquoise	0.944530437
ENSG00000147654.10	EBAG9	turquoise	0.959526573
ENSG00000164330.12	EBF1	turquoise	0.525824322
ENSG00000221818.4	EBF2	turquoise	0.792287503
ENSG00000088881.16	EBF4	yellow	0.753242168
ENSG00000105246.5	EBI3	magenta	0.902925833
ENSG00000117395.6	EBNA1BP2	turquoise	0.96028029
ENSG00000147155.6	EBP	turquoise	0.929129731
ENSG00000123179.9	EBPL	red	0.742819478
ENSG00000122882.6	ECD	turquoise	0.974930984
ENSG00000117298.10	ECE1	blue	0.763304718
ENSG00000145194.13	ECE2	turquoise	0.871316819
ENSG00000171551.7	ECEL1	midnightblue	0.758226278
ENSG00000104823.4	ECH1	blue	0.820788352
ENSG00000093144.14	ECHDC1	turquoise	0.927577389
ENSG00000121310.12	ECHDC2	green	0.705252645
ENSG00000134463.10	ECHDC3	grey	0.365760652
ENSG00000127884.4	ECHS1	red	0.897597506
ENSG00000167969.8	ECI1	green	0.659206207
ENSG00000198721.8	ECI2	blue	0.825524536
ENSG00000143369.10	ECM1	yellow	0.768197515
ENSG00000249751.1	ECSCR	black	0.738725703
ENSG00000130159.9	ECSIT	turquoise	0.917190621
ENSG00000114346.9	ECT2	turquoise	0.861458771
ENSG00000158813.13	EDA	brown	0.793184101
ENSG00000179151.7	EDC3	turquoise	0.933683983
ENSG00000038358.10	EDC4	turquoise	0.964304442
ENSG00000134109.6	EDEM1	turquoise	0.947886227
ENSG00000088298.8	EDEM2	turquoise	0.861591685
ENSG00000116406.14	EDEM3	turquoise	0.882761731
ENSG00000107223.8	EDF1	red	0.968900448
ENSG00000164176.8	EDIL3	brown	0.933004967
ENSG00000078401.6	EDN1	black	0.590511491
ENSG00000124205.11	EDN3	brown	0.476069427
ENSG00000151617.11	EDNRA	brown	0.494267976
ENSG00000136160.10	EDNRB	green	0.791616353
ENSG00000102189.12	EEA1	turquoise	0.838003859

ENSG00000074266.13	EED	turquoise	0.8964709
ENSG00000156508.13	EEF1A1	red	0.868646276
ENSG00000228502.1	EEF1A1P11	grey	0.181411985
ENSG00000101210.6	EEF1A2	turquoise	0.893820675
ENSG00000114942.9	EEF1B2	red	0.915553437
ENSG00000104529.13	EEF1D	red	0.851305966
ENSG00000124802.7	EEF1E1	turquoise	0.939762198
ENSG00000254772.5	EEF1G	red	0.920408539
ENSG00000167658.11	EEF2	turquoise	0.93207554
ENSG00000103319.7	EEF2K	turquoise	0.836209032
ENSG00000132394.6	EEFSEC	turquoise	0.916412494
ENSG00000122547.6	EEPD1	brown	0.798159845
ENSG00000185055.6	EFCAB10	pink	0.91637948
ENSG00000140025.11	EFCAB11	blue	0.802434569
ENSG00000172771.7	EFCAB12	pink	0.875318148
ENSG00000159658.6	EFCAB14	blue	0.918067871
ENSG00000177685.12	EFCAB4A	grey	0.65244383
ENSG00000186976.10	EFCAB6	pink	0.93421937
ENSG00000203965.8	EFCAB7	turquoise	0.926456114
ENSG00000114654.6	EFCC1	pink	0.606354306
ENSG00000115380.14	EFEMP1	green	0.853683878
ENSG00000172638.8	EFEMP2	green	0.754342052
ENSG00000096093.10	EFHC1	pink	0.964138878
ENSG00000115468.7	EFHD1	brown	0.847643796
ENSG00000142634.8	EFHD2	red	0.713142623
ENSG00000169242.7	EFNA1	blue	0.719599143
ENSG00000099617.2	EFNA2	turquoise	0.76027815
ENSG00000143590.9	EFNA3	turquoise	0.871172435
ENSG00000243364.3	EFNA4	green	0.603289328
ENSG00000184349.8	EFNA5	turquoise	0.909820244
ENSG00000090776.5	EFNB1	blue	0.62874357
ENSG00000125266.6	EFNB2	turquoise	0.905378331
ENSG00000108947.4	EFNB3	turquoise	0.902026436
ENSG00000132294.9	EFR3A	turquoise	0.856955227
ENSG00000084710.9	EFR3B	turquoise	0.863752313
ENSG00000100842.8	EFS	brown	0.89798163
ENSG00000140598.9	EFTUD1	turquoise	0.864576977
ENSG00000108883.8	EFTUD2	turquoise	0.955206288
ENSG00000172889.11	EGFL7	blue	0.568172604
ENSG00000241404.2	EGFL8	yellow	0.795108591
ENSG00000164318.13	EGFLAM	black	0.565655871
ENSG00000146648.11	EGFR	green	0.889896689
ENSG00000135766.8	EGLN1	turquoise	0.925573644
ENSG00000269858.1	EGLN2	turquoise	0.930896647
ENSG00000129521.9	EGLN3	blue	0.692305315
ENSG00000120738.7	EGR1	turquoise	0.553357197
ENSG00000122877.9	EGR2	turquoise	0.657414651
ENSG00000179388.8	EGR3	turquoise	0.869006001

ENSG00000135625.6	EGR4	turquoise	0.657744251
ENSG00000115504.10	EHBP1	turquoise	0.921702461
ENSG00000173442.7	EHBP1L1	blue	0.761674787
ENSG00000110047.13	EHD1	blue	0.840919709
ENSG00000024422.7	EHD2	black	0.757611082
ENSG00000013016.10	EHD3	turquoise	0.882193647
ENSG00000103966.5	EHD4	black	0.821214567
ENSG00000113790.6	EHHADH	green	0.895372074
ENSG00000181090.13	EHMT1	turquoise	0.868844841
ENSG00000204371.7	EHMT2	turquoise	0.931640946
ENSG00000149547.10	EI24	turquoise	0.965766024
ENSG00000255302.3	EID1	turquoise	0.928250164
ENSG00000176396.9	EID2	turquoise	0.962444114
ENSG00000176401.5	EID2B	turquoise	0.923114525
ENSG00000255150.1	EID3	turquoise	0.913249958
ENSG00000173812.6	EIF1	turquoise	0.906468877
ENSG00000175376.4	EIF1AD	turquoise	0.894411025
ENSG00000173674.6	EIF1AX	turquoise	0.920797636
ENSG00000198692.5	EIF1AY	grey	0.199249801
ENSG00000114784.3	EIF1B	turquoise	0.947192151
ENSG00000144895.7	EIF2A	turquoise	0.935087579
ENSG00000086232.8	EIF2AK1	turquoise	0.950306939
ENSG00000055332.12	EIF2AK2	turquoise	0.860999006
ENSG00000172071.7	EIF2AK3	turquoise	0.824206132
ENSG00000128829.7	EIF2AK4	turquoise	0.97052148
ENSG00000111361.8	EIF2B1	turquoise	0.96896137
ENSG00000119718.6	EIF2B2	turquoise	0.818032982
ENSG00000070785.12	EIF2B3	turquoise	0.961539768
ENSG00000115211.11	EIF2B4	turquoise	0.90828546
ENSG00000145191.7	EIF2B5	turquoise	0.962943567
ENSG00000143486.11	EIF2D	turquoise	0.912840377
ENSG00000134001.8	EIF2S1	turquoise	0.927913885
ENSG00000125977.6	EIF2S2	turquoise	0.861256957
ENSG00000130741.6	EIF2S3	turquoise	0.919343721
ENSG00000107581.8	EIF3A	blue	0.909710801
ENSG00000106263.13	EIF3B	turquoise	0.933784557
ENSG00000184110.10	EIF3C	turquoise	0.879285528
ENSG00000100353.13	EIF3D	turquoise	0.946595213
ENSG00000104408.5	EIF3E	turquoise	0.918389061
ENSG00000234882.1	EIF3EP1	turquoise	0.917158944
ENSG00000175390.8	EIF3F	turquoise	0.931373844
ENSG00000130811.6	EIF3G	turquoise	0.852383312
ENSG00000147677.6	EIF3H	red	0.912327555
ENSG00000084623.7	EIF3I	turquoise	0.932543038
ENSG00000104131.8	EIF3J	turquoise	0.883079251
ENSG00000179523.4	EIF3J-AS1	turquoise	0.942164766
ENSG00000178982.5	EIF3K	turquoise	0.920015297
ENSG00000175749.11	EIF3KP1	turquoise	0.653425496

ENSG00000100129.13	EIF3L	turquoise	0.92423813
ENSG00000149100.8	EIF3M	turquoise	0.970585454
ENSG00000161960.10	EIF4A1	turquoise	0.873781661
ENSG00000156976.10	EIF4A2	turquoise	0.966297314
ENSG00000141543.5	EIF4A3	turquoise	0.895756676
ENSG00000063046.13	EIF4B	turquoise	0.923412552
ENSG00000197258.5	EIF4BP6	turquoise	0.702684065
ENSG00000151247.8	EIF4E	turquoise	0.950159533
ENSG00000135930.9	EIF4E2	turquoise	0.978122281
ENSG00000163412.8	EIF4E3	turquoise	0.943390097
ENSG00000187840.4	EIF4EBP1	black	0.667792023
ENSG00000148730.6	EIF4EBP2	green	0.909180628
ENSG00000243056.1	EIF4EBP3	green	0.642765011
ENSG00000184708.13	EIF4ENIF1	turquoise	0.971097699
ENSG00000114867.15	EIF4G1	turquoise	0.844975776
ENSG00000110321.11	EIF4G2	turquoise	0.887717487
ENSG00000075151.15	EIF4G3	turquoise	0.95037841
ENSG00000106682.10	EIF4H	turquoise	0.977343683
ENSG00000237977.1	EIF4HP2	grey	0.686781494
ENSG00000100664.6	EIF5	turquoise	0.900631752
ENSG00000132507.13	EIF5A	turquoise	0.920186
ENSG00000163577.3	EIF5A2	turquoise	0.887190445
ENSG00000158417.6	EIF5B	red	0.65822132
ENSG00000242372.2	EIF6	turquoise	0.929127535
ENSG00000141642.4	ELAC1	blue	0.840003561
ENSG00000006744.14	ELAC2	turquoise	0.957505196
ENSG00000066044.9	ELAVL1	turquoise	0.971660963
ENSG00000107105.10	ELAVL2	greenyellow	0.895466331
ENSG00000196361.5	ELAVL3	turquoise	0.930135158
ENSG00000162374.12	ELAVL4	greenyellow	0.855494801
ENSG00000120690.9	ELF1	blue	0.814190389
ENSG00000109381.15	ELF2	turquoise	0.897145507
ENSG00000225968.4	ELFN1	midnightblue	0.72248788
ENSG00000166897.10	ELFN2	turquoise	0.871839538
ENSG00000126767.13	ELK1	turquoise	0.852262416
ENSG00000111145.3	ELK3	blue	0.767556025
ENSG00000158711.9	ELK4	turquoise	0.764978087
ENSG00000105656.8	ELL	turquoise	0.921993003
ENSG00000118985.10	ELL2	blue	0.699306866
ENSG00000128886.7	ELL3	pink	0.841126627
ENSG00000155849.11	ELMO1	turquoise	0.943794152
ENSG00000062598.13	ELMO2	turquoise	0.932349486
ENSG00000102890.10	ELMO3	yellow	0.72285664
ENSG00000110675.8	ELMOD1	turquoise	0.936560794
ENSG00000179387.5	ELMOD2	turquoise	0.906856155
ENSG00000115459.13	ELMOD3	yellow	0.927487105
ENSG00000156030.8	ELMSAN1	cyan	0.883046021
ENSG00000049540.12	ELN	pink	0.648086706

ENSG00000130165.6	ELOF1	red	0.934277434
ENSG00000066322.8	ELOVL1	brown	0.936278955
ENSG00000197977.3	ELOVL2	green	0.878629724
ENSG00000230314.2	ELOVL2-AS1	green	0.65553476
ENSG00000118402.5	ELOVL4	turquoise	0.942738129
ENSG00000012660.9	ELOVL5	blue	0.827171745
ENSG00000170522.5	ELOVL6	turquoise	0.919468552
ENSG00000164181.9	ELOVL7	black	0.807757862
ENSG00000134759.9	ELP2	turquoise	0.968230186
ENSG00000134014.12	ELP3	turquoise	0.972055686
ENSG00000109911.13	ELP4	blue	0.872428286
ENSG00000170291.10	ELP5	turquoise	0.95361933
ENSG00000163832.11	ELP6	turquoise	0.924295843
ENSG00000162618.8	ELTD1	black	0.791817575
ENSG00000170571.7	EMB	magenta	0.767364954
ENSG00000127463.9	EMC1	turquoise	0.960905616
ENSG00000161671.12	EMC10	blue	0.877970361
ENSG00000104412.3	EMC2	blue	0.879049535
ENSG00000125037.8	EMC3	turquoise	0.954169477
ENSG00000128463.8	EMC4	turquoise	0.964001035
ENSG00000127774.6	EMC6	red	0.916306442
ENSG00000134153.5	EMC7	turquoise	0.907447666
ENSG00000131148.4	EMC8	turquoise	0.886779943
ENSG00000100908.9	EMC9	turquoise	0.919268125
ENSG00000164035.5	EMCN	black	0.533164188
ENSG00000102119.6	EMD	turquoise	0.883121816
ENSG00000197774.8	EME2	yellow	0.897604626
ENSG00000126749.10	EMG1	turquoise	0.858962322
ENSG00000186998.11	EMID1	green	0.618067572
ENSG00000138080.9	EMILIN1	black	0.703298649
ENSG00000132205.6	EMILIN2	magenta	0.616951351
ENSG00000183798.4	EMILIN3	grey	0.398145318
ENSG00000066629.12	EML1	turquoise	0.923068393
ENSG00000125746.10	EML2	brown	0.933512013
ENSG00000149499.7	EML3	blue	0.788860763
ENSG00000143924.14	EML4	turquoise	0.87423154
ENSG00000165521.11	EML5	turquoise	0.947549496
ENSG00000214595.7	EML6	turquoise	0.889389948
ENSG00000134531.5	EMP1	black	0.784563984
ENSG00000213853.5	EMP2	black	0.755228778
ENSG00000142227.6	EMP3	black	0.755813705
ENSG00000170370.10	EMX2	green	0.79472714
ENSG00000229847.4	EMX2OS	green	0.844226108
ENSG00000154380.12	ENAH	blue	0.881080974
ENSG00000171617.9	ENC1	turquoise	0.888090328
ENSG00000149218.4	ENDOD1	brown	0.868545035
ENSG00000167136.6	ENDOG	red	0.838434343
ENSG00000111405.4	ENDOU	green	0.51521049

ENSG00000173818.12	ENDOV	turquoise	0.962418033
ENSG00000106991.9	ENG	black	0.720992553
ENSG00000167280.12	ENGASE	blue	0.773248511
ENSG00000168913.6	ENHO	grey	0.06798214
ENSG00000124074.7	ENKD1	pink	0.871569429
ENSG00000074800.9	ENO1	blue	0.781435748
ENSG00000111674.4	ENO2	turquoise	0.961034859
ENSG00000108515.13	ENO3	blue	0.580093008
ENSG00000188316.9	ENO4	pink	0.767577336
ENSG00000145293.10	ENOPH1	turquoise	0.906739426
ENSG00000132199.14	ENOSF1	yellow	0.843775768
ENSG00000120658.8	ENOX1	turquoise	0.936558465
ENSG00000165675.12	ENOX2	turquoise	0.853741233
ENSG00000136960.8	ENPP2	brown	0.935367233
ENSG00000001561.6	ENPP4	turquoise	0.849969216
ENSG00000112796.5	ENPP5	turquoise	0.82127443
ENSG00000164303.6	ENPP6	turquoise	0.773676561
ENSG00000241278.1	ENPP7P4	turquoise	0.784482446
ENSG00000143420.13	ENSA	turquoise	0.913519268
ENSG00000167302.5	ENTHD2	yellow	0.901326871
ENSG00000138185.12	ENTPD1	turquoise	0.869714845
ENSG00000168032.4	ENTPD3	turquoise	0.818336006
ENSG00000223797.1	ENTPD3-AS1	red	0.797098406
ENSG00000197217.8	ENTPD4	turquoise	0.943369682
ENSG00000187097.8	ENTPD5	blue	0.888435522
ENSG00000197586.8	ENTPD6	turquoise	0.941946077
ENSG00000120533.8	ENY2	turquoise	0.956636942
ENSG00000163378.9	EOGT	blue	0.792631787
ENSG00000100393.9	EP300	turquoise	0.906622417
ENSG00000183495.9	EP400	turquoise	0.951792942
ENSG00000185684.8	EP400NL	turquoise	0.89131482
ENSG00000116016.9	EPAS1	black	0.668843936
ENSG00000159023.14	EPB41	turquoise	0.859139088
ENSG00000088367.16	EPB41L1	turquoise	0.940164922
ENSG00000079819.12	EPB41L2	turquoise	0.882684311
ENSG00000082397.11	EPB41L3	turquoise	0.878008204
ENSG00000129595.8	EPB41L4A	turquoise	0.775966276
ENSG00000224032.2	EPB41L4A-AS1	turquoise	0.858798804
ENSG00000268358.1	EPB41L4A-AS2	turquoise	0.73211062
ENSG00000115109.9	EPB41L5	green	0.929750852
ENSG00000166947.7	EPB42	turquoise	0.842450336
ENSG00000120616.11	EPC1	turquoise	0.926743606
ENSG00000135999.7	EPC2	turquoise	0.907742238
ENSG00000119888.6	EPCAM	turquoise	0.901961716
ENSG00000086289.7	EPDR1	greenyellow	0.88075921
ENSG00000152223.8	EPG5	turquoise	0.955145368
ENSG00000183317.12	EPHA10	turquoise	0.877657662
ENSG00000142627.9	EPHA2	black	0.797981705

ENSG00000116106.7	EPHA4	turquoise	0.844601671
ENSG00000080224.13	EPHA6	turquoise	0.821323342
ENSG00000135333.9	EPHA7	turquoise	0.90014436
ENSG00000070886.6	EPHA8	turquoise	0.804831747
ENSG00000154928.12	EPHB1	midnightblue	0.795870971
ENSG00000133216.12	EPHB2	turquoise	0.763461931
ENSG00000182580.2	EPHB3	green	0.65263653
ENSG00000196411.5	EPHB4	black	0.851600523
ENSG00000106123.7	EPHB6	turquoise	0.804704444
ENSG00000143819.8	EPHX1	green	0.714183992
ENSG00000120915.9	EPHX2	green	0.869511486
ENSG00000172031.6	EPHX4	turquoise	0.891580677
ENSG00000112425.9	EPM2A	brown	0.780213153
ENSG00000178567.5	EPM2AIP1	turquoise	0.96899739
ENSG00000063245.10	EPN1	turquoise	0.904206025
ENSG00000072134.11	EPN2	brown	0.811713871
ENSG00000187266.9	EPOR	turquoise	0.732701012
ENSG00000136628.13	EPRS	turquoise	0.949912139
ENSG00000085832.12	EPS15	blue	0.887110218
ENSG00000127527.9	EPS15L1	turquoise	0.913786202
ENSG00000151491.8	EPS8	green	0.78148626
ENSG00000177106.10	EPS8L2	turquoise	0.762882695
ENSG00000133106.10	EPSTI1	brown	0.615011192
ENSG00000138018.13	EPT1	turquoise	0.973643218
ENSG00000132591.7	ERAL1	turquoise	0.963440898
ENSG00000164307.8	ERAP1	turquoise	0.842914275
ENSG00000164308.12	ERAP2	grey	0.419621739
ENSG00000187682.1	ERAS	yellow	0.689350912
ENSG00000141736.9	ERBB2	green	0.782213289
ENSG00000112851.10	ERBB2IP	brown	0.807756433
ENSG00000065361.10	ERBB3	brown	0.927162381
ENSG00000178568.9	ERBB4	turquoise	0.872245389
ENSG00000082805.15	ERC1	turquoise	0.940719761
ENSG00000187672.8	ERC2	turquoise	0.856679621
ENSG00000012061.11	ERCC1	turquoise	0.938444647
ENSG00000104884.10	ERCC2	turquoise	0.940645947
ENSG00000163161.8	ERCC3	turquoise	0.97740554
ENSG00000175595.10	ERCC4	turquoise	0.898584165
ENSG00000134899.13	ERCC5	turquoise	0.919502074
ENSG00000225830.6	ERCC6	turquoise	0.933713126
ENSG00000182150.11	ERCC6L2	turquoise	0.916260235
ENSG00000049167.9	ERCC8	blue	0.870767199
ENSG00000105722.5	ERF	blue	0.792645875
ENSG00000157554.14	ERG	black	0.751334325
ENSG00000113719.11	ERGIC1	turquoise	0.93960778
ENSG00000087502.13	ERGIC2	turquoise	0.920902495
ENSG00000125991.14	ERGIC3	turquoise	0.937183467
ENSG00000100632.6	ERH	turquoise	0.902204929

ENSG00000104626.10	ERI1	blue	0.92180839
ENSG00000196678.9	ERI2	turquoise	0.793844637
ENSG00000117419.10	ERI3	turquoise	0.927943481
ENSG00000104714.9	ERICH1	turquoise	0.92128879
ENSG00000204334.7	ERICH2	turquoise	0.733449299
ENSG00000068912.9	ERLEC1	turquoise	0.959484315
ENSG00000107566.9	ERLIN1	turquoise	0.936241379
ENSG00000147475.10	ERLIN2	green	0.925384023
ENSG00000164010.9	ERMAP	turquoise	0.880606866
ENSG00000130023.11	ERMARD	yellow	0.883969246
ENSG00000136541.10	ERMN	brown	0.7452644
ENSG00000099219.9	ERMP1	brown	0.912587688
ENSG00000178607.11	ERN1	blue	0.846723034
ENSG00000197930.8	ERO1L	turquoise	0.948111951
ENSG00000086619.9	ERO1LB	turquoise	0.934685299
ENSG00000089248.6	ERP29	turquoise	0.938013924
ENSG00000023318.7	ERP44	turquoise	0.913849581
ENSG00000116285.8	ERRFI1	turquoise	0.884405841
ENSG00000213462.4	ERV3-1	turquoise	0.858211934
ENSG00000260565.2	ERVK13-1	turquoise	0.848700743
ENSG00000142396.6	ERVK3-1	turquoise	0.950033233
ENSG00000149564.7	ESAM	black	0.738551726
ENSG00000141446.6	ESCO1	turquoise	0.904693078
ENSG00000139684.9	ESD	turquoise	0.913236698
ENSG00000089048.10	ESF1	turquoise	0.867683447
ENSG00000173153.9	ESRRA	blue	0.803514939
ENSG00000196482.12	ESRRG	turquoise	0.79689467
ENSG00000139641.8	ESYT1	blue	0.888002625
ENSG00000117868.11	ESYT2	turquoise	0.899698494
ENSG00000158220.9	ESYT3	turquoise	0.821359605
ENSG00000143971.7	ETAA1	turquoise	0.830327742
ENSG00000120705.8	ETF1	turquoise	0.902692591
ENSG00000140374.11	ETFA	blue	0.865632193
ENSG00000105379.5	ETFB	red	0.933572525
ENSG00000171503.7	ETFDH	green	0.886046522
ENSG00000105755.3	ETHE1	turquoise	0.868730454
ENSG00000139163.11	ETNK1	turquoise	0.941302923
ENSG00000143845.10	ETNK2	turquoise	0.806594637
ENSG00000164089.4	ETNPPL	green	0.795986568
ENSG00000134954.10	ETS1	black	0.696460206
ENSG00000157557.7	ETS2	blue	0.62108067
ENSG00000006468.9	ETV1	turquoise	0.821790751
ENSG00000117036.7	ETV3	turquoise	0.86036213
ENSG00000244405.3	ETV5	turquoise	0.763568734
ENSG00000139083.6	ETV6	black	0.820972433
ENSG00000142694.6	EVA1B	grey	0.484028414
ENSG00000166979.8	EVA1C	blue	0.681788967
ENSG00000072840.8	EVC	green	0.896360584

ENSG00000126860.7	EVI2A	brown	0.869831057
ENSG00000185862.5	EVI2B	magenta	0.833500236
ENSG00000067208.10	EVI5	blue	0.897617504
ENSG00000142459.4	EVI5L	turquoise	0.881527073
ENSG00000196405.8	EVL	turquoise	0.921944829
ENSG00000182944.13	EWSR1	turquoise	0.946136925
ENSG00000081177.14	EXD2	blue	0.928252803
ENSG00000187609.11	EXD3	yellow	0.891070435
ENSG00000164002.7	EXO5	turquoise	0.927936461
ENSG00000090989.13	EXOC1	turquoise	0.945754885
ENSG00000112685.9	EXOC2	turquoise	0.953277674
ENSG00000180104.11	EXOC3	turquoise	0.92170276
ENSG00000179044.11	EXOC3L1	turquoise	0.809347323
ENSG00000131558.10	EXOC4	turquoise	0.963045659
ENSG00000070367.11	EXOC5	blue	0.905684976
ENSG00000138190.12	EXOC6	turquoise	0.940785591
ENSG00000144036.10	EXOC6B	turquoise	0.948734325
ENSG00000182473.16	EXOC7	turquoise	0.971710538
ENSG00000116903.6	EXOC8	turquoise	0.95581881
ENSG00000157036.8	EXOG	turquoise	0.858761243
ENSG00000171311.8	EXOSC1	turquoise	0.943398174
ENSG00000171824.9	EXOSC10	turquoise	0.878565372
ENSG00000130713.11	EXOSC2	turquoise	0.978784215
ENSG00000107371.8	EXOSC3	turquoise	0.93930093
ENSG00000178896.6	EXOSC4	red	0.943695874
ENSG00000077348.4	EXOSC5	turquoise	0.929524747
ENSG00000223496.1	EXOSC6	turquoise	0.93252782
ENSG00000075914.8	EXOSC7	turquoise	0.932338034
ENSG00000120699.8	EXOSC8	blue	0.893375119
ENSG00000123737.8	EXOSC9	turquoise	0.942777479
ENSG00000182197.6	EXT1	turquoise	0.870930562
ENSG00000151348.9	EXT2	turquoise	0.934583809
ENSG00000158008.5	EXTL1	turquoise	0.840598882
ENSG00000162694.9	EXTL2	turquoise	0.854910212
ENSG00000012232.4	EXTL3	turquoise	0.964058583
ENSG00000246339.4	EXTL3-AS1	yellow	0.872625664
ENSG00000104313.13	EYA1	turquoise	0.743683853
ENSG00000064655.14	EYA2	green	0.755204877
ENSG00000158161.11	EYA3	turquoise	0.923981979
ENSG00000108799.8	EZH1	turquoise	0.925761146
ENSG00000106462.6	EZH2	yellow	0.837343791
ENSG00000092820.13	EZR	green	0.875508095
ENSG00000126218.7	F10	brown	0.606834758
ENSG00000158769.13	F11R	blue	0.657101763
ENSG00000131187.5	F12	turquoise	0.836947628
ENSG00000124491.11	F13A1	magenta	0.732184048
ENSG00000117525.9	F3	blue	0.577275768
ENSG00000185010.9	F8	turquoise	0.907678921

ENSG00000197932.3	F8A1	turquoise	0.817680412
ENSG00000103089.4	FA2H	brown	0.898083668
ENSG00000117480.11	FAAH	turquoise	0.89226273
ENSG00000165591.6	FAAH2	turquoise	0.831466249
ENSG00000121769.3	FABP3	turquoise	0.883747712
ENSG00000170323.4	FABP4	grey	0.108436801
ENSG00000164687.6	FABP5	cyan	0.776387805
ENSG00000234964.3	FABP5P7	grey	0.280817485
ENSG00000170231.11	FABP6	turquoise	0.846440529
ENSG00000168040.4	FADD	turquoise	0.742381735
ENSG00000149485.12	FADS1	blue	0.850386745
ENSG00000134824.9	FADS2	green	0.841243308
ENSG00000221968.4	FADS3	yellow	0.842470909
ENSG00000172782.7	FADS6	turquoise	0.894848916
ENSG00000185104.15	FAF1	turquoise	0.95445376
ENSG00000113194.8	FAF2	turquoise	0.949027911
ENSG00000103876.7	FAH	green	0.823919287
ENSG00000180185.7	FAHD1	turquoise	0.897301837
ENSG00000115042.5	FAHD2A	turquoise	0.9539728
ENSG00000144199.7	FAHD2B	turquoise	0.903487317
ENSG00000231584.4	FAHD2CP	brown	0.817258387
ENSG00000158234.8	FAIM	blue	0.862704401
ENSG00000135472.4	FAIM2	turquoise	0.942323729
ENSG00000178882.9	FAM101A	turquoise	0.82239694
ENSG00000183688.4	FAM101B	turquoise	0.614030018
ENSG00000167106.7	FAM102A	turquoise	0.932402416
ENSG00000162636.11	FAM102B	turquoise	0.876963117
ENSG00000169612.3	FAM103A1	turquoise	0.937892697
ENSG00000133193.8	FAM104A	turquoise	0.959538873
ENSG00000182518.9	FAM104B	blue	0.878521065
ENSG00000145569.5	FAM105A	magenta	0.832157337
ENSG00000154124.4	FAM105B	blue	0.856008037
ENSG00000168309.12	FAM107A	green	0.637015966
ENSG00000065809.9	FAM107B	brown	0.846503065
ENSG00000198324.10	FAM109A	blue	0.801860842
ENSG00000177096.4	FAM109B	blue	0.650659821
ENSG00000125898.8	FAM110A	turquoise	0.864130595
ENSG00000169122.7	FAM110B	turquoise	0.933795158
ENSG00000184731.5	FAM110C	turquoise	0.598052617
ENSG00000197245.4	FAM110D	black	0.624515029
ENSG00000166801.11	FAM111A	blue	0.729339348
ENSG00000197712.7	FAM114A1	blue	0.775289669
ENSG00000055147.13	FAM114A2	turquoise	0.832803124
ENSG00000198420.5	FAM115A	turquoise	0.939143343
ENSG00000223459.4	FAM115B	turquoise	0.798327969
ENSG00000121104.3	FAM117A	turquoise	0.903028302
ENSG00000138439.10	FAM117B	turquoise	0.924821365
ENSG00000100376.7	FAM118A	turquoise	0.672318319

ENSG00000197798.4	FAM118B	turquoise	0.863788017
ENSG00000048828.12	FAM120A	turquoise	0.966695593
ENSG00000188938.11	FAM120AOS	blue	0.888079745
ENSG00000112584.9	FAM120B	turquoise	0.966154124
ENSG00000184083.7	FAM120C	blue	0.883380325
ENSG00000187866.6	FAM122A	turquoise	0.938163071
ENSG00000156504.12	FAM122B	turquoise	0.937915894
ENSG00000156500.10	FAM122C	turquoise	0.697627337
ENSG00000150510.11	FAM124A	brown	0.957957863
ENSG00000122591.7	FAM126A	brown	0.774238778
ENSG00000155744.5	FAM126B	turquoise	0.954493985
ENSG00000134590.9	FAM127A	turquoise	0.915850061
ENSG00000203950.6	FAM127B	turquoise	0.905962017
ENSG00000212747.3	FAM127C	turquoise	0.950272959
ENSG00000135842.12	FAM129A	magenta	0.724166285
ENSG00000136830.7	FAM129B	black	0.82605218
ENSG00000175182.9	FAM131A	turquoise	0.946680371
ENSG00000159784.13	FAM131B	turquoise	0.900161346
ENSG00000185519.8	FAM131C	grey	0.466128111
ENSG00000179083.5	FAM133A	turquoise	0.828790184
ENSG00000234545.3	FAM133B	turquoise	0.900040418
ENSG00000144567.6	FAM134A	turquoise	0.977500588
ENSG00000154153.9	FAM134B	brown	0.881994415
ENSG00000141699.6	FAM134C	turquoise	0.933676849
ENSG00000082269.12	FAM135A	turquoise	0.834641632
ENSG00000147724.7	FAM135B	turquoise	0.910881117
ENSG00000035141.3	FAM136A	turquoise	0.951766987
ENSG00000248019.2	FAM13A-AS1	yellow	0.876751032
ENSG00000031003.6	FAM13B	turquoise	0.932302844
ENSG00000148541.8	FAM13C	brown	0.873984409
ENSG00000109794.9	FAM149A	turquoise	0.873655075
ENSG00000138286.10	FAM149B1	blue	0.888165425
ENSG00000170074.15	FAM153A	turquoise	0.746491092
ENSG00000182230.7	FAM153B	turquoise	0.843403535
ENSG00000204677.6	FAM153C	turquoise	0.769736274
ENSG00000188659.5	FAM154B	pink	0.903061573
ENSG00000204442.2	FAM155A	greenyellow	0.850122754
ENSG00000130054.4	FAM155B	turquoise	0.817780291
ENSG00000051009.6	FAM160A2	turquoise	0.928843314
ENSG00000151553.10	FAM160B1	turquoise	0.956340927
ENSG00000158863.17	FAM160B2	turquoise	0.896304589
ENSG00000170264.8	FAM161A	turquoise	0.91974286
ENSG00000156050.4	FAM161B	turquoise	0.955747974
ENSG00000114023.11	FAM162A	turquoise	0.952239508
ENSG00000183807.6	FAM162B	grey	0.254508647
ENSG00000196990.4	FAM163B	greenyellow	0.870216517
ENSG00000154319.10	FAM167A	green	0.789143869
ENSG00000183615.5	FAM167B	black	0.598307688

ENSG00000054965.6	FAM168A	turquoise	0.953278598
ENSG00000152102.13	FAM168B	turquoise	0.948011139
ENSG00000198780.7	FAM169A	turquoise	0.938781174
ENSG00000148468.12	FAM171A1	turquoise	0.93521185
ENSG00000161682.10	FAM171A2	turquoise	0.902268394
ENSG00000144369.8	FAM171B	blue	0.703194632
ENSG00000113391.12	FAM172A	turquoise	0.949092752
ENSG00000103254.5	FAM173A	red	0.7261399
ENSG00000150756.9	FAM173B	blue	0.822957362
ENSG00000174132.8	FAM174A	turquoise	0.953202435
ENSG00000185442.8	FAM174B	turquoise	0.93049786
ENSG00000165660.7	FAM175B	turquoise	0.970114848
ENSG00000151327.8	FAM177A1	turquoise	0.75173701
ENSG00000119906.7	FAM178A	turquoise	0.879671664
ENSG00000168754.9	FAM178B	brown	0.822898522
ENSG00000198718.8	FAM179B	turquoise	0.954788055
ENSG00000196666.3	FAM180B	turquoise	0.628854032
ENSG00000182103.3	FAM181B	green	0.636014755
ENSG00000175170.10	FAM182B	turquoise	0.750018023
ENSG00000111879.14	FAM184A	blue	0.829501492
ENSG00000047662.4	FAM184B	turquoise	0.82168394
ENSG00000222011.4	FAM185A	turquoise	0.919076778
ENSG00000148481.9	FAM188A	turquoise	0.953864461
ENSG00000106125.14	FAM188B	pink	0.928915132
ENSG00000214237.5	FAM188B2	turquoise	0.790494489
ENSG00000104059.4	FAM189A1	turquoise	0.82222819
ENSG00000135063.13	FAM189A2	blue	0.640111656
ENSG00000160767.16	FAM189B	turquoise	0.929088444
ENSG00000172775.12	FAM192A	turquoise	0.960434585
ENSG00000125386.10	FAM193A	turquoise	0.946625129
ENSG00000146067.11	FAM193B	yellow	0.939352568
ENSG00000172366.15	FAM195A	grey	0.557273875
ENSG00000225663.3	FAM195B	red	0.81866884
ENSG00000188916.4	FAM196A	turquoise	0.926956205
ENSG00000204767.3	FAM196B	brown	0.803819346
ENSG00000144649.4	FAM198A	blue	0.733245708
ENSG00000164125.11	FAM198B	green	0.829574476
ENSG00000123575.8	FAM199X	turquoise	0.896594527
ENSG00000163377.10	FAM19A4	brown	0.810081451
ENSG00000219438.4	FAM19A5	turquoise	0.853314944
ENSG00000221909.2	FAM200A	turquoise	0.925896587
ENSG00000237765.2	FAM200B	turquoise	0.920976113
ENSG00000204860.4	FAM201A	turquoise	0.924748472
ENSG00000230992.2	FAM201B	turquoise	0.826135964
ENSG00000165669.9	FAM204A	turquoise	0.884667665
ENSG00000119328.7	FAM206A	turquoise	0.958417123
ENSG00000160256.8	FAM207A	turquoise	0.909654154
ENSG00000163946.9	FAM208A	turquoise	0.91706777

ENSG00000108021.15	FAM208B	turquoise	0.932656752
ENSG00000116199.7	FAM20B	turquoise	0.970463324
ENSG00000177706.8	FAM20C	turquoise	0.854672707
ENSG00000177150.8	FAM210A	turquoise	0.876064157
ENSG00000124098.9	FAM210B	turquoise	0.885013059
ENSG00000181350.7	FAM211A	turquoise	0.752428812
ENSG00000178026.8	FAM211B	turquoise	0.656208469
ENSG00000185614.4	FAM212A	turquoise	0.74852264
ENSG00000197852.8	FAM212B	turquoise	0.854513193
ENSG00000122378.9	FAM213A	blue	0.801658202
ENSG00000157870.10	FAM213B	turquoise	0.921504181
ENSG00000047346.8	FAM214A	turquoise	0.892241282
ENSG00000005238.15	FAM214B	turquoise	0.91903212
ENSG00000204856.7	FAM216A	turquoise	0.95471785
ENSG00000196227.6	FAM217B	turquoise	0.90269839
ENSG00000250486.2	FAM218A	turquoise	0.832069813
ENSG00000164970.10	FAM219A	brown	0.75601512
ENSG00000178761.10	FAM219B	turquoise	0.96309115
ENSG00000099290.11	FAM21A	turquoise	0.721153482
ENSG00000152726.14	FAM21B	grey	0.276992138
ENSG00000178397.8	FAM220A	turquoise	0.965298721
ENSG00000188732.6	FAM221A	turquoise	0.773129028
ENSG00000139438.5	FAM222A	brown	0.860747935
ENSG00000173065.9	FAM222B	turquoise	0.956139871
ENSG00000184949.11	FAM227A	pink	0.93098356
ENSG00000166262.11	FAM227B	blue	0.799162256
ENSG00000186453.8	FAM228A	turquoise	0.807088023
ENSG00000219626.4	FAM228B	turquoise	0.84093785
ENSG00000225828.1	FAM229A	grey	0.539048404
ENSG00000203778.3	FAM229B	turquoise	0.848036765
ENSG00000188820.8	FAM26F	turquoise	0.833873457
ENSG00000182368.4	FAM27A	turquoise	0.646850982
ENSG00000204807.1	FAM27E2	turquoise	0.404198125
ENSG00000232833.4	FAM27E3	turquoise	0.595276349
ENSG00000204805.7	FAM27E4	turquoise	0.454464399
ENSG00000105058.7	FAM32A	turquoise	0.940028193
ENSG00000122376.7	FAM35A	turquoise	0.887946424
ENSG00000071889.12	FAM3A	red	0.936776855
ENSG00000196937.6	FAM3C	turquoise	0.946894987
ENSG00000174028.6	FAM3C2	turquoise	0.835726045
ENSG00000185112.4	FAM43A	blue	0.557402181
ENSG00000183114.6	FAM43B	turquoise	0.835178238
ENSG00000119979.11	FAM45A	turquoise	0.961412177
ENSG00000112773.11	FAM46A	blue	0.539728115
ENSG00000189157.9	FAM47E	pink	0.794921433
ENSG00000118804.7	FAM47E-STBD1	blue	0.735699016
ENSG00000197872.7	FAM49A	turquoise	0.950053263
ENSG00000153310.14	FAM49B	turquoise	0.947140946

ENSG00000071859.10	FAM50A	blue	0.815175629
ENSG00000145945.5	FAM50B	turquoise	0.955479793
ENSG00000189319.9	FAM53B	brown	0.912784651
ENSG00000120709.6	FAM53C	turquoise	0.954186942
ENSG00000167695.10	FAM57A	turquoise	0.771401145
ENSG00000149926.9	FAM57B	turquoise	0.875320784
ENSG00000147382.14	FAM58A	turquoise	0.887392183
ENSG00000139146.9	FAM60A	turquoise	0.661377947
ENSG00000143409.11	FAM63A	brown	0.91701153
ENSG00000128923.6	FAM63B	turquoise	0.853121899
ENSG00000039523.13	FAM65A	turquoise	0.941388398
ENSG00000111913.11	FAM65B	turquoise	0.928879506
ENSG00000042062.7	FAM65C	grey	0.417606025
ENSG00000227888.3	FAM66A	turquoise	0.576564635
ENSG00000215374.5	FAM66B	turquoise	0.760058527
ENSG00000226711.2	FAM66C	turquoise	0.895362935
ENSG00000255052.3	FAM66D	turquoise	0.651645244
ENSG00000154511.7	FAM69A	turquoise	0.929475096
ENSG00000165716.5	FAM69B	turquoise	0.860740585
ENSG00000187773.7	FAM69C	brown	0.675341622
ENSG00000142530.6	FAM71E1	turquoise	0.828706189
ENSG00000205085.4	FAM71F2	turquoise	0.77377809
ENSG00000180488.10	FAM73A	turquoise	0.870899508
ENSG00000148343.14	FAM73B	turquoise	0.943412559
ENSG00000009780.11	FAM76A	turquoise	0.921091466
ENSG00000077458.8	FAM76B	turquoise	0.837677697
ENSG00000126882.8	FAM78A	midnightblue	0.898482396
ENSG00000188859.5	FAM78B	turquoise	0.870120196
ENSG00000157470.7	FAM81A	turquoise	0.86140683
ENSG00000101447.9	FAM83D	turquoise	0.797657001
ENSG00000180921.6	FAM83H	pink	0.793331558
ENSG00000162981.12	FAM84A	turquoise	0.903910259
ENSG00000168672.3	FAM84B	brown	0.818297407
ENSG00000255495.1	FAM85A	turquoise	0.501546
ENSG00000118894.10	FAM86A	turquoise	0.901003366
ENSG00000186523.10	FAM86B1	turquoise	0.745563303
ENSG00000173295.3	FAM86B3P	turquoise	0.802097469
ENSG00000158483.11	FAM86C1	turquoise	0.840145812
ENSG00000160172.6	FAM86C2P	turquoise	0.801244298
ENSG00000244026.2	FAM86DP	turquoise	0.833376494
ENSG00000251669.1	FAM86EP	turquoise	0.86968995
ENSG00000164845.12	FAM86FP	pink	0.78043942
ENSG00000253540.1	FAM86HP	turquoise	0.841436879
ENSG00000171084.11	FAM86JP	turquoise	0.694227983
ENSG00000182118.5	FAM89A	turquoise	0.619826754
ENSG00000176973.7	FAM89B	turquoise	0.721403793
ENSG00000137414.5	FAM8A1	turquoise	0.959721963
ENSG00000171847.6	FAM90A1	turquoise	0.879110729

ENSG00000176853.11	FAM91A1	turquoise	0.892373692
ENSG00000188343.8	FAM92A1	turquoise	0.959284507
ENSG00000157021.9	FAM92A1P1	turquoise	0.830704969
ENSG00000166797.6	FAM96A	turquoise	0.881810936
ENSG00000166595.7	FAM96B	red	0.966607846
ENSG00000119812.14	FAM98A	turquoise	0.858599568
ENSG00000171262.7	FAM98B	turquoise	0.96103536
ENSG00000130244.8	FAM98C	turquoise	0.901820094
ENSG00000198690.5	FAN1	blue	0.905697451
ENSG00000187741.10	FANCA	turquoise	0.899181317
ENSG00000158169.7	FANCC	yellow	0.786069577
ENSG00000112039.3	FANCE	blue	0.71371334
ENSG00000183161.3	FANCF	turquoise	0.891525874
ENSG00000221829.5	FANCG	yellow	0.939554247
ENSG00000140525.13	FANCI	blue	0.814627098
ENSG00000115392.7	FANCL	turquoise	0.951199609
ENSG00000187790.6	FANCM	turquoise	0.89463186
ENSG00000197601.8	FAR1	turquoise	0.897869668
ENSG00000064763.6	FAR2	turquoise	0.911179532
ENSG00000152767.11	FARP1	blue	0.742486247
ENSG00000006607.9	FARP2	turquoise	0.88142003
ENSG00000145982.7	FARS2	turquoise	0.92163244
ENSG00000179115.6	FARSA	turquoise	0.96287469
ENSG00000116120.8	FARSB	turquoise	0.970191365
ENSG00000026103.15	FAS	green	0.570466834
ENSG00000169710.6	FASN	turquoise	0.937359212
ENSG00000164896.15	FASTK	turquoise	0.896709434
ENSG00000138399.13	FASTKD1	turquoise	0.901407352
ENSG00000118246.9	FASTKD2	turquoise	0.901516973
ENSG00000124279.7	FASTKD3	turquoise	0.92709315
ENSG00000215251.3	FASTKD5	turquoise	0.922474074
ENSG00000083857.9	FAT1	green	0.875386488
ENSG00000165323.11	FAT3	turquoise	0.865780867
ENSG00000196159.7	FAT4	blue	0.694385201
ENSG00000149806.6	FAU	red	0.970191325
ENSG00000146267.11	FAXC	turquoise	0.9375843
ENSG00000170271.6	FAXDC2	brown	0.891553792
ENSG00000188878.12	FBF1	turquoise	0.865428045
ENSG00000105202.3	FBL	red	0.949693836
ENSG00000162458.8	FBLIM1	black	0.775918114
ENSG00000188573.7	FBLL1	turquoise	0.858075343
ENSG00000077942.13	FBLN1	green	0.621847669
ENSG00000163520.9	FBLN2	brown	0.581515152
ENSG00000140092.10	FBLN5	turquoise	0.892038557
ENSG00000166147.9	FBN1	turquoise	0.856660535
ENSG00000165140.5	FBP1	red	0.473015981
ENSG00000156860.11	FBRS	turquoise	0.88684716
ENSG00000112787.8	FBRSL1	turquoise	0.892873612

ENSG00000127452.4	FBXL12	yellow	0.914964207
ENSG00000171823.6	FBXL14	cyan	0.897780184
ENSG00000107872.8	FBXL15	turquoise	0.831958723
ENSG00000127585.7	FBXL16	turquoise	0.829519346
ENSG00000145743.11	FBXL17	turquoise	0.933121417
ENSG00000155034.14	FBXL18	turquoise	0.86726987
ENSG00000099364.12	FBXL19	turquoise	0.899504789
ENSG00000260852.1	FBXL19-AS1	brown	0.654660213
ENSG00000153558.9	FBXL2	turquoise	0.9462645
ENSG00000108306.7	FBXL20	turquoise	0.889724733
ENSG00000164616.10	FBXL21	turquoise	0.769309719
ENSG00000197361.5	FBXL22	turquoise	0.804688646
ENSG00000005812.6	FBXL3	turquoise	0.901275759
ENSG00000112234.4	FBXL4	turquoise	0.87965404
ENSG00000118564.10	FBXL5	blue	0.917461374
ENSG00000182325.6	FBXL6	yellow	0.916742964
ENSG00000183580.8	FBXL7	green	0.812052944
ENSG00000135722.4	FBXL8	yellow	0.84514188
ENSG00000147912.8	FBXO10	turquoise	0.948631734
ENSG00000141665.7	FBXO15	pink	0.92269448
ENSG00000214050.3	FBXO16	turquoise	0.894411137
ENSG00000269190.1	FBXO17	turquoise	0.912900127
ENSG00000134452.15	FBXO18	turquoise	0.972632673
ENSG00000116661.9	FBXO2	red	0.786609901
ENSG00000135108.10	FBXO21	turquoise	0.958748698
ENSG00000167196.9	FBXO22	turquoise	0.91512517
ENSG00000147364.12	FBXO25	turquoise	0.955537769
ENSG00000161243.4	FBXO27	turquoise	0.799507318
ENSG00000143756.7	FBXO28	turquoise	0.954753905
ENSG00000110429.9	FBXO3	turquoise	0.966395555
ENSG00000118496.4	FBXO30	green	0.811637067
ENSG00000103264.11	FBXO31	turquoise	0.956015027
ENSG00000156804.3	FBXO32	brown	0.837429417
ENSG00000165355.7	FBXO33	turquoise	0.931475338
ENSG00000178974.5	FBXO34	turquoise	0.957495384
ENSG00000153832.7	FBXO36	blue	0.811447632
ENSG00000145868.12	FBXO38	turquoise	0.936111728
ENSG00000151876.8	FBXO4	turquoise	0.849569259
ENSG00000163013.7	FBXO41	turquoise	0.889858325
ENSG00000037637.6	FBXO42	turquoise	0.939797474
ENSG00000132879.9	FBXO44	turquoise	0.930297961
ENSG00000174013.7	FBXO45	turquoise	0.893949089
ENSG00000177051.5	FBXO46	turquoise	0.886547273
ENSG00000204923.3	FBXO48	turquoise	0.826238413
ENSG00000112029.5	FBXO5	turquoise	0.821485464
ENSG00000116663.6	FBXO6	turquoise	0.892634082
ENSG00000100225.13	FBXO7	blue	0.893676019
ENSG00000164117.8	FBXO8	blue	0.867039826

ENSG00000112146.12	FBXO9	turquoise	0.934474513
ENSG00000072803.13	FBXW11	turquoise	0.926503535
ENSG00000119402.12	FBXW2	turquoise	0.962025825
ENSG00000107829.9	FBXW4	brown	0.6945796
ENSG00000159069.9	FBXW5	turquoise	0.914272246
ENSG00000109670.9	FBXW7	turquoise	0.899535079
ENSG00000174989.8	FBXW8	turquoise	0.889907056
ENSG00000132004.8	FBXW9	pink	0.935454969
ENSG00000158869.6	FCER1G	magenta	0.931004555
ENSG00000119616.7	FCF1	turquoise	0.874428908
ENSG00000228638.1	FCF1P2	turquoise	0.783705468
ENSG00000090920.9	FCGBP	magenta	0.739198613
ENSG00000150337.9	FCGR1A	magenta	0.861869644
ENSG00000198019.8	FCGR1B	magenta	0.799953111
ENSG00000143226.9	FCGR2A	magenta	0.905760687
ENSG00000203747.5	FCGR3A	magenta	0.915006801
ENSG00000162747.5	FCGR3B	grey	0.120624668
ENSG00000104870.8	FCGRT	blue	0.705529698
ENSG00000130475.10	FCHO1	brown	0.885782387
ENSG00000157107.9	FCHO2	turquoise	0.772091778
ENSG00000197948.6	FCHSD1	yellow	0.915844853
ENSG00000137478.10	FCHSD2	brown	0.865337374
ENSG00000085265.6	FCN1	grey	0.057421377
ENSG00000079459.8	FDFT1	turquoise	0.896261307
ENSG00000160752.10	FDPS	turquoise	0.930281982
ENSG00000137714.2	FDX1	turquoise	0.908303946
ENSG00000267673.2	FDX1L	turquoise	0.906312929
ENSG00000255561.2	FDXACB1	turquoise	0.877825963
ENSG00000161513.7	FDXR	green	0.678520454
ENSG00000066926.6	FECH	blue	0.897627812
ENSG00000141965.3	FEM1A	turquoise	0.634963131
ENSG00000169018.5	FEM1B	turquoise	0.959202206
ENSG00000145780.6	FEM1C	turquoise	0.877377747
ENSG00000168496.3	FEN1	turquoise	0.889115886
ENSG00000268388.1	FENDRR	black	0.655722601
ENSG00000151422.8	FER	turquoise	0.858725265
ENSG00000088340.11	FER1L4	midnightblue	0.79683589
ENSG00000249715.5	FER1L5	turquoise	0.802385663
ENSG00000101311.11	FERMT1	greenyellow	0.63855512
ENSG00000073712.9	FERMT2	green	0.927588153
ENSG00000149781.8	FERMT3	magenta	0.782103054
ENSG00000182511.7	FES	blue	0.692732632
ENSG00000163497.2	FEV	turquoise	0.530190035
ENSG00000149557.8	FEZ1	brown	0.886515295
ENSG00000171055.10	FEZ2	blue	0.909650442
ENSG00000128610.7	FEZF1	green	0.617961297
ENSG00000230316.2	FEZF1-AS1	green	0.684610105
ENSG00000153266.8	FEZF2	grey	0.163521241

ENSG00000126266.2	FFAR1	brown	0.709950882
ENSG00000102302.7	FGD1	turquoise	0.950091165
ENSG00000146192.10	FGD2	magenta	0.90274979
ENSG00000127084.13	FGD3	magenta	0.816331394
ENSG00000139132.10	FGD4	turquoise	0.845696397
ENSG00000154783.6	FGD5	black	0.678417062
ENSG00000225733.1	FGD5-AS1	blue	0.89189774
ENSG00000180263.9	FGD6	green	0.847646146
ENSG00000113578.13	FGF1	brown	0.726791551
ENSG00000161958.6	FGF11	blue	0.554445238
ENSG00000114279.9	FGF12	turquoise	0.887963812
ENSG00000129682.9	FGF13	turquoise	0.910506808
ENSG00000102466.11	FGF14	turquoise	0.921179464
ENSG00000272143.1	FGF14-AS2	brown	0.639668745
ENSG00000158815.6	FGF17	greenyellow	0.571531174
ENSG00000138685.8	FGF2	green	0.807643644
ENSG00000070388.7	FGF22	turquoise	0.702937694
ENSG00000102678.6	FGF9	turquoise	0.886797466
ENSG00000137441.7	FGFBP2	grey	0.193678664
ENSG00000174721.9	FGFBP3	turquoise	0.730055696
ENSG00000077782.15	FGFR1	blue	0.798055585
ENSG00000213066.7	FGFR1OP	pink	0.933705281
ENSG00000111790.9	FGFR1OP2	turquoise	0.937821383
ENSG00000066468.16	FGFR2	brown	0.898400206
ENSG00000068078.13	FGFR3	green	0.76644008
ENSG00000160867.10	FGFR4	grey	0.705894402
ENSG00000127418.10	FGFRL1	green	0.672982257
ENSG00000172456.12	FGGY	turquoise	0.688846679
ENSG00000127951.5	FGL2	magenta	0.789580705
ENSG00000000938.8	FGR	black	0.835067238
ENSG00000091483.6	FH	turquoise	0.941596046
ENSG00000137460.4	FHDC1	turquoise	0.874362754
ENSG00000189283.5	FHIT	turquoise	0.878743032
ENSG00000022267.12	FHL1	turquoise	0.856183624
ENSG00000115641.14	FHL2	turquoise	0.619660579
ENSG00000183386.5	FHL3	black	0.806894115
ENSG00000112214.6	FHL5	grey	0.318712778
ENSG00000135723.9	FHOD1	turquoise	0.880481699
ENSG00000134775.11	FHOD3	turquoise	0.848529573
ENSG00000130720.8	FIBCD1	turquoise	0.699978224
ENSG00000176971.2	FIBIN	green	0.863647406
ENSG00000172500.8	FIBP	turquoise	0.909933986
ENSG00000198855.2	FICD	turquoise	0.71231685
ENSG00000112367.6	FIG4	turquoise	0.958016542
ENSG00000165197.4	FIGF	yellow	0.847980308
ENSG00000182263.9	FIGN	blue	0.752443137
ENSG00000132436.7	FIGNL1	turquoise	0.925867232
ENSG00000118407.10	FILIP1	turquoise	0.796922193

ENSG00000168386.14	FILIP1L	grey	0.315998579
ENSG00000145216.11	FIP1L1	turquoise	0.956106342
ENSG00000214253.4	FIS1	red	0.953857274
ENSG00000197296.5	FITM2	turquoise	0.928440901
ENSG00000179943.6	FIZ1	turquoise	0.854235493
ENSG00000179431.5	FJX1	green	0.502353378
ENSG00000141756.14	FKBP10	green	0.822381636
ENSG00000134285.6	FKBP11	turquoise	0.882265327
ENSG00000106080.6	FKBP14	turquoise	0.899397522
ENSG00000119321.4	FKBP15	turquoise	0.91415164
ENSG00000088832.10	FKBP1A	turquoise	0.915313255
ENSG00000119782.9	FKBP1B	turquoise	0.840164014
ENSG00000198225.4	FKBP1C	turquoise	0.736904427
ENSG00000173486.8	FKBP2	red	0.938187537
ENSG00000100442.6	FKBP3	turquoise	0.960519138
ENSG00000004478.5	FKBP4	cyan	0.927341596
ENSG00000096060.10	FKBP5	green	0.5172695
ENSG00000079150.13	FKBP7	turquoise	0.658787522
ENSG00000105701.11	FKBP8	turquoise	0.888330781
ENSG00000122642.6	FKBP9	green	0.740999982
ENSG00000204315.3	FKBPL	turquoise	0.882392056
ENSG00000181027.6	FKRP	turquoise	0.955004302
ENSG00000106692.9	FKTN	turquoise	0.931584426
ENSG00000160688.14	FLAD1	turquoise	0.930057262
ENSG00000154803.8	FLCN	turquoise	0.897743319
ENSG00000237975.2	FLG-AS1	turquoise	0.657918814
ENSG00000151702.12	FLI1	black	0.694510139
ENSG00000177731.11	FLII	turquoise	0.863437987
ENSG00000205047.3	FLJ00104	turquoise	0.88910258
ENSG00000268927.1	FLJ00418	green	0.663287979
ENSG00000197182.8	FLJ27365	yellow	0.512956574
ENSG00000272268.1	FLJ30594	brown	0.911702642
ENSG00000196924.10	FLNA	blue	0.732924987
ENSG00000136068.10	FLNB	yellow	0.866573945
ENSG00000128591.11	FLNC	brown	0.694558841
ENSG00000137312.10	FLOT1	turquoise	0.887420082
ENSG00000132589.11	FLOT2	turquoise	0.886178742
ENSG00000126500.3	FLRT1	turquoise	0.88131619
ENSG00000185070.6	FLRT2	blue	0.73191397
ENSG00000125848.9	FLRT3	turquoise	0.794006051
ENSG00000102755.6	FLT1	black	0.709632701
ENSG00000090554.8	FLT3LG	black	0.705787867
ENSG00000037280.11	FLT4	midnightblue	0.775902242
ENSG00000162769.8	FLVCR1	turquoise	0.910613314
ENSG00000198468.3	FLVCR1-AS1	turquoise	0.688611938
ENSG00000119686.5	FLVCR2	black	0.756865957
ENSG00000059122.12	FLYWCH1	turquoise	0.883567441
ENSG00000162076.8	FLYWCH2	turquoise	0.856156558

ENSG00000155816.15	FMN2	blue	0.871811201
ENSG00000184922.9	FMNL1	turquoise	0.883395192
ENSG00000157827.15	FMNL2	brown	0.855431344
ENSG00000161791.9	FMNL3	blue	0.774853331
ENSG00000094963.9	FMO2	grey	0.301185486
ENSG00000076258.5	FMO4	blue	0.662964618
ENSG00000131781.8	FMO5	blue	0.7853998
ENSG00000122176.10	FMOD	brown	0.556341106
ENSG00000102081.9	FMR1	blue	0.88581202
ENSG00000115414.14	FN1	black	0.694722149
ENSG00000167363.9	FN3K	turquoise	0.895592992
ENSG00000141560.10	FN3KRP	turquoise	0.91362166
ENSG00000187239.12	FNBP1	brown	0.931291583
ENSG00000137942.12	FNBP1L	turquoise	0.915403156
ENSG00000109920.8	FNBP4	yellow	0.951758156
ENSG00000102531.12	FNDC3A	turquoise	0.947357218
ENSG00000075420.8	FNDC3B	blue	0.780447485
ENSG00000115226.5	FNDC4	turquoise	0.870160227
ENSG00000160097.11	FNDC5	turquoise	0.819707525
ENSG00000217128.7	FNIP1	turquoise	0.893395967
ENSG00000052795.8	FNIP2	turquoise	0.814143258
ENSG00000168522.8	FNTA	brown	0.840207389
ENSG00000257365.3	FNTB	turquoise	0.886555339
ENSG00000188352.8	FOCAD	turquoise	0.961535763
ENSG00000086205.12	FOLH1	brown	0.872040324
ENSG00000165457.9	FOLR2	magenta	0.78463504
ENSG00000133393.8	FOPNL	turquoise	0.934721233
ENSG00000170345.5	FOS	black	0.528283976
ENSG00000125740.9	FOSB	turquoise	0.625194941
ENSG00000175592.4	FOSL1	black	0.659534605
ENSG00000075426.7	FOSL2	black	0.718639459
ENSG00000054598.5	FOXC1	black	0.654664665
ENSG00000170122.4	FOXD4	turquoise	0.612387568
ENSG00000103241.5	FOXF1	grey	0.30968199
ENSG00000137273.3	FOXF2	grey	0.362960111
ENSG00000176165.7	FOXG1	turquoise	0.866339227
ENSG00000065970.4	FOXJ2	turquoise	0.938809092
ENSG00000198815.4	FOXJ3	turquoise	0.974540407
ENSG00000164916.9	FOXK1	turquoise	0.916418525
ENSG00000141568.15	FOXK2	turquoise	0.969480446
ENSG00000111206.8	FOXM1	turquoise	0.69933711
ENSG00000170802.11	FOXN2	brown	0.86573449
ENSG00000053254.11	FOXN3	blue	0.830780644
ENSG00000258920.1	FOXN3-AS1	grey	0.439983788
ENSG00000150907.6	FOXO1	turquoise	0.911818035
ENSG00000118689.10	FOXO3	turquoise	0.943616118
ENSG00000184481.12	FOXO4	brown	0.598076821
ENSG00000204060.4	FOXO6	turquoise	0.808693597

ENSG00000114861.14	FOXP1	turquoise	0.887427686
ENSG00000128573.18	FOXP2	turquoise	0.794028951
ENSG00000137166.10	FOXP4	turquoise	0.900103166
ENSG00000164379.4	FOXQ1	grey	0.236688853
ENSG00000110074.6	FOXRED1	turquoise	0.937641154
ENSG00000100350.10	FOXRED2	turquoise	0.901152053
ENSG00000179772.6	FOXS1	grey	0.245569133
ENSG00000136877.10	FPGS	blue	0.805878779
ENSG00000254685.2	FPGT	blue	0.848993779
ENSG00000171051.4	FPR1	magenta	0.821611921
ENSG00000148690.10	FRA10AC1	turquoise	0.855089192
ENSG00000138759.13	FRAS1	turquoise	0.852421764
ENSG00000165879.7	FRAT1	turquoise	0.878354138
ENSG00000181274.5	FRAT2	turquoise	0.886180009
ENSG00000109536.7	FRG1	turquoise	0.935190343
ENSG00000149531.10	FRG1B	turquoise	0.834636353
ENSG00000172159.11	FRMD3	blue	0.552836002
ENSG00000151474.15	FRMD4A	turquoise	0.792262508
ENSG00000114541.10	FRMD4B	brown	0.939938657
ENSG00000171877.15	FRMD5	brown	0.898926056
ENSG00000139926.11	FRMD6	blue	0.499815477
ENSG00000126391.9	FRMD8	blue	0.827740591
ENSG00000070601.5	FRMPD1	midnightblue	0.887925635
ENSG00000147234.6	FRMPD3	turquoise	0.725849432
ENSG00000169933.8	FRMPD4	turquoise	0.911929514
ENSG00000260230.2	FRRS1L	turquoise	0.935727917
ENSG00000166225.4	FRS2	turquoise	0.892465084
ENSG00000137218.6	FRS3	turquoise	0.900155297
ENSG00000073910.15	FRY	turquoise	0.953065426
ENSG00000075539.9	FRYL	brown	0.932475826
ENSG00000162998.4	FRZB	brown	0.598705454
ENSG00000075618.13	FSCN1	brown	0.795516078
ENSG00000105255.6	FSD1	turquoise	0.855233137
ENSG00000106701.7	FSD1L	turquoise	0.893418222
ENSG00000150667.6	FSIP1	pink	0.884070134
ENSG00000163430.5	FSTL1	green	0.658406983
ENSG00000070404.5	FSTL3	blue	0.685837533
ENSG00000053108.12	FSTL4	turquoise	0.868540405
ENSG00000168843.9	FSTL5	turquoise	0.757720672
ENSG00000160282.9	FTCD	grey	0.710408899
ENSG00000237338.1	FTCD-AS1	turquoise	0.590447343
ENSG00000226124.2	FTCDNL1	turquoise	0.69968606
ENSG00000167996.11	FTH1	red	0.802577888
ENSG00000087086.9	FTL	black	0.577778797
ENSG00000068438.10	FTSJ1	turquoise	0.960829586
ENSG00000122687.13	FTSJ2	turquoise	0.916620083
ENSG00000108592.12	FTSJ3	turquoise	0.79475428
ENSG00000162613.12	FUBP1	turquoise	0.928781202

ENSG00000107164.15	FUBP3	blue	0.931216243
ENSG00000179163.11	FUCA1	turquoise	0.921409573
ENSG00000001036.9	FUCA2	blue	0.90317385
ENSG00000157353.12	FUK	turquoise	0.874975602
ENSG00000069509.5	FUNDC1	turquoise	0.945805617
ENSG00000165775.13	FUNDC2	turquoise	0.914401002
ENSG00000148803.7	FUOM	magenta	0.587925224
ENSG00000140564.6	FURIN	blue	0.81208678
ENSG00000089280.14	FUS	turquoise	0.940746984
ENSG00000172728.11	FUT10	green	0.821539775
ENSG00000196968.6	FUT11	turquoise	0.858359301
ENSG00000176920.10	FUT2	yellow	0.699043464
ENSG00000196371.2	FUT4	turquoise	0.887441416
ENSG00000033170.12	FUT8	brown	0.920161258
ENSG00000172461.6	FUT9	blue	0.758603795
ENSG00000010361.9	FUZ	turquoise	0.860779705
ENSG00000165060.7	FXN	turquoise	0.90187482
ENSG00000114416.13	FXR1	blue	0.900521041
ENSG00000129245.7	FXR2	turquoise	0.953077855
ENSG00000266964.1	FXYD1	green	0.654398456
ENSG00000089356.12	FXYD3	black	0.522846029
ENSG00000150201.10	FXYD4	brown	0.678883948
ENSG00000089327.10	FXYD5	black	0.835246348
ENSG00000137726.11	FXYD6	greenyellow	0.869912888
ENSG00000221946.3	FXYD7	turquoise	0.63445036
ENSG00000082074.11	FYB	magenta	0.898383709
ENSG00000163820.10	FYCO1	blue	0.776086362
ENSG00000010810.13	FYN	turquoise	0.852052291
ENSG00000122068.8	FYTTD1	turquoise	0.933830555
ENSG00000157240.2	FZD1	green	0.703589079
ENSG00000180340.5	FZD2	grey	0.200235537
ENSG00000104290.6	FZD3	turquoise	0.929104342
ENSG00000174804.3	FZD4	blue	0.691597706
ENSG00000163251.3	FZD5	blue	0.811790566
ENSG00000164930.7	FZD6	blue	0.691851963
ENSG00000155760.1	FZD7	green	0.739575037
ENSG00000177283.4	FZD8	green	0.663047222
ENSG00000188763.3	FZD9	grey	0.471278538
ENSG00000105325.9	FZR1	turquoise	0.940833649
ENSG00000123689.5	G0S2	black	0.366207708
ENSG00000092140.10	G2E3	turquoise	0.921658006
ENSG00000145907.10	G3BP1	turquoise	0.854658873
ENSG00000138757.10	G3BP2	turquoise	0.962417243
ENSG00000141349.4	G6PC3	blue	0.830657665
ENSG00000160211.11	G6PD	turquoise	0.873043378
ENSG00000171298.8	GAA	turquoise	0.907270073
ENSG00000109458.4	GAB1	brown	0.881364067
ENSG00000033327.8	GAB2	brown	0.677810056

ENSG00000160219.7	GAB3	magenta	0.904516459
ENSG00000170296.5	GABARAP	turquoise	0.872948276
ENSG00000139112.6	GABARAPL1	turquoise	0.95207525
ENSG00000034713.3	GABARAPL2	turquoise	0.943391939
ENSG00000204681.6	GABBR1	turquoise	0.889220894
ENSG00000136928.4	GABBR2	greenyellow	0.931627532
ENSG00000154727.6	GABPA	turquoise	0.849548525
ENSG00000104064.12	GABPB1	cyan	0.918402104
ENSG00000244879.3	GABPB1-AS1	turquoise	0.885271244
ENSG00000143458.7	GABPB2	turquoise	0.824196088
ENSG00000022355.10	GABRA1	turquoise	0.874242551
ENSG00000151834.11	GABRA2	blue	0.726780793
ENSG00000011677.8	GABRA3	turquoise	0.745143233
ENSG00000109158.6	GABRA4	turquoise	0.872853724
ENSG00000186297.7	GABRA5	turquoise	0.78177999
ENSG00000163288.9	GABRB1	blue	0.676504392
ENSG00000145864.8	GABRB2	turquoise	0.900043224
ENSG00000166206.9	GABRB3	turquoise	0.928368229
ENSG00000187730.6	GABRD	turquoise	0.851618767
ENSG00000102287.12	GABRE	black	0.578805512
ENSG00000163285.7	GABRG1	green	0.854210667
ENSG00000113327.10	GABRG2	turquoise	0.922901171
ENSG00000147402.7	GABRQ	greenyellow	0.653748873
ENSG00000128683.9	GAD1	turquoise	0.920861285
ENSG00000136750.7	GAD2	turquoise	0.917980915
ENSG00000116717.7	GADD45A	cyan	0.7491916
ENSG00000099860.4	GADD45B	black	0.757183354
ENSG00000130222.6	GADD45G	black	0.491257376
ENSG00000179271.1	GADD45GIP1	red	0.950596342
ENSG00000215274.5	GAGE10	grey	0.574524438
ENSG00000178950.12	GAK	turquoise	0.929317706
ENSG00000128242.8	GAL3ST1	brown	0.791534089
ENSG00000175229.6	GAL3ST3	turquoise	0.833210862
ENSG00000197093.6	GAL3ST4	magenta	0.667778606
ENSG00000054983.12	GALC	blue	0.940589844
ENSG00000117308.10	GALE	turquoise	0.905530756
ENSG00000108479.7	GALK1	blue	0.775663768
ENSG00000156958.10	GALK2	turquoise	0.876539064
ENSG00000143891.12	GALM	green	0.796074785
ENSG00000141012.8	GALNS	pink	0.893767076
ENSG00000141429.9	GALNT1	turquoise	0.893229564
ENSG00000164574.11	GALNT10	blue	0.867269626
ENSG00000178234.8	GALNT11	turquoise	0.968740752
ENSG00000119514.5	GALNT12	brown	0.778705796
ENSG00000144278.10	GALNT13	turquoise	0.901517809
ENSG00000158089.10	GALNT14	greenyellow	0.791512753
ENSG00000131386.13	GALNT15	black	0.70034939
ENSG00000100626.12	GALNT16	turquoise	0.754595557

ENSG00000110328.5	GALNT18	turquoise	0.83003121
ENSG00000143641.8	GALNT2	turquoise	0.82674232
ENSG00000257594.2	GALNT4	blue	0.805121628
ENSG00000139629.11	GALNT6	brown	0.811683611
ENSG00000109586.7	GALNT7	turquoise	0.875533953
ENSG00000182870.8	GALNT9	greenyellow	0.751580276
ENSG00000174473.11	GALNTL6	turquoise	0.842883262
ENSG00000213930.7	GALT	turquoise	0.933591524
ENSG00000130005.7	GAMT	red	0.9247896
ENSG00000261609.3	GAN	blue	0.839262196
ENSG00000089597.12	GANAB	turquoise	0.955133774
ENSG00000214013.5	GANC	turquoise	0.793123924
ENSG00000172020.8	GAP43	turquoise	0.880732161
ENSG00000111640.10	GAPDH	turquoise	0.92286616
ENSG00000165219.17	GAPVD1	turquoise	0.94252962
ENSG00000109534.12	GAR1	turquoise	0.908548523
ENSG00000141441.11	GAREM	green	0.803197641
ENSG00000157833.8	GAREML	green	0.637642445
ENSG00000136895.14	GARNL3	turquoise	0.892246756
ENSG00000106105.9	GARS	turquoise	0.959960458
ENSG00000159131.12	GART	turquoise	0.911432506
ENSG00000180447.5	GAS1	turquoise	0.689021576
ENSG00000148935.6	GAS2	turquoise	0.794491249
ENSG00000185340.11	GAS2L1	turquoise	0.733976068
ENSG00000132139.8	GAS2L2	pink	0.884074106
ENSG00000234741.3	GAS5	red	0.86246762
ENSG00000270084.1	GAS5-AS1	blue	0.743690933
ENSG00000183087.10	GAS6	turquoise	0.876837283
ENSG00000272695.1	GAS6-AS2	turquoise	0.725297717
ENSG00000007237.14	GAS7	turquoise	0.926154581
ENSG00000141013.10	GAS8	pink	0.890667871
ENSG00000179348.7	GATA2	black	0.571696655
ENSG00000157259.6	GATAD1	turquoise	0.840735321
ENSG00000167491.13	GATAD2A	turquoise	0.890994704
ENSG00000143614.7	GATAD2B	turquoise	0.966938803
ENSG00000171766.11	GATM	blue	0.811809532
ENSG00000239521.3	GATS	brown	0.836983887
ENSG00000239282.3	GATSL3	green	0.630585164
ENSG00000177628.11	GBA	turquoise	0.939606742
ENSG00000070610.10	GBA2	turquoise	0.943551839
ENSG00000160766.10	GBAP1	turquoise	0.883550385
ENSG00000146729.5	GBAS	blue	0.82274765
ENSG00000114480.8	GBE1	blue	0.841322809
ENSG00000107862.4	GBF1	turquoise	0.958218124
ENSG00000148288.7	GBGT1	blue	0.686955253
ENSG00000117228.9	GBP1	black	0.800021524
ENSG00000162645.8	GBP2	black	0.860524709
ENSG00000117226.7	GBP3	black	0.641145976

ENSG00000162654.8	GBP4	grey	0.37810078
ENSG00000168505.6	GBX2	midnightblue	0.754564032
ENSG00000115271.6	GCA	blue	0.855450688
ENSG00000100116.12	GCAT	turquoise	0.812995486
ENSG00000179562.2	GCC1	turquoise	0.847091037
ENSG00000135968.15	GCC2	turquoise	0.888615666
ENSG00000105607.8	GCDH	blue	0.74568708
ENSG00000005436.9	GCFC2	turquoise	0.895247929
ENSG00000131979.14	GCH1	turquoise	0.871470822
ENSG00000137880.4	GCHFR	turquoise	0.822689549
ENSG00000106633.11	GCK	turquoise	0.600283676
ENSG00000001084.6	GCLC	turquoise	0.90693009
ENSG00000023909.5	GCLM	turquoise	0.785252292
ENSG00000089154.6	GCN1L1	turquoise	0.88769746
ENSG00000187210.8	GCNT1	turquoise	0.827482566
ENSG00000140905.5	GCSH	blue	0.676537026
ENSG00000104381.8	GDAP1	turquoise	0.955307611
ENSG00000124194.11	GDAP1L1	turquoise	0.87942997
ENSG00000196505.6	GDAP2	turquoise	0.82375335
ENSG00000006007.7	GDE1	turquoise	0.913322034
ENSG00000107623.4	GDF10	green	0.623776247
ENSG00000135414.5	GDF11	turquoise	0.619399484
ENSG00000164404.4	GDF9	turquoise	0.785583227
ENSG00000203879.7	GDI1	turquoise	0.96622194
ENSG00000057608.12	GDI2	turquoise	0.916900947
ENSG00000248587.2	GDNF-AS1	yellow	0.709422655
ENSG00000153982.6	GDPD1	turquoise	0.938799337
ENSG00000102886.10	GDPD3	grey	0.723269587
ENSG00000158555.10	GDPD5	turquoise	0.819413383
ENSG00000183208.8	GDPGP1	turquoise	0.854386972
ENSG00000164949.3	GEM	blue	0.588560468
ENSG00000092208.12	GEMIN2	turquoise	0.944856892
ENSG00000179409.6	GEMIN4	turquoise	0.929333487
ENSG00000082516.8	GEMIN5	turquoise	0.96083016
ENSG00000152147.6	GEMIN6	turquoise	0.915984429
ENSG00000142252.6	GEMIN7	turquoise	0.827496548
ENSG00000046647.9	GEMIN8	turquoise	0.841960147
ENSG00000228175.2	GEMIN8P4	turquoise	0.766577938
ENSG00000178295.10	GEN1	turquoise	0.7706726
ENSG00000239857.2	GET4	turquoise	0.84315217
ENSG00000131095.7	GFAP	magenta	0.619566935
ENSG00000127554.12	GFER	turquoise	0.896132722
ENSG00000168827.10	GFM1	turquoise	0.895936991
ENSG00000164347.13	GFM2	turquoise	0.953041667
ENSG00000145990.6	GFOD1	turquoise	0.937378883
ENSG00000141098.8	GFOD2	turquoise	0.955902376
ENSG00000198380.8	GFPT1	turquoise	0.93278659
ENSG00000131459.8	GFPT2	pink	0.844643349

ENSG00000151892.10	GFRA1	turquoise	0.728475257
ENSG00000168546.6	GFRA2	greenyellow	0.803152828
ENSG00000100083.14	GGA1	turquoise	0.88702996
ENSG00000103365.11	GGA2	turquoise	0.948589906
ENSG00000125447.12	GGA3	turquoise	0.967821776
ENSG00000134864.6	GGACT	blue	0.716585387
ENSG00000006625.13	GGCT	turquoise	0.925750611
ENSG00000115486.7	GGCX	turquoise	0.941169582
ENSG00000137563.7	GGH	turquoise	0.914699502
ENSG00000179168.10	GGN	turquoise	0.69116711
ENSG00000005955.8	GGNBP2	turquoise	0.974139337
ENSG00000152904.7	GGPS1	turquoise	0.947261645
ENSG00000100031.14	GGT1	yellow	0.761705424
ENSG00000099998.13	GGT5	black	0.736111306
ENSG00000131067.12	GGT7	turquoise	0.955050816
ENSG00000204136.6	GGTA1P	green	0.474657037
ENSG00000259384.2	GH1	grey	0.117133006
ENSG00000167925.11	GHDC	turquoise	0.88124244
ENSG00000165678.15	GHITM	turquoise	0.949934921
ENSG00000240288.3	GHRLOS	turquoise	0.69700442
ENSG00000141034.5	GID4	turquoise	0.949933141
ENSG00000101193.6	GID8	turquoise	0.956619764
ENSG00000146830.9	GIGYF1	yellow	0.960937743
ENSG00000204120.10	GIGYF2	turquoise	0.919036868
ENSG00000213203.2	GIMAP1	grey	0.496496105
ENSG00000106560.6	GIMAP2	magenta	0.746178463
ENSG00000133574.5	GIMAP4	grey	0.462964664
ENSG00000196329.6	GIMAP5	black	0.781854274
ENSG00000133561.11	GIMAP6	black	0.623535826
ENSG00000179144.4	GIMAP7	grey	0.344687347
ENSG00000171115.3	GIMAP8	black	0.639528263
ENSG00000145723.12	GIN1	turquoise	0.91932315
ENSG00000055211.8	GINM1	blue	0.931023577
ENSG00000101003.8	GINS1	turquoise	0.896252962
ENSG00000131153.4	GINS2	blue	0.714594519
ENSG00000181938.9	GINS3	midnightblue	0.734045695
ENSG00000123159.11	GIPC1	red	0.92188162
ENSG00000108262.11	GIT1	turquoise	0.862657365
ENSG00000139436.16	GIT2	turquoise	0.921180049
ENSG00000152661.7	GJA1	green	0.858511442
ENSG00000187513.8	GJA4	black	0.670852819
ENSG00000143140.6	GJA5	grey	0.262126456
ENSG00000169562.9	GJB1	brown	0.856963965
ENSG00000165474.5	GJB2	green	0.58433017
ENSG00000121742.11	GJB6	green	0.724313372
ENSG00000182963.5	GJC1	grey	0.30622635
ENSG00000198835.3	GJC2	brown	0.868292007
ENSG00000159248.4	GJD2	turquoise	0.753055995

ENSG00000198814.8	GK	turquoise	0.873484319
ENSG00000175066.11	GK5	yellow	0.885211791
ENSG00000165113.8	GKAP1	blue	0.803471726
ENSG00000102393.5	GLA	blue	0.866024628
ENSG00000170266.11	GLB1	turquoise	0.926451284
ENSG00000163521.11	GLB1L	pink	0.926759732
ENSG00000149328.10	GLB1L2	pink	0.922872729
ENSG00000166105.11	GLB1L3	turquoise	0.907301696
ENSG00000106415.8	GLCCI1	turquoise	0.899091214
ENSG00000138604.5	GLCE	turquoise	0.905736817
ENSG00000178445.8	GLDC	green	0.893256743
ENSG00000186417.9	GLDN	brown	0.860795708
ENSG00000119392.10	GLE1	turquoise	0.970699424
ENSG00000090863.7	GLG1	turquoise	0.948861562
ENSG00000106571.8	GLI3	green	0.759441007
ENSG00000250571.2	GLI4	turquoise	0.790883841
ENSG00000139278.5	GLIPR1	turquoise	0.824735304
ENSG00000180481.6	GLIPR1L2	blue	0.448813645
ENSG00000122694.11	GLIPR2	brown	0.782063539
ENSG00000174332.3	GLIS1	turquoise	0.711917075
ENSG00000126603.4	GLIS2	blue	0.644818246
ENSG00000107249.17	GLIS3	blue	0.646655276
ENSG00000174842.12	GLMN	turquoise	0.941436774
ENSG00000124767.6	GLO1	turquoise	0.933938762
ENSG00000167699.9	GLOD4	turquoise	0.964430561
ENSG00000112164.5	GLP1R	turquoise	0.622472917
ENSG00000101958.9	GLRA2	turquoise	0.747624872
ENSG00000109738.6	GLRB	turquoise	0.949413252
ENSG00000173221.9	GLRX	cyan	0.847083327
ENSG00000023572.4	GLRX2	turquoise	0.892707446
ENSG00000108010.7	GLRX3	turquoise	0.958443684
ENSG00000182512.4	GLRX5	turquoise	0.921343168
ENSG00000115419.8	GLS	turquoise	0.963047756
ENSG00000135423.8	GLS2	turquoise	0.922763142
ENSG00000151948.7	GLT1D1	turquoise	0.921806803
ENSG00000016864.12	GLT8D1	turquoise	0.96553487
ENSG00000120820.8	GLT8D2	turquoise	0.890098265
ENSG00000139433.5	GLTP	brown	0.87634255
ENSG00000224051.2	GLTPD1	turquoise	0.852238533
ENSG00000182327.7	GLTPD2	turquoise	0.809933551
ENSG00000063169.6	GLTSCR1	turquoise	0.863767466
ENSG00000112624.8	GLTSCR1L	turquoise	0.935830088
ENSG00000105373.14	GLTSCR2	red	0.923399908
ENSG00000148672.7	GLUD1	green	0.864616407
ENSG00000250959.2	GLUD1P3	turquoise	0.845869791
ENSG00000135821.12	GLUL	green	0.792551751
ENSG00000168237.13	GLYCTK	blue	0.655082453
ENSG00000140632.12	GLYR1	turquoise	0.940218805

ENSG00000196743.4	GM2A	brown	0.857450811
ENSG00000087338.4	GMCL1	turquoise	0.875649189
ENSG00000112699.6	GMDS	turquoise	0.917529543
ENSG00000162419.8	GMEB1	turquoise	0.910858855
ENSG00000101216.6	GMEB2	turquoise	0.930047449
ENSG00000197045.8	GMFB	blue	0.750037884
ENSG00000130755.8	GMFG	red	0.738361274
ENSG00000089639.6	GMIP	turquoise	0.730182516
ENSG00000112312.5	GMNN	green	0.626603689
ENSG00000144591.13	GMPPA	blue	0.855443408
ENSG00000173540.8	GMPPB	blue	0.576933314
ENSG00000100938.13	GMPR2	turquoise	0.971974124
ENSG00000163655.11	GMPS	turquoise	0.96708038
ENSG00000088256.4	GNA11	turquoise	0.936317507
ENSG00000146535.9	GNA12	blue	0.761181555
ENSG00000120063.5	GNA13	green	0.798539916
ENSG00000156049.6	GNA14	green	0.733049189
ENSG00000060558.3	GNA15	magenta	0.879792734
ENSG00000127955.11	GNAI1	brown	0.816963426
ENSG00000114353.12	GNAI2	blue	0.861987482
ENSG00000065135.7	GNAI3	blue	0.94375059
ENSG00000141404.11	GNAL	turquoise	0.910442471
ENSG00000087258.9	GNAO1	turquoise	0.904557174
ENSG00000156052.6	GNAQ	turquoise	0.940336699
ENSG00000087460.19	GNAS	greenyellow	0.887910165
ENSG00000128266.7	GNAZ	turquoise	0.90043996
ENSG00000078369.13	GNB1	turquoise	0.958775441
ENSG00000185838.9	GNB1L	turquoise	0.860371278
ENSG00000172354.5	GNB2	turquoise	0.909366425
ENSG00000204628.7	GNB2L1	red	0.922290398
ENSG00000114450.5	GNB4	blue	0.75985983
ENSG00000069966.14	GNB5	turquoise	0.941433888
ENSG00000159921.10	GNE	turquoise	0.932717594
ENSG00000242616.3	GNG10	turquoise	0.891973124
ENSG00000127920.5	GNG11	black	0.556104823
ENSG00000172380.5	GNG12	green	0.872310911
ENSG00000186469.4	GNG2	greenyellow	0.879567477
ENSG00000162188.5	GNG3	turquoise	0.925630033
ENSG00000168243.6	GNG4	turquoise	0.861069077
ENSG00000174021.6	GNG5	turquoise	0.723498288
ENSG00000176533.8	GNG7	turquoise	0.773605788
ENSG00000167414.4	GNG8	brown	0.531114901
ENSG00000204590.8	GNL1	turquoise	0.960954811
ENSG00000134697.8	GNL2	turquoise	0.841708338
ENSG00000163938.12	GNL3	turquoise	0.960200556
ENSG00000130119.11	GNL3L	blue	0.851116708
ENSG00000115523.12	GNLY	grey	0.354553149
ENSG00000124713.5	GNMT	turquoise	0.721426144

ENSG00000116906.7	GNPAT	turquoise	0.884766452
ENSG00000113552.11	GNPDA1	turquoise	0.970300127
ENSG00000163281.7	GNPDA2	turquoise	0.951554312
ENSG00000100522.4	GNPNAT1	turquoise	0.879308514
ENSG00000111670.10	GNPTAB	turquoise	0.947345157
ENSG00000090581.5	GNPTG	red	0.913909227
ENSG00000147437.5	GNRH1	brown	0.546063283
ENSG00000211451.7	GNRHR2	turquoise	0.905457458
ENSG00000135677.6	GNS	turquoise	0.875430928
ENSG00000136935.9	GOLGA1	turquoise	0.909561757
ENSG00000167110.12	GOLGA2	turquoise	0.963867331
ENSG00000090615.8	GOLGA3	turquoise	0.895076409
ENSG00000144674.12	GOLGA4	turquoise	0.911513717
ENSG00000066455.8	GOLGA5	turquoise	0.879302343
ENSG00000230373.4	GOLGA6L5	yellow	0.757548223
ENSG00000147533.12	GOLGA7	brown	0.893440886
ENSG00000269783.1	GOLGA7B	turquoise	0.721536255
ENSG00000155265.6	GOLGA7B	turquoise	0.775782457
ENSG00000175265.13	GOLGA8A	yellow	0.912220089
ENSG00000215252.7	GOLGA8B	turquoise	0.766128216
ENSG00000173230.11	GOLGB1	turquoise	0.934981851
ENSG00000173905.4	GOLIM4	green	0.910014946
ENSG00000135052.12	GOLM1	turquoise	0.840718486
ENSG00000113384.9	GOLPH3	turquoise	0.906408401
ENSG00000143457.6	GOLPH3L	turquoise	0.862500321
ENSG00000174567.7	GOLT1A	turquoise	0.781146369
ENSG00000111711.5	GOLT1B	turquoise	0.932635545
ENSG00000116580.14	GON4L	turquoise	0.915012095
ENSG00000047932.9	GOPC	turquoise	0.922518618
ENSG00000120370.8	GORAB	blue	0.885543585
ENSG00000114745.9	GORASP1	turquoise	0.959438174
ENSG00000115806.8	GORASP2	turquoise	0.972328149
ENSG00000108587.10	GOSR1	turquoise	0.956577413
ENSG00000108433.11	GOSR2	turquoise	0.922736247
ENSG00000120053.9	GOT1	turquoise	0.957343692
ENSG00000125166.8	GOT2	turquoise	0.977754275
ENSG00000088053.7	GP6	turquoise	0.744409683
ENSG00000197858.6	GPAA1	turquoise	0.921482856
ENSG00000133114.13	GPALPP1	turquoise	0.964435509
ENSG00000119927.9	GPAM	green	0.784612517
ENSG00000204438.6	GPANK1	turquoise	0.860345486
ENSG00000186281.8	GPAT2	turquoise	0.718400081
ENSG00000076650.2	GPATCH1	turquoise	0.96913574
ENSG00000152133.10	GPATCH11	turquoise	0.954760763
ENSG00000092978.6	GPATCH2	turquoise	0.92539545
ENSG00000089916.13	GPATCH2L	turquoise	0.924246937
ENSG00000198746.8	GPATCH3	brown	0.75687885
ENSG00000160818.12	GPATCH4	turquoise	0.902261594

ENSG00000062194.11	GPBP1	turquoise	0.911825514
ENSG00000159592.6	GPBP1L1	turquoise	0.949127108
ENSG00000063660.4	GPC1	turquoise	0.893523534
ENSG00000213420.3	GPC2	blue	0.673626103
ENSG00000147257.9	GPC3	grey	0.362720982
ENSG00000179399.9	GPC5	blue	0.703266007
ENSG00000183098.6	GPC6	blue	0.687525517
ENSG00000125772.8	GPCPD1	brown	0.666347991
ENSG00000167588.8	GPD1	brown	0.752819856
ENSG00000152642.6	GPD1L	turquoise	0.940751
ENSG00000115159.11	GPD2	turquoise	0.930891798
ENSG00000164850.10	GPER1	grey	0.362582366
ENSG00000171723.11	GPHN	turquoise	0.957798257
ENSG00000105220.10	GPI	turquoise	0.962324922
ENSG00000182851.2	GPIHBP1	brown	0.680532796
ENSG00000068394.9	GPKOW	turquoise	0.894904031
ENSG00000112293.10	GPLD1	turquoise	0.880305981
ENSG00000150625.12	GPM6A	blue	0.763304228
ENSG00000046653.10	GPM6B	blue	0.875254352
ENSG00000198522.9	GPN1	turquoise	0.919899322
ENSG00000142751.10	GPN2	turquoise	0.971899392
ENSG00000111231.4	GPN3	turquoise	0.896829907
ENSG00000136235.11	GPNMB	grey	0.389846842
ENSG00000183671.8	GPR1	turquoise	0.751781559
ENSG00000165370.1	GPR101	turquoise	0.721088385
ENSG00000148358.15	GPR107	turquoise	0.938328656
ENSG00000125734.9	GPR108	blue	0.870484507
ENSG00000069122.14	GPR116	brown	0.55223956
ENSG00000132975.6	GPR12	turquoise	0.894169129
ENSG00000197177.11	GPR123	greenyellow	0.794304573
ENSG00000020181.13	GPR124	brown	0.57256554
ENSG00000152990.9	GPR125	green	0.896553272
ENSG00000111452.8	GPR133	black	0.573322958
ENSG00000181619.11	GPR135	pink	0.940140496
ENSG00000173264.9	GPR137	turquoise	0.875099602
ENSG00000077585.9	GPR137B	blue	0.793178408
ENSG00000180998.7	GPR137C	turquoise	0.928994719
ENSG00000101850.8	GPR143	blue	0.664134359
ENSG00000164849.7	GPR146	black	0.636433396
ENSG00000174948.5	GPR149	turquoise	0.840345196
ENSG00000158292.6	GPR153	turquoise	0.769981129
ENSG00000163328.9	GPR155	turquoise	0.93740535
ENSG00000151025.9	GPR158	turquoise	0.896182694
ENSG00000143147.10	GPR161	green	0.759575587
ENSG00000250510.3	GPR162	turquoise	0.865526357
ENSG00000144230.12	GPR17	grey	0.210705709
ENSG00000184194.5	GPR173	turquoise	0.937918681
ENSG00000166073.4	GPR176	turquoise	0.869764173

ENSG00000188888.7	GPR179	turquoise	0.864230978
ENSG00000152749.7	GPR180	turquoise	0.923023757
ENSG00000169508.6	GPR183	magenta	0.693758124
ENSG00000183150.3	GPR19	turquoise	0.844753357
ENSG00000188394.6	GPR21	turquoise	0.765987614
ENSG00000172209.4	GPR22	turquoise	0.801415809
ENSG00000170837.2	GPR27	turquoise	0.89734093
ENSG00000181773.6	GPR3	turquoise	0.41470129
ENSG00000171659.9	GPR34	magenta	0.811491551
ENSG00000178623.7	GPR35	yellow	0.789587619
ENSG00000170775.2	GPR37	brown	0.917395517
ENSG00000170075.8	GPR37L1	green	0.753656441
ENSG00000177464.4	GPR4	black	0.81011573
ENSG00000135973.1	GPR45	turquoise	0.820013202
ENSG00000203737.2	GPR52	turquoise	0.84016
ENSG00000135898.5	GPR55	turquoise	0.836457886
ENSG00000205336.7	GPR56	blue	0.609748577
ENSG00000146360.7	GPR6	turquoise	0.820588966
ENSG00000156097.8	GPR61	turquoise	0.903464767
ENSG00000180929.4	GPR62	brown	0.874174618
ENSG00000112218.7	GPR63	turquoise	0.781755172
ENSG00000119737.5	GPR75	green	0.86679559
ENSG00000115239.17	GPR75-ASB3	green	0.883936544
ENSG00000123901.4	GPR83	turquoise	0.811413018
ENSG00000164604.8	GPR85	grey	0.426571491
ENSG00000181656.6	GPR88	turquoise	0.76304886
ENSG00000117262.14	GPR89A	turquoise	0.785240977
ENSG00000164199.11	GPR98	green	0.733218011
ENSG00000198932.8	GPRASP1	turquoise	0.95801976
ENSG00000158301.14	GPRASP2	turquoise	0.961132484
ENSG00000167191.7	GPRC5B	brown	0.825409814
ENSG00000170412.12	GPRC5C	green	0.681217797
ENSG00000169258.6	GPRIN1	turquoise	0.840948083
ENSG00000185477.4	GPRIN3	turquoise	0.803248513
ENSG00000169727.8	GPS1	turquoise	0.932383923
ENSG00000132522.11	GPS2	turquoise	0.869278557
ENSG00000160360.7	GPSM1	turquoise	0.812739639
ENSG00000121957.8	GPSM2	blue	0.713877705
ENSG00000213654.5	GPSM3	magenta	0.919429704
ENSG00000167701.9	GPT	green	0.658011482
ENSG00000166123.9	GPT2	green	0.891291585
ENSG00000233276.2	GPX1	red	0.741202798
ENSG00000197582.5	GPX1P1	grey	0.252221495
ENSG00000211445.7	GPX3	turquoise	0.730286291
ENSG00000167468.12	GPX4	red	0.939058807
ENSG00000116157.5	GPX7	blue	0.845911756
ENSG00000089351.10	GRAMD1A	turquoise	0.851269325
ENSG00000023171.10	GRAMD1B	turquoise	0.929053577

ENSG00000178075.15	GRAMD1C	green	0.841965528
ENSG00000175318.7	GRAMD2	pink	0.915951521
ENSG00000155324.5	GRAMD3	green	0.86744768
ENSG00000075240.12	GRAMD4	turquoise	0.72551857
ENSG00000161835.6	GRASP	turquoise	0.77809782
ENSG00000106070.13	GRB10	turquoise	0.941021898
ENSG00000115290.5	GRB14	brown	0.691946658
ENSG00000177885.9	GRB2	turquoise	0.976975739
ENSG00000196208.9	GREB1	turquoise	0.911405518
ENSG00000141449.10	GREB1L	turquoise	0.815878849
ENSG00000166923.6	GREM1	brown	0.733530098
ENSG00000180875.4	GREM2	turquoise	0.817816661
ENSG00000137106.13	GRHPR	turquoise	0.86818072
ENSG00000155511.13	GRIA1	turquoise	0.911956815
ENSG00000120251.14	GRIA2	turquoise	0.925423679
ENSG00000125675.13	GRIA3	turquoise	0.919606469
ENSG00000152578.8	GRIA4	greenyellow	0.934246367
ENSG00000182771.13	GRID1	turquoise	0.937794637
ENSG00000152208.7	GRID2	turquoise	0.801099337
ENSG00000171189.12	GRIK1	greenyellow	0.828550571
ENSG00000164418.15	GRIK2	turquoise	0.895540533
ENSG00000163873.5	GRIK3	greenyellow	0.874590796
ENSG00000149403.7	GRIK4	midnightblue	0.923622768
ENSG00000105737.5	GRIK5	turquoise	0.914246654
ENSG00000176884.10	GRIN1	turquoise	0.856466764
ENSG00000183454.9	GRIN2A	turquoise	0.805001795
ENSG00000273079.1	GRIN2B	turquoise	0.89094908
ENSG00000161509.9	GRIN2C	green	0.657110783
ENSG00000105464.3	GRIN2D	greenyellow	0.856420786
ENSG00000178719.12	GRINA	turquoise	0.927025405
ENSG00000155974.7	GRIP1	turquoise	0.899353859
ENSG00000144596.7	GRIP2	greenyellow	0.886795811
ENSG00000068400.9	GRIPAP1	turquoise	0.967906273
ENSG00000125388.15	GRK4	turquoise	0.922899434
ENSG00000198873.10	GRK5	turquoise	0.890956618
ENSG00000198055.6	GRK6	turquoise	0.949986161
ENSG00000152822.9	GRM1	turquoise	0.846613078
ENSG00000164082.10	GRM2	greenyellow	0.718213301
ENSG00000198822.6	GRM3	midnightblue	0.785499883
ENSG00000124493.9	GRM4	turquoise	0.84546604
ENSG00000168959.10	GRM5	turquoise	0.796877798
ENSG00000196277.11	GRM7	turquoise	0.863837967
ENSG00000179603.13	GRM8	turquoise	0.705030688
ENSG00000030582.12	GRN	blue	0.858893294
ENSG00000109519.8	GRPEL1	turquoise	0.940627302
ENSG00000164284.10	GRPEL2	turquoise	0.896396681
ENSG00000132463.9	GRSF1	turquoise	0.952961149
ENSG00000139835.9	GRTP1	green	0.816904288

ENSG00000105447.8	GRWD1	turquoise	0.897791731
ENSG00000229180.4	GS1-124K5.11	yellow	0.923420728
ENSG00000232559.2	GS1-124K5.12	turquoise	0.924993914
ENSG00000230189.2	GS1-124K5.2	blue	0.744120758
ENSG00000237310.1	GS1-124K5.4	turquoise	0.810388428
ENSG00000251451.1	GS1-124K5.6	blue	0.680682365
ENSG00000272361.1	GS1-166A23.2	turquoise	0.614912635
ENSG00000224223.1	GS1-18A18.1	turquoise	0.822477192
ENSG00000253738.1	GS1-251I9.4	turquoise	0.934291516
ENSG00000244219.2	GS1-259H13.2	turquoise	0.699074961
ENSG00000233196.2	GS1-304P7.1	turquoise	0.682178216
ENSG00000260822.1	GS1-358P8.4	turquoise	0.949999216
ENSG00000254690.1	GS1-393G12.12	grey	0.780945278
ENSG00000254239.1	GS1-39E22.2	grey	0.265970667
ENSG00000272163.1	GS1-72M22.1	turquoise	0.90671949
ENSG00000186088.11	GSAP	yellow	0.811909224
ENSG00000073605.14	GSDMB	grey	0.823302757
ENSG00000104518.6	GSDMD	magenta	0.711943563
ENSG00000131149.13	GSE1	turquoise	0.920560865
ENSG00000169181.8	GSG1L	turquoise	0.905452283
ENSG00000105723.7	GSK3A	turquoise	0.961147999
ENSG00000082701.10	GSK3B	turquoise	0.965738254
ENSG00000100744.10	GSKIP	turquoise	0.942493634
ENSG00000148180.12	GSN	brown	0.819301
ENSG00000103342.8	GSPT1	turquoise	0.91119397
ENSG00000189369.7	GSPT2	turquoise	0.949926867
ENSG00000104687.8	GSR	turquoise	0.961015606
ENSG00000100983.5	GSS	turquoise	0.945526672
ENSG00000170899.6	GSTA4	turquoise	0.96183056
ENSG00000138780.10	GSTCD	turquoise	0.868060099
ENSG00000197448.9	GSTK1	turquoise	0.920368589
ENSG00000134184.8	GSTM1	grey	0.065986072
ENSG00000213366.8	GSTM2	turquoise	0.912812324
ENSG00000134202.6	GSTM3	turquoise	0.822512421
ENSG00000227693.1	GSTM3P1	brown	0.819121504
ENSG00000168765.11	GSTM4	turquoise	0.914928669
ENSG00000134201.6	GSTM5	grey	0.278883136
ENSG00000148834.8	GSTO1	turquoise	0.915901745
ENSG00000065621.10	GSTO2	turquoise	0.91139393
ENSG00000084207.11	GSTP1	black	0.596209268
ENSG00000184674.8	GSTT1	grey	0.161679107
ENSG00000099984.6	GSTT2	grey	0.273919628
ENSG00000133433.6	GSTT2B	grey	0.217119686
ENSG00000100577.14	GSTZ1	brown	0.699005822
ENSG00000121964.10	GTDC1	brown	0.818857366
ENSG00000165417.7	GTF2A1	turquoise	0.919919841
ENSG00000140307.6	GTF2A2	turquoise	0.907383377
ENSG00000137947.7	GTF2B	turquoise	0.950340444

ENSG00000153767.5	GTF2E1	blue	0.89014041
ENSG00000197265.4	GTF2E2	turquoise	0.893106501
ENSG00000125651.9	GTF2F1	turquoise	0.956559377
ENSG00000188342.7	GTF2F2	turquoise	0.911275798
ENSG00000110768.7	GTF2H1	turquoise	0.961933154
ENSG00000111358.9	GTF2H3	turquoise	0.925451279
ENSG00000213780.6	GTF2H4	turquoise	0.934389377
ENSG00000272047.1	GTF2H5	turquoise	0.945673872
ENSG00000006704.6	GTF2IRD1	turquoise	0.930734072
ENSG00000196275.9	GTF2IRD2	blue	0.746234938
ENSG00000174428.12	GTF2IRD2B	blue	0.783653852
ENSG00000122034.8	GTF3A	turquoise	0.906710808
ENSG00000077235.13	GTF3C1	turquoise	0.938585293
ENSG00000115207.9	GTF3C2	turquoise	0.973300098
ENSG00000119041.6	GTF3C3	turquoise	0.916021491
ENSG00000125484.7	GTF3C4	turquoise	0.944963149
ENSG00000148308.13	GTF3C5	turquoise	0.912088051
ENSG00000155115.6	GTF3C6	turquoise	0.789984055
ENSG00000100226.11	GTPBP1	turquoise	0.921553939
ENSG00000105793.11	GTPBP10	turquoise	0.930116859
ENSG00000172432.14	GTPBP2	turquoise	0.907058734
ENSG00000130299.12	GTPBP3	turquoise	0.892524683
ENSG00000107937.14	GTPBP4	turquoise	0.934970601
ENSG00000178605.8	GTPBP6	turquoise	0.824660866
ENSG00000163607.10	GTPBP8	turquoise	0.913813948
ENSG00000048545.9	GUCA1A	turquoise	0.798628389
ENSG00000112599.8	GUCA1B	yellow	0.786516014
ENSG00000138867.12	GUCD1	turquoise	0.783756557
ENSG00000152402.6	GUCY1A2	turquoise	0.829246744
ENSG00000164116.12	GUCY1A3	turquoise	0.883009597
ENSG00000061918.8	GUCY1B3	turquoise	0.942663819
ENSG00000151806.9	GUF1	turquoise	0.950281413
ENSG00000143774.12	GUK1	red	0.923745427
ENSG00000144366.11	GULP1	turquoise	0.853695587
ENSG00000169919.12	GUSB	yellow	0.91643846
ENSG00000183666.12	GUSBP1	turquoise	0.743208885
ENSG00000253203.2	GUSBP3	brown	0.644158241
ENSG00000241043.1	GVQW1	turquoise	0.651812238
ENSG00000151233.6	GXYLT1	blue	0.879737838
ENSG00000172986.8	GXYLT2	blue	0.759448477
ENSG00000163754.13	GYG1	turquoise	0.811004011
ENSG00000206159.6	GYG2P1	grey	0.281761517
ENSG00000136732.10	GYPC	magenta	0.81153052
ENSG00000104812.10	GYS1	blue	0.83179621
ENSG00000125812.11	GZF1	turquoise	0.847494333
ENSG00000197540.3	GZMM	grey	0.378347436
ENSG00000130600.11	H19	turquoise	0.509913455
ENSG00000189060.4	H1F0	brown	0.708713932

ENSG00000184897.4	H1FX	turquoise	0.821086579
ENSG00000206417.4	H1FX-AS1	yellow	0.785753713
ENSG00000246705.3	H2AFJ	turquoise	0.912419429
ENSG00000105968.14	H2AFV	turquoise	0.915994664
ENSG00000188486.3	H2AFX	blue	0.714753768
ENSG00000113648.12	H2AFY	turquoise	0.983849797
ENSG00000099284.9	H2AFY2	turquoise	0.92318961
ENSG00000164032.7	H2AFZ	turquoise	0.940101425
ENSG00000163041.5	H3F3A	blue	0.894057713
ENSG00000235655.2	H3F3AP4	blue	0.596911808
ENSG00000132475.4	H3F3B	blue	0.844892404
ENSG00000049239.8	H6PD	turquoise	0.832279052
ENSG00000130956.9	HABP4	turquoise	0.920631337
ENSG00000085382.7	HACE1	turquoise	0.954921096
ENSG00000131373.10	HACL1	turquoise	0.910306241
ENSG00000138796.11	HADH	blue	0.908959478
ENSG00000084754.6	HADHA	blue	0.879035345
ENSG00000138029.9	HADHB	green	0.878310556
ENSG00000063854.8	HAGH	turquoise	0.927032379
ENSG00000103253.13	HAGHL	midnightblue	0.788283482
ENSG00000105697.3	HAMP	magenta	0.78291688
ENSG00000173805.11	HAP1	blue	0.559994985
ENSG00000145681.6	HAPLN1	greenyellow	0.703144128
ENSG00000132702.8	HAPLN2	brown	0.846438794
ENSG00000140511.7	HAPLN3	black	0.73340285
ENSG00000187664.8	HAPLN4	greenyellow	0.847619734
ENSG00000225978.2	HAR1A	turquoise	0.853075857
ENSG00000231133.2	HAR1B	turquoise	0.830039923
ENSG00000180423.4	HARBI1	turquoise	0.793501532
ENSG00000170445.8	HARS	turquoise	0.970989878
ENSG00000112855.10	HARS2	turquoise	0.944439463
ENSG00000105509.6	HAS1	turquoise	0.820854652
ENSG00000103044.6	HAS3	turquoise	0.780307322
ENSG00000128708.8	HAT1	turquoise	0.755175064
ENSG00000152240.8	HAUS1	turquoise	0.858112656
ENSG00000137814.5	HAUS2	turquoise	0.893563768
ENSG00000214367.3	HAUS3	turquoise	0.888479991
ENSG00000092036.12	HAUS4	turquoise	0.821211235
ENSG00000249115.4	HAUS5	yellow	0.915589455
ENSG00000147874.6	HAUS6	turquoise	0.848093079
ENSG00000213397.6	HAUS7	blue	0.578311084
ENSG00000131351.10	HAUS8	turquoise	0.801470609
ENSG00000268442.1	HAVCR1P1	blue	0.550761441
ENSG00000135077.4	HAVCR2	magenta	0.943117601
ENSG00000143575.10	HAX1	turquoise	0.942590986
ENSG00000206172.4	HBA1	grey	0.011890686
ENSG00000188536.8	HBA2	grey	-0.02380906
ENSG00000244734.2	HBB	grey	-0.016766324

ENSG00000223609.3	HBD	grey	-0.106169269
ENSG00000113070.6	HBEGF	blue	0.654775808
ENSG00000105856.9	HBP1	blue	0.910600886
ENSG00000086506.2	HBQ1	turquoise	0.645378593
ENSG00000112339.10	HBS1L	turquoise	0.827332816
ENSG00000269235.1	HCCAT3	turquoise	0.82650816
ENSG00000004961.10	HCCS	turquoise	0.952855
ENSG00000172534.9	HCFC1	turquoise	0.926641959
ENSG00000103145.6	HCFC1R1	turquoise	0.818229727
ENSG00000111727.7	HCFC2	turquoise	0.874391104
ENSG00000228223.1	HCG11	blue	0.810815963
ENSG00000270604.1	HCG17	turquoise	0.745508563
ENSG00000231074.4	HCG18	turquoise	0.881988329
ENSG00000228962.1	HCG23	turquoise	0.619755504
ENSG00000230313.1	HCG24	turquoise	0.538909404
ENSG00000206344.6	HCG27	yellow	0.624786146
ENSG00000176998.3	HCG4	turquoise	0.865202806
ENSG00000237669.1	HCG4P3	turquoise	0.505444112
ENSG00000101336.8	HCK	magenta	0.934293752
ENSG00000180353.6	HCLS1	magenta	0.94291677
ENSG00000164588.4	HCN1	turquoise	0.783150908
ENSG00000099822.2	HCN2	turquoise	0.852399182
ENSG00000143630.5	HCN3	turquoise	0.870764459
ENSG00000206337.6	HCP5	black	0.702359188
ENSG00000126264.5	HCST	magenta	0.890032687
ENSG00000116478.7	HDAC1	blue	0.897181114
ENSG00000100429.13	HDAC10	turquoise	0.529031283
ENSG00000163517.10	HDAC11	brown	0.830792939
ENSG00000196591.7	HDAC2	turquoise	0.968597309
ENSG00000171720.5	HDAC3	turquoise	0.971677854
ENSG00000068024.12	HDAC4	blue	0.893230837
ENSG00000108840.11	HDAC5	turquoise	0.86436725
ENSG00000094631.14	HDAC6	turquoise	0.865048289
ENSG00000061273.13	HDAC7	yellow	0.874730372
ENSG00000147099.15	HDAC8	turquoise	0.932576893
ENSG00000048052.17	HDAC9	greenyellow	0.862237976
ENSG00000111906.13	HDDC2	red	0.856328138
ENSG00000184508.6	HDDC3	turquoise	0.887192904
ENSG00000143321.14	HDGF	turquoise	0.86424017
ENSG00000167674.10	HDGFRP2	turquoise	0.930718015
ENSG00000166503.4	HDGFRP3	turquoise	0.906851999
ENSG00000130021.9	HDHD1	turquoise	0.865034316
ENSG00000167220.7	HDHD2	blue	0.883391003
ENSG00000119431.5	HDHD3	turquoise	0.880278548
ENSG00000115677.12	HDLBP	turquoise	0.948076619
ENSG00000165259.9	HDX	turquoise	0.902696545
ENSG00000119285.6	HEATR1	turquoise	0.894744552
ENSG00000164818.11	HEATR2	pink	0.884818477

ENSG00000155393.8	HEATR3	turquoise	0.94728863
ENSG00000129493.10	HEATR5A	blue	0.840402975
ENSG00000008869.7	HEATR5B	turquoise	0.973583268
ENSG00000068097.10	HEATR6	turquoise	0.937589733
ENSG00000013583.4	HEBP1	turquoise	0.911880276
ENSG00000051620.6	HEBP2	black	0.754669587
ENSG00000112406.4	HECA	turquoise	0.735499066
ENSG00000092148.8	HECTD1	turquoise	0.969973441
ENSG00000165338.12	HECTD2	turquoise	0.931532595
ENSG00000126107.10	HECTD3	turquoise	0.948607877
ENSG00000173064.6	HECTD4	turquoise	0.965654612
ENSG00000002746.10	HECW1	turquoise	0.900128539
ENSG00000138411.6	HECW2	turquoise	0.874154923
ENSG00000173706.8	HEG1	brown	0.64690097
ENSG00000163312.6	HELQ	turquoise	0.938882679
ENSG00000198265.7	HELZ	turquoise	0.91889759
ENSG00000130589.12	HELZ2	black	0.845610529
ENSG00000114735.5	HEMK1	turquoise	0.870170207
ENSG00000162639.11	HENMT1	turquoise	0.943331569
ENSG00000165478.6	HEPACAM	green	0.90940785
ENSG00000089472.12	HEPH	green	0.90874585
ENSG00000103657.9	HERC1	turquoise	0.973533585
ENSG00000128731.11	HERC2	turquoise	0.959213677
ENSG00000140181.10	HERC2P2	yellow	0.647581627
ENSG00000180229.8	HERC2P3	grey	0.422188271
ENSG00000206149.6	HERC2P9	yellow	0.719843767
ENSG00000138641.11	HERC3	turquoise	0.954203845
ENSG00000148634.11	HERC4	blue	0.872982161
ENSG00000138646.4	HERC5	blue	0.780262432
ENSG00000138642.10	HERC6	turquoise	0.787957469
ENSG00000051108.10	HERPUD1	cyan	0.826720292
ENSG00000122557.5	HERPUD2	blue	0.895783268
ENSG00000114315.3	HES1	grey	0.288827114
ENSG00000188290.6	HES4	turquoise	0.62539405
ENSG00000197921.5	HES5	grey	0.103448647
ENSG00000144485.6	HES6	brown	0.477354518
ENSG00000163666.4	HESX1	blue	0.570062851
ENSG00000213614.5	HEXA	turquoise	0.935147679
ENSG00000049860.9	HEXB	turquoise	0.957639005
ENSG00000169660.11	HEXDC	turquoise	0.870259964
ENSG00000186834.2	HEXIM1	turquoise	0.88547771
ENSG00000168517.6	HEXIM2	turquoise	0.868534685
ENSG00000164683.12	HEY1	turquoise	0.923077197
ENSG00000135547.4	HEY2	brown	0.638748077
ENSG00000163909.6	HEYL	green	0.579661893
ENSG00000010704.14	HFE	blue	0.760590768
ENSG00000113924.7	HGD	grey	0.301585452
ENSG00000109758.4	HGFAC	grey	0.670581421

ENSG00000185359.8	HGS	turquoise	0.944025266
ENSG00000165102.10	HGSNAT	turquoise	0.915619986
ENSG00000054392.8	HHAT	turquoise	0.805865981
ENSG00000010282.10	HHATL	brown	0.643613801
ENSG00000230970.2	HHATL-AS1	blue	0.539750944
ENSG00000152804.6	HHEX	magenta	0.615758729
ENSG00000164161.5	HHIP	brown	0.882422435
ENSG00000248890.1	HHIP-AS1	turquoise	0.670624277
ENSG00000182218.5	HHIPL1	green	0.755056797
ENSG00000197568.9	HHLA3	turquoise	0.785267064
ENSG00000156875.9	HIAT1	turquoise	0.937986991
ENSG00000148110.11	HIATL1	turquoise	0.908899139
ENSG00000196312.7	HIATL2	turquoise	0.901438136
ENSG00000106049.4	HIBADH	blue	0.891870527
ENSG00000198130.10	HIBCH	blue	0.796963434
ENSG00000177374.8	HIC1	grey	0.360774946
ENSG00000169635.5	HIC2	yellow	0.87325203
ENSG00000167861.11	HID1	yellow	0.912620107
ENSG00000100644.12	HIF1A	blue	0.855073337
ENSG00000166135.9	HIF1AN	turquoise	0.950167499
ENSG00000124440.11	HIF3A	green	0.553649169
ENSG00000181061.9	HIGD1A	blue	0.811221844
ENSG00000131097.2	HIGD1B	grey	0.127186579
ENSG00000146066.2	HIGD2A	red	0.945484191
ENSG00000135245.9	HILPDA	black	0.712146255
ENSG00000188662.5	HILS1	turquoise	0.635342011
ENSG00000172273.8	HINFP	turquoise	0.965001701
ENSG00000169567.7	HINT1	turquoise	0.935945174
ENSG00000137133.6	HINT2	red	0.906088725
ENSG00000111911.5	HINT3	brown	0.821145481
ENSG00000127946.12	HIP1	brown	0.912470592
ENSG00000130787.9	HIP1R	brown	0.800591567
ENSG00000064393.11	HIPK2	brown	0.574848157
ENSG00000110422.7	HIPK3	blue	0.878181286
ENSG00000160396.8	HIPK4	turquoise	0.838483182
ENSG00000100084.10	HIRA	turquoise	0.914898465
ENSG00000149929.11	HIRIP3	brown	0.764056658
ENSG00000187837.2	HIST1H1C	black	0.542797599
ENSG00000180573.8	HIST1H2AC	blue	0.698502727
ENSG00000168274.3	HIST1H2AE	magenta	0.661401288
ENSG00000184348.2	HIST1H2AK	turquoise	0.726895928
ENSG00000180596.7	HIST1H2BC	blue	0.657902691
ENSG00000158373.7	HIST1H2BD	turquoise	0.670541591
ENSG00000187990.4	HIST1H2BG	magenta	0.677731435
ENSG00000197903.6	HIST1H2BK	turquoise	0.876005198
ENSG00000198518.5	HIST1H4E	brown	0.504914873
ENSG00000158406.2	HIST1H4H	red	0.691166662
ENSG00000184678.8	HIST2H2BE	blue	0.717443651

ENSG00000181218.4	HIST3H2A	turquoise	0.896675438
ENSG00000196890.3	HIST3H2BB	turquoise	0.759206972
ENSG00000197837.3	HIST4H4	turquoise	0.879081307
ENSG00000095951.12	HIVEP1	blue	0.89790088
ENSG00000010818.4	HIVEP2	turquoise	0.918195611
ENSG00000127124.9	HIVEP3	turquoise	0.807841641
ENSG00000156515.17	HK1	turquoise	0.948735122
ENSG00000159399.5	HK2	magenta	0.835912543
ENSG00000181666.13	HKR1	turquoise	0.960551046
ENSG00000206503.7	HLA-A	blue	0.727099737
ENSG00000234745.5	HLA-B	black	0.829544388
ENSG00000204525.10	HLA-C	black	0.757524191
ENSG00000204257.10	HLA-DMA	magenta	0.886641213
ENSG00000242574.4	HLA-DMB	magenta	0.883393556
ENSG00000204252.8	HLA-DOA	magenta	0.866962645
ENSG00000241106.2	HLA-DOB	grey	0.412678782
ENSG00000231389.3	HLA-DPA1	magenta	0.909349247
ENSG00000223865.6	HLA-DPB1	magenta	0.884385406
ENSG00000196735.7	HLA-DQA1	magenta	0.832442671
ENSG00000237541.3	HLA-DQA2	magenta	0.57341818
ENSG00000179344.12	HLA-DQB1	magenta	0.696302107
ENSG00000204287.9	HLA-DRA	magenta	0.917517312
ENSG00000196126.6	HLA-DRB1	magenta	0.864938858
ENSG00000198502.5	HLA-DRB5	magenta	0.689184866
ENSG00000229391.3	HLA-DRB6	magenta	0.717527773
ENSG00000204592.5	HLA-E	black	0.900048823
ENSG00000204642.9	HLA-F	yellow	0.867258987
ENSG00000214922.5	HLA-F-AS1	turquoise	0.776581376
ENSG00000206341.6	HLA-H	blue	0.641763012
ENSG00000204622.6	HLA-J	blue	0.575598239
ENSG00000230795.2	HLA-K	grey	0.318575677
ENSG00000243753.1	HLA-L	turquoise	0.766891053
ENSG00000228078.1	HLA-U	grey	0.254040323
ENSG00000159267.10	HLCS	turquoise	0.961944005
ENSG00000108924.9	HLF	turquoise	0.898740243
ENSG00000071794.11	HLTF	turquoise	0.913399187
ENSG00000101294.12	HM13	turquoise	0.895418976
ENSG00000147421.13	HMBOX1	blue	0.780903416
ENSG00000256269.2	HMBS	red	0.849082708
ENSG00000183624.9	HMCES	turquoise	0.951386047
ENSG00000140382.10	HMG20A	turquoise	0.97036969
ENSG00000064961.14	HMG20B	brown	0.675233089
ENSG00000137309.15	HMGA1	turquoise	0.89471901
ENSG00000189403.10	HMGB1	blue	0.882040307
ENSG00000213707.2	HMGB1P10	blue	0.468447407
ENSG00000132967.9	HMGB1P5	blue	0.567528944
ENSG00000164104.7	HMGB2	green	0.663745074
ENSG00000029993.10	HMGB3	turquoise	0.735117272

ENSG00000225051.4	HMGB3P22	red	0.571515075
ENSG00000215283.3	HMGB3P24	turquoise	0.645372391
ENSG00000117305.10	HMGCL	blue	0.837745381
ENSG00000146151.8	HMGCLL1	turquoise	0.890489722
ENSG00000113161.11	HMGCR	turquoise	0.950259854
ENSG00000112972.10	HMGCS1	turquoise	0.887222964
ENSG00000205581.6	HMGN1	blue	0.874805521
ENSG00000258967.1	HMGN1P3	turquoise	0.748757629
ENSG00000198830.6	HMGN2	blue	0.88044739
ENSG00000214578.4	HMGN2P15	blue	0.536525153
ENSG00000230330.1	HMGN2P3	blue	0.52346578
ENSG00000249014.2	HMGN2P4	grey	0.505864595
ENSG00000234664.1	HMGN2P5	turquoise	0.730466324
ENSG00000118418.10	HMGN3	turquoise	0.863809641
ENSG00000270362.1	HMGN3-AS1	turquoise	0.859765498
ENSG00000182952.4	HMGN4	turquoise	0.943592682
ENSG00000198157.6	HMGN5	blue	0.638548694
ENSG00000113716.8	HMGXB3	turquoise	0.963803075
ENSG00000100281.9	HMGXB4	turquoise	0.886346218
ENSG00000180448.6	HMHA1	turquoise	0.783958871
ENSG00000100292.12	HMOX1	black	0.767043884
ENSG00000103415.7	HMOX2	turquoise	0.94272744
ENSG00000215612.5	HMX1	grey	0.234751328
ENSG00000189159.11	HN1	turquoise	0.939738531
ENSG00000206053.8	HN1L	blue	0.726376999
ENSG00000150540.9	HNMT	blue	0.769793974
ENSG00000177733.4	HNRNPA0	turquoise	0.963496136
ENSG00000135486.13	HNRNPA1	turquoise	0.907811701
ENSG00000139675.10	HNRNPA1L2	turquoise	0.909994832
ENSG00000262333.1	HNRNPA1P16	yellow	0.781115995
ENSG00000233680.4	HNRNPA1P27	turquoise	0.656916367
ENSG00000224578.3	HNRNPA1P48	red	0.749232319
ENSG00000122566.16	HNRNPA2B1	turquoise	0.927628752
ENSG00000170144.14	HNRNPA3	turquoise	0.930933116
ENSG00000197451.6	HNRNPAB	turquoise	0.859653654
ENSG00000092199.13	HNRNPC	turquoise	0.931219072
ENSG00000204253.3	HNRNPCP2	blue	0.735251416
ENSG00000138668.14	HNRNPD	turquoise	0.956518155
ENSG00000152795.13	HNRNPDL	turquoise	0.958066412
ENSG00000169813.12	HNRNPF	blue	0.870491217
ENSG00000169045.13	HNRNPH1	turquoise	0.901079459
ENSG00000126945.8	HNRNPH2	turquoise	0.976245482
ENSG00000096746.13	HNRNPH3	turquoise	0.880085464
ENSG00000165119.14	HNRNPK	turquoise	0.957704134
ENSG00000104824.12	HNRNPL	turquoise	0.955151324
ENSG00000143889.11	HNRNPLL	turquoise	0.94978359
ENSG00000099783.7	HNRNPM	turquoise	0.878024459
ENSG00000125944.14	HNRNPR	turquoise	0.945915762

ENSG00000153187.12	HNRNPU	turquoise	0.944474219
ENSG00000188206.5	HNRNPU-AS1	turquoise	0.69461149
ENSG00000105323.12	HNRNPUL1	turquoise	0.877796862
ENSG00000214753.2	HNRNPUL2	turquoise	0.962833074
ENSG00000241935.4	HOGA1	green	0.721281055
ENSG00000152413.10	HOMER1	turquoise	0.891445675
ENSG00000103942.8	HOMER2	blue	0.836254443
ENSG00000051128.14	HOMER3	brown	0.599904281
ENSG00000215271.6	HOMEZ	blue	0.907534024
ENSG00000134709.6	HOOK1	turquoise	0.903931808
ENSG00000095066.7	HOOK2	yellow	0.948045381
ENSG00000168172.4	HOOK3	turquoise	0.902375573
ENSG00000171476.17	HOPX	red	0.656104643
ENSG00000224189.2	HOXD-AS1	brown	0.740168285
ENSG00000127483.13	HP1BP3	turquoise	0.907596633
ENSG00000121905.5	HPCA	turquoise	0.741120303
ENSG00000115756.8	HPCAL1	greenyellow	0.75064358
ENSG00000116983.8	HPCAL4	turquoise	0.854133257
ENSG00000186603.4	HPDL	green	0.518628229
ENSG00000163106.6	HPGDS	magenta	0.823282917
ENSG00000105707.9	HPN	brown	0.85401216
ENSG00000227392.1	HPN-AS1	brown	0.760437775
ENSG00000261701.2	HPR	grey	0.196764263
ENSG00000165704.10	HPRT1	turquoise	0.947380224
ENSG00000107521.14	HPS1	turquoise	0.959758593
ENSG00000163755.4	HPS3	turquoise	0.820154647
ENSG00000100099.16	HPS4	turquoise	0.964988153
ENSG00000110756.13	HPS5	blue	0.916841529
ENSG00000166189.6	HPS6	turquoise	0.943141984
ENSG00000172987.8	HPSE2	brown	0.479074331
ENSG00000110169.6	HPX	turquoise	0.69564137
ENSG00000168453.10	HR	brown	0.836118987
ENSG00000174775.12	HRAS	turquoise	0.836829093
ENSG00000127252.4	HRASLS	turquoise	0.864584003
ENSG00000168004.5	HRASLS5	blue	0.708587013
ENSG00000130528.7	HRC	grey	0.422188804
ENSG00000196196.2	HRCT1	grey	0.436392657
ENSG00000196639.6	HRH1	cyan	0.757399159
ENSG00000113749.5	HRH2	turquoise	0.862839306
ENSG00000101180.11	HRH3	turquoise	0.768434365
ENSG00000135116.5	HRK	pink	0.575371917
ENSG00000132541.6	HRSP12	green	0.742109104
ENSG00000118960.8	HS1BP3	blue	0.822314227
ENSG00000153936.12	HS2ST1	turquoise	0.904571469
ENSG00000182601.6	HS3ST4	turquoise	0.686904737
ENSG00000249853.3	HS3ST5	turquoise	0.802449035
ENSG00000136720.6	HS6ST1	turquoise	0.917933817
ENSG00000171004.13	HS6ST2	turquoise	0.861837855

ENSG00000185352.7	HS6ST3	turquoise	0.738897004
ENSG00000141854.3	hsa-mir-1199	red	0.856561062
ENSG00000215769.4	hsa-mir-6080	yellow	0.891502446
ENSG00000237973.1	hsa-mir-6723	turquoise	-0.523353037
ENSG00000256092.2	hsa-mir-8072	red	0.632713941
ENSG00000230989.2	HSBP1	turquoise	0.911777462
ENSG00000226742.3	HSBP1L1	red	0.862226567
ENSG00000100209.5	HSCB	turquoise	0.782984083
ENSG00000117594.5	HSD11B1	brown	0.715089203
ENSG00000167733.9	HSD11B1L	turquoise	0.829589
ENSG00000176387.6	HSD11B2	midnightblue	0.677604172
ENSG00000108786.6	HSD17B1	turquoise	0.746947975
ENSG00000072506.8	HSD17B10	blue	0.79243994
ENSG00000198189.6	HSD17B11	blue	0.805134687
ENSG00000149084.7	HSD17B12	blue	0.85203841
ENSG00000087076.4	HSD17B14	red	0.74578679
ENSG00000108785.7	HSD17B1P1	turquoise	0.794112499
ENSG00000130948.5	HSD17B3	brown	0.780517519
ENSG00000133835.10	HSD17B4	blue	0.946832038
ENSG00000025423.7	HSD17B6	green	0.80105083
ENSG00000132196.9	HSD17B7	cyan	0.814605623
ENSG00000099251.10	HSD17B7P2	yellow	0.761412843
ENSG00000204228.3	HSD17B8	brown	0.533551718
ENSG00000099377.9	HSD3B7	black	0.740260969
ENSG00000103160.7	HSDL1	turquoise	0.964934018
ENSG00000119471.10	HSDL2	green	0.910188149
ENSG00000185122.6	HSF1	blue	0.820771569
ENSG00000025156.8	HSF2	turquoise	0.949403782
ENSG00000102878.11	HSF4	yellow	0.890129353
ENSG00000080824.14	HSP90AA1	cyan	0.944847255
ENSG00000096384.15	HSP90AB1	cyan	0.953347627
ENSG00000166598.8	HSP90B1	cyan	0.944830962
ENSG00000165868.8	HSPA12A	turquoise	0.9637537
ENSG00000132622.6	HSPA12B	grey	0.462420495
ENSG00000155304.4	HSPA13	turquoise	0.951786469
ENSG00000187522.9	HSPA14	turquoise	0.964706687
ENSG00000204389.8	HSPA1A	black	0.53602946
ENSG00000204388.5	HSPA1B	black	0.579564645
ENSG00000204390.8	HSPA1L	cyan	0.854214601
ENSG00000126803.8	HSPA2	brown	0.920802487
ENSG00000170606.9	HSPA4	cyan	0.948052659
ENSG00000164070.7	HSPA4L	cyan	0.910923226
ENSG00000044574.7	HSPA5	cyan	0.942031617
ENSG00000173110.6	HSPA6	black	0.551209905
ENSG00000225217.1	HSPA7	black	0.604849116
ENSG00000109971.9	HSPA8	cyan	0.926828253
ENSG00000113013.8	HSPA9	turquoise	0.896721373
ENSG00000106211.8	HSPB1	black	0.635965296

ENSG00000081870.7	HSPB11	turquoise	0.937713817
ENSG00000236060.2	HSPB1P1	black	0.533445452
ENSG00000170276.4	HSPB2	red	0.665689703
ENSG00000004776.7	HSPB6	green	0.593318394
ENSG00000173641.13	HSPB7	grey	0.36152136
ENSG00000152137.2	HSPB8	green	0.748447384
ENSG00000197723.3	HSPB9	turquoise	0.494019031
ENSG00000169087.6	HSPBAP1	turquoise	0.805581121
ENSG00000133265.6	HSPBP1	turquoise	0.854935204
ENSG00000144381.12	HSPD1	cyan	0.915161327
ENSG00000115541.6	HSPE1	cyan	0.90664687
ENSG00000142798.12	HSPG2	black	0.778551658
ENSG00000120694.15	HSPH1	turquoise	0.806121448
ENSG00000109854.9	HTATIP2	brown	0.782788001
ENSG00000102241.7	HTATSF1	turquoise	0.923988275
ENSG00000271361.1	HTATSF1P2	turquoise	0.842295241
ENSG00000135312.4	HTR1B	turquoise	0.796124113
ENSG00000179546.3	HTR1D	turquoise	0.840963492
ENSG00000168830.6	HTR1E	turquoise	0.880645514
ENSG00000102468.6	HTR2A	turquoise	0.680011988
ENSG00000147246.5	HTR2C	turquoise	0.897912681
ENSG00000166736.7	HTR3A	turquoise	0.709146681
ENSG00000164270.13	HTR4	turquoise	0.830707311
ENSG00000157219.3	HTR5A	turquoise	0.91201632
ENSG00000220575.3	HTR5A-AS1	turquoise	0.860576042
ENSG00000158748.3	HTR6	turquoise	0.836828464
ENSG00000183935.3	HTR7P1	turquoise	0.801145668
ENSG00000166033.7	HTRA1	green	0.757435055
ENSG00000115317.7	HTRA2	turquoise	0.937426817
ENSG00000197386.6	HTT	turquoise	0.970213131
ENSG00000142149.4	HUNK	turquoise	0.80804408
ENSG00000136273.7	HUS1	turquoise	0.936173143
ENSG00000086758.11	HUWE1	turquoise	0.939342071
ENSG00000122986.9	HVCN1	green	0.652190763
ENSG00000114378.12	HYAL1	black	0.636701306
ENSG00000068001.9	HYAL2	black	0.741065104
ENSG00000186792.11	HYAL3	turquoise	0.749776203
ENSG00000157423.13	HYDIN	pink	0.942547959
ENSG00000178922.12	HYI	red	0.809373384
ENSG00000188266.9	HYKK	turquoise	0.88596129
ENSG00000198331.6	HYLS1	turquoise	0.937284231
ENSG00000149428.14	HYOU1	turquoise	0.927427347
ENSG00000242028.1	HYPK	yellow	0.874168513
ENSG00000134330.14	IAH1	turquoise	0.94961502
ENSG00000196305.13	IARS	turquoise	0.956374751
ENSG00000067704.8	IARS2	turquoise	0.926598276
ENSG00000181873.8	IBA57	turquoise	0.870766518
ENSG00000203684.5	IBA57-AS1	turquoise	0.782031469

ENSG00000005700.10	IBTK	turquoise	0.942272752
ENSG00000003147.13	ICA1	turquoise	0.915379808
ENSG00000163596.12	ICA1L	turquoise	0.934994551
ENSG00000090339.4	ICAM1	black	0.798454301
ENSG00000108622.6	ICAM2	black	0.806875884
ENSG00000076662.5	ICAM3	red	0.592176987
ENSG00000105376.4	ICAM5	turquoise	0.850691961
ENSG00000112144.11	ICK	brown	0.89368446
ENSG00000116237.11	ICMT	turquoise	0.92601968
ENSG00000160223.12	ICOSLG	brown	0.84145166
ENSG00000167862.5	ICT1	turquoise	0.901890293
ENSG00000125968.7	ID1	grey	0.353073425
ENSG00000115738.5	ID2	green	0.629509933
ENSG00000117318.8	ID3	black	0.672568336
ENSG00000172201.6	ID4	green	0.816317484
ENSG00000119912.11	IDE	turquoise	0.897841431
ENSG00000138413.9	IDH1	blue	0.882858188
ENSG00000231908.1	IDH1-AS1	grey	0.369077485
ENSG00000182054.5	IDH2	blue	0.736450399
ENSG00000101365.16	IDH3B	turquoise	0.973230352
ENSG00000067829.14	IDH3G	turquoise	0.943428675
ENSG00000067064.6	IDI1	turquoise	0.896445127
ENSG00000232656.3	IDI2-AS1	brown	0.648262741
ENSG00000148057.11	IDNK	turquoise	0.870352587
ENSG00000010404.13	IDS	turquoise	0.934099986
ENSG00000127415.8	IDUA	yellow	0.932295808
ENSG00000160888.6	IER2	grey	0.654002185
ENSG00000137331.11	IER3	blue	0.579446241
ENSG00000134049.3	IER3IP1	turquoise	0.941070705
ENSG00000162783.8	IER5	turquoise	0.578443464
ENSG00000188483.6	IER5L	black	0.578761031
ENSG00000010295.15	IFFO1	turquoise	0.916278126
ENSG00000169991.6	IFFO2	blue	0.777414552
ENSG00000163565.14	IFI16	magenta	0.81705311
ENSG00000165949.8	IFI27	black	0.519733303
ENSG00000165948.6	IFI27L1	red	0.890478415
ENSG00000119632.3	IFI27L2	red	0.6969042
ENSG00000216490.3	IFI30	magenta	0.839503564
ENSG00000068079.3	IFI35	black	0.718668479
ENSG00000137965.6	IFI44	brown	0.639189618
ENSG00000137959.11	IFI44L	brown	0.562589682
ENSG00000126709.10	IFI6	brown	0.588700746
ENSG00000115267.5	IFIH1	blue	0.655122917
ENSG00000185745.8	IFIT1	blue	0.708720899
ENSG00000119922.7	IFIT2	blue	0.605756992
ENSG00000119917.9	IFIT3	blue	0.656062641
ENSG00000152778.7	IFIT5	turquoise	0.806400731
ENSG00000185885.11	IFITM1	black	0.77327392

ENSG00000244242.1	IFITM10	greenyellow	0.784043389
ENSG00000185201.12	IFITM2	black	0.801365542
ENSG00000142089.11	IFITM3	black	0.799656748
ENSG00000142166.8	IFNAR1	turquoise	0.904971595
ENSG00000159110.15	IFNAR2	blue	0.88529984
ENSG00000027697.8	IFNGR1	blue	0.780526707
ENSG00000159128.10	IFNGR2	turquoise	0.923058033
ENSG00000185436.7	IFNLR1	pink	0.82466823
ENSG00000006652.9	IFRD1	cyan	0.921762301
ENSG00000214706.6	IFRD2	blue	0.876824204
ENSG00000163913.7	IFT122	turquoise	0.931294874
ENSG00000187535.9	IFT140	pink	0.937808651
ENSG00000138002.10	IFT172	turquoise	0.90053655
ENSG00000109083.9	IFT20	turquoise	0.93804483
ENSG00000100360.10	IFT27	turquoise	0.895959002
ENSG00000119650.8	IFT43	turquoise	0.915281444
ENSG00000118096.3	IFT46	pink	0.940806273
ENSG00000101052.8	IFT52	turquoise	0.928582894
ENSG00000114446.4	IFT57	blue	0.889784391
ENSG00000096872.11	IFT74	pink	0.904276325
ENSG00000068885.10	IFT80	turquoise	0.85432817
ENSG00000122970.11	IFT81	pink	0.949179169
ENSG00000032742.13	IFT88	blue	0.883282142
ENSG00000089289.11	IGBP1	turquoise	0.947558946
ENSG00000174498.9	IGDCC3	green	0.565262967
ENSG00000140443.9	IGF1R	turquoise	0.902059735
ENSG00000167244.13	IGF2	grey	0.281665247
ENSG00000197081.8	IGF2R	blue	0.911635916
ENSG00000099769.5	IGFALS	yellow	0.726409489
ENSG00000115457.5	IGFBP2	magenta	0.6178407
ENSG00000146674.10	IGFBP3	grey	0.271859245
ENSG00000141753.5	IGFBP4	black	0.728601597
ENSG00000115461.4	IGFBP5	turquoise	0.805523889
ENSG00000167779.3	IGFBP6	turquoise	0.755584666
ENSG00000163453.7	IGFBP7	black	0.719697175
ENSG00000137142.4	IGFBPL1	turquoise	0.831344311
ENSG00000204869.4	IGFL4	turquoise	0.851122613
ENSG00000126246.5	IGFLR1	black	0.774762907
ENSG00000163395.12	IGFN1	grey	0.238681775
ENSG00000211895.3	IGHA1	grey	0.164058444
ENSG00000211896.2	IGHG1	grey	0.226403533
ENSG00000240041.1	IGHJ4	grey	0.107743289
ENSG00000211899.3	IGHM	grey	0.121274761
ENSG00000132740.4	IGHMBP2	turquoise	0.94579443
ENSG00000182700.3	IGIP	turquoise	0.829570483
ENSG00000211595.2	IGKJ3	grey	0.133141596
ENSG00000211594.2	IGKJ4	grey	0.078960799
ENSG00000211593.2	IGKJ5	grey	0.1515756

ENSG00000142549.9	IGLON5	greenyellow	0.904283059
ENSG00000211644.2	IGLV1-51	turquoise	0.579207054
ENSG00000211643.2	IGLV5-52	turquoise	0.86986688
ENSG00000144847.8	IGSF11	blue	0.87587478
ENSG00000117154.7	IGSF21	greenyellow	0.745839111
ENSG00000179057.9	IGSF22	turquoise	0.809956392
ENSG00000143061.13	IGSF3	turquoise	0.910245062
ENSG00000183067.5	IGSF5	turquoise	0.885211687
ENSG00000140749.7	IGSF6	magenta	0.838179844
ENSG00000162729.9	IGSF8	turquoise	0.921161864
ENSG00000080854.10	IGSF9B	turquoise	0.732036612
ENSG00000113141.11	IK	turquoise	0.931117295
ENSG00000166130.10	IKBIP	turquoise	0.824172683
ENSG00000070061.10	IKBKAP	turquoise	0.962625274
ENSG00000104365.9	IKBKB	blue	0.920833289
ENSG00000143466.9	IKBKE	turquoise	0.805179461
ENSG00000073009.8	IKBKG	turquoise	0.923239865
ENSG00000030419.12	IKZF2	blue	0.805560037
ENSG00000123411.10	IKZF4	turquoise	0.900158681
ENSG00000095574.7	IKZF5	turquoise	0.877687314
ENSG00000110324.5	IL10RA	magenta	0.87854763
ENSG00000243646.4	IL10RB	blue	0.841707942
ENSG00000223799.1	IL10RB-AS1	blue	0.66515767
ENSG00000137070.13	IL11RA	yellow	0.92991416
ENSG00000168811.2	IL12A	black	0.560610932
ENSG00000131724.6	IL13RA1	black	0.835717078
ENSG00000123496.3	IL13RA2	turquoise	0.83981205
ENSG00000134470.15	IL15RA	black	0.876969407
ENSG00000172458.4	IL17D	blue	0.666987123
ENSG00000177663.9	IL17RA	turquoise	0.848064512
ENSG00000056736.5	IL17RB	green	0.839489854
ENSG00000163702.14	IL17RC	green	0.73488828
ENSG00000144730.12	IL17RD	green	0.888794852
ENSG00000163701.14	IL17RE	turquoise	0.857974376
ENSG00000150782.7	IL18	magenta	0.889524714
ENSG00000137496.13	IL18BP	blue	0.727481369
ENSG00000115604.6	IL18R1	black	0.653743878
ENSG00000125538.7	IL1B	magenta	0.566487581
ENSG00000115594.7	IL1R1	black	0.891991377
ENSG00000196083.5	IL1RAP	blue	0.683644717
ENSG00000115602.12	IL1RL1	black	0.605535827
ENSG00000016402.8	IL20RA	green	0.749819609
ENSG00000259954.1	IL21R-AS1	turquoise	0.504641746
ENSG00000104998.2	IL27RA	blue	0.739604065
ENSG00000147168.8	IL2RG	magenta	0.632251191
ENSG00000008517.12	IL32	black	0.653759379
ENSG00000137033.7	IL33	green	0.781613514
ENSG00000157368.6	IL34	turquoise	0.877996361

ENSG00000185291.6	IL3RA	blue	0.577760219
ENSG00000077238.9	IL4R	black	0.87041979
ENSG00000136244.7	IL6	black	0.608638057
ENSG00000160712.8	IL6R	black	0.908796884
ENSG00000134352.15	IL6ST	blue	0.865008506
ENSG00000169429.6	IL8	black	0.532933646
ENSG00000143621.12	ILF2	turquoise	0.934145748
ENSG00000129351.13	ILF3	turquoise	0.957247511
ENSG00000267100.1	ILF3-AS1	turquoise	0.928506347
ENSG00000166333.9	ILK	blue	0.91105596
ENSG00000132323.4	ILKAP	turquoise	0.92516062
ENSG00000105135.11	ILVBL	turquoise	0.934313034
ENSG00000148950.5	IMMP1L	turquoise	0.906522244
ENSG00000184903.5	IMMP2L	turquoise	0.910637413
ENSG00000132305.16	IMMT	turquoise	0.96638608
ENSG00000177971.7	IMP3	turquoise	0.94940585
ENSG00000136718.5	IMP4	turquoise	0.924589663
ENSG00000133731.5	IMPA1	turquoise	0.904073834
ENSG00000141401.7	IMPA2	green	0.661737445
ENSG00000154059.5	IMPACT	turquoise	0.862764438
ENSG00000104331.4	IMPAD1	turquoise	0.951254451
ENSG00000106348.12	IMPDH1	turquoise	0.903033148
ENSG00000178035.7	IMPDH2	turquoise	0.856074713
ENSG00000112706.7	IMPG1	turquoise	0.757541381
ENSG00000148798.5	INA	turquoise	0.921486839
ENSG00000132849.14	INADL	turquoise	0.911492659
ENSG00000196388.4	INCA1	turquoise	0.805697831
ENSG00000224975.1	INE1	blue	0.722933175
ENSG00000203485.8	INF2	midnightblue	0.900591795
ENSG00000153487.11	ING1	turquoise	0.878159066
ENSG00000168556.5	ING2	turquoise	0.899188433
ENSG00000071243.11	ING3	turquoise	0.873114989
ENSG00000111653.15	ING4	turquoise	0.950097312
ENSG00000168395.10	ING5	turquoise	0.941294741
ENSG00000123999.4	INHA	turquoise	0.662667399
ENSG00000122641.9	INHBA	turquoise	0.649120024
ENSG00000224116.2	INHBA-AS1	turquoise	0.846222147
ENSG00000163083.5	INHBB	green	0.797908301
ENSG00000148153.9	INIP	turquoise	0.907651087
ENSG00000128908.11	INO80	turquoise	0.936480888
ENSG00000115274.10	INO80B	turquoise	0.949599303
ENSG00000153391.11	INO80C	turquoise	0.936050069
ENSG00000114933.11	INO80D	turquoise	0.92483634
ENSG00000169592.10	INO80E	red	0.909912538
ENSG00000151689.8	INPP1	brown	0.818116155
ENSG00000040933.11	INPP4A	turquoise	0.965552168
ENSG00000109452.8	INPP4B	turquoise	0.873717355
ENSG00000068383.14	INPP5A	turquoise	0.903674834

ENSG00000204084.8	INPP5B	blue	0.903471133
ENSG00000168918.9	INPP5D	magenta	0.8651265
ENSG00000148384.11	INPP5E	turquoise	0.886046376
ENSG00000198825.7	INPP5F	turquoise	0.960809012
ENSG00000185133.9	INPP5J	turquoise	0.886356321
ENSG00000132376.15	INPP5K	turquoise	0.934617104
ENSG00000165458.9	INPPL1	blue	0.808445616
ENSG00000186480.8	INSIG1	brown	0.790602481
ENSG00000125629.10	INSIG2	turquoise	0.947081297
ENSG00000173404.3	INSM1	turquoise	0.864356442
ENSG00000168348.3	INSM2	turquoise	0.843696739
ENSG00000171105.9	INSR	blue	0.894873634
ENSG00000164880.11	INTS1	turquoise	0.91074173
ENSG00000104613.7	INTS10	turquoise	0.958058194
ENSG00000138785.10	INTS12	turquoise	0.896318654
ENSG00000108506.7	INTS2	turquoise	0.909980197
ENSG00000143624.9	INTS3	yellow	0.962176327
ENSG00000149262.12	INTS4	turquoise	0.899562026
ENSG00000164669.8	INTS4L1	turquoise	0.624611065
ENSG00000185085.2	INTS5	turquoise	0.932747184
ENSG00000102786.10	INTS6	turquoise	0.92032618
ENSG00000143493.8	INTS7	turquoise	0.962381877
ENSG00000164941.9	INTS8	turquoise	0.919424226
ENSG00000104299.10	INTS9	turquoise	0.908773072
ENSG00000164066.8	INTU	pink	0.88972403
ENSG00000119509.8	INVS	turquoise	0.851313943
ENSG00000176095.7	IP6K1	turquoise	0.97487332
ENSG00000068745.10	IP6K2	turquoise	0.947243373
ENSG00000161896.6	IP6K3	brown	0.812870009
ENSG00000074706.9	IPCEF1	turquoise	0.906871746
ENSG00000151151.5	IPMK	turquoise	0.868254274
ENSG00000086200.12	IPO11	blue	0.910223833
ENSG00000117408.6	IPO13	brown	0.87477623
ENSG00000196497.11	IPO4	blue	0.856165083
ENSG00000065150.14	IPO5	turquoise	0.930714115
ENSG00000205339.5	IPO7	turquoise	0.918483091
ENSG00000133704.5	IPO8	turquoise	0.908833606
ENSG00000198700.5	IPO9	turquoise	0.979878911
ENSG00000197429.6	IPP	blue	0.908450668
ENSG00000127080.5	IPPK	turquoise	0.925104941
ENSG00000132321.12	IQCA1	green	0.785237921
ENSG00000173226.12	IQCB1	turquoise	0.925091399
ENSG00000160051.7	IQCC	turquoise	0.92539272
ENSG00000106012.13	IQCE	turquoise	0.916652108
ENSG00000114473.9	IQCG	pink	0.914320167
ENSG00000259673.1	IQCH-AS1	turquoise	0.773627992
ENSG00000250588.2	IQCJ-SCHIP1	brown	0.830676728
ENSG00000174628.12	IQCK	blue	0.827686761

ENSG00000140575.8	IQGAP1	blue	0.820900371
ENSG00000144711.9	IQSEC1	turquoise	0.938035053
ENSG00000124313.8	IQSEC2	turquoise	0.921727185
ENSG00000120645.7	IQSEC3	turquoise	0.850679275
ENSG00000184216.7	IRAK1	blue	0.826342089
ENSG00000146243.9	IRAK1BP1	pink	0.877365029
ENSG00000134070.4	IRAK2	blue	0.650996955
ENSG00000090376.4	IRAK3	black	0.811586605
ENSG00000198001.9	IRAK4	blue	0.92858872
ENSG00000136381.8	IREB2	turquoise	0.953356493
ENSG00000125347.9	IRF1	black	0.76626304
ENSG00000168310.6	IRF2	blue	0.930096035
ENSG00000170604.3	IRF2BP1	turquoise	0.927618472
ENSG00000168264.6	IRF2BP2	blue	0.8250284
ENSG00000119669.3	IRF2BPL	turquoise	0.893429705
ENSG00000126456.11	IRF3	blue	0.860593645
ENSG00000128604.14	IRF5	magenta	0.928533198
ENSG00000185507.15	IRF7	black	0.867995629
ENSG00000140968.6	IRF8	magenta	0.90758342
ENSG00000213928.4	IRF9	blue	0.742001825
ENSG00000167378.4	IRGQ	turquoise	0.958048745
ENSG00000169047.5	IRS1	turquoise	0.892091205
ENSG00000185950.7	IRS2	turquoise	0.886587315
ENSG00000135070.9	ISCA1	turquoise	0.961892389
ENSG00000165898.9	ISCA2	turquoise	0.914714797
ENSG00000136003.11	ISCU	turquoise	0.946634614
ENSG00000187608.5	ISG15	brown	0.452667832
ENSG00000172183.10	ISG20	black	0.79347808
ENSG00000143319.12	ISG20L2	turquoise	0.927363032
ENSG00000016082.10	ISL1	turquoise	0.793401594
ENSG00000129009.8	ISLR	brown	0.511541151
ENSG00000167178.11	ISLR2	turquoise	0.843517779
ENSG00000101230.5	ISM1	greenyellow	0.608317102
ENSG00000100593.13	ISM2	turquoise	0.795167464
ENSG00000066583.7	ISOC1	turquoise	0.927047971
ENSG00000063241.3	ISOC2	red	0.891964362
ENSG00000182149.16	IST1	turquoise	0.883642996
ENSG00000240682.5	ISY1	turquoise	0.932857478
ENSG00000105655.14	ISYNA1	turquoise	0.677640491
ENSG00000078747.8	ITCH	turquoise	0.859541734
ENSG00000129636.8	ITFG1	turquoise	0.953238178
ENSG00000111203.7	ITFG2	turquoise	0.932935537
ENSG00000167930.11	ITFG3	blue	0.848154661
ENSG00000213949.4	ITGA1	black	0.732155416
ENSG00000143127.8	ITGA10	black	0.654522062
ENSG00000164171.6	ITGA2	blue	0.688538471
ENSG00000005961.13	ITGA2B	yellow	0.807107286
ENSG00000005884.13	ITGA3	turquoise	0.916460906

ENSG00000161638.6	ITGA5	black	0.893014527
ENSG00000091409.10	ITGA6	black	0.773604407
ENSG00000135424.11	ITGA7	green	0.77955855
ENSG00000077943.7	ITGA8	grey	0.46995618
ENSG00000144668.7	ITGA9	turquoise	0.773160459
ENSG00000083457.7	ITGAE	turquoise	0.868615676
ENSG00000005844.13	ITGAL	magenta	0.857117211
ENSG00000169896.12	ITGAM	magenta	0.939359897
ENSG00000138448.7	ITGAV	turquoise	0.877874457
ENSG00000140678.12	ITGAX	magenta	0.805807215
ENSG00000150093.14	ITGB1	blue	0.660339439
ENSG00000119185.8	ITGB1BP1	turquoise	0.904254954
ENSG00000160255.12	ITGB2	magenta	0.929992108
ENSG00000227039.2	ITGB2-AS1	turquoise	0.609358315
ENSG00000142856.12	ITGB3BP	turquoise	0.773714642
ENSG00000132470.9	ITGB4	black	0.739712238
ENSG00000082781.7	ITGB5	green	0.728568218
ENSG00000139626.11	ITGB7	green	0.722442352
ENSG00000105855.5	ITGB8	green	0.824798159
ENSG00000198542.9	ITGBL1	turquoise	0.834506967
ENSG00000151655.13	ITIH2	brown	0.557000864
ENSG00000055955.11	ITIH4	yellow	0.828853945
ENSG00000123243.10	ITIH5	brown	0.552977684
ENSG00000078596.6	ITM2A	brown	0.597143774
ENSG00000136156.8	ITM2B	blue	0.916211551
ENSG00000135916.11	ITM2C	turquoise	0.70254562
ENSG00000125877.8	ITPA	turquoise	0.920739866
ENSG00000100605.12	ITPK1	brown	0.821377361
ENSG00000137825.6	ITPKA	turquoise	0.766525101
ENSG00000143772.5	ITPKB	green	0.710168102
ENSG00000086544.2	ITPKC	black	0.821528543
ENSG00000150995.13	ITPR1	turquoise	0.897924658
ENSG00000231249.1	ITPR1-AS1	turquoise	0.866717329
ENSG00000123104.7	ITPR2	green	0.879939692
ENSG00000096433.6	ITPR3	blue	0.731113134
ENSG00000148841.11	ITPRIP	black	0.887423156
ENSG00000205730.5	ITPRIPL2	black	0.740710602
ENSG00000205726.9	ITSN1	turquoise	0.911035245
ENSG00000198399.10	ITSN2	turquoise	0.912061601
ENSG00000128928.4	IVD	turquoise	0.921232308
ENSG00000116679.11	IVNS1ABP	midnightblue	0.926970962
ENSG00000163166.9	IWS1	turquoise	0.873171321
ENSG00000099840.9	IZUMO4	blue	0.662821141
ENSG00000101384.7	JAG1	blue	0.756704886
ENSG00000184916.4	JAG2	turquoise	0.835751425
ENSG00000171135.10	JAGN1	turquoise	0.913668276
ENSG00000162434.7	JAK1	turquoise	0.873136531
ENSG00000096968.8	JAK2	turquoise	0.889841832

ENSG00000105639.14	JAK3	magenta	0.806199894
ENSG00000152969.12	JAKMIP1	turquoise	0.915775489
ENSG00000176049.11	JAKMIP2	turquoise	0.919393133
ENSG00000188385.7	JAKMIP3	turquoise	0.846257034
ENSG00000154721.10	JAM2	green	0.775267888
ENSG00000166086.8	JAM3	brown	0.936239679
ENSG00000008083.9	JARID2	turquoise	0.869733568
ENSG00000153814.7	JAZF1	turquoise	0.954801299
ENSG00000234336.2	JAZF1-AS1	turquoise	0.876214899
ENSG00000140044.8	JDP2	cyan	0.814059213
ENSG00000006459.6	JHDM1D	turquoise	0.805832026
ENSG00000260231.1	JHDM1D-AS1	red	0.564938249
ENSG00000050130.13	JKAMP	turquoise	0.943753776
ENSG00000171988.13	JMJD1C	turquoise	0.880790992
ENSG00000081692.8	JMJD4	turquoise	0.822299944
ENSG00000070495.10	JMJD6	cyan	0.918073211
ENSG00000243789.6	JMJD7	turquoise	0.876694916
ENSG00000161999.7	JMJD8	turquoise	0.929093613
ENSG00000152409.8	JMY	turquoise	0.944963868
ENSG00000100221.6	JOSD1	turquoise	0.944917739
ENSG00000161677.7	JOSD2	red	0.789624529
ENSG00000104369.4	JPH1	turquoise	0.859417665
ENSG00000154118.8	JPH3	turquoise	0.858545947
ENSG00000092051.12	JPH4	turquoise	0.887739114
ENSG00000225470.2	JPX	turquoise	0.806315618
ENSG00000234616.4	JRK	turquoise	0.935121527
ENSG00000183340.5	JRKL	turquoise	0.828051373
ENSG00000143543.10	JTB	turquoise	0.906780854
ENSG00000177606.5	JUN	black	0.620139824
ENSG00000171223.4	JUNB	black	0.484731909
ENSG00000130522.4	JUND	turquoise	0.718758985
ENSG00000173801.12	JUP	brown	0.635303843
ENSG00000011201.6	KAL1	green	0.846253159
ENSG00000160145.11	KALRN	turquoise	0.916631014
ENSG00000107104.14	KANK1	blue	0.818287832
ENSG00000197256.6	KANK2	brown	0.636894746
ENSG00000186994.7	KANK3	black	0.652522415
ENSG00000120071.8	KANSL1	turquoise	0.936596941
ENSG00000214401.4	KANSL1-AS1	grey	0.223964076
ENSG00000139620.8	KANSL2	turquoise	0.915695012
ENSG00000114982.13	KANSL3	turquoise	0.933246415
ENSG00000065427.10	KARS	turquoise	0.947048025
ENSG00000108773.6	KAT2A	yellow	0.953868586
ENSG00000114166.7	KAT2B	blue	0.800793246
ENSG00000172977.8	KAT5	turquoise	0.954957212
ENSG00000083168.5	KAT6A	blue	0.926604067
ENSG00000136504.7	KAT7	turquoise	0.980509438
ENSG00000103510.15	KAT8	turquoise	0.924690279

ENSG00000186625.9	KATNA1	blue	0.903712669
ENSG00000102781.9	KATNAL1	turquoise	0.894294495
ENSG00000167216.12	KATNAL2	green	0.791663105
ENSG00000140854.8	KATNB1	turquoise	0.947486378
ENSG00000134152.6	KATNBL1	blue	0.864148863
ENSG00000189337.11	KAZN	turquoise	0.921332707
ENSG00000272973.1	KB-1125A3.11	turquoise	0.784636046
ENSG00000245970.2	KB-1208A12.3	blue	0.785519398
ENSG00000271882.1	KB-1410C5.5	turquoise	0.779971076
ENSG00000261087.1	KB-1460A1.5	yellow	0.836025248
ENSG00000253320.1	KB-1507C5.2	turquoise	0.724890647
ENSG00000254281.1	KB-1507C5.4	turquoise	0.706492498
ENSG00000272321.1	KB-1517D11.4	midnightblue	0.860136213
ENSG00000253633.1	KB-1980E6.3	turquoise	0.816968272
ENSG00000272733.1	KB-208E9.1	turquoise	0.627866393
ENSG00000272955.1	KB-226F1.1	grey	0.305553829
ENSG00000272787.1	KB-226F1.2	blue	0.624881418
ENSG00000246263.2	KB-431C1.4	turquoise	0.871391068
ENSG00000272037.1	KB-431C1.5	blue	0.749302623
ENSG00000176595.3	KBTBD11	turquoise	0.94853094
ENSG00000253696.2	KBTBD11-OT1	black	0.565907628
ENSG00000187715.9	KBTBD12	turquoise	0.712136219
ENSG00000170852.6	KBTBD2	turquoise	0.855263262
ENSG00000182359.10	KBTBD3	turquoise	0.837274977
ENSG00000123444.9	KBTBD4	turquoise	0.939845481
ENSG00000165572.6	KBTBD6	turquoise	0.935306222
ENSG00000120696.8	KBTBD7	turquoise	0.928391838
ENSG00000163376.7	KBTBD8	turquoise	0.866665998
ENSG00000176407.13	KCMF1	turquoise	0.959802646
ENSG00000111262.4	KCNA1	turquoise	0.861266985
ENSG00000177301.9	KCNA2	turquoise	0.829210347
ENSG00000182255.6	KCNA4	turquoise	0.857654598
ENSG00000130037.3	KCNA5	turquoise	0.843206903
ENSG00000169282.13	KCNAB1	turquoise	0.829021495
ENSG00000069424.10	KCNAB2	turquoise	0.868949311
ENSG00000170049.5	KCNAB3	greenyellow	0.866994982
ENSG00000158445.7	KCNB1	turquoise	0.905809206
ENSG00000129159.6	KCNC1	turquoise	0.915158385
ENSG00000166006.8	KCNC2	greenyellow	0.853468486
ENSG00000131398.9	KCNC3	turquoise	0.909438131
ENSG00000116396.9	KCNC4	greenyellow	0.843933111
ENSG00000102057.5	KCND1	green	0.656759374
ENSG00000184408.5	KCND2	turquoise	0.902924553
ENSG00000171385.5	KCND3	turquoise	0.942492156
ENSG00000176076.6	KCNE1L	grey	0.16316604
ENSG00000159197.3	KCNE2	turquoise	0.716010588
ENSG00000175538.6	KCNE3	blue	0.681150539
ENSG00000152049.5	KCNE4	black	0.659500943

ENSG00000162975.3	KCNF1	turquoise	0.840445301
ENSG00000171126.7	KCNG3	turquoise	0.853578435
ENSG00000143473.7	KCNH1	turquoise	0.809079036
ENSG00000234233.1	KCNH1-IT1	turquoise	0.720125775
ENSG00000055118.10	KCNH2	turquoise	0.908081894
ENSG00000135519.6	KCNH3	turquoise	0.81457651
ENSG00000089558.4	KCNH4	turquoise	0.746636043
ENSG00000183960.4	KCNH8	brown	0.848008631
ENSG00000182132.8	KCNIP1	turquoise	0.852968024
ENSG00000120049.14	KCNIP2	turquoise	0.844053186
ENSG00000115041.8	KCNIP3	turquoise	0.695029173
ENSG00000185774.10	KCNIP4	turquoise	0.888012295
ENSG00000177807.6	KCNJ10	brown	0.678667642
ENSG00000187486.5	KCNJ11	turquoise	0.841582205
ENSG00000184185.5	KCNJ12	turquoise	0.828941999
ENSG00000182324.5	KCNJ14	grey	0.267311042
ENSG00000123700.4	KCNJ2	turquoise	0.816667462
ENSG00000267365.1	KCNJ2-AS1	brown	0.705882124
ENSG00000162989.3	KCNJ3	greenyellow	0.797565798
ENSG00000168135.4	KCNJ4	turquoise	0.832976581
ENSG00000121361.3	KCNJ8	black	0.765969521
ENSG00000162728.4	KCNJ9	turquoise	0.821973358
ENSG00000135750.10	KCNK1	turquoise	0.897375785
ENSG00000100433.11	KCNK10	brown	0.731698664
ENSG00000184261.4	KCNK12	greenyellow	0.850621474
ENSG00000152315.4	KCNK13	magenta	0.791333795
ENSG00000082482.9	KCNK2	turquoise	0.823029077
ENSG00000171303.5	KCNK3	turquoise	0.814815708
ENSG00000182450.8	KCNK4	turquoise	0.804235996
ENSG00000173338.8	KCNK7	blue	0.528440937
ENSG00000156113.16	KCNMA1	turquoise	0.943656493
ENSG00000135643.4	KCNMB4	turquoise	0.784005376
ENSG00000105642.11	KCNN1	turquoise	0.766319979
ENSG00000080709.10	KCNN2	pink	0.799910983
ENSG00000143603.14	KCNN3	green	0.861836604
ENSG00000104783.7	KCNN4	turquoise	0.6722262
ENSG00000053918.11	KCNQ1	magenta	0.811989711
ENSG00000075043.13	KCNQ2	turquoise	0.88739108
ENSG00000184156.11	KCNQ3	turquoise	0.913970076
ENSG00000117013.10	KCNQ4	turquoise	0.852031704
ENSG00000185760.11	KCNQ5	turquoise	0.897120772
ENSG00000156486.7	KCNS2	turquoise	0.722933918
ENSG00000170745.7	KCNS3	brown	0.683824034
ENSG00000107147.7	KCNT1	turquoise	0.753640499
ENSG00000162687.12	KCNT2	greenyellow	0.791068219
ENSG00000164794.4	KCNV1	turquoise	0.834447897
ENSG00000134504.8	KCTD1	turquoise	0.878850315
ENSG00000110906.8	KCTD10	turquoise	0.886250594

ENSG00000213859.3	KCTD11	cyan	0.860951112
ENSG00000178695.4	KCTD12	turquoise	0.751148798
ENSG00000174943.5	KCTD13	turquoise	0.932764564
ENSG00000153885.10	KCTD15	green	0.808164153
ENSG00000183775.6	KCTD16	turquoise	0.882855456
ENSG00000100379.13	KCTD17	turquoise	0.775298548
ENSG00000155729.8	KCTD18	blue	0.889338826
ENSG00000180901.6	KCTD2	turquoise	0.965162858
ENSG00000112078.9	KCTD20	blue	0.946323937
ENSG00000188997.3	KCTD21	blue	0.770935057
ENSG00000246174.3	KCTD21-AS1	turquoise	0.831450936
ENSG00000136636.8	KCTD3	turquoise	0.857986009
ENSG00000180332.5	KCTD4	turquoise	0.74454982
ENSG00000167977.4	KCTD5	turquoise	0.817804953
ENSG00000168301.8	KCTD6	blue	0.862314696
ENSG00000243335.4	KCTD7	turquoise	0.924673315
ENSG00000183783.6	KCTD8	turquoise	0.936852208
ENSG00000104756.11	KCTD9	blue	0.897107371
ENSG00000134901.8	KDELC1	blue	0.822725133
ENSG00000178202.8	KDELC2	green	0.69717554
ENSG00000105438.4	KDELR1	turquoise	0.921348561
ENSG00000136240.5	KDELR2	turquoise	0.850886787
ENSG00000100196.6	KDELR3	pink	0.792188549
ENSG00000004487.11	KDM1A	turquoise	0.979230553
ENSG00000165097.9	KDM1B	blue	0.904446357
ENSG00000173120.10	KDM2A	turquoise	0.934387088
ENSG00000089094.12	KDM2B	turquoise	0.9520649
ENSG00000115548.12	KDM3A	blue	0.90732974
ENSG00000120733.9	KDM3B	turquoise	0.941786011
ENSG00000066135.8	KDM4A	turquoise	0.971388783
ENSG00000236200.1	KDM4A-AS1	yellow	0.863949683
ENSG00000127663.10	KDM4B	turquoise	0.929514433
ENSG00000107077.13	KDM4C	turquoise	0.932040269
ENSG00000186280.5	KDM4D	turquoise	0.830472706
ENSG00000073614.7	KDM5A	blue	0.9177192
ENSG00000117139.12	KDM5B	turquoise	0.95945309
ENSG00000126012.7	KDM5C	turquoise	0.928715066
ENSG00000012817.11	KDM5D	grey	0.283419688
ENSG00000147050.10	KDM6A	turquoise	0.770769179
ENSG00000132510.6	KDM6B	yellow	0.806252476
ENSG00000155666.7	KDM8	turquoise	0.926529255
ENSG00000128052.8	KDR	brown	0.70293358
ENSG00000119537.11	KDSR	turquoise	0.776431631
ENSG00000079999.9	KEAP1	turquoise	0.877741572
ENSG00000197993.3	KEL	brown	0.781540783
ENSG00000135314.8	KHDC1	turquoise	0.920955606
ENSG00000256980.4	KHDC1L	turquoise	0.698167055
ENSG00000121774.13	KHDRBS1	turquoise	0.966699616

ENSG00000112232.8	KHDRBS2	turquoise	0.885229846
ENSG00000131773.9	KHDRBS3	turquoise	0.781692659
ENSG00000138030.8	KHK	turquoise	0.942492366
ENSG00000100441.5	KHNYN	blue	0.893677126
ENSG00000088247.11	KHSRP	turquoise	0.960322877
ENSG00000080608.9	KIAA0020	cyan	0.898892378
ENSG00000235750.5	KIAA0040	black	0.799289778
ENSG00000007202.10	KIAA0100	turquoise	0.957631271
ENSG00000081791.4	KIAA0141	turquoise	0.891168026
ENSG00000177728.10	KIAA0195	turquoise	0.853438977
ENSG00000164961.11	KIAA0196	turquoise	0.894889545
ENSG00000145016.9	KIAA0226	turquoise	0.965506705
ENSG00000102445.14	KIAA0226L	blue	0.714748672
ENSG00000170871.7	KIAA0232	turquoise	0.910445473
ENSG00000100647.7	KIAA0247	blue	0.841274114
ENSG00000137261.9	KIAA0319	turquoise	0.884374101
ENSG00000142687.13	KIAA0319L	turquoise	0.976512072
ENSG00000166398.8	KIAA0355	blue	0.861313411
ENSG00000136813.10	KIAA0368	turquoise	0.956642037
ENSG00000100890.11	KIAA0391	blue	0.922027276
ENSG00000189367.10	KIAA0408	turquoise	0.89521368
ENSG00000166783.15	KIAA0430	turquoise	0.927158038
ENSG00000135709.8	KIAA0513	turquoise	0.906276137
ENSG00000047578.8	KIAA0556	turquoise	0.863149661
ENSG00000100578.10	KIAA0586	turquoise	0.917605165
ENSG00000198920.5	KIAA0753	turquoise	0.947561573
ENSG00000185261.9	KIAA0825	turquoise	0.790108982
ENSG00000164542.8	KIAA0895	turquoise	0.945084665
ENSG00000196123.8	KIAA0895L	turquoise	0.923079534
ENSG00000132680.6	KIAA0907	turquoise	0.932369393
ENSG00000121210.11	KIAA0922	turquoise	0.92161745
ENSG00000100364.14	KIAA0930	brown	0.834815641
ENSG00000164151.7	KIAA0947	turquoise	0.950495045
ENSG00000135315.7	KIAA1009	yellow	0.86967637
ENSG00000136051.9	KIAA1033	blue	0.908618553
ENSG00000122733.11	KIAA1045	turquoise	0.895188285
ENSG00000069712.9	KIAA1107	turquoise	0.932166413
ENSG00000138688.11	KIAA1109	turquoise	0.915968056
ENSG00000163807.4	KIAA1143	turquoise	0.886247288
ENSG00000257093.2	KIAA1147	brown	0.814892925
ENSG00000164976.8	KIAA1161	green	0.737975529
ENSG00000122203.10	KIAA1191	turquoise	0.955995996
ENSG00000103888.11	KIAA1199	grey	0.646353497
ENSG00000196872.6	KIAA1211L	turquoise	0.890711104
ENSG00000120549.11	KIAA1217	turquoise	0.878732059
ENSG00000112379.8	KIAA1244	turquoise	0.922931772
ENSG00000198954.4	KIAA1279	turquoise	0.974143624
ENSG00000116299.12	KIAA1324	turquoise	0.905338753

ENSG00000164659.10	KIAA1324L	brown	0.828507081
ENSG00000150477.10	KIAA1328	turquoise	0.864179429
ENSG00000110318.9	KIAA1377	turquoise	0.909906025
ENSG00000163617.6	KIAA1407	pink	0.912539638
ENSG00000164944.7	KIAA1429	turquoise	0.907693957
ENSG00000164323.8	KIAA1430	blue	0.892776882
ENSG00000107036.7	KIAA1432	turquoise	0.892589441
ENSG00000250305.4	KIAA1456	pink	0.927066881
ENSG00000165757.8	KIAA1462	turquoise	0.77172473
ENSG00000084444.9	KIAA1467	turquoise	0.946567766
ENSG00000134444.9	KIAA1468	turquoise	0.95499264
ENSG00000162522.6	KIAA1522	turquoise	0.901173048
ENSG00000163507.9	KIAA1524	turquoise	0.892548469
ENSG00000122778.5	KIAA1549	turquoise	0.908958539
ENSG00000110427.10	KIAA1549L	turquoise	0.920777779
ENSG00000174718.7	KIAA1551	turquoise	0.835609897
ENSG00000168116.9	KIAA1586	turquoise	0.898061074
ENSG00000187164.13	KIAA1598	brown	0.738034348
ENSG00000135835.6	KIAA1614	blue	0.821902411
ENSG00000138944.7	KIAA1644	greenyellow	0.907401012
ENSG00000197077.8	KIAA1671	turquoise	0.9155799
ENSG00000130518.12	KIAA1683	turquoise	0.498049465
ENSG00000144320.9	KIAA1715	turquoise	0.946141756
ENSG00000166004.10	KIAA1731	turquoise	0.91521901
ENSG00000198894.3	KIAA1737	turquoise	0.901909652
ENSG00000149633.7	KIAA1755	brown	0.860759836
ENSG00000162929.9	KIAA1841	turquoise	0.923525736
ENSG00000179698.9	KIAA1875	turquoise	0.741729398
ENSG00000173214.5	KIAA1919	turquoise	0.810499965
ENSG00000165185.10	KIAA1958	blue	0.894415466
ENSG00000213213.9	KIAA1984	turquoise	0.890650031
ENSG00000228544.1	KIAA1984-AS1	turquoise	0.849323313
ENSG00000116685.11	KIAA2013	blue	0.89858187
ENSG00000176542.5	KIAA2018	turquoise	0.934030123
ENSG00000050030.9	KIAA2022	turquoise	0.884123938
ENSG00000183354.7	KIAA2026	turquoise	0.901431287
ENSG00000134313.10	KIDINS220	turquoise	0.931041843
ENSG00000138160.4	KIF11	turquoise	0.870770543
ENSG00000136883.8	KIF12	turquoise	0.805220772
ENSG00000137177.14	KIF13A	brown	0.893908551
ENSG00000197892.8	KIF13B	brown	0.930408714
ENSG00000089177.13	KIF16B	blue	0.923330619
ENSG00000117245.8	KIF17	turquoise	0.887967243
ENSG00000196169.10	KIF19	brown	0.583833876
ENSG00000130294.10	KIF1A	turquoise	0.915719766
ENSG00000054523.12	KIF1B	turquoise	0.951391848
ENSG00000129250.7	KIF1C	brown	0.46092311
ENSG00000139116.13	KIF21A	turquoise	0.940916277

ENSG00000116852.10	KIF21B	turquoise	0.810230621
ENSG00000079616.8	KIF22	turquoise	0.92611668
ENSG00000186638.11	KIF24	pink	0.911143449
ENSG00000125337.12	KIF25	grey	0.39876416
ENSG00000066735.10	KIF26A	grey	0.550751171
ENSG00000162849.11	KIF26B	greenyellow	0.767796701
ENSG00000165115.10	KIF27	pink	0.875203205
ENSG00000068796.12	KIF2A	blue	0.863711867
ENSG00000131437.11	KIF3A	turquoise	0.968570889
ENSG00000101350.6	KIF3B	turquoise	0.936311538
ENSG00000084731.9	KIF3C	turquoise	0.941788866
ENSG00000155980.7	KIF5A	turquoise	0.757089287
ENSG00000170759.10	KIF5B	blue	0.535070274
ENSG00000168280.12	KIF5C	turquoise	0.846297776
ENSG00000164627.13	KIF6	blue	0.674900594
ENSG00000166813.10	KIF7	blue	0.768760929
ENSG00000088727.8	KIF9	pink	0.94013551
ENSG00000227398.3	KIF9-AS1	yellow	0.881822524
ENSG00000075945.8	KIFAP3	turquoise	0.976859759
ENSG00000167702.7	KIFC2	turquoise	0.887748713
ENSG00000140859.11	KIFC3	turquoise	0.824969386
ENSG00000151657.7	KIN	turquoise	0.934173034
ENSG00000183853.13	KIRREL	brown	0.579164174
ENSG00000126259.15	KIRREL2	turquoise	0.783302454
ENSG00000149571.6	KIRREL3	turquoise	0.902717405
ENSG00000170498.7	KISS1	turquoise	0.554961674
ENSG00000116014.5	KISS1R	turquoise	0.62863241
ENSG00000157404.11	KIT	turquoise	0.791634481
ENSG00000049130.9	KITLG	turquoise	0.770337947
ENSG00000133116.6	KL	turquoise	0.839453812
ENSG00000134962.6	KLB	turquoise	0.624639836
ENSG00000126214.16	KLC1	turquoise	0.957427896
ENSG00000174996.7	KLC2	turquoise	0.945106828
ENSG00000137171.10	KLC4	turquoise	0.946072369
ENSG00000155090.10	KLF10	blue	0.641349643
ENSG00000172059.6	KLF11	black	0.725973671
ENSG00000118922.12	KLF12	blue	0.718076236
ENSG00000169926.5	KLF13	brown	0.855605564
ENSG00000163884.3	KLF15	green	0.683439328
ENSG00000129911.4	KLF16	turquoise	0.845688145
ENSG00000127528.5	KLF2	grey	0.276449709
ENSG00000109787.8	KLF3	blue	0.90606933
ENSG00000136826.10	KLF4	grey	0.316193616
ENSG00000102554.9	KLF5	turquoise	0.780457195
ENSG00000067082.10	KLF6	black	0.848540139
ENSG00000118263.10	KLF7	blue	0.913141302
ENSG00000102349.10	KLF8	turquoise	0.893737065
ENSG00000119138.3	KLF9	turquoise	0.810065747

ENSG00000197776.3	KLHDC1	turquoise	0.90940217
ENSG00000128607.9	KLHDC10	turquoise	0.954349868
ENSG00000165516.6	KLHDC2	turquoise	0.970288008
ENSG00000124702.13	KLHDC3	turquoise	0.935885628
ENSG00000104731.9	KLHDC4	turquoise	0.869226538
ENSG00000162873.10	KLHDC8A	turquoise	0.783059442
ENSG00000185909.10	KLHDC8B	red	0.671892077
ENSG00000162755.9	KLHDC9	turquoise	0.907714706
ENSG00000150361.7	KLHL1	turquoise	0.735833873
ENSG00000117153.11	KLHL12	turquoise	0.95645228
ENSG00000003096.9	KLHL13	turquoise	0.921526127
ENSG00000174010.9	KLHL15	turquoise	0.895465285
ENSG00000187961.9	KLHL17	turquoise	0.751448763
ENSG00000114648.7	KLHL18	turquoise	0.967176865
ENSG00000109466.9	KLHL2	turquoise	0.920577663
ENSG00000076321.6	KLHL20	turquoise	0.899297067
ENSG00000162413.12	KLHL21	blue	0.844676893
ENSG00000099910.12	KLHL22	turquoise	0.953638678
ENSG00000213160.5	KLHL23	turquoise	0.865482497
ENSG00000114796.11	KLHL24	blue	0.893766458
ENSG00000183655.11	KLHL25	turquoise	0.778260191
ENSG00000167487.7	KLHL26	turquoise	0.882666605
ENSG00000179454.9	KLHL28	turquoise	0.858867411
ENSG00000119771.10	KLHL29	turquoise	0.923233984
ENSG00000146021.10	KLHL3	turquoise	0.933591015
ENSG00000186231.12	KLHL32	brown	0.902899461
ENSG00000149243.11	KLHL35	turquoise	0.863948215
ENSG00000135686.8	KLHL36	turquoise	0.842870759
ENSG00000102271.9	KLHL4	brown	0.853704404
ENSG00000239474.2	KLHL41	turquoise	0.679019033
ENSG00000087448.5	KLHL42	turquoise	0.971644513
ENSG00000109790.12	KLHL5	brown	0.730343471
ENSG00000122550.13	KLHL7	turquoise	0.972106931
ENSG00000145332.9	KLHL8	turquoise	0.869275984
ENSG00000198642.5	KLHL9	turquoise	0.898191069
ENSG00000129437.5	KLK14	grey	0.800813259
ENSG00000167755.9	KLK6	brown	0.87448059
ENSG00000164344.11	KLKB1	grey	0.598643706
ENSG00000256667.2	KLRAP1	turquoise	0.803110399
ENSG00000205809.5	KLRC2	grey	0.136546994
ENSG00000205810.4	KLRC3	brown	0.531127971
ENSG00000183542.4	KLRC4	grey	0.362549838
ENSG00000139187.5	KLRG1	turquoise	0.688234862
ENSG00000213809.4	KLRK1	grey	0.329423778
ENSG00000118058.16	KMT2A	turquoise	0.929377458
ENSG00000055609.13	KMT2C	turquoise	0.876255271
ENSG00000167548.10	KMT2D	turquoise	0.906487919
ENSG00000005483.15	KMT2E	turquoise	0.899707559

ENSG00000239569.2	KMT2E-AS1	blue	0.690875286
ENSG00000162456.5	KNCN	turquoise	0.780680496
ENSG00000171798.13	KNDC1	turquoise	0.845004725
ENSG00000103550.9	KNOP1	turquoise	0.792210853
ENSG00000128944.9	KNSTRN	turquoise	0.913921903
ENSG00000184445.7	KNTC1	blue	0.831273086
ENSG00000114030.8	KPNA1	turquoise	0.957635726
ENSG00000182481.4	KPNA2	turquoise	0.897110484
ENSG00000102753.5	KPNA3	turquoise	0.95508616
ENSG00000186432.4	KPNA4	turquoise	0.950489855
ENSG00000196911.5	KPNA5	turquoise	0.868050635
ENSG00000025800.9	KPNA6	turquoise	0.973317715
ENSG00000108424.5	KPNB1	turquoise	0.96374152
ENSG00000203786.5	KPRP	turquoise	0.544454199
ENSG00000118162.9	KPTN	turquoise	0.873240962
ENSG00000133703.7	KRAS	turquoise	0.957662572
ENSG00000133619.13	KRBA1	turquoise	0.934964435
ENSG00000240747.3	KRBOX1	red	0.655473772
ENSG00000147121.11	KRBOX4	turquoise	0.96603511
ENSG00000172086.7	KRCC1	blue	0.892528302
ENSG00000183762.8	KREMEN1	turquoise	0.796130398
ENSG00000129347.15	KRI1	blue	0.857108924
ENSG00000001631.10	KRIT1	turquoise	0.945206351
ENSG00000111615.8	KRR1	turquoise	0.942767067
ENSG00000167768.4	KRT1	grey	0.038496856
ENSG00000186395.6	KRT10	turquoise	0.74697917
ENSG00000171401.10	KRT13	grey	0.091755052
ENSG00000186847.5	KRT14	grey	0.160389563
ENSG00000171345.9	KRT19	grey	0.432727804
ENSG00000172867.3	KRT2	grey	0.036565804
ENSG00000213424.4	KRT222	turquoise	0.893581951
ENSG00000170477.8	KRT4	grey	0.071938855
ENSG00000186081.7	KRT5	turquoise	0.449060782
ENSG00000229320.3	KRT8P12	turquoise	0.769516082
ENSG00000214659.4	KRT8P26	turquoise	0.807303475
ENSG00000224928.2	KRT8P30	turquoise	0.811295955
ENSG00000205869.2	KRTAP5-1	turquoise	0.776475099
ENSG00000244411.2	KRTAP5-7	turquoise	0.646901374
ENSG00000254997.2	KRTAP5-9	turquoise	0.650570293
ENSG00000233930.3	KRTAP5-AS1	turquoise	0.770581013
ENSG00000163463.7	KRTCAP2	turquoise	0.805832937
ENSG00000157992.8	KRTCAP3	yellow	0.803917589
ENSG00000188508.6	KRTDAP	grey	0.366025375
ENSG00000141068.9	KSR1	blue	0.884427761
ENSG00000171435.9	KSR2	turquoise	0.905942579
ENSG00000198841.3	KTI12	turquoise	0.95207926
ENSG00000126777.13	KTN1	blue	0.876934331
ENSG00000105700.6	KXD1	turquoise	0.912339294

ENSG00000174611.7	KY	turquoise	0.820409508
ENSG00000198910.8	L1CAM	turquoise	0.935213595
ENSG00000087299.7	L2HGDH	turquoise	0.898471921
ENSG00000126790.7	L3HYPDH	blue	0.862998026
ENSG00000185513.10	L3MBTL1	yellow	0.953055022
ENSG00000100395.10	L3MBTL2	turquoise	0.964292317
ENSG00000198945.3	L3MBTL3	turquoise	0.77536904
ENSG00000154655.10	L3MBTL4	turquoise	0.842022928
ENSG00000260425.1	LA16c-316G12.2	yellow	0.915676112
ENSG00000261399.1	LA16c-329F2.1	grey	0.106869475
ENSG00000262528.1	LA16c-349E10.1	yellow	0.744001273
ENSG00000261505.1	LA16c-358B7.3	blue	0.784365188
ENSG00000261207.1	LA16c-361A3.3	yellow	0.767721928
ENSG00000259840.1	LA16c-380A1.1	turquoise	0.503051506
ENSG00000272079.1	LA16c-380H5.5	midnightblue	0.795122019
ENSG00000260051.1	LA16c-390E6.4	yellow	0.885691826
ENSG00000268836.1	LA16c-OS12.2	yellow	0.661258185
ENSG00000179630.6	LACC1	brown	0.838981081
ENSG00000135537.12	LACE1	turquoise	0.866922657
ENSG00000103642.7	LACTB	turquoise	0.925253328
ENSG00000147592.4	LACTB2	blue	0.813449545
ENSG00000196976.5	LAGE3	red	0.936841655
ENSG00000167613.11	LAIR1	magenta	0.951937116
ENSG00000101680.9	LAMA1	green	0.811112894
ENSG00000196569.7	LAMA2	brown	0.783346195
ENSG00000053747.11	LAMA3	blue	0.575766398
ENSG00000112769.14	LAMA4	green	0.595039616
ENSG00000130702.9	LAMA5	blue	0.639112907
ENSG00000091136.9	LAMB1	turquoise	0.881663024
ENSG00000172037.9	LAMB2	blue	0.783540971
ENSG00000196878.8	LAMB3	turquoise	0.795023666
ENSG00000135862.5	LAMC1	blue	0.837367252
ENSG00000050555.13	LAMC3	brown	0.569165834
ENSG00000185896.9	LAMP1	brown	0.876489483
ENSG00000005893.11	LAMP2	brown	0.926897574
ENSG00000125869.5	LAMP5	turquoise	0.858414432
ENSG00000149357.5	LAMTOR1	red	0.960107164
ENSG00000116586.7	LAMTOR2	red	0.962284795
ENSG00000109270.8	LAMTOR3	turquoise	0.953828822
ENSG00000188186.6	LAMTOR4	red	0.968928015
ENSG00000134248.9	LAMTOR5	turquoise	0.884506633
ENSG00000115365.7	LANCL1	turquoise	0.919476013
ENSG00000132434.5	LANCL2	turquoise	0.95204257
ENSG00000002549.8	LAP3	blue	0.836516958
ENSG00000068697.6	LAPTM4A	green	0.892339275
ENSG00000104341.12	LAPTM4B	turquoise	0.806923373
ENSG00000162511.7	LAPTM5	magenta	0.959067281
ENSG00000133424.16	LARGE	turquoise	0.882380301

ENSG00000155506.12	LARP1	turquoise	0.97183168
ENSG00000138709.13	LARP1B	turquoise	0.94130887
ENSG00000161813.16	LARP4	blue	0.890734392
ENSG00000107929.10	LARP4B	turquoise	0.966040805
ENSG00000166173.9	LARP6	brown	0.789391057
ENSG00000174720.11	LARP7	blue	0.88790992
ENSG00000133706.13	LARS	turquoise	0.954079282
ENSG00000011376.5	LARS2	blue	0.835235697
ENSG00000001497.12	LAS1L	yellow	0.946921081
ENSG00000002834.13	LASP1	turquoise	0.932805233
ENSG00000213658.6	LAT	grey	0.843052059
ENSG00000086730.12	LAT2	magenta	0.93803778
ENSG00000131023.8	LATS1	turquoise	0.913808626
ENSG00000150457.7	LATS2	black	0.843495614
ENSG00000204381.7	LAYN	brown	0.833144395
ENSG00000213626.7	LBH	grey	0.278806128
ENSG00000143815.10	LBR	blue	0.903630585
ENSG00000135338.9	LCA5	blue	0.849524198
ENSG00000213398.3	LCAT	blue	0.632809582
ENSG00000172954.9	LCLAT1	turquoise	0.923228333
ENSG00000205629.7	LCMT1	turquoise	0.960044328
ENSG00000168806.6	LCMT2	blue	0.880909976
ENSG00000187922.9	LCN10	grey	0.181944208
ENSG00000184925.7	LCN12	green	0.615855181
ENSG00000214402.6	LCNL1	brown	0.48139873
ENSG00000196233.7	LCOR	turquoise	0.858930242
ENSG00000178177.10	LCORL	blue	0.822573687
ENSG00000136167.9	LCP1	magenta	0.9062698
ENSG00000043462.7	LCP2	magenta	0.813188212
ENSG00000188501.7	LCTL	turquoise	0.731767275
ENSG00000198728.6	LDB1	turquoise	0.960979615
ENSG00000169744.8	LDB2	turquoise	0.901290791
ENSG00000122367.15	LDB3	brown	0.856316699
ENSG00000134333.9	LDHA	turquoise	0.912458969
ENSG00000214110.3	LDHAP4	turquoise	0.755338578
ENSG00000111716.8	LDHB	blue	0.82201623
ENSG00000166816.9	LDHD	green	0.775136243
ENSG00000130164.7	LDLR	black	0.798471888
ENSG00000179241.8	LDLRAD3	blue	0.779270002
ENSG00000168675.14	LDLRAD4	brown	0.843500729
ENSG00000157978.7	LDLRAP1	brown	0.847272916
ENSG00000182195.6	LDOC1	turquoise	0.941601827
ENSG00000188636.3	LDOC1L	turquoise	0.874941622
ENSG00000164406.7	LEAP2	blue	0.750240607
ENSG00000136110.8	LECT1	turquoise	0.887894945
ENSG00000138795.5	LEF1	black	0.559156075
ENSG00000243709.1	LEFTY1	turquoise	0.732360474
ENSG00000161904.7	LEMD2	turquoise	0.87563416

ENSG00000174106.2	LEMD3	turquoise	0.95174885
ENSG00000105617.3	LENG1	turquoise	0.760165319
ENSG00000167615.12	LENG8	yellow	0.961774783
ENSG00000226696.1	LENG8-AS1	yellow	0.901555404
ENSG00000166477.8	LEO1	turquoise	0.956804989
ENSG00000116678.14	LEPR	turquoise	0.529634204
ENSG00000117385.11	LEPRE1	yellow	0.944909785
ENSG00000090530.5	LEPREL1	blue	0.682992708
ENSG00000110811.15	LEPREL2	turquoise	0.799215731
ENSG00000141696.8	LEPREL4	turquoise	0.802725627
ENSG00000213625.4	LEPROT	green	0.881325226
ENSG00000104660.13	LEPROTL1	turquoise	0.958982386
ENSG00000168924.10	LETM1	turquoise	0.917118428
ENSG00000165046.8	LETM2	pink	0.858238237
ENSG00000050426.11	LETMD1	turquoise	0.95537813
ENSG00000106003.8	LFNG	green	0.590557535
ENSG00000100097.7	LGALS1	black	0.65466077
ENSG00000131981.11	LGALS3	black	0.661058006
ENSG00000108679.8	LGALS3BP	brown	0.777529041
ENSG00000116977.14	LGALS8	turquoise	0.973147469
ENSG00000168961.12	LGALS9	magenta	0.894209601
ENSG00000119862.8	LGALSL	turquoise	0.943721391
ENSG00000108231.7	LGI1	turquoise	0.883593423
ENSG00000153012.7	LGI2	greenyellow	0.728744067
ENSG00000168481.4	LGI3	brown	0.82067416
ENSG00000153902.9	LGI4	green	0.679514865
ENSG00000100600.10	LGMN	turquoise	0.925117476
ENSG00000205213.9	LGR4	green	0.894107357
ENSG00000139292.8	LGR5	brown	0.843057067
ENSG00000183722.7	LHFP	blue	0.693594922
ENSG00000182508.9	LHFPL1	green	0.667050195
ENSG00000145685.9	LHFPL2	blue	0.852063685
ENSG00000187416.7	LHFPL3	turquoise	0.874353344
ENSG00000156959.7	LHFPL4	turquoise	0.904230281
ENSG00000107902.9	LHPP	brown	0.761177358
ENSG00000106689.6	LHX2	green	0.577485892
ENSG00000106852.11	LHX6	greenyellow	0.822489561
ENSG00000162624.10	LHX8	midnightblue	0.661255705
ENSG00000121897.9	LIAS	turquoise	0.941608594
ENSG00000113594.5	LIFR	green	0.861729627
ENSG00000244968.2	LIFR-AS1	turquoise	0.808830069
ENSG00000105486.9	LIG1	turquoise	0.898729395
ENSG00000005156.7	LIG3	turquoise	0.952828088
ENSG00000174405.9	LIG4	turquoise	0.936427314
ENSG00000239998.1	LILRA2	magenta	0.903734589
ENSG00000104972.10	LILRB1	magenta	0.93793972
ENSG00000186818.8	LILRB4	magenta	0.924910345
ENSG00000050405.9	LIMA1	turquoise	0.779512406

ENSG00000064042.13	LIMCH1	brown	0.868275557
ENSG00000144791.5	LIMD1	blue	0.785571405
ENSG00000136490.4	LIMD2	turquoise	0.743909889
ENSG00000203896.5	LIME1	midnightblue	0.712068039
ENSG00000106683.10	LIMK1	greenyellow	0.920071088
ENSG00000182541.13	LIMK2	black	0.791096244
ENSG00000169756.12	LIMS1	blue	0.821922987
ENSG00000072163.14	LIMS2	yellow	0.785816831
ENSG00000267796.3	LIN37	turquoise	0.880746302
ENSG00000205659.6	LIN52	turquoise	0.93694103
ENSG00000189308.6	LIN54	turquoise	0.923817978
ENSG00000111052.3	LIN7A	turquoise	0.858975114
ENSG00000104863.7	LIN7B	turquoise	0.899171399
ENSG00000148943.7	LIN7C	turquoise	0.812351162
ENSG00000183814.11	LIN9	turquoise	0.868060914
ENSG00000182310.8	LINC00085	brown	0.6223306
ENSG00000178947.8	LINC00086	turquoise	0.899891655
ENSG00000196972.6	LINC00087	turquoise	0.878538665
ENSG00000225194.2	LINC00092	green	0.667992855
ENSG00000235106.4	LINC00094	turquoise	0.914467958
ENSG00000236871.2	LINC00106	yellow	0.835397169
ENSG00000225880.4	LINC00115	yellow	0.770922714
ENSG00000175701.6	LINC00116	red	0.89271051
ENSG00000222041.6	LINC00152	turquoise	0.592317281
ENSG00000185433.4	LINC00158	turquoise	0.734211239
ENSG00000196668.3	LINC00173	yellow	0.74578495
ENSG00000179406.6	LINC00174	yellow	0.841724232
ENSG00000196421.3	LINC00176	yellow	0.829178766
ENSG00000232224.1	LINC00202-1	yellow	0.850873286
ENSG00000223768.1	LINC00205	turquoise	0.822966058
ENSG00000229214.1	LINC00242	turquoise	0.899683211
ENSG00000235823.1	LINC00263	brown	0.749930164
ENSG00000188185.7	LINC00265	yellow	0.903058944
ENSG00000212766.5	LINC00277	turquoise	0.674163137
ENSG00000248197.1	LINC00290	turquoise	0.789662478
ENSG00000236790.1	LINC00299	green	0.642886287
ENSG00000224924.2	LINC00320	brown	0.669742963
ENSG00000178977.3	LINC00324	turquoise	0.649644369
ENSG00000234912.5	LINC00338	turquoise	0.885556563
ENSG00000218510.3	LINC00339	turquoise	0.834760983
ENSG00000229645.4	LINC00341	brown	0.802093289
ENSG00000232931.1	LINC00342	yellow	0.912101875
ENSG00000237879.1	LINC00398	turquoise	0.782500475
ENSG00000224243.1	LINC00403	turquoise	0.85126549
ENSG00000229520.1	LINC00404	turquoise	0.826041767
ENSG00000233532.1	LINC00460	turquoise	0.789980356
ENSG00000245526.4	LINC00461	blue	0.625196875
ENSG00000153363.8	LINC00467	brown	0.579539051

ENSG00000175611.7	LINC00476	turquoise	0.835608016
ENSG00000215386.6	LINC00478	green	0.866178043
ENSG00000232388.2	LINC00493	turquoise	0.915280615
ENSG00000251372.1	LINC00499	grey	0.188115214
ENSG00000225667.1	LINC00505	grey	0.378662512
ENSG00000227036.2	LINC00511	turquoise	0.748921375
ENSG00000260583.1	LINC00515	turquoise	0.729965173
ENSG00000196273.3	LINC00523	turquoise	0.698425609
ENSG00000264575.1	LINC00526	turquoise	0.895161597
ENSG00000232815.1	LINC00537	turquoise	0.768195099
ENSG00000228126.1	LINC00568	turquoise	0.725577447
ENSG00000224177.2	LINC00570	turquoise	0.718083389
ENSG00000231690.2	LINC00574	turquoise	0.863213765
ENSG00000203809.5	LINC00577	midnightblue	0.816180076
ENSG00000259714.1	LINC00594	turquoise	0.798428484
ENSG00000253230.2	LINC00599	turquoise	0.766925469
ENSG00000257585.1	LINC00609	pink	0.539072628
ENSG00000250366.2	LINC00617	turquoise	0.772964164
ENSG00000262619.1	LINC00621	turquoise	0.739182121
ENSG00000260941.1	LINC00622	turquoise	0.810688414
ENSG00000203930.6	LINC00632	turquoise	0.845095459
ENSG00000205704.5	LINC00634	turquoise	0.805292047
ENSG00000259070.1	LINC00639	brown	0.862744033
ENSG00000258441.1	LINC00641	turquoise	0.928778298
ENSG00000186369.5	LINC00643	turquoise	0.836161477
ENSG00000205181.5	LINC00654	turquoise	0.722596408
ENSG00000260032.1	LINC00657	turquoise	0.924700186
ENSG00000261824.2	LINC00662	turquoise	0.93370336
ENSG00000266904.1	LINC00663	turquoise	0.832449214
ENSG00000232677.2	LINC00665	turquoise	0.961785771
ENSG00000263753.2	LINC00667	turquoise	0.868489683
ENSG00000263874.1	LINC00672	turquoise	0.905528299
ENSG00000237854.3	LINC00674	blue	0.7362062
ENSG00000215190.4	LINC00680	turquoise	0.700959353
ENSG00000226179.1	LINC00685	grey	0.685253541
ENSG00000228214.2	LINC00693	turquoise	0.701506963
ENSG00000185904.7	LINC00839	turquoise	0.701976104
ENSG00000237949.1	LINC00844	brown	0.652271749
ENSG00000186842.4	LINC00846	turquoise	0.872252278
ENSG00000245060.2	LINC00847	turquoise	0.903983993
ENSG00000231177.4	LINC00852	turquoise	0.786904532
ENSG00000224805.2	LINC00853	grey	0.061407727
ENSG00000224914.2	LINC00863	turquoise	0.740651692
ENSG00000232229.1	LINC00865	greenyellow	0.64025806
ENSG00000242759.2	LINC00882	turquoise	0.89707633
ENSG00000243701.1	LINC00883	brown	0.838388488
ENSG00000240024.1	LINC00888	turquoise	0.942038662
ENSG00000241769.3	LINC00893	turquoise	0.825324861

ENSG00000235703.1	LINC00894	yellow	0.886712385
ENSG00000231711.2	LINC00899	blue	0.534400965
ENSG00000246100.3	LINC00900	turquoise	0.753210798
ENSG00000264247.1	LINC00909	brown	0.822192424
ENSG00000188825.9	LINC00910	blue	0.801552452
ENSG00000259134.1	LINC00924	brown	0.486643083
ENSG00000255571.2	LINC00925	turquoise	0.830705436
ENSG00000247982.2	LINC00926	yellow	0.766221779
ENSG00000271614.1	LINC00936	turquoise	0.89499529
ENSG00000273015.1	LINC00938	turquoise	0.864586966
ENSG00000235049.1	LINC00940	brown	0.768093964
ENSG00000235314.1	LINC00957	brown	0.686202329
ENSG00000237489.2	LINC00959	turquoise	0.817404407
ENSG00000235387.1	LINC00961	black	0.672217623
ENSG00000204054.7	LINC00963	blue	0.79755463
ENSG00000254377.1	LINC00966	turquoise	0.859332574
ENSG00000242086.4	LINC00969	yellow	0.753516113
ENSG00000177133.6	LINC00982	green	0.629173051
ENSG00000259330.1	LINC00984	turquoise	0.848850468
ENSG00000237248.3	LINC00987	turquoise	0.676230557
ENSG00000214194.4	LINC00998	turquoise	0.883419584
ENSG00000225119.3	LINC00999	blue	0.774434596
ENSG00000261455.1	LINC01003	turquoise	0.811807697
ENSG00000228393.3	LINC01004	yellow	0.875496664
ENSG00000182648.7	LINC01006	brown	0.537126242
ENSG00000244041.3	LINC01011	turquoise	0.818710445
ENSG00000250056.1	LINC01018	blue	0.686683376
ENSG00000272523.1	LINC01023	grey	0.481696806
ENSG00000245146.2	LINC01024	brown	0.726016157
ENSG00000224081.3	LINC01057	black	0.715126955
ENSG00000232065.1	LINC01063	turquoise	0.604466039
ENSG00000169783.8	LINGO1	turquoise	0.875324462
ENSG00000174482.6	LINGO2	turquoise	0.699377535
ENSG00000220008.2	LINGO3	turquoise	0.800729942
ENSG00000140471.12	LINS	turquoise	0.937592012
ENSG00000107798.13	LIPA	brown	0.925371612
ENSG00000079435.5	LIPE	brown	0.896145375
ENSG00000213904.4	LIPE-AS1	turquoise	0.679141255
ENSG00000144182.12	LIPT1	turquoise	0.803203125
ENSG00000175536.6	LIPT2	turquoise	0.78853402
ENSG00000189067.8	LITAF	blue	0.835236734
ENSG00000145721.7	LIX1	green	0.811012202
ENSG00000152022.7	LIX1L	green	0.837007318
ENSG00000273473.1	LL09NC01-139C3.1	turquoise	0.509642859
ENSG00000270012.1	LL0XNC01-7P3.1	blue	0.752537672
ENSG00000273027.1	LL21NC02-1C16.1	turquoise	0.546547427
ENSG00000272825.1	LL21NC02-1C16.2	grey	0.384904332
ENSG00000234630.1	LL22NC03-2H8.4	turquoise	0.715354781

ENSG00000272216.1	LL22NC03-2H8.5	turquoise	0.748315532
ENSG00000272779.1	LL22NC03-80A10.6	turquoise	0.927219404
ENSG00000224086.3	LL22NC03-86G7.1	turquoise	0.752037258
ENSG00000270041.1	LL22NC03-N27C7.1	blue	0.656720636
ENSG00000273116.1	LLfos-48D6.1	yellow	0.684599612
ENSG00000131899.6	LLGL1	brown	0.853675909
ENSG00000073350.9	LLGL2	brown	0.763569776
ENSG00000139233.2	LLPH	blue	0.873388897
ENSG00000074695.5	LMAN1	blue	0.930695834
ENSG00000169223.10	LMAN2	turquoise	0.874201449
ENSG00000114988.7	LMAN2L	cyan	0.870075464
ENSG00000105983.15	LMBR1	turquoise	0.960863712
ENSG00000139636.11	LMBR1L	yellow	0.958097401
ENSG00000168216.6	LMBRD1	blue	0.93752365
ENSG00000164187.6	LMBRD2	turquoise	0.931678309
ENSG00000071282.7	LMCD1	blue	0.592588483
ENSG00000103227.14	LMF1	turquoise	0.855806437
ENSG00000100258.13	LMF2	turquoise	0.848112865
ENSG00000185621.7	LMLN	turquoise	0.932497487
ENSG00000160789.15	LMNA	blue	0.695428728
ENSG00000113368.7	LMNB1	turquoise	0.768498892
ENSG00000176619.6	LMNB2	turquoise	0.882268849
ENSG00000166407.9	LMO1	turquoise	0.517083854
ENSG00000135363.7	LMO2	black	0.734603802
ENSG00000048540.10	LMO3	turquoise	0.891597314
ENSG00000143013.8	LMO4	turquoise	0.898373698
ENSG00000136153.15	LMO7	turquoise	0.917704045
ENSG00000163431.11	LMOD1	green	0.586648505
ENSG00000164715.5	LMTK2	turquoise	0.964514793
ENSG00000142235.4	LMTK3	turquoise	0.8666924
ENSG00000206535.3	LNP1	turquoise	0.913345475
ENSG00000072201.9	LNX1	turquoise	0.905844905
ENSG00000139517.6	LNX2	turquoise	0.86894011
ENSG00000165714.6	LOH12CR1	turquoise	0.945481679
ENSG00000205791.2	LOH12CR2	turquoise	0.749308062
ENSG00000196365.7	LONP1	turquoise	0.914295795
ENSG00000102910.9	LONP2	turquoise	0.949482887
ENSG00000154359.8	LONRF1	turquoise	0.923335024
ENSG00000170500.8	LONRF2	turquoise	0.941278973
ENSG00000113083.8	LOX	pink	0.841570585
ENSG00000167210.12	LOXHD1	midnightblue	0.853045851
ENSG00000129038.11	LOXL1	turquoise	0.650040491
ENSG00000261801.1	LOXL1-AS1	turquoise	0.897644251
ENSG00000134013.11	LOXL2	blue	0.698194705
ENSG00000115318.7	LOXL3	blue	0.802409716
ENSG00000198121.9	LPAR1	brown	0.499567867
ENSG00000064547.9	LPAR2	yellow	0.803676579
ENSG00000171517.5	LPAR3	pink	0.666122692

ENSG00000184574.5	LPAR5	magenta	0.88589186
ENSG00000139679.11	LPAR6	magenta	0.846001661
ENSG00000153395.5	LPCAT1	turquoise	0.891989842
ENSG00000087253.7	LPCAT2	magenta	0.885810067
ENSG00000111684.6	LPCAT3	blue	0.907150696
ENSG00000176454.9	LPCAT4	turquoise	0.911613976
ENSG00000123684.8	LPGAT1	turquoise	0.930189877
ENSG00000072071.12	LPHN1	turquoise	0.912506563
ENSG00000117114.15	LPHN2	turquoise	0.903563542
ENSG00000150471.11	LPHN3	blue	0.835060231
ENSG00000134324.7	LPIN1	blue	0.864486898
ENSG00000101577.5	LPIN2	turquoise	0.975590814
ENSG00000132793.7	LPIN3	green	0.709974296
ENSG00000175445.10	LPL	turquoise	0.842096538
ENSG00000145012.8	LPP	blue	0.774189574
ENSG00000270959.1	LPP-AS2	green	0.700100121
ENSG00000148123.10	LPPR1	turquoise	0.868092011
ENSG00000129951.14	LPPR3	turquoise	0.790121101
ENSG00000117600.8	LPPR4	turquoise	0.918589763
ENSG00000117598.7	LPPR5	turquoise	0.888325006
ENSG00000110031.8	LPXN	blue	0.880025391
ENSG00000198589.6	LRBA	turquoise	0.937525589
ENSG00000136141.10	LRCH1	blue	0.831462148
ENSG00000130224.10	LRCH2	turquoise	0.909308528
ENSG00000186001.8	LRCH3	turquoise	0.888064791
ENSG00000077454.11	LRCH4	yellow	0.948060443
ENSG00000128011.4	LRFN1	turquoise	0.860628982
ENSG00000126243.4	LRFN3	turquoise	0.928506472
ENSG00000173621.8	LRFN4	turquoise	0.845049121
ENSG00000165379.9	LRFN5	turquoise	0.894681017
ENSG00000121931.11	LRIF1	turquoise	0.864055386
ENSG00000144749.9	LRIG1	green	0.856851052
ENSG00000198799.7	LRIG2	turquoise	0.909313424
ENSG00000139263.7	LRIG3	blue	0.715008973
ENSG00000204033.5	LRIT2	turquoise	0.810408642
ENSG00000118308.10	LRMP	magenta	0.889350954
ENSG00000123384.9	LRP1	turquoise	0.848829635
ENSG00000197324.4	LRP10	blue	0.742851593
ENSG00000120256.5	LRP11	turquoise	0.970136574
ENSG00000147650.7	LRP12	turquoise	0.945363261
ENSG00000168702.12	LRP1B	blue	0.758457609
ENSG00000081479.8	LRP2	brown	0.729566863
ENSG00000109771.11	LRP2BP	pink	0.881598273
ENSG00000130881.9	LRP3	turquoise	0.828155894
ENSG00000134569.5	LRP4	green	0.832118254
ENSG00000162337.7	LRP5	green	0.770829637
ENSG00000100068.7	LRP5L	yellow	0.761283051
ENSG00000070018.4	LRP6	turquoise	0.780986848

ENSG00000157193.10	LRP8	turquoise	0.778725605
ENSG00000163956.6	LRPAP1	turquoise	0.904587644
ENSG00000138095.14	LRPPRC	turquoise	0.968142116
ENSG00000165501.12	LRR1	blue	0.793744527
ENSG00000137269.10	LRRC1	blue	0.87802621
ENSG00000204950.2	LRRC10B	turquoise	0.748682786
ENSG00000160959.3	LRRC14	turquoise	0.956170799
ENSG00000185028.3	LRRC14B	turquoise	0.796156754
ENSG00000079691.15	LRRC16A	green	0.879267543
ENSG00000186648.10	LRRC16B	turquoise	0.8366458
ENSG00000128606.8	LRRC17	greenyellow	0.609809792
ENSG00000172731.9	LRRC20	turquoise	0.91779097
ENSG00000010626.10	LRRC23	pink	0.901490041
ENSG00000175489.9	LRRC25	magenta	0.880634629
ENSG00000148814.13	LRRC27	pink	0.936626186
ENSG00000168904.10	LRRC28	turquoise	0.956894378
ENSG00000125122.10	LRRC29	turquoise	0.743336428
ENSG00000137507.7	LRRC32	black	0.834776033
ENSG00000171757.11	LRRC34	pink	0.924328343
ENSG00000159708.13	LRRC36	turquoise	0.770940849
ENSG00000267023.1	LRRC37A16P	blue	0.770971349
ENSG00000263142.1	LRRC37A17P	blue	0.765685061
ENSG00000238083.3	LRRC37A2	grey	0.568708098
ENSG00000176809.6	LRRC37A3	yellow	0.90820253
ENSG00000230445.4	LRRC37A6P	turquoise	0.766440038
ENSG00000185158.8	LRRC37B	turquoise	0.948980145
ENSG00000250462.4	LRRC37BP1	turquoise	0.948517315
ENSG00000122477.8	LRRC39	midnightblue	0.836649085
ENSG00000179796.7	LRRC3B	green	0.692684633
ENSG00000269430.1	LRRC3DN	grey	0.383625115
ENSG00000128594.3	LRRC4	turquoise	0.922400849
ENSG00000066557.5	LRRC40	turquoise	0.920233578
ENSG00000132128.12	LRRC41	turquoise	0.9537471
ENSG00000116212.10	LRRC42	turquoise	0.907876633
ENSG00000158113.8	LRRC43	pink	0.895976632
ENSG00000169683.3	LRRC45	turquoise	0.840124679
ENSG00000130764.5	LRRC47	turquoise	0.973608746
ENSG00000137821.7	LRRC49	turquoise	0.955797127
ENSG00000131409.8	LRRC4B	turquoise	0.866361628
ENSG00000148948.3	LRRC4C	turquoise	0.923928312
ENSG00000183908.5	LRRC55	greenyellow	0.766156375
ENSG00000161328.10	LRRC56	turquoise	0.853298479
ENSG00000180979.5	LRRC57	blue	0.896481691
ENSG00000163428.3	LRRC58	turquoise	0.870553501
ENSG00000108829.9	LRRC59	turquoise	0.917341174
ENSG00000129295.4	LRRC6	pink	0.957446456
ENSG00000127399.10	LRRC61	turquoise	0.847700805
ENSG00000188993.3	LRRC66	turquoise	0.629407732

ENSG00000033122.14	LRRC7	turquoise	0.924016814
ENSG00000204052.4	LRRC73	turquoise	0.883036649
ENSG00000136802.7	LRRC8A	green	0.860873
ENSG00000197147.8	LRRC8B	turquoise	0.948855603
ENSG00000171488.10	LRRC8C	turquoise	0.800487027
ENSG00000171492.10	LRRC8D	brown	0.894228325
ENSG00000133739.11	LRRCC1	blue	0.633917669
ENSG00000124831.14	LRRFIP1	pink	0.853660029
ENSG00000093167.13	LRRFIP2	turquoise	0.879374849
ENSG00000154237.8	LRRK1	turquoise	0.69976503
ENSG00000188906.9	LRRK2	turquoise	0.883549222
ENSG00000175928.5	LRRN1	turquoise	0.850587224
ENSG00000170382.7	LRRN2	turquoise	0.890492091
ENSG00000173114.8	LRRN3	turquoise	0.885519949
ENSG00000125872.7	LRRN4	turquoise	0.821402829
ENSG00000162951.6	LRRTM1	turquoise	0.904957925
ENSG00000146006.7	LRRTM2	turquoise	0.935429507
ENSG00000198739.6	LRRTM3	turquoise	0.888732754
ENSG00000176204.9	LRRTM4	turquoise	0.908433776
ENSG00000148356.9	LRSAM1	turquoise	0.912459754
ENSG00000166159.6	LRTM2	turquoise	0.794563385
ENSG00000161036.6	LRWD1	pink	0.867621599
ENSG00000185565.7	LSAMP	blue	0.819515685
ENSG00000041802.6	LSG1	turquoise	0.910300088
ENSG00000175324.5	LSM1	turquoise	0.854742041
ENSG00000181817.5	LSM10	red	0.926939763
ENSG00000155858.5	LSM11	turquoise	0.868133353
ENSG00000161654.5	LSM12	turquoise	0.897676535
ENSG00000257103.4	LSM14A	turquoise	0.844215142
ENSG00000149657.15	LSM14B	turquoise	0.97824997
ENSG00000204392.6	LSM2	turquoise	0.821569529
ENSG00000170860.3	LSM3	turquoise	0.922250359
ENSG00000130520.6	LSM4	red	0.954061344
ENSG00000106355.5	LSM5	turquoise	0.918220786
ENSG00000164167.5	LSM6	turquoise	0.83536157
ENSG00000130332.10	LSM7	red	0.914272682
ENSG00000183011.9	LSMD1	red	0.94346473
ENSG00000181016.5	LSMEM1	blue	0.54612878
ENSG00000179564.3	LSMEM2	brown	0.742648678
ENSG00000105699.12	LSR	brown	0.563034848
ENSG00000160285.10	LSS	brown	0.845365842
ENSG00000204482.6	LST1	magenta	0.913369449
ENSG00000111144.5	LTA4H	turquoise	0.945549649
ENSG00000227507.2	LTB	turquoise	0.644530314
ENSG00000213903.4	LTB4R	yellow	0.8847965
ENSG00000213906.5	LTB4R2	turquoise	0.714188961
ENSG00000049323.11	LTBP1	blue	0.699204745
ENSG00000119681.7	LTBP2	turquoise	0.826341668

ENSG00000168056.10	LTBP3	turquoise	0.82482251
ENSG00000090006.13	LTBP4	turquoise	0.83391914
ENSG00000111321.6	LTBR	black	0.897961743
ENSG00000062524.11	LTK	turquoise	0.671030678
ENSG00000198862.9	LTN1	turquoise	0.890537958
ENSG00000135521.8	LTV1	turquoise	0.912307785
ENSG00000007392.12	LUC7L	yellow	0.937542984
ENSG00000108848.11	LUC7L3	yellow	0.919109896
ENSG00000171357.5	LURAP1	turquoise	0.889485142
ENSG00000153714.5	LURAP1L	turquoise	0.546345064
ENSG00000169641.9	LUZP1	turquoise	0.944064343
ENSG00000187398.7	LUZP2	greenyellow	0.806130946
ENSG00000079257.3	LXN	turquoise	0.644566288
ENSG00000160932.6	LY6E	turquoise	0.877458266
ENSG00000240053.8	LY6G5B	yellow	0.873198498
ENSG00000204428.8	LY6G5C	grey	0.692776242
ENSG00000176956.8	LY6H	turquoise	0.732714149
ENSG00000160886.9	LY6K	turquoise	0.487488042
ENSG00000112799.4	LY86	magenta	0.899430628
ENSG00000154589.2	LY96	magenta	0.776983127
ENSG00000145220.9	LYAR	turquoise	0.843611503
ENSG00000144214.5	LYG1	yellow	0.782963673
ENSG00000104903.4	LYL1	magenta	0.833406885
ENSG00000254087.3	LYN	magenta	0.896909994
ENSG00000180155.14	LYNX1	turquoise	0.868869749
ENSG00000124466.8	LYPD3	turquoise	0.613194777
ENSG00000159871.10	LYPD5	turquoise	0.939652341
ENSG00000187123.10	LYPD6	green	0.728755954
ENSG00000266949.1	LYPD8	turquoise	0.42877512
ENSG00000120992.13	LYPLA1	turquoise	0.836822938
ENSG00000011009.6	LYPLA2	turquoise	0.931503772
ENSG00000143353.7	LYPLAL1	blue	0.846665934
ENSG00000102897.5	LYRM1	blue	0.849652699
ENSG00000083099.6	LYRM2	blue	0.904128341
ENSG00000214113.6	LYRM4	turquoise	0.911781403
ENSG00000205707.6	LYRM5	blue	0.829375206
ENSG00000186687.11	LYRM7	turquoise	0.929168638
ENSG00000232859.5	LYRM9	turquoise	0.849476413
ENSG00000163155.7	LYSMD1	turquoise	0.959192287
ENSG00000140280.9	LYSMD2	turquoise	0.938002637
ENSG00000176018.8	LYSMD3	turquoise	0.879987781
ENSG00000183060.11	LYSMD4	turquoise	0.916883597
ENSG00000143669.9	LYST	turquoise	0.93656298
ENSG00000133800.4	LYVE1	magenta	0.742982539
ENSG00000090382.2	LYZ	magenta	0.610767696
ENSG00000162441.7	LZIC	blue	0.906171915
ENSG00000163818.12	LZTFL1	pink	0.913095942
ENSG00000099949.14	LZTR1	turquoise	0.89006897

ENSG00000061337.11	LZTS1	turquoise	0.816869542
ENSG00000107816.13	LZTS2	blue	0.834542166
ENSG00000088899.10	LZTS3	turquoise	0.918640025
ENSG00000003056.3	M6PR	turquoise	0.920937755
ENSG00000183833.12	MAATS1	pink	0.956503195
ENSG00000127603.19	MACF1	blue	0.885292029
ENSG00000133315.6	MACROD1	red	0.796770409
ENSG00000172264.12	MACROD2	turquoise	0.900296805
ENSG00000002822.11	MAD1L1	turquoise	0.749164536
ENSG00000164109.9	MAD2L1	turquoise	0.902728692
ENSG00000124688.9	MAD2L1BP	turquoise	0.961995823
ENSG00000116670.10	MAD2L2	turquoise	0.8605318
ENSG00000099866.10	MADCAM1	yellow	0.771425061
ENSG00000110514.14	MADD	turquoise	0.972768962
ENSG00000090316.11	MAEA	turquoise	0.979702141
ENSG00000178573.6	MAF	magenta	0.690830359
ENSG00000179632.5	MAF1	red	0.931453274
ENSG00000204103.2	MAFB	magenta	0.851061711
ENSG00000185022.7	MAFF	black	0.765521319
ENSG00000197063.6	MAFG	turquoise	0.876481219
ENSG00000265688.1	MAFG-AS1	turquoise	0.799658562
ENSG00000198517.5	MAFK	brown	0.567571143
ENSG00000105695.10	MAG	brown	0.902270587
ENSG00000165509.9	MAGEC3	turquoise	0.732710375
ENSG00000179222.13	MAGED1	turquoise	0.944361203
ENSG00000102316.12	MAGED2	blue	0.892342595
ENSG00000198934.3	MAGEE1	turquoise	0.950229636
ENSG00000186675.5	MAGEE2	turquoise	0.872394791
ENSG00000177383.3	MAGEF1	turquoise	0.964554333
ENSG00000187601.3	MAGEH1	turquoise	0.950872181
ENSG00000151276.19	MAGI1	turquoise	0.901500691
ENSG00000187391.13	MAGI2	blue	0.844935332
ENSG00000234456.3	MAGI2-AS3	blue	0.84811527
ENSG00000081026.14	MAGI3	blue	0.868263495
ENSG00000017621.11	MAGIX	turquoise	0.813312689
ENSG00000162385.6	MAGOH	turquoise	0.905697107
ENSG00000111196.5	MAGOHB	turquoise	0.916417449
ENSG00000102158.15	MAGT1	blue	0.862546834
ENSG00000198042.6	MAK16	turquoise	0.937138855
ENSG00000172005.6	MAL	brown	0.816802547
ENSG00000147676.9	MAL2	turquoise	0.892641249
ENSG00000251562.3	MALAT1	grey	0.675479688
ENSG00000156928.4	MALSU1	turquoise	0.925923122
ENSG00000172175.8	MALT1	blue	0.867765804
ENSG00000177943.9	MAMDC4	yellow	0.833129319
ENSG00000161021.7	MAML1	turquoise	0.887738825
ENSG00000184384.9	MAML2	green	0.767683266
ENSG00000196782.8	MAML3	turquoise	0.883191985

ENSG00000013619.9	MAMLD1	turquoise	0.88789155
ENSG00000176909.7	MAMSTR	turquoise	0.810568136
ENSG00000111885.5	MAN1A1	turquoise	0.8647856
ENSG00000198162.8	MAN1A2	turquoise	0.941558075
ENSG00000177239.10	MAN1B1	turquoise	0.93144342
ENSG00000117643.10	MAN1C1	pink	0.878196771
ENSG00000112893.5	MAN2A1	brown	0.843055761
ENSG00000196547.10	MAN2A2	brown	0.843757342
ENSG00000104774.8	MAN2B1	blue	0.81324991
ENSG00000013288.4	MAN2B2	turquoise	0.896174224
ENSG00000140400.10	MAN2C1	yellow	0.963558722
ENSG00000109323.4	MANBA	turquoise	0.922741225
ENSG00000101363.8	MANBAL	turquoise	0.95051977
ENSG00000172469.10	MANEA	blue	0.865209532
ENSG00000261366.1	MANEA-AS1	turquoise	0.749968277
ENSG00000185090.10	MANEAL	turquoise	0.937899649
ENSG00000145050.11	MANF	cyan	0.897404096
ENSG00000111261.9	MANSC1	turquoise	0.953629732
ENSG00000189221.5	MAOA	green	0.814806893
ENSG00000069535.12	MAOB	cyan	0.8213063
ENSG00000212916.3	MAP10	turquoise	0.885119634
ENSG00000166963.8	MAP1A	turquoise	0.930649741
ENSG00000131711.10	MAP1B	turquoise	0.925001273
ENSG00000101460.8	MAP1LC3A	turquoise	0.875645824
ENSG00000140941.8	MAP1LC3B	turquoise	0.891040612
ENSG00000171471.5	MAP1LC3B2	turquoise	0.648489868
ENSG00000130479.6	MAP1S	turquoise	0.912294378
ENSG00000078018.15	MAP2	turquoise	0.928520511
ENSG00000169032.5	MAP2K1	turquoise	0.952278106
ENSG00000126934.9	MAP2K2	red	0.922117304
ENSG00000034152.14	MAP2K3	blue	0.707663112
ENSG00000065559.10	MAP2K4	turquoise	0.965317922
ENSG00000137764.15	MAP2K5	turquoise	0.96719725
ENSG00000108984.9	MAP2K6	turquoise	0.689929125
ENSG00000076984.13	MAP2K7	blue	0.784245197
ENSG00000095015.5	MAP3K1	turquoise	0.757421673
ENSG00000130758.3	MAP3K10	turquoise	0.851230526
ENSG00000173327.3	MAP3K11	brown	0.856466397
ENSG00000139625.8	MAP3K12	turquoise	0.949511318
ENSG00000073803.9	MAP3K13	turquoise	0.932073583
ENSG00000006062.9	MAP3K14	turquoise	0.85653046
ENSG00000169967.12	MAP3K2	turquoise	0.912625992
ENSG00000198909.3	MAP3K3	turquoise	0.898621353
ENSG00000085511.15	MAP3K4	turquoise	0.949133302
ENSG00000197442.8	MAP3K5	blue	0.843634943
ENSG00000142733.10	MAP3K6	black	0.848063048
ENSG00000135341.13	MAP3K7	turquoise	0.953133249
ENSG00000156265.11	MAP3K7CL	pink	0.723284469

ENSG00000107968.5	MAP3K8	black	0.835406051
ENSG00000006432.11	MAP3K9	turquoise	0.929915839
ENSG00000047849.17	MAP4	brown	0.727398977
ENSG00000104814.8	MAP4K1	yellow	0.851308009
ENSG00000168067.7	MAP4K2	yellow	0.855829494
ENSG00000011566.10	MAP4K3	turquoise	0.922207496
ENSG00000071054.11	MAP4K4	brown	0.642246242
ENSG00000012983.7	MAP4K5	brown	0.847045815
ENSG00000171533.7	MAP6	turquoise	0.95931484
ENSG00000180834.3	MAP6D1	brown	0.897863837
ENSG00000135525.14	MAP7	brown	0.887200421
ENSG00000116871.11	MAP7D1	turquoise	0.945675095
ENSG00000184368.11	MAP7D2	turquoise	0.92777044
ENSG00000129680.11	MAP7D3	turquoise	0.726936134
ENSG00000164114.14	MAP9	turquoise	0.90501514
ENSG00000100030.10	MAPK1	turquoise	0.958447025
ENSG00000109339.14	MAPK10	blue	0.837149932
ENSG00000185386.10	MAPK11	turquoise	0.865243187
ENSG00000188130.9	MAPK12	turquoise	0.822814596
ENSG00000156711.12	MAPK13	turquoise	0.8955515
ENSG00000112062.6	MAPK14	turquoise	0.96456364
ENSG00000168175.10	MAPK1IP1L	turquoise	0.925169005
ENSG00000102882.7	MAPK3	turquoise	0.914027763
ENSG00000141639.7	MAPK4	turquoise	0.765126918
ENSG00000069956.7	MAPK6	turquoise	0.951646547
ENSG00000166484.15	MAPK7	turquoise	0.888944493
ENSG00000107643.11	MAPK8	turquoise	0.943569278
ENSG00000121653.7	MAPK8IP1	turquoise	0.932346832
ENSG00000008735.10	MAPK8IP2	turquoise	0.910255905
ENSG00000138834.8	MAPK8IP3	turquoise	0.949020822
ENSG00000050748.13	MAPK9	turquoise	0.95673458
ENSG00000119487.12	MAPKAP1	turquoise	0.938741391
ENSG00000162889.6	MAPKAPK2	black	0.876709641
ENSG00000114738.6	MAPKAPK3	green	0.726889208
ENSG00000089022.9	MAPKAPK5	turquoise	0.936139832
ENSG00000234608.3	MAPKAPK5-AS1	turquoise	0.952024784
ENSG00000137802.9	MAPKBP1	yellow	0.931467015
ENSG00000101367.8	MAPRE1	blue	0.863676159
ENSG00000166974.8	MAPRE2	turquoise	0.965282682
ENSG00000084764.6	MAPRE3	turquoise	0.947444724
ENSG00000186868.11	MAPT	turquoise	0.939484427
ENSG00000264589.1	MAPT-AS1	red	0.757526221
ENSG00000155130.5	MARCKS	turquoise	0.801111578
ENSG00000175130.6	MARCKSL1	brown	0.794228061
ENSG00000116141.11	MARK1	turquoise	0.948830224
ENSG00000072518.16	MARK2	turquoise	0.957215747
ENSG00000075413.13	MARK3	turquoise	0.931393936
ENSG00000007047.10	MARK4	turquoise	0.919659254

ENSG00000166986.8	MARS	turquoise	0.942917486
ENSG00000247626.3	MARS2	turquoise	0.851956004
ENSG00000155254.8	MARVELD1	midnightblue	0.679276795
ENSG00000127241.12	MASP1	green	0.61168124
ENSG00000009724.12	MASP2	yellow	0.850728021
ENSG00000105613.5	MAST1	greenyellow	0.901177439
ENSG00000086015.16	MAST2	turquoise	0.886379672
ENSG00000099308.6	MAST3	turquoise	0.919669453
ENSG00000069020.14	MAST4	green	0.88068514
ENSG00000120539.10	MASTL	turquoise	0.885402778
ENSG00000168906.8	MAT2A	blue	0.908554523
ENSG00000038274.12	MAT2B	turquoise	0.956658995
ENSG00000007264.9	MATK	turquoise	0.828900662
ENSG00000132561.9	MATN2	blue	0.708720437
ENSG00000132031.8	MATN3	black	0.546829746
ENSG00000015479.13	MATR3	turquoise	0.977126534
ENSG00000129933.16	MAU2	turquoise	0.90149444
ENSG00000088888.13	MAVS	blue	0.881908427
ENSG00000125952.14	MAX	blue	0.899326618
ENSG00000103495.9	MAZ	turquoise	0.900920325
ENSG00000198125.8	MB	grey	0.064686643
ENSG00000164430.11	MB21D1	black	0.721167302
ENSG00000180611.6	MB21D2	turquoise	0.907733223
ENSG00000141644.13	MBD1	turquoise	0.979444592
ENSG00000134046.7	MBD2	turquoise	0.91498841
ENSG00000071655.13	MBD3	turquoise	0.801507246
ENSG00000129071.5	MBD4	turquoise	0.945893351
ENSG00000204406.7	MBD5	turquoise	0.94819312
ENSG00000166987.10	MBD6	yellow	0.910858947
ENSG00000151332.14	MBIP	turquoise	0.93562796
ENSG00000242600.2	MBL1P	pink	0.732810985
ENSG00000214309.3	MBLAC1	turquoise	0.864873234
ENSG00000176055.9	MBLAC2	turquoise	0.916692728
ENSG00000152601.13	MBNL1	turquoise	0.933997653
ENSG00000229619.3	MBNL1-AS1	turquoise	0.84475555
ENSG00000139793.14	MBNL2	turquoise	0.949725309
ENSG00000172197.9	MBOAT1	blue	0.508632654
ENSG00000143797.7	MBOAT2	blue	0.858468559
ENSG00000125505.12	MBOAT7	turquoise	0.86972207
ENSG00000197971.10	MBP	brown	0.485495412
ENSG00000011258.11	MBTD1	turquoise	0.882876772
ENSG00000140943.12	MBTPS1	turquoise	0.968433969
ENSG00000012174.7	MBTPS2	turquoise	0.859650021
ENSG00000258839.2	MC1R	turquoise	0.850356685
ENSG00000166603.3	MC4R	midnightblue	0.736680612
ENSG00000076706.10	MCAM	brown	0.851349363
ENSG00000100294.8	MCAT	turquoise	0.946160753
ENSG00000171444.13	MCC	green	0.76517146

ENSG00000078070.7	MCCC1	turquoise	0.938583321
ENSG00000243368.2	MCCC1-AS1	yellow	0.794795522
ENSG00000131844.11	MCCC2	turquoise	0.899602261
ENSG00000124370.6	MCEE	blue	0.851315527
ENSG00000101977.15	MCF2	turquoise	0.907752679
ENSG00000126217.16	MCF2L	turquoise	0.897439669
ENSG00000235280.2	MCF2L-AS1	turquoise	0.790055881
ENSG00000053524.7	MCF2L2	turquoise	0.850459057
ENSG00000180398.7	MCFD2	turquoise	0.939654876
ENSG00000143384.8	MCL1	black	0.815659418
ENSG00000073111.9	MCM2	turquoise	0.856806012
ENSG00000112118.13	MCM3	turquoise	0.911940103
ENSG00000160294.6	MCM3AP	turquoise	0.950714906
ENSG00000215424.5	MCM3AP-AS1	turquoise	0.898291673
ENSG00000104738.12	MCM4	turquoise	0.965596027
ENSG00000100297.11	MCM5	blue	0.789913023
ENSG00000076003.4	MCM6	turquoise	0.855137495
ENSG00000166508.13	MCM7	brown	0.6319666
ENSG00000125885.9	MCM8	blue	0.773275469
ENSG00000111877.13	MCM9	turquoise	0.879164916
ENSG00000197771.8	MCMBP	turquoise	0.857436248
ENSG00000178460.13	MCMDC2	turquoise	0.910872254
ENSG00000090674.11	MCOLN1	turquoise	0.938238616
ENSG00000147316.8	MCPH1	turquoise	0.950049498
ENSG00000187778.9	MCRS1	red	0.932256472
ENSG00000175471.15	MCTP1	turquoise	0.890251742
ENSG00000232119.3	MCTS1	turquoise	0.958231403
ENSG00000156026.10	MCU	turquoise	0.934700554
ENSG00000050393.7	MCUR1	blue	0.867372213
ENSG00000137337.10	MDC1	turquoise	0.884681243
ENSG00000112559.9	MDFI	grey	0.407781058
ENSG00000112139.10	MDGA1	midnightblue	0.696502397
ENSG00000014641.13	MDH1	turquoise	0.957162353
ENSG00000138400.8	MDH1B	pink	0.923594372
ENSG00000146701.7	MDH2	turquoise	0.962022402
ENSG00000110492.11	MDK	grey	0.449528937
ENSG00000111554.10	MDM1	pink	0.918298484
ENSG00000135679.17	MDM2	turquoise	0.827882912
ENSG00000198625.8	MDM4	yellow	0.919399496
ENSG00000112159.7	MDN1	turquoise	0.93435432
ENSG00000213920.4	MDP1	turquoise	0.901269809
ENSG00000065833.7	ME1	blue	0.785224577
ENSG00000082212.7	ME2	turquoise	0.888215505
ENSG00000151376.12	ME3	turquoise	0.926964375
ENSG00000124733.3	MEA1	red	0.955005874
ENSG00000163875.11	MEAF6	turquoise	0.97385173
ENSG00000085276.13	MECOM	black	0.699959999
ENSG00000169057.15	MECP2	turquoise	0.977929952

ENSG00000116353.11	MECR	turquoise	0.933274488
ENSG00000125686.7	MED1	turquoise	0.91131819
ENSG00000133398.3	MED10	turquoise	0.905193054
ENSG00000161920.5	MED11	brown	0.78976347
ENSG00000184634.11	MED12	yellow	0.914019633
ENSG00000108510.5	MED13	turquoise	0.881149469
ENSG00000123066.3	MED13L	turquoise	0.883406609
ENSG00000180182.6	MED14	turquoise	0.885229339
ENSG00000234636.1	MED14-AS1	turquoise	0.562629039
ENSG00000099917.13	MED15	turquoise	0.9384536
ENSG00000175221.10	MED16	turquoise	0.87553222
ENSG00000042429.6	MED17	turquoise	0.924989413
ENSG00000130772.9	MED18	turquoise	0.896679719
ENSG00000156603.10	MED19	turquoise	0.928893996
ENSG00000124641.10	MED20	turquoise	0.751908349
ENSG00000152944.4	MED21	turquoise	0.928118657
ENSG00000148297.11	MED22	turquoise	0.87986899
ENSG00000112282.13	MED23	turquoise	0.910150613
ENSG00000008838.13	MED24	turquoise	0.961293632
ENSG00000104973.10	MED25	blue	0.871023395
ENSG00000105085.6	MED26	turquoise	0.89620056
ENSG00000160563.9	MED27	turquoise	0.941988901
ENSG00000118579.7	MED28	turquoise	0.93421485
ENSG00000063322.9	MED29	blue	0.844175109
ENSG00000164758.3	MED30	turquoise	0.796842774
ENSG00000108590.6	MED31	turquoise	0.931580136
ENSG00000136146.10	MED4	turquoise	0.940596713
ENSG00000133997.7	MED6	turquoise	0.969578638
ENSG00000155868.7	MED7	turquoise	0.902859532
ENSG00000159479.12	MED8	turquoise	0.877145452
ENSG00000141026.4	MED9	turquoise	0.941763595
ENSG00000068305.13	MEF2A	turquoise	0.936170015
ENSG00000213999.11	MEF2B	brown	0.614043011
ENSG00000254901.3	MEF2BNB	turquoise	0.939314464
ENSG00000081189.9	MEF2C	turquoise	0.812533282
ENSG00000116604.13	MEF2D	turquoise	0.950337086
ENSG00000214548.10	MEG3	turquoise	0.794293852
ENSG00000258399.2	MEG8	turquoise	0.737877311
ENSG00000223403.3	MEG9	turquoise	0.705033505
ENSG00000145794.12	MEGF10	blue	0.846195279
ENSG00000157890.13	MEGF11	greenyellow	0.633117246
ENSG00000162591.11	MEGF6	brown	0.601412576
ENSG00000105429.8	MEGF8	turquoise	0.93190299
ENSG00000106780.7	MEGF9	blue	0.901554385
ENSG00000197889.5	MEIG1	pink	0.768508707
ENSG00000143995.15	MEIS1	brown	0.84916924
ENSG00000134138.15	MEIS2	turquoise	0.912472476
ENSG00000105419.13	MEIS3	greenyellow	0.686398311

ENSG00000179277.9	MEIS3P1	green	0.760217771
ENSG00000162959.9	MEMO1	turquoise	0.897781241
ENSG00000133895.10	MEN1	turquoise	0.942035335
ENSG00000146834.9	MEPCE	turquoise	0.928833153
ENSG00000153208.12	MERTK	green	0.808164288
ENSG00000140406.2	MESDC1	turquoise	0.856609305
ENSG00000117899.6	MESDC2	turquoise	0.921866646
ENSG00000166823.5	MESP1	turquoise	0.739613237
ENSG00000188095.3	MESP2	turquoise	0.741944199
ENSG00000106484.10	MEST	cyan	0.853341285
ENSG00000164024.7	METAP1	turquoise	0.968650713
ENSG00000172878.9	METAP1D	turquoise	0.853628977
ENSG00000111142.9	METAP2	turquoise	0.894533424
ENSG00000272019.1	Metazoa_SRP	turquoise	0.663403363
ENSG00000103260.4	METRN	grey	0.329657912
ENSG00000176845.8	METRNL	blue	0.564129351
ENSG00000037897.12	METTL1	turquoise	0.880731558
ENSG00000203791.9	METTL10	turquoise	0.931430008
ENSG00000214756.3	METTL12	turquoise	0.804262181
ENSG00000010165.15	METTL13	turquoise	0.9527711
ENSG00000145388.10	METTL14	turquoise	0.943054012
ENSG00000169519.15	METTL15	turquoise	0.912217664
ENSG00000127804.8	METTL16	turquoise	0.848457432
ENSG00000165792.13	METTL17	turquoise	0.888137363
ENSG00000171806.7	METTL18	turquoise	0.728692762
ENSG00000139160.9	METTL20	turquoise	0.924280274
ENSG00000144401.10	METTL21A	turquoise	0.843816459
ENSG00000123427.11	METTL21B	blue	0.784554716
ENSG00000250878.3	METTL21EP	turquoise	0.803167524
ENSG00000067365.10	METTL22	turquoise	0.89534683
ENSG00000181038.9	METTL23	turquoise	0.925130823
ENSG00000053328.8	METTL24	turquoise	0.807422647
ENSG00000127720.3	METTL25	turquoise	0.761656259
ENSG00000087995.11	METTL2A	turquoise	0.922383868
ENSG00000165055.11	METTL2B	turquoise	0.903989222
ENSG00000165819.7	METTL3	turquoise	0.946190882
ENSG00000101574.10	METTL4	turquoise	0.929279904
ENSG00000138382.9	METTL5	turquoise	0.937395119
ENSG00000206562.7	METTL6	turquoise	0.979440127
ENSG00000185432.10	METTL7A	green	0.936545391
ENSG00000170439.5	METTL7B	black	0.654961382
ENSG00000123600.14	METTL8	turquoise	0.900220724
ENSG00000197006.9	METTL9	turquoise	0.908467632
ENSG00000183496.5	MEX3B	turquoise	0.745003033
ENSG00000176624.9	MEX3C	turquoise	0.856361643
ENSG00000181588.15	MEX3D	brown	0.777186448
ENSG00000140259.6	MFAP1	turquoise	0.921052825
ENSG00000232489.1	MFAP1P1	grey	0.585029116

ENSG00000037749.7	MFAP3	blue	0.881248535
ENSG00000198948.7	MFAP3L	blue	0.784009191
ENSG00000166482.7	MFAP4	brown	0.532873922
ENSG00000168958.15	MFF	turquoise	0.94578495
ENSG00000140545.10	MFGE8	green	0.52945618
ENSG00000147324.6	MFHAS1	cyan	0.899498056
ENSG00000163975.7	MFI2	grey	0.533843352
ENSG00000228109.1	MFI2-AS1	yellow	0.663009488
ENSG00000171109.14	MFN1	turquoise	0.879709057
ENSG00000116688.12	MFN2	turquoise	0.952441916
ENSG00000100060.13	MFNG	black	0.745896053
ENSG00000118855.14	MFSD1	turquoise	0.776698881
ENSG00000109736.10	MFSD10	turquoise	0.830759863
ENSG00000092931.7	MFSD11	turquoise	0.933732773
ENSG00000161091.8	MFSD12	turquoise	0.888051097
ENSG00000168389.13	MFSD2A	grey	0.562381148
ENSG00000167700.4	MFSD3	turquoise	0.883816451
ENSG00000174514.8	MFSD4	turquoise	0.922239094
ENSG00000182544.8	MFSD5	turquoise	0.883398966
ENSG00000151690.10	MFSD6	turquoise	0.869914015
ENSG00000169026.8	MFSD7	blue	0.708418164
ENSG00000164073.5	MFSD8	turquoise	0.939094956
ENSG00000135953.6	MFSD9	turquoise	0.889180653
ENSG00000174197.12	MGA	turquoise	0.89172675
ENSG00000137463.4	MGARP	turquoise	0.648762461
ENSG00000131446.11	MGAT1	blue	0.880577528
ENSG00000168282.4	MGAT2	turquoise	0.729675364
ENSG00000128268.11	MGAT3	turquoise	0.925930416
ENSG00000071073.8	MGAT4A	turquoise	0.962953306
ENSG00000161013.12	MGAT4B	turquoise	0.942371485
ENSG00000182050.9	MGAT4C	green	0.814275547
ENSG00000152127.4	MGAT5	turquoise	0.933104155
ENSG00000167889.8	MGAT5B	turquoise	0.820563798
ENSG00000217702.1	MGC10955	grey	0.312274726
ENSG00000198408.9	MGEA5	turquoise	0.936501837
ENSG00000074416.9	MGLL	green	0.743277162
ENSG00000125871.9	MGME1	blue	0.85455401
ENSG00000170430.9	MGMT	red	0.819503051
ENSG00000111341.5	MGP	grey	0.296713579
ENSG00000102858.8	MGRN1	turquoise	0.930488056
ENSG00000008394.8	MGST1	green	0.736445604
ENSG00000085871.4	MGST2	magenta	0.696252073
ENSG00000143198.8	MGST3	turquoise	0.9004885
ENSG00000261857.2	MIA	grey	0.427522203
ENSG00000154305.12	MIA3	turquoise	0.98069768
ENSG00000225783.2	MIAT	turquoise	0.818836111
ENSG00000101752.7	MIB1	turquoise	0.876407942
ENSG00000197530.8	MIB2	turquoise	0.874552459

ENSG00000204520.8	MICA	blue	0.637257291
ENSG00000135596.13	MICAL1	yellow	0.919520444
ENSG00000133816.9	MICAL2	turquoise	0.917231345
ENSG00000243156.3	MICAL3	yellow	0.916793101
ENSG00000100139.9	MICALL1	brown	0.846081071
ENSG00000164877.14	MICALL2	blue	0.733431659
ENSG00000233265.1	MICF	turquoise	0.530960172
ENSG00000107745.12	MICU1	turquoise	0.973522334
ENSG00000165487.9	MICU2	turquoise	0.933408737
ENSG00000155970.7	MICU3	turquoise	0.909438925
ENSG00000101871.10	MID1	green	0.883786249
ENSG00000165175.11	MID1IP1	brown	0.772691815
ENSG00000238123.1	MID1IP1-AS1	green	0.650214887
ENSG00000167470.8	MIDN	black	0.781493637
ENSG00000100335.8	MIEF1	turquoise	0.937750103
ENSG00000177427.8	MIEF2	turquoise	0.945234055
ENSG00000141741.7	MIEN1	turquoise	0.82196729
ENSG00000198160.10	MIER1	turquoise	0.897838185
ENSG00000105556.6	MIER2	turquoise	0.862330852
ENSG00000155545.15	MIER3	turquoise	0.874548354
ENSG00000240972.1	MIF	turquoise	0.812881598
ENSG00000125457.9	MIF4GD	turquoise	0.766712928
ENSG00000116691.6	MIIP	turquoise	0.847978295
ENSG00000271605.1	MILR1	magenta	0.87401131
ENSG00000268654.1	MIMT1	turquoise	0.835548015
ENSG00000170854.13	MINA	blue	0.919516739
ENSG00000141503.11	MINK1	turquoise	0.961623624
ENSG00000173436.9	MINOS1	red	0.930807423
ENSG00000107789.11	MINPP1	turquoise	0.863242811
ENSG00000164654.11	MIOS	turquoise	0.947674013
ENSG00000027001.7	MIPEP	turquoise	0.882577856
ENSG00000233325.3	MIPEPP3	pink	0.810637465
ENSG00000221170.1	MIR1304	turquoise	0.56861845
ENSG00000225206.4	MIR137HG	turquoise	0.802437029
ENSG00000269936.2	MIR145	grey	0.33211751
ENSG00000247095.2	MIR210HG	grey	0.372912172
ENSG00000207955.2	MIR219-2	brown	0.697765107
ENSG00000186594.8	MIR22HG	blue	0.656202257
ENSG00000267519.2	MIR24-2	black	0.712085652
ENSG00000226380.3	MIR29B1	turquoise	0.755687249
ENSG00000265452.1	MIR3682	grey	0.206049158
ENSG00000264329.1	MIR3911	turquoise	0.681087207
ENSG00000227671.3	MIR3916	blue	0.761050769
ENSG00000266509.1	MIR3934	turquoise	0.522918101
ENSG00000263597.1	MIR3936	turquoise	0.713735784
ENSG00000268471.3	MIR4453	blue	0.843279868
ENSG00000247516.3	MIR4458HG	turquoise	0.904292677
ENSG00000198868.3	MIR4461	grey	-0.22910849

ENSG00000260083.1	MIR4519	turquoise	0.725814813
ENSG00000267532.2	MIR497HG	green	0.585703147
ENSG00000207770.1	MIR568	blue	0.62800313
ENSG00000207650.1	MIR570	grey	0.415031949
ENSG00000236901.3	MIR600HG	turquoise	0.921201021
ENSG00000207733.1	MIR637	yellow	0.56677691
ENSG00000207554.1	MIR647	yellow	0.602584669
ENSG00000176840.7	MIR7-3HG	turquoise	0.803525335
ENSG00000211574.1	MIR770	turquoise	0.855909071
ENSG00000260778.2	MIR940	turquoise	0.60910483
ENSG00000167842.11	MIS12	turquoise	0.812985843
ENSG00000159055.3	MIS18A	turquoise	0.942682976
ENSG00000129534.9	MIS18BP1	turquoise	0.852409921
ENSG00000158411.6	MITD1	turquoise	0.871575662
ENSG00000187098.10	MITF	brown	0.872608483
ENSG00000155438.7	MKI67IP	turquoise	0.913815431
ENSG00000125863.13	MKKS	turquoise	0.959368975
ENSG00000196588.10	MKL1	turquoise	0.924698535
ENSG00000186260.12	MKL2	turquoise	0.89865379
ENSG00000128585.13	MKLN1	blue	0.847377125
ENSG00000231721.2	MKLN1-AS1	turquoise	0.881796569
ENSG00000236753.1	MKLN1-AS2	turquoise	0.737273625
ENSG00000079277.15	MKNK1	yellow	0.906975937
ENSG00000269956.1	MKNK1-AS1	turquoise	0.772699885
ENSG00000099875.10	MKNK2	cyan	0.765956786
ENSG00000133606.6	MKRN1	turquoise	0.979749424
ENSG00000075975.11	MKRN2	turquoise	0.951676513
ENSG00000179455.6	MKRN3	brown	0.80794341
ENSG00000225849.1	MKRN7P	turquoise	0.609662776
ENSG00000011143.12	MKS1	turquoise	0.867551551
ENSG00000100427.11	MLC1	green	0.833042176
ENSG00000110917.3	MLEC	turquoise	0.908544584
ENSG00000178053.13	MLF1	turquoise	0.865244693
ENSG00000089693.6	MLF2	turquoise	0.955091805
ENSG00000076242.10	MLH1	turquoise	0.948663067
ENSG00000119684.11	MLH3	turquoise	0.904733052
ENSG00000143674.6	MLK4	turquoise	0.895126131
ENSG00000168404.8	MLKL	black	0.777818796
ENSG00000130382.7	MLLT1	turquoise	0.915569518
ENSG00000078403.12	MLLT10	turquoise	0.927115626
ENSG00000238151.1	MLLT10P1	turquoise	0.623503025
ENSG00000213190.2	MLLT11	turquoise	0.929537492
ENSG00000171843.11	MLLT3	turquoise	0.941437155
ENSG00000130396.16	MLLT4	turquoise	0.847029045
ENSG00000108292.12	MLLT6	turquoise	0.891118216
ENSG00000167965.13	MLST8	turquoise	0.918074013
ENSG00000091436.12	MLTK	blue	0.810879897
ENSG00000108788.7	MLX	turquoise	0.959235867

ENSG00000175727.9	MLXIP	turquoise	0.897763767
ENSG00000009950.11	MLXIPL	turquoise	0.689596148
ENSG00000103150.4	MLYCD	blue	0.863169471
ENSG00000151611.9	MMAA	turquoise	0.912339427
ENSG00000139428.7	MMAB	turquoise	0.922469949
ENSG00000132763.10	MMACHC	turquoise	0.853261877
ENSG00000168288.8	MMADHC	turquoise	0.941281626
ENSG00000108960.3	MMD	turquoise	0.856678844
ENSG00000136297.10	MMD2	green	0.608334471
ENSG00000196549.6	MME	midnightblue	0.892573612
ENSG00000169446.4	MMGT1	blue	0.81293128
ENSG00000157227.8	MMP14	magenta	0.763857534
ENSG00000102996.4	MMP15	brown	0.694303582
ENSG00000156103.11	MMP16	greenyellow	0.762753826
ENSG00000198598.2	MMP17	turquoise	0.78429008
ENSG00000087245.8	MMP2	magenta	0.603185443
ENSG00000125966.8	MMP24	turquoise	0.913505057
ENSG00000126005.11	MMP24-AS1	turquoise	0.887210092
ENSG00000129270.11	MMP28	green	0.641615579
ENSG00000173269.9	MMRN2	black	0.853272274
ENSG00000155229.16	MMS19	turquoise	0.971010359
ENSG00000169184.5	MN1	turquoise	0.886557098
ENSG00000020426.6	MNAT1	turquoise	0.932932736
ENSG00000163563.7	MNDA	magenta	0.790856498
ENSG00000070444.10	MNT	turquoise	0.905737012
ENSG00000165943.4	MOAP1	turquoise	0.955685401
ENSG00000114978.13	MOB1A	blue	0.926778555
ENSG00000173542.4	MOB1B	turquoise	0.897801356
ENSG00000182208.8	MOB2	turquoise	0.88574658
ENSG00000172081.9	MOB3A	blue	0.856256579
ENSG00000120162.9	MOB3B	turquoise	0.873197946
ENSG00000142961.10	MOB3C	blue	0.803610903
ENSG00000115540.10	MOB4	turquoise	0.929474983
ENSG00000168314.13	MOBP	brown	0.544417926
ENSG00000124615.13	MOCS1	blue	0.859204483
ENSG00000164172.14	MOCS2	turquoise	0.954908892
ENSG00000124217.3	MOCS3	turquoise	0.942574956
ENSG00000204655.7	MOG	brown	0.930240985
ENSG00000115275.7	MOGS	turquoise	0.931168209
ENSG00000080823.17	MOK	pink	0.917048017
ENSG00000164077.9	MON1A	turquoise	0.916432804
ENSG00000103111.10	MON1B	blue	0.927200388
ENSG00000061987.10	MON2	turquoise	0.903779128
ENSG00000133422.8	MORC2	turquoise	0.892503595
ENSG00000159256.8	MORC3	turquoise	0.84979789
ENSG00000133131.10	MORC4	blue	0.804794902
ENSG00000185787.10	MORF4L1	turquoise	0.950717114
ENSG00000218283.2	MORF4L1P1	turquoise	0.909705161

ENSG00000123562.12	MORF4L2	turquoise	0.963285014
ENSG00000116151.9	MORN1	pink	0.913945393
ENSG00000188010.8	MORN2	turquoise	0.879286336
ENSG00000139714.8	MORN3	pink	0.912594585
ENSG00000171160.13	MORN4	turquoise	0.946979692
ENSG00000101928.8	MOSPD1	turquoise	0.880240115
ENSG00000130150.7	MOSPD2	brown	0.892933561
ENSG00000106330.7	MOSPD3	red	0.897869622
ENSG00000155363.14	MOV10	yellow	0.8894519
ENSG00000079931.10	MOXD1	brown	0.483480224
ENSG00000060762.14	MPC1	blue	0.823311145
ENSG00000143158.6	MPC2	turquoise	0.9422006
ENSG00000129255.10	MPDU1	turquoise	0.885693512
ENSG00000107186.12	MPDZ	blue	0.875860247
ENSG00000197629.5	MPEG1	magenta	0.66872967
ENSG00000103152.7	MPG	turquoise	0.892921842
ENSG00000124383.4	MPHOSPH10	turquoise	0.936017511
ENSG00000135698.5	MPHOSPH6	turquoise	0.953285541
ENSG00000196199.9	MPHOSPH8	turquoise	0.939996109
ENSG00000051825.10	MPHOSPH9	turquoise	0.914368475
ENSG00000178802.13	MPI	turquoise	0.94305226
ENSG00000168303.6	MPLKIP	turquoise	0.918080261
ENSG00000008382.11	MPND	turquoise	0.909344124
ENSG00000005381.6	MPO	grey	0.467347608
ENSG00000130830.10	MPP1	turquoise	0.962830848
ENSG00000108852.10	MPP2	turquoise	0.909920559
ENSG00000161647.14	MPP3	turquoise	0.872056641
ENSG00000072415.4	MPP5	turquoise	0.903847322
ENSG00000105926.11	MPP6	turquoise	0.930090588
ENSG00000150054.14	MPP7	turquoise	0.892306637
ENSG00000154889.12	MPPE1	turquoise	0.912221289
ENSG00000186732.9	MPPED1	turquoise	0.723137991
ENSG00000066382.12	MPPED2	turquoise	0.918334028
ENSG00000133030.15	MPRIP	turquoise	0.93557704
ENSG00000128309.12	MPST	brown	0.694833947
ENSG00000115204.10	MPV17	turquoise	0.957435025
ENSG00000156968.8	MPV17L	turquoise	0.916546152
ENSG00000254858.5	MPV17L2	turquoise	0.822249033
ENSG00000158887.11	MPZ	turquoise	0.611047398
ENSG00000197965.7	MPZL1	blue	0.911371322
ENSG00000149573.4	MPZL2	black	0.77383054
ENSG00000160588.5	MPZL3	turquoise	0.859780046
ENSG00000153029.10	MR1	turquoise	0.800442674
ENSG00000135324.5	MRAP2	turquoise	0.866250238
ENSG00000158186.8	MRAS	turquoise	0.793136692
ENSG00000011028.9	MRC2	green	0.673719748
ENSG00000020922.8	MRE11A	pink	0.892576702
ENSG00000118242.11	MREG	turquoise	0.763132489

ENSG00000179010.10	MRFAP1	turquoise	0.945446946
ENSG00000178988.10	MRFAP1L1	turquoise	0.953282067
ENSG00000101189.5	MRGBP	turquoise	0.909046029
ENSG00000172935.8	MRGPRF	grey	0.481559525
ENSG00000037757.9	MRI1	turquoise	0.871869239
ENSG00000129282.8	MRM1	turquoise	0.899592136
ENSG00000134042.8	MRO	green	0.854055713
ENSG00000179832.13	MROH1	turquoise	0.883906212
ENSG00000204839.4	MROH6	grey	0.745957811
ENSG00000184313.15	MROH7	green	0.728332396
ENSG00000101353.10	MROH8	turquoise	0.856476782
ENSG00000173141.4	MRP63	turquoise	0.927983838
ENSG00000169288.13	MRPL1	turquoise	0.93469969
ENSG00000159111.8	MRPL10	turquoise	0.965827036
ENSG00000174547.9	MRPL11	turquoise	0.929589744
ENSG00000262814.2	MRPL12	red	0.927113436
ENSG00000172172.3	MRPL13	blue	0.849791145
ENSG00000180992.5	MRPL14	turquoise	0.910213852
ENSG00000137547.4	MRPL15	turquoise	0.919584163
ENSG00000166902.4	MRPL16	turquoise	0.870846271
ENSG00000158042.8	MRPL17	turquoise	0.942669209
ENSG00000112110.5	MRPL18	cyan	0.938676497
ENSG00000115364.9	MRPL19	blue	0.881255534
ENSG00000112651.7	MRPL2	turquoise	0.944108867
ENSG00000242485.1	MRPL20	turquoise	0.918632831
ENSG00000197345.8	MRPL21	turquoise	0.868829313
ENSG00000082515.12	MRPL22	turquoise	0.936791746
ENSG00000214026.6	MRPL23	red	0.90055122
ENSG00000226416.1	MRPL23-AS1	yellow	0.847580889
ENSG00000143314.8	MRPL24	red	0.962946647
ENSG00000108826.11	MRPL27	turquoise	0.865799418
ENSG00000086504.11	MRPL28	turquoise	0.890676172
ENSG00000114686.4	MRPL3	turquoise	0.964089282
ENSG00000106591.3	MRPL32	turquoise	0.969360648
ENSG00000243147.3	MRPL33	turquoise	0.893546481
ENSG00000130312.2	MRPL34	turquoise	0.888954054
ENSG00000132313.10	MRPL35	turquoise	0.954881754
ENSG00000171421.8	MRPL36	turquoise	0.897015754
ENSG00000116221.11	MRPL37	turquoise	0.955215551
ENSG00000204316.8	MRPL38	turquoise	0.910617309
ENSG00000154719.9	MRPL39	turquoise	0.907641559
ENSG00000105364.9	MRPL4	turquoise	0.928029895
ENSG00000185608.4	MRPL40	red	0.953492348
ENSG00000182154.7	MRPL41	turquoise	0.667159669
ENSG00000198015.8	MRPL42	turquoise	0.873572193
ENSG00000055950.12	MRPL43	turquoise	0.918158545
ENSG00000135900.3	MRPL44	turquoise	0.957841391
ENSG00000174100.5	MRPL45	turquoise	0.965109033

ENSG00000228782.3	MRPL45P2	turquoise	0.840427779
ENSG00000259494.1	MRPL46	turquoise	0.880172119
ENSG00000136522.9	MRPL47	turquoise	0.883580302
ENSG00000175581.9	MRPL48	turquoise	0.905814869
ENSG00000149792.4	MRPL49	turquoise	0.961990486
ENSG00000136897.6	MRPL50	turquoise	0.947593122
ENSG00000111639.3	MRPL51	red	0.959176889
ENSG00000172590.14	MRPL52	red	0.960254894
ENSG00000204822.6	MRPL53	turquoise	0.851899757
ENSG00000183617.4	MRPL54	red	0.952884281
ENSG00000162910.14	MRPL55	turquoise	0.905086303
ENSG00000143436.6	MRPL9	turquoise	0.963683599
ENSG00000048544.5	MRPS10	turquoise	0.96844865
ENSG00000181991.11	MRPS11	turquoise	0.934373937
ENSG00000128626.7	MRPS12	red	0.924037928
ENSG00000120333.4	MRPS14	blue	0.879269247
ENSG00000116898.7	MRPS15	turquoise	0.909659242
ENSG00000182180.9	MRPS16	turquoise	0.926551074
ENSG00000239789.1	MRPS17	turquoise	0.953111454
ENSG00000096080.7	MRPS18A	turquoise	0.915978066
ENSG00000204568.7	MRPS18B	turquoise	0.919701173
ENSG00000163319.6	MRPS18C	turquoise	0.902559689
ENSG00000122140.6	MRPS2	turquoise	0.915626123
ENSG00000187145.10	MRPS21	turquoise	0.940385405
ENSG00000175110.7	MRPS22	turquoise	0.925389263
ENSG00000181610.8	MRPS23	turquoise	0.948927467
ENSG00000062582.9	MRPS24	red	0.958762425
ENSG00000131368.3	MRPS25	turquoise	0.975288437
ENSG00000125901.5	MRPS26	red	0.955288062
ENSG00000113048.12	MRPS27	turquoise	0.975182283
ENSG00000147586.5	MRPS28	blue	0.851091236
ENSG00000112996.5	MRPS30	turquoise	0.963149649
ENSG00000102738.6	MRPS31	turquoise	0.926553368
ENSG00000243406.2	MRPS31P5	turquoise	0.764491903
ENSG00000090263.11	MRPS33	turquoise	0.938918374
ENSG00000074071.9	MRPS34	red	0.944524641
ENSG00000061794.8	MRPS35	turquoise	0.9546224
ENSG00000134056.7	MRPS36	turquoise	0.936946401
ENSG00000144029.7	MRPS5	turquoise	0.940471252
ENSG00000243927.1	MRPS6	cyan	0.877423022
ENSG00000125445.6	MRPS7	turquoise	0.921018893
ENSG00000135972.4	MRPS9	turquoise	0.96145513
ENSG00000148187.13	MRRF	turquoise	0.909875206
ENSG00000124532.10	MRS2	turquoise	0.95644848
ENSG00000053372.4	MRTO4	turquoise	0.938225892
ENSG00000072952.14	MRVI1	green	0.776053093
ENSG00000177112.3	MRVI1-AS1	turquoise	0.694115436
ENSG00000166928.6	MS4A14	magenta	0.713734842

ENSG00000110079.12	MS4A4A	magenta	0.871261985
ENSG00000110077.10	MS4A6A	magenta	0.833156229
ENSG00000166927.8	MS4A7	magenta	0.8782283
ENSG00000188981.6	MSANTD1	turquoise	0.880091743
ENSG00000120458.5	MSANTD2	yellow	0.903982626
ENSG00000066697.10	MSANTD3	turquoise	0.854920824
ENSG00000170903.6	MSANTD4	blue	0.890219252
ENSG00000095002.8	MSH2	turquoise	0.963536671
ENSG00000113318.9	MSH3	turquoise	0.942703489
ENSG00000204410.10	MSH5	yellow	0.907082939
ENSG00000116062.10	MSH6	turquoise	0.886149563
ENSG00000153944.6	MSI2	green	0.928689791
ENSG00000188895.7	MSL1	turquoise	0.970800906
ENSG00000174579.3	MSL2	turquoise	0.873696467
ENSG00000005302.13	MSL3	turquoise	0.931141333
ENSG00000224287.2	MSL3P1	turquoise	0.745752609
ENSG00000052802.8	MSMO1	turquoise	0.881070051
ENSG00000147065.12	MSN	blue	0.838679066
ENSG00000175806.10	MSRA	turquoise	0.919190868
ENSG00000198736.7	MSRB1	blue	0.74335403
ENSG00000148450.8	MSRB2	red	0.944669749
ENSG00000174099.6	MSRB3	green	0.81614183
ENSG00000166343.5	MSS51	yellow	0.899299395
ENSG00000173531.11	MST1	yellow	0.908687135
ENSG00000186715.6	MST1L	yellow	0.654981414
ENSG00000186301.7	MST1P2	yellow	0.796031118
ENSG00000134602.11	MST4	turquoise	0.8684514
ENSG00000125459.10	MSTO1	turquoise	0.912570587
ENSG00000203761.4	MSTO2P	turquoise	0.85610346
ENSG00000198899.2	MT-ATP6	cyan	-0.821743403
ENSG00000228253.1	MT-ATP8	red	-0.615889144
ENSG00000198804.2	MT-CO1	turquoise	-0.808953733
ENSG00000198712.1	MT-CO2	turquoise	-0.69223583
ENSG00000198938.2	MT-CO3	cyan	-0.762953345
ENSG00000198727.2	MT-CYB	turquoise	-0.799594782
ENSG00000198888.2	MT-ND1	turquoise	-0.821296027
ENSG00000198763.3	MT-ND2	turquoise	-0.689855622
ENSG00000198840.2	MT-ND3	turquoise	-0.660854711
ENSG00000198886.2	MT-ND4	turquoise	-0.837472596
ENSG00000212907.2	MT-ND4L	red	-0.757851843
ENSG00000198786.2	MT-ND5	turquoise	-0.814474727
ENSG00000198695.2	MT-ND6	turquoise	-0.822244079
ENSG00000211459.2	MT-RNR1	turquoise	-0.604321045
ENSG00000210082.2	MT-RNR2	turquoise	-0.683241786
ENSG00000210127.1	MT-TA	grey	-0.366603429
ENSG00000210140.1	MT-TC	turquoise	-0.564907684
ENSG00000210154.1	MT-TD	grey	-0.2341894
ENSG00000210194.1	MT-TE	turquoise	-0.546358368

ENSG00000210049.1	MT-TF	grey	-0.278023231
ENSG00000210164.1	MT-TG	grey	-0.220774298
ENSG00000210176.1	MT-TH	grey	-0.196485538
ENSG00000210100.1	MT-TI	grey	-0.287743925
ENSG00000210156.1	MT-TK	grey	-0.263091391
ENSG00000209082.1	MT-TL1	grey	-0.26303767
ENSG00000210191.1	MT-TL2	grey	-0.118763705
ENSG00000210112.1	MT-TM	grey	-0.259601567
ENSG00000210135.1	MT-TN	turquoise	-0.503097777
ENSG00000210196.2	MT-TP	turquoise	-0.461365222
ENSG00000210107.1	MT-TQ	grey	-0.289681754
ENSG00000210174.1	MT-TR	grey	-0.270729346
ENSG00000210151.2	MT-TS1	turquoise	-0.597831935
ENSG00000210184.1	MT-TS2	grey	-0.168806774
ENSG00000210195.2	MT-TT	turquoise	-0.452142059
ENSG00000210077.1	MT-TV	grey	-0.21192174
ENSG00000210117.1	MT-TW	grey	-0.268270492
ENSG00000210144.1	MT-TY	turquoise	-0.486100478
ENSG00000205362.6	MT1A	black	0.727293167
ENSG00000205361.4	MT1DP	grey	0.36030075
ENSG00000169715.10	MT1E	black	0.34583679
ENSG00000198417.5	MT1F	grey	0.19875473
ENSG00000125144.9	MT1G	grey	0.10607897
ENSG00000205358.3	MT1H	grey	0.115934618
ENSG00000260549.1	MT1L	black	0.746925299
ENSG00000205364.3	MT1M	black	0.574997918
ENSG00000187193.8	MT1X	black	0.639225352
ENSG00000233929.1	MT1XP1	grey	0.284409758
ENSG00000125148.6	MT2A	black	0.654460463
ENSG00000162840.3	MT2P1	grey	0.183500257
ENSG00000087250.4	MT3	green	0.616874251
ENSG00000182979.13	MTA1	turquoise	0.916064695
ENSG00000149480.2	MTA2	green	0.741470355
ENSG00000057935.9	MTA3	turquoise	0.968554803
ENSG00000099810.14	MTAP	turquoise	0.881790845
ENSG00000248527.1	MTATP6P1	grey	-0.282239189
ENSG00000270307.1	MTATP6P2	grey	-0.072379749
ENSG00000240409.1	MTATP8P1	grey	-0.349641706
ENSG00000229604.2	MTATP8P2	grey	-0.222605512
ENSG00000172167.3	MTBP	turquoise	0.726125137
ENSG00000137409.14	MTCH1	turquoise	0.946678863
ENSG00000109919.5	MTCH2	turquoise	0.899238879
ENSG00000214827.5	MTCP1	red	0.902666548
ENSG00000147649.5	MTDH	turquoise	0.906550482
ENSG00000127989.9	MTERF	turquoise	0.86822344
ENSG00000156469.4	MTERFD1	turquoise	0.930627614
ENSG00000122085.12	MTERFD2	turquoise	0.952000603
ENSG00000120832.5	MTERFD3	yellow	0.921838868

ENSG00000188786.9	MTF1	turquoise	0.829566045
ENSG00000143033.13	MTF2	turquoise	0.928814164
ENSG00000103707.5	MTFMT	turquoise	0.965375651
ENSG00000242114.1	MTFP1	turquoise	0.574804921
ENSG00000066855.11	MTFR1	brown	0.903556287
ENSG00000117640.13	MTFR1L	turquoise	0.972915523
ENSG00000148824.14	MTG1	brown	0.740876259
ENSG00000101181.13	MTG2	turquoise	0.923534651
ENSG00000100714.11	MTHFD1	turquoise	0.957018294
ENSG00000120254.11	MTHFD1L	turquoise	0.871681071
ENSG00000065911.7	MTHFD2	black	0.771725118
ENSG00000163738.14	MTHFD2L	turquoise	0.87269961
ENSG00000177000.6	MTHFR	turquoise	0.953002519
ENSG00000136371.5	MTHFS	turquoise	0.821327225
ENSG00000103248.13	MTHFSD	turquoise	0.844820398
ENSG00000085760.10	MTIF2	turquoise	0.921510261
ENSG00000122033.10	MTIF3	turquoise	0.895948613
ENSG00000132749.6	MTL5	turquoise	0.847819527
ENSG00000171100.10	MTM1	green	0.890843363
ENSG00000063601.12	MTMR1	turquoise	0.948643328
ENSG00000166912.12	MTMR10	blue	0.748230426
ENSG00000014914.15	MTMR11	pink	0.847073648
ENSG00000150712.6	MTMR12	turquoise	0.954312314
ENSG00000163719.14	MTMR14	turquoise	0.873655966
ENSG00000087053.14	MTMR2	turquoise	0.863714773
ENSG00000100330.11	MTMR3	turquoise	0.86765716
ENSG00000108389.5	MTMR4	turquoise	0.956685237
ENSG00000139505.10	MTMR6	turquoise	0.958491004
ENSG00000003987.9	MTMR7	turquoise	0.939066052
ENSG00000104643.5	MTMR9	turquoise	0.972324054
ENSG00000220785.3	MTMR9LP	yellow	0.78324994
ENSG00000225972.1	MTND1P23	grey	-0.130521887
ENSG00000225630.1	MTND2P28	grey	-0.218239823
ENSG00000247627.2	MTND4P12	grey	-0.260043921
ENSG00000232177.1	MTND4P24	grey	-0.1836612
ENSG00000248923.1	MTND5P11	grey	-0.283838481
ENSG00000249119.1	MTND6P4	grey	-0.349556897
ENSG00000135297.11	MTO1	turquoise	0.946084249
ENSG00000198793.8	MTOR	turquoise	0.951083131
ENSG00000107951.8	MTPAP	turquoise	0.928062797
ENSG00000116984.8	MTR	yellow	0.912151327
ENSG00000120662.11	MTRF1	turquoise	0.921149529
ENSG00000112031.11	MTRF1L	turquoise	0.680436605
ENSG00000256618.1	MTRNR2L1	grey	-0.083422006
ENSG00000256045.1	MTRNR2L10	grey	-0.175416912
ENSG00000269028.2	MTRNR2L12	grey	-0.224905999
ENSG00000271043.1	MTRNR2L2	grey	-0.233972445
ENSG00000255823.1	MTRNR2L8	grey	-0.175050515

ENSG00000255633.3	MTRNR2L9	grey	-0.188167434
ENSG00000124275.10	MTRR	turquoise	0.900507754
ENSG00000170873.14	MTSS1	turquoise	0.911613352
ENSG00000132613.10	MTSS1L	green	0.734127323
ENSG00000129422.9	MTUS1	brown	0.85306377
ENSG00000132938.14	MTUS2	turquoise	0.8782814
ENSG00000173171.10	MTX1	turquoise	0.884381381
ENSG00000236675.1	MTX1P1	turquoise	0.8693939
ENSG00000128654.9	MTX2	turquoise	0.963131068
ENSG00000185499.12	MUC1	turquoise	0.640274832
ENSG00000176945.12	MUC20	turquoise	0.755374209
ENSG00000184956.11	MUC6	grey	0.437911991
ENSG00000090432.5	MUL1	turquoise	0.925839782
ENSG00000160953.10	MUM1	turquoise	0.956445879
ENSG00000170681.6	MURC	brown	0.772265135
ENSG00000172732.7	MUS81	turquoise	0.913614915
ENSG00000272573.1	MUSTN1	grey	0.198221203
ENSG00000146085.7	MUT	blue	0.913296987
ENSG00000132781.13	MUTYH	yellow	0.839949241
ENSG00000141971.8	MVB12A	blue	0.785169912
ENSG00000196814.10	MVB12B	brown	0.876592947
ENSG00000167508.6	MVD	turquoise	0.866735444
ENSG00000110921.7	MVK	turquoise	0.846637628
ENSG00000013364.14	MVP	blue	0.706171843
ENSG00000157601.9	MX1	turquoise	0.700051733
ENSG00000183486.8	MX2	black	0.590515586
ENSG00000059728.6	MXD1	cyan	0.874243382
ENSG00000213347.6	MXD3	yellow	0.826125298
ENSG00000123933.10	MXD4	turquoise	0.710259965
ENSG00000119950.16	MXI1	blue	0.909851375
ENSG00000182534.9	MXRA7	turquoise	0.900035818
ENSG00000162576.12	MXRA8	red	0.651408899
ENSG00000179820.11	MYADM	turquoise	0.906155314
ENSG00000185105.4	MYADML2	turquoise	0.858185694
ENSG00000118513.14	MYB	turquoise	0.788098748
ENSG00000132382.10	MYBBP1A	turquoise	0.94574005
ENSG00000185697.12	MYBL1	blue	0.781943035
ENSG00000136997.10	MYC	black	0.815368336
ENSG00000005810.13	MYCBP2	turquoise	0.954407613
ENSG00000136449.9	MYCBPAP	pink	0.877072275
ENSG00000116990.9	MYCL	turquoise	0.827046213
ENSG00000134323.10	MYCN	turquoise	0.762113622
ENSG00000120279.6	MYCT1	black	0.669389521
ENSG00000172936.8	MYD88	blue	0.79913159
ENSG00000104177.13	MYEF2	turquoise	0.948436872
ENSG00000172428.6	MYEOV2	red	0.801388539
ENSG00000133026.8	MYH10	turquoise	0.947054963
ENSG00000133392.12	MYH11	grey	0.372955919

ENSG00000105357.11	MYH14	brown	0.817499857
ENSG00000144821.5	MYH15	blue	0.682770679
ENSG00000109063.10	MYH3	yellow	0.868672252
ENSG00000092054.12	MYH7	turquoise	0.681679245
ENSG00000078814.11	MYH7B	brown	0.849944689
ENSG00000100345.16	MYH9	black	0.871443248
ENSG00000101608.8	MYL12A	black	0.882791203
ENSG00000118680.8	MYL12B	turquoise	0.933029501
ENSG00000111245.10	MYL2	grey	0.062245384
ENSG00000160808.5	MYL3	grey	0.318535394
ENSG00000215375.2	MYL5	turquoise	0.813933012
ENSG00000092841.14	MYL6	turquoise	0.733657656
ENSG00000196465.6	MYL6B	turquoise	0.898009638
ENSG00000106631.4	MYL7	grey	0.298861827
ENSG00000101335.5	MYL9	grey	0.46442214
ENSG00000007944.10	MYLIP	brown	0.886960934
ENSG00000065534.14	MYLK	green	0.757517592
ENSG00000239523.1	MYLK-AS1	pink	0.79575954
ENSG00000101306.6	MYLK2	grey	0.285959272
ENSG00000145949.8	MYLK4	turquoise	0.841605439
ENSG00000085274.11	MYNN	turquoise	0.939884854
ENSG00000145555.10	MYO10	green	0.774941375
ENSG00000266714.2	MYO15B	yellow	0.810199811
ENSG00000196535.10	MYO18A	brown	0.833887345
ENSG00000141140.12	MYO19	yellow	0.934464094
ENSG00000128641.13	MYO1B	grey	0.523967634
ENSG00000197879.10	MYO1C	black	0.831617762
ENSG00000176658.12	MYO1D	brown	0.929883781
ENSG00000157483.4	MYO1E	brown	0.802345458
ENSG00000142347.12	MYO1F	magenta	0.961051289
ENSG00000095777.10	MYO3A	turquoise	0.790181906
ENSG00000197535.10	MYO5A	turquoise	0.954240535
ENSG00000238245.2	MYO5BP2	grey	0.165475222
ENSG00000128833.8	MYO5C	pink	0.87768824
ENSG00000196586.9	MYO6	turquoise	0.802708888
ENSG00000137474.15	MYO7A	magenta	0.799087063
ENSG00000066933.11	MYO9A	turquoise	0.947563178
ENSG00000099331.9	MYO9B	brown	0.908969043
ENSG00000138119.12	MYOF	black	0.841993332
ENSG00000101605.8	MYOM1	blue	0.759716184
ENSG00000036448.5	MYOM2	turquoise	0.806189316
ENSG00000120729.5	MYOT	brown	0.685195048
ENSG00000177791.11	MYOZ1	green	0.650485152
ENSG00000164591.9	MYOZ3	turquoise	0.814349052
ENSG00000176182.5	MYPOP	turquoise	0.9103141
ENSG00000124920.9	MYRF	brown	0.925982441
ENSG00000170011.9	MYRIP	turquoise	0.737397945
ENSG00000162601.5	MYSM1	turquoise	0.895224949

ENSG00000196132.7	MYT1	turquoise	0.765246663
ENSG00000186487.13	MYT1L	turquoise	0.908612571
ENSG00000099326.4	MZF1	turquoise	0.84154985
ENSG00000204899.5	MZT1	turquoise	0.89093533
ENSG00000173272.9	MZT2A	turquoise	0.798257622
ENSG00000152082.9	MZT2B	red	0.926612186
ENSG00000102921.3	N4BP1	turquoise	0.95388777
ENSG00000139597.12	N4BP2L1	turquoise	0.934356316
ENSG00000244754.4	N4BP2L2	turquoise	0.935844197
ENSG00000145911.5	N4BP3	turquoise	0.830574578
ENSG00000156239.7	N6AMT1	blue	0.796239379
ENSG00000150456.6	N6AMT2	red	0.867183086
ENSG00000184640.13	NA	black	0.787244312
ENSG00000168385.13	NA	blue	0.883373989
ENSG00000173926.5	NA	blue	0.628826503
ENSG00000122545.13	NA	blue	0.767831979
ENSG00000165406.11	NA	blue	0.922520159
ENSG00000138758.7	NA	blue	0.818601994
ENSG00000183291.11	NA	blue	0.912393399
ENSG00000145416.9	NA	brown	0.808055436
ENSG00000108387.10	NA	brown	0.652122421
ENSG00000164402.9	NA	brown	0.931888541
ENSG00000186522.10	NA	brown	0.813188426
ENSG00000186205.8	NA	green	0.776345865
ENSG00000117791.11	NA	green	0.861047679
ENSG00000173838.7	NA	pink	0.876653465
ENSG00000099785.6	NA	turquoise	0.752397391
ENSG00000100167.15	NA	turquoise	0.912975418
ENSG00000144583.4	NA	turquoise	0.86467929
ENSG00000198060.5	NA	turquoise	0.917360555
ENSG00000184702.13	NA	turquoise	0.870419073
ENSG00000145495.10	NA	turquoise	0.968791602
ENSG00000125354.18	NA	turquoise	0.83635565
ENSG00000136536.10	NA	turquoise	0.922002385
ENSG00000139266.5	NA	turquoise	0.938837702
ENSG00000183654.8	NA	turquoise	0.817167372
ENSG00000102030.11	NAA10	turquoise	0.880926207
ENSG00000164134.8	NAA15	turquoise	0.924962498
ENSG00000172766.14	NAA16	blue	0.819315852
ENSG00000173418.7	NAA20	turquoise	0.961780575
ENSG00000111300.5	NAA25	turquoise	0.937993525
ENSG00000139977.9	NAA30	turquoise	0.952551009
ENSG00000135040.11	NAA35	turquoise	0.913131636
ENSG00000128534.3	NAA38	turquoise	0.859844604
ENSG00000110583.8	NAA40	turquoise	0.894237434
ENSG00000121579.8	NAA50	turquoise	0.919578088
ENSG00000122390.13	NAA60	turquoise	0.892027944
ENSG00000138744.10	NAAA	turquoise	0.912691752

ENSG00000077616.6	NAALAD2	turquoise	0.860819468
ENSG00000138386.12	NAB1	turquoise	0.744361627
ENSG00000166886.8	NAB2	turquoise	0.903445227
ENSG00000139579.8	NABP2	turquoise	0.94239762
ENSG00000196531.6	NACA	red	0.939003247
ENSG00000136274.8	NACAD	brown	0.870269083
ENSG00000160877.5	NACC1	turquoise	0.916316836
ENSG00000148411.3	NACC2	green	0.86419812
ENSG00000008130.11	NADK	turquoise	0.92320188
ENSG00000152620.8	NADK2	green	0.917274747
ENSG00000172890.7	NADSYN1	yellow	0.95726691
ENSG00000159593.10	NAE1	turquoise	0.945785306
ENSG00000145414.4	NAF1	turquoise	0.943121314
ENSG00000198951.7	NAGA	blue	0.849593089
ENSG00000124357.8	NAGK	turquoise	0.912941642
ENSG00000108784.5	NAGLU	brown	0.794676549
ENSG00000103174.7	NAGPA	turquoise	0.870364518
ENSG00000161653.6	NAGS	black	0.726838282
ENSG00000171169.8	NAIF1	turquoise	0.910545079
ENSG00000102452.11	NALCN	turquoise	0.948113855
ENSG00000105835.7	NAMPT	black	0.849154814
ENSG00000188613.5	NANOS1	turquoise	0.877116589
ENSG00000187556.6	NANOS3	grey	0.293232361
ENSG00000170191.4	NANP	turquoise	0.914152299
ENSG00000095380.10	NANS	turquoise	0.904713523
ENSG00000187109.9	NAP1L1	turquoise	0.891918774
ENSG00000186462.7	NAP1L2	turquoise	0.950052386
ENSG00000186310.9	NAP1L3	turquoise	0.928517104
ENSG00000205531.8	NAP1L4	turquoise	0.941393045
ENSG00000177432.6	NAP1L5	turquoise	0.925789555
ENSG00000204118.1	NAP1L6	turquoise	0.562758071
ENSG00000105402.3	NAPA	turquoise	0.9501431
ENSG00000268061.1	NAPA-AS1	green	0.611168829
ENSG00000125814.13	NAPB	turquoise	0.934347312
ENSG00000161048.7	NAPEPLD	turquoise	0.871768249
ENSG00000134265.8	NAPG	turquoise	0.973309259
ENSG00000147813.11	NAPRT1	turquoise	0.620678674
ENSG00000131400.3	NAPSA	blue	0.599735879
ENSG00000131401.7	NAPSB	magenta	0.722008473
ENSG00000141562.13	NARF	turquoise	0.934898262
ENSG00000103245.9	NARFL	turquoise	0.879880407
ENSG00000128915.7	NARG2	turquoise	0.861944609
ENSG00000134440.7	NARS	turquoise	0.965445261
ENSG00000137513.5	NARS2	turquoise	0.919538114
ENSG00000132780.12	NASP	brown	0.83910482
ENSG00000135372.4	NAT10	turquoise	0.947252714
ENSG00000090971.4	NAT14	turquoise	0.835262323
ENSG00000243477.1	NAT6	turquoise	0.863091667

ENSG00000185818.7	NAT8L	turquoise	0.879029891
ENSG00000109065.7	NAT9	turquoise	0.921297825
ENSG00000134369.11	NAV1	blue	0.834483769
ENSG00000166833.15	NAV2	blue	0.871602242
ENSG00000067798.9	NAV3	turquoise	0.90145867
ENSG00000151779.8	NBAS	turquoise	0.968038759
ENSG00000172915.14	NBEA	turquoise	0.931549995
ENSG00000160796.12	NBEAL2	yellow	0.839674687
ENSG00000158747.9	NBL1	red	0.763028367
ENSG00000104320.9	NBN	turquoise	0.954559668
ENSG00000219481.6	NBPF1	turquoise	0.71393462
ENSG00000163386.16	NBPF10	green	0.766048816
ENSG00000186275.7	NBPF12	yellow	0.887591561
ENSG00000243452.3	NBPF15	turquoise	0.877904024
ENSG00000227001.2	NBPF2P	turquoise	0.669253171
ENSG00000142794.14	NBPF3	turquoise	0.713117403
ENSG00000168614.13	NBPF9	turquoise	0.853774924
ENSG00000188554.9	NBR1	blue	0.930952212
ENSG00000198496.6	NBR2	turquoise	0.656814828
ENSG00000104490.13	NCALD	greenyellow	0.829160701
ENSG00000149294.12	NCAM1	turquoise	0.928519032
ENSG00000227487.3	NCAM1-AS1	yellow	0.849753677
ENSG00000154654.10	NCAM2	turquoise	0.906779978
ENSG00000130287.9	NCAN	green	0.617424227
ENSG00000010292.8	NCAPD2	brown	0.884742223
ENSG00000151503.8	NCAPD3	turquoise	0.929559105
ENSG00000146918.15	NCAPG2	turquoise	0.872207691
ENSG00000025770.14	NCAPH2	turquoise	0.936552188
ENSG00000136937.8	NCBP1	turquoise	0.929092799
ENSG00000114503.6	NCBP2	turquoise	0.94085102
ENSG00000270170.1	NCBP2-AS2	turquoise	0.918586873
ENSG00000020129.11	NCDN	turquoise	0.872933191
ENSG00000144959.5	NCEH1	turquoise	0.925660254
ENSG00000116701.10	NCF2	magenta	0.870562041
ENSG00000100365.10	NCF4	magenta	0.91001494
ENSG00000158092.2	NCK1	turquoise	0.847398402
ENSG00000071051.9	NCK2	cyan	0.829622167
ENSG00000061676.10	NCKAP1	turquoise	0.961573877
ENSG00000123338.8	NCKAP1L	magenta	0.964109031
ENSG00000176771.11	NCKAP5	brown	0.892715194
ENSG00000167566.12	NCKAP5L	yellow	0.941712097
ENSG00000213672.3	NCKIPSD	turquoise	0.948152584
ENSG00000115053.11	NCL	turquoise	0.920976787
ENSG00000125912.6	NCLN	turquoise	0.919832171
ENSG00000084676.11	NCOA1	turquoise	0.969217047
ENSG00000140396.8	NCOA2	turquoise	0.949410928
ENSG00000124151.14	NCOA3	turquoise	0.765418739
ENSG00000138293.15	NCOA4	blue	0.943892643

ENSG00000124160.7	NCOA5	turquoise	0.903591512
ENSG00000198646.9	NCOA6	turquoise	0.956787526
ENSG00000111912.14	NCOA7	turquoise	0.935343716
ENSG00000141027.16	NCOR1	turquoise	0.939740734
ENSG00000196498.9	NCOR2	blue	0.81816045
ENSG00000188211.4	NCR3LG1	turquoise	0.860810829
ENSG00000107130.6	NCS1	turquoise	0.834610914
ENSG00000162736.11	NCSTN	turquoise	0.898068092
ENSG00000058804.10	NDC1	turquoise	0.899903324
ENSG00000072864.8	NDE1	brown	0.89012199
ENSG00000166579.11	NDEL1	turquoise	0.950823589
ENSG00000131507.9	NDFIP1	turquoise	0.975366834
ENSG00000102471.9	NDFIP2	turquoise	0.93004825
ENSG00000182636.4	NDN	turquoise	0.904377184
ENSG00000185115.4	NDNL2	turquoise	0.953693179
ENSG00000188566.7	NDOR1	yellow	0.95487834
ENSG00000124479.8	NDP	green	0.762683696
ENSG00000104419.10	NDRG1	brown	0.755417524
ENSG00000165795.16	NDRG2	green	0.724469351
ENSG00000101079.16	NDRG3	turquoise	0.967671575
ENSG00000103034.10	NDRG4	turquoise	0.926991154
ENSG00000070614.10	NDST1	turquoise	0.888929332
ENSG00000166507.12	NDST2	turquoise	0.945925568
ENSG00000164100.7	NDST3	turquoise	0.892662654
ENSG00000125356.6	NDUFA1	red	0.938856157
ENSG00000130414.7	NDUFA10	turquoise	0.978648288
ENSG00000174886.8	NDUFA11	red	0.972914369
ENSG00000184752.8	NDUFA12	turquoise	0.917643798
ENSG00000186010.14	NDUFA13	red	0.956072565
ENSG00000131495.4	NDUFA2	red	0.955062672
ENSG00000170906.11	NDUFA3	red	0.903544021
ENSG00000259262.1	NDUFA3P4	grey	0.618609706
ENSG00000189043.5	NDUFA4	turquoise	0.908343289
ENSG00000185633.6	NDUFA4L2	brown	0.437820953
ENSG00000128609.10	NDUFA5	turquoise	0.91978503
ENSG00000184983.5	NDUFA6	turquoise	0.890999876
ENSG00000237037.5	NDUFA6-AS1	turquoise	0.884995933
ENSG00000267855.1	NDUFA7	red	0.939984964
ENSG00000119421.5	NDUFA8	turquoise	0.920691296
ENSG00000139180.6	NDUFA9	turquoise	0.945024722
ENSG00000004779.5	NDUFAB1	turquoise	0.941104853
ENSG00000137806.4	NDUFAF1	turquoise	0.947786102
ENSG00000164182.6	NDUFAF2	turquoise	0.918340445
ENSG00000178057.10	NDUFAF3	red	0.940011034
ENSG00000123545.5	NDUFAF4	turquoise	0.922497901
ENSG00000236411.1	NDUFAF4P3	turquoise	0.754007235
ENSG00000101247.13	NDUFAF5	turquoise	0.956644973
ENSG00000156170.8	NDUFAF6	turquoise	0.939522743

ENSG00000003509.11	NDUFAF7	turquoise	0.948615371
ENSG00000183648.5	NDUFB1	red	0.924078414
ENSG00000140990.10	NDUFB10	red	0.943972187
ENSG00000147123.10	NDUFB11	red	0.955118164
ENSG00000090266.8	NDUFB2	red	0.938072395
ENSG00000240889.1	NDUFB2-AS1	turquoise	0.880444236
ENSG00000119013.4	NDUFB3	turquoise	0.880809611
ENSG00000065518.7	NDUFB4	turquoise	0.929247388
ENSG00000136521.8	NDUFB5	turquoise	0.912606739
ENSG00000165264.6	NDUFB6	turquoise	0.941330906
ENSG00000099795.2	NDUFB7	red	0.960085304
ENSG00000166136.11	NDUFB8	red	0.929682264
ENSG00000147684.3	NDUFB9	red	0.940384402
ENSG00000109390.7	NDUFC1	turquoise	0.919814758
ENSG00000151366.8	NDUFC2	turquoise	0.949471344
ENSG00000023228.9	NDUFS1	turquoise	0.95759528
ENSG00000158864.8	NDUFS2	turquoise	0.967775947
ENSG00000213619.5	NDUFS3	turquoise	0.853029237
ENSG00000164258.7	NDUFS4	turquoise	0.91510646
ENSG00000168653.6	NDUFS5	red	0.932296271
ENSG00000145494.7	NDUFS6	red	0.95624754
ENSG00000115286.15	NDUFS7	red	0.936513517
ENSG00000110717.6	NDUFS8	red	0.926884545
ENSG00000167792.7	NDUFV1	turquoise	0.940067424
ENSG00000178127.8	NDUFV2	turquoise	0.941964663
ENSG00000267809.1	NDUFV2P1	turquoise	0.729904139
ENSG00000160194.13	NDUFV3	turquoise	0.905863227
ENSG00000245532.4	NEAT1	blue	0.521331462
ENSG00000078114.14	NEBL	green	0.915005205
ENSG00000231920.1	NEBL-AS1	turquoise	0.594341178
ENSG00000123119.7	NECAB1	turquoise	0.912520719
ENSG00000103154.5	NECAB2	turquoise	0.880141135
ENSG00000125967.12	NECAB3	turquoise	0.90042902
ENSG00000089818.12	NECAP1	turquoise	0.968172874
ENSG00000157191.15	NECAP2	blue	0.835231571
ENSG00000139350.7	NEDD1	blue	0.848338283
ENSG00000069869.11	NEDD4	blue	0.791185682
ENSG00000049759.12	NEDD4L	turquoise	0.920305836
ENSG00000129559.8	NEDD8	turquoise	0.924021778
ENSG00000111859.12	NEDD9	black	0.808932389
ENSG00000100285.9	NEFH	turquoise	0.854285319
ENSG00000104725.9	NEFL	turquoise	0.922309122
ENSG00000104722.9	NEFM	turquoise	0.848402837
ENSG00000172260.9	NEGR1	turquoise	0.8863907
ENSG00000140398.9	NEIL1	yellow	0.947684642
ENSG00000154328.11	NEIL2	turquoise	0.872008279
ENSG00000137601.11	NEK1	blue	0.880002924
ENSG00000163491.12	NEK10	turquoise	0.808371821

ENSG00000136098.12	NEK3	brown	0.857334856
ENSG00000114904.8	NEK4	blue	0.895834503
ENSG00000119408.12	NEK6	blue	0.763867721
ENSG00000151414.10	NEK7	brown	0.604592352
ENSG00000160602.9	NEK8	green	0.546436325
ENSG00000119638.8	NEK9	yellow	0.933534755
ENSG00000185049.8	NELFA	turquoise	0.912018544
ENSG00000188986.4	NELFB	turquoise	0.921885038
ENSG00000101158.8	NELFCD	turquoise	0.892312753
ENSG00000204356.7	NELFE	turquoise	0.901586581
ENSG00000165973.13	NELL1	greenyellow	0.871388377
ENSG00000184613.6	NELL2	turquoise	0.926032518
ENSG00000165525.13	NEMF	turquoise	0.932703025
ENSG00000117691.5	NENF	red	0.915238952
ENSG00000067141.12	NEO1	blue	0.922493266
ENSG00000132688.10	NES	blue	0.511573264
ENSG00000173848.14	NET1	brown	0.609626643
ENSG00000166342.14	NETO1	turquoise	0.875959123
ENSG00000171208.5	NETO2	turquoise	0.896317875
ENSG00000204386.6	NEU1	turquoise	0.942137219
ENSG00000204099.7	NEU4	brown	0.515931344
ENSG00000107954.6	NEURL	turquoise	0.843183272
ENSG00000214357.4	NEURL1B	greenyellow	0.706626406
ENSG00000124257.6	NEURL2	turquoise	0.763084548
ENSG00000215041.5	NEURL4	turquoise	0.969443568
ENSG00000178403.3	NEUROG2	grey	0.093252913
ENSG00000162614.14	NEXN	midnightblue	0.790304905
ENSG00000196712.12	NF1	turquoise	0.943681559
ENSG00000186575.13	NF2	turquoise	0.902349628
ENSG00000163531.11	NFASC	brown	0.892130033
ENSG00000131196.13	NFATC1	green	0.758386711
ENSG00000101096.15	NFATC2	turquoise	0.63205146
ENSG00000176953.6	NFATC2IP	turquoise	0.926204263
ENSG00000072736.14	NFATC3	blue	0.847026206
ENSG00000100968.9	NFATC4	green	0.590340428
ENSG00000082641.11	NFE2L1	turquoise	0.941676852
ENSG00000116044.11	NFE2L2	green	0.805243242
ENSG00000050344.8	NFE2L3	yellow	0.840576275
ENSG00000162599.11	NFIA	green	0.878775694
ENSG00000147862.10	NFIB	green	0.782529403
ENSG00000141905.13	NFIC	blue	0.86114152
ENSG00000165030.3	NFIL3	cyan	0.854403411
ENSG00000008441.12	NFIX	brown	0.804244288
ENSG00000109320.7	NFKB1	blue	0.892690808
ENSG00000077150.13	NFKB2	black	0.866820213
ENSG00000100906.6	NFKBIA	black	0.801825732
ENSG00000104825.12	NFKBIB	blue	0.718740548
ENSG00000167604.9	NFKBID	yellow	0.738781337

ENSG00000146232.10	NFKBIE	cyan	0.740132944
ENSG00000204498.6	NFKBIL1	turquoise	0.911004641
ENSG00000144802.7	NFKBIZ	black	0.783498148
ENSG00000170322.10	NFRKB	turquoise	0.948242736
ENSG00000244005.8	NFS1	turquoise	0.951172622
ENSG00000169599.8	NFU1	turquoise	0.919931356
ENSG00000086102.14	NFX1	turquoise	0.968896027
ENSG00000170448.7	NFXL1	turquoise	0.905218252
ENSG00000001167.10	NFYA	blue	0.870156329
ENSG00000120837.3	NFYB	turquoise	0.790424141
ENSG00000066136.15	NFYC	turquoise	0.963409316
ENSG00000165553.4	NGB	greenyellow	0.664523401
ENSG00000129460.11	NGDN	turquoise	0.937269097
ENSG00000066248.10	NGEF	turquoise	0.835919978
ENSG00000134259.3	NGF	greenyellow	0.60692659
ENSG00000064300.4	NGFR	grey	0.343806789
ENSG00000166681.9	NGFRAP1	turquoise	0.938110273
ENSG00000151092.12	NGLY1	turquoise	0.9007817
ENSG00000182768.7	NGRN	turquoise	0.925693808
ENSG00000187736.8	NHEJ1	turquoise	0.848345054
ENSG00000171786.5	NHLH1	turquoise	0.837294625
ENSG00000187566.3	NHLRC1	turquoise	0.908362809
ENSG00000196865.4	NHLRC2	turquoise	0.749297628
ENSG00000188811.8	NHLRC3	turquoise	0.913487901
ENSG00000145912.4	NHP2	turquoise	0.930260011
ENSG00000100138.9	NHP2L1	turquoise	0.951946027
ENSG00000188158.10	NHS	green	0.735857563
ENSG00000135540.7	NHSL1	green	0.869901361
ENSG00000204131.6	NHSL2	turquoise	0.811155116
ENSG00000145029.7	NICN1	turquoise	0.956448014
ENSG00000116962.10	NID1	green	0.6688328
ENSG00000087303.12	NID2	brown	0.712017521
ENSG00000196290.10	NIF3L1	turquoise	0.967879041
ENSG00000177453.3	NIM1	turquoise	0.933684621
ENSG00000100503.19	NIN	turquoise	0.866942967
ENSG00000131669.5	NINJ1	magenta	0.669536345
ENSG00000171840.7	NINJ2	brown	0.879095609
ENSG00000101004.10	NINL	turquoise	0.864514841
ENSG00000132603.9	NIP7	turquoise	0.897697842
ENSG00000170113.11	NIPA1	brown	0.827813823
ENSG00000140157.10	NIPA2	turquoise	0.939090662
ENSG00000104361.5	NIPAL2	turquoise	0.882270006
ENSG00000001461.12	NIPAL3	brown	0.870406977
ENSG00000172548.10	NIPAL4	brown	0.877023779
ENSG00000164190.12	NIPBL	turquoise	0.870000047
ENSG00000184117.7	NIPSNAP1	turquoise	0.961132298
ENSG00000136783.9	NIPSNAP3A	blue	0.873007549
ENSG00000165028.7	NIPSNAP3B	turquoise	0.924190029

ENSG00000010322.11	NISCH	turquoise	0.961908918
ENSG00000158793.9	NIT1	turquoise	0.957295306
ENSG00000114021.7	NIT2	turquoise	0.939432395
ENSG00000084628.5	NKAIN1	brown	0.665791227
ENSG00000188580.9	NKAIN2	brown	0.802121289
ENSG00000185942.7	NKAIN3	green	0.742561373
ENSG00000101198.10	NKAIN4	green	0.489286155
ENSG00000101882.5	NKAP	turquoise	0.91407769
ENSG00000189134.3	NKAPL	turquoise	0.677499843
ENSG00000140807.4	NKD1	brown	0.827216103
ENSG00000145506.9	NKD2	brown	0.497988968
ENSG00000105374.5	NKG7	grey	0.125767015
ENSG00000197885.6	NKIRAS1	turquoise	0.96133434
ENSG00000168256.13	NKIRAS2	blue	0.874669217
ENSG00000186416.8	NKRF	turquoise	0.930244787
ENSG00000114857.13	NKTR	yellow	0.950016229
ENSG00000136352.13	NKX2-1	greenyellow	0.727923703
ENSG00000125820.5	NKX2-2	brown	0.662123761
ENSG00000167034.9	NKX3-1	cyan	0.81346552
ENSG00000148826.6	NKX6-2	brown	0.781562279
ENSG00000073536.13	NLE1	turquoise	0.944967373
ENSG00000169760.13	NLGN1	turquoise	0.899021194
ENSG00000169992.5	NLGN2	turquoise	0.944409003
ENSG00000196338.8	NLGN3	turquoise	0.788898451
ENSG00000146938.10	NLGN4X	turquoise	0.901852922
ENSG00000165246.8	NLGN4Y	grey	0.261243718
ENSG00000087095.8	NLK	turquoise	0.936497082
ENSG00000123213.18	NLN	turquoise	0.909246182
ENSG00000167984.12	NLRC3	turquoise	0.83759133
ENSG00000140853.11	NLRC5	blue	0.690463188
ENSG00000091592.11	NLRP1	turquoise	0.918485459
ENSG00000022556.11	NLRP2	turquoise	0.648143399
ENSG00000162711.12	NLRP3	turquoise	0.738923983
ENSG00000160703.11	NLRX1	turquoise	0.855091005
ENSG00000197696.5	NMB	brown	0.56053363
ENSG00000169251.8	NMD3	blue	0.910371201
ENSG00000239672.3	NME1	turquoise	0.901915035
ENSG00000243678.7	NME1-NME2	red	0.961589352
ENSG00000103024.3	NME3	red	0.890535895
ENSG00000103202.8	NME4	turquoise	0.746104318
ENSG00000112981.3	NME5	turquoise	0.904531448
ENSG00000172113.4	NME6	blue	0.926244107
ENSG00000143156.9	NME7	pink	0.879629162
ENSG00000181322.9	NME9	pink	0.94873942
ENSG00000123609.6	NMI	black	0.803777703
ENSG00000173614.9	NMNAT1	turquoise	0.875809533
ENSG00000157064.6	NMNAT2	turquoise	0.937772943
ENSG00000163864.10	NMNAT3	turquoise	0.907029655

ENSG00000153406.9	NMRAL1	turquoise	0.909862841
ENSG00000106733.16	NMRK1	turquoise	0.845188371
ENSG00000136448.7	NMT1	turquoise	0.944767065
ENSG00000152465.13	NMT2	turquoise	0.952644267
ENSG00000053438.7	NNAT	turquoise	0.823078144
ENSG00000112992.12	NNT	turquoise	0.925644939
ENSG00000248092.3	NNT-AS1	turquoise	0.874731675
ENSG00000084092.6	NOA1	turquoise	0.976162202
ENSG00000141101.8	NOB1	turquoise	0.913750902
ENSG00000188976.6	NOC2L	turquoise	0.96457701
ENSG00000173145.7	NOC3L	blue	0.864830487
ENSG00000184967.2	NOC4L	turquoise	0.901965953
ENSG00000106100.6	NOD1	black	0.870497317
ENSG00000183691.4	NOG	turquoise	0.828917177
ENSG00000115761.11	NOL10	turquoise	0.866145875
ENSG00000130935.5	NOL11	turquoise	0.925204823
ENSG00000100101.13	NOL12	turquoise	0.897886477
ENSG00000140939.10	NOL3	brown	0.653968194
ENSG00000101746.11	NOL4	turquoise	0.873817701
ENSG00000165271.12	NOL6	turquoise	0.939986177
ENSG00000225921.2	NOL7	turquoise	0.894423188
ENSG00000198000.7	NOL8	turquoise	0.899848577
ENSG00000162408.10	NOL9	turquoise	0.896375015
ENSG00000166197.12	NOLC1	turquoise	0.958065676
ENSG00000146909.3	NOM1	turquoise	0.928823408
ENSG00000103512.10	NOMO1	turquoise	0.934649064
ENSG00000103226.13	NOMO3	turquoise	0.672735278
ENSG00000147140.11	NONO	turquoise	0.958390927
ENSG00000182117.4	NOP10	turquoise	0.882180986
ENSG00000087269.11	NOP14	turquoise	0.920578858
ENSG00000249673.2	NOP14-AS1	turquoise	0.940656344
ENSG00000048162.15	NOP16	turquoise	0.900776579
ENSG00000111641.6	NOP2	turquoise	0.841971529
ENSG00000101361.10	NOP56	turquoise	0.928805884
ENSG00000055044.6	NOP58	turquoise	0.897785932
ENSG00000196943.8	NOP9	turquoise	0.921653514
ENSG00000089250.14	NOS1	midnightblue	0.928132351
ENSG00000198929.8	NOS1AP	turquoise	0.778392866
ENSG00000007171.12	NOS2	turquoise	0.712660115
ENSG00000164867.6	NOS3	black	0.825317131
ENSG00000142546.9	NOSIP	red	0.972951757
ENSG00000163072.10	NOSTRIN	black	0.58237204
ENSG00000148400.9	NOTCH1	brown	0.718590637
ENSG00000134250.13	NOTCH2	green	0.920758042
ENSG00000213240.7	NOTCH2NL	green	0.769005756
ENSG00000074181.4	NOTCH3	grey	0.41409374
ENSG00000204301.5	NOTCH4	black	0.784391201
ENSG00000185269.7	NOTUM	midnightblue	0.67835758

ENSG00000136999.4	NOV	turquoise	0.764180516
ENSG00000139910.15	NOVA1	turquoise	0.920567677
ENSG00000104967.6	NOVA2	turquoise	0.934017851
ENSG00000188747.4	NOXA1	turquoise	0.770284076
ENSG00000196408.7	NOXO1	turquoise	0.475678134
ENSG00000130751.5	NPAS1	greenyellow	0.778864338
ENSG00000170485.12	NPAS2	turquoise	0.926185099
ENSG00000151322.14	NPAS3	green	0.858295415
ENSG00000174576.4	NPAS4	grey	0.201036722
ENSG00000149308.12	NPAT	turquoise	0.928867524
ENSG00000141458.8	NPC1	brown	0.919012867
ENSG00000119655.4	NPC2	blue	0.797951099
ENSG00000107281.5	NPDC1	turquoise	0.875094292
ENSG00000215440.7	NPEPL1	yellow	0.895894454
ENSG00000141279.11	NPEPPS	turquoise	0.965699136
ENSG00000139574.7	NPFF	grey	0.698842842
ENSG00000144061.8	NPHP1	pink	0.963938484
ENSG00000113971.14	NPHP3	yellow	0.889335745
ENSG00000131697.13	NPHP4	turquoise	0.939473288
ENSG00000183426.11	NPIPA1	yellow	0.604599177
ENSG00000254852.4	NPIPA2	grey	0.628654816
ENSG00000183793.9	NPIPA5	grey	0.345603704
ENSG00000254206.1	NPIPB11	yellow	0.532428937
ENSG00000196436.7	NPIPB15	grey	0.157591772
ENSG00000169246.12	NPIPB3	yellow	0.843292288
ENSG00000185864.12	NPIPB4	yellow	0.85692301
ENSG00000243716.6	NPIPB5	yellow	0.86039256
ENSG00000198156.6	NPIPB6	grey	0.673938638
ENSG00000188599.13	NPIPP1	grey	0.80727
ENSG00000135838.9	NPL	green	0.741772047
ENSG00000182446.9	NPLOC4	turquoise	0.951117799
ENSG00000181163.9	NPM1	turquoise	0.929331362
ENSG00000158806.9	NPM2	turquoise	0.885169997
ENSG00000107833.6	NPM3	turquoise	0.867254106
ENSG00000168743.8	NPNT	turquoise	0.683405822
ENSG00000175206.6	NPPA	grey	0.109973932
ENSG00000242349.1	NPPA-AS1	yellow	0.694897865
ENSG00000163273.3	NPPC	brown	0.661581657
ENSG00000169418.9	NPR1	blue	0.431224019
ENSG00000159899.10	NPR2	turquoise	0.921935749
ENSG00000114388.8	NPRL2	turquoise	0.938683499
ENSG00000103148.11	NPRL3	turquoise	0.860474181
ENSG00000156642.12	NPTN	turquoise	0.934679601
ENSG00000171246.5	NPTX1	turquoise	0.874380553
ENSG00000106236.3	NPTX2	turquoise	0.764038274
ENSG00000221890.2	NPTXR	greenyellow	0.921338107
ENSG00000122585.3	NPY	midnightblue	0.857178797
ENSG00000164128.2	NPY1R	turquoise	0.862562979

ENSG00000164129.7	NPY5R	turquoise	0.888164533
ENSG00000181019.8	NQO1	blue	0.5346624
ENSG00000124588.15	NQO2	turquoise	0.858333799
ENSG00000126368.5	NR1D1	turquoise	0.703948978
ENSG00000174738.8	NR1D2	turquoise	0.915758808
ENSG00000131408.9	NR1H2	red	0.910666302
ENSG00000025434.14	NR1H3	red	0.717004705
ENSG00000143257.7	NR1I3	turquoise	0.797228199
ENSG00000120798.12	NR2C1	turquoise	0.888913207
ENSG00000177463.11	NR2C2	turquoise	0.916852894
ENSG00000184162.10	NR2C2AP	turquoise	0.888882239
ENSG00000112333.7	NR2E1	green	0.875078144
ENSG00000175745.7	NR2F1	green	0.742804149
ENSG00000237187.4	NR2F1-AS1	green	0.848953012
ENSG00000185551.8	NR2F2	grey	0.349780004
ENSG00000160113.5	NR2F6	turquoise	0.83375516
ENSG00000113580.10	NR3C1	turquoise	0.84519066
ENSG00000151623.10	NR3C2	blue	0.85670802
ENSG00000123358.15	NR4A1	grey	0.347720554
ENSG00000153234.9	NR4A2	grey	0.320726441
ENSG00000119508.13	NR4A3	turquoise	0.539147563
ENSG00000148200.12	NR6A1	yellow	0.901932423
ENSG00000198435.2	NRARP	grey	0.267862988
ENSG00000213281.4	NRAS	turquoise	0.857782797
ENSG00000148572.10	NRBF2	turquoise	0.943023155
ENSG00000115216.9	NRBP1	turquoise	0.955149845
ENSG00000185189.11	NRBP2	yellow	0.873520441
ENSG00000091129.15	NRCAM	blue	0.885047802
ENSG00000078618.15	NRD1	turquoise	0.964191976
ENSG00000119720.13	NRDE2	turquoise	0.953318985
ENSG00000134986.9	NREP	turquoise	0.686965235
ENSG00000106459.10	NRF1	turquoise	0.937775469
ENSG00000158458.15	NRG2	turquoise	0.881345658
ENSG00000185737.8	NRG3	blue	0.867603472
ENSG00000169752.12	NRG4	pink	0.901663361
ENSG00000154146.8	NRGN	turquoise	0.683706576
ENSG00000180530.5	NRIP1	turquoise	0.800586737
ENSG00000053702.10	NRIP2	midnightblue	0.918018238
ENSG00000175352.6	NRIP3	turquoise	0.896692743
ENSG00000129535.8	NRL	turquoise	0.707069002
ENSG00000137404.10	NRM	black	0.788225869
ENSG00000124785.4	NRN1	greenyellow	0.593624928
ENSG00000188038.3	NRN1L	turquoise	0.661731641
ENSG00000099250.13	NRP1	blue	0.689210769
ENSG00000118257.12	NRP2	blue	0.689543509
ENSG00000174004.5	NRROS	magenta	0.859022771
ENSG00000152954.7	NRSN1	turquoise	0.891098842
ENSG00000125841.8	NRSN2	turquoise	0.937020251

ENSG00000179915.16	NRXN1	blue	0.745223503
ENSG00000110076.14	NRXN2	turquoise	0.912154461
ENSG00000021645.13	NRXN3	turquoise	0.930456093
ENSG00000164346.5	NSA2	turquoise	0.896311702
ENSG00000165671.14	NSD1	turquoise	0.93786202
ENSG00000147383.6	NSDHL	turquoise	0.919494915
ENSG00000073969.14	NSF	turquoise	0.948284737
ENSG00000088833.13	NSFL1C	turquoise	0.950265669
ENSG00000168824.10	NSG1	turquoise	0.928906008
ENSG00000170091.6	NSG2	turquoise	0.905807003
ENSG00000117697.10	NSL1	blue	0.901440402
ENSG00000035681.3	NSMAF	turquoise	0.966481059
ENSG00000169189.12	NSMCE1	turquoise	0.804084249
ENSG00000156831.3	NSMCE2	turquoise	0.911805356
ENSG00000107672.10	NSMCE4A	turquoise	0.930615412
ENSG00000165802.15	NSMF	turquoise	0.852671697
ENSG00000126653.11	NSRP1	brown	0.858276172
ENSG00000037474.10	NSUN2	turquoise	0.884276781
ENSG00000178694.5	NSUN3	turquoise	0.929457426
ENSG00000117481.6	NSUN4	turquoise	0.943489257
ENSG00000130305.12	NSUN5	turquoise	0.911634169
ENSG00000223705.5	NSUN5P1	yellow	0.968478449
ENSG00000106133.13	NSUN5P2	turquoise	0.764904801
ENSG00000241058.1	NSUN6	yellow	0.899131657
ENSG00000125458.2	NT5C	brown	0.698985432
ENSG00000076685.14	NT5C2	blue	0.920237915
ENSG00000122643.14	NT5C3A	turquoise	0.938888926
ENSG00000141698.12	NT5C3B	turquoise	0.922353359
ENSG00000258408.1	NT5CP2	grey	0.369631255
ENSG00000178425.9	NT5DC1	turquoise	0.88408952
ENSG00000168268.6	NT5DC2	magenta	0.614731153
ENSG00000111696.7	NT5DC3	turquoise	0.881624194
ENSG00000135318.7	NT5E	green	0.778525925
ENSG00000205309.9	NT5M	turquoise	0.772003768
ENSG00000157045.4	NTAN1	turquoise	0.865154873
ENSG00000065057.3	NTHL1	turquoise	0.851917074
ENSG00000182667.10	NTM	turquoise	0.773251818
ENSG00000148335.10	NTMT1	turquoise	0.91881759
ENSG00000065320.4	NTN1	pink	0.705514983
ENSG00000074527.7	NTN4	blue	0.647075567
ENSG00000142233.7	NTN5	yellow	0.714598503
ENSG00000162631.14	NTNG1	turquoise	0.792121208
ENSG00000196358.6	NTNG2	turquoise	0.402911989
ENSG00000135778.7	NTPCR	turquoise	0.916414849
ENSG00000198400.7	NTRK1	midnightblue	0.765251508
ENSG00000148053.11	NTRK2	green	0.930758125
ENSG00000140538.12	NTRK3	turquoise	0.923474232
ENSG00000169006.6	NTSR2	green	0.652627384

ENSG00000074590.9	NUAK1	brown	0.83422349
ENSG00000163545.7	NUAK2	blue	0.616824146
ENSG00000013374.11	NUB1	turquoise	0.883984026
ENSG00000103274.6	NUBP1	turquoise	0.85158636
ENSG00000095906.12	NUBP2	red	0.93387414
ENSG00000151413.12	NUBPL	turquoise	0.900740996
ENSG00000104805.11	NUCB1	blue	0.850988032
ENSG00000070081.11	NUCB2	turquoise	0.946743404
ENSG00000069275.12	NUCKS1	turquoise	0.875725117
ENSG00000090273.9	NUDC	turquoise	0.851507952
ENSG00000120526.6	NUDCD1	turquoise	0.885782914
ENSG00000170584.6	NUDCD2	blue	0.877052278
ENSG00000015676.13	NUDCD3	turquoise	0.962727063
ENSG00000106268.11	NUDT1	turquoise	0.852976015
ENSG00000122824.6	NUDT10	turquoise	0.899600714
ENSG00000196368.4	NUDT11	turquoise	0.876360631
ENSG00000112874.5	NUDT12	blue	0.847607014
ENSG00000166321.9	NUDT13	turquoise	0.728627384
ENSG00000183828.10	NUDT14	turquoise	0.791109563
ENSG00000136159.3	NUDT15	turquoise	0.939180724
ENSG00000198585.7	NUDT16	brown	0.745819544
ENSG00000168101.10	NUDT16L1	turquoise	0.873859945
ENSG00000186364.7	NUDT17	turquoise	0.902349436
ENSG00000173566.9	NUDT18	turquoise	0.897035992
ENSG00000213965.3	NUDT19	turquoise	0.943948222
ENSG00000164978.13	NUDT2	turquoise	0.905188856
ENSG00000167005.9	NUDT21	turquoise	0.963169919
ENSG00000149761.4	NUDT22	red	0.868012537
ENSG00000272325.1	NUDT3	blue	0.821039511
ENSG00000173598.9	NUDT4	turquoise	0.829933038
ENSG00000165609.8	NUDT5	turquoise	0.943047044
ENSG00000170917.9	NUDT6	blue	0.825933923
ENSG00000140876.7	NUDT7	turquoise	0.875055716
ENSG00000167799.5	NUDT8	brown	0.501807192
ENSG00000170502.8	NUDT9	turquoise	0.922299924
ENSG00000083635.7	NUFIP1	turquoise	0.937550278
ENSG00000108256.4	NUFIP2	turquoise	0.866492589
ENSG00000137497.13	NUMA1	turquoise	0.920368805
ENSG00000133961.15	NUMB	turquoise	0.916940955
ENSG00000105245.5	NUMBL	turquoise	0.919136195
ENSG00000111581.5	NUP107	turquoise	0.837711078
ENSG00000069248.9	NUP133	turquoise	0.955260916
ENSG00000124789.7	NUP153	turquoise	0.923350414
ENSG00000113569.11	NUP155	turquoise	0.922636491
ENSG00000030066.9	NUP160	blue	0.942481986
ENSG00000095319.10	NUP188	turquoise	0.842165229
ENSG00000155561.10	NUP205	turquoise	0.912985947
ENSG00000132182.7	NUP210	turquoise	0.894623433

ENSG00000126883.12	NUP214	turquoise	0.922940205
ENSG00000163002.8	NUP35	turquoise	0.905257422
ENSG00000075188.4	NUP37	blue	0.868176052
ENSG00000120253.9	NUP43	blue	0.944409962
ENSG00000093000.14	NUP50	turquoise	0.975056605
ENSG00000138750.10	NUP54	turquoise	0.887098227
ENSG00000213024.6	NUP62	turquoise	0.939550667
ENSG00000125450.6	NUP85	turquoise	0.878171836
ENSG00000108559.7	NUP88	turquoise	0.959072822
ENSG00000102900.8	NUP93	turquoise	0.943474299
ENSG00000110713.11	NUP98	blue	0.939370896
ENSG00000139496.11	NUPL1	turquoise	0.934755377
ENSG00000136243.12	NUPL2	turquoise	0.947705112
ENSG00000176046.7	NUPR1	black	0.514112792
ENSG00000185290.3	NUPR1L	turquoise	0.708100903
ENSG00000153989.7	NUS1	turquoise	0.948121821
ENSG00000102898.7	NUTF2	turquoise	0.939078818
ENSG00000223482.3	NUTM2A-AS1	turquoise	0.749343102
ENSG00000143748.13	NVL	turquoise	0.944145829
ENSG00000188039.9	NWD1	green	0.69531487
ENSG00000162231.9	NXF1	turquoise	0.882060859
ENSG00000167693.12	NXN	black	0.794500087
ENSG00000144815.10	NXPE3	brown	0.869334298
ENSG00000122584.8	NXPH1	greenyellow	0.853012351
ENSG00000144227.4	NXPH2	greenyellow	0.701411541
ENSG00000182575.7	NXPH3	brown	0.534006214
ENSG00000182379.9	NXPH4	brown	0.67069488
ENSG00000132661.3	NXT1	cyan	0.865144902
ENSG00000101888.7	NXT2	blue	0.765961489
ENSG00000166924.4	NYAP1	turquoise	0.915103817
ENSG00000205978.5	NYNRIN	turquoise	0.673074729
ENSG00000184232.4	OAF	green	0.47758478
ENSG00000124596.12	OARD1	blue	0.841682128
ENSG00000089127.8	OAS1	grey	0.416349164
ENSG00000111335.8	OAS2	black	0.526435003
ENSG00000111331.8	OAS3	blue	0.604221689
ENSG00000065154.7	OAT	turquoise	0.936937691
ENSG00000104904.8	OAZ1	turquoise	0.935149326
ENSG00000180304.10	OAZ2	turquoise	0.949259369
ENSG00000143450.10	OAZ3	turquoise	0.878749092
ENSG00000107960.6	OBFC1	turquoise	0.940399178
ENSG00000154358.15	OBSCN	turquoise	0.799030999
ENSG00000124006.10	OBSL1	turquoise	0.876648102
ENSG00000099330.4	OCEL1	red	0.797312333
ENSG00000109180.10	OCIAD1	turquoise	0.973483551
ENSG00000145247.7	OCIAD2	turquoise	0.893092721
ENSG00000122543.6	OCM	turquoise	0.56358954
ENSG00000122126.11	OCRL	turquoise	0.972679915

ENSG00000115758.8	ODC1	cyan	0.829736205
ENSG00000155087.3	ODF1	turquoise	0.683193535
ENSG00000136811.12	ODF2	yellow	0.903686003
ENSG00000122417.11	ODF2L	pink	0.915729165
ENSG00000046651.10	OFD1	yellow	0.939814015
ENSG00000105953.10	OGDH	turquoise	0.902397386
ENSG00000197444.5	OGDHL	turquoise	0.925868947
ENSG00000087263.12	OGFOD1	turquoise	0.937872804
ENSG00000111325.12	OGFOD2	turquoise	0.918490225
ENSG00000181396.8	OGFOD3	blue	0.878277557
ENSG00000060491.12	OGFR	turquoise	0.884077073
ENSG00000229873.1	OGFR-AS1	turquoise	0.646272551
ENSG00000119900.7	OGFRL1	blue	0.765151957
ENSG00000114026.17	OGG1	turquoise	0.918159015
ENSG00000106809.6	OGN	brown	0.557704335
ENSG00000147162.9	OGT	turquoise	0.93327576
ENSG00000247556.2	OIP5-AS1	turquoise	0.90270428
ENSG00000138430.11	OLA1	turquoise	0.970165342
ENSG00000130558.14	OLFM1	turquoise	0.722219145
ENSG00000105088.4	OLFM2	green	0.56821731
ENSG00000118733.12	OLFM3	turquoise	0.890296185
ENSG00000183801.3	OLFML1	brown	0.489506244
ENSG00000185585.15	OLFML2A	grey	0.327688975
ENSG00000116774.7	OLFML3	magenta	0.628755853
ENSG00000184221.8	OLIG1	brown	0.649562939
ENSG00000205927.4	OLIG2	brown	0.785363921
ENSG00000173391.4	OLR1	magenta	0.750038231
ENSG00000162600.7	OMA1	blue	0.837818472
ENSG00000127083.7	OMD	grey	0.216847317
ENSG00000126861.4	OMG	turquoise	0.773395888
ENSG00000119547.5	ONECUT2	turquoise	0.749220754
ENSG00000198836.4	OPA1	turquoise	0.967348189
ENSG00000125741.4	OPA3	turquoise	0.950017366
ENSG00000197430.6	OPALIN	brown	0.773933395
ENSG00000183715.9	OPCML	turquoise	0.82811561
ENSG00000079482.11	OPHN1	green	0.85469076
ENSG00000178814.11	OPLAH	blue	0.728485187
ENSG00000128617.2	OPN1SW	turquoise	0.844842546
ENSG00000054277.8	OPN3	turquoise	0.796558082
ENSG00000122375.7	OPN4	turquoise	0.763280219
ENSG00000082556.6	OPRK1	turquoise	0.779029258
ENSG00000125510.11	OPRL1	turquoise	0.906076087
ENSG00000123240.12	OPTN	turquoise	0.86366204
ENSG00000236083.1	OR13E1P	turquoise	0.836301114
ENSG00000168124.2	OR1F1	turquoise	0.79759803
ENSG00000204657.2	OR2H2	turquoise	0.709346738
ENSG00000196071.3	OR2L13	turquoise	0.692986967
ENSG00000238243.2	OR2W3	turquoise	0.801865572

ENSG00000183444.10	OR7E38P	brown	0.677306848
ENSG00000238228.1	OR7E7P	turquoise	0.80464249
ENSG00000182500.7	ORAI1	black	0.788152183
ENSG00000160991.11	ORAI2	turquoise	0.93598209
ENSG00000175938.6	ORAI3	blue	0.750364259
ENSG00000149716.8	ORAOV1	turquoise	0.928485579
ENSG00000251008.1	ORAOV1P1	turquoise	0.683654742
ENSG00000115942.4	ORC2	turquoise	0.922912473
ENSG00000135336.10	ORC3	blue	0.88712697
ENSG00000115947.9	ORC4	turquoise	0.962505249
ENSG00000164815.6	ORC5	turquoise	0.963115458
ENSG00000091651.4	ORC6	turquoise	0.928423746
ENSG00000128699.9	ORMDL1	turquoise	0.939847875
ENSG00000123353.5	ORMDL2	blue	0.767443565
ENSG00000172057.5	ORMDL3	blue	0.871562701
ENSG00000135506.11	OS9	turquoise	0.913854474
ENSG00000110048.7	OSBP	turquoise	0.947935071
ENSG00000184792.11	OSBP2	turquoise	0.932844573
ENSG00000144645.9	OSBPL10	turquoise	0.887966513
ENSG00000144909.7	OSBPL11	green	0.760466387
ENSG00000141447.12	OSBPL1A	brown	0.900275553
ENSG00000130703.11	OSBPL2	turquoise	0.947089352
ENSG00000070882.8	OSBPL3	blue	0.707177174
ENSG00000021762.15	OSBPL5	yellow	0.906051797
ENSG00000079156.12	OSBPL6	turquoise	0.800909227
ENSG00000006025.7	OSBPL7	yellow	0.95701884
ENSG00000091039.12	OSBPL8	turquoise	0.934134799
ENSG00000117859.14	OSBPL9	turquoise	0.928512018
ENSG00000170909.9	OSCAR	magenta	0.885028872
ENSG00000116885.14	OSCP1	turquoise	0.929989468
ENSG00000132823.6	OSER1	turquoise	0.887638899
ENSG00000223891.1	OSER1-AS1	red	0.871060402
ENSG00000092094.6	OSGEP	turquoise	0.864652607
ENSG00000128694.7	OSGEPL1	turquoise	0.868858167
ENSG00000253559.1	OSGEPL1-AS1	turquoise	0.734413126
ENSG00000140961.8	OSGIN1	black	0.660404843
ENSG00000164823.5	OSGIN2	blue	0.849221053
ENSG00000145623.8	OSMR	black	0.899874038
ENSG00000228474.1	OST4	red	0.922368298
ENSG00000198856.8	OSTC	turquoise	0.907240825
ENSG00000134996.11	OSTF1	turquoise	0.900524201
ENSG00000081087.10	OSTM1	turquoise	0.946754704
ENSG00000225174.1	OSTM1-AS1	green	0.716712652
ENSG00000115155.12	OTOF	turquoise	0.803347607
ENSG00000178602.3	OTOS	grey	0.167378374
ENSG00000167770.7	OTUB1	turquoise	0.963754594
ENSG00000089723.5	OTUB2	turquoise	0.857500335
ENSG00000165312.5	OTUD1	turquoise	0.893150776

ENSG00000169914.5	OTUD3	turquoise	0.920771872
ENSG00000164164.11	OTUD4	turquoise	0.93702867
ENSG00000068308.9	OTUD5	turquoise	0.965906797
ENSG00000155100.6	OTUD6B	turquoise	0.889419127
ENSG00000169918.5	OTUD7A	turquoise	0.851435643
ENSG00000163113.10	OTUD7B	brown	0.825084649
ENSG00000115507.5	OTX1	grey	0.196848891
ENSG00000085465.11	OVGP1	turquoise	0.804652971
ENSG00000155463.8	OXA1L	turquoise	0.925570842
ENSG00000083720.8	OXCT1	turquoise	0.946684491
ENSG00000204237.4	OXLD1	turquoise	0.873257328
ENSG00000154814.9	OXNAD1	turquoise	0.926753676
ENSG00000164830.13	OXR1	blue	0.843297863
ENSG00000151093.3	OXSM	turquoise	0.936376743
ENSG00000172939.4	OXSR1	turquoise	0.901080035
ENSG00000180914.6	OXTR	grey	0.382474993
ENSG00000135124.10	P2RX4	blue	0.826563136
ENSG00000083454.17	P2RX5	turquoise	0.903966982
ENSG00000099957.12	P2RX6	greenyellow	0.820214028
ENSG00000206145.4	P2RX6P	grey	0.3663709
ENSG00000089041.12	P2RX7	brown	0.846588846
ENSG00000169860.4	P2RY1	turquoise	0.682884521
ENSG00000244165.1	P2RY11	turquoise	0.875538812
ENSG00000169313.9	P2RY12	magenta	0.610321805
ENSG00000181631.6	P2RY13	magenta	0.63726224
ENSG00000174944.4	P2RY14	grey	0.157799561
ENSG00000175591.7	P2RY2	grey	0.299384552
ENSG00000122884.8	P4HA1	cyan	0.782093551
ENSG00000072682.14	P4HA2	black	0.746294387
ENSG00000185624.10	P4HB	turquoise	0.855638605
ENSG00000178467.13	P4HTM	turquoise	0.951279995
ENSG00000170515.9	PA2G4	turquoise	0.963401893
ENSG00000175575.8	PAAF1	turquoise	0.752048059
ENSG00000070756.9	PABPC1	turquoise	0.905091333
ENSG00000101104.8	PABPC1L	turquoise	0.78151816
ENSG00000186288.4	PABPC1L2A	turquoise	0.899393378
ENSG00000184388.4	PABPC1L2B	turquoise	0.893915892
ENSG00000255642.1	PABPC1P4	blue	0.78125305
ENSG00000090621.9	PABPC4	turquoise	0.943495068
ENSG00000174740.7	PABPC5	blue	0.795161712
ENSG00000100836.6	PABPN1	turquoise	0.884162557
ENSG00000112530.7	PACRG	turquoise	0.857477308
ENSG00000163138.14	PACRGL	turquoise	0.930001387
ENSG00000175115.7	PACS1	turquoise	0.944281181
ENSG00000179364.9	PACS2	brown	0.844401189
ENSG00000124507.6	PACSIN1	turquoise	0.886929767
ENSG00000100266.13	PACSIN2	blue	0.887875414
ENSG00000165912.11	PACSIN3	brown	0.657347667

ENSG00000117115.8	PADI2	brown	0.852109535
ENSG00000006712.10	PAF1	turquoise	0.866449399
ENSG00000007168.8	PAFAH1B1	turquoise	0.974596721
ENSG00000168092.9	PAFAH1B2	turquoise	0.955112586
ENSG00000079462.3	PAFAH1B3	turquoise	0.892466849
ENSG00000158006.9	PAFAH2	blue	0.930057678
ENSG00000076641.4	PAG1	turquoise	0.714967768
ENSG00000128050.4	PAICS	turquoise	0.768898347
ENSG00000172239.9	PAIP1	turquoise	0.947010376
ENSG00000120727.8	PAIP2	turquoise	0.909710661
ENSG00000124374.8	PAIP2B	brown	0.445745516
ENSG00000149269.5	PAK1	turquoise	0.953744847
ENSG00000111845.4	PAK1IP1	turquoise	0.946813417
ENSG00000180370.6	PAK2	turquoise	0.810089533
ENSG00000077264.10	PAK3	turquoise	0.913549539
ENSG00000130669.13	PAK4	turquoise	0.862800289
ENSG00000137843.7	PAK6	turquoise	0.885891142
ENSG00000101349.12	PAK7	turquoise	0.919607287
ENSG00000083093.5	PALB2	turquoise	0.948288716
ENSG00000107719.8	PALD1	yellow	0.811990059
ENSG00000129116.13	PALLD	green	0.799902371
ENSG00000099864.13	PALM	turquoise	0.816158169
ENSG00000243444.3	PALM2	turquoise	0.868709005
ENSG00000099260.6	PALMD	black	0.524393414
ENSG00000145730.16	PAM	turquoise	0.949812194
ENSG00000217930.3	PAM16	turquoise	0.877319507
ENSG00000149090.7	PAMR1	green	0.734315108
ENSG00000135473.10	PAN2	yellow	0.949729516
ENSG00000152520.9	PAN3	turquoise	0.862524577
ENSG00000261485.1	PAN3-AS1	turquoise	0.871549368
ENSG00000152782.12	PANK1	turquoise	0.786705849
ENSG00000125779.17	PANK2	turquoise	0.968099224
ENSG00000120137.6	PANK3	turquoise	0.941524547
ENSG00000157881.9	PANK4	turquoise	0.962617541
ENSG00000110218.4	PANX1	turquoise	0.89745329
ENSG00000073150.9	PANX2	turquoise	0.835882379
ENSG00000148832.10	PAOX	blue	0.827019683
ENSG00000164329.9	PAPD4	turquoise	0.91754162
ENSG00000121274.8	PAPD5	cyan	0.907730135
ENSG00000112941.8	PAPD7	turquoise	0.934782494
ENSG00000183760.6	PAPL	brown	0.766192211
ENSG00000100767.11	PAPLN	green	0.779981051
ENSG00000090060.13	PAPOLA	cyan	0.917039231
ENSG00000115421.8	PAPOLG	turquoise	0.932047236
ENSG00000138801.4	PAPSS1	turquoise	0.967534645
ENSG00000198682.8	PAPSS2	black	0.793738573
ENSG00000163291.10	PAQR3	turquoise	0.93185265
ENSG00000162073.9	PAQR4	brown	0.931970356

ENSG00000137819.9	PAQR5	black	0.64671574
ENSG00000160781.11	PAQR6	brown	0.489669708
ENSG00000182749.5	PAQR7	pink	0.851879381
ENSG00000170915.8	PAQR8	brown	0.660311463
ENSG00000188582.4	PAQR9	turquoise	0.835818562
ENSG00000148498.11	PARD3	turquoise	0.798966192
ENSG00000226386.1	PARD3-AS1	turquoise	0.558588703
ENSG00000102981.5	PARD6A	turquoise	0.809597249
ENSG00000124171.4	PARD6B	pink	0.856979991
ENSG00000178184.11	PARD6G	blue	0.713769073
ENSG00000227345.4	PARG	turquoise	0.969177151
ENSG00000185345.14	PARK2	turquoise	0.930550526
ENSG00000116288.8	PARK7	turquoise	0.913416166
ENSG00000175193.8	PARL	turquoise	0.898511146
ENSG00000169116.7	PARM1	turquoise	0.939688856
ENSG00000140694.12	PARN	turquoise	0.947056543
ENSG00000143799.8	PARP1	turquoise	0.838409842
ENSG00000178685.9	PARP10	blue	0.71991592
ENSG00000111224.9	PARP11	turquoise	0.935875571
ENSG00000059378.8	PARP12	black	0.747793797
ENSG00000173193.9	PARP14	black	0.799623797
ENSG00000138617.10	PARP16	yellow	0.908268434
ENSG00000129484.9	PARP2	turquoise	0.967546379
ENSG00000041880.10	PARP3	turquoise	0.922488718
ENSG00000102699.5	PARP4	blue	0.842436916
ENSG00000137817.12	PARP6	turquoise	0.970266472
ENSG00000151883.12	PARP8	blue	0.83813055
ENSG00000138496.12	PARP9	black	0.864074957
ENSG00000162396.5	PARS2	turquoise	0.926862022
ENSG00000197702.7	PARVA	green	0.916216133
ENSG00000188677.10	PARVB	turquoise	0.90012438
ENSG00000138964.12	PARVG	magenta	0.948572281
ENSG00000115687.9	PASK	turquoise	0.808441398
ENSG00000166889.13	PATL1	blue	0.940081862
ENSG00000100105.13	PATZ1	turquoise	0.894891534
ENSG00000177425.6	PAWR	blue	0.736237133
ENSG00000007372.16	PAX6	green	0.887467531
ENSG00000159086.10	PAXBP1	blue	0.896500509
ENSG00000238197.1	PAXBP1-AS1	turquoise	0.87312117
ENSG00000157212.14	PAXIP1	turquoise	0.926314347
ENSG00000273344.1	PAXIP1-AS1	turquoise	0.937087666
ENSG00000214106.3	PAXIP1-AS2	green	0.825585748
ENSG00000102390.6	PBDC1	turquoise	0.935588258
ENSG00000108187.11	PBLD	brown	0.701413348
ENSG00000163939.14	PBRM1	turquoise	0.890396338
ENSG00000185630.14	PBX1	turquoise	0.918207972
ENSG00000204304.7	PBX2	turquoise	0.966112833
ENSG00000167081.12	PBX3	turquoise	0.953693587

ENSG00000105717.9	PBX4	yellow	0.828163956
ENSG00000163346.12	PBXIP1	green	0.846624611
ENSG00000173599.9	PC	turquoise	0.87134761
ENSG00000228288.2	PCAT6	turquoise	0.575626265
ENSG00000166228.4	PCBD1	cyan	0.857017268
ENSG00000132570.10	PCBD2	turquoise	0.860064099
ENSG00000169564.5	PCBP1	turquoise	0.867023003
ENSG00000179818.9	PCBP1-AS1	turquoise	0.809259371
ENSG00000197111.11	PCBP2	turquoise	0.936121952
ENSG00000183570.12	PCBP3	turquoise	0.855908282
ENSG00000090097.16	PCBP4	brown	0.900657234
ENSG00000175198.10	PCCA	turquoise	0.921133046
ENSG00000114054.9	PCCB	blue	0.885963728
ENSG00000156453.9	PCDH1	turquoise	0.884906337
ENSG00000138650.7	PCDH10	turquoise	0.931216281
ENSG00000102290.17	PCDH11X	turquoise	0.808428789
ENSG00000113555.4	PCDH12	turquoise	0.726390728
ENSG00000150275.13	PCDH15	greenyellow	0.640790385
ENSG00000118946.7	PCDH17	turquoise	0.873744587
ENSG00000189184.7	PCDH18	brown	0.524386164
ENSG00000165194.10	PCDH19	greenyellow	0.749103689
ENSG00000197991.10	PCDH20	turquoise	0.820414606
ENSG00000169851.11	PCDH7	turquoise	0.856020465
ENSG00000136099.9	PCDH8	turquoise	0.896003314
ENSG00000184226.10	PCDH9	blue	0.791725444
ENSG00000204970.5	PCDHA1	turquoise	0.845727471
ENSG00000243232.3	PCDHAC2	turquoise	0.859492031
ENSG00000120324.4	PCDHB10	turquoise	0.91261141
ENSG00000187372.9	PCDHB13	turquoise	0.833837981
ENSG00000120327.4	PCDHB14	turquoise	0.941965685
ENSG00000113248.3	PCDHB15	blue	0.854943847
ENSG00000196963.2	PCDHB16	blue	0.84509564
ENSG00000112852.4	PCDHB2	turquoise	0.834929497
ENSG00000113205.2	PCDHB3	turquoise	0.844039226
ENSG00000081818.1	PCDHB4	turquoise	0.816855789
ENSG00000113209.6	PCDHB5	blue	0.799137736
ENSG00000113211.3	PCDHB6	turquoise	0.807297231
ENSG00000113212.4	PCDHB7	green	0.687872297
ENSG00000204956.4	PCDHGA1	turquoise	0.822873239
ENSG00000253846.1	PCDHGA10	green	0.830877501
ENSG00000253873.1	PCDHGA11	green	0.786498102
ENSG00000253159.1	PCDHGA12	green	0.791843792
ENSG00000081853.13	PCDHGA2	turquoise	0.737859672
ENSG00000254245.1	PCDHGA3	green	0.793695186
ENSG00000262576.1	PCDHGA4	green	0.838555153
ENSG00000253485.1	PCDHGA5	turquoise	0.750703957
ENSG00000253731.1	PCDHGA6	turquoise	0.820653083
ENSG00000253537.1	PCDHGA7	turquoise	0.758845504

ENSG00000261934.1	PCDHGA9	green	0.84958537
ENSG00000254221.1	PCDHGB1	green	0.840001584
ENSG00000253910.1	PCDHGB2	green	0.846225606
ENSG00000262209.1	PCDHGB3	green	0.772604522
ENSG00000253953.1	PCDHGB4	turquoise	0.753616847
ENSG00000253305.1	PCDHGB6	green	0.857506675
ENSG00000254122.1	PCDHGB7	green	0.880005008
ENSG00000240184.2	PCDHGC3	green	0.887395184
ENSG00000242419.1	PCDHGC4	turquoise	0.846389792
ENSG00000240764.2	PCDHGC5	turquoise	0.89650916
ENSG00000163075.8	PCDP1	pink	0.94991217
ENSG00000132635.12	PCED1A	turquoise	0.877681584
ENSG00000179715.8	PCED1B	pink	0.75426783
ENSG00000247774.2	PCED1B-AS1	magenta	0.918663951
ENSG00000165494.6	PCF11	turquoise	0.881012456
ENSG00000115289.8	PCGF1	turquoise	0.93905858
ENSG00000056661.9	PCGF2	turquoise	0.878313089
ENSG00000185619.13	PCGF3	turquoise	0.939571439
ENSG00000180628.10	PCGF5	blue	0.895621192
ENSG00000156374.10	PCGF6	turquoise	0.901173959
ENSG00000126226.17	PCID2	turquoise	0.933127634
ENSG00000100982.7	PCIF1	turquoise	0.940093116
ENSG00000124253.9	PCK1	grey	0.282374203
ENSG00000100889.7	PCK2	blue	0.693930537
ENSG00000186472.15	PCLO	turquoise	0.909850421
ENSG00000078674.13	PCM1	turquoise	0.872531659
ENSG00000120265.12	PCMT1	turquoise	0.963666127
ENSG00000168300.9	PCMTD1	turquoise	0.897180478
ENSG00000203880.7	PCMTD2	turquoise	0.943892495
ENSG00000132646.6	PCNA	blue	0.897429913
ENSG00000081154.7	PCNP	turquoise	0.897827259
ENSG00000160299.12	PCNT	turquoise	0.945888517
ENSG00000100731.11	PCNX	turquoise	0.970455123
ENSG00000135749.14	PCNXL2	turquoise	0.93956786
ENSG00000197136.4	PCNXL3	turquoise	0.889305258
ENSG00000126773.8	PCNXL4	blue	0.920812795
ENSG00000106333.8	PCOLCE	brown	0.482165594
ENSG00000183036.6	PCP4	turquoise	0.8308095
ENSG00000248485.1	PCP4L1	turquoise	0.891257659
ENSG00000175426.6	PCSK1	turquoise	0.746508236
ENSG00000102109.7	PCSK1N	turquoise	0.670883669
ENSG00000125851.5	PCSK2	turquoise	0.908364537
ENSG00000115257.11	PCSK4	pink	0.860670454
ENSG00000099139.9	PCSK5	green	0.847963051
ENSG00000140479.12	PCSK6	brown	0.901405334
ENSG00000160613.8	PCSK7	yellow	0.921652938
ENSG00000141179.9	PCTP	green	0.710373661
ENSG00000116005.7	PCYOX1	turquoise	0.924538232

ENSG00000145882.6	PCYOX1L	turquoise	0.923228243
ENSG00000161217.7	PCYT1A	turquoise	0.878297616
ENSG00000102230.9	PCYT1B	turquoise	0.934977914
ENSG00000185813.6	PCYT2	turquoise	0.888574858
ENSG00000106244.8	PDAP1	turquoise	0.918212699
ENSG00000114209.10	PDCD10	turquoise	0.929750891
ENSG00000148843.9	PDCD11	turquoise	0.946713169
ENSG00000071994.6	PDCD2	turquoise	0.960989283
ENSG00000126249.3	PDCD2L	turquoise	0.961852186
ENSG00000150593.11	PDCD4	turquoise	0.869405334
ENSG00000203497.2	PDCD4-AS1	turquoise	0.7527132
ENSG00000105185.7	PDCD5	turquoise	0.933158809
ENSG00000249915.3	PDCD6	turquoise	0.947999948
ENSG00000170248.9	PDCD6IP	turquoise	0.922301507
ENSG00000090470.10	PDCD7	turquoise	0.958002583
ENSG00000136940.9	PDCL	blue	0.899416579
ENSG00000115539.9	PDCL3	turquoise	0.841233702
ENSG00000244119.1	PDCL3P4	yellow	0.771396026
ENSG00000177225.12	PDDC1	turquoise	0.853712257
ENSG00000112541.9	PDE10A	turquoise	0.791492917
ENSG00000174840.8	PDE12	turquoise	0.939438723
ENSG00000115252.14	PDE1A	greenyellow	0.681930197
ENSG00000123360.7	PDE1B	turquoise	0.781986809
ENSG00000154678.12	PDE1C	brown	0.899788503
ENSG00000186642.11	PDE2A	turquoise	0.877234472
ENSG00000172572.6	PDE3A	green	0.739155749
ENSG00000065989.11	PDE4A	turquoise	0.898278909
ENSG00000184588.13	PDE4B	turquoise	0.864610065
ENSG00000105650.17	PDE4C	grey	0.53543837
ENSG00000113448.12	PDE4D	turquoise	0.823726606
ENSG00000178104.15	PDE4DIP	turquoise	0.942688379
ENSG00000138735.11	PDE5A	brown	0.532624348
ENSG00000133256.8	PDE6B	yellow	0.819157472
ENSG00000156973.9	PDE6D	turquoise	0.897122883
ENSG00000205268.6	PDE7A	turquoise	0.848166206
ENSG00000171408.9	PDE7B	turquoise	0.918415455
ENSG00000073417.10	PDE8A	brown	0.933994778
ENSG00000113231.9	PDE8B	turquoise	0.884358817
ENSG00000160191.13	PDE9A	midnightblue	0.863087246
ENSG00000258429.1	PDF	turquoise	0.771287298
ENSG00000197461.9	PDGFA	blue	0.784954545
ENSG00000100311.12	PDGFB	turquoise	0.793113075
ENSG00000145431.6	PDGFC	green	0.839003391
ENSG00000170962.8	PDGFD	greenyellow	0.694621926
ENSG00000134853.7	PDGFRA	greenyellow	0.643024081
ENSG00000113721.9	PDGFRB	grey	0.578364843
ENSG00000104213.8	PDGFRL	turquoise	0.85605622
ENSG00000131828.9	PDHA1	turquoise	0.963234846

ENSG00000168291.8	PDHB	turquoise	0.939929625
ENSG00000110435.7	PDHX	turquoise	0.96423157
ENSG00000185615.11	PDIA2	yellow	0.863676452
ENSG00000167004.8	PDIA3	turquoise	0.870558982
ENSG00000180867.9	PDIA3P	blue	0.842959792
ENSG00000155660.6	PDIA4	cyan	0.83890487
ENSG00000065485.13	PDIA5	pink	0.757325544
ENSG00000143870.8	PDIA6	turquoise	0.851341518
ENSG00000175087.5	PDIK1L	turquoise	0.945389792
ENSG00000152256.9	PDK1	turquoise	0.834990127
ENSG00000005882.7	PDK2	turquoise	0.921022162
ENSG00000067992.8	PDK3	turquoise	0.835933695
ENSG00000004799.7	PDK4	green	0.649900939
ENSG00000107438.4	PDLIM1	black	0.832189415
ENSG00000120913.19	PDLIM2	black	0.563381059
ENSG00000154553.9	PDLIM3	green	0.727535015
ENSG00000131435.8	PDLIM4	black	0.784906143
ENSG00000163110.10	PDLIM5	green	0.912124849
ENSG00000196923.9	PDLIM7	turquoise	0.811530419
ENSG00000164951.11	PDP1	turquoise	0.880442352
ENSG00000172840.2	PDP2	blue	0.841050365
ENSG00000140992.14	PDPK1	turquoise	0.95360921
ENSG00000162493.12	PDPN	blue	0.589334298
ENSG00000090857.9	PDPR	turquoise	0.826783498
ENSG00000088356.4	PDRG1	turquoise	0.906670811
ENSG00000121892.10	PDS5A	turquoise	0.937303908
ENSG00000083642.14	PDS5B	turquoise	0.944686033
ENSG00000148459.11	PDSS1	turquoise	0.900412702
ENSG00000164494.7	PDSS2	blue	0.90550193
ENSG00000179889.14	PDXDC1	turquoise	0.972353995
ENSG00000196696.8	PDXDC2P	yellow	0.825478966
ENSG00000160209.14	PDXK	turquoise	0.918744435
ENSG00000241360.1	PDXP	turquoise	0.837308654
ENSG00000101327.4	PDYN	turquoise	0.783634923
ENSG00000120509.6	PDZD11	turquoise	0.888538349
ENSG00000133401.11	PDZD2	turquoise	0.916403212
ENSG00000067840.8	PDZD4	turquoise	0.873900727
ENSG00000186862.13	PDZD7	turquoise	0.862066652
ENSG00000165650.7	PDZD8	turquoise	0.863688285
ENSG00000155714.9	PDZD9	turquoise	0.833192941
ENSG00000121440.10	PDZRN3	green	0.748070463
ENSG00000165966.10	PDZRN4	greenyellow	0.661656756
ENSG00000162734.8	PEA15	turquoise	0.74148175
ENSG00000173517.6	PEAK1	turquoise	0.936308794
ENSG00000187800.9	PEAR1	black	0.668707196
ENSG00000089220.4	PEBP1	turquoise	0.932187337
ENSG00000134020.6	PEBP4	brown	0.557651287
ENSG00000115425.9	PECR	blue	0.880194967

ENSG00000162517.8	PEF1	turquoise	0.949106661
ENSG00000242265.1	PEG10	turquoise	0.872544735
ENSG00000198300.8	PEG3	turquoise	0.937696812
ENSG00000197329.7	PELI1	turquoise	0.91658514
ENSG00000139946.5	PELI2	blue	0.782121103
ENSG00000174516.10	PELI3	turquoise	0.933405836
ENSG00000152684.10	PELO	turquoise	0.954167067
ENSG00000141456.10	PELP1	turquoise	0.897562349
ENSG00000133027.13	PEMT	red	0.885937954
ENSG00000181195.6	PENK	turquoise	0.820152863
ENSG00000124299.9	PEPD	turquoise	0.843212579
ENSG00000179094.9	PER1	yellow	0.773157989
ENSG00000132326.7	PER2	turquoise	0.805115086
ENSG00000049246.10	PER3	turquoise	0.853715031
ENSG00000112378.11	PERP	black	0.717372483
ENSG00000100029.13	PES1	turquoise	0.901702458
ENSG00000229833.5	PET100	red	0.914551689
ENSG00000059691.7	PET112	turquoise	0.939371659
ENSG00000232838.3	PET117	turquoise	0.865438167
ENSG00000127980.11	PEX1	turquoise	0.939542266
ENSG00000157911.5	PEX10	blue	0.840093084
ENSG00000166821.4	PEX11A	green	0.889361885
ENSG00000131779.6	PEX11B	turquoise	0.977258978
ENSG00000104883.3	PEX11G	brown	0.6082172
ENSG00000108733.5	PEX12	blue	0.893639582
ENSG00000162928.8	PEX13	turquoise	0.921346642
ENSG00000142655.8	PEX14	turquoise	0.953147108
ENSG00000121680.11	PEX16	turquoise	0.899792748
ENSG00000162735.14	PEX19	turquoise	0.945204299
ENSG00000164751.10	PEX2	blue	0.911416333
ENSG00000215193.8	PEX26	turquoise	0.971472262
ENSG00000034693.10	PEX3	turquoise	0.901513706
ENSG00000139197.6	PEX5	brown	0.752479249
ENSG00000114757.14	PEX5L	brown	0.897306977
ENSG00000124587.9	PEX6	yellow	0.8620893
ENSG00000112357.8	PEX7	turquoise	0.898863107
ENSG00000178921.9	PFAS	turquoise	0.953781808
ENSG00000113068.5	PFDN1	turquoise	0.953023076
ENSG00000143256.4	PFDN2	red	0.929579755
ENSG00000101132.5	PFDN4	turquoise	0.908869319
ENSG00000123349.9	PFDN5	red	0.949720534
ENSG00000204220.5	PFDN6	red	0.918488765
ENSG00000123836.10	PFKFB2	green	0.675630554
ENSG00000170525.14	PFKFB3	blue	0.732692157
ENSG00000114268.7	PFKFB4	blue	0.863889315
ENSG00000141959.12	PFKL	blue	0.836559973
ENSG00000152556.11	PFKM	blue	0.843793797
ENSG00000067057.12	PFKP	midnightblue	0.766744829

ENSG00000108518.7	PFN1	turquoise	0.752040569
ENSG00000070087.9	PFN2	turquoise	0.959061762
ENSG00000176732.6	PFN4	blue	0.645484685
ENSG00000171314.8	PGAM1	turquoise	0.880799791
ENSG00000247077.2	PGAM5	turquoise	0.937776094
ENSG00000197121.10	PGAP1	turquoise	0.8692537
ENSG00000148985.15	PGAP2	turquoise	0.876922138
ENSG00000161395.8	PGAP3	turquoise	0.894834475
ENSG00000137338.4	PGBD1	turquoise	0.868558338
ENSG00000185220.7	PGBD2	blue	0.786977411
ENSG00000243251.4	PGBD3	turquoise	0.816009569
ENSG00000177614.5	PGBD5	turquoise	0.941045221
ENSG00000096088.12	PGC	grey	0.134360649
ENSG00000142657.16	PGD	turquoise	0.820918865
ENSG00000119630.9	PGF	grey	0.30620839
ENSG00000164219.5	PGGT1B	blue	0.859654209
ENSG00000102144.9	PGK1	turquoise	0.946575381
ENSG00000130313.2	PGLS	red	0.902654554
ENSG00000079739.11	PGM1	blue	0.806502128
ENSG00000169299.9	PGM2	green	0.743933867
ENSG00000165434.6	PGM2L1	turquoise	0.850664508
ENSG00000013375.11	PGM3	turquoise	0.841988206
ENSG00000154330.8	PGM5	grey	0.411329137
ENSG00000184207.8	PGP	turquoise	0.935533306
ENSG00000130517.9	PGPEP1	blue	0.777303574
ENSG00000101856.8	PGRMC1	turquoise	0.952631868
ENSG00000164040.12	PGRMC2	turquoise	0.955733851
ENSG00000087157.14	PGS1	turquoise	0.932189916
ENSG00000112137.12	PHACTR1	turquoise	0.893444802
ENSG00000112419.10	PHACTR2	green	0.710164258
ENSG00000087495.12	PHACTR3	brown	0.824948201
ENSG00000204138.8	PHACTR4	blue	0.883100778
ENSG00000164902.9	PHAX	turquoise	0.929969892
ENSG00000167085.7	PHB	turquoise	0.909148541
ENSG00000215021.4	PHB2	turquoise	0.946121184
ENSG00000230224.1	PHBP9	turquoise	0.749604073
ENSG00000111752.6	PHC1	turquoise	0.917710965
ENSG00000179899.7	PHC1P1	turquoise	0.788803019
ENSG00000134686.12	PHC2	turquoise	0.843837193
ENSG00000173889.11	PHC3	turquoise	0.867810763
ENSG00000224204.1	PHEX-AS1	turquoise	0.680092474
ENSG00000112511.13	PHF1	turquoise	0.931477836
ENSG00000130024.10	PHF10	blue	0.809936225
ENSG00000136147.12	PHF11	blue	0.878295413
ENSG00000109118.9	PHF12	turquoise	0.928342509
ENSG00000116273.5	PHF13	turquoise	0.931779292
ENSG00000106443.10	PHF14	turquoise	0.90692381
ENSG00000043143.16	PHF15	turquoise	0.948697637

ENSG00000102221.9	PHF16	brown	0.807833513
ENSG00000077684.11	PHF17	turquoise	0.926165567
ENSG00000119403.9	PHF19	blue	0.843065544
ENSG00000197724.6	PHF2	blue	0.927788031
ENSG00000025293.11	PHF20	turquoise	0.959489742
ENSG00000129292.16	PHF20L1	turquoise	0.957072517
ENSG00000135365.11	PHF21A	turquoise	0.853616934
ENSG00000056487.11	PHF21B	green	0.755416892
ENSG00000040633.8	PHF23	turquoise	0.936455775
ENSG00000118482.7	PHF3	turquoise	0.924891121
ENSG00000100410.3	PHF5A	turquoise	0.874256119
ENSG00000156531.12	PHF6	turquoise	0.915728961
ENSG00000010318.15	PHF7	turquoise	0.874302162
ENSG00000172943.14	PHF8	blue	0.90475392
ENSG00000092621.7	PHGDH	blue	0.696477179
ENSG00000146247.13	PHIP	turquoise	0.868300496
ENSG00000067177.10	PHKA1	green	0.862764168
ENSG00000044446.7	PHKA2	turquoise	0.921047379
ENSG00000237836.1	PHKA2-AS1	turquoise	0.657278019
ENSG00000102893.11	PHKB	turquoise	0.952440773
ENSG00000156873.11	PHKG2	turquoise	0.948436503
ENSG00000139289.9	PHLDA1	turquoise	0.842106336
ENSG00000174307.5	PHLDA3	red	0.856901272
ENSG00000019144.12	PHLDB1	brown	0.944476335
ENSG00000144824.15	PHLDB2	brown	0.513830179
ENSG00000176531.6	PHLDB3	turquoise	0.849834291
ENSG00000081913.9	PHLPP1	brown	0.7885147
ENSG00000040199.14	PHLPP2	turquoise	0.962563961
ENSG00000173868.7	PHOSPHO1	turquoise	0.544523633
ENSG00000144362.7	PHOSPHO2	turquoise	0.784476139
ENSG00000054148.13	PHPT1	red	0.963441988
ENSG00000070047.7	PHRF1	turquoise	0.907268003
ENSG00000116793.11	PHTF1	turquoise	0.940603032
ENSG00000006576.12	PHTF2	turquoise	0.927307125
ENSG00000107537.9	PHYH	blue	0.833663648
ENSG00000175287.14	PHYHD1	green	0.666204235
ENSG00000168490.9	PHYHIP	turquoise	0.87258288
ENSG00000165443.7	PHYHIPL	blue	0.863245723
ENSG00000175309.10	PHYKPL	yellow	0.906894612
ENSG00000164530.9	PI16	blue	0.642637607
ENSG00000155252.12	PI4K2A	turquoise	0.964154944
ENSG00000038210.9	PI4K2B	blue	0.867276924
ENSG00000241973.6	PI4KA	turquoise	0.962382907
ENSG00000215513.7	PI4KAP1	turquoise	0.558963117
ENSG00000183506.12	PI4KAP2	turquoise	0.87534057
ENSG00000143393.12	PI4KB	turquoise	0.938990487
ENSG00000139200.9	PIANP	turquoise	0.889272906
ENSG00000078043.11	PIAS2	turquoise	0.975573357

ENSG00000131788.11	PIAS3	blue	0.933392086
ENSG00000105229.2	PIAS4	blue	0.810233608
ENSG00000083535.11	PIBF1	turquoise	0.891965857
ENSG00000073921.13	PICALM	turquoise	0.795118287
ENSG00000100151.11	PICK1	turquoise	0.888124059
ENSG00000153823.14	PID1	turquoise	0.868480662
ENSG00000177595.13	PIDD	yellow	0.929639989
ENSG00000103335.15	PIEZO1	blue	0.742922828
ENSG00000154864.7	PIEZO2	brown	0.766953746
ENSG00000173947.9	PIFO	pink	0.898743799
ENSG00000165195.9	PIGA	blue	0.811121752
ENSG00000069943.5	PIGB	turquoise	0.922021601
ENSG00000135845.5	PIGC	turquoise	0.965690651
ENSG00000151665.8	PIGF	turquoise	0.841392673
ENSG00000174227.11	PIGG	turquoise	0.921183618
ENSG00000100564.4	PIGH	turquoise	0.929930587
ENSG00000142892.10	PIGK	turquoise	0.953874831
ENSG00000108474.12	PIGL	turquoise	0.926118513
ENSG00000143315.5	PIGM	blue	0.897362821
ENSG00000197563.5	PIGN	turquoise	0.901056959
ENSG00000165282.9	PIGO	turquoise	0.869832639
ENSG00000185808.9	PIGP	turquoise	0.90324457
ENSG00000007541.10	PIGQ	turquoise	0.862829817
ENSG00000087111.16	PIGS	turquoise	0.948930132
ENSG00000124155.12	PIGT	brown	0.839183117
ENSG00000101464.6	PIGU	brown	0.834929901
ENSG00000060642.6	PIGV	blue	0.90238242
ENSG00000184886.3	PIGW	turquoise	0.829420264
ENSG00000163964.9	PIGX	turquoise	0.958711909
ENSG00000119227.3	PIGZ	turquoise	0.859863978
ENSG00000104872.6	PIH1D1	turquoise	0.932013472
ENSG00000150773.6	PIH1D2	pink	0.893753543
ENSG00000155629.10	PIK3AP1	magenta	0.915661831
ENSG00000011405.9	PIK3C2A	blue	0.658773378
ENSG00000133056.9	PIK3C2B	brown	0.911753399
ENSG00000078142.7	PIK3C3	turquoise	0.960940556
ENSG00000121879.3	PIK3CA	blue	0.905165081
ENSG00000051382.4	PIK3CB	turquoise	0.966831881
ENSG00000171608.11	PIK3CD	blue	0.689163898
ENSG00000100100.8	PIK3IP1	blue	0.76197394
ENSG00000145675.10	PIK3R1	brown	0.807660966
ENSG00000105647.10	PIK3R2	turquoise	0.919678873
ENSG00000117461.10	PIK3R3	turquoise	0.722479729
ENSG00000196455.3	PIK3R4	turquoise	0.92380158
ENSG00000141506.9	PIK3R5	magenta	0.90194458
ENSG00000174083.13	PIK3R6	brown	0.630120004
ENSG00000115020.12	PIKFYVE	turquoise	0.890869371
ENSG00000085514.11	PILRA	yellow	0.790452726

ENSG00000121716.14	PILRB	yellow	0.885362218
ENSG00000137193.9	PIM1	black	0.813723071
ENSG00000102096.9	PIM2	turquoise	0.93764116
ENSG00000198355.4	PIM3	blue	0.695490527
ENSG00000127445.9	PIN1	turquoise	0.900802603
ENSG00000102309.8	PIN4	turquoise	0.89298713
ENSG00000158828.5	PINK1	turquoise	0.858724149
ENSG00000234465.6	PINLYP	red	0.810700703
ENSG00000254093.4	PINX1	turquoise	0.837776957
ENSG00000150867.9	PIP4K2A	brown	0.896287847
ENSG00000141720.7	PIP4K2B	turquoise	0.958389295
ENSG00000166908.13	PIP4K2C	turquoise	0.975551359
ENSG00000143398.15	PIP5K1A	turquoise	0.930143734
ENSG00000107242.13	PIP5K1B	greenyellow	0.881519325
ENSG00000186111.4	PIP5K1C	turquoise	0.907635616
ENSG00000179761.7	PIPOX	green	0.683873884
ENSG00000087842.6	PIR	green	0.674062079
ENSG00000241878.5	PISD	turquoise	0.960759207
ENSG00000057757.5	PITHD1	turquoise	0.97081474
ENSG00000174238.10	PITPNA	turquoise	0.946253115
ENSG00000236618.2	PITPNA-AS1	turquoise	0.688731887
ENSG00000154217.10	PITPNC1	blue	0.815633586
ENSG00000110697.8	PITPNM1	turquoise	0.914075372
ENSG00000090975.8	PITPNM2	turquoise	0.886949423
ENSG00000091622.11	PITPNM3	turquoise	0.854874975
ENSG00000107959.11	PITRM1	turquoise	0.955105565
ENSG00000197181.7	PIWIL2	turquoise	0.650301161
ENSG00000134627.7	PIWIL4	turquoise	0.898493079
ENSG00000181191.11	PJA1	turquoise	0.952201002
ENSG00000198961.5	PJA2	turquoise	0.964757431
ENSG00000008710.13	PKD1	yellow	0.931424283
ENSG00000254681.2	PKD1P5	grey	0.410262022
ENSG00000250251.2	PKD1P6	turquoise	0.834522778
ENSG00000118762.3	PKD2	blue	0.908944042
ENSG00000162878.8	PKDCC	blue	0.563329741
ENSG00000171033.8	PKIA	turquoise	0.927994028
ENSG00000168734.9	PKIG	turquoise	0.82360811
ENSG00000067225.13	PKM	turquoise	0.938450924
ENSG00000220563.1	PKMP3	turquoise	0.904137698
ENSG00000123143.8	PKN1	turquoise	0.882344429
ENSG00000065243.14	PKN2	turquoise	0.815954794
ENSG00000160447.6	PKN3	black	0.825558766
ENSG00000160199.10	PKNOX1	turquoise	0.953831512
ENSG00000165495.11	PKNOX2	turquoise	0.830084529
ENSG00000057294.9	PKP2	turquoise	0.771198907
ENSG00000144283.17	PKP4	brown	0.628336637
ENSG00000144837.4	PLA1A	black	0.73905299
ENSG00000123739.6	PLA2G12A	turquoise	0.905762567

ENSG00000103066.8	PLA2G15	turquoise	0.931307035
ENSG00000176485.6	PLA2G16	brown	0.890235993
ENSG00000116711.8	PLA2G4A	magenta	0.679899482
ENSG00000243708.4	PLA2G4B	yellow	0.954280701
ENSG00000105499.9	PLA2G4C	blue	0.814349454
ENSG00000127472.6	PLA2G5	grey	0.087242967
ENSG00000184381.14	PLA2G6	turquoise	0.885342121
ENSG00000146070.12	PLA2G7	turquoise	0.809043179
ENSG00000137055.10	PLAA	turquoise	0.952993609
ENSG00000173261.4	PLAC8L1	turquoise	0.707430899
ENSG00000189129.9	PLAC9	grey	0.512415044
ENSG00000118495.14	PLAGL1	turquoise	0.82956454
ENSG00000126003.6	PLAGL2	turquoise	0.848670202
ENSG00000104368.13	PLAT	brown	0.569725244
ENSG00000122861.11	PLAU	magenta	0.571486734
ENSG00000011422.7	PLAUR	black	0.819780525
ENSG00000163803.8	PLB1	turquoise	0.867753845
ENSG00000121316.6	PLBD1	magenta	0.756964892
ENSG00000151176.3	PLBD2	turquoise	0.91641192
ENSG00000182621.12	PLCB1	turquoise	0.823110635
ENSG00000137841.7	PLCB2	turquoise	0.856414541
ENSG00000149782.7	PLCB3	blue	0.666222418
ENSG00000101333.12	PLCB4	brown	0.711260982
ENSG00000187091.9	PLCD1	green	0.808921034
ENSG00000161714.7	PLCD3	brown	0.503770235
ENSG00000115556.9	PLCD4	green	0.481685527
ENSG00000138193.10	PLCE1	blue	0.762785303
ENSG00000124181.10	PLCG1	turquoise	0.80495958
ENSG00000197943.5	PLCG2	blue	0.590982777
ENSG00000114805.12	PLCH1	turquoise	0.774094392
ENSG00000149527.13	PLCH2	turquoise	0.834663728
ENSG00000115896.11	PLCL1	brown	0.877170757
ENSG00000154822.11	PLCL2	turquoise	0.906505015
ENSG00000182378.8	PLCXD1	turquoise	0.747449794
ENSG00000182836.5	PLCXD3	turquoise	0.77411611
ENSG00000075651.11	PLD1	brown	0.919765341
ENSG00000129219.9	PLD2	blue	0.8068933
ENSG00000105223.14	PLD3	turquoise	0.932084671
ENSG00000166428.8	PLD4	magenta	0.761587801
ENSG00000180287.12	PLD5	greenyellow	0.831507859
ENSG00000179598.5	PLD6	turquoise	0.939914407
ENSG00000178209.10	PLEC	turquoise	0.854841983
ENSG00000115956.9	PLEK	midnightblue	0.827732241
ENSG00000107679.10	PLEKHA1	turquoise	0.93862791
ENSG00000169499.10	PLEKHA2	turquoise	0.942991798
ENSG00000116095.6	PLEKHA3	turquoise	0.954965289
ENSG00000105559.7	PLEKHA4	black	0.76240855
ENSG00000052126.10	PLEKHA5	turquoise	0.924298104

ENSG00000143850.8	PLEKHA6	turquoise	0.923958877
ENSG00000166689.10	PLEKHA7	blue	0.833062993
ENSG00000106086.14	PLEKHA8	turquoise	0.882963272
ENSG00000021300.9	PLEKHB1	brown	0.682050473
ENSG00000115762.12	PLEKHB2	turquoise	0.974935307
ENSG00000166289.5	PLEKHF1	black	0.716059196
ENSG00000175895.3	PLEKHF2	blue	0.772660827
ENSG00000120278.10	PLEKHG1	blue	0.714227024
ENSG00000090924.10	PLEKHG2	black	0.79664735
ENSG00000126822.11	PLEKHG3	brown	0.936862311
ENSG00000196155.8	PLEKHG4	yellow	0.846091984
ENSG00000171680.16	PLEKHG5	turquoise	0.879569591
ENSG00000054690.9	PLEKHH1	brown	0.934702037
ENSG00000152527.9	PLEKHH2	blue	0.620566
ENSG00000068137.10	PLEKHH3	blue	0.728242008
ENSG00000104886.5	PLEKHJ1	turquoise	0.877808671
ENSG00000225190.4	PLEKHM1	turquoise	0.88848954
ENSG00000214176.5	PLEKHM1P	yellow	0.915164259
ENSG00000116786.7	PLEKHM2	turquoise	0.878243266
ENSG00000023902.9	PLEKHO1	turquoise	0.768886851
ENSG00000241839.5	PLEKHO2	green	0.801624226
ENSG00000183281.10	PLGLB1	yellow	0.680106246
ENSG00000107020.5	PLGRKT	blue	0.838637795
ENSG00000166819.7	PLIN1	green	0.661310518
ENSG00000147872.5	PLIN2	black	0.823512019
ENSG00000105355.4	PLIN3	blue	0.811734274
ENSG00000167676.3	PLIN4	green	0.626050076
ENSG00000214456.4	PLIN5	green	0.738391172
ENSG00000166851.10	PLK1	yellow	0.8790187
ENSG00000088970.11	PLK1S1	turquoise	0.907157259
ENSG00000145632.10	PLK2	turquoise	0.897853081
ENSG00000173846.8	PLK3	turquoise	0.698642653
ENSG00000142731.6	PLK4	turquoise	0.808958988
ENSG00000185988.7	PLK5	midnightblue	0.768183797
ENSG00000102934.5	PLLP	brown	0.848301366
ENSG00000198523.5	PLN	grey	0.251313221
ENSG00000083444.12	PLOD1	blue	0.677559285
ENSG00000152952.7	PLOD2	green	0.787605791
ENSG00000106397.7	PLOD3	blue	0.692223207
ENSG00000123560.9	PLP1	brown	0.868418993
ENSG00000102007.6	PLP2	black	0.802882548
ENSG00000171566.7	PLRG1	turquoise	0.897157916
ENSG00000102024.13	PLS3	brown	0.82996731
ENSG00000188313.8	PLSCR1	black	0.886159804
ENSG00000114698.10	PLSCR4	green	0.786316177
ENSG00000130300.4	PLVAP	magenta	0.689637032
ENSG00000161381.9	PLXDC1	brown	0.606115768
ENSG00000120594.12	PLXDC2	blue	0.899238047

ENSG00000114554.7	PLXNA1	yellow	0.807463565
ENSG00000076356.6	PLXNA2	turquoise	0.914302777
ENSG00000130827.5	PLXNA3	turquoise	0.850017982
ENSG00000221866.5	PLXNA4	turquoise	0.916889429
ENSG00000164050.8	PLXNB1	blue	0.797186328
ENSG00000196576.10	PLXNB2	blue	0.837522972
ENSG00000198753.7	PLXNB3	brown	0.888649957
ENSG00000136040.4	PLXNC1	turquoise	0.870156498
ENSG00000004399.8	PLXND1	blue	0.689133589
ENSG00000146281.5	PM20D2	turquoise	0.862937151
ENSG00000141682.11	PMAIP1	grey	0.150757316
ENSG00000124225.11	PMEPA1	turquoise	0.841783896
ENSG00000160783.15	PMF1	red	0.927383305
ENSG00000140464.15	PML	blue	0.763767235
ENSG00000100417.7	PMM1	turquoise	0.914134863
ENSG00000140650.7	PMM2	turquoise	0.809147934
ENSG00000147588.6	PMP2	green	0.664558173
ENSG00000109099.9	PMP22	brown	0.829685121
ENSG00000165688.7	PMPCA	turquoise	0.929487131
ENSG00000105819.9	PMPCB	turquoise	0.967585399
ENSG00000064933.12	PMS1	turquoise	0.912704674
ENSG00000122512.10	PMS2	turquoise	0.949485812
ENSG00000187953.6	PMS2CL	turquoise	0.896273573
ENSG00000078319.7	PMS2P1	turquoise	0.901066628
ENSG00000127957.12	PMS2P3	turquoise	0.85985102
ENSG00000067601.6	PMS2P4	turquoise	0.89851486
ENSG00000123965.12	PMS2P5	turquoise	0.866233827
ENSG00000233448.2	PMS2P9	turquoise	0.630872353
ENSG00000163344.5	PMVK	red	0.835291074
ENSG00000130822.11	PNCK	turquoise	0.88970817
ENSG00000132424.10	PNISR	yellow	0.933765036
ENSG00000127838.9	PNKD	turquoise	0.892603731
ENSG00000039650.5	PNKP	turquoise	0.910400347
ENSG00000146453.8	PNLDC1	turquoise	0.699024341
ENSG00000175535.6	PNLIP	grey	0.088209504
ENSG00000176903.3	PNMA1	turquoise	0.957002265
ENSG00000240694.4	PNMA2	turquoise	0.950051834
ENSG00000183837.8	PNMA3	turquoise	0.884880386
ENSG00000235961.3	PNMA6C	turquoise	0.563572498
ENSG00000182013.13	PNMAL1	turquoise	0.956288081
ENSG00000204851.5	PNMAL2	turquoise	0.890894538
ENSG00000141744.3	PNMT	turquoise	0.638025922
ENSG00000100941.4	PNN	turquoise	0.892816991
ENSG00000115946.3	PNO1	turquoise	0.912053934
ENSG00000168081.4	PNOC	midnightblue	0.708424724
ENSG00000198805.7	PNP	black	0.780180187
ENSG00000177666.11	PNPLA2	turquoise	0.857261273
ENSG00000006757.7	PNPLA4	blue	0.785137966

ENSG00000100341.7	PNPLA5	turquoise	0.843876216
ENSG00000032444.11	PNPLA6	turquoise	0.925981276
ENSG00000130653.11	PNPLA7	turquoise	0.773071651
ENSG00000135241.12	PNPLA8	turquoise	0.804585045
ENSG00000108439.5	PNPO	blue	0.864038761
ENSG00000138035.10	PNPT1	turquoise	0.844996514
ENSG00000146278.10	PNRC1	blue	0.855160198
ENSG00000164087.3	POC1A	turquoise	0.87237144
ENSG00000139323.9	POC1B	blue	0.890589358
ENSG00000152359.10	POC5	turquoise	0.899779143
ENSG00000174348.9	PODN	black	0.760442404
ENSG00000132000.7	PODNL1	black	0.626520246
ENSG00000128567.12	PODXL	black	0.717561035
ENSG00000114631.10	PODXL2	turquoise	0.88152147
ENSG00000101346.7	POFUT1	turquoise	0.875614847
ENSG00000186866.12	POFUT2	turquoise	0.849948477
ENSG00000143157.7	POGK	brown	0.87212506
ENSG00000163389.6	POGLUT1	turquoise	0.891111034
ENSG00000143442.17	POGZ	yellow	0.966395665
ENSG00000101868.6	POLA1	blue	0.91154615
ENSG00000014138.4	POLA2	turquoise	0.904768711
ENSG00000070501.7	POLB	turquoise	0.956723374
ENSG00000062822.8	POLD1	blue	0.845448042
ENSG00000106628.6	POLD2	turquoise	0.910885109
ENSG00000175482.4	POLD4	turquoise	0.621673348
ENSG00000004142.7	POLDIP2	turquoise	0.942150266
ENSG00000100227.13	POLDIP3	turquoise	0.894013734
ENSG00000177084.12	POLE	turquoise	0.771007091
ENSG00000148229.8	POLE3	turquoise	0.96021467
ENSG00000115350.7	POLE4	red	0.91542329
ENSG00000140521.7	POLG	turquoise	0.944569108
ENSG00000256525.2	POLG2	blue	0.883983559
ENSG00000170734.7	POLH	turquoise	0.879139217
ENSG00000101751.6	POLI	turquoise	0.864398964
ENSG00000122008.11	POLK	turquoise	0.859435775
ENSG00000166169.12	POLL	turquoise	0.933260221
ENSG00000122678.10	POLM	yellow	0.887376879
ENSG00000130997.12	POLN	green	0.572127316
ENSG00000068654.11	POLR1A	turquoise	0.947292723
ENSG00000125630.11	POLR1B	turquoise	0.913359441
ENSG00000171453.13	POLR1C	turquoise	0.941228121
ENSG00000186184.11	POLR1D	turquoise	0.964912343
ENSG00000137054.11	POLR1E	turquoise	0.941327124
ENSG00000181222.10	POLR2A	turquoise	0.920294351
ENSG00000047315.10	POLR2B	turquoise	0.962664077
ENSG00000102978.8	POLR2C	turquoise	0.967508531
ENSG00000144231.6	POLR2D	turquoise	0.881391524
ENSG00000099817.7	POLR2E	turquoise	0.921257813

ENSG00000100142.10	POLR2F	red	0.933799085
ENSG00000168002.7	POLR2G	red	0.902888282
ENSG00000163882.5	POLR2H	blue	0.878448719
ENSG00000105258.4	POLR2I	red	0.963994301
ENSG00000005075.11	POLR2J	red	0.939068089
ENSG00000168255.14	POLR2J3	turquoise	0.600852317
ENSG00000272655.1	POLR2J4	turquoise	0.856109931
ENSG00000147669.6	POLR2K	turquoise	0.935218783
ENSG00000177700.5	POLR2L	red	0.902221252
ENSG00000255529.3	POLR2M	blue	0.880789503
ENSG00000148606.8	POLR3A	turquoise	0.975658854
ENSG00000013503.5	POLR3B	turquoise	0.959099691
ENSG00000186141.4	POLR3C	turquoise	0.963239682
ENSG00000168495.8	POLR3D	turquoise	0.862674562
ENSG00000058600.11	POLR3E	turquoise	0.962801791
ENSG00000132664.7	POLR3F	turquoise	0.960339906
ENSG00000113356.6	POLR3G	turquoise	0.859484448
ENSG00000121851.8	POLR3GL	turquoise	0.872160757
ENSG00000100413.12	POLR3H	turquoise	0.853357547
ENSG00000161980.5	POLR3K	turquoise	0.942436944
ENSG00000099821.9	POLRMT	turquoise	0.926752846
ENSG00000196313.7	POM121	turquoise	0.945747963
ENSG00000135213.8	POM121C	turquoise	0.962944863
ENSG00000115138.6	POMC	grey	0.267749266
ENSG00000085998.9	POMGNT1	turquoise	0.96361729
ENSG00000144647.5	POMGNT2	turquoise	0.957212753
ENSG00000185900.5	POMK	turquoise	0.853916495
ENSG00000132963.7	POMP	turquoise	0.868571552
ENSG00000130714.11	POMT1	turquoise	0.933402957
ENSG00000009830.7	POMT2	brown	0.858661964
ENSG00000146707.10	POMZP3	turquoise	0.66640297
ENSG00000105854.8	PON2	green	0.916807031
ENSG00000105852.6	PON3	green	0.610077098
ENSG00000104356.6	POP1	turquoise	0.878785815
ENSG00000105171.5	POP4	turquoise	0.947886397
ENSG00000167272.6	POP5	turquoise	0.785802544
ENSG00000172336.4	POP7	turquoise	0.932959202
ENSG00000121577.9	POPDC2	turquoise	0.65324145
ENSG00000132429.5	POPDC3	turquoise	0.628251853
ENSG00000127948.9	POR	turquoise	0.904996151
ENSG00000102312.16	PORCN	turquoise	0.941059576
ENSG00000128513.10	POT1	turquoise	0.927830271
ENSG00000143190.17	POU2F1	yellow	0.841974554
ENSG00000028277.16	POU2F2	turquoise	0.688637381
ENSG00000185668.5	POU3F1	turquoise	0.783439543
ENSG00000184486.7	POU3F2	green	0.773597114
ENSG00000198914.2	POU3F3	blue	0.850439799
ENSG00000196767.4	POU3F4	turquoise	0.843606409

ENSG00000184271.11	POU6F1	turquoise	0.949583758
ENSG00000180817.7	PPA1	turquoise	0.938049971
ENSG00000138777.15	PPA2	turquoise	0.88059223
ENSG00000130810.15	PPAN	turquoise	0.937241335
ENSG00000067113.12	PPAP2A	blue	0.860300999
ENSG00000162407.8	PPAP2B	green	0.916823161
ENSG00000141934.5	PPAP2C	brown	0.851825102
ENSG00000203805.6	PPAPDC1A	blue	0.582706175
ENSG00000147535.12	PPAPDC1B	blue	0.856101998
ENSG00000205808.4	PPAPDC2	turquoise	0.927137155
ENSG00000160539.4	PPAPDC3	turquoise	0.901184345
ENSG00000186951.12	PPARA	green	0.92700214
ENSG00000112033.9	PPARD	turquoise	0.867403148
ENSG00000132170.15	PPARG	turquoise	0.787392719
ENSG00000109819.4	PPARGC1A	turquoise	0.826462793
ENSG00000155846.12	PPARGC1B	turquoise	0.887054483
ENSG00000128059.4	PPAT	turquoise	0.904600509
ENSG00000138621.7	PPCDC	brown	0.619143844
ENSG00000127125.7	PPCS	turquoise	0.90704379
ENSG00000125534.5	PPDPF	red	0.871966178
ENSG00000131626.11	PPFIA1	blue	0.94879826
ENSG00000139220.12	PPFIA2	turquoise	0.93687887
ENSG00000177380.9	PPFIA3	turquoise	0.907387298
ENSG00000143847.11	PPFIA4	turquoise	0.913745801
ENSG00000110841.9	PPFIBP1	brown	0.761900051
ENSG00000166387.7	PPFIBP2	brown	0.868914438
ENSG00000134283.13	PPHLN1	turquoise	0.980423884
ENSG00000196262.9	PPIA	turquoise	0.935387682
ENSG00000226038.4	PPIAP21	turquoise	0.784767326
ENSG00000198618.4	PPIAP22	turquoise	0.867706205
ENSG00000166794.4	PPIB	turquoise	0.780393059
ENSG00000168938.5	PPIC	green	0.571014278
ENSG00000171497.4	PPID	cyan	0.922945561
ENSG00000084072.12	PPIE	brown	0.767044026
ENSG00000243970.1	PPIEL	turquoise	0.793256925
ENSG00000108179.9	PPIF	blue	0.883338206
ENSG00000138398.11	PPIG	turquoise	0.957798016
ENSG00000171960.6	PPIH	turquoise	0.947695997
ENSG00000137168.7	PPIL1	turquoise	0.932910191
ENSG00000100023.13	PPIL2	turquoise	0.941084121
ENSG00000240344.4	PPIL3	turquoise	0.895880666
ENSG00000131013.3	PPIL4	blue	0.931448538
ENSG00000185250.11	PPIL6	pink	0.952227937
ENSG00000168781.17	PPIP5K1	turquoise	0.965695916
ENSG00000145725.15	PPIP5K2	turquoise	0.913987551
ENSG00000100614.13	PPM1A	turquoise	0.922961692
ENSG00000138032.16	PPM1B	turquoise	0.892692746
ENSG00000170836.7	PPM1D	turquoise	0.839785647

ENSG00000175175.4	PPM1E	greenyellow	0.772905768
ENSG00000100034.9	PPM1F	pink	0.840055234
ENSG00000115241.9	PPM1G	turquoise	0.945873776
ENSG00000111110.7	PPM1H	turquoise	0.882207998
ENSG00000155367.11	PPM1J	pink	0.839678095
ENSG00000163644.10	PPM1K	turquoise	0.895796837
ENSG00000163590.9	PPM1L	blue	0.847172064
ENSG00000164088.13	PPM1M	turquoise	0.911859442
ENSG00000213889.6	PPM1N	turquoise	0.77878112
ENSG00000214517.4	PPME1	turquoise	0.976626798
ENSG00000143224.13	PPOX	turquoise	0.910089486
ENSG00000172531.10	PPP1CA	turquoise	0.922917147
ENSG00000213639.5	PPP1CB	turquoise	0.942809095
ENSG00000186298.7	PPP1CC	turquoise	0.899486746
ENSG00000204569.5	PPP1R10	turquoise	0.928658948
ENSG00000204619.3	PPP1R11	turquoise	0.976663396
ENSG00000058272.11	PPP1R12A	turquoise	0.930996338
ENSG00000077157.16	PPP1R12B	turquoise	0.93849359
ENSG00000125503.8	PPP1R12C	turquoise	0.915987686
ENSG00000088808.12	PPP1R13B	turquoise	0.934077558
ENSG00000104881.10	PPP1R13L	black	0.772587684
ENSG00000167641.6	PPP1R14A	brown	0.758460642
ENSG00000173457.6	PPP1R14B	black	0.49463294
ENSG00000179967.11	PPP1R14BP3	grey	0.47500186
ENSG00000198729.4	PPP1R14C	turquoise	0.877651824
ENSG00000087074.7	PPP1R15A	black	0.543562209
ENSG00000158615.8	PPP1R15B	turquoise	0.871674499
ENSG00000160972.5	PPP1R16A	turquoise	0.832024479
ENSG00000101445.5	PPP1R16B	turquoise	0.862492129
ENSG00000146112.7	PPP1R18	black	0.881917921
ENSG00000135447.12	PPP1R1A	turquoise	0.77208387
ENSG00000131771.9	PPP1R1B	turquoise	0.780617084
ENSG00000184203.3	PPP1R2	turquoise	0.944508759
ENSG00000162869.11	PPP1R21	turquoise	0.963577337
ENSG00000196422.6	PPP1R26	turquoise	0.747329263
ENSG00000162148.6	PPP1R32	pink	0.942124051
ENSG00000160813.2	PPP1R35	red	0.842159993
ENSG00000165807.3	PPP1R36	pink	0.817218559
ENSG00000104866.6	PPP1R37	turquoise	0.938612988
ENSG00000173281.4	PPP1R3B	blue	0.711939186
ENSG00000119938.8	PPP1R3C	green	0.576102474
ENSG00000132825.5	PPP1R3D	green	0.852003333
ENSG00000235194.4	PPP1R3E	yellow	0.930739673
ENSG00000049769.8	PPP1R3F	turquoise	0.952991768
ENSG00000219607.2	PPP1R3G	green	0.508605653
ENSG00000115685.10	PPP1R7	turquoise	0.921068651
ENSG00000117751.13	PPP1R8	turquoise	0.965744425
ENSG00000158528.7	PPP1R9A	turquoise	0.946007687

ENSG00000108819.9	PPP1R9B	turquoise	0.898999118
ENSG00000113575.5	PPP2CA	turquoise	0.964999502
ENSG00000104695.8	PPP2CB	blue	0.88881479
ENSG00000105568.13	PPP2R1A	turquoise	0.930096997
ENSG00000137713.11	PPP2R1B	blue	0.82817869
ENSG00000221914.4	PPP2R2A	turquoise	0.919644669
ENSG00000156475.14	PPP2R2B	turquoise	0.957093486
ENSG00000074211.9	PPP2R2C	turquoise	0.913197181
ENSG00000175470.14	PPP2R2D	turquoise	0.96804344
ENSG00000073711.6	PPP2R3A	blue	0.862671593
ENSG00000167393.12	PPP2R3B	yellow	0.778576082
ENSG00000092020.6	PPP2R3C	blue	0.862415795
ENSG00000119383.15	PPP2R4	turquoise	0.935310605
ENSG00000066027.7	PPP2R5A	blue	0.852441623
ENSG00000068971.9	PPP2R5B	turquoise	0.941444758
ENSG00000078304.15	PPP2R5C	turquoise	0.956906136
ENSG00000112640.10	PPP2R5D	turquoise	0.971042618
ENSG00000154001.9	PPP2R5E	turquoise	0.960510284
ENSG00000138814.12	PPP3CA	turquoise	0.881390742
ENSG00000107758.11	PPP3CB	turquoise	0.958181593
ENSG00000120910.10	PPP3CC	turquoise	0.885956713
ENSG00000221823.6	PPP3R1	turquoise	0.928062734
ENSG00000149923.9	PPP4C	turquoise	0.849049367
ENSG00000154845.11	PPP4R1	turquoise	0.816288781
ENSG00000163605.10	PPP4R2	turquoise	0.793507622
ENSG00000119698.7	PPP4R4	turquoise	0.901141091
ENSG00000011485.10	PPP5C	turquoise	0.962413183
ENSG00000230510.2	PPP5D1	turquoise	0.792577109
ENSG00000119414.7	PPP6C	turquoise	0.938650852
ENSG00000105063.14	PPP6R1	turquoise	0.923267839
ENSG00000100239.11	PPP6R2	turquoise	0.960718818
ENSG00000110075.10	PPP6R3	turquoise	0.907950461
ENSG00000148840.6	PPRC1	blue	0.85820863
ENSG00000131238.12	PPT1	turquoise	0.941835892
ENSG00000221988.8	PPT2	turquoise	0.90905825
ENSG00000196850.4	PPTC7	turquoise	0.941127571
ENSG00000113593.7	PPWD1	turquoise	0.905863715
ENSG00000102103.11	PQBP1	turquoise	0.931694202
ENSG00000122490.14	PQLC1	turquoise	0.919306679
ENSG00000040487.8	PQLC2	turquoise	0.873035518
ENSG00000162976.8	PQLC3	blue	0.849135881
ENSG00000135617.3	PRADC1	red	0.893524513
ENSG00000243279.2	PRAF2	turquoise	0.929507936
ENSG00000133246.7	PRAM1	magenta	0.858360634
ENSG00000121335.10	PRB2	turquoise	0.698158883
ENSG00000198901.9	PRC1	turquoise	0.920261574
ENSG00000258725.1	PRC1-AS1	turquoise	0.841586515
ENSG00000143294.10	PRCC	turquoise	0.967983744

ENSG00000214140.6	PRCD	brown	0.862486928
ENSG00000137509.6	PRCP	blue	0.842824823
ENSG00000057657.10	PRDM1	black	0.683243064
ENSG00000170325.10	PRDM10	turquoise	0.946199828
ENSG00000019485.8	PRDM11	turquoise	0.872511505
ENSG00000141956.9	PRDM15	turquoise	0.910111635
ENSG00000142611.12	PRDM16	green	0.882026501
ENSG00000116731.18	PRDM2	turquoise	0.966338755
ENSG00000110851.7	PRDM4	turquoise	0.960594062
ENSG00000138738.6	PRDM5	blue	0.795717643
ENSG00000152784.11	PRDM8	blue	0.646302762
ENSG00000117450.9	PRDX1	blue	0.820254649
ENSG00000167815.7	PRDX2	turquoise	0.909001549
ENSG00000165672.5	PRDX3	turquoise	0.914861738
ENSG00000123131.8	PRDX4	turquoise	0.848055677
ENSG00000126432.9	PRDX5	red	0.9447329
ENSG00000117592.8	PRDX6	green	0.827537196
ENSG00000138073.9	PREB	turquoise	0.926316155
ENSG00000169230.5	PRELID1	turquoise	0.839511238
ENSG00000188783.5	PRELP	green	0.608074662
ENSG00000085377.9	PREP	turquoise	0.955715974
ENSG00000138078.11	PREPL	turquoise	0.958543919
ENSG00000124126.9	PREX1	brown	0.888534064
ENSG00000046889.14	PREX2	green	0.748855758
ENSG00000139174.6	PRICKLE1	turquoise	0.873696656
ENSG00000163637.7	PRICKLE2	turquoise	0.87848327
ENSG00000012211.8	PRICKLE3	black	0.854781595
ENSG00000124593.10	PRICKLE4	yellow	0.888591619
ENSG00000198056.9	PRIM1	turquoise	0.852289197
ENSG00000146143.13	PRIM2	blue	0.779281978
ENSG00000175785.8	PRIMA1	brown	0.891755154
ENSG00000164306.6	PRIMPOL	blue	0.910329951
ENSG00000132356.7	PRKAA1	turquoise	0.883416576
ENSG00000162409.6	PRKAA2	turquoise	0.926085018
ENSG00000111725.6	PRKAB1	turquoise	0.844642596
ENSG00000131791.6	PRKAB2	turquoise	0.878536201
ENSG00000072062.9	PRKACA	turquoise	0.955861944
ENSG00000142875.15	PRKACB	turquoise	0.946136718
ENSG00000181929.7	PRKAG1	turquoise	0.936579063
ENSG00000106617.9	PRKAG2	turquoise	0.942718578
ENSG00000239911.1	PRKAG2-AS1	turquoise	0.886297164
ENSG00000108946.10	PRKAR1A	turquoise	0.953284735
ENSG00000188191.10	PRKAR1B	turquoise	0.880579208
ENSG00000114302.11	PRKAR2A	blue	0.917894435
ENSG00000224424.7	PRKAR2A-AS1	brown	0.623693954
ENSG00000005249.8	PRKAR2B	turquoise	0.919435944
ENSG00000154229.7	PRKCA	blue	0.865194466
ENSG00000166501.8	PRKCB	turquoise	0.818400904

ENSG00000163932.9	PRKCD	turquoise	0.915941509
ENSG00000170955.9	PRKCDBP	red	0.489722594
ENSG00000171132.9	PRKCE	turquoise	0.928307328
ENSG00000126583.6	PRKCG	turquoise	0.831004529
ENSG00000027075.9	PRKCH	turquoise	0.841305597
ENSG00000163558.8	PRKCI	turquoise	0.949641104
ENSG00000065675.10	PRKCQ	brown	0.907999482
ENSG00000237943.2	PRKCQ-AS1	brown	0.879353275
ENSG00000130175.5	PRKCSH	blue	0.863209233
ENSG00000067606.11	PRKCZ	turquoise	0.929658428
ENSG00000184304.10	PRKD1	blue	0.82357617
ENSG00000105287.8	PRKD2	blue	0.729125715
ENSG00000115825.5	PRKD3	green	0.810156712
ENSG00000253729.3	PRKDC	turquoise	0.963328317
ENSG00000185532.10	PRKG1	turquoise	0.656976199
ENSG00000180228.8	PRKRA	turquoise	0.952947294
ENSG00000128563.9	PRKRIP1	turquoise	0.910481895
ENSG00000137492.3	PRKRIR	turquoise	0.92554336
ENSG00000183943.5	PRKX	magenta	0.690595915
ENSG00000259205.2	PRKXP1	turquoise	0.561702836
ENSG00000099725.10	PRKY	grey	0.278906736
ENSG00000172179.7	PRL	grey	0.148526276
ENSG00000113494.12	PRLR	turquoise	0.714948039
ENSG00000126457.16	PRMT1	turquoise	0.949592078
ENSG00000164169.8	PRMT10	turquoise	0.928215879
ENSG00000160310.12	PRMT2	turquoise	0.870952041
ENSG00000185238.8	PRMT3	turquoise	0.892679344
ENSG00000100462.11	PRMT5	turquoise	0.939094951
ENSG00000198890.6	PRMT6	turquoise	0.926737044
ENSG00000132600.12	PRMT7	turquoise	0.915937577
ENSG00000111218.7	PRMT8	turquoise	0.879855245
ENSG00000171867.12	PRNP	turquoise	0.957885749
ENSG00000167525.9	PROCA1	pink	0.859466818
ENSG00000101000.4	PROCR	magenta	0.647548107
ENSG00000100033.12	PRODH	green	0.786041827
ENSG00000007062.7	PROM1	brown	0.533527106
ENSG00000155066.11	PROM2	yellow	0.901537116
ENSG00000162997.11	PRORSD1P	turquoise	0.791189751
ENSG00000184500.10	PROS1	pink	0.85539806
ENSG00000147471.7	PROSC	turquoise	0.944549412
ENSG00000120685.15	PROSER1	turquoise	0.941122891
ENSG00000225778.1	PROSER2-AS1	turquoise	0.614787646
ENSG00000117707.11	PROX1	brown	0.895479167
ENSG00000165630.9	PRPF18	turquoise	0.906450016
ENSG00000110107.4	PRPF19	turquoise	0.969110742
ENSG00000117360.8	PRPF3	yellow	0.929068914
ENSG00000105618.9	PRPF31	red	0.941945795
ENSG00000134748.12	PRPF38A	blue	0.954189496

ENSG00000134186.7	PRPF38B	turquoise	0.882494671
ENSG00000185246.13	PRPF39	turquoise	0.910946929
ENSG00000136875.8	PRPF4	turquoise	0.946828174
ENSG00000196504.11	PRPF40A	turquoise	0.855380038
ENSG00000110844.9	PRPF40B	turquoise	0.888548872
ENSG00000112739.12	PRPF4B	turquoise	0.93662011
ENSG00000101161.6	PRPF6	turquoise	0.940715577
ENSG00000174231.12	PRPF8	turquoise	0.963405422
ENSG00000112619.6	PRPH2	turquoise	0.762021402
ENSG00000147224.6	PRPS1	turquoise	0.960152117
ENSG00000232630.1	PRPS1P2	blue	0.808761251
ENSG00000101911.8	PRPS2	turquoise	0.944491308
ENSG00000161542.12	PRPSAP1	turquoise	0.917911887
ENSG00000141127.10	PRPSAP2	turquoise	0.927367327
ENSG00000126464.9	PRR12	turquoise	0.929262928
ENSG00000205352.6	PRR13	turquoise	0.866692468
ENSG00000156858.7	PRR14	yellow	0.926161097
ENSG00000183530.9	PRR14L	turquoise	0.949158934
ENSG00000184838.10	PRR16	turquoise	0.891134692
ENSG00000176381.4	PRR18	brown	0.887308502
ENSG00000188368.5	PRR19	turquoise	0.828836003
ENSG00000212123.3	PRR22	turquoise	0.61375153
ENSG00000257704.2	PRR24	black	0.625635303
ENSG00000204576.7	PRR3	turquoise	0.9263947
ENSG00000111215.7	PRR4	turquoise	0.860876516
ENSG00000186654.16	PRR5	grey	0.480643422
ENSG00000135362.9	PRR5L	brown	0.887810871
ENSG00000131188.7	PRR7	turquoise	0.719179859
ENSG00000164244.16	PRRC1	turquoise	0.841201855
ENSG00000204469.8	PRRC2A	turquoise	0.951899329
ENSG00000130723.13	PRRC2B	turquoise	0.958663022
ENSG00000117523.11	PRRC2C	turquoise	0.916955795
ENSG00000130962.13	PRRG1	brown	0.929217516
ENSG00000130032.11	PRRG3	turquoise	0.842639098
ENSG00000135378.3	PRRG4	turquoise	0.587463637
ENSG00000204314.6	PRRT1	turquoise	0.893315651
ENSG00000167371.12	PRRT2	turquoise	0.915750849
ENSG00000163704.7	PRRT3	turquoise	0.897254538
ENSG00000230082.1	PRRT3-AS1	grey	0.484706015
ENSG00000224940.4	PRRT4	turquoise	0.784539267
ENSG00000116132.7	PRRX1	green	0.776235628
ENSG00000204983.8	PRSS1	grey	0.070682182
ENSG00000164099.3	PRSS12	turquoise	0.6396972
ENSG00000150687.7	PRSS23	blue	0.645828925
ENSG00000172382.5	PRSS27	turquoise	0.704694542
ENSG00000010438.12	PRSS3	turquoise	0.672162845
ENSG00000146250.5	PRSS35	green	0.607470757
ENSG00000178226.6	PRSS36	turquoise	0.720000515

ENSG00000206549.8	PRSS50	blue	0.464234751
ENSG00000253649.1	PRSS51	turquoise	0.758361372
ENSG00000151006.7	PRSS53	yellow	0.926088827
ENSG00000237412.2	PRSS56	grey	0.426762352
ENSG00000099256.14	PRTFDC1	blue	0.85880337
ENSG00000196415.5	PRTN3	turquoise	0.567287917
ENSG00000143363.11	PRUNE	turquoise	0.963678288
ENSG00000106772.13	PRUNE2	brown	0.862007886
ENSG00000105227.10	PRX	black	0.7062773
ENSG00000197746.9	PSAP	turquoise	0.933515862
ENSG00000135069.9	PSAT1	green	0.873387816
ENSG00000059915.12	PSD	turquoise	0.814339508
ENSG00000146005.3	PSD2	green	0.782524764
ENSG00000156011.12	PSD3	turquoise	0.943951476
ENSG00000125637.11	PSD4	green	0.764327371
ENSG00000080815.14	PSEN1	brown	0.942341821
ENSG00000143801.12	PSEN2	turquoise	0.936294038
ENSG00000205155.3	PSENEN	pink	0.841549203
ENSG00000164985.10	PSIP1	turquoise	0.975620942
ENSG00000159792.5	PSKH1	blue	0.862579916
ENSG00000129084.13	PSMA1	turquoise	0.968435679
ENSG00000106588.6	PSMA2	turquoise	0.919327625
ENSG00000100567.8	PSMA3	turquoise	0.928748107
ENSG00000041357.11	PSMA4	turquoise	0.952474444
ENSG00000143106.8	PSMA5	turquoise	0.966273292
ENSG00000100902.6	PSMA6	turquoise	0.918186347
ENSG00000101182.10	PSMA7	turquoise	0.923877547
ENSG00000008018.8	PSMB1	turquoise	0.93519141
ENSG00000205220.7	PSMB10	red	0.856299534
ENSG00000126067.7	PSMB2	turquoise	0.970987981
ENSG00000108294.4	PSMB3	turquoise	0.925760537
ENSG00000159377.6	PSMB4	turquoise	0.920772726
ENSG00000100804.14	PSMB5	turquoise	0.929901763
ENSG00000142507.5	PSMB6	turquoise	0.921395685
ENSG00000136930.8	PSMB7	turquoise	0.957616415
ENSG00000204264.4	PSMB8	black	0.83847247
ENSG00000240065.3	PSMB9	black	0.799719074
ENSG00000100764.9	PSMC1	turquoise	0.944054942
ENSG00000161057.6	PSMC2	turquoise	0.957745544
ENSG00000165916.4	PSMC3	turquoise	0.852112975
ENSG00000131470.10	PSMC3IP	turquoise	0.794268361
ENSG00000013275.3	PSMC4	turquoise	0.937778979
ENSG00000087191.8	PSMC5	turquoise	0.926837405
ENSG00000100519.7	PSMC6	turquoise	0.95539055
ENSG00000173692.8	PSMD1	turquoise	0.967866228
ENSG00000101843.14	PSMD10	turquoise	0.940164339
ENSG00000108671.5	PSMD11	turquoise	0.946065797
ENSG00000197170.5	PSMD12	turquoise	0.920401436

ENSG00000185627.13	PSMD13	turquoise	0.941942664
ENSG00000115233.7	PSMD14	turquoise	0.949808581
ENSG00000175166.12	PSMD2	turquoise	0.960352365
ENSG00000108344.10	PSMD3	turquoise	0.970367404
ENSG00000159352.11	PSMD4	red	0.972444303
ENSG00000095261.9	PSMD5	turquoise	0.940804855
ENSG00000226752.3	PSMD5-AS1	turquoise	0.712102489
ENSG00000163636.6	PSMD6	turquoise	0.942636938
ENSG00000103035.6	PSMD7	turquoise	0.960689143
ENSG00000099341.7	PSMD8	turquoise	0.944276094
ENSG00000110801.9	PSMD9	turquoise	0.893704065
ENSG00000092010.10	PSME1	turquoise	0.754349272
ENSG00000100911.9	PSME2	blue	0.826357788
ENSG00000225131.1	PSME2P2	blue	0.571288085
ENSG00000131467.6	PSME3	turquoise	0.94368748
ENSG00000068878.10	PSME4	turquoise	0.849068337
ENSG00000125818.13	PSMF1	turquoise	0.96358246
ENSG00000183527.7	PSMG1	turquoise	0.929222745
ENSG00000128789.16	PSMG2	turquoise	0.920791567
ENSG00000157778.4	PSMG3	red	0.91779678
ENSG00000230487.3	PSMG3-AS1	turquoise	0.845666269
ENSG00000180822.7	PSMG4	turquoise	0.934923737
ENSG00000121390.13	PSPC1	turquoise	0.964322455
ENSG00000146733.9	PSPH	turquoise	0.85836247
ENSG00000226278.1	PSPHP1	grey	0.161333291
ENSG00000125650.4	PSPN	turquoise	0.749230056
ENSG00000134222.12	PSRC1	brown	0.769960789
ENSG00000179988.9	PSTK	turquoise	0.9556431
ENSG00000140368.8	PSTPIP1	grey	0.455657896
ENSG00000152229.14	PSTPIP2	magenta	0.73830881
ENSG00000169403.7	PTAFR	magenta	0.904293619
ENSG00000188647.8	PTAR1	green	0.807614912
ENSG00000011304.12	PTBP1	blue	0.86427942
ENSG00000117569.14	PTBP2	turquoise	0.921098916
ENSG00000119314.11	PTBP3	turquoise	0.954965986
ENSG00000106246.13	PTCD1	turquoise	0.937917509
ENSG00000049883.10	PTCD2	turquoise	0.916833192
ENSG00000132300.14	PTCD3	turquoise	0.951331768
ENSG00000185920.11	PTCH1	turquoise	0.715729782
ENSG00000117425.9	PTCH2	turquoise	0.83925828
ENSG00000165186.9	PTCHD1	turquoise	0.847536959
ENSG00000204624.6	PTCHD2	midnightblue	0.722406857
ENSG00000224597.5	PTCHD3P1	blue	0.866752896
ENSG00000156471.8	PTDSS1	turquoise	0.973480217
ENSG00000174915.7	PTDSS2	turquoise	0.828214704
ENSG00000171862.5	PTEN	turquoise	0.915041053
ENSG00000183134.4	PTGDR2	grey	0.240021117
ENSG00000107317.7	PTGDS	brown	0.700245017

ENSG00000160951.3	PTGER1	turquoise	0.503610901
ENSG00000148344.10	PTGES	grey	0.400536445
ENSG00000148334.10	PTGES2	turquoise	0.916939039
ENSG00000110958.11	PTGES3	cyan	0.946980391
ENSG00000267060.1	PTGES3L	turquoise	0.853563863
ENSG00000234518.1	PTGES3P1	cyan	0.899935697
ENSG00000217643.1	PTGES3P2	turquoise	0.84822687
ENSG00000134247.9	PTGFRN	blue	0.820210768
ENSG00000124212.5	PTGIS	turquoise	0.843497539
ENSG00000106853.12	PTGR1	turquoise	0.927256426
ENSG00000140043.7	PTGR2	blue	0.872839492
ENSG00000095303.10	PTGS1	magenta	0.909576295
ENSG00000073756.7	PTGS2	black	0.620416662
ENSG00000160801.9	PTH1R	grey	0.440391855
ENSG00000087494.11	PTHLH	turquoise	0.785277044
ENSG00000169398.15	PTK2	brown	0.929244082
ENSG00000120899.13	PTK2B	turquoise	0.88682062
ENSG00000101213.5	PTK6	blue	0.677591903
ENSG00000112655.11	PTK7	pink	0.879192463
ENSG00000187514.10	PTMA	brown	0.854659962
ENSG00000214182.5	PTMAP5	blue	0.527980397
ENSG00000159335.11	PTMS	turquoise	0.877696402
ENSG00000105894.7	PTN	green	0.647270615
ENSG00000104960.11	PTOV1	turquoise	0.900533706
ENSG00000268006.1	PTOV1-AS1	turquoise	0.889057874
ENSG00000112245.6	PTP4A1	turquoise	0.81578542
ENSG00000184007.13	PTP4A2	brown	0.797567418
ENSG00000184489.7	PTP4A3	yellow	0.759708197
ENSG00000158079.10	PTPDC1	turquoise	0.933255832
ENSG00000165996.9	PTPLA	turquoise	0.751105727
ENSG00000074696.8	PTPLAD1	blue	0.899578017
ENSG00000188921.12	PTPLAD2	turquoise	0.792546341
ENSG00000206527.5	PTPLB	turquoise	0.936750729
ENSG00000110536.9	PTPMT1	blue	0.863040212
ENSG00000196396.5	PTPN1	turquoise	0.863028461
ENSG00000179295.11	PTPN11	brown	0.77573502
ENSG00000127947.11	PTPN12	blue	0.868940607
ENSG00000163629.8	PTPN13	turquoise	0.817389304
ENSG00000152104.7	PTPN14	green	0.757702127
ENSG00000072135.8	PTPN18	yellow	0.903928349
ENSG00000175354.14	PTPN2	turquoise	0.926485693
ENSG00000070778.8	PTPN21	green	0.784613997
ENSG00000076201.10	PTPN23	turquoise	0.921655141
ENSG00000088179.4	PTPN4	turquoise	0.932628228
ENSG00000110786.13	PTPN5	turquoise	0.855990203
ENSG00000111679.12	PTPN6	magenta	0.951546911
ENSG00000143851.11	PTPN7	midnightblue	0.85088172
ENSG00000169410.5	PTPN9	turquoise	0.875008625

ENSG00000132670.16	PTPRA	blue	0.86578122
ENSG00000127329.10	PTPRB	brown	0.603252224
ENSG00000081237.14	PTPRC	magenta	0.907531753
ENSG00000213402.2	PTPRCAP	yellow	0.587137257
ENSG00000153707.11	PTPRD	brown	0.92769605
ENSG00000132334.12	PTPRE	turquoise	0.917770563
ENSG00000142949.12	PTPRF	turquoise	0.906429182
ENSG00000144724.14	PTPRG	turquoise	0.798738652
ENSG00000241472.2	PTPRG-AS1	turquoise	0.776049326
ENSG00000080031.5	PTPRH	brown	0.740743484
ENSG00000152894.10	PTPRK	brown	0.896068636
ENSG00000173482.12	PTPRM	turquoise	0.952758522
ENSG00000054356.9	PTPRN	turquoise	0.883041124
ENSG00000155093.13	PTPRN2	turquoise	0.934092367
ENSG00000151490.9	PTPRO	turquoise	0.871062204
ENSG00000153233.8	PTPRR	turquoise	0.867028361
ENSG00000105426.10	PTPRS	turquoise	0.891883149
ENSG00000196090.8	PTPRT	turquoise	0.855615555
ENSG00000060656.15	PTPRU	turquoise	0.761072767
ENSG00000106278.7	PTPRZ1	green	0.875842767
ENSG00000177469.12	PTRF	brown	0.659739929
ENSG00000141378.13	PTRH2	turquoise	0.945233425
ENSG00000184924.5	PTRHD1	red	0.925085349
ENSG00000150787.3	PTS	turquoise	0.904709617
ENSG00000164611.8	PTTG1	turquoise	0.701667296
ENSG00000183255.7	PTTG1IP	black	0.841513467
ENSG00000179950.9	PUF60	turquoise	0.919828892
ENSG00000134644.11	PUM1	turquoise	0.969726732
ENSG00000055917.11	PUM2	turquoise	0.950185079
ENSG00000185129.4	PURA	turquoise	0.860511477
ENSG00000146676.5	PURB	turquoise	0.97029525
ENSG00000172733.10	PURG	turquoise	0.849966865
ENSG00000177192.9	PUS1	turquoise	0.847233177
ENSG00000162927.9	PUS10	turquoise	0.840590098
ENSG00000110060.4	PUS3	turquoise	0.863448841
ENSG00000091127.9	PUS7	turquoise	0.914772042
ENSG00000129317.10	PUS7L	turquoise	0.876625326
ENSG00000169972.7	PUSL1	red	0.881514484
ENSG00000100362.8	PVALB	brown	0.535934314
ENSG00000073008.10	PVR	turquoise	0.846569056
ENSG00000213413.2	PVRIG	blue	0.684509402
ENSG00000110400.6	PVRL1	brown	0.897913272
ENSG00000130202.5	PVRL2	blue	0.668516696
ENSG00000177707.6	PVRL3	blue	0.793570357
ENSG00000257151.1	PWAR6	turquoise	0.931382661
ENSG00000136045.7	PWP1	turquoise	0.959482597
ENSG00000241945.3	PWP2	turquoise	0.926324796
ENSG00000259905.1	PWRN1	turquoise	0.74232081

ENSG00000170234.8	PWWP2A	turquoise	0.893911258
ENSG00000171813.10	PWWP2B	turquoise	0.859763049
ENSG00000168994.9	PXDC1	black	0.843300476
ENSG00000130508.6	PXDN	blue	0.550038942
ENSG00000168297.11	PXK	brown	0.946183115
ENSG00000176894.5	PXMP2	blue	0.677174124
ENSG00000089159.11	PXN	blue	0.694718707
ENSG00000255857.1	PXN-AS1	blue	0.800398239
ENSG00000103490.13	PYCARD	magenta	0.862857906
ENSG00000183010.12	PYCR1	cyan	0.838436087
ENSG00000143811.12	PYCR2	blue	0.92028908
ENSG00000104524.9	PYCRL	turquoise	0.81675049
ENSG00000100994.7	PYGB	turquoise	0.773843683
ENSG00000100504.12	PYGL	black	0.670186337
ENSG00000068976.9	PYGM	green	0.450336841
ENSG00000171016.7	PYGO1	blue	0.813484212
ENSG00000163348.3	PYGO2	turquoise	0.932742748
ENSG00000121350.11	PYROXD1	blue	0.828369789
ENSG00000119943.6	PYROXD2	yellow	0.835245871
ENSG00000145337.4	PYURF	grey	0.390180408
ENSG00000237575.4	PYY2	grey	0.600772133
ENSG00000172053.10	QARS	turquoise	0.892919143
ENSG00000151552.7	QDPR	brown	0.848348193
ENSG00000112531.12	QKI	blue	0.777925536
ENSG00000115828.11	QPCT	greenyellow	0.788168613
ENSG00000011478.7	QPCTL	turquoise	0.941933479
ENSG00000103485.13	QPRT	blue	0.643984047
ENSG00000198218.6	QRICH1	turquoise	0.982582898
ENSG00000129646.9	QRICH2	greenyellow	0.615915107
ENSG00000130348.7	QRSL1	turquoise	0.908382313
ENSG00000060749.10	QSER1	blue	0.885850505
ENSG00000116260.12	QSOX1	turquoise	0.941650483
ENSG00000165661.11	QSOX2	turquoise	0.958062679
ENSG00000213339.4	QTRT1	yellow	0.923474532
ENSG00000151576.6	QTRTD1	turquoise	0.913357381
ENSG00000104679.6	R3HCC1	red	0.88229429
ENSG00000166024.9	R3HCC1L	turquoise	0.907409158
ENSG00000048991.12	R3HDM1	turquoise	0.956113827
ENSG00000179912.15	R3HDM2	turquoise	0.962193466
ENSG00000198858.5	R3HDM4	turquoise	0.921332709
ENSG00000084733.6	RAB10	turquoise	0.889573896
ENSG00000103769.5	RAB11A	turquoise	0.971817898
ENSG00000185236.7	RAB11B	red	0.913226207
ENSG00000269386.1	RAB11B-AS1	brown	0.667183948
ENSG00000107560.6	RAB11FIP2	turquoise	0.958033025
ENSG00000090565.11	RAB11FIP3	turquoise	0.946830703
ENSG00000131242.11	RAB11FIP4	brown	0.733907436
ENSG00000135631.11	RAB11FIP5	turquoise	0.953065558

ENSG00000206418.3	RAB12	turquoise	0.964390168
ENSG00000143545.4	RAB13	black	0.796575775
ENSG00000119396.6	RAB14	turquoise	0.95625554
ENSG00000139998.10	RAB15	turquoise	0.90298252
ENSG00000099246.12	RAB18	turquoise	0.949578176
ENSG00000138069.12	RAB1A	turquoise	0.962796766
ENSG00000174903.10	RAB1B	turquoise	0.940348997
ENSG00000139832.3	RAB20	black	0.804587688
ENSG00000080371.4	RAB21	turquoise	0.921873637
ENSG00000124209.3	RAB22A	turquoise	0.970775984
ENSG00000112210.7	RAB23	blue	0.814399802
ENSG00000169228.9	RAB24	turquoise	0.940713592
ENSG00000167964.8	RAB26	turquoise	0.817549365
ENSG00000069974.11	RAB27A	blue	0.785107057
ENSG00000041353.5	RAB27B	turquoise	0.758645895
ENSG00000157869.10	RAB28	blue	0.893685955
ENSG00000104388.10	RAB2A	turquoise	0.972582695
ENSG00000129472.8	RAB2B	turquoise	0.967365258
ENSG00000137502.5	RAB30	blue	0.835398829
ENSG00000246067.3	RAB30-AS1	blue	0.839679268
ENSG00000168461.8	RAB31	green	0.898813864
ENSG00000118508.4	RAB32	magenta	0.802175222
ENSG00000134594.4	RAB33A	brown	0.868379593
ENSG00000172007.5	RAB33B	turquoise	0.800273489
ENSG00000109113.13	RAB34	blue	0.619088075
ENSG00000111737.7	RAB35	turquoise	0.943125916
ENSG00000100228.8	RAB36	pink	0.915294334
ENSG00000172794.15	RAB37	greenyellow	0.776451584
ENSG00000155961.4	RAB39B	turquoise	0.963985663
ENSG00000105649.5	RAB3A	turquoise	0.9122878
ENSG00000169213.6	RAB3B	greenyellow	0.87311652
ENSG00000152932.6	RAB3C	turquoise	0.916996176
ENSG00000105514.3	RAB3D	turquoise	0.913251586
ENSG00000115839.13	RAB3GAP1	turquoise	0.972196971
ENSG00000118873.11	RAB3GAP2	turquoise	0.95206364
ENSG00000167994.7	RAB3IL1	blue	0.678654064
ENSG00000127328.17	RAB3IP	brown	0.759903085
ENSG00000141542.6	RAB40B	turquoise	0.932162894
ENSG00000197562.5	RAB40C	turquoise	0.906286002
ENSG00000147127.4	RAB41	green	0.783684847
ENSG00000172780.12	RAB43	turquoise	0.901860299
ENSG00000168118.7	RAB4A	blue	0.876432124
ENSG00000167578.12	RAB4B	red	0.8911879
ENSG00000144566.6	RAB5A	blue	0.926367453
ENSG00000108774.10	RAB5C	blue	0.865580038
ENSG00000175582.15	RAB6A	turquoise	0.97290984
ENSG00000154917.6	RAB6B	turquoise	0.950945745
ENSG00000222014.4	RAB6C	turquoise	0.721112756

ENSG00000075785.8	RAB7A	turquoise	0.937336734
ENSG00000117280.8	RAB7L1	green	0.841716431
ENSG00000167461.7	RAB8A	turquoise	0.874837755
ENSG00000166128.8	RAB8B	turquoise	0.906013124
ENSG00000123595.5	RAB9A	blue	0.917344222
ENSG00000123570.3	RAB9B	turquoise	0.924209254
ENSG00000105404.6	RABAC1	red	0.962258517
ENSG00000029725.12	RABEP1	turquoise	0.873456435
ENSG00000177548.8	RABEP2	blue	0.767488145
ENSG00000136933.12	RABEPK	turquoise	0.954511811
ENSG00000011454.12	RABGAP1	blue	0.912854955
ENSG00000152061.17	RABGAP1L	turquoise	0.951272727
ENSG00000154710.11	RABGEF1	red	0.800465647
ENSG00000100949.10	RABGGTA	turquoise	0.94997051
ENSG00000137955.11	RABGGTB	turquoise	0.921588817
ENSG00000183155.4	RABIF	turquoise	0.934569996
ENSG00000144134.14	RABL2A	pink	0.932871254
ENSG00000079974.13	RABL2B	pink	0.95898967
ENSG00000144840.4	RABL3	blue	0.907247667
ENSG00000128581.11	RABL5	turquoise	0.930762605
ENSG00000196642.11	RABL6	turquoise	0.911822248
ENSG00000136238.13	RAC1	turquoise	0.87962517
ENSG00000128340.10	RAC2	magenta	0.826439954
ENSG00000169750.4	RAC3	greenyellow	0.835779413
ENSG00000161800.8	RACGAP1	turquoise	0.949167915
ENSG00000113456.14	RAD1	turquoise	0.966044349
ENSG00000152942.14	RAD17	turquoise	0.958655324
ENSG00000070950.5	RAD18	turquoise	0.907186939
ENSG00000164754.8	RAD21	turquoise	0.904262453
ENSG00000179262.5	RAD23A	turquoise	0.954740065
ENSG00000119318.8	RAD23B	turquoise	0.971384531
ENSG00000113522.9	RAD50	turquoise	0.958406696
ENSG00000245849.2	RAD51-AS1	yellow	0.90460758
ENSG00000111247.10	RAD51AP1	turquoise	0.775071251
ENSG00000108384.10	RAD51C	turquoise	0.926809043
ENSG00000185379.16	RAD51D	turquoise	0.962554112
ENSG00000002016.12	RAD52	yellow	0.916562844
ENSG00000197275.8	RAD54B	turquoise	0.802651362
ENSG00000085999.7	RAD54L	grey	0.784803963
ENSG00000164080.9	RAD54L2	turquoise	0.87074065
ENSG00000172613.3	RAD9A	yellow	0.928701138
ENSG00000157927.12	RADIL	turquoise	0.754173948
ENSG00000101146.8	RAE1	turquoise	0.967779048
ENSG00000132155.7	RAF1	turquoise	0.945014655
ENSG00000108557.13	RAI1	turquoise	0.92484517
ENSG00000039560.9	RAI14	brown	0.740639956
ENSG00000131831.13	RAI2	turquoise	0.822421275
ENSG00000006451.3	RALA	turquoise	0.915508741

ENSG00000144118.9	RALB	turquoise	0.884224831
ENSG00000017797.7	RALBP1	blue	0.89579932
ENSG00000174373.11	RALGAPA1	turquoise	0.926980484
ENSG00000188559.9	RALGAPA2	blue	0.906909146
ENSG00000170471.10	RALGAPB	turquoise	0.968516247
ENSG00000160271.10	RALGDS	turquoise	0.813642901
ENSG00000136828.14	RALGPS1	turquoise	0.944306281
ENSG00000116191.13	RALGPS2	turquoise	0.870506823
ENSG00000125970.7	RALY	blue	0.813993755
ENSG00000184672.7	RALYL	turquoise	0.930949968
ENSG00000132329.6	RAMP1	red	0.83368147
ENSG00000131477.6	RAMP2	grey	0.360806524
ENSG00000197291.4	RAMP2-AS1	blue	0.674322316
ENSG00000122679.4	RAMP3	black	0.767725931
ENSG00000132341.7	RAN	turquoise	0.957730724
ENSG00000099901.12	RANBP1	turquoise	0.909361411
ENSG00000141084.6	RANBP10	turquoise	0.922402767
ENSG00000153201.11	RANBP2	turquoise	0.934057271
ENSG00000031823.10	RANBP3	turquoise	0.948233139
ENSG00000164188.4	RANBP3L	green	0.857608169
ENSG00000137040.8	RANBP6	turquoise	0.940959597
ENSG00000010017.9	RANBP9	turquoise	0.944884149
ENSG00000100401.15	RANGAP1	turquoise	0.925413864
ENSG00000108961.9	RANGRF	turquoise	0.903949032
ENSG00000225125.2	RANP4	turquoise	0.787507631
ENSG00000116473.10	RAP1A	brown	0.866923878
ENSG00000127314.13	RAP1B	blue	0.921590342
ENSG00000076864.15	RAP1GAP	turquoise	0.855577424
ENSG00000132359.9	RAP1GAP2	turquoise	0.91322925
ENSG00000138698.10	RAP1GDS1	brown	0.791605822
ENSG00000125249.6	RAP2A	turquoise	0.935886465
ENSG00000181467.2	RAP2B	turquoise	0.947186708
ENSG00000123728.5	RAP2C	turquoise	0.908869425
ENSG00000107263.14	RAPGEF1	turquoise	0.945211427
ENSG00000109756.4	RAPGEF2	turquoise	0.94403836
ENSG00000079337.11	RAPGEF3	brown	0.759663041
ENSG00000091428.13	RAPGEF4	turquoise	0.764043216
ENSG00000228016.1	RAPGEF4-AS1	turquoise	0.826954649
ENSG00000136237.14	RAPGEF5	brown	0.823945688
ENSG00000158987.15	RAPGEF6	turquoise	0.906955929
ENSG00000108352.7	RAPGEFL1	turquoise	0.882963939
ENSG00000131759.13	RARA	turquoise	0.71359457
ENSG00000077092.14	RARB	turquoise	0.887004355
ENSG00000172819.12	RARG	green	0.746069338
ENSG00000133321.6	RARRES3	black	0.688516966
ENSG00000113643.4	RARS	turquoise	0.955824875
ENSG00000146282.13	RARS2	turquoise	0.899839431
ENSG00000145715.10	RASA1	turquoise	0.946175518

ENSG00000155903.7	RASA2	turquoise	0.877033049
ENSG00000185989.9	RASA3	blue	0.825438376
ENSG00000228903.2	RASA4CP	turquoise	0.862957531
ENSG00000233297.4	RASA4DP	turquoise	0.567335581
ENSG00000111344.7	RASAL1	turquoise	0.829450443
ENSG00000075391.12	RASAL2	turquoise	0.938195535
ENSG00000224687.1	RASAL2-AS1	turquoise	0.854797569
ENSG00000105122.8	RASAL3	magenta	0.910877727
ENSG00000108551.4	RASD1	magenta	0.53667057
ENSG00000100302.6	RASD2	turquoise	0.624849527
ENSG00000198915.7	RASGEF1A	turquoise	0.932127654
ENSG00000138670.12	RASGEF1B	turquoise	0.894237205
ENSG00000146090.11	RASGEF1C	magenta	0.614346723
ENSG00000058335.11	RASGRF1	turquoise	0.829278363
ENSG00000113319.7	RASGRF2	turquoise	0.735776847
ENSG00000172575.7	RASGRP1	turquoise	0.891870794
ENSG00000068831.14	RASGRP2	turquoise	0.867922211
ENSG00000152689.13	RASGRP3	brown	0.820031957
ENSG00000105538.4	RASIP1	black	0.787617953
ENSG00000100276.9	RASL10A	grey	0.347871661
ENSG00000141150.3	RASL10B	turquoise	0.851676918
ENSG00000122035.6	RASL11A	turquoise	0.794847077
ENSG00000128045.5	RASL11B	greenyellow	0.817213926
ENSG00000068028.13	RASSF1	blue	0.820139766
ENSG00000101265.11	RASSF2	brown	0.818305745
ENSG00000153179.7	RASSF3	brown	0.548261274
ENSG00000107551.16	RASSF4	brown	0.614075015
ENSG00000136653.15	RASSF5	turquoise	0.778145551
ENSG00000099849.10	RASSF7	turquoise	0.828620686
ENSG00000123094.11	RASSF8	green	0.768271821
ENSG00000246695.3	RASSF8-AS1	blue	0.80146058
ENSG00000161847.9	RAVER1	blue	0.875695349
ENSG00000162437.10	RAVER2	blue	0.845242532
ENSG00000139687.9	RB1	blue	0.915885691
ENSG00000023287.8	RB1CC1	turquoise	0.868383418
ENSG00000146587.13	RBAK	turquoise	0.93204775
ENSG00000273313.1	RBAKDN	turquoise	0.582727096
ENSG00000162521.14	RBBP4	turquoise	0.943085885
ENSG00000117222.9	RBBP5	turquoise	0.966786957
ENSG00000122257.14	RBBP6	turquoise	0.938598924
ENSG00000102054.13	RBBP7	turquoise	0.968940768
ENSG00000101773.12	RBBP8	blue	0.850081883
ENSG00000089050.10	RBBP9	blue	0.903155787
ENSG00000125826.15	RBCK1	yellow	0.926822063
ENSG00000101546.8	RBFA	turquoise	0.94034298
ENSG00000261126.3	RBFADN	yellow	0.831894183
ENSG00000078328.15	RBFOX1	turquoise	0.899936218
ENSG00000100320.18	RBFOX2	turquoise	0.962464907

ENSG00000167281.14	RBFOX3	turquoise	0.851885692
ENSG00000171174.9	RBKS	turquoise	0.723014752
ENSG00000080839.7	RBL1	blue	0.87574298
ENSG00000103479.10	RBL2	turquoise	0.869867013
ENSG00000182872.11	RBM10	turquoise	0.921920172
ENSG00000185272.9	RBM11	turquoise	0.914194755
ENSG00000244462.3	RBM12	turquoise	0.883894916
ENSG00000183808.7	RBM12B	turquoise	0.881231388
ENSG00000239306.4	RBM14	cyan	0.880908914
ENSG00000162775.10	RBM15	turquoise	0.867155267
ENSG00000179837.6	RBM15B	turquoise	0.974570896
ENSG00000134453.11	RBM17	turquoise	0.800869435
ENSG00000119446.9	RBM18	turquoise	0.941608615
ENSG00000122965.6	RBM19	turquoise	0.920025267
ENSG00000203867.7	RBM20	turquoise	0.84816897
ENSG00000086589.7	RBM22	turquoise	0.955760257
ENSG00000213411.2	RBM22P2	grey	0.667445464
ENSG00000100461.13	RBM23	turquoise	0.944828767
ENSG00000112183.10	RBM24	turquoise	0.87522152
ENSG00000119707.9	RBM25	turquoise	0.92923886
ENSG00000139746.11	RBM26	turquoise	0.964321265
ENSG00000227354.2	RBM26-AS1	turquoise	0.845941301
ENSG00000091009.6	RBM27	turquoise	0.935669291
ENSG00000106344.4	RBM28	blue	0.910377082
ENSG00000102317.13	RBM3	turquoise	0.706870372
ENSG00000184863.6	RBM33	turquoise	0.901847397
ENSG00000188739.10	RBM34	turquoise	0.766626341
ENSG00000132819.12	RBM38	blue	0.727087891
ENSG00000131051.16	RBM39	turquoise	0.943955784
ENSG00000173933.15	RBM4	turquoise	0.880577337
ENSG00000089682.12	RBM41	turquoise	0.788807259
ENSG00000126254.7	RBM42	turquoise	0.851203934
ENSG00000184898.6	RBM43	blue	0.835798811
ENSG00000155636.10	RBM45	turquoise	0.931318291
ENSG00000127993.10	RBM48	turquoise	0.880338955
ENSG00000173914.7	RBM4B	turquoise	0.859660228
ENSG00000003756.12	RBM5	yellow	0.97033544
ENSG00000004534.10	RBM6	yellow	0.906692563
ENSG00000076053.6	RBM7	turquoise	0.821977308
ENSG00000131795.8	RBM8A	turquoise	0.948101878
ENSG00000153250.13	RBMS1	turquoise	0.813392333
ENSG00000076067.7	RBMS2	black	0.829590378
ENSG00000144642.16	RBMS3	green	0.593562246
ENSG00000147274.10	RBMX	turquoise	0.942457152
ENSG00000134597.9	RBMX2	turquoise	0.876295958
ENSG00000213516.5	RBMXL1	blue	0.853498101
ENSG00000249465.1	RBMXP4	turquoise	0.747730352
ENSG00000114115.5	RBP1	grey	0.429513312

ENSG00000138207.8	RBP4	turquoise	0.882470835
ENSG00000139194.3	RBP5	turquoise	0.773397205
ENSG00000162444.11	RBP7	brown	0.776087154
ENSG00000168214.16	RBPJ	brown	0.897793171
ENSG00000157110.11	RBPMS	black	0.820702896
ENSG00000166831.4	RBPMS2	grey	0.454492281
ENSG00000100387.8	RBX1	red	0.932481688
ENSG00000135870.7	RC3H1	blue	0.887629941
ENSG00000056586.11	RC3H2	turquoise	0.960171788
ENSG00000159200.13	RCAN1	turquoise	0.870864937
ENSG00000172348.10	RCAN2	turquoise	0.914794009
ENSG00000117602.7	RCAN3	brown	0.786327058
ENSG00000136144.7	RCBTB1	brown	0.887100083
ENSG00000136161.8	RCBTB2	blue	0.833879194
ENSG00000180198.11	RCC1	cyan	0.898377807
ENSG00000179051.9	RCC2	turquoise	0.835665872
ENSG00000166965.8	RCCD1	turquoise	0.909152711
ENSG00000173653.3	RCE1	turquoise	0.852100961
ENSG00000163743.9	RCHY1	turquoise	0.950097564
ENSG00000120158.7	RCL1	turquoise	0.866709448
ENSG00000049449.4	RCN1	turquoise	0.933068231
ENSG00000214455.3	RCN1P2	turquoise	0.830047544
ENSG00000117906.9	RCN2	turquoise	0.943957308
ENSG00000142552.3	RCN3	blue	0.666227438
ENSG00000089902.8	RCOR1	turquoise	0.910078604
ENSG00000167771.5	RCOR2	turquoise	0.908354833
ENSG00000117625.9	RCOR3	turquoise	0.936503342
ENSG00000198771.6	RCSD1	brown	0.710377587
ENSG00000121039.5	RDH10	cyan	0.862004937
ENSG00000072042.8	RDH11	turquoise	0.885956657
ENSG00000139988.5	RDH12	midnightblue	0.71331582
ENSG00000160439.11	RDH13	turquoise	0.856870424
ENSG00000240857.1	RDH14	turquoise	0.955619799
ENSG00000135437.5	RDH5	blue	0.72133626
ENSG00000137710.10	RDX	blue	0.818043796
ENSG00000100918.8	REC8	yellow	0.934558784
ENSG00000122707.7	RECK	turquoise	0.8873616
ENSG00000004700.11	RECQL	turquoise	0.759523049
ENSG00000160957.8	RECQL4	turquoise	0.847890832
ENSG00000108469.10	RECQL5	turquoise	0.908235707
ENSG00000068615.12	REEP1	turquoise	0.936314219
ENSG00000132563.11	REEP2	turquoise	0.902161316
ENSG00000165476.8	REEP3	brown	0.873780423
ENSG00000168476.7	REEP4	blue	0.770907381
ENSG00000129625.8	REEP5	turquoise	0.958054901
ENSG00000115255.6	REEP6	turquoise	0.780534961
ENSG00000115386.5	REG1A	grey	0.034352016
ENSG00000173039.14	RELA	blue	0.841353803

ENSG00000104856.9	RELB	cyan	0.680309263
ENSG00000181826.5	RELL1	black	0.808611809
ENSG00000164620.4	RELL2	turquoise	0.915816591
ENSG00000189056.9	RELN	turquoise	0.88506047
ENSG00000054967.8	RELT	blue	0.786942047
ENSG00000088320.3	REM1	grey	0.162169004
ENSG00000139890.5	REM2	turquoise	0.821311464
ENSG00000102032.8	RENBP	turquoise	0.843389678
ENSG00000214022.7	REPIN1	turquoise	0.917145743
ENSG00000135597.14	REPS1	turquoise	0.927973083
ENSG00000169891.13	REPS2	turquoise	0.934869391
ENSG00000157916.14	RER1	turquoise	0.919184932
ENSG00000142599.13	RERE	turquoise	0.9452757
ENSG00000134533.2	RERG	turquoise	0.862486658
ENSG00000111404.2	RERGL	grey	0.061438405
ENSG00000182698.7	RESP18	turquoise	0.569910878
ENSG00000084093.11	REST	blue	0.76510088
ENSG00000042445.9	RETSAT	blue	0.86679488
ENSG00000135945.5	REV1	turquoise	0.960650475
ENSG00000009413.11	REV3L	turquoise	0.893267612
ENSG00000079313.7	REXO1	turquoise	0.855475044
ENSG00000076043.5	REXO2	turquoise	0.862212406
ENSG00000148300.7	REXO4	turquoise	0.897447003
ENSG00000035928.10	RFC1	turquoise	0.897399786
ENSG00000049541.6	RFC2	turquoise	0.925142874
ENSG00000133119.8	RFC3	turquoise	0.836418775
ENSG00000163918.6	RFC4	turquoise	0.919265902
ENSG00000111445.9	RFC5	turquoise	0.943290588
ENSG00000092871.12	RFFL	brown	0.917585743
ENSG00000135002.7	RFK	turquoise	0.942282382
ENSG00000169733.7	RFNG	turquoise	0.841350717
ENSG00000225465.6	RFPL1S	turquoise	0.910407027
ENSG00000128253.9	RFPL2	turquoise	0.800623845
ENSG00000205853.6	RFPL3S	grey	0.712270324
ENSG00000163933.5	RFT1	turquoise	0.931884886
ENSG00000131378.9	RFTN1	black	0.763853774
ENSG00000162944.6	RFTN2	blue	0.650447779
ENSG00000143207.15	RFWD2	turquoise	0.963977289
ENSG00000168411.9	RFWD3	turquoise	0.921533833
ENSG00000132005.4	RFX1	yellow	0.90028009
ENSG00000080298.11	RFX3	pink	0.911906874
ENSG00000111783.8	RFX4	green	0.84014427
ENSG00000143390.13	RFX5	turquoise	0.874721516
ENSG00000181827.10	RFX7	turquoise	0.925643407
ENSG00000064490.9	RFXANK	blue	0.768261148
ENSG00000133111.3	RFXAP	blue	0.866904756
ENSG00000242732.3	RGAG4	turquoise	0.945922296
ENSG00000102760.12	RGCC	brown	0.712347803

ENSG00000143344.11	RGL1	turquoise	0.900386672
ENSG00000237441.5	RGL2	turquoise	0.849648715
ENSG00000205517.8	RGL3	magenta	0.677542498
ENSG00000182175.9	RGMA	green	0.683770595
ENSG00000174136.7	RGMB	turquoise	0.908114274
ENSG00000130988.8	RGN	green	0.848026512
ENSG00000107185.8	RGP1	turquoise	0.908671644
ENSG00000148604.9	RGR	green	0.519079693
ENSG00000090104.7	RGS1	magenta	0.678383012
ENSG00000148908.10	RGS10	magenta	0.869209218
ENSG00000076344.11	RGS11	yellow	0.897427626
ENSG00000159788.14	RGS12	turquoise	0.885169882
ENSG00000169220.13	RGS14	turquoise	0.764108755
ENSG00000143333.6	RGS16	cyan	0.708301476
ENSG00000091844.3	RGS17	turquoise	0.918831806
ENSG00000171700.9	RGS19	magenta	0.904230009
ENSG00000116741.6	RGS2	turquoise	0.769508384
ENSG00000147509.9	RGS20	turquoise	0.685441195
ENSG00000138835.18	RGS3	blue	0.719938681
ENSG00000117152.9	RGS4	midnightblue	0.86820388
ENSG00000143248.8	RGS5	brown	0.43515038
ENSG00000182901.11	RGS7	turquoise	0.918416065
ENSG00000186479.4	RGS7BP	turquoise	0.818512569
ENSG00000135824.8	RGS8	turquoise	0.829479837
ENSG00000108370.11	RGS9	turquoise	0.715903965
ENSG00000144468.12	RHBDD1	blue	0.954170805
ENSG00000005486.12	RHBDD2	turquoise	0.927225864
ENSG00000100263.9	RHBDD3	blue	0.766942198
ENSG00000007384.11	RHBDF1	yellow	0.711136254
ENSG00000129667.8	RHBDF2	magenta	0.937514619
ENSG00000103269.9	RHBDL1	turquoise	0.809878787
ENSG00000158315.6	RHBDL2	brown	0.856500643
ENSG00000141314.8	RHBDL3	turquoise	0.685445923
ENSG00000188672.12	RHCE	turquoise	0.692596143
ENSG00000140519.8	RHCG	green	0.600070148
ENSG00000106615.5	RHEB	turquoise	0.895065048
ENSG00000167550.6	RHEBL1	turquoise	0.581904765
ENSG00000171792.6	RHNO1	turquoise	0.956309718
ENSG00000067560.6	RHOA	blue	0.934818273
ENSG00000143878.8	RHOB	blue	0.753119874
ENSG00000072422.12	RHOBTB1	brown	0.820229502
ENSG00000008853.12	RHOBTB2	turquoise	0.801701035
ENSG00000164292.8	RHOBTB3	green	0.863153705
ENSG00000155366.12	RHOC	black	0.769733938
ENSG00000173156.2	RHOD	grey	0.299848742
ENSG00000139725.3	RHOF	turquoise	0.877915528
ENSG00000177105.9	RHOG	brown	0.857973131
ENSG00000126785.8	RHOJ	green	0.699448958

ENSG00000119729.6	RHOQ	turquoise	0.887221674
ENSG00000126858.12	RHOT1	turquoise	0.955816565
ENSG00000266145.1	RHOT1P1	turquoise	0.625580619
ENSG00000203616.2	RHOT1P2	grey	0.32399145
ENSG00000140983.9	RHOT2	turquoise	0.89483831
ENSG00000116574.4	RHOU	brown	0.87761911
ENSG00000104140.6	RHOV	turquoise	0.806689007
ENSG00000101883.4	RHOXF1	turquoise	0.461692203
ENSG00000158106.8	RHPN1	turquoise	0.861525873
ENSG00000131941.3	RHPN2	green	0.651707282
ENSG00000166405.10	RIC3	turquoise	0.946259456
ENSG00000177963.8	RIC8A	turquoise	0.954817183
ENSG00000111785.14	RIC8B	turquoise	0.891729404
ENSG00000164327.8	RICTOR	turquoise	0.848423286
ENSG00000080345.13	RIF1	turquoise	0.939637457
ENSG00000178796.8	RIIAD1	turquoise	0.747033478
ENSG00000167705.7	RILP	red	0.805322078
ENSG00000188026.7	RILPL1	brown	0.819422782
ENSG00000150977.9	RILPL2	blue	0.855849498
ENSG00000060709.9	RIMBP2	turquoise	0.895560186
ENSG00000177181.10	RIMKLA	turquoise	0.924189596
ENSG00000166532.11	RIMKLB	turquoise	0.852167513
ENSG00000079841.14	RIMS1	turquoise	0.860713616
ENSG00000176406.16	RIMS2	turquoise	0.830104924
ENSG00000117016.5	RIMS3	turquoise	0.904265111
ENSG00000101098.8	RIMS4	turquoise	0.817729741
ENSG00000174791.6	RIN1	turquoise	0.819096338
ENSG00000132669.8	RIN2	green	0.848686043
ENSG00000100599.11	RIN3	magenta	0.81346018
ENSG00000204227.4	RING1	turquoise	0.931774858
ENSG00000187994.9	RINL	turquoise	0.933122173
ENSG00000135249.3	RINT1	turquoise	0.943059865
ENSG00000124784.4	RIOK1	turquoise	0.927685038
ENSG00000058729.6	RIOK2	turquoise	0.908566843
ENSG00000101782.10	RIOK3	turquoise	0.864537002
ENSG00000137275.9	RIPK1	blue	0.866856517
ENSG00000104312.6	RIPK2	blue	0.84139821
ENSG00000203877.3	RIPPLY2	turquoise	0.909365581
ENSG00000143622.6	RIT1	blue	0.919931463
ENSG00000152214.8	RIT2	turquoise	0.86060027
ENSG00000140522.7	RLBP1	grey	0.223141862
ENSG00000117000.8	RLF	turquoise	0.933356506
ENSG00000131263.8	RLIM	turquoise	0.970599058
ENSG00000159753.9	RLTPR	turquoise	0.839159434
ENSG00000176623.7	RMDN1	blue	0.70492365
ENSG00000115841.15	RMDN2	turquoise	0.898034121
ENSG00000137824.11	RMDN3	blue	0.921554939
ENSG00000178966.11	RMI1	turquoise	0.777364192

ENSG00000175643.7	RMI2	turquoise	0.763260502
ENSG00000155906.12	RMND1	turquoise	0.95670294
ENSG00000153561.8	RMND5A	turquoise	0.930879767
ENSG00000145916.14	RMND5B	turquoise	0.944277108
ENSG00000269900.2	RMRP	grey	0.175885003
ENSG00000202198.1	RN7SK	grey	0.28711941
ENSG00000201078.1	RN7SKP214	turquoise	0.527330758
ENSG00000252051.1	RN7SKP276	turquoise	0.776452042
ENSG00000202058.1	RN7SKP80	turquoise	0.701328942
ENSG00000258486.2	RN7SL1	grey	0.206235559
ENSG00000265150.1	RN7SL2	grey	0.21427517
ENSG00000244425.2	RN7SL268P	turquoise	0.846376424
ENSG00000266037.1	RN7SL3	grey	0.1271122
ENSG00000263386.1	RN7SL358P	turquoise	0.686798058
ENSG00000263426.1	RN7SL471P	grey	0.220396027
ENSG00000240877.2	RN7SL521P	grey	0.400018881
ENSG00000239607.2	RN7SL573P	yellow	0.804693187
ENSG00000242037.2	RN7SL585P	green	0.610565278
ENSG00000239884.2	RN7SL608P	blue	0.611229727
ENSG00000265945.1	RN7SL721P	turquoise	0.605785492
ENSG00000243015.2	RN7SL737P	turquoise	0.838255204
ENSG00000243819.3	RN7SL832P	turquoise	0.561005701
ENSG00000223318.1	RNA5SP111	grey	0.265472311
ENSG00000207186.1	RNA5SP122	grey	0.467187962
ENSG00000252793.1	RNA5SP196	turquoise	0.699650493
ENSG00000199804.1	RNA5SP383	turquoise	0.451242793
ENSG00000201440.1	RNA5SP515	turquoise	0.651207401
ENSG00000129538.9	RNASE1	brown	0.851891021
ENSG00000169385.2	RNASE2	magenta	0.802668025
ENSG00000169413.2	RNASE6	magenta	0.863174055
ENSG00000104889.4	RNASEH2A	red	0.869851778
ENSG00000136104.14	RNASEH2B	turquoise	0.945431657
ENSG00000172922.4	RNASEH2C	turquoise	0.857490376
ENSG00000219200.6	RNASEK	turquoise	0.928676282
ENSG00000135828.7	RNASEL	turquoise	0.887709768
ENSG00000026297.11	RNASET2	turquoise	0.749050551
ENSG00000172602.5	RND1	turquoise	0.7200176
ENSG00000108830.7	RND2	turquoise	0.78422248
ENSG00000115963.9	RND3	turquoise	0.670432373
ENSG00000022840.11	RNF10	turquoise	0.970737918
ENSG00000239305.2	RNF103	turquoise	0.932335799
ENSG00000123091.4	RNF11	turquoise	0.930622436
ENSG00000157450.11	RNF111	turquoise	0.954917613
ENSG00000128482.11	RNF112	turquoise	0.880825194
ENSG00000125352.4	RNF113A	turquoise	0.940679921
ENSG00000124226.7	RNF114	turquoise	0.852176803
ENSG00000121848.9	RNF115	turquoise	0.973220199
ENSG00000137522.13	RNF121	turquoise	0.955339859

ENSG00000133874.1	RNF122	black	0.636369444
ENSG00000164068.11	RNF123	turquoise	0.965016299
ENSG00000101695.4	RNF125	brown	0.872732252
ENSG00000070423.13	RNF126	turquoise	0.863429492
ENSG00000133135.9	RNF128	turquoise	0.855399728
ENSG00000082996.15	RNF13	brown	0.919753443
ENSG00000113269.9	RNF130	turquoise	0.857978658
ENSG00000181481.9	RNF135	blue	0.719586073
ENSG00000134758.9	RNF138	turquoise	0.839219573
ENSG00000170881.4	RNF139	turquoise	0.93993902
ENSG00000245149.3	RNF139-AS1	yellow	0.883377086
ENSG00000013561.13	RNF14	turquoise	0.918383167
ENSG00000110315.2	RNF141	blue	0.796503772
ENSG00000151692.10	RNF144A	turquoise	0.923458798
ENSG00000228203.2	RNF144A-AS1	turquoise	0.87259781
ENSG00000137393.8	RNF144B	black	0.794013382
ENSG00000145860.7	RNF145	turquoise	0.946590942
ENSG00000118518.11	RNF146	turquoise	0.948613807
ENSG00000163162.4	RNF149	blue	0.879802409
ENSG00000170153.6	RNF150	turquoise	0.890801381
ENSG00000176641.6	RNF152	blue	0.518945142
ENSG00000141576.10	RNF157	turquoise	0.90894819
ENSG00000267128.1	RNF157-AS1	green	0.672081192
ENSG00000141622.9	RNF165	turquoise	0.894150969
ENSG00000158717.6	RNF166	turquoise	0.901649421
ENSG00000108523.11	RNF167	turquoise	0.940171684
ENSG00000163961.4	RNF168	turquoise	0.933176798
ENSG00000166439.5	RNF169	blue	0.863131836
ENSG00000120925.9	RNF170	turquoise	0.910320404
ENSG00000145428.10	RNF175	turquoise	0.928045501
ENSG00000164197.7	RNF180	turquoise	0.877038016
ENSG00000168894.5	RNF181	turquoise	0.855652641
ENSG00000180537.8	RNF182	grey	0.272754556
ENSG00000138942.11	RNF185	turquoise	0.918747005
ENSG00000168159.9	RNF187	turquoise	0.959565986
ENSG00000034677.7	RNF19A	blue	0.879944181
ENSG00000116514.12	RNF19B	turquoise	0.889416854
ENSG00000121481.6	RNF2	turquoise	0.919652532
ENSG00000155827.7	RNF20	blue	0.917303971
ENSG00000158286.8	RNF207	turquoise	0.83897925
ENSG00000212864.2	RNF208	turquoise	0.80348421
ENSG00000178222.8	RNF212	turquoise	0.829651046
ENSG00000173821.15	RNF213	blue	0.866657453
ENSG00000167257.6	RNF214	turquoise	0.914226082
ENSG00000099999.10	RNF215	turquoise	0.876404149
ENSG00000011275.14	RNF216	turquoise	0.958524848
ENSG00000196204.7	RNF216P1	turquoise	0.853038409
ENSG00000146373.12	RNF217	turquoise	0.855265183

ENSG00000152193.7	RNF219	turquoise	0.961160459
ENSG00000234377.3	RNF219-AS1	green	0.785639545
ENSG00000187147.13	RNF220	brown	0.792939732
ENSG00000233198.2	RNF224	pink	0.833698746
ENSG00000101236.12	RNF24	turquoise	0.897623299
ENSG00000163481.3	RNF25	turquoise	0.934274664
ENSG00000173456.4	RNF26	turquoise	0.943421645
ENSG00000092098.12	RNF31	turquoise	0.895700055
ENSG00000105982.12	RNF32	pink	0.809815747
ENSG00000170633.12	RNF34	turquoise	0.982370655
ENSG00000137075.13	RNF38	turquoise	0.956302974
ENSG00000204618.4	RNF39	turquoise	0.849962671
ENSG00000063978.11	RNF4	turquoise	0.941348242
ENSG00000103549.17	RNF40	turquoise	0.93148389
ENSG00000181852.13	RNF41	turquoise	0.970491235
ENSG00000108375.8	RNF43	grey	0.31888842
ENSG00000146083.7	RNF44	turquoise	0.957181141
ENSG00000204308.6	RNF5	turquoise	0.929381527
ENSG00000253570.1	RNF5P1	grey	0.27532189
ENSG00000127870.12	RNF6	turquoise	0.942663281
ENSG00000114125.9	RNF7	turquoise	0.92582257
ENSG00000112130.12	RNF8	turquoise	0.955954401
ENSG00000189050.10	RNFT1	turquoise	0.889914096
ENSG00000135119.10	RNFT2	turquoise	0.919337539
ENSG00000111880.11	RNGTT	turquoise	0.934343675
ENSG00000023191.12	RNH1	turquoise	0.83746182
ENSG00000184719.7	RNLS	turquoise	0.888244687
ENSG00000101654.13	RNMT	turquoise	0.981504492
ENSG00000171861.6	RNMTL1	turquoise	0.937593679
ENSG00000185946.11	RNPC3	turquoise	0.899947893
ENSG00000176393.6	RNPEP	turquoise	0.881538603
ENSG00000142327.7	RNPEPL1	blue	0.772897904
ENSG00000205937.7	RNPS1	turquoise	0.968176586
ENSG00000202408.1	RNU1-122P	grey	0.316986147
ENSG00000202538.1	RNU4-2	grey	0.116053941
ENSG00000264229.1	RNU4ATAC	grey	0.207802468
ENSG00000207357.1	RNU6-2	grey	0.293645019
ENSG00000207291.1	RNU6-30P	turquoise	0.660377257
ENSG00000201499.1	RNU6-312P	turquoise	0.586491058
ENSG00000201136.1	RNU6-353P	turquoise	0.524239732
ENSG00000223313.1	RNU6-516P	turquoise	0.409183673
ENSG00000200253.1	RNU6-529P	grey	0.195541761
ENSG00000202337.1	RNU6-8	grey	0.224697964
ENSG00000221571.2	RNU6ATAC35P	grey	0.21201377
ENSG00000201098.1	RNY1	grey	0.06590567
ENSG00000223298.1	RNY3P8	grey	0.275725165
ENSG00000169855.15	ROBO1	turquoise	0.917142317
ENSG00000185008.13	ROBO2	turquoise	0.871398926

ENSG00000154134.10	ROBO3	green	0.608615252
ENSG00000154133.10	ROBO4	black	0.666112152
ENSG00000067900.6	ROCK1	turquoise	0.752967096
ENSG00000263006.2	ROCK1P1	turquoise	0.659522515
ENSG00000134318.9	ROCK2	turquoise	0.939037922
ENSG00000067836.8	ROGDI	turquoise	0.864454449
ENSG00000149489.4	ROM1	black	0.656605324
ENSG00000125995.11	ROMO1	red	0.931012546
ENSG00000114547.5	ROPN1B	green	0.529260545
ENSG00000069667.11	RORA	turquoise	0.842214853
ENSG00000198963.6	RORB	green	0.836368711
ENSG00000224849.2	RP1-104O17.1	turquoise	0.689423337
ENSG00000271913.1	RP1-111C20.4	turquoise	0.8192895
ENSG00000233330.1	RP1-12G14.7	turquoise	0.604784921
ENSG00000241528.1	RP1-130H16.16	turquoise	0.728972317
ENSG00000272848.1	RP1-137D17.2	turquoise	0.571254948
ENSG00000231050.1	RP1-140A9.1	turquoise	0.723402027
ENSG00000229931.1	RP1-151F17.1	turquoise	0.853739674
ENSG00000272341.1	RP1-151F17.2	turquoise	0.911037909
ENSG00000271755.1	RP1-153G14.4	turquoise	0.737726667
ENSG00000235912.1	RP1-159A19.3	grey	0.296615376
ENSG00000235357.1	RP1-159G19.1	turquoise	0.725512879
ENSG00000266911.1	RP1-161P9.5	turquoise	0.651414315
ENSG00000261420.1	RP1-168L15.5	turquoise	0.782885543
ENSG00000246982.2	RP1-179N16.6	yellow	0.807426991
ENSG00000236173.1	RP1-182D15.2	turquoise	0.646715642
ENSG00000267194.1	RP1-193H18.2	turquoise	0.818207926
ENSG00000257433.1	RP1-197B17.3	brown	0.615186745
ENSG00000226648.1	RP1-1J6.2	green	0.573897507
ENSG00000272223.1	RP1-20C7.6	turquoise	0.595698739
ENSG00000226852.2	RP1-212P9.2	turquoise	0.692797264
ENSG00000228793.1	RP1-223B1.1	brown	0.734921129
ENSG00000261071.1	RP1-223E5.4	turquoise	0.799732864
ENSG00000260920.1	RP1-228H13.5	turquoise	0.92254205
ENSG00000240399.1	RP1-228P16.1	yellow	0.519069997
ENSG00000231533.1	RP1-232L24.3	brown	0.691526048
ENSG00000260196.1	RP1-239B22.5	turquoise	0.753709357
ENSG00000271857.1	RP1-244F24.1	turquoise	0.636370878
ENSG00000227502.2	RP1-249H1.4	magenta	0.544997445
ENSG00000232311.1	RP1-249I4.2	turquoise	0.708976386
ENSG00000272379.1	RP1-257A7.5	turquoise	0.766414511
ENSG00000270083.1	RP1-257I20.14	turquoise	0.688016052
ENSG00000259146.2	RP1-261D10.2	pink	0.773414538
ENSG00000219392.1	RP1-265C24.5	brown	0.58988162
ENSG00000232640.1	RP1-266L20.2	turquoise	0.62908756
ENSG00000233508.1	RP1-269M15.3	turquoise	0.860547628
ENSG00000227775.3	RP1-283E3.4	turquoise	0.820281823
ENSG00000272153.1	RP1-286D6.5	yellow	0.810211263

ENSG00000234688.1	RP1-293L6.1	turquoise	0.771848909
ENSG00000272345.1	RP1-30M3.5	turquoise	0.606940993
ENSG00000272402.1	RP1-30M3.6	blue	0.59067725
ENSG00000272009.1	RP1-313I6.12	grey	0.434166953
ENSG00000232412.1	RP1-315G1.3	red	0.551806155
ENSG00000259943.1	RP1-39G22.7	turquoise	0.935835441
ENSG00000272277.1	RP1-40E16.12	turquoise	0.564814386
ENSG00000230269.2	RP1-40E16.9	turquoise	0.859637722
ENSG00000230424.1	RP1-43E13.2	grey	0.284483891
ENSG00000258240.1	RP1-46F2.3	turquoise	0.784960843
ENSG00000263345.1	RP1-59D14.5	yellow	0.916795113
ENSG00000243902.2	RP1-63G5.5	turquoise	0.776008938
ENSG00000231769.2	RP1-8B1.4	turquoise	0.803608105
ENSG00000261015.1	RP1-90J20.11	yellow	0.866584492
ENSG00000270346.1	RP1-90J20.12	turquoise	0.85405258
ENSG00000229431.1	RP1-92O14.6	turquoise	0.765264704
ENSG00000272403.1	RP1-93H18.7	grey	0.272567287
ENSG00000217648.1	RP1-95L4.4	turquoise	0.657094138
ENSG00000260534.1	RP11-1006G14.4	turquoise	0.931982554
ENSG00000261187.1	RP11-1007O24.2	turquoise	0.727410747
ENSG00000259168.1	RP11-100M12.3	turquoise	0.700424103
ENSG00000271780.1	RP11-1017G21.5	turquoise	0.829170763
ENSG00000272444.1	RP11-1017G21.6	turquoise	0.657868752
ENSG00000272579.1	RP11-101E13.5	blue	0.652078338
ENSG00000269894.1	RP11-1020A11.1	yellow	0.675078131
ENSG00000269982.1	RP11-1020A11.2	turquoise	0.899237939
ENSG00000249109.1	RP11-1026M7.2	turquoise	0.619039751
ENSG00000258919.1	RP11-1029J19.4	turquoise	0.820761762
ENSG00000258404.1	RP11-1029J19.5	turquoise	0.721225749
ENSG00000244668.1	RP11-103G8.1	turquoise	0.777849834
ENSG00000240137.1	RP11-103G8.2	turquoise	0.792447941
ENSG00000230317.1	RP11-104D21.3	turquoise	0.758734394
ENSG00000229273.1	RP11-104G3.2	turquoise	0.696652778
ENSG00000262223.2	RP11-1055B8.3	brown	0.850957344
ENSG00000262877.3	RP11-1055B8.4	brown	0.644285054
ENSG00000263053.3	RP11-1055B8.6	turquoise	0.877369085
ENSG00000171282.9	RP11-1055B8.7	turquoise	0.915956106
ENSG00000270659.1	RP11-105N14.1	turquoise	0.666133812
ENSG00000228484.1	RP11-106M7.1	green	0.578425156
ENSG00000272457.1	RP11-1070A24.2	turquoise	0.470071827
ENSG00000248714.2	RP11-1079K10.3	turquoise	0.708255932
ENSG00000225208.1	RP11-107I14.2	turquoise	0.727903393
ENSG00000254966.1	RP11-1081L13.4	turquoise	0.835970494
ENSG00000273476.1	RP11-108L7.14	turquoise	0.41649868
ENSG00000238142.1	RP11-108M9.4	grey	0.388427349
ENSG00000235241.1	RP11-108M9.5	grey	0.461336438
ENSG00000267010.1	RP11-108P20.1	turquoise	0.867071518
ENSG00000267321.1	RP11-1094M14.11	turquoise	0.919593611

ENSG00000267369.1	RP11-1094M14.8	grey	0.216413118
ENSG00000261447.1	RP11-109D9.4	turquoise	0.66604539
ENSG00000258813.1	RP11-109N23.4	turquoise	0.700670074
ENSG00000230916.2	RP11-10B2.1	grey	-0.314809284
ENSG00000245213.2	RP11-10K16.1	blue	0.704362154
ENSG00000246560.2	RP11-10L12.4	blue	0.658241859
ENSG00000246375.2	RP11-10L7.1	grey	0.153981791
ENSG00000253854.1	RP11-10N23.2	turquoise	0.82796241
ENSG00000259884.1	RP11-1100L3.8	grey	0.297315242
ENSG00000228801.5	RP11-110G21.1	turquoise	0.846700694
ENSG00000254428.1	RP11-110I1.11	turquoise	0.685803721
ENSG00000272186.1	RP11-110I1.13	turquoise	0.582149168
ENSG00000254909.1	RP11-110I1.5	turquoise	0.667881791
ENSG00000232611.1	RP11-1114A5.4	turquoise	0.883552073
ENSG00000225400.1	RP11-1114A5.5	turquoise	0.729264926
ENSG00000231995.2	RP11-111F5.2	turquoise	0.739458623
ENSG00000260331.1	RP11-111J6.2	turquoise	0.694220161
ENSG00000261019.1	RP11-111K18.2	turquoise	0.784296193
ENSG00000255135.3	RP11-111M22.3	turquoise	0.810333745
ENSG00000272301.1	RP11-111M22.4	turquoise	0.749665729
ENSG00000271757.1	RP11-111M22.5	turquoise	0.692172023
ENSG00000227388.2	RP11-112J3.16	turquoise	0.759507561
ENSG00000231742.1	RP11-112L6.4	grey	0.421391167
ENSG00000254348.1	RP11-1134I14.4	turquoise	0.862561799
ENSG00000255366.1	RP11-1134I14.8	turquoise	0.939808808
ENSG00000256552.2	RP11-113C12.4	turquoise	0.646692451
ENSG00000228343.1	RP11-1148L6.5	yellow	0.811991322
ENSG00000273488.1	RP11-114I8.4	turquoise	0.85021751
ENSG00000271320.1	RP11-1152H14.1	turquoise	0.413011521
ENSG00000246089.3	RP11-115C21.2	turquoise	0.884057463
ENSG00000273125.1	RP11-115H18.1	turquoise	0.817625064
ENSG00000267671.1	RP11-115K3.2	turquoise	0.863985458
ENSG00000267088.1	RP11-115N12.1	turquoise	0.734961187
ENSG00000255038.1	RP11-1167A19.2	yellow	0.768238962
ENSG00000259174.1	RP11-1180F24.1	grey	0.619773761
ENSG00000256427.1	RP11-118B22.4	turquoise	0.540639039
ENSG00000273001.1	RP11-118K6.3	grey	0.529689159
ENSG00000242686.2	RP11-1191J2.2	yellow	0.848838955
ENSG00000272927.1	RP11-1191J2.5	yellow	0.836837938
ENSG00000229539.1	RP11-119B16.2	turquoise	0.610903169
ENSG00000226381.3	RP11-119F19.2	turquoise	0.859403718
ENSG00000260400.1	RP11-119F7.5	turquoise	0.490121132
ENSG00000247134.2	RP11-11N9.4	blue	0.631603916
ENSG00000225472.1	RP11-120J1.1	green	0.674525133
ENSG00000267868.1	RP11-120K24.3	turquoise	0.806491151
ENSG00000259684.1	RP11-120K9.2	turquoise	0.717382569
ENSG00000267009.2	RP11-120M18.2	turquoise	0.730994296
ENSG00000205746.5	RP11-1212A22.1	turquoise	0.753140593

ENSG00000259959.1	RP11-121C2.2	turquoise	0.91209577
ENSG00000253250.2	RP11-122A3.2	turquoise	0.679679264
ENSG00000235477.2	RP11-122G18.5	turquoise	0.92409017
ENSG00000235245.1	RP11-122K13.12	yellow	0.844052926
ENSG00000270235.1	RP11-122K13.14	turquoise	0.710742972
ENSG00000273230.1	RP11-1246C19.1	turquoise	0.849818221
ENSG00000250012.1	RP11-124N2.1	turquoise	0.812534865
ENSG00000236404.4	RP11-125B21.2	turquoise	0.791689281
ENSG00000273238.1	RP11-1263C18.1	turquoise	0.825952498
ENSG00000237976.1	RP11-126K1.6	grey	0.264757812
ENSG00000234432.3	RP11-1275H24.1	turquoise	0.82620677
ENSG00000272953.1	RP11-1275H24.2	grey	0.67644575
ENSG00000273084.1	RP11-1275H24.3	turquoise	0.75628994
ENSG00000247679.2	RP11-1277A3.1	turquoise	0.877501553
ENSG00000246596.2	RP11-1277A3.2	turquoise	0.765577084
ENSG00000272459.1	RP11-1277A3.3	turquoise	0.654506354
ENSG00000273156.1	RP11-127B20.2	turquoise	0.745824445
ENSG00000272677.1	RP11-127B20.3	turquoise	0.677919285
ENSG00000267077.1	RP11-127I20.5	brown	0.571635675
ENSG00000228261.1	RP11-127L20.3	turquoise	0.64987952
ENSG00000226644.1	RP11-128M1.1	pink	0.79225626
ENSG00000271868.1	RP11-1293J14.1	grey	0.598443806
ENSG00000260641.1	RP11-1299A16.3	brown	0.754051638
ENSG00000238099.1	RP11-12A2.3	green	0.569642183
ENSG00000271143.1	RP11-12L8.2	turquoise	0.554092556
ENSG00000269976.1	RP11-130L8.2	grey	0.497646164
ENSG00000273264.1	RP11-131L23.2	grey	0.065089288
ENSG00000232445.1	RP11-132A1.4	turquoise	0.752378366
ENSG00000248996.1	RP11-1334A24.6	turquoise	0.669135042
ENSG00000224536.1	RP11-134G8.7	brown	0.567760188
ENSG00000224818.1	RP11-134G8.8	red	0.624611565
ENSG00000271967.1	RP11-134K13.4	turquoise	0.753166731
ENSG00000272941.1	RP11-134L10.1	brown	0.692508135
ENSG00000231507.1	RP11-134P9.3	turquoise	0.69886785
ENSG00000228063.1	RP11-135J2.4	blue	0.637023032
ENSG00000265678.1	RP11-1376P16.2	turquoise	0.786660401
ENSG00000271738.1	RP11-137H2.6	turquoise	0.84858323
ENSG00000221817.5	RP11-137L10.6	blue	0.79441271
ENSG00000270755.1	RP11-138E2.1	turquoise	0.505429722
ENSG00000228704.1	RP11-138M12.1	midnightblue	0.719657898
ENSG00000250397.2	RP11-1391J7.1	grey	0.727433704
ENSG00000225973.3	RP11-139H15.1	turquoise	0.865863536
ENSG00000269707.1	RP11-13J10.1	turquoise	0.813421403
ENSG00000267466.1	RP11-13K12.5	turquoise	0.618229624
ENSG00000263350.1	RP11-13N13.2	turquoise	0.470668536
ENSG00000269908.1	RP11-1406H17.1	turquoise	0.622493428
ENSG00000174093.6	RP11-1407O15.2	turquoise	0.873308216
ENSG00000272343.1	RP11-140I16.3	turquoise	0.660516428

ENSG00000272288.1	RP11-140K17.3	brown	0.515818238
ENSG00000253816.2	RP11-1415C14.3	brown	0.757818626
ENSG00000261113.1	RP11-141O15.1	turquoise	0.445245742
ENSG00000266053.2	RP11-143J12.2	turquoise	0.892670705
ENSG00000260248.1	RP11-143K11.1	turquoise	0.753212289
ENSG00000269728.1	RP11-145M9.4	magenta	0.652586576
ENSG00000261840.1	RP11-146F11.1	red	0.600056949
ENSG00000241170.2	RP11-147I3.1	grey	0.540933018
ENSG00000265100.1	RP11-147L13.2	blue	0.483399954
ENSG00000267731.1	RP11-147L13.8	turquoise	0.748569729
ENSG00000242428.1	RP11-147N17.1	turquoise	0.800360634
ENSG00000244151.1	RP11-148K1.12	yellow	0.773745671
ENSG00000232110.3	RP11-149I23.3	brown	0.570876652
ENSG00000232527.3	RP11-14N7.2	turquoise	0.847921213
ENSG00000272599.1	RP11-152N13.16	turquoise	0.715987972
ENSG00000227540.1	RP11-152N13.5	pink	0.841787612
ENSG00000270562.1	RP11-154H23.3	turquoise	0.765632794
ENSG00000272601.1	RP11-155G14.5	black	0.675187606
ENSG00000272100.1	RP11-155O18.6	turquoise	0.559621142
ENSG00000259623.1	RP11-156E6.1	turquoise	0.949987183
ENSG00000272195.1	RP11-156E8.1	turquoise	0.642406525
ENSG00000253944.1	RP11-156K13.1	grey	0.263192772
ENSG00000262879.1	RP11-156P1.3	turquoise	0.839255053
ENSG00000272279.1	RP11-157J24.2	grey	0.216124102
ENSG00000226332.2	RP11-157P1.4	yellow	0.614660386
ENSG00000253106.1	RP11-158K1.3	yellow	0.891414297
ENSG00000259407.1	RP11-158M2.3	turquoise	0.679026342
ENSG00000259762.1	RP11-158M2.4	turquoise	0.609674769
ENSG00000259416.2	RP11-158M2.5	turquoise	0.617160912
ENSG00000264112.1	RP11-159D12.2	yellow	0.918109271
ENSG00000256757.1	RP11-159N11.3	turquoise	0.721139748
ENSG00000270179.1	RP11-159N11.4	turquoise	0.626450272
ENSG00000267058.1	RP11-15A1.3	turquoise	0.916922607
ENSG00000269837.1	RP11-15H20.5	turquoise	0.87947202
ENSG00000213971.3	RP11-15H20.6	turquoise	0.878347385
ENSG00000204778.4	RP11-15J10.1	turquoise	0.496749365
ENSG00000261533.1	RP11-15N24.4	turquoise	0.898343851
ENSG00000260807.2	RP11-161M6.2	grey	0.782060437
ENSG00000264278.1	RP11-162A12.2	turquoise	0.748971583
ENSG00000273107.1	RP11-165A20.3	grey	0.446993078
ENSG00000227603.1	RP11-165J3.6	turquoise	0.831279611
ENSG00000234719.4	RP11-166B2.1	grey	0.270213458
ENSG00000255248.2	RP11-166D19.1	cyan	0.850536588
ENSG00000227253.2	RP11-166N17.1	yellow	0.697978199
ENSG00000235475.1	RP11-166O4.5	turquoise	0.698575568
ENSG00000273448.1	RP11-166O4.6	yellow	0.603964003
ENSG00000263004.1	RP11-166P13.3	turquoise	0.642151374
ENSG00000267834.1	RP11-167N5.5	grey	0.808539445

ENSG00000251050.1	RP11-168A11.4	grey	0.521686205
ENSG00000249599.1	RP11-168E14.1	grey	0.342892689
ENSG00000255087.2	RP11-168K9.2	turquoise	0.741796163
ENSG00000227467.3	RP11-169D4.1	turquoise	0.703028338
ENSG00000233954.4	RP11-169K16.7	turquoise	0.842749944
ENSG00000179743.2	RP11-169K16.9	brown	0.748161267
ENSG00000259448.2	RP11-16E12.1	turquoise	0.854824865
ENSG00000261542.1	RP11-16E18.3	turquoise	0.825868348
ENSG00000260572.1	RP11-16N11.2	turquoise	0.820324836
ENSG00000261428.1	RP11-16P6.1	turquoise	0.801973232
ENSG00000273007.1	RP11-170N16.3	yellow	0.808484651
ENSG00000228444.2	RP11-173B14.4	turquoise	0.600803715
ENSG00000265683.1	RP11-173M1.5	turquoise	0.770370559
ENSG00000264469.1	RP11-173M1.8	yellow	0.709038297
ENSG00000250327.1	RP11-173M11.2	turquoise	0.731180514
ENSG00000231025.1	RP11-175O19.4	turquoise	0.91059476
ENSG00000203546.3	RP11-176H8.1	turquoise	0.632466792
ENSG00000272137.1	RP11-177G23.2	grey	0.466418955
ENSG00000273257.1	RP11-177J6.1	turquoise	0.608656169
ENSG00000267957.1	RP11-178G16.4	turquoise	0.828911566
ENSG00000269772.1	RP11-178G16.5	turquoise	0.890337871
ENSG00000270006.1	RP11-178L8.7	grey	0.437746716
ENSG00000254254.1	RP11-17A4.2	turquoise	0.686376069
ENSG00000272864.1	RP11-17E13.2	grey	0.409987595
ENSG00000259234.1	RP11-17L5.4	turquoise	0.799833785
ENSG00000265778.1	RP11-17M16.2	turquoise	0.792628727
ENSG00000272511.1	RP11-180N14.1	turquoise	0.754456301
ENSG00000263931.1	RP11-180P8.1	turquoise	0.771171945
ENSG00000254370.1	RP11-181B11.1	turquoise	0.67659054
ENSG00000182873.4	RP11-181G12.2	yellow	0.920752034
ENSG00000254414.1	RP11-182J1.1	turquoise	0.48301293
ENSG00000259683.1	RP11-182J1.14	turquoise	0.749509072
ENSG00000272447.1	RP11-182L21.6	turquoise	0.76348435
ENSG00000240497.2	RP11-185E8.1	turquoise	0.39295998
ENSG00000228302.2	RP11-186N15.3	yellow	0.879586993
ENSG00000271427.1	RP11-188D8.1	turquoise	0.703076572
ENSG00000272646.1	RP11-188P17.2	turquoise	0.446290798
ENSG00000271941.1	RP11-188P20.3	grey	0.579945082
ENSG00000269609.1	RP11-18I14.10	turquoise	0.878583195
ENSG00000272668.1	RP11-190A12.8	grey	0.517929297
ENSG00000272663.1	RP11-191L17.1	turquoise	0.738024357
ENSG00000258472.4	RP11-192H23.4	turquoise	0.856055475
ENSG00000264608.1	RP11-192H23.8	turquoise	0.750806436
ENSG00000272148.1	RP11-195B17.1	turquoise	0.819747612
ENSG00000230847.4	RP11-195E2.1	grey	0.255541716
ENSG00000254353.1	RP11-195E2.4	grey	0.221760505
ENSG00000187186.10	RP11-195F19.5	turquoise	0.69861141
ENSG00000230074.1	RP11-195F19.9	turquoise	0.684528407

ENSG00000254561.2	RP11-196E1.3	turquoise	0.742994677
ENSG00000260911.1	RP11-196G11.2	brown	0.735552617
ENSG00000262766.1	RP11-196G11.4	blue	0.741518814
ENSG00000261716.1	RP11-196G18.22	turquoise	0.731861074
ENSG00000273477.1	RP11-196O16.1	turquoise	0.817324686
ENSG00000223812.1	RP11-197K6.1	turquoise	0.618734015
ENSG00000262251.1	RP11-199F11.2	blue	0.622483935
ENSG00000253477.1	RP11-1C8.4	turquoise	0.849437406
ENSG00000271830.1	RP11-1C8.7	turquoise	0.748282937
ENSG00000227218.3	RP11-203J24.8	turquoise	0.635751317
ENSG00000258515.1	RP11-203M5.7	yellow	0.839948518
ENSG00000258908.1	RP11-203M5.8	black	0.519637757
ENSG00000231754.1	RP11-204E9.1	turquoise	0.822319863
ENSG00000204837.2	RP11-204M4.2	turquoise	0.776385285
ENSG00000224956.5	RP11-206L10.1	yellow	0.731647118
ENSG00000228794.4	RP11-206L10.11	turquoise	0.95616756
ENSG00000228327.2	RP11-206L10.2	turquoise	0.671626981
ENSG00000204894.4	RP11-208G20.2	grey	0.210438759
ENSG00000270405.1	RP11-208G20.3	grey	0.222064163
ENSG00000261033.1	RP11-209D14.2	turquoise	0.671056418
ENSG00000257526.1	RP11-20E24.1	grey	0.232786014
ENSG00000273179.1	RP11-20I20.4	grey	0.536863701
ENSG00000229116.1	RP11-20J15.3	turquoise	0.718908149
ENSG00000258010.2	RP11-210M15.1	turquoise	-0.396232135
ENSG00000259495.1	RP11-210M15.2	turquoise	0.783006096
ENSG00000223401.1	RP11-211G3.2	black	0.755934147
ENSG00000273270.1	RP11-212P7.2	turquoise	0.800147382
ENSG00000165121.10	RP11-213G2.3	blue	0.752280315
ENSG00000271855.1	RP11-214N9.1	turquoise	0.71913993
ENSG00000272016.1	RP11-215G15.5	turquoise	0.837785386
ENSG00000261340.1	RP11-215H22.1	turquoise	0.860046422
ENSG00000228395.1	RP11-216B9.6	turquoise	0.751201575
ENSG00000237846.1	RP11-216M21.1	turquoise	0.498042117
ENSG00000271769.1	RP11-216P16.2	turquoise	0.637656972
ENSG00000270001.1	RP11-218C14.8	grey	0.093722983
ENSG00000273449.1	RP11-218F10.3	yellow	0.575748553
ENSG00000177406.4	RP11-218M22.1	turquoise	0.800873279
ENSG00000273097.1	RP11-219C20.3	turquoise	0.435715713
ENSG00000256481.1	RP11-21A7A.4	red	0.579395734
ENSG00000261744.1	RP11-21B21.4	turquoise	0.686769694
ENSG00000233137.2	RP11-220I1.1	turquoise	0.949517511
ENSG00000261829.1	RP11-223I10.1	turquoise	0.421417051
ENSG00000273014.1	RP11-225B17.2	turquoise	0.721884805
ENSG00000260766.1	RP11-226L15.5	turquoise	0.835235181
ENSG00000265139.1	RP11-227G15.2	turquoise	0.569328242
ENSG00000225032.1	RP11-228B15.4	yellow	0.863363838
ENSG00000235117.2	RP11-229P13.20	turquoise	0.793648492
ENSG00000111788.9	RP11-22B23.1	grey	0.422964121

ENSG00000261766.1	RP11-22P6.2	turquoise	0.732888138
ENSG00000260442.1	RP11-22P6.3	red	0.459130304
ENSG00000235545.1	RP11-230B22.1	turquoise	0.6904315
ENSG00000271551.1	RP11-230C9.2	turquoise	0.840171311
ENSG00000271265.1	RP11-230C9.4	turquoise	0.734453037
ENSG00000169203.12	RP11-231C14.4	turquoise	0.690079161
ENSG00000248184.1	RP11-231C18.1	grey	0.179028283
ENSG00000272308.1	RP11-231G3.1	turquoise	0.524645503
ENSG00000257279.1	RP11-231I16.1	midnightblue	0.803564027
ENSG00000232406.2	RP11-234K24.3	turquoise	0.834687384
ENSG00000262903.1	RP11-235E17.6	blue	0.709867992
ENSG00000273124.1	RP11-236B18.5	grey	0.265389797
ENSG00000254921.1	RP11-236J17.6	turquoise	0.773297562
ENSG00000271239.1	RP11-238F2.1	grey	0.06510294
ENSG00000272003.1	RP11-23P13.7	grey	0.31611387
ENSG00000267002.1	RP11-242D8.1	turquoise	0.943547175
ENSG00000267340.1	RP11-242D8.3	turquoise	0.688850555
ENSG00000271948.1	RP11-242F4.2	turquoise	0.886283859
ENSG00000261617.1	RP11-243A14.1	brown	0.690441211
ENSG00000241014.1	RP11-244H3.1	turquoise	0.727789125
ENSG00000261534.1	RP11-244O19.1	midnightblue	0.945009615
ENSG00000271952.1	RP11-245G13.2	turquoise	0.737502233
ENSG00000238276.1	RP11-245J24.1	grey	0.144494262
ENSG00000271843.1	RP11-245J9.5	turquoise	0.71122033
ENSG00000204055.4	RP11-247A12.2	turquoise	0.804943183
ENSG00000268707.1	RP11-247A12.7	grey	0.386409924
ENSG00000248540.2	RP11-247C2.2	turquoise	0.751863706
ENSG00000259071.1	RP11-247L20.4	turquoise	0.651115895
ENSG00000269906.1	RP11-248J18.2	brown	0.495518299
ENSG00000270101.1	RP11-24B19.3	turquoise	0.861670764
ENSG00000269965.1	RP11-24B19.4	turquoise	0.664905672
ENSG00000233967.2	RP11-250B2.3	yellow	0.734255293
ENSG00000260645.1	RP11-250B2.5	yellow	0.749570796
ENSG00000272129.1	RP11-250B2.6	turquoise	0.868124625
ENSG00000272604.1	RP11-251G23.5	turquoise	0.897672696
ENSG00000214331.4	RP11-252A24.2	turquoise	0.850061135
ENSG00000260539.1	RP11-252A24.7	turquoise	0.868200021
ENSG00000260077.1	RP11-254F7.2	yellow	0.7222445
ENSG00000273190.1	RP11-255C15.4	turquoise	0.545541284
ENSG00000268896.1	RP11-256I23.1	turquoise	0.640037697
ENSG00000267919.1	RP11-256I23.3	turquoise	0.712466785
ENSG00000270189.1	RP11-258C19.7	yellow	0.900742912
ENSG00000226578.1	RP11-258F22.1	turquoise	0.691949657
ENSG00000262539.1	RP11-259G18.3	grey	0.221193708
ENSG00000258844.1	RP11-259K15.2	pink	0.610070936
ENSG00000271653.1	RP11-259K5.1	grey	0.369270425
ENSG00000167912.5	RP11-25K19.1	blue	0.521212849
ENSG00000272807.1	RP11-260M2.1	turquoise	0.805075033

ENSG00000238113.2	RP11-262H14.1	pink	0.754740673
ENSG00000234665.4	RP11-262H14.3	grey	0.262775537
ENSG00000170161.6	RP11-262H14.4	brown	0.693348168
ENSG00000229422.1	RP11-262H14.5	turquoise	0.798457479
ENSG00000227582.2	RP11-262H14.7	grey	0.297702213
ENSG00000224222.1	RP11-262I2.2	turquoise	0.857091866
ENSG00000236263.1	RP11-263K19.6	yellow	0.818827509
ENSG00000267207.1	RP11-264B14.1	turquoise	0.477286855
ENSG00000260853.1	RP11-264B17.2	yellow	0.834983902
ENSG00000260367.1	RP11-264B17.4	turquoise	0.646001934
ENSG00000256164.1	RP11-264F23.3	grey	0.664362177
ENSG00000255920.1	RP11-264F23.4	turquoise	0.783904351
ENSG00000236199.1	RP11-264I13.2	blue	0.547597611
ENSG00000254680.1	RP11-265D17.2	yellow	0.779446863
ENSG00000272905.1	RP11-265E18.1	turquoise	0.481264517
ENSG00000269886.1	RP11-266J6.2	grey	0.408494833
ENSG00000244050.2	RP11-266K4.1	turquoise	0.717884221
ENSG00000215241.3	RP11-266K4.9	turquoise	0.869652378
ENSG00000262587.1	RP11-266L9.4	turquoise	0.829445696
ENSG00000263326.1	RP11-266L9.6	turquoise	0.692924539
ENSG00000249306.1	RP11-267A15.1	brown	0.626335121
ENSG00000227115.2	RP11-267C16.1	brown	0.791029896
ENSG00000116883.8	RP11-268J15.5	turquoise	0.857885771
ENSG00000250166.2	RP11-268P4.5	green	0.512458331
ENSG00000265971.1	RP11-269G24.6	grey	0.579001295
ENSG00000270111.1	RP11-271F18.4	turquoise	0.81440597
ENSG00000256967.1	RP11-273B20.1	turquoise	0.743053938
ENSG00000247317.3	RP11-273G15.2	turquoise	0.853355228
ENSG00000242588.2	RP11-274B21.1	blue	0.808809059
ENSG00000271553.1	RP11-274B21.10	grey	0.683199971
ENSG00000243302.3	RP11-274B21.2	yellow	0.868468814
ENSG00000243679.1	RP11-274B21.3	yellow	0.815767064
ENSG00000230715.2	RP11-274B21.4	yellow	0.856946253
ENSG00000270810.1	RP11-274B21.8	yellow	0.626495169
ENSG00000241231.1	RP11-275H4.1	grey	0.2481082
ENSG00000272205.1	RP11-277B15.3	grey	0.590797376
ENSG00000257511.1	RP11-278C7.1	yellow	0.857141351
ENSG00000226828.1	RP11-278H7.1	turquoise	0.826333023
ENSG00000245750.3	RP11-279F6.1	turquoise	0.842953733
ENSG00000249679.1	RP11-279O9.4	turquoise	0.674491612
ENSG00000272138.1	RP11-27N21.3	turquoise	0.721589179
ENSG00000254035.1	RP11-281O15.4	turquoise	0.759294458
ENSG00000235423.4	RP11-282O18.3	blue	0.719991831
ENSG00000256894.1	RP11-283G6.3	turquoise	0.724519321
ENSG00000255671.1	RP11-283I3.1	grey	0.391999415
ENSG00000256577.1	RP11-283I3.2	brown	0.657816295
ENSG00000261799.1	RP11-283I3.6	turquoise	0.915259535
ENSG00000272405.1	RP11-284F21.10	brown	0.558899354

ENSG00000272971.1	RP11-284F21.11	turquoise	0.573393597
ENSG00000229953.1	RP11-284F21.7	brown	0.515833005
ENSG00000259834.1	RP11-284N8.3	turquoise	0.833503085
ENSG00000273030.1	RP11-285F16.1	brown	0.481152891
ENSG00000242861.1	RP11-285F7.2	brown	0.829535777
ENSG00000271993.1	RP11-285J16.1	brown	0.563223728
ENSG00000272789.1	RP11-286H15.1	grey	0.381577102
ENSG00000255931.1	RP11-286N22.10	turquoise	0.790043874
ENSG00000234531.2	RP11-288G11.3	brown	0.669768212
ENSG00000223612.2	RP11-289I10.2	blue	0.666619296
ENSG00000273149.1	RP11-290D2.6	cyan	0.763859939
ENSG00000267940.1	RP11-290F24.6	turquoise	0.818935901
ENSG00000257941.1	RP11-290L1.4	turquoise	0.676875625
ENSG00000240005.1	RP11-293A21.1	red	0.501944255
ENSG00000259319.1	RP11-293M10.6	turquoise	0.831818356
ENSG00000262712.1	RP11-295D4.1	turquoise	0.64907134
ENSG00000233461.1	RP11-295G20.2	black	0.686010793
ENSG00000239665.4	RP11-295P9.3	turquoise	0.923301119
ENSG00000247228.2	RP11-296I10.3	turquoise	0.763173288
ENSG00000261556.4	RP11-296I10.6	yellow	0.860337343
ENSG00000270049.1	RP11-297D21.4	turquoise	0.772684729
ENSG00000260432.1	RP11-297M9.2	turquoise	0.787127867
ENSG00000260788.1	RP11-298D21.1	brown	0.736645978
ENSG00000257285.1	RP11-298I3.1	blue	0.653046152
ENSG00000269387.1	RP11-298J23.8	grey	0.40107732
ENSG00000259172.1	RP11-299G20.2	brown	0.756425449
ENSG00000272758.1	RP11-299J3.8	red	0.62882808
ENSG00000269942.1	RP11-29B2.5	yellow	0.738715096
ENSG00000269946.1	RP11-2B6.3	grey	0.342866167
ENSG00000259920.1	RP11-2E11.5	green	0.586729903
ENSG00000270953.1	RP11-2E11.9	turquoise	0.785379739
ENSG00000259417.2	RP11-2E17.1	turquoise	0.576224352
ENSG00000272885.1	RP11-2H3.6	turquoise	0.558953062
ENSG00000272479.1	RP11-301G7.1	turquoise	0.716615747
ENSG00000273080.1	RP11-301O19.1	turquoise	0.578045552
ENSG00000239617.1	RP11-302B13.1	turquoise	0.869365519
ENSG00000273100.1	RP11-302L19.3	black	0.628086725
ENSG00000261061.1	RP11-303E16.2	brown	0.751112684
ENSG00000260213.1	RP11-303E16.3	brown	0.62815899
ENSG00000261141.1	RP11-303E16.5	turquoise	0.695459404
ENSG00000259933.2	RP11-304L19.1	yellow	0.751373702
ENSG00000261663.1	RP11-304L19.11	yellow	0.794071405
ENSG00000261123.1	RP11-304L19.3	yellow	0.824531421
ENSG00000260260.1	RP11-304L19.5	red	0.740432232
ENSG00000224616.1	RP11-305E17.6	blue	0.730897603
ENSG00000259994.1	RP11-305E6.4	turquoise	0.885571629
ENSG00000272990.1	RP11-305K5.1	grey	0.352484637
ENSG00000273455.1	RP11-305O4.3	turquoise	0.543088357

ENSG00000223774.1	RP11-307B6.3	grey	0.307094236
ENSG00000232093.1	RP11-307C12.11	turquoise	0.700250739
ENSG00000271380.1	RP11-307C12.12	turquoise	0.618993492
ENSG00000272950.1	RP11-307C18.1	turquoise	0.571830419
ENSG00000272370.1	RP11-307L14.1	turquoise	0.771192698
ENSG00000261728.1	RP11-307O13.1	turquoise	0.762381618
ENSG00000234062.3	RP11-308D16.4	pink	0.9201896
ENSG00000272198.1	RP11-309G3.3	turquoise	0.506153016
ENSG00000239815.1	RP11-309L24.4	turquoise	0.754850315
ENSG00000272899.1	RP11-309L24.9	turquoise	0.826802063
ENSG00000245975.2	RP11-30K9.6	turquoise	0.613650065
ENSG00000232186.1	RP11-311P8.2	yellow	0.841774101
ENSG00000229874.2	RP11-312O7.2	turquoise	0.626728391
ENSG00000215146.4	RP11-313J2.1	turquoise	0.739142907
ENSG00000213939.4	RP11-314A20.1	red	0.551231009
ENSG00000273301.1	RP11-314B1.2	turquoise	0.860669586
ENSG00000230939.1	RP11-314C16.1	turquoise	0.787636197
ENSG00000229728.1	RP11-314N13.3	turquoise	0.789007235
ENSG00000260981.1	RP11-315A16.1	grey	0.242167257
ENSG00000249006.1	RP11-317B7.2	grey	0.330462518
ENSG00000253549.1	RP11-317J10.2	brown	0.644184572
ENSG00000253642.1	RP11-317N12.1	grey	0.268497082
ENSG00000257272.1	RP11-317N8.3	turquoise	0.684087107
ENSG00000261185.1	RP11-317P15.5	turquoise	0.853177437
ENSG00000227740.1	RP11-318C24.2	grey	0.681585636
ENSG00000261252.1	RP11-318L16.6	grey	0.296424431
ENSG00000247081.3	RP11-318M2.2	black	0.614328803
ENSG00000248932.1	RP11-319G6.1	turquoise	0.769535664
ENSG00000238198.1	RP11-31F15.2	blue	0.700022556
ENSG00000226403.1	RP11-31F19.1	brown	0.578747871
ENSG00000260878.1	RP11-320H14.1	turquoise	0.743268649
ENSG00000254783.1	RP11-320L11.2	turquoise	0.77653445
ENSG00000259045.1	RP11-320M16.1	grey	-0.268649606
ENSG00000257543.1	RP11-321F8.4	yellow	0.536926849
ENSG00000233200.1	RP11-324I22.2	grey	0.205766625
ENSG00000269952.1	RP11-324I22.3	grey	0.46977498
ENSG00000271335.1	RP11-324I22.4	turquoise	0.759112706
ENSG00000272551.1	RP11-324L17.1	turquoise	0.687580901
ENSG00000261114.1	RP11-325K4.2	blue	0.575832318
ENSG00000261270.1	RP11-325K4.3	blue	0.559177706
ENSG00000251661.3	RP11-326C3.11	grey	0.372089465
ENSG00000255328.1	RP11-326C3.12	grey	0.245415081
ENSG00000254910.1	RP11-326C3.7	grey	0.386427994
ENSG00000233554.1	RP11-326F20.5	red	0.529408138
ENSG00000272755.1	RP11-326G21.1	brown	0.402680886
ENSG00000271646.1	RP11-326I11.3	brown	0.739596237
ENSG00000271538.1	RP11-326I11.4	turquoise	0.562265614
ENSG00000270426.1	RP11-326I11.5	yellow	0.712522542

ENSG00000263823.1	RP11-326K13.4	turquoise	0.823038985
ENSG00000231856.1	RP11-327P2.5	turquoise	0.587969755
ENSG00000273403.1	RP11-329B9.3	turquoise	0.541720188
ENSG00000272970.1	RP11-329B9.4	turquoise	0.571375362
ENSG00000272922.1	RP11-329B9.5	grey	0.395067101
ENSG00000237161.3	RP11-32B5.1	turquoise	0.711974659
ENSG00000272861.1	RP11-332H14.1	turquoise	0.426752785
ENSG00000272994.1	RP11-332H14.2	turquoise	0.904034165
ENSG00000267280.1	RP11-332H18.4	grey	0.328105861
ENSG00000261879.1	RP11-333E1.1	turquoise	0.853967985
ENSG00000263164.1	RP11-333E1.2	turquoise	0.673369714
ENSG00000260977.1	RP11-333I13.1	turquoise	0.779805905
ENSG00000262580.1	RP11-334C17.5	turquoise	0.634859007
ENSG00000237188.3	RP11-337C18.8	turquoise	0.889275697
ENSG00000271721.1	RP11-337C18.9	blue	0.749066491
ENSG00000273267.1	RP11-338K13.1	turquoise	0.394999553
ENSG00000270171.1	RP11-338N10.1	brown	0.596172836
ENSG00000270035.1	RP11-338N10.2	brown	0.624687089
ENSG00000269978.1	RP11-338N10.3	turquoise	0.61784722
ENSG00000272593.1	RP11-339B21.11	grey	0.719852623
ENSG00000272696.1	RP11-339B21.13	grey	0.723954621
ENSG00000273281.1	RP11-339B21.14	turquoise	0.787239656
ENSG00000272960.1	RP11-339B21.15	red	0.664822037
ENSG00000228510.2	RP11-339B21.8	brown	0.722922873
ENSG00000245958.2	RP11-33B1.1	turquoise	0.908265256
ENSG00000260091.1	RP11-33B1.4	yellow	0.769305706
ENSG00000272880.1	RP11-33M22.2	turquoise	0.603826382
ENSG00000272644.1	RP11-33O4.1	grey	0.504342046
ENSG00000261827.1	RP11-340F14.5	brown	0.794711668
ENSG00000261186.1	RP11-341N2.1	turquoise	0.57674538
ENSG00000270696.1	RP11-342K6.1	turquoise	0.870570555
ENSG00000271452.1	RP11-342K6.2	turquoise	0.644914523
ENSG00000270996.1	RP11-342K6.4	turquoise	0.527612296
ENSG00000233597.3	RP11-343B5.1	grey	0.617608009
ENSG00000224114.1	RP11-343H5.4	grey	0.102619351
ENSG00000269996.1	RP11-343N15.5	turquoise	0.887760694
ENSG00000272630.1	RP11-344N10.5	yellow	0.842196541
ENSG00000272583.1	RP11-344P13.6	turquoise	0.740503296
ENSG00000272106.1	RP11-345P4.9	turquoise	0.921269851
ENSG00000271009.2	RP11-346C20.3	yellow	0.690421091
ENSG00000272250.1	RP11-346C20.4	blue	0.588547035
ENSG00000272509.1	RP11-347C18.5	turquoise	0.555368754
ENSG00000272849.1	RP11-347I19.8	yellow	0.848664969
ENSG00000229151.1	RP11-348F1.3	turquoise	0.779656531
ENSG00000236905.2	RP11-348H3.2	grey	0.507184025
ENSG00000270589.1	RP11-348N5.7	yellow	0.807074382
ENSG00000272077.1	RP11-348P10.2	yellow	0.838272099
ENSG00000257621.3	RP11-349A22.5	yellow	0.947732524

ENSG00000257742.1	RP11-350F4.2	yellow	0.893679176
ENSG00000255201.1	RP11-350N15.4	blue	0.570364115
ENSG00000272092.1	RP11-350N15.5	turquoise	0.686689244
ENSG00000272159.1	RP11-350N15.6	grey	0.197332852
ENSG00000273008.1	RP11-351D16.3	turquoise	0.759161553
ENSG00000249780.1	RP11-352E6.2	grey	-0.1044883
ENSG00000260773.1	RP11-352G18.2	yellow	0.774819838
ENSG00000273009.1	RP11-352G9.1	grey	0.641080878
ENSG00000261597.1	RP11-353B9.1	turquoise	0.914861028
ENSG00000273265.1	RP11-353K11.1	brown	0.606790115
ENSG00000262188.1	RP11-353N14.4	brown	0.811487227
ENSG00000261696.1	RP11-354P17.15	brown	0.714229949
ENSG00000270820.1	RP11-355B11.2	turquoise	0.825383434
ENSG00000259796.1	RP11-355I22.7	turquoise	0.910592853
ENSG00000261094.2	RP11-355O1.11	yellow	0.889465061
ENSG00000271833.1	RP11-356B19.11	brown	0.525745782
ENSG00000250303.2	RP11-356J5.12	turquoise	0.81301456
ENSG00000235437.3	RP11-357C3.3	turquoise	0.93588977
ENSG00000255618.1	RP11-357K6.1	turquoise	0.755616886
ENSG00000250132.2	RP11-359B12.2	turquoise	0.90404433
ENSG00000270607.1	RP11-359E10.1	turquoise	0.889074101
ENSG00000272631.1	RP11-359E3.4	turquoise	0.818452247
ENSG00000259110.1	RP11-359N5.1	turquoise	0.709802561
ENSG00000249685.1	RP11-360F5.3	brown	0.543130651
ENSG00000272130.1	RP11-360I2.1	grey	0.262914372
ENSG00000225135.1	RP11-361F15.2	turquoise	0.874132677
ENSG00000272995.1	RP11-362J17.1	turquoise	0.514803636
ENSG00000269984.1	RP11-362K14.5	turquoise	0.790455488
ENSG00000270096.1	RP11-362K14.6	turquoise	0.725738988
ENSG00000254027.1	RP11-363E6.3	turquoise	0.609890539
ENSG00000272425.1	RP11-363E6.4	turquoise	0.663627539
ENSG00000260912.1	RP11-363E7.4	black	0.658172025
ENSG00000258520.1	RP11-363J20.2	grey	0.283232142
ENSG00000230707.1	RP11-364B14.3	turquoise	0.827315118
ENSG00000272576.1	RP11-365H22.2	turquoise	0.611679903
ENSG00000259515.1	RP11-365N19.2	yellow	0.781482998
ENSG00000248632.1	RP11-366M4.11	blue	0.554625492
ENSG00000273113.1	RP11-367H1.1	grey	0.385551187
ENSG00000272744.1	RP11-367N14.3	turquoise	0.521161675
ENSG00000242268.2	RP11-368I23.2	turquoise	0.687534976
ENSG00000272797.1	RP11-368I23.3	turquoise	0.631775066
ENSG00000273177.1	RP11-368I23.4	turquoise	0.514336376
ENSG00000258985.1	RP11-368P15.3	turquoise	0.601676141
ENSG00000241570.4	RP11-372E1.6	turquoise	0.832322238
ENSG00000270681.1	RP11-372K14.2	grey	0.322787445
ENSG00000270640.1	RP11-373D23.2	grey	0.256867935
ENSG00000248859.2	RP11-375B1.3	red	0.539053241
ENSG00000272267.1	RP11-375N15.2	blue	0.510544444

ENSG00000261338.1	RP11-378A13.1	turquoise	0.855365446
ENSG00000261402.1	RP11-378I6.1	midnightblue	0.808679481
ENSG00000225265.1	RP11-378J18.3	turquoise	0.743744015
ENSG00000272750.1	RP11-378J18.8	turquoise	0.641296
ENSG00000239804.1	RP11-379B18.1	pink	0.558471162
ENSG00000241288.3	RP11-379B18.5	turquoise	0.762178089
ENSG00000240207.2	RP11-379F4.4	turquoise	0.820830957
ENSG00000272440.1	RP11-379F4.6	grey	0.258550027
ENSG00000271778.1	RP11-379F4.8	turquoise	0.677658414
ENSG00000272247.1	RP11-379F4.9	turquoise	0.53776872
ENSG00000271122.1	RP11-379H18.1	blue	0.866660039
ENSG00000261136.1	RP11-37C7.3	turquoise	0.775291332
ENSG00000230633.1	RP11-37E23.5	turquoise	0.795131049
ENSG00000270028.1	RP11-380L11.4	turquoise	0.746604001
ENSG00000267541.1	RP11-380M21.2	grey	-0.178541621
ENSG00000250159.2	RP11-381K20.2	brown	0.773547459
ENSG00000270237.1	RP11-381K20.5	turquoise	0.505732117
ENSG00000260579.1	RP11-382A20.2	turquoise	0.702202272
ENSG00000271387.1	RP11-382D12.2	yellow	0.547848944
ENSG00000271975.1	RP11-383J24.6	turquoise	0.832278413
ENSG00000239268.2	RP11-384F7.2	turquoise	0.735823232
ENSG00000225892.3	RP11-384K6.2	yellow	0.866680978
ENSG00000260404.2	RP11-384K6.6	turquoise	0.83741644
ENSG00000267034.1	RP11-384O8.1	turquoise	0.856678695
ENSG00000260947.1	RP11-384P7.7	turquoise	0.669446562
ENSG00000230325.1	RP11-385F5.4	yellow	0.720416413
ENSG00000273058.1	RP11-385F5.5	yellow	0.6328125
ENSG00000270761.1	RP11-385F7.1	red	0.587937068
ENSG00000257913.1	RP11-386G11.5	turquoise	0.795087684
ENSG00000273338.1	RP11-386I14.4	grey	0.397681895
ENSG00000258741.2	RP11-386M24.4	turquoise	0.664159004
ENSG00000262410.1	RP11-388C12.8	yellow	0.817269982
ENSG00000260060.1	RP11-388M20.1	turquoise	0.544729932
ENSG00000260304.1	RP11-388M20.6	blue	0.639692268
ENSG00000232682.1	RP11-388P9.2	brown	0.7683676
ENSG00000261269.1	RP11-389C8.2	grey	0.34442344
ENSG00000272365.1	RP11-389C8.3	turquoise	0.806596791
ENSG00000261292.1	RP11-389G6.3	turquoise	0.847693285
ENSG00000236066.1	RP11-389O22.1	grey	0.561138588
ENSG00000231187.2	RP11-38L15.3	turquoise	0.833355663
ENSG00000273297.1	RP11-38M8.1	turquoise	0.793591283
ENSG00000272686.1	RP11-390E23.6	blue	0.841139207
ENSG00000236924.1	RP11-390F4.6	grey	0.29347764
ENSG00000270060.1	RP11-390K5.6	yellow	0.868695686
ENSG00000225177.1	RP11-390P2.4	grey	0.349569179
ENSG00000272452.1	RP11-391M1.4	turquoise	0.795353706
ENSG00000229789.1	RP11-391M20.1	turquoise	0.79058437
ENSG00000273036.1	RP11-392E22.12	grey	0.747062218

ENSG00000250989.1	RP11-392E22.5	turquoise	0.846738741
ENSG00000270109.1	RP11-393M11.2	grey	0.178834896
ENSG00000272667.1	RP11-395A13.2	turquoise	0.740225701
ENSG00000227053.1	RP11-395B7.4	grey	0.370594417
ENSG00000260336.1	RP11-395B7.7	blue	0.695270267
ENSG00000254615.2	RP11-395G23.3	turquoise	0.74593721
ENSG00000260296.1	RP11-395I6.3	turquoise	0.737160555
ENSG00000234771.2	RP11-395P17.3	yellow	0.938014931
ENSG00000263033.1	RP11-396B14.2	yellow	0.735277279
ENSG00000225518.2	RP11-396C23.2	turquoise	0.722191313
ENSG00000257718.1	RP11-396F22.1	black	0.504063876
ENSG00000233369.3	RP11-396K3.1	blue	0.747620565
ENSG00000272349.1	RP11-397O8.7	turquoise	0.623032822
ENSG00000272853.1	RP11-398C13.6	turquoise	0.852784808
ENSG00000229852.2	RP11-398K22.12	turquoise	0.943510048
ENSG00000270087.1	RP11-399K21.11	grey	0.217622947
ENSG00000273248.1	RP11-399K21.13	turquoise	0.447081465
ENSG00000272692.1	RP11-399K21.14	turquoise	0.825839127
ENSG00000205898.3	RP11-3B12.4	turquoise	0.659558997
ENSG00000259408.1	RP11-3D4.3	turquoise	0.786469812
ENSG00000214652.4	RP11-3N2.13	turquoise	0.670252658
ENSG00000267632.1	RP11-400F19.18	yellow	0.841354974
ENSG00000266962.1	RP11-400F19.6	turquoise	0.923428647
ENSG00000261685.2	RP11-401P9.4	brown	0.77124835
ENSG00000226067.2	RP11-403I13.7	turquoise	0.524574232
ENSG00000235999.1	RP11-403I13.8	turquoise	0.700432448
ENSG00000273153.1	RP11-406H21.2	grey	0.451932919
ENSG00000260038.1	RP11-407G23.4	grey	0.424883141
ENSG00000258940.2	RP11-407N17.5	turquoise	0.773445596
ENSG00000272871.1	RP11-408A13.4	yellow	0.863436389
ENSG00000265888.1	RP11-408H20.2	turquoise	0.70948281
ENSG00000219928.2	RP11-40C6.2	grey	0.280969419
ENSG00000253948.1	RP11-410L14.2	red	0.882739899
ENSG00000267756.1	RP11-411B10.4	turquoise	0.7511093
ENSG00000260329.1	RP11-412D9.4	turquoise	0.906898529
ENSG00000271964.1	RP11-415F23.2	turquoise	0.520242227
ENSG00000272529.1	RP11-415F23.4	turquoise	0.616736879
ENSG00000225313.2	RP11-415J8.3	yellow	0.894114837
ENSG00000260328.1	RP11-416I2.1	turquoise	0.852585378
ENSG00000273108.1	RP11-416N2.4	turquoise	0.797842507
ENSG00000256955.1	RP11-417L19.2	turquoise	0.548429516
ENSG00000255992.1	RP11-417L19.4	turquoise	0.733078479
ENSG00000231365.1	RP11-418J17.1	yellow	0.858762194
ENSG00000226232.4	RP11-419C5.2	yellow	0.617275742
ENSG00000251432.2	RP11-420A23.1	pink	0.764419455
ENSG00000230438.5	RP11-420G6.4	grey	0.266943405
ENSG00000270504.1	RP11-420L9.5	black	0.775410049
ENSG00000247903.1	RP11-421F16.3	turquoise	0.80782675

ENSG00000233184.2	RP11-421L21.3	turquoise	0.748634984
ENSG00000272097.1	RP11-421M1.8	turquoise	0.818519505
ENSG00000233621.1	RP11-422J8.1	brown	0.551637028
ENSG00000273026.1	RP11-422P24.10	turquoise	0.523649987
ENSG00000272654.1	RP11-422P24.11	turquoise	0.890865449
ENSG00000260987.1	RP11-423G4.7	turquoise	0.892149928
ENSG00000170089.11	RP11-423H2.1	turquoise	0.748010688
ENSG00000260197.1	RP11-424G14.1	grey	0.338902099
ENSG00000262890.1	RP11-424M24.5	pink	0.827151092
ENSG00000196951.6	RP11-425I13.3	turquoise	0.854402761
ENSG00000260953.1	RP11-426C22.6	turquoise	0.623930611
ENSG00000188971.4	RP11-427H3.3	yellow	0.93493229
ENSG00000271978.1	RP11-428J1.4	turquoise	0.736169258
ENSG00000256790.1	RP11-429A20.3	turquoise	0.783491015
ENSG00000256654.1	RP11-429A20.4	grey	0.717505648
ENSG00000271992.1	RP11-42O15.3	grey	0.471472094
ENSG00000259577.1	RP11-430B1.2	turquoise	0.836170478
ENSG00000271200.1	RP11-430G17.3	grey	0.589207934
ENSG00000254427.1	RP11-430H10.1	red	0.516690567
ENSG00000238058.1	RP11-432J22.2	turquoise	0.833428886
ENSG00000272774.1	RP11-433A10.3	brown	0.49477167
ENSG00000258702.1	RP11-433J8.1	turquoise	0.871425266
ENSG00000258979.1	RP11-433J8.2	turquoise	0.712236172
ENSG00000260837.1	RP11-434B12.1	turquoise	0.910820613
ENSG00000247157.2	RP11-434C1.1	turquoise	0.818329137
ENSG00000273437.1	RP11-434H6.7	turquoise	0.45474919
ENSG00000271659.1	RP11-435O5.4	grey	0.576053087
ENSG00000271860.1	RP11-436D23.1	turquoise	0.551473482
ENSG00000231427.1	RP11-436F9.1	brown	0.603826217
ENSG00000231252.1	RP11-436K8.1	green	0.768330913
ENSG00000272800.1	RP11-438L19.1	turquoise	0.758465399
ENSG00000257231.1	RP11-438N16.2	turquoise	0.553559718
ENSG00000253764.1	RP11-439C15.4	green	0.61269565
ENSG00000260855.1	RP11-439E19.10	turquoise	0.881968159
ENSG00000227953.2	RP11-439E19.3	turquoise	0.79947666
ENSG00000271947.1	RP11-439M11.1	turquoise	0.679290441
ENSG00000188002.6	RP11-43F13.1	blue	0.721194877
ENSG00000272913.1	RP11-440D17.3	turquoise	0.891180188
ENSG00000273305.1	RP11-440D17.4	turquoise	0.824562051
ENSG00000249592.1	RP11-440L14.1	turquoise	0.749157663
ENSG00000272372.1	RP11-441F2.5	turquoise	0.733979814
ENSG00000224934.2	RP11-441O15.3	yellow	0.884051473
ENSG00000229388.1	RP11-442N24__B.:	brown	0.56688978
ENSG00000132832.5	RP11-445H22.3	turquoise	0.86081497
ENSG00000244558.1	RP11-445H22.4	turquoise	0.877397402
ENSG00000273293.1	RP11-445N20.3	midnightblue	0.649129814
ENSG00000236814.1	RP11-446E9.1	turquoise	0.647917299
ENSG00000241218.1	RP11-446H18.1	yellow	0.789631352

ENSG00000272597.1	RP11-446H18.6	brown	0.573175184
ENSG00000273329.1	RP11-448A19.1	turquoise	0.791532101
ENSG00000261490.1	RP11-448G15.3	blue	0.662379261
ENSG00000260448.1	RP11-449H11.1	brown	0.705223563
ENSG00000273151.1	RP11-449P15.2	blue	0.829869699
ENSG00000184414.2	RP11-44M6.3	grey	0.115279197
ENSG00000272384.1	RP11-44N11.2	grey	0.277295593
ENSG00000257556.1	RP11-44N21.1	turquoise	0.83344015
ENSG00000272702.1	RP11-44N22.3	red	0.595230415
ENSG00000240045.1	RP11-451G4.2	midnightblue	0.611959206
ENSG00000203876.5	RP11-451M19.3	turquoise	0.555051692
ENSG00000228106.1	RP11-452F19.3	red	0.534447438
ENSG00000231970.1	RP11-452K12.7	turquoise	0.641512848
ENSG00000261474.1	RP11-452L6.1	yellow	0.776458418
ENSG00000260625.1	RP11-452L6.7	brown	0.603557936
ENSG00000265015.1	RP11-454P7.3	turquoise	0.6512884
ENSG00000250616.2	RP11-455F5.3	turquoise	0.843569855
ENSG00000273240.1	RP11-455J20.3	turquoise	0.631674957
ENSG00000261584.1	RP11-457M11.5	yellow	0.809443432
ENSG00000233396.3	RP11-458D21.1	turquoise	0.818496907
ENSG00000272709.1	RP11-458D21.6	grey	0.308303907
ENSG00000230295.1	RP11-458F8.2	blue	0.668667698
ENSG00000273142.1	RP11-458F8.4	yellow	0.847773706
ENSG00000272048.1	RP11-458N5.1	grey	0.626970146
ENSG00000259775.1	RP11-45P15.4	turquoise	0.838046417
ENSG00000263235.1	RP11-461A8.4	grey	0.580519411
ENSG00000272791.1	RP11-464F9.22	pink	0.682330297
ENSG00000217801.5	RP11-465B22.3	turquoise	0.682268148
ENSG00000234678.1	RP11-465N4.4	yellow	0.808334159
ENSG00000273478.1	RP11-465N4.5	yellow	0.731488947
ENSG00000255651.2	RP11-466C23.4	turquoise	0.369977036
ENSG00000256280.1	RP11-466C23.5	turquoise	0.68729617
ENSG00000249755.2	RP11-466G12.2	turquoise	0.78348557
ENSG00000266045.1	RP11-466P24.7	turquoise	0.88761333
ENSG00000270457.1	RP11-467C18.1	turquoise	0.553751236
ENSG00000259826.1	RP11-467D6.1	turquoise	0.889910927
ENSG00000213542.3	RP11-467H10.1	turquoise	0.7537857
ENSG00000253973.2	RP11-467K18.2	turquoise	0.821446058
ENSG00000259321.1	RP11-468E2.5	yellow	0.813210494
ENSG00000260006.1	RP11-469M7.1	turquoise	0.768564316
ENSG00000243155.1	RP11-46A10.5	yellow	0.710934857
ENSG00000259877.1	RP11-46C24.7	turquoise	0.884922668
ENSG00000261512.2	RP11-46D6.1	turquoise	0.791323484
ENSG00000238260.1	RP11-46F15.2	grey	0.761375659
ENSG00000250790.3	RP11-46H11.3	pink	0.8376449
ENSG00000258424.1	RP11-471B22.2	yellow	0.815206374
ENSG00000224097.5	RP11-472B18.1	turquoise	0.645914624
ENSG00000237797.1	RP11-472N13.3	grey	0.251267038

ENSG00000263244.1	RP11-473I1.10	turquoise	0.943351923
ENSG00000262944.1	RP11-473I1.9	turquoise	0.846201921
ENSG00000263072.1	RP11-473M20.14	turquoise	0.886078023
ENSG00000261971.2	RP11-473M20.7	yellow	0.910604001
ENSG00000273064.1	RP11-474G23.3	grey	0.760293459
ENSG00000270225.1	RP11-475J5.4	grey	-0.113108209
ENSG00000270906.1	RP11-475J5.6	grey	-0.211901095
ENSG00000235381.1	RP11-477D19.2	turquoise	0.822679507
ENSG00000272156.1	RP11-477N3.1	grey	0.359843911
ENSG00000251188.1	RP11-478C6.6	turquoise	0.703222809
ENSG00000273372.1	RP11-479O17.10	turquoise	0.755022083
ENSG00000272933.1	RP11-47A8.5	turquoise	0.76312297
ENSG00000258926.1	RP11-47I22.1	green	0.547910271
ENSG00000260261.1	RP11-480A16.1	yellow	0.867415316
ENSG00000273409.1	RP11-480C22.1	magenta	0.473057975
ENSG00000272049.1	RP11-480D4.6	turquoise	0.549278013
ENSG00000254507.2	RP11-481A20.10	turquoise	0.571837456
ENSG00000255098.1	RP11-481A20.11	turquoise	0.655183816
ENSG00000260186.1	RP11-481J2.2	turquoise	0.712413853
ENSG00000257918.1	RP11-482D24.3	turquoise	0.769229024
ENSG00000260750.1	RP11-482M8.1	turquoise	0.827954359
ENSG00000269935.1	RP11-482M8.3	turquoise	0.758526213
ENSG00000260879.1	RP11-483I13.5	turquoise	0.777234694
ENSG00000272565.1	RP11-485G4.2	turquoise	0.897219716
ENSG00000271576.1	RP11-486G15.2	yellow	0.903248413
ENSG00000258301.2	RP11-488C13.5	turquoise	0.868258535
ENSG00000259081.1	RP11-488C13.6	turquoise	0.688410336
ENSG00000259865.1	RP11-488L18.10	blue	0.645020917
ENSG00000259446.1	RP11-489D6.2	grey	0.167158367
ENSG00000253988.1	RP11-489O18.1	magenta	0.607493738
ENSG00000240898.1	RP11-48B14.1	blue	0.628124016
ENSG00000260317.1	RP11-48B3.4	turquoise	0.675344948
ENSG00000259946.1	RP11-490G2.2	turquoise	0.644595677
ENSG00000229447.2	RP11-490K7.4	blue	0.650334366
ENSG00000260025.1	RP11-490M8.1	turquoise	0.804357608
ENSG00000235016.1	RP11-493K19.3	yellow	0.878787984
ENSG00000261121.1	RP11-496D24.2	brown	0.745003476
ENSG00000249626.1	RP11-496H1.1	yellow	0.801403558
ENSG00000273308.1	RP11-496H1.2	grey	0.442110324
ENSG00000205562.1	RP11-497E19.1	turquoise	0.858296832
ENSG00000263731.1	RP11-498C9.15	turquoise	0.720854849
ENSG00000263320.1	RP11-498D10.6	turquoise	0.624468379
ENSG00000270102.1	RP11-498E2.8	turquoise	0.478780459
ENSG00000203279.3	RP11-498P14.5	turquoise	0.662375898
ENSG00000248161.1	RP11-499E18.1	turquoise	0.761469363
ENSG00000240291.1	RP11-499P20.2	yellow	0.879919049
ENSG00000260023.1	RP11-49C24.1	turquoise	0.710760997
ENSG00000260359.1	RP11-4F5.2	turquoise	0.729156068

ENSG00000243659.1	RP11-4K3__A.3	turquoise	0.589084753
ENSG00000259953.1	RP11-4O1.2	turquoise	0.909551979
ENSG00000272269.1	RP11-500C11.3	turquoise	0.742465556
ENSG00000226864.1	RP11-500G22.2	grey	0.363620198
ENSG00000272233.1	RP11-503E24.3	turquoise	0.646278338
ENSG00000272056.1	RP11-503P10.1	turquoise	0.71632907
ENSG00000272645.1	RP11-504P24.8	yellow	0.84550372
ENSG00000260018.1	RP11-505K9.1	turquoise	0.663073405
ENSG00000242798.1	RP11-506M12.1	turquoise	0.76406461
ENSG00000258789.1	RP11-507K2.3	turquoise	0.852916491
ENSG00000272983.1	RP11-508N22.12	turquoise	0.834141522
ENSG00000273019.1	RP11-508N22.13	turquoise	0.543537839
ENSG00000261452.1	RP11-509E16.1	turquoise	0.81955435
ENSG00000235326.1	RP11-509J21.3	blue	0.590647978
ENSG00000259250.1	RP11-50C13.1	midnightblue	0.723028839
ENSG00000224127.1	RP11-510C10.2	midnightblue	0.638534644
ENSG00000261213.1	RP11-510C10.4	midnightblue	0.563565585
ENSG00000272842.1	RP11-513M16.7	blue	0.571114764
ENSG00000273226.1	RP11-513M16.8	turquoise	0.73441584
ENSG00000261167.1	RP11-517B11.7	turquoise	0.894232037
ENSG00000230555.2	RP11-517P14.2	turquoise	0.854114747
ENSG00000236114.1	RP11-517P14.7	pink	0.842273802
ENSG00000270587.1	RP11-51F16.9	turquoise	0.748473839
ENSG00000271869.1	RP11-51J9.5	red	0.70732491
ENSG00000272375.1	RP11-51J9.6	turquoise	0.748876893
ENSG00000228376.3	RP11-520B13.4	pink	0.744838775
ENSG00000251602.2	RP11-521B24.3	turquoise	0.857821544
ENSG00000257270.1	RP11-521B24.5	yellow	0.821885612
ENSG00000260634.1	RP11-521O16.1	turquoise	0.807776137
ENSG00000254473.1	RP11-522I20.3	turquoise	0.801114245
ENSG00000226352.2	RP11-523H24.3	yellow	0.776521719
ENSG00000265511.1	RP11-524F11.1	turquoise	0.746163985
ENSG00000270127.1	RP11-526I2.5	turquoise	0.535479584
ENSG00000225554.1	RP11-527D7.1	turquoise	0.843169428
ENSG00000273306.1	RP11-527J8.1	turquoise	0.783636427
ENSG00000261643.1	RP11-529E10.6	midnightblue	0.798702389
ENSG00000261372.1	RP11-529H20.6	yellow	0.781849256
ENSG00000253636.1	RP11-531A24.5	yellow	0.824026957
ENSG00000273261.1	RP11-531F16.4	blue	0.548340837
ENSG00000272906.1	RP11-533E19.7	brown	0.52487329
ENSG00000261000.1	RP11-534L20.5	grey	0.306461071
ENSG00000224848.1	RP11-535M15.1	turquoise	0.859891976
ENSG00000233820.2	RP11-535M15.2	turquoise	0.805550609
ENSG00000232807.2	RP11-536K7.3	yellow	0.870508292
ENSG00000251580.1	RP11-539L10.3	turquoise	0.689277424
ENSG00000251141.1	RP11-53O19.1	turquoise	0.880913815
ENSG00000272335.1	RP11-53O19.3	turquoise	0.935893591
ENSG00000270015.1	RP11-540B6.6	blue	0.845950711

ENSG00000260461.1	RP11-541N10.3	turquoise	0.92382554
ENSG00000247970.2	RP11-543C4.1	turquoise	0.833252544
ENSG00000223478.1	RP11-545E17.3	turquoise	0.83673518
ENSG00000235652.3	RP11-545I5.3	turquoise	0.903918917
ENSG00000258591.2	RP11-545M17.3	turquoise	0.768150339
ENSG00000270557.1	RP11-546J1.1	turquoise	0.662889635
ENSG00000237457.2	RP11-547I7.2	blue	0.527783411
ENSG00000273466.1	RP11-548H3.1	yellow	0.756815456
ENSG00000272564.1	RP11-548P2.2	brown	0.703224596
ENSG00000259985.1	RP11-549B18.1	turquoise	0.887681375
ENSG00000213433.5	RP11-54C4.1	brown	0.535325151
ENSG00000215237.5	RP11-54D18.2	turquoise	0.615460691
ENSG00000251196.1	RP11-54F2.1	turquoise	0.825390737
ENSG00000224764.1	RP11-54O15.3	brown	0.476115559
ENSG00000272512.1	RP11-54O7.17	grey	0.411636294
ENSG00000228058.1	RP11-552D4.1	magenta	0.752196347
ENSG00000267342.1	RP11-552F3.10	yellow	0.855358397
ENSG00000243960.1	RP11-552M11.4	turquoise	0.640172693
ENSG00000260948.1	RP11-552M11.8	turquoise	0.88332362
ENSG00000259976.1	RP11-553L6.5	green	0.728160868
ENSG00000259799.1	RP11-554A11.9	turquoise	0.789578241
ENSG00000241770.1	RP11-555M1.3	turquoise	0.625934472
ENSG00000232310.2	RP11-557H15.4	brown	0.781494565
ENSG00000272002.1	RP11-557L19.1	turquoise	0.644069187
ENSG00000229212.3	RP11-561C5.4	red	0.479102134
ENSG00000224525.2	RP11-561I11.3	turquoise	0.55288115
ENSG00000272865.1	RP11-561I11.4	grey	0.130430457
ENSG00000213642.3	RP11-561N12.5	turquoise	0.713102198
ENSG00000235349.1	RP11-561N12.6	turquoise	0.571650122
ENSG00000215244.2	RP11-563J2.2	blue	0.501197965
ENSG00000272619.1	RP11-563K23.1	turquoise	0.757451679
ENSG00000255624.1	RP11-564D11.3	turquoise	0.841514766
ENSG00000233626.2	RP11-565J7.1	turquoise	0.561483163
ENSG00000261453.1	RP11-565N2.1	midnightblue	0.696903185
ENSG00000260804.2	RP11-566E18.3	turquoise	0.938397621
ENSG00000259344.1	RP11-566K19.6	turquoise	0.910701005
ENSG00000242193.5	RP11-568K15.1	brown	0.698169222
ENSG00000260101.1	RP11-568N6.1	turquoise	0.843064487
ENSG00000226862.1	RP11-569A11.1	turquoise	0.412385467
ENSG00000237212.1	RP11-569G13.2	turquoise	0.485991368
ENSG00000259556.2	RP11-56B16.2	turquoise	0.782754652
ENSG00000234043.3	RP11-56M3.1	turquoise	0.758992917
ENSG00000272650.1	RP11-571I18.4	grey	0.409306067
ENSG00000272777.1	RP11-571L19.8	turquoise	0.815190038
ENSG00000270039.1	RP11-571M6.17	turquoise	0.864674395
ENSG00000269903.1	RP11-571M6.18	turquoise	0.692789757
ENSG00000257499.2	RP11-571M6.8	turquoise	0.77023987
ENSG00000272761.1	RP11-572C15.6	green	0.440839381

ENSG00000270195.1	RP11-572O17.1	grey	0.534919056
ENSG00000231982.1	RP11-573D15.1	turquoise	0.736136034
ENSG00000232233.1	RP11-573D15.2	grey	0.548517523
ENSG00000263826.1	RP11-573D15.9	turquoise	0.867467798
ENSG00000261671.1	RP11-573G6.6	turquoise	0.8313027
ENSG00000255445.1	RP11-573M3.3	turquoise	0.763134864
ENSG00000272140.2	RP11-574K11.29	turquoise	0.765789373
ENSG00000224195.1	RP11-574K11.5	turquoise	0.672193659
ENSG00000261600.1	RP11-575H3.1	turquoise	0.414957916
ENSG00000235298.1	RP11-575L7.8	blue	0.775127529
ENSG00000261377.1	RP11-578F21.12	turquoise	0.76021798
ENSG00000254398.1	RP11-578F21.2	blue	0.508921634
ENSG00000260844.2	RP11-578F21.9	grey	0.530808796
ENSG00000260917.1	RP11-57H14.4	turquoise	0.938181647
ENSG00000253200.1	RP11-582J16.5	turquoise	0.840363493
ENSG00000249896.1	RP11-586D19.1	turquoise	0.88826512
ENSG00000260244.1	RP11-588K22.2	turquoise	0.878607858
ENSG00000269899.1	RP11-589N15.2	magenta	0.667371231
ENSG00000262089.1	RP11-589P10.5	turquoise	0.655199667
ENSG00000269975.1	RP11-58B17.2	turquoise	0.870194404
ENSG00000260398.1	RP11-594N15.3	turquoise	0.835666105
ENSG00000251429.1	RP11-597D13.7	brown	0.794831941
ENSG00000256694.1	RP11-598F7.5	grey	0.424065195
ENSG00000256540.1	RP11-598F7.6	grey	0.509144047
ENSG00000256673.1	RP11-599J14.2	grey	0.164258804
ENSG00000272760.1	RP11-5C23.1	green	0.647072134
ENSG00000273183.1	RP11-5C23.2	grey	0.610280302
ENSG00000232456.1	RP11-5P18.10	grey	0.407247454
ENSG00000260285.1	RP11-600F24.7	turquoise	0.831541703
ENSG00000272795.1	RP11-602N24.3	turquoise	0.562221238
ENSG00000255145.1	RP11-60I3.5	turquoise	0.649201213
ENSG00000257815.1	RP11-611E13.2	turquoise	0.798361423
ENSG00000260054.1	RP11-611L7.1	turquoise	0.850646271
ENSG00000256185.1	RP11-612B6.2	green	0.633833304
ENSG00000234160.1	RP11-613M10.6	turquoise	0.730010505
ENSG00000231160.5	RP11-617D20.1	turquoise	0.765852461
ENSG00000259291.1	RP11-617F23.1	turquoise	0.49502602
ENSG00000255362.1	RP11-619A14.3	turquoise	0.709024419
ENSG00000258122.1	RP11-61A14.1	turquoise	0.681705802
ENSG00000261705.1	RP11-61A14.2	blue	0.788938761
ENSG00000261088.1	RP11-61A14.3	blue	0.755530498
ENSG00000235033.3	RP11-61I13.3	brown	0.826015821
ENSG00000260805.1	RP11-61J19.4	turquoise	0.753129321
ENSG00000257698.1	RP11-620J15.3	turquoise	0.875403093
ENSG00000273321.1	RP11-621L6.3	brown	0.512786264
ENSG00000204272.6	RP11-622K12.1	turquoise	0.937127367
ENSG00000237742.2	RP11-624M8.1	grey	0.117069042
ENSG00000247416.2	RP11-629G13.1	brown	0.603788629

ENSG00000261220.2	RP11-629O1.2	grey	0.57325158
ENSG00000233609.3	RP11-62H7.2	turquoise	0.668796182
ENSG00000272915.1	RP11-62J1.4	grey	0.459543101
ENSG00000257354.1	RP11-631N16.2	blue	0.569250866
ENSG00000230202.1	RP11-632C17__A.1	grey	0.229398675
ENSG00000223509.4	RP11-632K20.7	turquoise	0.747704276
ENSG00000273391.1	RP11-634H22.1	turquoise	0.666760941
ENSG00000267390.1	RP11-635N19.1	red	0.799251253
ENSG00000258666.1	RP11-638I2.8	blue	0.494045745
ENSG00000272556.1	RP11-638I8.1	grey	0.563813862
ENSG00000260465.1	RP11-63M22.2	blue	0.781641149
ENSG00000225241.3	RP11-640M9.2	turquoise	0.850232202
ENSG00000259251.2	RP11-643M14.1	pink	0.868260024
ENSG00000258056.1	RP11-644F5.11	brown	0.618665029
ENSG00000260306.1	RP11-645C24.5	turquoise	0.732646218
ENSG00000269921.1	RP11-646I6.5	turquoise	0.62251534
ENSG00000270147.1	RP11-646I6.6	yellow	0.694498996
ENSG00000263843.1	RP11-649A18.12	turquoise	0.859466581
ENSG00000265242.1	RP11-649A18.7	turquoise	0.877785427
ENSG00000213144.2	RP11-64B16.2	green	0.538023172
ENSG00000261374.1	RP11-64K12.10	turquoise	0.877889585
ENSG00000238287.1	RP11-656D10.3	turquoise	0.597374337
ENSG00000238186.1	RP11-656D10.6	turquoise	0.675198716
ENSG00000240086.1	RP11-657O9.1	turquoise	0.808869656
ENSG00000258297.1	RP11-658F2.8	turquoise	0.871526357
ENSG00000262454.1	RP11-65J21.3	turquoise	0.738865679
ENSG00000233901.1	RP11-65J3.1	red	0.71710673
ENSG00000260931.1	RP11-65L3.1	turquoise	0.859041235
ENSG00000254682.1	RP11-660L16.2	turquoise	0.5721875
ENSG00000271912.1	RP11-661A12.14	turquoise	0.720432209
ENSG00000255050.1	RP11-661A12.9	yellow	0.750553913
ENSG00000248801.3	RP11-664D7.4	pink	0.899569056
ENSG00000249460.1	RP11-665C14.2	turquoise	0.715863635
ENSG00000261543.1	RP11-665J16.1	turquoise	0.664976673
ENSG00000267543.1	RP11-666A8.7	yellow	0.74159423
ENSG00000263050.1	RP11-667K14.3	turquoise	0.751506279
ENSG00000259604.1	RP11-66B24.1	brown	0.743447161
ENSG00000223356.1	RP11-66D17.5	turquoise	0.812901475
ENSG00000260081.1	RP11-66N11.8	red	0.856894006
ENSG00000258727.1	RP11-66N24.3	yellow	0.912492887
ENSG00000157306.10	RP11-66N24.4	turquoise	0.777689376
ENSG00000262951.1	RP11-670E13.2	midnightblue	0.779663644
ENSG00000259781.1	RP11-673C5.1	blue	0.75644925
ENSG00000265962.1	RP11-674N23.1	grey	0.361334331
ENSG00000263958.1	RP11-676J15.1	turquoise	0.799121975
ENSG00000254612.2	RP11-676M6.1	turquoise	0.703804609
ENSG00000254941.1	RP11-677M14.5	turquoise	0.688421624
ENSG00000273033.1	RP11-67L2.2	turquoise	0.905791598

ENSG00000257605.1	RP11-680A11.5	yellow	0.816082439
ENSG00000263906.1	RP11-680F20.11	turquoise	0.877411991
ENSG00000255027.2	RP11-680F20.9	turquoise	0.772648829
ENSG00000260872.1	RP11-680G24.5	grey	0.566213276
ENSG00000246308.1	RP11-685M7.3	turquoise	0.874522425
ENSG00000273456.1	RP11-686O6.2	turquoise	0.795798674
ENSG00000267167.1	RP11-687F6.1	yellow	0.694802006
ENSG00000205959.3	RP11-689P11.2	turquoise	0.83596565
ENSG00000260966.1	RP11-690D19.3	turquoise	0.881285524
ENSG00000266753.2	RP11-690G19.3	turquoise	0.890037508
ENSG00000237641.1	RP11-690I21.1	blue	0.734813207
ENSG00000250220.1	RP11-692D12.1	turquoise	0.787792098
ENSG00000270441.1	RP11-694I15.7	turquoise	0.762154446
ENSG00000228998.3	RP11-697E2.7	turquoise	0.937646209
ENSG00000261355.1	RP11-698N11.4	yellow	0.901444239
ENSG00000182109.3	RP11-69E11.4	yellow	0.867330306
ENSG00000273061.1	RP11-6J24.6	green	0.536630355
ENSG00000250673.1	RP11-6L6.2	turquoise	0.528389343
ENSG00000264920.1	RP11-6N17.4	turquoise	0.750803431
ENSG00000261616.1	RP11-6O2.3	turquoise	0.702576274
ENSG00000262006.1	RP11-700H6.4	turquoise	0.789108699
ENSG00000273369.1	RP11-700J17.1	turquoise	0.732261278
ENSG00000266954.1	RP11-701H16.4	brown	0.671074709
ENSG00000257647.1	RP11-701H24.3	turquoise	0.410862144
ENSG00000271347.1	RP11-701H24.7	turquoise	0.673232722
ENSG00000256594.3	RP11-705C15.2	blue	0.705168058
ENSG00000205664.6	RP11-706O15.1	turquoise	0.817796524
ENSG00000205035.4	RP11-707M1.1	turquoise	0.777362627
ENSG00000272155.1	RP11-707M3.3	grey	0.485232791
ENSG00000263503.1	RP11-707O23.5	grey	0.089317123
ENSG00000259668.1	RP11-707P17.2	brown	0.742548384
ENSG00000260236.1	RP11-708J19.1	brown	0.717849822
ENSG00000271474.1	RP11-710C12.1	turquoise	0.673957856
ENSG00000270689.1	RP11-712B9.4	turquoise	0.740224969
ENSG00000270578.1	RP11-712B9.5	turquoise	0.697091161
ENSG00000255062.1	RP11-712L6.5	turquoise	0.542154641
ENSG00000265579.1	RP11-713C5.1	turquoise	0.875228639
ENSG00000273233.1	RP11-713D19.1	grey	0.487373863
ENSG00000204241.3	RP11-713P17.3	turquoise	0.671049971
ENSG00000260095.1	RP11-715J22.3	turquoise	0.507286393
ENSG00000260293.1	RP11-715J22.6	green	0.551175424
ENSG00000250934.1	RP11-71E19.1	turquoise	0.704990012
ENSG00000272947.1	RP11-71H17.9	turquoise	0.807716265
ENSG00000239922.1	RP11-71N10.1	brown	0.825587106
ENSG00000264433.1	RP11-720L2.3	grey	0.147425286
ENSG00000264514.1	RP11-720L2.4	grey	0.299578179
ENSG00000259434.1	RP11-720L8.1	turquoise	0.692249011
ENSG00000272338.1	RP11-722E23.2	grey	0.313418353

ENSG00000187695.6	RP11-723O4.6	pink	0.913956127
ENSG00000261159.1	RP11-723O4.9	yellow	0.642236863
ENSG00000272769.1	RP11-725P16.2	turquoise	0.654421451
ENSG00000254676.1	RP11-727A23.4	blue	0.730033447
ENSG00000247137.4	RP11-727A23.5	turquoise	0.853023765
ENSG00000267811.1	RP11-727F15.11	turquoise	0.563594723
ENSG00000269176.1	RP11-727F15.12	turquoise	0.798203181
ENSG00000269463.1	RP11-727F15.13	grey	0.470164425
ENSG00000224016.2	RP11-728K20.1	turquoise	0.757365813
ENSG00000234175.1	RP11-730A19.9	blue	0.509052123
ENSG00000253341.1	RP11-730G20.2	turquoise	0.790609583
ENSG00000273486.1	RP11-731C17.2	brown	0.65261987
ENSG00000260918.1	RP11-731J8.2	turquoise	0.750630595
ENSG00000254641.1	RP11-732A19.2	turquoise	0.637677457
ENSG00000236537.1	RP11-732M18.3	brown	0.600589133
ENSG00000270344.1	RP11-734K2.4	red	0.57721417
ENSG00000226409.1	RP11-735G4.1	midnightblue	0.789690156
ENSG00000255471.1	RP11-736K20.5	blue	0.546052671
ENSG00000246523.3	RP11-736K20.6	blue	0.650833329
ENSG00000258738.1	RP11-73E17.2	turquoise	0.583025025
ENSG00000260526.1	RP11-73K9.2	turquoise	0.849737438
ENSG00000270108.1	RP11-73M18.6	cyan	0.616741092
ENSG00000269940.1	RP11-73M18.7	blue	0.606391354
ENSG00000269958.1	RP11-73M18.8	brown	0.555832524
ENSG00000269963.1	RP11-73M18.9	grey	0.628382379
ENSG00000235790.3	RP11-73M7.6	yellow	0.825182824
ENSG00000270112.2	RP11-742D12.2	turquoise	0.68996538
ENSG00000227097.4	RP11-742N3.1	grey	0.385370244
ENSG00000239335.4	RP11-745O10.2	grey	0.370246633
ENSG00000262050.1	RP11-74E22.3	blue	0.586680409
ENSG00000261963.2	RP11-74E22.4	turquoise	0.777597078
ENSG00000262902.1	RP11-750B16.1	grey	-0.3185929
ENSG00000255197.1	RP11-750H9.5	turquoise	0.794036613
ENSG00000250988.3	RP11-752G15.6	turquoise	0.937361569
ENSG00000259315.1	RP11-752G15.9	yellow	0.715963466
ENSG00000255320.1	RP11-755F10.1	grey	0.455373673
ENSG00000254461.1	RP11-755F10.3	yellow	0.855730694
ENSG00000264222.1	RP11-757O6.1	turquoise	0.681791168
ENSG00000225706.1	RP11-75C9.1	blue	0.698777148
ENSG00000213443.2	RP11-75L1.2	yellow	0.635456065
ENSG00000254343.2	RP11-760H22.2	brown	0.803119782
ENSG00000259562.2	RP11-762H8.2	yellow	0.799688047
ENSG00000257509.1	RP11-762I7.4	yellow	0.767235251
ENSG00000215548.2	RP11-764K9.4	blue	0.440580414
ENSG00000255693.1	RP11-766N7.3	turquoise	0.845656737
ENSG00000272263.1	RP11-767C1.2	turquoise	0.779086993
ENSG00000230896.1	RP11-767N6.7	turquoise	0.801796117
ENSG00000255435.2	RP11-770J1.3	turquoise	0.59056681

ENSG00000255384.1	RP11-770J1.4	turquoise	0.795708575
ENSG00000225484.2	RP11-773D16.1	turquoise	0.857334265
ENSG00000251615.3	RP11-774O3.3	yellow	0.907559526
ENSG00000226476.2	RP11-776H12.1	turquoise	0.752583004
ENSG00000249072.1	RP11-777B9.5	grey	-0.187140226
ENSG00000203472.3	RP11-77K12.4	blue	0.632078513
ENSG00000249846.2	RP11-77P16.4	turquoise	0.700288752
ENSG00000227896.2	RP11-77P6.2	yellow	0.835484562
ENSG00000256116.1	RP11-783K16.14	turquoise	0.604110174
ENSG00000271730.1	RP11-787I22.3	turquoise	0.591002006
ENSG00000263970.1	RP11-789C17.5	grey	0.335771255
ENSG00000270091.1	RP11-78O7.2	turquoise	0.796355401
ENSG00000251442.1	RP11-792D21.2	magenta	0.7599829
ENSG00000270175.1	RP11-793H13.11	turquoise	0.830524236
ENSG00000256443.1	RP11-794G24.1	turquoise	0.730038156
ENSG00000267127.2	RP11-795F19.5	turquoise	0.625052188
ENSG00000258512.1	RP11-796G6.2	turquoise	0.778335899
ENSG00000269951.1	RP11-797A18.6	turquoise	0.714778939
ENSG00000213640.3	RP11-797H7.3	turquoise	0.770284912
ENSG00000189316.3	RP11-797H7.5	turquoise	0.781786197
ENSG00000253520.1	RP11-798K23.5	grey	0.499390472
ENSG00000259347.1	RP11-798K3.2	grey	0.290544088
ENSG00000272870.1	RP11-798M19.6	blue	0.784525399
ENSG00000265750.1	RP11-799B12.4	brown	0.619212895
ENSG00000272525.1	RP11-79P5.9	turquoise	0.821505693
ENSG00000237950.1	RP11-7O11.3	turquoise	0.657766752
ENSG00000260401.1	RP11-800A3.4	grey	0.250944447
ENSG00000255031.1	RP11-802E16.3	blue	0.519595435
ENSG00000272182.1	RP11-802O23.3	brown	0.782935393
ENSG00000272690.1	RP11-803B1.8	turquoise	0.691163979
ENSG00000273375.1	RP11-803P9.1	turquoise	0.626980589
ENSG00000249106.1	RP11-806K15.1	grey	0.186671944
ENSG00000266456.1	RP11-806L2.2	brown	0.796534377
ENSG00000253671.1	RP11-806O11.1	green	0.5710938
ENSG00000225140.1	RP11-809C18.3	brown	0.616193893
ENSG00000272992.1	RP11-809C18.4	brown	0.543348904
ENSG00000258102.1	RP11-809C9.2	turquoise	0.630893286
ENSG00000259222.1	RP11-809H16.5	turquoise	0.515330686
ENSG00000256148.1	RP11-809N8.5	grey	-0.309320701
ENSG00000273493.1	RP11-80H18.4	turquoise	0.442181576
ENSG00000241679.2	RP11-80H8.4	turquoise	0.757480765
ENSG00000270581.1	RP11-811P12.3	turquoise	0.797218697
ENSG00000272862.1	RP11-814H16.2	turquoise	0.464663185
ENSG00000250007.2	RP11-814P5.1	turquoise	0.782302724
ENSG00000260591.1	RP11-817O13.6	yellow	0.683618374
ENSG00000260274.1	RP11-817O13.8	turquoise	0.845559769
ENSG00000261098.1	RP11-819C21.1	turquoise	0.907580079
ENSG00000262165.1	RP11-81A22.5	grey	0.235744323

ENSG00000266050.2	RP11-822E23.6	turquoise	0.893046638
ENSG00000248469.1	RP11-826N14.2	turquoise	0.705997041
ENSG00000258525.1	RP11-829H16.3	turquoise	0.806902853
ENSG00000224945.1	RP11-82L18.2	turquoise	0.573741719
ENSG00000231632.1	RP11-82L18.4	turquoise	0.777472652
ENSG00000250742.1	RP11-834C11.4	magenta	0.852980288
ENSG00000255129.1	RP11-839D17.3	turquoise	0.755419455
ENSG00000273247.1	RP11-83A24.2	turquoise	0.84178569
ENSG00000272158.1	RP11-840I19.5	grey	0.288678173
ENSG00000265743.1	RP11-848P1.3	magenta	0.568498968
ENSG00000264107.1	RP11-848P1.5	red	0.625090974
ENSG00000264148.1	RP11-848P1.7	red	0.607956774
ENSG00000266865.2	RP11-848P1.9	blue	0.869727728
ENSG00000269928.1	RP11-849F2.8	grey	0.812880545
ENSG00000269947.1	RP11-849F2.9	turquoise	0.578660392
ENSG00000251143.1	RP11-849H4.4	yellow	0.824293361
ENSG00000263146.2	RP11-849I19.1	green	0.634170965
ENSG00000271359.1	RP11-84C13.1	turquoise	0.763293378
ENSG00000270720.1	RP11-84C13.2	turquoise	0.576785737
ENSG00000256124.1	RP11-84E24.3	green	0.622749312
ENSG00000260475.1	RP11-85A1.3	turquoise	0.609035104
ENSG00000244128.1	RP11-85M11.2	turquoise	0.754833855
ENSG00000267108.1	RP11-861E21.1	yellow	0.58516236
ENSG00000233170.3	RP11-863K10.4	turquoise	0.816344765
ENSG00000257057.1	RP11-867G2.2	turquoise	0.707077531
ENSG00000254855.1	RP11-867G23.1	yellow	0.650795881
ENSG00000254510.1	RP11-867G23.10	turquoise	0.496400253
ENSG00000255468.2	RP11-867G23.8	turquoise	0.822189864
ENSG00000269290.1	RP11-869B15.1	turquoise	0.84983511
ENSG00000235565.1	RP11-86H7.7	black	0.614185623
ENSG00000263013.1	RP11-876N24.5	blue	0.561560132
ENSG00000233961.1	RP11-87H9.2	turquoise	0.788405398
ENSG00000271976.1	RP11-884K10.7	turquoise	0.890798473
ENSG00000270059.1	RP11-88H12.2	brown	0.684823891
ENSG00000231437.3	RP11-88H9.2	turquoise	0.846926489
ENSG00000266413.2	RP11-88L24.4	turquoise	0.726043311
ENSG00000255455.2	RP11-890B15.3	turquoise	0.930043574
ENSG00000253730.1	RP11-893F2.13	turquoise	0.567501391
ENSG00000261959.1	RP11-893F2.14	turquoise	0.587018039
ENSG00000246451.2	RP11-894P9.1	yellow	0.915859461
ENSG00000259658.3	RP11-89K11.1	grey	0.406864463
ENSG00000259439.1	RP11-89K21.1	turquoise	0.771463142
ENSG00000271417.1	RP11-909M7.3	turquoise	0.797075691
ENSG00000266441.1	RP11-91I8.3	turquoise	0.610468875
ENSG00000272293.1	RP11-91J19.3	grey	0.41345933
ENSG00000272005.1	RP11-91J19.4	turquoise	0.937913754
ENSG00000272832.1	RP11-91K8.5	grey	0.599282656
ENSG00000261586.2	RP11-923I11.6	turquoise	0.812748672

ENSG00000265982.1	RP11-927P21.4	grey	0.410717175
ENSG00000260088.1	RP11-92G12.3	grey	0.310505548
ENSG00000247728.2	RP11-932O9.7	turquoise	0.650618813
ENSG00000236869.1	RP11-944L7.4	turquoise	0.611274483
ENSG00000253877.1	RP11-946L20.2	brown	0.796103969
ENSG00000250286.1	RP11-94C24.8	yellow	0.594225083
ENSG00000258646.1	RP11-950C14.3	yellow	0.774999439
ENSG00000261710.1	RP11-953B20.1	brown	0.790701738
ENSG00000183458.9	RP11-958N24.1	turquoise	0.866666705
ENSG00000269973.1	RP11-95D17.1	turquoise	0.76084825
ENSG00000267651.1	RP11-95O2.1	turquoise	0.772864941
ENSG00000267707.1	RP11-95O2.5	turquoise	0.776808401
ENSG00000262468.1	RP11-95P2.1	pink	0.78744902
ENSG00000257126.1	RP11-966I7.1	turquoise	0.761000394
ENSG00000261011.1	RP11-96C23.11	blue	0.553650878
ENSG00000269986.1	RP11-96H17.3	turquoise	0.50615607
ENSG00000257261.1	RP11-96H19.1	grey	0.344235636
ENSG00000267249.1	RP11-973H7.3	turquoise	0.811546477
ENSG00000257303.1	RP11-977G19.11	turquoise	0.840262185
ENSG00000257740.1	RP11-977G19.12	turquoise	0.835054411
ENSG00000258260.1	RP11-977G19.14	turquoise	0.667738791
ENSG00000258199.1	RP11-977G19.5	grey	0.685961986
ENSG00000271870.1	RP11-97C16.1	turquoise	0.848011791
ENSG00000173867.8	RP11-97O12.7	grey	0.299447741
ENSG00000253641.1	RP11-981G7.2	turquoise	0.733334562
ENSG00000272505.1	RP11-981G7.6	turquoise	0.836456069
ENSG00000258302.1	RP11-981P6.1	turquoise	0.80700891
ENSG00000256050.2	RP11-982M15.6	turquoise	0.784009514
ENSG00000257337.2	RP11-983P16.4	brown	0.743346921
ENSG00000269621.1	RP11-98D18.15	turquoise	0.756120628
ENSG00000269489.1	RP11-98D18.17	turquoise	0.746868207
ENSG00000249602.1	RP11-98D18.3	turquoise	0.77750018
ENSG00000203288.3	RP11-98D18.9	turquoise	0.823144685
ENSG00000228506.1	RP11-98I9.4	turquoise	0.732438077
ENSG00000257176.1	RP11-996F15.2	turquoise	0.789057864
ENSG00000259969.1	RP11-999E24.3	turquoise	0.63025519
ENSG00000250241.1	RP11-9G1.3	turquoise	0.873767866
ENSG00000273123.1	RP11-9N20.3	green	0.539228393
ENSG00000262049.1	RP13-1032I1.7	blue	0.684282761
ENSG00000265298.1	RP13-104F24.3	yellow	0.946637738
ENSG00000223343.1	RP13-131K19.2	pink	0.751504935
ENSG00000272434.1	RP13-131K19.6	grey	0.349118582
ENSG00000232903.2	RP13-137A17.4	greenyellow	0.743035375
ENSG00000260011.2	RP13-20L14.1	grey	0.694109331
ENSG00000228906.1	RP13-216E22.4	turquoise	0.605601654
ENSG00000269962.1	RP13-238F13.5	turquoise	0.591028598
ENSG00000234969.1	RP13-33H18.1	turquoise	0.671754201
ENSG00000270223.1	RP13-36G14.4	blue	0.633752221

ENSG00000226465.1	RP13-401N8.1	turquoise	0.588250102
ENSG00000261496.1	RP13-514E23.1	turquoise	0.92620583
ENSG00000273389.1	RP13-514E23.2	grey	0.325251913
ENSG00000260563.2	RP13-516M14.1	yellow	0.834529124
ENSG00000253716.1	RP13-582O9.5	turquoise	0.814427872
ENSG00000272172.1	RP13-582O9.7	grey	0.512580877
ENSG00000264569.1	RP13-650J16.1	grey	0.302315535
ENSG00000254872.2	RP13-870H17.3	turquoise	0.676000265
ENSG00000225460.1	RP13-93L13.1	brown	0.610814145
ENSG00000102218.5	RP2	blue	0.836469106
ENSG00000232645.1	RP3-322G13.5	brown	0.542551926
ENSG00000227954.2	RP3-323P13.2	turquoise	0.567932173
ENSG00000101898.5	RP3-324O17.4	turquoise	0.7922777
ENSG00000272294.1	RP3-326L13.3	turquoise	0.75500917
ENSG00000227285.1	RP3-327A19.5	turquoise	0.86683783
ENSG00000245261.1	RP3-330M21.5	yellow	0.847981601
ENSG00000271754.1	RP3-337H4.9	turquoise	0.607512923
ENSG00000233250.1	RP3-339A18.6	turquoise	0.587932093
ENSG00000228477.1	RP3-342P20.2	blue	0.61356299
ENSG00000271430.1	RP3-368A4.5	turquoise	0.79956502
ENSG00000271533.1	RP3-368A4.6	turquoise	0.835719187
ENSG00000234084.1	RP3-388E23.2	turquoise	0.749286161
ENSG00000218631.1	RP3-395C13.1	turquoise	0.502042978
ENSG00000224387.1	RP3-395M20.2	turquoise	0.594202334
ENSG00000229393.1	RP3-395M20.3	turquoise	0.649616419
ENSG00000228839.1	RP3-400N23.6	brown	0.561149173
ENSG00000273188.1	RP3-402G11.25	turquoise	0.690992113
ENSG00000273253.1	RP3-402G11.26	turquoise	0.689890203
ENSG00000272836.1	RP3-402G11.27	yellow	0.845876164
ENSG00000273137.1	RP3-402G11.28	yellow	0.811013492
ENSG00000260418.1	RP3-406A7.7	brown	0.60197838
ENSG00000273387.1	RP3-412A9.16	red	0.599236945
ENSG00000258603.1	RP3-414A15.10	turquoise	0.591834049
ENSG00000270031.1	RP3-426I6.6	turquoise	0.477345578
ENSG00000272841.1	RP3-428L16.2	turquoise	0.830822075
ENSG00000244627.1	RP3-449O17.1	yellow	0.868756526
ENSG00000257877.1	RP3-462E2.3	green	0.730924419
ENSG00000270018.1	RP3-462E2.5	turquoise	0.828090724
ENSG00000272432.1	RP3-465N24.6	turquoise	0.653191832
ENSG00000270638.1	RP3-466P17.1	turquoise	0.576321898
ENSG00000236266.1	RP3-467L1.4	grey	0.477382812
ENSG00000269925.1	RP3-467L1.6	turquoise	0.799304683
ENSG00000260000.2	RP3-467N11.1	turquoise	0.882444657
ENSG00000231113.2	RP3-475N16.1	yellow	0.817215492
ENSG00000268340.1	RP3-477O4.16	turquoise	0.552488831
ENSG00000230149.2	RP3-508I15.19	blue	0.694237558
ENSG00000273096.1	RP3-508I15.20	brown	0.578142298
ENSG00000272669.1	RP3-508I15.21	yellow	0.730000784

ENSG00000228274.3	RP3-508I15.9	turquoise	0.865116557
ENSG00000224167.1	RP3-522D1.1	turquoise	0.510577486
ENSG00000271218.1	RP3-523E19.2	blue	0.627720495
ENSG00000230910.2	RP3-525N10.2	yellow	0.898930626
ENSG00000235189.1	RP4-537K23.4	brown	0.720113048
ENSG00000273350.1	RP4-539M6.20	grey	0.556072334
ENSG00000272689.1	RP4-539M6.21	grey	0.45704164
ENSG00000261101.1	RP4-545K15.5	turquoise	0.677957797
ENSG00000261786.1	RP4-555D20.2	turquoise	0.820391425
ENSG00000272121.1	RP4-555D20.4	turquoise	0.801375093
ENSG00000260464.1	RP4-561L24.3	turquoise	0.877551202
ENSG00000203650.3	RP4-562J12.2	brown	0.736956385
ENSG00000273044.1	RP4-569D19.8	midnightblue	0.772027904
ENSG00000225938.1	RP4-575N6.4	green	0.715446298
ENSG00000229291.1	RP4-580N22.2	grey	0.378188466
ENSG00000240449.1	RP4-584D14.5	turquoise	0.840200983
ENSG00000260938.1	RP4-593C16.3	turquoise	0.808993538
ENSG00000273416.1	RP4-597N16.4	turquoise	0.520481245
ENSG00000272368.1	RP4-605O3.4	brown	0.550053226
ENSG00000272782.1	RP4-607J23.2	turquoise	0.642153381
ENSG00000225490.1	RP4-610C12.3	grey	0.316251662
ENSG00000205611.3	RP4-610C12.4	turquoise	0.641779184
ENSG00000261737.1	RP4-612B15.3	turquoise	0.843743801
ENSG00000230084.1	RP4-613B23.1	brown	0.776619802
ENSG00000269202.1	RP4-614O4.12	blue	0.49348656
ENSG00000273487.1	RP4-621B10.8	turquoise	0.707422723
ENSG00000224066.1	RP4-622L5.7	yellow	0.807754318
ENSG00000230337.1	RP4-635E18.6	turquoise	0.667229135
ENSG00000226849.1	RP4-635E18.7	yellow	0.913796605
ENSG00000271895.1	RP4-635E18.8	yellow	0.870114503
ENSG00000235501.1	RP4-639F20.1	black	0.507272308
ENSG00000258944.1	RP4-647C14.3	green	0.563631015
ENSG00000266993.1	RP4-657D16.3	blue	0.743897845
ENSG00000233593.2	RP4-665J23.1	grey	0.339100844
ENSG00000232710.1	RP4-669P10.16	turquoise	0.65138875
ENSG00000227370.1	RP4-669P10.19	turquoise	0.740363661
ENSG00000273483.1	RP4-671G15.2	grey	0.677673817
ENSG00000232528.3	RP4-673D20.3	green	0.783615637
ENSG00000272768.1	RP4-673M15.1	turquoise	0.835726607
ENSG00000255542.1	RP4-683L5.1	grey	0.113759565
ENSG00000213742.2	RP4-694B14.5	turquoise	0.840844325
ENSG00000229635.1	RP4-713B5.2	turquoise	0.722649432
ENSG00000261254.1	RP4-714D9.5	turquoise	0.870577841
ENSG00000223745.3	RP4-717I23.3	yellow	0.82988699
ENSG00000238194.1	RP4-719C8.1	brown	0.698409228
ENSG00000239636.1	RP4-728D4.2	turquoise	0.770588472
ENSG00000232450.1	RP4-730K3.3	pink	0.769070493
ENSG00000241720.2	RP4-735C1.4	turquoise	0.789558065

ENSG00000230725.1	RP4-738P15.1	turquoise	0.766136072
ENSG00000272145.1	RP4-739H11.4	turquoise	0.846866802
ENSG00000269896.1	RP4-740C4.6	pink	0.781590619
ENSG00000272420.1	RP4-740C4.7	turquoise	0.732410961
ENSG00000272715.1	RP4-753F5.1	turquoise	0.508903529
ENSG00000235513.1	RP4-756G23.5	blue	0.777313348
ENSG00000226824.2	RP4-756H11.3	grey	0.265011283
ENSG00000225285.1	RP4-758J18.10	grey	0.38969341
ENSG00000272455.1	RP4-758J18.13	yellow	0.874660152
ENSG00000224870.3	RP4-758J18.2	brown	0.801547045
ENSG00000225905.1	RP4-758J18.7	brown	0.66276732
ENSG00000272091.1	RP4-758J24.5	turquoise	0.918958625
ENSG00000269892.1	RP4-761J14.10	grey	0.344277717
ENSG00000213058.3	RP4-765C7.2	grey	0.105516821
ENSG00000271147.3	RP4-769N13.6	turquoise	0.90764645
ENSG00000258634.2	RP4-773N10.4	blue	0.798315512
ENSG00000237096.1	RP4-782G3.1	greenyellow	0.585674791
ENSG00000271914.1	RP4-789D17.5	turquoise	0.701914529
ENSG00000254539.1	RP4-791M13.3	yellow	0.851896221
ENSG00000255148.2	RP4-791M13.4	turquoise	0.720384413
ENSG00000232453.1	RP4-794H19.1	green	0.536419417
ENSG00000234807.4	RP4-794H19.2	brown	0.633847761
ENSG00000226029.1	RP4-798A10.2	yellow	0.878791882
ENSG00000261135.1	RP4-798A10.7	turquoise	0.536227489
ENSG00000244560.2	RP4-800G7.2	yellow	0.919707283
ENSG00000226308.1	RP4-813D12.3	grey	0.270410343
ENSG00000273081.1	RP4-813F11.4	turquoise	0.777991373
ENSG00000231999.2	RP5-1007M22.2	brown	0.793138654
ENSG00000259065.1	RP5-1021I20.1	grey	0.431418381
ENSG00000226754.1	RP5-1024G6.5	blue	0.662649315
ENSG00000270172.1	RP5-1024G6.8	turquoise	0.745014576
ENSG00000222044.1	RP5-1039K5.16	brown	0.736578887
ENSG00000272582.1	RP5-1039K5.17	grey	0.547049453
ENSG00000261662.1	RP5-1042I8.7	green	0.63411467
ENSG00000262227.1	RP5-1050D4.5	turquoise	0.732898452
ENSG00000261326.2	RP5-1057J7.6	yellow	0.881499836
ENSG00000250770.2	RP5-1063M23.1	turquoise	0.743843905
ENSG00000273382.1	RP5-1065J22.8	grey	0.365621146
ENSG00000226920.1	RP5-1068B5.3	turquoise	0.612600579
ENSG00000273148.1	RP5-1068E13.7	yellow	0.845306074
ENSG00000273373.1	RP5-1074L1.4	yellow	0.894163393
ENSG00000260257.1	RP5-1085F17.3	yellow	0.539375726
ENSG00000270605.1	RP5-1092A3.4	turquoise	0.52955117
ENSG00000225377.1	RP5-1103G7.4	blue	0.789355063
ENSG00000227188.1	RP5-1104E15.6	turquoise	0.686949988
ENSG00000232022.2	RP5-1109J22.1	turquoise	0.585056274
ENSG00000230177.1	RP5-1112D6.4	turquoise	0.798273198
ENSG00000272356.1	RP5-1112D6.8	turquoise	0.823818223

ENSG00000261675.1	RP5-1119A7.17	turquoise	0.87806866
ENSG00000237686.2	RP5-1120P11.1	turquoise	0.802473208
ENSG00000273314.1	RP5-1136G13.2	turquoise	0.77599478
ENSG00000224888.3	RP5-1142A6.2	turquoise	0.715304876
ENSG00000260121.1	RP5-1142A6.9	blue	0.798983115
ENSG00000272894.1	RP5-1159O4.1	yellow	0.800421119
ENSG00000272732.1	RP5-1159O4.2	yellow	0.684234376
ENSG00000228703.1	RP5-1160K1.6	turquoise	0.724296989
ENSG00000184068.2	RP5-821D11.7	blue	0.82096162
ENSG00000236358.1	RP5-827C21.2	turquoise	0.713195958
ENSG00000231663.1	RP5-827C21.4	turquoise	0.819467829
ENSG00000273367.1	RP5-827C21.6	turquoise	0.729135181
ENSG00000224113.2	RP5-837J1.2	turquoise	0.8405664
ENSG00000243961.2	RP5-839B4.8	turquoise	0.568307435
ENSG00000271782.1	RP5-850O15.4	turquoise	0.651170838
ENSG00000272240.1	RP5-855D21.1	yellow	0.790778986
ENSG00000273402.1	RP5-855D21.2	yellow	0.626334134
ENSG00000272812.1	RP5-855D21.3	yellow	0.778838431
ENSG00000198744.5	RP5-857K21.11	grey	-0.260458713
ENSG00000229344.1	RP5-857K21.7	grey	-0.217354669
ENSG00000228436.2	RP5-864K19.4	turquoise	0.749492136
ENSG00000197062.7	RP5-874C20.3	turquoise	0.891468783
ENSG00000260793.2	RP5-882C2.2	turquoise	0.691043309
ENSG00000226499.1	RP5-882O7.1	grey	0.337486505
ENSG00000224592.1	RP5-884C9.2	turquoise	0.851745799
ENSG00000228084.1	RP5-884G6.2	turquoise	0.836509606
ENSG00000236810.1	RP5-886K2.3	turquoise	0.827822592
ENSG00000240731.1	RP5-890O3.9	yellow	0.917553326
ENSG00000244701.1	RP5-894A10.2	brown	0.671074474
ENSG00000270157.1	RP5-894A10.6	brown	0.731614347
ENSG00000230415.1	RP5-902P8.10	yellow	0.874798436
ENSG00000260426.1	RP5-912I13.1	grey	0.261827093
ENSG00000259456.2	RP5-914P20.5	turquoise	0.863976638
ENSG00000270081.1	RP5-935K16.1	turquoise	0.876445945
ENSG00000255966.1	RP5-940J5.3	turquoise	0.562873903
ENSG00000272931.1	RP5-943J3.2	turquoise	0.629241202
ENSG00000230839.1	RP5-968J1.1	green	0.630665465
ENSG00000259716.1	RP5-977B1.11	grey	0.008541088
ENSG00000229591.1	RP5-981O7.2	turquoise	0.758095674
ENSG00000259901.1	RP5-991G20.4	pink	0.836722916
ENSG00000234917.1	RP5-994D16.3	turquoise	0.804774272
ENSG00000228452.1	RP5-994D16.9	turquoise	0.793405054
ENSG00000230163.1	RP5-997D16.2	turquoise	0.745522761
ENSG00000241990.1	RP6-109B7.3	turquoise	0.749098487
ENSG00000235159.1	RP6-109B7.4	turquoise	0.61032945
ENSG00000236064.1	RP6-191P20.4	turquoise	0.876206495
ENSG00000260118.1	RP6-201G10.2	turquoise	0.490528332
ENSG00000223804.1	RP6-206I17.1	red	0.695720214

ENSG00000237863.2	RP6-24A23.3	greenyellow	0.655822445
ENSG00000273131.1	RP6-42F4.1	yellow	0.878348684
ENSG00000259153.1	RP6-65G23.3	turquoise	0.91899827
ENSG00000272824.1	RP6-74O6.6	turquoise	0.450422403
ENSG00000269945.1	RP6-91H8.5	turquoise	0.701171375
ENSG00000270069.1	RP6-99M1.2	turquoise	0.829861731
ENSG00000164610.4	RP9	turquoise	0.81275718
ENSG00000205763.9	RP9P	turquoise	0.716922766
ENSG00000132383.7	RPA1	turquoise	0.93614483
ENSG00000117748.5	RPA2	turquoise	0.91883936
ENSG00000106399.7	RPA3	turquoise	0.931887723
ENSG00000219545.5	RPA3-AS1	turquoise	0.930536008
ENSG00000129197.10	RPAIN	turquoise	0.981585572
ENSG00000103932.7	RPAP1	turquoise	0.935025788
ENSG00000122484.8	RPAP2	turquoise	0.959849398
ENSG00000005175.5	RPAP3	turquoise	0.914412833
ENSG00000197713.10	RPE	turquoise	0.954594797
ENSG00000117133.6	RPF1	turquoise	0.947057484
ENSG00000197498.8	RPF2	turquoise	0.922486229
ENSG00000156313.8	RPGR	pink	0.80479885
ENSG00000103494.8	RPGRIP1L	turquoise	0.915744577
ENSG00000089169.10	RPH3A	turquoise	0.780632376
ENSG00000181031.11	RPH3AL	grey	0.465656963
ENSG00000153574.8	RPIA	turquoise	0.863197657
ENSG00000147403.12	RPL10	red	0.940213997
ENSG00000198755.6	RPL10A	red	0.918657338
ENSG00000267119.1	RPL10P15	pink	0.528231906
ENSG00000230734.1	RPL10P3	grey	0.200042621
ENSG00000142676.8	RPL11	red	0.924472317
ENSG00000197958.8	RPL12	red	0.907920632
ENSG00000167526.9	RPL13	red	0.90106343
ENSG00000142541.12	RPL13A	red	0.942851553
ENSG00000244053.1	RPL13AP2	turquoise	-0.424804673
ENSG00000136149.6	RPL13AP25	red	0.579006918
ENSG00000213885.3	RPL13AP7	grey	0.354050847
ENSG00000215030.4	RPL13P12	grey	0.101697703
ENSG00000240370.2	RPL13P5	blue	0.818931909
ENSG00000188846.9	RPL14	red	0.935118552
ENSG00000174748.14	RPL15	red	0.903070928
ENSG00000265681.2	RPL17	red	0.910605562
ENSG00000228331.2	RPL17P43	turquoise	0.713658134
ENSG00000213700.3	RPL17P50	cyan	0.564819175
ENSG00000063177.8	RPL18	red	0.884928499
ENSG00000105640.8	RPL18A	red	0.922508871
ENSG00000108298.5	RPL19	red	0.956117007
ENSG00000122026.6	RPL21	red	0.868213063
ENSG00000220749.3	RPL21P28	red	0.633144058
ENSG00000213860.3	RPL21P75	red	0.448165623

ENSG00000116251.5	RPL22	red	0.918538974
ENSG00000163584.13	RPL22L1	turquoise	0.716811672
ENSG00000125691.8	RPL23	red	0.930175761
ENSG00000198242.9	RPL23A	red	0.926783131
ENSG00000239257.1	RPL23AP1	turquoise	0.61960502
ENSG00000223508.5	RPL23AP53	turquoise	0.829131208
ENSG00000240356.2	RPL23AP7	blue	0.65406842
ENSG00000213753.6	RPL23AP79	turquoise	0.931805131
ENSG00000213035.4	RPL23AP80	turquoise	0.458026479
ENSG00000176054.6	RPL23P2	yellow	0.860549444
ENSG00000114391.8	RPL24	red	0.932770313
ENSG00000235065.1	RPL24P2	red	0.655514214
ENSG00000161970.8	RPL26	red	0.905061621
ENSG00000037241.3	RPL26L1	turquoise	0.944597066
ENSG00000131469.8	RPL27	red	0.948583693
ENSG00000166441.8	RPL27A	red	0.946037468
ENSG00000108107.8	RPL28	red	0.889487111
ENSG00000162244.6	RPL29	red	0.933948629
ENSG00000224858.4	RPL29P11	grey	0.223172166
ENSG00000100316.11	RPL3	red	0.934550604
ENSG00000156482.6	RPL30	red	0.904715768
ENSG00000071082.6	RPL31	red	0.930135821
ENSG00000229865.1	RPL31P54	grey	0.098293575
ENSG00000144713.8	RPL32	red	0.927833138
ENSG00000251474.2	RPL32P3	yellow	0.903196262
ENSG00000109475.12	RPL34	red	0.912053029
ENSG00000136942.10	RPL35	red	0.86512666
ENSG00000182899.10	RPL35A	red	0.948144805
ENSG00000220583.1	RPL35P2	blue	0.499562927
ENSG00000130255.8	RPL36	red	0.875701795
ENSG00000241343.5	RPL36A	red	0.927348943
ENSG00000165502.6	RPL36AL	red	0.932640874
ENSG00000241362.2	RPL36AP43	turquoise	0.704419968
ENSG00000145592.9	RPL37	red	0.863570229
ENSG00000197756.5	RPL37A	red	0.910502623
ENSG00000172809.8	RPL38	red	0.922925503
ENSG00000198918.7	RPL39	red	0.893382838
ENSG00000163923.5	RPL39L	turquoise	0.860629968
ENSG00000226580.1	RPL39P40	turquoise	0.482214884
ENSG00000174444.10	RPL4	red	0.926173618
ENSG00000229117.4	RPL41	red	0.89823371
ENSG00000227063.4	RPL41P1	red	0.46082539
ENSG00000122406.8	RPL5	red	0.923917754
ENSG00000243431.1	RPL5P30	pink	0.815068876
ENSG00000234009.1	RPL5P34	turquoise	0.644392537
ENSG00000229994.1	RPL5P4	turquoise	0.63397405
ENSG00000089009.11	RPL6	red	0.931563301
ENSG00000147604.9	RPL7	red	0.896352896

ENSG00000148303.12	RPL7A	red	0.949173515
ENSG00000146223.10	RPL7L1	turquoise	0.977395303
ENSG00000161016.11	RPL8	red	0.940871804
ENSG00000163682.11	RPL9	red	0.879941779
ENSG00000267355.2	RPL9P29	brown	0.606946873
ENSG00000237550.4	RPL9P9	red	0.617556053
ENSG00000089157.11	RPLP0	red	0.878598061
ENSG00000137818.7	RPLP1	red	0.923094064
ENSG00000177600.4	RPLP2	red	0.945022519
ENSG00000163902.7	RPN1	turquoise	0.910261613
ENSG00000118705.12	RPN2	turquoise	0.881741908
ENSG00000163684.7	RPP14	turquoise	0.972915957
ENSG00000241370.1	RPP21	turquoise	0.892731124
ENSG00000178718.5	RPP25	turquoise	0.826925098
ENSG00000164967.5	RPP25L	turquoise	0.854767359
ENSG00000148688.9	RPP30	turquoise	0.941249227
ENSG00000152464.10	RPP38	turquoise	0.951083652
ENSG00000124787.9	RPP40	turquoise	0.920777558
ENSG00000259001.2	RPPH1	grey	0.228161197
ENSG00000141425.13	RPRD1A	turquoise	0.941542957
ENSG00000101413.7	RPRD1B	turquoise	0.92828033
ENSG00000163125.11	RPRD2	turquoise	0.970620266
ENSG00000177519.3	RPRM	greenyellow	0.622541703
ENSG00000179673.3	RPRML	turquoise	0.7674955
ENSG00000124614.9	RPS10	red	0.920047019
ENSG00000223396.2	RPS10P7	brown	0.765275649
ENSG00000142534.2	RPS11	red	0.935109529
ENSG00000243024.2	RPS11P6	red	0.696408724
ENSG00000112306.7	RPS12	red	0.866683827
ENSG00000240494.2	RPS12P28	blue	0.571665948
ENSG00000110700.2	RPS13	red	0.891916802
ENSG00000164587.7	RPS14	red	0.939938387
ENSG00000226396.1	RPS14P3	grey	0.538951129
ENSG00000115268.5	RPS15	red	0.912552579
ENSG00000134419.11	RPS15A	red	0.893952924
ENSG00000105193.4	RPS16	red	0.925110407
ENSG00000231500.2	RPS18	red	0.871213259
ENSG00000220848.4	RPS18P9	blue	0.778806061
ENSG00000105372.2	RPS19	red	0.873153953
ENSG00000187051.4	RPS19BP1	red	0.891862796
ENSG00000140988.11	RPS2	red	0.921398654
ENSG00000008988.5	RPS20	red	0.909290133
ENSG00000242085.1	RPS20P33	blue	0.649428001
ENSG00000171858.13	RPS21	red	0.918689019
ENSG00000186468.8	RPS23	red	0.889430849
ENSG00000138326.14	RPS24	red	0.909980619
ENSG00000118181.6	RPS25	red	0.93616315
ENSG00000197728.5	RPS26	red	0.638543259

ENSG00000177954.7	RPS27	red	0.894934881
ENSG00000143947.8	RPS27A	red	0.892781309
ENSG00000185088.8	RPS27L	turquoise	0.729519446
ENSG00000233927.4	RPS28	red	0.880302168
ENSG00000213741.4	RPS29	red	0.8757326
ENSG00000232818.2	RPS2P32	turquoise	0.721250315
ENSG00000189343.6	RPS2P46	turquoise	0.544248308
ENSG00000240342.2	RPS2P5	red	0.596124694
ENSG00000149273.10	RPS3	red	0.932150749
ENSG00000145425.5	RPS3A	red	0.883486463
ENSG00000242607.1	RPS3AP34	pink	0.823050018
ENSG00000198034.6	RPS4X	red	0.901253823
ENSG00000224892.2	RPS4XP16	pink	0.681737393
ENSG00000129824.11	RPS4Y1	grey	0.132198021
ENSG00000083845.4	RPS5	red	0.927683756
ENSG00000137154.8	RPS6	red	0.872517976
ENSG00000117676.9	RPS6KA1	magenta	0.84803828
ENSG00000071242.7	RPS6KA2	turquoise	0.943543888
ENSG00000177189.8	RPS6KA3	turquoise	0.920393749
ENSG00000162302.8	RPS6KA4	turquoise	0.928115512
ENSG00000100784.5	RPS6KA5	turquoise	0.913048131
ENSG00000072133.6	RPS6KA6	turquoise	0.82564082
ENSG00000108443.9	RPS6KB1	turquoise	0.924955663
ENSG00000175634.10	RPS6KB2	blue	0.837042857
ENSG00000136643.7	RPS6KC1	turquoise	0.922689458
ENSG00000198208.7	RPS6KL1	turquoise	0.899518716
ENSG00000171863.8	RPS7	red	0.949995667
ENSG00000142937.7	RPS8	red	0.935821862
ENSG00000170889.9	RPS9	red	0.908342989
ENSG00000168028.9	RPSA	red	0.907975162
ENSG00000205246.4	RPSAP58	turquoise	0.406302845
ENSG00000234618.1	RPSAP9	pink	0.79185495
ENSG00000141564.9	RPTOR	turquoise	0.925373059
ENSG00000007376.3	RPUSD1	turquoise	0.855059748
ENSG00000166133.13	RPUSD2	turquoise	0.939442093
ENSG00000156990.10	RPUSD3	turquoise	0.949175435
ENSG00000165526.4	RPUSD4	turquoise	0.96850144
ENSG00000144580.9	RQCD1	turquoise	0.963063328
ENSG00000166592.7	RRAD	grey	0.311791619
ENSG00000155876.4	RRAGA	turquoise	0.977590102
ENSG00000083750.8	RRAGB	turquoise	0.963248442
ENSG00000116954.7	RRAGC	turquoise	0.922100473
ENSG00000025039.10	RRAGD	turquoise	0.915203648
ENSG00000126458.3	RRAS	brown	0.530222965
ENSG00000133818.8	RRAS2	turquoise	0.849720273
ENSG00000125844.11	RRBP1	blue	0.759305656
ENSG00000124782.15	RREB1	green	0.718449838
ENSG00000167325.10	RRM1	turquoise	0.91912196

ENSG00000048392.7	RRM2B	turquoise	0.837765769
ENSG00000085721.8	RRN3	turquoise	0.953446043
ENSG00000248124.3	RRN3P1	turquoise	0.897851678
ENSG00000103472.5	RRN3P2	blue	0.737282786
ENSG00000257122.1	RRN3P3	turquoise	0.826807978
ENSG00000143303.7	RRNAD1	blue	0.908412527
ENSG00000160214.8	RRP1	turquoise	0.923775883
ENSG00000052749.9	RRP12	turquoise	0.936016184
ENSG00000067533.5	RRP15	turquoise	0.877542738
ENSG00000160208.11	RRP1B	turquoise	0.966219908
ENSG00000124541.6	RRP36	turquoise	0.937064354
ENSG00000189306.6	RRP7A	turquoise	0.896259616
ENSG00000182841.8	RRP7B	yellow	0.90819964
ENSG00000132275.6	RRP8	turquoise	0.783198196
ENSG00000114767.6	RRP9	turquoise	0.919713456
ENSG00000179041.2	RRS1	turquoise	0.840055196
ENSG00000102104.8	RS1	turquoise	0.691637701
ENSG00000136444.5	RSAD1	turquoise	0.938319505
ENSG00000134321.7	RSAD2	turquoise	0.770253718
ENSG00000081019.9	RSBN1	turquoise	0.931949626
ENSG00000187257.10	RSBN1L	turquoise	0.945364812
ENSG00000214293.4	RSBN1L-AS1	turquoise	0.84006245
ENSG00000048649.9	RSF1	turquoise	0.944995743
ENSG00000132881.7	RSG1	turquoise	0.801802167
ENSG00000171490.8	RSL1D1	turquoise	0.954638282
ENSG00000137876.5	RSL24D1	turquoise	0.932845882
ENSG00000160188.5	RSPH1	pink	0.921578419
ENSG00000130363.7	RSPH3	pink	0.94736423
ENSG00000101282.4	RSPO4	turquoise	0.868012581
ENSG00000159579.9	RSPRY1	turquoise	0.937156741
ENSG00000174891.8	RSRC1	turquoise	0.945830917
ENSG00000111011.13	RSRC2	turquoise	0.947041767
ENSG00000148484.13	RSU1	turquoise	0.870321026
ENSG00000137996.8	RTCA	turquoise	0.962543635
ENSG00000100220.7	RTCB	turquoise	0.920264969
ENSG00000100218.7	RTDR1	turquoise	0.849708116
ENSG00000258366.3	RTEL1	yellow	0.929207019
ENSG00000137815.10	RTF1	turquoise	0.94808764
ENSG00000022277.8	RTFDC1	turquoise	0.889654048
ENSG00000114993.11	RTKN	brown	0.898299105
ENSG00000139970.12	RTN1	turquoise	0.948493479
ENSG00000125744.7	RTN2	turquoise	0.848788434
ENSG00000133318.9	RTN3	turquoise	0.977891049
ENSG00000115310.13	RTN4	turquoise	0.948730333
ENSG00000130347.8	RTN4IP1	turquoise	0.938522042
ENSG00000040608.9	RTN4R	turquoise	0.704900068
ENSG00000185924.6	RTN4RL1	turquoise	0.880680016
ENSG00000186907.3	RTN4RL2	grey	0.323156193

ENSG00000136514.2	RTP4	grey	0.375017488
ENSG00000176225.8	RTTN	turquoise	0.888514788
ENSG00000176783.10	RUFY1	blue	0.915393085
ENSG00000204130.8	RUFY2	turquoise	0.907179349
ENSG00000018189.8	RUFY3	turquoise	0.960366084
ENSG00000198863.3	RUNDC1	turquoise	0.95853658
ENSG00000108309.8	RUNDC3A	turquoise	0.878373451
ENSG00000105784.11	RUNDC3B	turquoise	0.915840328
ENSG00000159216.14	RUNX1	magenta	0.812168067
ENSG00000079102.12	RUNX1T1	turquoise	0.928011186
ENSG00000160753.11	RUSC1	turquoise	0.943510055
ENSG00000225855.2	RUSC1-AS1	yellow	0.9181086
ENSG00000198853.7	RUSC2	turquoise	0.959927982
ENSG00000175792.7	RUVBL1	pink	0.907692343
ENSG00000183207.8	RUVBL2	turquoise	0.919832232
ENSG00000111832.8	RWDD1	turquoise	0.823670905
ENSG00000013392.6	RWDD2A	turquoise	0.955758495
ENSG00000156253.2	RWDD2B	turquoise	0.913244376
ENSG00000122481.12	RWDD3	blue	0.831762453
ENSG00000182552.10	RWDD4	turquoise	0.858174714
ENSG00000171509.11	RXFP1	turquoise	0.652258475
ENSG00000186350.8	RXRA	green	0.846736729
ENSG00000204231.6	RXRB	turquoise	0.974623856
ENSG00000143171.8	RXRG	turquoise	0.804624619
ENSG00000163602.9	RYBP	turquoise	0.894394723
ENSG00000163785.8	RYK	blue	0.919906958
ENSG00000196218.7	RYR1	turquoise	0.767353288
ENSG00000198626.11	RYR2	turquoise	0.910057786
ENSG00000198838.7	RYR3	turquoise	0.821751151
ENSG00000160678.7	S100A1	grey	0.326702742
ENSG00000197747.4	S100A10	blue	0.660684393
ENSG00000163191.5	S100A11	magenta	0.837210697
ENSG00000163221.7	S100A12	black	0.50594018
ENSG00000189171.9	S100A13	green	0.567219419
ENSG00000189334.4	S100A14	grey	0.184121785
ENSG00000188643.6	S100A16	green	0.549604274
ENSG00000196154.7	S100A4	grey	0.336332045
ENSG00000197956.5	S100A6	black	0.674040175
ENSG00000143546.5	S100A8	black	0.610212925
ENSG00000163220.10	S100A9	magenta	0.530055715
ENSG00000160307.5	S100B	green	0.54090781
ENSG00000116497.13	S100PBP	turquoise	0.914640896
ENSG00000170989.8	S1PR1	green	0.862822935
ENSG00000213694.3	S1PR3	black	0.824931787
ENSG00000180739.12	S1PR5	brown	0.745965534
ENSG00000173432.6	SAA1	grey	0.136135029
ENSG00000166788.5	SAAL1	turquoise	0.910450526
ENSG00000168061.9	SAC3D1	turquoise	0.813978552

ENSG00000211456.6	SACM1L	turquoise	0.959347787
ENSG00000151835.9	SACS	turquoise	0.942235144
ENSG00000142230.7	SAE1	turquoise	0.950272997
ENSG00000160633.8	SAFB	turquoise	0.94024486
ENSG00000130254.7	SAFB2	turquoise	0.853947722
ENSG00000130561.12	SAG	turquoise	0.706658066
ENSG00000103449.7	SALL1	blue	0.843136608
ENSG00000165821.7	SALL2	blue	0.824274946
ENSG00000256463.4	SALL3	green	0.803997727
ENSG00000141858.7	SAMD1	brown	0.686860158
ENSG00000130590.9	SAMD10	turquoise	0.835894673
ENSG00000187634.6	SAMD11	turquoise	0.642533925
ENSG00000177570.9	SAMD12	turquoise	0.934413943
ENSG00000203943.4	SAMD13	turquoise	0.765629378
ENSG00000167100.10	SAMD14	turquoise	0.871774848
ENSG00000100583.4	SAMD15	pink	0.909475358
ENSG00000020577.9	SAMD4A	blue	0.5482487
ENSG00000179134.10	SAMD4B	blue	0.908234989
ENSG00000203727.3	SAMD5	midnightblue	0.867061336
ENSG00000156671.8	SAMD8	turquoise	0.919922178
ENSG00000177409.7	SAMD9L	green	0.647037132
ENSG00000101347.7	SAMHD1	magenta	0.778365695
ENSG00000100347.10	SAMM50	turquoise	0.941264683
ENSG00000155307.13	SAMSN1	magenta	0.901325752
ENSG00000136715.13	SAP130	turquoise	0.970114566
ENSG00000150459.8	SAP18	turquoise	0.796585405
ENSG00000205307.6	SAP25	grey	0.709348086
ENSG00000164105.3	SAP30	blue	0.865053666
ENSG00000161526.10	SAP30BP	turquoise	0.890721106
ENSG00000164576.7	SAP30L	blue	0.870677273
ENSG00000228727.4	SAPCD1	yellow	0.862463738
ENSG00000235663.1	SAPCD1-AS1	turquoise	0.543311296
ENSG00000186193.7	SAPCD2	brown	0.52014482
ENSG00000079332.10	SAR1A	turquoise	0.924311448
ENSG00000152700.9	SAR1B	turquoise	0.83898141
ENSG00000123453.12	SARDH	green	0.778296474
ENSG00000004139.9	SARM1	turquoise	0.927973722
ENSG00000205323.4	SARNP	turquoise	0.921184105
ENSG00000031698.8	SARS	turquoise	0.957867606
ENSG00000104835.10	SARS2	turquoise	0.915190858
ENSG00000175467.10	SART1	turquoise	0.87850755
ENSG00000075856.7	SART3	turquoise	0.971567754
ENSG00000111961.12	SASH1	green	0.871648726
ENSG00000122122.9	SASH3	magenta	0.95264744
ENSG00000156876.8	SASS6	turquoise	0.733464294
ENSG00000130066.12	SAT1	black	0.760215078
ENSG00000141504.7	SAT2	turquoise	0.889611833
ENSG00000182568.12	SATB1	turquoise	0.834392534

ENSG00000119042.12	SATB2	blue	0.620732669
ENSG00000184788.8	SATL1	turquoise	0.919818505
ENSG00000151748.10	SAV1	turquoise	0.844996248
ENSG00000112167.5	SAYSD1	turquoise	0.93351448
ENSG00000126524.5	SBDS	turquoise	0.912535345
ENSG00000225648.1	SBDSP1	turquoise	0.796673004
ENSG00000100241.16	SBF1	turquoise	0.933624836
ENSG00000133812.10	SBF2	turquoise	0.875714536
ENSG00000246273.2	SBF2-AS1	turquoise	0.907763248
ENSG00000188322.4	SBK1	turquoise	0.844310101
ENSG00000139697.7	SBNO1	turquoise	0.9569357
ENSG00000064932.11	SBNO2	black	0.887825717
ENSG00000269490.1	SBP1	turquoise	0.850170727
ENSG00000189001.6	SBSN	turquoise	0.699111529
ENSG00000109929.5	SC5D	turquoise	0.91807322
ENSG00000126461.10	SCAF1	turquoise	0.936722431
ENSG00000139218.13	SCAF11	turquoise	0.84578151
ENSG00000156304.10	SCAF4	turquoise	0.91569858
ENSG00000213079.5	SCAF8	turquoise	0.939963974
ENSG00000173611.13	SCAI	turquoise	0.933499033
ENSG00000085365.13	SCAMP1	turquoise	0.955202129
ENSG00000140497.12	SCAMP2	blue	0.817710501
ENSG00000116521.6	SCAMP3	turquoise	0.899907515
ENSG00000227500.5	SCAMP4	turquoise	0.869372195
ENSG00000198794.7	SCAMP5	turquoise	0.947850678
ENSG00000171222.6	SCAND1	red	0.921898436
ENSG00000176700.15	SCAND2P	turquoise	0.868631619
ENSG00000114650.14	SCAP	turquoise	0.951359384
ENSG00000140386.8	SCAPER	turquoise	0.877915575
ENSG00000168077.9	SCARA3	green	0.659162181
ENSG00000073060.11	SCARB1	midnightblue	0.926059311
ENSG00000138760.4	SCARB2	brown	0.894850183
ENSG00000074660.11	SCARF1	blue	0.697158113
ENSG00000244486.3	SCARF2	yellow	0.797839556
ENSG00000238795.1	SCARNA12	yellow	0.708182121
ENSG00000252481.1	SCARNA13	blue	0.573525421
ENSG00000251992.1	SCARNA17	grey	0.47364862
ENSG00000252139.1	SCARNA18	grey	0.267466188
ENSG00000270066.2	SCARNA2	grey	0.27929131
ENSG00000252010.1	SCARNA5	grey	0.167632151
ENSG00000238741.1	SCARNA7	grey	0.109378967
ENSG00000254911.2	SCARNA9	grey	0.481024837
ENSG00000143653.9	SCCPDH	turquoise	0.932419281
ENSG00000099194.5	SCD	brown	0.807154033
ENSG00000145284.7	SCD5	blue	0.8576158
ENSG00000092108.16	SCFD1	turquoise	0.968026737
ENSG00000184178.11	SCFD2	turquoise	0.961976764
ENSG00000171951.4	SCG2	turquoise	0.913938783

ENSG00000104112.4	SCG3	turquoise	0.753319292
ENSG00000166922.4	SCG5	turquoise	0.935597133
ENSG00000268751.1	SCGB1B2P	turquoise	0.620607223
ENSG00000124935.3	SCGB1D2	grey	0.252544593
ENSG00000151967.14	SCHIP1	blue	0.844019694
ENSG00000006747.10	SCIN	magenta	0.831881403
ENSG00000151466.7	SCLT1	turquoise	0.903574241
ENSG00000132330.12	SCLY	blue	0.879875003
ENSG00000010803.12	SCMH1	turquoise	0.948705925
ENSG00000047634.10	SCML1	blue	0.821041186
ENSG00000144285.11	SCN1A	turquoise	0.88885279
ENSG00000105711.6	SCN1B	turquoise	0.726286358
ENSG00000136531.9	SCN2A	turquoise	0.921634883
ENSG00000149575.5	SCN2B	turquoise	0.923062052
ENSG00000153253.11	SCN3A	turquoise	0.893093877
ENSG00000166257.4	SCN3B	turquoise	0.923975017
ENSG00000177098.4	SCN4B	turquoise	0.737163333
ENSG00000196876.9	SCN8A	turquoise	0.911858299
ENSG00000169432.10	SCN9A	turquoise	0.723445635
ENSG00000163156.7	SCNM1	turquoise	0.883636831
ENSG00000162572.15	SCNN1D	yellow	0.927878281
ENSG00000133028.6	SCO1	turquoise	0.935295137
ENSG00000130489.8	SCO2	pink	0.866217559
ENSG00000153130.13	SCOC	turquoise	0.935132313
ENSG00000116171.12	SCP2	blue	0.92288807
ENSG00000121064.8	SCPEP1	blue	0.889358874
ENSG00000164106.3	SCRG1	green	0.735847351
ENSG00000180900.12	SCRIB	blue	0.832598553
ENSG00000136193.12	SCRN1	turquoise	0.918842749
ENSG00000141295.9	SCRN2	turquoise	0.891364191
ENSG00000144306.9	SCRN3	turquoise	0.887773257
ENSG00000170616.9	SCRT1	turquoise	0.760089452
ENSG00000175356.8	SCUBE2	greenyellow	0.718446808
ENSG00000142186.12	SCYL1	turquoise	0.957271445
ENSG00000136021.13	SCYL2	turquoise	0.933771973
ENSG00000000457.9	SCYL3	turquoise	0.892712536
ENSG00000198301.7	SDAD1	turquoise	0.973038118
ENSG00000228451.3	SDAD1P1	turquoise	0.8095713
ENSG00000169439.7	SDC2	blue	0.808492581
ENSG00000162512.11	SDC3	turquoise	0.67674344
ENSG00000124145.5	SDC4	green	0.724696859
ENSG00000137575.7	SDCBP	turquoise	0.944816976
ENSG00000125775.10	SDCBP2	turquoise	0.860480733
ENSG00000234684.2	SDCBP2-AS1	turquoise	0.865395052
ENSG00000165689.12	SDCCAG3	turquoise	0.863260323
ENSG00000054282.11	SDCCAG8	turquoise	0.906143517
ENSG00000143751.9	SDE2	blue	0.878481193
ENSG00000132581.5	SDF2	turquoise	0.914208129

ENSG00000128228.4	SDF2L1	cyan	0.801097773
ENSG00000078808.12	SDF4	turquoise	0.86877779
ENSG00000073578.12	SDHA	turquoise	0.906155633
ENSG00000205138.2	SDHAF1	turquoise	0.9179191
ENSG00000167985.2	SDHAF2	turquoise	0.902718022
ENSG00000185485.10	SDHAP1	yellow	0.895449095
ENSG00000185986.10	SDHAP3	turquoise	0.910612431
ENSG00000117118.5	SDHB	turquoise	0.931202635
ENSG00000143252.10	SDHC	blue	0.893987823
ENSG00000204370.4	SDHD	blue	0.885294752
ENSG00000168497.4	SDPR	grey	0.332098002
ENSG00000170786.8	SDR16C5	turquoise	0.74570176
ENSG00000100445.12	SDR39U1	turquoise	0.925881326
ENSG00000135094.6	SDS	green	0.526053634
ENSG00000139410.10	SDSL	green	0.526885514
ENSG00000140612.9	SEC11A	blue	0.898466628
ENSG00000166562.4	SEC11C	turquoise	0.862171952
ENSG00000157020.13	SEC13	turquoise	0.934245962
ENSG00000129657.10	SEC14L1	turquoise	0.851605614
ENSG00000100003.13	SEC14L2	green	0.721625885
ENSG00000103184.7	SEC14L5	brown	0.846261224
ENSG00000214491.4	SEC14L6	green	0.739169489
ENSG00000148396.14	SEC16A	turquoise	0.953412378
ENSG00000120341.14	SEC16B	brown	0.747177889
ENSG00000121542.7	SEC22A	turquoise	0.948105573
ENSG00000223380.3	SEC22B	turquoise	0.906910875
ENSG00000093183.9	SEC22C	turquoise	0.941740215
ENSG00000100934.10	SEC23A	turquoise	0.959589469
ENSG00000101310.10	SEC23B	turquoise	0.898470891
ENSG00000107651.8	SEC23IP	turquoise	0.94769033
ENSG00000113615.8	SEC24A	turquoise	0.848631313
ENSG00000138802.7	SEC24B	turquoise	0.930364114
ENSG00000247950.2	SEC24B-AS1	turquoise	0.841004737
ENSG00000176986.10	SEC24C	turquoise	0.945875086
ENSG00000150961.10	SEC24D	black	0.835988548
ENSG00000138674.12	SEC31A	turquoise	0.945566869
ENSG00000075826.12	SEC31B	yellow	0.953713048
ENSG00000058262.5	SEC61A1	blue	0.889949802
ENSG00000065665.16	SEC61A2	turquoise	0.962120989
ENSG00000106803.5	SEC61B	turquoise	0.871267447
ENSG00000132432.9	SEC61G	turquoise	0.890721581
ENSG00000008952.12	SEC62	turquoise	0.861811169
ENSG00000025796.9	SEC63	turquoise	0.919542923
ENSG00000187742.10	SECISBP2	turquoise	0.979664733
ENSG00000138593.4	SECISBP2L	brown	0.873650009
ENSG00000085415.11	SEH1L	turquoise	0.971802229
ENSG00000071537.9	SEL1L	turquoise	0.938979963
ENSG00000091490.6	SEL1L3	green	0.755491366

ENSG00000143416.16	SELENBP1	green	0.79631597
ENSG00000113811.6	SELK	turquoise	0.783005127
ENSG00000188404.4	SELL	magenta	0.744179477
ENSG00000198832.6	SELM	red	0.906499945
ENSG00000073169.9	SELO	turquoise	0.827855695
ENSG00000110876.8	SELPLG	magenta	0.768481902
ENSG00000162377.4	SELRC1	turquoise	0.907470642
ENSG00000198843.8	SELT	turquoise	0.937187682
ENSG00000075213.6	SEMA3A	turquoise	0.838572146
ENSG00000012171.13	SEMA3B	brown	0.853946356
ENSG00000232352.1	SEMA3B-AS1	brown	0.617185436
ENSG00000075223.9	SEMA3C	greenyellow	0.683265847
ENSG00000153993.9	SEMA3D	turquoise	0.675868067
ENSG00000170381.8	SEMA3E	turquoise	0.569743319
ENSG00000001617.7	SEMA3F	blue	0.633089068
ENSG00000010319.2	SEMA3G	brown	0.51619981
ENSG00000196189.8	SEMA4A	turquoise	0.769187443
ENSG00000185033.10	SEMA4B	green	0.813229775
ENSG00000168758.6	SEMA4C	brown	0.900710368
ENSG00000187764.7	SEMA4D	brown	0.907886793
ENSG00000135622.8	SEMA4F	turquoise	0.901115622
ENSG00000095539.11	SEMA4G	turquoise	0.88073939
ENSG00000112902.7	SEMA5A	greenyellow	0.850414669
ENSG00000082684.10	SEMA5B	turquoise	0.793977907
ENSG00000092421.12	SEMA6A	brown	0.933433823
ENSG00000167680.11	SEMA6B	turquoise	0.87921428
ENSG00000143434.11	SEMA6C	turquoise	0.888797934
ENSG00000137872.11	SEMA6D	turquoise	0.854263954
ENSG00000138623.5	SEMA7A	brown	0.822647591
ENSG00000079387.9	SENP1	blue	0.914370625
ENSG00000163904.8	SENP2	turquoise	0.973554336
ENSG00000161956.8	SENP3	turquoise	0.965023322
ENSG00000119231.6	SENP5	turquoise	0.965057189
ENSG00000112701.13	SENP6	turquoise	0.911124698
ENSG00000138468.11	SENP7	turquoise	0.78422022
ENSG00000166192.10	SENP8	blue	0.847623351
ENSG00000086475.10	SEPHS1	blue	0.889113572
ENSG00000182722.5	SEPHS1P1	turquoise	0.822763516
ENSG00000213938.3	SEPHS1P6	turquoise	0.869623903
ENSG00000179918.15	SEPHS2	turquoise	0.930579174
ENSG00000162430.12	SEPN1	blue	0.858452959
ENSG00000250722.1	SEPP1	brown	0.863147082
ENSG00000109618.7	SEPSECS	turquoise	0.750376215
ENSG00000214765.4	SEPT7P2	turquoise	0.8754575
ENSG00000178980.10	SEPW1	red	0.923927385
ENSG00000122335.9	SERAC1	turquoise	0.946608696
ENSG00000142864.10	SERBP1	turquoise	0.897330047
ENSG00000242142.1	SERBP1P3	grey	0.034560928

ENSG00000140264.15	SERF2	brown	0.633032954
ENSG00000129158.6	SERGEF	turquoise	0.8870674
ENSG00000183569.13	SERHL2	yellow	0.813962167
ENSG00000111897.6	SERINC1	turquoise	0.926041323
ENSG00000132824.9	SERINC3	turquoise	0.936628765
ENSG00000164300.11	SERINC5	blue	0.803429512
ENSG00000120742.6	SERP1	turquoise	0.78005724
ENSG00000151778.6	SERP2	turquoise	0.900738382
ENSG00000197249.8	SERPINA1	magenta	0.839631846
ENSG00000196136.12	SERPINA3	black	0.76482385
ENSG00000021355.8	SERPINB1	black	0.869736817
ENSG00000124570.13	SERPINB6	blue	0.818921145
ENSG00000166401.9	SERPINB8	blue	0.740453678
ENSG00000170542.5	SERPINB9	blue	0.768909781
ENSG00000099937.6	SERPIND1	turquoise	0.670330985
ENSG00000106366.7	SERPINE1	black	0.646360666
ENSG00000135919.8	SERPINE2	green	0.851967398
ENSG00000132386.6	SERPINF1	magenta	0.691956035
ENSG00000167711.9	SERPINF2	grey	0.697517054
ENSG00000149131.11	SERPING1	black	0.829875554
ENSG00000149257.9	SERPINH1	black	0.635189709
ENSG00000163536.8	SERPINI1	turquoise	0.944008115
ENSG00000114204.10	SERPINI2	pink	0.837123446
ENSG00000197019.4	SERTAD1	black	0.768250086
ENSG00000179833.4	SERTAD2	turquoise	0.881663054
ENSG00000167565.8	SERTAD3	blue	0.65965409
ENSG00000082497.7	SERTAD4	turquoise	0.899239214
ENSG00000203706.4	SERTAD4-AS1	turquoise	0.78935198
ENSG00000180440.3	SERTM1	greenyellow	0.728908756
ENSG00000080546.9	SESN1	turquoise	0.929176025
ENSG00000130766.4	SESN2	cyan	0.809544253
ENSG00000149212.6	SESN3	turquoise	0.893484451
ENSG00000187231.9	SESTD1	brown	0.894737553
ENSG00000119335.12	SET	turquoise	0.86757116
ENSG00000152217.12	SETBP1	turquoise	0.874748578
ENSG00000099381.12	SETD1A	turquoise	0.904267889
ENSG00000139718.6	SETD1B	turquoise	0.93705681
ENSG00000181555.15	SETD2	turquoise	0.936903069
ENSG00000183576.8	SETD3	turquoise	0.8926401
ENSG00000185917.9	SETD4	turquoise	0.948033658
ENSG00000168137.11	SETD5	turquoise	0.900591727
ENSG00000206573.4	SETD5-AS1	turquoise	0.912981953
ENSG00000103037.7	SETD6	turquoise	0.940057075
ENSG00000145391.9	SETD7	blue	0.864696752
ENSG00000183955.8	SETD8	blue	0.935587977
ENSG00000155542.7	SETD9	blue	0.69182611
ENSG00000143379.8	SETDB1	turquoise	0.931645692
ENSG00000136169.12	SETDB2	turquoise	0.919560901

ENSG00000170364.8	SETMAR	turquoise	0.92078376
ENSG00000107290.9	SETX	turquoise	0.904823157
ENSG00000063015.15	SEZ6	turquoise	0.873009534
ENSG00000100095.14	SEZ6L	greenyellow	0.817010142
ENSG00000174938.10	SEZ6L2	turquoise	0.905190509
ENSG00000168066.16	SF1	turquoise	0.932949321
ENSG00000099995.14	SF3A1	turquoise	0.919628091
ENSG00000104897.5	SF3A2	turquoise	0.862111838
ENSG00000183431.7	SF3A3	turquoise	0.961062414
ENSG00000115524.11	SF3B1	turquoise	0.918417369
ENSG00000115128.6	SF3B14	turquoise	0.855763096
ENSG00000087365.10	SF3B2	turquoise	0.953697403
ENSG00000189091.8	SF3B3	turquoise	0.947460206
ENSG00000143368.9	SF3B4	turquoise	0.887337307
ENSG00000169976.5	SF3B5	red	0.966505773
ENSG00000198089.10	SFI1	yellow	0.925047198
ENSG00000163935.9	SFMBT1	blue	0.911210063
ENSG00000198879.7	SFMBT2	blue	0.883726265
ENSG00000175793.10	SFN	turquoise	0.586995871
ENSG00000116560.6	SFPQ	turquoise	0.916026291
ENSG00000156384.10	SFR1	blue	0.805608602
ENSG00000104332.7	SFRP1	brown	0.821977201
ENSG00000145423.4	SFRP2	pink	0.752269392
ENSG00000061936.5	SFSWAP	yellow	0.932431149
ENSG00000198818.5	SFT2D1	blue	0.901507403
ENSG00000213064.5	SFT2D2	turquoise	0.815826622
ENSG00000225383.2	SFTA1P	grey	0.10975128
ENSG00000229415.5	SFTA3	greenyellow	0.666395957
ENSG00000168484.8	SFTPC	brown	0.646036636
ENSG00000133661.11	SFTPD	turquoise	0.820666708
ENSG00000164466.8	SFXN1	turquoise	0.97167179
ENSG00000156398.8	SFXN2	turquoise	0.839343837
ENSG00000107819.9	SFXN3	turquoise	0.949735834
ENSG00000183605.12	SFXN4	turquoise	0.902671253
ENSG00000144040.8	SFXN5	green	0.758589172
ENSG00000108823.11	SGCA	grey	0.523707378
ENSG00000163069.8	SGCB	turquoise	0.894957402
ENSG00000170624.9	SGCD	turquoise	0.727937144
ENSG00000127990.11	SGCE	turquoise	0.941431268
ENSG00000118473.17	SGIP1	turquoise	0.946734325
ENSG00000118515.7	SGK1	brown	0.773317371
ENSG00000101049.10	SGK2	brown	0.924982996
ENSG00000182319.5	SGK223	turquoise	0.879047752
ENSG00000104205.8	SGK3	turquoise	0.876729264
ENSG00000167524.10	SGK494	turquoise	0.860976708
ENSG00000198964.9	SGMS1	turquoise	0.799557886
ENSG00000166224.12	SGPL1	blue	0.906343181
ENSG00000126821.7	SGPP1	turquoise	0.942198678

ENSG00000163082.9	SGPP2	turquoise	0.882873714
ENSG00000181523.8	SGSH	turquoise	0.866544297
ENSG00000167037.14	SGSM1	turquoise	0.871205247
ENSG00000141258.8	SGSM2	turquoise	0.897516514
ENSG00000100359.16	SGSM3	turquoise	0.949311294
ENSG00000104969.5	SGTA	blue	0.830759914
ENSG00000197860.5	SGTB	turquoise	0.925600808
ENSG00000178188.10	SH2B1	turquoise	0.937933406
ENSG00000160999.8	SH2B2	turquoise	0.539743038
ENSG00000111252.6	SH2B3	blue	0.841634126
ENSG00000095370.15	SH2D3C	blue	0.695683529
ENSG00000104611.7	SH2D4A	blue	0.677833132
ENSG00000189410.7	SH2D5	turquoise	0.873806955
ENSG00000185437.9	SH3BGR	turquoise	0.800109823
ENSG00000131171.8	SH3BGRL	blue	0.861187878
ENSG00000198478.6	SH3BGRL2	turquoise	0.920881497
ENSG00000142669.9	SH3BGRL3	red	0.843257704
ENSG00000100092.16	SH3BP1	magenta	0.909226673
ENSG00000087266.11	SH3BP2	green	0.632899471
ENSG00000130147.11	SH3BP4	brown	0.833499935
ENSG00000131370.11	SH3BP5	turquoise	0.853178404
ENSG00000224660.1	SH3BP5-AS1	yellow	0.910805101
ENSG00000175137.9	SH3BP5L	turquoise	0.933074285
ENSG00000109686.12	SH3D19	brown	0.869404045
ENSG00000214193.5	SH3D21	turquoise	0.873567397
ENSG00000141985.5	SH3GL1	turquoise	0.913915876
ENSG00000267352.1	SH3GL1P3	grey	0.800083543
ENSG00000107295.8	SH3GL2	turquoise	0.927086879
ENSG00000140600.12	SH3GL3	brown	0.7937062
ENSG00000097033.10	SH3GLB1	turquoise	0.844727801
ENSG00000148341.13	SH3GLB2	turquoise	0.90043834
ENSG00000147010.13	SH3KBP1	turquoise	0.939200472
ENSG00000107957.12	SH3PXD2A	turquoise	0.728043981
ENSG00000174705.7	SH3PXD2B	blue	0.778772047
ENSG00000154447.10	SH3RF1	turquoise	0.827621109
ENSG00000156463.13	SH3RF2	turquoise	0.761240273
ENSG00000172985.8	SH3RF3	turquoise	0.771782731
ENSG00000125089.12	SH3TC1	magenta	0.812267321
ENSG00000169247.7	SH3TC2	brown	0.898748645
ENSG00000035115.17	SH3YL1	turquoise	0.959960338
ENSG00000161681.11	SHANK1	turquoise	0.707879556
ENSG00000162105.12	SHANK2	turquoise	0.893683817
ENSG00000251322.3	SHANK3	turquoise	0.891047555
ENSG00000179526.12	SHARPIN	red	0.927720671
ENSG00000107338.8	SHB	turquoise	0.796944166
ENSG00000129214.10	SHBG	turquoise	0.747068266
ENSG00000160691.14	SHC1	blue	0.692000792
ENSG00000129946.6	SHC2	yellow	0.73036302

ENSG00000148082.5	SHC3	turquoise	0.75654413
ENSG00000185634.7	SHC4	brown	0.905454305
ENSG00000171241.4	SHCBP1	turquoise	0.780470365
ENSG00000105251.6	SHD	grey	0.315791387
ENSG00000169291.5	SHE	black	0.730075594
ENSG00000138606.15	SHF	turquoise	0.896586639
ENSG00000127922.5	SHFM1	turquoise	0.882914598
ENSG00000180730.4	SHISA2	turquoise	0.664628218
ENSG00000178343.4	SHISA3	grey	0.349925154
ENSG00000198892.6	SHISA4	blue	0.664994609
ENSG00000164054.11	SHISA5	turquoise	0.88486833
ENSG00000187902.7	SHISA7	turquoise	0.870943295
ENSG00000237515.6	SHISA9	turquoise	0.895824702
ENSG00000160410.10	SHKBP1	blue	0.742762513
ENSG00000176974.13	SHMT1	blue	0.844341879
ENSG00000182199.6	SHMT2	blue	0.693637796
ENSG00000108061.7	SHOC2	turquoise	0.961713316
ENSG00000197417.7	SHPK	blue	0.832078706
ENSG00000146414.11	SHPRH	turquoise	0.875579618
ENSG00000144736.9	SHQ1	turquoise	0.916597004
ENSG00000164403.10	SHROOM1	brown	0.775328415
ENSG00000146950.8	SHROOM2	turquoise	0.910438915
ENSG00000158352.11	SHROOM4	brown	0.853389689
ENSG00000110013.8	SIAE	turquoise	0.921458457
ENSG00000196470.7	SIAH1	turquoise	0.890686494
ENSG00000181788.3	SIAH2	turquoise	0.868468817
ENSG00000215475.3	SIAH3	turquoise	0.784759416
ENSG00000072858.6	SIDT1	greenyellow	0.832676389
ENSG00000149577.11	SIDT2	turquoise	0.94072246
ENSG00000185187.8	SIGIRR	red	0.762412532
ENSG00000088827.8	SIGLEC1	yellow	0.616083529
ENSG00000142512.10	SIGLEC10	magenta	0.873491128
ENSG00000254415.3	SIGLEC14	magenta	0.790814141
ENSG00000105366.11	SIGLEC8	magenta	0.783808364
ENSG00000129450.4	SIGLEC9	magenta	0.855504644
ENSG00000147955.12	SIGMAR1	turquoise	0.94382034
ENSG00000142178.7	SIK1	black	0.639434769
ENSG00000170145.4	SIK2	turquoise	0.968099605
ENSG00000160584.11	SIK3	brown	0.775695062
ENSG00000052723.7	SIKE1	turquoise	0.891972793
ENSG00000120725.8	SIL1	turquoise	0.902636418
ENSG00000159263.11	SIM2	turquoise	0.810629217
ENSG00000170085.13	SIMC1	turquoise	0.955785276
ENSG00000169375.11	SIN3A	turquoise	0.950080198
ENSG00000127511.5	SIN3B	turquoise	0.947624148
ENSG00000213445.4	SIPA1	magenta	0.787023528
ENSG00000197555.5	SIPA1L1	turquoise	0.951255347
ENSG00000116991.6	SIPA1L2	blue	0.840861814

ENSG00000105738.6	SIPA1L3	turquoise	0.903089468
ENSG00000198053.7	SIRPA	blue	0.823547017
ENSG00000096717.7	SIRT1	turquoise	0.876529797
ENSG00000068903.15	SIRT2	brown	0.929745547
ENSG00000142082.10	SIRT3	turquoise	0.953272101
ENSG00000089163.4	SIRT4	turquoise	0.764019503
ENSG00000124523.10	SIRT5	turquoise	0.951674781
ENSG00000077463.10	SIRT6	turquoise	0.847259642
ENSG00000187531.9	SIRT7	blue	0.8451882
ENSG00000184990.8	SIVA1	red	0.948848293
ENSG00000138083.3	SIX3	turquoise	0.857246649
ENSG00000236502.1	SIX3-AS1	turquoise	0.762931673
ENSG00000177045.6	SIX5	green	0.689886259
ENSG00000182628.8	SKA2	blue	0.772878654
ENSG00000005020.8	SKAP2	turquoise	0.807255495
ENSG00000157933.9	SKI	turquoise	0.943743923
ENSG00000180592.12	SKIDA1	turquoise	0.887107439
ENSG00000136603.9	SKIL	turquoise	0.818422963
ENSG00000204351.7	SKIV2L	turquoise	0.930988919
ENSG00000039123.11	SKIV2L2	turquoise	0.956775966
ENSG00000188779.6	SKOR1	turquoise	0.766256087
ENSG00000113558.14	SKP1	turquoise	0.94015998
ENSG00000231234.1	SKP1P1	turquoise	0.638440163
ENSG00000145604.11	SKP2	blue	0.908068828
ENSG00000155926.9	SLA	turquoise	0.687732942
ENSG00000139737.17	SLAIN1	brown	0.924411026
ENSG00000109171.10	SLAIN2	turquoise	0.833732751
ENSG00000158714.6	SLAMF8	magenta	0.761861133
ENSG00000163950.8	SLBP	turquoise	0.952898504
ENSG00000126903.11	SLC10A3	blue	0.748081162
ENSG00000145248.6	SLC10A4	midnightblue	0.799781299
ENSG00000253598.1	SLC10A5	turquoise	0.8775037
ENSG00000120519.10	SLC10A7	turquoise	0.805516855
ENSG00000018280.12	SLC11A1	magenta	0.784552827
ENSG00000110911.10	SLC11A2	turquoise	0.892059485
ENSG00000064651.9	SLC12A2	brown	0.926262416
ENSG00000124067.12	SLC12A4	blue	0.829713614
ENSG00000124140.8	SLC12A5	turquoise	0.920621417
ENSG00000113504.15	SLC12A7	brown	0.527170191
ENSG00000221955.6	SLC12A8	turquoise	0.858817661
ENSG00000146828.13	SLC12A9	yellow	0.872047204
ENSG00000158296.9	SLC13A3	yellow	0.863681753
ENSG00000141485.11	SLC13A5	green	0.515427776
ENSG00000141469.12	SLC14A1	grey	0.308990507
ENSG00000163406.6	SLC15A2	green	0.870702068
ENSG00000110446.5	SLC15A3	magenta	0.75181846
ENSG00000139370.6	SLC15A4	turquoise	0.902352679
ENSG00000155380.7	SLC16A1	black	0.515720137

ENSG00000112394.12	SLC16A10	turquoise	0.865514343
ENSG00000174326.7	SLC16A11	turquoise	0.672303875
ENSG00000163053.6	SLC16A14	turquoise	0.844802818
ENSG00000147100.5	SLC16A2	turquoise	0.936480528
ENSG00000141526.10	SLC16A3	magenta	0.780812609
ENSG00000168679.13	SLC16A4	green	0.577174317
ENSG00000108932.7	SLC16A6	grey	0.498054238
ENSG00000100156.6	SLC16A8	turquoise	0.862708183
ENSG00000165449.7	SLC16A9	green	0.778501187
ENSG00000119899.11	SLC17A5	turquoise	0.959790704
ENSG00000104888.5	SLC17A7	grey	0.059433896
ENSG00000179520.6	SLC17A8	midnightblue	0.689681739
ENSG00000101194.13	SLC17A9	magenta	0.82259305
ENSG00000165646.7	SLC18A2	turquoise	0.879054283
ENSG00000187714.5	SLC18A3	midnightblue	0.70521664
ENSG00000146409.6	SLC18B1	green	0.89858182
ENSG00000173638.14	SLC19A1	brown	0.550514712
ENSG00000117479.8	SLC19A2	turquoise	0.867320786
ENSG00000135917.9	SLC19A3	black	0.56424783
ENSG00000106688.7	SLC1A1	turquoise	0.901909033
ENSG00000110436.7	SLC1A2	green	0.766727381
ENSG00000079215.9	SLC1A3	green	0.900754788
ENSG00000115902.6	SLC1A4	green	0.825276468
ENSG00000105281.8	SLC1A5	magenta	0.83262072
ENSG00000105143.8	SLC1A6	turquoise	0.860934595
ENSG00000162383.7	SLC1A7	grey	0.229814856
ENSG00000144136.6	SLC20A1	turquoise	0.896987514
ENSG00000168575.5	SLC20A2	brown	0.841223812
ENSG00000163393.8	SLC22A15	brown	0.876765594
ENSG00000092096.10	SLC22A17	turquoise	0.945410544
ENSG00000137266.10	SLC22A23	turquoise	0.890028048
ENSG00000146477.4	SLC22A3	grey	0.440443687
ENSG00000197375.8	SLC22A5	blue	0.805583507
ENSG00000197901.7	SLC22A6	grey	0.142993414
ENSG00000089057.10	SLC23A2	turquoise	0.928588903
ENSG00000074621.9	SLC24A1	turquoise	0.841210076
ENSG00000155886.7	SLC24A2	midnightblue	0.912448195
ENSG00000185052.7	SLC24A3	turquoise	0.925479824
ENSG00000100075.5	SLC25A1	blue	0.790557979
ENSG00000183048.7	SLC25A10	turquoise	0.78367345
ENSG00000108528.9	SLC25A11	turquoise	0.904371549
ENSG00000115840.9	SLC25A12	turquoise	0.968074342
ENSG00000004864.9	SLC25A13	brown	0.915352112
ENSG00000102078.11	SLC25A14	turquoise	0.956900524
ENSG00000102743.10	SLC25A15	turquoise	0.862946894
ENSG00000122912.10	SLC25A16	turquoise	0.934559089
ENSG00000100372.10	SLC25A17	turquoise	0.967720665
ENSG00000182902.9	SLC25A18	green	0.762574093

ENSG00000125454.7	SLC25A19	turquoise	0.879711144
ENSG00000178537.5	SLC25A20	green	0.889264624
ENSG00000258708.1	SLC25A21-AS1	blue	0.688411494
ENSG00000177542.6	SLC25A22	turquoise	0.907928706
ENSG00000125648.10	SLC25A23	turquoise	0.853861902
ENSG00000085491.11	SLC25A24	turquoise	0.715695399
ENSG00000241361.4	SLC25A24P1	midnightblue	0.78612304
ENSG00000148339.8	SLC25A25	turquoise	0.864925785
ENSG00000144741.13	SLC25A26	turquoise	0.898956809
ENSG00000153291.11	SLC25A27	turquoise	0.917019127
ENSG00000155287.6	SLC25A28	turquoise	0.855722309
ENSG00000197119.8	SLC25A29	yellow	0.925283264
ENSG00000075415.8	SLC25A3	turquoise	0.952150641
ENSG00000174032.12	SLC25A30	turquoise	0.897507464
ENSG00000164933.7	SLC25A32	turquoise	0.945665548
ENSG00000171612.6	SLC25A33	green	0.700552191
ENSG00000162461.7	SLC25A34	yellow	0.820628814
ENSG00000125434.6	SLC25A35	blue	0.740512114
ENSG00000114120.7	SLC25A36	turquoise	0.912694665
ENSG00000147454.9	SLC25A37	turquoise	0.815542041
ENSG00000144659.6	SLC25A38	turquoise	0.96565003
ENSG00000013306.11	SLC25A39	blue	0.806929266
ENSG00000151729.6	SLC25A4	turquoise	0.962868144
ENSG00000075303.8	SLC25A40	turquoise	0.946626031
ENSG00000181035.9	SLC25A42	turquoise	0.939912709
ENSG00000077713.14	SLC25A43	green	0.764872821
ENSG00000160785.9	SLC25A44	turquoise	0.957172538
ENSG00000162241.8	SLC25A45	turquoise	0.786952809
ENSG00000164209.12	SLC25A46	turquoise	0.960083528
ENSG00000145832.8	SLC25A48	green	0.530476821
ENSG00000005022.5	SLC25A5	turquoise	0.844829324
ENSG00000224281.4	SLC25A5-AS1	turquoise	0.814286677
ENSG00000122696.8	SLC25A51	turquoise	0.847944042
ENSG00000176274.6	SLC25A53	turquoise	0.781214863
ENSG00000169100.8	SLC25A6	turquoise	0.885707699
ENSG00000145217.9	SLC26A1	yellow	0.850467426
ENSG00000135502.12	SLC26A10	turquoise	0.901784739
ENSG00000181045.10	SLC26A11	turquoise	0.886134551
ENSG00000155850.7	SLC26A2	black	0.819669048
ENSG00000233705.2	SLC26A4-AS1	turquoise	0.745409237
ENSG00000225697.6	SLC26A6	yellow	0.912560795
ENSG00000112053.9	SLC26A8	turquoise	0.8339573
ENSG00000130304.12	SLC27A1	brown	0.758171748
ENSG00000143554.9	SLC27A3	green	0.645415557
ENSG00000167114.8	SLC27A4	turquoise	0.925245271
ENSG00000083807.5	SLC27A5	red	0.908283659
ENSG00000112759.12	SLC29A1	turquoise	0.931595029
ENSG00000174669.7	SLC29A2	yellow	0.879197155

ENSG00000198246.7	SLC29A3	blue	0.765703513
ENSG00000164638.6	SLC29A4	greenyellow	0.657297841
ENSG00000117394.15	SLC2A1	black	0.720927703
ENSG00000227533.1	SLC2A1-AS1	turquoise	0.746963348
ENSG00000197496.4	SLC2A10	green	0.906897251
ENSG00000133460.15	SLC2A11	turquoise	0.940468117
ENSG00000146411.5	SLC2A12	green	0.820876908
ENSG00000151229.8	SLC2A13	turquoise	0.918021351
ENSG00000059804.11	SLC2A3	turquoise	0.6916095
ENSG00000181856.10	SLC2A4	grey	0.380456124
ENSG00000125520.9	SLC2A4RG	green	0.607474706
ENSG00000142583.13	SLC2A5	magenta	0.916247471
ENSG00000160326.9	SLC2A6	turquoise	0.824356102
ENSG00000136856.13	SLC2A8	turquoise	0.881816529
ENSG00000170385.9	SLC30A1	turquoise	0.724218994
ENSG00000196660.6	SLC30A10	green	0.557608771
ENSG00000104154.5	SLC30A4	turquoise	0.867318517
ENSG00000145740.14	SLC30A5	turquoise	0.938178116
ENSG00000152683.10	SLC30A6	turquoise	0.889346139
ENSG00000162695.7	SLC30A7	turquoise	0.841832742
ENSG00000014824.9	SLC30A9	turquoise	0.947940204
ENSG00000136868.9	SLC31A1	turquoise	0.807418012
ENSG00000136867.6	SLC31A2	brown	0.89006646
ENSG00000101438.3	SLC32A1	turquoise	0.909759319
ENSG00000169359.9	SLC33A1	turquoise	0.91512118
ENSG00000164414.12	SLC35A1	turquoise	0.94442847
ENSG00000102100.10	SLC35A2	turquoise	0.934284984
ENSG00000117620.8	SLC35A3	turquoise	0.869681396
ENSG00000176087.10	SLC35A4	blue	0.89812183
ENSG00000138459.4	SLC35A5	turquoise	0.899469645
ENSG00000121073.9	SLC35B1	turquoise	0.942520758
ENSG00000157593.12	SLC35B2	brown	0.835200367
ENSG00000124786.8	SLC35B3	turquoise	0.820563927
ENSG00000205060.6	SLC35B4	turquoise	0.975221422
ENSG00000181830.7	SLC35C1	turquoise	0.852507182
ENSG00000080189.10	SLC35C2	blue	0.764403665
ENSG00000116704.6	SLC35D1	turquoise	0.924448546
ENSG00000130958.7	SLC35D2	brown	0.870481913
ENSG00000182747.4	SLC35D3	turquoise	0.818483424
ENSG00000127526.9	SLC35E1	turquoise	0.969053947
ENSG00000215790.2	SLC35E2	turquoise	0.851116816
ENSG00000189339.7	SLC35E2B	turquoise	0.918172222
ENSG00000175782.8	SLC35E3	turquoise	0.898607159
ENSG00000100036.11	SLC35E4	turquoise	0.907704335
ENSG00000196376.6	SLC35F1	turquoise	0.898949862
ENSG00000110660.10	SLC35F2	turquoise	0.702669026
ENSG00000183780.8	SLC35F3	turquoise	0.901313549
ENSG00000151812.10	SLC35F4	turquoise	0.844813292

ENSG00000115084.8	SLC35F5	blue	0.887300442
ENSG00000213699.4	SLC35F6	turquoise	0.845769637
ENSG00000176273.10	SLC35G1	turquoise	0.771940038
ENSG00000168917.8	SLC35G2	blue	0.825035855
ENSG00000123643.8	SLC36A1	turquoise	0.865625147
ENSG00000180773.10	SLC36A4	turquoise	0.933282629
ENSG00000160190.9	SLC37A1	turquoise	0.955395449
ENSG00000157800.13	SLC37A3	turquoise	0.955495288
ENSG00000137700.12	SLC37A4	turquoise	0.839264472
ENSG00000111371.11	SLC38A1	turquoise	0.946654007
ENSG00000157637.8	SLC38A10	blue	0.875854356
ENSG00000169507.5	SLC38A11	brown	0.598246294
ENSG00000134294.9	SLC38A2	turquoise	0.648367331
ENSG00000188338.10	SLC38A3	brown	0.589885654
ENSG00000017483.10	SLC38A5	brown	0.505918227
ENSG00000139974.11	SLC38A6	yellow	0.905860219
ENSG00000103042.4	SLC38A7	turquoise	0.945609898
ENSG00000177058.7	SLC38A9	turquoise	0.898762514
ENSG00000143570.13	SLC39A1	blue	0.80268233
ENSG00000196950.9	SLC39A10	turquoise	0.880305404
ENSG00000133195.7	SLC39A11	green	0.669416305
ENSG00000148482.7	SLC39A12	green	0.583350472
ENSG00000165915.9	SLC39A13	turquoise	0.895868091
ENSG00000104635.9	SLC39A14	cyan	0.800765518
ENSG00000141873.6	SLC39A3	turquoise	0.883442101
ENSG00000147804.5	SLC39A4	turquoise	0.747898584
ENSG00000141424.8	SLC39A6	turquoise	0.879005152
ENSG00000112473.12	SLC39A7	turquoise	0.846066477
ENSG00000138821.8	SLC39A8	brown	0.610147443
ENSG00000029364.7	SLC39A9	turquoise	0.942776267
ENSG00000168003.12	SLC3A2	blue	0.673773563
ENSG00000138449.6	SLC40A1	blue	0.744785881
ENSG00000133065.6	SLC41A1	blue	0.915992645
ENSG00000136052.5	SLC41A2	turquoise	0.931454067
ENSG00000114544.11	SLC41A3	turquoise	0.95405096
ENSG00000149150.4	SLC43A1	yellow	0.852054251
ENSG00000167703.10	SLC43A2	blue	0.736780007
ENSG00000134802.13	SLC43A3	blue	0.647346494
ENSG00000070214.11	SLC44A1	brown	0.943378403
ENSG00000129353.10	SLC44A2	green	0.896511995
ENSG00000143036.12	SLC44A3	green	0.754245599
ENSG00000162426.10	SLC45A1	turquoise	0.925657867
ENSG00000158715.5	SLC45A3	brown	0.917038451
ENSG00000022567.5	SLC45A4	turquoise	0.903357247
ENSG00000076351.8	SLC46A1	blue	0.876671712
ENSG00000139508.10	SLC46A3	turquoise	0.755295482
ENSG00000142494.9	SLC47A1	midnightblue	0.697387307
ENSG00000211584.9	SLC48A1	brown	0.868474974

ENSG00000144290.12	SLC4A10	turquoise	0.909242802
ENSG00000088836.8	SLC4A11	turquoise	0.529767512
ENSG00000163798.9	SLC4A1AP	turquoise	0.977304225
ENSG00000164889.8	SLC4A2	brown	0.846622798
ENSG00000114923.12	SLC4A3	turquoise	0.887616777
ENSG00000080493.9	SLC4A4	green	0.772650525
ENSG00000188687.11	SLC4A5	blue	0.746008378
ENSG00000033867.12	SLC4A7	turquoise	0.80922795
ENSG00000050438.12	SLC4A8	turquoise	0.866601276
ENSG00000169241.13	SLC50A1	blue	0.791432866
ENSG00000185803.4	SLC52A2	blue	0.855118821
ENSG00000101276.10	SLC52A3	black	0.69133596
ENSG00000158865.8	SLC5A11	brown	0.769127563
ENSG00000148942.10	SLC5A12	midnightblue	0.808229982
ENSG00000138074.10	SLC5A6	brown	0.619136965
ENSG00000115665.4	SLC5A7	midnightblue	0.749681728
ENSG00000157103.6	SLC6A1	turquoise	0.765188387
ENSG00000232287.2	SLC6A1-AS1	brown	0.537335381
ENSG00000111181.8	SLC6A12	brown	0.567874193
ENSG00000010379.11	SLC6A13	brown	0.471218918
ENSG00000072041.12	SLC6A15	turquoise	0.878331425
ENSG00000197106.6	SLC6A17	turquoise	0.918976674
ENSG00000131389.12	SLC6A6	turquoise	0.777926507
ENSG00000130821.11	SLC6A8	brown	0.842087554
ENSG00000196517.7	SLC6A9	blue	0.719037347
ENSG00000139514.8	SLC7A1	blue	0.753918948
ENSG00000130876.7	SLC7A10	green	0.646896505
ENSG00000151012.9	SLC7A11	green	0.871287266
ENSG00000013293.5	SLC7A14	turquoise	0.906497168
ENSG00000003989.12	SLC7A2	green	0.664958332
ENSG00000099960.8	SLC7A4	turquoise	0.838795503
ENSG00000103257.4	SLC7A5	black	0.741885979
ENSG00000260727.1	SLC7A5P1	blue	0.482615167
ENSG00000103064.9	SLC7A6	turquoise	0.90493554
ENSG00000103061.7	SLC7A6OS	turquoise	0.846099812
ENSG00000155465.14	SLC7A7	magenta	0.940392325
ENSG00000092068.14	SLC7A8	turquoise	0.838935434
ENSG00000021488.8	SLC7A9	grey	0.598093591
ENSG00000183023.14	SLC8A1	turquoise	0.908719239
ENSG00000118160.9	SLC8A2	turquoise	0.877988306
ENSG00000100678.14	SLC8A3	turquoise	0.879969088
ENSG00000089060.7	SLC8B1	blue	0.810623228
ENSG00000090020.6	SLC9A1	midnightblue	0.944503353
ENSG00000066230.6	SLC9A3	yellow	0.754124325
ENSG00000109062.5	SLC9A3R1	green	0.644885895
ENSG00000065054.9	SLC9A3R2	brown	0.626672782
ENSG00000135740.12	SLC9A5	turquoise	0.877091331
ENSG00000198689.5	SLC9A6	turquoise	0.857827326

ENSG00000065923.5	SLC9A7	turquoise	0.86085796
ENSG00000197818.7	SLC9A8	turquoise	0.927669362
ENSG00000181804.10	SLC9A9	blue	0.81272792
ENSG00000164038.10	SLC9B2	turquoise	0.93878139
ENSG00000084453.12	SLCO1A2	brown	0.700278556
ENSG00000139155.4	SLCO1C1	green	0.794718376
ENSG00000137491.10	SLCO2B1	magenta	0.755572922
ENSG00000176463.9	SLCO3A1	brown	0.94656768
ENSG00000101187.11	SLCO4A1	black	0.791445463
ENSG00000172716.12	SLFN11	blue	0.666496222
ENSG00000166750.5	SLFN5	brown	0.601151091
ENSG00000119705.5	SLIRP	red	0.928850534
ENSG00000187122.12	SLIT1	blue	0.581990428
ENSG00000145147.15	SLIT2	turquoise	0.922067577
ENSG00000184347.10	SLIT3	turquoise	0.894206959
ENSG00000178235.6	SLITRK1	brown	0.839887974
ENSG00000185985.7	SLITRK2	turquoise	0.833352877
ENSG00000121871.3	SLITRK3	turquoise	0.917453735
ENSG00000179542.11	SLITRK4	turquoise	0.843492552
ENSG00000165300.6	SLITRK5	turquoise	0.924482747
ENSG00000184564.8	SLITRK6	turquoise	0.818504073
ENSG00000065613.9	SLK	turquoise	0.947090967
ENSG00000163681.10	SLMAP	turquoise	0.920046825
ENSG00000141391.9	SLMO1	turquoise	0.848842877
ENSG00000101166.11	SLMO2	blue	0.896728502
ENSG00000124107.5	SLPI	grey	0.235281282
ENSG00000137776.12	SLTM	turquoise	0.881487793
ENSG00000164609.5	SLU7	turquoise	0.961599064
ENSG00000188827.6	SLX4	turquoise	0.857940796
ENSG00000170365.5	SMAD1	turquoise	0.886780256
ENSG00000175387.11	SMAD2	blue	0.94643872
ENSG00000166949.11	SMAD3	turquoise	0.885280898
ENSG00000141646.9	SMAD4	turquoise	0.921363753
ENSG00000113658.12	SMAD5	blue	0.9153908
ENSG00000137834.10	SMAD6	grey	0.465465524
ENSG00000101665.4	SMAD7	brown	0.712242379
ENSG00000120693.9	SMAD9	blue	0.829782081
ENSG00000170545.12	SMAGP	turquoise	0.639521103
ENSG00000084070.7	SMAP2	turquoise	0.95991615
ENSG00000102038.11	SMARCA1	turquoise	0.927049711
ENSG00000080503.15	SMARCA2	turquoise	0.9471214
ENSG00000127616.13	SMARCA4	turquoise	0.967488297
ENSG00000153147.5	SMARCA5	blue	0.958153882
ENSG00000163104.13	SMARCAD1	turquoise	0.942547121
ENSG00000138375.8	SMARCAL1	turquoise	0.930441685
ENSG00000099956.13	SMARCB1	turquoise	0.931802343
ENSG00000173473.6	SMARCC1	brown	0.798558852
ENSG00000139613.7	SMARCC2	turquoise	0.940314398

ENSG00000066117.10	SMARCD1	turquoise	0.947563552
ENSG00000108604.11	SMARCD2	yellow	0.874676602
ENSG00000082014.12	SMARCD3	turquoise	0.883145815
ENSG00000073584.14	SMARCE1	turquoise	0.929970089
ENSG00000230793.1	SMARCE1P5	grey	0.561803118
ENSG00000072501.13	SMC1A	turquoise	0.807168118
ENSG00000136824.14	SMC2	turquoise	0.837391379
ENSG00000108055.9	SMC3	turquoise	0.912642122
ENSG00000113810.11	SMC4	turquoise	0.702143074
ENSG00000198887.7	SMC5	blue	0.905029358
ENSG00000163029.11	SMC6	turquoise	0.859199313
ENSG00000101596.10	SMCHD1	turquoise	0.86180833
ENSG00000183172.8	SMDT1	red	0.957026386
ENSG00000100796.13	SMEK1	turquoise	0.873657316
ENSG00000138041.11	SMEK2	turquoise	0.925791334
ENSG00000157106.12	SMG1	turquoise	0.936676633
ENSG00000198952.7	SMG5	turquoise	0.921953788
ENSG00000070366.9	SMG6	turquoise	0.867894246
ENSG00000116698.16	SMG7	turquoise	0.973615261
ENSG00000167447.8	SMG8	turquoise	0.941012099
ENSG00000105771.9	SMG9	turquoise	0.874121372
ENSG00000184785.4	SMIM10	blue	0.667817994
ENSG00000205670.6	SMIM11	turquoise	0.940867218
ENSG00000163866.8	SMIM12	turquoise	0.94639561
ENSG00000224531.4	SMIM13	turquoise	0.928548809
ENSG00000163683.7	SMIM14	turquoise	0.940916418
ENSG00000188725.3	SMIM15	turquoise	0.912499363
ENSG00000268182.1	SMIM17	turquoise	0.86173273
ENSG00000253457.1	SMIM18	turquoise	0.820756692
ENSG00000176209.7	SMIM19	turquoise	0.937548443
ENSG00000250317.4	SMIM20	blue	0.739487457
ENSG00000168273.3	SMIM4	turquoise	0.871747588
ENSG00000204323.5	SMIM5	brown	0.69139281
ENSG00000259120.2	SMIM6	brown	0.765612428
ENSG00000214046.4	SMIM7	turquoise	0.907805078
ENSG00000111850.6	SMIM8	turquoise	0.91010341
ENSG00000240204.2	SMKR1	turquoise	0.901701576
ENSG00000172062.12	SMN1	turquoise	0.839481572
ENSG00000205571.8	SMN2	grey	0.377461097
ENSG00000119953.8	SMNDC1	turquoise	0.918526389
ENSG00000128602.5	SMO	green	0.728183206
ENSG00000198732.6	SMOC1	grey	0.46988257
ENSG00000112562.14	SMOC2	turquoise	0.714254635
ENSG00000088826.13	SMOX	green	0.73585605
ENSG00000166311.5	SMPD1	blue	0.842166441
ENSG00000135587.4	SMPD2	pink	0.891965761
ENSG00000103056.7	SMPD3	turquoise	0.862186739
ENSG00000136699.15	SMPD4	turquoise	0.921779463

ENSG00000172594.8	SMPDL3A	turquoise	0.864636145
ENSG00000102172.11	SMS	turquoise	0.835104614
ENSG00000183963.14	SMTN	black	0.775050203
ENSG00000122692.7	SMU1	turquoise	0.927567963
ENSG00000123415.10	SMUG1	turquoise	0.946959815
ENSG00000198742.5	SMURF1	turquoise	0.953317543
ENSG00000108854.11	SMURF2	turquoise	0.890850489
ENSG00000248121.4	SMURF2P1	turquoise	0.821849884
ENSG00000143499.9	SMYD2	greenyellow	0.857803607
ENSG00000185420.14	SMYD3	turquoise	0.938723403
ENSG00000186532.7	SMYD4	turquoise	0.939444766
ENSG00000135632.7	SMYD5	turquoise	0.954537874
ENSG00000124216.3	SNAI1	black	0.673011864
ENSG00000185669.5	SNAI3	turquoise	0.829147735
ENSG00000260630.2	SNAI3-AS1	turquoise	0.772575124
ENSG00000092531.5	SNAP23	blue	0.848040067
ENSG00000132639.8	SNAP25	turquoise	0.923141785
ENSG00000227906.3	SNAP25-AS1	turquoise	0.848783185
ENSG00000099940.7	SNAP29	turquoise	0.903736778
ENSG00000143740.10	SNAP47	turquoise	0.949340706
ENSG00000065609.10	SNAP91	turquoise	0.943555927
ENSG00000023608.4	SNAPC1	blue	0.899866425
ENSG00000104976.7	SNAPC2	turquoise	0.922935485
ENSG00000164975.11	SNAPC3	turquoise	0.954794411
ENSG00000165684.3	SNAPC4	yellow	0.917139559
ENSG00000174446.8	SNAPC5	turquoise	0.952066115
ENSG00000143553.6	SNAPIN	blue	0.89234565
ENSG00000145335.11	SNCA	turquoise	0.919136764
ENSG00000064692.14	SNCAIP	turquoise	0.834004705
ENSG00000074317.6	SNCB	turquoise	0.821369711
ENSG00000173267.9	SNCG	grey	0.403676335
ENSG00000197157.6	SND1	turquoise	0.951538893
ENSG00000162804.9	SNED1	blue	0.742399363
ENSG00000159210.5	SNF8	red	0.952465251
ENSG00000255717.2	SNHG1	blue	0.819516872
ENSG00000247092.2	SNHG10	pink	0.880680898
ENSG00000174365.15	SNHG11	turquoise	0.889112649
ENSG00000197989.9	SNHG12	blue	0.81365202
ENSG00000224078.8	SNHG14	turquoise	0.932707445
ENSG00000232956.4	SNHG15	turquoise	0.879984847
ENSG00000163597.10	SNHG16	turquoise	0.875917593
ENSG00000196756.7	SNHG17	blue	0.631531634
ENSG00000250786.1	SNHG18	grey	0.417091763
ENSG00000242125.2	SNHG3	grey	0.207631278
ENSG00000203875.6	SNHG5	red	0.558926465
ENSG00000245910.4	SNHG6	red	0.895636321
ENSG00000233016.2	SNHG7	turquoise	0.744089589
ENSG00000269893.2	SNHG8	turquoise	0.817107638

ENSG00000255198.3	SNHG9	red	0.749400525
ENSG00000163877.9	SNIP1	turquoise	0.935651007
ENSG00000184602.5	SNN	turquoise	0.860416477
ENSG00000221716.1	SNORA11	grey	0.361710861
ENSG00000212293.1	SNORA16	turquoise	0.443647473
ENSG00000207145.1	SNORA18	turquoise	0.685937867
ENSG00000202343.1	SNORA2	grey	0.451270805
ENSG00000199293.1	SNORA21	turquoise	0.594994905
ENSG00000212588.1	SNORA26	turquoise	0.431992735
ENSG00000207051.1	SNORA27	blue	0.542018613
ENSG00000253051.1	SNORA31	grey	0.197395181
ENSG00000200534.1	SNORA33	grey	0.436523932
ENSG00000212607.1	SNORA45	grey	0.434466317
ENSG00000209582.1	SNORA48	blue	0.608165645
ENSG00000199785.1	SNORA52	yellow	0.600232916
ENSG00000212443.1	SNORA53	turquoise	0.553489979
ENSG00000199266.1	SNORA60	grey	0.426139021
ENSG00000202363.1	SNORA62	turquoise	0.714318617
ENSG00000199363.1	SNORA63	turquoise	0.676874588
ENSG00000201448.1	SNORA63	turquoise	0.440354919
ENSG00000200320.1	SNORA63	turquoise	0.719833744
ENSG00000207405.1	SNORA64	yellow	0.692112103
ENSG00000201302.1	SNORA65	blue	0.532544257
ENSG00000207523.1	SNORA66	blue	0.529021391
ENSG00000207165.1	SNORA70	yellow	0.876497907
ENSG00000252531.1	SNORA73	yellow	0.565281496
ENSG00000200087.1	SNORA73B	grey	0.175493592
ENSG00000266402.2	SNORA76	grey	0.342893978
ENSG00000207304.1	SNORA8	turquoise	0.840006734
ENSG00000221420.2	SNORA81	turquoise	0.710718239
ENSG00000238917.1	SNORD10	yellow	0.815483838
ENSG00000261069.2	SNORD116-20	turquoise	0.65307392
ENSG00000200879.1	SNORD14E	grey	0.271211935
ENSG00000200530.1	SNORD35B	grey	0.445445389
ENSG00000263934.2	SNORD3A	grey	0.156070691
ENSG00000270704.2	SNORD64	turquoise	0.592948372
ENSG00000212283.1	SNORD89	turquoise	0.570266727
ENSG00000208772.1	SNORD94	turquoise	0.610820044
ENSG00000238896.1	snoU13	grey	0.166186418
ENSG00000238465.1	snoU13	turquoise	0.779425705
ENSG00000238556.1	snoU13	turquoise	0.487789092
ENSG00000238922.1	snoU13	turquoise	0.561526535
ENSG00000101298.9	SNPH	turquoise	0.888856519
ENSG00000163788.9	SNRK	turquoise	0.865068489
ENSG00000144028.10	SNRNP200	turquoise	0.940377042
ENSG00000161981.6	SNRNP25	red	0.931348732
ENSG00000124380.6	SNRNP27	turquoise	0.936500769
ENSG00000184209.14	SNRNP35	brown	0.773233468

ENSG00000060688.8	SNRNP40	turquoise	0.911743823
ENSG00000168566.11	SNRNP48	blue	0.86340535
ENSG00000104852.10	SNRNP70	yellow	0.945940565
ENSG00000077312.4	SNRPA	turquoise	0.893584819
ENSG00000131876.12	SNRPA1	turquoise	0.922841311
ENSG00000125835.13	SNRPB	blue	0.820131223
ENSG00000125870.6	SNRPB2	turquoise	0.949797655
ENSG00000124562.5	SNRPC	turquoise	0.919776599
ENSG00000167088.6	SNRPD1	turquoise	0.921998578
ENSG00000125743.6	SNRPD2	red	0.957681095
ENSG00000100028.7	SNRPD3	turquoise	0.918902886
ENSG00000182004.8	SNRPE	turquoise	0.909553072
ENSG00000233270.1	SNRPEP4	turquoise	0.615531982
ENSG00000139343.6	SNRPF	turquoise	0.903769602
ENSG00000143977.9	SNRPG	turquoise	0.783349638
ENSG00000235363.1	SNRPGP10	turquoise	0.660569379
ENSG00000264350.1	SNRPGP2	turquoise	0.546896928
ENSG00000128739.16	SNRPN	turquoise	0.930804554
ENSG00000101400.5	SNTA1	green	0.654178131
ENSG00000172164.9	SNTB1	green	0.835401205
ENSG00000168807.12	SNTB2	turquoise	0.853869614
ENSG00000147481.9	SNTG1	turquoise	0.842121152
ENSG00000169371.9	SNUPN	turquoise	0.932791075
ENSG00000273173.1	SNURF	turquoise	0.767190509
ENSG00000100603.9	SNW1	turquoise	0.938523158
ENSG00000028528.10	SNX1	brown	0.893385021
ENSG00000086300.11	SNX10	turquoise	0.906005474
ENSG00000002919.10	SNX11	turquoise	0.950222657
ENSG00000147164.7	SNX12	turquoise	0.940397638
ENSG00000071189.17	SNX13	turquoise	0.916074302
ENSG00000135317.8	SNX14	turquoise	0.945368435
ENSG00000110025.8	SNX15	turquoise	0.950439733
ENSG00000104497.9	SNX16	turquoise	0.766665251
ENSG00000115234.6	SNX17	turquoise	0.948513127
ENSG00000178996.8	SNX18	blue	0.847612684
ENSG00000230965.1	SNX18P13	turquoise	0.526683941
ENSG00000225345.3	SNX18P3	turquoise	0.781005841
ENSG00000120451.6	SNX19	blue	0.931498957
ENSG00000205302.2	SNX2	turquoise	0.929110527
ENSG00000124104.14	SNX21	turquoise	0.90574161
ENSG00000157734.9	SNX22	brown	0.737375948
ENSG00000064652.6	SNX24	blue	0.894743163
ENSG00000109762.11	SNX25	turquoise	0.962270488
ENSG00000143376.8	SNX27	turquoise	0.964187711
ENSG00000048471.9	SNX29	blue	0.89167709
ENSG00000112335.10	SNX3	turquoise	0.887712698
ENSG00000148158.12	SNX30	brown	0.901013843
ENSG00000172803.13	SNX32	turquoise	0.838171777

ENSG00000173548.8	SNX33	green	0.821568264
ENSG00000114520.6	SNX4	turquoise	0.890163835
ENSG00000089006.12	SNX5	blue	0.871759457
ENSG00000129515.14	SNX6	brown	0.850583386
ENSG00000162627.12	SNX7	blue	0.743464497
ENSG00000106266.4	SNX8	brown	0.750868552
ENSG00000130340.10	SNX9	turquoise	0.869262341
ENSG00000057252.8	SOAT1	cyan	0.912492744
ENSG00000112320.7	SOBP	turquoise	0.92583373
ENSG00000185338.4	SOCS1	black	0.452553574
ENSG00000120833.9	SOCS2	blue	0.673006682
ENSG00000184557.3	SOCS3	black	0.755524923
ENSG00000180008.8	SOCS4	turquoise	0.829085816
ENSG00000171150.7	SOCS5	turquoise	0.917296096
ENSG00000170677.5	SOCS6	turquoise	0.839437244
ENSG00000142168.10	SOD1	turquoise	0.936381311
ENSG00000112096.12	SOD2	cyan	0.873678715
ENSG00000109610.5	SOD3	green	0.617203971
ENSG00000149639.10	SOGA1	blue	0.867733354
ENSG00000168502.13	SOGA2	turquoise	0.887265375
ENSG00000165643.6	SOHLH1	brown	0.560562872
ENSG00000159140.13	SON	turquoise	0.929807235
ENSG00000095637.16	SORBS1	turquoise	0.883805273
ENSG00000154556.13	SORBS2	turquoise	0.921937178
ENSG00000120896.9	SORBS3	blue	0.638133443
ENSG00000108018.11	SORCS1	turquoise	0.898036953
ENSG00000184985.12	SORCS2	turquoise	0.824039398
ENSG00000156395.8	SORCS3	turquoise	0.879657732
ENSG00000140263.9	SORD	turquoise	0.886351053
ENSG00000137642.8	SORL1	blue	0.873788177
ENSG00000134243.7	SORT1	brown	0.914328634
ENSG00000115904.8	SOS1	turquoise	0.904434952
ENSG00000229692.3	SOS1-IT1	turquoise	0.706569251
ENSG00000100485.7	SOS2	brown	0.882157474
ENSG00000198944.4	SOWAHA	turquoise	0.836853732
ENSG00000198142.3	SOWAHC	blue	0.797355137
ENSG00000182968.3	SOX1	turquoise	0.782495363
ENSG00000100146.12	SOX10	brown	0.880587416
ENSG00000176887.5	SOX11	turquoise	0.846108681
ENSG00000177732.6	SOX12	turquoise	0.853425647
ENSG00000143842.10	SOX13	blue	0.774284858
ENSG00000129194.3	SOX15	turquoise	0.774492368
ENSG00000164736.5	SOX17	grey	0.417624988
ENSG00000203883.5	SOX18	grey	0.25204193
ENSG00000181449.2	SOX2	green	0.895520855
ENSG00000242808.3	SOX2-OT	brown	0.915157267
ENSG00000125285.4	SOX21	green	0.775685352
ENSG00000227640.2	SOX21-AS1	green	0.802200906

ENSG00000124766.4	SOX4	grey	0.388064257
ENSG00000134532.11	SOX5	green	0.860615636
ENSG00000110693.11	SOX6	blue	0.771419174
ENSG00000171056.6	SOX7	blue	0.590185029
ENSG00000005513.9	SOX8	brown	0.758490476
ENSG00000125398.5	SOX9	green	0.829230639
ENSG00000185591.5	SP1	blue	0.932608807
ENSG00000067066.12	SP100	black	0.874538917
ENSG00000135899.12	SP110	black	0.839778586
ENSG00000185404.12	SP140L	black	0.845687433
ENSG00000167182.11	SP2	turquoise	0.948239791
ENSG00000172845.9	SP3	turquoise	0.900214433
ENSG00000105866.9	SP4	turquoise	0.907500499
ENSG00000217236.1	SP9	turquoise	0.859899856
ENSG00000064199.2	SPA17	pink	0.947595367
ENSG00000104450.8	SPAG1	pink	0.931415852
ENSG00000144451.14	SPAG16	blue	0.792335154
ENSG00000061656.5	SPAG4	blue	0.660737118
ENSG00000076382.12	SPAG5	yellow	0.899517025
ENSG00000227543.3	SPAG5-AS1	turquoise	0.856623977
ENSG00000091640.3	SPAG7	turquoise	0.93555239
ENSG00000008294.16	SPAG9	blue	0.883498376
ENSG00000113140.6	SPARC	green	0.714024329
ENSG00000152583.8	SPARCL1	green	0.889845163
ENSG00000021574.7	SPAST	turquoise	0.888380468
ENSG00000182957.11	SPATA13	turquoise	0.936984876
ENSG00000158480.6	SPATA2	turquoise	0.925398433
ENSG00000006282.15	SPATA20	red	0.689946612
ENSG00000170469.6	SPATA24	pink	0.838854272
ENSG00000149634.4	SPATA25	yellow	0.824674464
ENSG00000158792.11	SPATA2L	turquoise	0.784081141
ENSG00000167523.9	SPATA33	pink	0.918467368
ENSG00000171763.13	SPATA5L1	turquoise	0.905646642
ENSG00000132122.7	SPATA6	blue	0.822421688
ENSG00000106686.12	SPATA6L	pink	0.931198504
ENSG00000042317.12	SPATA7	turquoise	0.90164323
ENSG00000160284.10	SPATC1L	turquoise	0.747551774
ENSG00000123352.13	SPATS2	turquoise	0.964664393
ENSG00000196141.8	SPATS2L	turquoise	0.736165339
ENSG00000114902.9	SPCS1	turquoise	0.926914062
ENSG00000118363.7	SPCS2	blue	0.901024897
ENSG00000129128.8	SPCS3	turquoise	0.954458309
ENSG00000124664.6	SPDEF	turquoise	0.659050634
ENSG00000040275.12	SPDL1	brown	0.755208676
ENSG00000128487.12	SPECC1	brown	0.920364758
ENSG00000100014.15	SPECC1L	blue	0.910707676
ENSG00000101222.8	SPEF1	turquoise	0.773352491
ENSG00000152582.8	SPEF2	pink	0.934305342

ENSG00000072195.10	SPEG	turquoise	0.888320409
ENSG00000065526.6	SPEN	blue	0.912997592
ENSG00000258484.3	SPESP1	turquoise	0.560794155
ENSG00000104133.10	SPG11	turquoise	0.865172366
ENSG00000133104.8	SPG20	turquoise	0.860264393
ENSG00000090487.6	SPG21	turquoise	0.946861441
ENSG00000197912.9	SPG7	turquoise	0.937264558
ENSG00000176170.9	SPHK1	black	0.744701813
ENSG00000063176.11	SPHK2	brown	0.746695965
ENSG00000153820.8	SPHKAP	midnightblue	0.794803662
ENSG00000066336.7	SPI1	magenta	0.929758987
ENSG00000163611.7	SPICE1	pink	0.919511311
ENSG00000164808.12	SPIDR	turquoise	0.944095391
ENSG00000106723.12	SPIN1	turquoise	0.969751128
ENSG00000186787.7	SPIN2B	turquoise	0.938654082
ENSG00000204271.6	SPIN3	turquoise	0.941087077
ENSG00000186767.5	SPIN4	turquoise	0.84661464
ENSG00000167642.8	SPINT2	turquoise	0.930970677
ENSG00000204991.6	SPIRE2	turquoise	0.911545139
ENSG00000169682.13	SPNS1	turquoise	0.889357693
ENSG00000183018.4	SPNS2	turquoise	0.892852503
ENSG00000152377.8	SPOCK1	brown	0.877921499
ENSG00000107742.8	SPOCK2	turquoise	0.931691718
ENSG00000196104.6	SPOCK3	brown	0.791012868
ENSG00000152268.8	SPON1	green	0.885965115
ENSG00000159674.7	SPON2	turquoise	0.741644788
ENSG00000121067.13	SPOP	turquoise	0.87689479
ENSG00000144228.4	SPOPL	blue	0.768609152
ENSG00000118785.9	SPP1	blue	0.731265755
ENSG00000138600.5	SPPL2A	blue	0.828013999
ENSG00000005206.12	SPPL2B	yellow	0.939691803
ENSG00000157837.11	SPPL3	turquoise	0.959802686
ENSG00000116096.5	SPR	cyan	0.845194206
ENSG00000166068.8	SPRED1	turquoise	0.845141944
ENSG00000203772.6	SPRN	turquoise	0.910011418
ENSG00000163209.10	SPRR3	grey	0.067178442
ENSG00000010072.11	SPRTN	blue	0.907416588
ENSG00000164056.6	SPRY1	turquoise	0.748146584
ENSG00000136158.6	SPRY2	green	0.81195458
ENSG00000168939.6	SPRY3	turquoise	0.884450049
ENSG00000187678.8	SPRY4	grey	0.48087193
ENSG00000167778.4	SPRYD3	turquoise	0.92887411
ENSG00000176422.10	SPRYD4	turquoise	0.928411343
ENSG00000123178.10	SPRYD7	turquoise	0.97259242
ENSG00000171621.9	SPSB1	blue	0.705743246
ENSG00000111671.5	SPSB2	red	0.57443727
ENSG00000162032.11	SPSB3	turquoise	0.898696062
ENSG00000175093.4	SPSB4	greenyellow	0.799375472

ENSG00000197694.9	SPTAN1	turquoise	0.972848983
ENSG00000070182.13	SPTB	turquoise	0.914493873
ENSG00000115306.11	SPTBN1	brown	0.790154264
ENSG00000173898.7	SPTBN2	turquoise	0.889717622
ENSG00000160460.11	SPTBN4	turquoise	0.912553268
ENSG00000090054.9	SPTLC1	turquoise	0.918215875
ENSG00000100596.2	SPTLC2	brown	0.908680222
ENSG00000165389.6	SPTSSA	turquoise	0.785272938
ENSG00000196542.4	SPTSSB	turquoise	0.838711336
ENSG00000179119.10	SPTY2D1	turquoise	0.900181108
ENSG00000104549.7	SQLE	turquoise	0.911207192
ENSG00000137767.9	SQRDL	black	0.889780014
ENSG00000161011.15	SQSTM1	turquoise	0.785889601
ENSG00000213523.5	SRA1	turquoise	0.873004615
ENSG00000068784.8	SRBD1	blue	0.864508153
ENSG00000197122.7	SRC	turquoise	0.923919397
ENSG00000080603.12	SRCAP	turquoise	0.899626848
ENSG00000017373.11	SRCIN1	brown	0.882669629
ENSG00000146700.8	SRCRB4D	red	0.585803498
ENSG00000145545.7	SRD5A1	turquoise	0.941807732
ENSG00000128039.6	SRD5A3	brown	0.815584362
ENSG00000072310.12	SREBF1	red	0.605917626
ENSG00000198911.7	SREBF2	turquoise	0.946389204
ENSG00000153914.11	SREK1	turquoise	0.90832759
ENSG00000153006.10	SREK1IP1	turquoise	0.892906217
ENSG00000112658.6	SRF	turquoise	0.937199504
ENSG00000151304.5	SRFBP1	turquoise	0.815510106
ENSG00000196935.4	SRGAP1	blue	0.891741173
ENSG00000163486.8	SRGAP2	turquoise	0.905189381
ENSG00000196220.11	SRGAP3	turquoise	0.887120417
ENSG00000122862.4	SRGN	black	0.854901756
ENSG00000075142.9	SRI	blue	0.745192962
ENSG00000116649.5	SRM	turquoise	0.922685895
ENSG00000140319.6	SRP14	turquoise	0.898909776
ENSG00000248508.2	SRP14-AS1	blue	0.648179992
ENSG00000153037.8	SRP19	turquoise	0.962186279
ENSG00000100883.7	SRP54	turquoise	0.95695615
ENSG00000167881.10	SRP68	turquoise	0.959267767
ENSG00000174780.11	SRP72	turquoise	0.94153954
ENSG00000143742.8	SRP9	blue	0.869519733
ENSG00000096063.10	SRPK1	turquoise	0.920331683
ENSG00000135250.12	SRPK2	turquoise	0.956292255
ENSG00000184343.6	SRPK3	brown	0.800414206
ENSG00000182934.7	SRPR	turquoise	0.955015506
ENSG00000144867.7	SRPRB	turquoise	0.899821595
ENSG00000101955.10	SRPX	grey	0.333002125
ENSG00000167720.8	SRR	blue	0.85255587
ENSG00000100104.8	SRRD	turquoise	0.970959049

ENSG00000133226.12	SRRM1	turquoise	0.904104824
ENSG00000167978.12	SRRM2	yellow	0.941292105
ENSG00000177679.14	SRRM3	turquoise	0.840534463
ENSG00000139767.4	SRRM4	turquoise	0.8696685
ENSG00000226763.3	SRRM5	grey	0.785668994
ENSG00000087087.14	SRRT	turquoise	0.868458286
ENSG00000136450.8	SRSF1	turquoise	0.933002055
ENSG00000116754.9	SRSF11	turquoise	0.937749801
ENSG00000154548.8	SRSF12	turquoise	0.940248225
ENSG00000161547.10	SRSF2	turquoise	0.940115933
ENSG00000112081.12	SRSF3	turquoise	0.853580946
ENSG00000116350.11	SRSF4	turquoise	0.934712178
ENSG00000100650.11	SRSF5	turquoise	0.919734238
ENSG00000124193.10	SRSF6	turquoise	0.826039263
ENSG00000115875.14	SRSF7	turquoise	0.947698388
ENSG00000180771.10	SRSF8	turquoise	0.885770026
ENSG00000111786.4	SRSF9	turquoise	0.92776518
ENSG00000271303.1	SRXN1	turquoise	0.805869691
ENSG00000141380.9	SS18	blue	0.932830415
ENSG00000184402.10	SS18L1	turquoise	0.966054711
ENSG00000008324.6	SS18L2	turquoise	0.93431457
ENSG00000138385.11	SSB	turquoise	0.963427505
ENSG00000106028.6	SSBP1	turquoise	0.934182174
ENSG00000145687.11	SSBP2	turquoise	0.960836414
ENSG00000157216.11	SSBP3	turquoise	0.934255032
ENSG00000130511.11	SSBP4	turquoise	0.836335917
ENSG00000179954.10	SSC5D	midnightblue	0.692965266
ENSG00000138434.12	SSFA2	blue	0.787597822
ENSG00000084112.10	SSH1	turquoise	0.888789621
ENSG00000141298.13	SSH2	turquoise	0.900158093
ENSG00000172830.8	SSH3	blue	0.768823935
ENSG00000176101.7	SSNA1	red	0.907084139
ENSG00000123096.7	SSPN	green	0.832778451
ENSG00000197558.7	SSPO	midnightblue	0.781160333
ENSG00000124783.8	SSR1	turquoise	0.936375948
ENSG00000163479.9	SSR2	turquoise	0.930579696
ENSG00000114850.2	SSR3	turquoise	0.854467723
ENSG00000180879.9	SSR4	turquoise	0.883377835
ENSG00000149136.3	SSRP1	turquoise	0.956608261
ENSG00000173465.3	SSSCA1	turquoise	0.918868294
ENSG00000260233.2	SSSCA1-AS1	turquoise	0.89366589
ENSG00000157005.3	SST	turquoise	0.752280862
ENSG00000139874.5	SSTR1	turquoise	0.722770319
ENSG00000180616.4	SSTR2	turquoise	0.816115644
ENSG00000160075.10	SSU72	turquoise	0.944688713
ENSG00000117155.12	SSX2IP	turquoise	0.938647756
ENSG00000100380.8	ST13	turquoise	0.812158841
ENSG00000147488.7	ST18	brown	0.905633517

ENSG00000180953.7	ST20	turquoise	0.681388066
ENSG00000008513.10	ST3GAL1	blue	0.685120819
ENSG00000157350.8	ST3GAL2	turquoise	0.905796299
ENSG00000126091.15	ST3GAL3	turquoise	0.931206875
ENSG00000110080.14	ST3GAL4	brown	0.869744585
ENSG00000115525.12	ST3GAL5	turquoise	0.951282174
ENSG00000064225.8	ST3GAL6	brown	0.871558054
ENSG00000239445.1	ST3GAL6-AS1	brown	0.606688628
ENSG00000166444.13	ST5	blue	0.848784328
ENSG00000073849.10	ST6GAL1	magenta	0.856503619
ENSG00000144057.11	ST6GAL2	turquoise	0.59677031
ENSG00000070526.10	ST6GALNAC1	grey	0.357210794
ENSG00000184005.9	ST6GALNAC3	brown	0.759208711
ENSG00000136840.14	ST6GALNAC4	blue	0.751350633
ENSG00000117069.10	ST6GALNAC5	turquoise	0.819361646
ENSG00000160408.10	ST6GALNAC6	turquoise	0.875240481
ENSG00000004866.14	ST7	blue	0.875748256
ENSG00000227199.1	ST7-AS1	brown	0.558673777
ENSG00000007341.14	ST7L	turquoise	0.931087757
ENSG00000111728.6	ST8SIA1	pink	0.839073142
ENSG00000177511.5	ST8SIA3	turquoise	0.86820976
ENSG00000113532.8	ST8SIA4	turquoise	0.676292614
ENSG00000101638.9	ST8SIA5	turquoise	0.80783706
ENSG00000148488.11	ST8SIA6	grey	0.253096392
ENSG00000010327.6	STAB1	magenta	0.885324247
ENSG00000144681.6	STAC	turquoise	0.619885296
ENSG00000141750.6	STAC2	turquoise	0.858140665
ENSG00000185482.3	STAC3	magenta	0.641676341
ENSG00000118007.8	STAG1	blue	0.886764159
ENSG00000101972.14	STAG2	blue	0.904183717
ENSG00000066923.13	STAG3	blue	0.785944383
ENSG00000160828.13	STAG3L2	turquoise	0.827239864
ENSG00000106610.10	STAG3L4	turquoise	0.793888497
ENSG00000242294.2	STAG3L5P	turquoise	0.836155691
ENSG00000136738.10	STAM	turquoise	0.938013656
ENSG00000115145.5	STAM2	blue	0.943473803
ENSG00000124356.11	STAMBP	turquoise	0.877371813
ENSG00000138134.7	STAMBPL1	turquoise	0.870846689
ENSG00000178078.7	STAP2	turquoise	0.724006703
ENSG00000147465.7	STAR	turquoise	0.866535942
ENSG00000214530.3	STARD10	red	0.754502827
ENSG00000133121.16	STARD13	turquoise	0.798000416
ENSG00000131748.11	STARD3	turquoise	0.846410025
ENSG00000010270.9	STARD3NL	turquoise	0.929124432
ENSG00000164211.8	STARD4	turquoise	0.866965314
ENSG00000246859.2	STARD4-AS1	turquoise	0.740397723
ENSG00000172345.9	STARD5	turquoise	0.677223116
ENSG00000084090.9	STARD7	turquoise	0.93735426

ENSG00000130052.9	STARD8	black	0.841364364
ENSG00000159433.7	STARD9	brown	0.854558664
ENSG00000115415.14	STAT1	turquoise	0.774043902
ENSG00000170581.9	STAT2	yellow	0.93705717
ENSG00000168610.10	STAT3	blue	0.81649912
ENSG00000138378.13	STAT4	turquoise	0.839061783
ENSG00000126561.12	STAT5A	black	0.863524996
ENSG00000173757.5	STAT5B	turquoise	0.941699751
ENSG00000166888.6	STAT6	turquoise	0.851919307
ENSG00000124214.15	STAU1	turquoise	0.950985224
ENSG00000040341.13	STAU2	turquoise	0.950632381
ENSG00000159167.7	STC1	black	0.706791273
ENSG00000105889.10	STEAP1B	brown	0.740033882
ENSG00000157214.9	STEAP2	turquoise	0.925019124
ENSG00000115107.15	STEAP3	magenta	0.847871768
ENSG00000167323.5	STIM1	turquoise	0.945494851
ENSG00000109689.10	STIM2	turquoise	0.968452215
ENSG00000168439.12	STIP1	cyan	0.952043592
ENSG00000072786.8	STK10	blue	0.823890466
ENSG00000118046.10	STK11	turquoise	0.895594964
ENSG00000144589.16	STK11IP	yellow	0.950652959
ENSG00000115661.9	STK16	turquoise	0.940013154
ENSG00000164543.5	STK17A	turquoise	0.85242707
ENSG00000081320.6	STK17B	green	0.762483545
ENSG00000204344.10	STK19	turquoise	0.908897057
ENSG00000250535.1	STK19P	grey	0.330222908
ENSG00000102572.10	STK24	turquoise	0.96820707
ENSG00000115694.10	STK25	turquoise	0.958788595
ENSG00000104375.11	STK3	blue	0.782342734
ENSG00000196335.8	STK31	midnightblue	0.810679901
ENSG00000169302.10	STK32A	green	0.513071757
ENSG00000152953.8	STK32B	greenyellow	0.819175391
ENSG00000165752.12	STK32C	turquoise	0.89791774
ENSG00000125834.8	STK35	turquoise	0.938988112
ENSG00000163482.7	STK36	yellow	0.939582939
ENSG00000112079.8	STK38	yellow	0.84986812
ENSG00000211455.3	STK38L	blue	0.89900651
ENSG00000198648.6	STK39	brown	0.864107711
ENSG00000101109.7	STK4	turquoise	0.932245237
ENSG00000196182.6	STK40	blue	0.748396881
ENSG00000117632.16	STMN1	greenyellow	0.863563005
ENSG00000104435.9	STMN2	turquoise	0.896281706
ENSG00000197457.5	STMN3	turquoise	0.893000917
ENSG00000015592.12	STMN4	brown	0.785334999
ENSG00000148175.8	STOM	green	0.808879398
ENSG00000067221.9	STOML1	turquoise	0.935457763
ENSG00000165283.11	STOML2	turquoise	0.947703511
ENSG00000140022.5	STON2	green	0.716267435

ENSG00000165730.10	STOX1	green	0.881653464
ENSG00000173320.5	STOX2	turquoise	0.927742712
ENSG00000001460.13	STPG1	pink	0.885247322
ENSG00000169689.10	STRA13	red	0.922808406
ENSG00000266173.1	STRADA	turquoise	0.928652961
ENSG00000082146.8	STRADB	blue	0.914540743
ENSG00000023734.6	STRAP	turquoise	0.94741004
ENSG00000165209.14	STRBP	turquoise	0.932061495
ENSG00000143093.10	STRIP1	turquoise	0.894326642
ENSG00000128578.5	STRIP2	turquoise	0.879482428
ENSG00000115808.7	STRN	brown	0.825536575
ENSG00000196792.7	STRN3	turquoise	0.927849333
ENSG00000090372.10	STRN4	turquoise	0.938800754
ENSG00000101846.6	STS	turquoise	0.903359686
ENSG00000134910.8	STT3A	turquoise	0.888473455
ENSG00000163527.5	STT3B	turquoise	0.957896735
ENSG00000103266.6	STUB1	red	0.954553884
ENSG00000104915.10	STX10	blue	0.815801061
ENSG00000135604.9	STX11	black	0.810204935
ENSG00000117758.9	STX12	turquoise	0.940208823
ENSG00000124222.17	STX16	yellow	0.957119711
ENSG00000136874.6	STX17	blue	0.892590882
ENSG00000168818.5	STX18	turquoise	0.943168182
ENSG00000106089.7	STX1A	turquoise	0.857493361
ENSG00000099365.5	STX1B	turquoise	0.889864728
ENSG00000111450.9	STX2	blue	0.900211741
ENSG00000166900.10	STX3	turquoise	0.867675299
ENSG00000103496.10	STX4	blue	0.866956711
ENSG00000162236.7	STX5	turquoise	0.929333519
ENSG00000135823.9	STX6	turquoise	0.965875538
ENSG00000079950.9	STX7	turquoise	0.91019449
ENSG00000170310.10	STX8	turquoise	0.863007059
ENSG00000136854.13	STXBP1	turquoise	0.95492242
ENSG00000076944.10	STXBP2	turquoise	0.785899466
ENSG00000116266.6	STXBP3	blue	0.882366993
ENSG00000164506.10	STXBP5	turquoise	0.912072817
ENSG00000233452.2	STXBP5-AS1	turquoise	0.821173195
ENSG00000145087.8	STXBP5L	turquoise	0.903567757
ENSG00000168952.11	STXBP6	turquoise	0.721594915
ENSG00000060140.4	STYK1	turquoise	0.84897257
ENSG00000198252.7	STYX	turquoise	0.847111483
ENSG00000127952.12	STYXL1	cyan	0.843972922
ENSG00000113387.7	SUB1	turquoise	0.950602294
ENSG00000227203.2	SUB1P1	turquoise	0.56034174
ENSG00000136143.9	SUCLA2	turquoise	0.952421819
ENSG00000227848.1	SUCLA2-AS1	turquoise	0.719349813
ENSG00000163541.7	SUCLG1	turquoise	0.944826421
ENSG00000172340.10	SUCLG2	green	0.886427219

ENSG00000094975.9	SUCO	turquoise	0.948517885
ENSG00000111707.7	SUDS3	turquoise	0.970401256
ENSG00000107882.7	SUFU	turquoise	0.875540533
ENSG00000105705.11	SUGP1	turquoise	0.939399612
ENSG00000064607.12	SUGP2	turquoise	0.97580691
ENSG00000165416.10	SUGT1	turquoise	0.938559906
ENSG00000226823.1	SUGT1P	blue	0.618030123
ENSG00000239827.4	SUGT1P3	turquoise	0.828379871
ENSG00000137573.9	SULF1	pink	0.785784061
ENSG00000196562.10	SULF2	turquoise	0.761242027
ENSG00000196502.7	SULT1A1	turquoise	0.738758897
ENSG00000197165.6	SULT1A2	turquoise	0.63512167
ENSG00000198075.5	SULT1C4	green	0.874127093
ENSG00000130540.9	SULT4A1	turquoise	0.920350893
ENSG00000144455.9	SUMF1	turquoise	0.837990553
ENSG00000129103.13	SUMF2	turquoise	0.889352866
ENSG00000116030.12	SUMO1	turquoise	0.944415021
ENSG00000188612.7	SUMO2	turquoise	0.941812423
ENSG00000184900.11	SUMO3	turquoise	0.950996661
ENSG00000164828.13	SUN1	turquoise	0.934232694
ENSG00000100242.11	SUN2	brown	0.912594692
ENSG00000139531.8	SUOX	turquoise	0.862606622
ENSG00000092201.5	SUPT16H	turquoise	0.974894785
ENSG00000102710.15	SUPT20H	turquoise	0.88764448
ENSG00000196284.9	SUPT3H	turquoise	0.909693169
ENSG00000213246.2	SUPT4H1	turquoise	0.946230492
ENSG00000196235.9	SUPT5H	turquoise	0.942747719
ENSG00000109111.10	SUPT6H	turquoise	0.945056442
ENSG00000119760.11	SUPT7L	turquoise	0.973419509
ENSG00000156502.9	SUPV3L1	turquoise	0.904548105
ENSG00000148290.5	SURF1	red	0.849110245
ENSG00000148291.5	SURF2	red	0.933776213
ENSG00000148248.9	SURF4	turquoise	0.895549805
ENSG00000148296.5	SURF6	brown	0.74648165
ENSG00000106868.12	SUSD1	turquoise	0.926615226
ENSG00000099994.10	SUSD2	grey	0.496710822
ENSG00000157303.6	SUSD3	magenta	0.746399177
ENSG00000143502.10	SUSD4	turquoise	0.900479229
ENSG00000173705.4	SUSD5	greenyellow	0.776157618
ENSG00000101945.12	SUV39H1	turquoise	0.767820315
ENSG00000152455.11	SUV39H2	turquoise	0.862488899
ENSG00000110066.10	SUV420H1	turquoise	0.899434247
ENSG00000133247.9	SUV420H2	yellow	0.897936736
ENSG00000178691.6	SUZ12	turquoise	0.937567788
ENSG00000264538.2	SUZ12P	turquoise	0.940154838
ENSG00000159164.5	SV2A	turquoise	0.936184003
ENSG00000185518.7	SV2B	turquoise	0.869024897
ENSG00000122012.9	SV2C	turquoise	0.858235354

ENSG00000165124.13	SVEP1	brown	0.766594695
ENSG00000197321.10	SVIL	blue	0.673304641
ENSG00000198168.4	SVIP	turquoise	0.943376336
ENSG00000166111.5	SVOP	turquoise	0.90017843
ENSG00000133789.10	SWAP70	blue	0.734499246
ENSG00000175854.7	SWI5	turquoise	0.850973161
ENSG00000173928.2	SWSAP1	turquoise	0.707320811
ENSG00000116668.8	SWT1	turquoise	0.822554844
ENSG00000169895.5	SYAP1	turquoise	0.88518493
ENSG00000147642.12	SYBU	turquoise	0.91263222
ENSG00000171772.11	SYCE1	turquoise	0.780572414
ENSG00000205078.5	SYCE1L	turquoise	0.436268391
ENSG00000161860.7	SYCE2	yellow	0.825695578
ENSG00000217442.3	SYCE3	grey	0.282246716
ENSG00000196074.8	SYCP2	turquoise	0.690058534
ENSG00000153157.8	SYCP2L	turquoise	0.696239536
ENSG00000105137.8	SYDE1	black	0.767875431
ENSG00000117614.5	SYF2	turquoise	0.914168193
ENSG00000165025.10	SYK	magenta	0.943175855
ENSG00000125755.14	SYMPK	turquoise	0.946628847
ENSG00000008056.8	SYN1	turquoise	0.898590366
ENSG00000157152.12	SYN2	turquoise	0.890047575
ENSG00000185666.10	SYN3	turquoise	0.892696912
ENSG00000162520.10	SYNC	green	0.788555116
ENSG00000135316.13	SYNCRIP	turquoise	0.822189947
ENSG00000101463.5	SYNDIG1	midnightblue	0.892792056
ENSG00000183379.4	SYNDIG1L	turquoise	0.632835777
ENSG00000131018.18	SYNE1	turquoise	0.926378532
ENSG00000054654.11	SYNE2	blue	0.751847944
ENSG00000176438.8	SYNE3	blue	0.734109923
ENSG00000181392.10	SYNE4	greenyellow	0.656750901
ENSG00000197283.8	SYNGAP1	turquoise	0.911260972
ENSG00000100321.10	SYNGR1	turquoise	0.938176553
ENSG00000108639.3	SYNGR2	brown	0.820704
ENSG00000127561.10	SYNGR3	turquoise	0.901685873
ENSG00000159082.13	SYNJ1	turquoise	0.961708806
ENSG00000078269.9	SYNJ2	brown	0.833137461
ENSG00000182253.10	SYNM	blue	0.752944617
ENSG00000171992.8	SYNPO	turquoise	0.873092826
ENSG00000172403.6	SYNPO2	green	0.706359133
ENSG00000163630.6	SYNPR	turquoise	0.87119743
ENSG00000006114.11	SYNRG	turquoise	0.941787941
ENSG00000102003.6	SYP	turquoise	0.921232215
ENSG00000008282.3	SYPL1	brown	0.857141717
ENSG00000143028.7	SYPL2	green	0.621198772
ENSG00000204070.5	SYS1	turquoise	0.875823661
ENSG00000067715.9	SYT1	turquoise	0.922246318
ENSG00000132718.7	SYT11	turquoise	0.905011745

ENSG00000173227.9	SYT12	turquoise	0.858141128
ENSG00000019505.3	SYT13	greenyellow	0.933972784
ENSG00000143469.12	SYT14	turquoise	0.856348929
ENSG00000204176.9	SYT15	turquoise	0.765589146
ENSG00000103528.12	SYT17	blue	0.762685765
ENSG00000143858.7	SYT2	turquoise	0.812786434
ENSG00000213023.5	SYT3	turquoise	0.905457297
ENSG00000132872.7	SYT4	turquoise	0.916034004
ENSG00000129990.10	SYT5	turquoise	0.864382904
ENSG00000134207.10	SYT6	turquoise	0.870535904
ENSG00000011347.5	SYT7	turquoise	0.896161801
ENSG00000170743.12	SYT9	brown	0.816588522
ENSG00000137501.12	SYTL2	greenyellow	0.730473568
ENSG00000102362.11	SYTL4	green	0.620392665
ENSG00000147041.7	SYTL5	turquoise	0.902717792
ENSG00000162298.12	SYVN1	turquoise	0.890387091
ENSG00000055070.12	SZRD1	blue	0.887675501
ENSG00000198198.9	SZT2	yellow	0.966325411
ENSG00000100324.9	TAB1	turquoise	0.898332942
ENSG00000055208.13	TAB2	blue	0.891185899
ENSG00000157625.11	TAB3	turquoise	0.924609768
ENSG00000006128.7	TAC1	turquoise	0.822307634
ENSG00000166863.7	TAC3	greenyellow	0.793371717
ENSG00000147526.15	TACC1	turquoise	0.845105047
ENSG00000138162.13	TACC2	turquoise	0.946794982
ENSG00000013810.14	TACC3	blue	0.733137267
ENSG00000136463.7	TACO1	turquoise	0.928964987
ENSG00000115353.6	TACR1	midnightblue	0.887737196
ENSG00000075073.10	TACR2	turquoise	0.804600103
ENSG00000152382.5	TADA1	turquoise	0.966495045
ENSG00000108264.12	TADA2A	turquoise	0.966295152
ENSG00000173011.11	TADA2B	turquoise	0.971782358
ENSG00000171148.9	TADA3	blue	0.753916212
ENSG00000147133.11	TAF1	turquoise	0.873830028
ENSG00000166337.5	TAF10	red	0.938465425
ENSG00000064995.12	TAF11	turquoise	0.956995453
ENSG00000120656.7	TAF12	turquoise	0.83596737
ENSG00000197780.5	TAF13	blue	0.79342063
ENSG00000172660.7	TAF15	turquoise	0.913395845
ENSG00000143498.13	TAF1A	turquoise	0.8319198
ENSG00000115750.12	TAF1B	blue	0.844168583
ENSG00000103168.12	TAF1C	yellow	0.952786563
ENSG00000166012.10	TAF1D	turquoise	0.897288384
ENSG00000064313.7	TAF2	turquoise	0.930464558
ENSG00000165632.7	TAF3	turquoise	0.814880467
ENSG00000130699.12	TAF4	turquoise	0.91585532
ENSG00000141384.7	TAF4B	turquoise	0.939693899
ENSG00000148835.9	TAF5	turquoise	0.872986303

ENSG00000135801.5	TAF5L	turquoise	0.915119842
ENSG00000106290.10	TAF6	turquoise	0.96577042
ENSG00000162227.3	TAF6L	turquoise	0.872778124
ENSG00000178913.5	TAF7	turquoise	0.942364096
ENSG00000137413.11	TAF8	turquoise	0.869364202
ENSG00000085231.9	TAF9	turquoise	0.960541779
ENSG00000187325.4	TAF9B	turquoise	0.928473279
ENSG00000267692.1	TAF9P3	turquoise	0.657228996
ENSG00000149591.12	TAGLN	grey	0.604292946
ENSG00000158710.10	TAGLN2	black	0.933044875
ENSG00000144834.8	TAGLN3	turquoise	0.879443576
ENSG00000162367.7	TAL1	magenta	0.80692519
ENSG00000177156.6	TALDO1	turquoise	0.813018543
ENSG00000144559.6	TAMM41	turquoise	0.944899225
ENSG00000115183.9	TANC1	blue	0.84658754
ENSG00000170921.10	TANC2	turquoise	0.950339987
ENSG00000183597.11	TANGO2	turquoise	0.920868986
ENSG00000103047.3	TANGO6	turquoise	0.957846885
ENSG00000136560.9	TANK	turquoise	0.926911349
ENSG00000160551.5	TAOK1	turquoise	0.9285503
ENSG00000149930.13	TAOK2	turquoise	0.9600161
ENSG00000135090.9	TAOK3	turquoise	0.93691441
ENSG00000168394.9	TAP1	black	0.909286592
ENSG00000204267.9	TAP2	blue	0.836485799
ENSG00000231925.7	TAPBP	turquoise	0.892121479
ENSG00000139192.7	TAPBPL	blue	0.830748167
ENSG00000204261.4	TAPSAR1	magenta	0.7776415
ENSG00000169762.12	TAPT1	blue	0.909680527
ENSG00000263327.2	TAPT1-AS1	grey	0.678865175
ENSG00000059588.5	TARBP1	turquoise	0.874583069
ENSG00000139546.6	TARBP2	turquoise	0.922765526
ENSG00000120948.11	TARDBP	turquoise	0.941117862
ENSG00000113407.9	TARS	cyan	0.938524385
ENSG00000143374.10	TARS2	turquoise	0.95124522
ENSG00000185418.11	TARSL2	turquoise	0.914073795
ENSG00000212127.5	TAS2R14	turquoise	0.828962838
ENSG00000212125.2	TAS2R15	turquoise	0.848184411
ENSG00000255837.1	TAS2R20	yellow	0.732139328
ENSG00000127364.2	TAS2R4	turquoise	0.802237075
ENSG00000127366.4	TAS2R5	turquoise	0.747654034
ENSG00000089123.11	TASP1	turquoise	0.914660096
ENSG00000147687.12	TATDN1	turquoise	0.907811423
ENSG00000157014.6	TATDN2	turquoise	0.959585082
ENSG00000203705.6	TATDN3	turquoise	0.950801234
ENSG00000106052.9	TAX1BP1	turquoise	0.941418813
ENSG00000213977.3	TAX1BP3	brown	0.680448576
ENSG00000102125.11	TAZ	yellow	0.922740391
ENSG00000065882.11	TBC1D1	blue	0.848079511

ENSG00000099992.11	TBC1D10A	turquoise	0.698084286
ENSG00000169221.9	TBC1D10B	turquoise	0.912361246
ENSG00000175463.7	TBC1D10C	turquoise	0.824836713
ENSG00000108239.8	TBC1D12	brown	0.919003699
ENSG00000107021.11	TBC1D13	turquoise	0.949631327
ENSG00000121749.11	TBC1D15	turquoise	0.893880793
ENSG00000167291.11	TBC1D16	turquoise	0.894410695
ENSG00000104946.6	TBC1D17	turquoise	0.911236177
ENSG00000095383.15	TBC1D2	brown	0.859875696
ENSG00000125875.9	TBC1D20	turquoise	0.938397808
ENSG00000054611.9	TBC1D22A	turquoise	0.937604382
ENSG00000065491.8	TBC1D22B	turquoise	0.963273911
ENSG00000036054.8	TBC1D23	turquoise	0.896729659
ENSG00000162065.7	TBC1D24	turquoise	0.9107918
ENSG00000068354.11	TBC1D25	turquoise	0.962743778
ENSG00000214946.9	TBC1D26	turquoise	0.693690601
ENSG00000167202.7	TBC1D2B	blue	0.881138555
ENSG00000111490.8	TBC1D30	turquoise	0.92987094
ENSG00000156787.12	TBC1D31	turquoise	0.925455069
ENSG00000146350.9	TBC1D32	turquoise	0.901623451
ENSG00000185128.11	TBC1D3F	turquoise	0.799632368
ENSG00000136111.8	TBC1D4	turquoise	0.722559331
ENSG00000131374.10	TBC1D5	turquoise	0.913113758
ENSG00000145979.13	TBC1D7	turquoise	0.901011799
ENSG00000204634.8	TBC1D8	turquoise	0.843687218
ENSG00000133138.15	TBC1D8B	turquoise	0.74900986
ENSG00000109436.7	TBC1D9	turquoise	0.956687782
ENSG00000197226.8	TBC1D9B	turquoise	0.862437979
ENSG00000171530.9	TBCA	turquoise	0.933671692
ENSG00000105254.7	TBCB	red	0.956839291
ENSG00000124659.5	TBCC	turquoise	0.948959622
ENSG00000113838.8	TBCCD1	blue	0.917867606
ENSG00000141556.16	TBCD	turquoise	0.967425561
ENSG00000116957.8	TBCE	turquoise	0.948265491
ENSG00000154114.8	TBCEL	blue	0.822639621
ENSG00000145348.12	TBCK	turquoise	0.906512904
ENSG00000183735.5	TBK1	turquoise	0.918197216
ENSG00000198933.5	TBKBP1	greenyellow	0.815090328
ENSG00000101849.11	TBL1X	blue	0.884840709
ENSG00000177565.11	TBL1XR1	turquoise	0.960052648
ENSG00000106638.11	TBL2	turquoise	0.915223121
ENSG00000183751.10	TBL3	turquoise	0.930358899
ENSG00000112592.8	TBP	turquoise	0.962791866
ENSG00000028839.5	TBPL1	turquoise	0.932118021
ENSG00000154144.8	TBRG1	turquoise	0.921491672
ENSG00000136270.9	TBRG4	turquoise	0.937318042
ENSG00000112837.12	TBX18	grey	0.34019452
ENSG00000143178.8	TBX19	yellow	0.612749728

ENSG00000121068.9	TBX2	grey	0.473090469
ENSG00000135111.10	TBX3	grey	0.430307062
ENSG00000149922.6	TBX6	brown	0.534739654
ENSG00000059377.11	TBXAS1	magenta	0.94006095
ENSG00000179152.14	TCAIM	turquoise	0.95044508
ENSG00000173991.5	TCAP	grey	0.685296677
ENSG00000187735.8	TCEA1	blue	0.904349281
ENSG00000171703.12	TCEA2	turquoise	0.922398422
ENSG00000204219.5	TCEA3	green	0.602155822
ENSG00000172465.9	TCEAL1	turquoise	0.955176943
ENSG00000184905.4	TCEAL2	turquoise	0.928358299
ENSG00000196507.6	TCEAL3	red	0.889565472
ENSG00000133142.13	TCEAL4	turquoise	0.943829788
ENSG00000204065.2	TCEAL5	turquoise	0.915367853
ENSG00000204071.5	TCEAL6	turquoise	0.880133633
ENSG00000182916.7	TCEAL7	turquoise	0.884761426
ENSG00000180964.12	TCEAL8	turquoise	0.935148606
ENSG00000176896.7	TCEANC	turquoise	0.819874802
ENSG00000116205.6	TCEANC2	brown	0.795988837
ENSG00000154582.12	TCEB1	turquoise	0.934664029
ENSG00000103363.10	TCEB2	red	0.960920478
ENSG00000011007.8	TCEB3	turquoise	0.932019051
ENSG00000113649.7	TCERG1	turquoise	0.961954926
ENSG00000140262.13	TCF12	blue	0.910286478
ENSG00000137310.7	TCF19	blue	0.666957267
ENSG00000100207.14	TCF20	turquoise	0.969112528
ENSG00000141002.14	TCF25	turquoise	0.967030214
ENSG00000071564.10	TCF3	blue	0.888886274
ENSG00000196628.9	TCF4	blue	0.844023743
ENSG00000152284.4	TCF7L1	blue	0.721430068
ENSG00000148737.11	TCF7L2	green	0.807151211
ENSG00000101190.8	TCFL5	brown	0.673729156
ENSG00000139437.13	TCHP	turquoise	0.832810753
ENSG00000110719.5	TCIRG1	blue	0.64856939
ENSG00000185339.4	TCN2	black	0.646121601
ENSG00000070814.13	TCOF1	turquoise	0.899122783
ENSG00000120438.7	TCP1	cyan	0.939145767
ENSG00000242220.2	TCP10L	turquoise	0.803615013
ENSG00000166046.6	TCP11L2	blue	0.758408217
ENSG00000145022.4	TCTA	turquoise	0.897008327
ENSG00000146221.8	TCTE1	pink	0.934280873
ENSG00000213123.6	TCTEX1D2	turquoise	0.917620456
ENSG00000204852.11	TCTN1	pink	0.937719034
ENSG00000168778.7	TCTN2	pink	0.944199794
ENSG00000119977.16	TCTN3	turquoise	0.874139783
ENSG00000139372.10	TDG	turquoise	0.920144561
ENSG00000154316.10	TDH	pink	0.762805851
ENSG00000042088.9	TDP1	turquoise	0.938605656

ENSG00000111802.9	TDP2	turquoise	0.939990193
ENSG00000163239.8	TDRD10	blue	0.527388884
ENSG00000173809.11	TDRD12	turquoise	0.760367418
ENSG00000083544.9	TDRD3	turquoise	0.870023698
ENSG00000162782.11	TDRD5	turquoise	0.866981704
ENSG00000180113.11	TDRD6	turquoise	0.777217209
ENSG00000196116.6	TDRD7	blue	0.9098539
ENSG00000156414.14	TDRD9	turquoise	0.82052699
ENSG00000182134.11	TDRKH	turquoise	0.94845735
ENSG00000180190.7	TDRP	turquoise	0.920654529
ENSG00000187079.10	TEAD1	green	0.897443932
ENSG00000074219.9	TEAD2	black	0.817483712
ENSG00000007866.14	TEAD3	black	0.836731384
ENSG00000197905.4	TEAD4	black	0.850158064
ENSG00000205356.5	TECPR1	turquoise	0.959755529
ENSG00000196663.11	TECPR2	turquoise	0.888096413
ENSG00000099797.7	TECR	turquoise	0.845036791
ENSG00000109927.5	TECTA	midnightblue	0.906075777
ENSG00000167074.10	TEF	turquoise	0.926211011
ENSG00000172171.6	TEFM	turquoise	0.873765987
ENSG00000120156.16	TEK	black	0.545958985
ENSG00000125409.8	TEKT3	pink	0.805193598
ENSG00000188681.7	TEKT4P2	turquoise	0.481015441
ENSG00000100726.10	TELO2	turquoise	0.869539792
ENSG00000257949.2	TEN1	turquoise	0.634941976
ENSG00000111077.13	TENC1	brown	0.719443621
ENSG00000009694.9	TENM1	turquoise	0.80841994
ENSG00000145934.11	TENM2	turquoise	0.883787608
ENSG00000218336.3	TENM3	turquoise	0.884379079
ENSG00000149256.10	TENM4	turquoise	0.881604424
ENSG00000129566.8	TEP1	blue	0.896334086
ENSG00000147601.9	TERF1	turquoise	0.949971796
ENSG00000132604.6	TERF2	turquoise	0.983119605
ENSG00000166848.5	TERF2IP	turquoise	0.974618484
ENSG00000135269.13	TES	black	0.795063289
ENSG00000088992.13	TESC	turquoise	0.772671105
ENSG00000107140.11	TESK1	turquoise	0.953259076
ENSG00000070759.12	TESK2	brown	0.908497068
ENSG00000135426.10	TESPA1	midnightblue	0.769868199
ENSG00000168769.8	TET2	turquoise	0.842222399
ENSG00000187605.11	TET3	turquoise	0.80297602
ENSG00000136891.9	TEX10	turquoise	0.965082286
ENSG00000136478.3	TEX2	turquoise	0.813017872
ENSG00000175664.5	TEX26	pink	0.833459993
ENSG00000144043.7	TEX261	blue	0.912834485
ENSG00000164081.8	TEX264	red	0.903336075
ENSG00000153495.6	TEX29	red	0.475547096
ENSG00000151287.12	TEX30	turquoise	0.83088914

ENSG00000219435.3	TEX40	turquoise	0.754773
ENSG00000151575.10	TEX9	pink	0.875096325
ENSG00000091513.10	TF	brown	0.930852234
ENSG00000108064.6	TFAM	turquoise	0.894661614
ENSG00000230444.1	TFAMP1	turquoise	0.737150368
ENSG00000116819.6	TFAP2E	yellow	0.904490459
ENSG00000090447.7	TFAP4	turquoise	0.878447816
ENSG00000029639.6	TFB1M	turquoise	0.922200347
ENSG00000162851.6	TFB2M	turquoise	0.944976042
ENSG00000135457.5	TFCP2	turquoise	0.952784456
ENSG00000115112.7	TFCP2L1	green	0.475049392
ENSG00000198176.8	TFDP1	turquoise	0.875521174
ENSG00000114126.13	TFDP2	turquoise	0.924568359
ENSG00000068323.12	TFE3	turquoise	0.902868488
ENSG00000112561.13	TFEB	brown	0.869147491
ENSG00000114354.8	TFG	turquoise	0.961478922
ENSG00000100109.12	TFIP11	turquoise	0.910238751
ENSG00000003436.10	TFPI	black	0.685492495
ENSG00000105619.9	TFPT	red	0.908580514
ENSG00000106327.8	TFR2	turquoise	0.835950875
ENSG00000072274.8	TFRC	black	0.580820733
ENSG00000088451.6	TGDS	blue	0.906874065
ENSG00000163235.11	TGFA	brown	0.908551219
ENSG00000105329.5	TGFB1	magenta	0.838822333
ENSG00000140682.14	TGFB1I1	black	0.641848766
ENSG00000092969.7	TGFB2	green	0.841504525
ENSG00000119699.3	TGFB3	brown	0.542842181
ENSG00000120708.12	TGFBI	magenta	0.688103424
ENSG00000106799.8	TGFBR1	magenta	0.884581141
ENSG00000163513.13	TGFBR2	black	0.730393107
ENSG00000069702.6	TGFBR3	green	0.797254681
ENSG00000260001.2	TGFBR3L	turquoise	0.670949752
ENSG00000135966.8	TGFBRAP1	turquoise	0.952910596
ENSG00000118707.5	TGIF2	blue	0.722679495
ENSG00000092295.7	TGM1	yellow	0.80517302
ENSG00000198959.7	TGM2	black	0.839138897
ENSG00000152291.9	TGOLN2	turquoise	0.910006006
ENSG00000137574.6	TGS1	turquoise	0.927911652
ENSG00000180176.10	TH	turquoise	0.65795434
ENSG00000115970.14	THADA	turquoise	0.912028534
ENSG00000131931.4	THAP1	turquoise	0.839376978
ENSG00000129028.4	THAP10	turquoise	0.939552534
ENSG00000168286.1	THAP11	turquoise	0.950489773
ENSG00000173451.2	THAP2	turquoise	0.770890955
ENSG00000041988.11	THAP3	turquoise	0.924403812
ENSG00000176946.7	THAP4	turquoise	0.93686426
ENSG00000177683.9	THAP5	turquoise	0.893460645
ENSG00000174796.8	THAP6	turquoise	0.890717697

ENSG00000184436.7	THAP7	red	0.901793091
ENSG00000230513.1	THAP7-AS1	turquoise	0.665534002
ENSG00000161277.9	THAP8	brown	0.680377418
ENSG00000168152.8	THAP9	blue	0.859267756
ENSG00000251022.2	THAP9-AS1	turquoise	0.865339196
ENSG00000178726.6	THBD	black	0.701856271
ENSG00000137801.9	THBS1	black	0.670243291
ENSG00000186340.10	THBS2	brown	0.86519318
ENSG00000169231.9	THBS3	yellow	0.887242111
ENSG00000113296.10	THBS4	turquoise	0.751353425
ENSG00000159445.8	THEM4	turquoise	0.935410411
ENSG00000196407.7	THEM5	brown	0.687237167
ENSG00000130193.7	THEM6	turquoise	0.891404786
ENSG00000130775.11	THEMIS2	magenta	0.819389277
ENSG00000113272.9	THG1L	turquoise	0.84409711
ENSG00000185875.8	THNSL1	turquoise	0.919607551
ENSG00000144115.12	THNSL2	grey	0.306447266
ENSG00000079134.7	THOC1	yellow	0.94610865
ENSG00000125676.15	THOC2	turquoise	0.929252022
ENSG00000051596.5	THOC3	turquoise	0.867345336
ENSG00000100296.9	THOC5	turquoise	0.934604175
ENSG00000131652.9	THOC6	turquoise	0.841212578
ENSG00000163634.7	THOC7	turquoise	0.924808698
ENSG00000172009.10	THOP1	turquoise	0.928575066
ENSG00000090534.13	THPO	turquoise	0.480878017
ENSG00000126351.8	THRA	turquoise	0.897123013
ENSG00000054118.9	THRAP3	turquoise	0.952783648
ENSG00000151090.13	THRB	turquoise	0.900005995
ENSG00000136114.11	THSD1	green	0.756716514
ENSG00000187720.10	THSD4	black	0.591657385
ENSG00000005108.11	THSD7A	turquoise	0.865675515
ENSG00000259431.1	THTPA	blue	0.852456245
ENSG00000066654.8	THUMPD1	turquoise	0.974574958
ENSG00000138050.10	THUMPD2	turquoise	0.905036752
ENSG00000134077.11	THUMPD3	turquoise	0.917007496
ENSG00000154096.9	THY1	turquoise	0.863356487
ENSG00000151500.10	THYN1	turquoise	0.965724468
ENSG00000116001.11	TIA1	turquoise	0.880451712
ENSG00000221995.4	TIAF1	yellow	0.89280486
ENSG00000151923.13	TIAL1	turquoise	0.852390029
ENSG00000156299.8	TIAM1	turquoise	0.955476501
ENSG00000146426.13	TIAM2	turquoise	0.894975506
ENSG00000127666.8	TICAM1	turquoise	0.900930864
ENSG00000243414.4	TICAM2	blue	0.750101951
ENSG00000066056.9	TIE1	black	0.717509913
ENSG00000145365.10	TIFA	cyan	0.786009433
ENSG00000221944.3	TIGD1	yellow	0.900905149
ENSG00000180346.2	TIGD2	turquoise	0.886957325

ENSG00000173825.6	TIGD3	turquoise	0.86815327
ENSG00000179886.4	TIGD5	turquoise	0.827251914
ENSG00000164296.6	TIGD6	turquoise	0.903126975
ENSG00000140993.6	TIGD7	turquoise	0.870876724
ENSG00000111602.7	TIMELESS	blue	0.763790009
ENSG00000134809.4	TIMM10	turquoise	0.897647694
ENSG00000132286.7	TIMM10B	turquoise	0.834703203
ENSG00000099800.3	TIMM13	red	0.934836654
ENSG00000134375.6	TIMM17A	turquoise	0.92770505
ENSG00000126768.8	TIMM17B	turquoise	0.925012892
ENSG00000075336.7	TIMM21	turquoise	0.926433631
ENSG00000177370.4	TIMM22	turquoise	0.945447277
ENSG00000138297.9	TIMM23	turquoise	0.916738953
ENSG00000104980.3	TIMM44	turquoise	0.889574638
ENSG00000105197.6	TIMM50	turquoise	0.96420025
ENSG00000126953.5	TIMM8A	turquoise	0.92640637
ENSG00000150779.7	TIMM8B	red	0.941525507
ENSG00000100575.9	TIMM9	turquoise	0.935593802
ENSG00000113845.5	TIMMDC1	blue	0.904351427
ENSG00000102265.7	TIMP1	black	0.805542366
ENSG00000035862.8	TIMP2	turquoise	0.796540631
ENSG00000100234.11	TIMP3	green	0.86900765
ENSG00000157150.4	TIMP4	green	0.521443414
ENSG00000142910.11	TINAGL1	black	0.638828893
ENSG00000223573.2	TINCR	midnightblue	0.748073753
ENSG00000092330.11	TINF2	turquoise	0.86941333
ENSG00000163659.8	TIPARP	blue	0.70233522
ENSG00000075131.5	TIPIN	blue	0.794349183
ENSG00000143155.8	TIPRL	turquoise	0.950377212
ENSG00000150455.9	TIRAP	turquoise	0.878565165
ENSG00000137221.10	TJAP1	brown	0.923271819
ENSG00000104067.12	TJP1	blue	0.840777587
ENSG00000119139.12	TJP2	brown	0.875184762
ENSG00000167900.7	TK1	turquoise	0.801652631
ENSG00000166548.11	TK2	turquoise	0.918113157
ENSG00000163931.11	TKT	blue	0.814878177
ENSG00000160606.6	TLCD1	green	0.581993449
ENSG00000140950.11	TLDC1	turquoise	0.937606956
ENSG00000196781.9	TLE1	turquoise	0.856561098
ENSG00000228158.1	TLE1P1	turquoise	0.660146706
ENSG00000065717.10	TLE2	greenyellow	0.869327735
ENSG00000140332.11	TLE3	blue	0.628645099
ENSG00000106829.14	TLE4	turquoise	0.953633436
ENSG00000198586.9	TLK1	turquoise	0.966454831
ENSG00000146872.13	TLK2	turquoise	0.924285202
ENSG00000095587.8	TLL2	turquoise	0.90720262
ENSG00000137076.14	TLN1	blue	0.849445997
ENSG00000171914.10	TLN2	turquoise	0.953678186

ENSG00000137462.6	TLR2	magenta	0.873453237
ENSG00000136869.13	TLR4	green	0.865394372
ENSG00000187554.7	TLR5	magenta	0.815877852
ENSG00000239732.2	TLR9	grey	0.665692313
ENSG00000162604.8	TM2D1	turquoise	0.921792737
ENSG00000169490.12	TM2D2	turquoise	0.941130538
ENSG00000184277.8	TM2D3	turquoise	0.969757661
ENSG00000169908.6	TM4SF1	black	0.826665566
ENSG00000163762.2	TM4SF18	black	0.620238044
ENSG00000136404.11	TM6SF1	blue	0.785975751
ENSG00000149809.10	TM7SF2	turquoise	0.83342389
ENSG00000100926.10	TM9SF1	turquoise	0.813347167
ENSG00000125304.8	TM9SF2	turquoise	0.96159479
ENSG00000077147.10	TM9SF3	turquoise	0.888744624
ENSG00000101337.11	TM9SF4	turquoise	0.974351966
ENSG00000198498.5	TMA16	turquoise	0.896012317
ENSG00000232112.3	TMA7	red	0.935943205
ENSG00000135926.8	TMBIM1	black	0.820184243
ENSG00000155957.12	TMBIM4	turquoise	0.901759824
ENSG00000139644.8	TMBIM6	green	0.943529743
ENSG00000167608.7	TMC4	grey	0.458779148
ENSG00000141524.11	TMC6	brown	0.900954861
ENSG00000170537.8	TMC7	brown	0.928085281
ENSG00000167895.10	TMC8	magenta	0.875050307
ENSG00000172765.12	TMCC1	turquoise	0.94070421
ENSG00000271270.1	TMCC1-AS1	turquoise	0.849241586
ENSG00000133069.10	TMCC2	turquoise	0.821002854
ENSG00000057704.6	TMCC3	brown	0.896561547
ENSG00000143183.12	TMCO1	turquoise	0.795447997
ENSG00000150403.13	TMCO3	blue	0.81912436
ENSG00000162542.9	TMCO4	turquoise	0.78630008
ENSG00000113119.8	TMCO6	turquoise	0.840691384
ENSG00000099203.2	TMED1	blue	0.824145852
ENSG00000170348.4	TMED10	turquoise	0.884749589
ENSG00000086598.6	TMED2	turquoise	0.923006532
ENSG00000166557.8	TMED3	turquoise	0.904697965
ENSG00000158604.10	TMED4	turquoise	0.945138694
ENSG00000117500.8	TMED5	turquoise	0.775785015
ENSG00000134970.13	TMED7	turquoise	0.858984298
ENSG00000100580.7	TMED8	turquoise	0.917740788
ENSG00000184840.7	TMED9	turquoise	0.864771034
ENSG00000241697.3	TMEFF1	turquoise	0.85892467
ENSG00000144339.7	TMEFF2	turquoise	0.834485514
ENSG00000166292.7	TMEM100	green	0.466394118
ENSG00000091947.5	TMEM101	turquoise	0.922410993
ENSG00000181284.2	TMEM102	black	0.660956184
ENSG00000109066.9	TMEM104	blue	0.856067815
ENSG00000184988.4	TMEM106A	magenta	0.89236865

ENSG00000106460.14	TMEM106B	turquoise	0.87608908
ENSG00000134291.7	TMEM106C	turquoise	0.871477251
ENSG00000179029.10	TMEM107	pink	0.90131985
ENSG00000144868.9	TMEM108	green	0.560964814
ENSG00000110108.5	TMEM109	turquoise	0.785181613
ENSG00000178307.5	TMEM11	turquoise	0.906713901
ENSG00000213533.7	TMEM110	blue	0.902205628
ENSG00000126062.3	TMEM115	turquoise	0.884756948
ENSG00000198270.8	TMEM116	blue	0.872238018
ENSG00000139173.5	TMEM117	turquoise	0.864357086
ENSG00000183160.8	TMEM119	magenta	0.851094992
ENSG00000189077.6	TMEM120A	turquoise	0.923596435
ENSG00000188735.8	TMEM120B	turquoise	0.850859461
ENSG00000184986.6	TMEM121	turquoise	0.834445326
ENSG00000152558.10	TMEM123	blue	0.693653788
ENSG00000179178.6	TMEM125	brown	0.838255825
ENSG00000171202.2	TMEM126A	turquoise	0.898349599
ENSG00000171204.8	TMEM126B	turquoise	0.930968489
ENSG00000135956.4	TMEM127	turquoise	0.95415082
ENSG00000132406.7	TMEM128	blue	0.877896868
ENSG00000168936.6	TMEM129	turquoise	0.806089968
ENSG00000166448.10	TMEM130	turquoise	0.906327818
ENSG00000075568.12	TMEM131	turquoise	0.974624965
ENSG00000006118.10	TMEM132A	green	0.666243641
ENSG00000139364.6	TMEM132B	turquoise	0.898387869
ENSG00000181234.8	TMEM132C	green	0.644806911
ENSG00000151952.10	TMEM132D	turquoise	0.892136679
ENSG00000181291.5	TMEM132E	greenyellow	0.807575365
ENSG00000170647.2	TMEM133	green	0.664220407
ENSG00000172663.4	TMEM134	red	0.893845391
ENSG00000166575.12	TMEM135	blue	0.913024178
ENSG00000181264.4	TMEM136	blue	0.835547737
ENSG00000149483.7	TMEM138	pink	0.90591053
ENSG00000146859.6	TMEM140	brown	0.71663374
ENSG00000244187.3	TMEM141	red	0.924313125
ENSG00000161558.6	TMEM143	turquoise	0.9149542
ENSG00000164124.6	TMEM144	brown	0.915511143
ENSG00000167619.7	TMEM145	turquoise	0.842055615
ENSG00000105677.7	TMEM147	turquoise	0.884622579
ENSG00000096092.5	TMEM14A	turquoise	0.948362207
ENSG00000137210.9	TMEM14B	turquoise	0.911158527
ENSG00000111843.9	TMEM14C	blue	0.855360238
ENSG00000168890.9	TMEM150A	blue	0.788255429
ENSG00000249242.3	TMEM150C	blue	0.810992994
ENSG00000179292.4	TMEM151A	turquoise	0.819119002
ENSG00000178233.13	TMEM151B	greenyellow	0.923439955
ENSG00000249992.1	TMEM158	turquoise	0.800306261
ENSG00000011638.6	TMEM159	turquoise	0.799884839

ENSG00000130748.6	TMEM160	red	0.692038671
ENSG00000064545.10	TMEM161A	blue	0.818702501
ENSG00000164180.9	TMEM161B	turquoise	0.918934339
ENSG00000247828.3	TMEM161B-AS1	turquoise	0.931433331
ENSG00000152128.13	TMEM163	turquoise	0.836274139
ENSG00000157600.7	TMEM164	turquoise	0.843318208
ENSG00000134851.8	TMEM165	brown	0.921155667
ENSG00000174695.5	TMEM167A	turquoise	0.918222569
ENSG00000215717.5	TMEM167B	turquoise	0.960765393
ENSG00000146802.8	TMEM168	turquoise	0.897175354
ENSG00000163449.6	TMEM169	turquoise	0.85643535
ENSG00000186889.5	TMEM17	turquoise	0.933407785
ENSG00000166822.8	TMEM170A	blue	0.90881413
ENSG00000205269.4	TMEM170B	turquoise	0.912355667
ENSG00000157111.8	TMEM171	brown	0.7460134
ENSG00000184584.8	TMEM173	turquoise	0.831149064
ENSG00000127419.12	TMEM175	turquoise	0.888203193
ENSG00000002933.3	TMEM176A	black	0.606962202
ENSG00000106565.13	TMEM176B	black	0.661993759
ENSG00000144120.8	TMEM177	turquoise	0.937787757
ENSG00000152154.6	TMEM178A	turquoise	0.880164784
ENSG00000261115.1	TMEM178B	turquoise	0.93727802
ENSG00000258986.2	TMEM179	turquoise	0.818034422
ENSG00000185475.4	TMEM179B	blue	0.884439764
ENSG00000151353.10	TMEM18	turquoise	0.962166697
ENSG00000138111.10	TMEM180	turquoise	0.88020809
ENSG00000146433.8	TMEM181	turquoise	0.967970974
ENSG00000170417.10	TMEM182	turquoise	0.813461199
ENSG00000163444.7	TMEM183A	turquoise	0.960251787
ENSG00000227609.2	TMEM183AP1	turquoise	0.63395838
ENSG00000198792.8	TMEM184B	turquoise	0.845603356
ENSG00000164168.3	TMEM184C	turquoise	0.910923977
ENSG00000155984.10	TMEM185A	turquoise	0.903484096
ENSG00000226479.3	TMEM185B	turquoise	0.767194605
ENSG00000184857.7	TMEM186	turquoise	0.943265738
ENSG00000177854.7	TMEM187	blue	0.763865009
ENSG00000240849.6	TMEM189	blue	0.829291302
ENSG00000139291.9	TMEM19	turquoise	0.946069185
ENSG00000226287.3	TMEM191A	turquoise	0.77110569
ENSG00000206140.5	TMEM191C	turquoise	0.711983016
ENSG00000170088.9	TMEM192	blue	0.91549781
ENSG00000166881.5	TMEM194A	turquoise	0.867302714
ENSG00000189362.7	TMEM194B	brown	0.826302208
ENSG00000173452.9	TMEM196	turquoise	0.849374552
ENSG00000188760.6	TMEM198	turquoise	0.913363264
ENSG00000182796.8	TMEM198B	blue	0.857797628
ENSG00000244045.6	TMEM199	turquoise	0.947325228
ENSG00000135048.9	TMEM2	black	0.815345226

ENSG00000253304.1	TMEM200B	turquoise	0.777831656
ENSG00000188807.8	TMEM201	turquoise	0.840428133
ENSG00000187713.5	TMEM203	turquoise	0.959490674
ENSG00000131634.9	TMEM204	black	0.538386397
ENSG00000105518.9	TMEM205	turquoise	0.925006094
ENSG00000065600.8	TMEM206	brown	0.928628037
ENSG00000168701.14	TMEM208	red	0.94597672
ENSG00000146842.12	TMEM209	blue	0.891350755
ENSG00000119777.14	TMEM214	turquoise	0.894880757
ENSG00000187049.5	TMEM216	turquoise	0.725607249
ENSG00000150433.5	TMEM218	blue	0.883286354
ENSG00000149932.12	TMEM219	red	0.916621595
ENSG00000187824.4	TMEM220	blue	0.751399098
ENSG00000188051.6	TMEM221	grey	0.385055926
ENSG00000186501.10	TMEM222	turquoise	0.88072176
ENSG00000168569.7	TMEM223	turquoise	0.923158268
ENSG00000234224.1	TMEM229A	brown	0.758041113
ENSG00000198133.4	TMEM229B	turquoise	0.893923495
ENSG00000089063.10	TMEM230	blue	0.885962047
ENSG00000205084.6	TMEM231	pink	0.951530102
ENSG00000186952.10	TMEM232	pink	0.887764051
ENSG00000160055.15	TMEM234	pink	0.933604027
ENSG00000204278.8	TMEM235	brown	0.850491763
ENSG00000155755.14	TMEM237	blue	0.898022177
ENSG00000205090.4	TMEM240	turquoise	0.867208451
ENSG00000134490.9	TMEM241	turquoise	0.936492438
ENSG00000215712.6	TMEM242	turquoise	0.962901439
ENSG00000135185.7	TMEM243	turquoise	0.852701152
ENSG00000106771.8	TMEM245	turquoise	0.960128799
ENSG00000165152.4	TMEM246	turquoise	0.957806443
ENSG00000106609.12	TMEM248	turquoise	0.975452869
ENSG00000214597.4	TMEM249	turquoise	0.709392694
ENSG00000149582.11	TMEM25	turquoise	0.937056767
ENSG00000153485.5	TMEM251	turquoise	0.92437011
ENSG00000133678.9	TMEM254	pink	0.90217418
ENSG00000230091.2	TMEM254-AS1	turquoise	0.777875017
ENSG00000184497.8	TMEM255B	magenta	0.669120562
ENSG00000205544.3	TMEM256	red	0.915208987
ENSG00000187838.12	TMEM256-PLSCR3	yellow	0.887959882
ENSG00000221870.2	TMEM257	turquoise	0.808389812
ENSG00000134825.9	TMEM258	turquoise	0.784830121
ENSG00000182087.8	TMEM259	turquoise	0.881824176
ENSG00000070269.9	TMEM260	turquoise	0.928297722
ENSG00000137038.7	TMEM261	turquoise	0.936483328
ENSG00000147003.5	TMEM27	turquoise	0.686390069
ENSG00000112697.11	TMEM30A	turquoise	0.971604132
ENSG00000182107.5	TMEM30B	turquoise	0.74941687
ENSG00000179363.6	TMEM31	turquoise	0.578412787

ENSG00000109133.8	TMEM33	turquoise	0.968728238
ENSG00000126950.7	TMEM35	turquoise	0.910871514
ENSG00000072954.2	TMEM38A	turquoise	0.864502172
ENSG00000095209.7	TMEM38B	turquoise	0.783725435
ENSG00000176142.8	TMEM39A	blue	0.869084806
ENSG00000121775.13	TMEM39B	turquoise	0.916268171
ENSG00000163900.6	TMEM41A	turquoise	0.960454552
ENSG00000166471.6	TMEM41B	turquoise	0.902694284
ENSG00000169964.5	TMEM42	turquoise	0.897992569
ENSG00000170876.7	TMEM43	blue	0.925667652
ENSG00000145014.13	TMEM44	turquoise	0.754924695
ENSG00000231770.1	TMEM44-AS1	turquoise	0.768583613
ENSG00000181458.6	TMEM45A	turquoise	0.883166559
ENSG00000147027.3	TMEM47	blue	0.804247313
ENSG00000118600.7	TMEM5	turquoise	0.917185111
ENSG00000183726.6	TMEM50A	turquoise	0.937812518
ENSG00000142188.12	TMEM50B	blue	0.909096086
ENSG00000171729.9	TMEM51	green	0.566559736
ENSG00000178821.8	TMEM52	pink	0.754126516
ENSG00000165685.4	TMEM52B	midnightblue	0.807473739
ENSG00000126106.9	TMEM53	turquoise	0.912668183
ENSG00000121900.14	TMEM54	red	0.690781438
ENSG00000155099.3	TMEM55A	turquoise	0.902795324
ENSG00000165782.6	TMEM55B	turquoise	0.9750584
ENSG00000152078.5	TMEM56	turquoise	0.885665468
ENSG00000204178.5	TMEM57	turquoise	0.966239465
ENSG00000116209.7	TMEM59	blue	0.931729033
ENSG00000105696.4	TMEM59L	turquoise	0.90599541
ENSG00000135211.5	TMEM60	turquoise	0.916418132
ENSG00000137842.2	TMEM62	turquoise	0.940511384
ENSG00000196187.7	TMEM63A	brown	0.917629999
ENSG00000137216.14	TMEM63B	turquoise	0.966974099
ENSG00000165548.6	TMEM63C	turquoise	0.906136197
ENSG00000180694.9	TMEM64	turquoise	0.853855664
ENSG00000164983.6	TMEM65	greenyellow	0.890319245
ENSG00000133872.9	TMEM66	turquoise	0.945900477
ENSG00000164953.11	TMEM67	pink	0.969907137
ENSG00000167904.10	TMEM68	turquoise	0.923583875
ENSG00000159596.6	TMEM69	turquoise	0.921181069
ENSG00000175606.6	TMEM70	turquoise	0.938075003
ENSG00000164841.4	TMEM74	blue	0.630628797
ENSG00000125895.5	TMEM74B	turquoise	0.81403062
ENSG00000163472.14	TMEM79	blue	0.722125346
ENSG00000177042.10	TMEM80	turquoise	0.858755907
ENSG00000174529.6	TMEM81	turquoise	0.812201491
ENSG00000151117.4	TMEM86A	turquoise	0.850424539
ENSG00000180089.4	TMEM86B	grey	0.765798924
ENSG00000103978.11	TMEM87A	turquoise	0.808372526

ENSG00000153214.5	TMEM87B	turquoise	0.871936042
ENSG00000167874.6	TMEM88	grey	0.471317075
ENSG00000205116.3	TMEM88B	brown	0.777423289
ENSG00000129925.6	TMEM8A	turquoise	0.844773675
ENSG00000137103.12	TMEM8B	turquoise	0.92383347
ENSG00000116857.12	TMEM9	turquoise	0.928376173
ENSG00000142046.10	TMEM91	turquoise	0.850693048
ENSG00000109084.9	TMEM97	turquoise	0.890281078
ENSG00000006042.7	TMEM98	brown	0.858353094
ENSG00000167920.4	TMEM99	blue	0.813909152
ENSG00000175348.6	TMEM9B	blue	0.898274672
ENSG00000254860.1	TMEM9B-AS1	blue	0.651016042
ENSG00000144747.10	TMF1	turquoise	0.865603225
ENSG00000181585.3	TMIE	black	0.519108508
ENSG00000167664.4	TMIGD2	magenta	0.718996769
ENSG00000185973.6	TMLHE	blue	0.851021753
ENSG00000136842.9	TMOD1	turquoise	0.926515796
ENSG00000128872.5	TMOD2	turquoise	0.887648844
ENSG00000138594.8	TMOD3	turquoise	0.846813386
ENSG00000120802.9	TMPO	turquoise	0.777258297
ENSG00000160183.9	TMPRSS3	green	0.623798338
ENSG00000166682.6	TMPRSS5	blue	0.586389431
ENSG00000187045.12	TMPRSS6	turquoise	0.696243548
ENSG00000034510.4	TMSB10	turquoise	0.85861742
ENSG00000205542.6	TMSB4X	turquoise	0.863705428
ENSG00000188765.6	TMSB4XP2	grey	0.313291762
ENSG00000223551.1	TMSB4XP4	red	0.634157773
ENSG00000187653.10	TMSB4XP8	turquoise	0.735564597
ENSG00000154620.5	TMSB4Y	grey	0.235942208
ENSG00000133687.11	TMTC1	turquoise	0.924724014
ENSG00000179104.4	TMTC2	brown	0.925925165
ENSG00000139324.7	TMTC3	turquoise	0.907757246
ENSG00000125247.11	TMTC4	brown	0.938655288
ENSG00000164897.8	TMUB1	red	0.843521723
ENSG00000168591.11	TMUB2	turquoise	0.951637598
ENSG00000139921.8	TMX1	green	0.807140888
ENSG00000213593.5	TMX2	turquoise	0.918925245
ENSG00000166479.5	TMX3	turquoise	0.959632888
ENSG00000125827.4	TMX4	turquoise	0.946357256
ENSG00000109079.5	TNFAIP1	turquoise	0.889173008
ENSG00000185215.4	TNFAIP2	turquoise	0.870247875
ENSG00000118503.10	TNFAIP3	black	0.738206485
ENSG00000123610.3	TNFAIP6	brown	0.652034071
ENSG00000185361.4	TNFAIP8L1	turquoise	0.648432255
ENSG00000163154.5	TNFAIP8L2	magenta	0.859513288
ENSG00000104689.5	TNFRSF10A	black	0.871748646
ENSG00000120889.8	TNFRSF10B	black	0.904534801
ENSG00000173530.5	TNFRSF10D	black	0.80622784

ENSG00000006327.9	TNFRSF12A	black	0.732933655
ENSG00000157873.13	TNFRSF14	blue	0.60238558
ENSG00000127863.11	TNFRSF19	pink	0.788574609
ENSG00000067182.3	TNFRSF1A	black	0.865441982
ENSG00000028137.12	TNFRSF1B	magenta	0.831621827
ENSG00000146072.6	TNFRSF21	turquoise	0.849561645
ENSG00000215788.5	TNFRSF25	yellow	0.82329037
ENSG00000243509.4	TNFRSF6B	yellow	0.587033365
ENSG00000120949.10	TNFRSF8	turquoise	0.752533744
ENSG00000121858.6	TNFSF10	grey	0.26675413
ENSG00000239697.6	TNFSF12	red	0.804677385
ENSG00000161955.12	TNFSF13	green	0.823020958
ENSG00000102524.7	TNFSF13B	magenta	0.712221553
ENSG00000117586.6	TNFSF4	blue	0.770395144
ENSG00000125657.3	TNFSF9	blue	0.618819807
ENSG00000154310.12	TNIK	green	0.900992928
ENSG00000145901.10	TNIP1	turquoise	0.834850289
ENSG00000168884.10	TNIP2	black	0.879505701
ENSG00000174292.8	TNK1	turquoise	0.801918865
ENSG00000061938.12	TNK2	turquoise	0.87276965
ENSG00000173273.11	TNKS	turquoise	0.946641592
ENSG00000149115.9	TNKS1BP1	turquoise	0.852446552
ENSG00000107854.5	TNKS2	turquoise	0.944177627
ENSG00000228701.1	TNKS2-AS1	grey	0.292822424
ENSG00000114854.3	TNNC1	grey	0.185346971
ENSG00000101470.5	TNNC2	turquoise	0.742298552
ENSG00000116783.10	TNNI3K	turquoise	0.822698453
ENSG00000105048.12	TNNT1	grey	0.32024848
ENSG00000083312.13	TNPO1	turquoise	0.857893326
ENSG00000105576.11	TNPO2	turquoise	0.961944376
ENSG00000064419.9	TNPO3	turquoise	0.935486903
ENSG00000116147.12	TNR	greenyellow	0.703280478
ENSG00000182095.10	TNRC18	turquoise	0.905644343
ENSG00000090905.13	TNRC6A	turquoise	0.903099469
ENSG00000100354.16	TNRC6B	turquoise	0.913822336
ENSG00000078687.12	TNRC6C	turquoise	0.932094845
ENSG00000204282.3	TNRC6C-AS1	brown	0.863505914
ENSG00000079308.12	TNS1	blue	0.656635089
ENSG00000136205.12	TNS3	green	0.593341742
ENSG00000168477.13	TNXB	turquoise	0.816237203
ENSG00000229980.4	TOB1-AS1	turquoise	0.907512533
ENSG00000183864.4	TOB2	blue	0.85813668
ENSG00000132773.7	TOE1	turquoise	0.909901012
ENSG00000078902.11	TOLLIP	turquoise	0.954862111
ENSG00000255153.1	TOLLIP-AS1	turquoise	0.761271065
ENSG00000100284.16	TOM1	turquoise	0.93007571
ENSG00000141198.9	TOM1L1	turquoise	0.876141945
ENSG00000175662.13	TOM1L2	turquoise	0.903971107

ENSG00000173726.6	TOMM20	turquoise	0.965114311
ENSG00000100216.4	TOMM22	turquoise	0.935500875
ENSG00000025772.7	TOMM34	turquoise	0.919165437
ENSG00000130204.8	TOMM40	turquoise	0.914736209
ENSG00000158882.8	TOMM40L	turquoise	0.950715298
ENSG00000175768.8	TOMM5	turquoise	0.932148256
ENSG00000196683.6	TOMM7	red	0.941249576
ENSG00000154174.7	TOMM70A	turquoise	0.943109884
ENSG00000160949.12	TONSL	turquoise	0.80337797
ENSG00000198900.5	TOP1	turquoise	0.944001998
ENSG00000184428.8	TOP1MT	turquoise	0.902938974
ENSG00000077097.9	TOP2B	turquoise	0.932679239
ENSG00000177302.10	TOP3A	turquoise	0.944315492
ENSG00000100038.15	TOP3B	turquoise	0.893256284
ENSG00000163781.8	TOPBP1	turquoise	0.954295083
ENSG00000197579.3	TOPORS	turquoise	0.941406271
ENSG00000235453.3	TOPORS-AS1	red	0.916214355
ENSG00000136827.11	TOR1A	turquoise	0.979777896
ENSG00000143337.14	TOR1AIP1	blue	0.917301205
ENSG00000169905.8	TOR1AIP2	turquoise	0.910220243
ENSG00000136816.11	TOR1B	turquoise	0.93851402
ENSG00000160404.13	TOR2A	turquoise	0.90414586
ENSG00000186283.9	TOR3A	turquoise	0.911651947
ENSG00000198113.2	TOR4A	black	0.792329299
ENSG00000198846.5	TOX	green	0.815508217
ENSG00000124191.13	TOX2	greenyellow	0.790991662
ENSG00000103460.12	TOX3	greenyellow	0.836800066
ENSG00000092203.9	TOX4	turquoise	0.962262276
ENSG00000141510.11	TP53	blue	0.66550726
ENSG00000067369.9	TP53BP1	turquoise	0.944297955
ENSG00000143514.12	TP53BP2	green	0.926919147
ENSG00000175274.14	TP53I11	turquoise	0.740452851
ENSG00000167543.11	TP53I13	blue	0.673965658
ENSG00000115129.9	TP53I3	brown	0.540898345
ENSG00000164938.9	TP53INP1	blue	0.749746257
ENSG00000078804.8	TP53INP2	brown	0.515995835
ENSG00000172315.5	TP53RK	turquoise	0.875584788
ENSG00000182165.13	TP53TG1	red	0.882987542
ENSG00000124251.6	TP53TG5	brown	0.887229337
ENSG00000227372.6	TP73-AS1	turquoise	0.9062396
ENSG00000146242.5	TPBG	turquoise	0.742355346
ENSG00000261594.3	TPBGL	turquoise	0.845514328
ENSG00000186815.8	TPCN1	green	0.767054176
ENSG00000162341.11	TPCN2	yellow	0.896852433
ENSG00000076554.11	TPD52	turquoise	0.929680398
ENSG00000111907.16	TPD52L1	turquoise	0.801593259
ENSG00000101150.13	TPD52L2	turquoise	0.906308845
ENSG00000141933.8	TPGS1	turquoise	0.674034503

ENSG00000134779.10	TPGS2	turquoise	0.927214259
ENSG00000111669.10	TPI1	turquoise	0.93383369
ENSG00000196511.9	TPK1	turquoise	0.862954785
ENSG00000140416.15	TPM1	turquoise	0.913843714
ENSG00000198467.9	TPM2	grey	0.72686255
ENSG00000143549.15	TPM3	turquoise	0.952715384
ENSG00000241015.2	TPM3P9	turquoise	0.604135346
ENSG00000167460.10	TPM4	blue	0.793148807
ENSG00000137364.4	TPMT	turquoise	0.930342735
ENSG00000166340.10	TPP1	green	0.887249619
ENSG00000134900.7	TPP2	turquoise	0.930548037
ENSG00000171368.10	TPPP	brown	0.819956374
ENSG00000047410.9	TPR	turquoise	0.912817998
ENSG00000163870.10	TPRA1	blue	0.857046577
ENSG00000158109.10	TPRG1L	turquoise	0.952482211
ENSG00000144034.10	TPRKB	turquoise	0.88681049
ENSG00000176058.7	TPRN	brown	0.8866287
ENSG00000116176.6	TPSG1	turquoise	0.553380017
ENSG00000169902.9	TPST1	cyan	0.841804038
ENSG00000128294.11	TPST2	cyan	0.765659669
ENSG00000133112.12	TPT1	red	0.89379118
ENSG00000170919.11	TPT1-AS1	turquoise	0.909604811
ENSG00000253771.1	TPTE2P1	turquoise	0.757634126
ENSG00000168852.8	TPTE2P5	turquoise	0.872137203
ENSG00000100181.17	TPTEP1	brown	0.837040761
ENSG00000088325.11	TPX2	turquoise	0.870155638
ENSG00000164548.6	TRA2A	turquoise	0.899094139
ENSG00000136527.13	TRA2B	turquoise	0.886847056
ENSG00000170638.5	TRABD	turquoise	0.867081027
ENSG00000229164.5	TRAC	turquoise	0.799511011
ENSG00000102871.11	TRADD	blue	0.681030479
ENSG00000056558.6	TRAF1	blue	0.78200787
ENSG00000127191.13	TRAF2	turquoise	0.854146852
ENSG00000131323.10	TRAF3	blue	0.872792439
ENSG00000204104.7	TRAF3IP1	turquoise	0.875269082
ENSG00000056972.14	TRAF3IP2	cyan	0.80716345
ENSG00000231889.3	TRAF3IP2-AS1	turquoise	0.936349288
ENSG00000076604.10	TRAF4	brown	0.660851558
ENSG00000082512.10	TRAF5	turquoise	0.815643704
ENSG00000175104.10	TRAF6	turquoise	0.839981524
ENSG00000131653.8	TRAF7	turquoise	0.939131631
ENSG00000135148.7	TRAFD1	turquoise	0.819056114
ENSG00000183763.4	TRAIP	turquoise	0.778941996
ENSG00000182606.10	TRAK1	turquoise	0.932116224
ENSG00000115993.7	TRAK2	brown	0.704523637
ENSG00000067167.3	TRAM1	blue	0.855056144
ENSG00000174599.4	TRAM1L1	turquoise	0.864526945
ENSG00000065308.4	TRAM2	black	0.84770072

ENSG00000225791.2	TRAM2-AS1	brown	0.680804324
ENSG00000168016.9	TRANK1	turquoise	0.890870612
ENSG00000126602.6	TRAP1	turquoise	0.969659332
ENSG00000170043.7	TRAPPC1	turquoise	0.89283368
ENSG00000160218.8	TRAPPC10	turquoise	0.939301654
ENSG00000168538.11	TRAPPC11	turquoise	0.971756284
ENSG00000171853.11	TRAPPC12	turquoise	0.935084577
ENSG00000225234.1	TRAPPC12-AS1	turquoise	0.864983159
ENSG00000113597.13	TRAPPC13	turquoise	0.960101555
ENSG00000196459.9	TRAPPC2	turquoise	0.955269684
ENSG00000167515.6	TRAPPC2L	turquoise	0.918563499
ENSG00000256060.2	TRAPPC2P1	turquoise	0.884276654
ENSG00000054116.7	TRAPPC3	turquoise	0.974915589
ENSG00000196655.5	TRAPPC4	turquoise	0.915426688
ENSG00000181029.7	TRAPPC5	red	0.860934803
ENSG00000007255.6	TRAPPC6A	red	0.87898514
ENSG00000182400.10	TRAPPC6B	turquoise	0.962898735
ENSG00000153339.9	TRAPPC8	turquoise	0.961703336
ENSG00000167632.10	TRAPPC9	turquoise	0.933078937
ENSG00000211772.4	TRBC2	grey	0.254389836
ENSG00000211750.2	TRBV24-1	turquoise	0.703800536
ENSG00000211751.3	TRBV25-1	turquoise	0.722314733
ENSG00000107614.17	TRDMT1	turquoise	0.837532305
ENSG00000095970.12	TREM2	magenta	0.903041187
ENSG00000124496.8	TRERF1	turquoise	0.914736988
ENSG00000213689.5	TREX1	blue	0.657941108
ENSG00000211689.2	TRGC1	grey	0.256492469
ENSG00000227191.2	TRGC2	grey	0.223161006
ENSG00000211692.1	TRGJP1	grey	0.151638564
ENSG00000211688.1	TRGJP2	grey	0.232342928
ENSG00000170893.3	TRH	greenyellow	0.755872481
ENSG00000072657.4	TRHDE	greenyellow	0.80610564
ENSG00000236333.3	TRHDE-AS1	turquoise	0.631020244
ENSG00000170855.3	TRIAP1	turquoise	0.908053267
ENSG00000173334.3	TRIB1	green	0.632227808
ENSG00000071575.7	TRIB2	green	0.597862489
ENSG00000101255.6	TRIB3	grey	0.15455749
ENSG00000154370.9	TRIM11	turquoise	0.876136755
ENSG00000204977.5	TRIM13	turquoise	0.875328038
ENSG00000106785.10	TRIM14	blue	0.845086556
ENSG00000221926.7	TRIM16	cyan	0.896864387
ENSG00000108448.16	TRIM16L	blue	0.788877368
ENSG00000162931.7	TRIM17	turquoise	0.866210313
ENSG00000109654.10	TRIM2	brown	0.847263246
ENSG00000132109.8	TRIM21	blue	0.695102165
ENSG00000132274.11	TRIM22	black	0.869831738
ENSG00000113595.10	TRIM23	turquoise	0.951653832
ENSG00000122779.12	TRIM24	turquoise	0.952921375

ENSG00000121060.10	TRIM25	turquoise	0.854267681
ENSG00000234127.4	TRIM26	cyan	0.873367683
ENSG00000204713.6	TRIM27	turquoise	0.939979837
ENSG00000130726.7	TRIM28	turquoise	0.911207553
ENSG00000110171.14	TRIM3	turquoise	0.955547169
ENSG00000119401.10	TRIM32	turquoise	0.934219203
ENSG00000197323.6	TRIM33	turquoise	0.939360188
ENSG00000104228.8	TRIM35	turquoise	0.95888447
ENSG00000152503.5	TRIM36	greenyellow	0.815907647
ENSG00000108395.9	TRIM37	turquoise	0.965943471
ENSG00000112343.8	TRIM38	black	0.831068896
ENSG00000204599.10	TRIM39	turquoise	0.909921987
ENSG00000146833.11	TRIM4	blue	0.915709658
ENSG00000146063.14	TRIM41	brown	0.731485765
ENSG00000134253.5	TRIM45	yellow	0.872776013
ENSG00000163462.13	TRIM46	turquoise	0.888134629
ENSG00000132481.2	TRIM47	black	0.705116567
ENSG00000132256.14	TRIM5	black	0.90252477
ENSG00000255190.2	TRIM51DP	turquoise	0.528862513
ENSG00000183718.4	TRIM52	turquoise	0.915253325
ENSG00000248275.1	TRIM52-AS1	turquoise	0.749008562
ENSG00000169871.8	TRIM56	blue	0.729075435
ENSG00000213186.3	TRIM59	brown	0.896106436
ENSG00000227986.1	TRIM60P18	turquoise	0.897593168
ENSG00000116525.9	TRIM62	brown	0.878933115
ENSG00000141569.6	TRIM65	blue	0.874760893
ENSG00000166436.11	TRIM66	turquoise	0.89138391
ENSG00000119283.11	TRIM67	turquoise	0.756235561
ENSG00000167333.8	TRIM68	turquoise	0.931738768
ENSG00000185880.8	TRIM69	turquoise	0.908446207
ENSG00000146054.13	TRIM7	yellow	0.83877955
ENSG00000206557.5	TRIM71	turquoise	0.812727895
ENSG00000155428.8	TRIM74	turquoise	0.737950648
ENSG00000171206.9	TRIM8	turquoise	0.87541459
ENSG00000100505.9	TRIM9	turquoise	0.937343196
ENSG00000038382.13	TRIO	turquoise	0.923890329
ENSG00000100106.15	TRIOBP	blue	0.832800605
ENSG00000125733.13	TRIP10	black	0.863568443
ENSG00000100815.8	TRIP11	blue	0.905095359
ENSG00000153827.9	TRIP12	turquoise	0.936435062
ENSG00000103671.5	TRIP4	turquoise	0.873325478
ENSG00000087077.7	TRIP6	green	0.722273219
ENSG00000043514.11	TRIT1	turquoise	0.931135696
ENSG00000104907.8	TRMT1	turquoise	0.874892282
ENSG00000145331.9	TRMT10A	turquoise	0.752817544
ENSG00000165275.5	TRMT10B	turquoise	0.893737101
ENSG00000174173.6	TRMT10C	turquoise	0.92469726
ENSG00000066651.13	TRMT11	turquoise	0.916329046

ENSG00000173113.2	TRMT112	turquoise	0.886730879
ENSG00000183665.4	TRMT12	turquoise	0.916862452
ENSG00000122435.5	TRMT13	brown	0.857640641
ENSG00000121486.7	TRMT1L	turquoise	0.934257065
ENSG00000099899.10	TRMT2A	turquoise	0.907273789
ENSG00000188917.10	TRMT2B	turquoise	0.903890671
ENSG00000155275.14	TRMT44	turquoise	0.902426741
ENSG00000126814.6	TRMT5	turquoise	0.94609655
ENSG00000089195.10	TRMT6	turquoise	0.953921842
ENSG00000166166.8	TRMT61A	turquoise	0.87927893
ENSG00000171103.6	TRMT61B	turquoise	0.930819436
ENSG00000100416.8	TRMU	turquoise	0.902720545
ENSG00000180098.5	TRNAU1AP	turquoise	0.866133738
ENSG00000253368.3	TRNP1	turquoise	0.899415972
ENSG00000072756.12	TRNT1	turquoise	0.935068745
ENSG00000067445.16	TRO	turquoise	0.918256197
ENSG00000116747.8	TROVE2	blue	0.900750731
ENSG00000144935.10	TRPC1	turquoise	0.900773383
ENSG00000138741.6	TRPC3	midnightblue	0.789173288
ENSG00000100991.7	TRPC4AP	turquoise	0.933485401
ENSG00000142185.12	TRPM2	turquoise	0.851146271
ENSG00000083067.18	TRPM3	green	0.689444679
ENSG00000130529.11	TRPM4	blue	0.736407126
ENSG00000092439.9	TRPM7	turquoise	0.887360664
ENSG00000104447.7	TRPS1	green	0.875162477
ENSG00000149743.9	TRPT1	red	0.855358405
ENSG00000196689.6	TRPV1	yellow	0.887089105
ENSG00000187688.10	TRPV2	blue	0.660155351
ENSG00000167723.10	TRPV3	yellow	0.814572455
ENSG00000165125.13	TRPV6	turquoise	0.78055909
ENSG00000196367.8	TRRAP	turquoise	0.949617442
ENSG00000165832.4	TRUB1	turquoise	0.950078662
ENSG00000167112.7	TRUB2	turquoise	0.887576796
ENSG00000165699.9	TSC1	turquoise	0.946926919
ENSG00000103197.12	TSC2	turquoise	0.928361046
ENSG00000102804.10	TSC22D1	green	0.613532016
ENSG00000196428.8	TSC22D2	turquoise	0.879933548
ENSG00000157514.12	TSC22D3	green	0.607005395
ENSG00000166925.4	TSC22D4	brown	0.567049707
ENSG00000198860.7	TSEN15	turquoise	0.857982918
ENSG00000154743.13	TSEN2	turquoise	0.974668219
ENSG00000170892.6	TSEN34	blue	0.843207048
ENSG00000182173.8	TSEN54	yellow	0.917024694
ENSG00000123297.12	TSFM	turquoise	0.952524221
ENSG00000074319.8	TSG101	turquoise	0.943362362
ENSG00000135951.10	TSGA10	pink	0.889779485
ENSG00000179981.9	TSHZ1	turquoise	0.880031798
ENSG00000121297.6	TSHZ3	turquoise	0.8375857

ENSG00000182704.6	TSKU	black	0.615248209
ENSG00000211460.7	TSN	turquoise	0.958702399
ENSG00000171045.10	TSNARE1	turquoise	0.895099403
ENSG00000116918.9	TSNAX	turquoise	0.941385214
ENSG00000102904.10	TSNAXIP1	pink	0.936512542
ENSG00000110900.10	TSPAN11	grey	0.379215462
ENSG00000106025.4	TSPAN12	green	0.660020505
ENSG00000106537.7	TSPAN13	turquoise	0.913201033
ENSG00000108219.10	TSPAN14	blue	0.861265965
ENSG00000099282.5	TSPAN15	brown	0.875757205
ENSG00000048140.13	TSPAN17	turquoise	0.909148236
ENSG00000157570.7	TSPAN18	brown	0.658408264
ENSG00000231738.6	TSPAN19	blue	0.618840215
ENSG00000134198.5	TSPAN2	turquoise	0.6844714
ENSG00000140391.10	TSPAN3	turquoise	0.895272518
ENSG00000135452.5	TSPAN31	turquoise	0.927750943
ENSG00000158457.4	TSPAN33	blue	0.71804444
ENSG00000214063.6	TSPAN4	blue	0.6576424
ENSG00000168785.3	TSPAN5	turquoise	0.957889107
ENSG00000156298.8	TSPAN7	turquoise	0.911745281
ENSG00000011105.7	TSPAN9	turquoise	0.79921638
ENSG00000100300.13	TSPO	black	0.700850748
ENSG00000235217.5	TSPY26P	turquoise	0.908580979
ENSG00000189241.6	TSPYL1	turquoise	0.9439568
ENSG00000184205.10	TSPYL2	turquoise	0.895278037
ENSG00000187189.9	TSPYL4	turquoise	0.961815316
ENSG00000180543.3	TSPYL5	turquoise	0.971123578
ENSG00000167721.6	TSR1	turquoise	0.913016133
ENSG00000158526.7	TSR2	turquoise	0.849206649
ENSG00000007520.3	TSR3	red	0.950170945
ENSG00000032389.8	TSSC1	turquoise	0.936663638
ENSG00000184281.10	TSSC4	turquoise	0.812888197
ENSG00000162526.6	TSSK3	yellow	0.892234374
ENSG00000178093.12	TSSK6	blue	0.761297373
ENSG00000128311.9	TST	green	0.651206949
ENSG00000104522.11	TSTA3	turquoise	0.904296432
ENSG00000215845.6	TSTD1	turquoise	0.848191043
ENSG00000136925.10	TSTD2	blue	0.915377215
ENSG00000228439.3	TSTD3	blue	0.783211652
ENSG00000146216.7	TTBK1	turquoise	0.934308406
ENSG00000128881.12	TTBK2	turquoise	0.931412196
ENSG00000113312.6	TTC1	turquoise	0.94296698
ENSG00000149292.12	TTC12	turquoise	0.785200262
ENSG00000143643.8	TTC13	turquoise	0.946789691
ENSG00000163728.6	TTC14	yellow	0.930369629
ENSG00000052841.10	TTC17	turquoise	0.923547074
ENSG00000011295.11	TTC19	turquoise	0.948447911
ENSG00000168026.12	TTC21A	pink	0.899246049

ENSG00000123607.10	TTC21B	turquoise	0.95926281
ENSG00000006555.6	TTC22	turquoise	0.759248624
ENSG00000103852.8	TTC23	blue	0.828813522
ENSG00000204815.4	TTC25	pink	0.917728563
ENSG00000105948.9	TTC26	pink	0.95673359
ENSG00000018699.7	TTC27	turquoise	0.822188143
ENSG00000100154.10	TTC28	turquoise	0.86943328
ENSG00000235954.2	TTC28-AS1	turquoise	0.883621056
ENSG00000182670.9	TTC3	turquoise	0.963848442
ENSG00000197557.6	TTC30A	blue	0.758272864
ENSG00000196659.9	TTC30B	pink	0.866606014
ENSG00000115282.15	TTC31	yellow	0.931158667
ENSG00000183891.5	TTC32	turquoise	0.78184686
ENSG00000113638.8	TTC33	turquoise	0.928522792
ENSG00000172425.6	TTC36	turquoise	0.66695723
ENSG00000198677.6	TTC37	turquoise	0.925449567
ENSG00000075234.12	TTC38	blue	0.780034121
ENSG00000085831.11	TTC39A	turquoise	0.876738347
ENSG00000155158.16	TTC39B	turquoise	0.867044496
ENSG00000168234.8	TTC39C	turquoise	0.862783167
ENSG00000215105.3	TTC3P1	turquoise	0.864051562
ENSG00000243725.2	TTC4	pink	0.875270863
ENSG00000171811.8	TTC40	pink	0.905425932
ENSG00000136319.7	TTC5	turquoise	0.873667923
ENSG00000068724.11	TTC7A	yellow	0.903708067
ENSG00000165914.10	TTC7B	turquoise	0.96752132
ENSG00000165533.14	TTC8	turquoise	0.939605645
ENSG00000133985.2	TTC9	turquoise	0.918058694
ENSG00000174521.7	TTC9B	turquoise	0.654275114
ENSG00000162222.9	TTC9C	turquoise	0.966567614
ENSG00000125482.8	TTF1	turquoise	0.92917958
ENSG00000116830.7	TTF2	blue	0.885149624
ENSG00000101407.8	TTI1	turquoise	0.965436215
ENSG00000129696.8	TTI2	turquoise	0.917995339
ENSG00000114999.7	TTL	turquoise	0.866851395
ENSG00000100271.12	TTLL1	turquoise	0.934522928
ENSG00000175764.10	TTLL11	brown	0.844099737
ENSG00000100304.12	TTLL12	turquoise	0.92170564
ENSG00000214021.11	TTLL3	yellow	0.936865282
ENSG00000135912.6	TTLL4	blue	0.859023546
ENSG00000119685.15	TTLL5	turquoise	0.900123737
ENSG00000137941.12	TTLL7	brown	0.89717964
ENSG00000131044.12	TTLL9	pink	0.896310542
ENSG00000137561.4	TTPA	green	0.777576483
ENSG00000124120.6	TTPAL	turquoise	0.910251631
ENSG00000118271.5	TTR	grey	0.053188376
ENSG00000176728.3	TTTY14	grey	0.281091598
ENSG00000233864.3	TTTY15	grey	0.242020082

ENSG00000167614.9	TTYH1	green	0.756650675
ENSG00000141540.6	TTYH2	brown	0.952729198
ENSG00000136295.10	TTYH3	turquoise	0.845263725
ENSG00000166402.4	TUB	blue	0.829006547
ENSG00000167552.9	TUBA1A	turquoise	0.907266599
ENSG00000123416.11	TUBA1B	turquoise	0.864818743
ENSG00000167553.10	TUBA1C	black	0.848998895
ENSG00000161149.7	TUBA3FP	pink	0.860773336
ENSG00000127824.9	TUBA4A	turquoise	0.918678479
ENSG00000183785.10	TUBA8	turquoise	0.874915869
ENSG00000196230.8	TUBB	turquoise	0.910739375
ENSG00000137267.5	TUBB2A	turquoise	0.862263762
ENSG00000137285.9	TUBB2B	green	0.692125406
ENSG00000216819.1	TUBB2BP1	turquoise	0.477582026
ENSG00000258947.2	TUBB3	turquoise	0.882411337
ENSG00000104833.6	TUBB4A	turquoise	0.897403746
ENSG00000188229.5	TUBB4B	pink	0.826940746
ENSG00000176014.8	TUBB6	black	0.83339549
ENSG00000108423.10	TUBD1	turquoise	0.865781087
ENSG00000074935.9	TUBE1	yellow	0.923420631
ENSG00000131462.3	TUBG1	turquoise	0.918181166
ENSG00000037042.8	TUBG2	turquoise	0.92549283
ENSG00000130640.9	TUBGCP2	turquoise	0.956418042
ENSG00000126216.8	TUBGCP3	turquoise	0.948525758
ENSG00000137822.8	TUBGCP4	turquoise	0.965669774
ENSG00000153575.6	TUBGCP5	turquoise	0.927211281
ENSG00000128159.7	TUBGCP6	yellow	0.957831051
ENSG00000178952.4	TUFM	blue	0.862232169
ENSG00000143367.11	TUFT1	turquoise	0.887401551
ENSG00000253352.4	TUG1	turquoise	0.902369803
ENSG00000078246.11	TULP3	blue	0.878221117
ENSG00000130338.8	TULP4	brown	0.764735968
ENSG00000198680.3	TUSC1	turquoise	0.948045334
ENSG00000114383.5	TUSC2	turquoise	0.955086375
ENSG00000104723.16	TUSC3	turquoise	0.939437968
ENSG00000149016.11	TUT1	turquoise	0.85931788
ENSG00000166676.10	TVP23A	turquoise	0.930539731
ENSG00000171928.9	TVP23B	blue	0.915213183
ENSG00000175106.12	TVP23C	blue	0.792638534
ENSG00000151239.9	TWF1	blue	0.881353811
ENSG00000247596.4	TWF2	turquoise	0.888795723
ENSG00000105849.5	TWISTNB	turquoise	0.916364905
ENSG00000128791.7	TWSG1	blue	0.901294437
ENSG00000084652.11	TXLNA	turquoise	0.876778866
ENSG00000086712.8	TXLNG	turquoise	0.958268415
ENSG00000131002.7	TXLNG2P	grey	0.308974717
ENSG00000136810.8	TXN	turquoise	0.886962164
ENSG00000100348.5	TXN2	turquoise	0.893410282

ENSG00000153066.8	TXNDC11	turquoise	0.941772506
ENSG00000117862.7	TXNDC12	turquoise	0.919431649
ENSG00000113621.10	TXNDC15	turquoise	0.98277421
ENSG00000087301.4	TXNDC16	turquoise	0.944714176
ENSG00000129235.6	TXNDC17	turquoise	0.847156255
ENSG00000239264.4	TXNDC5	turquoise	0.867550276
ENSG00000115514.7	TXNDC9	turquoise	0.906958308
ENSG00000117289.7	TXNIP	black	0.63067783
ENSG00000091164.8	TXNL1	turquoise	0.958006483
ENSG00000141759.10	TXNL4A	turquoise	0.90008433
ENSG00000140830.4	TXNL4B	turquoise	0.861608254
ENSG00000198431.11	TXNRD1	turquoise	0.815483111
ENSG00000184470.15	TXNRD2	turquoise	0.851162156
ENSG00000197763.8	TXNRD3	blue	0.762852349
ENSG00000105397.9	TYK2	turquoise	0.878147238
ENSG00000025708.8	TYMP	magenta	0.789052683
ENSG00000176890.11	TYMS	brown	0.859821683
ENSG00000092445.7	TYRO3	turquoise	0.886301235
ENSG00000011600.7	TYROBP	magenta	0.942777219
ENSG00000156521.9	TYSND1	turquoise	0.915097414
ENSG00000198874.8	TYW1	turquoise	0.840742838
ENSG00000254184.3	TYW1B	turquoise	0.552259896
ENSG00000162623.11	TYW3	turquoise	0.906873264
ENSG00000162971.6	TYW5	turquoise	0.880894658
ENSG00000160201.7	U2AF1	turquoise	0.875428862
ENSG00000161265.10	U2AF1L4	turquoise	0.916749932
ENSG00000063244.8	U2AF2	turquoise	0.952178073
ENSG00000163714.13	U2SURP	turquoise	0.931021357
ENSG00000271817.1	U3	blue	0.546358323
ENSG00000200693.1	U3	red	0.521115742
ENSG00000272201.1	U47924.30	turquoise	0.831266147
ENSG00000272173.1	U47924.31	turquoise	0.85025471
ENSG00000269553.1	U62631.5	brown	0.842360875
ENSG00000259886.1	U82695.10	turquoise	0.906847807
ENSG00000224963.2	U82695.9	grey	0.367575005
ENSG00000272462.2	U91328.19	turquoise	0.847603862
ENSG00000137831.10	UACA	brown	0.764300095
ENSG00000117143.9	UAP1	turquoise	0.873811973
ENSG00000197355.6	UAP1L1	yellow	0.869774068
ENSG00000130985.12	UBA1	turquoise	0.921415203
ENSG00000126261.8	UBA2	turquoise	0.954015983
ENSG00000144744.12	UBA3	turquoise	0.930078943
ENSG00000081307.8	UBA5	turquoise	0.95486416
ENSG00000221983.3	UBA52	red	0.966136624
ENSG00000033178.8	UBA6	turquoise	0.893353343
ENSG00000248049.2	UBA6-AS1	brown	0.746257401
ENSG00000182179.6	UBA7	yellow	0.783324876
ENSG00000130560.4	UBAC1	turquoise	0.829236624

ENSG00000134882.11	UBAC2	turquoise	0.881516374
ENSG00000228889.2	UBAC2-AS1	green	0.586796114
ENSG00000153443.8	UBALD1	turquoise	0.893231393
ENSG00000185262.7	UBALD2	blue	0.705631877
ENSG00000165006.9	UBAP1	turquoise	0.934141318
ENSG00000246922.4	UBAP1L	yellow	0.882639654
ENSG00000137073.16	UBAP2	turquoise	0.952494676
ENSG00000143569.14	UBAP2L	turquoise	0.973623145
ENSG00000154127.5	UBASH3B	turquoise	0.823608837
ENSG00000170315.9	UBB	turquoise	0.851778264
ENSG00000263563.1	UBBP4	turquoise	0.722574563
ENSG00000150991.10	UBC	cyan	0.909540561
ENSG00000077721.11	UBE2A	turquoise	0.949418277
ENSG00000119048.3	UBE2B	turquoise	0.917929776
ENSG00000072401.10	UBE2D1	turquoise	0.91275581
ENSG00000131508.11	UBE2D2	turquoise	0.967542712
ENSG00000109332.15	UBE2D3	turquoise	0.90271885
ENSG00000078967.8	UBE2D4	turquoise	0.922959964
ENSG00000170142.7	UBE2E1	blue	0.903921773
ENSG00000182247.5	UBE2E2	turquoise	0.960265921
ENSG00000170035.11	UBE2E3	turquoise	0.944245227
ENSG00000184182.14	UBE2F	turquoise	0.879895306
ENSG00000233337.1	UBE2FP3	turquoise	0.832914837
ENSG00000132388.8	UBE2G1	turquoise	0.94417197
ENSG00000184787.14	UBE2G2	turquoise	0.941964125
ENSG00000186591.7	UBE2H	turquoise	0.899117613
ENSG00000103275.14	UBE2I	turquoise	0.966342996
ENSG00000198833.6	UBE2J1	turquoise	0.934906022
ENSG00000160087.16	UBE2J2	turquoise	0.898245361
ENSG00000078140.9	UBE2K	turquoise	0.978526395
ENSG00000185651.10	UBE2L3	turquoise	0.935934541
ENSG00000156587.11	UBE2L6	blue	0.828140409
ENSG00000130725.3	UBE2M	turquoise	0.891421294
ENSG00000177889.5	UBE2N	turquoise	0.969652694
ENSG00000175931.8	UBE2O	turquoise	0.971522788
ENSG00000160714.5	UBE2Q1	turquoise	0.979237805
ENSG00000140367.7	UBE2Q2	blue	0.824007356
ENSG00000189136.4	UBE2Q2P1	yellow	0.798471097
ENSG00000215218.3	UBE2QL1	turquoise	0.886389511
ENSG00000107341.4	UBE2R2	turquoise	0.978250826
ENSG00000108106.9	UBE2S	turquoise	0.87836122
ENSG00000077152.5	UBE2T	turquoise	0.935520023
ENSG00000244687.7	UBE2V1	turquoise	0.975185339
ENSG00000169139.7	UBE2V2	turquoise	0.952943739
ENSG00000104343.15	UBE2W	turquoise	0.94766176
ENSG00000159202.13	UBE2Z	turquoise	0.947291374
ENSG00000114062.13	UBE3A	turquoise	0.963129497
ENSG00000151148.9	UBE3B	turquoise	0.957373702

ENSG00000009335.13	UBE3C	turquoise	0.973002676
ENSG00000118420.12	UBE3D	turquoise	0.835357489
ENSG00000130939.14	UBE4B	turquoise	0.955015307
ENSG00000103353.11	UBFD1	turquoise	0.974546351
ENSG00000120942.9	UBIAD1	turquoise	0.907912376
ENSG00000122042.9	UBL3	turquoise	0.942867501
ENSG00000102178.8	UBL4A	turquoise	0.945131058
ENSG00000198258.6	UBL5	red	0.954535334
ENSG00000138629.11	UBL7	turquoise	0.915090285
ENSG00000247240.3	UBL7-AS1	blue	0.683380268
ENSG00000164332.6	UBLCP1	turquoise	0.950476511
ENSG00000118900.10	UBN1	turquoise	0.939884177
ENSG00000157741.10	UBN2	yellow	0.931659417
ENSG00000185019.12	UBOX5	turquoise	0.912718518
ENSG00000153560.7	UBP1	turquoise	0.973569517
ENSG00000135018.9	UBQLN1	turquoise	0.965417717
ENSG00000188021.7	UBQLN2	turquoise	0.979434522
ENSG00000160803.7	UBQLN4	turquoise	0.926198568
ENSG00000159459.7	UBR1	turquoise	0.942744239
ENSG00000024048.6	UBR2	turquoise	0.922707446
ENSG00000144357.12	UBR3	turquoise	0.957864138
ENSG00000127481.10	UBR4	turquoise	0.924842409
ENSG00000104517.8	UBR5	turquoise	0.936320728
ENSG00000012963.9	UBR7	turquoise	0.973781837
ENSG00000165886.4	UBTD1	turquoise	0.721553111
ENSG00000168246.5	UBTD2	turquoise	0.932127111
ENSG00000108312.10	UBTF	turquoise	0.939343627
ENSG00000162191.9	UBXN1	red	0.968928921
ENSG00000158062.16	UBXN11	turquoise	0.846983305
ENSG00000173960.8	UBXN2A	blue	0.910645684
ENSG00000215114.3	UBXN2B	turquoise	0.964233958
ENSG00000144224.12	UBXN4	turquoise	0.895247789
ENSG00000167671.7	UBXN6	turquoise	0.925669406
ENSG00000163960.7	UBXN7	turquoise	0.923151314
ENSG00000104691.10	UBXN8	turquoise	0.946204237
ENSG00000154277.8	UCHL1	turquoise	0.933932137
ENSG00000118939.13	UCHL3	turquoise	0.912554829
ENSG00000116750.9	UCHL5	turquoise	0.955805734
ENSG00000130717.8	UCK1	blue	0.850447346
ENSG00000143179.8	UCK2	turquoise	0.909713281
ENSG00000198276.9	UCKL1	turquoise	0.878884232
ENSG00000163794.6	UCN	grey	0.76635189
ENSG00000175567.4	UCP2	magenta	0.815125714
ENSG00000175564.8	UCP3	grey	0.777700597
ENSG00000151116.12	UEVLD	blue	0.946653717
ENSG00000143222.7	UFC1	turquoise	0.928882596
ENSG00000070010.14	UFD1L	turquoise	0.906106269
ENSG00000014123.9	UFL1	blue	0.924715775

ENSG00000120686.7	UFM1	turquoise	0.906614069
ENSG00000176125.3	UFSP1	turquoise	0.750404487
ENSG00000109775.6	UFSP2	turquoise	0.894637515
ENSG00000148154.5	UGCG	turquoise	0.941000912
ENSG00000109814.7	UGDH	cyan	0.876172979
ENSG00000249348.1	UGDH-AS1	blue	0.82089387
ENSG00000136731.8	UGGT1	turquoise	0.939575981
ENSG00000102595.14	UGGT2	turquoise	0.786545805
ENSG00000169764.10	UGP2	turquoise	0.91088595
ENSG00000174607.6	UGT8	brown	0.879510706
ENSG00000152332.11	UHMK1	turquoise	0.94139683
ENSG00000065060.12	UHRF1BP1	turquoise	0.923553384
ENSG00000111647.8	UHRF1BP1L	turquoise	0.957555989
ENSG00000147854.12	UHRF2	turquoise	0.906459794
ENSG00000087206.12	UIMC1	blue	0.900104145
ENSG00000177169.5	ULK1	turquoise	0.886790283
ENSG00000083290.15	ULK2	turquoise	0.927886287
ENSG00000140474.8	ULK3	turquoise	0.836564903
ENSG00000114491.9	UMPS	turquoise	0.873742904
ENSG00000109103.7	UNC119	turquoise	0.869852261
ENSG00000175970.7	UNC119B	blue	0.863047897
ENSG00000130477.10	UNC13A	turquoise	0.910312233
ENSG00000198722.8	UNC13B	turquoise	0.938060393
ENSG00000137766.12	UNC13C	turquoise	0.682182344
ENSG00000092929.7	UNC13D	yellow	0.823230542
ENSG00000140553.12	UNC45A	turquoise	0.904638862
ENSG00000115446.7	UNC50	turquoise	0.934157293
ENSG00000113763.6	UNC5A	turquoise	0.865636684
ENSG00000107731.8	UNC5B	brown	0.914587126
ENSG00000237512.2	UNC5B-AS1	red	0.488209261
ENSG00000182168.10	UNC5C	turquoise	0.870172073
ENSG00000124602.5	UNC5CL	yellow	0.709297025
ENSG00000156687.6	UNC5D	turquoise	0.700336784
ENSG00000133958.9	UNC79	turquoise	0.942643906
ENSG00000144406.14	UNC80	turquoise	0.924957099
ENSG00000110057.3	UNC93B1	red	0.722093834
ENSG00000076248.6	UNG	green	0.862816751
ENSG00000132478.5	UNK	turquoise	0.900354279
ENSG00000059145.14	UNKL	turquoise	0.9015109
ENSG00000005007.8	UPF1	turquoise	0.934349687
ENSG00000151461.15	UPF2	turquoise	0.916027065
ENSG00000169062.10	UPF3A	turquoise	0.931817266
ENSG00000214832.4	UPF3AP2	turquoise	0.570740942
ENSG00000125351.6	UPF3B	turquoise	0.944508817
ENSG00000114638.3	UPK1B	turquoise	0.694246259
ENSG00000110375.2	UPK2	turquoise	0.731719292
ENSG00000243566.2	UPK3B	grey	0.606331513
ENSG00000267368.1	UPK3BL	turquoise	0.480517691

ENSG00000183696.9	UPP1	blue	0.710974452
ENSG00000094841.9	UPRT	turquoise	0.957718516
ENSG00000101019.17	UQCC1	turquoise	0.980790208
ENSG00000137288.5	UQCC2	red	0.930034426
ENSG00000184076.10	UQCR10	red	0.951316561
ENSG00000127540.7	UQCR11	red	0.952788016
ENSG00000156467.5	UQCRB	turquoise	0.899019414
ENSG00000010256.6	UQCRC1	turquoise	0.933012588
ENSG00000140740.6	UQCRC2	turquoise	0.964984528
ENSG00000169021.4	UQCRFS1	turquoise	0.911914994
ENSG00000226085.2	UQCRFS1P1	turquoise	0.449912528
ENSG00000173660.7	UQCRH	turquoise	0.922358178
ENSG00000164405.6	UQCRQ	red	0.956423661
ENSG00000222019.3	URAHP	yellow	0.839601766
ENSG00000142207.5	URB1	turquoise	0.948074232
ENSG00000135763.5	URB2	blue	0.892189525
ENSG00000106608.12	URGCP	turquoise	0.965300796
ENSG00000105176.13	URI1	turquoise	0.790418091
ENSG00000167118.6	URM1	turquoise	0.840312134
ENSG00000126088.8	UROD	turquoise	0.935889424
ENSG00000188690.8	UROS	turquoise	0.818812809
ENSG00000103005.7	USB1	turquoise	0.931644578
ENSG00000053501.8	USE1	red	0.945711942
ENSG00000158773.10	USF1	turquoise	0.959830658
ENSG00000105698.11	USF2	red	0.922076226
ENSG00000006611.11	USH1C	brown	0.526035286
ENSG00000130307.7	USHBP1	brown	0.568160561
ENSG00000173915.8	USMG5	turquoise	0.884295272
ENSG00000138768.10	USO1	turquoise	0.942958649
ENSG00000162607.8	USP1	blue	0.907113306
ENSG00000103194.11	USP10	turquoise	0.97574173
ENSG00000102226.5	USP11	turquoise	0.943764053
ENSG00000152484.9	USP12	turquoise	0.863853677
ENSG00000230641.1	USP12-AS2	turquoise	0.625590754
ENSG00000058056.4	USP13	turquoise	0.910025539
ENSG00000101557.10	USP14	turquoise	0.974794945
ENSG00000135655.9	USP15	turquoise	0.925277091
ENSG00000156256.10	USP16	turquoise	0.898679944
ENSG00000184979.9	USP18	turquoise	0.592750004
ENSG00000172046.14	USP19	turquoise	0.942109142
ENSG00000036672.11	USP2	turquoise	0.888604137
ENSG00000136878.8	USP20	turquoise	0.954739645
ENSG00000143258.11	USP21	turquoise	0.894356232
ENSG00000124422.7	USP22	turquoise	0.961024926
ENSG00000162402.8	USP24	turquoise	0.907222187
ENSG00000155313.11	USP25	turquoise	0.952343352
ENSG00000242013.4	USP27X	turquoise	0.901183547
ENSG00000234390.4	USP27X-AS1	blue	0.804371317

ENSG00000048028.7	USP28	turquoise	0.902178798
ENSG00000140455.12	USP3	blue	0.8441519
ENSG00000259248.1	USP3-AS1	turquoise	0.849906297
ENSG00000135093.8	USP30	turquoise	0.939787132
ENSG00000103404.10	USP31	turquoise	0.886836793
ENSG00000170832.8	USP32	brown	0.808637862
ENSG00000233327.6	USP32P2	turquoise	0.506652022
ENSG00000189423.7	USP32P3	blue	0.766831969
ENSG00000077254.10	USP33	turquoise	0.961750451
ENSG00000115464.10	USP34	blue	0.859385528
ENSG00000118369.8	USP35	turquoise	0.919473342
ENSG00000055483.15	USP36	turquoise	0.912329118
ENSG00000170185.5	USP38	turquoise	0.799556962
ENSG00000168883.15	USP39	turquoise	0.932708037
ENSG00000114316.8	USP4	turquoise	0.952800669
ENSG00000085982.9	USP40	blue	0.880160411
ENSG00000106346.7	USP42	turquoise	0.941714559
ENSG00000123552.13	USP45	turquoise	0.925418485
ENSG00000109189.8	USP46	turquoise	0.765705572
ENSG00000248866.1	USP46-AS1	turquoise	0.723494903
ENSG00000170242.13	USP47	turquoise	0.963673549
ENSG00000090686.11	USP48	turquoise	0.966795885
ENSG00000164663.9	USP49	yellow	0.891916556
ENSG00000111667.9	USP5	turquoise	0.966615614
ENSG00000247746.4	USP51	turquoise	0.88576274
ENSG00000145390.7	USP53	blue	0.850130112
ENSG00000166348.13	USP54	brown	0.926249746
ENSG00000148429.10	USP6NL	blue	0.88809319
ENSG00000187555.10	USP7	turquoise	0.981946324
ENSG00000138592.9	USP8	blue	0.945547446
ENSG00000124486.8	USP9X	turquoise	0.934281928
ENSG00000114374.8	USP9Y	grey	0.282354563
ENSG00000132952.7	USPL1	turquoise	0.852311505
ENSG00000111962.7	UST	turquoise	0.849777254
ENSG00000183520.7	UTP11L	turquoise	0.950656174
ENSG00000156697.8	UTP14A	turquoise	0.934269326
ENSG00000253797.2	UTP14C	turquoise	0.891110829
ENSG00000164338.5	UTP15	turquoise	0.851155266
ENSG00000011260.9	UTP18	turquoise	0.948912322
ENSG00000120800.4	UTP20	turquoise	0.890937091
ENSG00000147679.7	UTP23	turquoise	0.903453709
ENSG00000132467.2	UTP3	turquoise	0.921923046
ENSG00000108651.5	UTP6	turquoise	0.912155327
ENSG00000152818.14	UTRN	turquoise	0.870833266
ENSG00000183878.11	UTY	grey	0.321827731
ENSG00000198382.4	UVRAG	turquoise	0.882957124
ENSG00000163945.11	UVSSA	yellow	0.884084278
ENSG00000115652.10	UXS1	turquoise	0.959951008

ENSG00000126756.7	UXT	red	0.932883185
ENSG00000103043.10	VAC14	turquoise	0.961845107
ENSG00000139190.12	VAMP1	brown	0.801434841
ENSG00000220205.4	VAMP2	turquoise	0.94205321
ENSG00000049245.8	VAMP3	brown	0.904707597
ENSG00000117533.10	VAMP4	turquoise	0.965962731
ENSG00000168899.4	VAMP5	black	0.697328045
ENSG00000124333.10	VAMP7	turquoise	0.804785945
ENSG00000118640.6	VAMP8	magenta	0.904458155
ENSG00000162738.5	VANGL2	green	0.830797956
ENSG00000101558.9	VAPA	turquoise	0.961470943
ENSG00000124164.11	VAPB	turquoise	0.920037191
ENSG00000204394.8	VARS	turquoise	0.949716753
ENSG00000137411.12	VARS2	yellow	0.927320257
ENSG00000071246.6	VASH1	brown	0.798566858
ENSG00000168140.4	VASN	black	0.70602532
ENSG00000125753.9	VASP	black	0.901785219
ENSG00000108828.11	VAT1	turquoise	0.897955108
ENSG00000171724.2	VAT1L	turquoise	0.923840304
ENSG00000141968.3	VAV1	turquoise	0.772543434
ENSG00000160293.12	VAV2	blue	0.826318489
ENSG00000134215.11	VAV3	turquoise	0.744245813
ENSG00000148704.8	VAX1	cyan	0.62935971
ENSG00000116035.2	VAX2	grey	0.273976209
ENSG00000155959.6	VBP1	turquoise	0.900502252
ENSG00000162692.6	VCAM1	grey	0.256042392
ENSG00000038427.11	VCAN	green	0.566357431
ENSG00000035403.12	VCL	blue	0.845025879
ENSG00000165280.11	VCP	turquoise	0.958909346
ENSG00000175073.7	VCPIP1	turquoise	0.939848898
ENSG00000100483.9	VCPKMT	turquoise	0.925720788
ENSG00000213585.6	VDAC1	turquoise	0.962666312
ENSG00000165637.9	VDAC2	turquoise	0.952742122
ENSG00000078668.9	VDAC3	turquoise	0.969025874
ENSG00000112715.16	VEGFA	blue	0.555507675
ENSG00000173511.5	VEGFB	green	0.556778481
ENSG00000136451.4	VEZF1	brown	0.900324188
ENSG00000028203.13	VEZT	turquoise	0.949176389
ENSG00000128564.5	VGF	turquoise	0.726843343
ENSG00000134086.7	VHL	turquoise	0.949693704
ENSG00000136059.10	VILL	grey	0.598237169
ENSG00000026025.9	VIM	black	0.639845548
ENSG00000131871.10	VIMP	turquoise	0.722200548
ENSG00000146469.8	VIP	grey	0.245906703
ENSG00000151445.11	VIPAS39	turquoise	0.958091978
ENSG00000114812.8	VIPR1	turquoise	0.775506172
ENSG00000167397.10	VKORC1	turquoise	0.837666973
ENSG00000196715.5	VKORC1L1	turquoise	0.954363995

ENSG00000147852.11	VLDLR	turquoise	0.882906792
ENSG00000160131.9	VMA21	turquoise	0.934923544
ENSG00000187650.3	VMAC	blue	0.853574834
ENSG00000182853.7	VMO1	red	0.676587306
ENSG00000062716.6	VMP1	turquoise	0.89422467
ENSG00000178201.3	VN1R1	turquoise	0.893127465
ENSG00000230772.1	VN1R108P	turquoise	0.7222769
ENSG00000233121.1	VN1R20P	turquoise	0.846301297
ENSG00000228038.1	VN1R51P	pink	0.837073984
ENSG00000268357.1	VN1R81P	turquoise	0.78564242
ENSG00000268995.1	VN1R82P	turquoise	0.597964605
ENSG00000268521.1	VN1R83P	turquoise	0.668174044
ENSG00000154978.8	VOPP1	blue	0.838697058
ENSG00000145041.11	VPRBP	turquoise	0.932631689
ENSG00000128218.7	VPREB3	turquoise	0.464532812
ENSG00000160695.10	VPS11	turquoise	0.956055555
ENSG00000197969.7	VPS13A	turquoise	0.923419096
ENSG00000132549.14	VPS13B	turquoise	0.930590527
ENSG00000129003.11	VPS13C	turquoise	0.831219043
ENSG00000048707.9	VPS13D	turquoise	0.953807966
ENSG00000215305.5	VPS16	turquoise	0.950039955
ENSG00000104142.6	VPS18	turquoise	0.936190011
ENSG00000131475.2	VPS25	turquoise	0.943160278
ENSG00000122958.10	VPS26A	turquoise	0.942553214
ENSG00000151502.6	VPS26B	turquoise	0.979504721
ENSG00000160948.9	VPS28	turquoise	0.900181422
ENSG00000111237.14	VPS29	turquoise	0.955336311
ENSG00000139719.5	VPS33A	turquoise	0.976416271
ENSG00000184056.10	VPS33B	turquoise	0.966899412
ENSG00000069329.11	VPS35	turquoise	0.970229414
ENSG00000136100.8	VPS36	turquoise	0.955774186
ENSG00000155975.5	VPS37A	turquoise	0.957068458
ENSG00000139722.2	VPS37B	turquoise	0.82343897
ENSG00000167987.6	VPS37C	turquoise	0.916654679
ENSG00000176428.5	VPS37D	turquoise	0.851065172
ENSG00000166887.11	VPS39	turquoise	0.971911834
ENSG00000006715.11	VPS41	turquoise	0.949698946
ENSG00000136631.8	VPS45	turquoise	0.963995556
ENSG00000132612.14	VPS4A	turquoise	0.948972206
ENSG00000119541.5	VPS4B	turquoise	0.870576084
ENSG00000149823.3	VPS51	turquoise	0.939679809
ENSG00000223501.4	VPS52	turquoise	0.953371766
ENSG00000141252.15	VPS53	turquoise	0.9659728
ENSG00000143952.15	VPS54	turquoise	0.804218019
ENSG00000163159.7	VPS72	turquoise	0.923284594
ENSG00000156931.11	VPS8	turquoise	0.940501805
ENSG00000075399.8	VPS9D1	turquoise	0.908985568
ENSG00000261373.1	VPS9D1-AS1	turquoise	0.813363375

ENSG00000100749.3	VRK1	turquoise	0.934877185
ENSG00000028116.12	VRK2	brown	0.842193154
ENSG00000105053.6	VRK3	turquoise	0.956265962
ENSG00000101842.9	VSIG1	turquoise	0.848331482
ENSG00000176834.9	VSIG10	blue	0.837269129
ENSG00000186806.5	VSIG10L	turquoise	0.905797043
ENSG00000019102.7	VSIG2	midnightblue	0.759388725
ENSG00000155659.10	VSIG4	magenta	0.847409766
ENSG00000163032.7	VSNL1	turquoise	0.732579488
ENSG00000170419.6	VSTM2A	turquoise	0.904682504
ENSG00000187135.7	VSTM2B	turquoise	0.86880257
ENSG00000132821.7	VSTM2L	turquoise	0.818221663
ENSG00000165633.8	VSTM4	blue	0.867612028
ENSG00000214376.5	VSTM5	greenyellow	0.848893932
ENSG00000100987.10	VSX1	green	0.582066258
ENSG00000009844.11	VTA1	turquoise	0.950047169
ENSG00000151532.9	VTI1A	turquoise	0.902445182
ENSG00000100568.6	VTI1B	turquoise	0.918437969
ENSG00000109072.9	VTN	yellow	0.878488849
ENSG00000179403.10	VWA1	brown	0.804234433
ENSG00000110002.11	VWA5A	turquoise	0.92016813
ENSG00000145198.10	VWA5B2	turquoise	0.785638452
ENSG00000204396.6	VWA7	turquoise	0.889796495
ENSG00000102763.11	VWA8	turquoise	0.939081246
ENSG00000138614.10	VWA9	blue	0.914014685
ENSG00000188730.4	VWC2	turquoise	0.744466063
ENSG00000167992.8	VWCE	pink	0.864463243
ENSG00000110799.9	VWF	black	0.755957081
ENSG00000095787.17	WAC	turquoise	0.900708226
ENSG00000254635.1	WAC-AS1	turquoise	0.927415711
ENSG00000062650.13	WAPAL	turquoise	0.890520318
ENSG00000140105.13	WARS	cyan	0.883177759
ENSG00000116874.7	WARS2	turquoise	0.867233457
ENSG00000015285.6	WAS	magenta	0.950438699
ENSG00000112290.8	WASF1	turquoise	0.928226693
ENSG00000158195.6	WASF2	blue	0.838329411
ENSG00000132970.8	WASF3	blue	0.805381368
ENSG00000146556.10	WASH2P	turquoise	0.8065239
ENSG00000185596.12	WASH3P	blue	0.654466532
ENSG00000234769.4	WASH4P	yellow	0.872651452
ENSG00000227232.4	WASH7P	yellow	0.754171193
ENSG00000231439.3	WASIR2	cyan	0.721297348
ENSG00000106299.7	WASL	turquoise	0.969417254
ENSG00000239779.2	WBP1	turquoise	0.931055221
ENSG00000084463.3	WBP11	turquoise	0.977339813
ENSG00000166272.12	WBP1L	turquoise	0.905182645
ENSG00000132471.7	WBP2	turquoise	0.932090013
ENSG00000120688.7	WBP4	turquoise	0.957350895

ENSG00000185222.7	WBP5	cyan	0.817566531
ENSG00000174374.9	WBSCR16	blue	0.844633476
ENSG00000185274.7	WBSCR17	turquoise	0.901112502
ENSG00000071462.7	WBSCR22	turquoise	0.955111711
ENSG00000165171.6	WBSCR27	pink	0.600385709
ENSG00000085449.10	WDFY1	turquoise	0.878173442
ENSG00000139668.7	WDFY2	blue	0.909570898
ENSG00000163625.11	WDFY3	turquoise	0.886437182
ENSG00000180769.4	WDFY3-AS2	green	0.848637204
ENSG00000198554.7	WDHD1	turquoise	0.89854054
ENSG00000143951.11	WDPCP	turquoise	0.922616236
ENSG00000071127.12	WDR1	blue	0.866231964
ENSG00000120008.11	WDR11	turquoise	0.928264937
ENSG00000138442.5	WDR12	turquoise	0.964964549
ENSG00000101940.13	WDR13	turquoise	0.911111835
ENSG00000150627.11	WDR17	turquoise	0.915433887
ENSG00000065268.6	WDR18	turquoise	0.867127828
ENSG00000157796.13	WDR19	pink	0.950042783
ENSG00000140153.13	WDR20	turquoise	0.963923404
ENSG00000127580.11	WDR24	turquoise	0.924552023
ENSG00000176473.9	WDR25	turquoise	0.918927479
ENSG00000162923.10	WDR26	turquoise	0.961514622
ENSG00000184465.11	WDR27	yellow	0.93021219
ENSG00000065183.11	WDR3	turquoise	0.939977045
ENSG00000148225.11	WDR31	blue	0.796842263
ENSG00000136709.7	WDR33	turquoise	0.953541259
ENSG00000119333.7	WDR34	pink	0.864777459
ENSG00000118965.10	WDR35	pink	0.9076783
ENSG00000134987.7	WDR36	turquoise	0.888402051
ENSG00000047056.10	WDR37	turquoise	0.941447046
ENSG00000160193.7	WDR4	turquoise	0.89988765
ENSG00000164253.8	WDR41	turquoise	0.954215515
ENSG00000163811.7	WDR43	turquoise	0.873746738
ENSG00000131725.9	WDR44	turquoise	0.947495871
ENSG00000196998.11	WDR45	turquoise	0.938448109
ENSG00000141580.11	WDR45B	turquoise	0.880645793
ENSG00000227057.3	WDR46	turquoise	0.952065975
ENSG00000085433.11	WDR47	turquoise	0.970243272
ENSG00000114742.9	WDR48	turquoise	0.954636258
ENSG00000196363.5	WDR5	turquoise	0.936231188
ENSG00000185798.6	WDR53	turquoise	0.938894192
ENSG00000005448.12	WDR54	turquoise	0.916430759
ENSG00000120314.14	WDR55	turquoise	0.916374822
ENSG00000103091.10	WDR59	turquoise	0.912656495
ENSG00000196981.2	WDR5B	blue	0.844740999
ENSG00000178252.13	WDR6	turquoise	0.862912573
ENSG00000126870.11	WDR60	turquoise	0.890152975
ENSG00000140395.4	WDR61	turquoise	0.961489688

ENSG00000243710.3	WDR65	pink	0.916296446
ENSG00000158023.5	WDR66	pink	0.935183666
ENSG00000091157.9	WDR7	turquoise	0.976167667
ENSG00000082068.4	WDR70	turquoise	0.954286999
ENSG00000177082.8	WDR73	turquoise	0.903369245
ENSG00000133316.11	WDR74	turquoise	0.943361959
ENSG00000115368.5	WDR75	turquoise	0.901902323
ENSG00000116455.9	WDR77	turquoise	0.935222007
ENSG00000167716.14	WDR81	turquoise	0.874456681
ENSG00000164091.7	WDR82	turquoise	0.984241935
ENSG00000123154.7	WDR83	red	0.929248656
ENSG00000105583.5	WDR83OS	turquoise	0.910705833
ENSG00000187260.11	WDR86	grey	0.382625016
ENSG00000140006.7	WDR89	turquoise	0.931269705
ENSG00000161996.13	WDR90	pink	0.911169884
ENSG00000105875.9	WDR91	turquoise	0.920731727
ENSG00000243667.2	WDR92	turquoise	0.920195769
ENSG00000196151.6	WDSUB1	turquoise	0.914731635
ENSG00000142784.11	WDTC1	turquoise	0.955248691
ENSG00000156795.2	WDYHV1	turquoise	0.872277973
ENSG00000103175.6	WFDC1	grey	0.347617246
ENSG00000101443.13	WFDC2	midnightblue	0.780440048
ENSG00000127578.6	WFIKKN1	grey	0.565122689
ENSG00000109501.9	WFS1	turquoise	0.74364324
ENSG00000156232.6	WHAMM	turquoise	0.899796201
ENSG00000248334.2	WHAMMP2	turquoise	0.826182787
ENSG00000187667.6	WHAMMP3	turquoise	0.856476334
ENSG00000109685.13	WHSC1	turquoise	0.956478608
ENSG00000147548.12	WHSC1L1	turquoise	0.957631672
ENSG00000215861.4	WI2-1896O14.1	turquoise	0.787886601
ENSG00000270301.1	WI2-2334D6.1	turquoise	0.678972605
ENSG00000232637.3	WI2-3658N16.1	turquoise	0.715113168
ENSG00000205632.2	WI2-81516E3.1	turquoise	0.729805126
ENSG00000170473.12	WIBG	turquoise	0.913854214
ENSG00000156076.5	WIF1	green	0.609417167
ENSG00000115935.12	WIPF1	brown	0.882879765
ENSG00000171475.9	WIPF2	turquoise	0.940678251
ENSG00000122574.6	WIPF3	turquoise	0.885919648
ENSG00000070540.8	WIPI1	blue	0.824413426
ENSG00000157954.10	WIPI2	turquoise	0.979843033
ENSG00000011451.13	WIZ	turquoise	0.898202006
ENSG00000116729.9	WLS	green	0.914619853
ENSG00000060237.12	WNK1	brown	0.835644455
ENSG00000165238.12	WNK2	turquoise	0.926403694
ENSG00000196632.6	WNK3	turquoise	0.912424142
ENSG00000125084.7	WNT1	turquoise	0.812864014
ENSG00000135925.4	WNT10A	turquoise	0.764163164
ENSG00000169884.9	WNT10B	turquoise	0.780252238

ENSG00000085741.8	WNT11	turquoise	0.727947104
ENSG00000134245.13	WNT2B	turquoise	0.923850381
ENSG00000108379.5	WNT3	turquoise	0.812255523
ENSG00000114251.9	WNT5A	magenta	0.808532488
ENSG00000111186.8	WNT5B	turquoise	0.877041816
ENSG00000115596.3	WNT6	turquoise	0.664375058
ENSG00000154764.5	WNT7A	green	0.50479118
ENSG00000188064.5	WNT7B	grey	0.279079578
ENSG00000061492.7	WNT8A	turquoise	0.765175513
ENSG00000141499.12	WRAP53	pink	0.891683192
ENSG00000116213.11	WRAP73	turquoise	0.914480402
ENSG00000182093.10	WRB	turquoise	0.942255245
ENSG00000165392.5	WRN	turquoise	0.852208302
ENSG00000124535.11	WRNIP1	turquoise	0.973299131
ENSG00000109046.10	WSB1	yellow	0.894869898
ENSG00000176871.4	WSB2	turquoise	0.908811338
ENSG00000179314.9	WSCD1	brown	0.640909628
ENSG00000075035.5	WSCD2	turquoise	0.893658042
ENSG00000184937.8	WT1	turquoise	0.773763346
ENSG00000146457.10	WTAP	turquoise	0.890364404
ENSG00000142279.8	WTIP	grey	0.60204102
ENSG00000113645.9	WWC1	blue	0.810203248
ENSG00000151718.11	WWC2	turquoise	0.693161928
ENSG00000047644.13	WWC3	brown	0.863439556
ENSG00000186153.12	WWOX	blue	0.834303361
ENSG00000123124.9	WWP1	turquoise	0.91413657
ENSG00000198373.8	WWP2	turquoise	0.910388009
ENSG00000018408.10	WWTR1	green	0.560248474
ENSG00000076924.7	XAB2	turquoise	0.916829698
ENSG00000132530.12	XAF1	brown	0.575831068
ENSG00000100219.12	XBP1	cyan	0.938117942
ENSG00000101966.8	XIAP	turquoise	0.963304105
ENSG00000229807.5	XIST	grey	0.132777203
ENSG00000047597.5	XK	turquoise	0.914899148
ENSG00000206579.7	XKR4	turquoise	0.869529768
ENSG00000158156.7	XKR8	turquoise	0.869889299
ENSG00000136936.6	XPA	turquoise	0.906784325
ENSG00000154767.10	XPC	turquoise	0.894912636
ENSG00000108039.13	XPNPEP1	turquoise	0.972184659
ENSG00000196236.8	XPNPEP3	blue	0.904461981
ENSG00000082898.12	XPO1	blue	0.86343689
ENSG00000132953.12	XPO4	turquoise	0.909513186
ENSG00000124571.13	XPO5	turquoise	0.973178446
ENSG00000169180.7	XPO6	turquoise	0.945031415
ENSG00000130227.12	XPO7	turquoise	0.985061017
ENSG00000184575.7	XPOT	turquoise	0.86358126
ENSG00000143324.9	XPR1	turquoise	0.94663729
ENSG00000073050.7	XRCC1	turquoise	0.944351475

ENSG00000126215.9	XRCC3	yellow	0.88416292
ENSG00000152422.11	XRCC4	turquoise	0.81258349
ENSG00000079246.11	XRCC5	turquoise	0.947937503
ENSG00000196419.8	XRCC6	turquoise	0.963559021
ENSG00000166896.3	XRCC6BP1	turquoise	0.876811937
ENSG00000114127.6	XRN1	turquoise	0.898092178
ENSG00000088930.6	XRN2	turquoise	0.915509705
ENSG00000166435.11	XRRA1	turquoise	0.786055154
ENSG00000232694.2	XX-CR54.3	turquoise	0.600257777
ENSG00000237476.1	XXbac-B135H6.15	grey	0.457922954
ENSG00000272829.1	XXbac-B135H6.18	turquoise	0.740653644
ENSG00000273343.1	XXbac-B444P24.13	turquoise	0.808022353
ENSG00000272217.1	XXbac-BPG157A10	grey	0.485901403
ENSG00000272221.1	XXbac-BPG181B23	turquoise	0.616692784
ENSG00000272273.1	XXbac-BPG252P9.1	turquoise	0.60921742
ENSG00000272540.1	XXbac-BPG252P9.9	yellow	0.594489418
ENSG00000272501.1	XXbac-BPG299F13.	turquoise	0.885485801
ENSG00000272541.1	XXbac-BPGBPG55C	grey	0.707976875
ENSG00000272316.1	XXbac-BPGBPG55C	brown	0.794601646
ENSG00000271761.1	XXbac-BPGBPG55C	turquoise	0.400309736
ENSG00000271858.1	XXcos-LUCA11.4	turquoise	0.738116763
ENSG00000181404.13	XXyac-YRM2039.2	turquoise	0.60601912
ENSG00000226445.1	XXyac-YX65C7_A.2	turquoise	0.831297797
ENSG00000173950.11	XXYLT1	turquoise	0.94170524
ENSG00000103489.7	XYLT1	greenyellow	0.794579013
ENSG00000015532.5	XYLT2	turquoise	0.901914558
ENSG00000201134.1	Y_RNA	grey	0.210395469
ENSG00000207425.1	Y_RNA	grey	0.476072737
ENSG00000200610.1	Y_RNA	turquoise	0.582280765
ENSG00000201678.1	Y_RNA	turquoise	0.625235103
ENSG00000199476.1	Y_RNA	turquoise	0.383722287
ENSG00000251867.2	Y_RNA	turquoise	0.921283587
ENSG00000200714.1	Y_RNA	turquoise	0.469935527
ENSG00000241127.3	YAE1D1	turquoise	0.899357824
ENSG00000015153.10	YAF2	turquoise	0.905355197
ENSG00000137693.9	YAP1	green	0.860994117
ENSG00000134684.6	YARS	turquoise	0.887675935
ENSG00000139131.8	YARS2	turquoise	0.95721313
ENSG00000182362.9	YBEY	turquoise	0.697033076
ENSG00000065978.13	YBX1	blue	0.817748325
ENSG00000213866.3	YBX1P10	blue	0.585365459
ENSG00000060138.8	YBX3	black	0.873498762
ENSG00000161179.9	YDJC	turquoise	0.850054133
ENSG00000163872.11	YEATS2	turquoise	0.973153387
ENSG00000233885.3	YEATS2-AS1	yellow	0.818857139
ENSG00000127337.2	YEATS4	turquoise	0.904921222
ENSG00000176105.9	YES1	green	0.723731342
ENSG00000174851.10	YIF1A	blue	0.827366829

ENSG00000167645.12	YIF1B	red	0.925314747
ENSG00000058799.9	YIPF1	cyan	0.934661585
ENSG00000130733.6	YIPF2	red	0.892326395
ENSG00000137207.7	YIPF3	turquoise	0.950934616
ENSG00000119820.6	YIPF4	turquoise	0.953719526
ENSG00000145817.12	YIPF5	turquoise	0.925596956
ENSG00000181704.7	YIPF6	turquoise	0.916362413
ENSG00000250067.7	YJEFN3	turquoise	0.783142032
ENSG00000106636.3	YKT6	turquoise	0.896504616
ENSG00000119596.13	YLPM1	turquoise	0.96089781
ENSG00000136758.14	YME1L1	turquoise	0.956725089
ENSG00000180667.6	YOD1	turquoise	0.816942275
ENSG00000100027.10	YPEL1	turquoise	0.914441931
ENSG00000175155.4	YPEL2	turquoise	0.929925261
ENSG00000090238.7	YPEL3	turquoise	0.843789216
ENSG00000166793.6	YPEL4	turquoise	0.859202922
ENSG00000119801.8	YPEL5	turquoise	0.973348678
ENSG00000196449.3	YRDC	turquoise	0.924982018
ENSG00000083896.8	YTHDC1	turquoise	0.882454516
ENSG00000047188.11	YTHDC2	turquoise	0.933306777
ENSG00000149658.13	YTHDF1	turquoise	0.888540366
ENSG00000198492.10	YTHDF2	turquoise	0.967237715
ENSG00000185728.12	YTHDF3	turquoise	0.948264
ENSG00000270673.1	YTHDF3-AS1	grey	0.496507502
ENSG00000166913.8	YWHAB	turquoise	0.971067627
ENSG00000108953.12	YWHAE	turquoise	0.932404415
ENSG00000170027.5	YWHAG	turquoise	0.963195171
ENSG00000128245.10	YWHAH	turquoise	0.948343948
ENSG00000134308.9	YWHAQ	turquoise	0.894815221
ENSG00000164924.13	YWHAZ	turquoise	0.964082268
ENSG00000100811.6	YY1	turquoise	0.979159997
ENSG00000163374.15	YY1AP1	turquoise	0.951296747
ENSG00000269482.1	Z69720.2	yellow	0.728071624
ENSG00000229891.1	Z83851.1	red	0.752443338
ENSG00000265106.1	Z93241.1	turquoise	0.409546097
ENSG00000180011.6	ZADH2	turquoise	0.954354689
ENSG00000115085.9	ZAP70	brown	0.522190456
ENSG00000169064.8	ZBBX	pink	0.896069954
ENSG00000214717.5	ZBED1	blue	0.8726662
ENSG00000177494.5	ZBED2	turquoise	0.537638487
ENSG00000132846.5	ZBED3	brown	0.918169037
ENSG00000100426.6	ZBED4	turquoise	0.9274643
ENSG00000236287.3	ZBED5	turquoise	0.943115483
ENSG00000247271.2	ZBED5-AS1	turquoise	0.783087178
ENSG00000188707.4	ZBED6CL	pink	0.79279299
ENSG00000126804.9	ZBTB1	turquoise	0.894476525
ENSG00000205189.7	ZBTB10	turquoise	0.885619794
ENSG00000066422.4	ZBTB11	turquoise	0.956087634

ENSG00000256628.2	ZBTB11-AS1	turquoise	0.760833055
ENSG00000204366.3	ZBTB12	turquoise	0.714488538
ENSG00000198081.6	ZBTB14	turquoise	0.821359431
ENSG00000109906.9	ZBTB16	green	0.644252431
ENSG00000116809.7	ZBTB17	blue	0.841013586
ENSG00000179456.9	ZBTB18	turquoise	0.837046097
ENSG00000181472.4	ZBTB2	turquoise	0.898341872
ENSG00000173276.9	ZBTB21	turquoise	0.872169743
ENSG00000236104.2	ZBTB22	turquoise	0.946424777
ENSG00000112365.4	ZBTB24	turquoise	0.963958891
ENSG00000089775.7	ZBTB25	turquoise	0.901922113
ENSG00000171448.8	ZBTB26	turquoise	0.838683916
ENSG00000185670.7	ZBTB3	blue	0.812207939
ENSG00000177485.6	ZBTB33	turquoise	0.893100278
ENSG00000177125.5	ZBTB34	turquoise	0.846089183
ENSG00000185278.10	ZBTB37	turquoise	0.787496124
ENSG00000177311.6	ZBTB38	turquoise	0.893381989
ENSG00000166860.2	ZBTB39	turquoise	0.852773339
ENSG00000174282.7	ZBTB4	turquoise	0.954333157
ENSG00000184677.12	ZBTB40	turquoise	0.884739275
ENSG00000177888.7	ZBTB41	turquoise	0.901209192
ENSG00000169155.5	ZBTB43	turquoise	0.920153329
ENSG00000196323.7	ZBTB44	turquoise	0.90702729
ENSG00000119574.8	ZBTB45	turquoise	0.907176485
ENSG00000130584.6	ZBTB46	turquoise	0.862026965
ENSG00000114853.9	ZBTB47	brown	0.77239996
ENSG00000204859.7	ZBTB48	turquoise	0.864962781
ENSG00000168826.11	ZBTB49	turquoise	0.925792846
ENSG00000168795.4	ZBTB5	turquoise	0.840412525
ENSG00000186130.4	ZBTB6	turquoise	0.880114889
ENSG00000178951.4	ZBTB7A	turquoise	0.919747661
ENSG00000160685.9	ZBTB7B	blue	0.661708394
ENSG00000160062.10	ZBTB8A	turquoise	0.779930118
ENSG00000176261.11	ZBTB8OS	turquoise	0.929093684
ENSG00000213588.4	ZBTB9	turquoise	0.931462854
ENSG00000104427.7	ZC2HC1A	turquoise	0.934501869
ENSG00000119703.12	ZC2HC1C	pink	0.915990586
ENSG00000135482.2	ZC3H10	blue	0.877661127
ENSG00000058673.11	ZC3H11A	turquoise	0.885244712
ENSG00000163874.8	ZC3H12A	black	0.720259288
ENSG00000102053.11	ZC3H12B	turquoise	0.93974677
ENSG00000149289.6	ZC3H12C	turquoise	0.856135186
ENSG00000123200.12	ZC3H13	turquoise	0.859237195
ENSG00000100722.14	ZC3H14	turquoise	0.951639333
ENSG00000065548.13	ZC3H15	turquoise	0.966552735
ENSG00000158545.11	ZC3H18	turquoise	0.960402149
ENSG00000014164.6	ZC3H3	turquoise	0.875977327
ENSG00000130749.5	ZC3H4	turquoise	0.835667565

ENSG00000188177.9	ZC3H6	turquoise	0.909516474
ENSG00000122299.7	ZC3H7A	turquoise	0.929684748
ENSG00000100403.10	ZC3H7B	turquoise	0.92738507
ENSG00000144161.8	ZC3H8	turquoise	0.96495752
ENSG00000105939.8	ZC3HAV1	blue	0.733692614
ENSG00000146858.7	ZC3HAV1L	turquoise	0.789665126
ENSG00000091732.11	ZC3HC1	turquoise	0.941585527
ENSG00000126970.11	ZC4H2	turquoise	0.93736196
ENSG00000155329.7	ZCCHC10	turquoise	0.898721502
ENSG00000134744.9	ZCCHC11	turquoise	0.87097667
ENSG00000174460.3	ZCCHC12	turquoise	0.868868065
ENSG00000140948.7	ZCCHC14	turquoise	0.955613253
ENSG00000121766.10	ZCCHC17	turquoise	0.924705535
ENSG00000166707.6	ZCCHC18	turquoise	0.847022998
ENSG00000141664.5	ZCCHC2	turquoise	0.906136655
ENSG00000165424.6	ZCCHC24	brown	0.711835016
ENSG00000177764.6	ZCCHC3	turquoise	0.761732615
ENSG00000168228.10	ZCCHC4	turquoise	0.838044351
ENSG00000083223.13	ZCCHC6	turquoise	0.880478034
ENSG00000147905.13	ZCCHC7	turquoise	0.936055231
ENSG00000033030.9	ZCCHC8	yellow	0.931640856
ENSG00000131732.7	ZCCHC9	turquoise	0.868536771
ENSG00000139168.3	ZCRB1	turquoise	0.817418142
ENSG00000078487.13	ZCWPW1	brown	0.785711604
ENSG00000204186.3	ZDBF2	turquoise	0.89633024
ENSG00000159714.6	ZDHHC1	pink	0.896352429
ENSG00000188818.8	ZDHHC11	yellow	0.674649588
ENSG00000206077.6	ZDHHC11B	yellow	0.805825514
ENSG00000160446.14	ZDHHC12	red	0.814006847
ENSG00000177054.9	ZDHHC13	turquoise	0.934333626
ENSG00000175048.12	ZDHHC14	turquoise	0.898721546
ENSG00000102383.9	ZDHHC15	turquoise	0.918653172
ENSG00000171307.14	ZDHHC16	turquoise	0.932188663
ENSG00000186908.10	ZDHHC17	turquoise	0.960554206
ENSG00000204160.7	ZDHHC18	turquoise	0.877549917
ENSG00000163958.9	ZDHHC19	turquoise	0.581958977
ENSG00000104219.8	ZDHHC2	blue	0.889840093
ENSG00000180776.11	ZDHHC20	blue	0.722167693
ENSG00000175893.7	ZDHHC21	turquoise	0.924733795
ENSG00000177108.5	ZDHHC22	greenyellow	0.88885467
ENSG00000184307.9	ZDHHC23	turquoise	0.858236179
ENSG00000174165.3	ZDHHC24	turquoise	0.896726176
ENSG00000163812.9	ZDHHC3	turquoise	0.820219741
ENSG00000136247.10	ZDHHC4	turquoise	0.92555666
ENSG00000156599.6	ZDHHC5	turquoise	0.950634643
ENSG00000023041.7	ZDHHC6	turquoise	0.939196644
ENSG00000153786.8	ZDHHC7	turquoise	0.905047068
ENSG00000099904.11	ZDHHC8	turquoise	0.893956031

ENSG00000133519.8	ZDHHC8P1	turquoise	0.709353573
ENSG00000188706.8	ZDHHC9	brown	0.933451339
ENSG00000148516.17	ZEB1	blue	0.821434825
ENSG00000169554.12	ZEB2	brown	0.721008358
ENSG00000160445.6	ZER1	turquoise	0.944954127
ENSG00000104231.6	ZFAND1	turquoise	0.942952474
ENSG00000178381.7	ZFAND2A	turquoise	0.753743516
ENSG00000158552.8	ZFAND2B	turquoise	0.919529754
ENSG00000156639.7	ZFAND3	blue	0.922270935
ENSG00000172671.15	ZFAND4	blue	0.875333085
ENSG00000107372.8	ZFAND5	turquoise	0.957587283
ENSG00000086666.14	ZFAND6	blue	0.920406295
ENSG00000177410.8	ZFAS1	red	0.883811771
ENSG00000066827.11	ZFAT	turquoise	0.865434227
ENSG00000133858.11	ZFC3H1	turquoise	0.919956124
ENSG00000136367.12	ZFHX2	pink	0.818226008
ENSG00000140836.10	ZFHX3	turquoise	0.896099384
ENSG00000091656.11	ZFHX4	turquoise	0.836975172
ENSG00000253661.1	ZFHX4-AS1	blue	0.620539399
ENSG00000184517.7	ZFP1	turquoise	0.944332601
ENSG00000142065.9	ZFP14	turquoise	0.884134004
ENSG00000198939.3	ZFP2	yellow	0.84544793
ENSG00000196867.3	ZFP28	turquoise	0.924788835
ENSG00000180787.5	ZFP3	blue	0.868584992
ENSG00000120784.11	ZFP30	turquoise	0.898943346
ENSG00000128016.4	ZFP36	black	0.75565353
ENSG00000185650.8	ZFP36L1	green	0.740824121
ENSG00000152518.5	ZFP36L2	blue	0.705170131
ENSG00000136866.9	ZFP37	turquoise	0.908679298
ENSG00000181638.13	ZFP41	turquoise	0.803696096
ENSG00000204644.5	ZFP57	brown	0.702338603
ENSG00000196670.9	ZFP62	turquoise	0.855077777
ENSG00000020256.15	ZFP64	turquoise	0.949040251
ENSG00000187815.5	ZFP69	turquoise	0.883352446
ENSG00000187801.10	ZFP69B	turquoise	0.90207755
ENSG00000181007.7	ZFP82	turquoise	0.915539292
ENSG00000184939.11	ZFP90	turquoise	0.929242169
ENSG00000186660.14	ZFP91	blue	0.778076856
ENSG00000162300.7	ZFPL1	red	0.916439471
ENSG00000179588.4	ZFPM1	turquoise	0.664823817
ENSG00000169946.9	ZFPM2	turquoise	0.803318153
ENSG00000056097.11	ZFR	turquoise	0.943580175
ENSG00000105278.6	ZFR2	turquoise	0.815813071
ENSG00000005889.11	ZFX	turquoise	0.742698183
ENSG00000067646.7	ZFY	grey	0.262764052
ENSG00000165861.9	ZFYVE1	turquoise	0.949958186
ENSG00000039319.12	ZFYVE16	turquoise	0.799335709
ENSG00000166140.13	ZFYVE19	turquoise	0.873004041

ENSG00000131381.8	ZFYVE20	turquoise	0.932822729
ENSG00000100711.9	ZFYVE21	blue	0.775031969
ENSG00000072121.11	ZFYVE26	blue	0.94358263
ENSG00000155256.13	ZFYVE27	turquoise	0.965766589
ENSG00000159733.9	ZFYVE28	turquoise	0.917311743
ENSG00000157077.10	ZFYVE9	turquoise	0.948872446
ENSG00000220201.3	ZGLP1	yellow	0.790339175
ENSG00000197114.7	ZGPAT	turquoise	0.910970666
ENSG00000165156.10	ZHX1	blue	0.846031649
ENSG00000178764.6	ZHX2	brown	0.752044384
ENSG00000174306.17	ZHX3	blue	0.852401532
ENSG00000152977.5	ZIC1	blue	0.592928681
ENSG00000043355.6	ZIC2	grey	0.404084495
ENSG00000156925.7	ZIC3	brown	0.608869595
ENSG00000174963.13	ZIC4	turquoise	0.597166674
ENSG00000139800.8	ZIC5	grey	0.400065604
ENSG00000171649.7	ZIK1	turquoise	0.92668857
ENSG00000106261.12	ZKSCAN1	turquoise	0.873340997
ENSG00000155592.11	ZKSCAN2	turquoise	0.932323506
ENSG00000189298.9	ZKSCAN3	blue	0.856417408
ENSG00000187626.7	ZKSCAN4	turquoise	0.904627917
ENSG00000196652.7	ZKSCAN5	turquoise	0.931369107
ENSG00000196345.8	ZKSCAN7	turquoise	0.8362583
ENSG00000198315.6	ZKSCAN8	turquoise	0.803532842
ENSG00000166432.10	ZMAT1	yellow	0.829420761
ENSG00000146007.6	ZMAT2	turquoise	0.940537758
ENSG00000172667.6	ZMAT3	turquoise	0.928718236
ENSG00000165061.10	ZMAT4	turquoise	0.852584708
ENSG00000100319.11	ZMAT5	red	0.8491962
ENSG00000108175.12	ZMIZ1	blue	0.911506485
ENSG00000122515.10	ZMIZ2	turquoise	0.937642231
ENSG00000084073.4	ZMPSTE24	turquoise	0.857513459
ENSG00000197056.5	ZMYM1	blue	0.829415687
ENSG00000121741.12	ZMYM2	turquoise	0.954084343
ENSG00000147130.10	ZMYM3	turquoise	0.959729445
ENSG00000146463.7	ZMYM4	turquoise	0.959665488
ENSG00000132950.14	ZMYM5	turquoise	0.800498716
ENSG00000163867.12	ZMYM6	turquoise	0.963084448
ENSG00000243749.1	ZMYM6NB	blue	0.751840481
ENSG00000004838.9	ZMYND10	pink	0.904579007
ENSG00000015171.14	ZMYND11	turquoise	0.902063328
ENSG00000141497.9	ZMYND15	blue	0.688475059
ENSG00000165724.5	ZMYND19	turquoise	0.940233021
ENSG00000101040.15	ZMYND8	turquoise	0.930308379
ENSG00000256223.1	ZNF10	turquoise	0.894653778
ENSG00000197020.6	ZNF100	blue	0.772214115
ENSG00000181896.7	ZNF101	turquoise	0.899378647
ENSG00000103994.12	ZNF106	turquoise	0.909954973

ENSG00000196247.7	ZNF107	turquoise	0.797750196
ENSG00000062370.12	ZNF112	turquoise	0.811493615
ENSG00000152926.10	ZNF117	blue	0.80607215
ENSG00000164631.14	ZNF12	turquoise	0.918652775
ENSG00000197961.7	ZNF121	blue	0.839954318
ENSG00000172262.7	ZNF131	turquoise	0.948752126
ENSG00000131849.10	ZNF132	turquoise	0.93669734
ENSG00000125846.11	ZNF133	yellow	0.950149684
ENSG00000213762.6	ZNF134	turquoise	0.920722875
ENSG00000176293.15	ZNF135	turquoise	0.904259301
ENSG00000196646.7	ZNF136	turquoise	0.87884329
ENSG00000123870.9	ZNF137P	turquoise	0.797094561
ENSG00000197008.5	ZNF138	turquoise	0.897226391
ENSG00000105708.8	ZNF14	turquoise	0.937231239
ENSG00000196387.5	ZNF140	turquoise	0.926125324
ENSG00000131127.9	ZNF141	yellow	0.88014859
ENSG00000115568.11	ZNF142	turquoise	0.954142198
ENSG00000166478.5	ZNF143	turquoise	0.761284883
ENSG00000167635.7	ZNF146	brown	0.877475038
ENSG00000163848.14	ZNF148	turquoise	0.938532631
ENSG00000179909.11	ZNF154	turquoise	0.920612368
ENSG00000204920.6	ZNF155	turquoise	0.813184835
ENSG00000170631.10	ZNF16	turquoise	0.933761143
ENSG00000170949.13	ZNF160	blue	0.921438722
ENSG00000197279.3	ZNF165	turquoise	0.867559548
ENSG00000175787.12	ZNF169	turquoise	0.938846587
ENSG00000186272.8	ZNF17	turquoise	0.944702241
ENSG00000103343.8	ZNF174	turquoise	0.971895433
ENSG00000105497.3	ZNF175	turquoise	0.896845044
ENSG00000188629.7	ZNF177	blue	0.823135406
ENSG00000154957.9	ZNF18	turquoise	0.879627137
ENSG00000167384.6	ZNF180	turquoise	0.910601777
ENSG00000197841.10	ZNF181	blue	0.882674671
ENSG00000147118.6	ZNF182	yellow	0.934132735
ENSG00000096654.11	ZNF184	turquoise	0.920191786
ENSG00000147394.14	ZNF185	turquoise	0.878542853
ENSG00000136870.6	ZNF189	turquoise	0.843467397
ENSG00000157429.11	ZNF19	pink	0.892676979
ENSG00000226314.3	ZNF192P1	yellow	0.799889374
ENSG00000005801.12	ZNF195	turquoise	0.905622527
ENSG00000186448.10	ZNF197	turquoise	0.930741366
ENSG00000163067.10	ZNF2	turquoise	0.9206143
ENSG00000010539.7	ZNF200	turquoise	0.929855872
ENSG00000166261.6	ZNF202	yellow	0.899929651
ENSG00000204789.3	ZNF204P	turquoise	0.927915607
ENSG00000122386.6	ZNF205	red	0.862465335
ENSG00000263214.1	ZNF205-AS1	turquoise	0.761942586
ENSG00000010244.12	ZNF207	turquoise	0.956513254

ENSG00000121417.9	ZNF211	yellow	0.951680192
ENSG00000170260.4	ZNF212	turquoise	0.90902798
ENSG00000085644.9	ZNF213	turquoise	0.928511912
ENSG00000149050.5	ZNF214	pink	0.919959157
ENSG00000171940.9	ZNF217	blue	0.819742176
ENSG00000165804.11	ZNF219	red	0.709294235
ENSG00000165512.4	ZNF22	blue	0.825818798
ENSG00000159885.9	ZNF222	turquoise	0.904016849
ENSG00000178386.8	ZNF223	turquoise	0.949656755
ENSG00000267680.1	ZNF224	blue	0.839080376
ENSG00000256294.3	ZNF225	turquoise	0.882581087
ENSG00000167380.12	ZNF226	turquoise	0.835013637
ENSG00000131115.11	ZNF227	turquoise	0.857023205
ENSG00000167383.4	ZNF229	turquoise	0.918881423
ENSG00000167377.13	ZNF23	turquoise	0.896626433
ENSG00000159882.8	ZNF230	turquoise	0.917846156
ENSG00000167840.9	ZNF232	turquoise	0.763364676
ENSG00000159915.8	ZNF233	turquoise	0.898838153
ENSG00000263002.3	ZNF234	blue	0.891198937
ENSG00000159917.10	ZNF235	turquoise	0.913344251
ENSG00000130856.11	ZNF236	turquoise	0.94265318
ENSG00000196793.9	ZNF239	turquoise	0.889649821
ENSG00000172466.11	ZNF24	blue	0.881157394
ENSG00000198105.7	ZNF248	turquoise	0.935429962
ENSG00000175395.11	ZNF25	turquoise	0.959102817
ENSG00000196150.9	ZNF250	turquoise	0.913135085
ENSG00000198169.4	ZNF251	yellow	0.936388987
ENSG00000196922.6	ZNF252P	blue	0.798805006
ENSG00000256771.2	ZNF253	turquoise	0.855385946
ENSG00000213096.5	ZNF254	turquoise	0.868082116
ENSG00000152454.3	ZNF256	turquoise	0.874246831
ENSG00000109917.6	ZNF259	turquoise	0.876504755
ENSG00000254004.2	ZNF260	blue	0.922589271
ENSG00000006194.6	ZNF263	turquoise	0.95190816
ENSG00000174652.13	ZNF266	turquoise	0.80011387
ENSG00000185947.10	ZNF267	turquoise	0.844169069
ENSG00000090612.16	ZNF268	turquoise	0.863155791
ENSG00000257267.1	ZNF271	blue	0.870956048
ENSG00000198039.7	ZNF273	pink	0.936367885
ENSG00000171606.13	ZNF274	blue	0.881096032
ENSG00000063587.12	ZNF275	turquoise	0.934548011
ENSG00000158805.7	ZNF276	yellow	0.933949872
ENSG00000198839.5	ZNF277	turquoise	0.889477703
ENSG00000198538.6	ZNF28	blue	0.877903081
ENSG00000198477.3	ZNF280B	turquoise	0.943689688
ENSG00000056277.11	ZNF280C	turquoise	0.788499135
ENSG00000137871.15	ZNF280D	turquoise	0.882880519
ENSG00000162702.7	ZNF281	brown	0.791661561

ENSG00000170265.7	ZNF282	turquoise	0.919119869
ENSG00000167637.12	ZNF283	turquoise	0.899248053
ENSG00000186026.6	ZNF284	turquoise	0.863043162
ENSG00000267508.1	ZNF285	turquoise	0.898252761
ENSG00000187607.11	ZNF286A	turquoise	0.927821365
ENSG00000141040.10	ZNF287	turquoise	0.877156192
ENSG00000188994.8	ZNF292	turquoise	0.874429744
ENSG00000166526.12	ZNF3	turquoise	0.868344542
ENSG00000168661.10	ZNF30	turquoise	0.923877367
ENSG00000145908.8	ZNF300	turquoise	0.762195466
ENSG00000197083.7	ZNF300P1	blue	0.49047602
ENSG00000089335.16	ZNF302	turquoise	0.906351844
ENSG00000131845.10	ZNF304	turquoise	0.948503046
ENSG00000205903.2	ZNF316	yellow	0.822368546
ENSG00000130803.10	ZNF317	turquoise	0.946110575
ENSG00000171467.11	ZNF318	turquoise	0.929079034
ENSG00000166188.2	ZNF319	blue	0.883545473
ENSG00000169740.9	ZNF32	turquoise	0.903352586
ENSG00000226245.1	ZNF32-AS1	yellow	0.771583549
ENSG00000230565.1	ZNF32-AS2	blue	0.793147286
ENSG00000182986.8	ZNF320	turquoise	0.900589669
ENSG00000181315.6	ZNF322	blue	0.818393746
ENSG00000083812.7	ZNF324	turquoise	0.950049832
ENSG00000249471.3	ZNF324B	turquoise	0.927507085
ENSG00000162664.12	ZNF326	brown	0.798988412
ENSG00000181894.10	ZNF329	turquoise	0.961513208
ENSG00000109445.6	ZNF330	turquoise	0.950580408
ENSG00000130844.12	ZNF331	turquoise	0.927182724
ENSG00000160961.7	ZNF333	blue	0.911230926
ENSG00000198185.7	ZNF334	yellow	0.890609684
ENSG00000198026.6	ZNF335	turquoise	0.950689486
ENSG00000130684.9	ZNF337	turquoise	0.895897029
ENSG00000189180.11	ZNF33A	turquoise	0.917598144
ENSG00000196693.10	ZNF33B	turquoise	0.888853222
ENSG00000196378.7	ZNF34	turquoise	0.864463742
ENSG00000131061.9	ZNF341	turquoise	0.893978407
ENSG00000088876.7	ZNF343	turquoise	0.896308016
ENSG00000251247.5	ZNF345	turquoise	0.863409676
ENSG00000113761.7	ZNF346	turquoise	0.966301888
ENSG00000197937.8	ZNF347	blue	0.85445023
ENSG00000169981.6	ZNF35	turquoise	0.845886972
ENSG00000256683.2	ZNF350	turquoise	0.901507394
ENSG00000169131.6	ZNF354A	turquoise	0.926378082
ENSG00000178338.6	ZNF354B	turquoise	0.872726845
ENSG00000177932.6	ZNF354C	blue	0.848956967
ENSG00000198816.5	ZNF358	red	0.850910272
ENSG00000160094.10	ZNF362	turquoise	0.945348877
ENSG00000138311.11	ZNF365	turquoise	0.90237159

ENSG00000178175.7	ZNF366	black	0.541736967
ENSG00000165244.6	ZNF367	turquoise	0.875798681
ENSG00000075407.13	ZNF37A	turquoise	0.896837923
ENSG00000234420.3	ZNF37BP	turquoise	0.86901842
ENSG00000161298.12	ZNF382	turquoise	0.91171936
ENSG00000188283.7	ZNF383	turquoise	0.848531812
ENSG00000126746.13	ZNF384	turquoise	0.919575948
ENSG00000161642.13	ZNF385A	green	0.596468075
ENSG00000144331.14	ZNF385B	midnightblue	0.898883151
ENSG00000187595.10	ZNF385C	turquoise	0.752032275
ENSG00000151789.5	ZNF385D	turquoise	0.788355428
ENSG00000124613.4	ZNF391	turquoise	0.900144402
ENSG00000160908.14	ZNF394	turquoise	0.840628689
ENSG00000186918.9	ZNF395	turquoise	0.80528298
ENSG00000186496.6	ZNF396	pink	0.847210719
ENSG00000186812.8	ZNF397	turquoise	0.796775428
ENSG00000197024.4	ZNF398	turquoise	0.948490528
ENSG00000176222.7	ZNF404	blue	0.758510742
ENSG00000215421.5	ZNF407	turquoise	0.9422236
ENSG00000175213.2	ZNF408	turquoise	0.829515327
ENSG00000147124.8	ZNF41	turquoise	0.949933404
ENSG00000119725.13	ZNF410	turquoise	0.835835962
ENSG00000133250.9	ZNF414	turquoise	0.882734689
ENSG00000170954.7	ZNF415	turquoise	0.913409524
ENSG00000083817.8	ZNF416	turquoise	0.932392229
ENSG00000173480.6	ZNF417	turquoise	0.872041882
ENSG00000196724.8	ZNF418	turquoise	0.909588213
ENSG00000105136.15	ZNF419	turquoise	0.894094229
ENSG00000197050.6	ZNF420	turquoise	0.934888325
ENSG00000102935.7	ZNF423	green	0.896053183
ENSG00000204947.4	ZNF425	turquoise	0.928114763
ENSG00000131116.7	ZNF428	turquoise	0.837266048
ENSG00000197013.5	ZNF429	turquoise	0.866214502
ENSG00000198521.7	ZNF43	turquoise	0.808359631
ENSG00000118620.8	ZNF430	turquoise	0.920459494
ENSG00000196705.4	ZNF431	turquoise	0.836702831
ENSG00000256087.2	ZNF432	turquoise	0.907402157
ENSG00000197647.7	ZNF433	blue	0.770582823
ENSG00000125945.10	ZNF436	turquoise	0.91425442
ENSG00000183621.11	ZNF438	blue	0.917479202
ENSG00000171291.4	ZNF439	turquoise	0.936645149
ENSG00000197857.9	ZNF44	blue	0.852389018
ENSG00000171295.8	ZNF440	turquoise	0.928957851
ENSG00000197044.6	ZNF441	turquoise	0.867302006
ENSG00000180855.11	ZNF443	turquoise	0.807361553
ENSG00000167685.10	ZNF444	turquoise	0.836287747
ENSG00000185219.11	ZNF445	turquoise	0.933580084
ENSG00000083838.11	ZNF446	turquoise	0.940387713

ENSG00000173275.8	ZNF449	turquoise	0.852510936
ENSG00000124459.7	ZNF45	turquoise	0.937229762
ENSG00000112200.12	ZNF451	turquoise	0.890720573
ENSG00000178187.3	ZNF454	turquoise	0.869348088
ENSG00000197808.7	ZNF461	turquoise	0.948302665
ENSG00000148143.8	ZNF462	turquoise	0.870000948
ENSG00000181444.8	ZNF467	turquoise	0.665487111
ENSG00000204604.5	ZNF468	turquoise	0.833893717
ENSG00000225614.2	ZNF469	grey	0.668928251
ENSG00000197016.7	ZNF470	turquoise	0.878345351
ENSG00000196263.3	ZNF471	turquoise	0.849599744
ENSG00000142528.11	ZNF473	cyan	0.89076357
ENSG00000180035.6	ZNF48	turquoise	0.951783271
ENSG00000198464.9	ZNF480	blue	0.871549025
ENSG00000173258.8	ZNF483	turquoise	0.751934476
ENSG00000127081.9	ZNF484	turquoise	0.928421816
ENSG00000198298.8	ZNF485	turquoise	0.861499712
ENSG00000243660.5	ZNF487	pink	0.943062018
ENSG00000165388.8	ZNF488	brown	0.854002793
ENSG00000188033.5	ZNF490	turquoise	0.877301131
ENSG00000177599.8	ZNF491	turquoise	0.864655569
ENSG00000196268.7	ZNF493	turquoise	0.930151449
ENSG00000162714.8	ZNF496	turquoise	0.890710099
ENSG00000174586.6	ZNF497	turquoise	0.907805753
ENSG00000103199.9	ZNF500	turquoise	0.909474541
ENSG00000186446.7	ZNF501	blue	0.823781413
ENSG00000196653.7	ZNF502	turquoise	0.906151025
ENSG00000165655.14	ZNF503	turquoise	0.868442117
ENSG00000237149.4	ZNF503-AS2	turquoise	0.848557856
ENSG00000081665.9	ZNF506	turquoise	0.940092485
ENSG00000168813.12	ZNF507	turquoise	0.926311886
ENSG00000081386.8	ZNF510	turquoise	0.95750643
ENSG00000198546.10	ZNF511	turquoise	0.872014083
ENSG00000243943.5	ZNF512	turquoise	0.948139352
ENSG00000196700.3	ZNF512B	turquoise	0.938708213
ENSG00000163795.9	ZNF513	turquoise	0.909198308
ENSG00000144026.7	ZNF514	yellow	0.862754159
ENSG00000101493.6	ZNF516	turquoise	0.866913813
ENSG00000197363.5	ZNF517	turquoise	0.927209299
ENSG00000177853.10	ZNF518A	turquoise	0.836359338
ENSG00000178163.3	ZNF518B	turquoise	0.905086064
ENSG00000175322.7	ZNF519	turquoise	0.873108639
ENSG00000198795.6	ZNF521	blue	0.797855283
ENSG00000171443.6	ZNF524	red	0.766089477
ENSG00000203326.5	ZNF525	turquoise	0.892176931
ENSG00000167625.6	ZNF526	turquoise	0.901634045
ENSG00000189164.10	ZNF527	turquoise	0.923106738
ENSG00000167555.9	ZNF528	turquoise	0.876186779

ENSG00000186020.8	ZNF529	turquoise	0.961950187
ENSG00000183647.6	ZNF530	turquoise	0.904072288
ENSG00000074657.9	ZNF532	turquoise	0.863319144
ENSG00000198633.6	ZNF534	turquoise	0.787462626
ENSG00000198597.4	ZNF536	brown	0.94147731
ENSG00000171817.12	ZNF540	turquoise	0.921111472
ENSG00000240225.6	ZNF542	turquoise	0.966623463
ENSG00000178229.7	ZNF543	turquoise	0.868714705
ENSG00000198131.9	ZNF544	turquoise	0.946630724
ENSG00000187187.9	ZNF546	turquoise	0.890594768
ENSG00000152433.10	ZNF547	yellow	0.849400604
ENSG00000188785.7	ZNF548	blue	0.899785292
ENSG00000121406.4	ZNF549	turquoise	0.918058524
ENSG00000251369.4	ZNF550	turquoise	0.935015776
ENSG00000204519.6	ZNF551	turquoise	0.790166385
ENSG00000178935.5	ZNF552	turquoise	0.887809403
ENSG00000172006.7	ZNF554	turquoise	0.954413236
ENSG00000186300.7	ZNF555	turquoise	0.862178132
ENSG00000130544.7	ZNF557	turquoise	0.867301295
ENSG00000167785.4	ZNF558	turquoise	0.839963065
ENSG00000188321.9	ZNF559	turquoise	0.868600709
ENSG00000171469.6	ZNF561	turquoise	0.899381122
ENSG00000171466.5	ZNF562	turquoise	0.941112317
ENSG00000188868.9	ZNF563	turquoise	0.84813562
ENSG00000249709.3	ZNF564	blue	0.83846433
ENSG00000196357.7	ZNF565	turquoise	0.89692861
ENSG00000186017.10	ZNF566	turquoise	0.921609901
ENSG00000189042.9	ZNF567	turquoise	0.918599714
ENSG00000198453.8	ZNF568	turquoise	0.870210963
ENSG00000196437.6	ZNF569	turquoise	0.931776973
ENSG00000171970.8	ZNF57	turquoise	0.919740676
ENSG00000180479.9	ZNF571	blue	0.869461094
ENSG00000267470.1	ZNF571-AS1	turquoise	0.907348219
ENSG00000180938.5	ZNF572	blue	0.789238829
ENSG00000105732.9	ZNF574	turquoise	0.924693295
ENSG00000176472.6	ZNF575	turquoise	0.885362438
ENSG00000124444.11	ZNF576	turquoise	0.955842781
ENSG00000161551.8	ZNF577	yellow	0.81627018
ENSG00000218891.2	ZNF579	turquoise	0.773236519
ENSG00000213015.4	ZNF580	turquoise	0.818092973
ENSG00000171425.5	ZNF581	turquoise	0.863401908
ENSG00000018869.12	ZNF582	turquoise	0.919101162
ENSG00000267454.1	ZNF582-AS1	turquoise	0.929834075
ENSG00000198440.5	ZNF583	turquoise	0.890113106
ENSG00000171574.13	ZNF584	turquoise	0.947226158
ENSG00000196967.6	ZNF585A	turquoise	0.9417923
ENSG00000245680.5	ZNF585B	turquoise	0.937900936
ENSG00000083828.11	ZNF586	turquoise	0.778656529

ENSG00000198466.8	ZNF587	turquoise	0.929645944
ENSG00000269343.2	ZNF587B	turquoise	0.86409335
ENSG00000164048.9	ZNF589	turquoise	0.880516783
ENSG00000166716.5	ZNF592	turquoise	0.933505825
ENSG00000180626.9	ZNF594	turquoise	0.906423977
ENSG00000172748.8	ZNF596	yellow	0.821443688
ENSG00000167962.8	ZNF598	turquoise	0.908563542
ENSG00000153896.13	ZNF599	turquoise	0.91038799
ENSG00000189190.7	ZNF600	blue	0.684533305
ENSG00000196458.6	ZNF605	turquoise	0.885120475
ENSG00000166704.7	ZNF606	turquoise	0.872774674
ENSG00000198182.8	ZNF607	turquoise	0.936838745
ENSG00000168916.11	ZNF608	blue	0.866147962
ENSG00000180357.5	ZNF609	turquoise	0.91346442
ENSG00000167554.10	ZNF610	turquoise	0.912664201
ENSG00000213020.4	ZNF611	turquoise	0.926090101
ENSG00000176024.12	ZNF613	turquoise	0.903767119
ENSG00000142556.14	ZNF614	turquoise	0.886930934
ENSG00000197619.9	ZNF615	turquoise	0.944230021
ENSG00000204611.2	ZNF616	turquoise	0.907879402
ENSG00000157657.10	ZNF618	yellow	0.865005061
ENSG00000177873.8	ZNF619	blue	0.87732062
ENSG00000177842.7	ZNF620	turquoise	0.821170876
ENSG00000172888.7	ZNF621	blue	0.846868068
ENSG00000173545.4	ZNF622	turquoise	0.903323846
ENSG00000183309.7	ZNF623	turquoise	0.909707215
ENSG00000197566.5	ZNF624	turquoise	0.883228973
ENSG00000188171.10	ZNF626	turquoise	0.910416958
ENSG00000198551.5	ZNF627	turquoise	0.862861896
ENSG00000197483.8	ZNF628	turquoise	0.829127852
ENSG00000102870.4	ZNF629	turquoise	0.915427651
ENSG00000221994.6	ZNF630	turquoise	0.810666825
ENSG00000075292.14	ZNF638	turquoise	0.933844007
ENSG00000121864.5	ZNF639	turquoise	0.930676222
ENSG00000167528.8	ZNF641	blue	0.882163804
ENSG00000122482.16	ZNF644	turquoise	0.825534528
ENSG00000167395.10	ZNF646	turquoise	0.874430476
ENSG00000198093.6	ZNF649	turquoise	0.838234917
ENSG00000198740.4	ZNF652	brown	0.823730487
ENSG00000161914.5	ZNF653	turquoise	0.922109222
ENSG00000175105.5	ZNF654	turquoise	0.851445725
ENSG00000197343.6	ZNF655	turquoise	0.96983375
ENSG00000196409.7	ZNF658	turquoise	0.862848849
ENSG00000144792.5	ZNF660	blue	0.754212656
ENSG00000182983.10	ZNF662	blue	0.81285807
ENSG00000215452.3	ZNF663P	yellow	0.802550716
ENSG00000179195.11	ZNF664	blue	0.757859105
ENSG00000198046.7	ZNF667	turquoise	0.936690835

ENSG00000166770.6	ZNF667-AS1	turquoise	0.9425576
ENSG00000167394.8	ZNF668	turquoise	0.902312842
ENSG00000188295.10	ZNF669	turquoise	0.776456488
ENSG00000083814.8	ZNF671	turquoise	0.936968081
ENSG00000171161.8	ZNF672	turquoise	0.912797207
ENSG00000251192.3	ZNF674	turquoise	0.911692255
ENSG00000230844.2	ZNF674-AS1	turquoise	0.927251756
ENSG00000197372.5	ZNF675	turquoise	0.924998318
ENSG00000181450.13	ZNF678	blue	0.847856932
ENSG00000173041.7	ZNF680	blue	0.774946329
ENSG00000197124.7	ZNF682	turquoise	0.853366358
ENSG00000117010.11	ZNF684	turquoise	0.824165526
ENSG00000143373.13	ZNF687	turquoise	0.879578833
ENSG00000229809.4	ZNF688	red	0.852782619
ENSG00000156853.7	ZNF689	turquoise	0.962308119
ENSG00000198429.5	ZNF69	yellow	0.824282286
ENSG00000164011.13	ZNF691	blue	0.859237646
ENSG00000171163.11	ZNF692	yellow	0.913116201
ENSG00000185730.3	ZNF696	turquoise	0.907651926
ENSG00000143067.4	ZNF697	turquoise	0.861254018
ENSG00000147789.11	ZNF7	turquoise	0.934642943
ENSG00000187792.3	ZNF70	blue	0.775273621
ENSG00000196757.3	ZNF700	blue	0.871118321
ENSG00000167562.7	ZNF701	blue	0.860593892
ENSG00000242779.2	ZNF702P	turquoise	0.787531238
ENSG00000183779.5	ZNF703	green	0.66221927
ENSG00000164684.9	ZNF704	turquoise	0.82224783
ENSG00000120963.7	ZNF706	turquoise	0.97068398
ENSG00000181135.11	ZNF707	turquoise	0.938280846
ENSG00000182141.5	ZNF708	turquoise	0.807263716
ENSG00000242852.2	ZNF709	pink	0.888359646
ENSG00000197951.4	ZNF71	turquoise	0.901682795
ENSG00000140548.5	ZNF710	turquoise	0.895468619
ENSG00000147180.12	ZNF711	turquoise	0.915035639
ENSG00000160352.11	ZNF714	turquoise	0.850811799
ENSG00000250312.2	ZNF718	turquoise	0.794520742
ENSG00000197302.6	ZNF720	turquoise	0.944178316
ENSG00000182903.11	ZNF721	turquoise	0.962562721
ENSG00000234444.5	ZNF736	turquoise	0.875730407
ENSG00000237440.4	ZNF737	turquoise	0.925584687
ENSG00000172687.9	ZNF738	brown	0.828702316
ENSG00000185252.13	ZNF74	turquoise	0.949393864
ENSG00000139651.9	ZNF740	turquoise	0.946271242
ENSG00000181220.11	ZNF746	turquoise	0.949400006
ENSG00000169955.6	ZNF747	turquoise	0.961637135
ENSG00000186230.6	ZNF749	turquoise	0.822398094
ENSG00000162086.10	ZNF75A	turquoise	0.914356895
ENSG00000186376.10	ZNF75D	turquoise	0.913573347

ENSG00000065029.10	ZNF76	turquoise	0.935595171
ENSG00000160336.10	ZNF761	blue	0.845684224
ENSG00000197054.7	ZNF763	blue	0.778933623
ENSG00000169951.5	ZNF764	turquoise	0.927813305
ENSG00000196417.8	ZNF765	turquoise	0.756772983
ENSG00000196214.6	ZNF766	blue	0.910713154
ENSG00000133624.9	ZNF767	yellow	0.968158898
ENSG00000169957.8	ZNF768	blue	0.766465166
ENSG00000175691.8	ZNF77	turquoise	0.80398151
ENSG00000179965.7	ZNF771	turquoise	0.757432146
ENSG00000197128.7	ZNF772	blue	0.84785068
ENSG00000152439.8	ZNF773	turquoise	0.897613967
ENSG00000196391.6	ZNF774	turquoise	0.924107686
ENSG00000196456.4	ZNF775	turquoise	0.913477177
ENSG00000152443.8	ZNF776	turquoise	0.946617427
ENSG00000196453.7	ZNF777	turquoise	0.922165771
ENSG00000197782.10	ZNF780A	turquoise	0.873641075
ENSG00000128000.11	ZNF780B	turquoise	0.905360749
ENSG00000196381.6	ZNF781	turquoise	0.844337239
ENSG00000196597.7	ZNF782	turquoise	0.843717527
ENSG00000204946.5	ZNF783	yellow	0.962978638
ENSG00000179922.5	ZNF784	blue	0.810721261
ENSG00000197162.8	ZNF785	turquoise	0.941742232
ENSG00000197362.9	ZNF786	turquoise	0.913512503
ENSG00000142409.4	ZNF787	red	0.899565592
ENSG00000198556.9	ZNF789	yellow	0.912348323
ENSG00000196152.6	ZNF79	turquoise	0.881955961
ENSG00000197863.4	ZNF790	turquoise	0.879427129
ENSG00000173875.9	ZNF791	turquoise	0.926683788
ENSG00000188227.8	ZNF793	turquoise	0.92901341
ENSG00000196466.6	ZNF799	blue	0.830443678
ENSG00000048405.5	ZNF800	blue	0.922051741
ENSG00000170396.6	ZNF804A	turquoise	0.880915298
ENSG00000198482.6	ZNF808	turquoise	0.880265974
ENSG00000197779.9	ZNF81	turquoise	0.740203155
ENSG00000204514.5	ZNF814	yellow	0.888809604
ENSG00000235944.3	ZNF815P	turquoise	0.883894451
ENSG00000180257.8	ZNF816	blue	0.818067166
ENSG00000269001.1	ZNF818P	turquoise	0.733015728
ENSG00000102984.10	ZNF821	turquoise	0.91548167
ENSG00000197933.8	ZNF823	turquoise	0.866873465
ENSG00000151612.11	ZNF827	turquoise	0.929567529
ENSG00000185869.9	ZNF829	turquoise	0.90176839
ENSG00000167766.14	ZNF83	turquoise	0.747250972
ENSG00000198783.4	ZNF830	turquoise	0.955893621
ENSG00000127903.12	ZNF835	turquoise	0.892722957
ENSG00000196267.8	ZNF836	turquoise	0.923179693
ENSG00000152475.6	ZNF837	turquoise	0.689621877

ENSG00000022976.11	ZNF839	turquoise	0.956021247
ENSG00000197608.7	ZNF841	turquoise	0.881439227
ENSG00000223547.5	ZNF844	blue	0.776648052
ENSG00000213799.6	ZNF845	turquoise	0.817382578
ENSG00000196605.3	ZNF846	blue	0.785219868
ENSG00000236609.3	ZNF853	red	0.692653873
ENSG00000106479.6	ZNF862	turquoise	0.909825303
ENSG00000261221.1	ZNF865	turquoise	0.886851055
ENSG00000198155.5	ZNF876P	turquoise	0.848358356
ENSG00000234284.2	ZNF879	blue	0.834633956
ENSG00000221923.4	ZNF880	blue	0.714822363
ENSG00000228623.2	ZNF883	turquoise	0.911127802
ENSG00000167232.9	ZNF91	turquoise	0.947283925
ENSG00000146757.9	ZNF92	turquoise	0.830197479
ENSG00000197360.5	ZNF98	grey	0.192053687
ENSG00000124201.10	ZNFX1	blue	0.923272348
ENSG00000106400.7	ZNHIT1	red	0.968727416
ENSG00000174276.5	ZNHIT2	turquoise	0.867974073
ENSG00000108278.7	ZNHIT3	turquoise	0.956527024
ENSG00000117174.6	ZNHIT6	turquoise	0.887288251
ENSG00000066379.10	ZNRD1	turquoise	0.764438772
ENSG00000204623.4	ZNRD1-AS1	yellow	0.876291564
ENSG00000186187.7	ZNRF1	turquoise	0.951187568
ENSG00000180233.9	ZNRF2	blue	0.815582891
ENSG00000183579.11	ZNRF3	green	0.850614872
ENSG00000188372.10	ZP3	turquoise	0.777079282
ENSG00000019995.6	ZRANB1	turquoise	0.929575509
ENSG00000132485.8	ZRANB2	turquoise	0.964000961
ENSG00000121988.13	ZRANB3	turquoise	0.920412623
ENSG00000212643.1	ZRSR1	blue	0.729979711
ENSG00000169249.8	ZRSR2	yellow	0.814694976
ENSG00000152467.5	ZSCAN1	turquoise	0.772225403
ENSG00000158691.10	ZSCAN12	turquoise	0.886619015
ENSG00000219891.2	ZSCAN12P1	turquoise	0.801648446
ENSG00000196812.4	ZSCAN16	brown	0.785573935
ENSG00000269293.2	ZSCAN16-AS1	red	0.938084877
ENSG00000121413.8	ZSCAN18	turquoise	0.949922828
ENSG00000176371.9	ZSCAN2	turquoise	0.942080961
ENSG00000166529.10	ZSCAN21	turquoise	0.855175144
ENSG00000182318.5	ZSCAN22	turquoise	0.899673244
ENSG00000197037.6	ZSCAN25	turquoise	0.919547847
ENSG00000140265.8	ZSCAN29	turquoise	0.849177637
ENSG00000186814.8	ZSCAN30	yellow	0.943038733
ENSG00000235109.3	ZSCAN31	turquoise	0.571332336
ENSG00000140987.15	ZSCAN32	brown	0.863939727
ENSG00000131848.5	ZSCAN5A	turquoise	0.925891746
ENSG00000137185.7	ZSCAN9	blue	0.813610342
ENSG00000168612.4	ZSWIM1	turquoise	0.926222283

ENSG00000132801.5	ZSWIM3	turquoise	0.923232052
ENSG00000132003.5	ZSWIM4	blue	0.788129253
ENSG00000162415.6	ZSWIM5	turquoise	0.932536737
ENSG00000130449.5	ZSWIM6	turquoise	0.934640883
ENSG00000214941.3	ZSWIM7	turquoise	0.877477135
ENSG00000214655.6	ZSWIM8	turquoise	0.878879666
ENSG00000272589.1	ZSWIM8-AS1	yellow	0.790313453
ENSG00000153975.5	ZUFSP	turquoise	0.921133112
ENSG00000086827.4	ZW10	turquoise	0.910537541
ENSG00000174442.7	ZWILCH	turquoise	0.923008849
ENSG00000122952.12	ZWINT	turquoise	0.873371588
ENSG00000198205.5	ZXDA	turquoise	0.900986944
ENSG00000198455.3	ZXDB	turquoise	0.965494506
ENSG00000070476.10	ZXDC	turquoise	0.884105195
ENSG00000162378.8	ZYG11B	turquoise	0.951916765
ENSG00000159840.11	ZYX	yellow	0.844968711
ENSG00000074755.10	ZZEF1	turquoise	0.916448006
ENSG00000036549.8	ZZZ3	turquoise	0.886385791
