# Supplementary material for: Dissecting genetic architecture of rare dystonia: genetic, molecular and clinical insights
Source: J Med Genet. 2024 Mar 8;61(5):443–51. doi: 10.1136/jmg-2022-109099 (PMC11041572; doi:10.1136/jmg-2022-109099)
Supplement: Supplementary data [file jmg-2022-109099supp005.pdf]

CEN Genes in the putamen region with Module Membership Values

Ensembl Gene id	Gene Symbol	Module	Module Membership
ENSG00000175899	A2M	tan	0.66306465
ENSG00000245105	A2M-AS1	grey	0.2248224
ENSG00000166535	A2ML1	turquoise	0.48169117
ENSG00000256069	A2MP1	grey	-0.0917287
ENSG00000094914	AAAS	brown	-0.3940654
ENSG00000081760	AACS	green	-0.6395562
ENSG00000158122	AAED1	grey	-0.0398908
ENSG00000103591	AAGAB	brown	0.68845451
ENSG00000115977	AAK1	blue	0.70930417
ENSG00000087884	AAMDC	grey	-0.032373
ENSG00000127837	AAMP	grey	-0.1921318
ENSG00000131043	AAR2	grey	0.09745929
ENSG00000090861	AARS	turquoise	-0.4965996
ENSG00000124608	AARS2	grey	-0.0092808
ENSG00000266967	AARSD1	grey	-0.0186746
ENSG00000157426	AASDH	grey	0.00466183
ENSG00000149313	AASDHPPT	grey	-0.3000486
ENSG00000108270	AATF	darkgrey	0.62714862
ENSG00000181409	AATK	yellow	0.59814422
ENSG00000254180	AB015752.3	grey	0.33362938
ENSG00000183044	ABAT	grey	-0.1511097
ENSG00000226210	ABC7-42389800N	grey	-0.0046703
ENSG00000165029	ABCA1	turquoise	0.76082906
ENSG00000154263	ABCA10	grey	0.07911545
ENSG00000251595	ABCA11P	grey	-0.0027151
ENSG00000238098	ABCA17P	brown	0.41595143
ENSG00000107331	ABCA2	yellow	0.69316399
ENSG00000154265	ABCA5	blue	0.49267416
ENSG00000154262	ABCA6	grey	0.13862655
ENSG00000064687	ABCA7	brown	0.53514416
ENSG00000141338	ABCA8	yellow	0.81121051
ENSG00000154258	ABCA9	grey	0.18368873
ENSG00000085563	ABCB1	tan	0.89412658
ENSG00000135776	ABCB10	turquoise	0.41542418
ENSG00000115657	ABCB6	yellow	-0.6368025
ENSG00000131269	ABCB7	blue	-0.3772324
ENSG00000197150	ABCB8	lightcyan	-0.5148213
ENSG00000150967	ABCB9	turquoise	-0.4642364
ENSG00000103222	ABCC1	grey	0.03488883
ENSG00000124574	ABCC10	turquoise	-0.4020056
ENSG00000125257	ABCC4	cyan	0.5781445
ENSG00000114770	ABCC5	turquoise	-0.6390018
ENSG00000006071	ABCC8	blue	0.37513075
ENSG00000101986	ABCD1	grey	0.26160627
ENSG00000173208	ABCD2	turquoise	0.5324914
ENSG00000117528	ABCD3	turquoise	0.62710109

ENSG00000164163	ABCE1	salmon	0.61454993
ENSG00000204574	ABCF1	magenta	0.44318456
ENSG00000033050	ABCF2	grey	0.30543226
ENSG00000228769	ABCF2P2	grey	0.43100019
ENSG00000161204	ABCF3	blue	0.46496193
ENSG00000160179	ABCG1	yellow	0.45772994
ENSG00000118777	ABCG2	tan	0.65981175
ENSG00000143994	ABHD1	grey	0.24435682
ENSG00000144827	ABHD10	salmon	0.66540119
ENSG00000106077	ABHD11	grey	0.12731706
ENSG00000100997	ABHD12	brown	0.71007631
ENSG00000131969	ABHD12B	grey60	0.76547811
ENSG00000139826	ABHD13	grey	0.14953947
ENSG00000248487	ABHD14A	blue	0.62122151
ENSG00000114779	ABHD14B	blue	-0.4769726
ENSG00000168792	ABHD15	turquoise	0.44026734
ENSG00000183260	ABHD16B	grey	-0.0019942
ENSG00000129968	ABHD17A	grey	0.0646542
ENSG00000107362	ABHD17B	yellow	0.69112248
ENSG00000136379	ABHD17C	grey	-0.1203904
ENSG00000140526	ABHD2	turquoise	0.59885591
ENSG00000158201	ABHD3	turquoise	0.62990405
ENSG00000011198	ABHD5	turquoise	0.63322885
ENSG00000163686	ABHD6	yellow	0.52593371
ENSG00000127220	ABHD8	grey	0.36497369
ENSG00000136754	ABI1	turquoise	0.67639673
ENSG00000108798	ABI3	royalblue	0.84093879
ENSG00000154175	ABI3BP	black	-0.6968476
ENSG00000097007	ABL1	yellow	0.74004071
ENSG00000099204	ABLIM1	turquoise	0.65530679
ENSG00000163995	ABLIM2	blue	0.73981299
ENSG00000173210	ABLIM3	grey	-0.2310852
ENSG00000159842	ABR	yellowgreen	0.62706645
ENSG00000146386	ABRACL	yellow	-0.5823227
ENSG00000146109	ABT1	grey	0.2502012
ENSG00000114626	ABTB1	turquoise	-0.6170091
ENSG00000264194	AC000003.2	grey	0.35433721
ENSG00000221069	AC000029.1	grey	0.08027023
ENSG00000185065	AC000068.5	grey	0.36392382
ENSG00000270393	AC000095.9	grey	0.28225358
ENSG00000243107	AC000120.7	grey	0.11396581
ENSG00000224138	AC000123.4	grey	0.26932808
ENSG00000234459	AC002064.4	grey	0.01318353
ENSG00000261588	AC002310.17	grey	0.3044656
ENSG00000266269	AC002395.1	grey	0.63233348
ENSG00000267439	AC002398.11	grey	0.08401437
ENSG00000261528	AC002400.1	grey	0.43204444
ENSG00000231153	AC002429.4	grey	0.47905062

ENSG00000231170	AC002451.3	grey	0.21330958
ENSG00000223969	AC002456.2	grey	0.19334891
ENSG00000241764	AC002467.7	grey	0.06568374
ENSG00000269103	AC002472.1	grey	0.477353
ENSG00000264392	AC002477.1	grey	0.28259697
ENSG00000227782	AC002553.1	grey	0.53740435
ENSG00000252111	AC002981.1	grey	0.35570331
ENSG00000234358	AC003080.4	grey	0.01818591
ENSG00000267750	AC003102.3	grey	0.04553082
ENSG00000234494	AC003665.1	grey	0.16957309
ENSG00000243621	AC003989.3	grey	0.31628992
ENSG00000227948	AC003989.4	grey	0.29722023
ENSG00000268948	AC004017.1	grey	0.35844606
ENSG00000223442	AC004041.2	grey	0.11868569
ENSG00000245384	AC004053.1	blue	0.48267965
ENSG00000248984	AC004054.1	grey	0.18498372
ENSG00000196656	AC004057.1	grey	0.05463128
ENSG00000250670	AC004063.1	grey	0.27503268
ENSG00000268533	AC004076.7	grey	0.46936137
ENSG00000226851	AC004112.4	greenyellow	-0.4739153
ENSG00000260664	AC004158.3	green	0.75852527
ENSG00000230435	AC004160.4	grey	0.33863492
ENSG00000184616	AC004166.6	grey	-0.0835972
ENSG00000267778	AC004221.2	grey	-0.0480691
ENSG00000230612	AC004237.1	grey	0.17573046
ENSG00000270143	AC004257.3	grey	0.067704
ENSG00000005189	AC004381.6	grey	-0.0319716
ENSG00000269131	AC004447.2	brown	0.57707836
ENSG00000227078	AC004448.2	grey	0.29375338
ENSG00000268069	AC004466.1	grey	0.15149018
ENSG00000223461	AC004471.9	grey	0.30517427
ENSG00000225792	AC004540.4	brown	-0.4793724
ENSG00000214870	AC004540.5	blue	0.36144057
ENSG00000235669	AC004593.3	grey	-0.1383115
ENSG00000225877	AC004603.4	yellow	0.48748844
ENSG00000228680	AC004692.4	grey	0.32416695
ENSG00000213717	AC004692.5	grey	0.4161221
ENSG00000229944	AC004797.1	grey	0.3278666
ENSG00000269468	AC004824.2	grey	0.28420415
ENSG00000175873	AC004840.9	grey	0.26293719
ENSG00000252378	AC004851.1	grey	0.36241645
ENSG00000224804	AC004878.2	grey	0.2020492
ENSG00000254977	AC004878.8	grey	0.27174693
ENSG00000242687	AC004893.11	grey	0.24808125
ENSG00000222024	AC004945.1	yellow	0.47391605
ENSG00000239556	AC004951.5	grey	0.22068349
ENSG00000243554	AC004967.7	grey	0.39173575
ENSG00000214243	AC004980.10	grey	0.0913521

ENSG00000185040	AC004980.11	grey	0.08382516
ENSG00000205485	AC004980.7	grey	0.01696404
ENSG00000213888	AC005003.1	grey	0.32392245
ENSG00000231255	AC005009.1	grey	0.32236875
ENSG00000233073	AC005009.2	yellow	0.48504901
ENSG00000228421	AC005013.5	grey	0.16890281
ENSG00000226658	AC005037.4	grey	0.43105244
ENSG00000227331	AC005042.2	grey	0.21656779
ENSG00000204380	AC005042.4	yellowgreen	-0.6590937
ENSG00000224046	AC005076.5	grey	0.01310102
ENSG00000213549	AC005077.8	grey	0.37544244
ENSG00000196295	AC005154.6	grey	-0.0713288
ENSG00000244480	AC005154.7	grey	0.37039017
ENSG00000226874	AC005154.8	grey	0.47748681
ENSG00000229452	AC005162.4	grey	0.16564474
ENSG00000233517	AC005162.5	grey	0.17098926
ENSG00000269694	AC005197.2	grey	0.02620228
ENSG00000236352	AC005220.3	green	0.65017897
ENSG00000231595	AC005224.2	grey	0.37534096
ENSG00000264918	AC005229.1	grey	0.42651335
ENSG00000197462	AC005276.1	grey	-0.1184435
ENSG00000265904	AC005288.1	grey	0.40592862
ENSG00000233852	AC005304.1	grey	0.37758094
ENSG00000267283	AC005306.3	grey	0.01423522
ENSG00000231812	AC005326.2	grey	0.35949454
ENSG00000267372	AC005330.2	grey	0.15089139
ENSG00000267056	AC005336.4	turquoise	0.40484635
ENSG00000248493	AC005351.1	grey	0.19945066
ENSG00000250994	AC005355.1	greenyellow	-0.451123
ENSG00000262686	AC005356.1	grey	-0.1322563
ENSG00000257040	AC005477.1	black	-0.6924207
ENSG00000222012	AC005481.5	grey	0.0015507
ENSG00000223878	AC005517.3	grey	0.0922031
ENSG00000230190	AC005518.2	yellow	0.47202116
ENSG00000258559	AC005519.4	grey	0.11884817
ENSG00000230825	AC005532.5	grey	0.27200704
ENSG00000228937	AC005539.2	grey	0.38512767
ENSG00000214719	AC005562.1	grey	0.08538804
ENSG00000231185	AC005592.2	greenyellow	-0.4741987
ENSG00000269296	AC005614.3	grey	0.02904314
ENSG00000263422	AC005702.1	grey	0.26235089
ENSG00000266701	AC005702.4	grey	0.54357869
ENSG00000225157	AC005779.1	grey	0.24806366
ENSG00000267473	AC005789.11	grey	0.14395196
ENSG00000267161	AC005943.5	grey	0.27417513
ENSG00000267205	AC005954.4	grey	0.11917119
ENSG00000231515	AC006003.3	grey	0.0732256
ENSG00000203335	AC006019.3	grey	0.24652296

ENSG00000227719	AC006042.6	grey	0.07398319
ENSG00000233108	AC006042.7	grey	0.18660379
ENSG00000233264	AC006042.8	grey	0.30209586
ENSG00000269793	AC006115.3	grey	-0.2402365
ENSG00000266631	AC006116.1	grey	0.53176228
ENSG00000267192	AC006116.12	grey	0.33131676
ENSG00000267145	AC006116.14	grey	0.30302061
ENSG00000267429	AC006116.15	grey	0.18796219
ENSG00000267298	AC006116.19	grey	0.32523852
ENSG00000267710	AC006116.20	grey	0.08366538
ENSG00000267606	AC006116.21	grey	0.46073395
ENSG00000267776	AC006116.24	grey	0.17389999
ENSG00000226323	AC006150.1	black	0.6296692
ENSG00000249502	AC006160.5	blue	-0.4324786
ENSG00000269729	AC006262.4	grey	0.22465481
ENSG00000268460	AC006262.6	grey	-0.1267811
ENSG00000269483	AC006272.1	grey	0.25659897
ENSG00000229110	AC006355.3	grey	0.13179885
ENSG00000223070	AC006377.1	grey	0.26575285
ENSG00000236861	AC006378.2	grey	0.4073998
ENSG00000248851	AC006427.2	grey	0.12967155
ENSG00000205821	AC006435.1	grey	0.31243343
ENSG00000214998	AC006465.4	grey	0.28697616
ENSG00000261342	AC006538.1	grey	0.25952459
ENSG00000264441	AC006539.1	grey	0.45049577
ENSG00000252234	AC006557.1	grey	0.58713449
ENSG00000235328	AC006946.12	grey	0.3355966
ENSG00000235478	AC006946.15	brown	0.31241343
ENSG00000234457	AC006960.5	grey	0.52050375
ENSG00000235859	AC006978.6	darkgrey	0.58308078
ENSG00000231063	AC006994.1	grey	0.34346124
ENSG00000225726	AC007000.10	grey	0.33328606
ENSG00000237815	AC007000.11	grey	0.34562828
ENSG00000205482	AC007000.12	grey	0.29452936
ENSG00000265212	AC007009.2	grey	0.36625114
ENSG00000249930	AC007016.3	pink	0.44629115
ENSG00000263653	AC007025.1	grey	0.07994827
ENSG00000230751	AC007036.4	grey	0.26330461
ENSG00000227017	AC007036.6	grey	0.14021754
ENSG00000229127	AC007038.7	green	0.52369761
ENSG00000237689	AC007064.24	grey	0.2805295
ENSG00000234718	AC007161.5	grey	0.53805567
ENSG00000237166	AC007163.3	yellow	0.76763889
ENSG00000269145	AC007192.6	grey	0.03735627
ENSG00000267922	AC007193.6	grey	0.2572483
ENSG00000269696	AC007228.11	grey	0.14446505
ENSG00000268352	AC007228.5	grey	0.25609904
ENSG00000268036	AC007228.8	grey	0.46662706

ENSG00000226230	AC007237.2	turquoise	0.44596355
ENSG00000231043	AC007238.1	grey	0.04883216
ENSG00000231312	AC007246.3	blue	0.39239147
ENSG00000223813	AC007255.8	grey	0.27489015
ENSG00000213090	AC007256.5	black	0.52527033
ENSG00000235579	AC007283.4	grey	0.38538101
ENSG00000234431	AC007283.5	turquoise	0.34464329
ENSG00000227014	AC007285.6	grey	0.20770158
ENSG00000231519	AC007285.7	grey	0.23552819
ENSG00000267980	AC007292.6	grey	0.18751107
ENSG00000172974	AC007318.5	grey	0.41592583
ENSG00000271275	AC007326.9	grey	0.17687503
ENSG00000229679	AC007327.5	grey	0.34017528
ENSG00000235728	AC007349.5	grey	0.05849083
ENSG00000180672	AC007362.1	grey	0.07794164
ENSG00000268898	AC007377.1	grey	0.20742381
ENSG00000268688	AC007382.1	black	0.55055791
ENSG00000227946	AC007383.3	turquoise	0.52346799
ENSG00000231955	AC007383.4	darkmagenta	-0.6143878
ENSG00000232613	AC007386.4	grey	0.09722709
ENSG00000235725	AC007389.3	grey	0.28447404
ENSG00000217075	AC007401.2	grey	0.24751484
ENSG00000234350	AC007405.4	grey	0.21410434
ENSG00000239467	AC007405.6	darkred	0.48853248
ENSG00000268503	AC007421.1	grey	0.28776709
ENSG00000267893	AC007461.1	black	-0.473652
ENSG00000221156	AC007551.1	grey	0.37064581
ENSG00000228878	AC007551.3	salmon	-0.5092436
ENSG00000223874	AC007557.1	turquoise	-0.4071035
ENSG00000237479	AC007557.2	grey	0.2929347
ENSG00000227021	AC007557.3	grey	0.30492373
ENSG00000231597	AC007557.4	grey	0.19745358
ENSG00000235993	AC007559.1	grey	0.5219291
ENSG00000238165	AC007560.1	grey	0.05367755
ENSG00000215968	AC007560.2	grey	0.26952508
ENSG00000236295	AC007563.1	red	-0.5250619
ENSG00000229352	AC007563.3	grey	0.06668089
ENSG00000237930	AC007563.4	grey	0.40419605
ENSG00000236886	AC007563.5	grey	0.14524981
ENSG00000244055	AC007566.10	green	0.59070658
ENSG00000203648	AC007618.3	grey	0.44701412
ENSG00000242539	AC007620.3	grey	0.24349316
ENSG00000216011	AC007682.2	grey	0.36540068
ENSG00000203632	AC007690.1	grey	-0.0253647
ENSG00000237750	AC007740.1	grey	0.14964946
ENSG00000263960	AC007773.1	grey	0.43366964
ENSG00000267213	AC007773.2	grey	0.12350786
ENSG00000226205	AC007790.4	grey	0.4161234

ENSG00000231859	AC007875.2	grey	0.24973778
ENSG00000235982	AC007875.3	grey	-0.0033614
ENSG00000224891	AC007899.3	grey	0.22784093
ENSG00000233762	AC007969.5	grey	-0.050832
ENSG00000234281	AC007970.1	yellow	0.61897815
ENSG00000224553	AC008065.1	grey	0.35263412
ENSG00000237031	AC008067.2	grey	0.19877708
ENSG00000223754	AC008073.9	grey	0.41426983
ENSG00000223935	AC008074.3	grey	0.20851608
ENSG00000237217	AC008074.5	grey	0.2923602
ENSG00000203433	AC008147.2	grey	0.43140519
ENSG00000266512	AC008166.1	grey	0.39440327
ENSG00000229321	AC008269.2	grey	0.01885484
ENSG00000235937	AC008280.1	grey	0.22712601
ENSG00000268208	AC008372.1	brown	-0.4025665
ENSG00000222316	AC008581.1	grey	0.38707212
ENSG00000267838	AC008746.12	grey	0.20425199
ENSG00000235681	AC008746.5	grey	0.44815537
ENSG00000265379	AC008752.2	grey	0.22630563
ENSG00000266247	AC008752.3	grey	0.41066193
ENSG00000268280	AC008914.1	grey	0.30682367
ENSG00000237705	AC008937.2	grey	0.23638577
ENSG00000269746	AC009060.1	grey	0.32048207
ENSG00000196927	AC009093.1	grey	0.19309824
ENSG00000239763	AC009120.3	grey	0.23865558
ENSG00000260884	AC009120.5	grey	0.14304709
ENSG00000259972	AC009120.6	grey	0.00258642
ENSG00000224675	AC009227.2	grey	0.42014177
ENSG00000242628	AC009228.1	grey	0.15708495
ENSG00000230327	AC009234.1	grey	0.26196546
ENSG00000216191	AC009234.2	grey	0.29118134
ENSG00000236431	AC009237.11	grey	0.21051285
ENSG00000229689	AC009237.8	grey	-0.0158088
ENSG00000225813	AC009299.4	greenyellow	-0.4136791
ENSG00000233287	AC009362.2	grey	0.29772438
ENSG00000216895	AC009403.2	grey	0.13467248
ENSG00000236255	AC009404.2	grey	0.22378179
ENSG00000223911	AC009480.3	grey	0.17141881
ENSG00000233143	AC009492.1	grey	0.15328605
ENSG00000231609	AC009501.4	green	0.61998766
ENSG00000224152	AC009506.1	turquoise	0.41474782
ENSG00000227197	AC009518.8	grey	0.13605693
ENSG00000231794	AC009542.2	yellow	0.40225091
ENSG00000223960	AC009948.5	grey	-0.0335459
ENSG00000238082	AC009948.7	grey	0.22900536
ENSG00000176824	AC009951.1	yellow	0.71100402
ENSG00000213410	AC009951.2	grey	0.45385096
ENSG00000226266	AC009961.3	grey	0.2390077

ENSG00000233953	AC009970.1	grey	0.34294948
ENSG00000266566	AC009994.2	grey	0.46970234
ENSG00000228763	AC010095.5	grey	0.27657443
ENSG00000236107	AC010127.3	turquoise	-0.4807459
ENSG00000249249	AC010226.4	grey	0.17351986
ENSG00000235253	AC010240.2	grey	0.31199437
ENSG00000266156	AC010329.1	grey	0.29144662
ENSG00000249318	AC010468.2	grey	-0.0306022
ENSG00000269304	AC010522.1	grey	0.33715983
ENSG00000228577	AC010731.2	grey	0.30431095
ENSG00000235118	AC010731.4	turquoise	-0.3577019
ENSG00000232713	AC010733.5	grey	0.1601732
ENSG00000269291	AC010877.1	grey	0.16177684
ENSG00000234936	AC010883.5	grey	0.39257044
ENSG00000224509	AC010884.1	grey	0.1783177
ENSG00000226953	AC010890.1	yellow	0.43584501
ENSG00000225840	AC010970.2	grey	0.00317221
ENSG00000230065	AC010974.3	grey	0.19107503
ENSG00000229385	AC010975.1	grey	0.23208931
ENSG00000230237	AC010975.2	grey	0.30179804
ENSG00000231731	AC010976.2	grey	0.46196592
ENSG00000237732	AC010980.2	blue	0.46605794
ENSG00000222586	AC010999.1	grey	0.48758694
ENSG00000215781	AC011043.1	grey	0.04792687
ENSG00000235518	AC011196.3	grey	0.34611259
ENSG00000235586	AC011247.3	grey	0.43895636
ENSG00000236015	AC011290.5	grey	0.38404611
ENSG00000233487	AC011322.1	blue	0.45444461
ENSG00000265875	AC011357.1	grey	0.34253617
ENSG00000232129	AC011385.2	grey	0.422278
ENSG00000265104	AC011416.1	grey	0.37987014
ENSG00000265339	AC011477.1	grey	0.43339054
ENSG00000268038	AC011516.2	turquoise	0.54341988
ENSG00000223436	AC011625.1	grey	0.15336213
ENSG00000231638	AC011738.4	turquoise	-0.3569562
ENSG00000235092	AC011747.7	grey	-0.0641302
ENSG00000269686	AC011755.1	yellow	0.61529254
ENSG00000212884	AC011841.1	grey	0.05137373
ENSG00000215705	AC011841.2	grey	0.17824353
ENSG00000263455	AC011841.8	grey	0.35736983
ENSG00000229233	AC011891.5	grey	-0.124312
ENSG00000233038	AC011899.9	grey	0.18843014
ENSG00000237720	AC011995.1	grey	0.16285888
ENSG00000228391	AC011995.3	grey	0.17790015
ENSG00000266259	AC012003.1	grey	0.19584382
ENSG00000233416	AC012065.5	grey	0.02969412
ENSG00000260396	AC012065.7	grey	0.17626405
ENSG00000213197	AC012066.1	grey	0.11128886

ENSG00000179061	AC012074.1	grey	-0.0053815
ENSG00000235072	AC012074.2	brown	-0.3210263
ENSG00000234327	AC012146.7	grey	-0.4170958
ENSG00000226686	AC012309.5	grey	0.04327136
ENSG00000225156	AC012354.6	grey	-0.1614096
ENSG00000227799	AC012358.4	grey	0.09201067
ENSG00000240401	AC012358.8	grey	0.34501558
ENSG00000235319	AC012360.4	grey	0.41861026
ENSG00000232693	AC012370.2	grey	0.45179049
ENSG00000228251	AC012442.6	grey	0.28578009
ENSG00000203643	AC012456.3	turquoise	0.33136199
ENSG00000268396	AC012485.1	grey	0.5153385
ENSG00000232520	AC012507.3	magenta	0.65827352
ENSG00000236478	AC012513.4	grey	0.37462131
ENSG00000231189	AC013448.1	grey	0.38617866
ENSG00000205500	AC013472.3	grey	0.25303389
ENSG00000230286	AC013472.4	grey	0.34551247
ENSG00000267347	AC015849.13	grey	0.57003352
ENSG00000267326	AC015849.14	grey	0.16055245
ENSG00000267025	AC015849.19	turquoise	-0.3682972
ENSG00000237805	AC015849.2	grey	0.43896054
ENSG00000265403	AC015884.1	grey	0.46708184
ENSG00000226797	AC015923.1	grey	0.35307434
ENSG00000227279	AC015933.2	grey	0.20662921
ENSG00000228363	AC015971.2	yellow	0.51998767
ENSG00000224746	AC015987.1	grey	0.29821972
ENSG00000215431	AC015989.1	grey	0.29330077
ENSG00000235361	AC016292.1	grey	0.30214998
ENSG00000221542	AC016405.2	grey	0.32101226
ENSG00000225868	AC016582.2	grey	0.19664755
ENSG00000267858	AC016629.8	grey	0.02675458
ENSG00000221179	AC016670.1	grey	0.47212694
ENSG00000231327	AC016700.5	grey	0.08549528
ENSG00000223427	AC016716.1	grey	0.30405018
ENSG00000224731	AC016716.2	grey	0.15586719
ENSG00000228206	AC016717.1	grey	0.30179987
ENSG00000235335	AC016723.4	grey	0.34714652
ENSG00000234997	AC016745.3	grey	0.07812301
ENSG00000233559	AC016831.7	grey	0.3290807
ENSG00000234624	AC016894.1	grey	0.25965661
ENSG00000233729	AC016909.1	yellow	0.68595853
ENSG00000228721	AC016909.2	grey	0.57105799
ENSG00000232377	AC016910.1	brown	-0.6947009
ENSG00000224959	AC017002.2	grey	0.24995142
ENSG00000232696	AC017006.2	grey	0.29089707
ENSG00000225964	AC017076.5	grey	0.03618853
ENSG00000233829	AC017078.1	grey	0.31319231
ENSG00000269633	AC017081.1	grey	-0.0270084

ENSG00000216115	AC017083.1	grey	0.26384736
ENSG00000236259	AC017083.2	grey	0.22885543
ENSG00000227227	AC017101.10	blue	-0.3612379
ENSG00000229839	AC018462.2	grey	0.41076841
ENSG00000234520	AC018464.3	grey	0.1932567
ENSG00000268488	AC018512.1	grey	0.37306513
ENSG00000268301	AC018630.1	grey	0.30574905
ENSG00000225144	AC018643.4	grey	0.11728676
ENSG00000227544	AC018647.3	yellow	0.60603368
ENSG00000230964	AC018696.4	grey	0.37879374
ENSG00000235388	AC018696.7	grey	0.45798417
ENSG00000230104	AC018712.2	grey	0.19101967
ENSG00000170092	AC018720.10	grey	-0.0894483
ENSG00000239587	AC018730.4	grey	0.11823752
ENSG00000167765	AC018755.1	green	0.35837855
ENSG00000269388	AC018755.16	grey	0.07933735
ENSG00000235978	AC018816.3	black	0.49397809
ENSG00000228351	AC018832.1	yellow	0.56309743
ENSG00000213953	AC018867.1	grey	0.16717997
ENSG00000268846	AC018867.2	grey	0.13613168
ENSG00000232893	AC019068.2	turquoise	0.51213078
ENSG00000224007	AC019070.1	grey	0.20731946
ENSG00000231822	AC019097.7	grey	0.15693205
ENSG00000234423	AC019118.2	grey	-0.0220575
ENSG00000226649	AC019118.4	tan	0.4550224
ENSG00000234584	AC019186.1	grey	0.37525161
ENSG00000268328	AC019206.1	grey	-0.1264448
ENSG00000269367	AC020629.1	grey	0.3063708
ENSG00000263437	AC020892.1	grey	0.51527034
ENSG00000264129	AC020900.2	grey	0.53681893
ENSG00000256383	AC020910.2	turquoise	0.57870179
ENSG00000237281	AC021016.8	grey	0.41556769
ENSG00000196355	AC021860.1	grey	0.14657912
ENSG00000206567	AC022007.5	grey	0.24883962
ENSG00000249214	AC022558.1	grey	0.33322559
ENSG00000264771	AC022819.1	grey	0.39751873
ENSG00000268730	AC022819.2	grey	0.24666809
ENSG00000223628	AC023449.2	grey	0.30655458
ENSG00000225885	AC023590.1	red	-0.5676239
ENSG00000238508	AC024569.1	grey	0.50960117
ENSG00000244063	AC024704.2	grey	0.15789782
ENSG00000231443	AC024937.6	grey	0.2560021
ENSG00000215068	AC025171.1	grey	0.13594542
ENSG00000214325	AC025287.1	grey	0.15558186
ENSG00000179859	AC025335.1	lightcyan	0.61177882
ENSG00000268830	AC025811.2	grey	0.31356158
ENSG00000270947	AC025811.3	grey	0.16938096
ENSG00000225930	AC026150.5	black	0.61194155

ENSG00000260693	AC026150.8	grey	0.10712477
ENSG00000233912	AC026202.3	grey	0.32746056
ENSG00000268885	AC026740.1	grey	0.08230481
ENSG00000212104	AC026877.1	grey	0.17548036
ENSG00000230398	AC026882.1	grey	0.38789743
ENSG00000265047	AC026992.1	grey	0.5081429
ENSG00000143429	AC027612.6	grey	0.03437601
ENSG00000215067	AC027763.2	yellow	0.42983884
ENSG00000233006	AC034220.3	grey	0.25902671
ENSG00000223548	AC034228.3	grey	0.25901251
ENSG00000196193	AC034229.1	grey	0.26174837
ENSG00000233635	AC037445.1	grey	0.24338612
ENSG00000229334	AC046143.3	grey	0.03372343
ENSG00000268085	AC051642.1	grey	0.43442741
ENSG00000234136	AC055764.1	cyan	-0.4066636
ENSG00000197825	AC061975.1	grey	0.39145116
ENSG00000222020	AC062017.1	grey	-0.1439178
ENSG00000226939	AC062021.1	grey	0.05668487
ENSG00000234028	AC062029.1	grey	0.24258312
ENSG00000213055	AC064852.5	grey	0.04227051
ENSG00000222007	AC064874.1	yellow	0.5064975
ENSG00000238259	AC067940.1	grey	0.36111968
ENSG00000231858	AC067945.4	black	-0.5085602
ENSG00000232784	AC067961.1	grey	0.26586974
ENSG00000221027	AC068020.1	grey	0.53125235
ENSG00000268821	AC068039.1	grey	0.40103087
ENSG00000228389	AC068039.4	turquoise	-0.473104
ENSG00000228528	AC068057.1	grey	0.14574811
ENSG00000251485	AC068134.10	grey	0.37474705
ENSG00000225049	AC068279.2	grey	0.2636274
ENSG00000224881	AC068279.3	grey	0.02886331
ENSG00000236682	AC068282.3	grey	0.23387952
ENSG00000227157	AC068535.2	yellowgreen	0.64131707
ENSG00000238460	AC068587.2	grey	0.02216162
ENSG00000268171	AC068620.1	grey	0.11296243
ENSG00000266347	AC068641.1	grey	0.29717133
ENSG00000223908	AC068657.2	grey	0.4821434
ENSG00000260415	AC068987.1	blue	0.71993252
ENSG00000233581	AC069155.1	grey	0.07500385
ENSG00000203615	AC069200.1	grey	0.40199676
ENSG00000224769	AC069213.1	grey	0.02191068
ENSG00000227433	AC069213.4	grey	0.37522606
ENSG00000237418	AC069257.6	grey	0.36847547
ENSG00000228028	AC069257.8	grey	0.333513
ENSG00000189229	AC069277.2	grey	0.42950293
ENSG00000267242	AC069278.4	grey	0.12504722
ENSG00000225507	AC069282.6	grey	0.31128379
ENSG00000221487	AC069294.1	grey	0.41217416

ENSG00000229178	AC069513.4	grey	-0.1003
ENSG00000269165	AC069547.1	green	0.60140825
ENSG00000269700	AC069547.2	grey	0.27904936
ENSG00000231359	AC072052.7	grey	0.46623265
ENSG00000228446	AC073052.1	grey	0.41450608
ENSG00000235920	AC073109.2	grey	0.40627851
ENSG00000234722	AC073236.3	darkgrey	0.56020493
ENSG00000232140	AC073257.1	grey	0.35217482
ENSG00000267906	AC073333.1	grey	-0.0773427
ENSG00000235837	AC073333.8	grey	0.26960148
ENSG00000231840	AC073342.12	green	0.66934051
ENSG00000198580	AC073343.1	grey	0.01671199
ENSG00000228010	AC073343.13	grey	-0.0516818
ENSG00000236047	AC073410.1	grey	0.39445001
ENSG00000227470	AC073415.2	grey	0.49251772
ENSG00000204717	AC073464.11	greenyellow	-0.4475101
ENSG00000269531	AC073569.1	grey	0.47312469
ENSG00000250990	AC073635.5	grey	0.29448123
ENSG00000268632	AC073657.1	grey	0.42669782
ENSG00000235077	AC073842.19	grey	0.46844502
ENSG00000234072	AC074117.10	grey	0.26145588
ENSG00000225975	AC074138.3	grey	0.10509493
ENSG00000263570	AC074264.1	grey	0.42976703
ENSG00000213963	AC074286.1	grey	0.15249249
ENSG00000225889	AC074289.1	grey	-0.3442202
ENSG00000229054	AC074338.4	grey	0.36342346
ENSG00000228222	AC074363.1	grey	0.19314764
ENSG00000233716	AC074367.1	grey	0.28642032
ENSG00000266798	AC078816.1	grey	0.48017011
ENSG00000231114	AC078842.4	grey	0.17490548
ENSG00000238890	AC078917.1	grey	0.20287346
ENSG00000225365	AC078942.1	grey	0.39604397
ENSG00000234653	AC079117.1	grey	0.03152407
ENSG00000211984	AC079117.2	grey	0.13206034
ENSG00000233611	AC079135.1	grey	0.27815817
ENSG00000252616	AC079140.1	grey	0.46476334
ENSG00000269657	AC079210.1	grey	0.38103061
ENSG00000263738	AC079233.1	grey	-0.1360765
ENSG00000196096	AC079610.2	grey	0.26426824
ENSG00000225342	AC079630.4	grey	0.18706244
ENSG00000180019	AC079741.2	grey	0.34781104
ENSG00000214100	AC079776.2	yellow	0.52267399
ENSG00000228643	AC079779.4	grey	0.49620416
ENSG00000221048	AC079781.1	grey	0.21478643
ENSG00000232032	AC079781.7	grey	0.22282285
ENSG00000232097	AC079781.8	grey	0.26935879
ENSG00000233230	AC079807.2	brown	-0.4218559
ENSG00000236549	AC079807.3	grey	0.42751018

ENSG00000221432	AC079834.2	grey	0.47548596
ENSG00000231747	AC079922.2	grey	0.13935264
ENSG00000237753	AC079922.3	grey	-0.1288679
ENSG00000239776	AC079949.1	grey	0.0589573
ENSG00000203644	AC083799.1	grey	0.21394931
ENSG00000259820	AC083843.1	grey	0.29752932
ENSG00000232729	AC083884.8	grey	0.02477311
ENSG00000212694	AC084018.1	grey	-0.1289493
ENSG00000267163	AC084219.3	grey	0.3811328
ENSG00000186019	AC084219.4	grey	0.18586127
ENSG00000222311	AC084882.1	grey	0.27996303
ENSG00000232344	AC087163.2	grey	0.20278148
ENSG00000268076	AC087239.1	grey	0.53263284
ENSG00000235530	AC087294.2	grey	0.21175496
ENSG00000236819	AC087393.1	grey	-0.0478809
ENSG00000225751	AC087501.1	grey	0.18807165
ENSG00000263660	AC087793.1	grey	0.3425688
ENSG00000224239	AC090044.2	grey	0.27103065
ENSG00000252846	AC090206.1	grey	0.34749458
ENSG00000235672	AC090286.2	grey	0.1692728
ENSG00000203573	AC090519.4	grey	0.45497711
ENSG00000256905	AC090571.1	blue	-0.428586
ENSG00000250404	AC090587.2	grey	0.20887343
ENSG00000242088	AC090602.2	grey	0.52223895
ENSG00000236838	AC090617.1	greenyellow	-0.431956
ENSG00000215380	AC090625.1	turquoise	0.44084223
ENSG00000221291	AC090660.1	grey	0.25244855
ENSG00000266526	AC090720.1	grey	0.11635285
ENSG00000230593	AC090804.1	grey	0.37954863
ENSG00000231780	AC090960.1	grey	0.09011324
ENSG00000236234	AC091132.1	grey	0.35780191
ENSG00000214433	AC091167.3	grey	0.2791058
ENSG00000229043	AC091729.9	grey	-0.0491372
ENSG00000268126	AC091948.1	grey	0.45927863
ENSG00000227785	AC092013.1	grey	0.43849288
ENSG00000221506	AC092038.1	grey	0.32694775
ENSG00000266026	AC092111.1	grey	0.13233821
ENSG00000232034	AC092168.2	grey	0.36307646
ENSG00000230733	AC092171.4	turquoise	-0.482047
ENSG00000267290	AC092192.1	red	-0.5385909
ENSG00000228629	AC092295.4	grey	0.23649598
ENSG00000233527	AC092295.7	grey	0.13265748
ENSG00000269148	AC092301.3	grey	0.11154692
ENSG00000265341	AC092332.1	grey	0.56605727
ENSG00000230872	AC092338.5	grey	0.18841188
ENSG00000263984	AC092380.1	grey	0.39936594
ENSG00000229948	AC092431.2	grey	0.42555234
ENSG00000231024	AC092431.3	grey	0.33766983

ENSG00000227189	AC092535.3	grey	0.21054551
ENSG00000211482	AC092574.1	grey	0.48044447
ENSG00000252150	AC092574.2	grey	0.32016502
ENSG00000236204	AC092594.1	grey	0.47082305
ENSG00000248254	AC092597.3	grey	0.18642686
ENSG00000227176	AC092641.2	grey	0.33744181
ENSG00000213126	AC092642.1	black	0.67921895
ENSG00000236922	AC092661.1	grey	0.08700037
ENSG00000232056	AC092687.4	grey	0.2261658
ENSG00000269795	AC092782.1	grey	0.4072802
ENSG00000265819	AC092832.1	grey	0.47808886
ENSG00000233757	AC092835.2	grey	0.12239084
ENSG00000228108	AC092839.3	grey	0.15494339
ENSG00000234943	AC092839.4	grey	0.30289399
ENSG00000222262	AC092846.1	grey	0.34141029
ENSG00000265001	AC092846.2	grey	0.36735258
ENSG00000221104	AC092865.1	grey	0.19846336
ENSG00000221681	AC092902.1	grey	0.50529046
ENSG00000213358	AC092933.4	grey	0.19361011
ENSG00000267979	AC092964.2	grey	0.33137856
ENSG00000238018	AC093110.3	blue	-0.4330963
ENSG00000228541	AC093159.1	grey	0.18702618
ENSG00000233654	AC093388.3	grey	0.19503365
ENSG00000232057	AC093390.1	grey	0.19410275
ENSG00000231890	AC093391.2	grey	0.22100982
ENSG00000235257	AC093415.2	blue	0.42965628
ENSG00000238217	AC093590.1	yellow	0.5616681
ENSG00000234231	AC093616.4	turquoise	-0.4528108
ENSG00000237940	AC093642.3	blue	0.36012778
ENSG00000220804	AC093642.5	grey	0.08240101
ENSG00000256604	AC093668.2	grey	-0.0461722
ENSG00000232533	AC093673.5	grey	0.09076529
ENSG00000223522	AC093690.1	grey	0.12500062
ENSG00000213222	AC093724.2	grey	-0.0282844
ENSG00000226886	AC093787.1	grey	0.36266515
ENSG00000225205	AC093818.1	blue	-0.532992
ENSG00000152117	AC093838.4	grey	-0.1602837
ENSG00000224819	AC093843.1	red	0.47891466
ENSG00000221388	AC093865.1	grey	0.33771351
ENSG00000236732	AC094019.4	grey	0.45449675
ENSG00000269690	AC096677.1	grey	0.25904766
ENSG00000263566	AC096753.1	grey	0.34219578
ENSG00000244567	AC096772.6	purple	0.49739567
ENSG00000231449	AC097359.2	grey	0.2752121
ENSG00000238470	AC097361.1	grey	0.51923218
ENSG00000265550	AC097382.1	grey	0.34479576
ENSG00000234193	AC097461.4	grey	0.28966994
ENSG00000235024	AC097468.7	grey	0.00327327

ENSG00000237877	AC097500.2	blue	-0.4389686
ENSG00000236432	AC097662.2	grey	0.07175439
ENSG00000213846	AC098614.2	grey	0.05140501
ENSG00000233766	AC098617.1	grey	0.1169196
ENSG00000216169	AC098650.1	grey	0.49276092
ENSG00000227745	AC098826.5	blue	0.50939698
ENSG00000221573	AC099535.1	grey	0.33914164
ENSG00000228521	AC099552.3	grey	0.42492454
ENSG00000225386	AC099754.1	turquoise	0.42331924
ENSG00000238952	AC099757.1	grey	0.48359813
ENSG00000224738	AC099850.1	grey	0.35023422
ENSG00000264937	AC100830.5	grey	0.47531139
ENSG00000233850	AC103563.8	yellow	0.49744622
ENSG00000221185	AC103816.1	grey	0.51265861
ENSG00000254879	AC103828.1	grey	0.29200895
ENSG00000226410	AC104058.1	grey	0.34114174
ENSG00000233847	AC104113.3	grey	0.26915704
ENSG00000233133	AC104451.2	grey	0.25598049
ENSG00000268756	AC104534.2	grey	0.26694649
ENSG00000203435	AC104600.1	grey	0.16517004
ENSG00000236307	AC104651.1	black	0.48653991
ENSG00000225496	AC104651.2	grey	0.35604956
ENSG00000228857	AC104653.1	grey	0.02263807
ENSG00000231851	AC104655.2	grey	0.48995945
ENSG00000226508	AC104655.3	grey	0.13217251
ENSG00000224220	AC104699.1	grey	0.18681392
ENSG00000231334	AC104781.1	grey	0.39766557
ENSG00000266621	AC104841.1	grey	0.20942398
ENSG00000238149	AC104978.1	grey	0.31465729
ENSG00000263842	AC104986.1	grey	0.45690643
ENSG00000232504	AC105053.4	yellow	0.69433042
ENSG00000216089	AC105316.1	grey	0.25972369
ENSG00000231079	AC105402.4	turquoise	-0.4823628
ENSG00000227252	AC105760.2	grey	0.21552245
ENSG00000197644	AC106870.2	grey	0.44807001
ENSG00000228368	AC106873.4	grey	0.46260426
ENSG00000177822	AC108142.1	blue	0.41407993
ENSG00000227992	AC108463.2	grey	0.23899445
ENSG00000234295	AC109135.1	grey	0.0253537
ENSG00000216125	AC109496.1	grey	0.40078454
ENSG00000234945	AC109828.1	grey	0.28200538
ENSG00000233438	AC109829.1	grey	0.3703622
ENSG00000268656	AC110084.1	grey	0.37744575
ENSG00000230044	AC110754.3	grey	0.26776167
ENSG00000268800	AC112205.1	grey	0.40257082
ENSG00000233223	AC113189.5	grey	0.19804891
ENSG00000266671	AC113342.1	grey	0.29953634
ENSG00000234165	AC114877.3	turquoise	0.46782405

ENSG00000268601	AC115522.3	grey	0.26876594
ENSG00000204620	AC115618.1	grey	0.29287392
ENSG00000233991	AC116050.1	grey	0.01227602
ENSG00000238160	AC116366.5	grey	0.37191713
ENSG00000234290	AC116366.6	grey	0.43700376
ENSG00000238512	AC117401.1	grey	0.3595268
ENSG00000236440	AC117947.1	grey	0.26909742
ENSG00000211553	AC118278.1	grey	0.3716021
ENSG00000221051	AC118344.1	grey	0.35447899
ENSG00000228171	AC121251.1	grey	0.29566413
ENSG00000223082	AC121336.1	grey	0.4985345
ENSG00000197815	AC122129.1	grey	0.29452233
ENSG00000262728	AC123768.4	grey	0.19133106
ENSG00000231536	AC123886.2	grey	-0.0957181
ENSG00000231395	AC124914.3	grey	0.25353909
ENSG00000226155	AC124944.3	grey	0.26035541
ENSG00000231259	AC125232.1	turquoise	-0.4137372
ENSG00000268367	AC126614.1	grey	0.10655376
ENSG00000230732	AC127904.2	grey	0.15203809
ENSG00000250443	AC128709.1	grey	0.01483541
ENSG00000235126	AC128709.3	grey	0.07416683
ENSG00000236457	AC130689.5	grey	0.47930439
ENSG00000253864	AC131025.8	grey	0.11755747
ENSG00000216921	AC131097.4	turquoise	0.44132721
ENSG00000183562	AC131971.1	yellow	0.37197718
ENSG00000213519	AC132008.1	grey	0.46034019
ENSG00000221799	AC132068.1	grey	0.29517004
ENSG00000268403	AC132192.1	grey	0.49804469
ENSG00000268852	AC132872.2	grey	0.22526938
ENSG00000228989	AC133528.2	turquoise	0.4342082
ENSG00000237838	AC133680.1	magenta	0.53588215
ENSG00000198150	AC135178.1	grey	-0.0491542
ENSG00000256579	AC135776.1	grey	0.34077693
ENSG00000228740	AC136896.1	cyan	-0.3912631
ENSG00000260279	AC137932.5	grey	0.06109656
ENSG00000261253	AC137932.6	grey	0.13057054
ENSG00000260923	AC137934.1	yellow	0.51173765
ENSG00000238035	AC138035.2	grey	0.22718741
ENSG00000269358	AC138393.1	grey	0.34682947
ENSG00000235760	AC138655.4	grey	0.23785981
ENSG00000236558	AC138655.6	grey	0.31943751
ENSG00000203563	AC138951.1	grey	0.2623682
ENSG00000267251	AC139100.3	grey	0.0514319
ENSG00000233812	AC139426.2	red	-0.4859375
ENSG00000263853	AC139530.1	grey	0.09114705
ENSG00000233799	AC139887.4	grey	0.18979708
ENSG00000269529	AC140061.12	grey	0.29702878
ENSG00000227309	AC140076.1	grey	0.23661788

ENSG00000261227	AC140912.1	grey	0.0308428
ENSG00000215154	AC141586.5	grey	-0.1151106
ENSG00000250986	AC141928.1	magenta	0.70296296
ENSG00000265613	AC143336.1	grey	0.48526311
ENSG00000231969	AC144449.1	grey	0.35440614
ENSG00000221246	AC144522.1	grey	0.46395356
ENSG00000234742	AC144530.1	grey	0.21222077
ENSG00000264965	AC144573.1	grey	0.20152643
ENSG00000261426	AC144833.1	grey	0.04026261
ENSG00000215750	AC145212.1	grey	0.09217554
ENSG00000221485	AC145212.2	grey	0.1864123
ENSG00000223061	AC145212.3	grey	0.22953869
ENSG00000230606	AC159540.1	blue	0.61759977
ENSG00000235833	AC159540.14	grey	0.06103718
ENSG00000269396	AC187652.1	green	0.36826053
ENSG00000238424	AC190387.1	grey	0.38457197
ENSG00000255748	AC226150.4	grey	0.25905313
ENSG00000251775	ACA59	grey	0.4356828
ENSG00000238685	ACA64	grey	0.34821877
ENSG00000239005	ACA64	grey	0.35948126
ENSG00000060971	ACAA1	red	0.58176462
ENSG00000167315	ACAA2	turquoise	0.84090654
ENSG00000132142	ACACA	green	-0.6410608
ENSG00000076555	ACACB	turquoise	0.46363615
ENSG00000111271	ACAD10	turquoise	-0.4382146
ENSG00000151498	ACAD8	darkred	0.53095179
ENSG00000177646	ACAD9	grey	0.14165045
ENSG00000117054	ACADM	brown	-0.4747934
ENSG00000122971	ACADS	turquoise	0.40742037
ENSG00000196177	ACADSB	grey	0.10356667
ENSG00000072778	ACADVL	turquoise	0.46716418
ENSG00000072818	ACAP1	grey	-0.2542422
ENSG00000114331	ACAP2	turquoise	0.48339731
ENSG00000229325	ACAP2-IT1	grey	-0.0615045
ENSG00000131584	ACAP3	turquoise	-0.6146882
ENSG00000120437	ACAT2	blue	0.59863086
ENSG00000182827	ACBD3	brown	-0.6539836
ENSG00000181513	ACBD4	grey	0.17437371
ENSG00000107897	ACBD5	brown	-0.9006124
ENSG00000135847	ACBD6	turquoise	-0.7428107
ENSG00000176244	ACBD7	turquoise	0.69205353
ENSG00000102977	ACD	brown	0.33269231
ENSG00000159640	ACE	red	-0.583009
ENSG00000177076	ACER2	tan	0.68686103
ENSG00000078124	ACER3	grey	0.14949168
ENSG00000087085	ACHE	blue	0.501955
ENSG00000100813	ACIN1	grey	-0.2037929
ENSG00000144476	ACKR3	turquoise	0.63727948

ENSG00000129048	ACKR4	grey	0.23927461
ENSG00000131473	ACLY	greenyellow	-0.3941014
ENSG00000196636	ACN9	yellow	-0.3979964
ENSG00000122729	ACO1	turquoise	0.56752808
ENSG00000100412	ACO2	grey	0.29304945
ENSG00000184227	ACOT1	grey	-0.0187928
ENSG00000162390	ACOT11	turquoise	0.49374809
ENSG00000112304	ACOT13	turquoise	0.52667123
ENSG00000119673	ACOT2	grey	-0.0686145
ENSG00000177465	ACOT4	grey	0.13827166
ENSG00000205669	ACOT6	grey	0.28460924
ENSG00000097021	ACOT7	blue	0.85932208
ENSG00000101473	ACOT8	yellow	-0.4477553
ENSG00000123130	ACOT9	red	0.56655917
ENSG00000161533	ACOX1	turquoise	0.71490469
ENSG00000168306	ACOX2	red	0.5648336
ENSG00000087008	ACOX3	grey	0.06572464
ENSG00000143727	ACP1	yellow	-0.6074332
ENSG00000134575	ACP2	blue	0.38460278
ENSG00000162836	ACP6	grey	-0.1134678
ENSG00000155893	ACPL2	purple	0.52822596
ENSG00000134940	ACRV1	grey	0.11271389
ENSG00000103740	ACSBG1	turquoise	0.81111771
ENSG00000167107	ACSF2	grey	-0.0273721
ENSG00000151726	ACSL1	darkred	-0.6086359
ENSG00000068366	ACSL4	turquoise	-0.5506924
ENSG00000197142	ACSL5	pink	0.81661366
ENSG00000164398	ACSL6	darkred	0.70854442
ENSG00000183549	ACSM5	grey	-0.18279
ENSG00000154930	ACSS1	turquoise	0.51999795
ENSG00000111058	ACSS3	turquoise	0.70135384
ENSG00000107796	ACTA2	grey	-0.0327261
ENSG00000180139	ACTA2-AS1	grey	-0.2194502
ENSG00000075624	ACTB	grey	-0.2201947
ENSG00000185607	ACTBP7	yellow	0.61246969
ENSG00000159251	ACTC1	purple	0.53173489
ENSG00000178631	ACTG1P1	grey	0.13343332
ENSG00000231340	ACTG1P10	grey	0.16737283
ENSG00000226642	ACTG1P12	grey	0.27628105
ENSG00000215388	ACTG1P3	grey	0.0802172
ENSG00000136518	ACTL6A	turquoise	0.48971877
ENSG00000077080	ACTL6B	blue	0.72427458
ENSG00000077522	ACTN2	darkmagenta	0.88156128
ENSG00000130402	ACTN4	red	-0.7804419
ENSG00000138107	ACTR1A	blue	0.49787919
ENSG00000115073	ACTR1B	blue	0.36283174
ENSG00000138071	ACTR2	grey	-0.3333568
ENSG00000115091	ACTR3	blue	-0.3218525

ENSG00000133627	ACTR3B	turquoise	-0.6228451
ENSG00000106526	ACTR3C	grey	0.24982595
ENSG00000101442	ACTR5	lightcyan	-0.4927193
ENSG00000075089	ACTR6	grey	0.05497238
ENSG00000113812	ACTR8	grey	0.22940254
ENSG00000115170	ACVR1	tan	0.6811847
ENSG00000135503	ACVR1B	grey	0.29837064
ENSG00000123612	ACVR1C	turquoise	-0.4507325
ENSG00000121989	ACVR2A	black	-0.5623514
ENSG00000114739	ACVR2B	brown	-0.4201887
ENSG00000139567	ACVRL1	tan	0.86666046
ENSG00000132744	ACY3	pink	-0.544441
ENSG00000119640	ACYP1	grey	-0.2109157
ENSG00000170634	ACYP2	grey	-0.1863431
ENSG00000236144	AD000090.2	grey	-0.3563863
ENSG00000240616	AD000092.3	grey	0.31497501
ENSG00000268947	AD000684.2	grey	0.12021086
ENSG00000196839	ADA	yellow	0.62915831
ENSG00000168803	ADAL	brown	0.44087515
ENSG00000137845	ADAM10	yellow	0.60593173
ENSG00000148848	ADAM12	grey	0.09364336
ENSG00000143537	ADAM15	grey	0.20030343
ENSG00000151694	ADAM17	turquoise	0.45901809
ENSG00000135074	ADAM19	grey	0.42660646
ENSG00000229186	ADAM1A	grey	0.01442402
ENSG00000226469	ADAM1B	grey	0.36321076
ENSG00000134007	ADAM20	grey	0.15582956
ENSG00000259158	ADAM20P1	turquoise	-0.4373136
ENSG00000139985	ADAM21	grey	0.19240095
ENSG00000008277	ADAM22	grey	-0.0755391
ENSG00000114948	ADAM23	blue	0.84217115
ENSG00000042980	ADAM28	cyan	0.85103388
ENSG00000197140	ADAM32	grey	0.3151727
ENSG00000149451	ADAM33	grey	0.04690703
ENSG00000168615	ADAM9	blue	-0.5422631
ENSG00000142303	ADAMTS10	grey	-0.3199769
ENSG00000160323	ADAMTS13	grey	0.07777262
ENSG00000140470	ADAMTS17	turquoise	0.45813308
ENSG00000145808	ADAMTS19	turquoise	-0.421364
ENSG00000249421	ADAMTS19-AS1	grey	0.13301118
ENSG00000156140	ADAMTS3	magenta	0.66750671
ENSG00000158859	ADAMTS4	yellow	0.73238621
ENSG00000134917	ADAMTS8	grey	-0.1789713
ENSG00000163638	ADAMTS9	pink	0.83031549
ENSG00000241684	ADAMTS9-AS2	magenta	-0.4153531
ENSG00000197859	ADAMTSL2	grey	-0.033224
ENSG00000156218	ADAMTSL3	grey	-0.0018706
ENSG00000105963	ADAP1	turquoise	-0.5455038

ENSG00000184060	ADAP2	cyan	0.69039666
ENSG00000160710	ADAR	brown	0.5343446
ENSG00000197381	ADARB1	brown	0.65509023
ENSG00000185736	ADARB2	grey	0.07578534
ENSG00000205696	ADARB2-AS1	grey	0.17356181
ENSG00000065457	ADAT1	grey	0.21471141
ENSG00000189007	ADAT2	turquoise	-0.4267277
ENSG00000142920	ADC	pink	-0.551871
ENSG00000063761	ADCK1	turquoise	-0.491308
ENSG00000133597	ADCK2	turquoise	-0.6248794
ENSG00000163050	ADCK3	brown	0.68241665
ENSG00000123815	ADCK4	grey	0.05156606
ENSG00000173137	ADCK5	grey	-0.0471209
ENSG00000164742	ADCY1	black	0.69330895
ENSG00000161912	ADCY10P1	darkred	-0.5778191
ENSG00000078295	ADCY2	turquoise	0.58978973
ENSG00000138031	ADCY3	grey	0.16358618
ENSG00000129467	ADCY4	tan	0.73669459
ENSG00000173175	ADCY5	magenta	0.85567304
ENSG00000174233	ADCY6	grey	0.05514205
ENSG00000162104	ADCY9	grey60	0.62653166
ENSG00000087274	ADD1	salmon	0.56914764
ENSG00000075340	ADD2	blue	0.82124659
ENSG00000148700	ADD3	turquoise	0.79724472
ENSG00000197894	ADH5	turquoise	0.62836354
ENSG00000223694	ADH5P3	grey	0.51963452
ENSG00000147576	ADHFE1	grey	0.04710143
ENSG00000182551	ADI1	turquoise	0.74464271
ENSG00000159346	ADIPOR1	brown	-0.6286498
ENSG00000006831	ADIPOR2	blue	-0.6801229
ENSG00000156110	ADK	darkred	0.56549441
ENSG00000148926	ADM	pink	0.49532388
ENSG00000101126	ADNP	grey	0.16052348
ENSG00000101544	ADNP2	lightgreen	0.5527832
ENSG00000163485	ADORA1	grey	0.19188789
ENSG00000170425	ADORA2B	turquoise	0.50384967
ENSG00000121933	ADORA3	cyan	0.77184122
ENSG00000159322	ADPGK	yellow	-0.3066775
ENSG00000144843	ADPRH	tan	0.52084577
ENSG00000153531	ADPRHL1	grey	0.41665902
ENSG00000116863	ADPRHL2	salmon	0.58423635
ENSG00000170222	ADPRM	grey	-0.0662785
ENSG00000120907	ADRA1A	brown	-0.3186123
ENSG00000184160	ADRA2C	magenta	0.77012028
ENSG00000043591	ADRB1	grey	-0.0451283
ENSG00000169252	ADRB2	brown	-0.3277619
ENSG00000173020	ADRBK1	purple	0.68658871
ENSG00000100077	ADRBK2	yellow	-0.6535636

ENSG00000130706	ADRM1	grey	0.07517253
ENSG00000239900	ADSL	grey	-0.0398797
ENSG00000035687	ADSS	yellow	0.43228272
ENSG00000185100	ADSSL1	grey	0.25893928
ENSG00000106624	AEBP1	grey	-0.1412238
ENSG00000139154	AEBP2	turquoise	-0.5070417
ENSG00000181026	AEN	grey	0.05741197
ENSG00000104964	AES	blue	0.34858641
ENSG00000263335	AF001548.5	yellow	0.40779738
ENSG00000267388	AF038458.5	grey	0.31697632
ENSG00000237721	AF064858.11	grey	0.1104245
ENSG00000225330	AF064860.5	grey	0.28674555
ENSG00000260721	AF067845.1	grey	-0.1128023
ENSG00000231965	AF131215.1	grey	0.242375
ENSG00000255310	AF131215.2	grey	0.38393465
ENSG00000254936	AF131215.3	grey	0.21841218
ENSG00000254556	AF131215.4	grey	0.16833973
ENSG00000215346	AF131215.5	grey	0.10038134
ENSG00000254839	AF131215.6	grey	0.43081699
ENSG00000269918	AF131215.9	grey	0.20862833
ENSG00000255020	AF131216.5	grey	-0.014885
ENSG00000246477	AF131216.6	grey	0.2088083
ENSG00000245685	AF146191.4	grey	0.06155886
ENSG00000227170	AF178030.2	grey	0.14250455
ENSG00000255085	AF186192.5	brown	-0.628755
ENSG00000238553	AF213884.3	grey	0.49294339
ENSG00000196526	AFAP1	grey	-0.2195348
ENSG00000169129	AFAP1L2	grey	-0.0428213
ENSG00000172493	AFF1	blue	-0.7695174
ENSG00000144218	AFF3	black	0.6941599
ENSG00000072364	AFF4	brown	0.25559321
ENSG00000223959	AFG3L1P	grey	0.02313947
ENSG00000141385	AFG3L2	brown	0.63859716
ENSG00000183077	AFMID	yellow	0.6335031
ENSG00000119844	AFTPH	grey	0.1202897
ENSG00000038002	AGA	grey	-0.2248605
ENSG00000157985	AGAP1	darkred	-0.6248536
ENSG00000235529	AGAP1-IT1	grey	0.19982355
ENSG00000204172	AGAP10	grey	-0.1171889
ENSG00000135439	AGAP2	darkorange	0.71470431
ENSG00000255737	AGAP2-AS1	grey	0.2596033
ENSG00000133612	AGAP3	blue	0.69424346
ENSG00000204149	AGAP6	turquoise	-0.436294
ENSG00000146856	AGBL3	grey	0.28406963
ENSG00000186094	AGBL4	blue	0.75947847
ENSG00000225623	AGBL4-IT1	grey	0.28908233
ENSG00000084693	AGBL5	grey	0.0174527
ENSG00000204305	AGER	grey	-0.0996876

ENSG00000173744	AGFG1	grey	0.18968193
ENSG00000106351	AGFG2	grey	0.00082334
ENSG00000164252	AGGF1	grey	0.0334531
ENSG00000006530	AGK	turquoise	-0.4806424
ENSG00000226555	AGKP1	grey	-0.1163609
ENSG00000162688	AGL	turquoise	0.50305647
ENSG00000092847	AGO1	brown	-0.7169225
ENSG00000123908	AGO2	grey	0.16481817
ENSG00000126070	AGO3	brown	-0.5625407
ENSG00000134698	AGO4	grey	-0.0581406
ENSG00000204310	AGPAT1	grey	0.34052241
ENSG00000169692	AGPAT2	magenta	-0.3947109
ENSG00000160216	AGPAT3	blue	-0.4777307
ENSG00000026652	AGPAT4	darkmagenta	0.70827823
ENSG00000155189	AGPAT5	turquoise	0.79549435
ENSG00000158669	AGPAT6	turquoise	-0.4216454
ENSG00000018510	AGPS	turquoise	0.64881779
ENSG00000188157	AGRN	grey	-0.0985952
ENSG00000135744	AGT	turquoise	0.68271889
ENSG00000135049	AGTPBP1	lightgreen	0.78762512
ENSG00000177674	AGTRAP	pink	0.4718479
ENSG00000153207	AHCTF1	blue	-0.3756503
ENSG00000168710	AHCYL1	turquoise	0.70009353
ENSG00000158467	AHCYL2	turquoise	0.75992647
ENSG00000126705	AHDC1	grey	0.14795449
ENSG00000135541	AHI1	grey	-0.0096538
ENSG00000124942	AHNAK	turquoise	0.6302791
ENSG00000185567	AHNAK2	yellow	-0.6310715
ENSG00000106546	AHR	floralwhite	0.53313062
ENSG00000204472	AIF1	cyan	0.90663966
ENSG00000126878	AIF1L	green	0.83759137
ENSG00000156709	AIFM1	grey	-0.0690957
ENSG00000042286	AIFM2	grey	0.17307566
ENSG00000183773	AIFM3	turquoise	0.61564366
ENSG00000146416	AIG1	turquoise	0.46146096
ENSG00000249089	AIG1P1	grey	0.23541576
ENSG00000112297	AIM1	grey	0.42388117
ENSG00000164022	AIMP1	lightgreen	0.68031752
ENSG00000234187	AIMP1P1	grey	-0.1235293
ENSG00000106305	AIMP2	grey	0.1483855
ENSG00000110711	AIP	grey	-0.195603
ENSG00000106992	AK1	turquoise	0.39799555
ENSG00000004455	AK2	red	-0.5994727
ENSG00000147853	AK3	turquoise	0.77886922
ENSG00000230042	AK3P3	grey	0.2586145
ENSG00000228816	AK3P5	grey	0.42507049
ENSG00000162433	AK4	turquoise	0.8550188
ENSG00000154027	AK5	purple	0.77277142

ENSG00000140057	AK7	brown	0.50308842
ENSG00000165695	AK8	grey	-0.1518856
ENSG00000155085	AK9	blue	0.45505765
ENSG00000121057	AKAP1	grey	-0.0312378
ENSG00000108599	AKAP10	grey	0.10237404
ENSG00000131016	AKAP12	darkmagenta	-0.5577043
ENSG00000170776	AKAP13	green	-0.6428947
ENSG00000186471	AKAP14	grey	-0.0235568
ENSG00000197976	AKAP17A	grey	-0.1394007
ENSG00000111254	AKAP3	grey	0.22240531
ENSG00000179841	AKAP5	turquoise	-0.4082133
ENSG00000151320	AKAP6	turquoise	-0.7579033
ENSG00000118507	AKAP7	lightcyan	0.70453974
ENSG00000105127	AKAP8	blue	-0.3142405
ENSG00000011243	AKAP8L	grey	0.04386263
ENSG00000127914	AKAP9	turquoise	-0.5280752
ENSG00000166452	AKIP1	grey	0.1224157
ENSG00000174574	AKIRIN1	blue	-0.6678525
ENSG00000135334	AKIRIN2	brown	0.59932399
ENSG00000106948	AKNA	grey	-0.3562589
ENSG00000117448	AKR1A1	green	-0.6614024
ENSG00000085662	AKR1B1	salmon	0.67135593
ENSG00000187134	AKR1C1	grey	0.15919565
ENSG00000196139	AKR1C3	turquoise	0.4423777
ENSG00000165568	AKR1E2	grey	0.13315258
ENSG00000053371	AKR7A2	turquoise	0.82670803
ENSG00000162482	AKR7A3	turquoise	0.37498201
ENSG00000211454	AKR7L	grey	0.02798537
ENSG00000142208	AKT1	grey	0.08888654
ENSG00000204673	AKT1S1	grey	0.15335209
ENSG00000105221	AKT2	yellow	0.48440398
ENSG00000228939	AKT3-IT1	grey	0.39223737
ENSG00000166971	AKTIP	turquoise	0.41869814
ENSG00000265978	AL008721.1	grey	0.18551102
ENSG00000213062	AL021068.1	grey	0.14829919
ENSG00000216901	AL022393.7	grey	0.28328173
ENSG00000203518	AL023807.1	grey	0.4408269
ENSG00000268402	AL031320.1	grey	0.3508746
ENSG00000221201	AL031655.1	grey	0.34800869
ENSG00000265642	AL034375.1	grey	0.32678553
ENSG00000268302	AL035252.1	grey	0.31297588
ENSG00000221132	AL035422.1	grey	0.49965081
ENSG00000266568	AL049610.1	grey	0.36736285
ENSG00000221748	AL049647.1	grey	0.4193261
ENSG00000251713	AL050321.1	grey	0.39164766
ENSG00000225683	AL078585.1	yellow	0.43266109
ENSG00000263645	AL079339.1	grey	0.45995496
ENSG00000221378	AL096864.1	grey	0.21732984

ENSG00000252748	AL096869.1	grey	0.38147305
ENSG00000244676	AL109761.5	yellow	0.53437281
ENSG00000235785	AL109767.1	magenta	0.49621088
ENSG00000239160	AL109947.1	grey	0.31672234
ENSG00000267918	AL117190.2	grey	-0.053022
ENSG00000259977	AL121578.2	grey	-0.0112375
ENSG00000224339	AL121578.5	grey	0.06170626
ENSG00000212111	AL121584.1	grey	0.30173469
ENSG00000236854	AL121656.5	yellow	0.55680681
ENSG00000263576	AL121748.1	grey	0.11495467
ENSG00000225746	AL132709.5	blue	0.55929212
ENSG00000232018	AL132709.8	turquoise	-0.3120847
ENSG00000221470	AL132875.1	grey	0.12538766
ENSG00000268466	AL132989.1	blue	-0.5141401
ENSG00000265856	AL133153.1	grey	0.33997673
ENSG00000197146	AL133458.1	cyan	0.50077479
ENSG00000212673	AL136115.1	grey	0.16937232
ENSG00000252949	AL136303.1	grey	0.48681296
ENSG00000266294	AL136380.1	grey	0.24244802
ENSG00000268130	AL137002.1	grey	0.49051794
ENSG00000203602	AL137059.1	grey	0.30460393
ENSG00000266098	AL137127.1	grey	0.42661927
ENSG00000221100	AL138706.1	grey	0.55747715
ENSG00000221473	AL138710.1	grey	0.38518079
ENSG00000215549	AL138815.1	grey	0.38765853
ENSG00000268344	AL138815.2	grey	0.29099528
ENSG00000268735	AL139080.1	grey	0.55446819
ENSG00000248458	AL139147.1	turquoise	0.3902056
ENSG00000214479	AL139333.1	grey	0.51031302
ENSG00000221645	AL157791.1	grey	0.37383642
ENSG00000265498	AL158051.1	grey	0.42905175
ENSG00000269305	AL158147.2	grey	0.45020895
ENSG00000251989	AL160011.1	grey	0.48962275
ENSG00000253017	AL160037.1	grey	0.49132495
ENSG00000268457	AL160175.1	grey	0.2061
ENSG00000268155	AL161450.1	grey	0.48535573
ENSG00000241781	AL161626.1	grey	-0.2142049
ENSG00000268925	AL161645.2	grey	0.53859135
ENSG00000264234	AL161751.1	grey	0.23724216
ENSG00000252731	AL161793.1	grey	0.24817488
ENSG00000214645	AL162389.1	blue	-0.513506
ENSG00000221465	AL162502.1	grey	0.30391074
ENSG00000212071	AL162511.1	grey	0.38434058
ENSG00000252324	AL163193.1	grey	0.31877395
ENSG00000213622	AL163952.1	grey	0.1062825
ENSG00000215902	AL353354.1	grey	0.27937029
ENSG00000269879	AL353583.1	grey	0.49853318
ENSG00000238638	AL353626.1	grey	0.17972002

ENSG00000264898	AL353626.2	grey	0.39159096
ENSG00000203512	AL353662.2	grey	0.33730251
ENSG00000203543	AL353671.2	grey	0.22555978
ENSG00000214006	AL353671.4	grey	0.3516849
ENSG00000238919	AL353763.1	grey	0.32705244
ENSG00000266021	AL353763.2	grey	0.06412041
ENSG00000227921	AL353791.1	purple	0.46181332
ENSG00000263475	AL353805.1	grey	0.45654851
ENSG00000203276	AL353898.2	grey	0.35702788
ENSG00000237611	AL353898.3	brown	-0.3632728
ENSG00000223291	AL354680.1	grey	0.18092052
ENSG00000269591	AL354808.2	grey	0.27923402
ENSG00000215615	AL354822.1	grey	0.01825194
ENSG00000264677	AL355490.2	grey	0.25792876
ENSG00000265082	AL356213.1	grey	0.34857655
ENSG00000266687	AL356261.1	grey	0.3593832
ENSG00000221159	AL356741.1	grey	0.50409319
ENSG00000251969	AL356776.1	grey	0.30133041
ENSG00000248835	AL357673.1	grey	0.03751637
ENSG00000238826	AL359253.1	grey	0.35099358
ENSG00000264650	AL359473.1	grey	0.45815865
ENSG00000216004	AL359832.1	grey	0.43009949
ENSG00000266732	AL359922.1	turquoise	0.46199698
ENSG00000215642	AL360154.1	grey	-0.0175792
ENSG00000203286	AL365202.1	grey	0.41055665
ENSG00000221519	AL390071.1	grey	0.32409601
ENSG00000252146	AL390776.1	grey	0.21384306
ENSG00000220981	AL391384.1	grey	0.41324752
ENSG00000252309	AL391416.1	grey	0.44762754
ENSG00000264567	AL391803.1	grey	0.40711051
ENSG00000234086	AL391994.1	grey	0.1534443
ENSG00000268059	AL441883.1	grey	-0.0251994
ENSG00000221319	AL441988.1	grey	0.28591573
ENSG00000222596	AL445523.1	grey	0.30998047
ENSG00000234614	AL450992.2	brown	-0.3921152
ENSG00000222121	AL512290.1	grey	0.31489579
ENSG00000263391	AL512428.1	grey	0.29084853
ENSG00000203438	AL512625.1	grey	-0.005567
ENSG00000203614	AL512652.1	grey	0.02324576
ENSG00000238758	AL512791.1	grey	0.33486238
ENSG00000221717	AL583860.1	grey	0.41573363
ENSG00000269753	AL589739.1	brown	-0.4562943
ENSG00000252817	AL590431.1	grey	0.12796809
ENSG00000268172	AL590452.1	grey	0.27123684
ENSG00000265803	AL590703.1	grey	0.53640407
ENSG00000213018	AL590762.11	grey	0.30804416
ENSG00000214239	AL591025.1	grey	0.03924217
ENSG00000269337	AL591479.1	grey	-0.0595674

ENSG00000251962	AL591668.1	grey	0.42929778
ENSG00000220023	AL592183.1	grey	0.07149465
ENSG00000239060	AL592183.5	grey	0.35931467
ENSG00000263936	AL592183.7	grey	0.56135426
ENSG00000231360	AL592284.1	grey	-0.1470702
ENSG00000227082	AL592494.5	grey	-0.1778397
ENSG00000221670	AL596092.1	grey	0.52841677
ENSG00000268662	AL596220.1	grey	0.44603676
ENSG00000268141	AL606500.1	grey	0.56193126
ENSG00000197502	AL627171.1	grey	0.55940529
ENSG00000269832	AL627171.2	grey	0.16456421
ENSG00000215014	AL645728.1	grey	0.27249054
ENSG00000221746	AL645730.1	grey	0.40567271
ENSG00000268365	AL646016.1	grey	0.39619942
ENSG00000222894	AL662800.1	grey	0.49986813
ENSG00000269831	AL669831.1	grey	0.36576187
ENSG00000203257	AL691479.1	grey	0.22124721
ENSG00000216109	AL713999.1	grey	0.31079763
ENSG00000265601	AL845321.1	grey	0.49911972
ENSG00000148218	ALAD	green	0.73742857
ENSG00000023330	ALAS1	lightcyan	-0.7060577
ENSG00000170017	ALCAM	darkmagenta	0.80611459
ENSG00000161618	ALDH16A1	grey	-0.1477477
ENSG00000059573	ALDH18A1	grey	0.25157533
ENSG00000165092	ALDH1A1	purple	-0.7425471
ENSG00000137124	ALDH1B1	turquoise	0.56842387
ENSG00000144908	ALDH1L1	turquoise	0.59391157
ENSG00000246022	ALDH1L1-AS2	turquoise	0.40358152
ENSG00000136010	ALDH1L2	black	-0.5454827
ENSG00000111275	ALDH2	turquoise	0.65582996
ENSG00000159423	ALDH4A1	turquoise	0.67286997
ENSG00000112294	ALDH5A1	yellowgreen	0.69568885
ENSG00000119711	ALDH6A1	turquoise	0.8832417
ENSG00000164904	ALDH7A1	turquoise	0.77853144
ENSG00000251400	ALDH7A1P1	black	0.74013221
ENSG00000118514	ALDH8A1	grey	0.17375144
ENSG00000143149	ALDH9A1	turquoise	0.75395954
ENSG00000149925	ALDOA	yellow	-0.6991073
ENSG00000136872	ALDOB	grey	0.3863183
ENSG00000109107	ALDOC	turquoise	0.70818614
ENSG00000033011	ALG1	grey	0.23259971
ENSG00000175548	ALG10B	turquoise	0.45869562
ENSG00000253710	ALG11	grey	0.49914648
ENSG00000101901	ALG13	grey	-0.1235209
ENSG00000229487	ALG13-AS1	grey	0.21481057
ENSG00000172339	ALG14	grey	0.10860772
ENSG00000254016	ALG1L10P	grey	0.06088599
ENSG00000249889	ALG1L11P	grey	0.11449445

ENSG00000251287	ALG1L2	brown	-0.3623669
ENSG00000238278	ALG1L6P	grey	-0.0229709
ENSG00000119523	ALG2	grey	0.03707913
ENSG00000214160	ALG3	grey	0.05238692
ENSG00000251568	ALG3P1	grey	0.1843065
ENSG00000088035	ALG6	grey	0.17211824
ENSG00000159063	ALG8	grey	-0.1485764
ENSG00000086848	ALG9	grey	0.08845427
ENSG00000254450	ALG9-IT1	grey	0.18477824
ENSG00000171094	ALK	grey	-0.2766548
ENSG00000100601	ALKBH1	grey	0.07375842
ENSG00000189046	ALKBH2	grey	0.17939835
ENSG00000166199	ALKBH3	turquoise	-0.459313
ENSG00000244926	ALKBH3-AS1	grey	0.24608989
ENSG00000160993	ALKBH4	grey	0.24508838
ENSG00000091542	ALKBH5	grey	-0.0316751
ENSG00000239382	ALKBH6	grey	-0.1446009
ENSG00000125652	ALKBH7	brown	0.61159698
ENSG00000137760	ALKBH8	grey	-0.0080588
ENSG00000116127	ALMS1	salmon	-0.5952725
ENSG00000230002	ALMS1-IT1	yellow	0.62389103
ENSG00000163016	ALMS1P	grey	0.01400253
ENSG00000108839	ALOX12	yellow	0.59681147
ENSG00000262943	ALOX12P2	grey	-0.1667013
ENSG00000012779	ALOX5	royalblue	0.77674275
ENSG00000132965	ALOX5AP	royalblue	0.87000304
ENSG00000073331	ALPK1	grey	0.13132585
ENSG00000136383	ALPK3	grey	0.38981009
ENSG00000162551	ALPL	tan	0.69681641
ENSG00000003393	ALS2	grey	0.22474627
ENSG00000155754	ALS2CR11	grey	0.03895764
ENSG00000155749	ALS2CR12	grey	0.06627617
ENSG00000183684	ALYREF	grey	-0.260335
ENSG00000178522	AMBN	black	0.77397202
ENSG00000110497	AMBRA1	turquoise	-0.6672589
ENSG00000123505	AMD1	lightgreen	0.66611598
ENSG00000184675	AMER1	brown	-0.3380541
ENSG00000234056	AMER2-AS1	grey	0.32162917
ENSG00000178171	AMER3	blue	0.48500082
ENSG00000159461	AMFR	grey	0.01149401
ENSG00000181754	AMIGO1	red	0.51759451
ENSG00000139211	AMIGO2	turquoise	0.4381332
ENSG00000101935	AMMECR1	grey	0.22706148
ENSG00000144233	AMMECR1L	turquoise	0.41015669
ENSG00000233273	AMMECR1LP1	grey	0.30999584
ENSG00000151743	AMN1	grey	0.10791043
ENSG00000126016	AMOT	turquoise	0.75876142
ENSG00000166025	AMOTL1	darkmagenta	-0.8748425

ENSG00000114019	AMOTL2	yellow	0.83232341
ENSG00000116337	AMPD2	blue	0.64198927
ENSG00000133805	AMPD3	yellow	0.70168168
ENSG00000078053	AMPH	blue	0.68217371
ENSG00000145020	AMT	turquoise	0.4213764
ENSG00000240038	AMY2B	turquoise	-0.5157214
ENSG00000174945	AMZ1	grey	-0.0202561
ENSG00000196704	AMZ2	grey	-0.1809342
ENSG00000214174	AMZ2P1	turquoise	-0.4576825
ENSG00000153107	ANAPC1	grey60	0.63306321
ENSG00000164162	ANAPC10	turquoise	0.51420964
ENSG00000141552	ANAPC11	greenyellow	0.65569288
ENSG00000129055	ANAPC13	grey	-0.2161201
ENSG00000110200	ANAPC15	grey	0.0299052
ENSG00000166295	ANAPC16	grey	-0.1091171
ENSG00000176248	ANAPC2	grey	0.04383618
ENSG00000053900	ANAPC4	grey	-0.2519094
ENSG00000089053	ANAPC5	lightcyan	-0.7404404
ENSG00000196510	ANAPC7	brown	0.81256975
ENSG00000013523	ANGEL1	yellow	0.34815013
ENSG00000174606	ANGEL2	blue	-0.4121745
ENSG00000091879	ANGPT2	tan	0.70927618
ENSG00000116194	ANGPTL1	grey	0.03848842
ENSG00000136859	ANGPTL2	yellow	0.62420143
ENSG00000167772	ANGPTL4	pink	0.7673264
ENSG00000171819	ANGPTL7	magenta	-0.3887346
ENSG00000029534	ANK1	grey	-0.1330355
ENSG00000145362	ANK2	purple	0.83503208
ENSG00000151150	ANK3	turquoise	-0.5736563
ENSG00000151687	ANKAR	brown	-0.6896522
ENSG00000166839	ANKDD1A	mediumpurp	0.72521086
ENSG00000189045	ANKDD1B	grey	-0.1257758
ENSG00000185722	ANKFY1	turquoise	0.71832349
ENSG00000154122	ANKH	blue	-0.3192318
ENSG00000131503	ANKHD1	grey	0.48012542
ENSG00000001629	ANKIB1	yellow	0.74059264
ENSG00000170209	ANKK1	black	0.55636614
ENSG00000176915	ANKLE2	green	-0.6876605
ENSG00000144504	ANKMY1	grey	-0.0019626
ENSG00000106524	ANKMY2	grey	-0.2295673
ENSG00000164331	ANKRA2	grey	-0.0917454
ENSG00000088448	ANKRD10	grey	-0.26914
ENSG00000167522	ANKRD11	grey	-0.3067726
ENSG00000101745	ANKRD12	lightgreen	0.6953983
ENSG00000076513	ANKRD13A	red	-0.8621793
ENSG00000198720	ANKRD13B	turquoise	-0.5080946
ENSG00000118454	ANKRD13C	grey	-0.095108
ENSG00000172932	ANKRD13D	grey	-0.1511581

ENSG00000134461	ANKRD16	blue	0.39664242
ENSG00000132466	ANKRD17	grey	0.07408342
ENSG00000180071	ANKRD18A	floralwhite	0.62124099
ENSG00000159712	ANKRD18CP	purple	0.45015201
ENSG00000226435	ANKRD18DP	brown	0.57257689
ENSG00000187984	ANKRD19P	darkorange	0.65641774
ENSG00000165887	ANKRD2	yellow	-0.3840349
ENSG00000196774	ANKRD20A1	grey	0.16009799
ENSG00000215559	ANKRD20A11P	grey	-0.062645
ENSG00000172014	ANKRD20A4	grey	-0.0195786
ENSG00000186481	ANKRD20A5P	grey	-0.1105044
ENSG00000236816	ANKRD20A7P	grey	1.44E-05
ENSG00000229089	ANKRD20A8P	grey	0.19807582
ENSG00000089847	ANKRD24	grey	0.09767906
ENSG00000107890	ANKRD26	lightgreen	0.45991502
ENSG00000237636	ANKRD26P3	grey	-0.0371161
ENSG00000229427	ANKRD26P4	grey	0.19279373
ENSG00000105186	ANKRD27	grey	0.18536576
ENSG00000206560	ANKRD28	red	-0.6691254
ENSG00000154065	ANKRD29	grey	-0.0141107
ENSG00000180777	ANKRD30B	purple	0.60852055
ENSG00000163046	ANKRD30BL	blue	0.62914422
ENSG00000164236	ANKRD33B	grey	0.38314201
ENSG00000181039	ANKRD34A	blue	0.7759144
ENSG00000189127	ANKRD34B	darkmagenta	0.72491568
ENSG00000198483	ANKRD35	red	0.51953311
ENSG00000196912	ANKRD36B	grey	-0.0030629
ENSG00000214262	ANKRD36BP1	grey60	-0.6471165
ENSG00000230006	ANKRD36BP2	turquoise	-0.4896894
ENSG00000213337	ANKRD39	grey	-0.3047181
ENSG00000154945	ANKRD40	green	0.93719376
ENSG00000137494	ANKRD42	blue	0.57559171
ENSG00000065413	ANKRD44	grey	-0.0606787
ENSG00000236977	ANKRD44-IT1	grey	0.00153347
ENSG00000183831	ANKRD45	grey	-0.2115611
ENSG00000186106	ANKRD46	red	0.6578423
ENSG00000168876	ANKRD49	green	0.55454865
ENSG00000151458	ANKRD50	grey	-0.1756228
ENSG00000100124	ANKRD54	salmon	-0.5106394
ENSG00000164512	ANKRD55	purple	0.72059731
ENSG00000135299	ANKRD6	floralwhite	-0.5002191
ENSG00000157999	ANKRD61	grey	0.11145394
ENSG00000230778	ANKRD63	magenta	0.40140365
ENSG00000235098	ANKRD65	grey	-0.0496732
ENSG00000106013	ANKRD7	grey	0.04680633
ENSG00000156381	ANKRD9	grey	0.25410406
ENSG00000064999	ANKS1A	grey	0.24651892
ENSG00000185046	ANKS1B	blue	0.63959282

ENSG00000168096	ANKS3	brown	0.49019537
ENSG00000165138	ANKS6	yellow	0.43126055
ENSG00000163516	ANKZF1	brown	0.49368046
ENSG00000011426	ANLN	darkred	-0.8216552
ENSG00000160746	ANO10	brown	0.37347363
ENSG00000047617	ANO2	grey	-0.1399358
ENSG00000134343	ANO3	magenta	0.93185691
ENSG00000151572	ANO4	yellow	0.61172664
ENSG00000171714	ANO5	yellow	-0.5174466
ENSG00000177119	ANO6	turquoise	0.68771481
ENSG00000237276	ANO7P1	grey	0.16050521
ENSG00000074855	ANO8	grey	0.07578057
ENSG00000140350	ANP32A	darkgrey	0.73063183
ENSG00000259516	ANP32AP1	grey	-0.0834118
ENSG00000136938	ANP32B	green	0.8124944
ENSG00000248546	ANP32C	grey	0.24654627
ENSG00000139223	ANP32D	grey	0.54796587
ENSG00000143401	ANP32E	brown	-0.7331673
ENSG00000169604	ANTXR1	turquoise	0.58894766
ENSG00000163297	ANTXR2	turquoise	0.60200854
ENSG00000122359	ANXA11	magenta	0.61354983
ENSG00000182718	ANXA2	magenta	-0.4426828
ENSG00000213406	ANXA2P1	yellow	0.55566169
ENSG00000138772	ANXA3	tan	0.71179848
ENSG00000196975	ANXA4	yellow	0.34541647
ENSG00000164111	ANXA5	turquoise	0.50401575
ENSG00000197043	ANXA6	grey60	0.80081639
ENSG00000138279	ANXA7	grey	0.14567547
ENSG00000143412	ANXA9	green	0.46732339
ENSG00000136250	AOAH	cyan	0.71282768
ENSG00000230539	AOAH-IT1	grey	0.19110044
ENSG00000131480	AOC2	grey	0.11849087
ENSG00000244301	AOX3P	yellow	0.74166536
ENSG00000234509	AP000253.1	grey	0.36810262
ENSG00000265590	AP000275.65	grey	0.28378941
ENSG00000231355	AP000302.58	grey	0.34291778
ENSG00000268265	AP000304.1	grey	0.49712717
ENSG00000243627	AP000322.53	cyan	-0.455941
ENSG00000236611	AP000343.2	grey	0.22830159
ENSG00000240906	AP000356.1	grey	0.24523424
ENSG00000257002	AP000438.2	grey	-0.0073566
ENSG00000255139	AP000442.1	grey	0.36817372
ENSG00000256315	AP000462.1	grey	0.09773837
ENSG00000255580	AP000462.2	turquoise	0.47232751
ENSG00000256972	AP000462.3	grey	0.38924625
ENSG00000228798	AP000473.5	grey	0.00909836
ENSG00000270093	AP000473.8	turquoise	0.5224852
ENSG00000237484	AP000476.1	grey	0.08124611

ENSG00000254495	AP000487.4	grey	0.3178072
ENSG00000246889	AP000487.5	magenta	-0.4649329
ENSG00000254604	AP000487.6	turquoise	0.40535773
ENSG00000237325	AP000563.2	grey	0.15653014
ENSG00000219529	AP000580.1	grey	0.05612647
ENSG00000213592	AP000662.9	grey	0.18465833
ENSG00000233393	AP000688.29	yellow	0.70628328
ENSG00000228107	AP000692.9	grey	0.41296329
ENSG00000173727	AP000769.1	grey	0.26086609
ENSG00000257067	AP000797.4	grey	-0.2049453
ENSG00000212093	AP000807.1	grey	0.49702437
ENSG00000222339	AP000807.2	grey	0.28024918
ENSG00000255396	AP000857.2	grey	0.16731761
ENSG00000254758	AP000889.1	blue	0.36771306
ENSG00000255483	AP000889.2	grey	0.08579987
ENSG00000221112	AP000908.1	grey	0.30713868
ENSG00000235286	AP000925.2	grey	0.20859843
ENSG00000234268	AP000936.1	grey	0.39722043
ENSG00000264344	AP000936.2	grey	0.54359596
ENSG00000269950	AP000962.2	turquoise	0.72268188
ENSG00000215504	AP000974.1	grey	-0.0255189
ENSG00000203306	AP001007.1	grey	-0.0404978
ENSG00000215458	AP001053.11	black	0.66921157
ENSG00000267913	AP001055.1	grey	0.27845834
ENSG00000184441	AP001062.7	grey	0.30131291
ENSG00000226771	AP001136.2	grey	0.01492819
ENSG00000221210	AP001137.1	grey	0.45010589
ENSG00000270071	AP001172.2	turquoise	0.47312495
ENSG00000270139	AP001172.3	grey	0.24560337
ENSG00000203400	AP001187.1	grey	0.27646956
ENSG00000264991	AP001205.1	grey	0.50217927
ENSG00000245571	AP001258.4	grey	-0.0220482
ENSG00000227726	AP001271.3	grey	0.32028779
ENSG00000224905	AP001347.6	grey	0.07769184
ENSG00000254837	AP001372.2	turquoise	0.58989819
ENSG00000267937	AP001421.1	grey	0.40733891
ENSG00000270116	AP001429.1	grey	0.16923008
ENSG00000242553	AP001432.14	grey	0.22255015
ENSG00000224541	AP001439.2	grey	0.22842101
ENSG00000233783	AP001442.2	grey	0.35452872
ENSG00000269038	AP001462.6	grey	0.33912221
ENSG00000235878	AP001468.1	grey	0.18559117
ENSG00000223901	AP001469.5	grey	0.38162488
ENSG00000239415	AP001469.9	grey	0.05663257
ENSG00000216113	AP001572.1	grey	0.4387088
ENSG00000197934	AP001597.1	grey	-0.0469374
ENSG00000227698	AP001619.2	grey	0.22804878
ENSG00000216197	AP001623.1	grey	0.08496585

ENSG00000239930	AP001625.4	grey	-0.1318821
ENSG00000235772	AP001625.6	grey	0.242845
ENSG00000225731	AP001627.1	grey	0.05028133
ENSG00000225218	AP001628.6	grey	0.46093579
ENSG00000254531	AP001816.1	yellow	-0.4242048
ENSG00000256885	AP001877.1	brown	-0.3706057
ENSG00000179038	AP001885.1	grey	0.36795668
ENSG00000234751	AP002381.2	grey	0.35806627
ENSG00000218536	AP002530.2	grey	0.25348867
ENSG00000237612	AP002856.7	black	0.58158532
ENSG00000255176	AP002954.3	yellow	0.73136347
ENSG00000237654	AP003025.2	black	0.57617233
ENSG00000224795	AP003039.3	yellow	0.73378956
ENSG00000215565	AP003041.1	grey	0.5004536
ENSG00000267981	AP003041.2	grey	0.49998821
ENSG00000254614	AP003068.23	grey	0.13174022
ENSG00000264838	AP003305.1	grey	0.45203494
ENSG00000265898	AP003354.1	grey	0.24677235
ENSG00000269089	AP003733.1	grey	0.33880587
ENSG00000237704	AP004289.2	grey	0.31707918
ENSG00000212067	AP005227.1	grey	0.31211322
ENSG00000215527	AP005482.1	grey	0.04807757
ENSG00000238504	AP005638.1	grey	0.38125576
ENSG00000215512	AP005901.1	grey	0.05974634
ENSG00000226645	AP006216.10	grey	0.0903177
ENSG00000231611	AP006216.11	grey	0.44416058
ENSG00000228463	AP006222.2	grey	0.26924491
ENSG00000177236	AP006621.1	grey	0.05716694
ENSG00000255284	AP006621.5	grey	-0.1181656
ENSG00000138660	AP1AR	greenyellow	0.49293493
ENSG00000100280	AP1B1	brown	0.57242075
ENSG00000166747	AP1G1	grey	0.07445869
ENSG00000213983	AP1G2	red	-0.5589623
ENSG00000072958	AP1M1	blue	0.58320502
ENSG00000106367	AP1S1	turquoise	-0.5133976
ENSG00000182287	AP1S2	magenta	0.47313904
ENSG00000152056	AP1S3	grey	0.20924737
ENSG00000196961	AP2A1	yellow	-0.5794015
ENSG00000183020	AP2A2	blue	0.69792826
ENSG00000006125	AP2B1	lightgreen	-0.6075933
ENSG00000161203	AP2M1	brown	0.85691363
ENSG00000042753	AP2S1	brown	0.87992737
ENSG00000132842	AP3B1	green	0.62255439
ENSG00000103723	AP3B2	blue	0.87260682
ENSG00000065000	AP3D1	grey60	0.63504821
ENSG00000185009	AP3M1	turquoise	0.60267653
ENSG00000070718	AP3M2	yellow	-0.6386617
ENSG00000177879	AP3S1	mediumpurp	0.54524753

ENSG00000134262	AP4B1	brown	0.73256706
ENSG00000081014	AP4E1	turquoise	0.3822368
ENSG00000221838	AP4M1	green	-0.6412758
ENSG00000100478	AP4S1	grey	0.0616137
ENSG00000053770	AP5M1	grey	0.00882866
ENSG00000242802	AP5Z1	brown	0.49204825
ENSG00000120868	APAF1	turquoise	-0.3943397
ENSG00000107282	APBA1	blue	0.64726271
ENSG00000034053	APBA2	yellow	-0.6132013
ENSG00000166313	APBB1	turquoise	-0.4858669
ENSG00000077420	APBB1IP	cyan	0.85802313
ENSG00000163697	APBB2	lightcyan	0.52066333
ENSG00000113108	APBB3	grey	0.01147413
ENSG00000134982	APC	yellowgreen	0.80071878
ENSG00000115266	APC2	grey	0.0573674
ENSG00000154856	APCDD1	turquoise	0.73918047
ENSG00000164062	APEH	darkred	0.718144
ENSG00000100823	APEX1	grey	0.08748962
ENSG00000169188	APEX2	grey	0.21712892
ENSG00000117362	APH1A	greenyellow	-0.4252484
ENSG00000138613	APH1B	red	0.53069766
ENSG00000166181	API5	grey	0.20567585
ENSG00000149089	APIP	yellow	0.38832337
ENSG00000175279	APITD1	brown	0.54751735
ENSG00000169621	APLF	green	0.5215628
ENSG00000171388	APLN	grey	-0.2043507
ENSG00000134817	APLNR	grey	-0.3007733
ENSG00000105290	APLP1	floralwhite	0.86091564
ENSG00000084234	APLP2	red	0.4822779
ENSG00000101474	APMAP	salmon	0.59431109
ENSG00000235910	APOA1-AS	blue	-0.4702715
ENSG00000124701	APOBEC2	grey	0.23933162
ENSG00000128383	APOBEC3A	grey	0.43329377
ENSG00000244509	APOBEC3C	grey	-0.0704209
ENSG00000243811	APOBEC3D	grey	0.29249253
ENSG00000173627	APOBEC4	grey	0.24706397
ENSG00000224916	APOC4-APOC2	grey	0.01472215
ENSG00000189058	APOD	yellow	0.59747428
ENSG00000130203	APOE	turquoise	0.49311349
ENSG00000100342	APOL1	tan	0.63361467
ENSG00000128335	APOL2	pink	0.72038979
ENSG00000128284	APOL3	tan	0.73571806
ENSG00000100336	APOL4	pink	0.80988883
ENSG00000221963	APOL6	pink	0.61443971
ENSG00000178878	APOLD1	pink	0.64092095
ENSG00000204444	APOM	brown	-0.3682154
ENSG00000184831	APOO	grey	0.07401772
ENSG00000155008	APOOL	grey	0.23389806

ENSG00000256053	APOPT1	brown	0.65205491
ENSG00000142192	APP	grey60	0.59550596
ENSG00000062725	APPBP2	turquoise	0.63682925
ENSG00000157500	APPL1	brown	-0.6406483
ENSG00000136044	APPL2	turquoise	0.75508023
ENSG00000198931	APRT	yellow	-0.6133437
ENSG00000137074	APTX	yellow	0.35918524
ENSG00000240583	AQP1	grey	-0.1092283
ENSG00000178301	AQP11	red	0.53654552
ENSG00000171885	AQP4	turquoise	0.68567481
ENSG00000260372	AQP4-AS1	grey	0.04655545
ENSG00000161798	AQP5	grey	0.02954049
ENSG00000021776	AQR	grey	0.20138836
ENSG00000169083	AR	grey	0.07100295
ENSG00000078061	ARAF	blue	-0.6075758
ENSG00000186635	ARAP1	grey	-0.10713
ENSG00000245148	ARAP1-AS2	grey	0.03756407
ENSG00000047365	ARAP2	red	-0.7938849
ENSG00000120318	ARAP3	tan	0.57769297
ENSG00000198576	ARC	grey	0.23079469
ENSG00000119682	AREL1	turquoise	-0.6835487
ENSG00000143761	ARF1	blue	0.31293015
ENSG00000168374	ARF4	grey	-0.1217739
ENSG00000165527	ARF6	brown	-0.8720152
ENSG00000101199	ARFGAP1	brown	0.74423591
ENSG00000149182	ARFGAP2	grey	0.11363514
ENSG00000242247	ARFGAP3	blue	-0.4293717
ENSG00000066777	ARFGEF1	yellow	-0.509962
ENSG00000124198	ARFGEF2	turquoise	-0.3644189
ENSG00000132254	ARFIP2	brown	0.52547806
ENSG00000101246	ARFRP1	lightcyan	-0.6315183
ENSG00000081181	ARG2	grey	0.21698843
ENSG00000265293	ARGFXP2	grey	0.16825486
ENSG00000175220	ARHGAP1	yellow	0.63661336
ENSG00000071205	ARHGAP10	black	0.79130089
ENSG00000198826	ARHGAP11A	green	0.57171969
ENSG00000165322	ARHGAP12	yellowgreen	0.55019488
ENSG00000140750	ARHGAP17	grey	-0.0236748
ENSG00000146376	ARHGAP18	black	-0.4796005
ENSG00000213390	ARHGAP19	brown	-0.6498483
ENSG00000137727	ARHGAP20	blue	0.85407531
ENSG00000107863	ARHGAP21	yellow	0.75885409
ENSG00000128805	ARHGAP22	darkred	-0.6701431
ENSG00000225485	ARHGAP23	yellow	0.83582456
ENSG00000138639	ARHGAP24	grey	-0.0753561
ENSG00000163219	ARHGAP25	cyan	0.60146461
ENSG00000145819	ARHGAP26	yellowgreen	-0.6375782
ENSG00000226272	ARHGAP26-AS1	grey	0.21910218

ENSG00000230789	ARHGAP26-IT1	grey	0.4258559
ENSG00000159314	ARHGAP27	black	0.55724521
ENSG00000137962	ARHGAP29	tan	0.79696425
ENSG00000186517	ARHGAP30	royalblue	0.580975
ENSG00000031081	ARHGAP31	turquoise	0.69540126
ENSG00000241155	ARHGAP31-AS1	turquoise	0.40885773
ENSG00000134909	ARHGAP32	grey60	0.61031851
ENSG00000160007	ARHGAP35	turquoise	0.5987281
ENSG00000147799	ARHGAP39	pink	0.46289965
ENSG00000165895	ARHGAP42	turquoise	0.68123212
ENSG00000006740	ARHGAP44	blue	0.80477361
ENSG00000100852	ARHGAP5	turquoise	0.84658674
ENSG00000258655	ARHGAP5-AS1	turquoise	0.67923097
ENSG00000047648	ARHGAP6	black	-0.7866949
ENSG00000123329	ARHGAP9	royalblue	0.70344293
ENSG00000141522	ARHGDIA	grey	0.00154847
ENSG00000111348	ARHGDIB	royalblue	0.75220931
ENSG00000242173	ARHGDIG	purple	0.72783002
ENSG00000076928	ARHGEF1	grey	-0.2858317
ENSG00000104728	ARHGEF10	yellow	0.68189476
ENSG00000074964	ARHGEF10L	turquoise	0.30234601
ENSG00000196914	ARHGEF12	darkred	0.70473189
ENSG00000198844	ARHGEF15	tan	0.7127279
ENSG00000110237	ARHGEF17	grey60	0.4806477
ENSG00000104880	ARHGEF18	grey	0.19206951
ENSG00000116584	ARHGEF2	red	-0.7537369
ENSG00000114790	ARHGEF26	turquoise	0.82718159
ENSG00000243069	ARHGEF26-AS1	turquoise	0.51300234
ENSG00000163947	ARHGEF3	blue	0.33424846
ENSG00000214694	ARHGEF33	grey	-0.0505277
ENSG00000183111	ARHGEF37	yellow	0.79851223
ENSG00000136002	ARHGEF4	grey	-0.3119416
ENSG00000165801	ARHGEF40	turquoise	0.62501471
ENSG00000129675	ARHGEF6	turquoise	0.61018484
ENSG00000102606	ARHGEF7	turquoise	-0.7647477
ENSG00000131089	ARHGEF9	blue	0.75068748
ENSG00000231729	ARHGEF9-IT1	grey	0.41308037
ENSG00000117713	ARID1A	lightcyan	0.68174892
ENSG00000049618	ARID1B	grey	0.03468483
ENSG00000189079	ARID2	grey	0.36616758
ENSG00000179361	ARID3B	grey	0.0695943
ENSG00000032219	ARID4A	lightgreen	0.73970938
ENSG00000054267	ARID4B	bisque4	0.79178784
ENSG00000196843	ARID5A	pink	0.78343476
ENSG00000150347	ARID5B	grey	0.29967753
ENSG00000166233	ARIH1	grey	0.03540149
ENSG00000177479	ARIH2	grey	0.03145981
ENSG00000221883	ARIH2OS	grey	0.30476155

ENSG00000175414	ARL10	black	-0.5948455
ENSG00000174225	ARL13A	grey	0.15911147
ENSG00000169379	ARL13B	blue	-0.508722
ENSG00000152219	ARL14EP	grey	-0.0756747
ENSG00000185305	ARL15	grey	0.03934725
ENSG00000214087	ARL16	green	-0.5128161
ENSG00000185829	ARL17A	grey	-0.0046981
ENSG00000228696	ARL17B	grey	0.08573153
ENSG00000213465	ARL2	turquoise	-0.67943
ENSG00000225225	ARL2BPP10	grey	0.35552416
ENSG00000248355	ARL2BPP6	grey	0.28019227
ENSG00000138175	ARL3	darkred	0.73404002
ENSG00000122644	ARL4A	lightgreen	0.58288651
ENSG00000188042	ARL4C	grey	0.01391515
ENSG00000175906	ARL4D	grey	0.03701437
ENSG00000162980	ARL5A	grey	-0.177835
ENSG00000165997	ARL5B	turquoise	0.48711107
ENSG00000113966	ARL6	green	-0.5617724
ENSG00000170540	ARL6IP1	mediumpurp	0.70950397
ENSG00000144746	ARL6IP5	grey	-0.1390349
ENSG00000177917	ARL6IP6	turquoise	0.69211913
ENSG00000143862	ARL8A	grey	-0.0832909
ENSG00000134108	ARL8B	red	0.57562613
ENSG00000196503	ARL9	blue	0.5386159
ENSG00000104442	ARMC1	green	0.64009949
ENSG00000170632	ARMC10	turquoise	-0.5810862
ENSG00000157343	ARMC12	grey	-0.0006063
ENSG00000118690	ARMC2	grey	-0.2508561
ENSG00000238021	ARMC4P1	grey	0.03430461
ENSG00000140691	ARMC5	brown	0.46509004
ENSG00000105676	ARMC6	brown	0.58806054
ENSG00000125449	ARMC7	grey	-0.0942725
ENSG00000114098	ARMC8	grey	0.02647939
ENSG00000135931	ARMC9	darkorange	0.43139581
ENSG00000126947	ARMCX1	mediumpurp	0.58948074
ENSG00000184867	ARMCX2	purple	0.76510342
ENSG00000102401	ARMCX3	turquoise	0.45143716
ENSG00000196440	ARMCX4	brown	0.54157769
ENSG00000125962	ARMCX5	turquoise	-0.6279231
ENSG00000198960	ARMCX6	grey	0.1910693
ENSG00000143437	ARNT	brown	-0.5246663
ENSG00000172379	ARNT2	magenta	0.55415048
ENSG00000133794	ARNTL	grey60	0.51101376
ENSG00000029153	ARNTL2	blue	0.38938917
ENSG00000241685	ARPC1A	brown	0.76254406
ENSG00000130429	ARPC1B	royalblue	0.70356573
ENSG00000163466	ARPC2	salmon	0.73392616
ENSG00000111229	ARPC3	salmon	0.68124779

ENSG00000241553	ARPC4	blue	0.37700085
ENSG00000162704	ARPC5	grey	-0.1292482
ENSG00000136950	ARPC5L	brown	0.79901399
ENSG00000128989	ARPP19	magenta	0.59519416
ENSG00000230830	ARPP21-AS1	black	0.4647509
ENSG00000120500	ARR3	grey	0.10348976
ENSG00000141480	ARRB2	cyan	0.69834469
ENSG00000197070	ARRDC1	turquoise	-0.3961823
ENSG00000105643	ARRDC2	yellow	0.66301345
ENSG00000113369	ARRDC3	blue	-0.5185054
ENSG00000140450	ARRDC4	grey	-0.1090865
ENSG00000100299	ARSA	grey	0.19264254
ENSG00000113273	ARSB	grey	0.16810604
ENSG00000006756	ARSD	red	0.5042311
ENSG00000225117	ARSDP1	grey	0.007798
ENSG00000157399	ARSE	grey	0.15416648
ENSG00000062096	ARSF	turquoise	0.58532845
ENSG00000141337	ARSG	turquoise	0.49881153
ENSG00000180801	ARSJ	turquoise	0.57880947
ENSG00000164291	ARSK	grey	0.12807732
ENSG00000156219	ART3	grey	-0.2366219
ENSG00000173409	ARV1	grey	-0.1528464
ENSG00000099889	ARVCF	grey	-0.0999873
ENSG00000004848	ARX	grey	-0.2436554
ENSG00000104763	ASAH1	grey	-0.1116381
ENSG00000204147	ASAH2B	blue	0.51332661
ENSG00000151693	ASAP2	grey	-0.1193439
ENSG00000088280	ASAP3	turquoise	0.43299154
ENSG00000065802	ASB1	brown	-0.5833716
ENSG00000196372	ASB13	grey	0.23148806
ENSG00000239388	ASB14	grey	0.3208494
ENSG00000267080	ASB16-AS1	brown	-0.3996265
ENSG00000005981	ASB4	grey	0.29481585
ENSG00000148331	ASB6	grey	0.14721476
ENSG00000183475	ASB7	red	0.40902909
ENSG00000177981	ASB8	grey	-0.0063759
ENSG00000138303	ASCC1	turquoise	0.49868825
ENSG00000100325	ASCC2	grey	0.13333239
ENSG00000112249	ASCC3	turquoise	-0.3348246
ENSG00000139352	ASCL1	turquoise	0.55414869
ENSG00000111875	ASF1A	turquoise	0.7678509
ENSG00000116539	ASH1L	magenta	0.51262864
ENSG00000235919	ASH1L-AS1	grey	0.14754934
ENSG00000129691	ASH2L	grey	-0.0587415
ENSG00000110881	ASIC1	grey	0.01681582
ENSG00000108684	ASIC2	purple	0.74482774
ENSG00000126522	ASL	brown	0.42708668
ENSG00000169093	ASMTL	green	-0.562724

ENSG00000236017	ASMTL-AS1	grey	-0.1730092
ENSG00000198356	ASNA1	red	0.5706864
ENSG00000070669	ASNS	blue	0.72475669
ENSG00000138381	ASNSD1	grey	0.01033152
ENSG00000229683	ASNSP5	grey	0.17957711
ENSG00000108381	ASPA	yellow	0.77224666
ENSG00000204653	ASPDH	grey	0.21954094
ENSG00000198363	ASPH	turquoise	0.69756617
ENSG00000174939	ASPHD1	turquoise	-0.6423428
ENSG00000106819	ASPN	grey	0.06726346
ENSG00000244617	ASPRV1	grey	0.02071458
ENSG00000169696	ASPSCR1	lightcyan	-0.6093133
ENSG00000162174	ASRGL1	blue	-0.6761025
ENSG00000130707	ASS1	purple	0.7453848
ENSG00000237437	ASS1P12	grey	0.22625641
ENSG00000223922	ASS1P2	grey	0.50352531
ENSG00000034533	ASTE1	grey	0.06825932
ENSG00000152092	ASTN1	red	0.74011724
ENSG00000064102	ASUN	yellow	0.54875867
ENSG00000171456	ASXL1	lightcyan	-0.6949675
ENSG00000141431	ASXL3	purple	0.45542968
ENSG00000138138	ATAD1	yellowgreen	0.64301244
ENSG00000119778	ATAD2B	lightgreen	0.69193806
ENSG00000197785	ATAD3A	brown	0.34638427
ENSG00000215915	ATAD3C	grey	0.28140114
ENSG00000176208	ATAD5	grey	-0.003011
ENSG00000167654	ATCAY	blue	0.76215573
ENSG00000107669	ATE1	blue	0.39012592
ENSG00000226864	ATE1-AS1	grey	0.26755977
ENSG00000123268	ATF1	grey	0.12306825
ENSG00000115966	ATF2	salmon	-0.631315
ENSG00000169136	ATF5	grey	-0.3049839
ENSG00000118217	ATF6	blue	-0.4963755
ENSG00000170653	ATF7	blue	-0.7700451
ENSG00000171681	ATF7IP	turquoise	0.67750312
ENSG00000166669	ATF7IP2	green	-0.4952474
ENSG00000152348	ATG10	brown	-0.3864783
ENSG00000175224	ATG13	turquoise	-0.4460368
ENSG00000126775	ATG14	turquoise	-0.3627506
ENSG00000085978	ATG16L1	grey	-0.1380767
ENSG00000168010	ATG16L2	cyan	0.55910228
ENSG00000066739	ATG2B	brown	0.60056709
ENSG00000144848	ATG3	grey	-0.0152691
ENSG00000101844	ATG4A	grey	0.23930611
ENSG00000168397	ATG4B	brown	0.66779044
ENSG00000125703	ATG4C	yellow	0.80558487
ENSG00000130734	ATG4D	grey	0.28899751
ENSG00000057663	ATG5	turquoise	0.38454123

ENSG00000197548	ATG7	magenta	-0.3828812
ENSG00000198925	ATG9A	turquoise	-0.4091185
ENSG00000181652	ATG9B	black	-0.5110119
ENSG00000138363	ATIC	brown	0.69455648
ENSG00000198513	ATL1	blue	0.79969615
ENSG00000119787	ATL2	grey	-0.057804
ENSG00000184743	ATL3	blue	-0.7312371
ENSG00000149311	ATM	royalblue	0.57705891
ENSG00000166454	ATMIN	grey	0.06873444
ENSG00000111676	ATN1	grey	0.02228643
ENSG00000168874	ATOH8	yellow	0.44884475
ENSG00000177556	ATOX1	brown	0.61505909
ENSG00000206190	ATP10A	tan	0.75001391
ENSG00000118322	ATP10B	yellow	0.7621581
ENSG00000145246	ATP10D	grey	0.13819376
ENSG00000058063	ATP11B	darkred	-0.6095827
ENSG00000101974	ATP11C	turquoise	-0.3681754
ENSG00000105726	ATP13A1	turquoise	-0.6025786
ENSG00000159363	ATP13A2	purple	0.73896648
ENSG00000133657	ATP13A3	grey	-0.1803575
ENSG00000127249	ATP13A4	turquoise	0.68842927
ENSG00000225473	ATP13A4-AS1	turquoise	0.42064682
ENSG00000187527	ATP13A5	grey	0.11650006
ENSG00000236508	ATP13A5-AS1	grey	0.32774146
ENSG00000163399	ATP1A1	turquoise	-0.7394641
ENSG00000203865	ATP1A1OS	grey	0.06788693
ENSG00000018625	ATP1A2	turquoise	0.69369636
ENSG00000105409	ATP1A3	blue	0.79716741
ENSG00000143153	ATP1B1	blue	0.64868792
ENSG00000249212	ATP1B1P1	grey	0.12569014
ENSG00000129244	ATP1B2	turquoise	0.59723807
ENSG00000069849	ATP1B3	turquoise	-0.5145967
ENSG00000244124	ATP1B3-AS1	grey	0.27598897
ENSG00000174437	ATP2A2	grey60	0.68352681
ENSG00000070961	ATP2B1	grey60	0.82632682
ENSG00000157087	ATP2B2	grey60	0.61194034
ENSG00000224771	ATP2B2-IT2	grey	0.21462942
ENSG00000067842	ATP2B3	blue	0.77306098
ENSG00000058668	ATP2B4	red	0.77411078
ENSG00000017260	ATP2C1	magenta	-0.5819311
ENSG00000152234	ATP5A1	green	-0.7228848
ENSG00000110955	ATP5B	darkred	0.73645683
ENSG00000165629	ATP5C1	green	-0.7185649
ENSG00000099624	ATP5D	blue	0.49677588
ENSG00000124172	ATP5E	brown	0.65418517
ENSG00000116459	ATP5F1	darkred	0.65539029
ENSG00000224451	ATP5F1P1	grey	0.20862139
ENSG00000254944	ATP5F1P5	blue	0.33883142

ENSG00000159199	ATP5G1	blue	0.70519961
ENSG00000135390	ATP5G2	darkgrey	-0.3866432
ENSG00000154518	ATP5G3	brown	0.75598669
ENSG00000169020	ATP5I	brown	0.52204358
ENSG00000154723	ATP5J	greenyellow	0.60560619
ENSG00000241468	ATP5J2	blue	0.62394344
ENSG00000261102	ATP5J2P6	grey	0.21168822
ENSG00000167283	ATP5L	brown	0.85749116
ENSG00000241837	ATP5O	brown	0.83728001
ENSG00000105341	ATP5SL	grey	0.20919098
ENSG00000071553	ATP6AP1	blue	0.76178172
ENSG00000205464	ATP6AP1L	darkgrey	0.53400765
ENSG00000182220	ATP6AP2	red	0.57758867
ENSG00000033627	ATP6V0A1	blue	0.80779612
ENSG00000185344	ATP6V0A2	grey	0.22447349
ENSG00000117410	ATP6V0B	blue	0.53886192
ENSG00000185883	ATP6V0C	purple	0.39647749
ENSG00000230201	ATP6V0CP1	grey	0.24493103
ENSG00000159720	ATP6V0D1	brown	0.82843933
ENSG00000113732	ATP6V0E1	blue	-0.6449119
ENSG00000225364	ATP6V0E1P1	grey	0.49776512
ENSG00000223513	ATP6V0E1P2	grey	0.39411825
ENSG00000171130	ATP6V0E2	blue	0.36888759
ENSG00000204934	ATP6V0E2-AS1	grey	0.18489775
ENSG00000114573	ATP6V1A	purple	0.75874252
ENSG00000147416	ATP6V1B2	brown	0.84701211
ENSG00000155097	ATP6V1C1	yellow	-0.7126791
ENSG00000143882	ATP6V1C2	grey	0.16149484
ENSG00000100554	ATP6V1D	yellow	-0.7738037
ENSG00000131100	ATP6V1E1	blue	0.77459888
ENSG00000250565	ATP6V1E2	grey	-0.0240513
ENSG00000128524	ATP6V1F	yellow	-0.7555695
ENSG00000136888	ATP6V1G1	red	0.68345911
ENSG00000233343	ATP6V1G1P4	grey	0.40638418
ENSG00000213760	ATP6V1G2	lightcyan	0.60017389
ENSG00000047249	ATP6V1H	green	-0.8369232
ENSG00000165240	ATP7A	grey	0.11142713
ENSG00000123191	ATP7B	tan	0.54417253
ENSG00000124406	ATP8A1	darkmagenta	0.87703333
ENSG00000132932	ATP8A2	blue	0.76244793
ENSG00000229800	ATP8A2P2	grey	0.41417784
ENSG00000081923	ATP8B1	tan	0.52021806
ENSG00000143515	ATP8B2	grey	0.30510223
ENSG00000054793	ATP9A	turquoise	-0.4077288
ENSG00000123472	ATPAF1	grey	0.02138683
ENSG00000171953	ATPAF2	grey	0.41495829
ENSG00000175054	ATR	turquoise	-0.7286723
ENSG00000138085	ATRAID	brown	0.4015705

ENSG00000164053	ATRIP	grey	0.27842812
ENSG00000088812	ATRN	brown	0.61204568
ENSG00000107518	ATRNL1	blue	0.55691817
ENSG00000124788	ATXN1	darkorange	0.54490855
ENSG00000130638	ATXN10	brown	0.722565
ENSG00000224470	ATXN1L	brown	-0.8369974
ENSG00000204842	ATXN2	grey	0.04208165
ENSG00000168488	ATXN2L	grey	0.07073254
ENSG00000163635	ATXN7	grey	-0.1329844
ENSG00000146776	ATXN7L1	grey	-0.139141
ENSG00000162650	ATXN7L2	grey	0.17818363
ENSG00000087152	ATXN7L3	darkorange	0.52479634
ENSG00000253719	ATXN7L3B	grey	-0.092177
ENSG00000148090	AUH	brown	0.54892272
ENSG00000127423	AUNIP	grey	0.30226936
ENSG00000087586	AURKA	grey	0.4495446
ENSG00000175756	AURKAIP1	brown	0.53909698
ENSG00000213033	AURKAPS1	grey	0.41739414
ENSG00000105146	AURKC	grey	0.15286377
ENSG00000158321	AUTS2	grey	0.04748543
ENSG00000169857	AVEN	grey	0.19309172
ENSG00000105778	AVL9	brown	0.48683582
ENSG00000119986	AVPI1	grey	0.11461168
ENSG00000103126	AXIN1	brown	-0.4386849
ENSG00000168646	AXIN2	turquoise	0.47165824
ENSG00000167601	AXL	turquoise	0.87365691
ENSG00000141577	AZI1	turquoise	-0.2645988
ENSG00000163512	AZI2	blue	0.62486992
ENSG00000162885	B3GALNT2	grey	0.3162448
ENSG00000172318	B3GALT1	blue	0.37632382
ENSG00000162630	B3GALT2	purple	0.69363266
ENSG00000235863	B3GALT4	grey	0.15200385
ENSG00000183778	B3GALT5	magenta	0.72047632
ENSG00000176022	B3GALT6	grey	-0.1008223
ENSG00000187676	B3GALTL	turquoise	-0.459636
ENSG00000109956	B3GAT1	grey	-0.0745884
ENSG00000112309	B3GAT2	grey	-0.1179728
ENSG00000174684	B3GNT1	brown	0.76399219
ENSG00000170340	B3GNT2	grey	0.02217236
ENSG00000176383	B3GNT4	turquoise	-0.3414234
ENSG00000176597	B3GNT5	grey	-0.2331807
ENSG00000237172	B3GNT9	grey	-0.0690121
ENSG00000175711	B3GNTL1	grey60	0.42682216
ENSG00000135454	B4GALNT1	grey	-0.0975029
ENSG00000182272	B4GALNT4	brown	0.65590899
ENSG00000086062	B4GALT1	pink	0.70616084
ENSG00000117411	B4GALT2	lightcyan	0.77645238
ENSG00000158850	B4GALT3	brown	0.37965645

ENSG00000121578	B4GALT4	grey	-0.1720648
ENSG00000158470	B4GALT5	turquoise	-0.3924389
ENSG00000118276	B4GALT6	blue	0.6746859
ENSG00000027847	B4GALT7	blue	0.52992989
ENSG00000108641	B9D1	blue	0.73465747
ENSG00000123810	B9D2	yellow	-0.4532905
ENSG00000164929	BAALC	turquoise	0.81291323
ENSG00000105393	BABAM1	brown	0.69300817
ENSG00000186318	BACE1	yellow	0.56036113
ENSG00000182240	BACE2	pink	0.78286111
ENSG00000156273	BACH1	blue	-0.5523285
ENSG00000248476	BACH1-IT1	grey	0.28391579
ENSG00000112182	BACH2	grey60	-0.4679604
ENSG00000002330	BAD	yellow	-0.4421714
ENSG00000107262	BAG1	brown	0.62625365
ENSG00000151929	BAG3	pink	0.74287793
ENSG00000156735	BAG4	blue	0.5630213
ENSG00000166170	BAG5	brown	-0.692715
ENSG00000204463	BAG6	blue	0.45129566
ENSG00000140320	BAHD1	grey	-0.0848116
ENSG00000181790	BAI1	blue	0.69283885
ENSG00000121753	BAI2	blue	0.4831821
ENSG00000135298	BAI3	turquoise	-0.3625051
ENSG00000175866	BAIAP2	darkmagenta	0.84797733
ENSG00000226137	BAIAP2-AS1	magenta	0.63273503
ENSG00000006453	BAIAP2L1	grey	0.14280116
ENSG00000128298	BAIAP2L2	yellow	0.45992952
ENSG00000030110	BAK1	grey	0.17272712
ENSG00000095739	BAMBI	turquoise	0.52678296
ENSG00000175334	BANF1	darkgrey	0.38092173
ENSG00000258531	BANF1P1	grey	0.30326282
ENSG00000237758	BANF1P3	grey	0.39769653
ENSG00000223828	BANF1P4	grey	0.01305544
ENSG00000163930	BAP1	grey	-0.0059877
ENSG00000138376	BARD1	brown	-0.5725887
ENSG00000176788	BASP1	purple	0.78635432
ENSG00000156127	BATF	grey	0.25861512
ENSG00000123685	BATF3	grey	-0.2540392
ENSG00000087088	BAX	grey	-0.0107119
ENSG00000198604	BAZ1A	darkgrey	0.72395268
ENSG00000009954	BAZ1B	darkgrey	0.70434955
ENSG00000076108	BAZ2A	lightgreen	0.5567508
ENSG00000123636	BAZ2B	yellow	0.73350607
ENSG00000214413	BBIP1	yellow	0.30963021
ENSG00000129151	BBOX1	turquoise	0.58592965
ENSG00000174483	BBS1	grey	0.21009529
ENSG00000179941	BBS10	turquoise	0.70683769
ENSG00000181004	BBS12	green	-0.5217203

ENSG00000125124	BBS2	turquoise	0.23713803
ENSG00000140463	BBS4	red	0.57680133
ENSG00000163093	BBS5	grey	-0.0186875
ENSG00000138686	BBS7	blue	0.54426786
ENSG00000122507	BBS9	grey	-0.0383149
ENSG00000114439	BBX	yellow	0.64795171
ENSG00000187244	BCAM	grey	0.09355271
ENSG00000132692	BCAN	yellowgreen	0.73095181
ENSG00000075790	BCAP29	grey	0.03305893
ENSG00000185825	BCAP31	grey	0.17132309
ENSG00000050820	BCAR1	turquoise	-0.3355127
ENSG00000137936	BCAR3	turquoise	0.75131439
ENSG00000064787	BCAS1	green	0.8824966
ENSG00000116752	BCAS2	grey	0.07457607
ENSG00000141376	BCAS3	blue	0.44040678
ENSG00000124243	BCAS4	grey	-0.0999991
ENSG00000060982	BCAT1	grey	0.28002006
ENSG00000105552	BCAT2	grey	0.11534411
ENSG00000107949	BCCIP	grey	0.22370051
ENSG00000186666	BCDIN3D	grey	0.30884429
ENSG00000258057	BCDIN3D-AS1	grey	0.22691614
ENSG00000114200	BCHE	blue	0.42959595
ENSG00000083123	BCKDHB	turquoise	0.66574944
ENSG00000103507	BCKDK	grey	0.29532534
ENSG00000142867	BCL10	grey	0.29588755
ENSG00000119866	BCL11A	magenta	0.61487611
ENSG00000127152	BCL11B	magenta	0.85730154
ENSG00000171791	BCL2	turquoise	0.7737798
ENSG00000171552	BCL2L1	yellow	0.60401934
ENSG00000153094	BCL2L11	brown	-0.6166986
ENSG00000099968	BCL2L13	grey	0.20593194
ENSG00000129473	BCL2L2	brown	-0.8221609
ENSG00000069399	BCL3	pink	0.76634825
ENSG00000113916	BCL6	pink	0.82443041
ENSG00000161940	BCL6B	tan	0.66846389
ENSG00000110987	BCL7A	grey	0.01771869
ENSG00000106635	BCL7B	brown	0.47526143
ENSG00000099385	BCL7C	grey	-0.002899
ENSG00000186174	BCL9L	lightcyan	0.53376834
ENSG00000029363	BCLAF1	turquoise	-0.4563511
ENSG00000135697	BCMO1	grey	-0.0361565
ENSG00000197580	BCO2	turquoise	-0.5402862
ENSG00000183337	BCOR	grey	0.08507313
ENSG00000085185	BCORL1	grey	0.11908404
ENSG00000186716	BCR	black	0.54009517
ENSG00000169668	BCRP2	grey	-0.041207
ENSG00000074582	BCS1L	brown	0.37481764
ENSG00000236824	BCYRN1	grey	-0.258675

ENSG00000164039	BDH2	turquoise	0.54623683
ENSG00000245573	BDNF-AS	grey	0.20588004
ENSG00000145734	BDP1	grey	-0.0383904
ENSG00000126581	BECN1	grey	0.11361075
ENSG00000183092	BEGAIN	grey	-0.1219667
ENSG00000162373	BEND5	blue	0.3645094
ENSG00000165626	BEND7	grey	0.29135734
ENSG00000167995	BEST1	yellow	0.61483756
ENSG00000142959	BEST4	brown	0.53210488
ENSG00000105829	BET1	grey	-0.09672
ENSG00000177951	BET1L	blue	-0.6287477
ENSG00000102409	BEX4	turquoise	0.46791467
ENSG00000103429	BFAR	magenta	-0.4497147
ENSG00000125864	BFSP1	grey	-0.1550113
ENSG00000182492	BGN	brown	-0.5705653
ENSG00000198908	BHLHB9	grey	-0.1961612
ENSG00000134107	BHLHE40	grey	0.39189811
ENSG00000235831	BHLHE40-AS1	grey	0.04866268
ENSG00000123095	BHLHE41	grey	-0.3692222
ENSG00000132840	BHMT2	turquoise	0.60393663
ENSG00000122870	BICC1	turquoise	0.42742158
ENSG00000151746	BICD1	yellow	-0.4705142
ENSG00000185963	BICD2	lightgreen	0.6424132
ENSG00000015475	BID	grey	0.02666889
ENSG00000136717	BIN1	turquoise	-0.454548
ENSG00000110934	BIN2	cyan	0.76125581
ENSG00000147439	BIN3	grey	0.34652888
ENSG00000110330	BIRC2	brown	-0.5612653
ENSG00000089685	BIRC5	grey	0.21787531
ENSG00000115760	BIRC6	grey	0.2802381
ENSG00000230046	BIRC6-AS1	grey	0.15200791
ENSG00000134897	BIVM	grey	0.00213391
ENSG00000166619	BLCAP	purple	0.73491616
ENSG00000259571	BLID	grey	0.26444422
ENSG00000197299	BLM	grey	-0.0908518
ENSG00000095585	BLNK	cyan	0.86372539
ENSG00000135441	BLOC1S1	grey	-0.2603109
ENSG00000196072	BLOC1S2	brown	-0.3312991
ENSG00000189114	BLOC1S3	grey	0.1382955
ENSG00000186222	BLOC1S4	green	-0.6069464
ENSG00000188428	BLOC1S5	brown	-0.3318063
ENSG00000090013	BLVRB	grey	-0.1153929
ENSG00000117475	BLZF1	grey	0.10219417
ENSG00000168283	BMI1	brown	-0.5795377
ENSG00000168487	BMP1	black	0.58698436
ENSG00000125845	BMP2	pink	0.50528393
ENSG00000138756	BMP2K	turquoise	0.51934756
ENSG00000204113	BMP2KL	grey	0.22232392

ENSG00000153162	BMP6	tan	0.56548084
ENSG00000101144	BMP7	turquoise	0.75075841
ENSG00000116985	BMP8B	grey	-0.2315061
ENSG00000164619	BMPER	red	0.49755203
ENSG00000107779	BMPR1A	turquoise	0.64933081
ENSG00000138696	BMPR1B	turquoise	0.88058914
ENSG00000204217	BMPR2	yellow	-0.7319403
ENSG00000165733	BMS1	turquoise	-0.5443749
ENSG00000237238	BMS1P10	grey	0.11985395
ENSG00000258780	BMS1P15	grey	0.28109322
ENSG00000251079	BMS1P2	grey	0.17523773
ENSG00000236850	BMS1P20	grey	-0.0456364
ENSG00000243899	BMS1P7	grey	0.07650809
ENSG00000260518	BMS1P8	grey	0.06057453
ENSG00000113734	BNIP1	blue	0.40773255
ENSG00000140299	BNIP2	brown	-0.8446041
ENSG00000176171	BNIP3	turquoise	0.6921801
ENSG00000104765	BNIP3L	yellow	0.66494472
ENSG00000197358	BNIP3P1	turquoise	0.59960551
ENSG00000144857	BOC	pink	0.63984843
ENSG00000145919	BOD1	salmon	0.70573505
ENSG00000038219	BOD1L1	darkgrey	0.67826044
ENSG00000176720	BOK	darkred	-0.6980614
ENSG00000178096	BOLA1	green	-0.4563035
ENSG00000163170	BOLA3	brown	0.73283719
ENSG00000225439	BOLA3-AS1	grey	0.07680968
ENSG00000136122	BORA	green	0.37005384
ENSG00000172331	BPGM	turquoise	0.61031786
ENSG00000137274	BPHL	grey	0.0312382
ENSG00000125999	BPIFB1	grey	0.06903729
ENSG00000125997	BPIFB9P	grey	0.20151788
ENSG00000171634	BPTF	lightgreen	0.65466085
ENSG00000157764	BRAF	turquoise	-0.5294499
ENSG00000089234	BRAP	lightgreen	0.50892432
ENSG00000106009	BRAT1	brown	0.67609261
ENSG00000012048	BRCA1	yellow	0.6830872
ENSG00000185515	BRCC3	blue	0.51209349
ENSG00000100425	BRD1	lightgreen	0.58103947
ENSG00000204256	BRD2	grey	-0.0769899
ENSG00000169925	BRD3	bisque4	0.79144668
ENSG00000141867	BRD4	grey	-0.0523371
ENSG00000166164	BRD7	darkgrey	0.65354407
ENSG00000169075	BRD7P3	grey	0.11398596
ENSG00000218676	BRD7P4	grey	0.45457073
ENSG00000112983	BRD8	lightcyan	-0.587144
ENSG00000028310	BRD9	turquoise	-0.4592823
ENSG00000158019	BRE	brown	0.70461415
ENSG00000185024	BRF1	brown	0.48356703

ENSG00000164713	BRI3	greenyellow	0.5463222
ENSG00000184992	BRI3BP	turquoise	0.44986852
ENSG00000182685	BRICD5	grey	-0.0283939
ENSG00000078725	BRINP1	grey60	0.4269962
ENSG00000198797	BRINP2	red	0.7042976
ENSG00000162670	BRINP3	red	0.71838193
ENSG00000113460	BRIX1	grey	0.13608817
ENSG00000254999	BRK1	grey	-0.0376952
ENSG00000174744	BRMS1	grey	0.08825565
ENSG00000100916	BRMS1L	brown	0.43385403
ENSG00000162819	BROX	grey	-0.2318567
ENSG00000156983	BRPF1	black	0.71729489
ENSG00000096070	BRPF3	brown	-0.610532
ENSG00000160469	BRSK1	turquoise	-0.5233731
ENSG00000174672	BRSK2	blue	0.739929
ENSG00000185658	BRWD1	grey	-0.1048779
ENSG00000238141	BRWD1-AS1	grey	0.4095351
ENSG00000165288	BRWD3	grey	0.05611145
ENSG00000168000	BSCL2	blue	0.58342194
ENSG00000160058	BSDC1	grey	-0.1585774
ENSG00000172270	BSG	brown	-0.6085746
ENSG00000164061	BSN	darkorange	0.81154359
ENSG00000119411	BSPRY	grey	0.12584739
ENSG00000130303	BST2	blue	-0.6328017
ENSG00000095564	BTAF1	grey	-0.025699
ENSG00000064726	BTBD1	grey	0.02621292
ENSG00000148925	BTBD10	brown	0.56846031
ENSG00000151136	BTBD11	purple	0.84541748
ENSG00000233436	BTBD18	grey	0.23471502
ENSG00000222009	BTBD19	grey	0.03981899
ENSG00000132640	BTBD3	yellow	0.49114318
ENSG00000184887	BTBD6	grey	-0.2368871
ENSG00000011114	BTBD7	turquoise	0.50110052
ENSG00000203414	BTBD7P1	grey	0.37957901
ENSG00000189195	BTBD8	grey	0.23852363
ENSG00000183826	BTBD9	blue	0.75642129
ENSG00000226533	BTBD9-AS1	grey	0.31719118
ENSG00000169814	BTD	grey	-0.171957
ENSG00000145741	BTF3	brown	0.61983689
ENSG00000232260	BTF3L4P1	grey	0.27972458
ENSG00000213189	BTF3L4P2	grey	0.53377479
ENSG00000213003	BTF3P12	grey	0.43237402
ENSG00000133639	BTG1	darkorange	-0.5177355
ENSG00000154640	BTG3	darkmagenta	0.64113196
ENSG00000112763	BTN2A1	salmon	0.61229848
ENSG00000026950	BTN3A1	grey	-0.0440639
ENSG00000186470	BTN3A2	grey	-0.187059
ENSG00000111801	BTN3A3	grey	-0.1657876

ENSG00000165810	BTNL9	tan	0.79285714
ENSG00000166167	BTRC	blue	0.82748237
ENSG00000154473	BUB3	grey	-0.0317287
ENSG00000137656	BUD13	grey	-0.1283404
ENSG00000112276	BVES	turquoise	0.39083237
ENSG00000215933	BX119917.1	grey	0.13935665
ENSG00000196400	BX255923.1	grey	0.06981357
ENSG00000215447	BX322557.10	grey	-0.0041151
ENSG00000197180	BX936347.1	grey	0.24704366
ENSG00000112578	BYSL	salmon	0.52151665
ENSG00000005379	BZRAP1	grey	-0.0887218
ENSG00000082153	BZW1	grey	0.06333556
ENSG00000165507	C10orf10	pink	0.85177192
ENSG00000183346	C10orf107	grey	-0.1915923
ENSG00000148655	C10orf11	black	-0.6694183
ENSG00000204683	C10orf113	turquoise	0.49677188
ENSG00000165813	C10orf118	grey	-0.0884935
ENSG00000155640	C10orf12	grey	0.04872265
ENSG00000204161	C10orf128	darkred	-0.5996616
ENSG00000173124	C10orf129	grey	0.01575908
ENSG00000173088	C10orf131	grey	0.20909216
ENSG00000107815	C10orf2	grey	0.25107681
ENSG00000165511	C10orf25	grey	0.04370631
ENSG00000166275	C10orf32	mediumpurp	0.80552848
ENSG00000107738	C10orf54	blue	-0.623672
ENSG00000150076	C10ORF68	grey	0.02216117
ENSG00000120029	C10orf76	mediumpurp	-0.6054204
ENSG00000177234	C10orf85	turquoise	0.34000829
ENSG00000119965	C10orf88	grey	-0.2589844
ENSG00000154493	C10orf90	yellow	0.89956986
ENSG00000137720	C11orf1	grey	-0.269954
ENSG00000171067	C11orf24	black	0.70754512
ENSG00000158636	C11orf30	grey	0.1835487
ENSG00000211450	C11orf31	brown	0.85513654
ENSG00000180878	C11orf42	grey	0.12513857
ENSG00000174370	C11orf45	red	-0.5351471
ENSG00000149179	C11orf49	brown	0.74150974
ENSG00000182919	C11orf54	grey	0.08860805
ENSG00000150776	C11orf57	turquoise	-0.3949906
ENSG00000110696	C11orf58	grey	-0.1831896
ENSG00000109944	C11orf63	blue	0.76214934
ENSG00000166323	C11orf65	brown	-0.4516792
ENSG00000175573	C11orf68	grey	0.16165587
ENSG00000180425	C11orf71	yellow	0.33880001
ENSG00000184224	C11orf72	grey	0.03669841
ENSG00000166352	C11orf74	turquoise	-0.6627556
ENSG00000173715	C11orf80	blue	0.47951633
ENSG00000204922	C11orf83	lightcyan	-0.6273635

ENSG00000168005	C11orf84	grey	-0.046209
ENSG00000185742	C11orf87	blue	0.48759743
ENSG00000188070	C11orf95	floralwhite	-0.6211953
ENSG00000187479	C11orf96	pink	0.67171558
ENSG00000139637	C12orf10	blue	0.66506304
ENSG00000151135	C12orf23	mediumpurp	0.66846432
ENSG00000133641	C12orf29	grey	-0.0774111
ENSG00000134548	C12orf39	brown	-0.7559611
ENSG00000047621	C12orf4	turquoise	-0.4193773
ENSG00000157895	C12orf43	blue	0.53382768
ENSG00000123395	C12orf44	salmon	0.55606865
ENSG00000111412	C12orf49	turquoise	0.68506996
ENSG00000078237	C12orf5	grey	0.27533645
ENSG00000111678	C12orf57	brown	0.45013358
ENSG00000130921	C12orf65	grey	-0.0746064
ENSG00000174206	C12orf66	brown	0.33185051
ENSG00000204954	C12orf73	green	-0.4004964
ENSG00000235162	C12orf75	grey	-0.2484394
ENSG00000174456	C12orf76	darkgrey	0.51743092
ENSG00000133935	C14orf1	grey	0.08904761
ENSG00000179933	C14orf119	grey	-0.1012309
ENSG00000227051	C14orf132	brown	-0.5459846
ENSG00000170270	C14orf142	grey	0.03031614
ENSG00000133943	C14orf159	turquoise	0.40644921
ENSG00000215277	C14orf164	grey	0.30919475
ENSG00000087302	C14orf166	salmon	0.82555749
ENSG00000156411	C14orf2	brown	0.78516413
ENSG00000186960	C14orf23	magenta	0.82365427
ENSG00000139971	C14orf37	turquoise	0.49182241
ENSG00000246223	C14orf64	grey	-0.1458575
ENSG00000140104	C14orf79	yellow	-0.629551
ENSG00000185347	C14orf80	turquoise	-0.5343531
ENSG00000100802	C14orf93	grey	-0.0243295
ENSG00000242498	C15orf38	brown	-0.6636044
ENSG00000167173	C15orf39	black	-0.5965225
ENSG00000169609	C15orf40	grey	0.15547306
ENSG00000186073	C15orf41	grey	-0.252716
ENSG00000188549	C15orf52	turquoise	0.48372968
ENSG00000128891	C15orf57	grey	-0.062519
ENSG00000205363	C15orf59	grey	0.25905172
ENSG00000183324	C15orf60	grey	0.39152556
ENSG00000189227	C15orf61	greenyellow	0.57409204
ENSG00000188277	C15orf62	yellow	0.43995508
ENSG00000261652	C15orf65	grey	-0.0220223
ENSG00000130731	C16orf13	blue	0.53775118
ENSG00000221819	C16orf3	grey	0.29671894
ENSG00000166780	C16orf45	blue	0.74751451
ENSG00000166455	C16orf46	grey	0.29136532

ENSG00000185716	C16orf52	grey	-0.0905385
ENSG00000140688	C16orf58	turquoise	-0.4521486
ENSG00000103544	C16orf62	grey	0.4019957
ENSG00000125149	C16orf70	brown	0.47760169
ENSG00000182831	C16orf72	brown	-0.604718
ENSG00000154102	C16orf74	grey	0.1962932
ENSG00000070761	C16orf80	green	-0.8025309
ENSG00000159761	C16orf86	grey	-0.0187426
ENSG00000155330	C16orf87	grey	-0.0187464
ENSG00000153446	C16orf89	turquoise	0.65601645
ENSG00000154035	C17orf103	blue	-0.7285637
ENSG00000205710	C17orf107	grey	-0.2871646
ENSG00000212719	C17orf51	grey	-0.0330482
ENSG00000186665	C17orf58	grey	0.11416209
ENSG00000196544	C17orf59	grey	0.2516675
ENSG00000178927	C17orf62	royalblue	0.59916739
ENSG00000172653	C17orf66	grey	0.15376717
ENSG00000214226	C17orf67	grey	0.16214674
ENSG00000185504	C17orf70	green	-0.3525728
ENSG00000108666	C17orf75	turquoise	-0.4553022
ENSG00000167230	C17orf78	grey	0.31920768
ENSG00000141219	C17orf80	turquoise	0.67656148
ENSG00000074356	C17orf85	darkred	-0.56914
ENSG00000224877	C17orf89	greenyellow	0.62014728
ENSG00000187624	C17orf97	grey	-0.0662002
ENSG00000141428	C18orf21	grey	0.12475224
ENSG00000152242	C18orf25	grey	0.17827425
ENSG00000177576	C18orf32	grey	-0.0254297
ENSG00000166845	C18orf54	grey	0.18693948
ENSG00000176912	C18orf56	yellow	0.61513605
ENSG00000141452	C18orf8	yellow	0.45832285
ENSG00000074842	C19orf10	grey	0.10041444
ENSG00000131943	C19orf12	green	-0.4249613
ENSG00000228300	C19orf24	turquoise	-0.4369481
ENSG00000119559	C19orf25	grey	0.28227311
ENSG00000099625	C19orf26	grey	0.0297822
ENSG00000123144	C19orf43	grey	-0.0380863
ENSG00000105072	C19orf44	grey	0.16041231
ENSG00000160392	C19orf47	green	0.53968861
ENSG00000167747	C19orf48	grey	0.22699243
ENSG00000104979	C19orf53	yellow	-0.4925168
ENSG00000188493	C19orf54	brown	-0.6999967
ENSG00000167595	C19orf55	turquoise	-0.368499
ENSG00000006015	C19orf60	brown	0.54263402
ENSG00000130813	C19orf66	grey	0.01401198
ENSG00000185453	C19orf68	green	0.56857448
ENSG00000174917	C19orf70	brown	0.57946724
ENSG00000221916	C19orf73	grey	0.10040316

ENSG00000235034	C19orf81	yellow	-0.5708757
ENSG00000267106	C19orf82	green	0.48651923
ENSG00000197223	C1D	blue	0.32262522
ENSG00000106392	C1GALT1	yellow	0.54535925
ENSG00000171155	C1GALT1C1	grey	0.22199786
ENSG00000179397	C1orf101	grey	0.16074669
ENSG00000116922	C1orf109	grey	0.23563847
ENSG00000000460	C1orf112	grey	0.26637196
ENSG00000197982	C1orf122	grey	-0.1164765
ENSG00000162384	C1orf123	brown	0.56214282
ENSG00000143633	C1orf131	grey	0.14631495
ENSG00000203709	C1orf132	black	0.60306186
ENSG00000162913	C1orf145	magenta	0.71618744
ENSG00000131591	C1orf159	grey	0.02014696
ENSG00000143110	C1orf162	royalblue	0.68316853
ENSG00000178965	C1orf173	blue	0.69378029
ENSG00000198912	C1orf174	grey	-0.2868285
ENSG00000162398	C1orf177	grey	-0.0067155
ENSG00000188931	C1orf192	brown	-0.520123
ENSG00000119280	C1orf198	turquoise	0.65966866
ENSG00000188004	C1orf204	brown	-0.3952438
ENSG00000116667	C1orf21	yellowgreen	0.73692211
ENSG00000249087	C1orf213	grey	-0.0054027
ENSG00000142686	C1orf216	blue	0.57207934
ENSG00000198520	C1orf228	grey	-0.0453138
ENSG00000228594	C1orf233	blue	0.45484818
ENSG00000157181	C1orf27	grey	-0.0577428
ENSG00000143793	C1orf35	grey	-0.1906987
ENSG00000143612	C1orf43	grey	-0.2994641
ENSG00000164008	C1orf50	brown	0.37379562
ENSG00000159208	C1orf51	grey	0.22102241
ENSG00000162642	C1orf52	black	-0.5592127
ENSG00000203724	C1orf53	grey	0.13994248
ENSG00000118292	C1orf54	grey	0.00565828
ENSG00000143443	C1orf56	grey	0.18470099
ENSG00000125462	C1orf61	yellowgreen	0.76262093
ENSG00000117616	C1orf63	grey	-0.2205031
ENSG00000183888	C1orf64	tan	0.73121529
ENSG00000198715	C1orf85	grey	0.00958654
ENSG00000162585	C1orf86	grey	0.12848099
ENSG00000173372	C1QA	royalblue	0.84307678
ENSG00000173369	C1QB	royalblue	0.9177343
ENSG00000159189	C1QC	royalblue	0.91108851
ENSG00000131094	C1QL1	grey	-0.1920675
ENSG00000173918	C1QTNF1	purple	-0.7370774
ENSG00000082196	C1QTNF3	turquoise	0.54158793
ENSG00000172247	C1QTNF4	brown	0.78170288
ENSG00000133466	C1QTNF6	grey	-0.0459663

ENSG00000163145	C1QTNF7	grey	0.27190263
ENSG00000159403	C1R	pink	0.79168126
ENSG00000139178	C1RL	grey	0.245193
ENSG00000182326	C1S	pink	0.68679507
ENSG00000197183	C20orf112	darkred	-0.4324766
ENSG00000088854	C20orf194	turquoise	0.35088481
ENSG00000171984	C20orf196	grey	0.11653008
ENSG00000198547	C20orf203	turquoise	-0.546448
ENSG00000101084	C20orf24	green	-0.5666621
ENSG00000089101	C20orf26	blue	0.51567637
ENSG00000101220	C20orf27	magenta	0.54838658
ENSG00000256073	C21orf119	red	0.57182556
ENSG00000222018	C21orf140	grey	0.22745997
ENSG00000160226	C21orf2	brown	0.58309902
ENSG00000160221	C21orf33	grey	0.19185037
ENSG00000232560	C21orf37	grey	-0.0921111
ENSG00000205930	C21orf49	grey	0.09969517
ENSG00000159079	C21orf59	grey	-0.0598338
ENSG00000205929	C21orf62	grey	0.06160477
ENSG00000183250	C21orf67	grey	0.17508026
ENSG00000184809	C21orf88	magenta	0.59177835
ENSG00000154642	C21orf91	yellow	0.84450691
ENSG00000128346	C22orf23	grey60	-0.5049195
ENSG00000215012	C22orf29	darkred	0.6401133
ENSG00000100249	C22orf31	grey	0.35799272
ENSG00000242259	C22orf39	grey	0.0544707
ENSG00000184208	C22orf46	green	0.7154036
ENSG00000157617	C2CD2	grey	-0.0589978
ENSG00000172375	C2CD2L	turquoise	-0.6292645
ENSG00000168014	C2CD3	brown	0.51288194
ENSG00000111731	C2CD5	turquoise	-0.5760072
ENSG00000241962	C2orf15	grey	0.10314764
ENSG00000197927	C2orf27A	grey	-0.0761385
ENSG00000119147	C2orf40	grey	-0.1283025
ENSG00000115998	C2orf42	brown	0.39993526
ENSG00000118961	C2orf43	grey	-0.0377675
ENSG00000163026	C2orf44	grey	0.10907374
ENSG00000162972	C2orf47	grey	0.32197566
ENSG00000135974	C2orf49	turquoise	0.78655748
ENSG00000150873	C2orf50	grey	-0.0272083
ENSG00000168887	C2orf68	yellow	0.53567662
ENSG00000178074	C2orf69	turquoise	0.83433314
ENSG00000204128	C2orf72	turquoise	0.76237449
ENSG00000177994	C2orf73	grey	0.18676134
ENSG00000237651	C2orf74	grey	0.292148
ENSG00000186132	C2orf76	grey	0.00798708
ENSG00000159239	C2orf81	grey	-0.1485603
ENSG00000182600	C2orf82	grey	0.02574921

ENSG00000187699	C2orf88	grey	-0.0304963
ENSG00000125730	C3	cyan	0.89004871
ENSG00000171860	C3AR1	cyan	0.87818208
ENSG00000114405	C3orf14	blue	0.67490672
ENSG00000163608	C3orf17	grey	-0.1176209
ENSG00000088543	C3orf18	grey	0.11711897
ENSG00000174928	C3orf33	grey	0.2457052
ENSG00000198590	C3orf35	grey	0.37631412
ENSG00000179021	C3orf38	turquoise	0.44180712
ENSG00000163632	C3orf49	grey	0.14890356
ENSG00000181744	C3orf58	brown	-0.6308939
ENSG00000188315	C3orf62	turquoise	-0.4978768
ENSG00000187068	C3orf70	turquoise	0.88445236
ENSG00000154274	C4orf19	pink	0.65137489
ENSG00000138658	C4orf21	green	0.55858206
ENSG00000164074	C4orf29	grey	0.06137214
ENSG00000164096	C4orf3	brown	-0.8027719
ENSG00000174749	C4orf32	yellow	0.67399325
ENSG00000151470	C4orf33	blue	0.36559731
ENSG00000163633	C4orf36	grey	0.0764439
ENSG00000205208	C4orf46	turquoise	0.54932935
ENSG00000205129	C4orf47	grey	-0.1051861
ENSG00000243449	C4orf48	grey	-0.1994542
ENSG00000106804	C5	grey	0.06071698
ENSG00000197405	C5AR1	royalblue	0.63480619
ENSG00000113583	C5orf15	blue	-0.5354409
ENSG00000082213	C5orf22	blue	0.65073786
ENSG00000181904	C5orf24	brown	-0.6148482
ENSG00000236882	C5orf27	grey	0.23469266
ENSG00000151881	C5orf28	grey	0.15437935
ENSG00000181751	C5orf30	grey	0.03987059
ENSG00000172244	C5orf34	grey	-0.1313686
ENSG00000197603	C5orf42	grey	-0.1238705
ENSG00000161010	C5orf45	grey	-0.0670383
ENSG00000215217	C5orf49	black	-0.6447563
ENSG00000205765	C5orf51	turquoise	0.64241088
ENSG00000221886	C5orf54	blue	-0.3601589
ENSG00000197536	C5orf56	blue	-0.6247755
ENSG00000178722	C5orf64	yellow	0.57523938
ENSG00000224186	C5orf66	grey	0.09868323
ENSG00000186577	C6orf1	pink	0.51818269
ENSG00000204296	C6orf10	grey	-0.0725882
ENSG00000196821	C6orf106	turquoise	-0.5293698
ENSG00000185127	C6orf120	grey	0.13852311
ENSG00000204564	C6orf136	blue	0.61234602
ENSG00000197261	C6orf141	magenta	0.63520275
ENSG00000203872	C6orf163	grey	0.08353769
ENSG00000203871	C6orf164	grey	0.24034099

ENSG00000130349	C6orf203	lightgreen	0.55692591
ENSG00000146476	C6orf211	grey	0.09055842
ENSG00000255389	C6orf3	grey	0.11781833
ENSG00000204439	C6orf47	turquoise	0.57505826
ENSG00000204387	C6orf48	grey	-0.3192435
ENSG00000137434	C6orf52	grey	-0.0183569
ENSG00000112308	C6orf62	blue	-0.5942978
ENSG00000198663	C6orf89	grey	0.17612871
ENSG00000203711	C6orf99	grey	0.18556281
ENSG00000244291	C7orf13	grey	-0.1134733
ENSG00000146576	C7orf26	turquoise	-0.5030147
ENSG00000153790	C7orf31	grey	0.06571732
ENSG00000165131	C7orf34	yellow	0.70191027
ENSG00000146826	C7orf43	blue	0.57039695
ENSG00000122783	C7orf49	yellow	0.71359373
ENSG00000146540	C7orf50	yellow	-0.3482781
ENSG00000164898	C7orf55	grey	-0.0319646
ENSG00000146963	C7orf55-LUC7L2	lightgreen	0.70042676
ENSG00000164746	C7orf57	grey	-0.1872365
ENSG00000164603	C7orf60	grey	0.16441283
ENSG00000185955	C7orf61	grey	0.2059993
ENSG00000105792	C7orf63	blue	0.56060154
ENSG00000243317	C7orf73	brown	-0.5954339
ENSG00000177335	C8orf31	grey	0.21433716
ENSG00000182307	C8orf33	turquoise	0.71921931
ENSG00000165084	C8orf34	darkmagenta	0.63934538
ENSG00000156172	C8orf37	turquoise	0.4848656
ENSG00000176907	C8orf4	grey	0.31816814
ENSG00000213865	C8orf44	brown	0.45245388
ENSG00000169085	C8orf46	grey	-0.1923073
ENSG00000177459	C8orf47	brown	0.44784286
ENSG00000164743	C8orf48	grey	0.0168518
ENSG00000236939	C8orf56	grey	0.15792995
ENSG00000241852	C8orf58	turquoise	0.4701971
ENSG00000176731	C8orf59	grey	-0.0854991
ENSG00000189376	C8orf76	lightcyan	-0.5248288
ENSG00000213563	C8orf82	grey60	-0.4971133
ENSG00000179082	C9orf106	grey	0.05577848
ENSG00000198917	C9orf114	grey	0.04689416
ENSG00000160345	C9orf116	grey	-0.0968075
ENSG00000174038	C9orf131	grey	0.25774758
ENSG00000148362	C9orf142	grey	-0.0192474
ENSG00000136932	C9orf156	grey	-0.0685306
ENSG00000171159	C9orf16	turquoise	-0.4023519
ENSG00000232434	C9orf172	grey	0.01114141
ENSG00000148120	C9orf3	yellow	0.66375125
ENSG00000203993	C9orf37	grey	0.26999886
ENSG00000215066	C9orf38	grey	0.15955008

ENSG00000135045	C9orf40	grey	-0.2122754
ENSG00000156017	C9orf41	black	0.65890904
ENSG00000165118	C9orf64	turquoise	0.5367811
ENSG00000238227	C9orf69	yellow	0.35954745
ENSG00000147894	C9orf72	grey60	-0.4169503
ENSG00000136819	C9orf78	grey	-0.105274
ENSG00000165181	C9orf84	pink	0.52828978
ENSG00000155621	C9orf85	blue	-0.5211919
ENSG00000165233	C9orf89	turquoise	0.3558575
ENSG00000165698	C9orf9	grey	-0.1441492
ENSG00000157693	C9orf91	blue	0.56351119
ENSG00000198870	C9orf96	grey	0.01324251
ENSG00000063180	CA11	magenta	0.65992996
ENSG00000074410	CA12	grey60	0.62903823
ENSG00000185015	CA13	grey	0.26387026
ENSG00000118298	CA14	yellow	0.47788853
ENSG00000167434	CA4	grey	0.18647109
ENSG00000169239	CA5B	turquoise	0.35111734
ENSG00000168748	CA7	black	0.32972894
ENSG00000178538	CA8	turquoise	0.48842482
ENSG00000120159	CAAP1	turquoise	0.3534088
ENSG00000135932	CAB39	grey	0.04842303
ENSG00000102547	CAB39L	grey	-0.0889868
ENSG00000099991	CABIN1	turquoise	-0.5669926
ENSG00000134508	CABLES1	turquoise	0.66482464
ENSG00000149679	CABLES2	grey	0.03546404
ENSG00000157782	CABP1	turquoise	-0.4561076
ENSG00000100314	CABP7	grey	-0.0297739
ENSG00000154040	CABYR	grey	0.14706605
ENSG00000160325	CACFD1	blue	-0.4570635
ENSG00000158966	CACHD1	grey	0.24740803
ENSG00000141837	CACNA1A	grey	-0.0098868
ENSG00000148408	CACNA1B	grey60	0.66985748
ENSG00000151067	CACNA1C	grey60	0.66401502
ENSG00000256271	CACNA1C-AS2	grey	0.22533544
ENSG00000256025	CACNA1C-AS4	grey	0.09747563
ENSG00000256721	CACNA1C-IT3	grey	0.41591193
ENSG00000157388	CACNA1D	blue	0.76035562
ENSG00000198216	CACNA1E	turquoise	-0.533484
ENSG00000196557	CACNA1H	magenta	0.74930889
ENSG00000100346	CACNA1I	magenta	0.6947975
ENSG00000153956	CACNA2D1	green	-0.6082017
ENSG00000157445	CACNA2D3	blue	0.66090988
ENSG00000243715	CACNA2D3-AS1	grey	0.15729694
ENSG00000067191	CACNB1	blue	0.867308
ENSG00000165995	CACNB2	blue	0.63502324
ENSG00000167535	CACNB3	blue	0.54736471
ENSG00000182389	CACNB4	blue	0.58582042

ENSG00000006116	CACNG3	darkmagenta	0.77058876
ENSG00000075461	CACNG4	darkorange	0.74556628
ENSG00000075429	CACNG5	red	0.34711934
ENSG00000105605	CACNG7	salmon	-0.7703099
ENSG00000142408	CACNG8	blue	0.71325012
ENSG00000105298	CACTIN	grey	0.13177225
ENSG00000151893	CACUL1	yellow	-0.5667363
ENSG00000225928	CACYBPP1	grey	0.35469862
ENSG00000084774	CAD	grey	0.139338
ENSG00000182985	CADM1	turquoise	0.54192449
ENSG00000175161	CADM2	turquoise	0.58514014
ENSG00000239519	CADM2-AS1	grey	0.08206845
ENSG00000241648	CADM2-AS2	yellow	0.37891291
ENSG00000162706	CADM3	brown	0.61003598
ENSG00000105767	CADM4	brown	-0.6016402
ENSG00000163618	CADPS	blue	0.82546635
ENSG00000081803	CADPS2	grey	0.13317971
ENSG00000270419	CAHM	blue	-0.5396822
ENSG00000104327	CALB1	blue	0.60764287
ENSG00000172137	CALB2	blue	0.51190861
ENSG00000012822	CALCOCO1	turquoise	-0.4826869
ENSG00000136436	CALCOCO2	blue	-0.7752244
ENSG00000064989	CALCRL	grey	-0.0113836
ENSG00000122786	CALD1	brown	-0.5112213
ENSG00000138172	CALHM2	tan	0.49409609
ENSG00000198668	CALM1	blue	0.51242455
ENSG00000143933	CALM2	grey	-0.1344359
ENSG00000229097	CALM2P2	grey	0.09289419
ENSG00000160014	CALM3	blue	0.67277651
ENSG00000129007	CALML4	grey	-0.2216564
ENSG00000183166	CALN1	magenta	0.63010803
ENSG00000128595	CALU	brown	-0.6102337
ENSG00000130643	CALY	blue	0.8094047
ENSG00000134072	CAMK1	grey	0.09844017
ENSG00000183049	CAMK1D	magenta	0.64044463
ENSG00000070808	CAMK2A	darkorange	0.77513008
ENSG00000058404	CAMK2B	blue	0.66089351
ENSG00000145349	CAMK2D	magenta	-0.6391069
ENSG00000148660	CAMK2G	turquoise	0.6563554
ENSG00000162545	CAMK2N1	brown	-0.4922284
ENSG00000163888	CAMK2N2	grey	0.14345138
ENSG00000004660	CAMKK1	grey60	0.70975073
ENSG00000110931	CAMKK2	magenta	0.61855215
ENSG00000143919	CAMKMT	blue	0.32223058
ENSG00000164076	CAMKV	blue	0.60143503
ENSG00000164615	CAMLG	greenyellow	0.47861717
ENSG00000130559	CAMSAP1	brown	0.64421829
ENSG00000118200	CAMSAP2	grey	-0.3625011

ENSG00000076826	CAMSAP3	blue	0.66523913
ENSG00000171735	CAMTA1	grey60	0.75150237
ENSG00000108509	CAMTA2	darkorange	0.68612822
ENSG00000111530	CAND1	brown	0.5781686
ENSG00000144712	CAND2	blue	0.4434463
ENSG00000127022	CANX	turquoise	0.55927688
ENSG00000131236	CAP1	black	0.74634681
ENSG00000112186	CAP2	blue	0.87237191
ENSG00000259151	CAP2P1	grey	0.06548858
ENSG00000042493	CAPG	cyan	0.53491933
ENSG00000014216	CAPN1	yellow	-0.5774429
ENSG00000142330	CAPN10	yellow	-0.4113928
ENSG00000260942	CAPN10-AS1	grey	0.15492639
ENSG00000103326	CAPN15	grey	0.14632066
ENSG00000162909	CAPN2	turquoise	0.61256252
ENSG00000092529	CAPN3	yellow	0.7202261
ENSG00000149260	CAPN5	red	0.62602355
ENSG00000131375	CAPN7	grey	0.02512165
ENSG00000135773	CAPN9	grey	0.17792856
ENSG00000126247	CAPNS1	grey	-0.1232808
ENSG00000135387	CAPRIN1	grey	-0.0810941
ENSG00000110888	CAPRIN2	blue	0.65439866
ENSG00000180881	CAPS2	grey	0.19432964
ENSG00000116489	CAPZA1	turquoise	0.40359682
ENSG00000198898	CAPZA2	turquoise	0.56657221
ENSG00000077549	CAPZB	grey	-0.3229862
ENSG00000132357	CARD6	pink	0.57512016
ENSG00000105483	CARD8	blue	-0.6415509
ENSG00000138380	CARF	grey	0.17477153
ENSG00000153048	CARHSP1	black	-0.5144683
ENSG00000213995	CARKD	turquoise	0.51054328
ENSG00000142453	CARM1	blue	0.46379869
ENSG00000227835	CARM1P1	grey	-0.0389392
ENSG00000172508	CARNS1	yellow	0.83648522
ENSG00000134905	CARS2	green	-0.5882025
ENSG00000204682	CASC10	turquoise	0.4700991
ENSG00000260455	CASC14	grey	0.02705701
ENSG00000177640	CASC2	grey	-0.0273213
ENSG00000108349	CASC3	green	0.76659655
ENSG00000166734	CASC4	lightcyan	0.8444284
ENSG00000249395	CASC9	grey	0.31223409
ENSG00000127995	CASD1	blue	0.47502943
ENSG00000147044	CASK	yellow	-0.7050529
ENSG00000167971	CASKIN1	blue	0.47635051
ENSG00000177303	CASKIN2	blue	-0.6171366
ENSG00000003400	CASP10	tan	0.64948554
ENSG00000164305	CASP3	turquoise	0.67785581
ENSG00000138794	CASP6	grey	0.18006714

ENSG00000165806	CASP7	turquoise	0.52580689
ENSG00000064012	CASP8	grey	0.0306451
ENSG00000132906	CASP9	grey	0.21238563
ENSG00000143318	CASQ1	yellow	-0.4475784
ENSG00000118729	CASQ2	grey	0.15915898
ENSG00000153113	CAST	pink	0.48289312
ENSG00000121691	CAT	turquoise	0.60101157
ENSG00000166762	CATSPER2	grey	0.02077408
ENSG00000205771	CATSPER2P1	grey	0.00170335
ENSG00000105974	CAV1	brown	-0.5548792
ENSG00000105971	CAV2	brown	-0.3251037
ENSG00000078699	CBFA2T2	blue	0.60038479
ENSG00000129993	CBFA2T3	grey	-0.1328761
ENSG00000067955	CBFB	brown	-0.8161918
ENSG00000110395	CBL	turquoise	0.63757015
ENSG00000114423	CBLB	grey	0.02591689
ENSG00000105879	CBLL1	grey	-0.0501636
ENSG00000102924	CBLN1	grey	0.26369511
ENSG00000139899	CBLN3	grey	0.04345298
ENSG00000159228	CBR1	yellow	0.60996171
ENSG00000159231	CBR3	grey	-0.0078374
ENSG00000145439	CBR4	brown	-0.638343
ENSG00000160200	CBS	turquoise	0.71871646
ENSG00000172785	CBWD1	brown	0.40023372
ENSG00000136682	CBWD2	green	-0.3831236
ENSG00000196873	CBWD3	grey	-0.0890055
ENSG00000147996	CBWD5	turquoise	-0.6211579
ENSG00000204790	CBWD6	brown	0.49005088
ENSG00000108468	CBX1	lightgreen	0.63437306
ENSG00000230823	CBX1P1	grey	0.36633556
ENSG00000214322	CBX1P2	grey	0.16491856
ENSG00000122565	CBX3	mediumpurp	0.67371325
ENSG00000250045	CBX3P3	grey	-0.0197127
ENSG00000141582	CBX4	grey	0.0605513
ENSG00000094916	CBX5	turquoise	0.28225357
ENSG00000241535	CBX5P1	blue	-0.4156266
ENSG00000100307	CBX7	greenyellow	0.40077301
ENSG00000100211	CBY1	grey	-0.1830248
ENSG00000132024	CC2D1A	turquoise	-0.3740706
ENSG00000154222	CC2D1B	grey	0.1046526
ENSG00000048342	CC2D2A	grey	0.04744264
ENSG00000060339	CCAR1	green	0.69404667
ENSG00000158941	CCAR2	brown	0.42308818
ENSG00000171097	CCBL1	grey	-0.0181555
ENSG00000137944	CCBL2	grey	0.06527058
ENSG00000176476	CCDC101	grey	-0.1412168
ENSG00000150636	CCDC102B	grey	-0.2261351
ENSG00000173581	CCDC106	brown	0.60786798

ENSG00000005059	CCDC109B	grey	0.1497989
ENSG00000168491	CCDC110	grey	-0.1240679
ENSG00000164221	CCDC112	grey	-0.2384445
ENSG00000103021	CCDC113	red	0.65046528
ENSG00000136710	CCDC115	greenyellow	0.50829612
ENSG00000159873	CCDC117	grey	-0.1228225
ENSG00000160799	CCDC12	salmon	0.71085602
ENSG00000147144	CCDC120	grey	0.20301179
ENSG00000176714	CCDC121	turquoise	0.71190754
ENSG00000151773	CCDC122	grey	0.08263663
ENSG00000007080	CCDC124	grey	0.25286243
ENSG00000183323	CCDC125	grey	0.17404036
ENSG00000169193	CCDC126	grey	0.34403017
ENSG00000164366	CCDC127	turquoise	0.64682489
ENSG00000173811	CCDC13-AS1	grey	0.05620272
ENSG00000104957	CCDC130	brown	0.42636338
ENSG00000004766	CCDC132	turquoise	-0.4040896
ENSG00000100147	CCDC134	grey	0.36142588
ENSG00000185298	CCDC137	red	0.56751023
ENSG00000163006	CCDC138	grey	0.12506979
ENSG00000175455	CCDC14	grey	-0.321653
ENSG00000135637	CCDC142	grey	-0.0287197
ENSG00000170160	CCDC144A	grey	-0.0731111
ENSG00000154874	CCDC144B	grey	-0.2945825
ENSG00000154898	CCDC144CP	yellow	0.62540165
ENSG00000231233	CCDC147-AS1	grey	0.08378981
ENSG00000153237	CCDC148	turquoise	-0.3802585
ENSG00000227480	CCDC148-AS1	grey	0.21576922
ENSG00000181982	CCDC149	grey	-0.1319253
ENSG00000198003	CCDC151	grey	-0.0069255
ENSG00000198865	CCDC152	grey	-0.0056485
ENSG00000183401	CCDC159	turquoise	-0.401815
ENSG00000203952	CCDC160	turquoise	0.58251077
ENSG00000236624	CCDC163P	grey	-0.0174684
ENSG00000198937	CCDC167	grey	0.0001063
ENSG00000164989	CCDC171	grey	-0.0237436
ENSG00000154781	CCDC174	grey	0.08578686
ENSG00000119636	CCDC176	turquoise	-0.3749098
ENSG00000255994	CCDC177	grey	0.08544124
ENSG00000122483	CCDC18	grey	0.0754374
ENSG00000197816	CCDC180	blue	0.39417253
ENSG00000117477	CCDC181	grey	-0.1232336
ENSG00000213213	CCDC183	grey	0.24383442
ENSG00000228544	CCDC183-AS1	grey	0.06403318
ENSG00000101997	CCDC22	grey	-0.1665689
ENSG00000177868	CCDC23	grey	-0.2099585
ENSG00000159214	CCDC24	black	-0.6778052
ENSG00000147419	CCDC25	grey	0.06088378

ENSG00000024862	CCDC28A	brown	0.75191739
ENSG00000160050	CCDC28B	brown	0.59831275
ENSG00000151468	CCDC3	black	0.79058159
ENSG00000186409	CCDC30	brown	0.48821842
ENSG00000109881	CCDC34	turquoise	-0.7312547
ENSG00000145075	CCDC39	magenta	0.69176201
ENSG00000180329	CCDC43	grey	0.02838782
ENSG00000108588	CCDC47	salmon	0.53470684
ENSG00000152492	CCDC50	turquoise	0.67927961
ENSG00000164051	CCDC51	darkred	0.60196866
ENSG00000120860	CCDC53	grey	-0.3524326
ENSG00000176155	CCDC57	turquoise	-0.631699
ENSG00000160124	CCDC58	grey	-0.0507315
ENSG00000133773	CCDC59	grey	-0.1621557
ENSG00000108091	CCDC6	green	-0.5636891
ENSG00000104983	CCDC61	grey	0.15020208
ENSG00000130783	CCDC62	grey	-0.0845198
ENSG00000135127	CCDC64	turquoise	-0.5922093
ENSG00000139537	CCDC65	turquoise	-0.4735405
ENSG00000180376	CCDC66	grey	0.17987324
ENSG00000198624	CCDC69	grey	0.01748115
ENSG00000216937	CCDC7	green	0.60890776
ENSG00000177352	CCDC71	brown	-0.3981518
ENSG00000163040	CCDC74A	blue	0.5899196
ENSG00000162004	CCDC78	magenta	0.48557577
ENSG00000169515	CCDC8	turquoise	0.51227222
ENSG00000091986	CCDC80	turquoise	0.41642747
ENSG00000149201	CCDC81	grey	0.22941599
ENSG00000149231	CCDC82	bisque4	0.86301051
ENSG00000186166	CCDC84	turquoise	-0.4950428
ENSG00000055813	CCDC85A	purple	0.68367841
ENSG00000175602	CCDC85B	brown	0.79511405
ENSG00000205476	CCDC85C	grey	0.19716095
ENSG00000110104	CCDC86	grey	0.06337009
ENSG00000182791	CCDC87	grey	0.2125479
ENSG00000115355	CCDC88A	turquoise	0.55253618
ENSG00000015133	CCDC88C	magenta	0.71337648
ENSG00000105321	CCDC9	grey	0.20601952
ENSG00000137500	CCDC90B	grey	-0.1833667
ENSG00000123106	CCDC91	grey	-0.127011
ENSG00000119242	CCDC92	grey	-0.2090512
ENSG00000125633	CCDC93	grey	-0.1280507
ENSG00000105248	CCDC94	grey	0.18882467
ENSG00000173013	CCDC96	turquoise	-0.3373443
ENSG00000142039	CCDC97	brown	-0.6626317
ENSG00000262484	CCER2	grey	-0.0500179
ENSG00000204536	CCHCR1	green	-0.5279858
ENSG00000187094	CCK	blue	0.53576213

ENSG00000110148	CCKBR	blue	0.51141596
ENSG00000108691	CCL2	pink	0.54755714
ENSG00000161570	CCL5	grey	0.23437991
ENSG00000136280	CCM2	blue	0.70678797
ENSG00000101331	CCM2L	tan	0.64329864
ENSG00000133101	CCNA1	blue	0.81466111
ENSG00000145386	CCNA2	grey	0.25143678
ENSG00000134057	CCNB1	turquoise	-0.5042163
ENSG00000100814	CCNB1IP1	grey	-0.024042
ENSG00000157456	CCNB2	grey	0.21833399
ENSG00000112237	CCNC	grey	0.04289122
ENSG00000110092	CCND1	turquoise	0.4270282
ENSG00000118971	CCND2	grey	-0.2163639
ENSG00000256164	CCND2-AS1	green	-0.3789586
ENSG00000255920	CCND2-AS2	grey	0.16564191
ENSG00000112576	CCND3	blue	-0.3319495
ENSG00000166946	CCNDBP1	brown	0.74278655
ENSG00000105173	CCNE1	grey	0.18740281
ENSG00000175305	CCNE2	yellow	0.73462561
ENSG00000162063	CCNF	grey	0.28237755
ENSG00000113328	CCNG1	turquoise	0.61375373
ENSG00000138764	CCNG2	blue	-0.4531196
ENSG00000134480	CCNH	grey	-0.1270431
ENSG00000205089	CCNI2	yellow	0.37253145
ENSG00000107443	CCNJ	brown	-0.6952233
ENSG00000135083	CCNJL	grey	0.23144444
ENSG00000090061	CCNK	grey	-0.0462182
ENSG00000152669	CCNO	grey	-0.1813178
ENSG00000129315	CCNT1	brown	-0.5165494
ENSG00000082258	CCNT2	grey	-0.1271995
ENSG00000224043	CCNT2-AS1	brown	-0.4679651
ENSG00000108100	CCNY	greenyellow	0.49114169
ENSG00000163249	CCNYL1	grey	0.02021678
ENSG00000103540	CCP110	yellow	0.81553038
ENSG00000260916	CCPG1	grey	0.10647295
ENSG00000163823	CCR1	royalblue	0.81686691
ENSG00000151014	CCRN4L	blue	-0.6169753
ENSG00000173992	CCS	green	-0.4772125
ENSG00000154429	CCSAP	grey	-0.1399306
ENSG00000184305	CCSER1	turquoise	-0.5794238
ENSG00000107771	CCSER2	grey	0.00180775
ENSG00000225569	CCT4P2	grey	0.33060477
ENSG00000132141	CCT6B	grey	0.15269539
ENSG00000228409	CCT6P1	yellow	0.38630902
ENSG00000135624	CCT7	brown	0.7684414
ENSG00000156261	CCT8	brown	0.66275517
ENSG00000226015	CCT8P1	grey	0.20863331
ENSG00000122674	CCZ1	grey	0.26245857

ENSG00000146574	CCZ1B	grey	0.33512362
ENSG00000156535	CD109	turquoise	0.59570117
ENSG00000170458	CD14	royalblue	0.83888004
ENSG00000177697	CD151	grey	0.01811541
ENSG00000117281	CD160	grey	0.18751653
ENSG00000135535	CD164	grey60	-0.7156078
ENSG00000091972	CD200	purple	0.67521774
ENSG00000012124	CD22	yellow	0.69846774
ENSG00000150637	CD226	red	-0.4226918
ENSG00000174807	CD248	brown	-0.3930973
ENSG00000139193	CD27	grey	-0.0513427
ENSG00000215039	CD27-AS1	darkmagenta	0.65399756
ENSG00000120217	CD274	grey	-0.0433453
ENSG00000103855	CD276	grey	-0.164632
ENSG00000198087	CD2AP	darkred	-0.5907468
ENSG00000169217	CD2BP2	grey	-0.020369
ENSG00000167851	CD300A	royalblue	0.8182539
ENSG00000241399	CD302	turquoise	0.67866494
ENSG00000167775	CD320	tan	0.57610095
ENSG00000174059	CD34	tan	0.74761352
ENSG00000004468	CD38	turquoise	0.62390704
ENSG00000117877	CD3EAP	grey	0.07755927
ENSG00000010610	CD4	cyan	0.75607117
ENSG00000101017	CD40	grey	0.06849461
ENSG00000026508	CD44	grey	-0.1161337
ENSG00000117335	CD46	grey	0.0716732
ENSG00000196776	CD47	yellow	-0.2801266
ENSG00000169442	CD52	black	0.52333821
ENSG00000143119	CD53	royalblue	0.87838505
ENSG00000196352	CD55	yellow	0.60301779
ENSG00000116815	CD58	royalblue	0.44039108
ENSG00000085063	CD59	turquoise	0.56357307
ENSG00000135404	CD63	turquoise	0.53844199
ENSG00000019582	CD74	cyan	0.90230795
ENSG00000110651	CD81	grey	0.07758226
ENSG00000085117	CD82	yellow	0.3826668
ENSG00000112149	CD83	grey	0.18956724
ENSG00000066294	CD84	cyan	0.90392787
ENSG00000114013	CD86	cyan	0.72485663
ENSG00000153563	CD8A	grey	-0.2932313
ENSG00000010278	CD9	purple	-0.6376153
ENSG00000125810	CD93	pink	0.77511791
ENSG00000123146	CD97	yellow	0.60195915
ENSG00000002586	CD99	turquoise	0.37289416
ENSG00000102181	CD99L2	brown	0.78537627
ENSG00000158825	CDA	grey	0.29619136
ENSG00000102543	CDADC1	red	0.67725057
ENSG00000140326	CDAN1	grey	0.038448

ENSG00000151465	CDC123	darkred	0.4660302
ENSG00000079335	CDC14A	turquoise	0.58145529
ENSG00000081377	CDC14B	blue	-0.6080182
ENSG00000130177	CDC16	salmon	0.59193134
ENSG00000094880	CDC23	grey	0.31819194
ENSG00000004897	CDC27	grey	-0.072997
ENSG00000099804	CDC34	grey	0.17238796
ENSG00000105401	CDC37	turquoise	-0.5573163
ENSG00000106993	CDC37L1	grey	-0.0412817
ENSG00000168438	CDC40	turquoise	-0.5028099
ENSG00000070831	CDC42	grey	-0.1291199
ENSG00000230068	CDC42-IT1	grey	0.35588357
ENSG00000143776	CDC42BPA	lightgreen	0.45036827
ENSG00000198752	CDC42BPB	grey	0.17633161
ENSG00000128283	CDC42EP1	yellow	0.8472994
ENSG00000149798	CDC42EP2	yellow	0.78875503
ENSG00000163171	CDC42EP3	brown	0.43597245
ENSG00000179604	CDC42EP4	turquoise	0.869422
ENSG00000253971	CDC42P3	grey	0.1229804
ENSG00000197622	CDC42SE1	pink	0.77234678
ENSG00000158985	CDC42SE2	lightgreen	0.68257098
ENSG00000096401	CDC5L	grey	-0.0746702
ENSG00000094804	CDC6	green	-0.3604792
ENSG00000097046	CDC7	grey	0.21215152
ENSG00000134371	CDC73	brown	-0.7156496
ENSG00000164649	CDCA7L	purple	-0.6386773
ENSG00000134690	CDCA8	grey	0.11995218
ENSG00000039068	CDH1	yellow	0.53522431
ENSG00000040731	CDH10	red	0.71844585
ENSG00000140937	CDH11	grey	0.26740503
ENSG00000154162	CDH12	purple	0.84854534
ENSG00000140945	CDH13	blue	0.48502283
ENSG00000145526	CDH18	turquoise	-0.4930719
ENSG00000071991	CDH19	yellow	0.77633231
ENSG00000170558	CDH2	red	0.76706069
ENSG00000101542	CDH20	turquoise	0.755001
ENSG00000149654	CDH22	grey60	0.56148158
ENSG00000107736	CDH23	grey	0.16582419
ENSG00000139880	CDH24	grey	0.15809587
ENSG00000124215	CDH26	grey	0.12998905
ENSG00000062038	CDH3	grey	-0.0150424
ENSG00000179242	CDH4	purple	0.39221787
ENSG00000179776	CDH5	tan	0.87981176
ENSG00000113361	CDH6	grey60	0.6786922
ENSG00000081138	CDH7	grey60	0.55197715
ENSG00000150394	CDH8	blue	0.78314755
ENSG00000113100	CDH9	blue	0.65591438
ENSG00000148600	CDHR1	darkmagenta	0.81288692

ENSG00000074276	CDHR2	yellow	-0.4885724
ENSG00000128536	CDHR3	grey	0.03876736
ENSG00000089486	CDIP1	red	0.60770093
ENSG00000103502	CDIPT	brown	0.55980105
ENSG00000214725	CDIPT-AS1	grey	-0.0602128
ENSG00000185324	CDK10	brown	0.77948433
ENSG00000008128	CDK11A	grey	0.0528549
ENSG00000248333	CDK11B	grey	0.19840069
ENSG00000167258	CDK12	grey	-0.0408159
ENSG00000065883	CDK13	grey	-0.1241412
ENSG00000058091	CDK14	grey60	0.67628049
ENSG00000117266	CDK18	yellow	0.77532187
ENSG00000155111	CDK19	green	0.86696937
ENSG00000156345	CDK20	yellow	-0.6678412
ENSG00000111328	CDK2AP1	turquoise	0.82257736
ENSG00000234299	CDK2AP2P1	grey	0.02055864
ENSG00000237451	CDK2AP2P2	grey	-0.0557244
ENSG00000135446	CDK4	turquoise	0.77433193
ENSG00000164885	CDK5	blue	0.8033546
ENSG00000176749	CDK5R1	blue	0.36090689
ENSG00000171450	CDK5R2	darkorange	0.70758972
ENSG00000101391	CDK5RAP1	brown	0.44190062
ENSG00000136861	CDK5RAP2	yellow	0.69685243
ENSG00000105810	CDK6	green	0.89381001
ENSG00000134058	CDK7	green	-0.6738433
ENSG00000132964	CDK8	grey	-0.0574226
ENSG00000136807	CDK9	grey	-0.2866957
ENSG00000145996	CDKAL1	grey	0.16660225
ENSG00000100490	CDKL1	grey	0.00349154
ENSG00000138769	CDKL2	grey60	0.62361172
ENSG00000006837	CDKL3	grey	0.12301268
ENSG00000008086	CDKL5	grey60	0.72138578
ENSG00000124762	CDKN1A	pink	0.77777048
ENSG00000111276	CDKN1B	yellow	0.62609714
ENSG00000129757	CDKN1C	yellow	0.58745051
ENSG00000168564	CDKN2AIP	blue	-0.5066805
ENSG00000237190	CDKN2AIPNL	blue	0.68288326
ENSG00000147883	CDKN2B	brown	-0.4873665
ENSG00000123080	CDKN2C	brown	-0.6377941
ENSG00000100526	CDKN3	grey	0.31034353
ENSG00000185267	CDNF	grey	0.29796746
ENSG00000129596	CDO1	grey	-0.1113364
ENSG00000064309	CDON	grey	0.1262338
ENSG00000205643	CDPF1	grey	0.22363825
ENSG00000140743	CDR2	grey60	0.71275616
ENSG00000109089	CDR2L	yellow	0.61837904
ENSG00000239704	CDRT4	grey	0.12970786
ENSG00000163624	CDS1	brown	0.57233315

ENSG00000101290	CDS2	red	0.60403663
ENSG00000091527	CDV3	blue	-0.7880734
ENSG00000228411	CDY4P	grey	0.16813442
ENSG00000153046	CDYL	blue	-0.5245038
ENSG00000166446	CDYL2	grey	0.27221048
ENSG00000186567	CEACAM19	turquoise	-0.3924764
ENSG00000231561	CEACAMP5	grey	0.10758707
ENSG00000267296	CEBPA-AS1	grey	0.00407023
ENSG00000172216	CEBPB	pink	0.75197265
ENSG00000221869	CEBPD	pink	0.83372922
ENSG00000153879	CEBPG	pink	0.63473746
ENSG00000115816	CEBPZ	brown	-0.7364256
ENSG00000093072	CECR1	turquoise	0.46009361
ENSG00000099954	CECR2	grey	0.02876669
ENSG00000069998	CECR5	salmon	0.72503093
ENSG00000183307	CECR6	magenta	0.7379548
ENSG00000237438	CECR7	grey	0.07917833
ENSG00000215704	CELA2B	grey	0.3228201
ENSG00000149187	CELF1	magenta	0.45229519
ENSG00000048740	CELF2	darkorange	0.43660517
ENSG00000181800	CELF2-AS1	grey	-0.0190252
ENSG00000159409	CELF3	blue	0.67524189
ENSG00000101489	CELF4	blue	0.71461779
ENSG00000161082	CELF5	blue	0.58767089
ENSG00000143126	CELSR2	floralwhite	0.67798893
ENSG00000008300	CELSR3	blue	0.57480489
ENSG00000184524	CEND1	grey	0.1766463
ENSG00000125817	CENPB	green	0.82292592
ENSG00000177946	CENPBD1	grey	0.18876562
ENSG00000145241	CENPC	grey	-0.106128
ENSG00000138778	CENPE	grey	-0.0997629
ENSG00000151849	CENPJ	yellowgreen	-0.4956864
ENSG00000120334	CENPL	grey	0.48665987
ENSG00000166451	CENPN	grey	0.33842804
ENSG00000138092	CENPO	grey	0.13600161
ENSG00000188312	CENPP	turquoise	-0.3786349
ENSG00000031691	CENPQ	turquoise	0.39798765
ENSG00000102901	CENPT	turquoise	-0.4914967
ENSG00000166582	CENPV	grey	-0.0955728
ENSG00000223591	CENPVP1	blue	0.55314564
ENSG00000203760	CENPW	grey	0.24386371
ENSG00000116198	CEP104	green	0.66476107
ENSG00000154240	CEP112	red	0.52873707
ENSG00000168944	CEP120	red	-0.4818187
ENSG00000100629	CEP128	darkgrey	0.61251249
ENSG00000174799	CEP135	darkgrey	0.50979697
ENSG00000103995	CEP152	purple	-0.5826996
ENSG00000110274	CEP164	turquoise	-0.5947361

ENSG00000143702	CEP170	grey	-0.1845071
ENSG00000099814	CEP170B	blue	0.84244316
ENSG00000174007	CEP19	grey	0.04552103
ENSG00000101639	CEP192	green	0.51462485
ENSG00000126001	CEP250	turquoise	-0.3842082
ENSG00000198707	CEP290	lightgreen	0.57208054
ENSG00000135837	CEP350	blue	-0.6665784
ENSG00000106477	CEP41	brown	0.74574118
ENSG00000164118	CEP44	grey	0.15928914
ENSG00000166037	CEP57	grey	-0.263865
ENSG00000183137	CEP57L1	grey	-0.0565938
ENSG00000182923	CEP63	darkgrey	0.57563736
ENSG00000011523	CEP68	grey	0.06645478
ENSG00000112877	CEP72	grey	-0.0315812
ENSG00000101624	CEP76	grey	0.15389014
ENSG00000148019	CEP78	grey	-0.1631122
ENSG00000130695	CEP85	turquoise	-0.5786077
ENSG00000111860	CEP85L	turquoise	0.72791503
ENSG00000121289	CEP89	brown	0.43978753
ENSG00000258890	CEP95	grey	0.2289511
ENSG00000182504	CEP97	grey	0.06121245
ENSG00000134255	CEPT1	grey60	-0.5982478
ENSG00000167123	CERCAM	yellow	0.73703915
ENSG00000100422	CERK	grey	0.0551774
ENSG00000223802	CERS1	turquoise	0.62102095
ENSG00000143418	CERS2	yellow	0.73834026
ENSG00000090661	CERS4	red	-0.6239265
ENSG00000139624	CERS5	turquoise	-0.6364792
ENSG00000172292	CERS6	blue	0.51476057
ENSG00000172824	CES4A	turquoise	-0.4061054
ENSG00000153140	CETN3	blue	-0.3901386
ENSG00000224786	CETN4P	grey	0.07903893
ENSG00000153774	CFDP1	brown	0.54141374
ENSG00000000971	CFH	tan	0.74227313
ENSG00000172757	CFL1	yellow	-0.5365164
ENSG00000223820	CFL1P1	grey	0.18103279
ENSG00000213830	CFL1P5	grey	0.2948984
ENSG00000213877	CFL1P7	grey	0.18695396
ENSG00000165410	CFL2	yellow	0.75364441
ENSG00000003402	CFLAR	blue	-0.5152286
ENSG00000001626	CFTR	turquoise	-0.4881275
ENSG00000163320	CGGBP1	grey	-0.020684
ENSG00000143375	CGN	yellow	0.56592895
ENSG00000128849	CGNL1	turquoise	0.59707231
ENSG00000138028	CGREF1	blue	0.7983024
ENSG00000100532	CGRRF1	turquoise	0.56856142
ENSG00000143942	CHAC2	red	0.56355586
ENSG00000100399	CHADL	yellow	0.59709447

ENSG00000167670	CHAF1A	grey	-0.1056679
ENSG00000159259	CHAF1B	grey	-0.0727612
ENSG00000198824	CHAMP1	grey	-0.0007543
ENSG00000070748	CHAT	grey	0.15484941
ENSG00000172586	CHCHD1	brown	0.31895629
ENSG00000250479	CHCHD10	blue	0.32467802
ENSG00000106153	CHCHD2	green	-0.658757
ENSG00000270809	CHCHD2P11	grey	0.17521386
ENSG00000106554	CHCHD3	turquoise	-0.5385659
ENSG00000163528	CHCHD4	brown	0.57072826
ENSG00000236156	CHCHD4P3	grey	0.37228097
ENSG00000225222	CHCHD4P5	grey	0.47923117
ENSG00000125611	CHCHD5	grey	0.08959145
ENSG00000159685	CHCHD6	brown	0.79723893
ENSG00000170791	CHCHD7	greenyellow	0.49508463
ENSG00000153922	CHD1	lightgreen	0.62097275
ENSG00000131778	CHD1L	yellow	0.60691222
ENSG00000173575	CHD2	darkgrey	0.65824755
ENSG00000170004	CHD3	blue	0.63188153
ENSG00000111642	CHD4	darkgrey	0.35673782
ENSG00000124177	CHD6	grey	0.02124808
ENSG00000171316	CHD7	green	0.79684751
ENSG00000100888	CHD8	turquoise	-0.6284468
ENSG00000177200	CHD9	brown	-0.6231973
ENSG00000016391	CHDH	turquoise	0.76737979
ENSG00000085872	CHERP	grey	0.16676628
ENSG00000072609	CHFR	grey	0.27295857
ENSG00000100604	CHGA	blue	0.69028643
ENSG00000089199	CHGB	blue	0.61582407
ENSG00000133048	CHI3L1	pink	0.7340976
ENSG00000204116	CHIC1	grey	-0.2994508
ENSG00000109220	CHIC2	turquoise	0.5333539
ENSG00000177830	CHID1	yellow	-0.6535955
ENSG00000110721	CHKA	salmon	0.52317125
ENSG00000100288	CHKB	blue	0.36341821
ENSG00000205559	CHKB-AS1	grey	0.31353597
ENSG00000134121	CHL1	black	-0.6701969
ENSG00000234661	CHL1-AS1	grey	0.14829854
ENSG00000224318	CHL1-AS2	grey	0.18320157
ENSG00000188419	CHM	blue	0.74153054
ENSG00000203668	CHML	yellow	-0.5349613
ENSG00000131165	CHMP1A	blue	0.32406477
ENSG00000255112	CHMP1B	grey	0.04189519
ENSG00000130724	CHMP2A	grey	-0.4602812
ENSG00000083937	CHMP2B	mediumpurp	0.70289691
ENSG00000115561	CHMP3	blue	0.38622928
ENSG00000254505	CHMP4A	grey	0.11875453
ENSG00000101421	CHMP4B	turquoise	-0.3692651

ENSG00000258469	CHMP4BP1	grey	0.39565286
ENSG00000086065	CHMP5	salmon	0.57799637
ENSG00000176108	CHMP6	grey	0.16538969
ENSG00000147457	CHMP7	brown	0.71822245
ENSG00000128656	CHN1	grey	-0.2164736
ENSG00000106069	CHN2	grey	-0.1790398
ENSG00000154645	CHODL	grey	0.01399382
ENSG00000187446	CHP1	red	0.55932109
ENSG00000123989	CHPF	grey	0.30599679
ENSG00000033100	CHPF2	turquoise	-0.3669457
ENSG00000111666	CHPT1	turquoise	0.77124682
ENSG00000104472	CHRAC1	yellow	0.62997309
ENSG00000101938	CHRDL1	turquoise	0.60716981
ENSG00000168539	CHRM1	magenta	0.82247625
ENSG00000133019	CHRM3	grey60	0.75352616
ENSG00000234601	CHRM3-AS1	grey	0.43200518
ENSG00000180720	CHRM4	black	0.66602428
ENSG00000080644	CHRNA3	grey	0.04331482
ENSG00000175344	CHRNA7	grey	0.12040713
ENSG00000170175	CHRNB1	turquoise	0.57804858
ENSG00000160716	CHRNB2	blue	0.71179151
ENSG00000175264	CHST1	darkmagenta	-0.7210745
ENSG00000115526	CHST10	darkred	0.60366965
ENSG00000136213	CHST12	grey	0.17059646
ENSG00000169105	CHST14	turquoise	0.64813975
ENSG00000182022	CHST15	turquoise	-0.3849946
ENSG00000175040	CHST2	grey	-0.2213713
ENSG00000122863	CHST3	blue	-0.7162725
ENSG00000183196	CHST6	yellow	0.51262488
ENSG00000147119	CHST7	turquoise	0.63961071
ENSG00000154080	CHST9	grey	-0.1074864
ENSG00000131873	CHSY1	blue	-0.8243547
ENSG00000198108	CHSY3	turquoise	-0.5104914
ENSG00000168802	CHTF8	grey	-0.0886862
ENSG00000160679	CHTOP	grey	-0.3047094
ENSG00000213341	CHUK	grey	0.31051552
ENSG00000258289	CHURC1	turquoise	0.43835543
ENSG00000144021	CIAO1	grey	0.24999775
ENSG00000005194	CIAPIN1	brown	0.66465037
ENSG00000263846	CIAPIN1P	grey	0.22172087
ENSG00000185043	CIB1	grey	0.05274851
ENSG00000136425	CIB2	red	0.46753682
ENSG00000079432	CIC	turquoise	-0.4512027
ENSG00000271529	CICP14	grey	0.02106949
ENSG00000233750	CICP27	grey	-0.0132424
ENSG00000186162	CIDECP	grey	0.08747039
ENSG00000179583	CIITA	royalblue	0.70175577
ENSG00000100865	CINP	blue	0.37052668

ENSG00000198894	CIPC	brown	-0.8219008
ENSG00000138433	CIR1	grey	-0.2436577
ENSG00000141076	CIRH1A	grey	-0.025318
ENSG00000122873	CISD1	green	-0.674599
ENSG00000145354	CISD2	grey	-0.0579572
ENSG00000230055	CISD3	greenyellow	0.61062544
ENSG00000122966	CIT	darkorange	0.73330261
ENSG00000125931	CITED1	yellow	-0.2941172
ENSG00000164442	CITED2	grey	0.19458609
ENSG00000179862	CITED4	grey	0.04498273
ENSG00000148337	CIZ1	salmon	-0.6402628
ENSG00000136108	CKAP2	red	0.53616095
ENSG00000136026	CKAP4	turquoise	0.52780367
ENSG00000175216	CKAP5	brown	0.38037256
ENSG00000166165	CKB	salmon	0.59163141
ENSG00000217555	CKLF	royalblue	0.42719182
ENSG00000223572	CKMT1A	yellow	-0.5985702
ENSG00000237289	CKMT1B	turquoise	-0.3668054
ENSG00000131730	CKMT2	blue	-0.5300664
ENSG00000247572	CKMT2-AS1	grey	-0.0395609
ENSG00000173207	CKS1B	salmon	0.64220214
ENSG00000250972	CKS1BP5	grey	0.3310136
ENSG00000123975	CKS2	grey	0.09953943
ENSG00000074054	CLASP1	grey	0.15990171
ENSG00000163539	CLASP2	brown	0.65975645
ENSG00000104859	CLASRP	yellow	0.3910568
ENSG00000016602	CLCA4	yellow	0.73748099
ENSG00000121940	CLCC1	brown	-0.6270189
ENSG00000114859	CLCN2	grey	0.05110518
ENSG00000109572	CLCN3	turquoise	0.69128679
ENSG00000073464	CLCN4	turquoise	-0.31395
ENSG00000171365	CLCN5	red	0.58559446
ENSG00000011021	CLCN6	blue	0.66536464
ENSG00000103249	CLCN7	brown	0.48221491
ENSG00000232305	CLCP1	grey	0.48972506
ENSG00000134873	CLDN10	turquoise	0.7138124
ENSG00000013297	CLDN11	yellow	0.74966612
ENSG00000106404	CLDN15	grey	0.01873177
ENSG00000171217	CLDN20	grey	0.28113436
ENSG00000184113	CLDN5	tan	0.8412895
ENSG00000213937	CLDN9	grey	-0.0618998
ENSG00000080822	CLDND1	yellow	0.80758601
ENSG00000105472	CLEC11A	yellow	-0.4599179
ENSG00000176435	CLEC14A	tan	0.70305902
ENSG00000038532	CLEC16A	lightgreen	-0.5090309
ENSG00000069493	CLEC2D	grey	-0.1685531
ENSG00000163815	CLEC3B	grey	0.17728365
ENSG00000111729	CLEC4A	grey	0.08321224

ENSG00000172243	CLEC7A	cyan	0.75445587
ENSG00000197992	CLEC9A	cyan	0.6356951
ENSG00000153132	CLGN	blue	0.43893021
ENSG00000162994	CLHC1	turquoise	0.51091134
ENSG00000213719	CLIC1	royalblue	0.72286538
ENSG00000155962	CLIC2	grey	0.05492466
ENSG00000169504	CLIC4	yellow	0.72232429
ENSG00000112782	CLIC5	tan	0.63234223
ENSG00000113282	CLINT1	grey	-0.1421423
ENSG00000130779	CLIP1	grey	0.21705806
ENSG00000106665	CLIP2	darkred	-0.7643178
ENSG00000105270	CLIP3	blue	0.65221124
ENSG00000115295	CLIP4	blue	-0.2609833
ENSG00000013441	CLK1	yellow	0.49304981
ENSG00000176444	CLK2	yellow	0.3266309
ENSG00000179335	CLK3	grey	0.26732576
ENSG00000113240	CLK4	lightgreen	0.69990053
ENSG00000165959	CLMN	yellow	0.63350858
ENSG00000166250	CLMP	grey	0.10717447
ENSG00000188603	CLN3	grey	0.17356817
ENSG00000102805	CLN5	blue	-0.4708667
ENSG00000128973	CLN6	grey	0.10843118
ENSG00000182372	CLN8	grey	0.23023584
ENSG00000074201	CLNS1A	greenyellow	0.40454153
ENSG00000134852	CLOCK	grey	0.15415308
ENSG00000172409	CLP1	grey	0.26913503
ENSG00000162129	CLPB	grey	0.24764634
ENSG00000125656	CLPP	grey	0.19618624
ENSG00000104853	CLPTM1	grey	0.32576157
ENSG00000049656	CLPTM1L	lightcyan	-0.7138996
ENSG00000166855	CLPX	grey	0.17042925
ENSG00000171603	CLSTN1	brown	0.64351518
ENSG00000158258	CLSTN2	grey	-0.1038617
ENSG00000139182	CLSTN3	blue	0.77505082
ENSG00000122705	CLTA	brown	0.77425272
ENSG00000175416	CLTB	brown	0.84580502
ENSG00000141367	CLTC	brown	0.87170336
ENSG00000070371	CLTCL1	brown	0.33687964
ENSG00000120885	CLU	turquoise	0.53429215
ENSG00000103351	CLUAP1	yellow	-0.5748357
ENSG00000132361	CLUH	grey	0.1440293
ENSG00000131797	CLUHP3	turquoise	-0.4891803
ENSG00000079101	CLUL1	grey	0.12071099
ENSG00000177182	CLVS1	blue	0.70373561
ENSG00000146352	CLVS2	grey	-0.1113519
ENSG00000125246	CLYBL	grey	-0.0257514
ENSG00000227659	CLYBL-AS2	grey	0.26105613
ENSG00000168405	CMAHP	tan	0.59026434

ENSG00000111726	CMAS	yellow	-0.6177706
ENSG00000164237	CMBL	grey	0.01539851
ENSG00000187118	CMC1	green	-0.5836999
ENSG00000103121	CMC2	greenyellow	0.53282993
ENSG00000153815	CMIP	purple	0.67481433
ENSG00000174600	CMKLR1	cyan	0.66913179
ENSG00000162368	CMPK1	grey	-0.0980958
ENSG00000134326	CMPK2	grey	-0.1679658
ENSG00000184220	CMSS1	brown	0.75958831
ENSG00000089505	CMTM1	brown	0.39358871
ENSG00000140932	CMTM2	grey	0.06654013
ENSG00000140931	CMTM3	grey	-0.102662
ENSG00000183723	CMTM4	turquoise	0.68724383
ENSG00000166091	CMTM5	yellow	0.78214883
ENSG00000091317	CMTM6	grey	0.10330278
ENSG00000170293	CMTM8	pink	0.47509478
ENSG00000137200	CMTR1	blue	0.45409928
ENSG00000180917	CMTR2	turquoise	0.48169024
ENSG00000164309	CMYA5	grey	-0.1227656
ENSG00000169714	CNBP	turquoise	0.63677706
ENSG00000150656	CNDP1	yellow	0.8057725
ENSG00000133313	CNDP2	grey	0.14939856
ENSG00000205423	CNEP1R1	grey	0.18418143
ENSG00000105427	CNFN	grey	-0.2315149
ENSG00000100528	CNIH1	turquoise	0.45609862
ENSG00000174871	CNIH2	blue	0.63066314
ENSG00000143786	CNIH3	blue	0.51101113
ENSG00000143771	CNIH4	grey	-0.021747
ENSG00000142675	CNKSR1	grey	-0.1215014
ENSG00000153721	CNKSR3	purple	-0.4842566
ENSG00000213355	CNN2P8	grey	0.03482879
ENSG00000117519	CNN3	turquoise	0.71546749
ENSG00000119946	CNNM1	grey60	0.68916487
ENSG00000148842	CNNM2	blue	0.72400941
ENSG00000168763	CNNM3	yellow	0.54646692
ENSG00000158158	CNNM4	grey	-0.082878
ENSG00000125107	CNOT1	grey	0.0837888
ENSG00000182973	CNOT10	turquoise	-0.4505319
ENSG00000158435	CNOT11	blue	-0.3679981
ENSG00000111596	CNOT2	grey	-0.0069229
ENSG00000088038	CNOT3	grey	-0.3255546
ENSG00000080802	CNOT4	lightcyan	0.6737716
ENSG00000113300	CNOT6	blue	-0.6421169
ENSG00000138767	CNOT6L	brown	-0.6895826
ENSG00000198791	CNOT7	greenyellow	0.465106
ENSG00000155508	CNOT8	tan	0.70903997
ENSG00000173786	CNP	yellow	0.88711886
ENSG00000115649	CNPPD1	blue	-0.532424

ENSG00000257727	CNPY2	turquoise	-0.4617175
ENSG00000137161	CNPY3	royalblue	0.6452423
ENSG00000166997	CNPY4	grey	-0.0716511
ENSG00000118432	CNR1	magenta	0.74654871
ENSG00000119865	CNRIP1	black	-0.6824406
ENSG00000162852	CNST	turquoise	-0.736998
ENSG00000176563	CNTD1	grey	0.17520691
ENSG00000242689	CNTF	green	0.5194259
ENSG00000122756	CNTFR	turquoise	0.74156025
ENSG00000237159	CNTFR-AS1	turquoise	0.4964054
ENSG00000044459	CNTLN	grey	-0.0673027
ENSG00000018236	CNTN1	grey	-0.0380506
ENSG00000184144	CNTN2	yellow	0.79905588
ENSG00000113805	CNTN3	purple	0.67996028
ENSG00000144619	CNTN4	grey	0.12230295
ENSG00000227588	CNTN4-AS2	grey	0.37364239
ENSG00000149972	CNTN5	blue	0.47950468
ENSG00000108797	CNTNAP1	blue	0.69175046
ENSG00000174469	CNTNAP2	grey	-0.1161404
ENSG00000106714	CNTNAP3	purple	0.60604455
ENSG00000154529	CNTNAP3B	grey	-0.0223997
ENSG00000152910	CNTNAP4	darkred	-0.5849021
ENSG00000155052	CNTNAP5	purple	0.87093723
ENSG00000119397	CNTRL	red	-0.5167066
ENSG00000170037	CNTROB	grey	0.03963933
ENSG00000106603	COA1	brown	0.73955519
ENSG00000183978	COA3	brown	0.41514667
ENSG00000183513	COA5	turquoise	0.7639831
ENSG00000168275	COA6	grey	-0.2635756
ENSG00000068120	COASY	bisque4	-0.8113022
ENSG00000106078	COBL	green	0.76620217
ENSG00000082438	COBLL1	tan	0.74798685
ENSG00000166685	COG1	blue	0.75146081
ENSG00000135775	COG2	grey	0.24840012
ENSG00000136152	COG3	grey	0.0742284
ENSG00000103051	COG4	grey	0.14935998
ENSG00000164597	COG5	grey	0.28389285
ENSG00000133103	COG6	grey	-0.1046287
ENSG00000168434	COG7	grey	-0.0278509
ENSG00000213380	COG8	grey	0.27096458
ENSG00000121058	COIL	brown	0.45078704
ENSG00000123500	COL10A1	yellow	0.52485567
ENSG00000204248	COL11A2	grey	-0.0394757
ENSG00000111799	COL12A1	grey	-0.0619632
ENSG00000084636	COL16A1	grey	-0.4863823
ENSG00000065618	COL17A1	grey	-0.0963483
ENSG00000182871	COL18A1	grey	-0.0293539
ENSG00000082293	COL19A1	grey	0.0153792

ENSG00000108821	COL1A1	grey	0.00620367
ENSG00000164692	COL1A2	grey	0.07196888
ENSG00000171502	COL24A1	grey	-0.0096393
ENSG00000188517	COL25A1	black	0.62407315
ENSG00000160963	COL26A1	blue	0.47634597
ENSG00000215018	COL28A1	grey	0.29036487
ENSG00000187498	COL4A1	pink	0.7561239
ENSG00000134871	COL4A2	pink	0.72546416
ENSG00000169031	COL4A3	brown	-0.3891849
ENSG00000113163	COL4A3BP	lightcyan	-0.7251424
ENSG00000188153	COL4A5	yellow	0.87853408
ENSG00000204262	COL5A2	grey	0.17240465
ENSG00000080573	COL5A3	grey	-0.0240524
ENSG00000142156	COL6A1	grey	0.07639906
ENSG00000163359	COL6A3	grey	0.03910118
ENSG00000206384	COL6A6	grey	0.50271483
ENSG00000114270	COL7A1	grey	-0.1115676
ENSG00000049089	COL9A2	yellow	0.71517474
ENSG00000092758	COL9A3	yellow	0.69656169
ENSG00000196167	COLCA1	brown	-0.4895658
ENSG00000214290	COLCA2	turquoise	0.69224382
ENSG00000158270	COLEC12	grey	0.00814321
ENSG00000130309	COLGALT1	royalblue	0.65863818
ENSG00000198756	COLGALT2	yellow	0.63707597
ENSG00000206561	COLQ	grey	-0.0886548
ENSG00000173163	COMMD1	brown	0.63837278
ENSG00000145781	COMMD10	grey	0.07089036
ENSG00000114744	COMMD2	grey	-0.1907136
ENSG00000148444	COMMD3	brown	0.4926165
ENSG00000140365	COMMD4	brown	0.68411855
ENSG00000170619	COMMD5	grey	0.06433379
ENSG00000188243	COMMD6	greenyellow	0.70880509
ENSG00000149600	COMMD7	turquoise	-0.4597115
ENSG00000169019	COMMD8	turquoise	0.51344291
ENSG00000110442	COMMD9	lightgreen	-0.5954193
ENSG00000093010	COMT	turquoise	0.75219247
ENSG00000165644	COMTD1	brown	0.40465739
ENSG00000122218	COPA	turquoise	-0.4332113
ENSG00000129083	COPB1	grey	0.06121546
ENSG00000184432	COPB2	turquoise	0.33714219
ENSG00000105669	COPE	grey	0.1465318
ENSG00000181789	COPG1	brown	0.7282365
ENSG00000158623	COPG2	grey	-0.0686694
ENSG00000172301	COPRS	brown	0.68477986
ENSG00000141030	COPS3	turquoise	-0.6058649
ENSG00000138663	COPS4	green	-0.8303545
ENSG00000121022	COPS5	green	-0.4891558
ENSG00000168090	COPS6	yellow	-0.6447426

ENSG00000111652	COPS7A	red	0.60212587
ENSG00000144524	COPS7B	blue	0.46463857
ENSG00000198612	COPS8	lightgreen	-0.6733073
ENSG00000111481	COPZ1	grey	0.0402971
ENSG00000005243	COPZ2	grey	-0.0757317
ENSG00000115520	COQ10B	darkred	0.58903201
ENSG00000251552	COQ10BP2	grey	0.15657516
ENSG00000173085	COQ2	grey	-0.0179636
ENSG00000132423	COQ3	brown	0.71510849
ENSG00000167113	COQ4	blue	0.49156313
ENSG00000110871	COQ5	grey	0.07929991
ENSG00000119723	COQ6	brown	0.58290249
ENSG00000167186	COQ7	grey	-0.019427
ENSG00000088682	COQ9	grey	-0.0079036
ENSG00000102879	CORO1A	cyan	0.67460106
ENSG00000172725	CORO1B	grey	0.12891963
ENSG00000110880	CORO1C	lightgreen	0.7162882
ENSG00000106789	CORO2A	grey	0.19085963
ENSG00000103647	CORO2B	grey	0.05814795
ENSG00000167549	CORO6	blue	0.70166421
ENSG00000262246	CORO7	lightcyan	-0.604009
ENSG00000241563	CORT	magenta	0.49538762
ENSG00000103187	COTL1	royalblue	0.42381316
ENSG00000006695	COX10	grey	0.302702
ENSG00000236088	COX10-AS1	blue	0.37387951
ENSG00000166260	COX11	brown	0.61054489
ENSG00000178449	COX14	brown	0.69458456
ENSG00000014919	COX15	grey	0.18772922
ENSG00000133983	COX16	red	0.57634796
ENSG00000138495	COX17	turquoise	-0.5119118
ENSG00000163626	COX18	grey	0.1799277
ENSG00000240230	COX19	grey	-0.036376
ENSG00000203667	COX20	grey	-0.1474609
ENSG00000131143	COX4I1	brown	0.77965004
ENSG00000131055	COX4I2	grey	0.10930543
ENSG00000178741	COX5A	brown	0.46349333
ENSG00000135940	COX5B	brown	0.59839561
ENSG00000213111	COX5BP2	grey	0.23307678
ENSG00000235967	COX5BP3	grey	0.23264378
ENSG00000237082	COX5BP6	yellow	0.52608004
ENSG00000111775	COX6A1	brown	0.50465763
ENSG00000226976	COX6A1P2	yellow	-0.364114
ENSG00000126267	COX6B1	yellow	-0.6317825
ENSG00000249047	COX6B1P5	grey	0.35176268
ENSG00000164919	COX6C	brown	0.67444815
ENSG00000260318	COX6CP1	grey	0.39470701
ENSG00000238008	COX6CP10	yellow	0.40505854
ENSG00000234144	COX6CP13	grey	0.10757486

ENSG00000228092	COX6CP15	grey	0.38355181
ENSG00000235141	COX6CP17	grey	0.24420697
ENSG00000161281	COX7A1	grey	0.00461724
ENSG00000112695	COX7A2	brown	0.80526004
ENSG00000258626	COX7A2P1	grey	0.50405584
ENSG00000131174	COX7B	yellow	-0.6657773
ENSG00000127184	COX7C	greenyellow	0.814509
ENSG00000235957	COX7CP1	grey	0.36418803
ENSG00000176340	COX8A	red	0.67751142
ENSG00000047457	CP	pink	0.70224519
ENSG00000080618	CPB2	yellow	0.72810035
ENSG00000235903	CPB2-AS1	yellow	0.70507139
ENSG00000108582	CPD	yellowgreen	-0.7246288
ENSG00000109472	CPE	turquoise	0.67739515
ENSG00000214575	CPEB1	turquoise	-0.4734676
ENSG00000137449	CPEB2	grey	-0.1076989
ENSG00000107864	CPEB3	yellow	-0.5342396
ENSG00000113742	CPEB4	turquoise	0.59516367
ENSG00000106034	CPED1	cyan	0.62275529
ENSG00000168993	CPLX1	blue	0.44129103
ENSG00000145920	CPLX2	grey	-0.2793529
ENSG00000214078	CPNE1	brown	0.47753787
ENSG00000140848	CPNE2	darkred	-0.4823823
ENSG00000085719	CPNE3	turquoise	0.88854759
ENSG00000196353	CPNE4	blue	0.51895746
ENSG00000124772	CPNE5	magenta	0.52235596
ENSG00000100884	CPNE6	blue	0.58143523
ENSG00000178773	CPNE7	blue	0.39464986
ENSG00000139117	CPNE8	pink	0.49945879
ENSG00000144550	CPNE9	grey60	0.49961153
ENSG00000144410	CPO	turquoise	-0.5136089
ENSG00000080819	CPOX	yellow	0.59086062
ENSG00000103381	CPPED1	green	0.66422502
ENSG00000021826	CPS1	grey	-0.0205428
ENSG00000071894	CPSF1	grey	-0.2264932
ENSG00000214076	CPSF1P1	grey	0.03212598
ENSG00000165934	CPSF2	blue	-0.6200059
ENSG00000119203	CPSF3	grey60	0.51664244
ENSG00000127054	CPSF3L	brown	0.54919098
ENSG00000160917	CPSF4	grey	-0.2050747
ENSG00000111605	CPSF6	grey	-0.0179043
ENSG00000149532	CPSF7	grey	0.13497014
ENSG00000110090	CPT1A	turquoise	0.71899596
ENSG00000169169	CPT1C	blue	0.79032778
ENSG00000157184	CPT2	turquoise	0.46149051
ENSG00000106066	CPVL	grey	-0.4196762
ENSG00000143320	CRABP2	grey	0.01801082
ENSG00000169372	CRADD	blue	-0.4463664

ENSG00000007545	CRAMP1L	grey	0.22264047
ENSG00000095321	CRAT	darkmagenta	-0.6851827
ENSG00000134376	CRB1	turquoise	0.51662594
ENSG00000148204	CRB2	darkorange	-0.5945135
ENSG00000113851	CRBN	grey	0.13312484
ENSG00000118260	CREB1	turquoise	0.73396448
ENSG00000107175	CREB3	grey	0.25977278
ENSG00000182158	CREB3L2	yellow	0.57919049
ENSG00000146592	CREB5	yellow	0.86760144
ENSG00000005339	CREBBP	grey	0.13344766
ENSG00000111269	CREBL2	grey	-0.2359325
ENSG00000164463	CREBRF	brown	-0.6546527
ENSG00000137504	CREBZF	bisque4	0.74229237
ENSG00000175874	CREG2	grey	-0.0970747
ENSG00000095794	CREM	grey	-0.0669736
ENSG00000145708	CRHBP	grey	-0.2675897
ENSG00000204650	CRHR1-IT1	green	-0.4736386
ENSG00000106113	CRHR2	grey	0.04531505
ENSG00000150938	CRIM1	grey	0.13790852
ENSG00000182809	CRIP2	grey	-0.261585
ENSG00000146215	CRIP3	grey	-0.1641743
ENSG00000179979	CRIPAK	grey	0.04527813
ENSG00000119878	CRIPT	grey	-0.1279812
ENSG00000121005	CRISPLD1	pink	0.49088256
ENSG00000103196	CRISPLD2	pink	0.65610039
ENSG00000167193	CRK	blue	-0.740712
ENSG00000099942	CRKL	turquoise	0.70686121
ENSG00000006016	CRLF1	black	-0.5514628
ENSG00000176390	CRLF3	grey	-0.0627722
ENSG00000088766	CRLS1	grey	-0.0030551
ENSG00000072832	CRMP1	yellow	-0.7941661
ENSG00000245694	CRNDE	grey	-0.0748902
ENSG00000101343	CRNKL1	yellow	0.36177875
ENSG00000058453	CROCC	grey	-0.2062128
ENSG00000215908	CROCCP2	grey	-0.0583169
ENSG00000080947	CROCCP3	grey	-0.2173619
ENSG00000005469	CROT	turquoise	0.30445908
ENSG00000095713	CRTAC1	blue	0.61749612
ENSG00000170275	CRTAP	green	0.73786938
ENSG00000105662	CRTC1	darkorange	0.37975263
ENSG00000160741	CRTC2	yellow	0.5846937
ENSG00000008405	CRY1	blue	-0.4102051
ENSG00000121671	CRY2	grey	0.19973744
ENSG00000109846	CRYAB	yellow	0.67204558
ENSG00000108255	CRYBA1	grey	0.39542217
ENSG00000100058	CRYBB2P1	grey	0.1167731
ENSG00000080200	CRYBG3	grey	0.01325771
ENSG00000213139	CRYGS	grey	0.19001137

ENSG00000165475	CRYL1	turquoise	0.53444124
ENSG00000103316	CRYM	purple	0.42147939
ENSG00000116791	CRYZ	grey	0.07040601
ENSG00000205758	CRYZL1	grey	0.08962362
ENSG00000233025	CRYZP1	grey	0.22124951
ENSG00000062485	CS	lightgreen	-0.6259678
ENSG00000139631	CSAD	grey	-0.0412141
ENSG00000172346	CSDC2	grey	0.1945968
ENSG00000009307	CSDE1	turquoise	0.44650369
ENSG00000184371	CSF1	pink	0.80334325
ENSG00000182578	CSF1R	cyan	0.95071284
ENSG00000198223	CSF2RA	cyan	0.72356429
ENSG00000119535	CSF3R	royalblue	0.7434011
ENSG00000147408	CSGALNACT1	turquoise	0.75358585
ENSG00000169826	CSGALNACT2	lightgreen	0.66906291
ENSG00000103653	CSK	grey	0.07195415
ENSG00000164796	CSMD3	purple	0.88416146
ENSG00000113712	CSNK1A1	lightgreen	-0.5392457
ENSG00000213923	CSNK1E	brown	-0.4288204
ENSG00000169118	CSNK1G1	brown	0.61695528
ENSG00000133275	CSNK1G2	grey	-0.0815767
ENSG00000151292	CSNK1G3	red	0.47049354
ENSG00000101266	CSNK2A1	grey	-0.0806514
ENSG00000254598	CSNK2A3	grey	0.47110352
ENSG00000204435	CSNK2B	brown	0.70390155
ENSG00000259295	CSPG4P12	lightcyan	0.57295847
ENSG00000114646	CSPG5	yellowgreen	0.75493488
ENSG00000144655	CSRNP1	pink	0.63211491
ENSG00000110925	CSRNP2	blue	0.57959194
ENSG00000178662	CSRNP3	blue	0.75425784
ENSG00000175183	CSRP2	grey	0.05790765
ENSG00000149474	CSRP2BP	grey	0.08650777
ENSG00000101439	CST3	turquoise	0.66585909
ENSG00000077984	CST7	grey	0.29271411
ENSG00000160213	CSTB	grey	-0.1884283
ENSG00000101138	CSTF1	grey	-0.0800826
ENSG00000101811	CSTF2	grey	0.06147473
ENSG00000177613	CSTF2T	grey	-0.2270832
ENSG00000232464	CTA-125H2.1	grey	0.36496251
ENSG00000225670	CTA-134P22.2	turquoise	-0.3438472
ENSG00000261353	CTA-14H9.5	red	0.54723472
ENSG00000259891	CTA-204B4.2	lightgreen	0.67187679
ENSG00000244625	CTA-211A9.5	grey	-0.1595931
ENSG00000226328	CTA-217C2.1	grey	-0.031366
ENSG00000203280	CTA-221G9.11	grey	-0.0068662
ENSG00000230637	CTA-246H3.8	grey	0.22624948
ENSG00000231084	CTA-253N17.1	grey	0.33523958
ENSG00000261462	CTA-254O6.1	grey	0.10866493

ENSG00000262529	CTA-276F8.2	grey	0.37862645
ENSG00000235354	CTA-276O3.4	grey	0.30355874
ENSG00000226471	CTA-292E10.6	grey	0.27663614
ENSG00000224256	CTA-292E10.7	grey	0.20942021
ENSG00000271330	CTA-298G8.2	grey	0.15456407
ENSG00000260708	CTA-29F11.1	grey	-0.1461824
ENSG00000260865	CTA-331F8.1	grey	0.22601916
ENSG00000226624	CTA-351J1.1	grey	0.3422322
ENSG00000243193	CTA-360L10.1	grey	0.37397265
ENSG00000254153	CTA-398F10.2	grey	0.28977966
ENSG00000224003	CTA-407F11.6	grey	0.26123465
ENSG00000261188	CTA-445C9.14	salmon	0.55953292
ENSG00000260065	CTA-445C9.15	grey	0.05839747
ENSG00000226772	CTA-747E2.10	grey	0.32669665
ENSG00000254484	CTA-797E19.1	grey	0.09696915
ENSG00000227117	CTA-85E5.10	turquoise	0.55983109
ENSG00000230051	CTA-929C8.8	magenta	0.74270717
ENSG00000238120	CTA-941F9.9	grey	0.30293678
ENSG00000250318	CTA-963H5.5	grey	-0.0304797
ENSG00000231405	CTA-992D9.7	grey	0.00088676
ENSG00000232872	CTAGE3P	grey	0.3362934
ENSG00000269653	CTB-102L5.7	grey	0.08713035
ENSG00000253527	CTB-105L4.2	grey	0.0372785
ENSG00000251405	CTB-109A12.1	green	0.45663783
ENSG00000249035	CTB-113P19.1	grey	0.00125228
ENSG00000239528	CTB-118N6.1	grey	0.34633821
ENSG00000248445	CTB-118N6.3	yellow	0.76017237
ENSG00000248367	CTB-129O4.1	grey	0.10875225
ENSG00000267114	CTB-129P6.11	grey	0.16726691
ENSG00000250069	CTB-131B5.2	grey	0.32751074
ENSG00000266469	CTB-131K11.1	brown	-0.7121662
ENSG00000260545	CTB-134F13.1	grey	0.26091544
ENSG00000267696	CTB-151G24.1	grey	0.40195316
ENSG00000271482	CTB-152G17.4	grey	0.41841627
ENSG00000270764	CTB-152G17.5	grey	0.60009745
ENSG00000254003	CTB-167B5.1	magenta	-0.4313411
ENSG00000248222	CTB-174D11.1	grey	0.10897234
ENSG00000248965	CTB-174D11.2	grey	0.26868012
ENSG00000267934	CTB-176F20.3	grey	-0.1292257
ENSG00000253978	CTB-178M22.2	yellow	-0.4248908
ENSG00000254365	CTB-180C19.1	grey	-0.0577986
ENSG00000267375	CTB-186G2.4	grey	0.27110376
ENSG00000263726	CTB-187M2.3	grey	0.08704643
ENSG00000228341	CTB-20D2.1	grey	0.3108966
ENSG00000267759	CTB-25B13.13	grey	0.46420592
ENSG00000267715	CTB-25B13.6	grey	0.27020868
ENSG00000261596	CTB-31N19.3	grey	0.18213026
ENSG00000261526	CTB-31O20.2	grey	0.32926055

ENSG00000267498	CTB-32O4.2	grey	0.26701771
ENSG00000253768	CTB-33O18.1	grey	0.14808772
ENSG00000260686	CTB-36H16.2	grey	-0.0579493
ENSG00000244921	CTB-36O1.7	grey	0.12213315
ENSG00000250409	CTB-3M24.3	grey	0.34133939
ENSG00000254211	CTB-43E15.4	turquoise	0.33219791
ENSG00000242683	CTB-46B19.1	grey	0.39693869
ENSG00000249593	CTB-46B19.2	grey	0.3433882
ENSG00000248544	CTB-47B11.3	grey	0.22308395
ENSG00000248245	CTB-49A3.2	grey	0.32879533
ENSG00000250244	CTB-49A3.4	grey	0.17070259
ENSG00000249478	CTB-49A3.5	grey	0.26318977
ENSG00000269374	CTB-50E14.5	grey	0.25316999
ENSG00000267169	CTB-55O6.12	grey	0.04678275
ENSG00000229119	CTB-63M22.1	brown	0.39225728
ENSG00000267299	CTB-66B24.1	grey	0.10711984
ENSG00000267067	CTB-75G16.3	grey	0.18267517
ENSG00000254187	CTB-78F1.1	purple	0.51781746
ENSG00000253357	CTB-78F1.2	black	-0.4826691
ENSG00000230551	CTB-89H12.4	blue	-0.4674333
ENSG00000250407	CTB-99A3.1	grey	0.39178469
ENSG00000159692	CTBP1	grey	-0.3290319
ENSG00000196810	CTBP1-AS2	grey	0.33952707
ENSG00000175029	CTBP2	grey60	-0.670886
ENSG00000234383	CTBP2P8	grey	0.19690816
ENSG00000117151	CTBS	blue	-0.6060088
ENSG00000270137	CTC-137K3.1	grey	0.11392808
ENSG00000270021	CTC-203F4.2	green	0.74396022
ENSG00000250328	CTC-210G5.1	cyan	-0.4927214
ENSG00000269172	CTC-218B8.3	grey	0.27842559
ENSG00000249605	CTC-218H9.1	grey	0.19560638
ENSG00000230561	CTC-228N24.1	grey	0.00779835
ENSG00000254011	CTC-229L21.1	grey	0.24823888
ENSG00000266983	CTC-232P5.1	grey	0.11860463
ENSG00000267262	CTC-232P5.3	grey	0.29963869
ENSG00000248127	CTC-235G5.3	grey	0.17855762
ENSG00000245317	CTC-241N9.1	grey	0.17162661
ENSG00000268051	CTC-244M17.1	grey	0.38818905
ENSG00000269246	CTC-246B18.10	grey	0.37821717
ENSG00000271524	CTC-260E6.10	grey	0.21494924
ENSG00000267383	CTC-260E6.6	lightgreen	0.58960908
ENSG00000267771	CTC-260E6.7	grey	0.4603517
ENSG00000253713	CTC-264O10.2	grey	0.29417666
ENSG00000267749	CTC-265F19.1	grey	0.32371591
ENSG00000253591	CTC-265N9.1	grey	0.11666676
ENSG00000269814	CTC-273B12.10	grey	0.26405967
ENSG00000269423	CTC-273B12.6	turquoise	0.30018493
ENSG00000262691	CTC-277H1.7	grey	-0.0131509

ENSG00000261996	CTC-281F24.1	grey	0.02535774
ENSG00000249068	CTC-287O8.1	blue	-0.3726484
ENSG00000248109	CTC-295J13.3	grey	0.58488393
ENSG00000270133	CTC-303L1.2	grey	0.32166855
ENSG00000264791	CTC-304I17.2	grey	0.18613615
ENSG00000265775	CTC-304I17.3	grey	0.09502348
ENSG00000263485	CTC-304I17.4	grey	0.05835517
ENSG00000263571	CTC-304I17.5	grey	-0.0897344
ENSG00000266912	CTC-325H20.2	blue	-0.3355278
ENSG00000267510	CTC-325H20.4	grey	0.27213955
ENSG00000270614	CTC-325H20.7	grey	0.0843964
ENSG00000251221	CTC-325J23.3	grey	0.32753986
ENSG00000249637	CTC-329D1.2	grey	0.15731418
ENSG00000271228	CTC-336P14.1	turquoise	0.5978576
ENSG00000233937	CTC-338M12.4	grey	0.07581718
ENSG00000250222	CTC-338M12.5	grey	0.0724367
ENSG00000250015	CTC-339F2.2	blue	-0.3954477
ENSG00000241956	CTC-340A15.2	blue	0.56393283
ENSG00000249335	CTC-340D7.1	grey	-0.0537175
ENSG00000268401	CTC-344H19.4	grey	0.35719188
ENSG00000248371	CTC-347C20.2	grey	0.30032011
ENSG00000253687	CTC-348L5.1	yellow	0.66016416
ENSG00000253660	CTC-353G13.1	grey	0.28696045
ENSG00000242615	CTC-359D24.3	grey	0.28752942
ENSG00000269486	CTC-360G5.9	grey	0.19347376
ENSG00000213303	CTC-398G3.2	grey	0.37183393
ENSG00000270402	CTC-412M14.5	grey	0.412408
ENSG00000271283	CTC-412M14.6	grey	0.31295362
ENSG00000260115	CTC-420A11.2	grey	0.33100282
ENSG00000267562	CTC-421K24.1	grey	0.3577365
ENSG00000253946	CTC-425K20.1	grey	0.05263021
ENSG00000205041	CTC-425O23.2	grey	0.022862
ENSG00000248175	CTC-428G20.3	grey	-0.0402928
ENSG00000268087	CTC-429P9.2	grey	0.23807717
ENSG00000269044	CTC-429P9.3	grey	0.03873633
ENSG00000267871	CTC-444N24.6	grey	-0.0405266
ENSG00000268713	CTC-444N24.8	grey	0.17718769
ENSG00000267006	CTC-448F2.4	salmon	-0.4890296
ENSG00000266910	CTC-448F2.6	grey	0.29148474
ENSG00000268764	CTC-450M9.1	grey	0.39983048
ENSG00000213757	CTC-451P13.1	grey	0.43718775
ENSG00000251675	CTC-458I2.2	yellow	0.69989178
ENSG00000261770	CTC-459F4.1	grey	0.19638255
ENSG00000267575	CTC-459F4.3	grey	0.12999428
ENSG00000266977	CTC-459F4.5	grey	0.37813241
ENSG00000267264	CTC-459F4.6	grey	0.31536668
ENSG00000267389	CTC-459F4.7	grey	0.14137118
ENSG00000267768	CTC-459F4.8	grey	0.32450894

ENSG00000251450	CTC-459I6.1	grey	-0.0632173
ENSG00000250882	CTC-459M5.1	grey	0.2384627
ENSG00000251187	CTC-459M5.2	grey	0.10911866
ENSG00000261589	CTC-462L7.1	grey	0.0906791
ENSG00000187534	CTC-471F3.4	grey	-0.0564047
ENSG00000269069	CTC-471F3.5	grey	-0.3184204
ENSG00000268166	CTC-471F3.6	grey	-0.0390024
ENSG00000261884	CTC-479C5.12	grey	-0.0274157
ENSG00000249175	CTC-484P3.3	red	-0.5118028
ENSG00000270067	CTC-487M23.5	grey	0.44221231
ENSG00000269843	CTC-490E21.10	grey	0.19766836
ENSG00000261939	CTC-496I23.1	grey	0.48706296
ENSG00000250156	CTC-498M16.2	turquoise	0.52097938
ENSG00000250306	CTC-498M16.3	grey	0.40991707
ENSG00000266922	CTC-499B15.6	grey	0.53369169
ENSG00000248268	CTC-499J9.1	grey	0.4885629
ENSG00000267427	CTC-503J8.6	grey	0.10382014
ENSG00000267003	CTC-507E2.1	grey	0.530019
ENSG00000271109	CTC-523E23.11	grey	0.31635508
ENSG00000263272	CTC-524C5.2	grey	-0.1020197
ENSG00000266248	CTC-525D6.2	grey	-0.0408407
ENSG00000254391	CTC-529G1.1	yellow	0.48350175
ENSG00000253251	CTC-534A2.2	grey	0.11439613
ENSG00000253512	CTC-535M15.1	grey	0.23021607
ENSG00000253693	CTC-535M15.2	blue	0.34386609
ENSG00000249588	CTC-537E7.1	grey	0.15469521
ENSG00000248359	CTC-537E7.2	grey	0.08820244
ENSG00000248884	CTC-537E7.3	grey	0.22480547
ENSG00000263990	CTC-542B22.2	grey	0.36242595
ENSG00000251293	CTC-552D5.1	blue	0.33823639
ENSG00000249545	CTC-558O19.1	grey	0.15488771
ENSG00000254042	CTC-558O2.1	grey	0.26667236
ENSG00000270270	CTC-559E9.10	grey	0.53958037
ENSG00000267481	CTC-559E9.5	grey	0.30118698
ENSG00000267419	CTC-559E9.6	grey	0.30549791
ENSG00000267565	CTC-559E9.8	grey	0.38402158
ENSG00000249412	CTC-563A5.2	grey	0.21667151
ENSG00000251680	CTC-575N7.1	grey	0.17349963
ENSG00000262601	CTC-786C10.1	grey	0.38793358
ENSG00000255489	CTC-830E23.1	grey	0.33802422
ENSG00000178971	CTC1	turquoise	-0.5879027
ENSG00000102974	CTCF	green	0.5201361
ENSG00000258682	CTD-2002H8.2	turquoise	0.58269965
ENSG00000254731	CTD-2005H7.1	grey	-0.0376144
ENSG00000255250	CTD-2005H7.2	grey	0.27216912
ENSG00000267274	CTD-2006C1.12	grey	0.40832607
ENSG00000219665	CTD-2006C1.2	grey	0.25430631
ENSG00000241597	CTD-2007H13.1	grey	0.28152979

ENSG00000248489	CTD-2007H13.3	grey	0.08573423
ENSG00000260848	CTD-2009A10.1	grey	0.200519
ENSG00000254595	CTD-2010I16.1	grey	0.25039743
ENSG00000271119	CTD-2012J19.3	grey	0.04645689
ENSG00000261386	CTD-2012K14.6	grey	0.09578858
ENSG00000244513	CTD-2013N24.2	grey	0.12331304
ENSG00000258847	CTD-2014B16.3	grey	-0.0663516
ENSG00000249042	CTD-2015H6.3	grey	-0.0510255
ENSG00000256705	CTD-2017C7.1	grey	0.40018142
ENSG00000259088	CTD-2017C7.2	grey	0.24552832
ENSG00000259161	CTD-2017C7.3	grey	0.16451987
ENSG00000268362	CTD-2017D11.1	grey	0.40415733
ENSG00000267121	CTD-2020K17.1	blue	0.51708567
ENSG00000233175	CTD-2020K17.3	blue	0.3569203
ENSG00000228897	CTD-2021A8.2	grey	0.42328412
ENSG00000227080	CTD-2021A8.3	lightcyan	0.54023417
ENSG00000258053	CTD-2021H9.3	grey	0.45612899
ENSG00000253347	CTD-2026D20.2	grey	0.18553318
ENSG00000264281	CTD-2031P19.4	grey	-0.1551572
ENSG00000269961	CTD-2033C11.1	grey	0.37738461
ENSG00000245556	CTD-2037K23.2	grey	0.10220195
ENSG00000263069	CTD-2047H16.4	turquoise	-0.5578025
ENSG00000261329	CTD-2049O4.1	yellow	0.59378409
ENSG00000259221	CTD-2050N2.1	grey	0.08682672
ENSG00000259048	CTD-2058B24.2	grey	-0.1349071
ENSG00000258696	CTD-2058B24.3	grey	0.15235706
ENSG00000258500	CTD-2062F14.2	grey	0.35454754
ENSG00000267014	CTD-2081K17.2	turquoise	0.53332533
ENSG00000260774	CTD-2083E4.4	grey	0.18284074
ENSG00000250848	CTD-2083E4.5	grey	0.25714858
ENSG00000261434	CTD-2083E4.7	green	-0.4341497
ENSG00000266930	CTD-2085J24.4	yellow	-0.504497
ENSG00000267381	CTD-2086O20.3	grey	-0.0475777
ENSG00000267093	CTD-2105E13.13	grey	0.39049764
ENSG00000250127	CTD-2117L12.1	grey	0.26813225
ENSG00000269091	CTD-2126E3.3	grey	0.31376812
ENSG00000267890	CTD-2126E3.4	grey	0.02866763
ENSG00000270010	CTD-2132N18.4	grey	0.3255043
ENSG00000248896	CTD-2135J3.3	grey	0.34080112
ENSG00000249781	CTD-2143L24.1	black	0.56874373
ENSG00000265478	CTD-2145A24.3	grey	0.23150917
ENSG00000259485	CTD-2147F2.1	grey	0.29292312
ENSG00000250764	CTD-2152M20.2	grey	0.5691091
ENSG00000267260	CTD-2162K18.4	brown	0.4047746
ENSG00000267254	CTD-2162K18.5	grey	0.27895875
ENSG00000261360	CTD-2165H16.4	grey	0.26223007
ENSG00000251575	CTD-2170G1.2	yellow	0.5376057
ENSG00000259712	CTD-2184D3.5	grey	0.17891132

ENSG00000259327	CTD-2184D3.6	grey60	0.58906342
ENSG00000259709	CTD-2184D3.7	grey	0.25679708
ENSG00000243295	CTD-2185K10.1	grey	0.24489984
ENSG00000178464	CTD-2192J16.15	grey	0.20953753
ENSG00000269560	CTD-2192J16.21	grey	0.22695247
ENSG00000249772	CTD-2193P3.2	grey	0.13786168
ENSG00000249577	CTD-2195M15.1	turquoise	0.43167376
ENSG00000259806	CTD-2196E14.4	grey	0.30934548
ENSG00000260751	CTD-2196E14.6	grey	0.31571911
ENSG00000260482	CTD-2196E14.9	salmon	-0.4532506
ENSG00000251189	CTD-2196P11.2	grey	0.23165363
ENSG00000249807	CTD-2199O4.1	grey	0.33783778
ENSG00000260515	CTD-2199O4.3	grey	0.30298311
ENSG00000177738	CTD-2201E18.3	grey	0.28277187
ENSG00000250001	CTD-2201E9.2	grey	0.2548457
ENSG00000249825	CTD-2201I18.1	grey	0.1454873
ENSG00000249572	CTD-2203K17.1	grey	0.30669283
ENSG00000249774	CTD-2206G10.2	grey	0.37996471
ENSG00000249286	CTD-2210P15.2	grey	0.19685817
ENSG00000205106	CTD-2210P24.4	grey	0.2371014
ENSG00000250365	CTD-2213F21.2	yellow	0.52498795
ENSG00000248758	CTD-2215E18.2	grey	0.32479226
ENSG00000188242	CTD-2228K2.5	grey	0.07599487
ENSG00000225138	CTD-2228K2.7	grey	0.06218298
ENSG00000259143	CTD-2240H23.2	grey	0.4683613
ENSG00000258829	CTD-2243E23.1	grey	-0.0783377
ENSG00000269051	CTD-2245F17.3	grey	0.0631966
ENSG00000248794	CTD-2248H3.1	grey	0.30176291
ENSG00000259802	CTD-2256P15.2	grey	0.19491751
ENSG00000248734	CTD-2260A17.1	grey	0.26543678
ENSG00000247121	CTD-2260A17.2	grey	0.01039422
ENSG00000259630	CTD-2262B20.1	grey	0.25880799
ENSG00000249514	CTD-2266L18.1	grey	0.39260349
ENSG00000250320	CTD-2269F5.1	grey	0.23132014
ENSG00000258882	CTD-2277K2.1	grey	0.24864144
ENSG00000269815	CTD-2278I10.4	grey	0.27241107
ENSG00000249916	CTD-2280E9.1	grey	0.26963163
ENSG00000253640	CTD-2281E23.1	turquoise	-0.3533452
ENSG00000254269	CTD-2281E23.2	green	0.485755
ENSG00000251330	CTD-2283N19.1	grey	0.26257825
ENSG00000254231	CTD-2284J15.1	grey	0.29261541
ENSG00000267655	CTD-2286N8.2	grey	0.35227964
ENSG00000185641	CTD-2287O16.1	grey	-0.0766135
ENSG00000224282	CTD-2290C23.1	grey	0.33463699
ENSG00000241739	CTD-2290C23.2	grey	0.26375733
ENSG00000267886	CTD-2291D10.4	grey	0.12445116
ENSG00000253741	CTD-2292P10.4	grey	0.02391003
ENSG00000258636	CTD-2298J14.2	grey	0.11359479

ENSG00000240729	CTD-2301A4.3	grey	0.16600422
ENSG00000270706	CTD-2301A4.5	grey	0.23003522
ENSG00000259377	CTD-2308G16.1	grey	0.36998713
ENSG00000240535	CTD-2313F11.1	grey	0.42436999
ENSG00000248671	CTD-2313N18.5	grey	0.14042004
ENSG00000225805	CTD-2313N18.7	grey	0.28932215
ENSG00000253236	CTD-2313P7.1	purple	0.50124107
ENSG00000259685	CTD-2315E11.1	grey	0.22948917
ENSG00000267248	CTD-2319I12.4	grey	0.05674615
ENSG00000253596	CTD-2320G14.2	grey	0.31511295
ENSG00000260269	CTD-2323K18.1	grey	0.13938764
ENSG00000250490	CTD-2324F15.2	grey	0.18057009
ENSG00000267939	CTD-2325M2.1	grey	0.15514616
ENSG00000259038	CTD-2325P2.4	grey	-0.0960017
ENSG00000226958	CTD-2328D6.1	grey	-0.0653055
ENSG00000259563	CTD-2329K10.1	grey	0.4679259
ENSG00000268225	CTD-2331H12.5	grey	0.37725003
ENSG00000268560	CTD-2332E11.2	grey	0.34481911
ENSG00000253982	CTD-2336O2.1	turquoise	0.63535545
ENSG00000268164	CTD-2337J16.1	grey	0.12998087
ENSG00000242602	CTD-2339M3.1	grey	0.24339198
ENSG00000258733	CTD-2341M24.1	grey	0.15502996
ENSG00000263603	CTD-2349P21.5	grey	0.18644665
ENSG00000265443	CTD-2349P21.6	grey	0.19427963
ENSG00000266490	CTD-2349P21.9	grey	0.07090698
ENSG00000264098	CTD-2350C19.1	grey	0.20551387
ENSG00000250155	CTD-2353F22.1	blue	-0.6833681
ENSG00000261780	CTD-2354A18.1	greenyellow	-0.4940443
ENSG00000261731	CTD-2358C21.4	grey	0.14237155
ENSG00000247796	CTD-2366F13.1	grey	-0.0581965
ENSG00000176593	CTD-2368P22.1	grey	-0.0736381
ENSG00000175898	CTD-2369P2.2	grey	0.28061713
ENSG00000267491	CTD-2373H9.5	grey	0.07794838
ENSG00000253112	CTD-2373N4.5	grey	0.30130474
ENSG00000261195	CTD-2380F24.1	grey	0.11210982
ENSG00000270177	CTD-2410N18.3	grey	0.43914743
ENSG00000228979	CTD-2501B8.5	grey	0.14261214
ENSG00000259274	CTD-2501E16.2	grey	0.33777345
ENSG00000257120	CTD-2503I6.1	grey	0.43635163
ENSG00000204556	CTD-2514C3.1	grey	0.3501062
ENSG00000246731	CTD-2514K5.2	green	0.57897334
ENSG00000264272	CTD-2514K5.4	yellow	0.52992695
ENSG00000261669	CTD-2515A14.1	grey	0.42671281
ENSG00000251364	CTD-2516F10.2	yellow	0.33518323
ENSG00000269640	CTD-2521M24.9	turquoise	0.48859451
ENSG00000260037	CTD-2524L6.3	grey	0.40650149
ENSG00000268015	CTD-2525I3.3	grey	0.18990442
ENSG00000269102	CTD-2525I3.5	grey	0.27723884

ENSG00000267319	CTD-2528L19.3	grey	0.23839545
ENSG00000267152	CTD-2528L19.6	grey	0.17897296
ENSG00000254826	CTD-2530H12.2	grey	0.25538653
ENSG00000255326	CTD-2530H12.4	grey	0.34063501
ENSG00000254630	CTD-2530H12.7	grey	0.35009497
ENSG00000255434	CTD-2530H12.8	grey	0.22830364
ENSG00000254064	CTD-2530N21.4	grey	0.30946119
ENSG00000251670	CTD-2532K18.1	grey	0.23754929
ENSG00000254787	CTD-2537O9.1	grey	0.21814858
ENSG00000267475	CTD-2538C1.2	blue	0.41080997
ENSG00000268743	CTD-2538G9.5	grey	0.32906532
ENSG00000267714	CTD-2540B15.6	turquoise	0.53155807
ENSG00000259888	CTD-2540M10.1	grey	0.21621559
ENSG00000260578	CTD-2541J13.1	grey	0.23226569
ENSG00000249898	CTD-2541M15.1	grey	0.14595562
ENSG00000269373	CTD-2542C24.3	grey	0.25952147
ENSG00000249621	CTD-2544H17.1	grey	0.18385609
ENSG00000268518	CTD-2545M3.8	yellow	0.56689379
ENSG00000180747	CTD-2547E10.2	grey	0.21197861
ENSG00000254988	CTD-2547H18.1	grey	0.48941249
ENSG00000245281	CTD-2547L16.1	grey	0.16164538
ENSG00000258875	CTD-2547L24.3	grey	0.1742751
ENSG00000267422	CTD-2554C21.1	grey	0.38051306
ENSG00000267640	CTD-2554C21.2	grey	0.12466472
ENSG00000229481	CTD-2554C21.3	grey	-0.0147475
ENSG00000256982	CTD-2555A7.2	grey	-0.1825338
ENSG00000258824	CTD-2555O16.2	brown	-0.6553361
ENSG00000258796	CTD-2560C21.1	grey	0.19484461
ENSG00000268231	CTD-2560K21.6	grey	0.25504121
ENSG00000262098	CTD-2561B21.10	grey	0.14700968
ENSG00000262873	CTD-2561B21.11	magenta	0.53269826
ENSG00000213976	CTD-2561J22.2	grey	0.28876082
ENSG00000268119	CTD-2561J22.5	grey	0.24417987
ENSG00000261714	CTD-2562G15.2	grey	0.06173583
ENSG00000255136	CTD-2562J17.4	turquoise	-0.4972222
ENSG00000254429	CTD-2562J17.7	yellow	-0.6583213
ENSG00000269656	CTD-2571L23.6	grey	0.28493967
ENSG00000247735	CTD-2574D22.2	grey	0.19388659
ENSG00000260114	CTD-2574D22.4	grey	0.13084848
ENSG00000270804	CTD-2583A14.11	grey	0.21958878
ENSG00000269867	CTD-2583A14.8	grey	0.2908663
ENSG00000268496	CTD-2587H19.2	grey	-0.0156557
ENSG00000269873	CTD-2587H19.3	grey	0.10524466
ENSG00000249908	CTD-2589H19.4	grey	0.1537336
ENSG00000268438	CTD-2607J13.1	grey	0.25249929
ENSG00000268593	CTD-2611O12.6	grey	0.31550753
ENSG00000268543	CTD-2619J13.16	grey	0.35663463
ENSG00000268912	CTD-2619J13.17	grey	0.27756581

ENSG00000269473	CTD-2619J13.19	grey	0.36127712
ENSG00000268049	CTD-2619J13.9	grey	0.13938873
ENSG00000268613	CTD-2620I22.7	grey	0.29437512
ENSG00000267370	CTD-2623N2.3	grey	0.18482033
ENSG00000267309	CTD-2630F21.1	grey	0.1982179
ENSG00000261604	CTD-2636A23.2	grey	0.13143305
ENSG00000267898	CTD-2639E6.9	grey	-0.2318132
ENSG00000254129	CTD-2647L4.1	grey	0.41364137
ENSG00000259366	CTD-2647L4.4	turquoise	0.67829441
ENSG00000259520	CTD-2651B20.3	grey	0.00909957
ENSG00000234773	CTD-2666L21.1	cyan	-0.4336736
ENSG00000234750	CTD-2666L21.2	grey	0.20917797
ENSG00000269806	CTD-3001H11.1	grey	0.3912822
ENSG00000269834	CTD-3018O17.3	grey	0.00470236
ENSG00000254081	CTD-3025N20.2	yellow	0.51563818
ENSG00000268322	CTD-3030D20.2	grey	0.28677894
ENSG00000269836	CTD-3032J10.4	grey	0.37681588
ENSG00000213315	CTD-3035D6.1	grey	0.2031414
ENSG00000259876	CTD-3037G24.4	grey	0.24777568
ENSG00000259067	CTD-3051D23.3	grey	0.173346
ENSG00000266916	CTD-3064H18.1	brown	0.50816922
ENSG00000259212	CTD-3065B20.2	grey	0.34365853
ENSG00000259314	CTD-3065B20.3	grey	0.27051533
ENSG00000255224	CTD-3065J16.9	grey	0.1342247
ENSG00000269990	CTD-3074O7.12	greenyellow	-0.5517738
ENSG00000255517	CTD-3074O7.5	grey	0.22826007
ENSG00000215302	CTD-3092A11.1	grey	-0.0474473
ENSG00000270055	CTD-3092A11.2	grey	0.01201236
ENSG00000266288	CTD-3096M3.2	grey	0.40911347
ENSG00000268970	CTD-3099C6.11	grey	0.21105363
ENSG00000269825	CTD-3099C6.9	black	-0.5254401
ENSG00000236483	CTD-3105H18.8	grey	0.26852159
ENSG00000267612	CTD-3116E22.7	grey	0.01582314
ENSG00000269439	CTD-3131K8.2	grey	0.41845108
ENSG00000268516	CTD-3138B18.5	brown	-0.5656669
ENSG00000268201	CTD-3138B18.6	grey	0.16251731
ENSG00000265519	CTD-3157E16.1	turquoise	0.6547014
ENSG00000267053	CTD-3162L10.1	grey	-0.2547687
ENSG00000232442	CTD-3184A7.4	grey	0.04195979
ENSG00000261351	CTD-3185P2.1	grey	0.27433911
ENSG00000183248	CTD-3193O13.9	blue	0.65772031
ENSG00000262194	CTD-3195I5.5	salmon	0.57314364
ENSG00000271555	CTD-3214K23.1	grey	0.25426378
ENSG00000267605	CTD-3220F14.1	grey	-0.159975
ENSG00000267682	CTD-3220F14.2	grey	0.34116741
ENSG00000240808	CTD-3236F5.1	grey	0.30031851
ENSG00000060069	CTDP1	grey	0.27254783
ENSG00000144579	CTDSP1	brown	-0.6179041

ENSG00000175215	CTDSP2	blue	-0.9013013
ENSG00000144677	CTDSPL	black	0.61118806
ENSG00000137770	CTDSPL2	turquoise	0.42304491
ENSG00000150281	CTF1	turquoise	0.5286587
ENSG00000118523	CTGF	blue	-0.3878396
ENSG00000179296	CTGLF12P	grey	-0.0672107
ENSG00000116761	CTH	grey	0.33336832
ENSG00000164932	CTHRC1	grey	0.32273447
ENSG00000134030	CTIF	grey60	0.4465182
ENSG00000066032	CTNNA2	yellow	-0.4929657
ENSG00000183230	CTNNA3	green	0.88692549
ENSG00000119326	CTNNAL1	grey	0.04629712
ENSG00000168036	CTNNB1	darkmagenta	-0.6335376
ENSG00000178585	CTNNBIP1	red	0.55545401
ENSG00000132792	CTNNBL1	salmon	0.61446699
ENSG00000198561	CTNND1	darkmagenta	-0.6580156
ENSG00000169862	CTNND2	turquoise	0.76520142
ENSG00000040531	CTNS	grey	0.20100733
ENSG00000171793	CTPS1	brown	0.77574599
ENSG00000047230	CTPS2	black	0.64947011
ENSG00000198730	CTR9	darkred	0.58594904
ENSG00000164733	CTSB	black	0.59095606
ENSG00000109861	CTSC	royalblue	0.54918026
ENSG00000117984	CTSD	grey	0.06011478
ENSG00000174080	CTSF	salmon	0.65714896
ENSG00000103811	CTSH	grey	-0.0759333
ENSG00000143387	CTSK	grey	0.10805775
ENSG00000135047	CTSL	salmon	0.56981301
ENSG00000163131	CTSS	royalblue	0.84365776
ENSG00000101160	CTSZ	grey	-0.1702234
ENSG00000085733	CTTN	red	-0.5546797
ENSG00000077063	CTTNBP2	floralwhite	0.76790945
ENSG00000143079	CTTNBP2NL	grey	-0.0443983
ENSG00000174177	CTU2	brown	0.54674205
ENSG00000178531	CTXN1	magenta	0.49149856
ENSG00000233932	CTXN2	grey	-0.027541
ENSG00000222670	CU459211.1	grey	0.26610488
ENSG00000107611	CUBN	grey	0.28731449
ENSG00000180891	CUEDC1	yellow	0.63065296
ENSG00000055130	CUL1	brown	0.6091184
ENSG00000108094	CUL2	brown	0.74478796
ENSG00000036257	CUL3	darkred	0.72645439
ENSG00000139842	CUL4A	grey	-0.0170055
ENSG00000158290	CUL4B	turquoise	-0.370205
ENSG00000166266	CUL5	grey	-0.1204995
ENSG00000044090	CUL7	brown	0.49541574
ENSG00000112514	CUTA	brown	0.89920289
ENSG00000119929	CUTC	grey	-0.0989965

ENSG00000257923	CUX1	brown	-0.6287446
ENSG00000150316	CWC15	greenyellow	0.45660772
ENSG00000163510	CWC22	green	0.5510783
ENSG00000108296	CWC25	grey	-0.0738157
ENSG00000153015	CWC27	lightgreen	0.62166829
ENSG00000006210	CX3CL1	turquoise	-0.548233
ENSG00000168329	CX3CR1	cyan	0.82053024
ENSG00000107562	CXCL12	grey	-0.2836865
ENSG00000145824	CXCL14	turquoise	0.66491972
ENSG00000161921	CXCL16	cyan	0.68971767
ENSG00000121966	CXCR4	grey	0.0812219
ENSG00000173681	CXorf23	grey	0.24261115
ENSG00000196741	CXorf24	turquoise	0.57904453
ENSG00000228459	CXorf28	grey	0.24140477
ENSG00000147113	CXorf36	tan	0.76123731
ENSG00000185753	CXorf38	grey	0.29508643
ENSG00000197620	CXorf40A	grey	0.23208693
ENSG00000197021	CXorf40B	brown	0.43604236
ENSG00000147231	CXorf57	purple	0.77467098
ENSG00000154832	CXXC1	grey	0.04110503
ENSG00000188011	CXXC11	purple	0.78366354
ENSG00000168772	CXXC4	salmon	-0.4984421
ENSG00000171604	CXXC5	yellow	0.58785423
ENSG00000008283	CYB561	blue	0.70726157
ENSG00000162144	CYB561A3	grey	0.26110867
ENSG00000174151	CYB561D1	grey	0.19817426
ENSG00000114395	CYB561D2	grey	0.23018792
ENSG00000166347	CYB5A	grey	0.30203011
ENSG00000103018	CYB5B	turquoise	0.4257905
ENSG00000182224	CYB5D1	grey	-0.1699399
ENSG00000159348	CYB5R1	grey	-0.0005817
ENSG00000166394	CYB5R2	yellow	0.58900414
ENSG00000100243	CYB5R3	lightcyan	0.62901507
ENSG00000065615	CYB5R4	red	-0.6172422
ENSG00000215883	CYB5RL	grey	0.2471827
ENSG00000051523	CYBA	cyan	0.66687486
ENSG00000165168	CYBB	cyan	0.87851944
ENSG00000071967	CYBRD1	turquoise	0.80916025
ENSG00000179091	CYC1	red	0.61837141
ENSG00000172115	CYCS	turquoise	0.67435609
ENSG00000232901	CYCSP10	grey	0.46452116
ENSG00000254358	CYCSP22	grey	0.13735147
ENSG00000227666	CYCSP24	grey	0.40422961
ENSG00000176268	CYCSP34	grey	0.17357761
ENSG00000259513	CYCSP38	grey	0.24862294
ENSG00000229120	CYCSP4	grey	0.36620912
ENSG00000262870	CYCSP40	grey	0.55209524
ENSG00000235700	CYCSP52	grey	0.32925473

ENSG00000232503	CYCSP8	grey	0.38652994
ENSG00000068793	CYFIP1	cyan	0.64873504
ENSG00000055163	CYFIP2	yellow	-0.5884048
ENSG00000161544	CYGB	black	0.55894066
ENSG00000083799	CYLD	brown	0.46631986
ENSG00000138061	CYP1B1	brown	-0.4748763
ENSG00000119004	CYP20A1	brown	-0.4996875
ENSG00000003137	CYP26B1	grey	-0.1908934
ENSG00000135929	CYP27A1	yellow	0.65037407
ENSG00000255974	CYP2A6	grey	0.10118854
ENSG00000138115	CYP2C8	blue	0.41409061
ENSG00000100197	CYP2D6	grey	-0.0707032
ENSG00000205702	CYP2D7P	grey	-0.0488182
ENSG00000226450	CYP2D8P	grey	-0.0062974
ENSG00000130649	CYP2E1	blue	0.44648428
ENSG00000237118	CYP2F2P	grey	0.03735555
ENSG00000186104	CYP2R1	grey	0.03857108
ENSG00000233622	CYP2T2P	grey	0.33576444
ENSG00000155016	CYP2U1	turquoise	0.642788
ENSG00000160868	CYP3A4	grey	0.2800213
ENSG00000160870	CYP3A7	grey	0.37927218
ENSG00000036530	CYP46A1	grey60	0.47590105
ENSG00000261593	CYP4A27P	grey	0.24361877
ENSG00000171903	CYP4F11	turquoise	0.6002335
ENSG00000228314	CYP4F29P	grey	-0.0667213
ENSG00000223564	CYP4F32P	grey	0.13063798
ENSG00000265787	CYP4F35P	grey	0.08052399
ENSG00000145476	CYP4V2	turquoise	0.73464877
ENSG00000186160	CYP4Z1	blue	0.40016846
ENSG00000001630	CYP51A1	grey	0.19970196
ENSG00000215223	CYP51A1P3	grey	0.22419882
ENSG00000172817	CYP7B1	grey	0.16768095
ENSG00000205795	CYS1	turquoise	0.51742153
ENSG00000152207	CYSLTR2	grey	0.10956492
ENSG00000120306	CYSTM1	turquoise	0.66127426
ENSG00000108669	CYTH1	turquoise	-0.5438027
ENSG00000105443	CYTH2	yellow	-0.5996203
ENSG00000008256	CYTH3	grey	-0.0003989
ENSG00000170891	CYTL1	grey	-0.124141
ENSG00000166265	CYYR1	tan	0.71003834
ENSG00000180902	D2HGDH	brown	0.57764769
ENSG00000100592	DAAM1	turquoise	0.48344704
ENSG00000146122	DAAM2	green	0.90767284
ENSG00000153071	DAB2	grey	0.03614118
ENSG00000136848	DAB2IP	black	0.81325692
ENSG00000165659	DACH1	magenta	0.8466713
ENSG00000165617	DACT1	grey	0.08154459
ENSG00000164488	DACT2	magenta	0.55257369

ENSG00000197380	DACT3	grey	-0.2606941
ENSG00000129562	DAD1	turquoise	0.64713898
ENSG00000173402	DAG1	turquoise	0.80645707
ENSG00000134780	DAGLA	grey	0.27904096
ENSG00000164535	DAGLB	turquoise	-0.5131352
ENSG00000149476	DAK	grey	-0.1039113
ENSG00000178149	DALRD3	red	0.55940017
ENSG00000226950	DANCR	grey	0.05675968
ENSG00000110887	DAO	grey	-0.2370298
ENSG00000112977	DAP	grey	0.22990201
ENSG00000132676	DAP3	brown	0.77039208
ENSG00000196730	DAPK1	turquoise	-0.6362805
ENSG00000236709	DAPK1-IT1	grey	0.20711127
ENSG00000035664	DAPK2	yellow	0.65516652
ENSG00000167657	DAPK3	turquoise	-0.6099014
ENSG00000213088	DARC	grey	0.03751646
ENSG00000115866	DARS	turquoise	0.73268086
ENSG00000117593	DARS2	red	0.44484485
ENSG00000123977	DAW1	brown	0.5577651
ENSG00000204209	DAXX	green	0.53227069
ENSG00000071626	DAZAP1	yellowgreen	-0.6068616
ENSG00000183283	DAZAP2	turquoise	0.68925982
ENSG00000006634	DBF4	grey	0.10386867
ENSG00000161692	DBF4B	grey	-0.072883
ENSG00000155368	DBI	turquoise	0.51226337
ENSG00000231784	DBIL5P	grey	0.1454656
ENSG00000113758	DBN1	blue	0.47036627
ENSG00000244274	DBNDD2	yellow	0.82412746
ENSG00000136279	DBNL	grey	-0.0090673
ENSG00000105516	DBP	grey	0.04760469
ENSG00000138231	DBR1	turquoise	0.52529234
ENSG00000137992	DBT	turquoise	0.68702617
ENSG00000185610	DBX2	turquoise	0.47569072
ENSG00000122741	DCAF10	grey	0.13873001
ENSG00000198876	DCAF12	turquoise	0.5878772
ENSG00000164934	DCAF13	grey	0.08389041
ENSG00000132017	DCAF15	grey	0.05653987
ENSG00000115827	DCAF17	grey	0.26742627
ENSG00000119599	DCAF4	brown	0.5298801
ENSG00000139990	DCAF5	grey	0.19543138
ENSG00000143164	DCAF6	turquoise	-0.5018608
ENSG00000136485	DCAF7	turquoise	0.71039489
ENSG00000172992	DCAKD	lightcyan	0.76960104
ENSG00000164465	DCBLD1	grey	0.42613158
ENSG00000057019	DCBLD2	black	0.45255444
ENSG00000187323	DCC	blue	0.59811897
ENSG00000161634	DCD	grey	0.3108168
ENSG00000222046	DCDC2B	grey	-0.0159334

ENSG00000166341	DCHS1	darkmagenta	-0.6504257
ENSG00000197410	DCHS2	purple	0.78302545
ENSG00000156136	DCK	grey	-0.3512043
ENSG00000133083	DCLK1	turquoise	-0.529671
ENSG00000170390	DCLK2	turquoise	-0.3393303
ENSG00000198924	DCLRE1A	grey	0.2118116
ENSG00000118655	DCLRE1B	turquoise	0.52455232
ENSG00000152457	DCLRE1C	grey	0.16268625
ENSG00000011465	DCN	grey	0.02477733
ENSG00000162290	DCP1A	grey	0.37700286
ENSG00000172795	DCP2	grey	-0.0964256
ENSG00000110063	DCPS	grey	-0.0356216
ENSG00000080166	DCT	grey	0.16280723
ENSG00000129187	DCTD	red	0.59926188
ENSG00000204843	DCTN1	turquoise	-0.8049085
ENSG00000237737	DCTN1-AS1	grey	-0.2214911
ENSG00000175203	DCTN2	brown	0.91760043
ENSG00000137100	DCTN3	green	-0.6615797
ENSG00000132912	DCTN4	lightgreen	-0.6060735
ENSG00000166847	DCTN5	turquoise	-0.4418889
ENSG00000104671	DCTN6	red	0.59740565
ENSG00000043093	DCUN1D1	brown	-0.7585312
ENSG00000150401	DCUN1D2	grey	0.13861129
ENSG00000233613	DCUN1D2-AS	grey	0.37935102
ENSG00000188215	DCUN1D3	grey	0.21122545
ENSG00000109184	DCUN1D4	lightcyan	0.63445816
ENSG00000137692	DCUN1D5	grey	0.08917684
ENSG00000169738	DCXR	grey	0.23250817
ENSG00000130311	DDA1	purple	0.48057793
ENSG00000153904	DDAH1	red	0.8317616
ENSG00000213722	DDAH2	turquoise	0.54779187
ENSG00000167986	DDB1	darkred	0.70413912
ENSG00000134574	DDB2	yellow	0.53269082
ENSG00000178404	DDC8	grey	-0.0722491
ENSG00000100523	DDHD1	grey	0.09467287
ENSG00000197312	DDI2	grey	0.06198007
ENSG00000168209	DDIT4	blue	-0.5311051
ENSG00000145358	DDIT4L	black	0.78033284
ENSG00000181418	DDN	magenta	0.52273825
ENSG00000203797	DDO	grey	-0.3319523
ENSG00000244038	DDOST	grey	0.1987827
ENSG00000204580	DDR1	grey60	-0.6378635
ENSG00000162733	DDR2	brown	-0.4626576
ENSG00000198171	DDRGK1	turquoise	-0.3351431
ENSG00000099977	DDT	yellow	-0.4467181
ENSG00000099974	DDTL	blue	-0.4688829
ENSG00000230683	DDTP1	grey	0.23092899
ENSG00000079785	DDX1	brown	0.7784798

ENSG00000178105	DDX10	turquoise	-0.6207089
ENSG00000237135	DDX10P1	grey	0.36520491
ENSG00000245614	DDX11-AS1	grey	0.15356276
ENSG00000088205	DDX18	blue	-0.4603012
ENSG00000259165	DDX18P1	grey	0.12912761
ENSG00000255220	DDX18P5	yellow	0.53319148
ENSG00000157349	DDX19B	grey	-0.0185446
ENSG00000064703	DDX20	salmon	0.67703647
ENSG00000165732	DDX21	pink	0.69068463
ENSG00000174243	DDX23	pink	0.47166584
ENSG00000089737	DDX24	brown	0.88417452
ENSG00000109832	DDX25	blue	0.66513285
ENSG00000165359	DDX26B	grey	0.05554957
ENSG00000124228	DDX27	turquoise	-0.5674835
ENSG00000182810	DDX28	brown	0.47576024
ENSG00000125485	DDX31	grey	0.23383669
ENSG00000123136	DDX39A	brown	0.36522706
ENSG00000198563	DDX39B	grey	0.04570283
ENSG00000215301	DDX3X	turquoise	0.65766033
ENSG00000067048	DDX3Y	grey	-0.071505
ENSG00000230986	DDX3YP2	grey	0.42066074
ENSG00000183258	DDX41	blue	0.64757887
ENSG00000198231	DDX42	darkred	0.46208059
ENSG00000145833	DDX46	grey	0.03830402
ENSG00000105671	DDX49	brown	0.53804926
ENSG00000108654	DDX5	lightcyan	-0.6877628
ENSG00000229816	DDX50P1	grey	0.39366309
ENSG00000240048	DDX50P2	grey	0.02877882
ENSG00000185163	DDX51	grey	-0.0266158
ENSG00000141141	DDX52	grey	0.23207366
ENSG00000111364	DDX55	grey	0.10944546
ENSG00000136271	DDX56	grey	0.04835496
ENSG00000107201	DDX58	turquoise	0.46447138
ENSG00000118197	DDX59	turquoise	0.50814511
ENSG00000110367	DDX6	grey	-0.1481622
ENSG00000137628	DDX60	grey	0.01251881
ENSG00000181381	DDX60L	grey	0.14881445
ENSG00000177030	DEAF1	blue	0.60038557
ENSG00000104325	DECR1	turquoise	0.61284131
ENSG00000242612	DECR2	grey	0.00137548
ENSG00000158796	DEDD	brown	-0.6668904
ENSG00000023892	DEF6	cyan	0.63073586
ENSG00000140995	DEF8	yellow	-0.5630181
ENSG00000242296	DEFB109P1	grey	-0.0811
ENSG00000206034	DEFB109P1B	grey	0.37917144
ENSG00000254866	DEFB109P3	grey	0.32541698
ENSG00000143753	DEGS1	brown	-0.7427679
ENSG00000168350	DEGS2	grey	-0.0571177

ENSG00000124795	DEK	bisque4	0.77475175
ENSG00000119522	DENND1A	brown	0.70832315
ENSG00000213047	DENND1B	grey	-0.0598968
ENSG00000205744	DENND1C	grey	-0.261048
ENSG00000146966	DENND2A	green	0.56328674
ENSG00000175984	DENND2C	grey	0.18386871
ENSG00000105339	DENND3	royalblue	0.7962745
ENSG00000198837	DENND4B	darkorange	0.56268326
ENSG00000184014	DENND5A	green	0.74483448
ENSG00000170456	DENND5B	yellow	-0.6354296
ENSG00000205593	DENND6B	blue	0.78227918
ENSG00000139726	DENR	red	0.53025128
ENSG00000100150	DEPDC5	grey	0.00901931
ENSG00000155792	DEPTOR	yellow	0.67017932
ENSG00000023697	DERA	grey	0.05901505
ENSG00000136986	DERL1	grey	-0.0597834
ENSG00000072849	DERL2	grey	0.18243618
ENSG00000100418	DESI1	grey	-0.083842
ENSG00000121644	DESI2	grey	0.16672505
ENSG00000140543	DET1	grey	0.18330295
ENSG00000182108	DEXI	grey	0.38236989
ENSG00000160049	DFFA	brown	-0.5131108
ENSG00000169598	DFFB	grey	-0.1626535
ENSG00000105928	DFNA5	royalblue	0.40204914
ENSG00000095397	DFNB31	darkred	-0.5650718
ENSG00000185000	DGAT1	grey	0.04033644
ENSG00000062282	DGAT2	turquoise	-0.6374584
ENSG00000100056	DGCR14	grey	0.20379872
ENSG00000070413	DGCR2	darkred	0.56614961
ENSG00000183628	DGCR6	blue	0.44591934
ENSG00000128185	DGCR6L	grey	0.00892423
ENSG00000128191	DGCR8	grey	0.15380192
ENSG00000065357	DGKA	purple	0.74213081
ENSG00000136267	DGKB	magenta	0.87141248
ENSG00000077044	DGKD	turquoise	-0.5352631
ENSG00000153933	DGKE	grey60	0.71945521
ENSG00000058866	DGKG	grey	0.07202515
ENSG00000102780	DGKH	grey60	0.73678565
ENSG00000157680	DGKI	turquoise	-0.7803465
ENSG00000145214	DGKQ	yellow	-0.514618
ENSG00000149091	DGKZ	turquoise	-0.4903189
ENSG00000114956	DGUOK	brown	0.72924325
ENSG00000237883	DGUOK-AS1	grey	0.02873363
ENSG00000172893	DHCR7	grey	0.25707617
ENSG00000117682	DHDDS	blue	0.53154997
ENSG00000228716	DHFR	brown	-0.3996132
ENSG00000178700	DHFRL1	turquoise	0.34093165
ENSG00000102967	DHODH	yellow	0.44220042

ENSG00000095059	DHPS	brown	0.80131251
ENSG00000157379	DHRS1	grey	-0.0734679
ENSG00000108272	DHRS11	grey	0.0900596
ENSG00000102796	DHRS12	grey	-0.075263
ENSG00000162496	DHRS3	blue	-0.6259321
ENSG00000157326	DHRS4	grey	-0.0350926
ENSG00000215256	DHRS4-AS1	turquoise	0.76310408
ENSG00000187630	DHRS4L2	grey	-0.0093909
ENSG00000100612	DHRS7	turquoise	0.56858907
ENSG00000109016	DHRS7B	lightgreen	-0.6171066
ENSG00000169084	DHRSX	grey	0.11320163
ENSG00000181192	DHTKD1	turquoise	0.69492276
ENSG00000109606	DHX15	blue	-0.7325054
ENSG00000204560	DHX16	lightcyan	-0.5788056
ENSG00000067248	DHX29	red	-0.5951157
ENSG00000132153	DHX30	brown	0.82270444
ENSG00000089876	DHX32	grey	0.24628503
ENSG00000005100	DHX33	grey	0.06727969
ENSG00000134815	DHX34	grey	0.04179174
ENSG00000101452	DHX35	grey	-0.085857
ENSG00000174953	DHX36	turquoise	-0.4534275
ENSG00000150990	DHX37	grey	0.12660242
ENSG00000140829	DHX38	darkgrey	0.4690441
ENSG00000108406	DHX40	grey	0.09606475
ENSG00000163214	DHX57	brown	0.59152579
ENSG00000108771	DHX58	grey	0.00087475
ENSG00000067596	DHX8	turquoise	-0.4526554
ENSG00000135829	DHX9	salmon	0.80977683
ENSG00000184047	DIABLO	grey	0.22309828
ENSG00000131504	DIAPH1	turquoise	-0.7241535
ENSG00000236256	DIAPH2-AS1	grey	0.26153352
ENSG00000100697	DICER1	yellow	0.80398168
ENSG00000235706	DICER1-AS1	turquoise	-0.4978083
ENSG00000101191	DIDO1	grey	-0.0296595
ENSG00000117597	DIEXF	purple	0.72742554
ENSG00000086189	DIMT1	brown	-0.7122449
ENSG00000211448	DIO2	purple	-0.6711535
ENSG00000160305	DIP2A	red	-0.5711269
ENSG00000223692	DIP2A-IT1	grey	0.19422074
ENSG00000066084	DIP2B	yellow	0.80372485
ENSG00000151240	DIP2C	grey	0.19939438
ENSG00000165023	DIRAS2	grey	-0.0418497
ENSG00000162595	DIRAS3	black	-0.7242972
ENSG00000138463	DIRC2	turquoise	0.60177634
ENSG00000231672	DIRC3	grey	-0.2219023
ENSG00000083520	DIS3	lightgreen	0.51038204
ENSG00000166938	DIS3L	red	0.48166697
ENSG00000144535	DIS3L2	turquoise	-0.4973937

ENSG00000162946	DISC1	brown	-0.44711
ENSG00000226758	DISC1-IT1	grey	0.03282359
ENSG00000154309	DISP1	turquoise	0.37934737
ENSG00000140323	DISP2	blue	0.41618777
ENSG00000150764	DIXDC1	yellow	0.48157406
ENSG00000130826	DKC1	salmon	0.58885235
ENSG00000212743	DKFZP667F0711	grey	-0.0466714
ENSG00000105520	DKFZP761J1410	black	-0.6361009
ENSG00000050165	DKK3	salmon	0.7170965
ENSG00000150768	DLAT	grey	-0.0494124
ENSG00000164741	DLC1	purple	-0.3998054
ENSG00000091140	DLD	turquoise	0.50754262
ENSG00000231607	DLEU2	yellow	0.65421061
ENSG00000116652	DLEU2L	grey	0.24285507
ENSG00000237152	DLEU7-AS1	grey	-0.1901309
ENSG00000075711	DLG1	yellow	0.67667878
ENSG00000082458	DLG3	blue	0.8217096
ENSG00000132535	DLG4	blue	0.72455605
ENSG00000151208	DLG5	turquoise	0.5271684
ENSG00000233871	DLG5-AS1	grey	0.18291767
ENSG00000170579	DLGAP1	blue	0.76833605
ENSG00000177337	DLGAP1-AS1	grey	0.12810783
ENSG00000262001	DLGAP1-AS2	grey	0.24788474
ENSG00000263724	DLGAP1-AS3	grey	0.25354388
ENSG00000263878	DLGAP1-AS4	grey	0.04110802
ENSG00000261520	DLGAP1-AS5	darkorange	0.53534493
ENSG00000198010	DLGAP2	magenta	0.84206328
ENSG00000116544	DLGAP3	darkorange	0.72608113
ENSG00000171462	DLK2	blue	0.50628577
ENSG00000198719	DLL1	yellow	0.39306372
ENSG00000090932	DLL3	lightcyan	0.44732717
ENSG00000119689	DLST	grey	0.27280159
ENSG00000144355	DLX1	magenta	0.40454494
ENSG00000105880	DLX5	magenta	0.73462805
ENSG00000006377	DLX6	magenta	0.7857518
ENSG00000231764	DLX6-AS1	magenta	0.70532069
ENSG00000178028	DMAP1	brown	0.71308704
ENSG00000198947	DMD	turquoise	0.34317587
ENSG00000132837	DMGDH	turquoise	0.39580811
ENSG00000161249	DMKN	grey60	0.51388892
ENSG00000104936	DMPK	grey	0.15891478
ENSG00000158856	DMTN	darkorange	0.76797153
ENSG00000185800	DMWD	blue	0.46023907
ENSG00000172869	DMXL1	grey	0.18246969
ENSG00000104093	DMXL2	blue	0.83996512
ENSG00000138346	DNA2	yellow	0.3391045
ENSG00000165506	DNAAF2	yellow	-0.3074621
ENSG00000114841	DNAH1	turquoise	-0.4869394

ENSG00000197653	DNAH10	blue	0.48431862
ENSG00000250091	DNAH10OS	grey	0.12035574
ENSG00000115423	DNAH6	blue	0.61164184
ENSG00000118997	DNAH7	red	0.66082137
ENSG00000122735	DNAI1	magenta	0.39395303
ENSG00000218089	DNAJA1P4	black	0.48896166
ENSG00000069345	DNAJA2	yellow	-0.7271687
ENSG00000103423	DNAJA3	green	-0.7163414
ENSG00000140403	DNAJA4	brown	0.49619674
ENSG00000148719	DNAJB12	grey	0.04692055
ENSG00000164031	DNAJB14	grey	-0.1851637
ENSG00000135924	DNAJB2	grey	-0.0301429
ENSG00000137094	DNAJB5	grey	0.16572977
ENSG00000105993	DNAJB6	grey	0.05267487
ENSG00000128590	DNAJB9	grey	-0.0295282
ENSG00000136770	DNAJC1	lightgreen	0.59126576
ENSG00000007923	DNAJC11	blue	0.47320258
ENSG00000108176	DNAJC12	brown	0.74248464
ENSG00000138246	DNAJC13	grey	0.09550371
ENSG00000135392	DNAJC14	greenyellow	-0.5195296
ENSG00000120675	DNAJC15	brown	-0.677294
ENSG00000116138	DNAJC16	brown	0.43489392
ENSG00000104129	DNAJC17	grey	-0.1809298
ENSG00000170464	DNAJC18	green	-0.550836
ENSG00000205981	DNAJC19	grey	-0.331222
ENSG00000225808	DNAJC19P5	blue	-0.3620311
ENSG00000220871	DNAJC19P6	grey	0.2122668
ENSG00000105821	DNAJC2	grey	-0.245637
ENSG00000168724	DNAJC21	darkgrey	0.66911948
ENSG00000170946	DNAJC24	grey	0.24190905
ENSG00000059769	DNAJC25	grey	-0.0208753
ENSG00000115137	DNAJC27	grey	-0.0356784
ENSG00000224165	DNAJC27-AS1	grey	0.25740954
ENSG00000177692	DNAJC28	grey	0.00584157
ENSG00000102580	DNAJC3	blue	-0.7012997
ENSG00000176410	DNAJC30	yellow	-0.5947124
ENSG00000110011	DNAJC4	lightcyan	0.50474104
ENSG00000101152	DNAJC5	brown	0.77038239
ENSG00000116675	DNAJC6	turquoise	-0.7479905
ENSG00000126698	DNAJC8	grey	0.03870924
ENSG00000213551	DNAJC9	grey	0.18385467
ENSG00000119661	DNAL1	brown	-0.3075876
ENSG00000100246	DNAL4	brown	0.49783175
ENSG00000213918	DNASE1	grey	0.10835279
ENSG00000013563	DNASE1L1	grey	0.21369185
ENSG00000105612	DNASE2	turquoise	0.43938147
ENSG00000187957	DNER	grey	-0.0395316
ENSG00000179532	DNHD1	grey	0.11990795

ENSG00000106976	DNM1	blue	0.89318971
ENSG00000087470	DNM1L	green	-0.7781257
ENSG00000261491	DNM1P31	black	0.55784419
ENSG00000235370	DNM1P51	grey	0.0756137
ENSG00000079805	DNM2	yellow	0.67475439
ENSG00000197959	DNM3	floralwhite	0.62643549
ENSG00000233540	DNM3-IT1	grey	0.29508848
ENSG00000230630	DNM3OS	yellow	0.51771762
ENSG00000107554	DNMBP	red	0.58629137
ENSG00000130816	DNMT1	turquoise	-0.4197438
ENSG00000119772	DNMT3A	red	-0.644789
ENSG00000123992	DNPEP	grey	0.08014475
ENSG00000101457	DNTTIP1	grey	-0.2323591
ENSG00000067334	DNTTIP2	lightgreen	0.56329535
ENSG00000149927	DOC2A	blue	0.41774792
ENSG00000150760	DOCK1	green	0.85546094
ENSG00000135905	DOCK10	darkred	-0.7748638
ENSG00000134516	DOCK2	cyan	0.83162727
ENSG00000088538	DOCK3	blue	0.69707499
ENSG00000128512	DOCK4	lightcyan	-0.6157533
ENSG00000225572	DOCK4-AS1	yellow	0.49014813
ENSG00000147459	DOCK5	yellow	0.90553241
ENSG00000130158	DOCK6	tan	0.57006012
ENSG00000116641	DOCK7	turquoise	0.69944771
ENSG00000107099	DOCK8	cyan	0.8505543
ENSG00000088387	DOCK9	yellowgreen	-0.7710103
ENSG00000229918	DOCK9-AS1	green	0.44805849
ENSG00000260992	DOCK9-AS2	brown	0.57014887
ENSG00000129932	DOHH	yellow	0.39878792
ENSG00000115325	DOK1	royalblue	0.62126413
ENSG00000146094	DOK3	royalblue	0.58990536
ENSG00000125170	DOK4	purple	0.6997188
ENSG00000101134	DOK5	turquoise	0.67490937
ENSG00000206052	DOK6	purple	0.82757761
ENSG00000175920	DOK7	grey	0.10917237
ENSG00000175283	DOLK	greenyellow	-0.4383398
ENSG00000167130	DOLPP1	cyan	-0.435938
ENSG00000159147	DONSON	grey	0.01129533
ENSG00000083097	DOPEY1	turquoise	-0.6284187
ENSG00000142197	DOPEY2	turquoise	-0.5994133
ENSG00000172269	DPAGT1	grey	-0.0530088
ENSG00000166171	DPCD	green	-0.6519119
ENSG00000011332	DPF1	blue	0.4510242
ENSG00000133884	DPF2	grey	-0.0726957
ENSG00000205683	DPF3	turquoise	0.61592531
ENSG00000108963	DPH1	brown	0.65664218
ENSG00000132768	DPH2	grey	0.02630476
ENSG00000154813	DPH3	brown	-0.6705165

ENSG00000117543	DPH5	grey	-0.003387
ENSG00000134146	DPH6	grey	-0.2748104
ENSG00000248079	DPH6-AS1	grey	-0.0386388
ENSG00000000419	DPM1	red	0.62401823
ENSG00000136908	DPM2	grey	0.2823153
ENSG00000179085	DPM3	grey	-0.1470059
ENSG00000175497	DPP10	grey	-0.0076033
ENSG00000235026	DPP10-AS1	grey	0.1711775
ENSG00000235717	DPP10-AS2	grey	0.17138602
ENSG00000231538	DPP10-AS3	grey	0.08867775
ENSG00000254986	DPP3	grey	0.17922745
ENSG00000130226	DPP6	blue	0.78903051
ENSG00000176978	DPP7	brown	0.59339404
ENSG00000074603	DPP8	yellowgreen	0.64498527
ENSG00000269776	DPPA5P1	grey	0.3597237
ENSG00000173852	DPY19L1	turquoise	0.46139051
ENSG00000229358	DPY19L1P1	brown	-0.4034413
ENSG00000231952	DPY19L1P2	grey	0.39999202
ENSG00000177990	DPY19L2	blue	0.65012698
ENSG00000189212	DPY19L2P1	turquoise	-0.7210443
ENSG00000170629	DPY19L2P2	blue	0.61049507
ENSG00000227855	DPY19L2P3	grey	0.16735469
ENSG00000235436	DPY19L2P4	grey	0.12829503
ENSG00000178904	DPY19L3	turquoise	0.68036116
ENSG00000156162	DPY19L4	turquoise	0.75368737
ENSG00000162961	DPY30	grey	-0.167628
ENSG00000188641	DPYD	darkred	-0.6488859
ENSG00000232878	DPYD-AS1	yellow	0.45099493
ENSG00000232542	DPYD-IT1	grey	0.2884606
ENSG00000092964	DPYSL2	blue	-0.6251462
ENSG00000113657	DPYSL3	turquoise	0.56554823
ENSG00000151640	DPYSL4	grey	-0.1184179
ENSG00000157851	DPYSL5	floralwhite	0.69200565
ENSG00000117505	DR1	grey	0.10005719
ENSG00000136048	DRAM1	pink	0.62301942
ENSG00000156171	DRAM2	grey	-0.1409947
ENSG00000175550	DRAP1	salmon	0.48916837
ENSG00000184845	DRD1	magenta	0.84053251
ENSG00000149295	DRD2	darkorange	0.5649953
ENSG00000151577	DRD3	blue	0.44660909
ENSG00000169676	DRD5	grey	-0.0254558
ENSG00000185721	DRG1	grey	-0.0730993
ENSG00000108591	DRG2	grey	0.09866591
ENSG00000113360	DROSHA	turquoise	-0.7483504
ENSG00000102385	DRP2	blue	0.74404042
ENSG00000134755	DSC2	turquoise	0.38261193
ENSG00000171587	DSCAM	darkorange	0.65953482
ENSG00000233756	DSCAM-IT1	grey	-0.1174602

ENSG00000177103	DSCAML1	floralwhite	0.8779028
ENSG00000157538	DSCR3	grey	0.18381749
ENSG00000111817	DSE	pink	0.71889982
ENSG00000171451	DSEL	salmon	-0.6609973
ENSG00000149636	DSN1	salmon	0.58881022
ENSG00000096696	DSP	tan	0.5334254
ENSG00000151914	DST	blue	-0.6229212
ENSG00000230982	DSTNP1	grey	0.36632585
ENSG00000248593	DSTNP2	red	0.59046257
ENSG00000133059	DSTYK	grey	0.0411741
ENSG00000129480	DTD2	turquoise	0.4359685
ENSG00000134769	DTNA	turquoise	0.66669679
ENSG00000138101	DTNB	turquoise	-0.7624841
ENSG00000047579	DTNBP1	brown	0.81496125
ENSG00000104047	DTWD1	grey	0.02604108
ENSG00000135144	DTX1	red	0.57583261
ENSG00000091073	DTX2	turquoise	-0.5086495
ENSG00000186704	DTX2P1	grey	-0.095543
ENSG00000178498	DTX3	turquoise	-0.5841469
ENSG00000163840	DTX3L	pink	0.85840951
ENSG00000110042	DTX4	grey	-0.134534
ENSG00000168393	DTYMK	turquoise	0.33791084
ENSG00000169718	DUS1L	brown	0.61973018
ENSG00000167264	DUS2	brown	0.45989507
ENSG00000141994	DUS3L	brown	0.43695651
ENSG00000105865	DUS4L	grey	0.04878352
ENSG00000143507	DUSP10	yellow	0.69049227
ENSG00000144048	DUSP11	grey	-0.1876471
ENSG00000081721	DUSP12	grey	-0.0932409
ENSG00000161326	DUSP14	royalblue	-0.4323781
ENSG00000149599	DUSP15	grey	-0.0176914
ENSG00000111266	DUSP16	yellow	0.73337891
ENSG00000167065	DUSP18	grey	0.1041467
ENSG00000158050	DUSP2	royalblue	-0.4398906
ENSG00000112679	DUSP22	grey	-0.1317985
ENSG00000158716	DUSP23	grey	-0.1489761
ENSG00000133878	DUSP26	blue	0.77206171
ENSG00000188542	DUSP28	grey	-0.0961562
ENSG00000108861	DUSP3	magenta	-0.4525098
ENSG00000138166	DUSP5	grey	0.28643001
ENSG00000139318	DUSP6	grey	0.22117935
ENSG00000164086	DUSP7	yellow	0.65456961
ENSG00000184545	DUSP8	darkorange	0.66965319
ENSG00000235316	DUSP8P5	grey	0.16481387
ENSG00000130829	DUSP9	grey	0.00655463
ENSG00000128951	DUT	greenyellow	0.43264775
ENSG00000229048	DUTP1	grey	0.47799854
ENSG00000226857	DUTP3	grey	0.35705967

ENSG00000225171	DUTP6	grey	0.14501821
ENSG00000250473	DUTP7	grey	0.40310182
ENSG00000107404	DVL1	brown	0.51306367
ENSG00000004975	DVL2	grey	0.03093142
ENSG00000161202	DVL3	brown	-0.6103498
ENSG00000204348	DXO	grey	0.19018167
ENSG00000141627	DYM	grey	0.15445601
ENSG00000197102	DYNC1H1	brown	0.53760972
ENSG00000158560	DYNC1I1	turquoise	-0.7219791
ENSG00000077380	DYNC1I2	grey	0.10002776
ENSG00000144635	DYNC1LI1	grey	0.10638089
ENSG00000135720	DYNC1LI2	green	0.91515583
ENSG00000138036	DYNC2LI1	blue	0.56756799
ENSG00000088986	DYNLL1	black	-0.5572393
ENSG00000248008	DYNLL1-AS1	grey	0.04489625
ENSG00000264364	DYNLL2	darkgrey	0.56123376
ENSG00000125971	DYNLRB1	blue	0.65404262
ENSG00000146425	DYNLT1	blue	-0.4082287
ENSG00000165169	DYNLT3	grey	-0.1347871
ENSG00000157540	DYRK1A	yellow	-0.5472605
ENSG00000105204	DYRK1B	blue	0.53582653
ENSG00000010219	DYRK4	brown	0.39680634
ENSG00000135636	DYSF	yellow	0.78198942
ENSG00000089091	DZANK1	blue	0.64068276
ENSG00000134874	DZIP1	black	-0.7183002
ENSG00000198919	DZIP3	blue	0.47422565
ENSG00000101412	E2F1	yellow	0.31697168
ENSG00000112242	E2F3	grey	-0.1982362
ENSG00000224707	E2F3-IT1	grey	0.27271777
ENSG00000205250	E2F4	grey	0.10877408
ENSG00000169016	E2F6	blue	-0.4295368
ENSG00000167967	E4F1	yellow	-0.4687976
ENSG00000144597	EAF1	grey	0.06641125
ENSG00000145088	EAF2	turquoise	0.53515124
ENSG00000129518	EAPP	grey	-0.0263616
ENSG00000103356	EARS2	grey	0.15769581
ENSG00000147654	EBAG9	grey	-0.3202832
ENSG00000233690	EBAG9P1	grey	0.22924298
ENSG00000164330	EBF1	grey	0.04965538
ENSG00000088881	EBF4	grey	-0.0374113
ENSG00000255423	EBLN2	grey	0.24163951
ENSG00000117395	EBNA1BP2	brown	0.8362422
ENSG00000147155	EBP	blue	0.65252063
ENSG00000123179	EBPL	yellow	-0.3304447
ENSG00000122882	ECD	brown	0.31280119
ENSG00000117298	ECE1	purple	-0.8257487
ENSG00000145194	ECE2	blue	0.5796879
ENSG00000171551	ECEL1	grey	0.00305332

ENSG00000104823	ECH1	turquoise	0.5716436
ENSG00000093144	ECHDC1	grey	-0.3279125
ENSG00000121310	ECHDC2	grey	-0.2315016
ENSG00000127884	ECHS1	darkred	0.58472218
ENSG00000167969	ECI1	grey	0.0562013
ENSG00000198721	ECI2	salmon	0.71833754
ENSG00000143369	ECM1	tan	0.49713479
ENSG00000106823	ECM2	grey	-0.1454615
ENSG00000249751	ECSCR	tan	0.60223574
ENSG00000130159	ECSIT	brown	0.66817631
ENSG00000114346	ECT2	grey	0.18163574
ENSG00000179151	EDC3	green	-0.526058
ENSG00000038358	EDC4	turquoise	-0.5661103
ENSG00000088298	EDEM2	grey	0.29497064
ENSG00000116406	EDEM3	turquoise	0.43339396
ENSG00000107223	EDF1	blue	0.6012226
ENSG00000164176	EDIL3	yellow	0.72695545
ENSG00000078401	EDN1	tan	0.68053395
ENSG00000124205	EDN3	tan	0.42561253
ENSG00000151617	EDNRA	brown	-0.394533
ENSG00000136160	EDNRB	turquoise	0.6562884
ENSG00000107938	EDRF1	brown	0.45018152
ENSG00000236991	EDRF1-AS1	grey	0.20415636
ENSG00000102189	EEA1	grey	-0.2006392
ENSG00000074266	EED	grey	-0.0123372
ENSG00000156508	EEF1A1	greenyellow	0.70749344
ENSG00000233167	EEF1A1P26	black	0.4816712
ENSG00000237757	EEF1A1P30	grey	0.32876037
ENSG00000245205	EEF1A1P4	grey	0.37074003
ENSG00000196205	EEF1A1P5	grey	0.04385904
ENSG00000233476	EEF1A1P6	grey	0.22905305
ENSG00000101210	EEF1A2	blue	0.71420286
ENSG00000114942	EEF1B2	greenyellow	0.78988924
ENSG00000231169	EEF1B2P1	grey	0.17998339
ENSG00000232472	EEF1B2P3	grey	0.03318891
ENSG00000104529	EEF1D	greenyellow	0.6693753
ENSG00000229715	EEF1DP3	red	-0.5513835
ENSG00000124802	EEF1E1	yellow	-0.4393506
ENSG00000167658	EEF2	turquoise	-0.4086458
ENSG00000103319	EEF2K	grey	0.19994384
ENSG00000132394	EEFSEC	blue	0.41226268
ENSG00000122547	EEPD1	black	0.63490746
ENSG00000140025	EFCAB11	grey	0.22546847
ENSG00000178852	EFCAB13	yellow	0.37170151
ENSG00000159658	EFCAB14	turquoise	0.61984527
ENSG00000228237	EFCAB14-AS1	grey	0.34488993
ENSG00000203666	EFCAB2	grey	0.05245128
ENSG00000177685	EFCAB4A	grey	-0.0676771

ENSG00000203965	EFCAB7	blue	0.44823007
ENSG00000115380	EFEMP1	turquoise	0.55811472
ENSG00000172638	EFEMP2	turquoise	0.71207441
ENSG00000096093	EFHC1	brown	0.55748354
ENSG00000183690	EFHC2	turquoise	0.37621299
ENSG00000115468	EFHD1	yellow	0.8357919
ENSG00000142634	EFHD2	grey	-0.1027937
ENSG00000169242	EFNA1	pink	0.70165726
ENSG00000099617	EFNA2	grey	0.1620347
ENSG00000143590	EFNA3	blue	0.47909918
ENSG00000243364	EFNA4	blue	-0.5214918
ENSG00000184349	EFNA5	yellow	-0.4203061
ENSG00000090776	EFNB1	grey	0.15007907
ENSG00000125266	EFNB2	purple	-0.489736
ENSG00000108947	EFNB3	blue	0.62643801
ENSG00000132294	EFR3A	darkmagenta	-0.5826911
ENSG00000084710	EFR3B	black	0.85284385
ENSG00000100842	EFS	yellow	0.76295476
ENSG00000140598	EFTUD1	salmon	0.53649394
ENSG00000259404	EFTUD1P1	grey	0.15198966
ENSG00000108883	EFTUD2	brown	0.81379269
ENSG00000206120	EGFEM1P	purple	0.74956368
ENSG00000172889	EGFL7	tan	0.77264874
ENSG00000146648	EGFR	turquoise	0.75420619
ENSG00000224057	EGFR-AS1	grey	0.36644488
ENSG00000135766	EGLN1	turquoise	0.67070539
ENSG00000269858	EGLN2	turquoise	-0.6033498
ENSG00000129521	EGLN3	blue	-0.3561479
ENSG00000235947	EGOT	black	0.55131642
ENSG00000120738	EGR1	grey	-0.0551022
ENSG00000122877	EGR2	grey	-0.0777057
ENSG00000179388	EGR3	magenta	0.7338985
ENSG00000135625	EGR4	grey	0.07271918
ENSG00000115504	EHBP1	yellow	-0.4636375
ENSG00000173442	EHBP1L1	royalblue	0.65880073
ENSG00000110047	EHD1	grey	0.12972375
ENSG00000024422	EHD2	turquoise	0.61678541
ENSG00000013016	EHD3	yellow	-0.4932099
ENSG00000103966	EHD4	pink	0.67696435
ENSG00000113790	EHHADH	turquoise	0.770603
ENSG00000181090	EHMT1	turquoise	0.44275425
ENSG00000204371	EHMT2	blue	0.47627855
ENSG00000149547	EI24	grey	0.03406017
ENSG00000255302	EID1	grey	-0.1754314
ENSG00000176396	EID2	green	-0.5786535
ENSG00000176401	EID2B	brown	0.62325718
ENSG00000255150	EID3	grey	0.32620669
ENSG00000173812	EIF1	lightcyan	-0.7081983

ENSG00000175376	EIF1AD	grey	-0.1593911
ENSG00000173674	EIF1AX	lightcyan	0.64212724
ENSG00000258980	EIF1AXP2	greenyellow	-0.4653506
ENSG00000198692	EIF1AY	grey	-0.0939774
ENSG00000114784	EIF1B	green	-0.752199
ENSG00000086232	EIF2AK1	grey	-0.0305727
ENSG00000055332	EIF2AK2	red	0.75213569
ENSG00000172071	EIF2AK3	grey	0.19946961
ENSG00000128829	EIF2AK4	brown	0.70002447
ENSG00000111361	EIF2B1	grey	-0.0557388
ENSG00000119718	EIF2B2	grey	0.41044397
ENSG00000070785	EIF2B3	turquoise	-0.6613239
ENSG00000115211	EIF2B4	salmon	0.60036049
ENSG00000145191	EIF2B5	turquoise	-0.7612581
ENSG00000143486	EIF2D	yellow	0.33941017
ENSG00000125977	EIF2S2	salmon	0.59772458
ENSG00000225650	EIF2S2P5	grey	0.34292226
ENSG00000130741	EIF2S3	grey	0.15521545
ENSG00000107581	EIF3A	darkgrey	0.71891173
ENSG00000106263	EIF3B	brown	0.77718482
ENSG00000184110	EIF3C	grey	-0.0918148
ENSG00000100353	EIF3D	brown	0.71501765
ENSG00000234882	EIF3EP1	grey	0.06892135
ENSG00000175390	EIF3F	grey	-0.1636786
ENSG00000130811	EIF3G	grey	0.07843649
ENSG00000147677	EIF3H	greenyellow	0.68233936
ENSG00000104131	EIF3J	brown	-0.6568886
ENSG00000179523	EIF3J-AS1	grey	0.22163859
ENSG00000178982	EIF3K	brown	0.68634901
ENSG00000175749	EIF3KP1	grey	0.22158724
ENSG00000100129	EIF3L	blue	0.70055786
ENSG00000149100	EIF3M	darkred	0.68136818
ENSG00000231564	EIF4A1P11	grey	0.38320967
ENSG00000213484	EIF4A1P8	grey	0.21514967
ENSG00000141543	EIF4A3	green	-0.6016052
ENSG00000063046	EIF4B	grey	-0.2695176
ENSG00000228753	EIF4BP2	grey	0.17794324
ENSG00000261336	EIF4BP5	grey	0.38815727
ENSG00000197258	EIF4BP6	grey	0.3612189
ENSG00000151247	EIF4E	brown	-0.2086345
ENSG00000135930	EIF4E2	salmon	0.55493063
ENSG00000163412	EIF4E3	bisque4	0.74187285
ENSG00000187840	EIF4EBP1	pink	0.49819232
ENSG00000148730	EIF4EBP2	turquoise	0.85872931
ENSG00000220505	EIF4EBP2P3	grey	0.15640014
ENSG00000184708	EIF4ENIF1	grey	0.05485284
ENSG00000114867	EIF4G1	turquoise	0.54796023
ENSG00000075151	EIF4G3	turquoise	-0.4834659

ENSG00000106682	EIF4H	grey	-0.1135379
ENSG00000237977	EIF4HP2	grey	0.22598343
ENSG00000100664	EIF5	grey	-0.1237022
ENSG00000132507	EIF5A	brown	-0.4291988
ENSG00000163577	EIF5A2	lightcyan	0.57393593
ENSG00000267346	EIF5AP3	grey	0.32604147
ENSG00000158417	EIF5B	darkgrey	0.82084254
ENSG00000242372	EIF6	darkred	0.60847905
ENSG00000141642	ELAC1	turquoise	0.48272613
ENSG00000006744	ELAC2	brown	0.8472671
ENSG00000066044	ELAVL1	grey	-0.2301819
ENSG00000120690	ELF1	yellow	0.69293817
ENSG00000109381	ELF2	red	-0.6956113
ENSG00000225968	ELFN1	magenta	0.41437106
ENSG00000166897	ELFN2	darkorange	0.74280686
ENSG00000126767	ELK1	grey	-0.1020277
ENSG00000111145	ELK3	turquoise	0.41101294
ENSG00000158711	ELK4	brown	-0.8642626
ENSG00000105656	ELL	yellowgreen	-0.540111
ENSG00000118985	ELL2	yellow	0.73095895
ENSG00000155849	ELMO1	blue	0.71920526
ENSG00000224101	ELMO1-AS1	yellow	0.64205292
ENSG00000062598	ELMO2	grey	0.45539291
ENSG00000110675	ELMOD1	blue	0.74868658
ENSG00000179387	ELMOD2	grey	-0.0727663
ENSG00000115459	ELMOD3	turquoise	-0.4574587
ENSG00000156030	ELMSAN1	pink	0.55681055
ENSG00000049540	ELN	grey	-0.1828972
ENSG00000130165	ELOF1	greenyellow	0.54781779
ENSG00000066322	ELOVL1	yellow	0.86672286
ENSG00000197977	ELOVL2	turquoise	0.79720649
ENSG00000170522	ELOVL6	grey	0.11849178
ENSG00000164181	ELOVL7	tan	0.8072112
ENSG00000134759	ELP2	grey	0.02488995
ENSG00000134014	ELP3	salmon	0.70651018
ENSG00000170291	ELP5	grey	0.04720768
ENSG00000163832	ELP6	brown	0.48596252
ENSG00000162618	ELTD1	tan	0.83085295
ENSG00000170571	EMB	grey	0.16313778
ENSG00000127463	EMC1	grey	0.17871975
ENSG00000161671	EMC10	lightcyan	0.55118413
ENSG00000104412	EMC2	grey	-0.0166593
ENSG00000125037	EMC3	brown	0.74879651
ENSG00000180385	EMC3-AS1	grey	0.18826258
ENSG00000128463	EMC4	blue	0.71359051
ENSG00000127774	EMC6	grey	0.28602465
ENSG00000131148	EMC8	grey	-0.0443587
ENSG00000164035	EMCN	tan	0.70067554

ENSG00000102119	EMD	greenyellow	0.39054201
ENSG00000197774	EME2	blue	0.41614072
ENSG00000126749	EMG1	grey	0.14575991
ENSG00000186998	EMID1	grey	-0.3227026
ENSG00000066629	EML1	red	-0.6009666
ENSG00000125746	EML2	grey	-0.2127407
ENSG00000143924	EML4	grey	-0.0143798
ENSG00000165521	EML5	grey	0.25227034
ENSG00000214595	EML6	grey	0.0487591
ENSG00000134531	EMP1	pink	0.76393015
ENSG00000213853	EMP2	tan	0.69689611
ENSG00000142227	EMP3	grey	-0.0278146
ENSG00000170370	EMX2	turquoise	0.65467985
ENSG00000229847	EMX2OS	turquoise	0.64236553
ENSG00000154380	ENAH	salmon	-0.7558581
ENSG00000167136	ENDOG	grey	0.19778436
ENSG00000111405	ENDOU	blue	-0.3777177
ENSG00000173818	ENDOV	black	-0.5835555
ENSG00000106991	ENG	tan	0.86883877
ENSG00000167280	ENGASE	grey	0.17503146
ENSG00000168913	ENHO	turquoise	0.58701483
ENSG00000124074	ENKD1	grey	-0.0782702
ENSG00000151023	ENKUR	turquoise	0.5596282
ENSG00000074800	ENO1	darkred	0.70218433
ENSG00000236269	ENO1-IT1	brown	-0.4441071
ENSG00000111674	ENO2	blue	0.75224412
ENSG00000188316	ENO4	grey	0.0286886
ENSG00000145293	ENOPH1	grey	-0.0043095
ENSG00000132199	ENOSF1	yellow	0.47951366
ENSG00000120658	ENOX1	grey	0.01410008
ENSG00000233821	ENOX1-AS1	grey	0.1482263
ENSG00000165675	ENOX2	red	-0.505429
ENSG00000136960	ENPP2	yellow	0.77331893
ENSG00000001561	ENPP4	yellow	0.71089715
ENSG00000112796	ENPP5	grey	0.1404114
ENSG00000164303	ENPP6	darkmagenta	0.57568222
ENSG00000249188	ENPP7P1	purple	0.40352921
ENSG00000249767	ENPP7P10	grey	0.30065983
ENSG00000254527	ENPP7P12	grey	0.11820366
ENSG00000239959	ENPP7P2	grey	0.06505258
ENSG00000241278	ENPP7P4	grey	-0.0584597
ENSG00000256589	ENPP7P5	grey	0.0661753
ENSG00000255549	ENPP7P6	grey	0.30328755
ENSG00000255319	ENPP7P8	grey	0.09404632
ENSG00000143420	ENSA	turquoise	-0.6816501
ENSG00000167302	ENTHD2	grey	-0.1389686
ENSG00000138185	ENTPD1	turquoise	0.41422309
ENSG00000226688	ENTPD1-AS1	grey	0.4735937

ENSG00000054179	ENTPD2	grey	-0.1351715
ENSG00000168032	ENTPD3	black	0.83503693
ENSG00000223797	ENTPD3-AS1	red	-0.7598215
ENSG00000197217	ENTPD4	turquoise	-0.5841267
ENSG00000187097	ENTPD5	lightcyan	0.5439841
ENSG00000197586	ENTPD6	brown	0.84579121
ENSG00000198018	ENTPD7	black	0.50294119
ENSG00000163378	EOGT	tan	0.54605183
ENSG00000100393	EP300	grey	-0.0067488
ENSG00000183495	EP400	turquoise	-0.507432
ENSG00000185684	EP400NL	grey	-0.0669789
ENSG00000116016	EPAS1	tan	0.78541513
ENSG00000159023	EPB41	black	0.89591266
ENSG00000079819	EPB41L2	red	-0.7098908
ENSG00000082397	EPB41L3	grey	-0.1175888
ENSG00000129595	EPB41L4A	grey	-0.0119167
ENSG00000095203	EPB41L4B	purple	0.88516253
ENSG00000115109	EPB41L5	turquoise	0.76158639
ENSG00000166947	EPB42	grey	0.20169055
ENSG00000120616	EPC1	grey	-0.0531295
ENSG00000135999	EPC2	brown	-0.4310967
ENSG00000086289	EPDR1	turquoise	0.48438034
ENSG00000152223	EPG5	brown	0.62877914
ENSG00000183317	EPHA10	blue	0.67825604
ENSG00000044524	EPHA3	grey	0.06991601
ENSG00000145242	EPHA5	grey	0.04548314
ENSG00000135333	EPHA7	turquoise	-0.5233821
ENSG00000154928	EPHB1	turquoise	0.41777912
ENSG00000133216	EPHB2	grey	-0.0313675
ENSG00000182580	EPHB3	red	0.5687013
ENSG00000106123	EPHB6	grey60	0.76694737
ENSG00000143819	EPHX1	turquoise	0.57559769
ENSG00000120915	EPHX2	turquoise	0.49595742
ENSG00000112425	EPM2A	bisque4	0.72056194
ENSG00000178567	EPM2AIP1	grey	-0.189338
ENSG00000063245	EPN1	turquoise	-0.5931534
ENSG00000072134	EPN2	grey	-0.2072804
ENSG00000235397	EPN2-AS1	grey	0.00582808
ENSG00000187266	EPOR	blue	-0.3521952
ENSG00000136628	EPRS	salmon	0.49999518
ENSG00000085832	EPS15	salmon	-0.4309353
ENSG00000151491	EPS8	turquoise	0.80040015
ENSG00000133106	EPSTI1	tan	0.6492628
ENSG00000138018	EPT1	green	-0.6575516
ENSG00000121053	EPX	brown	-0.2812
ENSG00000120160	EQTN	grey	0.23163149
ENSG00000132591	ERAL1	grey	0.25121883
ENSG00000164307	ERAP1	grey	0.03832153

ENSG00000164308	ERAP2	grey	-0.0186287
ENSG00000141736	ERBB2	turquoise	0.33003735
ENSG00000112851	ERBB2IP	green	0.8878153
ENSG00000065361	ERBB3	yellow	0.83929469
ENSG00000178568	ERBB4	magenta	0.51124717
ENSG00000082805	ERC1	grey	-0.0988607
ENSG00000187672	ERC2	purple	0.70180313
ENSG00000012061	ERCC1	yellow	-0.5165423
ENSG00000104884	ERCC2	darkred	0.56436495
ENSG00000163161	ERCC3	brown	0.79334177
ENSG00000175595	ERCC4	grey	0.49124135
ENSG00000134899	ERCC5	turquoise	-0.4166996
ENSG00000182150	ERCC6L2	turquoise	-0.5976989
ENSG00000049167	ERCC8	grey	0.2122644
ENSG00000105722	ERF	red	-0.5953626
ENSG00000157554	ERG	tan	0.87143832
ENSG00000113719	ERGIC1	magenta	-0.3950416
ENSG00000087502	ERGIC2	grey	0.26137235
ENSG00000125991	ERGIC3	green	-0.3987883
ENSG00000100632	ERH	grey	-0.2380446
ENSG00000104626	ERI1	blue	-0.485388
ENSG00000117419	ERI3	green	-0.4692144
ENSG00000233602	ERI3-IT1	grey	0.31387007
ENSG00000104714	ERICH1	grey	-0.0444451
ENSG00000237647	ERICH1-AS1	grey	-0.0303631
ENSG00000204334	ERICH2	blue	0.39313966
ENSG00000068912	ERLEC1	grey	-0.0018517
ENSG00000107566	ERLIN1	salmon	0.63779966
ENSG00000147475	ERLIN2	turquoise	0.79038321
ENSG00000136541	ERMN	yellow	0.83401377
ENSG00000099219	ERMP1	yellow	0.500453
ENSG00000178607	ERN1	grey	0.45274237
ENSG00000197930	ERO1L	grey	0.20306889
ENSG00000086619	ERO1LB	grey	-0.1154942
ENSG00000089248	ERP29	brown	0.67987429
ENSG00000023318	ERP44	grey	0.1522473
ENSG00000116285	ERRFI1	blue	-0.5502165
ENSG00000213462	ERV3-1	grey	-0.0766762
ENSG00000233056	ERVH48-1	grey	0.09560972
ENSG00000260565	ERVK13-1	grey	-0.1189295
ENSG00000142396	ERVK3-1	grey	0.0198559
ENSG00000242950	ERVW-1	yellow	0.6131474
ENSG00000149564	ESAM	tan	0.87148991
ENSG00000141446	ESCO1	salmon	-0.6964483
ENSG00000139684	ESD	turquoise	0.33941206
ENSG00000089048	ESF1	bisque4	0.88410583
ENSG00000265992	ESRG	grey	-0.1662155
ENSG00000173153	ESRRA	turquoise	0.5547323

ENSG00000139641	ESYT1	magenta	-0.509248
ENSG00000117868	ESYT2	tan	0.66006193
ENSG00000158220	ESYT3	magenta	-0.5266391
ENSG00000143971	ETAA1	grey	0.10425668
ENSG00000120705	ETF1	grey	0.37496387
ENSG00000240132	ETF1P2	grey	0.37518965
ENSG00000105379	ETFB	grey	-0.2172605
ENSG00000171503	ETFDH	turquoise	0.5596039
ENSG00000105755	ETHE1	grey	0.08902186
ENSG00000139163	ETNK1	grey	-0.3367614
ENSG00000143845	ETNK2	grey	0.04522768
ENSG00000164089	ETNPPL	turquoise	0.52346093
ENSG00000134954	ETS1	brown	-0.6448245
ENSG00000157557	ETS2	tan	0.64964882
ENSG00000006468	ETV1	red	0.62872772
ENSG00000117036	ETV3	bisque4	0.7075981
ENSG00000244405	ETV5	pink	-0.4499215
ENSG00000234197	ETV5-AS1	grey	0.0018736
ENSG00000139083	ETV6	pink	0.66415672
ENSG00000166979	EVA1C	yellowgreen	-0.6906288
ENSG00000072840	EVC	turquoise	0.55767559
ENSG00000126860	EVI2A	yellow	0.83218066
ENSG00000185862	EVI2B	royalblue	0.6640715
ENSG00000067208	EVI5	brown	-0.5940703
ENSG00000142459	EVI5L	turquoise	-0.4135104
ENSG00000196405	EVL	blue	0.75619104
ENSG00000182944	EWSR1	grey	-0.3087779
ENSG00000081177	EXD2	turquoise	0.66975418
ENSG00000187609	EXD3	turquoise	-0.3646114
ENSG00000164002	EXO5	grey	-0.0058484
ENSG00000090989	EXOC1	grey	-0.2271549
ENSG00000112685	EXOC2	grey	-0.0162653
ENSG00000180104	EXOC3	grey	0.17652563
ENSG00000131558	EXOC4	turquoise	-0.6831211
ENSG00000070367	EXOC5	grey	0.05500995
ENSG00000138190	EXOC6	grey	-0.3652843
ENSG00000144036	EXOC6B	grey	0.15023153
ENSG00000182473	EXOC7	blue	0.47504692
ENSG00000116903	EXOC8	grey	0.22091033
ENSG00000171311	EXOSC1	turquoise	-0.5971457
ENSG00000171824	EXOSC10	grey	-0.1373707
ENSG00000130713	EXOSC2	brown	0.74379356
ENSG00000229007	EXOSC3P1	grey	0.30012622
ENSG00000178896	EXOSC4	grey	-0.0014067
ENSG00000077348	EXOSC5	brown	0.54229464
ENSG00000075914	EXOSC7	salmon	0.72737398
ENSG00000120699	EXOSC8	grey	-0.146303
ENSG00000123737	EXOSC9	grey	-0.1719932

ENSG00000110723	EXPH5	black	0.49813194
ENSG00000182197	EXT1	grey	0.04818437
ENSG00000151348	EXT2	grey	0.02294734
ENSG00000158008	EXTL1	blue	0.59450109
ENSG00000162694	EXTL2	grey60	0.75846766
ENSG00000012232	EXTL3	brown	0.62791899
ENSG00000246339	EXTL3-AS1	grey	0.01557601
ENSG00000104313	EYA1	grey	-0.2602231
ENSG00000064655	EYA2	turquoise	0.63074656
ENSG00000112319	EYA4	grey	0.24264512
ENSG00000108799	EZH1	turquoise	-0.5152906
ENSG00000106462	EZH2	grey	0.23398692
ENSG00000092820	EZR	turquoise	0.70366293
ENSG00000158769	F11R	tan	0.6854
ENSG00000124491	F13A1	grey	-0.1157992
ENSG00000181104	F2R	brown	-0.3777609
ENSG00000117525	F3	turquoise	0.5756758
ENSG00000198734	F5	grey	-0.1673312
ENSG00000185010	F8	turquoise	0.4876453
ENSG00000197932	F8A1	grey	0.03543178
ENSG00000103089	FA2H	yellow	0.79685893
ENSG00000117480	FAAH	darkmagenta	0.64333902
ENSG00000165591	FAAH2	grey	0.06755946
ENSG00000121769	FABP3	purple	0.72634077
ENSG00000164687	FABP5	grey	-0.1689934
ENSG00000236044	FABP5P2	grey	0.50151624
ENSG00000229287	FABP5P4	grey	0.18770199
ENSG00000226766	FABP7P1	grey	0.39721277
ENSG00000168040	FADD	grey	0.22662819
ENSG00000149485	FADS1	turquoise	0.5506415
ENSG00000134824	FADS2	turquoise	0.39729937
ENSG00000221968	FADS3	grey	-0.1116071
ENSG00000172782	FADS6	yellow	-0.5934098
ENSG00000185104	FAF1	grey	-0.0192855
ENSG00000113194	FAF2	grey	0.27552472
ENSG00000103876	FAH	turquoise	0.59309395
ENSG00000180185	FAHD1	grey	0.13931014
ENSG00000115042	FAHD2A	turquoise	-0.5254574
ENSG00000144199	FAHD2B	turquoise	-0.4347214
ENSG00000231584	FAHD2CP	yellow	0.47394745
ENSG00000158234	FAIM	turquoise	0.45985263
ENSG00000135472	FAIM2	blue	0.69266206
ENSG00000183688	FAM101B	blue	-0.3918046
ENSG00000167106	FAM102A	yellow	0.47165961
ENSG00000162636	FAM102B	purple	0.65330091
ENSG00000169612	FAM103A1	grey	-0.0106856
ENSG00000133193	FAM104A	grey	0.13456201
ENSG00000182518	FAM104B	turquoise	0.38312997

ENSG00000145569	FAM105A	cyan	0.80518053
ENSG00000154124	FAM105B	blue	-0.7367367
ENSG00000205217	FAM106B	grey	0.09186917
ENSG00000168309	FAM107A	blue	-0.6561952
ENSG00000065809	FAM107B	yellow	0.82755782
ENSG00000125898	FAM110A	turquoise	-0.4159326
ENSG00000169122	FAM110B	yellow	-0.5877814
ENSG00000166801	FAM111A	grey	-0.0933313
ENSG00000197712	FAM114A1	yellow	0.52260333
ENSG00000055147	FAM114A2	grey	0.17180658
ENSG00000223459	FAM115B	brown	0.4226872
ENSG00000121104	FAM117A	blue	0.44023953
ENSG00000138439	FAM117B	darkorange	0.57059843
ENSG00000100376	FAM118A	grey	4.24E-05
ENSG00000188938	FAM120AOS	lightcyan	0.68035199
ENSG00000112584	FAM120B	lightcyan	0.70833696
ENSG00000184083	FAM120C	grey60	-0.6330612
ENSG00000187866	FAM122A	grey	0.17865736
ENSG00000156504	FAM122B	bisque4	0.7049102
ENSG00000156500	FAM122C	brown	0.45029808
ENSG00000150510	FAM124A	yellow	0.86118456
ENSG00000122591	FAM126A	green	0.71922906
ENSG00000155744	FAM126B	brown	0.58358871
ENSG00000134590	FAM127A	purple	0.67607146
ENSG00000203950	FAM127B	lightcyan	0.65449105
ENSG00000135842	FAM129A	royalblue	0.66135953
ENSG00000136830	FAM129B	pink	0.51337106
ENSG00000159784	FAM131B	red	-0.6051924
ENSG00000185519	FAM131C	grey	0.14124685
ENSG00000234545	FAM133B	purple	-0.5197051
ENSG00000144567	FAM134A	brown	0.69735223
ENSG00000154153	FAM134B	yellowgreen	-0.6033461
ENSG00000141699	FAM134C	grey	0.16743696
ENSG00000082269	FAM135A	turquoise	0.54371829
ENSG00000147724	FAM135B	grey60	0.7222524
ENSG00000035141	FAM136A	floralwhite	-0.4964267
ENSG00000138640	FAM13A	grey	0.18092538
ENSG00000248019	FAM13A-AS1	grey	0.05673083
ENSG00000031003	FAM13B	grey	-0.1713337
ENSG00000148541	FAM13C	yellow	0.66791623
ENSG00000109794	FAM149A	blue	0.53076609
ENSG00000138286	FAM149B1	brown	-0.6924413
ENSG00000152380	FAM151B	grey	0.07462676
ENSG00000170074	FAM153A	grey	-0.1049165
ENSG00000182230	FAM153B	grey	-0.1537083
ENSG00000204677	FAM153C	grey	-0.2302514
ENSG00000188659	FAM154B	grey	-0.0399251
ENSG00000204442	FAM155A	grey	0.27036381

ENSG00000227248	FAM155A-IT1	grey	0.29128824
ENSG00000130054	FAM155B	grey	0.0581619
ENSG00000260528	FAM157C	grey	0.07930459
ENSG00000051009	FAM160A2	grey	-0.2200115
ENSG00000151553	FAM160B1	grey	0.02105831
ENSG00000158863	FAM160B2	grey	0.28156144
ENSG00000170264	FAM161A	grey	-0.2211411
ENSG00000156050	FAM161B	yellow	-0.6921568
ENSG00000114023	FAM162A	yellow	-0.5410046
ENSG00000183807	FAM162B	turquoise	0.37164613
ENSG00000154319	FAM167A	turquoise	0.73358719
ENSG00000054965	FAM168A	grey	-0.0324372
ENSG00000152102	FAM168B	black	0.65459354
ENSG00000198780	FAM169A	yellow	-0.3948159
ENSG00000148468	FAM171A1	black	0.5659513
ENSG00000161682	FAM171A2	grey	0.10954889
ENSG00000144369	FAM171B	turquoise	0.60884309
ENSG00000113391	FAM172A	grey	0.01447963
ENSG00000103254	FAM173A	grey	-0.016488
ENSG00000150756	FAM173B	grey	0.25883143
ENSG00000174132	FAM174A	grey	0.08228667
ENSG00000185442	FAM174B	blue	0.38841475
ENSG00000163322	FAM175A	grey	0.19450163
ENSG00000165660	FAM175B	grey	0.13043627
ENSG00000151327	FAM177A1	green	0.77299702
ENSG00000197520	FAM177B	cyan	0.75410815
ENSG00000119906	FAM178A	green	0.51667478
ENSG00000168754	FAM178B	yellow	0.6090986
ENSG00000198718	FAM179B	brown	0.65744441
ENSG00000196666	FAM180B	magenta	-0.3622835
ENSG00000140067	FAM181A	turquoise	0.29590708
ENSG00000182103	FAM181B	turquoise	0.59573136
ENSG00000125804	FAM182A	grey	0.02018117
ENSG00000175170	FAM182B	grey	0.20679729
ENSG00000111879	FAM184A	grey	0.02220125
ENSG00000047662	FAM184B	brown	-0.6421993
ENSG00000222011	FAM185A	grey	0.32176358
ENSG00000214439	FAM185BP	cyan	-0.437708
ENSG00000148481	FAM188A	turquoise	-0.5693133
ENSG00000106125	FAM188B	grey	0.15632578
ENSG00000214237	FAM188B2	purple	0.53795449
ENSG00000104059	FAM189A1	blue	0.4775638
ENSG00000135063	FAM189A2	pink	0.68850363
ENSG00000160767	FAM189B	brown	0.4993655
ENSG00000172775	FAM192A	grey	0.09426837
ENSG00000125386	FAM193A	grey	0.09453864
ENSG00000146067	FAM193B	grey	0.02193315
ENSG00000225663	FAM195B	grey	0.22358265

ENSG00000188916	FAM196A	grey	-0.0805834
ENSG00000204767	FAM196B	yellow	0.64251697
ENSG00000144649	FAM198A	turquoise	0.66305758
ENSG00000164125	FAM198B	turquoise	0.79227486
ENSG00000123575	FAM199X	turquoise	0.83361451
ENSG00000219438	FAM19A5	brown	-0.6209709
ENSG00000221909	FAM200A	turquoise	0.54440889
ENSG00000237765	FAM200B	brown	-0.4383338
ENSG00000204860	FAM201A	grey	-0.3998416
ENSG00000230992	FAM201B	grey	-0.0370397
ENSG00000233040	FAM204BP	grey	0.48863412
ENSG00000119328	FAM206A	grey	-0.151772
ENSG00000160256	FAM207A	brown	0.5540685
ENSG00000163946	FAM208A	blue	-0.6259307
ENSG00000108021	FAM208B	grey	0.26132487
ENSG00000116199	FAM20B	blue	0.34573591
ENSG00000177706	FAM20C	turquoise	-0.3672769
ENSG00000124098	FAM210B	red	0.74453343
ENSG00000181350	FAM211A	turquoise	0.54535911
ENSG00000175061	FAM211A-AS1	lightcyan	0.5399381
ENSG00000178026	FAM211B	grey	0.16732328
ENSG00000185614	FAM212A	yellow	-0.3269233
ENSG00000197852	FAM212B	grey	-0.1218723
ENSG00000122378	FAM213A	turquoise	0.62500128
ENSG00000157870	FAM213B	purple	0.45792866
ENSG00000047346	FAM214A	yellow	0.64871815
ENSG00000005238	FAM214B	grey	0.06506858
ENSG00000232300	FAM215B	grey	0.02500443
ENSG00000204856	FAM216A	blue	0.53603534
ENSG00000196227	FAM217B	turquoise	0.34214292
ENSG00000250486	FAM218A	grey	0.11624998
ENSG00000164970	FAM219A	green	0.71032542
ENSG00000178761	FAM219B	brown	0.43590069
ENSG00000099290	FAM21A	grey	-0.0561945
ENSG00000172661	FAM21C	grey	0.05709595
ENSG00000178397	FAM220A	grey	0.05684928
ENSG00000188732	FAM221A	blue	-0.4347778
ENSG00000139438	FAM222A	floralwhite	0.76754731
ENSG00000255650	FAM222A-AS1	yellow	0.46770878
ENSG00000173065	FAM222B	grey	0.08628642
ENSG00000269911	FAM226B	grey	0.10456869
ENSG00000184949	FAM227A	grey	0.19029241
ENSG00000166262	FAM227B	grey	0.09341111
ENSG00000186453	FAM228A	grey	0.25861295
ENSG00000219626	FAM228B	grey	-0.1137246
ENSG00000225828	FAM229A	grey	0.21688254
ENSG00000203778	FAM229B	brown	-0.5958922
ENSG00000203815	FAM231D	grey	0.21298221

ENSG00000213185	FAM24B	grey	-0.1037446
ENSG00000182368	FAM27A	grey	0.12044804
ENSG00000232833	FAM27E3	grey	0.05597185
ENSG00000105058	FAM32A	blue	0.37573119
ENSG00000122376	FAM35A	grey	0.06138187
ENSG00000071889	FAM3A	grey	-0.0359323
ENSG00000174028	FAM3C2	grey	0.41725539
ENSG00000185112	FAM43A	tan	0.6292275
ENSG00000119979	FAM45A	blue	0.40659865
ENSG00000112773	FAM46A	brown	-0.4031505
ENSG00000189157	FAM47E	black	-0.6469687
ENSG00000197872	FAM49A	blue	0.65294047
ENSG00000153310	FAM49B	grey60	0.60363884
ENSG00000071859	FAM50A	grey	-0.1127724
ENSG00000145945	FAM50B	salmon	0.77120312
ENSG00000189319	FAM53B	yellow	0.67814507
ENSG00000233334	FAM53B-AS1	grey	0.24475798
ENSG00000120709	FAM53C	grey	0.26516365
ENSG00000167695	FAM57A	blue	-0.4616046
ENSG00000147382	FAM58A	turquoise	-0.4592237
ENSG00000139146	FAM60A	black	0.67146503
ENSG00000143409	FAM63A	yellow	0.76595641
ENSG00000128923	FAM63B	turquoise	0.82350941
ENSG00000111913	FAM65B	magenta	0.57065116
ENSG00000042062	FAM65C	grey	0.20661849
ENSG00000227888	FAM66A	grey	-0.2852885
ENSG00000215374	FAM66B	turquoise	-0.4477766
ENSG00000226711	FAM66C	grey	-0.1834457
ENSG00000255052	FAM66D	grey	0.12816732
ENSG00000225725	FAM66E	grey	0.12453959
ENSG00000165716	FAM69B	red	0.51881495
ENSG00000187773	FAM69C	turquoise	0.45036995
ENSG00000180219	FAM71C	grey	0.05150398
ENSG00000142530	FAM71E1	yellow	-0.5244422
ENSG00000205085	FAM71F2	grey	0.01271754
ENSG00000180488	FAM73A	grey	0.11271016
ENSG00000148343	FAM73B	brown	0.50142425
ENSG00000009780	FAM76A	grey	0.0496317
ENSG00000077458	FAM76B	grey	-0.0257709
ENSG00000126882	FAM78A	black	0.6905431
ENSG00000188859	FAM78B	grey	-0.2223887
ENSG00000101447	FAM83D	black	0.54232814
ENSG00000180921	FAM83H	grey	0.05624882
ENSG00000168672	FAM84B	purple	-0.7664023
ENSG00000255495	FAM85A	grey	-0.2974187
ENSG00000253893	FAM85B	grey	0.18953639
ENSG00000118894	FAM86A	brown	0.42363754
ENSG00000186523	FAM86B1	grey	-0.156321

ENSG00000173295	FAM86B3P	grey	0.14623609
ENSG00000158483	FAM86C1	grey	0.23476333
ENSG00000244026	FAM86DP	blue	0.39069243
ENSG00000251669	FAM86EP	grey	0.1558912
ENSG00000164845	FAM86FP	grey	0.06879987
ENSG00000253540	FAM86HP	grey	0.15371465
ENSG00000171084	FAM86JP	grey	-0.1202847
ENSG00000177757	FAM87B	grey	0.1629021
ENSG00000182118	FAM89A	brown	-0.4838849
ENSG00000176973	FAM89B	grey	0.09421186
ENSG00000137414	FAM8A1	grey	-0.2402261
ENSG00000171847	FAM90A1	grey	-0.0446785
ENSG00000233115	FAM90A11P	grey	-0.1002324
ENSG00000233295	FAM90A20P	grey	0.10521696
ENSG00000251402	FAM90A25P	grey	-0.1764499
ENSG00000205879	FAM90A2P	grey	-0.1812135
ENSG00000176853	FAM91A1	turquoise	0.30494502
ENSG00000188343	FAM92A1	red	0.45229053
ENSG00000223839	FAM95B1	grey	-0.1753081
ENSG00000166797	FAM96A	turquoise	0.46119301
ENSG00000224566	FAM96AP2	grey	0.22397611
ENSG00000166595	FAM96B	grey	0.04682592
ENSG00000119812	FAM98A	grey	0.11284224
ENSG00000171262	FAM98B	grey	-0.2727205
ENSG00000181544	FANCB	green	0.82356377
ENSG00000158169	FANCC	grey	0.1273423
ENSG00000144554	FANCD2	grey	0.06291831
ENSG00000183161	FANCF	red	0.64624303
ENSG00000221829	FANCG	grey	-0.1442196
ENSG00000140525	FANCI	grey	0.27521188
ENSG00000115392	FANCL	red	-0.778757
ENSG00000187790	FANCM	grey	-0.1284559
ENSG00000197601	FAR1	yellow	0.5389041
ENSG00000254791	FAR1-IT1	grey	0.16705106
ENSG00000064763	FAR2	grey60	0.76948754
ENSG00000178162	FAR2P2	yellow	-0.5688708
ENSG00000152767	FARP1	red	0.73691408
ENSG00000006607	FARP2	turquoise	-0.472154
ENSG00000145982	FARS2	grey	0.08265229
ENSG00000179115	FARSA	blue	0.63562457
ENSG00000116120	FARSB	blue	0.83786709
ENSG00000026103	FAS	grey	0.08128906
ENSG00000169710	FASN	blue	0.47928038
ENSG00000164896	FASTK	turquoise	-0.4365215
ENSG00000138399	FASTKD1	grey	0.0097863
ENSG00000118246	FASTKD2	brown	0.32114629
ENSG00000124279	FASTKD3	grey	-0.0013525
ENSG00000215251	FASTKD5	grey	-0.0903436

ENSG00000083857	FAT1	turquoise	0.55629179
ENSG00000086570	FAT2	grey	-0.0323363
ENSG00000165323	FAT3	grey	-0.1447841
ENSG00000196159	FAT4	yellow	0.4728657
ENSG00000149806	FAU	greenyellow	0.86906771
ENSG00000237806	FAUP2	grey	0.10515395
ENSG00000146267	FAXC	blue	0.66738218
ENSG00000170271	FAXDC2	yellow	0.6583206
ENSG00000188878	FBF1	grey	0.13080939
ENSG00000105202	FBL	grey	-0.0520042
ENSG00000162458	FBLIM1	grey	0.32091014
ENSG00000188573	FBLL1	blue	0.71652992
ENSG00000077942	FBLN1	turquoise	0.67861034
ENSG00000163520	FBLN2	grey	0.114261
ENSG00000140092	FBLN5	black	-0.5400764
ENSG00000166147	FBN1	grey	0.13147816
ENSG00000156860	FBRS	grey	-0.0360942
ENSG00000112787	FBRSL1	grey	-0.2107116
ENSG00000127452	FBXL12	grey	0.18934186
ENSG00000161040	FBXL13	grey	0.20738327
ENSG00000171823	FBXL14	turquoise	-0.3013453
ENSG00000107872	FBXL15	brown	0.76757507
ENSG00000127585	FBXL16	darkorange	0.53653431
ENSG00000145743	FBXL17	grey	-0.0790572
ENSG00000155034	FBXL18	grey	-0.2586302
ENSG00000099364	FBXL19	turquoise	-0.4593776
ENSG00000260852	FBXL19-AS1	green	0.53003995
ENSG00000153558	FBXL2	blue	0.83030342
ENSG00000108306	FBXL20	grey	0.16627414
ENSG00000164616	FBXL21	grey	-0.0349651
ENSG00000197361	FBXL22	grey	0.17319919
ENSG00000005812	FBXL3	turquoise	0.64205729
ENSG00000112234	FBXL4	grey	0.03051913
ENSG00000118564	FBXL5	turquoise	0.56901836
ENSG00000182325	FBXL6	grey	0.2891837
ENSG00000147912	FBXO10	blue	0.49690916
ENSG00000138081	FBXO11	grey	-0.0651272
ENSG00000214050	FBXO16	blue	0.67108443
ENSG00000269190	FBXO17	grey	-0.0555674
ENSG00000134452	FBXO18	brown	0.44468579
ENSG00000116661	FBXO2	yellow	-0.5452945
ENSG00000135108	FBXO21	grey	0.09693845
ENSG00000167196	FBXO22	grey	0.07766164
ENSG00000147364	FBXO25	grey	0.07201683
ENSG00000161243	FBXO27	grey	0.08127524
ENSG00000143756	FBXO28	grey	0.09815087
ENSG00000110429	FBXO3	grey	-0.1903633
ENSG00000118496	FBXO30	turquoise	0.87010341

ENSG00000103264	FBXO31	brown	0.40686616
ENSG00000156804	FBXO32	yellow	0.58749825
ENSG00000165355	FBXO33	grey	0.33773654
ENSG00000178974	FBXO34	purple	0.61094323
ENSG00000153832	FBXO36	grey	0.21158881
ENSG00000145868	FBXO38	grey	0.12613002
ENSG00000151876	FBXO4	grey	0.1025057
ENSG00000163013	FBXO41	darkorange	0.76406175
ENSG00000037637	FBXO42	grey	-0.0284548
ENSG00000132879	FBXO44	yellow	-0.7056386
ENSG00000174013	FBXO45	brown	-0.7575013
ENSG00000177051	FBXO46	grey	-0.0157611
ENSG00000204923	FBXO48	grey	0.23550718
ENSG00000112029	FBXO5	grey	0.15110201
ENSG00000116663	FBXO6	grey	0.14845416
ENSG00000100225	FBXO7	yellow	0.48267405
ENSG00000164117	FBXO8	turquoise	0.62140383
ENSG00000112146	FBXO9	pink	-0.6985414
ENSG00000072803	FBXW11	brown	-0.6332056
ENSG00000230870	FBXW11P1	grey	0.29516099
ENSG00000119402	FBXW2	grey	0.08808855
ENSG00000107829	FBXW4	green	0.69449416
ENSG00000230701	FBXW4P1	grey	0.23297915
ENSG00000159069	FBXW5	yellow	-0.4317307
ENSG00000109670	FBXW7	blue	0.65059731
ENSG00000174989	FBXW8	black	0.77263575
ENSG00000132004	FBXW9	yellow	-0.5156411
ENSG00000158869	FCER1G	royalblue	0.89176696
ENSG00000227436	FCF1P1	grey	0.2955812
ENSG00000228638	FCF1P2	grey	0.21732094
ENSG00000239997	FCF1P3	grey	0.34942701
ENSG00000220392	FCF1P5	grey	0.19292943
ENSG00000224727	FCF1P7	grey	0.2333934
ENSG00000150337	FCGR1A	royalblue	0.79600481
ENSG00000198019	FCGR1B	royalblue	0.64965235
ENSG00000143226	FCGR2A	royalblue	0.90438149
ENSG00000203747	FCGR3A	royalblue	0.89444705
ENSG00000104870	FCGRT	cyan	0.69583342
ENSG00000130475	FCHO1	yellow	0.65286478
ENSG00000157107	FCHO2	blue	-0.534615
ENSG00000197948	FCHSD1	grey	-0.0945524
ENSG00000137478	FCHSD2	green	0.80792073
ENSG00000079459	FDFT1	grey	-0.0661
ENSG00000160752	FDPS	yellow	-0.4798717
ENSG00000137714	FDX1	grey	0.2230159
ENSG00000267673	FDX1L	blue	0.54156697
ENSG00000161513	FDXR	grey	-0.1302244
ENSG00000066926	FECH	yellow	0.40168824

ENSG00000169018	FEM1B	grey	0.09377809
ENSG00000145780	FEM1C	turquoise	0.70260258
ENSG00000168496	FEN1	grey	-0.1538682
ENSG00000151422	FER	turquoise	0.54463899
ENSG00000253868	FER1L6-AS2	grey	0.14334567
ENSG00000073712	FERMT2	turquoise	0.6125618
ENSG00000149781	FERMT3	royalblue	0.66133715
ENSG00000182511	FES	grey	0.01411251
ENSG00000149557	FEZ1	yellow	0.55781269
ENSG00000171055	FEZ2	grey	-0.3000612
ENSG00000230316	FEZF1-AS1	turquoise	0.57651264
ENSG00000153266	FEZF2	turquoise	0.47822778
ENSG00000102302	FGD1	grey	0.19581595
ENSG00000146192	FGD2	cyan	0.73089922
ENSG00000139132	FGD4	turquoise	0.50271032
ENSG00000154783	FGD5	tan	0.75112208
ENSG00000225733	FGD5-AS1	turquoise	0.76258637
ENSG00000180263	FGD6	turquoise	0.76748454
ENSG00000113578	FGF1	green	0.88838804
ENSG00000114279	FGF12	blue	0.79736172
ENSG00000231383	FGF12-AS1	blue	0.43938548
ENSG00000230126	FGF12-AS2	grey	0.39228549
ENSG00000226709	FGF12-AS3	grey	0.41022359
ENSG00000129682	FGF13	blue	0.67814677
ENSG00000102466	FGF14	grey60	0.62559487
ENSG00000234445	FGF14-AS1	green	0.34640069
ENSG00000243319	FGF14-IT1	green	0.51695483
ENSG00000158815	FGF17	grey	-0.0803579
ENSG00000138685	FGF2	turquoise	0.82767876
ENSG00000140285	FGF7	grey	0.0020248
ENSG00000102678	FGF9	blue	0.49370544
ENSG00000174721	FGFBP3	brown	-0.6341722
ENSG00000077782	FGFR1	red	0.60948659
ENSG00000213066	FGFR1OP	black	-0.4287516
ENSG00000111790	FGFR1OP2	grey60	-0.5124302
ENSG00000068078	FGFR3	turquoise	0.69945081
ENSG00000127418	FGFRL1	pink	0.77135556
ENSG00000172456	FGGY	grey	0.09817493
ENSG00000127951	FGL2	cyan	0.6521347
ENSG00000000938	FGR	pink	0.67691013
ENSG00000091483	FH	grey	-0.0888844
ENSG00000137460	FHDC1	turquoise	-0.6174393
ENSG00000189283	FHIT	grey	-0.1363589
ENSG00000022267	FHL1	black	-0.7094021
ENSG00000115641	FHL2	grey	0.26095139
ENSG00000183386	FHL3	brown	-0.5161807
ENSG00000112214	FHL5	grey	0.07051428
ENSG00000135723	FHOD1	brown	0.58458968

ENSG00000134775	FHOD3	red	0.50658343
ENSG00000176971	FIBIN	turquoise	0.62801236
ENSG00000172500	FIBP	blue	0.68708766
ENSG00000198855	FICD	turquoise	0.64972807
ENSG00000112367	FIG4	brown	0.83077703
ENSG00000165197	FIGF	grey	0.21191187
ENSG00000182263	FIGN	turquoise	0.35537024
ENSG00000132436	FIGNL1	brown	-0.3391145
ENSG00000168386	FILIP1L	blue	-0.3533685
ENSG00000145216	FIP1L1	grey	0.09026367
ENSG00000214253	FIS1	brown	0.53036058
ENSG00000197296	FITM2	turquoise	0.62845832
ENSG00000179943	FIZ1	green	0.48087792
ENSG00000179431	FJX1	red	0.796446
ENSG00000141756	FKBP10	turquoise	0.66878743
ENSG00000134285	FKBP11	turquoise	-0.4842073
ENSG00000119321	FKBP15	grey	0.01843857
ENSG00000088832	FKBP1A	turquoise	-0.4445188
ENSG00000259691	FKBP1AP2	blue	-0.4836232
ENSG00000119782	FKBP1B	blue	0.55708693
ENSG00000173486	FKBP2	grey	-0.2493828
ENSG00000100442	FKBP3	grey	-0.049705
ENSG00000251463	FKBP4P1	blue	0.33241321
ENSG00000096060	FKBP5	blue	-0.572935
ENSG00000079150	FKBP7	grey	0.35326812
ENSG00000105701	FKBP8	blue	0.42316162
ENSG00000122642	FKBP9	turquoise	0.72717136
ENSG00000204315	FKBPL	grey	0.02366473
ENSG00000181027	FKRP	grey	0.11904077
ENSG00000224838	FKSG52	grey	-0.2240798
ENSG00000268156	FKSG61	grey	0.01890963
ENSG00000259752	FKSG62	grey	0.31968045
ENSG00000269866	FKSG63	grey	0.27269512
ENSG00000267812	FKSG68	grey	0.03678974
ENSG00000106692	FKTN	green	-0.5060064
ENSG00000160688	FLAD1	grey	0.15236633
ENSG00000154803	FLCN	grey	-0.0391816
ENSG00000237975	FLG-AS1	grey	0.28281558
ENSG00000151702	FLI1	tan	0.82521636
ENSG00000177731	FLII	lightcyan	-0.5197275
ENSG00000269510	FLJ00273	grey	0.39993735
ENSG00000197182	FLJ27365	grey	-0.1226651
ENSG00000196924	FLNA	pink	0.51701493
ENSG00000136068	FLNB	red	-0.6466015
ENSG00000128591	FLNC	yellow	0.56884818
ENSG00000137312	FLOT1	grey	0.18668095
ENSG00000132589	FLOT2	pink	0.47430112
ENSG00000126500	FLRT1	blue	0.41537856

ENSG00000185070	FLRT2	turquoise	0.71637478
ENSG00000125848	FLRT3	blue	0.64645488
ENSG00000102755	FLT1	tan	0.89691606
ENSG00000037280	FLT4	grey	0.24570197
ENSG00000162769	FLVCR1	blue	0.45320284
ENSG00000059122	FLYWCH1	blue	0.37854827
ENSG00000162076	FLYWCH2	grey	0.15556207
ENSG00000248905	FMN1	turquoise	-0.4296874
ENSG00000184922	FMNL1	blue	0.67111779
ENSG00000157827	FMNL2	darkred	-0.7408053
ENSG00000161791	FMNL3	cyan	0.65122772
ENSG00000094963	FMO2	grey	0.01652426
ENSG00000076258	FMO4	grey	0.13701739
ENSG00000131781	FMO5	grey	-0.0760054
ENSG00000122176	FMOD	grey	0.12484627
ENSG00000102081	FMR1	bisque4	0.78042292
ENSG00000236337	FMR1-IT1	grey	0.34210493
ENSG00000115414	FN1	tan	0.60908514
ENSG00000167363	FN3K	grey	-0.1511153
ENSG00000141560	FN3KRP	grey	0.02880142
ENSG00000187239	FNBP1	yellow	0.79890258
ENSG00000137942	FNBP1L	black	0.5284309
ENSG00000109920	FNBP4	red	-0.6682432
ENSG00000102531	FNDC3A	red	0.53246814
ENSG00000075420	FNDC3B	turquoise	0.51816473
ENSG00000115226	FNDC4	grey	0.09864023
ENSG00000160097	FNDC5	darkmagenta	-0.6807418
ENSG00000172568	FNDC9	brown	0.53634181
ENSG00000217128	FNIP1	turquoise	0.47094623
ENSG00000052795	FNIP2	yellow	0.59918148
ENSG00000257365	FNTB	grey	0.15825643
ENSG00000188352	FOCAD	turquoise	-0.6802596
ENSG00000086205	FOLH1	yellow	0.85831357
ENSG00000165457	FOLR2	cyan	0.70611162
ENSG00000133393	FOPNL	grey	0.24406697
ENSG00000248394	FOSL1P1	grey	0.33663712
ENSG00000075426	FOSL2	pink	0.58882826
ENSG00000054598	FOXC1	tan	0.69292773
ENSG00000137273	FOXF2	tan	0.62795631
ENSG00000176165	FOXG1	magenta	0.68558196
ENSG00000065970	FOXJ2	brown	-0.4434802
ENSG00000198815	FOXJ3	lightgreen	-0.5952216
ENSG00000164916	FOXK1	grey	0.12870737
ENSG00000170802	FOXN2	yellow	0.73762422
ENSG00000053254	FOXN3	brown	-0.5812006
ENSG00000258920	FOXN3-AS1	brown	-0.3574023
ENSG00000150907	FOXO1	grey	-0.0382299
ENSG00000118689	FOXO3	turquoise	0.69169725

ENSG00000240445	FOXO3B	grey	0.33028466
ENSG00000184481	FOXO4	green	0.9022558
ENSG00000204060	FOXO6	magenta	0.44122643
ENSG00000114861	FOXP1	red	-0.5485806
ENSG00000244203	FOXP1-AS1	grey	0.37487585
ENSG00000242094	FOXP1-IT1	grey	-0.0511808
ENSG00000128573	FOXP2	purple	0.6565065
ENSG00000137166	FOXP4	darkmagenta	0.59817711
ENSG00000164379	FOXQ1	tan	0.49687844
ENSG00000110074	FOXRED1	yellow	-0.4682711
ENSG00000100350	FOXRED2	red	0.57445073
ENSG00000136877	FPGS	grey	0.11977592
ENSG00000254685	FPGT	grey	0.07238291
ENSG00000171051	FPR1	royalblue	0.75334964
ENSG00000148690	FRA10AC1	grey	-0.2193102
ENSG00000138759	FRAS1	blue	0.67480267
ENSG00000165879	FRAT1	grey	-0.0343612
ENSG00000181274	FRAT2	blue	-0.6008027
ENSG00000109536	FRG1	grey	-0.2499808
ENSG00000149531	FRG1B	grey	-0.1978017
ENSG00000111816	FRK	red	-0.5000762
ENSG00000172159	FRMD3	grey	0.16037178
ENSG00000151474	FRMD4A	grey	0.11287815
ENSG00000114541	FRMD4B	yellow	0.86448736
ENSG00000171877	FRMD5	yellow	0.78570627
ENSG00000139926	FRMD6	turquoise	0.61845249
ENSG00000126391	FRMD8	grey	0.21108636
ENSG00000070601	FRMPD1	black	0.57854758
ENSG00000170324	FRMPD2	red	0.60309726
ENSG00000147234	FRMPD3	grey	0.01019301
ENSG00000169933	FRMPD4	blue	0.79705158
ENSG00000223487	FRMPD4-AS1	grey	0.23650464
ENSG00000260230	FRRS1L	brown	0.78833862
ENSG00000166225	FRS2	brown	-0.7461795
ENSG00000137218	FRS3	darkorange	0.60149999
ENSG00000073910	FRY	blue	0.86936118
ENSG00000237637	FRY-AS1	grey	0.11100614
ENSG00000075539	FRYL	yellow	0.8365497
ENSG00000162998	FRZB	grey	-0.056846
ENSG00000075618	FSCN1	floralwhite	0.76402158
ENSG00000105255	FSD1	turquoise	-0.6237436
ENSG00000106701	FSD1L	brown	-0.332227
ENSG00000186628	FSD2	grey	0.13195069
ENSG00000150667	FSIP1	blue	0.55835476
ENSG00000163430	FSTL1	turquoise	0.48261956
ENSG00000070404	FSTL3	pink	0.57839632
ENSG00000053108	FSTL4	darkorange	0.74328797
ENSG00000160282	FTCD	grey	-0.1172325

ENSG00000226124	FTCDNL1	grey	0.09160652
ENSG00000218980	FTH1P15	grey	0.47005722
ENSG00000227376	FTH1P16	grey	0.21482638
ENSG00000234975	FTH1P2	turquoise	-0.4149378
ENSG00000249302	FTH1P24	grey	0.48616897
ENSG00000213453	FTH1P3	grey	0.41164956
ENSG00000242992	FTH1P4	grey	0.45761685
ENSG00000087086	FTL	turquoise	0.4939023
ENSG00000254984	FTLP6	grey	0.3719205
ENSG00000140718	FTO	grey	-0.1166162
ENSG00000260936	FTO-IT1	grey	0.22784082
ENSG00000226491	FTOP1	grey	0.28820053
ENSG00000068438	FTSJ1	grey	-0.2399511
ENSG00000122687	FTSJ2	grey	0.16874813
ENSG00000108592	FTSJ3	grey	0.07070828
ENSG00000230590	FTX	grey	-0.3521558
ENSG00000107164	FUBP3	turquoise	0.67200459
ENSG00000179163	FUCA1	grey	-0.1622133
ENSG00000001036	FUCA2	grey	0.18958182
ENSG00000157353	FUK	grey	0.22898044
ENSG00000069509	FUNDC1	blue	0.49989952
ENSG00000140564	FURIN	pink	0.55115827
ENSG00000089280	FUS	salmon	0.60504302
ENSG00000172728	FUT10	turquoise	0.74789364
ENSG00000196968	FUT11	magenta	-0.3618528
ENSG00000196371	FUT4	turquoise	0.41378376
ENSG00000033170	FUT8	yellow	0.72903276
ENSG00000172461	FUT9	turquoise	0.65535771
ENSG00000010361	FUZ	grey	0.00481095
ENSG00000165060	FXN	grey	0.01759025
ENSG00000129245	FXR2	blue	0.45839093
ENSG00000266964	FXYD1	turquoise	0.47228625
ENSG00000089356	FXYD3	blue	-0.5818516
ENSG00000150201	FXYD4	grey	0.1807589
ENSG00000089327	FXYD5	grey	-0.2394346
ENSG00000137726	FXYD6	yellow	-0.6144612
ENSG00000221946	FXYD7	blue	0.54386455
ENSG00000082074	FYB	royalblue	0.8670712
ENSG00000163820	FYCO1	green	0.72504962
ENSG00000010810	FYN	blue	-0.4562339
ENSG00000122068	FYTTD1	brown	-0.6681267
ENSG00000234502	FYTTD1P1	grey	0.09718681
ENSG00000157240	FZD1	turquoise	0.6868773
ENSG00000104290	FZD3	purple	0.56768617
ENSG00000174804	FZD4	blue	-0.6988465
ENSG00000163251	FZD5	turquoise	0.37369175
ENSG00000164930	FZD6	blue	-0.6235662
ENSG00000155760	FZD7	turquoise	0.39300558

ENSG00000177283	FZD8	red	0.63343825
ENSG00000105325	FZR1	grey	0.11325833
ENSG00000123689	G0S2	grey	0.12429224
ENSG00000092140	G2E3	grey	-0.1764336
ENSG00000145907	G3BP1	turquoise	0.76521379
ENSG00000138757	G3BP2	brown	0.77022818
ENSG00000141349	G6PC3	red	0.54787496
ENSG00000160211	G6PD	grey	0.17345185
ENSG00000171298	GAA	cyan	0.56022216
ENSG00000109458	GAB1	yellow	0.80108621
ENSG00000033327	GAB2	green	0.93034805
ENSG00000160219	GAB3	cyan	0.57786569
ENSG00000170296	GABARAP	lightcyan	0.48892085
ENSG00000139112	GABARAPL1	blue	0.82646878
ENSG00000034713	GABARAPL2	mediumpurp	0.73509532
ENSG00000204681	GABBR1	red	0.56029519
ENSG00000136928	GABBR2	blue	0.6332324
ENSG00000104064	GABPB1	brown	0.67313816
ENSG00000244879	GABPB1-AS1	grey	0.07405533
ENSG00000143458	GABPB2	grey	0.00808741
ENSG00000151834	GABRA2	turquoise	0.6321261
ENSG00000011677	GABRA3	purple	0.79874602
ENSG00000109158	GABRA4	blue	0.28346989
ENSG00000186297	GABRA5	purple	0.80441644
ENSG00000163288	GABRB1	red	0.71788238
ENSG00000166206	GABRB3	blue	0.82447566
ENSG00000187730	GABRD	turquoise	-0.5472337
ENSG00000163285	GABRG1	turquoise	0.80694163
ENSG00000113327	GABRG2	blue	0.76849546
ENSG00000182256	GABRG3	magenta	0.50346038
ENSG00000147402	GABRQ	black	-0.6446174
ENSG00000116717	GADD45A	pink	0.83535235
ENSG00000099860	GADD45B	pink	0.76084554
ENSG00000130222	GADD45G	grey	0.23102093
ENSG00000179271	GADD45GIP1	green	-0.5647919
ENSG00000178950	GAK	brown	0.63376423
ENSG00000128242	GAL3ST1	yellow	0.54848476
ENSG00000197093	GAL3ST4	cyan	0.74485743
ENSG00000054983	GALC	blue	-0.4485215
ENSG00000117308	GALE	grey	0.05159199
ENSG00000108479	GALK1	grey	-0.0188711
ENSG00000156958	GALK2	grey	0.08892698
ENSG00000143891	GALM	grey	0.23115588
ENSG00000141012	GALNS	grey	0.09752362
ENSG00000164574	GALNT10	grey	-0.181627
ENSG00000178234	GALNT11	brown	0.63285611
ENSG00000144278	GALNT13	grey	-0.1302975
ENSG00000158089	GALNT14	blue	0.43815252

ENSG00000131386	GALNT15	yellow	0.51196913
ENSG00000100626	GALNT16	grey	0.21486876
ENSG00000110328	GALNT18	grey	0.34385856
ENSG00000143641	GALNT2	turquoise	0.3440537
ENSG00000139629	GALNT6	yellow	0.71219306
ENSG00000109586	GALNT7	grey	-0.0705972
ENSG00000174473	GALNTL6	purple	0.76791541
ENSG00000213930	GALT	blue	0.67843647
ENSG00000130005	GAMT	brown	0.54051994
ENSG00000089597	GANAB	grey	-0.002684
ENSG00000214013	GANC	yellow	0.67319076
ENSG00000172020	GAP43	purple	0.87373463
ENSG00000111640	GAPDH	grey	0.08195623
ENSG00000240244	GAPDHP33	grey	-0.063087
ENSG00000243033	GAPDHP47	grey	0.05807433
ENSG00000234285	GAPDHP49	grey	0.4587465
ENSG00000224055	GAPDHP55	grey	0.11351814
ENSG00000231739	GAPDHP59	grey	0.32158481
ENSG00000248180	GAPDHP60	grey	0.29916684
ENSG00000250933	GAPDHP66	grey	0.20821947
ENSG00000223460	GAPDHP69	grey	0.38073047
ENSG00000213376	GAPDHP71	yellow	0.62759745
ENSG00000165219	GAPVD1	grey	0.38085303
ENSG00000109534	GAR1	grey	-0.4014838
ENSG00000141441	GAREM	turquoise	0.69134042
ENSG00000157833	GAREML	turquoise	0.65604091
ENSG00000136895	GARNL3	green	-0.5334246
ENSG00000106105	GARS	brown	0.83947881
ENSG00000159131	GART	salmon	0.51960234
ENSG00000180447	GAS1	blue	-0.3499199
ENSG00000148935	GAS2	blue	0.3514291
ENSG00000185340	GAS2L1	grey	0.23533916
ENSG00000234741	GAS5	greenyellow	0.62118376
ENSG00000270084	GAS5-AS1	grey	0.44909937
ENSG00000183087	GAS6	brown	0.53395067
ENSG00000007237	GAS7	grey	-0.2651645
ENSG00000141013	GAS8	blue	0.50281634
ENSG00000167491	GATAD2A	grey	0.10903251
ENSG00000143614	GATAD2B	yellow	0.40453495
ENSG00000257218	GATC	brown	-0.4247022
ENSG00000171766	GATM	turquoise	0.67733695
ENSG00000239521	GATS	yellow	0.67371076
ENSG00000198750	GATSL2	grey	0.31525901
ENSG00000239282	GATSL3	turquoise	0.36896024
ENSG00000177628	GBA	pink	-0.4635238
ENSG00000070610	GBA2	grey	0.36608214
ENSG00000160766	GBAP1	turquoise	-0.370342
ENSG00000146729	GBAS	turquoise	0.59287287

ENSG00000114480	GBE1	grey	0.33391047
ENSG00000107862	GBF1	brown	0.61587495
ENSG00000117228	GBP1	pink	0.71379017
ENSG00000225492	GBP1P1	grey	0.22041491
ENSG00000162645	GBP2	pink	0.72891165
ENSG00000117226	GBP3	grey	0.10818883
ENSG00000162654	GBP4	tan	0.57330175
ENSG00000168505	GBX2	grey	0.26983503
ENSG00000115271	GCA	yellow	0.70029771
ENSG00000100116	GCAT	grey	0.15231326
ENSG00000179562	GCC1	turquoise	0.54568517
ENSG00000135968	GCC2	grey	-0.2070318
ENSG00000105607	GCDH	turquoise	0.3263147
ENSG00000005436	GCFC2	turquoise	0.45472199
ENSG00000131979	GCH1	grey	-0.0991062
ENSG00000137880	GCHFR	grey	-0.0136793
ENSG00000001084	GCLC	grey	0.19378997
ENSG00000023909	GCLM	turquoise	0.4893155
ENSG00000124827	GCM2	grey	0.24954793
ENSG00000089154	GCN1L1	grey	0.17977717
ENSG00000187210	GCNT1	grey	-0.1478664
ENSG00000231047	GCNT1P3	grey	0.35873537
ENSG00000111846	GCNT2	grey	-0.0308328
ENSG00000176928	GCNT4	black	0.79636376
ENSG00000124091	GCNT7	grey	0.25395628
ENSG00000140905	GCSH	turquoise	0.73095085
ENSG00000104381	GDAP1	pink	-0.540752
ENSG00000124194	GDAP1L1	purple	0.81370072
ENSG00000196505	GDAP2	turquoise	-0.4490621
ENSG00000006007	GDE1	turquoise	-0.417897
ENSG00000135414	GDF11	brown	-0.8239472
ENSG00000203879	GDI1	blue	0.69557094
ENSG00000057608	GDI2	grey	-0.0330305
ENSG00000233994	GDI2P2	grey	-0.0460023
ENSG00000248587	GDNF-AS1	grey	0.14282772
ENSG00000153982	GDPD1	darkred	0.46479334
ENSG00000130055	GDPD2	black	-0.5854923
ENSG00000102886	GDPD3	grey	-0.0041784
ENSG00000158555	GDPD5	grey	0.24665488
ENSG00000183208	GDPGP1	grey	0.2462183
ENSG00000164949	GEM	turquoise	0.55586917
ENSG00000092208	GEMIN2	grey	0.00994925
ENSG00000179409	GEMIN4	grey	0.04868047
ENSG00000082516	GEMIN5	grey	0.14429489
ENSG00000152147	GEMIN6	brown	0.37578913
ENSG00000142252	GEMIN7	grey	0.23871783
ENSG00000046647	GEMIN8	grey	0.06685371
ENSG00000178295	GEN1	grey	0.22227292

ENSG00000131095	GFAP	grey	-0.2656281
ENSG00000127554	GFER	grey	-0.033269
ENSG00000168827	GFM1	darkred	0.52392669
ENSG00000145990	GFOD1	grey	-0.0048413
ENSG00000141098	GFOD2	yellow	-0.6475679
ENSG00000198380	GFPT1	red	0.70180363
ENSG00000131459	GFPT2	grey	-0.1370032
ENSG00000151892	GFRA1	turquoise	0.54331133
ENSG00000100083	GGA1	grey	0.01137382
ENSG00000103365	GGA2	yellow	-0.600551
ENSG00000125447	GGA3	turquoise	-0.5938227
ENSG00000006625	GGCT	grey	-0.0460644
ENSG00000115486	GGCX	salmon	0.55207121
ENSG00000137563	GGH	brown	0.63665478
ENSG00000152904	GGPS1	brown	-0.4660459
ENSG00000100031	GGT1	grey	-0.0028147
ENSG00000099998	GGT5	tan	0.66979119
ENSG00000131067	GGT7	grey	-0.1891788
ENSG00000204136	GGTA1P	turquoise	0.51769152
ENSG00000237766	GGTA2P	grey	0.32096977
ENSG00000167925	GHDC	grey	-0.0309113
ENSG00000165678	GHITM	red	0.61987708
ENSG00000112964	GHR	red	0.58169023
ENSG00000157017	GHRL	grey	0.05631324
ENSG00000240288	GHRLOS	grey	0.15647814
ENSG00000101193	GID8	darkred	-0.5372233
ENSG00000146830	GIGYF1	red	-0.5325545
ENSG00000204120	GIGYF2	darkgrey	0.75387389
ENSG00000213203	GIMAP1	tan	0.64459602
ENSG00000106560	GIMAP2	cyan	0.51895192
ENSG00000133574	GIMAP4	tan	0.74885503
ENSG00000196329	GIMAP5	tan	0.78473756
ENSG00000133561	GIMAP6	tan	0.79706526
ENSG00000179144	GIMAP7	tan	0.77415892
ENSG00000171115	GIMAP8	tan	0.68414817
ENSG00000145723	GIN1	turquoise	0.54532106
ENSG00000055211	GINM1	blue	-0.6724911
ENSG00000131153	GINS2	grey	-0.0983919
ENSG00000181938	GINS3	grey	-0.1355322
ENSG00000123159	GIPC1	grey	-0.0535829
ENSG00000108262	GIT1	grey60	0.50195072
ENSG00000139436	GIT2	red	-0.4775416
ENSG00000152661	GJA1	turquoise	0.69174448
ENSG00000176857	GJA1P1	grey	0.28316127
ENSG00000187513	GJA4	pink	0.6598127
ENSG00000143140	GJA5	tan	0.47590063
ENSG00000236187	GJA6P	grey	0.37430264
ENSG00000169562	GJB1	yellow	0.80565607

ENSG00000121742	GJB6	turquoise	0.80766232
ENSG00000182963	GJC1	brown	-0.6014571
ENSG00000198835	GJC2	yellow	0.66621639
ENSG00000176402	GJC3	grey	0.3336881
ENSG00000198814	GK	grey	0.23023251
ENSG00000243055	GK-AS1	grey	0.36099822
ENSG00000175066	GK5	grey	-0.1458976
ENSG00000165113	GKAP1	grey	-0.0129391
ENSG00000102393	GLA	grey	0.04330075
ENSG00000170266	GLB1	yellow	-0.324871
ENSG00000163521	GLB1L	grey	0.12825778
ENSG00000149328	GLB1L2	grey	-0.1274157
ENSG00000166105	GLB1L3	brown	0.6899959
ENSG00000106415	GLCCI1	purple	0.56150349
ENSG00000138604	GLCE	grey	-0.172954
ENSG00000178445	GLDC	turquoise	0.48784088
ENSG00000186417	GLDN	yellow	0.76787281
ENSG00000090863	GLG1	brown	0.73603624
ENSG00000106571	GLI3	turquoise	0.70324565
ENSG00000250571	GLI4	grey	-0.0745705
ENSG00000139278	GLIPR1	cyan	0.54212064
ENSG00000180481	GLIPR1L2	grey	0.06305159
ENSG00000122694	GLIPR2	yellow	0.74601613
ENSG00000174332	GLIS1	purple	0.66551003
ENSG00000126603	GLIS2	pink	0.47925547
ENSG00000107249	GLIS3	turquoise	0.545918
ENSG00000174842	GLMN	blue	0.4478448
ENSG00000124767	GLO1	grey	-0.0864133
ENSG00000112164	GLP1R	magenta	0.42406078
ENSG00000101958	GLRA2	black	-0.5334018
ENSG00000145451	GLRA3	purple	0.8017032
ENSG00000109738	GLRB	darkred	0.48539321
ENSG00000173221	GLRX	yellowgreen	-0.6076923
ENSG00000023572	GLRX2	green	-0.6024412
ENSG00000108010	GLRX3	brown	0.5962308
ENSG00000182512	GLRX5	greenyellow	0.45473395
ENSG00000115419	GLS	brown	0.84579097
ENSG00000135423	GLS2	blue	0.67565261
ENSG00000151948	GLT1D1	grey60	0.61433084
ENSG00000016864	GLT8D1	turquoise	-0.5361785
ENSG00000120820	GLT8D2	blue	0.6165241
ENSG00000139433	GLTP	yellow	0.79060171
ENSG00000224051	GLTPD1	grey	0.17748074
ENSG00000182327	GLTPD2	grey	0.19227562
ENSG00000063169	GLTSCR1	grey	0.04112604
ENSG00000112624	GLTSCR1L	grey	0.03570374
ENSG00000105373	GLTSCR2	grey	-0.3547447
ENSG00000148672	GLUD1	turquoise	0.6224941

ENSG00000227781	GLUD1P2	grey	-0.0400192
ENSG00000250959	GLUD1P3	grey	0.16275448
ENSG00000182890	GLUD2	grey	-0.0384414
ENSG00000140632	GLYR1	grey	0.29680036
ENSG00000196743	GM2A	brown	-0.8166614
ENSG00000087338	GMCL1	turquoise	0.66329137
ENSG00000112699	GMDS	brown	0.71651188
ENSG00000250903	GMDS-AS1	grey	0.18247779
ENSG00000162419	GMEB1	grey	0.08995085
ENSG00000101216	GMEB2	grey	0.00779145
ENSG00000197045	GMFB	brown	-0.8159116
ENSG00000130755	GMFG	cyan	0.57990682
ENSG00000112312	GMNN	turquoise	0.53180657
ENSG00000144591	GMPPA	grey	-0.0320911
ENSG00000100938	GMPR2	green	-0.5743503
ENSG00000088256	GNA11	grey	0.24680822
ENSG00000146535	GNA12	turquoise	0.72887153
ENSG00000120063	GNA13	turquoise	0.78081975
ENSG00000156049	GNA14	turquoise	0.51005845
ENSG00000127955	GNAI1	yellow	0.5475893
ENSG00000114353	GNAI2	grey60	-0.6135134
ENSG00000065135	GNAI3	turquoise	0.51933648
ENSG00000141404	GNAL	blue	0.64181693
ENSG00000087258	GNAO1	turquoise	-0.446401
ENSG00000156052	GNAQ	turquoise	0.76467324
ENSG00000087460	GNAS	red	0.62363966
ENSG00000134183	GNAT2	grey	0.37631648
ENSG00000128266	GNAZ	salmon	-0.6273196
ENSG00000078369	GNB1	grey60	0.71916853
ENSG00000172354	GNB2	turquoise	-0.4541145
ENSG00000204628	GNB2L1	greenyellow	0.77638158
ENSG00000111664	GNB3	turquoise	-0.4422249
ENSG00000114450	GNB4	grey	-0.2035905
ENSG00000069966	GNB5	grey60	0.85683092
ENSG00000159921	GNE	grey	0.22083124
ENSG00000242616	GNG10	grey	-0.2582976
ENSG00000127920	GNG11	tan	0.73020019
ENSG00000172380	GNG12	turquoise	0.74371963
ENSG00000232284	GNG12-AS1	darkmagenta	-0.6331678
ENSG00000186469	GNG2	grey	-0.2399197
ENSG00000162188	GNG3	blue	0.78679775
ENSG00000168243	GNG4	black	-0.4928914
ENSG00000174021	GNG5	blue	-0.3440764
ENSG00000176533	GNG7	magenta	0.73318381
ENSG00000167414	GNG8	yellow	0.33502783
ENSG00000204590	GNL1	grey	-0.0828386
ENSG00000134697	GNL2	grey	0.2237056
ENSG00000163938	GNL3	brown	0.67822102

ENSG00000130119	GNL3L	grey	0.31019352
ENSG00000124713	GNMT	grey	0.08872929
ENSG00000116906	GNPAT	turquoise	0.46421133
ENSG00000113552	GNPDA1	brown	0.72651783
ENSG00000163281	GNPDA2	grey	-0.0632652
ENSG00000111670	GNPTAB	turquoise	-0.2895221
ENSG00000090581	GNPTG	grey	-0.0603951
ENSG00000147437	GNRH1	grey	0.18000629
ENSG00000109163	GNRHR	grey	0.27668977
ENSG00000135677	GNS	grey	0.29208387
ENSG00000136935	GOLGA1	grey	0.05830772
ENSG00000167110	GOLGA2	brown	0.60854671
ENSG00000225151	GOLGA2P7	grey	0.28042327
ENSG00000090615	GOLGA3	grey	-0.0139834
ENSG00000144674	GOLGA4	lightgreen	0.86161898
ENSG00000066455	GOLGA5	grey	-0.1332147
ENSG00000261649	GOLGA6L7P	grey	-0.0546485
ENSG00000147533	GOLGA7	yellow	0.70191791
ENSG00000175265	GOLGA8A	turquoise	-0.409101
ENSG00000179938	GOLGA8J	red	-0.4960795
ENSG00000188626	GOLGA8M	grey	0.07790783
ENSG00000186399	GOLGA8R	grey	-0.0717132
ENSG00000261739	GOLGA8S	grey	-0.1133567
ENSG00000261247	GOLGA8T	grey	0.08361266
ENSG00000173230	GOLGB1	lightgreen	0.79286766
ENSG00000173905	GOLIM4	turquoise	0.71886562
ENSG00000113384	GOLPH3	brown	-0.8130445
ENSG00000143457	GOLPH3L	red	0.53480206
ENSG00000174567	GOLT1A	purple	0.76787138
ENSG00000111711	GOLT1B	turquoise	0.37722655
ENSG00000116580	GON4L	turquoise	-0.5323871
ENSG00000120370	GORAB	grey	-0.1431223
ENSG00000114745	GORASP1	turquoise	0.34391449
ENSG00000115806	GORASP2	brown	0.45657733
ENSG00000108587	GOSR1	grey	0.00708585
ENSG00000108433	GOSR2	brown	0.49637807
ENSG00000120053	GOT1	brown	0.7636601
ENSG00000125166	GOT2	brown	0.82242843
ENSG00000185245	GP1BA	grey	0.15319248
ENSG00000088053	GP6	grey	0.18153517
ENSG00000197858	GPAA1	brown	0.70838024
ENSG00000133114	GPALPP1	grey	-0.3027558
ENSG00000119927	GPAM	turquoise	0.7961755
ENSG00000204438	GPANK1	grey	0.11076914
ENSG00000186281	GPAT2	grey	-0.1090913
ENSG00000076650	GPATCH1	blue	0.43247872
ENSG00000152133	GPATCH11	grey	-0.2066346
ENSG00000092978	GPATCH2	grey	-0.0549338

ENSG00000089916	GPATCH2L	grey	0.01145851
ENSG00000198746	GPATCH3	grey	0.28673536
ENSG00000160818	GPATCH4	darkgrey	0.57194378
ENSG00000186566	GPATCH8	grey	0.10994212
ENSG00000062194	GPBP1	lightgreen	0.51597907
ENSG00000063660	GPC1	yellow	-0.6912751
ENSG00000076716	GPC4	turquoise	0.39651018
ENSG00000179399	GPC5	turquoise	0.52188772
ENSG00000235984	GPC5-AS1	grey	0.34510073
ENSG00000232885	GPC5-AS2	turquoise	0.34027689
ENSG00000236240	GPC5-IT1	grey	0.22443173
ENSG00000183098	GPC6	red	0.54303997
ENSG00000236520	GPC6-AS1	grey	0.0786481
ENSG00000125772	GPCPD1	tan	0.62189929
ENSG00000167588	GPD1	grey	-0.2834586
ENSG00000152642	GPD1L	grey	-0.2681738
ENSG00000115159	GPD2	mediumpurp	0.69116433
ENSG00000164850	GPER1	tan	0.49480743
ENSG00000171723	GPHN	turquoise	-0.5251649
ENSG00000105220	GPI	blue	0.74931392
ENSG00000182851	GPIHBP1	yellow	0.51361728
ENSG00000068394	GPKOW	grey	0.00010588
ENSG00000112293	GPLD1	blue	0.43562806
ENSG00000150625	GPM6A	turquoise	0.64655203
ENSG00000046653	GPM6B	turquoise	0.7634918
ENSG00000198522	GPN1	green	-0.6294188
ENSG00000142751	GPN2	grey	0.30104045
ENSG00000136235	GPNMB	grey	-0.2280284
ENSG00000165370	GPR101	purple	0.64719577
ENSG00000148358	GPR107	green	-0.5541044
ENSG00000125734	GPR108	turquoise	0.52244358
ENSG00000069122	GPR116	tan	0.90955179
ENSG00000197177	GPR123	yellow	-0.5986384
ENSG00000020181	GPR124	tan	0.73958476
ENSG00000152990	GPR125	turquoise	0.60699227
ENSG00000181619	GPR135	blue	0.4024956
ENSG00000173264	GPR137	grey	0.20313216
ENSG00000077585	GPR137B	turquoise	0.83937716
ENSG00000180998	GPR137C	grey	0.02494696
ENSG00000180269	GPR139	grey	-0.1915583
ENSG00000101850	GPR143	turquoise	0.52117301
ENSG00000174948	GPR149	blue	0.47841528
ENSG00000158292	GPR153	yellow	-0.5967629
ENSG00000163328	GPR155	grey60	0.5784553
ENSG00000151025	GPR158	grey60	0.71264433
ENSG00000184194	GPR173	grey	0.07345651
ENSG00000166073	GPR176	purple	0.84884793
ENSG00000188888	GPR179	grey	0.09937243

ENSG00000125245	GPR18	blue	-0.3934407
ENSG00000152749	GPR180	grey	0.19103986
ENSG00000169508	GPR183	cyan	0.49689939
ENSG00000183150	GPR19	blue	0.4585522
ENSG00000188394	GPR21	grey	0.23257064
ENSG00000172209	GPR22	grey	0.00080136
ENSG00000170837	GPR27	grey	-0.2637167
ENSG00000181773	GPR3	grey	0.42225703
ENSG00000171659	GPR34	cyan	0.84644458
ENSG00000170775	GPR37	yellow	0.88579524
ENSG00000170075	GPR37L1	turquoise	0.80833045
ENSG00000177464	GPR4	pink	0.76060403
ENSG00000135973	GPR45	grey	-0.083929
ENSG00000203737	GPR52	magenta	0.64511923
ENSG00000135898	GPR55	magenta	0.5606467
ENSG00000205336	GPR56	turquoise	0.62835971
ENSG00000156097	GPR61	turquoise	-0.6883926
ENSG00000180929	GPR62	yellow	0.67361728
ENSG00000112218	GPR63	black	0.60483796
ENSG00000119714	GPR68	grey	0.11129889
ENSG00000119737	GPR75	turquoise	0.80864692
ENSG00000115239	GPR75-ASB3	grey	-0.0303622
ENSG00000123901	GPR83	grey	0.20818216
ENSG00000164604	GPR85	tan	0.50793532
ENSG00000138271	GPR87	grey	0.36699671
ENSG00000181656	GPR88	magenta	0.76581773
ENSG00000117262	GPR89A	brown	0.37900699
ENSG00000164199	GPR98	turquoise	0.52948496
ENSG00000198932	GPRASP1	blue	0.5382719
ENSG00000158301	GPRASP2	blue	0.66747597
ENSG00000170412	GPRC5C	turquoise	0.68291914
ENSG00000169258	GPRIN1	grey	-0.1239302
ENSG00000185477	GPRIN3	black	0.77967452
ENSG00000169727	GPS1	grey	-0.0931717
ENSG00000132522	GPS2	yellow	0.56312099
ENSG00000160360	GPSM1	yellow	-0.4369921
ENSG00000121957	GPSM2	green	0.76064668
ENSG00000213654	GPSM3	royalblue	0.68715412
ENSG00000166123	GPT2	turquoise	0.75348998
ENSG00000233276	GPX1	royalblue	0.53599101
ENSG00000176153	GPX2	grey	0.14978388
ENSG00000211445	GPX3	grey	-0.1888057
ENSG00000167468	GPX4	yellow	-0.5523805
ENSG00000116157	GPX7	turquoise	0.5063728
ENSG00000089351	GRAMD1A	grey	0.06987741
ENSG00000178075	GRAMD1C	turquoise	0.63907967
ENSG00000175318	GRAMD2	grey	0.0435506
ENSG00000155324	GRAMD3	turquoise	0.75789086

ENSG00000075240	GRAMD4	turquoise	0.38615283
ENSG00000257608	GRAMD4P3	green	0.39158684
ENSG00000161835	GRASP	grey	-0.010997
ENSG00000106070	GRB10	greenyellow	0.59665891
ENSG00000115290	GRB14	black	0.63160628
ENSG00000177885	GRB2	grey	-0.1071281
ENSG00000196208	GREB1	grey	0.06959479
ENSG00000141449	GREB1L	grey	-0.0765283
ENSG00000166923	GREM1	yellow	0.75812197
ENSG00000180875	GREM2	grey60	0.48223566
ENSG00000137106	GRHPR	salmon	0.59945118
ENSG00000155511	GRIA1	magenta	0.57002699
ENSG00000120251	GRIA2	magenta	0.43493912
ENSG00000125675	GRIA3	magenta	0.69428999
ENSG00000152578	GRIA4	yellow	-0.4333156
ENSG00000182771	GRID1	grey	0.24890668
ENSG00000174680	GRIK1-AS1	grey	0.29147949
ENSG00000164418	GRIK2	grey60	0.44060995
ENSG00000163873	GRIK3	turquoise	-0.3446597
ENSG00000149403	GRIK4	magenta	0.60665232
ENSG00000105737	GRIK5	grey	0.01641035
ENSG00000176884	GRIN1	blue	0.81471025
ENSG00000183454	GRIN2A	red	-0.5916291
ENSG00000161509	GRIN2C	turquoise	0.57502751
ENSG00000178719	GRINA	lightcyan	0.66691312
ENSG00000155974	GRIP1	blue	0.70243092
ENSG00000068400	GRIPAP1	turquoise	-0.7647814
ENSG00000125388	GRK4	blue	0.53259758
ENSG00000198873	GRK5	grey	-0.1357061
ENSG00000228485	GRK5-IT1	grey	0.3156993
ENSG00000198055	GRK6	turquoise	-0.3992117
ENSG00000198822	GRM3	grey	0.04748961
ENSG00000124493	GRM4	blue	0.40686722
ENSG00000168959	GRM5	blue	0.82283618
ENSG00000255082	GRM5-AS1	brown	0.5638886
ENSG00000196277	GRM7	purple	0.84340916
ENSG00000236202	GRM7-AS1	grey	0.2716424
ENSG00000237665	GRM7-AS2	grey	0.16996327
ENSG00000179603	GRM8	grey	-0.013681
ENSG00000030582	GRN	cyan	0.60498365
ENSG00000164284	GRPEL2	brown	-0.6713076
ENSG00000132463	GRSF1	grey	0.12672032
ENSG00000105447	GRWD1	green	-0.3719917
ENSG00000230470	GS1-115G20.1	grey	0.22597304
ENSG00000232036	GS1-115G20.2	grey	0.0463321
ENSG00000234500	GS1-124K5.10	grey	0.23271687
ENSG00000229180	GS1-124K5.11	grey	0.04857531
ENSG00000232559	GS1-124K5.12	grey	0.23417634

ENSG00000230189	GS1-124K5.2	grey	0.37335146
ENSG00000223473	GS1-124K5.3	grey	0.21994212
ENSG00000237310	GS1-124K5.4	grey	-0.0030102
ENSG00000251451	GS1-124K5.6	grey	0.28220929
ENSG00000224691	GS1-174L6.4	magenta	-0.4440689
ENSG00000228735	GS1-18A18.2	grey	0.07822924
ENSG00000253738	GS1-251I9.4	grey	-0.3046961
ENSG00000228238	GS1-304P7.2	grey	0.32762754
ENSG00000260822	GS1-358P8.4	turquoise	-0.6854926
ENSG00000254690	GS1-393G12.12	grey	0.18748626
ENSG00000228139	GS1-421I3.4	grey	0.35947727
ENSG00000237019	GS1-433O24.1	grey	0.38565639
ENSG00000253576	GS1-5L10.1	grey	0.21617488
ENSG00000186088	GSAP	grey	0.00582121
ENSG00000073605	GSDMB	grey	-0.2209808
ENSG00000104518	GSDMD	grey	0.14774759
ENSG00000131149	GSE1	grey	-0.1716083
ENSG00000111305	GSG1	grey	0.14651399
ENSG00000169181	GSG1L	darkred	0.4828738
ENSG00000105723	GSK3A	turquoise	-0.5961508
ENSG00000082701	GSK3B	grey	-0.0426059
ENSG00000100744	GSKIP	blue	0.61588414
ENSG00000235865	GSN-AS1	yellow	0.69922283
ENSG00000103342	GSPT1	darkorange	-0.7166716
ENSG00000189369	GSPT2	blue	0.48076232
ENSG00000104687	GSR	brown	0.6743183
ENSG00000100983	GSS	grey	-0.0339687
ENSG00000170899	GSTA4	grey	-0.2865056
ENSG00000138780	GSTCD	grey	0.05552889
ENSG00000197448	GSTK1	grey	0.26876084
ENSG00000213366	GSTM2	grey	-0.3437442
ENSG00000134202	GSTM3	grey	-0.3235229
ENSG00000227693	GSTM3P1	yellow	0.52944325
ENSG00000168765	GSTM4	grey	-0.3358489
ENSG00000134201	GSTM5	grey	0.06717232
ENSG00000148834	GSTO1	brown	0.69784711
ENSG00000065621	GSTO2	darkorange	0.48973285
ENSG00000084207	GSTP1	blue	-0.410116
ENSG00000184674	GSTT1	grey	0.17850137
ENSG00000100577	GSTZ1	grey	0.01303416
ENSG00000121964	GTDC1	darkgrey	0.65698119
ENSG00000165417	GTF2A1	salmon	-0.5527102
ENSG00000140307	GTF2A2	grey	-0.1102312
ENSG00000137947	GTF2B	darkred	0.56416322
ENSG00000153767	GTF2E1	turquoise	0.46467117
ENSG00000125651	GTF2F1	turquoise	-0.4105459
ENSG00000188342	GTF2F2	grey	-0.3927974
ENSG00000110768	GTF2H1	brown	0.67381947

ENSG00000145736	GTF2H2	grey	0.08296055
ENSG00000183474	GTF2H2C	grey	0.26715144
ENSG00000111358	GTF2H3	red	0.63196737
ENSG00000077809	GTF2I	yellow	0.40811186
ENSG00000232561	GTF2IP1	grey	0.44349534
ENSG00000006704	GTF2IRD1	yellow	-0.4698896
ENSG00000174428	GTF2IRD2B	grey	0.17608561
ENSG00000122034	GTF3A	turquoise	-0.6072324
ENSG00000077235	GTF3C1	lightcyan	-0.6481776
ENSG00000115207	GTF3C2	turquoise	-0.4273015
ENSG00000119041	GTF3C3	grey	0.27612023
ENSG00000125484	GTF3C4	grey	-0.2241042
ENSG00000155115	GTF3C6	mediumpurp	0.73666868
ENSG00000100226	GTPBP1	grey	0.15539456
ENSG00000105793	GTPBP10	grey	0.24465323
ENSG00000130299	GTPBP3	grey	0.03833021
ENSG00000107937	GTPBP4	brown	0.36150559
ENSG00000178605	GTPBP6	grey	-0.1394693
ENSG00000163607	GTPBP8	grey	0.18652653
ENSG00000048545	GUCA1A	blue	0.49351632
ENSG00000112599	GUCA1B	grey	0.08697546
ENSG00000138867	GUCD1	turquoise	0.56886414
ENSG00000152402	GUCY1A2	magenta	0.62333389
ENSG00000164116	GUCY1A3	turquoise	-0.5426916
ENSG00000061918	GUCY1B3	blue	0.81829477
ENSG00000151806	GUF1	grey	0.01211075
ENSG00000143774	GUK1	greenyellow	0.67133629
ENSG00000144366	GULP1	purple	0.63934207
ENSG00000169919	GUSB	grey	-0.1887277
ENSG00000183666	GUSBP1	grey	0.09979282
ENSG00000228315	GUSBP11	grey	-0.0244272
ENSG00000241549	GUSBP2	yellow	0.45439132
ENSG00000253203	GUSBP3	yellow	0.55671206
ENSG00000239650	GUSBP4	green	0.41031654
ENSG00000215630	GUSBP9	yellow	0.52898978
ENSG00000254838	GVINP1	grey	-0.014333
ENSG00000241043	GVQW1	grey	0.17181957
ENSG00000151233	GXYLT1	turquoise	0.78528895
ENSG00000172986	GXYLT2	brown	-0.483092
ENSG00000163754	GYG1	grey	0.15842068
ENSG00000056998	GYG2	turquoise	0.33258077
ENSG00000206159	GYG2P1	grey	-0.0798043
ENSG00000136732	GYPC	cyan	0.44023141
ENSG00000104812	GYS1	turquoise	0.35201243
ENSG00000125812	GZF1	brown	-0.6947469
ENSG00000189060	H1F0	brown	-0.531792
ENSG00000184897	H1FX	grey	0.0435102
ENSG00000206417	H1FX-AS1	grey	0.07646621

ENSG00000246705	H2AFJ	turquoise	-0.5264837
ENSG00000105968	H2AFV	brown	-0.8852411
ENSG00000188486	H2AFX	blue	-0.4466401
ENSG00000113648	H2AFY	greenyellow	0.59043787
ENSG00000099284	H2AFY2	grey	0.13961766
ENSG00000164032	H2AFZ	lightcyan	-0.7522276
ENSG00000213440	H2AFZP1	grey	0.35906565
ENSG00000255329	H2AFZP4	grey	0.16108849
ENSG00000163041	H3F3A	blue	-0.4711429
ENSG00000259389	H3F3AP1	grey	0.39153723
ENSG00000235655	H3F3AP4	turquoise	0.43534477
ENSG00000132475	H3F3B	blue	-0.6647943
ENSG00000236534	H3F3BP1	grey	0.42786114
ENSG00000049239	H6PD	grey	0.28029759
ENSG00000162882	HAAO	grey	-0.0309325
ENSG00000130956	HABP4	turquoise	-0.4987524
ENSG00000085382	HACE1	red	-0.6159549
ENSG00000131373	HACL1	turquoise	-0.6840029
ENSG00000138796	HADH	grey	0.00961146
ENSG00000084754	HADHA	turquoise	0.79496635
ENSG00000251596	HADHAP1	turquoise	0.32835089
ENSG00000138029	HADHB	turquoise	0.81628398
ENSG00000063854	HAGH	brown	0.5919709
ENSG00000103253	HAGHL	grey	-0.1101722
ENSG00000173805	HAP1	red	0.36690467
ENSG00000132702	HAPLN2	yellow	0.75340231
ENSG00000225978	HAR1A	blue	0.59247124
ENSG00000231133	HAR1B	blue	0.38758451
ENSG00000180423	HARBI1	grey	0.42898383
ENSG00000170445	HARS	yellow	-0.6457655
ENSG00000112855	HARS2	salmon	0.68399134
ENSG00000103044	HAS3	yellow	0.31329854
ENSG00000128708	HAT1	turquoise	0.43891071
ENSG00000152240	HAUS1	grey	-0.0574254
ENSG00000214367	HAUS3	turquoise	0.39201123
ENSG00000092036	HAUS4	grey	-0.0984039
ENSG00000249115	HAUS5	yellow	0.4784314
ENSG00000147874	HAUS6	blue	-0.3535753
ENSG00000268442	HAVCR1P1	grey	0.17685311
ENSG00000135077	HAVCR2	cyan	0.81754945
ENSG00000143575	HAX1	salmon	0.6319202
ENSG00000188536	HBA2	grey	0.11749341
ENSG00000244734	HBB	grey	0.10815998
ENSG00000113070	HBEGF	yellow	0.60292798
ENSG00000105856	HBP1	blue	-0.6300669
ENSG00000112339	HBS1L	grey	0.2130804
ENSG00000269235	HCCAT3	grey	9.59E-05
ENSG00000004961	HCCS	grey	-0.1205219

ENSG00000103145	HCFC1R1	blue	0.64444249
ENSG00000111727	HCFC2	turquoise	0.46690859
ENSG00000228223	HCG11	turquoise	0.53941792
ENSG00000270604	HCG17	grey	0.29748209
ENSG00000231074	HCG18	grey	0.16863715
ENSG00000228962	HCG23	grey	-0.0516507
ENSG00000232940	HCG25	grey	0.13167382
ENSG00000206344	HCG27	grey	0.29591632
ENSG00000176998	HCG4	grey	-0.060967
ENSG00000237669	HCG4P3	grey	0.37992476
ENSG00000204625	HCG9	grey	0.24202422
ENSG00000227758	HCG9P5	grey	0.13764572
ENSG00000101336	HCK	royalblue	0.86050252
ENSG00000180353	HCLS1	royalblue	0.88464736
ENSG00000164588	HCN1	blue	0.64036321
ENSG00000099822	HCN2	grey	0.0973341
ENSG00000143630	HCN3	blue	0.5017495
ENSG00000206337	HCP5	tan	0.56887739
ENSG00000126264	HCST	royalblue	0.50315082
ENSG00000116478	HDAC1	turquoise	0.71660529
ENSG00000163517	HDAC11	yellow	0.61931324
ENSG00000233012	HDAC1P2	grey	0.23252195
ENSG00000196591	HDAC2	turquoise	-0.3547483
ENSG00000171720	HDAC3	brown	0.63934542
ENSG00000068024	HDAC4	blue	-0.5703269
ENSG00000108840	HDAC5	purple	0.61965864
ENSG00000094631	HDAC6	blue	-0.2860318
ENSG00000061273	HDAC7	blue	-0.5638255
ENSG00000147099	HDAC8	turquoise	-0.3312596
ENSG00000048052	HDAC9	blue	0.56500072
ENSG00000111906	HDDC2	turquoise	0.42928666
ENSG00000184508	HDDC3	yellow	-0.4362536
ENSG00000143321	HDGF	brown	-0.7072346
ENSG00000167674	HDGFRP2	grey	-0.1117194
ENSG00000166503	HDGFRP3	grey	-0.1360069
ENSG00000130021	HDHD1	grey	-0.026125
ENSG00000167220	HDHD2	bisque4	0.73198158
ENSG00000119431	HDHD3	grey	0.18351102
ENSG00000115677	HDLBP	turquoise	-0.4231565
ENSG00000165259	HDX	purple	0.51488503
ENSG00000119285	HEATR1	grey	0.34130681
ENSG00000164818	HEATR2	grey	0.16086332
ENSG00000155393	HEATR3	turquoise	-0.427441
ENSG00000187105	HEATR4	grey	0.11142124
ENSG00000129493	HEATR5A	turquoise	0.69554573
ENSG00000008869	HEATR5B	brown	0.66338242
ENSG00000068097	HEATR6	grey	-0.0472831
ENSG00000013583	HEBP1	grey	-0.1825446

ENSG00000051620	HEBP2	darkmagenta	-0.7437408
ENSG00000112406	HECA	brown	-0.7413438
ENSG00000092148	HECTD1	salmon	0.78357628
ENSG00000126107	HECTD3	greenyellow	-0.4268233
ENSG00000173064	HECTD4	blue	0.73943663
ENSG00000002746	HECW1	blue	0.59116029
ENSG00000181211	HECW1-IT1	grey	0.29709867
ENSG00000138411	HECW2	red	-0.7592172
ENSG00000173706	HEG1	tan	0.64170437
ENSG00000127311	HELB	grey	0.14817412
ENSG00000163312	HELQ	grey	0.00871885
ENSG00000198265	HELZ	turquoise	0.50115645
ENSG00000114735	HEMK1	grey	-0.1971008
ENSG00000162639	HENMT1	brown	0.72880212
ENSG00000089472	HEPH	turquoise	0.71933421
ENSG00000103657	HERC1	turquoise	-0.740454
ENSG00000128731	HERC2	brown	0.61333557
ENSG00000140181	HERC2P2	tan	0.58039267
ENSG00000180229	HERC2P3	tan	0.63201995
ENSG00000206149	HERC2P9	tan	0.75126497
ENSG00000138641	HERC3	lightcyan	-0.7445622
ENSG00000138646	HERC5	grey	-0.186048
ENSG00000138642	HERC6	grey	-0.106754
ENSG00000051108	HERPUD1	tan	0.56051287
ENSG00000122557	HERPUD2	turquoise	0.38177476
ENSG00000114315	HES1	grey	-0.0720176
ENSG00000188290	HES4	grey	0.10732935
ENSG00000197921	HES5	yellowgreen	0.68848854
ENSG00000144485	HES6	blue	-0.3952429
ENSG00000213614	HEXA	green	-0.4659744
ENSG00000049860	HEXB	brown	0.7674292
ENSG00000169660	HEXDC	lightcyan	-0.6710781
ENSG00000186834	HEXIM1	grey	-0.0394883
ENSG00000168517	HEXIM2	grey	0.03276614
ENSG00000135547	HEY2	turquoise	0.61803341
ENSG00000010704	HFE	turquoise	0.44259808
ENSG00000185359	HGS	magenta	-0.4428631
ENSG00000054392	HHAT	turquoise	0.44366574
ENSG00000164161	HHIP	yellow	0.78676892
ENSG00000248890	HHIP-AS1	grey	0.27302334
ENSG00000182218	HHIPL1	turquoise	0.51326008
ENSG00000132297	HHLA1	grey	-0.1251518
ENSG00000156875	HIAT1	grey	0.20759111
ENSG00000148110	HIATL1	turquoise	0.62066537
ENSG00000196312	HIATL2	lightcyan	0.54664818
ENSG00000106049	HIBADH	turquoise	0.65673372
ENSG00000198130	HIBCH	turquoise	0.48616228
ENSG00000169635	HIC2	grey	-0.0783751

ENSG00000167861	HID1	turquoise	-0.3865882
ENSG00000100644	HIF1A	turquoise	0.48962263
ENSG00000258667	HIF1A-AS2	blue	-0.5021368
ENSG00000166135	HIF1AN	grey	0.27864546
ENSG00000124440	HIF3A	turquoise	0.41632773
ENSG00000181061	HIGD1A	turquoise	0.64299534
ENSG00000258886	HIGD1AP17	grey	0.37740366
ENSG00000237303	HIGD1AP8	grey	0.38460109
ENSG00000131097	HIGD1B	grey	0.13177742
ENSG00000146066	HIGD2A	yellow	-0.4078242
ENSG00000135245	HILPDA	pink	0.83537691
ENSG00000188662	HILS1	grey	0.16164546
ENSG00000172273	HINFP	brown	0.54766601
ENSG00000169567	HINT1	brown	0.85971339
ENSG00000137133	HINT2	blue	0.5117867
ENSG00000111911	HINT3	brown	-0.7004855
ENSG00000127946	HIP1	yellow	0.90304975
ENSG00000130787	HIP1R	yellow	0.52762089
ENSG00000163349	HIPK1	brown	-0.4838004
ENSG00000064393	HIPK2	green	0.93683509
ENSG00000110422	HIPK3	blue	-0.6659144
ENSG00000100084	HIRA	grey	0.32065262
ENSG00000149929	HIRIP3	darkgrey	0.73765642
ENSG00000124610	HIST1H1A	green	-0.3338558
ENSG00000187837	HIST1H1C	brown	-0.5710291
ENSG00000168298	HIST1H1E	salmon	-0.5610581
ENSG00000180573	HIST1H2AC	yellow	0.48162789
ENSG00000168274	HIST1H2AE	grey	0.10937591
ENSG00000184348	HIST1H2AK	grey	0.26222667
ENSG00000180596	HIST1H2BC	turquoise	0.46356379
ENSG00000197846	HIST1H2BF	royalblue	0.41718656
ENSG00000187990	HIST1H2BG	grey	-0.0359433
ENSG00000197903	HIST1H2BK	turquoise	-0.4790175
ENSG00000233822	HIST1H2BN	brown	0.32031109
ENSG00000197061	HIST1H4C	grey	-0.083777
ENSG00000188987	HIST1H4D	grey	-0.1496198
ENSG00000198518	HIST1H4E	brown	0.50886426
ENSG00000158406	HIST1H4H	grey	0.22558769
ENSG00000183558	HIST2H2AA3	blue	-0.5073099
ENSG00000184270	HIST2H2AB	grey	0.18053694
ENSG00000184260	HIST2H2AC	blue	-0.3477572
ENSG00000223345	HIST2H2BA	grey	0.27257754
ENSG00000184678	HIST2H2BE	blue	-0.5053264
ENSG00000203814	HIST2H2BF	yellow	0.42721204
ENSG00000181218	HIST3H2A	grey	0.07683927
ENSG00000197837	HIST4H4	grey	0.33208114
ENSG00000095951	HIVEP1	red	0.74676969
ENSG00000010818	HIVEP2	grey	0.14202676

ENSG00000127124	HIVEP3	grey	-0.1495898
ENSG00000156515	HK1	darkred	0.80947089
ENSG00000159399	HK2	blue	-0.460468
ENSG00000181666	HKR1	yellow	-0.4573657
ENSG00000206503	HLA-A	blue	-0.4787267
ENSG00000234745	HLA-B	blue	-0.4931634
ENSG00000204525	HLA-C	blue	-0.3926033
ENSG00000204257	HLA-DMA	cyan	0.77692775
ENSG00000242574	HLA-DMB	cyan	0.85021477
ENSG00000204252	HLA-DOA	cyan	0.77086108
ENSG00000231389	HLA-DPA1	cyan	0.83331094
ENSG00000237398	HLA-DPA3	grey	-0.126265
ENSG00000223865	HLA-DPB1	cyan	0.76359252
ENSG00000204287	HLA-DRA	cyan	0.86156673
ENSG00000196126	HLA-DRB1	royalblue	0.61321634
ENSG00000204592	HLA-E	tan	0.79832795
ENSG00000204642	HLA-F	lightcyan	-0.5853068
ENSG00000214922	HLA-F-AS1	grey	-0.0976011
ENSG00000204622	HLA-J	blue	-0.461118
ENSG00000230795	HLA-K	grey	0.23969708
ENSG00000243753	HLA-L	grey	-0.1612263
ENSG00000235290	HLA-W	grey	0.29716523
ENSG00000159267	HLCS	grey	-0.0239598
ENSG00000237646	HLCS-IT1	grey	0.3655668
ENSG00000108924	HLF	grey60	0.56172791
ENSG00000101294	HM13	brown	0.55009531
ENSG00000235313	HM13-IT1	grey	0.44696457
ENSG00000147421	HMBOX1	turquoise	0.65329691
ENSG00000256269	HMBS	grey	0.19555865
ENSG00000183624	HMCES	magenta	0.4009677
ENSG00000140382	HMG20A	blue	0.68657332
ENSG00000064961	HMG20B	blue	-0.5002334
ENSG00000137309	HMGA1	grey	-0.1042804
ENSG00000248641	HMGA1P2	blue	-0.4679105
ENSG00000189403	HMGB1	bisque4	0.80763271
ENSG00000180189	HMGB1P14	grey	0.38645971
ENSG00000253463	HMGB1P19	grey	0.39021344
ENSG00000248909	HMGB1P21	grey	0.13593962
ENSG00000253770	HMGB1P23	grey	0.13626011
ENSG00000254982	HMGB1P24	grey	0.43310058
ENSG00000233266	HMGB1P31	grey	0.29733864
ENSG00000253516	HMGB1P41	grey	0.30839144
ENSG00000164104	HMGB2	blue	-0.6088618
ENSG00000029993	HMGB3	grey	0.02935908
ENSG00000231845	HMGB3P14	grey	0.51371252
ENSG00000225051	HMGB3P22	grey	0.06505235
ENSG00000228808	HMGB3P4	grey	0.28732263
ENSG00000215399	HMGB3P7	grey	0.19016253

ENSG00000229992	HMGB3P9	grey	0.24916219
ENSG00000117305	HMGCL	grey	0.0606601
ENSG00000146151	HMGCLL1	blue	0.48843132
ENSG00000113161	HMGCR	brown	0.71897903
ENSG00000112972	HMGCS1	yellow	-0.4508113
ENSG00000205581	HMGN1	bisque4	0.76442852
ENSG00000247911	HMGN1P12	grey	0.40933859
ENSG00000249619	HMGN1P13	grey	0.3768798
ENSG00000249439	HMGN1P14	grey	-0.0524747
ENSG00000250197	HMGN1P15	grey	0.2514399
ENSG00000229046	HMGN1P2	grey	0.26666913
ENSG00000235734	HMGN1P36	grey	0.43035977
ENSG00000239614	HMGN1P7	grey	0.42762862
ENSG00000241120	HMGN1P8	grey	0.31373614
ENSG00000198830	HMGN2	blue	-0.4469114
ENSG00000214578	HMGN2P15	grey	0.46919669
ENSG00000232916	HMGN2P27	grey	0.4327596
ENSG00000236086	HMGN2P28	grey	0.38192047
ENSG00000232875	HMGN2P35	grey	0.47199599
ENSG00000231726	HMGN2P38	grey	0.4573258
ENSG00000179362	HMGN2P46	red	-0.6914033
ENSG00000234664	HMGN2P5	grey	0.55062564
ENSG00000118418	HMGN3	mediumpurp	0.68741072
ENSG00000270362	HMGN3-AS1	grey	0.06761854
ENSG00000182952	HMGN4	turquoise	0.69832265
ENSG00000113716	HMGXB3	grey	0.17611799
ENSG00000100281	HMGXB4	grey	0.09063701
ENSG00000100292	HMOX1	royalblue	0.66342423
ENSG00000103415	HMOX2	brown	0.7434783
ENSG00000189159	HN1	brown	0.87308324
ENSG00000206053	HN1L	darkred	-0.4303669
ENSG00000177733	HNRNPA0	mediumpurp	0.70451788
ENSG00000135486	HNRNPA1	grey	-0.2412397
ENSG00000139675	HNRNPA1L2	brown	0.42119147
ENSG00000227638	HNRNPA1P14	grey	0.02466181
ENSG00000262333	HNRNPA1P16	grey	0.22856158
ENSG00000225695	HNRNPA1P35	grey	0.20904593
ENSG00000212961	HNRNPA1P40	grey	0.3892964
ENSG00000233615	HNRNPA1P42	grey	0.0243465
ENSG00000259411	HNRNPA1P45	grey	-0.0515863
ENSG00000224578	HNRNPA1P48	brown	-0.4800019
ENSG00000233231	HNRNPA1P49	grey	0.27378919
ENSG00000257195	HNRNPA1P50	grey	0.31243453
ENSG00000259228	HNRNPA1P62	grey	0.27191004
ENSG00000230946	HNRNPA1P68	grey	0.40356642
ENSG00000215492	HNRNPA1P7	grey	0.28090774
ENSG00000254713	HNRNPA1P72	grey	0.2473762
ENSG00000255141	HNRNPA1P76	grey	0.22490045

ENSG00000122566	HNRNPA2B1	royalblue	0.32037746
ENSG00000170144	HNRNPA3	brown	-0.592515
ENSG00000257851	HNRNPA3P10	grey	0.0907734
ENSG00000213300	HNRNPA3P6	green	0.49690884
ENSG00000270903	HNRNPA3P9	grey	0.0308305
ENSG00000197451	HNRNPAB	pink	0.63945653
ENSG00000092199	HNRNPC	royalblue	0.48054998
ENSG00000258900	HNRNPCP1	grey	0.41780225
ENSG00000259419	HNRNPCP3	blue	0.45393319
ENSG00000213305	HNRNPCP6	grey	-0.0128973
ENSG00000228653	HNRNPCP7	grey	0.13244666
ENSG00000138668	HNRNPD	brown	-0.3409742
ENSG00000234890	HNRNPDLP2	grey	0.36706081
ENSG00000270837	HNRNPDLP4	grey	0.28315571
ENSG00000169813	HNRNPF	blue	-0.8387301
ENSG00000169045	HNRNPH1	blue	-0.7242519
ENSG00000220305	HNRNPH1P1	blue	-0.3649878
ENSG00000126945	HNRNPH2	grey	0.01595348
ENSG00000096746	HNRNPH3	turquoise	0.56225105
ENSG00000165119	HNRNPK	turquoise	-0.3112113
ENSG00000143889	HNRNPLL	grey	0.12305976
ENSG00000099783	HNRNPM	blue	-0.4655845
ENSG00000125944	HNRNPR	darkgrey	0.66863898
ENSG00000153187	HNRNPU	darkgrey	0.64508272
ENSG00000188206	HNRNPU-AS1	brown	-0.5329547
ENSG00000105323	HNRNPUL1	brown	-0.8594953
ENSG00000214753	HNRNPUL2	grey	-0.1508631
ENSG00000241935	HOGA1	cyan	-0.5104283
ENSG00000152413	HOMER1	magenta	0.43111304
ENSG00000103942	HOMER2	darkmagenta	-0.7341653
ENSG00000051128	HOMER3	darkorange	-0.5218732
ENSG00000215271	HOMEZ	blue	-0.5198908
ENSG00000134709	HOOK1	blue	0.57914594
ENSG00000095066	HOOK2	grey	-0.0239311
ENSG00000168172	HOOK3	black	-0.587605
ENSG00000171476	HOPX	red	0.45403386
ENSG00000224189	HOXD-AS1	yellow	0.67233662
ENSG00000121905	HPCA	magenta	0.7609309
ENSG00000115756	HPCAL1	magenta	-0.3535263
ENSG00000116983	HPCAL4	purple	0.73151492
ENSG00000186603	HPDL	turquoise	0.41109598
ENSG00000163106	HPGDS	cyan	0.75766508
ENSG00000105707	HPN	yellow	0.76852234
ENSG00000227392	HPN-AS1	yellow	0.63805616
ENSG00000261701	HPR	grey	0.12145873
ENSG00000165704	HPRT1	turquoise	-0.6918341
ENSG00000107521	HPS1	grey	0.05184503
ENSG00000163755	HPS3	cyan	0.50080472

ENSG00000100099	HPS4	brown	0.68505105
ENSG00000166189	HPS6	darkred	0.5699801
ENSG00000172987	HPSE2	turquoise	0.46543309
ENSG00000168453	HR	purple	-0.6044382
ENSG00000174775	HRAS	grey	-0.1504096
ENSG00000127252	HRASLS	blue	0.41487832
ENSG00000168004	HRASLS5	turquoise	0.57471903
ENSG00000196196	HRCT1	grey	0.20650788
ENSG00000113749	HRH2	magenta	0.67526994
ENSG00000101180	HRH3	grey60	0.45671707
ENSG00000132541	HRSP12	turquoise	0.5017739
ENSG00000118960	HS1BP3	grey	0.20394499
ENSG00000153936	HS2ST1	red	0.5948932
ENSG00000002587	HS3ST1	grey	0.14702973
ENSG00000182601	HS3ST4	grey	-0.4208882
ENSG00000249853	HS3ST5	floralwhite	0.75325212
ENSG00000136720	HS6ST1	turquoise	-0.5035526
ENSG00000171004	HS6ST2	blue	0.81144478
ENSG00000185352	HS6ST3	blue	0.49017304
ENSG00000141854	hsa-mir-1199	grey	-0.0500595
ENSG00000237973	hsa-mir-6723	grey	0.25444754
ENSG00000230989	HSBP1	red	0.62995095
ENSG00000226742	HSBP1L1	grey	0.23022748
ENSG00000100209	HSCB	darkred	0.5405652
ENSG00000117594	HSD11B1	black	0.64403595
ENSG00000167733	HSD11B1L	blue	0.69935672
ENSG00000108786	HSD17B1	brown	-0.4668926
ENSG00000072506	HSD17B10	grey	0.11981581
ENSG00000198189	HSD17B11	turquoise	0.43531928
ENSG00000149084	HSD17B12	turquoise	0.81957517
ENSG00000108785	HSD17B1P1	grey	0.22331857
ENSG00000130948	HSD17B3	yellow	0.5739314
ENSG00000133835	HSD17B4	blue	-0.4614386
ENSG00000025423	HSD17B6	turquoise	0.554241
ENSG00000132196	HSD17B7	grey	0.18693128
ENSG00000099251	HSD17B7P2	grey	0.08628984
ENSG00000099377	HSD3B7	pink	0.68047336
ENSG00000203855	HSD3BP4	grey	0.10028552
ENSG00000198857	HSD3BP5	grey	-0.1273223
ENSG00000103160	HSDL1	yellow	-0.7217836
ENSG00000119471	HSDL2	turquoise	0.82133808
ENSG00000025156	HSF2	grey	0.08865814
ENSG00000160207	HSF2BP	grey	-0.0650297
ENSG00000102878	HSF4	grey	-0.1257905
ENSG00000259706	HSP90B2P	salmon	-0.5504231
ENSG00000165868	HSPA12A	grey	0.24069808
ENSG00000132622	HSPA12B	tan	0.67675835
ENSG00000155304	HSPA13	green	-0.5751765

ENSG00000187522	HSPA14	brown	0.74346283
ENSG00000204390	HSPA1L	grey	0.03169911
ENSG00000126803	HSPA2	yellow	0.70785588
ENSG00000251614	HSPA8P19	grey	0.13066054
ENSG00000248610	HSPA8P4	grey	0.14086563
ENSG00000224773	HSPA8P7	blue	0.36115998
ENSG00000113013	HSPA9	grey	0.43324244
ENSG00000081870	HSPB11	grey	-0.3829415
ENSG00000170276	HSPB2	grey	0.07112276
ENSG00000004776	HSPB6	turquoise	0.65554491
ENSG00000152137	HSPB8	turquoise	0.49201369
ENSG00000169087	HSPBAP1	grey	-0.2170716
ENSG00000133265	HSPBP1	blue	0.60227835
ENSG00000251348	HSPD1P11	blue	-0.6205611
ENSG00000187762	HSPE1P11	grey	0.32266445
ENSG00000225900	HSPE1P13	grey	0.23775263
ENSG00000258645	HSPE1P2	grey	0.42057001
ENSG00000238059	HSPE1P21	grey	0.0519221
ENSG00000232015	HSPE1P25	grey	0.33402856
ENSG00000220867	HSPE1P26	grey	0.47702926
ENSG00000235112	HSPE1P27	grey	0.36236979
ENSG00000230081	HSPE1P28	salmon	0.58409234
ENSG00000120694	HSPH1	turquoise	-0.5674289
ENSG00000102241	HTATSF1	grey	-0.1005373
ENSG00000271361	HTATSF1P2	grey	0.25483349
ENSG00000135312	HTR1B	grey	-0.100309
ENSG00000179546	HTR1D	magenta	0.55500625
ENSG00000179097	HTR1F	grey	0.16478224
ENSG00000102468	HTR2A	purple	0.73271506
ENSG00000135914	HTR2B	grey	0.32070689
ENSG00000166736	HTR3A	grey	-0.0925511
ENSG00000164270	HTR4	magenta	0.74757965
ENSG00000253297	HTR4-IT1	grey	-0.0285292
ENSG00000157219	HTR5A	turquoise	-0.582765
ENSG00000158748	HTR6	magenta	0.73933284
ENSG00000183935	HTR7P1	grey	-0.0068039
ENSG00000115317	HTRA2	grey	0.10788318
ENSG00000197386	HTT	turquoise	-0.6433642
ENSG00000251164	HULC	grey	0.3912442
ENSG00000142149	HUNK	grey	0.05605393
ENSG00000136273	HUS1	grey	0.1849039
ENSG00000086758	HUWE1	grey	0.07834576
ENSG00000122986	HVCN1	turquoise	0.68551332
ENSG00000114378	HYAL1	tan	0.68311358
ENSG00000068001	HYAL2	pink	0.57579202
ENSG00000186792	HYAL3	grey	0.10295213
ENSG00000239475	HYDIN2	grey	0.02751375
ENSG00000178922	HYI	grey	-0.0032525

ENSG00000229348	HYI-AS1	grey	0.28190654
ENSG00000188266	HYKK	grey	0.15509292
ENSG00000198331	HYLS1	blue	0.58767611
ENSG00000149428	HYOU1	brown	0.6706537
ENSG00000242028	HYPK	grey	0.16705545
ENSG00000134330	IAH1	grey	-0.2907742
ENSG00000196305	IARS	brown	0.66817539
ENSG00000067704	IARS2	mediumpurp	0.5727294
ENSG00000181873	IBA57	turquoise	0.43842427
ENSG00000203684	IBA57-AS1	grey	0.15653678
ENSG00000005700	IBTK	green	0.61407707
ENSG00000003147	ICA1	blue	0.81396004
ENSG00000163596	ICA1L	yellow	-0.673359
ENSG00000090339	ICAM1	pink	0.66660289
ENSG00000108622	ICAM2	pink	0.53260882
ENSG00000076662	ICAM3	grey	0.22988936
ENSG00000112144	ICK	salmon	-0.5911089
ENSG00000116237	ICMT	black	0.74117385
ENSG00000160223	ICOSLG	yellow	0.74456894
ENSG00000167862	ICT1	grey	-0.0292433
ENSG00000125968	ID1	tan	0.4677347
ENSG00000115738	ID2	turquoise	0.54887454
ENSG00000117318	ID3	tan	0.57859381
ENSG00000172201	ID4	turquoise	0.74437506
ENSG00000119912	IDE	grey	0.25555146
ENSG00000138413	IDH1	turquoise	0.58522864
ENSG00000182054	IDH2	grey	0.03090862
ENSG00000166411	IDH3A	yellow	-0.3633721
ENSG00000101365	IDH3B	yellow	-0.4082855
ENSG00000067829	IDH3G	blue	0.74486903
ENSG00000067064	IDI1	grey	0.1617836
ENSG00000148377	IDI2	grey	0.30788409
ENSG00000232656	IDI2-AS1	purple	-0.4910542
ENSG00000010404	IDS	brown	0.62821014
ENSG00000241489	IDS	red	-0.5762353
ENSG00000127415	IDUA	grey	-0.0643375
ENSG00000137331	IER3	tan	0.49076685
ENSG00000134049	IER3IP1	grey	0.00868586
ENSG00000162783	IER5	grey	-0.0321991
ENSG00000188483	IER5L	grey	0.12424889
ENSG00000010295	IFFO1	turquoise	-0.468223
ENSG00000169991	IFFO2	turquoise	0.56814159
ENSG00000163565	IFI16	royalblue	0.8112443
ENSG00000165949	IFI27	tan	0.81734063
ENSG00000165948	IFI27L1	yellow	-0.5046671
ENSG00000119632	IFI27L2	brown	0.60904051
ENSG00000068079	IFI35	grey	0.18956352
ENSG00000137965	IFI44	grey	-0.1399114

ENSG00000137959	IFI44L	grey	-0.1468567
ENSG00000126709	IFI6	grey	0.05436389
ENSG00000115267	IFIH1	turquoise	0.42951393
ENSG00000185745	IFIT1	grey	-0.1413267
ENSG00000119922	IFIT2	yellow	0.69057126
ENSG00000152778	IFIT5	brown	-0.7871557
ENSG00000185885	IFITM1	pink	0.68546686
ENSG00000244242	IFITM10	darkorange	0.54130048
ENSG00000185201	IFITM2	pink	0.85522837
ENSG00000142089	IFITM3	pink	0.76557405
ENSG00000231195	IFNA11P	grey	0.42113112
ENSG00000226393	IFNA20P	grey	0.41656688
ENSG00000142166	IFNAR1	grey	0.3122422
ENSG00000159110	IFNAR2	yellowgreen	-0.6531201
ENSG00000027697	IFNGR1	grey	-0.0071236
ENSG00000159128	IFNGR2	turquoise	-0.3506608
ENSG00000147896	IFNK	grey	0.21028279
ENSG00000185436	IFNLR1	magenta	-0.4173313
ENSG00000214706	IFRD2	grey	0.26233319
ENSG00000163913	IFT122	brown	0.82088033
ENSG00000187535	IFT140	grey	0.06778148
ENSG00000138002	IFT172	brown	0.67943117
ENSG00000119650	IFT43	brown	0.66388001
ENSG00000118096	IFT46	yellow	-0.5219969
ENSG00000101052	IFT52	grey	-0.1039456
ENSG00000096872	IFT74	turquoise	0.39599068
ENSG00000068885	IFT80	grey	-0.1215066
ENSG00000122970	IFT81	grey	-0.0681228
ENSG00000032742	IFT88	grey	0.00222147
ENSG00000089289	IGBP1	turquoise	-0.5375196
ENSG00000203588	IGBP1-AS1	grey	0.40567166
ENSG00000017427	IGF1	grey	-0.3333335
ENSG00000140443	IGF1R	pink	0.54301231
ENSG00000167244	IGF2	grey	-0.1251298
ENSG00000073792	IGF2BP2	black	0.64135929
ENSG00000197081	IGF2R	blue	-0.5304636
ENSG00000141753	IGFBP4	blue	-0.4747248
ENSG00000115461	IGFBP5	grey	-0.2173669
ENSG00000167779	IGFBP6	brown	0.47289358
ENSG00000163453	IGFBP7	turquoise	0.46775274
ENSG00000137142	IGFBPL1	grey	-0.0201954
ENSG00000268879	IGFL1P1	grey	-0.0410963
ENSG00000204866	IGFL2	grey	0.24136549
ENSG00000188624	IGFL3	grey	0.12317679
ENSG00000204869	IGFL4	grey	-0.0490129
ENSG00000254017	IGHEP2	grey	0.11369444
ENSG00000132740	IGHMBP2	brown	0.63089513
ENSG00000182700	IGIP	darkgrey	0.68393024

ENSG00000254127	IGLCOR22-1	grey	0.15779013
ENSG00000142549	IGLON5	lightcyan	0.59043486
ENSG00000211645	IGLV1-50	grey	0.27513899
ENSG00000144847	IGSF11	yellow	0.65129738
ENSG00000239877	IGSF11-AS1	yellow	0.53594244
ENSG00000179057	IGSF22	brown	0.51043197
ENSG00000143061	IGSF3	blue	0.52913874
ENSG00000183067	IGSF5	red	-0.465853
ENSG00000140749	IGSF6	cyan	0.81664924
ENSG00000162729	IGSF8	yellow	0.51732148
ENSG00000080854	IGSF9B	turquoise	-0.5159992
ENSG00000113141	IK	salmon	0.63930783
ENSG00000166130	IKBIP	royalblue	0.47799096
ENSG00000070061	IKBKAP	turquoise	-0.7239481
ENSG00000104365	IKBKB	blue	-0.4770966
ENSG00000143466	IKBKE	grey	0.00107915
ENSG00000073009	IKBKG	grey	0.12327772
ENSG00000030419	IKZF2	blue	-0.6810638
ENSG00000123411	IKZF4	grey	0.06610018
ENSG00000095574	IKZF5	brown	-0.4484383
ENSG00000110324	IL10RA	cyan	0.75335565
ENSG00000243646	IL10RB	grey	0.21432834
ENSG00000223799	IL10RB-AS1	blue	-0.4211261
ENSG00000131724	IL13RA1	cyan	0.59862023
ENSG00000123496	IL13RA2	brown	0.6105427
ENSG00000172458	IL17D	turquoise	0.70361602
ENSG00000177663	IL17RA	red	-0.6337331
ENSG00000056736	IL17RB	turquoise	0.68084269
ENSG00000163702	IL17RC	grey	0.25592223
ENSG00000144730	IL17RD	turquoise	0.72609229
ENSG00000163701	IL17RE	grey	-0.0632774
ENSG00000150782	IL18	cyan	0.83258028
ENSG00000137496	IL18BP	pink	0.75270778
ENSG00000115594	IL1R1	pink	0.83364158
ENSG00000196083	IL1RAP	grey	0.24077266
ENSG00000169306	IL1RAPL1	turquoise	-0.480677
ENSG00000189108	IL1RAPL2	grey	0.17034036
ENSG00000137033	IL33	turquoise	0.60831046
ENSG00000157368	IL34	blue	0.56083995
ENSG00000185291	IL3RA	grey	-0.001153
ENSG00000077238	IL4R	tan	0.53192659
ENSG00000113525	IL5	grey	0.23262394
ENSG00000160712	IL6R	royalblue	0.70634477
ENSG00000134352	IL6ST	purple	-0.7482051
ENSG00000104432	IL7	grey	-0.0415444
ENSG00000145839	IL9	grey	0.06461483
ENSG00000143621	ILF2	lightgreen	-0.3999957
ENSG00000267100	ILF3-AS1	grey	-0.0652981

ENSG00000166333	ILK	grey	0.25274714
ENSG00000132323	ILKAP	lightcyan	-0.8158971
ENSG00000105135	ILVBL	green	-0.5655188
ENSG00000148950	IMMP1L	grey	-0.1382331
ENSG00000184903	IMMP2L	grey	0.0070677
ENSG00000132305	IMMT	brown	0.77154312
ENSG00000177971	IMP3	grey	-0.2649426
ENSG00000136718	IMP4	grey	0.0099575
ENSG00000133731	IMPA1	turquoise	0.43508775
ENSG00000141401	IMPA2	turquoise	0.67838915
ENSG00000154059	IMPACT	grey	-0.2137067
ENSG00000104331	IMPAD1	turquoise	0.59910561
ENSG00000106348	IMPDH1	grey60	0.68549935
ENSG00000178035	IMPDH2	brown	0.53051944
ENSG00000112706	IMPG1	grey	-0.1671454
ENSG00000081148	IMPG2	blue	0.38213249
ENSG00000148798	INA	blue	0.72243516
ENSG00000132849	INADL	blue	0.33473539
ENSG00000196388	INCA1	brown	-0.3905126
ENSG00000149503	INCENP	grey	0.231882
ENSG00000224975	INE1	grey	0.11398952
ENSG00000203485	INF2	red	-0.6533067
ENSG00000153487	ING1	blue	-0.4702828
ENSG00000168556	ING2	blue	-0.4570061
ENSG00000111653	ING4	brown	0.59161965
ENSG00000168395	ING5	grey	-0.1498834
ENSG00000122641	INHBA	grey	-0.0533199
ENSG00000224116	INHBA-AS1	blue	0.65111425
ENSG00000163083	INHBB	turquoise	0.81033536
ENSG00000148153	INIP	brown	-0.5815699
ENSG00000241644	INMT	purple	-0.6552331
ENSG00000128908	INO80	grey	-0.0303516
ENSG00000115274	INO80B	blue	0.52440731
ENSG00000153391	INO80C	brown	0.64676273
ENSG00000114933	INO80D	grey60	-0.6198405
ENSG00000151689	INPP1	grey	-0.279698
ENSG00000040933	INPP4A	blue	0.70797227
ENSG00000109452	INPP4B	blue	0.3957149
ENSG00000068383	INPP5A	grey60	0.72936589
ENSG00000204084	INPP5B	grey	0.43957713
ENSG00000168918	INPP5D	cyan	0.76181491
ENSG00000148384	INPP5E	grey	-0.1727695
ENSG00000198825	INPP5F	blue	0.82933756
ENSG00000132376	INPP5K	grey	0.17450055
ENSG00000165458	INPPL1	blue	-0.5945781
ENSG00000186480	INSIG1	grey	0.0720218
ENSG00000125629	INSIG2	grey	0.10009768
ENSG00000171105	INSR	blue	-0.6358791

ENSG00000164880	INTS1	grey	0.23313189
ENSG00000104613	INTS10	salmon	0.59670893
ENSG00000138785	INTS12	grey	0.06439017
ENSG00000108506	INTS2	blue	-0.6067097
ENSG00000143624	INTS3	turquoise	-0.6555511
ENSG00000149262	INTS4	brown	0.30510956
ENSG00000164669	INTS4L1	grey	0.10388953
ENSG00000232270	INTS4L2	grey	0.30688075
ENSG00000185085	INTS5	grey	0.21982645
ENSG00000102786	INTS6	grey	-0.0355635
ENSG00000143493	INTS7	brown	0.62481005
ENSG00000164941	INTS8	grey	0.01218373
ENSG00000104299	INTS9	grey	0.2999846
ENSG00000164066	INTU	brown	0.44692064
ENSG00000119509	INVS	grey	-0.0924499
ENSG00000176095	IP6K1	brown	0.5104719
ENSG00000068745	IP6K2	salmon	0.64284384
ENSG00000161896	IP6K3	yellow	0.75601201
ENSG00000074706	IPCEF1	blue	0.65913176
ENSG00000151151	IPMK	turquoise	0.57649814
ENSG00000086200	IPO11	lightgreen	-0.592626
ENSG00000117408	IPO13	floralwhite	0.77762297
ENSG00000065150	IPO5	grey	0.06259245
ENSG00000205339	IPO7	grey	-0.1154387
ENSG00000133704	IPO8	grey	0.08082876
ENSG00000224668	IPO8P1	magenta	0.36482415
ENSG00000198700	IPO9	blue	0.56126183
ENSG00000197429	IPP	grey	0.20760265
ENSG00000127080	IPPK	grey	0.3578267
ENSG00000132321	IQCA1	turquoise	0.35478127
ENSG00000173226	IQCB1	grey	0.13306653
ENSG00000160051	IQCC	brown	0.31014184
ENSG00000166578	IQCD	grey	0.05822335
ENSG00000106012	IQCE	grey	0.09914414
ENSG00000103599	IQCH	grey	0.3650193
ENSG00000259673	IQCH-AS1	turquoise	0.61999653
ENSG00000214216	IQCJ	grey	0.13435976
ENSG00000250588	IQCJ-SCHIP1	grey	0.31130343
ENSG00000241211	IQCJ-SCHIP1-AS1	grey	0.43477084
ENSG00000174628	IQCK	yellowgreen	0.58735065
ENSG00000140575	IQGAP1	yellow	0.79478835
ENSG00000144711	IQSEC1	grey	-0.2283274
ENSG00000124313	IQSEC2	turquoise	-0.6006113
ENSG00000184216	IRAK1	turquoise	0.41685831
ENSG00000146243	IRAK1BP1	grey	0.27953905
ENSG00000134070	IRAK2	yellow	0.48532054
ENSG00000090376	IRAK3	pink	0.68252648
ENSG00000198001	IRAK4	grey	-0.0758958

ENSG00000136381	IREB2	grey	0.09415512
ENSG00000125347	IRF1	pink	0.65314187
ENSG00000168310	IRF2	turquoise	0.64447492
ENSG00000170604	IRF2BP1	grey	0.08669804
ENSG00000168264	IRF2BP2	blue	-0.6906565
ENSG00000119669	IRF2BPL	brown	-0.4026804
ENSG00000126456	IRF3	brown	0.31764756
ENSG00000185507	IRF7	pink	0.74508762
ENSG00000140968	IRF8	cyan	0.72862678
ENSG00000213928	IRF9	grey	0.0670751
ENSG00000167378	IRGQ	turquoise	0.55223052
ENSG00000185950	IRS2	black	-0.5432291
ENSG00000135070	ISCA1	grey	0.11798495
ENSG00000165898	ISCA2	turquoise	0.72465496
ENSG00000136003	ISCU	brown	0.6447456
ENSG00000187608	ISG15	grey	0.0710777
ENSG00000143319	ISG20L2	grey	0.15332265
ENSG00000016082	ISL1	magenta	0.54134944
ENSG00000129009	ISLR	grey	0.01750609
ENSG00000167178	ISLR2	magenta	0.70279518
ENSG00000066583	ISOC1	grey	-0.2788057
ENSG00000063241	ISOC2	brown	0.38272929
ENSG00000214960	ISPD	grey	0.17187006
ENSG00000182149	IST1	grey	0.19973703
ENSG00000240682	ISY1	grey	0.21817757
ENSG00000105655	ISYNA1	yellowgreen	0.60650343
ENSG00000078747	ITCH	yellow	0.7714404
ENSG00000231795	ITCH-IT1	grey	0.3867034
ENSG00000129636	ITFG1	green	-0.8368819
ENSG00000111203	ITFG2	grey	-0.202138
ENSG00000167930	ITFG3	grey	0.01765367
ENSG00000213949	ITGA1	pink	0.59148448
ENSG00000143127	ITGA10	grey	0.36174838
ENSG00000164171	ITGA2	darkred	-0.7105986
ENSG00000005884	ITGA3	blue	0.68653449
ENSG00000161638	ITGA5	pink	0.6271243
ENSG00000091409	ITGA6	blue	-0.7368147
ENSG00000135424	ITGA7	grey	-0.1616959
ENSG00000077943	ITGA8	grey	0.12742046
ENSG00000169896	ITGAM	royalblue	0.85767718
ENSG00000138448	ITGAV	turquoise	0.64989066
ENSG00000140678	ITGAX	cyan	0.75306545
ENSG00000150093	ITGB1	brown	-0.861241
ENSG00000119185	ITGB1BP1	salmon	0.65110779
ENSG00000269378	ITGB1P1	grey	0.43090137
ENSG00000160255	ITGB2	cyan	0.78034654
ENSG00000142856	ITGB3BP	grey	0.05145348
ENSG00000132470	ITGB4	blue	-0.5999065

ENSG00000082781	ITGB5	turquoise	0.6311951
ENSG00000105855	ITGB8	blue	-0.7510454
ENSG00000198542	ITGBL1	red	-0.5782731
ENSG00000151655	ITIH2	grey	-0.0358484
ENSG00000123243	ITIH5	tan	0.47597448
ENSG00000078596	ITM2A	tan	0.78083276
ENSG00000136156	ITM2B	turquoise	0.47650229
ENSG00000135916	ITM2C	turquoise	0.37834868
ENSG00000125877	ITPA	brown	0.6744279
ENSG00000100605	ITPK1	yellow	0.55987933
ENSG00000137825	ITPKA	magenta	0.83967465
ENSG00000143772	ITPKB	turquoise	0.77609647
ENSG00000228548	ITPKB-AS1	grey	0.16218945
ENSG00000086544	ITPKC	pink	0.84877961
ENSG00000150995	ITPR1	grey60	0.73638982
ENSG00000231249	ITPR1-AS1	grey	0.2376611
ENSG00000123104	ITPR2	turquoise	0.54335787
ENSG00000096433	ITPR3	tan	0.68512449
ENSG00000148841	ITPRIP	pink	0.68093746
ENSG00000205730	ITPRIPL2	pink	0.64949102
ENSG00000205726	ITSN1	yellow	-0.4694358
ENSG00000198399	ITSN2	grey	0.2518569
ENSG00000128928	IVD	grey	0.22830238
ENSG00000116679	IVNS1ABP	black	0.88179793
ENSG00000163166	IWS1	red	-0.5648526
ENSG00000099840	IZUMO4	yellow	0.42643888
ENSG00000077684	JADE1	grey	0.19764235
ENSG00000043143	JADE2	turquoise	-0.4470229
ENSG00000102221	JADE3	brown	-0.5362755
ENSG00000101384	JAG1	grey	0.01865633
ENSG00000184916	JAG2	grey	-0.0505622
ENSG00000171135	JAGN1	grey	0.10735944
ENSG00000162434	JAK1	darkmagenta	-0.6385989
ENSG00000096968	JAK2	grey	-0.003586
ENSG00000105639	JAK3	royalblue	0.59855288
ENSG00000176049	JAKMIP2	grey	-0.1235626
ENSG00000188385	JAKMIP3	grey	-0.1421987
ENSG00000154721	JAM2	turquoise	0.67842694
ENSG00000166086	JAM3	yellow	0.84333067
ENSG00000008083	JARID2	yellow	0.57009791
ENSG00000153814	JAZF1	blue	0.55766334
ENSG00000234336	JAZF1-AS1	grey	0.08471223
ENSG00000140044	JDP2	blue	-0.5033009
ENSG00000260231	JHDM1D-AS1	grey	0.40137675
ENSG00000050130	JKAMP	grey	-0.1952333
ENSG00000171988	JMJD1C	blue	-0.3850327
ENSG00000081692	JMJD4	grey	0.25206895
ENSG00000161999	JMJD8	grey	0.13352902

ENSG00000152409	JMY	grey	0.1272193
ENSG00000100221	JOSD1	blue	-0.3774831
ENSG00000161677	JOSD2	grey	0.07015445
ENSG00000104369	JPH1	red	-0.4890335
ENSG00000154118	JPH3	darkorange	0.80419921
ENSG00000092051	JPH4	darkorange	0.7072012
ENSG00000225470	JPX	salmon	-0.5535775
ENSG00000234616	JRK	grey	0.07901814
ENSG00000183340	JRKL	turquoise	0.4289904
ENSG00000233603	JTBP1	grey	0.38726388
ENSG00000130522	JUND	red	-0.4798924
ENSG00000173801	JUP	yellow	0.61105577
ENSG00000011201	KAL1	turquoise	0.67361816
ENSG00000241859	KALP	grey	-0.0720111
ENSG00000160145	KALRN	blue	0.47841573
ENSG00000107104	KANK1	blue	-0.7124996
ENSG00000197256	KANK2	brown	-0.5974709
ENSG00000186994	KANK3	tan	0.81510934
ENSG00000120071	KANSL1	grey	0.0190375
ENSG00000214401	KANSL1-AS1	grey	0.06926697
ENSG00000144445	KANSL1L	grey	-0.0378833
ENSG00000139620	KANSL2	lightcyan	-0.4499084
ENSG00000114982	KANSL3	darkorange	-0.4712405
ENSG00000065427	KARS	brown	0.69087868
ENSG00000229696	KARSP1	grey	0.29314746
ENSG00000114166	KAT2B	blue	-0.692176
ENSG00000172977	KAT5	grey	0.08724284
ENSG00000083168	KAT6A	turquoise	0.45059968
ENSG00000156650	KAT6B	bisque4	0.63107925
ENSG00000136504	KAT7	blue	0.45841859
ENSG00000103510	KAT8	grey	0.14434695
ENSG00000186625	KATNA1	yellow	0.49612909
ENSG00000102781	KATNAL1	grey	0.0898268
ENSG00000167216	KATNAL2	turquoise	0.47681108
ENSG00000140854	KATNB1	brown	0.70750851
ENSG00000134152	KATNBL1	red	0.58773003
ENSG00000189337	KAZN	yellow	0.52196449
ENSG00000260640	KB-1000E4.2	grey	0.40603133
ENSG00000254112	KB-1205A7.1	yellow	0.33940427
ENSG00000261087	KB-1460A1.5	grey	0.06498007
ENSG00000253320	KB-1507C5.2	grey	-0.0276915
ENSG00000253263	KB-1507C5.3	grey	0.25198761
ENSG00000254281	KB-1507C5.4	grey	0.31083254
ENSG00000253633	KB-1980E6.3	grey	-0.0263374
ENSG00000232545	KB-318B8.7	grey	0.10522775
ENSG00000246263	KB-431C1.4	turquoise	0.455125
ENSG00000176595	KBTBD11	blue	-0.6745581
ENSG00000253696	KBTBD11-OT1	pink	0.50672977

ENSG00000187715	KBTBD12	red	0.56035345
ENSG00000170852	KBTBD2	darkorange	-0.5377175
ENSG00000182359	KBTBD3	grey	0.08314308
ENSG00000123444	KBTBD4	grey	0.29939547
ENSG00000165572	KBTBD6	black	-0.6097772
ENSG00000120696	KBTBD7	grey	0.0041212
ENSG00000163376	KBTBD8	grey	0.22957136
ENSG00000176407	KCMF1	grey	0.08170699
ENSG00000177301	KCNA2	grey	-0.0048269
ENSG00000177272	KCNA3	purple	0.73968667
ENSG00000182255	KCNA4	purple	0.40136775
ENSG00000130037	KCNA5	grey	-0.2238
ENSG00000151079	KCNA6	grey	0.06123202
ENSG00000169282	KCNAB1	magenta	0.82033281
ENSG00000242370	KCNAB1-AS1	black	0.58826899
ENSG00000158445	KCNB1	darkorange	0.69040896
ENSG00000182674	KCNB2	purple	0.57789408
ENSG00000102057	KCND1	turquoise	0.42573838
ENSG00000184408	KCND2	grey60	0.63186319
ENSG00000171385	KCND3	yellow	-0.5342404
ENSG00000237556	KCND3-AS1	grey	0.24269878
ENSG00000232558	KCND3-IT1	grey	0.24046139
ENSG00000152049	KCNE4	pink	0.46432319
ENSG00000162975	KCNF1	darkorange	0.71094764
ENSG00000171126	KCNG3	grey	-0.1772804
ENSG00000143473	KCNH1	magenta	0.73375349
ENSG00000234233	KCNH1-IT1	grey	0.09104162
ENSG00000055118	KCNH2	purple	0.46843609
ENSG00000135519	KCNH3	magenta	0.69179456
ENSG00000089558	KCNH4	magenta	0.80111269
ENSG00000184611	KCNH7	purple	0.49787406
ENSG00000183960	KCNH8	yellow	0.80270609
ENSG00000182132	KCNIP1	grey	-0.1428073
ENSG00000120049	KCNIP2	darkorange	0.66241332
ENSG00000226009	KCNIP2-AS1	grey	0.04162001
ENSG00000115041	KCNIP3	yellowgreen	0.77653393
ENSG00000185774	KCNIP4	blue	0.61406842
ENSG00000177807	KCNJ10	turquoise	0.77461934
ENSG00000187486	KCNJ11	grey	0.188335
ENSG00000184185	KCNJ12	blue	0.26069391
ENSG00000115474	KCNJ13	grey	0.40491497
ENSG00000123700	KCNJ2	yellow	0.79267065
ENSG00000267365	KCNJ2-AS1	brown	-0.4204619
ENSG00000162989	KCNJ3	grey	0.0600994
ENSG00000121361	KCNJ8	turquoise	0.64196039
ENSG00000162728	KCNJ9	darkgrey	0.54988024
ENSG00000135750	KCNK1	magenta	0.48180644
ENSG00000100433	KCNK10	yellow	0.51285749

ENSG00000184261	KCNK12	grey	0.25416269
ENSG00000082482	KCNK2	grey60	0.73954469
ENSG00000156113	KCNMA1	blue	0.58467238
ENSG00000135643	KCNMB4	grey	0.17754509
ENSG00000105642	KCNN1	magenta	0.53728262
ENSG00000080709	KCNN2	blue	0.2798748
ENSG00000143603	KCNN3	turquoise	0.60427725
ENSG00000269821	KCNQ1OT1	grey	-0.1075862
ENSG00000075043	KCNQ2	blue	0.69510754
ENSG00000184156	KCNQ3	blue	0.76723815
ENSG00000117013	KCNQ4	grey	0.1229308
ENSG00000185760	KCNQ5	magenta	0.82304734
ENSG00000229154	KCNQ5-AS1	magenta	0.53970682
ENSG00000233844	KCNQ5-IT1	magenta	0.44928531
ENSG00000198553	KCNRG	blue	-0.4526272
ENSG00000156486	KCNS2	black	0.62443079
ENSG00000170745	KCNS3	grey	0.04806617
ENSG00000107147	KCNT1	black	0.77109219
ENSG00000162687	KCNT2	magenta	0.34465847
ENSG00000164794	KCNV1	blue	0.3632547
ENSG00000168263	KCNV2	grey	0.27975156
ENSG00000134504	KCTD1	magenta	0.67970185
ENSG00000110906	KCTD10	grey	0.2195334
ENSG00000213859	KCTD11	grey60	-0.4243751
ENSG00000178695	KCTD12	grey	-0.320079
ENSG00000174943	KCTD13	blue	0.56161926
ENSG00000153885	KCTD15	turquoise	0.71698828
ENSG00000183775	KCTD16	darkorange	0.75906708
ENSG00000100379	KCTD17	grey60	0.80404748
ENSG00000155729	KCTD18	green	0.60848801
ENSG00000180901	KCTD2	brown	0.60625114
ENSG00000112078	KCTD20	darkorange	-0.6443588
ENSG00000188997	KCTD21	grey	0.0355886
ENSG00000136636	KCTD3	green	0.81159612
ENSG00000180332	KCTD4	purple	0.75892959
ENSG00000167977	KCTD5	grey	0.26331645
ENSG00000168301	KCTD6	blue	-0.6446772
ENSG00000243335	KCTD7	grey	-0.3045039
ENSG00000183783	KCTD8	blue	0.66187217
ENSG00000104756	KCTD9	turquoise	0.58125072
ENSG00000225014	KCTD9P1	blue	-0.3557108
ENSG00000250329	KDELC1P1	grey	0.20977324
ENSG00000178202	KDELC2	turquoise	0.72624363
ENSG00000105438	KDELR1	turquoise	0.29940108
ENSG00000136240	KDELR2	turquoise	0.55069102
ENSG00000100196	KDELR3	grey	0.03066766
ENSG00000004487	KDM1A	grey	-0.2057537
ENSG00000165097	KDM1B	brown	-0.4878339

ENSG00000173120	KDM2A	turquoise	-0.5103919
ENSG00000089094	KDM2B	grey	0.07996393
ENSG00000115548	KDM3A	grey	0.04917671
ENSG00000120733	KDM3B	grey	-0.0073874
ENSG00000066135	KDM4A	turquoise	-0.4645249
ENSG00000127663	KDM4B	grey	0.20362882
ENSG00000107077	KDM4C	grey	0.24392654
ENSG00000073614	KDM5A	darkgrey	0.59896995
ENSG00000117139	KDM5B	brown	0.60097681
ENSG00000126012	KDM5C	grey	0.03604243
ENSG00000012817	KDM5D	grey	-0.1384444
ENSG00000147050	KDM6A	blue	-0.4934887
ENSG00000132510	KDM6B	grey	0.26803982
ENSG00000006459	KDM7A	black	0.53017886
ENSG00000155666	KDM8	grey	0.13510016
ENSG00000128052	KDR	yellow	0.60622928
ENSG00000119537	KDSR	turquoise	0.61354133
ENSG00000079999	KEAP1	turquoise	0.55648898
ENSG00000197993	KEL	yellow	0.76652472
ENSG00000135314	KHDC1	salmon	0.50493646
ENSG00000121774	KHDRBS1	grey	-0.2952474
ENSG00000112232	KHDRBS2	purple	0.76129121
ENSG00000131773	KHDRBS3	grey	-0.0941479
ENSG00000138030	KHK	brown	0.46657726
ENSG00000100441	KHNYN	blue	-0.7638763
ENSG00000080608	KIAA0020	grey	-0.147782
ENSG00000122548	KIAA0087	blue	0.37763491
ENSG00000007202	KIAA0100	turquoise	-0.4555944
ENSG00000081791	KIAA0141	turquoise	0.46644106
ENSG00000177728	KIAA0195	turquoise	0.4220154
ENSG00000164961	KIAA0196	grey	0.04086275
ENSG00000253167	KIAA0196-AS1	yellow	0.38534717
ENSG00000145016	KIAA0226	turquoise	-0.5760682
ENSG00000170871	KIAA0232	greenyellow	-0.3719005
ENSG00000100647	KIAA0247	blue	-0.6896914
ENSG00000137261	KIAA0319	red	0.67594621
ENSG00000142687	KIAA0319L	brown	0.63292992
ENSG00000166398	KIAA0355	yellow	0.70060261
ENSG00000136813	KIAA0368	lightcyan	-0.7348876
ENSG00000166783	KIAA0430	grey	0.07562074
ENSG00000135709	KIAA0513	purple	0.74170141
ENSG00000047578	KIAA0556	grey	0.25810747
ENSG00000100578	KIAA0586	grey	0.12499528
ENSG00000198920	KIAA0753	turquoise	-0.7019959
ENSG00000255103	KIAA0754	grey	-0.0851165
ENSG00000185261	KIAA0825	purple	0.7626888
ENSG00000164542	KIAA0895	grey	0.27139232
ENSG00000132680	KIAA0907	grey	-0.0413752

ENSG00000121210	KIAA0922	darkmagenta	0.69955089
ENSG00000100364	KIAA0930	yellow	0.7383479
ENSG00000164151	KIAA0947	grey	0.17719088
ENSG00000136051	KIAA1033	grey	-0.2527188
ENSG00000122733	KIAA1045	blue	0.70125148
ENSG00000069712	KIAA1107	grey60	0.71943981
ENSG00000138688	KIAA1109	brown	0.3661865
ENSG00000163807	KIAA1143	lightcyan	0.78978989
ENSG00000257093	KIAA1147	green	0.80510011
ENSG00000164976	KIAA1161	turquoise	0.86287289
ENSG00000122203	KIAA1191	lightcyan	0.66225984
ENSG00000103888	KIAA1199	grey	-0.1099377
ENSG00000109265	KIAA1211	turquoise	0.46263067
ENSG00000196872	KIAA1211L	magenta	0.86059479
ENSG00000120549	KIAA1217	grey	0.14098097
ENSG00000112379	KIAA1244	pink	-0.523432
ENSG00000114656	KIAA1257	blue	0.67475167
ENSG00000164659	KIAA1324L	yellow	0.44319919
ENSG00000110318	KIAA1377	blue	0.70196466
ENSG00000164944	KIAA1429	grey	0.27603468
ENSG00000164323	KIAA1430	brown	-0.6042562
ENSG00000250305	KIAA1456	grey	0.19290154
ENSG00000165757	KIAA1462	black	0.72344599
ENSG00000134444	KIAA1468	turquoise	-0.7092193
ENSG00000162522	KIAA1522	grey	0.12597221
ENSG00000110427	KIAA1549L	darkorange	0.7080343
ENSG00000174718	KIAA1551	yellow	0.54263988
ENSG00000168116	KIAA1586	brown	-0.6392134
ENSG00000187164	KIAA1598	green	0.86742746
ENSG00000135835	KIAA1614	yellow	0.39728804
ENSG00000138944	KIAA1644	purple	0.64842635
ENSG00000268538	KIAA1658	grey	0.50036109
ENSG00000197077	KIAA1671	red	0.62381207
ENSG00000144320	KIAA1715	grey	0.14454145
ENSG00000166004	KIAA1731	grey	0.00773811
ENSG00000149633	KIAA1755	yellow	0.77809382
ENSG00000162929	KIAA1841	turquoise	-0.4555303
ENSG00000173214	KIAA1919	turquoise	-0.4542658
ENSG00000116685	KIAA2013	grey	0.01259858
ENSG00000176542	KIAA2018	grey	-0.2075314
ENSG00000050030	KIAA2022	purple	0.7993495
ENSG00000183354	KIAA2026	bisque4	0.84087391
ENSG00000134313	KIDINS220	lightcyan	-0.5030046
ENSG00000138160	KIF11	grey	-0.0303862
ENSG00000136883	KIF12	turquoise	-0.3940993
ENSG00000137177	KIF13A	green	0.8089264
ENSG00000197892	KIF13B	yellow	0.84454569
ENSG00000163808	KIF15	grey	0.17254031

ENSG00000089177	KIF16B	darkgrey	0.66521093
ENSG00000117245	KIF17	blue	0.63147574
ENSG00000130294	KIF1A	red	0.51640266
ENSG00000054523	KIF1B	brown	0.43896696
ENSG00000129250	KIF1C	green	0.86344897
ENSG00000138182	KIF20B	grey	-0.0822783
ENSG00000116852	KIF21B	purple	0.81384809
ENSG00000079616	KIF22	turquoise	-0.3850901
ENSG00000186638	KIF24	grey	0.09228321
ENSG00000223519	KIF28P	grey	0.04840636
ENSG00000068796	KIF2A	lightgreen	0.72623174
ENSG00000131437	KIF3A	blue	0.68253703
ENSG00000101350	KIF3B	turquoise	-0.3399996
ENSG00000084731	KIF3C	blue	0.85241286
ENSG00000155980	KIF5A	grey	-0.1181232
ENSG00000170759	KIF5B	green	0.86285652
ENSG00000168280	KIF5C	grey	-0.3804602
ENSG00000164627	KIF6	yellow	0.46973598
ENSG00000088727	KIF9	brown	0.62031189
ENSG00000227398	KIF9-AS1	grey	0.29166535
ENSG00000075945	KIFAP3	green	-0.8158938
ENSG00000167702	KIFC2	blue	0.42408502
ENSG00000140859	KIFC3	grey	0.03170548
ENSG00000151657	KIN	grey	-0.0378331
ENSG00000183853	KIRREL	grey	0.20211867
ENSG00000149571	KIRREL3	darkorange	0.58112388
ENSG00000257271	KIRREL3-AS1	grey	0.33029042
ENSG00000254960	KIRREL3-AS2	turquoise	-0.4476599
ENSG00000157404	KIT	grey	-0.0823726
ENSG00000133116	KL	blue	0.31344825
ENSG00000134962	KLB	grey	0.0976784
ENSG00000174996	KLC2	blue	0.54456466
ENSG00000137171	KLC4	grey	-0.1956831
ENSG00000155090	KLF10	pink	0.3447597
ENSG00000172059	KLF11	grey	0.30598712
ENSG00000118922	KLF12	red	0.75189537
ENSG00000163884	KLF15	blue	-0.7291325
ENSG00000129911	KLF16	grey	-0.4041015
ENSG00000127528	KLF2	tan	0.65638085
ENSG00000109787	KLF3	yellow	0.56320268
ENSG00000253218	KLF3P1	grey	-0.0300245
ENSG00000067082	KLF6	pink	0.69310142
ENSG00000118263	KLF7	turquoise	0.46777325
ENSG00000237892	KLF7-IT1	grey	0.30330066
ENSG00000102349	KLF8	brown	0.55546014
ENSG00000119138	KLF9	black	0.66213724
ENSG00000197776	KLHDC1	lightgreen	0.73120124
ENSG00000128607	KLHDC10	turquoise	0.65623511

ENSG00000124702	KLHDC3	turquoise	-0.6301479
ENSG00000104731	KLHDC4	brown	0.59471582
ENSG00000162873	KLHDC8A	purple	0.66024497
ENSG00000185909	KLHDC8B	red	0.74645968
ENSG00000150361	KLHL1	grey	-0.1634667
ENSG00000178502	KLHL11	turquoise	0.42859213
ENSG00000003096	KLHL13	blue	0.73135549
ENSG00000174010	KLHL15	blue	-0.6313748
ENSG00000114648	KLHL18	brown	0.60349691
ENSG00000109466	KLHL2	red	-0.6691092
ENSG00000076321	KLHL20	brown	-0.7266661
ENSG00000162413	KLHL21	blue	-0.5272567
ENSG00000099910	KLHL22	grey	-0.1496186
ENSG00000213160	KLHL23	turquoise	0.61370352
ENSG00000183655	KLHL25	grey	-0.1246183
ENSG00000167487	KLHL26	turquoise	0.66257854
ENSG00000179454	KLHL28	grey	0.14977575
ENSG00000119771	KLHL29	darkorange	0.51985062
ENSG00000250412	KLHL2P1	grey	0.25018536
ENSG00000146021	KLHL3	grey	0.06616494
ENSG00000186231	KLHL32	yellow	0.68658068
ENSG00000185915	KLHL34	green	0.64276396
ENSG00000149243	KLHL35	brown	0.58119636
ENSG00000135686	KLHL36	turquoise	0.56977784
ENSG00000102271	KLHL4	yellow	0.61503837
ENSG00000239474	KLHL41	grey	0.34833616
ENSG00000087448	KLHL42	grey	-0.2733758
ENSG00000109790	KLHL5	brown	-0.8352787
ENSG00000242522	KLHL6-AS1	grey	0.32610295
ENSG00000122550	KLHL7	blue	0.46032496
ENSG00000145332	KLHL8	turquoise	0.63735901
ENSG00000198642	KLHL9	red	0.53178445
ENSG00000167755	KLK6	yellow	0.75573009
ENSG00000164344	KLKB1	grey	-0.0871332
ENSG00000256667	KLRAP1	grey	0.13366128
ENSG00000139187	KLRG1	grey	0.16339265
ENSG00000118058	KMT2A	grey	-0.1571955
ENSG00000105663	KMT2B	grey	0.18317867
ENSG00000055609	KMT2C	grey	-0.1982719
ENSG00000167548	KMT2D	grey	-0.0510212
ENSG00000005483	KMT2E	green	0.82762609
ENSG00000162456	KNCN	blue	0.49500372
ENSG00000103550	KNOP1	yellow	0.41722455
ENSG00000270429	KNOP1P2	grey	0.41790639
ENSG00000232422	KNOP1P4	grey	0.31910645
ENSG00000128944	KNSTRN	grey	0.11659678
ENSG00000184445	KNTC1	grey	0.2340476
ENSG00000114030	KPNA1	turquoise	0.54824797

ENSG00000182481	KPNA2	grey	0.24008467
ENSG00000102753	KPNA3	turquoise	0.34560804
ENSG00000186432	KPNA4	red	0.5790181
ENSG00000196911	KPNA5	turquoise	0.63517767
ENSG00000025800	KPNA6	lightgreen	-0.6397703
ENSG00000108424	KPNB1	green	0.36641313
ENSG00000118162	KPTN	brown	0.40310769
ENSG00000133703	KRAS	lightcyan	0.65640562
ENSG00000133619	KRBA1	blue	0.42585226
ENSG00000184619	KRBA2	turquoise	0.38932609
ENSG00000240747	KRBOX1	grey	-0.0292077
ENSG00000147121	KRBOX4	grey	-0.4500021
ENSG00000172086	KRCC1	brown	-0.5605175
ENSG00000183762	KREMEN1	black	0.74160748
ENSG00000129347	KRI1	grey	-0.0180673
ENSG00000001631	KRIT1	grey	0.12517114
ENSG00000111615	KRR1	grey	-0.1160349
ENSG00000237672	KRR1P1	grey	0.3690665
ENSG00000186395	KRT10	grey	-0.1193011
ENSG00000249850	KRT18P31	grey	0.35022209
ENSG00000244515	KRT18P34	grey	0.33511955
ENSG00000229222	KRT18P4	grey	0.34183562
ENSG00000236670	KRT18P5	grey	0.28252496
ENSG00000215867	KRT18P57	grey	0.26463498
ENSG00000213424	KRT222	blue	0.59828029
ENSG00000170442	KRT86	grey	-0.1224445
ENSG00000258948	KRT8P1	yellow	0.43815634
ENSG00000229320	KRT8P12	turquoise	0.55451707
ENSG00000233579	KRT8P15	grey	0.2449419
ENSG00000214659	KRT8P26	grey	0.2153574
ENSG00000220378	KRT8P42	grey	0.0927682
ENSG00000248971	KRT8P46	grey	0.37356728
ENSG00000248568	KRT8P48	yellow	0.45562832
ENSG00000244411	KRTAP5-7	grey	0.01908488
ENSG00000233930	KRTAP5-AS1	grey	0.04877441
ENSG00000163463	KRTCAP2	grey	-0.1817582
ENSG00000141068	KSR1	grey	0.07314833
ENSG00000171435	KSR2	blue	0.82879377
ENSG00000198841	KTI12	grey	-0.1174257
ENSG00000126777	KTN1	lightgreen	0.73338455
ENSG00000186615	KTN1-AS1	grey	0.26160878
ENSG00000105700	KXD1	grey	0.17609493
ENSG00000174611	KY	grey	-0.0174103
ENSG00000198910	L1CAM	blue	0.81431794
ENSG00000087299	L2HGDH	grey	0.26415143
ENSG00000126790	L3HYPDH	grey	-0.1202499
ENSG00000185513	L3MBTL1	grey	-0.2322902
ENSG00000100395	L3MBTL2	brown	0.5171374

ENSG00000198945	L3MBTL3	grey	-0.0032864
ENSG00000154655	L3MBTL4	lightgreen	0.45495917
ENSG00000260022	LA16c-306A4.1	grey	-0.000737
ENSG00000260316	LA16c-306A4.2	grey	0.00536934
ENSG00000262482	LA16c-321D4.2	grey	0.24183805
ENSG00000261399	LA16c-329F2.1	turquoise	0.44609427
ENSG00000261207	LA16c-361A3.3	grey	0.33905894
ENSG00000260439	LA16c-366D3.1	grey	0.15298739
ENSG00000259840	LA16c-380A1.1	grey	0.12147459
ENSG00000179630	LACC1	green	0.74192288
ENSG00000135537	LACE1	grey	0.46635637
ENSG00000103642	LACTB	grey	0.09877589
ENSG00000147592	LACTB2	turquoise	0.59738265
ENSG00000196976	LAGE3	brown	0.62411049
ENSG00000167613	LAIR1	royalblue	0.83617713
ENSG00000101680	LAMA1	turquoise	0.4139587
ENSG00000196569	LAMA2	purple	-0.7509186
ENSG00000053747	LAMA3	grey	-0.0041826
ENSG00000130702	LAMA5	pink	0.63516152
ENSG00000091136	LAMB1	grey60	0.50378414
ENSG00000172037	LAMB2	turquoise	0.4500836
ENSG00000135862	LAMC1	pink	0.51843295
ENSG00000185896	LAMP1	turquoise	0.49423913
ENSG00000005893	LAMP2	yellow	0.73373551
ENSG00000125869	LAMP5	magenta	0.61956512
ENSG00000149357	LAMTOR1	grey	0.01976175
ENSG00000116586	LAMTOR2	brown	0.5781463
ENSG00000109270	LAMTOR3	yellow	-0.6751382
ENSG00000188186	LAMTOR4	brown	0.73468224
ENSG00000134248	LAMTOR5	yellow	-0.6112784
ENSG00000224699	LAMTOR5-AS1	grey	0.18778599
ENSG00000115365	LANCL1	floralwhite	0.66038565
ENSG00000132434	LANCL2	blue	0.5662062
ENSG00000147036	LANCL3	grey	0.16058549
ENSG00000002549	LAP3	pink	0.63524641
ENSG00000068697	LAPTM4A	turquoise	0.84501325
ENSG00000104341	LAPTM4B	grey	0.06115268
ENSG00000162511	LAPTM5	cyan	0.89195668
ENSG00000133424	LARGE	magenta	0.67931612
ENSG00000224973	LARGE-AS1	black	0.65992252
ENSG00000232081	LARGE-IT1	grey	0.33003817
ENSG00000155506	LARP1	purple	0.73524168
ENSG00000138709	LARP1B	yellow	-0.4158468
ENSG00000161813	LARP4	turquoise	0.66069057
ENSG00000107929	LARP4B	brown	0.43587281
ENSG00000261315	LARP4P	grey	0.35932861
ENSG00000166173	LARP6	red	-0.8387489
ENSG00000174720	LARP7	lightgreen	0.6777458

ENSG00000133706	LARS	salmon	0.58681586
ENSG00000011376	LARS2	grey	0.25891102
ENSG00000232455	LARS2-AS1	grey	0.30198258
ENSG00000001497	LAS1L	yellow	0.54033037
ENSG00000002834	LASP1	yellow	-0.3762574
ENSG00000086730	LAT2	royalblue	0.89428305
ENSG00000131023	LATS1	grey	0.2745404
ENSG00000150457	LATS2	blue	-0.75678
ENSG00000204381	LAYN	yellow	0.75989511
ENSG00000135338	LCA5	turquoise	0.58245824
ENSG00000157578	LCA5L	grey	0.04292639
ENSG00000213398	LCAT	grey	-0.0982764
ENSG00000172954	LCLAT1	yellow	-0.4386967
ENSG00000205629	LCMT1	brown	0.86642245
ENSG00000168806	LCMT2	turquoise	0.63437436
ENSG00000184925	LCN12	grey	0.01210594
ENSG00000214402	LCNL1	grey	0.10549682
ENSG00000196233	LCOR	brown	-0.6712879
ENSG00000178177	LCORL	grey	0.0151005
ENSG00000136167	LCP1	royalblue	0.91180876
ENSG00000043462	LCP2	royalblue	0.70968589
ENSG00000188501	LCTL	grey	0.01562459
ENSG00000198728	LDB1	grey	-0.2534246
ENSG00000169744	LDB2	blue	0.57076179
ENSG00000122367	LDB3	yellow	0.74450704
ENSG00000134333	LDHA	turquoise	-0.533024
ENSG00000250257	LDHAL6DP	yellow	0.39553243
ENSG00000270098	LDHAL6EP	grey	0.20092707
ENSG00000235674	LDHAP2	grey	0.34755144
ENSG00000214110	LDHAP4	grey	0.2705267
ENSG00000111716	LDHB	salmon	0.76599739
ENSG00000166816	LDHD	turquoise	0.48721558
ENSG00000130164	LDLR	pink	0.5991975
ENSG00000179241	LDLRAD3	grey60	-0.6236644
ENSG00000168675	LDLRAD4	lightgreen	0.56382615
ENSG00000267690	LDLRAD4-AS1	black	0.6479595
ENSG00000157978	LDLRAP1	yellow	0.70742944
ENSG00000182195	LDOC1	blue	0.67050201
ENSG00000188636	LDOC1L	turquoise	0.76428275
ENSG00000164406	LEAP2	yellow	0.48094366
ENSG00000136110	LECT1	green	-0.4652578
ENSG00000138795	LEF1	tan	0.66588766
ENSG00000161904	LEMD2	grey	-0.09962
ENSG00000174106	LEMD3	turquoise	-0.4175598
ENSG00000105617	LENG1	grey	0.01224962
ENSG00000167615	LENG8	darkred	-0.6868382
ENSG00000226696	LENG8-AS1	grey	0.07800833
ENSG00000166477	LEO1	grey	0.03475252

ENSG00000116678	LEPR	tan	0.48673696
ENSG00000117385	LEPRE1	turquoise	-0.4270177
ENSG00000090530	LEPREL1	cyan	0.54885684
ENSG00000225764	LEPREL1-AS1	grey	0.0074651
ENSG00000110811	LEPREL2	grey	0.07551236
ENSG00000141696	LEPREL4	grey	0.12234005
ENSG00000213625	LEPROT	turquoise	0.78338544
ENSG00000104660	LEPROTL1	turquoise	0.598628
ENSG00000168924	LETM1	grey	0.33532633
ENSG00000050426	LETMD1	grey	-0.2635877
ENSG00000100097	LGALS1	red	0.55453759
ENSG00000131981	LGALS3	grey	0.13958353
ENSG00000116977	LGALS8	grey	-0.1758594
ENSG00000168961	LGALS9	cyan	0.80310197
ENSG00000119862	LGALSL	grey	-0.1926165
ENSG00000108231	LGI1	grey	0.04328367
ENSG00000153012	LGI2	grey	-0.0822908
ENSG00000168481	LGI3	floralwhite	0.85926146
ENSG00000153902	LGI4	turquoise	0.51397592
ENSG00000100600	LGMN	cyan	0.47983912
ENSG00000205213	LGR4	turquoise	0.70811893
ENSG00000139292	LGR5	yellow	0.63122622
ENSG00000183722	LHFP	turquoise	0.67845397
ENSG00000145685	LHFPL2	royalblue	0.80373696
ENSG00000156959	LHFPL4	darkorange	0.68220733
ENSG00000107902	LHPP	darkred	-0.7138847
ENSG00000106689	LHX2	blue	-0.6695945
ENSG00000106852	LHX6	grey	-0.2951989
ENSG00000162624	LHX8	grey	0.00984995
ENSG00000121897	LIAS	grey	0.15100113
ENSG00000113594	LIFR	turquoise	0.81062088
ENSG00000105486	LIG1	grey	-0.4218945
ENSG00000005156	LIG3	turquoise	-0.4009394
ENSG00000174405	LIG4	turquoise	0.30011776
ENSG00000186818	LILRB4	cyan	0.86922988
ENSG00000050405	LIMA1	lightgreen	0.61548499
ENSG00000064042	LIMCH1	lightgreen	0.76714648
ENSG00000136490	LIMD2	grey	-0.092728
ENSG00000106683	LIMK1	grey	-0.0004406
ENSG00000182541	LIMK2	pink	0.85756078
ENSG00000169756	LIMS1	turquoise	0.72459066
ENSG00000072163	LIMS2	grey	-0.1052451
ENSG00000267796	LIN37	grey	0.25440682
ENSG00000205659	LIN52	grey	0.25029247
ENSG00000189308	LIN54	lightcyan	0.57053692
ENSG00000111052	LIN7A	purple	0.78965555
ENSG00000104863	LIN7B	blue	0.82913685
ENSG00000148943	LIN7C	brown	-0.6514865

ENSG00000183814	LIN9	grey	0.19768236
ENSG00000231721	LINC-PINT	grey	0.1619314
ENSG00000231459	LINC00032	red	-0.459452
ENSG00000178947	LINC00086	purple	0.5926209
ENSG00000196972	LINC00087	purple	0.55125578
ENSG00000225194	LINC00092	turquoise	0.59183625
ENSG00000235106	LINC00094	turquoise	0.66307931
ENSG00000236871	LINC00106	grey	0.09558149
ENSG00000225880	LINC00115	yellow	0.42757838
ENSG00000175701	LINC00116	brown	0.75767558
ENSG00000222041	LINC00152	blue	0.43433466
ENSG00000185433	LINC00158	grey	0.09176393
ENSG00000230323	LINC00159	grey	0.33534858
ENSG00000196668	LINC00173	grey	0.18363292
ENSG00000179406	LINC00174	grey	0.10860961
ENSG00000232224	LINC00202-1	grey	0.23760791
ENSG00000223768	LINC00205	grey	-0.0074195
ENSG00000224843	LINC00240	grey	0.46625908
ENSG00000229214	LINC00242	turquoise	-0.5200332
ENSG00000235823	LINC00263	yellow	0.58045466
ENSG00000149656	LINC00266-1	grey	0.13661235
ENSG00000231028	LINC00271	grey	0.00497185
ENSG00000248197	LINC00290	blue	0.52546082
ENSG00000236790	LINC00299	turquoise	0.28057939
ENSG00000224924	LINC00320	green	0.81417346
ENSG00000234912	LINC00338	grey	0.18875551
ENSG00000218510	LINC00339	grey	0.16278164
ENSG00000229645	LINC00341	grey	0.07462541
ENSG00000238230	LINC00391	grey	0.03868741
ENSG00000224243	LINC00403	purple	0.52747694
ENSG00000229520	LINC00404	grey	-0.1757617
ENSG00000237585	LINC00407	grey	0.13248629
ENSG00000224429	LINC00422	grey	0.08659873
ENSG00000238121	LINC00426	blue	0.38698655
ENSG00000236463	LINC00427	grey	0.11229068
ENSG00000203441	LINC00449	grey	0.38814238
ENSG00000233532	LINC00460	magenta	0.6561459
ENSG00000245526	LINC00461	turquoise	0.76853003
ENSG00000153363	LINC00467	grey	0.02736583
ENSG00000181798	LINC00471	grey	0.05915713
ENSG00000233237	LINC00472	purple	-0.7225428
ENSG00000175611	LINC00476	grey	-0.0483055
ENSG00000215386	LINC00478	turquoise	0.77354887
ENSG00000258169	LINC00485	yellow	0.39615369
ENSG00000232388	LINC00493	yellow	-0.643293
ENSG00000248397	LINC00498	grey	0.37888189
ENSG00000251372	LINC00499	grey	0.22479394
ENSG00000256193	LINC00507	grey	0.26277474

ENSG00000227036	LINC00511	turquoise	0.57969512
ENSG00000260583	LINC00515	grey	0.26847797
ENSG00000196273	LINC00523	grey	-0.2434128
ENSG00000264575	LINC00526	grey	0.16351641
ENSG00000232815	LINC00537	grey	0.0933923
ENSG00000231690	LINC00574	grey	0.1257596
ENSG00000203809	LINC00577	grey	0.28911853
ENSG00000259714	LINC00594	blue	0.37247258
ENSG00000227136	LINC00595	grey	0.06793034
ENSG00000253230	LINC00599	grey	0.01019386
ENSG00000235180	LINC00601	yellow	0.72347809
ENSG00000257585	LINC00609	green	0.3246993
ENSG00000196559	LINC00610	grey	0.21258687
ENSG00000236983	LINC00614	grey	0.28788141
ENSG00000250366	LINC00617	grey60	0.66620086
ENSG00000260941	LINC00622	magenta	0.42101392
ENSG00000223546	LINC00630	brown	0.28142583
ENSG00000203930	LINC00632	darkorange	0.62761416
ENSG00000205704	LINC00634	yellow	-0.4919155
ENSG00000259070	LINC00639	yellow	0.74908473
ENSG00000258441	LINC00641	darkgrey	0.45630587
ENSG00000186369	LINC00643	purple	0.63900974
ENSG00000259142	LINC00644	grey	-0.0390832
ENSG00000205181	LINC00654	grey	-0.081407
ENSG00000260032	LINC00657	grey	-0.1403482
ENSG00000261824	LINC00662	turquoise	-0.4197178
ENSG00000232677	LINC00665	grey	-0.0368956
ENSG00000263753	LINC00667	grey	0.21565745
ENSG00000237854	LINC00674	salmon	-0.6556787
ENSG00000215190	LINC00680	grey	0.02404319
ENSG00000226179	LINC00685	grey	0.04330571
ENSG00000228214	LINC00693	red	0.72400934
ENSG00000238266	LINC00707	grey	-0.1008703
ENSG00000229240	LINC00710	black	0.66014049
ENSG00000185904	LINC00839	grey	-0.2060672
ENSG00000178440	LINC00843	grey	0.15598429
ENSG00000237949	LINC00844	yellow	0.60006149
ENSG00000186842	LINC00846	grey	0.16004096
ENSG00000245060	LINC00847	grey	0.02387113
ENSG00000231177	LINC00852	grey	0.01107883
ENSG00000236383	LINC00854	grey	0.00012012
ENSG00000224914	LINC00863	grey	0.13448219
ENSG00000226067	LINC00869	grey	0.18969811
ENSG00000242759	LINC00882	grey	-0.1604795
ENSG00000243701	LINC00883	red	-0.72245
ENSG00000233058	LINC00884	grey	0.0792564
ENSG00000240875	LINC00886	grey	0.1344594
ENSG00000214145	LINC00887	grey	0.32066089

ENSG00000240024	LINC00888	yellow	-0.4835705
ENSG00000241769	LINC00893	red	-0.5641066
ENSG00000231711	LINC00899	yellow	0.37647096
ENSG00000246100	LINC00900	black	-0.6380294
ENSG00000242385	LINC00901	grey	-0.0807725
ENSG00000241397	LINC00903	grey	0.12746989
ENSG00000264247	LINC00909	yellow	0.55536809
ENSG00000251209	LINC00923	grey	0.23902295
ENSG00000259134	LINC00924	greenyellow	-0.5651688
ENSG00000255571	LINC00925	grey	-0.1098145
ENSG00000271614	LINC00936	purple	0.69280233
ENSG00000226091	LINC00937	blue	0.51536736
ENSG00000235884	LINC00941	blue	0.3027893
ENSG00000249628	LINC00942	grey	0.10548029
ENSG00000237489	LINC00959	grey	0.13548422
ENSG00000235387	LINC00961	tan	0.65180116
ENSG00000270074	LINC00965	grey	-0.198708
ENSG00000254377	LINC00966	darkorange	0.55376459
ENSG00000242086	LINC00969	grey	0.01553066
ENSG00000177133	LINC00982	turquoise	0.54228179
ENSG00000259330	LINC00984	grey	-0.0099853
ENSG00000237248	LINC00987	grey	-0.0208885
ENSG00000214194	LINC00998	red	0.63047637
ENSG00000225119	LINC00999	brown	-0.3566928
ENSG00000230724	LINC01001	grey	0.07973845
ENSG00000261455	LINC01003	turquoise	0.26097836
ENSG00000228393	LINC01004	grey	0.18282181
ENSG00000182648	LINC01006	grey	0.17877576
ENSG00000236700	LINC01010	yellow	0.59205343
ENSG00000244041	LINC01011	grey	-0.0774897
ENSG00000250056	LINC01018	turquoise	0.57916034
ENSG00000245146	LINC01024	grey	0.25123046
ENSG00000224081	LINC01057	pink	0.54405113
ENSG00000232065	LINC01063	grey	0.15942541
ENSG00000251442	LINC01094	grey	-0.2553232
ENSG00000235597	LINC01102	grey	-0.0483863
ENSG00000232044	LINC01105	floralwhite	0.76040715
ENSG00000175772	LINC01106	blue	0.53627046
ENSG00000234177	LINC01114	grey	0.05919223
ENSG00000163364	LINC01116	grey	0.16872655
ENSG00000233723	LINC01122	turquoise	-0.5686525
ENSG00000228486	LINC01125	grey	0.1498245
ENSG00000228794	LINC01128	blue	0.61260897
ENSG00000234807	LINC01135	green	0.65325197
ENSG00000236963	LINC01141	cyan	0.49588994
ENSG00000256124	LINC01152	black	-0.4597589
ENSG00000232593	LINC01155	cyan	-0.513586
ENSG00000233639	LINC01158	grey	0.03412211

ENSG00000229743	LINC01159	yellow	0.46733045
ENSG00000253807	LINC01170	yellow	0.61379018
ENSG00000257057	LINC01171	purple	0.70094372
ENSG00000169783	LINGO1	blue	0.615785
ENSG00000174482	LINGO2	purple	0.84425997
ENSG00000220008	LINGO3	magenta	0.57501499
ENSG00000140471	LINS	grey	0.02987626
ENSG00000107798	LIPA	yellow	0.86991816
ENSG00000079435	LIPE	floralwhite	0.81114705
ENSG00000144182	LIPT1	grey	0.05918808
ENSG00000229975	LIPT1P1	grey	0.3818472
ENSG00000189067	LITAF	yellow	0.55969937
ENSG00000145721	LIX1	turquoise	0.68637743
ENSG00000152022	LIX1L	turquoise	0.67596573
ENSG00000234345	LL0XNC01-116E7.	grey	0.21147068
ENSG00000231728	LL0XNC01-116E7.	grey	0.2261945
ENSG00000239407	LL0XNC01-237H1	magenta	-0.3967768
ENSG00000234050	LL0XNC01-237H1	grey	0.26119762
ENSG00000234405	LL0XNC01-250H1	grey	0.11538923
ENSG00000270012	LL0XNC01-7P3.1	blue	-0.5364146
ENSG00000229673	LL22NC01-116C6.	grey	0.39463873
ENSG00000236054	LL22NC03-104C7.	grey	0.4050284
ENSG00000234630	LL22NC03-2H8.4	grey	-0.1216033
ENSG00000224086	LL22NC03-86G7.1	grey	0.06793165
ENSG00000270041	LL22NC03-N27C7	grey	0.21037366
ENSG00000131899	LLGL1	yellow	0.58970046
ENSG00000139233	LLPH	turquoise	0.62392137
ENSG00000074695	LMAN1	turquoise	0.64299872
ENSG00000169223	LMAN2	pink	0.41255092
ENSG00000105983	LMBR1	grey	0.07653034
ENSG00000139636	LMBR1L	turquoise	-0.5540791
ENSG00000168216	LMBRD1	turquoise	0.51158879
ENSG00000164187	LMBRD2	red	0.50801676
ENSG00000071282	LMCD1	tan	0.53279579
ENSG00000227110	LMCD1-AS1	yellow	0.48214509
ENSG00000103227	LMF1	grey	-0.1147591
ENSG00000100258	LMF2	grey	0.22917063
ENSG00000185621	LMLN	blue	0.55267173
ENSG00000113368	LMNB1	grey	0.27284481
ENSG00000176619	LMNB2	grey	0.11406829
ENSG00000135363	LMO2	tan	0.67494704
ENSG00000048540	LMO3	grey	-0.482402
ENSG00000143013	LMO4	red	0.4963489
ENSG00000136153	LMO7	turquoise	-0.5819865
ENSG00000163431	LMOD1	grey	-0.0917095
ENSG00000164715	LMTK2	turquoise	-0.6933381
ENSG00000142235	LMTK3	turquoise	-0.6261939
ENSG00000206535	LNP1	turquoise	-0.6503722

ENSG00000113441	LNPEP	red	0.63416134
ENSG00000072201	LNX1	blue	0.69968333
ENSG00000250930	LNX1-AS1	grey	0.212038
ENSG00000139517	LNX2	grey	0.28214889
ENSG00000165714	LOH12CR1	yellow	0.51563245
ENSG00000205791	LOH12CR2	turquoise	0.66190308
ENSG00000196365	LONP1	brown	0.56019639
ENSG00000102910	LONP2	grey	0.17144078
ENSG00000154359	LONRF1	grey	0.35549403
ENSG00000170500	LONRF2	turquoise	-0.4604208
ENSG00000113083	LOX	grey	0.36798321
ENSG00000167210	LOXHD1	black	0.53562051
ENSG00000261801	LOXL1-AS1	grey	-0.1992932
ENSG00000134013	LOXL2	grey	0.1737622
ENSG00000115318	LOXL3	cyan	0.49054096
ENSG00000138131	LOXL4	yellow	0.37224429
ENSG00000198121	LPAR1	green	0.90735213
ENSG00000184574	LPAR5	cyan	0.86408765
ENSG00000139679	LPAR6	cyan	0.6523878
ENSG00000087253	LPCAT2	cyan	0.8376318
ENSG00000111684	LPCAT3	blue	-0.3303213
ENSG00000176454	LPCAT4	blue	0.87059793
ENSG00000123684	LPGAT1	red	-0.622042
ENSG00000072071	LPHN1	blue	0.35491397
ENSG00000117114	LPHN2	blue	0.48559943
ENSG00000150471	LPHN3	yellowgreen	0.62280191
ENSG00000134324	LPIN1	turquoise	0.55341786
ENSG00000101577	LPIN2	grey	-0.2384484
ENSG00000175445	LPL	magenta	0.85071609
ENSG00000145012	LPP	turquoise	0.71724334
ENSG00000129951	LPPR3	purple	0.54685228
ENSG00000117600	LPPR4	blue	0.79312429
ENSG00000117598	LPPR5	purple	0.77754115
ENSG00000110031	LPXN	grey	-0.2044446
ENSG00000136141	LRCH1	grey	0.31805657
ENSG00000130224	LRCH2	red	0.55930197
ENSG00000186001	LRCH3	grey	0.05809841
ENSG00000077454	LRCH4	grey	-0.2307125
ENSG00000128011	LRFN1	grey	0.00663355
ENSG00000126243	LRFN3	grey	0.23120042
ENSG00000173621	LRFN4	grey	0.2388051
ENSG00000165379	LRFN5	blue	0.68630414
ENSG00000121931	LRIF1	grey	0.06863946
ENSG00000144749	LRIG1	turquoise	0.76012949
ENSG00000198799	LRIG2	grey	-0.0235877
ENSG00000139263	LRIG3	yellow	0.65987353
ENSG00000118308	LRMP	cyan	0.69954113
ENSG00000123384	LRP1	turquoise	0.40563899

ENSG00000197324	LRP10	turquoise	0.78875677
ENSG00000120256	LRP11	grey	-0.4247337
ENSG00000168702	LRP1B	grey	-0.001608
ENSG00000081479	LRP2	yellow	0.62106142
ENSG00000109771	LRP2BP	grey	0.09287451
ENSG00000130881	LRP3	blue	0.47860309
ENSG00000134569	LRP4	turquoise	0.76291753
ENSG00000162337	LRP5	grey	0.28026742
ENSG00000070018	LRP6	brown	-0.6511814
ENSG00000157193	LRP8	darkmagenta	-0.5779102
ENSG00000163956	LRPAP1	grey60	0.76120629
ENSG00000138095	LRPPRC	brown	0.7539718
ENSG00000204950	LRRC10B	magenta	0.68584102
ENSG00000160959	LRRC14	grey	0.53798248
ENSG00000079691	LRRC16A	turquoise	0.70846105
ENSG00000186648	LRRC16B	grey	0.06079353
ENSG00000128606	LRRC17	royalblue	-0.4923803
ENSG00000172731	LRRC20	blue	0.5053438
ENSG00000148814	LRRC27	green	-0.6283401
ENSG00000168904	LRRC28	brown	0.39615525
ENSG00000125122	LRRC29	grey	0.19516085
ENSG00000137507	LRRC32	tan	0.85232199
ENSG00000171757	LRRC34	grey	0.09419982
ENSG00000159708	LRRC36	grey	0.08059153
ENSG00000176681	LRRC37A	grey	0.05156561
ENSG00000267023	LRRC37A16P	brown	-0.8256378
ENSG00000263142	LRRC37A17P	grey	0.16963172
ENSG00000238083	LRRC37A2	grey	-0.048501
ENSG00000176809	LRRC37A3	grey	-0.0463725
ENSG00000214425	LRRC37A4P	grey	0.08863327
ENSG00000230445	LRRC37A6P	tan	-0.4375686
ENSG00000185158	LRRC37B	grey	-0.0939348
ENSG00000250462	LRRC37BP1	grey	0.11499042
ENSG00000122477	LRRC39	red	-0.5748256
ENSG00000179796	LRRC3B	magenta	-0.6100753
ENSG00000128594	LRRC4	pink	-0.5400123
ENSG00000066557	LRRC40	grey	-0.0986077
ENSG00000132128	LRRC41	grey	0.31205633
ENSG00000116212	LRRC42	grey	0.04431799
ENSG00000158113	LRRC43	grey	0.02714262
ENSG00000130764	LRRC47	turquoise	-0.6388666
ENSG00000137821	LRRC49	blue	0.7239018
ENSG00000131409	LRRC4B	grey	0.07516234
ENSG00000183908	LRRC55	grey	-0.0226667
ENSG00000161328	LRRC56	grey	-0.329207
ENSG00000180979	LRRC57	grey	0.3714952
ENSG00000163428	LRRC58	turquoise	0.7104887
ENSG00000108829	LRRC59	brown	0.47900275

ENSG00000127399	LRRC61	grey	0.29094358
ENSG00000214954	LRRC69	grey	0.12888782
ENSG00000033122	LRRC7	darkorange	0.81841951
ENSG00000204052	LRRC73	blue	0.71985331
ENSG00000136802	LRRC8A	turquoise	0.74099602
ENSG00000197147	LRRC8B	red	-0.6397928
ENSG00000171488	LRRC8C	grey	-0.089109
ENSG00000171492	LRRC8D	yellow	0.79628567
ENSG00000133739	LRRCC1	green	0.64955393
ENSG00000124831	LRRFIP1	grey	-0.0411839
ENSG00000240429	LRRFIP1P1	yellow	0.43293584
ENSG00000093167	LRRFIP2	grey	0.15274572
ENSG00000154237	LRRK1	greenyellow	0.62505884
ENSG00000188906	LRRK2	grey	-0.0675287
ENSG00000175928	LRRN1	darkorange	0.64314665
ENSG00000170382	LRRN2	blue	0.7114661
ENSG00000173114	LRRN3	yellow	-0.6408972
ENSG00000125872	LRRN4	grey	0.06624355
ENSG00000162951	LRRTM1	blue	0.73227557
ENSG00000146006	LRRTM2	darkorange	0.72334182
ENSG00000198739	LRRTM3	blue	0.60190663
ENSG00000148356	LRSAM1	blue	0.66231536
ENSG00000144771	LRTM1	turquoise	-0.2523351
ENSG00000166159	LRTM2	black	0.63817223
ENSG00000184154	LRTOMT	brown	-0.5985919
ENSG00000161036	LRWD1	brown	0.59516208
ENSG00000185565	LSAMP	red	0.67255869
ENSG00000240922	LSAMP-AS1	turquoise	0.54514476
ENSG00000041802	LSG1	brown	0.5165204
ENSG00000175324	LSM1	red	0.56895826
ENSG00000181817	LSM10	grey	0.06779356
ENSG00000155858	LSM11	yellow	-0.558643
ENSG00000161654	LSM12	salmon	-0.7112426
ENSG00000257103	LSM14A	brown	-0.7796373
ENSG00000149657	LSM14B	grey	-0.0651501
ENSG00000204392	LSM2	grey	-0.0089041
ENSG00000170860	LSM3	turquoise	-0.5900136
ENSG00000250835	LSM3P4	grey	0.25221652
ENSG00000106355	LSM5	grey	-0.1272444
ENSG00000164167	LSM6	grey	-0.0206786
ENSG00000130332	LSM7	brown	0.64535188
ENSG00000183011	LSMD1	brown	0.74087568
ENSG00000181016	LSMEM1	grey	0.41345417
ENSG00000105699	LSR	tan	0.5602917
ENSG00000160285	LSS	black	0.56756294
ENSG00000204482	LST1	cyan	0.7756774
ENSG00000111144	LTA4H	salmon	0.69154177
ENSG00000213903	LTB4R	grey	0.15683052

ENSG00000049323	LTBP1	turquoise	0.473452
ENSG00000119681	LTBP2	grey	-0.0193925
ENSG00000168056	LTBP3	grey	-0.0945507
ENSG00000111321	LTBR	pink	0.64595982
ENSG00000198862	LTN1	grey	0.02410062
ENSG00000007392	LUC7L	brown	0.66781891
ENSG00000108848	LUC7L3	darkred	-0.7604845
ENSG00000171357	LURAP1	grey	-0.0060362
ENSG00000153714	LURAP1L	grey	0.02653282
ENSG00000169641	LUZP1	grey	0.03621209
ENSG00000079257	LXN	grey	0.00154838
ENSG00000160932	LY6E	turquoise	-0.4068117
ENSG00000204428	LY6G5C	grey	0.07477968
ENSG00000176956	LY6H	purple	0.65551908
ENSG00000112799	LY86	cyan	0.8534275
ENSG00000145220	LYAR	grey	0.00279262
ENSG00000144214	LYG1	grey	0.20562348
ENSG00000185674	LYG2	turquoise	-0.3668996
ENSG00000254087	LYN	royalblue	0.76685778
ENSG00000180155	LYNX1	grey	0.13536686
ENSG00000159871	LYPD5	blue	0.52670113
ENSG00000187123	LYPD6	darkmagenta	-0.7827433
ENSG00000266949	LYPD8	yellow	-0.3734786
ENSG00000120992	LYPLA1	turquoise	0.52838432
ENSG00000011009	LYPLA2	blue	0.63057135
ENSG00000143353	LYPLAL1	turquoise	0.51928372
ENSG00000102897	LYRM1	grey	0.13756583
ENSG00000214113	LYRM4	turquoise	-0.5234904
ENSG00000205707	LYRM5	lightcyan	0.56573386
ENSG00000186687	LYRM7	grey	0.07386846
ENSG00000232859	LYRM9	blue	0.68470204
ENSG00000163155	LYSMD1	turquoise	-0.3350025
ENSG00000140280	LYSMD2	yellow	-0.5259817
ENSG00000176018	LYSMD3	turquoise	0.64340613
ENSG00000183060	LYSMD4	grey	0.07811105
ENSG00000143669	LYST	turquoise	-0.4789629
ENSG00000133800	LYVE1	royalblue	0.59909219
ENSG00000090382	LYZ	royalblue	0.58698803
ENSG00000162441	LZIC	brown	-0.7925555
ENSG00000163818	LZTFL1	yellow	-0.4880949
ENSG00000099949	LZTR1	grey	0.14109907
ENSG00000061337	LZTS1	grey	-0.266773
ENSG00000253733	LZTS1-AS1	grey	-0.0181974
ENSG00000107816	LZTS2	grey	-0.119174
ENSG00000088899	LZTS3	magenta	0.63043632
ENSG00000003056	M6PR	brown	0.39323853
ENSG00000180660	MAB21L1	grey	0.25470388
ENSG00000173212	MAB21L3	green	0.52839353

ENSG00000127603	MACF1	blue	-0.5932839
ENSG00000133315	MACROD1	grey	0.00581139
ENSG00000172264	MACROD2	turquoise	-0.6841731
ENSG00000235914	MACROD2-AS1	grey	0.07024025
ENSG00000227927	MACROD2-IT1	grey	0.2675228
ENSG00000002822	MAD1L1	grey60	0.53696278
ENSG00000164109	MAD2L1	brown	0.32027839
ENSG00000124688	MAD2L1BP	grey	-0.0457948
ENSG00000116670	MAD2L2	grey	-0.0992409
ENSG00000110514	MADD	blue	0.77472532
ENSG00000090316	MAEA	green	-0.7945935
ENSG00000178573	MAF	cyan	0.71344077
ENSG00000179632	MAF1	blue	-0.4979896
ENSG00000204103	MAFB	royalblue	0.54349142
ENSG00000185022	MAFF	pink	0.82904195
ENSG00000197063	MAFG	grey60	-0.5119965
ENSG00000265688	MAFG-AS1	grey	0.04462035
ENSG00000198517	MAFK	brown	-0.5834835
ENSG00000105695	MAG	yellow	0.76550383
ENSG00000102316	MAGED2	black	-0.6864045
ENSG00000186675	MAGEE2	grey	-0.2019515
ENSG00000177383	MAGEF1	mediumpurp	0.77846502
ENSG00000187601	MAGEH1	green	-0.5806261
ENSG00000151276	MAGI1	pink	-0.5874504
ENSG00000240175	MAGI1-AS1	purple	-0.5183554
ENSG00000187391	MAGI2	turquoise	0.48259513
ENSG00000251276	MAGI2-AS1	blue	-0.551946
ENSG00000234456	MAGI2-AS3	turquoise	0.75640827
ENSG00000235751	MAGI2-IT1	grey	0.23878218
ENSG00000081026	MAGI3	red	0.61400267
ENSG00000162385	MAGOH	grey	0.15398819
ENSG00000264176	MAGOH2	grey	0.09275955
ENSG00000111196	MAGOHB	grey	0.00110834
ENSG00000102158	MAGT1	turquoise	0.59047595
ENSG00000111837	MAK	grey	0.15949611
ENSG00000198042	MAK16	grey	0.15043689
ENSG00000172005	MAL	yellow	0.84951696
ENSG00000147676	MAL2	turquoise	-0.4231864
ENSG00000251562	MALAT1	grey	-0.2285839
ENSG00000156928	MALSU1	salmon	0.71363529
ENSG00000172175	MALT1	grey	0.15194955
ENSG00000184384	MAML2	brown	-0.6489867
ENSG00000196782	MAML3	grey	-0.2994848
ENSG00000176909	MAMSTR	grey	0.04446973
ENSG00000111885	MAN1A1	purple	0.59632983
ENSG00000198162	MAN1A2	grey	-0.2696211
ENSG00000177239	MAN1B1	brown	0.74320498
ENSG00000117643	MAN1C1	black	-0.6215411

ENSG00000112893	MAN2A1	yellow	0.69838725
ENSG00000196547	MAN2A2	yellow	0.74892742
ENSG00000104774	MAN2B1	cyan	0.60453936
ENSG00000013288	MAN2B2	grey	-0.0779305
ENSG00000109323	MANBA	salmon	0.53425915
ENSG00000101363	MANBAL	magenta	-0.4235098
ENSG00000172469	MANEA	grey	0.02021707
ENSG00000261366	MANEA-AS1	grey	0.10433887
ENSG00000185090	MANEAL	blue	0.56438531
ENSG00000111261	MANSC1	grey	0.16043873
ENSG00000189221	MAOA	turquoise	0.75948791
ENSG00000069535	MAOB	grey	-0.2152883
ENSG00000212916	MAP10	green	-0.4624146
ENSG00000166963	MAP1A	blue	0.7206597
ENSG00000101460	MAP1LC3A	blue	0.56591121
ENSG00000140941	MAP1LC3B	grey	0.34558993
ENSG00000171471	MAP1LC3B2	grey	0.17842764
ENSG00000130479	MAP1S	blue	0.65231546
ENSG00000078018	MAP2	blue	0.81620982
ENSG00000126934	MAP2K2	grey	-0.0313035
ENSG00000034152	MAP2K3	grey	0.46268656
ENSG00000065559	MAP2K4	blue	0.79451511
ENSG00000137764	MAP2K5	brown	0.73535007
ENSG00000108984	MAP2K6	grey	0.05557194
ENSG00000076984	MAP2K7	green	0.71033312
ENSG00000095015	MAP3K1	grey	-0.1474895
ENSG00000130758	MAP3K10	turquoise	-0.4854846
ENSG00000173327	MAP3K11	yellowgreen	-0.7351555
ENSG00000139625	MAP3K12	turquoise	-0.4858518
ENSG00000073803	MAP3K13	blue	0.56320503
ENSG00000006062	MAP3K14	turquoise	-0.3294549
ENSG00000267278	MAP3K14-AS1	grey	0.08076438
ENSG00000169967	MAP3K2	turquoise	0.69611612
ENSG00000198909	MAP3K3	grey	0.31399124
ENSG00000085511	MAP3K4	grey	0.17045879
ENSG00000197442	MAP3K5	turquoise	0.53671475
ENSG00000142733	MAP3K6	pink	0.69252177
ENSG00000135341	MAP3K7	grey	-0.1499115
ENSG00000156265	MAP3K7CL	grey	-0.1209425
ENSG00000107968	MAP3K8	pink	0.57652199
ENSG00000006432	MAP3K9	blue	0.7611156
ENSG00000047849	MAP4	green	0.73986008
ENSG00000168067	MAP4K2	grey	0.0378471
ENSG00000011566	MAP4K3	grey	-0.2303942
ENSG00000071054	MAP4K4	green	0.92278068
ENSG00000012983	MAP4K5	yellow	0.80693837
ENSG00000171533	MAP6	yellow	-0.6045907
ENSG00000180834	MAP6D1	yellow	0.74700199

ENSG00000135525	MAP7	yellow	0.87785661
ENSG00000116871	MAP7D1	darkmagenta	0.6090965
ENSG00000184368	MAP7D2	blue	0.82321912
ENSG00000129680	MAP7D3	grey	-0.0160253
ENSG00000164114	MAP9	yellow	-0.4962223
ENSG00000100030	MAPK1	brown	0.63230348
ENSG00000109339	MAPK10	yellow	-0.6524714
ENSG00000185386	MAPK11	grey	0.06411761
ENSG00000188130	MAPK12	darkred	0.53721134
ENSG00000156711	MAPK13	grey	-0.0876721
ENSG00000168175	MAPK1IP1L	turquoise	0.59958145
ENSG00000102882	MAPK3	grey	-0.2417785
ENSG00000141639	MAPK4	pink	0.61014349
ENSG00000069956	MAPK6	lightgreen	0.61323983
ENSG00000237263	MAPK6PS3	grey	0.19857664
ENSG00000166484	MAPK7	grey	0.15714976
ENSG00000107643	MAPK8	grey	0.0168791
ENSG00000121653	MAPK8IP1	turquoise	-0.5789999
ENSG00000008735	MAPK8IP2	blue	0.73154668
ENSG00000138834	MAPK8IP3	blue	0.75522601
ENSG00000050748	MAPK9	blue	0.8214546
ENSG00000119487	MAPKAP1	grey	0.0203532
ENSG00000162889	MAPKAPK2	blue	-0.641474
ENSG00000114738	MAPKAPK3	grey	0.06316648
ENSG00000089022	MAPKAPK5	lightcyan	-0.6866436
ENSG00000234608	MAPKAPK5-AS1	brown	0.70983564
ENSG00000137802	MAPKBP1	turquoise	-0.4066831
ENSG00000101367	MAPRE1	blue	-0.8072567
ENSG00000253915	MAPRE1P1	grey	0.11871415
ENSG00000084764	MAPRE3	blue	0.84555588
ENSG00000186868	MAPT	grey	0.19294703
ENSG00000264589	MAPT-AS1	grey	0.21488421
ENSG00000155130	MARCKS	mediumpurp	0.75995564
ENSG00000175130	MARCKSL1	turquoise	0.58827277
ENSG00000213277	MARCKSL1P1	grey	0.38565128
ENSG00000116141	MARK1	purple	0.71481926
ENSG00000072518	MARK2	floralwhite	0.73055671
ENSG00000075413	MARK3	grey	0.06002518
ENSG00000007047	MARK4	grey60	0.59263499
ENSG00000247626	MARS2	blue	-0.4890211
ENSG00000155254	MARVELD1	turquoise	0.55974219
ENSG00000152939	MARVELD2	tan	0.67566972
ENSG00000127241	MASP1	turquoise	0.40254563
ENSG00000009724	MASP2	green	0.53905736
ENSG00000086015	MAST2	grey	0.11566615
ENSG00000099308	MAST3	darkmagenta	0.79205099
ENSG00000069020	MAST4	red	0.64731335
ENSG00000120539	MASTL	grey	0.19320294

ENSG00000168906	MAT2A	turquoise	0.43669529
ENSG00000038274	MAT2B	turquoise	0.54526235
ENSG00000007264	MATK	magenta	0.45114423
ENSG00000132561	MATN2	grey	-0.1449895
ENSG00000132031	MATN3	grey	0.37068389
ENSG00000015479	MATR3	turquoise	-0.4736864
ENSG00000129933	MAU2	lightcyan	-0.6273501
ENSG00000088888	MAVS	blue	-0.7605986
ENSG00000125952	MAX	greenyellow	0.58182321
ENSG00000103495	MAZ	grey	0.04932656
ENSG00000180611	MB21D2	grey	0.04690022
ENSG00000141644	MBD1	grey	-0.1480246
ENSG00000134046	MBD2	black	0.57301547
ENSG00000071655	MBD3	brown	0.54691317
ENSG00000129071	MBD4	lightgreen	0.81665305
ENSG00000204406	MBD5	lightgreen	-0.6180509
ENSG00000166987	MBD6	grey	-0.0745427
ENSG00000151332	MBIP	grey	-0.1824387
ENSG00000242600	MBL1P	grey	0.28359992
ENSG00000214309	MBLAC1	grey	-0.0066854
ENSG00000176055	MBLAC2	grey	-0.3208388
ENSG00000152601	MBNL1	grey	-0.1558174
ENSG00000139793	MBNL2	darkred	-0.7049272
ENSG00000076770	MBNL3	turquoise	0.3324551
ENSG00000172197	MBOAT1	grey	0.05459666
ENSG00000143797	MBOAT2	turquoise	0.85014459
ENSG00000197971	MBP	green	0.79766653
ENSG00000011258	MBTD1	lightgreen	0.69071045
ENSG00000140943	MBTPS1	brown	0.74417991
ENSG00000012174	MBTPS2	grey	0.32334764
ENSG00000166603	MC4R	grey	0.24939381
ENSG00000076706	MCAM	grey	0.11590263
ENSG00000100294	MCAT	blue	0.59985135
ENSG00000078070	MCCC1	grey	0.1059176
ENSG00000243368	MCCC1-AS1	brown	-0.4740923
ENSG00000131844	MCCC2	turquoise	0.56753751
ENSG00000235963	MCCD1P1	grey	0.25134271
ENSG00000224312	MCCD1P2	grey	0.25300509
ENSG00000124370	MCEE	grey	0.13995652
ENSG00000101977	MCF2	blue	0.85910171
ENSG00000235280	MCF2L-AS1	grey	0.13057244
ENSG00000180398	MCFD2	turquoise	0.63437086
ENSG00000152034	MCHR2	grey	-0.2547487
ENSG00000143384	MCL1	blue	-0.6874067
ENSG00000073111	MCM2	grey	0.09594077
ENSG00000112118	MCM3	grey	-0.0122736
ENSG00000160294	MCM3AP	turquoise	-0.5235817
ENSG00000215424	MCM3AP-AS1	grey	-0.1990771

ENSG00000104738	MCM4	grey	-0.0349363
ENSG00000100297	MCM5	grey	-0.1637062
ENSG00000076003	MCM6	brown	0.36633537
ENSG00000166508	MCM7	green	0.8132411
ENSG00000125885	MCM8	yellow	0.55504388
ENSG00000111877	MCM9	grey	0.23364551
ENSG00000197771	MCMBP	turquoise	0.42710446
ENSG00000178460	MCMDC2	turquoise	-0.3602808
ENSG00000090674	MCOLN1	turquoise	-0.3442638
ENSG00000147316	MCPH1	turquoise	-0.3661023
ENSG00000187778	MCRS1	turquoise	-0.4096098
ENSG00000175471	MCTP1	magenta	0.62510819
ENSG00000232119	MCTS1	brown	0.70947667
ENSG00000156026	MCU	grey	0.13594773
ENSG00000050393	MCUR1	grey	0.23574386
ENSG00000137337	MDC1	grey	-0.1267694
ENSG00000135272	MDFIC	turquoise	0.63339649
ENSG00000112139	MDGA1	grey	0.22004882
ENSG00000139915	MDGA2	purple	0.68082979
ENSG00000014641	MDH1	brown	0.86595305
ENSG00000138400	MDH1B	darkred	0.60188613
ENSG00000146701	MDH2	brown	0.76047965
ENSG00000110492	MDK	grey	-0.0587058
ENSG00000111554	MDM1	turquoise	0.46609586
ENSG00000135679	MDM2	brown	-0.515207
ENSG00000198625	MDM4	lightgreen	0.80720456
ENSG00000112159	MDN1	turquoise	-0.6752309
ENSG00000065833	ME1	turquoise	0.52797741
ENSG00000082212	ME2	brown	-0.5911794
ENSG00000151376	ME3	grey60	0.82373372
ENSG00000163875	MEAF6	blue	0.71454826
ENSG00000085276	MECOM	tan	0.8021265
ENSG00000169057	MECP2	blue	0.31644675
ENSG00000116353	MECR	blue	0.74021842
ENSG00000125686	MED1	grey	0.10621654
ENSG00000133398	MED10	grey	-0.2221217
ENSG00000161920	MED11	grey	0.17735712
ENSG00000184634	MED12	grey	0.17967838
ENSG00000108510	MED13	turquoise	0.72284053
ENSG00000123066	MED13L	black	0.53132273
ENSG00000180182	MED14	grey	-0.0477214
ENSG00000099917	MED15	turquoise	-0.4311103
ENSG00000175221	MED16	grey	0.2270808
ENSG00000042429	MED17	grey	0.29985246
ENSG00000156603	MED19	magenta	-0.5441436
ENSG00000124641	MED20	blue	-0.565179
ENSG00000152944	MED21	salmon	-0.6254798
ENSG00000148297	MED22	grey	0.21820288

ENSG00000112282	MED23	grey	0.13889381
ENSG00000008838	MED24	brown	0.75944067
ENSG00000104973	MED25	grey	-0.1722795
ENSG00000105085	MED26	grey	0.06024508
ENSG00000160563	MED27	brown	0.66439348
ENSG00000118579	MED28	darkgrey	0.53587099
ENSG00000227692	MED28P3	grey	0.39526438
ENSG00000259183	MED28P6	grey	0.29626675
ENSG00000063322	MED29	turquoise	0.42860631
ENSG00000164758	MED30	grey	0.06323181
ENSG00000108590	MED31	grey	-0.2171242
ENSG00000136146	MED4	turquoise	0.50608742
ENSG00000133997	MED6	grey	0.0980551
ENSG00000155868	MED7	turquoise	0.27017371
ENSG00000159479	MED8	grey	0.25687115
ENSG00000141026	MED9	grey	-0.0897152
ENSG00000068305	MEF2A	grey	-0.0696999
ENSG00000254901	MEF2BNB	blue	0.6662898
ENSG00000081189	MEF2C	yellow	-0.5415403
ENSG00000248309	MEF2C-AS1	grey	-0.0869622
ENSG00000116604	MEF2D	blue	0.65196174
ENSG00000214548	MEG3	grey	-0.3542322
ENSG00000258399	MEG8	turquoise	-0.3990469
ENSG00000105429	MEGF8	darkred	0.77952157
ENSG00000106780	MEGF9	brown	-0.6135645
ENSG00000143995	MEIS1	yellow	0.59647989
ENSG00000134138	MEIS2	grey	-0.4665891
ENSG00000105419	MEIS3	grey	-0.1880924
ENSG00000179277	MEIS3P1	turquoise	0.47411736
ENSG00000188013	MEIS3P2	turquoise	0.50388515
ENSG00000162959	MEMO1	grey	0.21724733
ENSG00000133895	MEN1	grey60	0.63826011
ENSG00000146834	MEPCE	grey	0.0247131
ENSG00000153208	MERTK	blue	-0.4785433
ENSG00000140406	MESDC1	grey	-0.105772
ENSG00000117899	MESDC2	mediumpurp	0.65293838
ENSG00000166823	MESP1	black	-0.563104
ENSG00000220884	MESTP1	purple	-0.5617005
ENSG00000164024	METAP1	grey60	0.53784921
ENSG00000172878	METAP1D	grey	-0.0739557
ENSG00000103260	METRN	turquoise	0.40189543
ENSG00000176845	METRNL	grey	0.3571845
ENSG00000037897	METTL1	blue	0.28026946
ENSG00000203791	METTL10	grey	-0.1395234
ENSG00000214756	METTL12	grey	0.11754345
ENSG00000010165	METTL13	brown	0.51111734
ENSG00000145388	METTL14	brown	-0.5882808
ENSG00000169519	METTL15	grey	0.14296174

ENSG00000127804	METTL16	grey	0.04447378
ENSG00000165792	METTL17	brown	0.51544879
ENSG00000171806	METTL18	turquoise	0.64718178
ENSG00000139160	METTL20	grey	0.18909728
ENSG00000144401	METTL21A	grey	0.00424663
ENSG00000229623	METTL21AP1	grey	0.31144845
ENSG00000123427	METTL21B	brown	-0.5301607
ENSG00000250878	METTL21EP	turquoise	-0.4622145
ENSG00000067365	METTL22	grey	0.0253109
ENSG00000181038	METTL23	grey	-0.0555125
ENSG00000053328	METTL24	grey	0.02065532
ENSG00000127720	METTL25	green	0.57024544
ENSG00000087995	METTL2A	blue	0.30358851
ENSG00000165819	METTL3	grey	0.00279888
ENSG00000101574	METTL4	grey	0.12192531
ENSG00000138382	METTL5	grey	-0.1936998
ENSG00000185432	METTL7A	turquoise	0.69417966
ENSG00000123600	METTL8	grey	-0.0176279
ENSG00000197006	METTL9	magenta	0.54121793
ENSG00000176624	MEX3C	turquoise	0.56817338
ENSG00000140259	MFAP1	darkgrey	0.59660412
ENSG00000232489	MFAP1P1	grey	0.19371345
ENSG00000037749	MFAP3	turquoise	0.47726836
ENSG00000166482	MFAP4	turquoise	0.51100777
ENSG00000168958	MFF	brown	0.64306886
ENSG00000140545	MFGE8	turquoise	0.50939534
ENSG00000147324	MFHAS1	grey	0.32492836
ENSG00000228109	MFI2-AS1	grey	0.07169827
ENSG00000171109	MFN1	grey	0.18803632
ENSG00000116688	MFN2	grey	0.23572809
ENSG00000118855	MFSD1	turquoise	0.70303234
ENSG00000109736	MFSD10	brown	0.43128046
ENSG00000092931	MFSD11	grey	0.25988031
ENSG00000168389	MFSD2A	tan	0.70274891
ENSG00000167700	MFSD3	brown	0.71871046
ENSG00000174514	MFSD4	purple	0.3895345
ENSG00000182544	MFSD5	grey	-0.0097576
ENSG00000135953	MFSD9	grey	0.44578565
ENSG00000174197	MGA	lightgreen	0.66423717
ENSG00000137463	MGARP	grey	0.05948748
ENSG00000131446	MGAT1	blue	-0.5195769
ENSG00000128268	MGAT3	brown	0.30897604
ENSG00000071073	MGAT4A	turquoise	-0.5104748
ENSG00000161013	MGAT4B	grey	0.12422544
ENSG00000182050	MGAT4C	red	0.74397851
ENSG00000152127	MGAT5	grey	-0.0893819
ENSG00000167889	MGAT5B	blue	0.29425033
ENSG00000198408	MGEA5	grey	-0.0502437

ENSG00000074416	MGLL	magenta	-0.5355099
ENSG00000125871	MGME1	turquoise	0.62441561
ENSG00000170430	MGMT	grey	-0.059123
ENSG00000111341	MGP	grey	-0.0563815
ENSG00000102858	MGRN1	grey	0.03297057
ENSG00000008394	MGST1	turquoise	0.67089999
ENSG00000085871	MGST2	tan	0.52573789
ENSG00000143198	MGST3	grey	-0.1600631
ENSG00000154305	MIA3	turquoise	-0.5716173
ENSG00000225783	MIAT	blue	0.25171878
ENSG00000101752	MIB1	brown	-0.5769161
ENSG00000197530	MIB2	brown	0.68407448
ENSG00000204520	MICA	grey	0.06059786
ENSG00000133816	MICAL2	grey	-0.0325458
ENSG00000243156	MICAL3	grey	-0.0518033
ENSG00000100139	MICALL1	yellow	0.46172831
ENSG00000164877	MICALL2	grey	-0.0469441
ENSG00000107745	MICU1	brown	0.75893569
ENSG00000165487	MICU2	grey	-0.1758054
ENSG00000155970	MICU3	blue	0.40722518
ENSG00000101871	MID1	turquoise	0.78805761
ENSG00000165175	MID1IP1	green	0.80324365
ENSG00000238123	MID1IP1-AS1	turquoise	0.68200725
ENSG00000080561	MID2	blue	0.63624564
ENSG00000167470	MIDN	pink	0.74441343
ENSG00000100335	MIEF1	turquoise	0.57995364
ENSG00000177427	MIEF2	grey	0.2137322
ENSG00000141741	MIEN1	grey	-0.1185407
ENSG00000198160	MIER1	brown	-0.6329314
ENSG00000105556	MIER2	grey	0.32755191
ENSG00000155545	MIER3	blue	-0.5186944
ENSG00000240972	MIF	black	-0.5635429
ENSG00000125457	MIF4GD	blue	-0.4634698
ENSG00000116691	MIIP	turquoise	-0.4030658
ENSG00000268654	MIMT1	turquoise	-0.4497696
ENSG00000170854	MINA	grey	0.02515981
ENSG00000141503	MINK1	grey	-0.2542555
ENSG00000173436	MINOS1	greenyellow	0.68323578
ENSG00000224237	MINOS1P3	grey	0.40651462
ENSG00000107789	MINPP1	grey	0.03203259
ENSG00000164654	MIOS	grey	0.00498351
ENSG00000027001	MIPEP	turquoise	-0.4147711
ENSG00000151338	MIPOL1	yellow	-0.4983615
ENSG00000265142	MIR1-2	grey	0.10553909
ENSG00000199024	MIR103A2	grey	0.07059182
ENSG00000221325	MIR1200	grey	0.57293348
ENSG00000221598	MIR1249	grey	0.50401587
ENSG00000221479	MIR1251	grey	0.51330837

ENSG00000221788	MIR1252	grey	0.36547176
ENSG00000221184	MIR1254-1	grey	0.33402712
ENSG00000221265	MIR1255A	grey	0.32490536
ENSG00000223770	MIR1255B1	grey	0.14497242
ENSG00000221808	MIR1256	grey	0.45230785
ENSG00000207971	MIR125B1	grey	0.45446229
ENSG00000221650	MIR1267	grey	0.3069522
ENSG00000265503	MIR1269B	grey	0.36707653
ENSG00000207654	MIR128-1	grey	0.42546529
ENSG00000221355	MIR1288	grey	0.34630031
ENSG00000221063	MIR1296	turquoise	0.46132511
ENSG00000239070	MIR1299	grey	0.17210655
ENSG00000221445	MIR1301	grey	0.20874397
ENSG00000221269	MIR1302-8	grey	0.25835084
ENSG00000227518	MIR1302-9	grey	0.0503855
ENSG00000221227	MIR1305	grey	0.34248873
ENSG00000251862	MIR1343	grey	0.39803017
ENSG00000225206	MIR137HG	purple	0.62299793
ENSG00000207954	MIR138-1	grey	0.39517255
ENSG00000207649	MIR138-2	grey	0.52772168
ENSG00000249669	MIR143HG	grey	-0.0904253
ENSG00000269936	MIR145	grey	-0.0957753
ENSG00000234883	MIR155HG	grey	0.2636692
ENSG00000229989	MIR181A1HG	yellow	0.68481812
ENSG00000207595	MIR181A2	grey	0.2540883
ENSG00000224020	MIR181A2HG	grey	0.21982977
ENSG00000207737	MIR181B2	grey	0.38439403
ENSG00000221476	MIR1827	grey	0.20080448
ENSG00000207695	MIR184	grey	0.38895417
ENSG00000222715	MIR1911	grey	0.1810672
ENSG00000222321	MIR1912	grey	0.16040628
ENSG00000222958	MIR1913	grey	0.4669284
ENSG00000207709	MIR197	grey	0.50425428
ENSG00000238728	MIR1972-1	grey	0.37462479
ENSG00000239118	MIR1972-2	grey	0.55966175
ENSG00000208024	MIR199A2	grey	0.40382851
ENSG00000238399	MIR2053	grey	0.25482633
ENSG00000215991	MIR208B	grey	0.38787872
ENSG00000238367	MIR2113	grey	0.57570135
ENSG00000207949	MIR214	grey	0.36937546
ENSG00000207955	MIR219-2	grey	0.33786542
ENSG00000252695	MIR2276	grey	0.37030929
ENSG00000252153	MIR2278	grey	0.59920514
ENSG00000186594	MIR22HG	grey	0.3577109
ENSG00000253008	MIR2355	grey	0.43516205
ENSG00000264292	MIR2467	grey	0.43378545
ENSG00000265164	MIR2681	grey	0.34625756
ENSG00000199075	MIR26A1	grey	0.33069222

ENSG00000266141	MIR2909	grey	0.5631452
ENSG00000226380	MIR29A	grey	0.31144049
ENSG00000215957	MIR300	grey	0.39630759
ENSG00000249532	MIR302B	grey	0.24115939
ENSG00000199094	MIR30C2	grey	0.53047525
ENSG00000264720	MIR3117	grey	0.3754971
ENSG00000265435	MIR3121	grey	0.27019629
ENSG00000265831	MIR3123	grey	0.32872149
ENSG00000264354	MIR3134	grey	0.3487701
ENSG00000265333	MIR3137	grey	0.43028805
ENSG00000265623	MIR3139	grey	0.4258908
ENSG00000265657	MIR3151	turquoise	0.52625054
ENSG00000264559	MIR3162	grey	0.2133054
ENSG00000266215	MIR3167	grey	0.20381587
ENSG00000264607	MIR3173	grey	0.19382015
ENSG00000265871	MIR3174	grey	0.3802367
ENSG00000267959	MIR3188	grey	0.12136445
ENSG00000265137	MIR3192	grey	0.30478009
ENSG00000264757	MIR3198-1	grey	0.35897913
ENSG00000207698	MIR32	grey	0.49863187
ENSG00000264661	MIR3200	grey	0.42897165
ENSG00000221406	MIR320B2	grey	0.52582077
ENSG00000211491	MIR320D1	grey	0.40214765
ENSG00000199090	MIR326	grey	0.40951169
ENSG00000266239	MIR3605	grey	0.55266395
ENSG00000266019	MIR3609	grey	0.33316896
ENSG00000266228	MIR3611	grey	0.37151665
ENSG00000265635	MIR3612	grey	0.26141871
ENSG00000266533	MIR3619	grey	0.43376117
ENSG00000199130	MIR365A	grey	0.54047941
ENSG00000265996	MIR3671	grey	0.03496255
ENSG00000263813	MIR3679	grey	0.28423235
ENSG00000265462	MIR3680-1	grey	0.57704775
ENSG00000265452	MIR3682	turquoise	0.60478571
ENSG00000265917	MIR3685	grey	0.37405958
ENSG00000212027	MIR374B	grey	0.35503803
ENSG00000264534	MIR378B	grey	0.30757078
ENSG00000264448	MIR378D2	grey	0.43549734
ENSG00000263831	MIR378E	grey	0.18138057
ENSG00000263526	MIR378G	turquoise	0.37536827
ENSG00000263361	MIR378H	grey	0.53711972
ENSG00000263463	MIR378I	grey	0.18930709
ENSG00000199088	MIR379	grey	0.6339448
ENSG00000199127	MIR383	grey	0.46253056
ENSG00000264329	MIR3911	grey	0.05243368
ENSG00000227671	MIR3916	brown	-0.6352019
ENSG00000263568	MIR3916	grey	0.26010898
ENSG00000266509	MIR3934	grey	0.39837771

ENSG00000265281	MIR3935	grey	0.42030365
ENSG00000263597	MIR3936	grey	0.22602748
ENSG00000264069	MIR3943	grey	0.51794174
ENSG00000265584	MIR3978	grey	0.41084038
ENSG00000202566	MIR421	grey	0.42699077
ENSG00000265321	MIR4263	grey	0.31615556
ENSG00000265215	MIR4269	grey	0.47000843
ENSG00000266180	MIR4282	grey	0.40416243
ENSG00000264763	MIR4295	grey	0.26551211
ENSG00000265140	MIR4301	grey	0.04848018
ENSG00000264171	MIR4305	grey	0.30400936
ENSG00000263512	MIR4311	grey	0.32000075
ENSG00000265195	MIR4312	grey	0.28956757
ENSG00000265957	MIR4319	grey	0.53304666
ENSG00000265744	MIR4427	grey	0.49967332
ENSG00000266262	MIR4428	grey	0.49373978
ENSG00000172965	MIR4435-1HG	turquoise	-0.371082
ENSG00000263445	MIR4450	grey	0.39064058
ENSG00000266421	MIR4451	grey	0.31284537
ENSG00000268471	MIR4453	grey	0.39881155
ENSG00000247516	MIR4458HG	grey	0.19893924
ENSG00000198868	MIR4461	grey	0.23953109
ENSG00000263790	MIR4473	grey	0.44960014
ENSG00000264941	MIR4474	grey	0.38922262
ENSG00000266017	MIR4477A	grey	0.42378042
ENSG00000266852	MIR4482-1	grey	0.3701764
ENSG00000265092	MIR4484	grey	0.50201781
ENSG00000265210	MIR4486	grey	-0.0110794
ENSG00000260083	MIR4519	grey	0.23613134
ENSG00000264468	MIR4520A	grey	0.38874149
ENSG00000263527	MIR4526	grey	0.40171557
ENSG00000264505	MIR4534	yellow	0.48051563
ENSG00000263712	MIR4639	grey	0.41373559
ENSG00000266245	MIR4644	grey	0.41837755
ENSG00000266038	MIR4659B	grey	0.26895294
ENSG00000264090	MIR4666B	grey	0.42738863
ENSG00000264377	MIR4671	grey	0.36678305
ENSG00000265201	MIR4677	grey	0.49380279
ENSG00000264309	MIR4694	grey	0.26582966
ENSG00000264919	MIR4697	grey	0.32490934
ENSG00000264482	MIR4705	grey	0.37423387
ENSG00000264109	MIR4712	grey	0.52586097
ENSG00000263857	MIR4729	grey	0.43205238
ENSG00000264961	MIR4730	grey	0.30806973
ENSG00000266392	MIR4740	grey	0.42351325
ENSG00000265879	MIR4748	grey	0.35402494
ENSG00000263973	MIR4760	grey	0.44025035
ENSG00000265694	MIR4774	grey	0.23345937

ENSG00000264279	MIR4786	grey	0.33379174
ENSG00000264658	MIR4798	grey	0.54345235
ENSG00000264099	MIR4803	grey	0.39175598
ENSG00000207609	MIR491	grey	0.38856048
ENSG00000267532	MIR497HG	grey	0.03388373
ENSG00000266383	MIR5002	grey	0.36853627
ENSG00000264796	MIR5009	grey	0.41632399
ENSG00000264966	MIR5094	grey	0.40226846
ENSG00000266270	MIR5096	grey	0.29365405
ENSG00000266146	MIR5190	grey	0.27720037
ENSG00000266097	MIR5192	grey	0.37399485
ENSG00000264653	MIR5194	grey	0.41189443
ENSG00000269842	MIR519A2	grey	0.22112416
ENSG00000207820	MIR545	grey	0.61498017
ENSG00000208032	MIR548A3	grey	0.12632375
ENSG00000265653	MIR548AK	grey	0.47747033
ENSG00000263741	MIR548AS	grey	0.39018723
ENSG00000221782	MIR548F2	grey	0.21653976
ENSG00000221737	MIR548I1	grey	0.10219388
ENSG00000221333	MIR548K	grey	0.28645726
ENSG00000221230	MIR548L	grey	0.26750431
ENSG00000265056	MIR548S	grey	0.46076265
ENSG00000221296	MIR548T	grey	0.58082482
ENSG00000212017	MIR548U	grey	0.36584058
ENSG00000265520	MIR548V	grey	0.30913311
ENSG00000207573	MIR550A2	grey	0.54580455
ENSG00000212024	MIR550A3	grey	0.15573005
ENSG00000207750	MIR553	grey	0.41285114
ENSG00000266542	MIR5572	grey	0.3530272
ENSG00000266570	MIR5579	grey	0.45947563
ENSG00000207653	MIR558	grey	0.4929449
ENSG00000263675	MIR5581	grey	0.54020674
ENSG00000263381	MIR5584	grey	0.42967093
ENSG00000264614	MIR5588	grey	0.37983427
ENSG00000207626	MIR562	grey	0.52446643
ENSG00000207815	MIR563	grey	0.42791302
ENSG00000207770	MIR568	turquoise	0.82433956
ENSG00000264477	MIR5682	grey	0.31329831
ENSG00000265135	MIR5687	grey	0.22372151
ENSG00000264084	MIR5688	grey	0.34787176
ENSG00000207963	MIR569	grey	0.28031506
ENSG00000265527	MIR5690	grey	0.42164572
ENSG00000207650	MIR570	grey	0.14611515
ENSG00000264536	MIR5706	grey	0.55255773
ENSG00000207642	MIR571	grey	0.31151735
ENSG00000207697	MIR573	grey	0.49739479
ENSG00000207746	MIR575	grey	0.30250662
ENSG00000207559	MIR578	grey	0.35349611

ENSG00000207756	MIR580	grey	0.48551017
ENSG00000207627	MIR581	grey	0.32261663
ENSG00000207741	MIR590	grey	0.33406982
ENSG00000207692	MIR592	grey	0.43247474
ENSG00000207637	MIR595	grey	0.53792605
ENSG00000207701	MIR597	grey	0.28620548
ENSG00000207600	MIR598	grey	0.48859729
ENSG00000236901	MIR600HG	grey	0.1264545
ENSG00000207991	MIR601	grey	0.32500987
ENSG00000207813	MIR605	grey	0.4630461
ENSG00000207583	MIR606	grey	0.27175632
ENSG00000208033	MIR609	grey	0.35132169
ENSG00000207622	MIR619	grey	0.31142073
ENSG00000207967	MIR620	grey	0.58061415
ENSG00000207652	MIR621	brown	-0.3604837
ENSG00000207719	MIR623	grey	0.36085168
ENSG00000207766	MIR626	grey	0.44440001
ENSG00000207561	MIR635	grey	0.55875182
ENSG00000207997	MIR644A	grey	0.37958277
ENSG00000208018	MIR645	grey	0.3628219
ENSG00000207802	MIR646	grey	0.02452236
ENSG00000207554	MIR647	grey	0.00570355
ENSG00000207696	MIR659	grey	0.41922303
ENSG00000176840	MIR7-3HG	yellow	-0.4361949
ENSG00000212100	MIR764	grey	0.36541328
ENSG00000211574	MIR770	grey	-0.1061484
ENSG00000216009	MIR874	grey	0.43089692
ENSG00000216192	MIR920	grey	0.42945204
ENSG00000260778	MIR940	grey	0.10818761
ENSG00000207807	MIR95	grey	0.41547558
ENSG00000207638	MIR99A	grey	0.58726068
ENSG00000199030	MIRLET7C	grey	0.47531841
ENSG00000199150	MIRLET7G	grey	0.50652782
ENSG00000167842	MIS12	turquoise	0.57978589
ENSG00000159055	MIS18A	grey	-0.2075613
ENSG00000129534	MIS18BP1	cyan	0.62818162
ENSG00000158411	MITD1	grey	-0.099083
ENSG00000187098	MITF	black	0.6142313
ENSG00000125863	MKKS	brown	0.69754438
ENSG00000196588	MKL1	grey	0.05490498
ENSG00000186260	MKL2	blue	0.48456095
ENSG00000128585	MKLN1	turquoise	0.74327399
ENSG00000079277	MKNK1	royalblue	0.60115688
ENSG00000099875	MKNK2	blue	-0.6214458
ENSG00000133606	MKRN1	grey	-0.3251371
ENSG00000075975	MKRN2	grey	0.15389038
ENSG00000179455	MKRN3	yellow	0.54560418
ENSG00000011143	MKS1	grey	-0.0532347

ENSG00000100427	MLC1	turquoise	0.59156468
ENSG00000110917	MLEC	turquoise	0.52297235
ENSG00000178053	MLF1	grey	-0.3644891
ENSG00000089693	MLF2	grey	-0.242602
ENSG00000076242	MLH1	grey	-0.0354536
ENSG00000119684	MLH3	lightgreen	0.62629989
ENSG00000143674	MLK4	blue	0.58171192
ENSG00000130382	MLLT1	grey	0.10403225
ENSG00000078403	MLLT10	grey	-0.0666314
ENSG00000238151	MLLT10P1	grey	0.3664271
ENSG00000213190	MLLT11	purple	0.81793867
ENSG00000171843	MLLT3	black	0.46403497
ENSG00000130396	MLLT4	grey	0.26679352
ENSG00000108292	MLLT6	green	0.59038501
ENSG00000167965	MLST8	turquoise	-0.5683957
ENSG00000091436	MLTK	turquoise	0.66939891
ENSG00000108788	MLX	turquoise	0.44522
ENSG00000175727	MLXIP	grey	-0.1269784
ENSG00000009950	MLXIPL	turquoise	-0.4699248
ENSG00000103150	MLYCD	grey	-0.0750351
ENSG00000139428	MMAB	grey	-0.0071504
ENSG00000132763	MMACHC	grey	0.2753414
ENSG00000168288	MMADHC	turquoise	0.59656982
ENSG00000108960	MMD	grey	-0.432286
ENSG00000136297	MMD2	yellowgreen	0.78961845
ENSG00000196549	MME	grey60	0.69896393
ENSG00000169446	MMGT1	darkmagenta	-0.5710641
ENSG00000102996	MMP15	grey	0.38128234
ENSG00000198598	MMP17	turquoise	-0.6147261
ENSG00000087245	MMP2	grey	-0.1094847
ENSG00000125966	MMP24	green	0.35976069
ENSG00000126005	MMP24-AS1	blue	0.45195937
ENSG00000129270	MMP28	turquoise	0.51139194
ENSG00000173269	MMRN2	tan	0.80868613
ENSG00000155229	MMS19	brown	0.65239844
ENSG00000146263	MMS22L	grey	0.16102864
ENSG00000169184	MN1	magenta	0.67910056
ENSG00000020426	MNAT1	turquoise	-0.471017
ENSG00000163563	MNDA	cyan	0.7330608
ENSG00000070444	MNT	black	-0.6045838
ENSG00000165943	MOAP1	brown	0.39840259
ENSG00000114978	MOB1A	turquoise	0.65676376
ENSG00000173542	MOB1B	cyan	0.39500614
ENSG00000182208	MOB2	grey	0.07938747
ENSG00000172081	MOB3A	blue	-0.6763605
ENSG00000120162	MOB3B	red	-0.5644043
ENSG00000142961	MOB3C	turquoise	0.4817558
ENSG00000115540	MOB4	red	0.67809136

ENSG00000168314	MOBP	green	0.79136823
ENSG00000124217	MOCS3	grey	0.10554381
ENSG00000204655	MOG	yellow	0.88256233
ENSG00000115275	MOGS	grey	-0.0504723
ENSG00000080823	MOK	yellow	-0.7056445
ENSG00000103111	MON1B	brown	-0.8575884
ENSG00000061987	MON2	grey	0.11887201
ENSG00000133422	MORC2	grey	0.18402275
ENSG00000235989	MORC2-AS1	grey	0.11322231
ENSG00000159256	MORC3	grey	0.20585045
ENSG00000185787	MORF4L1	black	-0.5988503
ENSG00000218283	MORF4L1P1	grey	0.37024774
ENSG00000231857	MORF4L1P5	grey	0.29838267
ENSG00000123562	MORF4L2	brown	0.65836263
ENSG00000188010	MORN2	darkred	0.57886615
ENSG00000171160	MORN4	yellow	-0.6333443
ENSG00000101928	MOSPD1	grey	0.15585443
ENSG00000130150	MOSPD2	yellow	0.47957661
ENSG00000106330	MOSPD3	grey	0.22023596
ENSG00000155363	MOV10	grey	-0.2078263
ENSG00000079931	MOXD1	turquoise	0.4442685
ENSG00000060762	MPC1	turquoise	0.57723732
ENSG00000143158	MPC2	grey	-0.2069153
ENSG00000129255	MPDU1	grey	0.16434903
ENSG00000107186	MPDZ	turquoise	0.35611108
ENSG00000197629	MPEG1	cyan	0.75465718
ENSG00000103152	MPG	yellow	-0.5189701
ENSG00000124383	MPHOSPH10	grey	0.08662471
ENSG00000135698	MPHOSPH6	grey	-0.1356527
ENSG00000196199	MPHOSPH8	red	-0.5860563
ENSG00000051825	MPHOSPH9	turquoise	-0.5692963
ENSG00000178802	MPI	yellow	-0.5317409
ENSG00000117400	MPL	grey	0.04233963
ENSG00000168303	MPLKIP	grey	0.09334959
ENSG00000008382	MPND	grey	0.03935559
ENSG00000130830	MPP1	turquoise	-0.7262727
ENSG00000108852	MPP2	grey	-0.0739125
ENSG00000161647	MPP3	grey	-0.0110983
ENSG00000072415	MPP5	grey	0.06887295
ENSG00000105926	MPP6	grey	-0.3097754
ENSG00000154889	MPPE1	grey	0.21745342
ENSG00000066382	MPPED2	darkmagenta	0.69424305
ENSG00000133030	MPRIP	black	0.56290858
ENSG00000225442	MPRIP-AS1	grey	0.26034893
ENSG00000128309	MPST	black	0.69392986
ENSG00000115204	MPV17	lightcyan	0.63180089
ENSG00000156968	MPV17L	salmon	-0.5873255
ENSG00000254858	MPV17L2	grey	0.22283763

ENSG00000158887	MPZ	grey	-0.0650699
ENSG00000197965	MPZL1	turquoise	0.50590816
ENSG00000160588	MPZL3	turquoise	-0.2685409
ENSG00000153029	MR1	grey	0.17500302
ENSG00000135324	MRAP2	grey	0.04839512
ENSG00000011028	MRC2	grey	-0.0602768
ENSG00000020922	MRE11A	grey	0.0548831
ENSG00000118242	MREG	grey	0.2041335
ENSG00000179010	MRFAP1	grey	-0.1932428
ENSG00000178988	MRFAP1L1	lightgreen	0.54603026
ENSG00000101189	MRGBP	turquoise	0.43678258
ENSG00000037757	MRI1	grey	-0.1368977
ENSG00000129282	MRM1	grey	0.12726808
ENSG00000134042	MRO	turquoise	0.80880753
ENSG00000179832	MROH1	grey	0.18350517
ENSG00000101353	MROH8	grey	-0.1944804
ENSG00000173141	MRP63	grey	-0.0955968
ENSG00000169288	MRPL1	grey	-0.0427962
ENSG00000159111	MRPL10	green	-0.4803182
ENSG00000174547	MRPL11	green	-0.612118
ENSG00000262814	MRPL12	brown	0.57290933
ENSG00000172172	MRPL13	grey	0.02302757
ENSG00000180992	MRPL14	salmon	0.60440008
ENSG00000137547	MRPL15	salmon	0.54734237
ENSG00000166902	MRPL16	grey	-0.0776982
ENSG00000158042	MRPL17	grey	-0.0008842
ENSG00000115364	MRPL19	brown	-0.8252674
ENSG00000112651	MRPL2	green	-0.6941321
ENSG00000242485	MRPL20	grey	-0.1466933
ENSG00000197345	MRPL21	blue	0.46818658
ENSG00000082515	MRPL22	lightgreen	-0.6377072
ENSG00000214026	MRPL23	brown	0.51622321
ENSG00000143314	MRPL24	brown	0.63263525
ENSG00000108826	MRPL27	brown	0.59522028
ENSG00000086504	MRPL28	brown	0.7713979
ENSG00000114686	MRPL3	brown	0.7005941
ENSG00000106591	MRPL32	brown	0.6531627
ENSG00000243147	MRPL33	salmon	0.75373745
ENSG00000130312	MRPL34	yellow	-0.4977154
ENSG00000132313	MRPL35	grey	0.18684635
ENSG00000232075	MRPL35P2	grey	0.34783357
ENSG00000226253	MRPL35P3	grey	0.2190356
ENSG00000171421	MRPL36	red	0.58536265
ENSG00000116221	MRPL37	green	-0.4271949
ENSG00000154719	MRPL39	turquoise	0.47418979
ENSG00000105364	MRPL4	brown	0.49485834
ENSG00000185608	MRPL40	green	-0.5725774
ENSG00000182154	MRPL41	yellow	-0.5015138

ENSG00000198015	MRPL42	salmon	-0.7035243
ENSG00000055950	MRPL43	grey	-0.1893966
ENSG00000135900	MRPL44	grey	0.10746116
ENSG00000174100	MRPL45	salmon	0.64839143
ENSG00000259494	MRPL46	salmon	0.59794914
ENSG00000136522	MRPL47	lightgreen	-0.6422564
ENSG00000175581	MRPL48	turquoise	-0.7310107
ENSG00000149792	MRPL49	grey	0.10067824
ENSG00000136897	MRPL50	grey	0.17470307
ENSG00000111639	MRPL51	brown	0.43953791
ENSG00000232777	MRPL51P2	grey	0.34716737
ENSG00000172590	MRPL52	brown	0.70611583
ENSG00000204822	MRPL53	grey	0.28420583
ENSG00000235299	MRPL53P1	grey	0.43223296
ENSG00000183617	MRPL54	greenyellow	0.53396551
ENSG00000162910	MRPL55	brown	0.79377299
ENSG00000143436	MRPL9	brown	0.65478301
ENSG00000048544	MRPS10	salmon	-0.5352926
ENSG00000181991	MRPS11	blue	0.63296541
ENSG00000128626	MRPS12	yellow	-0.5651594
ENSG00000120333	MRPS14	brown	-0.5479423
ENSG00000116898	MRPS15	blue	0.613286
ENSG00000182180	MRPS16	grey	0.06253933
ENSG00000239789	MRPS17	grey	0.02802101
ENSG00000096080	MRPS18A	grey	0.15723179
ENSG00000204568	MRPS18B	grey	-0.05618
ENSG00000163319	MRPS18C	grey	-0.0979267
ENSG00000122140	MRPS2	grey	0.29179586
ENSG00000187145	MRPS21	grey	-0.0719848
ENSG00000175110	MRPS22	grey	0.1917863
ENSG00000181610	MRPS23	brown	0.62354931
ENSG00000062582	MRPS24	grey	0.21008588
ENSG00000125901	MRPS26	red	0.44556006
ENSG00000113048	MRPS27	brown	0.63909934
ENSG00000147586	MRPS28	mediumpurp	0.62209144
ENSG00000112996	MRPS30	grey	-0.0195058
ENSG00000102738	MRPS31	grey	0.07585659
ENSG00000090263	MRPS33	green	-0.4779531
ENSG00000074071	MRPS34	yellow	-0.5281707
ENSG00000061794	MRPS35	grey	0.07291967
ENSG00000134056	MRPS36	red	0.60902902
ENSG00000255231	MRPS36P4	grey	0.31072892
ENSG00000144029	MRPS5	turquoise	-0.5611084
ENSG00000125445	MRPS7	green	-0.5773887
ENSG00000135972	MRPS9	brown	0.57109244
ENSG00000148187	MRRF	grey	0.20696283
ENSG00000124532	MRS2	green	-0.3927615
ENSG00000053372	MRTO4	brown	0.61001979

ENSG00000072952	MRVI1	turquoise	0.68187437
ENSG00000177112	MRVI1-AS1	grey	0.22892778
ENSG00000110079	MS4A4A	royalblue	0.69981772
ENSG00000166927	MS4A7	cyan	0.76544225
ENSG00000188981	MSANTD1	brown	0.61028375
ENSG00000120458	MSANTD2	green	0.55504196
ENSG00000066697	MSANTD3	brown	-0.396041
ENSG00000170903	MSANTD4	darkgrey	0.67873455
ENSG00000095002	MSH2	lightcyan	-0.7441144
ENSG00000113318	MSH3	grey	0.03148208
ENSG00000057468	MSH4	grey	0.41323116
ENSG00000116062	MSH6	blue	-0.561285
ENSG00000135097	MSI1	turquoise	0.67905667
ENSG00000153944	MSI2	turquoise	0.83448905
ENSG00000174579	MSL2	brown	-0.7593625
ENSG00000005302	MSL3	brown	0.45639307
ENSG00000224287	MSL3P1	grey	0.15504314
ENSG00000052802	MSMO1	grey	-0.1519689
ENSG00000147065	MSN	turquoise	0.51816146
ENSG00000175806	MSRA	turquoise	-0.470874
ENSG00000148450	MSRB2	blue	0.54929982
ENSG00000174099	MSRB3	turquoise	0.75089045
ENSG00000166343	MSS51	grey	0.14728446
ENSG00000173531	MST1	grey	-0.2623958
ENSG00000134602	MST4	turquoise	-0.592972
ENSG00000125459	MSTO1	grey	0.02104162
ENSG00000163132	MSX1	brown	-0.3932163
ENSG00000228253	MT-ATP8	grey	-0.0764647
ENSG00000198712	MT-CO2	grey	0.13061056
ENSG00000198727	MT-CYB	grey	0.0883853
ENSG00000198888	MT-ND1	grey	-0.0056243
ENSG00000198763	MT-ND2	grey	-0.0825735
ENSG00000212907	MT-ND4L	grey	0.15751147
ENSG00000198695	MT-ND6	grey	-0.0151458
ENSG00000205362	MT1A	pink	0.7878837
ENSG00000205361	MT1DP	grey	0.1056636
ENSG00000169715	MT1E	turquoise	0.56650832
ENSG00000198417	MT1F	grey	-0.168022
ENSG00000125144	MT1G	grey	0.00062818
ENSG00000205358	MT1H	grey	-0.184406
ENSG00000260549	MT1L	pink	0.61389002
ENSG00000205364	MT1M	pink	0.78887572
ENSG00000187193	MT1X	pink	0.80302256
ENSG00000125148	MT2A	pink	0.72435372
ENSG00000087250	MT3	brown	0.40842239
ENSG00000182979	MTA1	grey	-0.2674861
ENSG00000149480	MTA2	blue	-0.6611676
ENSG00000057935	MTA3	turquoise	-0.6234211

ENSG00000099810	MTAP	grey	0.17410138
ENSG00000248527	MTATP6P1	grey	0.0979033
ENSG00000229604	MTATP8P2	green	0.32096513
ENSG00000172167	MTBP	grey	0.33129213
ENSG00000137409	MTCH1	brown	0.81886854
ENSG00000147649	MTDH	lightcyan	-0.6949704
ENSG00000156469	MTERFD1	grey	0.02938083
ENSG00000122085	MTERFD2	grey	0.01989067
ENSG00000120832	MTERFD3	grey	0.01728036
ENSG00000188786	MTF1	brown	-0.4991934
ENSG00000143033	MTF2	grey	-0.1157079
ENSG00000103707	MTFMT	blue	0.48987787
ENSG00000066855	MTFR1	yellow	0.60407832
ENSG00000117640	MTFR1L	grey	0.09603075
ENSG00000148824	MTG1	grey	-0.2750328
ENSG00000101181	MTG2	grey	0.2083233
ENSG00000100714	MTHFD1	green	-0.6887838
ENSG00000120254	MTHFD1L	brown	0.47934886
ENSG00000065911	MTHFD2	pink	0.78624142
ENSG00000163738	MTHFD2L	grey	0.1767405
ENSG00000217169	MTHFD2P2	grey	0.09012379
ENSG00000177000	MTHFR	grey	0.03341604
ENSG00000136371	MTHFS	yellow	-0.452595
ENSG00000103248	MTHFSD	grey	0.23495899
ENSG00000085760	MTIF2	grey	0.08725086
ENSG00000122033	MTIF3	grey	0.08707531
ENSG00000132749	MTL5	blue	0.62833745
ENSG00000171100	MTM1	turquoise	0.80593174
ENSG00000063601	MTMR1	red	0.46037055
ENSG00000014914	MTMR11	grey	-0.0411729
ENSG00000150712	MTMR12	magenta	0.50574494
ENSG00000163719	MTMR14	grey	0.12514424
ENSG00000087053	MTMR2	yellow	0.69907813
ENSG00000100330	MTMR3	turquoise	0.58644415
ENSG00000139505	MTMR6	brown	0.51628426
ENSG00000003987	MTMR7	blue	0.70528471
ENSG00000104643	MTMR9	red	0.62472915
ENSG00000220785	MTMR9LP	grey	-0.2556036
ENSG00000251449	MTND1P19	turquoise	0.40516103
ENSG00000235940	MTND1P21	grey	0.30654357
ENSG00000225972	MTND1P23	brown	-0.2814406
ENSG00000232282	MTND1P32	grey	0.2483041
ENSG00000229954	MTND2P2	grey	0.08099594
ENSG00000232631	MTND2P23	grey	0.02814916
ENSG00000225630	MTND2P28	grey	0.11645125
ENSG00000247627	MTND4P12	grey	-0.1430837
ENSG00000241787	MTND4P16	grey	0.14599809
ENSG00000248923	MTND5P11	grey	-0.0755285

ENSG00000251544	MTND5P12	grey	0.1195278
ENSG00000243658	MTND5P16	grey	-0.0630416
ENSG00000254132	MTND6P3	grey	0.11560463
ENSG00000249119	MTND6P4	grey	0.05376716
ENSG00000227730	MTND6P5	grey	0.36694051
ENSG00000198793	MTOR	darkgrey	-0.4995106
ENSG00000225602	MTOR-AS1	grey	0.33296077
ENSG00000107951	MTPAP	grey	0.23022357
ENSG00000105887	MTPN	grey	-0.2272386
ENSG00000116984	MTR	darkgrey	0.47840016
ENSG00000120662	MTRF1	grey	0.20380099
ENSG00000256618	MTRNR2L1	grey	0.07349765
ENSG00000256045	MTRNR2L10	grey	0.16444694
ENSG00000270188	MTRNR2L11	grey	0.17899014
ENSG00000269028	MTRNR2L12	grey	-0.0155611
ENSG00000271043	MTRNR2L2	grey	0.04206735
ENSG00000256222	MTRNR2L3	grey	0.33275952
ENSG00000232196	MTRNR2L4	grey	0.3430931
ENSG00000249860	MTRNR2L5	grey	0.20107225
ENSG00000270672	MTRNR2L6	grey	0.25585084
ENSG00000255823	MTRNR2L8	grey	-0.036604
ENSG00000255633	MTRNR2L9	grey	0.182802
ENSG00000124275	MTRR	grey	0.00632969
ENSG00000170873	MTSS1	floralwhite	0.56622298
ENSG00000132613	MTSS1L	lightcyan	0.72824582
ENSG00000180354	MTURN	green	0.86965182
ENSG00000129422	MTUS1	pink	0.52888249
ENSG00000132938	MTUS2	grey60	0.68104881
ENSG00000179141	MTUS2-AS1	grey	0.27133788
ENSG00000173171	MTX1	greenyellow	-0.3864241
ENSG00000236675	MTX1P1	grey	0.20817331
ENSG00000128654	MTX2	brown	0.66496159
ENSG00000177034	MTX3	grey	-0.2367216
ENSG00000185499	MUC1	grey	-0.0601492
ENSG00000169550	MUC15	magenta	0.71082827
ENSG00000176945	MUC20	grey	-0.0223218
ENSG00000184956	MUC6	grey	-0.2702755
ENSG00000090432	MUL1	grey	0.41847505
ENSG00000160953	MUM1	brown	0.77772533
ENSG00000237906	MUPP	grey	0.33751621
ENSG00000170681	MURC	red	-0.6914929
ENSG00000172732	MUS81	grey	-0.2453047
ENSG00000030304	MUSK	yellow	0.42122578
ENSG00000146085	MUT	turquoise	0.52839843
ENSG00000132781	MUTYH	grey	0.03761022
ENSG00000141971	MVB12A	grey	0.10224589
ENSG00000196814	MVB12B	grey	-0.090124
ENSG00000167508	MVD	brown	0.73062078

ENSG00000110921	MVK	turquoise	-0.3989526
ENSG00000157601	MX1	grey	0.08084068
ENSG00000123933	MXD4	grey	0.15720665
ENSG00000119950	MXI1	brown	-0.8497498
ENSG00000182534	MXRA7	red	-0.4907505
ENSG00000162576	MXRA8	turquoise	0.64316514
ENSG00000179820	MYADM	grey	-0.1127001
ENSG00000185105	MYADML2	brown	0.38336754
ENSG00000118513	MYB	turquoise	-0.5863444
ENSG00000132382	MYBBP1A	grey60	0.54124099
ENSG00000185697	MYBL1	grey	0.39803248
ENSG00000196091	MYBPC1	turquoise	0.3538812
ENSG00000133055	MYBPH	pink	0.55833986
ENSG00000214114	MYCBP	grey	0.08381903
ENSG00000005810	MYCBP2	blue	0.81091034
ENSG00000236051	MYCBP2-AS1	grey	0.36122525
ENSG00000229521	MYCBP2-AS2	grey	0.31372594
ENSG00000116990	MYCL	grey	-0.0476197
ENSG00000134323	MYCN	grey	-0.0425434
ENSG00000120279	MYCT1	tan	0.81887541
ENSG00000172936	MYD88	pink	0.73530243
ENSG00000104177	MYEF2	bisque4	0.67555398
ENSG00000172428	MYEOV2	grey	-0.1573771
ENSG00000133026	MYH10	grey60	0.89828962
ENSG00000133392	MYH11	grey	-0.0307901
ENSG00000105357	MYH14	green	0.55943242
ENSG00000144821	MYH15	grey	0.0707579
ENSG00000109063	MYH3	grey	0.15332211
ENSG00000092054	MYH7	grey	-0.2299589
ENSG00000078814	MYH7B	turquoise	-0.4590438
ENSG00000100345	MYH9	tan	0.6235451
ENSG00000101608	MYL12A	pink	0.73886383
ENSG00000118680	MYL12B	yellow	-0.3478819
ENSG00000160808	MYL3	grey	0.0694209
ENSG00000215375	MYL5	brown	0.40464191
ENSG00000092841	MYL6	yellow	-0.3875988
ENSG00000196465	MYL6B	brown	0.6511611
ENSG00000235808	MYL6P2	grey	0.39750388
ENSG00000240677	MYL6P4	grey	0.25972977
ENSG00000101335	MYL9	brown	-0.5324703
ENSG00000007944	MYLIP	yellow	0.62731438
ENSG00000065534	MYLK	turquoise	0.75072733
ENSG00000239523	MYLK-AS1	grey	0.15402441
ENSG00000250174	MYLK-AS2	grey	0.25484159
ENSG00000145949	MYLK4	grey	0.18784993
ENSG00000085274	MYNN	grey	0.14510819
ENSG00000145555	MYO10	turquoise	0.6322118
ENSG00000196535	MYO18A	darkgrey	0.58857105

ENSG00000141140	MYO19	grey	0.21408287
ENSG00000128641	MYO1B	yellow	-0.5054861
ENSG00000197879	MYO1C	tan	0.70604271
ENSG00000176658	MYO1D	yellow	0.79497592
ENSG00000157483	MYO1E	yellow	0.82885371
ENSG00000142347	MYO1F	royalblue	0.74892618
ENSG00000095777	MYO3A	turquoise	-0.5735139
ENSG00000197535	MYO5A	blue	0.85348469
ENSG00000167306	MYO5B	grey	0.20167914
ENSG00000128833	MYO5C	grey	-0.199646
ENSG00000196586	MYO6	yellow	0.61067507
ENSG00000066933	MYO9A	brown	0.6273696
ENSG00000099331	MYO9B	yellow	0.76239261
ENSG00000138119	MYOF	tan	0.56245369
ENSG00000101605	MYOM1	grey	0.01627056
ENSG00000036448	MYOM2	grey	-0.1701447
ENSG00000120729	MYOT	yellow	0.73667803
ENSG00000177791	MYOZ1	turquoise	0.48372936
ENSG00000164591	MYOZ3	turquoise	-0.3383724
ENSG00000176182	MYPOP	grey	0.03694626
ENSG00000124920	MYRF	yellow	0.87728035
ENSG00000166268	MYRFL	grey	0.00435695
ENSG00000170011	MYRIP	purple	0.51575898
ENSG00000162601	MYSM1	red	-0.5453158
ENSG00000186487	MYT1L	grey60	0.77176245
ENSG00000225619	MYT1L-AS1	grey	0.29974201
ENSG00000099326	MZF1	grey	-0.3318337
ENSG00000204899	MZT1	grey	-0.4199504
ENSG00000173272	MZT2A	brown	0.7322041
ENSG00000152082	MZT2B	brown	0.71326904
ENSG00000102921	N4BP1	turquoise	-0.4217801
ENSG00000078177	N4BP2	blue	0.32159273
ENSG00000139597	N4BP2L1	greenyellow	0.52427151
ENSG00000244754	N4BP2L2	lightgreen	0.69143232
ENSG00000150456	N6AMT2	turquoise	0.55258467
ENSG00000145416	NA	darkred	-0.6491573
ENSG00000186205	NA	red	0.63394378
ENSG00000180096	NA	grey	0.04741823
ENSG00000099785	NA	grey	0.19640922
ENSG00000117791	NA	turquoise	0.64651567
ENSG00000168385	NA	turquoise	0.57216585
ENSG00000173926	NA	turquoise	0.52757566
ENSG00000100167	NA	purple	0.77636309
ENSG00000144583	NA	blue	0.69200273
ENSG00000108387	NA	yellow	0.77612703
ENSG00000198060	NA	turquoise	0.64508365
ENSG00000184702	NA	darkorange	0.75273284
ENSG00000145495	NA	grey	-0.0417751

ENSG00000125354	NA	yellow	-0.5661536
ENSG00000136536	NA	yellow	0.54143043
ENSG00000122545	NA	yellow	0.7758412
ENSG00000165406	NA	turquoise	0.46674799
ENSG00000164402	NA	yellow	0.68851067
ENSG00000139266	NA	green	-0.5142024
ENSG00000184640	NA	pink	0.56484936
ENSG00000186522	NA	green	0.69993008
ENSG00000183654	NA	blue	0.43480432
ENSG00000138758	NA	floralwhite	-0.5519224
ENSG00000183291	NA	turquoise	0.55749718
ENSG00000249194	NA	blue	0.52178721
ENSG00000253151	NA	blue	0.52726497
ENSG00000260356	NA	blue	-0.3147525
ENSG00000270348	NA	blue	0.74062897
ENSG00000270907	NA	blue	0.74782315
ENSG00000271341	NA	blue	0.64037125
ENSG00000112664	NA	brown	-0.7659019
ENSG00000224345	NA	brown	-0.3555105
ENSG00000261311	NA	brown	0.33451356
ENSG00000271051	NA	brown	0.58812385
ENSG00000150086	NA	darkorange	0.81303772
ENSG00000252701	NA	darkorange	0.5399541
ENSG00000259787	NA	darkorange	0.50779137
ENSG00000121083	NA	grey	-0.2387877
ENSG00000185068	NA	grey	-0.0150096
ENSG00000204804	NA	grey	0.11992784
ENSG00000205873	NA	grey	-0.1328365
ENSG00000207790	NA	grey	0.51313036
ENSG00000207966	NA	grey	0.32554314
ENSG00000213283	NA	grey	0.23210514
ENSG00000214117	NA	grey	0.12533842
ENSG00000223365	NA	grey	0.39807064
ENSG00000223444	NA	grey	0.1360717
ENSG00000223998	NA	grey	0.26874138
ENSG00000224119	NA	grey	0.36588306
ENSG00000225504	NA	grey	-0.1921855
ENSG00000225810	NA	grey	0.2295802
ENSG00000225994	NA	grey	0.30894183
ENSG00000226585	NA	grey	0.24310185
ENSG00000227280	NA	grey	0.12959888
ENSG00000228885	NA	grey	0.19723598
ENSG00000229033	NA	grey	0.37488809
ENSG00000229819	NA	grey	0.25351788
ENSG00000230028	NA	grey	0.24848546
ENSG00000230206	NA	grey	0.40147599
ENSG00000232584	NA	grey	0.36688219
ENSG00000232689	NA	grey	0.29929814

ENSG00000233202	NA	grey	0.2239725
ENSG00000233899	NA	grey	0.26043736
ENSG00000234133	NA	grey	0.42081983
ENSG00000234554	NA	grey	0.14968766
ENSG00000235167	NA	grey	0.18287959
ENSG00000236455	NA	grey	0.19859772
ENSG00000237030	NA	grey	0.42253352
ENSG00000238206	NA	grey	0.42726183
ENSG00000238274	NA	grey	0.19873784
ENSG00000238527	NA	grey	0.24634984
ENSG00000239235	NA	grey	0.31696959
ENSG00000239769	NA	grey	0.24288555
ENSG00000240368	NA	grey	0.20823631
ENSG00000240529	NA	grey	0.23469241
ENSG00000241238	NA	grey	0.32267644
ENSG00000242518	NA	grey	0.31066676
ENSG00000242629	NA	grey	0.2296986
ENSG00000242870	NA	grey	0.40725564
ENSG00000243077	NA	grey	0.21240581
ENSG00000243628	NA	grey	0.21972495
ENSG00000243754	NA	grey	0.29642328
ENSG00000243788	NA	grey	0.2748517
ENSG00000244054	NA	grey	0.13142408
ENSG00000244117	NA	grey	-0.0627296
ENSG00000244439	NA	grey	0.20797332
ENSG00000245562	NA	grey	0.27023706
ENSG00000248044	NA	grey	0.21696855
ENSG00000248201	NA	grey	0.18097953
ENSG00000248666	NA	grey	0.29844159
ENSG00000248865	NA	grey	0.19849191
ENSG00000249044	NA	grey	0.08761971
ENSG00000249179	NA	grey	0.09357033
ENSG00000249719	NA	grey	0.44503394
ENSG00000249823	NA	grey	0.35720556
ENSG00000249864	NA	grey	0.257111
ENSG00000250784	NA	grey	0.30355437
ENSG00000251267	NA	grey	0.37213417
ENSG00000251540	NA	grey	0.29616555
ENSG00000253718	NA	grey	0.31756554
ENSG00000255896	NA	grey	0.23246383
ENSG00000255906	NA	grey	0.2369511
ENSG00000256872	NA	grey	-0.1237509
ENSG00000257196	NA	grey	0.1914066
ENSG00000257424	NA	grey	0.02925301
ENSG00000259484	NA	grey	0.2578697
ENSG00000259812	NA	grey	0.20049565
ENSG00000260214	NA	grey	0.12939735
ENSG00000260512	NA	grey	-0.1042882

ENSG00000260556	NA	grey	0.23235654
ENSG00000260561	NA	grey	0.37423576
ENSG00000261050	NA	grey	0.06116261
ENSG00000261062	NA	grey	0.15313267
ENSG00000261179	NA	grey	-0.0821705
ENSG00000262359	NA	grey	0.05160628
ENSG00000263544	NA	grey	-0.0691938
ENSG00000265302	NA	grey	0.31032989
ENSG00000266471	NA	grey	0.49681293
ENSG00000266878	NA	grey	0.05199245
ENSG00000266937	NA	grey	0.04865831
ENSG00000267236	NA	grey	-0.0717178
ENSG00000267331	NA	grey	0.33315361
ENSG00000269932	NA	grey	0.08218109
ENSG00000269991	NA	grey	0.18322543
ENSG00000271463	NA	grey	0.25370019
ENSG00000260124	NA	grey60	0.66773713
ENSG00000251582	NA	magenta	-0.3945612
ENSG00000248603	NA	pink	0.48133009
ENSG00000240742	NA	purple	0.58451179
ENSG00000251160	NA	red	-0.662363
ENSG00000270155	NA	red	0.56258457
ENSG00000270567	NA	salmon	0.5647459
ENSG00000127688	NA	turquoise	0.72963796
ENSG00000136805	NA	turquoise	-0.3606673
ENSG00000233364	NA	turquoise	0.5462632
ENSG00000250970	NA	turquoise	0.57719371
ENSG00000254918	NA	turquoise	-0.3886945
ENSG00000259599	NA	turquoise	0.40704914
ENSG00000260800	NA	turquoise	0.67890575
ENSG00000261658	NA	turquoise	-0.4177329
ENSG00000263899	NA	turquoise	0.34807804
ENSG00000270674	NA	turquoise	0.47501776
ENSG00000234198	NA	yellow	0.69278819
ENSG00000243473	NA	yellow	0.31429939
ENSG00000250763	NA	yellow	0.4970534
ENSG00000267111	NA	yellow	0.38882228
ENSG00000267548	NA	yellow	0.88876865
ENSG00000102030	NAA10	brown	0.67459792
ENSG00000164134	NAA15	grey	0.07118339
ENSG00000172766	NAA16	grey	0.1503232
ENSG00000173418	NAA20	grey	-0.0587127
ENSG00000111300	NAA25	grey	-0.1803474
ENSG00000139977	NAA30	grey	-0.1356206
ENSG00000135040	NAA35	grey	-0.1485333
ENSG00000128534	NAA38	grey	0.1684677
ENSG00000110583	NAA40	grey	-0.3953143
ENSG00000121579	NAA50	grey	-0.0838914

ENSG00000122390	NAA60	lightgreen	-0.5691399
ENSG00000138744	NAAA	yellow	-0.5195321
ENSG00000077616	NAALAD2	grey	-0.1212174
ENSG00000177694	NAALADL2	grey	0.07223632
ENSG00000138386	NAB1	lightgreen	0.60779191
ENSG00000166886	NAB2	grey	-0.0317881
ENSG00000173559	NABP1	royalblue	0.59765389
ENSG00000139579	NABP2	grey	-0.0666146
ENSG00000196531	NACA	greenyellow	0.90961592
ENSG00000136274	NACAD	floralwhite	0.80075446
ENSG00000160877	NACC1	grey	0.25605654
ENSG00000148411	NACC2	turquoise	0.90630918
ENSG00000008130	NADK	purple	-0.6105812
ENSG00000152620	NADK2	turquoise	0.79286157
ENSG00000245711	NADK2-AS1	grey	0.19861807
ENSG00000172890	NADSYN1	yellowgreen	-0.6736013
ENSG00000145414	NAF1	brown	0.65722876
ENSG00000198951	NAGA	grey	-0.1518977
ENSG00000124357	NAGK	green	-0.4741876
ENSG00000108784	NAGLU	blue	-0.3914529
ENSG00000103174	NAGPA	turquoise	-0.4911362
ENSG00000171169	NAIF1	turquoise	0.36621062
ENSG00000249437	NAIP	grey	0.08378325
ENSG00000102452	NALCN	blue	0.65761699
ENSG00000105835	NAMPT	pink	0.80579965
ENSG00000111704	NANOG	grey	-0.2575259
ENSG00000237065	NANOGP4	grey	0.13151854
ENSG00000231697	NANOGP5	grey	0.11467949
ENSG00000255192	NANOGP8	grey	-0.1723678
ENSG00000188613	NANOS1	red	0.53220411
ENSG00000170191	NANP	grey	0.21996752
ENSG00000095380	NANS	brown	0.63088096
ENSG00000187109	NAP1L1	lightgreen	0.73313449
ENSG00000186462	NAP1L2	purple	0.54089872
ENSG00000186310	NAP1L3	blue	0.7187615
ENSG00000205531	NAP1L4	turquoise	-0.4579076
ENSG00000204118	NAP1L6	grey	0.52348008
ENSG00000105402	NAPA	blue	0.77507102
ENSG00000125814	NAPB	blue	0.80943619
ENSG00000161048	NAPEPLD	grey	0.21323279
ENSG00000134265	NAPG	yellow	-0.5629864
ENSG00000147813	NAPRT1	grey	-0.1367697
ENSG00000131400	NAPSA	grey	0.10883925
ENSG00000131401	NAPSB	royalblue	0.61352141
ENSG00000141562	NARF	lightcyan	-0.7434613
ENSG00000103245	NARFL	brown	0.43093217
ENSG00000128915	NARG2	blue	-0.3260919
ENSG00000134440	NARS	grey	0.15832294

ENSG00000137513	NARS2	grey	0.26758678
ENSG00000132780	NASP	darkred	-0.5874767
ENSG00000135372	NAT10	brown	0.48270057
ENSG00000090971	NAT14	blue	0.38086018
ENSG00000243477	NAT6	grey	0.09147801
ENSG00000144035	NAT8	grey	0.03981172
ENSG00000185818	NAT8L	turquoise	0.52778921
ENSG00000109065	NAT9	grey	0.14166946
ENSG00000134369	NAV1	grey	-0.0097667
ENSG00000166833	NAV2	grey	-0.0014076
ENSG00000254542	NAV2-AS3	grey	0.246021
ENSG00000254622	NAV2-AS4	grey	0.27976072
ENSG00000255043	NAV2-AS5	grey	-0.0861798
ENSG00000255270	NAV2-IT1	grey	-0.0632092
ENSG00000067798	NAV3	purple	0.69207332
ENSG00000151779	NBAS	brown	0.74221694
ENSG00000172915	NBEA	turquoise	-0.7053136
ENSG00000144426	NBEAL1	grey	-0.0203704
ENSG00000160796	NBEAL2	grey	0.18883368
ENSG00000158747	NBL1	turquoise	0.41537873
ENSG00000104320	NBN	grey	-0.2690069
ENSG00000219481	NBPF1	grey	-0.2260923
ENSG00000163386	NBPF10	red	0.58925379
ENSG00000186275	NBPF12	red	-0.3936053
ENSG00000122497	NBPF14	grey	0.16883347
ENSG00000243452	NBPF15	turquoise	-0.4019255
ENSG00000203836	NBPF24	grey	0.24697535
ENSG00000142794	NBPF3	grey	-0.0983478
ENSG00000162825	NBPF8	grey	0.15775753
ENSG00000168614	NBPF9	grey	-0.1738173
ENSG00000188554	NBR1	blue	-0.3974799
ENSG00000198496	NBR2	grey	0.10198587
ENSG00000149294	NCAM1	brown	0.39113232
ENSG00000227487	NCAM1-AS1	grey	0.10354589
ENSG00000154654	NCAM2	blue	-0.4239017
ENSG00000130287	NCAN	grey	0.04542035
ENSG00000010292	NCAPD2	grey	0.26085032
ENSG00000151503	NCAPD3	turquoise	-0.3781767
ENSG00000146918	NCAPG2	black	0.53833975
ENSG00000025770	NCAPH2	brown	0.50363073
ENSG00000136937	NCBP1	grey	0.11418012
ENSG00000114503	NCBP2	grey	-0.1084794
ENSG00000270170	NCBP2-AS2	yellow	-0.5427794
ENSG00000020129	NCDN	blue	0.65770184
ENSG00000144959	NCEH1	grey60	0.77825206
ENSG00000116701	NCF2	royalblue	0.74855254
ENSG00000100365	NCF4	royalblue	0.72894724
ENSG00000158092	NCK1	grey	0.00836252

ENSG00000071051	NCK2	pink	0.57830613
ENSG00000061676	NCKAP1	green	-0.7292717
ENSG00000123338	NCKAP1L	cyan	0.91970244
ENSG00000176771	NCKAP5	yellow	0.78916869
ENSG00000232474	NCKAP5-IT1	grey	0.20295151
ENSG00000167566	NCKAP5L	turquoise	-0.3288821
ENSG00000213672	NCKIPSD	blue	0.42815233
ENSG00000115053	NCL	darkgrey	0.6812044
ENSG00000125912	NCLN	grey	0.11256243
ENSG00000213212	NCLP1	turquoise	-0.3533158
ENSG00000084676	NCOA1	yellow	-0.3641256
ENSG00000140396	NCOA2	black	0.65235553
ENSG00000124151	NCOA3	brown	-0.5531479
ENSG00000138293	NCOA4	brown	-0.6847449
ENSG00000124160	NCOA5	grey	-0.1443726
ENSG00000198646	NCOA6	grey	0.02654006
ENSG00000111912	NCOA7	turquoise	-0.5262447
ENSG00000232131	NCOA7-AS1	grey	0.25194167
ENSG00000141027	NCOR1	grey	-0.1069891
ENSG00000196498	NCOR2	grey	0.00169372
ENSG00000188211	NCR3LG1	blue	0.58620649
ENSG00000107130	NCS1	purple	0.81705829
ENSG00000162736	NCSTN	grey	0.25947991
ENSG00000058804	NDC1	grey	0.03855331
ENSG00000072864	NDE1	yellow	0.84811637
ENSG00000166579	NDEL1	turquoise	-0.681889
ENSG00000131507	NDFIP1	yellow	-0.5181868
ENSG00000102471	NDFIP2	magenta	0.60625774
ENSG00000182636	NDN	purple	0.7475894
ENSG00000185115	NDNL2	grey	0.02590846
ENSG00000188566	NDOR1	yellow	0.39546384
ENSG00000104419	NDRG1	yellow	0.74059233
ENSG00000165795	NDRG2	turquoise	0.66408308
ENSG00000101079	NDRG3	blue	0.81507784
ENSG00000070614	NDST1	grey	0.28289449
ENSG00000166507	NDST2	grey	0.17813023
ENSG00000164100	NDST3	greenyellow	0.53851807
ENSG00000125356	NDUFA1	brown	0.63922776
ENSG00000130414	NDUFA10	turquoise	-0.667278
ENSG00000174886	NDUFA11	brown	0.79908993
ENSG00000184752	NDUFA12	yellow	-0.5452996
ENSG00000186010	NDUFA13	lightcyan	-0.7046357
ENSG00000131495	NDUFA2	brown	0.72164055
ENSG00000170906	NDUFA3	blue	0.58683335
ENSG00000267590	NDUFA3P1	grey	0.03362323
ENSG00000259262	NDUFA3P4	grey	0.22634451
ENSG00000189043	NDUFA4	brown	0.84135778
ENSG00000185633	NDUFA4L2	green	0.57146973

ENSG00000253454	NDUFA5P2	grey	0.10588895
ENSG00000184983	NDUFA6	yellow	-0.5426579
ENSG00000267855	NDUFA7	brown	0.55745786
ENSG00000119421	NDUFA8	yellow	-0.6496157
ENSG00000139180	NDUFA9	green	-0.5553282
ENSG00000237406	NDUFA9P1	grey	0.27169642
ENSG00000004779	NDUFAB1	brown	0.69980306
ENSG00000137806	NDUFAF1	salmon	0.56520363
ENSG00000164182	NDUFAF2	brown	0.82960679
ENSG00000178057	NDUFAF3	lightcyan	0.5917516
ENSG00000123545	NDUFAF4	grey	-0.162765
ENSG00000259467	NDUFAF4P1	grey	0.30612697
ENSG00000236411	NDUFAF4P3	grey	0.14821461
ENSG00000231378	NDUFAF4P4	grey	0.31336954
ENSG00000101247	NDUFAF5	turquoise	-0.6105721
ENSG00000156170	NDUFAF6	grey	0.08931454
ENSG00000003509	NDUFAF7	turquoise	-0.4111833
ENSG00000140990	NDUFB10	brown	0.54210158
ENSG00000147123	NDUFB11	brown	0.75090709
ENSG00000235547	NDUFB11P1	grey	0.28450369
ENSG00000234523	NDUFB1P2	grey	0.43230419
ENSG00000090266	NDUFB2	brown	0.8033659
ENSG00000240889	NDUFB2-AS1	grey	0.1794657
ENSG00000065518	NDUFB4	brown	0.41899358
ENSG00000251306	NDUFB4P2	grey	0.20174511
ENSG00000136521	NDUFB5	red	0.66291734
ENSG00000165264	NDUFB6	brown	0.79376276
ENSG00000099795	NDUFB7	brown	0.59808294
ENSG00000166136	NDUFB8	turquoise	-0.6393339
ENSG00000258972	NDUFB8P1	grey	0.3173641
ENSG00000147684	NDUFB9	brown	0.76168747
ENSG00000109390	NDUFC1	brown	0.60945259
ENSG00000023228	NDUFS1	darkred	0.77266106
ENSG00000158864	NDUFS2	brown	0.68261084
ENSG00000213619	NDUFS3	grey60	0.62093747
ENSG00000164258	NDUFS4	yellow	-0.501156
ENSG00000168653	NDUFS5	brown	0.74973555
ENSG00000145494	NDUFS6	brown	0.83152699
ENSG00000115286	NDUFS7	brown	0.58140683
ENSG00000110717	NDUFS8	brown	0.6987559
ENSG00000167792	NDUFV1	brown	0.80080635
ENSG00000178127	NDUFV2	turquoise	-0.5016815
ENSG00000160194	NDUFV3	grey	-0.1390255
ENSG00000245532	NEAT1	yellow	0.63330878
ENSG00000183091	NEB	black	0.75234036
ENSG00000078114	NEBL	turquoise	0.86375391
ENSG00000231920	NEBL-AS1	grey	-0.131072
ENSG00000123119	NECAB1	darkorange	0.57349935

ENSG00000103154	NECAB2	purple	0.83029292
ENSG00000125967	NECAB3	grey	0.12563814
ENSG00000089818	NECAP1	blue	0.79875767
ENSG00000157191	NECAP2	turquoise	0.36875604
ENSG00000139350	NEDD1	blue	-0.5686114
ENSG00000129559	NEDD8	brown	0.87398454
ENSG00000111859	NEDD9	pink	0.73694527
ENSG00000100285	NEFH	blue	0.23518514
ENSG00000104725	NEFL	blue	0.79941667
ENSG00000104722	NEFM	blue	0.63591774
ENSG00000172260	NEGR1	purple	0.79694661
ENSG00000140398	NEIL1	brown	0.51812365
ENSG00000154328	NEIL2	grey	0.0101943
ENSG00000137601	NEK1	turquoise	0.37494016
ENSG00000163491	NEK10	blue	0.7668976
ENSG00000136098	NEK3	yellow	0.64372362
ENSG00000114904	NEK4	turquoise	0.52244722
ENSG00000119408	NEK6	turquoise	0.86560562
ENSG00000151414	NEK7	green	0.89645274
ENSG00000119638	NEK9	blue	-0.3666154
ENSG00000185049	NELFA	turquoise	0.39189986
ENSG00000188986	NELFB	turquoise	-0.4272327
ENSG00000101158	NELFCD	salmon	0.51076876
ENSG00000204356	NELFE	salmon	0.7335212
ENSG00000165973	NELL1	blue	0.5943749
ENSG00000184613	NELL2	brown	0.69506134
ENSG00000165525	NEMF	lightgreen	0.72454985
ENSG00000117691	NENF	grey	-0.4121311
ENSG00000132688	NES	blue	-0.5940872
ENSG00000173848	NET1	tan	0.63998655
ENSG00000166342	NETO1	magenta	0.69243057
ENSG00000204386	NEU1	darkred	0.57708024
ENSG00000162139	NEU3	turquoise	-0.5894295
ENSG00000107954	NEURL1	darkorange	0.6103192
ENSG00000214357	NEURL1B	grey	-0.170004
ENSG00000215041	NEURL4	brown	0.5835098
ENSG00000162614	NEXN	black	0.42726849
ENSG00000196712	NF1	grey	0.23165678
ENSG00000186575	NF2	grey	0.15450993
ENSG00000163531	NFASC	floralwhite	0.6713436
ENSG00000102908	NFAT5	grey	0.11987575
ENSG00000131196	NFATC1	turquoise	0.48094992
ENSG00000176953	NFATC2IP	grey	0.16885692
ENSG00000072736	NFATC3	brown	-0.4808795
ENSG00000100968	NFATC4	turquoise	0.3870414
ENSG00000082641	NFE2L1	darkgrey	0.65857008
ENSG00000116044	NFE2L2	turquoise	0.72551871
ENSG00000162599	NFIA	turquoise	0.90732599

ENSG00000147862	NFIB	turquoise	0.84518737
ENSG00000141905	NFIC	brown	-0.8496744
ENSG00000165030	NFIL3	pink	0.75590148
ENSG00000008441	NFIX	brown	-0.7853423
ENSG00000109320	NFKB1	yellow	0.53919091
ENSG00000100906	NFKBIA	pink	0.65380065
ENSG00000104825	NFKBIB	green	0.469295
ENSG00000167604	NFKBID	grey	0.15093148
ENSG00000146232	NFKBIE	grey	0.21357513
ENSG00000204498	NFKBIL1	grey	-0.1757615
ENSG00000144802	NFKBIZ	pink	0.68883909
ENSG00000170322	NFRKB	grey	-0.167138
ENSG00000244005	NFS1	brown	0.3891149
ENSG00000169599	NFU1	grey	-0.1506969
ENSG00000086102	NFX1	grey	0.06343636
ENSG00000170448	NFXL1	darkgrey	0.54483586
ENSG00000001167	NFYA	green	0.84333325
ENSG00000120837	NFYB	bisque4	0.85541788
ENSG00000066136	NFYC	yellow	-0.4756696
ENSG00000165553	NGB	grey	0.24762601
ENSG00000129460	NGDN	salmon	0.83857444
ENSG00000066248	NGEF	magenta	0.92242405
ENSG00000166681	NGFRAP1	mediumpurp	0.71399048
ENSG00000151092	NGLY1	grey	0.28979441
ENSG00000182768	NGRN	grey	0.19256812
ENSG00000171786	NHLH1	grey	-0.0302983
ENSG00000187566	NHLRC1	grey	0.17846479
ENSG00000196865	NHLRC2	turquoise	0.76862167
ENSG00000188811	NHLRC3	yellow	0.43930566
ENSG00000145912	NHP2	yellow	-0.5465301
ENSG00000100138	NHP2L1	brown	0.77957063
ENSG00000188158	NHS	turquoise	0.63343014
ENSG00000135540	NHSL1	turquoise	0.73277916
ENSG00000145029	NICN1	grey	0.1968274
ENSG00000116962	NID1	turquoise	0.72928935
ENSG00000087303	NID2	grey	0.01396991
ENSG00000196290	NIF3L1	grey	0.11006999
ENSG00000155438	NIFK	grey	-0.1769054
ENSG00000236859	NIFK-AS1	green	0.86154767
ENSG00000177453	NIM1K	grey	0.14138382
ENSG00000100503	NIN	grey	0.16416567
ENSG00000131669	NINJ1	grey	0.00677198
ENSG00000171840	NINJ2	yellow	0.76001208
ENSG00000132603	NIP7	grey	0.36200104
ENSG00000218428	NIP7P3	yellow	0.54874989
ENSG00000170113	NIPA1	green	0.8154385
ENSG00000140157	NIPA2	darkmagenta	-0.6332697
ENSG00000104361	NIPAL2	blue	0.61062311

ENSG00000001461	NIPAL3	yellow	0.52946846
ENSG00000172548	NIPAL4	yellow	0.75353716
ENSG00000164190	NIPBL	blue	-0.5715497
ENSG00000184117	NIPSNAP1	brown	0.64873246
ENSG00000136783	NIPSNAP3A	grey	-0.0661293
ENSG00000165028	NIPSNAP3B	yellow	-0.3880753
ENSG00000010322	NISCH	blue	0.75104016
ENSG00000158793	NIT1	green	-0.6018461
ENSG00000084628	NKAIN1	yellow	0.3275172
ENSG00000188580	NKAIN2	grey	-0.2302549
ENSG00000185942	NKAIN3	turquoise	0.65725918
ENSG00000101198	NKAIN4	turquoise	0.48998749
ENSG00000101882	NKAP	lightcyan	-0.5693044
ENSG00000189134	NKAPL	grey	0.24642887
ENSG00000233382	NKAPP1	grey	0.30476891
ENSG00000140807	NKD1	salmon	-0.5991679
ENSG00000197885	NKIRAS1	blue	0.37325966
ENSG00000168256	NKIRAS2	grey	0.31843868
ENSG00000186416	NKRF	blue	0.7786236
ENSG00000114857	NKTR	red	-0.5004384
ENSG00000136352	NKX2-1	grey	-0.1757996
ENSG00000125820	NKX2-2	yellow	0.54644417
ENSG00000167034	NKX3-1	brown	-0.683196
ENSG00000148826	NKX6-2	yellow	0.7286346
ENSG00000073536	NLE1	yellow	-0.4072913
ENSG00000169760	NLGN1	blue	0.29191627
ENSG00000228213	NLGN1-AS1	grey	-0.1100663
ENSG00000169992	NLGN2	grey	-0.2491089
ENSG00000196338	NLGN3	yellowgreen	0.70877053
ENSG00000146938	NLGN4X	yellow	-0.6193557
ENSG00000165246	NLGN4Y	grey	-0.1263245
ENSG00000087095	NLK	turquoise	-0.3984127
ENSG00000123213	NLN	grey	0.11318891
ENSG00000167984	NLRC3	grey	-0.0345728
ENSG00000091106	NLRC4	grey	0.30794743
ENSG00000140853	NLRC5	pink	0.71821222
ENSG00000091592	NLRP1	yellow	-0.6136875
ENSG00000160703	NLRX1	grey	0.13417232
ENSG00000197696	NMB	grey	0.12812451
ENSG00000169251	NMD3	grey	-0.1954088
ENSG00000239672	NME1	brown	0.77399536
ENSG00000243678	NME1-NME2	grey	0.10261171
ENSG00000103024	NME3	grey	0.04841586
ENSG00000103202	NME4	lightcyan	0.65509232
ENSG00000112981	NME5	blue	0.70206896
ENSG00000172113	NME6	turquoise	0.45715042
ENSG00000181322	NME9	grey	-0.1648182
ENSG00000123609	NMI	pink	0.57346202

ENSG00000173614	NMNAT1	grey	0.1395755
ENSG00000157064	NMNAT2	blue	0.77083537
ENSG00000163864	NMNAT3	grey	0.04908718
ENSG00000153406	NMRAL1	brown	0.65809086
ENSG00000106733	NMRK1	grey	-0.0220803
ENSG00000136448	NMT1	grey	0.22716425
ENSG00000053438	NNAT	purple	0.58742548
ENSG00000112992	NNT	brown	0.42771711
ENSG00000248092	NNT-AS1	turquoise	0.40229818
ENSG00000141101	NOB1	black	0.55592799
ENSG00000188976	NOC2L	grey	0.20812943
ENSG00000173145	NOC3L	brown	-0.5934069
ENSG00000184967	NOC4L	brown	0.40094677
ENSG00000106100	NOD1	pink	0.6816645
ENSG00000115761	NOL10	grey	0.1664212
ENSG00000140939	NOL3	green	0.64810284
ENSG00000101746	NOL4	yellow	-0.7084848
ENSG00000225921	NOL7	turquoise	0.57250083
ENSG00000162408	NOL9	yellow	-0.4629404
ENSG00000166197	NOLC1	turquoise	-0.6831221
ENSG00000146909	NOM1	grey	0.25772363
ENSG00000103512	NOMO1	blue	0.6739596
ENSG00000185164	NOMO2	grey	0.12339158
ENSG00000103226	NOMO3	green	-0.4457942
ENSG00000147140	NONO	turquoise	0.37356599
ENSG00000237522	NONOP2	grey	0.27686697
ENSG00000182117	NOP10	green	-0.7371104
ENSG00000087269	NOP14	turquoise	-0.3651871
ENSG00000048162	NOP16	brown	0.53881549
ENSG00000111641	NOP2	grey	0.16447789
ENSG00000101361	NOP56	salmon	0.75628165
ENSG00000055044	NOP58	turquoise	-0.4095597
ENSG00000196943	NOP9	green	0.51988335
ENSG00000089250	NOS1	magenta	0.54083739
ENSG00000164867	NOS3	tan	0.69881461
ENSG00000142546	NOSIP	brown	0.68737891
ENSG00000163072	NOSTRIN	tan	0.76698241
ENSG00000134250	NOTCH2	turquoise	0.83245129
ENSG00000213240	NOTCH2NL	turquoise	0.64501171
ENSG00000074181	NOTCH3	grey	-0.0666446
ENSG00000204301	NOTCH4	tan	0.79151932
ENSG00000136999	NOV	yellow	-0.4247982
ENSG00000139910	NOVA1	grey60	0.31138165
ENSG00000104967	NOVA2	blue	0.35300412
ENSG00000188747	NOXA1	blue	0.53020459
ENSG00000165555	NOXRED1	grey	0.24294478
ENSG00000185823	NPAP1	grey	-0.1750307
ENSG00000170485	NPAS2	grey	0.09905896

ENSG00000151322	NPAS3	turquoise	0.58105204
ENSG00000149308	NPAT	grey	0.12777727
ENSG00000141458	NPC1	yellow	0.81691787
ENSG00000119655	NPC2	blue	-0.4769411
ENSG00000107281	NPDC1	brown	0.76871417
ENSG00000215440	NPEPL1	grey	-0.1023454
ENSG00000141279	NPEPPS	brown	0.71495069
ENSG00000139574	NPFF	grey	0.0225303
ENSG00000144061	NPHP1	green	-0.5674323
ENSG00000113971	NPHP3	blue	-0.3783163
ENSG00000131697	NPHP4	turquoise	-0.4240885
ENSG00000183426	NPIPA1	grey	-0.1304436
ENSG00000254206	NPIPB11	grey	0.27808908
ENSG00000196436	NPIPB15	grey	-0.0592424
ENSG00000169246	NPIPB3	grey	0.01275004
ENSG00000185864	NPIPB4	grey	0.00602938
ENSG00000243716	NPIPB5	grey	0.07220951
ENSG00000188599	NPIPP1	grey	0.0398667
ENSG00000135838	NPL	turquoise	0.45945499
ENSG00000182446	NPLOC4	brown	0.64531746
ENSG00000181163	NPM1	grey	-0.5063649
ENSG00000229417	NPM1P25	grey	-0.1200251
ENSG00000235677	NPM1P26	grey	0.13266585
ENSG00000249353	NPM1P27	grey	0.26331575
ENSG00000239351	NPM1P29	yellow	0.41278499
ENSG00000228219	NPM1P30	green	0.50241543
ENSG00000219085	NPM1P37	grey	0.13562462
ENSG00000236523	NPM1P40	grey	0.3371931
ENSG00000259440	NPM1P43	grey	0.18232658
ENSG00000158806	NPM2	blue	0.7608387
ENSG00000107833	NPM3	grey	-0.0284917
ENSG00000168743	NPNT	grey	-0.2658828
ENSG00000120937	NPPB	brown	0.34565683
ENSG00000163273	NPPC	grey	0.28940385
ENSG00000159899	NPR2	brown	0.54256866
ENSG00000114388	NPRL2	blue	0.62455266
ENSG00000103148	NPRL3	grey	0.01397917
ENSG00000171246	NPTX1	grey	-0.1808542
ENSG00000106236	NPTX2	black	-0.5156128
ENSG00000221890	NPTXR	yellow	-0.4204163
ENSG00000122585	NPY	magenta	0.56152308
ENSG00000164128	NPY1R	turquoise	-0.4819473
ENSG00000164129	NPY5R	grey	-0.185246
ENSG00000226306	NPY6R	darkred	-0.5925066
ENSG00000181019	NQO1	purple	-0.7227905
ENSG00000124588	NQO2	grey	0.02599284
ENSG00000126368	NR1D1	grey	0.310378
ENSG00000174738	NR1D2	grey	-0.0278095

ENSG00000131408	NR1H2	grey	0.31853787
ENSG00000025434	NR1H3	green	-0.5325923
ENSG00000143257	NR1I3	grey	0.0947243
ENSG00000120798	NR2C1	lightcyan	0.58455065
ENSG00000177463	NR2C2	grey	-0.0968131
ENSG00000112333	NR2E1	turquoise	0.67833205
ENSG00000175745	NR2F1	red	0.55085953
ENSG00000237187	NR2F1-AS1	red	0.79584937
ENSG00000185551	NR2F2	turquoise	0.4213074
ENSG00000160113	NR2F6	grey	0.16032986
ENSG00000113580	NR3C1	grey	-0.0242208
ENSG00000151623	NR3C2	turquoise	0.38952353
ENSG00000148200	NR6A1	grey	0.00182542
ENSG00000198435	NRARP	turquoise	0.58872648
ENSG00000148572	NRBF2	mediumpurp	-0.5777465
ENSG00000270427	NRBF2P5	grey	0.35808326
ENSG00000115216	NRBP1	yellow	-0.3979233
ENSG00000185189	NRBP2	darkred	-0.5229534
ENSG00000091129	NRCAM	red	0.73219642
ENSG00000078618	NRD1	turquoise	-0.5412592
ENSG00000119720	NRDE2	yellow	0.31163547
ENSG00000134986	NREP	red	0.54375422
ENSG00000106459	NRF1	grey	0.16067546
ENSG00000157168	NRG1	blue	0.36132234
ENSG00000253974	NRG1-IT1	grey	0.12859202
ENSG00000253222	NRG1-IT2	grey	0.42672068
ENSG00000158458	NRG2	turquoise	-0.3343523
ENSG00000225738	NRG3-AS1	grey	0.37581793
ENSG00000169752	NRG4	grey	0.04070948
ENSG00000154146	NRGN	magenta	0.90469425
ENSG00000180530	NRIP1	grey	-0.141542
ENSG00000053702	NRIP2	black	0.61358949
ENSG00000175352	NRIP3	blue	0.7877168
ENSG00000137404	NRM	grey	0.13120896
ENSG00000099250	NRP1	grey	0.33047365
ENSG00000118257	NRP2	grey	0.11242954
ENSG00000125841	NRSN2	red	0.4850366
ENSG00000179915	NRXN1	red	0.86624858
ENSG00000110076	NRXN2	yellow	-0.6280411
ENSG00000021645	NRXN3	blue	0.62695984
ENSG00000164346	NSA2	grey	-0.1385998
ENSG00000165671	NSD1	grey	0.19046162
ENSG00000147383	NSDHL	green	-0.4651422
ENSG00000073969	NSF	blue	0.88483187
ENSG00000088833	NSFL1C	blue	0.66443204
ENSG00000168824	NSG1	blue	0.80269668
ENSG00000170091	NSG2	blue	0.84906879
ENSG00000117697	NSL1	grey	-0.2998243

ENSG00000035681	NSMAF	brown	0.46603874
ENSG00000156831	NSMCE2	grey	0.16413105
ENSG00000126653	NSRP1	darkgrey	0.8679385
ENSG00000235613	NSRP1P1	darkgrey	0.7119904
ENSG00000037474	NSUN2	grey	-0.078354
ENSG00000178694	NSUN3	grey	0.08305854
ENSG00000117481	NSUN4	grey	0.04700335
ENSG00000130305	NSUN5	grey	0.02058638
ENSG00000106133	NSUN5P2	grey	-0.1095647
ENSG00000241058	NSUN6	grey	-0.1690705
ENSG00000125458	NT5C	grey	-0.185999
ENSG00000116981	NT5C1A	grey	0.00880632
ENSG00000076685	NT5C2	blue	-0.4303571
ENSG00000122643	NT5C3A	grey	0.05720509
ENSG00000141698	NT5C3B	blue	0.81603974
ENSG00000178425	NT5DC1	yellow	0.46633988
ENSG00000168268	NT5DC2	grey	0.04950026
ENSG00000111696	NT5DC3	turquoise	-0.4523255
ENSG00000135318	NT5E	turquoise	0.6833309
ENSG00000205309	NT5M	grey	0.13924853
ENSG00000157045	NTAN1	grey	-0.1223448
ENSG00000250569	NTAN1P2	grey	0.40623876
ENSG00000065057	NTHL1	brown	0.67775331
ENSG00000182667	NTM	greenyellow	-0.5171832
ENSG00000238262	NTM-IT	grey	0.33545115
ENSG00000148335	NTMT1	brown	0.53571547
ENSG00000065320	NTN1	turquoise	0.32218278
ENSG00000074527	NTN4	turquoise	0.43994606
ENSG00000142233	NTN5	green	0.35998081
ENSG00000196358	NTNG2	grey	-0.1340774
ENSG00000135778	NTPCR	darkred	0.60412907
ENSG00000148053	NTRK2	turquoise	0.77884572
ENSG00000140538	NTRK3	grey	-0.2664236
ENSG00000169006	NTSR2	turquoise	0.67177918
ENSG00000074590	NUAK1	darkgrey	0.56309808
ENSG00000013374	NUB1	grey	0.05550252
ENSG00000103274	NUBP1	lightcyan	0.48010926
ENSG00000095906	NUBP2	grey	0.09548993
ENSG00000151413	NUBPL	grey	-0.0967117
ENSG00000104805	NUCB1	grey	0.30721851
ENSG00000070081	NUCB2	mediumpurp	0.61576301
ENSG00000090273	NUDC	turquoise	-0.4730383
ENSG00000170584	NUDCD2	blue	-0.6919566
ENSG00000015676	NUDCD3	brown	0.75212738
ENSG00000122824	NUDT10	purple	0.75952226
ENSG00000196368	NUDT11	purple	0.74841319
ENSG00000112874	NUDT12	grey	0.14862712
ENSG00000166321	NUDT13	grey	0.0291708

ENSG00000183828	NUDT14	turquoise	-0.5296956
ENSG00000136159	NUDT15	yellow	-0.4642264
ENSG00000198585	NUDT16	green	0.88946896
ENSG00000168101	NUDT16L1	grey	0.02693501
ENSG00000186364	NUDT17	grey	-0.1377795
ENSG00000213965	NUDT19	grey	0.10706852
ENSG00000164978	NUDT2	grey	-0.0262802
ENSG00000167005	NUDT21	greenyellow	0.33734511
ENSG00000149761	NUDT22	darkred	0.55739825
ENSG00000173598	NUDT4	grey	0.0352785
ENSG00000165609	NUDT5	grey	-0.0457813
ENSG00000170917	NUDT6	grey	0.20422703
ENSG00000140876	NUDT7	grey	0.19812714
ENSG00000167799	NUDT8	grey	0.30984465
ENSG00000083635	NUFIP1	grey	0.02329055
ENSG00000108256	NUFIP2	turquoise	0.58725847
ENSG00000137497	NUMA1	yellow	0.29756727
ENSG00000133961	NUMB	grey	-0.0404688
ENSG00000105245	NUMBL	grey	0.0083634
ENSG00000069248	NUP133	turquoise	-0.3666951
ENSG00000124789	NUP153	brown	-0.6574632
ENSG00000030066	NUP160	blue	-0.6399819
ENSG00000155561	NUP205	grey	0.21034491
ENSG00000132182	NUP210	grey	0.21600269
ENSG00000126883	NUP214	grey	-0.0285154
ENSG00000163002	NUP35	grey	0.08361709
ENSG00000075188	NUP37	grey	0.12025316
ENSG00000093000	NUP50	brown	0.55373556
ENSG00000138750	NUP54	grey	0.28632962
ENSG00000213024	NUP62	grey	-0.0049553
ENSG00000125450	NUP85	turquoise	-0.3739996
ENSG00000108559	NUP88	grey	-0.0650291
ENSG00000102900	NUP93	blue	0.51746196
ENSG00000110713	NUP98	pink	0.52747733
ENSG00000139496	NUPL1	brown	0.5802259
ENSG00000136243	NUPL2	yellow	-0.5078902
ENSG00000176046	NUPR1	turquoise	0.30103378
ENSG00000185290	NUPR1L	green	-0.4881896
ENSG00000153989	NUS1	grey	-0.1935059
ENSG00000102898	NUTF2	lightcyan	0.80613295
ENSG00000223482	NUTM2A-AS1	grey	0.07838831
ENSG00000143748	NVL	grey	-0.0614644
ENSG00000188039	NWD1	turquoise	0.49227399
ENSG00000162231	NXF1	turquoise	-0.5661355
ENSG00000167693	NXN	tan	0.48286792
ENSG00000144815	NXPE3	yellow	0.54052782
ENSG00000132661	NXT1	grey	0.21191864
ENSG00000101888	NXT2	turquoise	0.70368662

ENSG00000166924	NYAP1	grey	-0.1186386
ENSG00000184232	OAF	turquoise	0.60682557
ENSG00000124596	OARD1	lightgreen	0.65049149
ENSG00000089127	OAS1	grey	-0.1943075
ENSG00000111335	OAS2	grey	0.00013348
ENSG00000111331	OAS3	tan	0.50328647
ENSG00000065154	OAT	pink	-0.4701448
ENSG00000104904	OAZ1	greenyellow	0.66491252
ENSG00000180304	OAZ2	grey	0.11834256
ENSG00000143450	OAZ3	salmon	-0.6055911
ENSG00000107960	OBFC1	grey	-0.0424367
ENSG00000154358	OBSCN	magenta	0.55366769
ENSG00000124006	OBSL1	red	0.7750705
ENSG00000099330	OCEL1	grey	0.12024154
ENSG00000109180	OCIAD1	green	-0.7366795
ENSG00000248256	OCIAD1-AS1	brown	-0.3648681
ENSG00000145247	OCIAD2	brown	0.78695247
ENSG00000197822	OCLN	tan	0.49343406
ENSG00000122126	OCRL	brown	0.77396589
ENSG00000136811	ODF2	grey	-0.0871666
ENSG00000122417	ODF2L	blue	0.75454421
ENSG00000046651	OFD1	red	-0.6384181
ENSG00000228212	OFD1P17	grey	0.11098344
ENSG00000105953	OGDH	grey	0.2379926
ENSG00000197444	OGDHL	blue	0.47195698
ENSG00000087263	OGFOD1	grey	-0.0784556
ENSG00000181396	OGFOD3	grey	0.15345579
ENSG00000229873	OGFR-AS1	grey	0.35660392
ENSG00000119900	OGFRL1	brown	-0.5492865
ENSG00000114026	OGG1	black	0.5211209
ENSG00000106809	OGN	grey	-0.0146456
ENSG00000147162	OGT	red	-0.593996
ENSG00000247556	OIP5-AS1	grey	0.19953512
ENSG00000138430	OLA1	green	-0.6565329
ENSG00000213790	OLA1P1	grey	0.23346678
ENSG00000105088	OLFM2	turquoise	0.82838718
ENSG00000118733	OLFM3	blue	0.63216468
ENSG00000183801	OLFML1	grey	0.00600171
ENSG00000116774	OLFML3	cyan	0.62728086
ENSG00000184221	OLIG1	turquoise	0.46694926
ENSG00000205927	OLIG2	yellow	0.39978275
ENSG00000173391	OLR1	cyan	0.62877977
ENSG00000127083	OMD	tan	0.68082314
ENSG00000126861	OMG	grey	-0.159013
ENSG00000119547	ONECUT2	magenta	0.70308668
ENSG00000198836	OPA1	brown	0.64289373
ENSG00000224855	OPA1-AS1	grey	0.40089079
ENSG00000125741	OPA3	grey	0.14407407

ENSG00000197430	OPALIN	yellow	0.7624441
ENSG00000183715	OPCML	grey60	0.66548754
ENSG00000254896	OPCML-IT1	black	0.63470712
ENSG00000255371	OPCML-IT2	grey	0.30649012
ENSG00000128617	OPN1SW	grey	0.17768855
ENSG00000122375	OPN4	magenta	0.40834982
ENSG00000125510	OPRL1	blue	0.69445142
ENSG00000112038	OPRM1	purple	0.84494417
ENSG00000123240	OPTN	lightcyan	-0.5642831
ENSG00000170790	OR10A2	grey	0.19083304
ENSG00000170782	OR10A4	grey	0.17554338
ENSG00000166363	OR10A5	grey	0.39458954
ENSG00000172640	OR10AD1	grey	0.09292697
ENSG00000236083	OR13E1P	grey	0.0995469
ENSG00000168828	OR13J1	grey	0.32401466
ENSG00000189181	OR14I1	blue	0.42227748
ENSG00000168124	OR1F1	grey	0.12741508
ENSG00000244479	OR2A1-AS1	grey	0.15408078
ENSG00000170803	OR2AG1	grey	0.2887669
ENSG00000187080	OR2AK2	grey	0.04379282
ENSG00000166368	OR2D2	grey	0.40521945
ENSG00000178358	OR2D3	grey	0.27419892
ENSG00000204657	OR2H2	grey	0.32421715
ENSG00000196071	OR2L13	grey	0.02563346
ENSG00000224227	OR2L1P	grey	0.20647914
ENSG00000197454	OR2L5	grey	0.22497251
ENSG00000238243	OR2W3	blue	0.41304691
ENSG00000221882	OR3A2	grey	0.06533207
ENSG00000268067	OR5AH1P	green	0.45301239
ENSG00000257763	OR5BK1P	grey	0.11363612
ENSG00000225781	OR6V1	yellow	0.66156553
ENSG00000254593	OR7E126P	grey	0.43388548
ENSG00000228915	OR7E128P	grey	0.13517103
ENSG00000184669	OR7E14P	grey	0.19654334
ENSG00000254715	OR7E154P	grey	0.20929939
ENSG00000183444	OR7E38P	grey	0.13526998
ENSG00000238228	OR7E7P	grey	0.05821624
ENSG00000226413	OR8T1P	grey	-0.0337273
ENSG00000179468	OR9A2	yellow	0.69664381
ENSG00000239293	OR9P1P	yellow	0.41648832
ENSG00000182500	ORAI1	turquoise	0.55259893
ENSG00000160991	ORAI2	yellow	-0.6709575
ENSG00000175938	ORAI3	turquoise	0.44940086
ENSG00000149716	ORAOV1	grey	-0.0146493
ENSG00000115942	ORC2	grey	0.03159465
ENSG00000135336	ORC3	grey	0.13024257
ENSG00000115947	ORC4	lightgreen	-0.7236334
ENSG00000164815	ORC5	brown	0.77906764

ENSG00000091651	ORC6	grey	-0.0978533
ENSG00000128699	ORMDL1	grey	-0.065357
ENSG00000123353	ORMDL2	blue	-0.3085221
ENSG00000172057	ORMDL3	grey	-0.2036498
ENSG00000135506	OS9	salmon	0.58493658
ENSG00000110048	OSBP	grey	0.19829423
ENSG00000184792	OSBP2	turquoise	-0.4861651
ENSG00000144645	OSBPL10	blue	0.46760355
ENSG00000144909	OSBPL11	turquoise	0.41235408
ENSG00000141447	OSBPL1A	yellow	0.77716897
ENSG00000130703	OSBPL2	greenyellow	-0.4914239
ENSG00000070882	OSBPL3	grey	0.00928508
ENSG00000021762	OSBPL5	grey	0.32436508
ENSG00000079156	OSBPL6	grey	0.20996772
ENSG00000006025	OSBPL7	grey	-0.2324768
ENSG00000117859	OSBPL9	yellow	0.50213331
ENSG00000116885	OSCP1	brown	0.72118867
ENSG00000132823	OSER1	grey	0.32312688
ENSG00000223891	OSER1-AS1	grey	0.22848189
ENSG00000092094	OSGEP	brown	0.44588451
ENSG00000128694	OSGEPL1	grey	-0.0796125
ENSG00000253559	OSGEPL1-AS1	grey	0.24092521
ENSG00000164823	OSGIN2	green	0.34356256
ENSG00000145623	OSMR	pink	0.92785346
ENSG00000228474	OST4	red	0.49649685
ENSG00000198856	OSTC	magenta	-0.3810206
ENSG00000219790	OSTCP6	grey	0.41950586
ENSG00000226801	OSTCP8	grey	0.36322791
ENSG00000134996	OSTF1	grey	-0.0902672
ENSG00000081087	OSTM1	grey	-0.0794489
ENSG00000115155	OTOF	magenta	0.63931445
ENSG00000178602	OTOS	grey	0.15730275
ENSG00000167770	OTUB1	blue	0.69467343
ENSG00000089723	OTUB2	blue	0.47140785
ENSG00000165312	OTUD1	grey	-0.2002266
ENSG00000169914	OTUD3	turquoise	-0.529353
ENSG00000068308	OTUD5	turquoise	-0.4719939
ENSG00000155100	OTUD6B	grey	0.09669464
ENSG00000169918	OTUD7A	grey	0.16559031
ENSG00000163113	OTUD7B	green	0.9338006
ENSG00000187950	OVCH1	turquoise	-0.4339612
ENSG00000085465	OVGP1	grey	-0.1952391
ENSG00000155463	OXA1L	grey	-0.0359619
ENSG00000204237	OXLD1	brown	0.587557
ENSG00000154814	OXNAD1	grey	0.16938579
ENSG00000164830	OXR1	grey	0.0354091
ENSG00000151093	OXSM	grey	0.01094394
ENSG00000172939	OXSR1	turquoise	0.42767475

ENSG00000180914	OXTR	turquoise	0.32781553
ENSG00000135124	P2RX4	grey	-0.0221463
ENSG00000083454	P2RX5	brown	0.74699414
ENSG00000099957	P2RX6	grey	-0.0849622
ENSG00000089041	P2RX7	yellow	0.75234463
ENSG00000169860	P2RY1	red	0.6362874
ENSG00000244165	P2RY11	brown	0.40378742
ENSG00000169313	P2RY12	cyan	0.73138393
ENSG00000181631	P2RY13	cyan	0.68947642
ENSG00000174944	P2RY14	turquoise	0.55013876
ENSG00000122884	P4HA1	turquoise	0.42222955
ENSG00000072682	P4HA2	pink	0.62250657
ENSG00000185624	P4HB	pink	0.60825293
ENSG00000178467	P4HTM	blue	0.80045227
ENSG00000170515	PA2G4	greenyellow	-0.3703597
ENSG00000230457	PA2G4P4	grey	0.10067708
ENSG00000175575	PAAF1	darkgrey	0.50646845
ENSG00000070756	PABPC1	royalblue	0.66113557
ENSG00000101104	PABPC1L	brown	0.4061198
ENSG00000186288	PABPC1L2A	blue	0.65714633
ENSG00000184388	PABPC1L2B	brown	0.67520255
ENSG00000231707	PABPC1P1	grey	0.33888999
ENSG00000228810	PABPC1P11	grey	0.32213206
ENSG00000230673	PABPC1P3	grey	0.3954663
ENSG00000255642	PABPC1P4	turquoise	0.38354165
ENSG00000250651	PABPC1P7	grey	0.17153984
ENSG00000090621	PABPC4	cyan	0.64165183
ENSG00000174740	PABPC5	brown	-0.6754989
ENSG00000100836	PABPN1	grey	-0.3574145
ENSG00000112530	PACRG	blue	0.55762937
ENSG00000163138	PACRGL	grey	0.03628681
ENSG00000175115	PACS1	turquoise	-0.5553068
ENSG00000179364	PACS2	grey	-0.0764605
ENSG00000124507	PACSIN1	darkorange	0.82234603
ENSG00000100266	PACSIN2	grey	0.03088495
ENSG00000165912	PACSIN3	blue	-0.3486003
ENSG00000117115	PADI2	yellow	0.57507892
ENSG00000006712	PAF1	lightcyan	-0.7040155
ENSG00000168092	PAFAH1B2	brown	-0.6139184
ENSG00000079462	PAFAH1B3	grey	-0.3854014
ENSG00000158006	PAFAH2	turquoise	0.46570572
ENSG00000076641	PAG1	blue	-0.458775
ENSG00000185928	PAGR1	lightgreen	0.52670765
ENSG00000128050	PAICS	turquoise	0.45206891
ENSG00000226055	PAICSP1	grey	-0.0796697
ENSG00000254244	PAICSP4	grey	-0.1454527
ENSG00000172239	PAIP1	lightgreen	0.55452154
ENSG00000120727	PAIP2	grey	-0.0048596

ENSG00000124374	PAIP2B	green	0.891404
ENSG00000149269	PAK1	blue	0.79044386
ENSG00000111845	PAK1IP1	grey	-0.055084
ENSG00000180370	PAK2	brown	-0.8214954
ENSG00000077264	PAK3	purple	0.82697825
ENSG00000130669	PAK4	grey	0.01789132
ENSG00000101349	PAK7	blue	0.57196168
ENSG00000083093	PALB2	grey	0.14504481
ENSG00000107719	PALD1	cyan	0.68140827
ENSG00000129116	PALLD	turquoise	0.69308312
ENSG00000099864	PALM	grey	-0.0485753
ENSG00000243444	PALM2	grey	0.09899126
ENSG00000099260	PALMD	tan	0.55494676
ENSG00000145730	PAM	brown	0.79990892
ENSG00000217930	PAM16	grey	0.06897139
ENSG00000149090	PAMR1	turquoise	0.5031155
ENSG00000152520	PAN3	brown	-0.3663686
ENSG00000261485	PAN3-AS1	grey	-0.1093579
ENSG00000152782	PANK1	red	0.46388253
ENSG00000125779	PANK2	grey	0.07449451
ENSG00000120137	PANK3	turquoise	0.75536614
ENSG00000157881	PANK4	brown	0.62279112
ENSG00000110218	PANX1	grey	0.03795199
ENSG00000148832	PAOX	grey	0.16572217
ENSG00000164329	PAPD4	grey	-0.1095647
ENSG00000112941	PAPD7	grey	0.11657072
ENSG00000100767	PAPLN	turquoise	0.5815347
ENSG00000115421	PAPOLG	grey	-0.0402842
ENSG00000138801	PAPSS1	darkmagenta	0.5506053
ENSG00000198682	PAPSS2	pink	0.65541256
ENSG00000163291	PAQR3	grey	0.24399008
ENSG00000162073	PAQR4	yellow	0.73548485
ENSG00000137819	PAQR5	tan	0.60885226
ENSG00000160781	PAQR6	green	0.82832448
ENSG00000182749	PAQR7	grey	-0.1733379
ENSG00000170915	PAQR8	turquoise	0.83442933
ENSG00000188582	PAQR9	blue	0.69067747
ENSG00000148498	PARD3	grey	-0.0052257
ENSG00000226386	PARD3-AS1	grey	0.28521443
ENSG00000116117	PARD3B	turquoise	0.43128869
ENSG00000102981	PARD6A	yellow	-0.4623111
ENSG00000124171	PARD6B	grey	-0.1299457
ENSG00000178184	PARD6G	grey	0.23383217
ENSG00000227345	PARG	grey	0.17615556
ENSG00000214982	PARGP1	grey	0.20139296
ENSG00000185345	PARK2	blue	0.48210441
ENSG00000116288	PARK7	salmon	0.86013792
ENSG00000175193	PARL	grey	-0.0103779

ENSG00000169116	PARM1	blue	0.51117265
ENSG00000140694	PARN	grey	-0.148004
ENSG00000111224	PARP11	grey	-0.0276358
ENSG00000059378	PARP12	tan	0.55301781
ENSG00000173193	PARP14	blue	-0.6103271
ENSG00000138617	PARP16	grey	0.18407876
ENSG00000129484	PARP2	grey	-0.1852151
ENSG00000041880	PARP3	grey	0.22057606
ENSG00000102699	PARP4	yellow	0.61519049
ENSG00000137817	PARP6	turquoise	-0.7470418
ENSG00000151883	PARP8	red	0.72407989
ENSG00000138496	PARP9	blue	-0.6628624
ENSG00000162396	PARS2	greenyellow	-0.4553239
ENSG00000197702	PARVA	turquoise	0.82017139
ENSG00000188677	PARVB	turquoise	-0.4184089
ENSG00000115687	PASK	grey	-0.1372172
ENSG00000171053	PATE1	grey	0.13882974
ENSG00000196844	PATE2	grey	0.11164962
ENSG00000236027	PATE3	grey	0.20334155
ENSG00000237353	PATE4	grey	0.08170733
ENSG00000166889	PATL1	brown	-0.674176
ENSG00000100105	PATZ1	grey	0.0204897
ENSG00000177425	PAWR	turquoise	0.5950542
ENSG00000007372	PAX6	turquoise	0.81171644
ENSG00000189223	PAX8-AS1	grey	0.03126231
ENSG00000159086	PAXBP1	red	-0.629461
ENSG00000238197	PAXBP1-AS1	grey	0.20595215
ENSG00000157212	PAXIP1	grey	0.35021354
ENSG00000214106	PAXIP1-AS2	turquoise	0.6738522
ENSG00000102390	PBDC1	grey	-0.173192
ENSG00000108187	PBLD	grey	0.11320865
ENSG00000254440	PBOV1	grey	0.11099085
ENSG00000163939	PBRM1	lightgreen	0.56674036
ENSG00000185630	PBX1	red	0.76315767
ENSG00000204304	PBX2	grey	-0.1867786
ENSG00000167081	PBX3	blue	0.50217531
ENSG00000105717	PBX4	grey	0.01344826
ENSG00000163346	PBXIP1	turquoise	0.69047795
ENSG00000173599	PC	salmon	0.51735028
ENSG00000225937	PCA3	yellow	0.72104803
ENSG00000228288	PCAT6	grey	0.03648259
ENSG00000169564	PCBP1	grey	-0.0107066
ENSG00000179818	PCBP1-AS1	yellow	0.52399931
ENSG00000183570	PCBP3	blue	0.5683194
ENSG00000090097	PCBP4	yellow	0.6582531
ENSG00000175198	PCCA	grey	0.20208795
ENSG00000234650	PCCA-AS1	turquoise	0.39899113
ENSG00000114054	PCCB	darkred	0.51196661

ENSG00000156453	PCDH1	blue	0.42289597
ENSG00000138650	PCDH10	grey	-0.1857261
ENSG00000102290	PCDH11X	purple	0.81265403
ENSG00000099715	PCDH11Y	grey	-0.1434479
ENSG00000113555	PCDH12	grey	0.15737919
ENSG00000118946	PCDH17	grey	-0.0940191
ENSG00000189184	PCDH18	turquoise	0.41305558
ENSG00000165194	PCDH19	purple	0.72910579
ENSG00000197991	PCDH20	blue	0.6291238
ENSG00000169851	PCDH7	grey60	0.68271687
ENSG00000136099	PCDH8	greenyellow	0.55732638
ENSG00000225263	PCDH9-AS3	yellow	0.52402686
ENSG00000204970	PCDHA1	grey	0.15016053
ENSG00000249158	PCDHA11	grey	0.21387161
ENSG00000255408	PCDHA3	grey	0.19049392
ENSG00000204965	PCDHA5	grey	0.29234822
ENSG00000081842	PCDHA6	brown	0.38838669
ENSG00000243232	PCDHAC2	blue	0.52708411
ENSG00000120324	PCDHB10	grey	0.17979207
ENSG00000197479	PCDHB11	grey	0.15690777
ENSG00000120328	PCDHB12	grey	0.13611989
ENSG00000187372	PCDHB13	grey	0.13408096
ENSG00000120327	PCDHB14	magenta	-0.3796141
ENSG00000113248	PCDHB15	grey	0.13015066
ENSG00000196963	PCDHB16	grey	0.21599626
ENSG00000255622	PCDHB17	grey	0.07770101
ENSG00000146001	PCDHB18	grey	0.09812299
ENSG00000262096	PCDHB19P	grey	0.36406842
ENSG00000112852	PCDHB2	magenta	-0.4096751
ENSG00000113205	PCDHB3	grey	0.1558432
ENSG00000081818	PCDHB4	grey	-0.0530734
ENSG00000113209	PCDHB5	grey	-0.0396483
ENSG00000113211	PCDHB6	grey	0.08064315
ENSG00000113212	PCDHB7	grey	0.12578496
ENSG00000120322	PCDHB8	grey	0.12694592
ENSG00000253846	PCDHGA10	turquoise	0.55044906
ENSG00000253873	PCDHGA11	turquoise	0.45929468
ENSG00000253159	PCDHGA12	turquoise	0.39286778
ENSG00000081853	PCDHGA2	turquoise	0.42140002
ENSG00000254245	PCDHGA3	turquoise	0.59675528
ENSG00000262576	PCDHGA4	turquoise	0.51188207
ENSG00000253485	PCDHGA5	grey	0.02600227
ENSG00000253731	PCDHGA6	grey	0.06002038
ENSG00000253537	PCDHGA7	grey	0.07390701
ENSG00000261934	PCDHGA9	turquoise	0.65309393
ENSG00000254221	PCDHGB1	turquoise	0.67597677
ENSG00000253910	PCDHGB2	turquoise	0.51372099
ENSG00000262209	PCDHGB3	brown	-0.5091825

ENSG00000253953	PCDHGB4	turquoise	0.39181771
ENSG00000253305	PCDHGB6	turquoise	0.62973701
ENSG00000254122	PCDHGB7	turquoise	0.82976685
ENSG00000248449	PCDHGB8P	grey	0.25525331
ENSG00000240184	PCDHGC3	turquoise	0.79888478
ENSG00000132635	PCED1A	yellow	0.57288662
ENSG00000179715	PCED1B	yellow	-0.3475701
ENSG00000247774	PCED1B-AS1	cyan	0.62066396
ENSG00000115289	PCGF1	green	-0.3928866
ENSG00000056661	PCGF2	grey	0.04947825
ENSG00000180628	PCGF5	turquoise	0.81371886
ENSG00000156374	PCGF6	grey	0.13541303
ENSG00000126226	PCID2	grey	-0.1214876
ENSG00000100982	PCIF1	greenyellow	-0.4576457
ENSG00000100889	PCK2	turquoise	0.50086681
ENSG00000186472	PCLO	darkorange	0.70732219
ENSG00000078674	PCM1	lightgreen	0.63504366
ENSG00000203880	PCMTD2	grey	-0.2019635
ENSG00000132646	PCNA	grey	0.07497342
ENSG00000081154	PCNP	brown	-0.7553224
ENSG00000258359	PCNPP1	grey	0.1783748
ENSG00000160299	PCNT	darkmagenta	0.62489153
ENSG00000100731	PCNX	turquoise	-0.5610679
ENSG00000197136	PCNXL3	greenyellow	-0.4989595
ENSG00000126773	PCNXL4	turquoise	0.4043554
ENSG00000106333	PCOLCE	grey	-0.0205524
ENSG00000183036	PCP4	turquoise	-0.5889622
ENSG00000248485	PCP4L1	magenta	0.66168306
ENSG00000175426	PCSK1	blue	0.43514165
ENSG00000102109	PCSK1N	blue	0.73642131
ENSG00000125851	PCSK2	blue	0.58535277
ENSG00000099139	PCSK5	turquoise	0.59060525
ENSG00000140479	PCSK6	red	-0.7958602
ENSG00000160613	PCSK7	grey	0.05360634
ENSG00000141179	PCTP	red	0.55357131
ENSG00000116005	PCYOX1	turquoise	0.64787489
ENSG00000161217	PCYT1A	turquoise	0.6453135
ENSG00000102230	PCYT1B	purple	0.49533484
ENSG00000185813	PCYT2	brown	0.61854708
ENSG00000106244	PDAP1	greenyellow	0.42461464
ENSG00000116703	PDC	grey	0.12225694
ENSG00000148843	PDCD11	lightcyan	-0.6456614
ENSG00000197646	PDCD1LG2	grey	0.18357783
ENSG00000071994	PDCD2	grey	-0.0634323
ENSG00000150593	PDCD4	grey	-0.2525336
ENSG00000105185	PDCD5	greenyellow	0.42626073
ENSG00000170248	PDCD6IP	brown	0.32882614
ENSG00000090470	PDCD7	grey	0.04839819

ENSG00000136940	PDCL	turquoise	0.65604042
ENSG00000115539	PDCL3	grey	0.15735305
ENSG00000244119	PDCL3P4	grey	0.15164362
ENSG00000177225	PDDC1	grey	-0.0699468
ENSG00000112541	PDE10A	magenta	0.74330486
ENSG00000174840	PDE12	grey	0.34038031
ENSG00000115252	PDE1A	grey	-0.1472979
ENSG00000123360	PDE1B	magenta	0.79633856
ENSG00000154678	PDE1C	yellow	0.80543087
ENSG00000186642	PDE2A	magenta	0.80034026
ENSG00000172572	PDE3A	red	0.70420068
ENSG00000152270	PDE3B	red	-0.5509768
ENSG00000065989	PDE4A	yellow	-0.5550121
ENSG00000113448	PDE4D	black	-0.6877596
ENSG00000178104	PDE4DIP	grey	-0.0123398
ENSG00000138735	PDE5A	grey	0.07344387
ENSG00000133256	PDE6B	grey	-0.2866173
ENSG00000095464	PDE6C	grey	0.37169524
ENSG00000156973	PDE6D	turquoise	0.61796214
ENSG00000171408	PDE7B	magenta	0.66448717
ENSG00000073417	PDE8A	yellow	0.87915296
ENSG00000113231	PDE8B	black	0.78813254
ENSG00000160191	PDE9A	grey60	0.5899194
ENSG00000197461	PDGFA	green	0.64877039
ENSG00000100311	PDGFB	grey	-0.1747052
ENSG00000145431	PDGFC	turquoise	0.36020348
ENSG00000170962	PDGFD	grey	0.04685345
ENSG00000113721	PDGFRB	grey	0.04079979
ENSG00000104213	PDGFRL	grey	0.10222667
ENSG00000131828	PDHA1	salmon	0.72281289
ENSG00000168291	PDHB	darkred	0.69929049
ENSG00000110435	PDHX	grey	0.15930135
ENSG00000185615	PDIA2	grey	-0.2242131
ENSG00000180867	PDIA3P1	grey	0.06953639
ENSG00000065485	PDIA5	grey	-0.0500178
ENSG00000175087	PDIK1L	brown	-0.4769063
ENSG00000152256	PDK1	pink	0.41847833
ENSG00000005882	PDK2	grey	0.07678638
ENSG00000067992	PDK3	blue	0.47611086
ENSG00000004799	PDK4	blue	-0.6769321
ENSG00000249274	PDLIM1P4	black	0.4934341
ENSG00000131435	PDLIM4	pink	0.66861699
ENSG00000163110	PDLIM5	turquoise	0.87069538
ENSG00000196923	PDLIM7	blue	0.50946951
ENSG00000164951	PDP1	turquoise	-0.4605714
ENSG00000172840	PDP2	turquoise	0.79646386
ENSG00000140992	PDPK1	brown	0.6547664
ENSG00000205918	PDPK2	yellow	-0.3368175

ENSG00000162493	PDPN	turquoise	0.37458374
ENSG00000090857	PDPR	grey	0.26108167
ENSG00000088356	PDRG1	brown	0.49999794
ENSG00000083642	PDS5B	turquoise	-0.4982954
ENSG00000148459	PDSS1	grey	0.07475132
ENSG00000164494	PDSS2	lightgreen	-0.5666993
ENSG00000179889	PDXDC1	grey	-0.1065684
ENSG00000255185	PDXDC2P	grey	0.30064635
ENSG00000241360	PDXP	blue	0.53477356
ENSG00000101327	PDYN	purple	0.5236368
ENSG00000120509	PDZD11	red	0.77858442
ENSG00000133401	PDZD2	magenta	0.58139146
ENSG00000067840	PDZD4	magenta	0.63586255
ENSG00000186862	PDZD7	blue	0.53761988
ENSG00000165650	PDZD8	blue	-0.4613014
ENSG00000155714	PDZD9	grey	0.13973472
ENSG00000121440	PDZRN3	red	0.57920839
ENSG00000165966	PDZRN4	grey	-0.0417172
ENSG00000162734	PEA15	grey60	-0.5858193
ENSG00000173517	PEAK1	darkorange	0.49880652
ENSG00000187800	PEAR1	tan	0.70352617
ENSG00000089220	PEBP1	grey	-0.2693246
ENSG00000134020	PEBP4	brown	-0.4814503
ENSG00000115425	PECR	turquoise	0.41288202
ENSG00000162517	PEF1	brown	0.79331809
ENSG00000198300	PEG3	blue	0.79168217
ENSG00000197329	PELI1	turquoise	-0.4260969
ENSG00000139946	PELI2	brown	-0.8523353
ENSG00000174516	PELI3	brown	0.61886537
ENSG00000152684	PELO	grey	0.32908534
ENSG00000141456	PELP1	turquoise	-0.5798628
ENSG00000133027	PEMT	red	0.53530188
ENSG00000124299	PEPD	turquoise	0.44210022
ENSG00000179094	PER1	grey	0.26384329
ENSG00000132326	PER2	grey	0.08205937
ENSG00000049246	PER3	grey	-0.007884
ENSG00000112378	PERP	blue	-0.4819364
ENSG00000100029	PES1	grey	0.08078312
ENSG00000059691	PET112	turquoise	-0.3364121
ENSG00000127980	PEX1	yellow	0.55786955
ENSG00000157911	PEX10	grey	0.0261931
ENSG00000166821	PEX11A	turquoise	0.58678377
ENSG00000131779	PEX11B	brown	-0.4940431
ENSG00000104883	PEX11G	grey	0.22579946
ENSG00000108733	PEX12	turquoise	0.49815045
ENSG00000162928	PEX13	grey	0.10193837
ENSG00000142655	PEX14	turquoise	-0.4852843
ENSG00000121680	PEX16	brown	0.52751238

ENSG00000162735	PEX19	turquoise	0.61419764
ENSG00000164751	PEX2	turquoise	0.58448161
ENSG00000215193	PEX26	yellow	-0.5665901
ENSG00000034693	PEX3	grey	-0.0229584
ENSG00000139197	PEX5	grey	-0.0047589
ENSG00000114757	PEX5L	yellow	0.74323951
ENSG00000243799	PEX5L-AS1	yellow	0.55692785
ENSG00000244302	PEX5L-AS2	yellow	0.59613721
ENSG00000124587	PEX6	grey	-0.0761315
ENSG00000112357	PEX7	grey	-0.0828945
ENSG00000178921	PFAS	grey	-0.0557166
ENSG00000113068	PFDN1	blue	0.49932434
ENSG00000101132	PFDN4	grey	-0.1755607
ENSG00000123349	PFDN5	brown	0.58581394
ENSG00000204220	PFDN6	green	-0.4184636
ENSG00000123836	PFKFB2	grey	0.08968279
ENSG00000170525	PFKFB3	turquoise	0.56089257
ENSG00000141959	PFKL	grey	0.18475179
ENSG00000152556	PFKM	darkred	0.88280903
ENSG00000067057	PFKP	turquoise	0.39886523
ENSG00000108518	PFN1	royalblue	0.59743108
ENSG00000203843	PFN1P2	grey	0.1990271
ENSG00000244371	PFN1P8	grey	0.08547104
ENSG00000176732	PFN4	grey	0.3518647
ENSG00000171314	PGAM1	grey	0.13218754
ENSG00000220515	PGAM1P10	grey	0.24108314
ENSG00000213997	PGAM1P7	grey	0.22151492
ENSG00000249255	PGAM1P9	grey	0.27953026
ENSG00000197121	PGAP1	black	-0.7809362
ENSG00000148985	PGAP2	yellow	0.43374951
ENSG00000161395	PGAP3	grey	-0.1771526
ENSG00000137338	PGBD1	grey	-0.038736
ENSG00000185220	PGBD2	grey	0.24754835
ENSG00000182405	PGBD4	grey	0.16472136
ENSG00000249834	PGBD4P3	grey	0.36453726
ENSG00000177614	PGBD5	blue	0.7080595
ENSG00000142657	PGD	turquoise	0.38247023
ENSG00000164219	PGGT1B	brown	-0.6530749
ENSG00000102144	PGK1	green	-0.6672414
ENSG00000130313	PGLS	grey	-0.0994745
ENSG00000008438	PGLYRP1	grey	0.147532
ENSG00000079739	PGM1	turquoise	0.58964892
ENSG00000169299	PGM2	grey	0.20637402
ENSG00000165434	PGM2L1	purple	0.84593651
ENSG00000013375	PGM3	turquoise	0.64621004
ENSG00000204794	PGM5P1	grey	-0.0720704
ENSG00000227558	PGM5P2	grey	0.00335695
ENSG00000184207	PGP	blue	0.5779126

ENSG00000130517	PGPEP1	bisque4	0.61276801
ENSG00000101856	PGRMC1	blue	0.48767806
ENSG00000164040	PGRMC2	grey	0.11007582
ENSG00000087157	PGS1	grey	-0.0907633
ENSG00000112137	PHACTR1	magenta	0.87335876
ENSG00000112419	PHACTR2	magenta	-0.6802
ENSG00000270300	PHACTR2P1	grey	0.18076204
ENSG00000087495	PHACTR3	green	0.74472761
ENSG00000204138	PHACTR4	turquoise	0.43018732
ENSG00000164902	PHAX	grey	0.22581151
ENSG00000167085	PHB	grey	0.2968099
ENSG00000215021	PHB2	yellow	-0.4158443
ENSG00000230585	PHBP12	grey	0.30795847
ENSG00000234835	PHBP13	grey	0.24668663
ENSG00000232210	PHBP15	grey	-0.0108729
ENSG00000230224	PHBP9	grey	0.2560536
ENSG00000111752	PHC1	salmon	-0.5541172
ENSG00000179899	PHC1P1	grey	0.14588441
ENSG00000173889	PHC3	grey	0.07232668
ENSG00000224204	PHEX-AS1	grey	-0.0045326
ENSG00000112511	PHF1	grey	0.03237927
ENSG00000130024	PHF10	brown	-0.8508651
ENSG00000136147	PHF11	grey60	-0.3515119
ENSG00000109118	PHF12	brown	-0.2733621
ENSG00000116273	PHF13	grey	0.07825559
ENSG00000106443	PHF14	salmon	0.66691205
ENSG00000119403	PHF19	blue	-0.5326155
ENSG00000197724	PHF2	blue	-0.7100121
ENSG00000025293	PHF20	turquoise	-0.5250699
ENSG00000129292	PHF20L1	grey	-0.2096431
ENSG00000135365	PHF21A	brown	-0.5800379
ENSG00000056487	PHF21B	turquoise	0.51235693
ENSG00000040633	PHF23	grey	0.04663675
ENSG00000118482	PHF3	lightgreen	0.77577879
ENSG00000100410	PHF5A	yellow	0.40362208
ENSG00000156531	PHF6	grey	-0.0006234
ENSG00000010318	PHF7	grey	-0.0281477
ENSG00000172943	PHF8	blue	-0.3867384
ENSG00000092621	PHGDH	turquoise	0.59844431
ENSG00000146247	PHIP	lightgreen	0.64405498
ENSG00000067177	PHKA1	turquoise	0.76904478
ENSG00000044446	PHKA2	lightcyan	-0.657114
ENSG00000102893	PHKB	green	-0.5346344
ENSG00000164776	PHKG1	grey	0.02176586
ENSG00000156873	PHKG2	yellow	-0.61614
ENSG00000019144	PHLDB1	yellow	0.83286591
ENSG00000144824	PHLDB2	tan	0.49386886
ENSG00000176531	PHLDB3	grey	0.1280438

ENSG00000040199	PHLPP2	yellow	-0.6173986
ENSG00000144362	PHOSPHO2	grey	0.13602199
ENSG00000054148	PHPT1	greenyellow	0.6180592
ENSG00000070047	PHRF1	lightcyan	-0.7411398
ENSG00000116793	PHTF1	brown	0.55710474
ENSG00000006576	PHTF2	grey	-0.0236932
ENSG00000107537	PHYH	turquoise	0.66213907
ENSG00000165443	PHYHIPL	turquoise	0.46001035
ENSG00000164530	PI16	grey	-0.1713564
ENSG00000155252	PI4K2A	blue	0.38613701
ENSG00000038210	PI4K2B	turquoise	0.51063274
ENSG00000241973	PI4KA	blue	0.84809687
ENSG00000183506	PI4KAP2	grey	-0.2424706
ENSG00000143393	PI4KB	grey	-0.0119653
ENSG00000139200	PIANP	darkorange	0.80071987
ENSG00000033800	PIAS1	turquoise	-0.4117375
ENSG00000131788	PIAS3	grey	0.07190413
ENSG00000105229	PIAS4	yellow	0.66454224
ENSG00000083535	PIBF1	grey	0.0962482
ENSG00000073921	PICALM	yellow	0.79591428
ENSG00000100151	PICK1	lightcyan	-0.6548528
ENSG00000153823	PID1	red	0.76504914
ENSG00000177595	PIDD	turquoise	-0.3576814
ENSG00000103335	PIEZO1	blue	-0.6024402
ENSG00000154864	PIEZO2	yellow	0.55354605
ENSG00000165195	PIGA	blue	-0.5293217
ENSG00000069943	PIGB	grey	0.08680503
ENSG00000213713	PIGCP1	grey	0.06205771
ENSG00000151665	PIGF	grey	0.12140349
ENSG00000234881	PIGFP2	grey	0.46407873
ENSG00000174227	PIGG	brown	0.5161447
ENSG00000100564	PIGH	grey	0.1984092
ENSG00000142892	PIGK	grey	-0.1477301
ENSG00000143315	PIGM	grey	0.06247823
ENSG00000197563	PIGN	grey	-0.0651461
ENSG00000165282	PIGO	grey	-0.0014759
ENSG00000185808	PIGP	brown	-0.5118213
ENSG00000007541	PIGQ	blue	0.49023409
ENSG00000087111	PIGS	grey	-0.1550513
ENSG00000124155	PIGT	floralwhite	0.66350136
ENSG00000101464	PIGU	floralwhite	0.64287667
ENSG00000224437	PIGUP1	grey	0.45850072
ENSG00000060642	PIGV	turquoise	0.67798173
ENSG00000184886	PIGW	grey	0.18923745
ENSG00000163964	PIGX	brown	0.33106686
ENSG00000119227	PIGZ	grey	0.12550134
ENSG00000268977	PIH1	grey	0.43871624
ENSG00000104872	PIH1D1	green	-0.6437812

ENSG00000150773	PIH1D2	grey	0.02265067
ENSG00000155629	PIK3AP1	royalblue	0.88801611
ENSG00000011405	PIK3C2A	turquoise	0.58841683
ENSG00000133056	PIK3C2B	yellow	0.76130135
ENSG00000078142	PIK3C3	grey	-0.0087758
ENSG00000121879	PIK3CA	brown	-0.5903104
ENSG00000051382	PIK3CB	turquoise	-0.6068703
ENSG00000171608	PIK3CD	grey	-0.0299561
ENSG00000100100	PIK3IP1	blue	-0.631293
ENSG00000145675	PIK3R1	blue	-0.5364742
ENSG00000117461	PIK3R3	pink	0.52563415
ENSG00000196455	PIK3R4	grey	0.14613474
ENSG00000174083	PIK3R6	black	0.76667362
ENSG00000115020	PIKFYVE	grey	0.43576363
ENSG00000085514	PILRA	grey	-0.2082672
ENSG00000121716	PILRB	grey	-0.103231
ENSG00000137193	PIM1	pink	0.8523608
ENSG00000102096	PIM2	green	-0.6070886
ENSG00000198355	PIM3	blue	-0.5108352
ENSG00000127445	PIN1	blue	0.69369746
ENSG00000229359	PIN1P1	cyan	-0.4300246
ENSG00000102309	PIN4	greenyellow	0.46719897
ENSG00000227973	PIN4P1	grey	0.35423348
ENSG00000158828	PINK1	greenyellow	-0.5741806
ENSG00000117242	PINK1-AS	grey	0.36298715
ENSG00000234465	PINLYP	grey	-0.1536083
ENSG00000254093	PINX1	grey	0.21925678
ENSG00000150867	PIP4K2A	yellow	0.85127657
ENSG00000141720	PIP4K2B	grey	0.01320204
ENSG00000166908	PIP4K2C	brown	0.58856046
ENSG00000143398	PIP5K1A	grey	0.04287161
ENSG00000107242	PIP5K1B	grey	-0.2478331
ENSG00000186111	PIP5K1C	grey	0.32749623
ENSG00000179761	PIPOX	yellowgreen	0.79650331
ENSG00000087842	PIR	grey	-0.0513663
ENSG00000241878	PISD	yellow	-0.5264196
ENSG00000057757	PITHD1	brown	0.72262889
ENSG00000174238	PITPNA	yellow	-0.7111439
ENSG00000236618	PITPNA-AS1	grey	0.29818157
ENSG00000180957	PITPNB	turquoise	-0.5349361
ENSG00000154217	PITPNC1	red	0.66092669
ENSG00000090975	PITPNM2	blue	0.46823972
ENSG00000091622	PITPNM3	purple	0.79000862
ENSG00000107959	PITRM1	turquoise	-0.4128993
ENSG00000237399	PITRM1-AS1	grey	0.44591416
ENSG00000134627	PIWIL4	grey	0.21344701
ENSG00000181191	PJA1	brown	0.81734379
ENSG00000198961	PJA2	turquoise	-0.4741243

ENSG00000008710	PKD1	turquoise	-0.4976345
ENSG00000158683	PKD1L1	grey	0.18847492
ENSG00000250251	PKD1P6	grey	0.20117441
ENSG00000118762	PKD2	blue	-0.7005105
ENSG00000171033	PKIA	grey	-0.5298316
ENSG00000135549	PKIB	grey	0.00440682
ENSG00000168734	PKIG	grey	0.06878896
ENSG00000067225	PKM	brown	0.59687399
ENSG00000220563	PKMP3	grey	0.15202921
ENSG00000065243	PKN2	lightgreen	0.74176421
ENSG00000165495	PKNOX2	grey	-0.0110797
ENSG00000057294	PKP2	purple	0.68645625
ENSG00000144283	PKP4	green	0.85826919
ENSG00000123739	PLA2G12A	brown	-0.4897235
ENSG00000103066	PLA2G15	grey	0.16121702
ENSG00000176485	PLA2G16	green	0.90135847
ENSG00000116711	PLA2G4A	grey	-0.0230096
ENSG00000105499	PLA2G4C	grey	-0.1870725
ENSG00000127472	PLA2G5	turquoise	0.56766644
ENSG00000184381	PLA2G6	floralwhite	0.67109412
ENSG00000146070	PLA2G7	blue	0.4337639
ENSG00000153246	PLA2R1	purple	-0.5592265
ENSG00000137055	PLAA	darkred	0.5788039
ENSG00000173261	PLAC8L1	grey	-0.0498245
ENSG00000189129	PLAC9	grey	0.10443446
ENSG00000181690	PLAG1	red	-0.5428543
ENSG00000118495	PLAGL1	grey	0.18069077
ENSG00000126003	PLAGL2	blue	-0.6731355
ENSG00000104368	PLAT	tan	0.6210908
ENSG00000011422	PLAUR	grey	0.2464407
ENSG00000163803	PLB1	turquoise	-0.552435
ENSG00000121316	PLBD1	grey	-0.0813293
ENSG00000151176	PLBD2	grey	0.1694142
ENSG00000182621	PLCB1	magenta	0.65425196
ENSG00000225479	PLCB1-IT1	black	0.6265483
ENSG00000149782	PLCB3	grey	-0.1420319
ENSG00000101333	PLCB4	grey	0.23536331
ENSG00000187091	PLCD1	turquoise	0.71829126
ENSG00000161714	PLCD3	turquoise	0.67197625
ENSG00000115556	PLCD4	grey	0.16633573
ENSG00000138193	PLCE1	pink	0.43584716
ENSG00000124181	PLCG1	yellow	0.402382
ENSG00000197943	PLCG2	grey	-0.0342617
ENSG00000114805	PLCH1	grey	0.12971791
ENSG00000149527	PLCH2	grey	-0.0888055
ENSG00000115896	PLCL1	yellow	0.79031895
ENSG00000154822	PLCL2	grey	0.00049491
ENSG00000226441	PLCL2-AS1	grey	0.15330535

ENSG00000182378	PLCXD1	grey	0.14113324
ENSG00000075651	PLD1	yellow	0.88653769
ENSG00000129219	PLD2	turquoise	0.41183251
ENSG00000105223	PLD3	blue	0.87884519
ENSG00000166428	PLD4	cyan	0.76929234
ENSG00000180287	PLD5	grey	-0.2226197
ENSG00000179598	PLD6	grey	-0.0001946
ENSG00000178209	PLEC	grey	-0.0416643
ENSG00000115956	PLEK	red	-0.5540907
ENSG00000116095	PLEKHA3	grey	0.05485666
ENSG00000105559	PLEKHA4	grey	-0.0662464
ENSG00000052126	PLEKHA5	turquoise	-0.4581542
ENSG00000143850	PLEKHA6	grey	0.22273467
ENSG00000166689	PLEKHA7	black	0.54173236
ENSG00000106086	PLEKHA8	grey	0.09776276
ENSG00000021300	PLEKHB1	green	0.77371307
ENSG00000115762	PLEKHB2	brown	0.59739937
ENSG00000175895	PLEKHF2	blue	-0.7281736
ENSG00000120278	PLEKHG1	magenta	-0.65975
ENSG00000126822	PLEKHG3	yellow	0.8001011
ENSG00000171680	PLEKHG5	blue	0.56997756
ENSG00000054690	PLEKHH1	yellow	0.88772456
ENSG00000152527	PLEKHH2	grey	-0.0510271
ENSG00000104886	PLEKHJ1	darkred	0.67237028
ENSG00000225190	PLEKHM1	grey	0.4294756
ENSG00000214176	PLEKHM1P	grey	0.22366483
ENSG00000116786	PLEKHM2	grey	-0.0480448
ENSG00000023902	PLEKHO1	grey	-0.4191167
ENSG00000241839	PLEKHO2	turquoise	0.67623225
ENSG00000188771	PLET1	grey	0.12247596
ENSG00000240935	PLGLA	grey	-0.0986466
ENSG00000183281	PLGLB1	brown	0.52284495
ENSG00000107020	PLGRKT	grey	0.0122544
ENSG00000166819	PLIN1	turquoise	0.485976
ENSG00000147872	PLIN2	blue	-0.652977
ENSG00000167676	PLIN4	turquoise	0.54748764
ENSG00000214456	PLIN5	turquoise	0.52626109
ENSG00000166851	PLK1	grey	0.04636826
ENSG00000088970	PLK1S1	turquoise	-0.596496
ENSG00000173846	PLK3	grey	0.07675037
ENSG00000185988	PLK5	grey	0.00140874
ENSG00000102934	PLLP	yellow	0.84446067
ENSG00000198523	PLN	grey	-0.0524369
ENSG00000152952	PLOD2	turquoise	0.69370852
ENSG00000123560	PLP1	yellow	0.77281202
ENSG00000102007	PLP2	pink	0.50431837
ENSG00000102024	PLS3	green	0.72110351
ENSG00000188313	PLSCR1	pink	0.91208894

ENSG00000114698	PLSCR4	turquoise	0.72046705
ENSG00000100979	PLTP	turquoise	0.67574537
ENSG00000161381	PLXDC1	grey	-0.0235394
ENSG00000120594	PLXDC2	cyan	0.5969549
ENSG00000076356	PLXNA2	yellow	-0.3379709
ENSG00000130827	PLXNA3	grey	-0.039283
ENSG00000196576	PLXNB2	turquoise	0.52407193
ENSG00000198753	PLXNB3	yellow	0.74419257
ENSG00000136040	PLXNC1	blue	0.6934229
ENSG00000004399	PLXND1	yellow	0.51748358
ENSG00000162877	PM20D1	grey	0.01824357
ENSG00000146281	PM20D2	brown	-0.3859973
ENSG00000141682	PMAIP1	tan	0.47731431
ENSG00000124225	PMEPA1	black	0.71549564
ENSG00000160783	PMF1	grey	-0.0518124
ENSG00000140464	PML	pink	0.82440965
ENSG00000100417	PMM1	yellow	-0.6555292
ENSG00000140650	PMM2	grey	0.06328327
ENSG00000147588	PMP2	turquoise	0.72573677
ENSG00000109099	PMP22	yellow	0.76403676
ENSG00000165688	PMPCA	green	-0.5973851
ENSG00000105819	PMPCB	darkred	0.63538022
ENSG00000064933	PMS1	grey	-0.1548494
ENSG00000122512	PMS2	blue	0.74722035
ENSG00000187953	PMS2CL	blue	0.49144473
ENSG00000078319	PMS2P1	grey	0.04911652
ENSG00000127957	PMS2P3	turquoise	-0.4510584
ENSG00000067601	PMS2P4	grey	-0.1010008
ENSG00000123965	PMS2P5	blue	0.48002073
ENSG00000130822	PNCK	magenta	0.50944788
ENSG00000132424	PNISR	lightgreen	0.72870875
ENSG00000127838	PNKD	blue	0.27043061
ENSG00000039650	PNKP	grey	-0.0092863
ENSG00000146453	PNLDC1	grey	-0.0593567
ENSG00000240694	PNMA2	blue	0.60030404
ENSG00000183837	PNMA3	blue	0.53977586
ENSG00000204851	PNMAL2	purple	0.78716402
ENSG00000141744	PNMT	grey	0.00500068
ENSG00000100941	PNN	darkgrey	0.87202537
ENSG00000115946	PNO1	grey	0.09422266
ENSG00000198805	PNP	pink	0.6510707
ENSG00000177666	PNPLA2	grey	0.11103336
ENSG00000006757	PNPLA4	grey	-0.0459047
ENSG00000100341	PNPLA5	grey	-0.0440371
ENSG00000032444	PNPLA6	turquoise	-0.5565341
ENSG00000130653	PNPLA7	turquoise	-0.4834696
ENSG00000135241	PNPLA8	turquoise	0.69896152
ENSG00000138035	PNPT1	yellow	0.4990146

ENSG00000229241	PNPT1P1	grey	0.34729931
ENSG00000146278	PNRC1	brown	-0.4946201
ENSG00000215700	PNRC2	grey	-0.0210823
ENSG00000139323	POC1B	brown	-0.4567366
ENSG00000152359	POC5	grey	0.07466506
ENSG00000174348	PODN	tan	0.58618711
ENSG00000128567	PODXL	tan	0.89110415
ENSG00000114631	PODXL2	blue	0.4672115
ENSG00000101346	POFUT1	turquoise	0.44836932
ENSG00000186866	POFUT2	green	-0.4198273
ENSG00000143157	POGK	green	0.84752756
ENSG00000163389	POGLUT1	grey	-0.1250391
ENSG00000143442	POGZ	grey	-0.1921139
ENSG00000101868	POLA1	green	0.69453725
ENSG00000014138	POLA2	turquoise	-0.5061175
ENSG00000070501	POLB	brown	0.61393178
ENSG00000106628	POLD2	brown	0.37240413
ENSG00000077514	POLD3	grey	0.04056725
ENSG00000175482	POLD4	grey	0.22650894
ENSG00000004142	POLDIP2	turquoise	0.41688494
ENSG00000100479	POLE2	grey	0.10920613
ENSG00000148229	POLE3	grey	-0.0180255
ENSG00000115350	POLE4	brown	0.710143
ENSG00000140521	POLG	lightcyan	-0.5831122
ENSG00000256525	POLG2	grey	-0.1423505
ENSG00000170734	POLH	grey	0.20263573
ENSG00000232970	POLHP1	grey	0.49351392
ENSG00000101751	POLI	turquoise	0.40464017
ENSG00000122008	POLK	yellow	0.47067692
ENSG00000166169	POLL	blue	0.4964805
ENSG00000122678	POLM	grey	0.18295816
ENSG00000130997	POLN	grey	0.22419947
ENSG00000068654	POLR1A	green	-0.5789163
ENSG00000125630	POLR1B	darkred	0.61581139
ENSG00000171453	POLR1C	grey	0.01262138
ENSG00000186184	POLR1D	grey	-0.2035015
ENSG00000137054	POLR1E	grey	-0.1154979
ENSG00000181222	POLR2A	grey	0.04659612
ENSG00000102978	POLR2C	brown	0.41420631
ENSG00000144231	POLR2D	grey	0.20867632
ENSG00000099817	POLR2E	brown	0.50643728
ENSG00000100142	POLR2F	greenyellow	0.49141534
ENSG00000168002	POLR2G	grey	-0.0925579
ENSG00000163882	POLR2H	grey	-0.0076981
ENSG00000105258	POLR2I	greenyellow	0.63480893
ENSG00000005075	POLR2J	grey	0.04509169
ENSG00000168255	POLR2J3	grey	0.13402735
ENSG00000214783	POLR2J4	grey	0.23680961

ENSG00000147669	POLR2K	grey	-0.1469735
ENSG00000258284	POLR2KP1	grey	0.28965827
ENSG00000177700	POLR2L	grey	-0.0375065
ENSG00000255529	POLR2M	brown	-0.7929484
ENSG00000148606	POLR3A	brown	0.73815147
ENSG00000013503	POLR3B	grey60	0.64082851
ENSG00000186141	POLR3C	brown	0.4260446
ENSG00000168495	POLR3D	green	0.54275774
ENSG00000214626	POLR3DP1	grey	0.31025194
ENSG00000058600	POLR3E	grey	-0.0239522
ENSG00000132664	POLR3F	yellow	-0.620773
ENSG00000113356	POLR3G	bisque4	0.73899224
ENSG00000121851	POLR3GL	mediumpurp	0.70163644
ENSG00000100413	POLR3H	grey	0.22890472
ENSG00000161980	POLR3K	yellow	-0.5587462
ENSG00000099821	POLRMT	brown	0.66376001
ENSG00000196313	POM121	grey	0.00272235
ENSG00000135213	POM121C	brown	0.39506955
ENSG00000115138	POMC	grey	0.22968806
ENSG00000085998	POMGNT1	grey	-0.0947114
ENSG00000144647	POMGNT2	magenta	-0.3726657
ENSG00000185900	POMK	turquoise	-0.5382459
ENSG00000132963	POMP	salmon	0.47730655
ENSG00000130714	POMT1	brown	0.67749147
ENSG00000009830	POMT2	yellow	0.34787223
ENSG00000146707	POMZP3	grey	0.00230324
ENSG00000105854	PON2	turquoise	0.76571786
ENSG00000105852	PON3	grey	0.26283367
ENSG00000104356	POP1	yellow	-0.3452605
ENSG00000105171	POP4	green	-0.7202885
ENSG00000167272	POP5	grey	0.012998
ENSG00000172336	POP7	yellow	-0.6354461
ENSG00000121577	POPDC2	grey	0.10938794
ENSG00000132429	POPDC3	grey	-0.1255097
ENSG00000102312	PORCN	grey	0.01807703
ENSG00000128513	POT1	grey	0.01124539
ENSG00000224897	POT1-AS1	grey	0.03352913
ENSG00000143190	POU2F1	turquoise	0.53169197
ENSG00000028277	POU2F2	grey	-0.4039937
ENSG00000185668	POU3F1	grey	-0.0023599
ENSG00000184486	POU3F2	turquoise	0.66528224
ENSG00000198914	POU3F3	turquoise	0.52994387
ENSG00000196767	POU3F4	grey	-0.2164235
ENSG00000184271	POU6F1	grey	-0.0228246
ENSG00000180817	PPA1	salmon	0.73457388
ENSG00000138777	PPA2	grey	0.22117648
ENSG00000067113	PPAP2A	turquoise	0.58711455
ENSG00000162407	PPAP2B	turquoise	0.80928951

ENSG00000141934	PPAP2C	yellow	0.78951956
ENSG00000203805	PPAPDC1A	turquoise	0.37361444
ENSG00000147535	PPAPDC1B	grey	0.05259142
ENSG00000160539	PPAPDC3	grey	0.14581846
ENSG00000186951	PPARA	turquoise	0.90762238
ENSG00000132170	PPARG	yellow	-0.4916767
ENSG00000109819	PPARGC1A	turquoise	0.29281424
ENSG00000155846	PPARGC1B	pink	-0.5380856
ENSG00000128059	PPAT	turquoise	0.65708768
ENSG00000138621	PPCDC	grey	0.34147704
ENSG00000127125	PPCS	mediumpurp	0.79292888
ENSG00000125534	PPDPF	magenta	-0.5275214
ENSG00000131626	PPFIA1	turquoise	0.58846391
ENSG00000139220	PPFIA2	blue	0.62858954
ENSG00000177380	PPFIA3	turquoise	-0.5471105
ENSG00000143847	PPFIA4	grey	-0.1175886
ENSG00000110841	PPFIBP1	tan	0.64257853
ENSG00000166387	PPFIBP2	yellow	0.70523355
ENSG00000134283	PPHLN1	green	-0.7527053
ENSG00000196262	PPIA	brown	0.85661812
ENSG00000228169	PPIAP19	grey	0.24812723
ENSG00000227379	PPIAP2	grey	0.18776234
ENSG00000226038	PPIAP21	grey	0.11602434
ENSG00000198618	PPIAP22	grey	0.23912314
ENSG00000166794	PPIB	turquoise	0.45315153
ENSG00000168938	PPIC	turquoise	0.60900794
ENSG00000084072	PPIE	grey	-0.1592445
ENSG00000243970	PPIEL	grey	-0.0262688
ENSG00000108179	PPIF	blue	-0.6131949
ENSG00000138398	PPIG	lightgreen	0.70237028
ENSG00000171960	PPIH	turquoise	-0.4784846
ENSG00000137168	PPIL1	green	-0.5319865
ENSG00000100023	PPIL2	grey	0.16309576
ENSG00000240344	PPIL3	grey	0.01159179
ENSG00000131013	PPIL4	bisque4	0.62172497
ENSG00000168781	PPIP5K1	blue	0.54155648
ENSG00000145725	PPIP5K2	grey	-0.1302063
ENSG00000118898	PPL	yellow	-0.3676288
ENSG00000100614	PPM1A	turquoise	0.75926363
ENSG00000250483	PPM1AP1	grey	0.39313976
ENSG00000138032	PPM1B	blue	-0.7280398
ENSG00000170836	PPM1D	grey	0.01251355
ENSG00000175175	PPM1E	grey	0.27275407
ENSG00000100034	PPM1F	black	-0.5291834
ENSG00000115241	PPM1G	grey	0.07723732
ENSG00000111110	PPM1H	grey	0.07816561
ENSG00000163644	PPM1K	turquoise	0.37096499
ENSG00000163590	PPM1L	red	0.73398044

ENSG00000164088	PPM1M	grey	-0.4099636
ENSG00000213889	PPM1N	grey	-0.0057659
ENSG00000214517	PPME1	blue	0.84646766
ENSG00000143224	PPOX	blue	0.62339214
ENSG00000172531	PPP1CA	grey	-0.0899557
ENSG00000213639	PPP1CB	brown	-0.5459916
ENSG00000186298	PPP1CC	turquoise	0.69264287
ENSG00000204569	PPP1R10	grey	-0.005025
ENSG00000204619	PPP1R11	grey	0.02796772
ENSG00000231765	PPP1R11P2	grey	0.19662019
ENSG00000058272	PPP1R12A	black	-0.6770224
ENSG00000077157	PPP1R12B	grey	-0.235014
ENSG00000125503	PPP1R12C	turquoise	-0.4261332
ENSG00000088808	PPP1R13B	blue	0.65422224
ENSG00000104881	PPP1R13L	pink	0.51520462
ENSG00000167641	PPP1R14A	yellow	0.74442791
ENSG00000173457	PPP1R14B	grey	0.13097589
ENSG00000198729	PPP1R14C	magenta	0.33832714
ENSG00000158615	PPP1R15B	blue	-0.5644621
ENSG00000160972	PPP1R16A	yellow	-0.5969421
ENSG00000101445	PPP1R16B	magenta	0.75100802
ENSG00000146112	PPP1R18	royalblue	0.73824514
ENSG00000135447	PPP1R1A	magenta	0.75308423
ENSG00000131771	PPP1R1B	magenta	0.78843991
ENSG00000184203	PPP1R2	blue	0.59327065
ENSG00000162869	PPP1R21	red	-0.5348454
ENSG00000196422	PPP1R26	turquoise	0.58652858
ENSG00000160813	PPP1R35	yellow	-0.5403999
ENSG00000104866	PPP1R37	turquoise	-0.6455566
ENSG00000173281	PPP1R3B	blue	-0.5128109
ENSG00000119938	PPP1R3C	brown	-0.5454098
ENSG00000132825	PPP1R3D	turquoise	0.61973685
ENSG00000235194	PPP1R3E	darkred	-0.599889
ENSG00000049769	PPP1R3F	turquoise	-0.6301645
ENSG00000219607	PPP1R3G	turquoise	0.57566642
ENSG00000117751	PPP1R8	lightgreen	0.53931904
ENSG00000224986	PPP1R8P1	grey	0.45033485
ENSG00000108819	PPP1R9B	turquoise	-0.4504976
ENSG00000113575	PPP2CA	green	-0.5597761
ENSG00000104695	PPP2CB	turquoise	0.69240973
ENSG00000105568	PPP2R1A	blue	0.74141194
ENSG00000137713	PPP2R1B	blue	-0.5061507
ENSG00000221914	PPP2R2A	red	-0.7158741
ENSG00000156475	PPP2R2B	darkmagenta	0.70786263
ENSG00000249553	PPP2R2B-IT1	yellow	0.70242738
ENSG00000074211	PPP2R2C	black	0.56312152
ENSG00000175470	PPP2R2D	brown	0.67128766
ENSG00000073711	PPP2R3A	yellow	0.62312789

ENSG00000167393	PPP2R3B	grey	-0.1619913
ENSG00000092020	PPP2R3C	grey	0.24316591
ENSG00000119383	PPP2R4	brown	0.70782147
ENSG00000066027	PPP2R5A	grey	0.13333056
ENSG00000068971	PPP2R5B	grey	0.01732372
ENSG00000078304	PPP2R5C	greenyellow	0.51006451
ENSG00000112640	PPP2R5D	blue	0.7006097
ENSG00000154001	PPP2R5E	grey	-0.318918
ENSG00000107758	PPP3CB	blue	0.76504713
ENSG00000120910	PPP3CC	grey	0.36404373
ENSG00000221823	PPP3R1	magenta	0.44850033
ENSG00000149923	PPP4C	grey	0.03805469
ENSG00000154845	PPP4R1	blue	-0.6584766
ENSG00000163605	PPP4R2	blue	-0.7095289
ENSG00000119698	PPP4R4	blue	0.7997231
ENSG00000011485	PPP5C	yellow	-0.5098296
ENSG00000230510	PPP5D1	grey	0.24370627
ENSG00000119414	PPP6C	turquoise	0.47203113
ENSG00000105063	PPP6R1	red	-0.7217317
ENSG00000100239	PPP6R2	turquoise	-0.6756089
ENSG00000110075	PPP6R3	blue	-0.6576514
ENSG00000148840	PPRC1	turquoise	-0.4368561
ENSG00000131238	PPT1	grey	-0.0410845
ENSG00000221988	PPT2	grey	0.23042975
ENSG00000196850	PPTC7	brown	-0.3691481
ENSG00000102103	PQBP1	brown	0.72400075
ENSG00000122490	PQLC1	turquoise	-0.6908865
ENSG00000040487	PQLC2	grey	0.22250627
ENSG00000162976	PQLC3	brown	-0.5025282
ENSG00000135617	PRADC1	grey	0.20499806
ENSG00000243279	PRAF2	grey	0.0489652
ENSG00000133246	PRAM1	royalblue	0.67569256
ENSG00000121335	PRB2	grey	0.23574746
ENSG00000198901	PRC1	blue	0.42234549
ENSG00000143294	PRCC	turquoise	-0.7159781
ENSG00000214140	PRCD	darkred	-0.6402056
ENSG00000137509	PRCP	turquoise	0.5982091
ENSG00000057657	PRDM1	grey	0.22221945
ENSG00000170325	PRDM10	grey	0.15951943
ENSG00000019485	PRDM11	grey	0.2827503
ENSG00000141956	PRDM15	grey	0.17786058
ENSG00000142611	PRDM16	turquoise	0.66453193
ENSG00000116731	PRDM2	grey	-0.2645015
ENSG00000110851	PRDM4	grey	0.1997848
ENSG00000117450	PRDX1	turquoise	0.51000093
ENSG00000167815	PRDX2	red	0.68009599
ENSG00000249140	PRDX2P3	grey	0.34211729
ENSG00000165672	PRDX3	red	0.62806426

ENSG00000229598	PRDX3P1	cyan	-0.4890898
ENSG00000239926	PRDX3P4	grey	0.46238298
ENSG00000123131	PRDX4	grey	-0.1241034
ENSG00000126432	PRDX5	yellow	-0.7222237
ENSG00000117592	PRDX6	turquoise	0.6976714
ENSG00000138073	PREB	grey	0.28421147
ENSG00000268805	PRED57	grey	0.38129827
ENSG00000267883	PRED60	grey	0.4881426
ENSG00000169230	PRELID1	grey	0.37545749
ENSG00000259592	PRELID1P4	grey	0.00438643
ENSG00000188783	PRELP	red	0.55497509
ENSG00000085377	PREP	grey60	0.395752
ENSG00000138078	PREPL	blue	0.78785083
ENSG00000124126	PREX1	yellow	0.90566308
ENSG00000046889	PREX2	turquoise	0.78848309
ENSG00000116690	PRG4	turquoise	0.44019363
ENSG00000134551	PRH2	grey	0.31809402
ENSG00000139174	PRICKLE1	yellow	-0.6389221
ENSG00000163637	PRICKLE2	turquoise	0.52824035
ENSG00000241101	PRICKLE2-AS2	grey	0.21183046
ENSG00000226017	PRICKLE2-AS3	grey	0.47151744
ENSG00000124593	PRICKLE4	grey	0.2282289
ENSG00000198056	PRIM1	grey	-0.1061978
ENSG00000146143	PRIM2	grey	0.1963458
ENSG00000175785	PRIMA1	yellow	0.81484873
ENSG00000164306	PRIMPOL	grey	0.23367
ENSG00000132356	PRKAA1	grey	0.08319257
ENSG00000162409	PRKAA2	blue	0.47449899
ENSG00000111725	PRKAB1	grey	0.31643163
ENSG00000131791	PRKAB2	green	0.73065667
ENSG00000072062	PRKACA	blue	0.4720597
ENSG00000181929	PRKAG1	blue	0.57591884
ENSG00000106617	PRKAG2	greenyellow	0.4468469
ENSG00000108946	PRKAR1A	red	0.64831757
ENSG00000233401	PRKAR1AP	grey	0.32367768
ENSG00000188191	PRKAR1B	blue	0.83819488
ENSG00000114302	PRKAR2A	magenta	-0.5311315
ENSG00000154229	PRKCA	turquoise	0.69539136
ENSG00000166501	PRKCB	magenta	0.78649992
ENSG00000170955	PRKCDBP	grey	0.02347658
ENSG00000171132	PRKCE	blue	0.60637258
ENSG00000126583	PRKCG	purple	0.79959166
ENSG00000027075	PRKCH	grey	-0.0015382
ENSG00000163558	PRKCI	purple	0.58564491
ENSG00000065675	PRKCQ	greenyellow	-0.5215634
ENSG00000237943	PRKCQ-AS1	yellow	0.74625296
ENSG00000130175	PRKCSH	grey	-0.0478852
ENSG00000067606	PRKCZ	grey60	0.54819497

ENSG00000184304	PRKD1	black	0.54394578
ENSG00000105287	PRKD2	grey	0.19508703
ENSG00000115825	PRKD3	turquoise	0.69853771
ENSG00000253729	PRKDC	brown	0.63453951
ENSG00000185532	PRKG1	red	0.61940844
ENSG00000138669	PRKG2	purple	0.58793944
ENSG00000180228	PRKRA	yellow	-0.4076597
ENSG00000128563	PRKRIP1	blue	0.55557127
ENSG00000137492	PRKRIR	grey60	-0.3934783
ENSG00000183943	PRKX	brown	-0.3609012
ENSG00000259205	PRKXP1	grey	0.09538931
ENSG00000113494	PRLR	purple	0.78613702
ENSG00000126457	PRMT1	blue	0.59722818
ENSG00000164169	PRMT10	grey	0.15250961
ENSG00000160310	PRMT2	greenyellow	0.60984718
ENSG00000185238	PRMT3	grey	-0.143592
ENSG00000100462	PRMT5	grey	0.09370446
ENSG00000198890	PRMT6	grey	-0.1081783
ENSG00000132600	PRMT7	grey60	0.71402319
ENSG00000111218	PRMT8	turquoise	-0.6157623
ENSG00000171867	PRNP	turquoise	0.74118814
ENSG00000167525	PROCA1	grey	-0.1449868
ENSG00000101000	PROCR	tan	0.50169833
ENSG00000100033	PRODH	turquoise	0.5335566
ENSG00000007062	PROM1	tan	0.59958334
ENSG00000155066	PROM2	grey	-0.2303848
ENSG00000162997	PRORSD1P	grey	0.17761378
ENSG00000184500	PROS1	grey	0.05032717
ENSG00000147471	PROSC	grey	-0.2137271
ENSG00000120685	PROSER1	grey	0.04407248
ENSG00000225778	PROSER2-AS1	grey	0.1931296
ENSG00000117707	PROX1	salmon	-0.4965998
ENSG00000230461	PROX1-AS1	green	0.58678162
ENSG00000119608	PROX2	grey	0.14447841
ENSG00000165630	PRPF18	grey	0.0953717
ENSG00000110107	PRPF19	turquoise	-0.7265667
ENSG00000117360	PRPF3	yellowgreen	-0.597668
ENSG00000105618	PRPF31	lightcyan	-0.5530107
ENSG00000134748	PRPF38A	brown	-0.8233624
ENSG00000134186	PRPF38B	darkgrey	0.79347531
ENSG00000185246	PRPF39	grey	-0.2521668
ENSG00000136875	PRPF4	grey	0.07551993
ENSG00000196504	PRPF40A	brown	-0.5277179
ENSG00000110844	PRPF40B	grey	0.29784788
ENSG00000112739	PRPF4B	lightgreen	0.67150198
ENSG00000101161	PRPF6	grey	-0.0630105
ENSG00000174231	PRPF8	bisque4	-0.7167236
ENSG00000112619	PRPH2	grey	-0.0819262

ENSG00000232630	PRPS1P2	grey	0.21831573
ENSG00000101911	PRPS2	blue	0.45670298
ENSG00000161542	PRPSAP1	red	0.56521331
ENSG00000141127	PRPSAP2	grey	-0.2167765
ENSG00000068489	PRR11	grey	0.0793952
ENSG00000126464	PRR12	blue	0.54605457
ENSG00000205352	PRR13	grey	-0.1353462
ENSG00000156858	PRR14	grey	-0.011385
ENSG00000183530	PRR14L	grey	0.03282545
ENSG00000184838	PRR16	grey	-0.0069997
ENSG00000176381	PRR18	yellow	0.79034366
ENSG00000188368	PRR19	grey	0.18020785
ENSG00000257704	PRR24	grey	0.18553327
ENSG00000180525	PRR26	yellow	0.64678373
ENSG00000204576	PRR3	black	0.62770688
ENSG00000111215	PRR4	grey	0.00794755
ENSG00000186654	PRR5	turquoise	0.5453406
ENSG00000135362	PRR5L	yellow	0.77990421
ENSG00000131188	PRR7	grey	-0.1923015
ENSG00000246334	PRR7-AS1	grey	0.11734704
ENSG00000164244	PRRC1	turquoise	0.67217302
ENSG00000204469	PRRC2A	salmon	-0.7158522
ENSG00000130723	PRRC2B	brown	0.59570253
ENSG00000117523	PRRC2C	grey	-0.1022132
ENSG00000130962	PRRG1	yellow	0.81174925
ENSG00000130032	PRRG3	purple	0.75304588
ENSG00000204314	PRRT1	blue	0.66565665
ENSG00000167371	PRRT2	magenta	0.56597627
ENSG00000163704	PRRT3	blue	0.47907863
ENSG00000230082	PRRT3-AS1	grey	0.16683709
ENSG00000116132	PRRX1	turquoise	0.67840048
ENSG00000150687	PRSS23	grey	0.33353809
ENSG00000010438	PRSS3	blue	0.46669947
ENSG00000146250	PRSS35	turquoise	0.56064099
ENSG00000165076	PRSS37	grey	0.0814503
ENSG00000189099	PRSS48	grey	0.18211415
ENSG00000253649	PRSS51	grey	0.21819302
ENSG00000099256	PRTFDC1	yellow	0.56132748
ENSG00000143363	PRUNE	grey	0.13246919
ENSG00000106772	PRUNE2	yellow	0.77292603
ENSG00000197746	PSAP	salmon	0.83704027
ENSG00000135069	PSAT1	turquoise	0.79390841
ENSG00000146005	PSD2	turquoise	0.69457646
ENSG00000156011	PSD3	turquoise	-0.4606462
ENSG00000125637	PSD4	turquoise	0.44544993
ENSG00000080815	PSEN1	yellow	0.78240197
ENSG00000143801	PSEN2	grey	-0.0267411
ENSG00000205155	PSENEN	grey	-0.0108516

ENSG00000164985	PSIP1	lightgreen	0.44373019
ENSG00000159792	PSKH1	brown	-0.41858
ENSG00000129084	PSMA1	brown	0.81154466
ENSG00000106588	PSMA2	salmon	0.54603314
ENSG00000254582	PSMA2P1	grey	0.20671217
ENSG00000251234	PSMA2P2	grey	0.50209133
ENSG00000100567	PSMA3	salmon	0.81353395
ENSG00000041357	PSMA4	brown	0.60185746
ENSG00000143106	PSMA5	brown	0.84319172
ENSG00000100902	PSMA6	grey	0.3812313
ENSG00000101182	PSMA7	salmon	0.76059906
ENSG00000008018	PSMB1	brown	0.7667596
ENSG00000205220	PSMB10	grey	-0.1744439
ENSG00000108294	PSMB3	brown	0.79767192
ENSG00000159377	PSMB4	brown	0.68932566
ENSG00000100804	PSMB5	brown	0.5321268
ENSG00000142507	PSMB6	brown	0.8329511
ENSG00000136930	PSMB7	grey	0.06712825
ENSG00000204264	PSMB8	pink	0.6511525
ENSG00000240065	PSMB9	turquoise	0.47703612
ENSG00000100764	PSMC1	brown	0.57747559
ENSG00000241506	PSMC1P1	grey	0.39964968
ENSG00000161057	PSMC2	salmon	0.71642317
ENSG00000165916	PSMC3	grey60	0.70585493
ENSG00000131470	PSMC3IP	darkgrey	0.52962634
ENSG00000013275	PSMC4	brown	0.41519931
ENSG00000087191	PSMC5	green	-0.6177119
ENSG00000100519	PSMC6	salmon	0.7589976
ENSG00000173692	PSMD1	brown	0.75716623
ENSG00000228663	PSMD10P1	grey	-0.0970505
ENSG00000108671	PSMD11	grey	0.19789939
ENSG00000185627	PSMD13	brown	0.80006957
ENSG00000115233	PSMD14	darkred	0.68639608
ENSG00000175166	PSMD2	brown	0.78484092
ENSG00000108344	PSMD3	turquoise	-0.7530145
ENSG00000159352	PSMD4	brown	0.8629539
ENSG00000095261	PSMD5	grey	0.1832213
ENSG00000226752	PSMD5-AS1	grey	-0.0108826
ENSG00000239653	PSMD6-AS2	blue	-0.4501644
ENSG00000103035	PSMD7	salmon	0.77660803
ENSG00000099341	PSMD8	brown	0.71850666
ENSG00000092010	PSME1	blue	-0.3937675
ENSG00000100911	PSME2	grey	-0.3701668
ENSG00000131467	PSME3	turquoise	0.49899519
ENSG00000068878	PSME4	red	-0.72207
ENSG00000125818	PSMF1	turquoise	0.43024395
ENSG00000183527	PSMG1	green	-0.5550839
ENSG00000128789	PSMG2	grey	0.27712701

ENSG00000157778	PSMG3	salmon	0.48281443
ENSG00000230487	PSMG3-AS1	darkred	0.5214741
ENSG00000180822	PSMG4	red	-0.3640163
ENSG00000121390	PSPC1	turquoise	-0.6221167
ENSG00000146733	PSPH	grey	0.28745321
ENSG00000125650	PSPN	grey	0.13653596
ENSG00000179988	PSTK	brown	0.58902994
ENSG00000169403	PTAFR	cyan	0.86290165
ENSG00000188647	PTAR1	turquoise	0.84982025
ENSG00000011304	PTBP1	blue	-0.5645126
ENSG00000117569	PTBP2	grey	0.08702712
ENSG00000119314	PTBP3	magenta	0.5363876
ENSG00000106246	PTCD1	grey	0.34839941
ENSG00000049883	PTCD2	brown	0.45439652
ENSG00000132300	PTCD3	grey	0.22607678
ENSG00000117425	PTCH2	blue	0.54976049
ENSG00000165186	PTCHD1	grey	0.22713583
ENSG00000204624	PTCHD2	grey	0.03767304
ENSG00000224597	PTCHD3P1	brown	-0.6155208
ENSG00000259104	PTCSC3	grey	0.13898503
ENSG00000156471	PTDSS1	brown	0.78110187
ENSG00000174915	PTDSS2	lightcyan	-0.5229123
ENSG00000171862	PTEN	grey	-0.035516
ENSG00000183134	PTGDR2	grey	0.1606271
ENSG00000148334	PTGES2	brown	0.73640274
ENSG00000217643	PTGES3P2	grey	0.31007146
ENSG00000134247	PTGFRN	red	0.69594769
ENSG00000124212	PTGIS	grey	-0.2161564
ENSG00000106853	PTGR1	salmon	0.59109764
ENSG00000140043	PTGR2	turquoise	0.45523276
ENSG00000095303	PTGS1	cyan	0.90291216
ENSG00000160801	PTH1R	grey	0.16700407
ENSG00000169398	PTK2	yellow	0.77857307
ENSG00000187514	PTMA	yellow	0.66181794
ENSG00000228415	PTMAP1	grey	-0.09813
ENSG00000159335	PTMS	blue	0.45917551
ENSG00000105894	PTN	turquoise	0.4159153
ENSG00000104960	PTOV1	grey	-0.0867802
ENSG00000268006	PTOV1-AS1	grey	0.22678
ENSG00000112245	PTP4A1	grey	0.09373287
ENSG00000184007	PTP4A2	green	0.89955583
ENSG00000254481	PTP4A2P2	grey	0.28221073
ENSG00000184489	PTP4A3	grey	0.07629344
ENSG00000158079	PTPDC1	yellow	0.50011649
ENSG00000165996	PTPLA	grey	0.20651768
ENSG00000074696	PTPLAD1	lightgreen	-0.6502188
ENSG00000188921	PTPLAD2	grey	-0.1155609
ENSG00000206527	PTPLB	magenta	0.67629862

ENSG00000110536	PTPMT1	turquoise	0.71043385
ENSG00000196396	PTPN1	yellow	0.60528904
ENSG00000179295	PTPN11	lightgreen	0.80597978
ENSG00000127947	PTPN12	turquoise	0.44407471
ENSG00000163629	PTPN13	turquoise	0.44322907
ENSG00000152104	PTPN14	turquoise	0.68840078
ENSG00000072135	PTPN18	cyan	0.52730093
ENSG00000175354	PTPN2	grey	-0.0047534
ENSG00000070778	PTPN21	brown	-0.7492552
ENSG00000076201	PTPN23	floralwhite	0.54183147
ENSG00000228196	PTPN2P1	grey	0.07180231
ENSG00000224117	PTPN2P2	grey	0.37798615
ENSG00000088179	PTPN4	red	0.70793947
ENSG00000110786	PTPN5	magenta	0.72512203
ENSG00000143851	PTPN7	black	0.85816874
ENSG00000169410	PTPN9	grey	0.25704488
ENSG00000127329	PTPRB	tan	0.85984037
ENSG00000081237	PTPRC	royalblue	0.86879374
ENSG00000213402	PTPRCAP	grey	0.02969359
ENSG00000153707	PTPRD	yellow	0.76064263
ENSG00000142949	PTPRF	black	-0.7271967
ENSG00000144724	PTPRG	tan	0.5654249
ENSG00000241472	PTPRG-AS1	grey	0.21681699
ENSG00000149177	PTPRJ	darkorange	0.46874144
ENSG00000152894	PTPRK	yellow	0.69943603
ENSG00000173482	PTPRM	grey60	0.77352323
ENSG00000054356	PTPRN	blue	0.75982986
ENSG00000155093	PTPRN2	blue	0.68162354
ENSG00000151490	PTPRO	blue	0.72481941
ENSG00000153233	PTPRR	blue	0.49782611
ENSG00000105426	PTPRS	blue	0.52601902
ENSG00000196090	PTPRT	blue	0.33886503
ENSG00000060656	PTPRU	purple	0.55311812
ENSG00000106278	PTPRZ1	turquoise	0.52418475
ENSG00000177469	PTRF	green	0.74739279
ENSG00000141378	PTRH2	turquoise	-0.5884772
ENSG00000184924	PTRHD1	yellow	-0.7309622
ENSG00000150787	PTS	yellow	-0.4205957
ENSG00000183255	PTTG1IP	blue	-0.8154552
ENSG00000213005	PTTG3P	grey	0.0846219
ENSG00000258571	PTTG4P	grey	0.20647972
ENSG00000179950	PUF60	salmon	0.73138828
ENSG00000134644	PUM1	brown	-0.3676538
ENSG00000055917	PUM2	grey	-0.1697221
ENSG00000185129	PURA	green	0.86326621
ENSG00000146676	PURB	grey	-0.0971453
ENSG00000172733	PURG	turquoise	0.49439287
ENSG00000177192	PUS1	brown	0.6324345

ENSG00000162927	PUS10	grey	0.26868974
ENSG00000110060	PUS3	grey	0.02594354
ENSG00000091127	PUS7	black	0.62468108
ENSG00000169972	PUSL1	grey	0.06375422
ENSG00000100362	PVALB	grey	0.12326039
ENSG00000073008	PVR	grey	0.29241365
ENSG00000213413	PVRIG	yellow	0.47948614
ENSG00000110400	PVRL1	floralwhite	0.62726882
ENSG00000130202	PVRL2	turquoise	0.53554362
ENSG00000177707	PVRL3	turquoise	0.44874237
ENSG00000257151	PWAR6	grey	-0.2678739
ENSG00000136045	PWP1	darkred	0.64263632
ENSG00000241945	PWP2	lightcyan	-0.5751509
ENSG00000259905	PWRN1	turquoise	-0.5856399
ENSG00000260551	PWRN2	turquoise	-0.494166
ENSG00000260760	PWRN3	grey	0.00078801
ENSG00000260232	PWRN4	grey	0.16613061
ENSG00000170234	PWWP2A	green	0.72412712
ENSG00000171813	PWWP2B	grey60	0.69080735
ENSG00000168994	PXDC1	pink	0.75334888
ENSG00000130508	PXDN	grey	0.0535142
ENSG00000168297	PXK	yellow	0.85337898
ENSG00000176894	PXMP2	turquoise	0.49521062
ENSG00000101417	PXMP4	brown	-0.3951444
ENSG00000089159	PXN	pink	0.83127106
ENSG00000255857	PXN-AS1	grey	0.03700781
ENSG00000103490	PYCARD	cyan	0.71004398
ENSG00000183010	PYCR1	grey	0.32008761
ENSG00000143811	PYCR2	grey	-0.2488022
ENSG00000104524	PYCRL	yellow	-0.5431042
ENSG00000100994	PYGB	red	0.58955153
ENSG00000100504	PYGL	pink	0.63285995
ENSG00000068976	PYGM	turquoise	0.5234395
ENSG00000171016	PYGO1	turquoise	0.70349576
ENSG00000163348	PYGO2	turquoise	0.6583304
ENSG00000121350	PYROXD1	grey	0.27335705
ENSG00000119943	PYROXD2	grey	-0.1728927
ENSG00000145337	PYURF	grey	0.00372718
ENSG00000126838	PZP	grey	0.05045129
ENSG00000151552	QDPR	yellow	0.77984856
ENSG00000112531	QKI	turquoise	0.6218388
ENSG00000115828	QPCT	grey	0.15505506
ENSG00000011478	QPCTL	grey	0.18731246
ENSG00000103485	QPRT	yellowgreen	0.75819253
ENSG00000198218	QRICH1	blue	0.69502665
ENSG00000129646	QRICH2	grey	0.19726895
ENSG00000130348	QRSL1	grey	0.21064789
ENSG00000257957	QRSL1P3	grey	0.58786983

ENSG00000060749	QSER1	brown	-0.7014049
ENSG00000116260	QSOX1	turquoise	-0.5289063
ENSG00000165661	QSOX2	grey	-0.0333348
ENSG00000213339	QTRT1	blue	0.72284943
ENSG00000151576	QTRTD1	brown	0.36795509
ENSG00000104679	R3HCC1	grey	0.07589485
ENSG00000166024	R3HCC1L	yellow	0.47228165
ENSG00000179912	R3HDM2	grey	0.01736901
ENSG00000248735	R3HDM2P1	yellow	0.43974272
ENSG00000198858	R3HDM4	salmon	-0.5415734
ENSG00000084733	RAB10	blue	-0.6216333
ENSG00000185236	RAB11B	grey	0.05099029
ENSG00000269386	RAB11B-AS1	yellow	0.44323153
ENSG00000228492	RAB11FIP1P1	grey	0.19024133
ENSG00000107560	RAB11FIP2	grey	-0.1811966
ENSG00000090565	RAB11FIP3	grey	-0.1414344
ENSG00000131242	RAB11FIP4	darkgrey	0.58560346
ENSG00000135631	RAB11FIP5	grey	0.10517549
ENSG00000206418	RAB12	turquoise	0.42808417
ENSG00000143545	RAB13	turquoise	0.57251396
ENSG00000119396	RAB14	grey	0.04731462
ENSG00000139998	RAB15	blue	0.54143332
ENSG00000099246	RAB18	mediumpurp	0.85735714
ENSG00000138069	RAB1A	red	0.7767347
ENSG00000080371	RAB21	blue	-0.6277605
ENSG00000124209	RAB22A	turquoise	0.40012596
ENSG00000112210	RAB23	grey	0.00061242
ENSG00000169228	RAB24	grey	0.0410465
ENSG00000167964	RAB26	grey60	0.62236383
ENSG00000069974	RAB27A	pink	0.7226615
ENSG00000041353	RAB27B	purple	0.87584571
ENSG00000157869	RAB28	turquoise	0.68088022
ENSG00000104388	RAB2A	grey	-0.2597718
ENSG00000129472	RAB2B	blue	0.40844156
ENSG00000137502	RAB30	turquoise	0.83216221
ENSG00000246067	RAB30-AS1	grey	0.12653434
ENSG00000168461	RAB31	turquoise	0.77607994
ENSG00000118508	RAB32	royalblue	0.54741189
ENSG00000134594	RAB33A	turquoise	-0.4032813
ENSG00000172007	RAB33B	lightcyan	0.74950774
ENSG00000109113	RAB34	turquoise	0.47319013
ENSG00000111737	RAB35	grey	0.13675195
ENSG00000100228	RAB36	red	0.62525186
ENSG00000155961	RAB39B	blue	0.55753199
ENSG00000105649	RAB3A	blue	0.57751843
ENSG00000152932	RAB3C	blue	0.76719236
ENSG00000115839	RAB3GAP1	brown	0.59250503
ENSG00000118873	RAB3GAP2	green	-0.7425985

ENSG00000167994	RAB3IL1	royalblue	0.53243054
ENSG00000127328	RAB3IP	brown	-0.6926282
ENSG00000172476	RAB40A	brown	0.30067291
ENSG00000141542	RAB40B	lightgreen	0.61793165
ENSG00000197562	RAB40C	grey60	0.63461807
ENSG00000147127	RAB41	darkmagenta	-0.5241168
ENSG00000168118	RAB4A	darkmagenta	-0.7899151
ENSG00000167578	RAB4B	grey	0.14806865
ENSG00000144566	RAB5A	turquoise	0.65921017
ENSG00000111540	RAB5B	black	-0.5938391
ENSG00000108774	RAB5C	turquoise	0.68559404
ENSG00000213731	RAB5CP1	grey	0.24676995
ENSG00000175582	RAB6A	blue	0.28545228
ENSG00000154917	RAB6B	yellow	-0.8017934
ENSG00000222014	RAB6C	grey	0.0151852
ENSG00000225449	RAB6C-AS1	grey	-0.0213088
ENSG00000075785	RAB7A	grey	-0.0579099
ENSG00000117280	RAB7L1	turquoise	0.65713867
ENSG00000167461	RAB8A	turquoise	0.45493958
ENSG00000123595	RAB9A	turquoise	0.60461638
ENSG00000123570	RAB9B	blue	0.64018637
ENSG00000105404	RABAC1	brown	0.67465656
ENSG00000029725	RABEP1	darkgrey	0.62997764
ENSG00000177548	RABEP2	grey	0.0816293
ENSG00000136933	RABEPK	brown	0.80327684
ENSG00000011454	RABGAP1	grey	0.02294734
ENSG00000152061	RABGAP1L	blue	0.53104205
ENSG00000100949	RABGGTA	grey	-0.090231
ENSG00000137955	RABGGTB	grey	-0.0700887
ENSG00000183155	RABIF	grey	-0.084739
ENSG00000144134	RABL2A	turquoise	-0.5631098
ENSG00000144840	RABL3	turquoise	0.76266456
ENSG00000128581	RABL5	yellow	-0.6539838
ENSG00000196642	RABL6	turquoise	-0.7735822
ENSG00000136238	RAC1	green	0.72252478
ENSG00000249936	RAC1P2	turquoise	0.41788844
ENSG00000236101	RAC1P7	grey	0.14332008
ENSG00000113456	RAD1	blue	0.61169214
ENSG00000152942	RAD17	grey	-0.1754444
ENSG00000070950	RAD18	grey	-0.1644335
ENSG00000164754	RAD21	darkgrey	0.57204566
ENSG00000179262	RAD23A	grey	-0.2909708
ENSG00000119318	RAD23B	turquoise	-0.6078668
ENSG00000113522	RAD50	darkmagenta	0.59715377
ENSG00000245849	RAD51-AS1	grey	0.30059308
ENSG00000111247	RAD51AP1	grey	0.08199064
ENSG00000182185	RAD51B	grey	0.0727872
ENSG00000108384	RAD51C	grey	-0.1108626

ENSG00000185379	RAD51D	grey	-0.1659484
ENSG00000002016	RAD52	grey	-0.0865108
ENSG00000085999	RAD54L	grey60	0.60110233
ENSG00000164080	RAD54L2	black	0.65207666
ENSG00000172613	RAD9A	grey	-0.028241
ENSG00000157927	RADIL	black	0.47611513
ENSG00000101146	RAE1	brown	0.80403439
ENSG00000108557	RAI1	darkorange	0.44242747
ENSG00000237328	RAI1-AS1	grey	0.16941596
ENSG00000039560	RAI14	brown	-0.5678064
ENSG00000131831	RAI2	black	-0.5112751
ENSG00000006451	RALA	grey	-0.0436476
ENSG00000144118	RALB	grey	0.1012161
ENSG00000017797	RALBP1	darkgrey	0.61044467
ENSG00000174373	RALGAPA1	grey	0.07828863
ENSG00000229419	RALGAPA1P	grey	0.17948567
ENSG00000188559	RALGAPA2	grey	0.48069529
ENSG00000170471	RALGAPB	grey	0.07018666
ENSG00000160271	RALGDS	pink	0.71620604
ENSG00000136828	RALGPS1	yellow	-0.3541462
ENSG00000116191	RALGPS2	purple	0.69733657
ENSG00000184672	RALYL	purple	0.71226922
ENSG00000132329	RAMP1	grey	-0.0099991
ENSG00000131477	RAMP2	tan	0.53639844
ENSG00000122679	RAMP3	tan	0.50292325
ENSG00000132341	RAN	yellow	-0.6378257
ENSG00000099901	RANBP1	grey	-0.2011754
ENSG00000141084	RANBP10	grey	0.18712694
ENSG00000153201	RANBP2	lightcyan	-0.6211204
ENSG00000031823	RANBP3	green	-0.6444817
ENSG00000164188	RANBP3L	turquoise	0.76195009
ENSG00000137040	RANBP6	lightcyan	-0.5729572
ENSG00000010017	RANBP9	grey	0.28461005
ENSG00000100401	RANGAP1	turquoise	-0.7173301
ENSG00000108961	RANGRF	salmon	0.49921363
ENSG00000225125	RANP4	grey	0.30667796
ENSG00000116473	RAP1A	green	0.80321607
ENSG00000127314	RAP1B	pink	0.54010688
ENSG00000076864	RAP1GAP	blue	0.59705682
ENSG00000132359	RAP1GAP2	purple	0.70168795
ENSG00000138698	RAP1GDS1	mediumpurp	-0.600236
ENSG00000125249	RAP2A	darkgrey	0.48609876
ENSG00000181467	RAP2B	yellow	-0.5276602
ENSG00000123728	RAP2C	grey60	-0.7090837
ENSG00000232160	RAP2C-AS1	grey	0.07347242
ENSG00000107263	RAPGEF1	black	0.71229845
ENSG00000109756	RAPGEF2	grey	0.174738
ENSG00000079337	RAPGEF3	blue	-0.4605756

ENSG00000091428	RAPGEF4	blue	0.45212813
ENSG00000228016	RAPGEF4-AS1	grey	-0.1227108
ENSG00000158987	RAPGEF6	grey	0.26428111
ENSG00000108352	RAPGEFL1	purple	0.73016437
ENSG00000173166	RAPH1	grey	-0.060526
ENSG00000131759	RARA	grey	0.05304801
ENSG00000172819	RARG	turquoise	0.4319769
ENSG00000118849	RARRES1	grey	0.31284959
ENSG00000106538	RARRES2	turquoise	0.41015023
ENSG00000231913	RARRES2P3	grey	0.36808807
ENSG00000133321	RARRES3	pink	0.59670863
ENSG00000113643	RARS	turquoise	-0.4056816
ENSG00000146282	RARS2	yellow	0.34648088
ENSG00000145715	RASA1	darkred	-0.53381
ENSG00000155903	RASA2	grey	-0.1865535
ENSG00000185989	RASA3	grey	-0.0169418
ENSG00000105808	RASA4	grey	-0.1758652
ENSG00000228903	RASA4CP	blue	0.51365466
ENSG00000111344	RASAL1	turquoise	-0.428593
ENSG00000075391	RASAL2	blue	0.55490013
ENSG00000108551	RASD1	pink	0.70786728
ENSG00000100302	RASD2	magenta	0.49546757
ENSG00000165105	RASEF	turquoise	-0.6750872
ENSG00000138670	RASGEF1B	turquoise	-0.5866103
ENSG00000146090	RASGEF1C	grey	-0.193617
ENSG00000058335	RASGRF1	blue	0.65423191
ENSG00000113319	RASGRF2	black	0.58788834
ENSG00000172575	RASGRP1	blue	0.62428167
ENSG00000068831	RASGRP2	grey60	0.68076683
ENSG00000152689	RASGRP3	yellow	0.78630396
ENSG00000171777	RASGRP4	royalblue	0.56877646
ENSG00000105538	RASIP1	tan	0.66801261
ENSG00000100276	RASL10A	yellowgreen	0.59460891
ENSG00000141150	RASL10B	darkorange	0.52527279
ENSG00000122035	RASL11A	grey	0.15233959
ENSG00000128045	RASL11B	grey	0.02500144
ENSG00000103710	RASL12	turquoise	0.36058855
ENSG00000068028	RASSF1	grey	0.00451733
ENSG00000101265	RASSF2	green	0.88728484
ENSG00000153179	RASSF3	salmon	-0.5620452
ENSG00000107551	RASSF4	blue	-0.5319968
ENSG00000136653	RASSF5	grey	0.12776993
ENSG00000099849	RASSF7	turquoise	-0.4957331
ENSG00000123094	RASSF8	turquoise	0.81609271
ENSG00000246695	RASSF8-AS1	turquoise	0.72252722
ENSG00000162437	RAVER2	darkred	0.54493016
ENSG00000139687	RB1	royalblue	0.55247408
ENSG00000023287	RB1CC1	grey	-0.1472895

ENSG00000146587	RBAK	turquoise	0.28696728
ENSG00000162521	RBBP4	grey	0.16648036
ENSG00000249485	RBBP4P1	grey	0.19752829
ENSG00000117222	RBBP5	brown	0.40025098
ENSG00000122257	RBBP6	brown	-0.2890087
ENSG00000102054	RBBP7	salmon	0.77388043
ENSG00000101773	RBBP8	grey	0.01625113
ENSG00000089050	RBBP9	red	0.78062332
ENSG00000125826	RBCK1	yellow	0.47187426
ENSG00000101546	RBFA	turquoise	-0.5167525
ENSG00000261126	RBFADN	grey	0.19956264
ENSG00000167281	RBFOX3	magenta	0.60401363
ENSG00000171174	RBKS	grey	0.1712726
ENSG00000080839	RBL1	turquoise	0.68403097
ENSG00000182872	RBM10	grey	-0.1926708
ENSG00000185272	RBM11	blue	0.69871114
ENSG00000244462	RBM12	green	0.48240716
ENSG00000183808	RBM12B	brown	-0.6387546
ENSG00000212998	RBM12B-AS1	grey	0.24669126
ENSG00000162775	RBM15	grey	0.14080489
ENSG00000179837	RBM15B	grey	0.10298176
ENSG00000134453	RBM17	lightgreen	0.7804743
ENSG00000119446	RBM18	grey	0.04334974
ENSG00000122965	RBM19	turquoise	-0.5597977
ENSG00000203867	RBM20	grey	-0.0742503
ENSG00000086589	RBM22	turquoise	-0.6401235
ENSG00000100461	RBM23	green	-0.6722015
ENSG00000112183	RBM24	purple	0.57156149
ENSG00000119707	RBM25	darkgrey	0.62995807
ENSG00000139746	RBM26	grey	-0.0007095
ENSG00000227354	RBM26-AS1	grey	0.24313671
ENSG00000091009	RBM27	grey	0.04818614
ENSG00000106344	RBM28	darkgrey	0.58379529
ENSG00000102317	RBM3	grey	0.04279789
ENSG00000184863	RBM33	turquoise	-0.444177
ENSG00000188739	RBM34	yellow	-0.3787413
ENSG00000132819	RBM38	brown	-0.6331127
ENSG00000131051	RBM39	blue	-0.4285871
ENSG00000173933	RBM4	lightgreen	0.63797655
ENSG00000089682	RBM41	brown	-0.6435096
ENSG00000126254	RBM42	grey	-0.1358782
ENSG00000184898	RBM43	turquoise	0.35373004
ENSG00000239263	RBM43P1	grey	0.36516495
ENSG00000155636	RBM45	grey	0.11155278
ENSG00000173914	RBM4B	lightcyan	0.73862836
ENSG00000003756	RBM5	salmon	0.75364666
ENSG00000076053	RBM7	turquoise	0.50732599
ENSG00000131795	RBM8A	grey	-0.0357356

ENSG00000153250	RBMS1	black	0.71029422
ENSG00000076067	RBMS2	pink	0.60336553
ENSG00000144642	RBMS3	grey	0.29281137
ENSG00000203506	RBMS3-AS2	grey	0.27163436
ENSG00000235904	RBMS3-AS3	grey	0.31995172
ENSG00000147274	RBMX	bisque4	0.72425122
ENSG00000134597	RBMX2	grey	-0.0315444
ENSG00000213516	RBMXL1	brown	-0.8266217
ENSG00000249465	RBMXP4	grey	0.19997278
ENSG00000114115	RBP1	grey	-0.065057
ENSG00000114113	RBP2	grey	0.07482368
ENSG00000162444	RBP7	yellow	0.70081188
ENSG00000168214	RBPJ	yellow	0.79399532
ENSG00000157110	RBPMS	turquoise	0.46795219
ENSG00000166831	RBPMS2	grey	0.24918773
ENSG00000100387	RBX1	grey	-0.1994355
ENSG00000236535	RC3H1-IT1	brown	-0.4193938
ENSG00000159200	RCAN1	darkgrey	0.55497526
ENSG00000172348	RCAN2	grey60	0.65630166
ENSG00000117602	RCAN3	green	0.42575525
ENSG00000136144	RCBTB1	brown	-0.7001315
ENSG00000136161	RCBTB2	turquoise	0.58035537
ENSG00000179051	RCC2	grey	0.15734297
ENSG00000166965	RCCD1	yellow	-0.4065454
ENSG00000173653	RCE1	grey	-0.1003141
ENSG00000163743	RCHY1	red	0.69310324
ENSG00000120158	RCL1	yellow	0.48493587
ENSG00000214455	RCN1P2	grey	0.13169634
ENSG00000142552	RCN3	royalblue	0.40437466
ENSG00000089902	RCOR1	grey	0.01510032
ENSG00000167771	RCOR2	grey	-0.1880231
ENSG00000198771	RCSD1	black	0.63451203
ENSG00000121039	RDH10	pink	0.47151732
ENSG00000072042	RDH11	grey	0.13089708
ENSG00000240857	RDH14	grey	-0.0592022
ENSG00000137710	RDX	yellow	0.7848492
ENSG00000100918	REC8	turquoise	-0.4887256
ENSG00000122707	RECK	grey	0.21729457
ENSG00000160957	RECQL4	grey	-0.025615
ENSG00000108469	RECQL5	turquoise	-0.3980675
ENSG00000068615	REEP1	blue	0.78455836
ENSG00000132563	REEP2	blue	0.80685511
ENSG00000165476	REEP3	yellow	0.80889729
ENSG00000168476	REEP4	cyan	0.55972621
ENSG00000129625	REEP5	grey	0.05859522
ENSG00000115255	REEP6	grey	0.22505674
ENSG00000162924	REL	darkred	-0.4719376
ENSG00000173039	RELA	blue	-0.6877321

ENSG00000181826	RELL1	blue	-0.7007836
ENSG00000164620	RELL2	blue	0.59941021
ENSG00000189056	RELN	grey	-0.0248914
ENSG00000054967	RELT	grey	-0.0325955
ENSG00000139890	REM2	grey	-0.1206912
ENSG00000135597	REPS1	red	0.51062063
ENSG00000169891	REPS2	blue	0.65311098
ENSG00000157916	RER1	grey	-0.0051828
ENSG00000142599	RERE	grey	-0.1999136
ENSG00000134533	RERG	grey	-0.0123419
ENSG00000255660	RERG-AS1	grey	0.36075175
ENSG00000256650	RERG-IT1	grey	0.31760065
ENSG00000111404	RERGL	grey	-0.0758051
ENSG00000182698	RESP18	grey	-0.0124882
ENSG00000084093	REST	brown	-0.8903596
ENSG00000042445	RETSAT	turquoise	0.43310576
ENSG00000135945	REV1	grey	0.24248505
ENSG00000079313	REXO1	black	0.63379642
ENSG00000076043	REXO2	grey	0.09547747
ENSG00000148300	REXO4	grey	0.03858474
ENSG00000035928	RFC1	yellow	0.4887336
ENSG00000049541	RFC2	grey	0.30071042
ENSG00000133119	RFC3	grey	-0.1312521
ENSG00000163918	RFC4	lightcyan	-0.5562157
ENSG00000111445	RFC5	grey	0.04920358
ENSG00000092871	RFFL	yellow	0.83930669
ENSG00000135002	RFK	grey	-0.2856945
ENSG00000215802	RFKP1	grey	0.25778347
ENSG00000169733	RFNG	grey	0.18193413
ENSG00000225465	RFPL1S	blue	0.80944557
ENSG00000128253	RFPL2	grey	-0.0318107
ENSG00000163933	RFT1	grey	0.01455733
ENSG00000131378	RFTN1	pink	0.50311238
ENSG00000162944	RFTN2	turquoise	0.6922939
ENSG00000143207	RFWD2	grey	-0.1521577
ENSG00000168411	RFWD3	grey	0.02402195
ENSG00000132005	RFX1	grey	-0.019409
ENSG00000087903	RFX2	turquoise	0.5411059
ENSG00000080298	RFX3	black	-0.6336582
ENSG00000111783	RFX4	turquoise	0.67551466
ENSG00000143390	RFX5	yellowgreen	0.72105094
ENSG00000181827	RFX7	blue	0.51765291
ENSG00000064490	RFXANK	turquoise	0.3317292
ENSG00000133111	RFXAP	grey	0.019566
ENSG00000243978	RGAG1	purple	0.73368136
ENSG00000242732	RGAG4	turquoise	-0.3858183
ENSG00000102760	RGCC	yellow	0.60230635
ENSG00000143344	RGL1	pink	0.59768393

ENSG00000237441	RGL2	grey	0.02822378
ENSG00000205517	RGL3	royalblue	0.44524307
ENSG00000159496	RGL4	grey	0.23186874
ENSG00000182175	RGMA	turquoise	0.50752976
ENSG00000174136	RGMB	purple	0.67217586
ENSG00000130988	RGN	turquoise	0.63683128
ENSG00000107185	RGP1	brown	-0.7363716
ENSG00000169629	RGPD8	turquoise	-0.4462528
ENSG00000148604	RGR	grey	0.18862271
ENSG00000148908	RGS10	cyan	0.8906541
ENSG00000076344	RGS11	grey	0.0443802
ENSG00000159788	RGS12	turquoise	-0.454851
ENSG00000169220	RGS14	magenta	0.88206104
ENSG00000143333	RGS16	magenta	-0.4384638
ENSG00000091844	RGS17	purple	0.40193714
ENSG00000229473	RGS17P1	grey	0.36428696
ENSG00000171700	RGS19	royalblue	0.7254412
ENSG00000116741	RGS2	grey	-0.1254947
ENSG00000147509	RGS20	grey	-0.023905
ENSG00000138835	RGS3	grey	0.50886529
ENSG00000117152	RGS4	grey60	0.63763923
ENSG00000143248	RGS5	grey	0.02027548
ENSG00000182732	RGS6	black	-0.7515796
ENSG00000182901	RGS7	blue	0.76800254
ENSG00000186479	RGS7BP	grey60	0.8242415
ENSG00000135824	RGS8	blue	0.69131771
ENSG00000108370	RGS9	magenta	0.80272977
ENSG00000144468	RHBDD1	turquoise	0.64549247
ENSG00000005486	RHBDD2	blue	0.81670513
ENSG00000100263	RHBDD3	grey	0.18192729
ENSG00000007384	RHBDF1	turquoise	0.47852538
ENSG00000129667	RHBDF2	royalblue	0.81212208
ENSG00000103269	RHBDL1	yellow	-0.5520804
ENSG00000158315	RHBDL2	yellow	0.69954106
ENSG00000141314	RHBDL3	grey	0.03969458
ENSG00000188672	RHCE	grey	0.07133181
ENSG00000140519	RHCG	grey	0.13769086
ENSG00000187010	RHD	grey	0.05751322
ENSG00000171792	RHNO1	turquoise	0.36654106
ENSG00000067560	RHOA	turquoise	0.77444225
ENSG00000143878	RHOB	turquoise	0.72681388
ENSG00000072422	RHOBTB1	grey	0.21763392
ENSG00000008853	RHOBTB2	grey60	0.61038532
ENSG00000164292	RHOBTB3	turquoise	0.77237748
ENSG00000155366	RHOC	turquoise	0.72635056
ENSG00000177105	RHOG	yellow	0.83522313
ENSG00000126785	RHOJ	red	0.58929137
ENSG00000119729	RHOQ	turquoise	0.48916406

ENSG00000126858	RHOT1	grey	-0.0515614
ENSG00000266145	RHOT1P1	grey	0.26404369
ENSG00000203616	RHOT1P2	grey	0.06123091
ENSG00000140983	RHOT2	blue	0.37187276
ENSG00000116574	RHOU	yellow	0.79436815
ENSG00000158106	RHPN1	grey	-0.1676184
ENSG00000131941	RHPN2	turquoise	0.68721607
ENSG00000166405	RIC3	grey	-0.0485737
ENSG00000177963	RIC8A	lightgreen	-0.5613384
ENSG00000111785	RIC8B	magenta	0.75344853
ENSG00000164327	RICTOR	grey	0.0160627
ENSG00000080345	RIF1	grey	-0.3581654
ENSG00000167705	RILP	greenyellow	-0.4769957
ENSG00000188026	RILPL1	grey	0.04625981
ENSG00000150977	RILPL2	grey	0.04790411
ENSG00000060709	RIMBP2	purple	0.76994015
ENSG00000177181	RIMKLA	grey	0.17143135
ENSG00000166532	RIMKLB	brown	-0.5030986
ENSG00000189089	RIMKLBP1	grey	0.25347369
ENSG00000225920	RIMKLBP2	grey	0.23879466
ENSG00000079841	RIMS1	blue	0.54798615
ENSG00000176406	RIMS2	darkorange	0.62625841
ENSG00000101098	RIMS4	blue	0.50113691
ENSG00000132669	RIN2	brown	-0.7030496
ENSG00000204227	RING1	grey	-0.0720614
ENSG00000135249	RINT1	grey	0.26008992
ENSG00000124784	RIOK1	grey	0.18162698
ENSG00000058729	RIOK2	grey	0.00366374
ENSG00000101782	RIOK3	grey	0.3293777
ENSG00000137275	RIPK1	blue	-0.5464043
ENSG00000104312	RIPK2	grey	0.11969465
ENSG00000203877	RIPPLY2	brown	0.60859549
ENSG00000183145	RIPPLY3	grey	0.05810001
ENSG00000143622	RIT1	blue	-0.604395
ENSG00000152214	RIT2	blue	0.65717894
ENSG00000139405	RITA1	blue	0.41611726
ENSG00000140522	RLBP1	turquoise	0.69628118
ENSG00000131263	RLIM	red	0.64853409
ENSG00000229456	RLIMP1	grey	0.35133377
ENSG00000159753	RLTPR	blue	0.78294925
ENSG00000176623	RMDN1	brown	-0.7780184
ENSG00000115841	RMDN2	grey	0.06847586
ENSG00000137824	RMDN3	grey	0.09104342
ENSG00000178966	RMI1	turquoise	0.80508233
ENSG00000175643	RMI2	grey	-0.2531952
ENSG00000155906	RMND1	darkred	0.53188598
ENSG00000153561	RMND5A	blue	-0.6222166
ENSG00000145916	RMND5B	grey	0.06691356

ENSG00000251950	RMRPP4	grey	0.3974267
ENSG00000255794	RMST	yellowgreen	0.67082324
ENSG00000202198	RN7SK	darkred	-0.6137986
ENSG00000223190	RN7SKP100	grey	0.36742003
ENSG00000223308	RN7SKP101	black	0.70526131
ENSG00000253001	RN7SKP105	grey	0.33977031
ENSG00000252084	RN7SKP12	grey	0.20974485
ENSG00000201600	RN7SKP124	grey	0.37082282
ENSG00000201140	RN7SKP128	grey	0.47325813
ENSG00000222343	RN7SKP139	grey	0.07001988
ENSG00000222162	RN7SKP151	grey	0.43115807
ENSG00000222068	RN7SKP154	yellow	0.53294248
ENSG00000222112	RN7SKP16	grey	0.41690753
ENSG00000200091	RN7SKP163	turquoise	0.46681504
ENSG00000199691	RN7SKP173	grey	0.33843058
ENSG00000252396	RN7SKP195	purple	-0.5379757
ENSG00000238324	RN7SKP198	grey	0.19496486
ENSG00000201078	RN7SKP214	grey	0.27472411
ENSG00000222543	RN7SKP220	grey	0.42337814
ENSG00000252838	RN7SKP23	grey	0.17233435
ENSG00000202512	RN7SKP230	grey	0.4199826
ENSG00000252814	RN7SKP233	grey	0.44117726
ENSG00000252982	RN7SKP234	grey	0.2256025
ENSG00000222594	RN7SKP235	grey	0.23393003
ENSG00000240294	RN7SKP241	grey	0.46029299
ENSG00000223026	RN7SKP247	grey	0.32998433
ENSG00000252233	RN7SKP253	grey	0.35547347
ENSG00000222164	RN7SKP266	grey	0.41994476
ENSG00000223039	RN7SKP268	grey	0.41238518
ENSG00000202415	RN7SKP269	grey	0.29713209
ENSG00000222460	RN7SKP271	grey	0.42392164
ENSG00000252797	RN7SKP272	grey	0.5513124
ENSG00000252051	RN7SKP276	grey	0.17641101
ENSG00000253006	RN7SKP283	grey	0.12714815
ENSG00000222069	RN7SKP285	grey	0.45839781
ENSG00000252355	RN7SKP287	grey	0.26986319
ENSG00000223117	RN7SKP296	grey	0.24737952
ENSG00000223305	RN7SKP30	grey	0.3017101
ENSG00000201364	RN7SKP37	grey	0.33251963
ENSG00000251982	RN7SKP43	grey	0.2447673
ENSG00000223269	RN7SKP53	grey	0.48777424
ENSG00000222445	RN7SKP56	grey	0.21628441
ENSG00000222942	RN7SKP58	grey	0.44667952
ENSG00000202260	RN7SKP69	grey	0.43826553
ENSG00000252464	RN7SKP70	grey	0.21053074
ENSG00000201428	RN7SKP71	grey	0.29985763
ENSG00000201289	RN7SKP76	grey	0.2548829
ENSG00000201581	RN7SKP78	grey	0.39049262

ENSG00000202058	RN7SKP80	grey	0.35301198
ENSG00000252730	RN7SKP84	grey	0.05401311
ENSG00000200966	RN7SKP87	grey	0.31276857
ENSG00000252656	RN7SKP88	grey	0.36786323
ENSG00000201793	RN7SKP9	grey	0.3365881
ENSG00000199730	RN7SKP95	grey	0.42177523
ENSG00000252757	RN7SKP96	grey	0.29633538
ENSG00000222898	RN7SKP97	grey	0.5350981
ENSG00000252837	RN7SKP99	grey	0.12730371
ENSG00000258486	RN7SL1	grey	-0.0737874
ENSG00000241064	RN7SL110P	grey	0.36874896
ENSG00000240474	RN7SL116P	grey	0.42867514
ENSG00000242175	RN7SL127P	grey	0.12968868
ENSG00000240869	RN7SL128P	grey	0.16778448
ENSG00000263360	RN7SL134P	grey	0.41047585
ENSG00000266628	RN7SL137P	grey	0.39519735
ENSG00000266453	RN7SL142P	brown	-0.3093844
ENSG00000244357	RN7SL145P	grey	0.35034208
ENSG00000266503	RN7SL162P	grey	0.40264726
ENSG00000242614	RN7SL164P	grey	0.45708993
ENSG00000265624	RN7SL166P	grey	0.31099091
ENSG00000242547	RN7SL169P	grey	0.38970075
ENSG00000243373	RN7SL173P	grey	0.20714044
ENSG00000242976	RN7SL177P	grey	0.2663664
ENSG00000264933	RN7SL190P	grey	0.24290366
ENSG00000243532	RN7SL19P	grey	0.10606336
ENSG00000265150	RN7SL2	grey	0.08755272
ENSG00000265123	RN7SL200P	grey	0.38577226
ENSG00000264018	RN7SL207P	grey	0.30732414
ENSG00000264706	RN7SL217P	grey	0.2318449
ENSG00000266467	RN7SL220P	grey	0.14112824
ENSG00000264916	RN7SL230P	turquoise	0.40976443
ENSG00000242971	RN7SL233P	grey	0.25617931
ENSG00000242999	RN7SL239P	turquoise	0.39662224
ENSG00000265093	RN7SL246P	grey	0.08428017
ENSG00000241652	RN7SL253P	turquoise	0.47428512
ENSG00000265033	RN7SL262P	grey	0.11463259
ENSG00000243051	RN7SL269P	grey	0.23885889
ENSG00000240490	RN7SL277P	grey	0.23495484
ENSG00000244671	RN7SL280P	grey	0.38025989
ENSG00000243313	RN7SL285P	grey	0.26750812
ENSG00000240183	RN7SL297P	grey	0.35608929
ENSG00000266037	RN7SL3	green	0.42751789
ENSG00000242241	RN7SL306P	grey	0.28818101
ENSG00000243951	RN7SL308P	grey	0.33024448
ENSG00000264046	RN7SL312P	grey	0.45816723
ENSG00000243900	RN7SL320P	grey	0.40463773
ENSG00000240853	RN7SL328P	grey	0.23739062

ENSG00000242170	RN7SL329P	grey	0.41997327
ENSG00000264017	RN7SL336P	grey	0.37552817
ENSG00000241568	RN7SL338P	grey	0.32659785
ENSG00000241395	RN7SL344P	grey	0.31525037
ENSG00000266345	RN7SL349P	grey	0.38914981
ENSG00000243254	RN7SL350P	grey	0.4303914
ENSG00000240098	RN7SL351P	grey	0.43891616
ENSG00000242707	RN7SL362P	cyan	-0.4406239
ENSG00000243437	RN7SL370P	grey	0.24313394
ENSG00000266122	RN7SL373P	grey	0.36455242
ENSG00000240723	RN7SL382P	grey	0.41965128
ENSG00000243723	RN7SL393P	grey	0.46312943
ENSG00000244307	RN7SL395P	grey	0.30342378
ENSG00000242893	RN7SL413P	brown	-0.419431
ENSG00000264384	RN7SL431P	grey	0.31247639
ENSG00000264963	RN7SL440P	grey	0.42708312
ENSG00000241413	RN7SL441P	blue	-0.4102929
ENSG00000243103	RN7SL452P	grey	0.36858518
ENSG00000266103	RN7SL458P	grey	0.32665227
ENSG00000240993	RN7SL459P	grey	0.35926356
ENSG00000264508	RN7SL473P	grey	0.42331462
ENSG00000265802	RN7SL49P	grey	0.21595561
ENSG00000263740	RN7SL4P	grey	0.11977436
ENSG00000244112	RN7SL508P	grey	0.30367859
ENSG00000266308	RN7SL510P	grey	0.47903294
ENSG00000242699	RN7SL516P	grey	0.35776121
ENSG00000241939	RN7SL517P	grey	0.50819105
ENSG00000243642	RN7SL526P	grey	0.46848103
ENSG00000240692	RN7SL538P	grey	0.42939803
ENSG00000239466	RN7SL552P	grey	0.25912302
ENSG00000243260	RN7SL558P	grey	0.23225557
ENSG00000240750	RN7SL559P	grey	0.29301452
ENSG00000264628	RN7SL565P	grey	0.21231606
ENSG00000241983	RN7SL566P	grey	0.36551842
ENSG00000265942	RN7SL577P	grey	0.31356525
ENSG00000242256	RN7SL57P	grey	0.13060925
ENSG00000241156	RN7SL582P	grey	0.44685383
ENSG00000242037	RN7SL585P	turquoise	0.57290326
ENSG00000239247	RN7SL589P	grey	0.44235264
ENSG00000264352	RN7SL602P	grey	0.3995201
ENSG00000243267	RN7SL614P	grey	0.07721995
ENSG00000263989	RN7SL615P	turquoise	0.54825837
ENSG00000241243	RN7SL629P	brown	-0.5955683
ENSG00000264978	RN7SL630P	grey	0.2806045
ENSG00000264531	RN7SL641P	grey	0.42583532
ENSG00000265727	RN7SL648P	brown	-0.5212803
ENSG00000263621	RN7SL650P	grey	0.32413207
ENSG00000265770	RN7SL660P	grey	0.2431423

ENSG00000239899	RN7SL674P	grey	0.2102819
ENSG00000242330	RN7SL683P	grey	0.4066711
ENSG00000239726	RN7SL688P	grey	0.40974856
ENSG00000263432	RN7SL689P	grey	0.33527625
ENSG00000242020	RN7SL68P	turquoise	0.40710281
ENSG00000240820	RN7SL695P	grey	0.24108907
ENSG00000241693	RN7SL704P	grey	0.3028385
ENSG00000264423	RN7SL718P	grey	0.42738604
ENSG00000244692	RN7SL724P	grey	0.1181604
ENSG00000244044	RN7SL735P	grey	0.21841489
ENSG00000243015	RN7SL737P	grey	0.14965811
ENSG00000240964	RN7SL751P	grey	0.23332758
ENSG00000239822	RN7SL754P	grey	0.22419869
ENSG00000244610	RN7SL756P	grey	0.49595292
ENSG00000266164	RN7SL758P	grey	0.16883708
ENSG00000244308	RN7SL762P	grey	0.1098709
ENSG00000266540	RN7SL772P	grey	0.35001469
ENSG00000241291	RN7SL791P	grey	0.32496852
ENSG00000242398	RN7SL800P	grey	0.43105853
ENSG00000241230	RN7SL801P	grey	0.26143417
ENSG00000241217	RN7SL809P	grey	0.28264048
ENSG00000243359	RN7SL815P	grey	0.3378924
ENSG00000244218	RN7SL81P	grey	0.29431363
ENSG00000243819	RN7SL832P	grey	0.33416531
ENSG00000243745	RN7SL844P	grey	0.13544862
ENSG00000240913	RN7SL856P	grey	0.45291368
ENSG00000244632	RN7SL863P	grey	0.46849833
ENSG00000239923	RN7SL864P	grey	0.47234661
ENSG00000129538	RNASE1	yellow	0.78143374
ENSG00000169413	RNASE6	cyan	0.77733032
ENSG00000171865	RNASEH1	grey	0.12690397
ENSG00000234171	RNASEH1-AS1	grey	0.13088703
ENSG00000104889	RNASEH2A	grey	-0.0023879
ENSG00000136104	RNASEH2B	grey	-0.0613368
ENSG00000172922	RNASEH2C	brown	0.43845176
ENSG00000135828	RNASEL	grey	0.04655827
ENSG00000026297	RNASET2	cyan	0.76248566
ENSG00000172602	RND1	grey	0.22464149
ENSG00000115963	RND3	grey	0.05049997
ENSG00000022840	RNF10	turquoise	-0.5787056
ENSG00000239305	RNF103	darkorange	-0.6626781
ENSG00000123091	RNF11	lightcyan	0.83153254
ENSG00000157450	RNF111	brown	0.44787671
ENSG00000128482	RNF112	blue	0.617714
ENSG00000125352	RNF113A	brown	0.65125281
ENSG00000124226	RNF114	blue	-0.809742
ENSG00000121848	RNF115	grey	0.00160722
ENSG00000137522	RNF121	grey	0.03663454

ENSG00000164068	RNF123	grey60	0.60874398
ENSG00000101695	RNF125	yellow	0.44821692
ENSG00000133135	RNF128	purple	0.67411153
ENSG00000082996	RNF13	yellow	0.68843741
ENSG00000113269	RNF130	turquoise	-0.5218937
ENSG00000181481	RNF135	grey	-0.1003093
ENSG00000134758	RNF138	grey	0.09350574
ENSG00000250853	RNF138P1	grey	0.34080825
ENSG00000170881	RNF139	grey	0.14887461
ENSG00000245149	RNF139-AS1	grey	0.15565387
ENSG00000013561	RNF14	bisque4	0.67415131
ENSG00000110315	RNF141	brown	-0.6454867
ENSG00000228203	RNF144A-AS1	turquoise	-0.5385596
ENSG00000137393	RNF144B	tan	0.79640555
ENSG00000145860	RNF145	lightcyan	-0.6465913
ENSG00000118518	RNF146	brown	-0.4568451
ENSG00000163162	RNF149	grey	0.21181947
ENSG00000170153	RNF150	brown	0.60224998
ENSG00000176641	RNF152	brown	-0.4631495
ENSG00000141576	RNF157	darkorange	0.49036612
ENSG00000267128	RNF157-AS1	grey	0.34450645
ENSG00000141622	RNF165	blue	0.70839349
ENSG00000158717	RNF166	cyan	0.59188004
ENSG00000108523	RNF167	grey	0.33968263
ENSG00000163961	RNF168	grey	0.18647176
ENSG00000166439	RNF169	brown	-0.5652907
ENSG00000120925	RNF170	grey	0.06733333
ENSG00000145428	RNF175	brown	0.83499903
ENSG00000164197	RNF180	yellowgreen	0.74647287
ENSG00000168894	RNF181	red	0.40389534
ENSG00000180537	RNF182	brown	-0.5004129
ENSG00000138942	RNF185	grey	0.23452805
ENSG00000168159	RNF187	blue	0.58400131
ENSG00000034677	RNF19A	blue	-0.5378548
ENSG00000116514	RNF19B	turquoise	-0.5532549
ENSG00000121481	RNF2	grey	-0.1947454
ENSG00000155827	RNF20	darkgrey	0.71729597
ENSG00000158286	RNF207	grey	0.00358744
ENSG00000178222	RNF212	blue	0.52108618
ENSG00000173821	RNF213	grey	-0.0218724
ENSG00000167257	RNF214	bisque4	0.78427936
ENSG00000099999	RNF215	red	0.49672345
ENSG00000011275	RNF216	turquoise	-0.7126812
ENSG00000237738	RNF216-IT1	grey	0.16802223
ENSG00000196204	RNF216P1	turquoise	-0.2966999
ENSG00000146373	RNF217	blue	-0.3718371
ENSG00000152193	RNF219	mediumpurp	0.69773625
ENSG00000234377	RNF219-AS1	turquoise	0.70907002

ENSG00000187147	RNF220	turquoise	-0.5650597
ENSG00000101236	RNF24	grey	0.2101245
ENSG00000163481	RNF25	grey	0.22614237
ENSG00000173456	RNF26	grey	0.08866213
ENSG00000092098	RNF31	grey	0.09934214
ENSG00000105982	RNF32	yellow	0.36325652
ENSG00000170633	RNF34	grey60	0.58621571
ENSG00000137075	RNF38	lightcyan	0.83515647
ENSG00000204618	RNF39	grey	0.19148269
ENSG00000063978	RNF4	turquoise	0.4685371
ENSG00000103549	RNF40	grey	0.33621326
ENSG00000146083	RNF44	lightcyan	0.64552965
ENSG00000204308	RNF5	grey	-0.1989186
ENSG00000127870	RNF6	grey60	0.55569705
ENSG00000114125	RNF7	salmon	0.80907634
ENSG00000112130	RNF8	brown	0.7095985
ENSG00000189050	RNFT1	grey	0.06854258
ENSG00000135119	RNFT2	blue	0.69167354
ENSG00000111880	RNGTT	grey	0.13096481
ENSG00000237731	RNGTTP1	turquoise	0.38289046
ENSG00000023191	RNH1	salmon	0.67839953
ENSG00000184719	RNLS	grey	0.09707375
ENSG00000101654	RNMT	brown	0.72846036
ENSG00000171861	RNMTL1	salmon	0.62322845
ENSG00000185946	RNPC3	grey	-0.1233242
ENSG00000176393	RNPEP	grey	-0.1636482
ENSG00000142327	RNPEPL1	yellow	0.55937652
ENSG00000205937	RNPS1	brown	0.43947231
ENSG00000252311	RNU1-103P	grey	0.36075699
ENSG00000206702	RNU1-11P	grey	0.32241101
ENSG00000202408	RNU1-122P	grey	0.19109526
ENSG00000200731	RNU1-124P	grey	0.36759255
ENSG00000252105	RNU1-143P	grey	0.29191383
ENSG00000212172	RNU1-149P	grey	0.38869961
ENSG00000202347	RNU1-16P	grey	0.18937355
ENSG00000201155	RNU1-24P	grey	0.10267503
ENSG00000199846	RNU1-72P	grey	0.15518272
ENSG00000201616	RNU1-91P	grey	0.47175739
ENSG00000201574	RNU1-93P	grey	0.45683368
ENSG00000199497	RNU1-94P	grey	0.18997424
ENSG00000201348	RNU105B	black	0.57186808
ENSG00000252707	RNU11-2P	grey	0.27545316
ENSG00000212429	RNU11-6P	grey	0.43166294
ENSG00000239122	RNU2-11P	grey	0.29447112
ENSG00000252639	RNU2-24P	grey	0.4264408
ENSG00000252763	RNU2-31P	grey	0.25454026
ENSG00000252343	RNU2-34P	grey	0.32547488
ENSG00000222788	RNU2-38P	grey	0.40262104

ENSG00000222629	RNU2-42P	grey	0.32648087
ENSG00000252847	RNU2-46P	grey	0.39448462
ENSG00000222414	RNU2-59P	grey	0.23070272
ENSG00000222650	RNU2-70P	grey	0.4110422
ENSG00000222726	RNU2-7P	grey	0.25399346
ENSG00000201607	RNU4-16P	grey	0.31566136
ENSG00000199672	RNU4-21P	grey	0.44230507
ENSG00000201435	RNU4-24P	grey	0.51381246
ENSG00000222202	RNU4-26P	grey	0.57615074
ENSG00000201164	RNU4-36P	grey	0.1680941
ENSG00000199325	RNU4-39P	grey	0.38391394
ENSG00000222808	RNU4-47P	grey	0.36388786
ENSG00000202429	RNU4-48P	grey	0.25895202
ENSG00000201570	RNU4-56P	grey	0.34341352
ENSG00000253048	RNU4-60P	grey	0.29899744
ENSG00000223175	RNU4-61P	grey	0.49286789
ENSG00000222872	RNU4-78P	grey	0.55280451
ENSG00000199313	RNU4-82P	grey	0.20287473
ENSG00000222067	RNU4-86P	grey	0.46471165
ENSG00000200974	RNU4-87P	grey	0.34359517
ENSG00000223152	RNU4-88P	grey	0.39669123
ENSG00000251748	RNU4ATAC11P	grey	0.3954361
ENSG00000221439	RNU4ATAC16P	grey	0.51065395
ENSG00000252508	RNU4ATAC3P	grey	0.35225333
ENSG00000251788	RNU5A-7P	grey	0.56327961
ENSG00000200372	RNU5E-8P	grey	0.18991453
ENSG00000252393	RNU6-1004P	grey	0.44402158
ENSG00000207248	RNU6-1005P	grey	0.55726815
ENSG00000252414	RNU6-100P	grey	0.42864912
ENSG00000207399	RNU6-1011P	grey	0.34990822
ENSG00000238456	RNU6-1014P	grey	0.0960585
ENSG00000252498	RNU6-1016P	greenyellow	-0.4116388
ENSG00000201294	RNU6-1019P	grey	0.40912953
ENSG00000222255	RNU6-101P	grey	0.44626137
ENSG00000207194	RNU6-1026P	grey	0.38162121
ENSG00000200556	RNU6-103P	grey	0.3092501
ENSG00000206701	RNU6-1040P	grey	0.4300962
ENSG00000222287	RNU6-1043P	grey	0.56271675
ENSG00000252130	RNU6-1045P	grey	0.15546618
ENSG00000251739	RNU6-1053P	grey	0.35425031
ENSG00000252971	RNU6-1057P	grey	0.41518821
ENSG00000252339	RNU6-1061P	grey	0.31295966
ENSG00000252898	RNU6-1096P	grey	0.50973881
ENSG00000200403	RNU6-1099P	grey	0.33120246
ENSG00000252271	RNU6-1110P	grey	0.27600586
ENSG00000202229	RNU6-1138P	grey	0.42525206
ENSG00000201390	RNU6-1141P	grey	0.38963505
ENSG00000252786	RNU6-1142P	grey	0.29739156

ENSG00000222924	RNU6-1148P	grey	0.26653995
ENSG00000222051	RNU6-1165P	grey	0.43183655
ENSG00000200097	RNU6-1167P	grey	0.30284738
ENSG00000206769	RNU6-116P	turquoise	0.60156653
ENSG00000251783	RNU6-1170P	grey	0.34017753
ENSG00000207318	RNU6-1184P	grey	0.51022933
ENSG00000200665	RNU6-1188P	grey	0.30029195
ENSG00000252361	RNU6-118P	grey	0.2709193
ENSG00000252017	RNU6-1194P	grey	0.52453739
ENSG00000207198	RNU6-1195P	grey	0.42778901
ENSG00000252030	RNU6-1196P	grey	0.28295263
ENSG00000206889	RNU6-1200P	grey	0.27417573
ENSG00000238482	RNU6-1208P	grey	0.58207153
ENSG00000200520	RNU6-1214P	grey	0.47466801
ENSG00000207345	RNU6-1222P	grey	0.46923859
ENSG00000252145	RNU6-1225P	grey	0.57062281
ENSG00000252627	RNU6-122P	grey	0.41373589
ENSG00000252686	RNU6-1234P	grey	0.42523333
ENSG00000207508	RNU6-1237P	grey	0.50923474
ENSG00000251972	RNU6-123P	grey	0.39148151
ENSG00000202025	RNU6-1240P	grey	0.30427136
ENSG00000252431	RNU6-1247P	grey	0.59290006
ENSG00000200304	RNU6-1255P	grey	0.58858316
ENSG00000252720	RNU6-1258P	grey	0.09502826
ENSG00000207234	RNU6-125P	grey	0.14279921
ENSG00000252026	RNU6-1262P	grey	0.31200235
ENSG00000252494	RNU6-126P	grey	0.38926146
ENSG00000202081	RNU6-1280P	grey	0.4389142
ENSG00000202184	RNU6-1283P	grey	0.29412763
ENSG00000200571	RNU6-1284P	grey	0.47333284
ENSG00000200350	RNU6-1285P	grey	0.48458311
ENSG00000206703	RNU6-128P	grey	0.59885001
ENSG00000199594	RNU6-1301P	grey	0.44045191
ENSG00000202089	RNU6-1306P	grey	0.29236191
ENSG00000223044	RNU6-130P	grey	0.35986296
ENSG00000206969	RNU6-1316P	grey	0.49609016
ENSG00000212140	RNU6-1320P	grey	0.38320412
ENSG00000201179	RNU6-1322P	grey	0.60061739
ENSG00000206949	RNU6-1324P	grey	0.38948727
ENSG00000252334	RNU6-1337P	grey	0.34686
ENSG00000252578	RNU6-135P	grey	0.49671599
ENSG00000200550	RNU6-137P	grey	0.41558477
ENSG00000222431	RNU6-141P	grey	0.48729333
ENSG00000206746	RNU6-142P	grey	0.17674239
ENSG00000252751	RNU6-143P	grey	0.52140164
ENSG00000201028	RNU6-151P	grey	0.3846288
ENSG00000206684	RNU6-157P	grey	0.44850302
ENSG00000212382	RNU6-159P	grey	0.33653147

ENSG00000207082	RNU6-171P	grey	0.40662888
ENSG00000207361	RNU6-178P	grey	0.41242931
ENSG00000252779	RNU6-182P	grey	0.3980295
ENSG00000206881	RNU6-190P	grey	0.41172214
ENSG00000223284	RNU6-195P	grey	0.29252779
ENSG00000199824	RNU6-199P	grey	0.34038193
ENSG00000200648	RNU6-226P	grey	0.31351087
ENSG00000199570	RNU6-228P	grey	0.35363146
ENSG00000200183	RNU6-238P	grey	0.45290757
ENSG00000251907	RNU6-240P	grey	0.43231392
ENSG00000206712	RNU6-26P	grey	0.22630909
ENSG00000252081	RNU6-277P	grey	0.39808116
ENSG00000200560	RNU6-288P	grey	0.40822204
ENSG00000223191	RNU6-293P	grey	0.26911278
ENSG00000202119	RNU6-302P	grey	0.4014164
ENSG00000207291	RNU6-30P	grey	0.50897593
ENSG00000207385	RNU6-310P	grey	0.377558
ENSG00000201499	RNU6-312P	grey	0.53928582
ENSG00000252383	RNU6-314P	grey	0.10225442
ENSG00000212329	RNU6-316P	grey	0.26057073
ENSG00000222844	RNU6-321P	grey	0.22782267
ENSG00000251819	RNU6-322P	grey	0.28198918
ENSG00000212189	RNU6-328P	grey	0.46989408
ENSG00000206675	RNU6-32P	grey	0.49951084
ENSG00000252782	RNU6-341P	grey	0.42686248
ENSG00000207251	RNU6-342P	grey	0.48403259
ENSG00000252717	RNU6-352P	grey	0.58140692
ENSG00000201136	RNU6-353P	grey	0.16094423
ENSG00000252373	RNU6-358P	grey	0.51894695
ENSG00000252922	RNU6-365P	grey	0.2769063
ENSG00000252980	RNU6-367P	grey	0.36925654
ENSG00000201080	RNU6-372P	blue	-0.392644
ENSG00000252859	RNU6-375P	grey	0.32118827
ENSG00000251774	RNU6-377P	grey	0.14425478
ENSG00000200683	RNU6-379P	grey	0.53792517
ENSG00000199562	RNU6-37P	grey	0.27952751
ENSG00000252035	RNU6-397P	grey	0.40812028
ENSG00000251777	RNU6-404P	grey	0.55785889
ENSG00000207180	RNU6-411P	grey	0.14643553
ENSG00000252061	RNU6-415P	grey	0.18739218
ENSG00000206762	RNU6-418P	grey	0.25553233
ENSG00000206604	RNU6-425P	grey	0.28101199
ENSG00000207312	RNU6-429P	grey	0.49985902
ENSG00000206601	RNU6-431P	grey	0.43955933
ENSG00000200086	RNU6-433P	grey	0.38683194
ENSG00000206766	RNU6-435P	grey	0.5544335
ENSG00000251980	RNU6-436P	grey	0.338061
ENSG00000206715	RNU6-444P	grey	0.53232477

ENSG00000199697	RNU6-446P	grey	0.45598171
ENSG00000252861	RNU6-448P	grey	0.3242508
ENSG00000207412	RNU6-455P	grey	0.44443098
ENSG00000200869	RNU6-457P	grey	0.07175875
ENSG00000207200	RNU6-45P	grey	0.4667999
ENSG00000212460	RNU6-460P	grey	0.32530754
ENSG00000206921	RNU6-481P	grey	0.39624595
ENSG00000212370	RNU6-482P	grey	0.4607542
ENSG00000206815	RNU6-483P	grey	0.26124181
ENSG00000206743	RNU6-484P	grey	0.50965844
ENSG00000199865	RNU6-495P	grey	0.40906848
ENSG00000252338	RNU6-503P	grey	0.5717364
ENSG00000252377	RNU6-504P	grey	0.40255581
ENSG00000199226	RNU6-50P	grey	0.40982463
ENSG00000223313	RNU6-516P	grey	0.3206041
ENSG00000201367	RNU6-522P	grey	0.40007035
ENSG00000212482	RNU6-530P	grey	0.33602646
ENSG00000252503	RNU6-531P	grey	0.41610067
ENSG00000200254	RNU6-536P	grey	0.60583436
ENSG00000207352	RNU6-540P	grey	0.20003399
ENSG00000252892	RNU6-548P	grey	0.31410312
ENSG00000200917	RNU6-553P	grey	0.50657337
ENSG00000222915	RNU6-564P	grey	0.23643201
ENSG00000206992	RNU6-574P	grey	0.36291834
ENSG00000223258	RNU6-575P	grey	0.42201924
ENSG00000252756	RNU6-577P	grey	0.17468454
ENSG00000223280	RNU6-57P	grey	0.52265666
ENSG00000202029	RNU6-580P	grey	0.53866553
ENSG00000252023	RNU6-581P	grey	0.35999205
ENSG00000252294	RNU6-589P	grey	0.4029256
ENSG00000201586	RNU6-593P	grey	0.39344792
ENSG00000200814	RNU6-595P	grey	0.28550723
ENSG00000223335	RNU6-603P	grey	0.3828574
ENSG00000207452	RNU6-606P	grey	0.49906356
ENSG00000201662	RNU6-60P	grey	0.48942721
ENSG00000207003	RNU6-611P	grey	0.44942635
ENSG00000222344	RNU6-613P	grey	0.24708663
ENSG00000207081	RNU6-616P	grey	0.31589487
ENSG00000202016	RNU6-619P	grey	0.37924521
ENSG00000201622	RNU6-621P	grey	0.49682207
ENSG00000238658	RNU6-625P	grey	0.44276834
ENSG00000252391	RNU6-638P	grey	0.28823416
ENSG00000212457	RNU6-644P	grey	0.28534874
ENSG00000252928	RNU6-64P	grey	0.46072379
ENSG00000202358	RNU6-652P	grey	0.46206736
ENSG00000199944	RNU6-653P	grey	0.42911175
ENSG00000207336	RNU6-658P	grey	0.43235062
ENSG00000252263	RNU6-659P	grey	0.18419335

ENSG00000252995	RNU6-667P	grey	0.29555245
ENSG00000253095	RNU6-676P	grey	0.38197077
ENSG00000252641	RNU6-678P	grey	0.40519399
ENSG00000200882	RNU6-681P	grey	0.20201085
ENSG00000206924	RNU6-689P	grey	0.234223
ENSG00000212136	RNU6-696P	grey	0.35734974
ENSG00000200218	RNU6-697P	grey	0.49274741
ENSG00000252055	RNU6-69P	grey	0.53909
ENSG00000206786	RNU6-701P	grey	0.3696177
ENSG00000252172	RNU6-720P	grey	0.28311381
ENSG00000206700	RNU6-723P	grey	0.33048503
ENSG00000207128	RNU6-729P	grey	0.14547827
ENSG00000252245	RNU6-736P	grey	0.51120927
ENSG00000202159	RNU6-742P	grey	0.38653391
ENSG00000222266	RNU6-757P	grey	0.4044312
ENSG00000252549	RNU6-759P	grey	0.44995703
ENSG00000251887	RNU6-760P	grey	0.47816645
ENSG00000252916	RNU6-762P	grey	0.59023866
ENSG00000206859	RNU6-767P	grey	0.53978101
ENSG00000212332	RNU6-780P	grey	0.41649244
ENSG00000252658	RNU6-786P	grey	0.4182265
ENSG00000207208	RNU6-790P	grey	0.41468298
ENSG00000252132	RNU6-795P	grey	0.47315006
ENSG00000202513	RNU6-805P	grey	0.46977328
ENSG00000212535	RNU6-808P	grey	0.55302426
ENSG00000199237	RNU6-834P	grey	0.37581345
ENSG00000253084	RNU6-840P	grey	0.51826704
ENSG00000252518	RNU6-846P	grey	0.31130842
ENSG00000201176	RNU6-853P	grey	0.4083975
ENSG00000252768	RNU6-856P	grey	0.41008733
ENSG00000199260	RNU6-874P	grey	0.34199124
ENSG00000252297	RNU6-875P	grey	0.43214463
ENSG00000201198	RNU6-879P	grey	0.40106231
ENSG00000252416	RNU6-885P	grey	0.43416879
ENSG00000207046	RNU6-886P	grey	0.36268317
ENSG00000206848	RNU6-890P	grey	0.34136208
ENSG00000222267	RNU6-892P	grey	0.22192814
ENSG00000252944	RNU6-897P	grey	0.25293347
ENSG00000252015	RNU6-904P	grey	0.35767196
ENSG00000201813	RNU6-915P	grey	0.37818992
ENSG00000199796	RNU6-924P	grey	0.21344735
ENSG00000207359	RNU6-925P	grey	0.20940148
ENSG00000222303	RNU6-935P	grey	0.33155854
ENSG00000206732	RNU6-936P	grey	0.27796924
ENSG00000199872	RNU6-942P	grey	0.44493245
ENSG00000206605	RNU6-946P	grey	0.52986581
ENSG00000223287	RNU6-954P	grey	0.11979727
ENSG00000200522	RNU6-957P	grey	0.34896032

ENSG00000252220	RNU6-977P	grey	0.35940854
ENSG00000206623	RNU6-979P	grey	0.4318472
ENSG00000200257	RNU6-97P	grey	0.33724018
ENSG00000200003	RNU6-986P	grey	0.5344494
ENSG00000207490	RNU6-987P	grey	0.48355668
ENSG00000221676	RNU6ATAC	grey	0.49460687
ENSG00000221562	RNU6ATAC10P	grey	0.16874029
ENSG00000252351	RNU6ATAC12P	grey	0.23682885
ENSG00000221518	RNU6ATAC16P	grey	0.39926642
ENSG00000252620	RNU6ATAC24P	grey	0.44495883
ENSG00000221571	RNU6ATAC35P	grey	0.33770394
ENSG00000252118	RNU6ATAC39P	grey	0.1760466
ENSG00000221564	RNU6ATAC42P	grey	0.32328631
ENSG00000206832	RNU6V	grey	0.47838835
ENSG00000252698	RNU7-101P	grey	0.34100281
ENSG00000238370	RNU7-103P	grey	0.314341
ENSG00000238523	RNU7-107P	grey	0.48324332
ENSG00000253062	RNU7-108P	grey	0.40393253
ENSG00000252579	RNU7-117P	grey	0.5415292
ENSG00000238310	RNU7-122P	grey	0.33157238
ENSG00000251720	RNU7-123P	grey	0.29707196
ENSG00000251745	RNU7-124P	grey	0.5214023
ENSG00000251964	RNU7-135P	grey	0.4483792
ENSG00000238427	RNU7-136P	grey	0.35228258
ENSG00000238777	RNU7-141P	grey	0.47947687
ENSG00000238357	RNU7-148P	grey	0.40973431
ENSG00000252739	RNU7-151P	grey	0.54292349
ENSG00000252702	RNU7-158P	grey	0.3470295
ENSG00000238924	RNU7-163P	grey	0.51412797
ENSG00000252934	RNU7-172P	grey	0.38057792
ENSG00000253043	RNU7-181P	grey	0.46280237
ENSG00000239102	RNU7-185P	grey	0.52136092
ENSG00000238998	RNU7-187P	grey	0.442634
ENSG00000252174	RNU7-18P	grey	0.43893367
ENSG00000238721	RNU7-194P	grey	0.26248278
ENSG00000239151	RNU7-195P	grey	0.38708021
ENSG00000239099	RNU7-23P	grey	0.49635799
ENSG00000251707	RNU7-37P	grey	0.54212195
ENSG00000238446	RNU7-38P	grey	0.43012039
ENSG00000238829	RNU7-45P	grey	0.2822973
ENSG00000238830	RNU7-46P	grey	0.42078197
ENSG00000251787	RNU7-47P	grey	0.52857384
ENSG00000238386	RNU7-48P	grey	0.27200743
ENSG00000252770	RNU7-4P	grey	0.567741
ENSG00000238365	RNU7-57P	grey	0.48400358
ENSG00000238880	RNU7-59P	grey	0.48112291
ENSG00000251747	RNU7-60P	grey	0.41135162
ENSG00000238417	RNU7-63P	grey	0.37685307

ENSG00000260764	RNU7-63P	grey	0.22849754
ENSG00000251868	RNU7-71P	grey	0.44701607
ENSG00000238606	RNU7-7P	grey	0.56193651
ENSG00000238884	RNU7-85P	grey	0.51746647
ENSG00000238500	RNU7-87P	grey	0.31627079
ENSG00000251767	RNU7-8P	grey	0.25745298
ENSG00000252966	RNU7-91P	grey	0.21363928
ENSG00000238719	RNU7-96P	grey	0.4666921
ENSG00000252080	RNU7-99P	grey	0.54903755
ENSG00000238782	RNU7-9P	grey	0.38912831
ENSG00000201690	RNY1P2	grey	0.40743787
ENSG00000223158	RNY1P3	grey	0.48866526
ENSG00000207325	RNY1P4	grey	0.53604926
ENSG00000199788	RNY3P2	grey	0.52246154
ENSG00000200686	RNY3P3	turquoise	0.40038178
ENSG00000201818	RNY4P17	grey	0.48741147
ENSG00000238711	RNY4P25	grey	0.34546599
ENSG00000201470	RNY4P7	grey	0.61338347
ENSG00000200735	RNY4P8	grey	0.52102954
ENSG00000202129	RNY5P1	grey	0.56805533
ENSG00000222411	RNY5P3	grey	0.52155344
ENSG00000222301	RNY5P8	grey	0.5640986
ENSG00000169855	ROBO1	red	0.72241164
ENSG00000185008	ROBO2	blue	0.64826618
ENSG00000154134	ROBO3	grey	-0.1791434
ENSG00000154133	ROBO4	tan	0.83505685
ENSG00000067900	ROCK1	green	0.78807936
ENSG00000134318	ROCK2	black	0.69643593
ENSG00000067836	ROGDI	turquoise	-0.5215174
ENSG00000149489	ROM1	blue	-0.6729056
ENSG00000125995	ROMO1	grey	-0.3757452
ENSG00000114547	ROPN1B	grey	0.00611211
ENSG00000185483	ROR1	grey	0.23121186
ENSG00000069667	RORA	darkmagenta	-0.8417238
ENSG00000198963	RORB	red	0.63995176
ENSG00000235869	RP1-102G20.4	grey	0.26610045
ENSG00000229531	RP1-102G20.5	grey	0.1621877
ENSG00000226524	RP1-102H19.8	grey	0.30522532
ENSG00000224849	RP1-104O17.1	grey	0.01580346
ENSG00000242486	RP1-104O17.2	grey	0.22596977
ENSG00000226207	RP1-104O17.3	grey	0.10495841
ENSG00000228408	RP1-111D6.3	grey	-0.1593933
ENSG00000236065	RP1-117O3.2	grey	0.23708234
ENSG00000220695	RP1-121G13.3	grey	0.28929108
ENSG00000212663	RP1-122K4.3	grey	0.45757201
ENSG00000233895	RP1-122P22.2	green	0.54426093
ENSG00000255692	RP1-127H14.3	grey	0.43463209
ENSG00000238055	RP1-128O3.6	grey	0.5654929

ENSG00000237502	RP1-12G14.6	grey	0.33819818
ENSG00000254537	RP1-130L23.1	grey	0.25999423
ENSG00000233044	RP1-137K2.2	grey	0.50001792
ENSG00000231050	RP1-140A9.1	grey	0.06377355
ENSG00000254708	RP1-145M24.1	grey	0.3505799
ENSG00000237994	RP1-146A15.1	grey	-0.0558129
ENSG00000234626	RP1-149A16.12	grey	0.43124245
ENSG00000232218	RP1-149A16.16	grey	0.04227418
ENSG00000229931	RP1-151F17.1	grey	0.14794537
ENSG00000236485	RP1-156L9.1	grey	0.15804304
ENSG00000237429	RP1-159A19.4	grey	0.2102801
ENSG00000235357	RP1-159G19.1	grey	-0.1475293
ENSG00000232599	RP1-161N10.1	grey	0.10989376
ENSG00000234557	RP1-164F3.8	grey	0.18756358
ENSG00000261692	RP1-168P16.1	grey	0.14130085
ENSG00000261574	RP1-168P16.2	grey	0.35393082
ENSG00000260647	RP1-178F10.1	grey	0.19559976
ENSG00000267350	RP1-178F10.3	grey	0.13148462
ENSG00000237719	RP1-179N16.3	grey	0.48144878
ENSG00000246982	RP1-179N16.6	yellow	0.4624586
ENSG00000254584	RP1-17K7.2	grey	0.20172104
ENSG00000217239	RP1-182O16.1	grey	0.48827666
ENSG00000216360	RP1-182O16.2	grey	0.1464467
ENSG00000228997	RP1-186E20.1	grey	0.39255702
ENSG00000257433	RP1-197B17.3	grey	0.16773165
ENSG00000226648	RP1-1J6.2	grey	0.41206991
ENSG00000234604	RP1-206D15.5	grey	0.43245216
ENSG00000230433	RP1-20B11.2	grey	0.22683172
ENSG00000229036	RP1-20N2.6	grey	0.18808695
ENSG00000228793	RP1-223B1.1	red	-0.526926
ENSG00000261071	RP1-223E5.4	grey	-0.094593
ENSG00000260920	RP1-228H13.5	blue	0.52221484
ENSG00000240399	RP1-228P16.1	grey	0.09722934
ENSG00000226138	RP1-228P16.7	grey	0.38148538
ENSG00000226436	RP1-22N22.1	grey	0.40194657
ENSG00000231533	RP1-232L24.3	grey	0.01055765
ENSG00000269985	RP1-232P20.1	grey	0.35552333
ENSG00000225793	RP1-234P15.4	grey	0.27323924
ENSG00000260196	RP1-239B22.5	brown	0.51766023
ENSG00000232311	RP1-249I4.2	grey	-0.0202088
ENSG00000231496	RP1-251M9.2	black	0.50368567
ENSG00000228027	RP1-251M9.3	grey60	0.48796418
ENSG00000270174	RP1-256G22.2	grey	0.2570435
ENSG00000270083	RP1-257I20.14	grey	0.30639038
ENSG00000259079	RP1-261D10.1	grey	0.1915254
ENSG00000259146	RP1-261D10.2	grey	0.11088868
ENSG00000219392	RP1-265C24.5	green	0.63137733
ENSG00000232640	RP1-266L20.2	grey	0.01779768

ENSG00000255575	RP1-267D11.1	grey	0.5164011
ENSG00000258323	RP1-267L14.3	grey	0.19687702
ENSG00000233508	RP1-269M15.3	grey	-0.0429608
ENSG00000249379	RP1-27K12.4	grey	0.41836007
ENSG00000219553	RP1-281H8.3	grey	0.20726127
ENSG00000227775	RP1-283E3.4	grey	0.00230874
ENSG00000268575	RP1-283E3.8	grey	0.12226068
ENSG00000234567	RP1-283K11.2	grey	0.22634502
ENSG00000223542	RP1-283K11.3	grey	0.18267808
ENSG00000234688	RP1-293L6.1	grey	0.1979504
ENSG00000255207	RP1-305G21.1	grey	0.29364144
ENSG00000229820	RP1-308E4.1	grey	0.56390995
ENSG00000225689	RP1-30E17.2	grey	0.02870057
ENSG00000232412	RP1-315G1.3	grey	0.09641903
ENSG00000231121	RP1-34H18.1	grey	0.1498602
ENSG00000235347	RP1-37M3.8	grey	0.34005772
ENSG00000259943	RP1-39G22.7	grey	-0.4321934
ENSG00000230269	RP1-40E16.9	blue	0.45042682
ENSG00000230424	RP1-43E13.2	yellow	0.32618007
ENSG00000227192	RP1-45I4.3	grey	-0.0073691
ENSG00000226698	RP1-50O24.6	darkred	0.58066685
ENSG00000263466	RP1-56K13.2	grey	0.3946102
ENSG00000263345	RP1-59D14.5	grey	0.28045007
ENSG00000229955	RP1-5O6.4	grey	0.11632468
ENSG00000235246	RP1-5O6.5	grey	0.26633224
ENSG00000229472	RP1-64K7.4	grey	0.37994132
ENSG00000234206	RP1-67A8.3	grey	0.17227481
ENSG00000237851	RP1-67K17.4	grey	0.3330289
ENSG00000255004	RP1-68D18.3	turquoise	0.49875069
ENSG00000226149	RP1-69D17.4	black	-0.4299145
ENSG00000257452	RP1-71H24.1	grey	0.12840295
ENSG00000225660	RP1-73A14.1	green	0.51784885
ENSG00000217120	RP1-76C18.1	grey	0.33470493
ENSG00000257894	RP1-78O14.1	grey	0.14206223
ENSG00000182404	RP1-86D1.3	blue	-0.4838557
ENSG00000227112	RP1-86D1.4	blue	-0.5726068
ENSG00000224733	RP1-86D1.5	brown	-0.4242791
ENSG00000231769	RP1-8B1.4	grey	0.01001835
ENSG00000242082	RP1-90G24.10	grey	0.17142113
ENSG00000224050	RP1-90G24.6	grey	0.08250088
ENSG00000261015	RP1-90J20.11	grey	-0.1322971
ENSG00000270346	RP1-90J20.12	grey	0.2213286
ENSG00000223704	RP1-90L6.2	grey	0.00658323
ENSG00000235271	RP1-90L6.3	grey	0.12470386
ENSG00000234694	RP1-92O14.3	grey	0.32494408
ENSG00000229431	RP1-92O14.6	grey	0.28600103
ENSG00000217648	RP1-95L4.4	grey	0.4250101
ENSG00000257835	RP1-97G4.1	grey	0.16385295

ENSG00000241818	RP11-1000B6.2	grey	0.14718454
ENSG00000261064	RP11-1000B6.3	grey	0.0447473
ENSG00000254131	RP11-1007J8.1	grey	0.21731002
ENSG00000261187	RP11-1007O24.2	grey	0.02201448
ENSG00000261423	RP11-1007O24.3	blue	-0.3936389
ENSG00000236914	RP11-1008C21.2	grey	0.032042
ENSG00000259168	RP11-100M12.3	magenta	0.44429041
ENSG00000259013	RP11-1017G21.3	grey	0.64298379
ENSG00000258959	RP11-1017G21.4	grey	0.28965518
ENSG00000260277	RP11-101E7.2	grey	0.28061674
ENSG00000269894	RP11-1020A11.1	grey	0.22063771
ENSG00000269982	RP11-1020A11.2	grey	-0.0589587
ENSG00000215158	RP11-1023L17.1	yellow	0.80504705
ENSG00000215156	RP11-1023L17.2	greenyellow	-0.4706784
ENSG00000253924	RP11-1023P17.2	yellow	0.73544904
ENSG00000249109	RP11-1026M7.2	grey	-0.0219011
ENSG00000255780	RP11-1029F8.1	grey	0.43854564
ENSG00000258404	RP11-1029J19.5	grey	0.09108812
ENSG00000259326	RP11-102L12.2	grey	0.11186242
ENSG00000240695	RP11-102M11.1	grey	0.10257044
ENSG00000227945	RP11-103C16.2	grey	0.12846062
ENSG00000244668	RP11-103G8.1	grey	0.09487588
ENSG00000240137	RP11-103G8.2	grey	-0.1124418
ENSG00000230317	RP11-104D21.3	brown	0.34177427
ENSG00000229273	RP11-104G3.2	grey	0.30082139
ENSG00000261118	RP11-104N10.1	grey	0.0214455
ENSG00000261868	RP11-104O19.2	grey	0.35100808
ENSG00000258536	RP11-1053O12.1	grey	0.22718039
ENSG00000262223	RP11-1055B8.3	yellow	0.65051033
ENSG00000262877	RP11-1055B8.4	yellow	0.64912778
ENSG00000263053	RP11-1055B8.6	grey	-0.0183256
ENSG00000171282	RP11-1055B8.7	grey	-0.3012933
ENSG00000260905	RP11-105C19.1	grey	0.45030354
ENSG00000257283	RP11-1060G2.1	grey	0.38703026
ENSG00000256557	RP11-1060J15.3	grey	0.40572605
ENSG00000228484	RP11-106M7.1	turquoise	0.44080031
ENSG00000244159	RP11-1070A24.1	grey	0.28869441
ENSG00000267160	RP11-1072C15.4	grey	0.35895755
ENSG00000258935	RP11-1078H9.2	grey	0.1719565
ENSG00000259163	RP11-1078H9.5	grey	0.45392758
ENSG00000248714	RP11-1079K10.3	grey	0.24795012
ENSG00000261598	RP11-107D24.2	grey	0.19785428
ENSG00000260814	RP11-107F6.3	grey	0.43262824
ENSG00000270702	RP11-107F6.4	grey	0.29669708
ENSG00000225208	RP11-107I14.2	grey	0.00764798
ENSG00000230967	RP11-107I14.5	grey	-0.0087925
ENSG00000253697	RP11-1081K18.1	grey	0.46479909
ENSG00000254966	RP11-1081L13.4	grey	0.32417387

ENSG00000243094	RP11-1084A12.1	grey	0.49832182
ENSG00000259468	RP11-1084A12.2	grey	0.33433768
ENSG00000258428	RP11-1085N6.2	grey	0.22736603
ENSG00000259194	RP11-108K3.4	yellow	0.58163996
ENSG00000270378	RP11-108K3.5	grey	0.28719381
ENSG00000271385	RP11-108M11.3	grey	0.2529135
ENSG00000238142	RP11-108M9.4	grey	0.16853994
ENSG00000267321	RP11-1094M14.1	green	-0.5711538
ENSG00000266368	RP11-1096G20.5	grey	0.22676969
ENSG00000227374	RP11-109A6.3	grey	0.41239665
ENSG00000260600	RP11-109D24.1	grey	0.15032862
ENSG00000235020	RP11-109I13.2	grey	0.05346071
ENSG00000258448	RP11-109N23.5	grey	-0.03958
ENSG00000259015	RP11-109N23.6	grey	0.26160559
ENSG00000253932	RP11-10C8.2	yellow	0.47364864
ENSG00000242103	RP11-10G15.4	grey	-0.0486844
ENSG00000267713	RP11-10I6.2	grey	0.39221483
ENSG00000245213	RP11-10K16.1	grey	0.05894065
ENSG00000224207	RP11-10L12.1	grey	0.21745035
ENSG00000251288	RP11-10L12.2	grey	0.30472327
ENSG00000246560	RP11-10L12.4	grey	0.27046293
ENSG00000246375	RP11-10L7.1	grey	0.14183137
ENSG00000260103	RP11-10O17.1	grey	0.27441428
ENSG00000241168	RP11-10O22.1	grey	0.0434079
ENSG00000257636	RP11-1103G16.1	grey	0.13004241
ENSG00000258172	RP11-1105G2.4	grey	0.3982536
ENSG00000253270	RP11-1105O14.1	grey	0.41453485
ENSG00000271327	RP11-1109F11.3	turquoise	0.47665836
ENSG00000271259	RP11-1109F11.5	grey	0.3455045
ENSG00000228801	RP11-110G21.1	grey	0.19116039
ENSG00000253475	RP11-110G21.2	grey	0.37095894
ENSG00000254428	RP11-110I1.11	grey	0.30769531
ENSG00000242986	RP11-1110J8.1	grey	0.38466065
ENSG00000266179	RP11-1113L8.6	grey	0.33563175
ENSG00000225400	RP11-1114A5.5	grey	0.24382934
ENSG00000253659	RP11-1114I9.1	grey	-0.1959283
ENSG00000258526	RP11-111A21.1	grey	0.32367094
ENSG00000231995	RP11-111F5.2	grey	0.11670928
ENSG00000231212	RP11-111F5.3	grey	0.04725455
ENSG00000204802	RP11-111F5.4	grey	0.27519889
ENSG00000226429	RP11-111F5.6	grey	0.22523012
ENSG00000260331	RP11-111J6.2	grey	0.09705314
ENSG00000261019	RP11-111K18.2	grey	0.09872593
ENSG00000179240	RP11-111M22.2	blue	0.55525503
ENSG00000255135	RP11-111M22.3	grey	-0.1886394
ENSG00000227388	RP11-112J3.16	brown	0.60330319
ENSG00000231742	RP11-112L6.4	grey	0.17479332
ENSG00000254348	RP11-1134I14.4	green	-0.3981616

ENSG00000241723	RP11-113A11.1	grey	0.41053546
ENSG00000256552	RP11-113C12.4	grey	-0.0282556
ENSG00000254522	RP11-113K21.1	grey	0.44383748
ENSG00000255503	RP11-113K21.4	grey	0.32415627
ENSG00000259055	RP11-1140I5.1	grey	0.46246617
ENSG00000228343	RP11-1148L6.5	grey	0.16957492
ENSG00000254177	RP11-1149M10.1	grey	0.40195922
ENSG00000257438	RP11-114F10.3	grey	-0.0402918
ENSG00000259792	RP11-114H24.6	grey	0.30667326
ENSG00000250031	RP11-114M5.1	grey	0.24786883
ENSG00000258999	RP11-114N19.3	grey	0.27295508
ENSG00000271320	RP11-1152H14.1	grey	0.36283251
ENSG00000254607	RP11-115C10.1	grey	0.17006194
ENSG00000246089	RP11-115C21.2	grey	0.17365714
ENSG00000253550	RP11-115C21.4	grey	0.23834409
ENSG00000258337	RP11-115H15.2	grey	0.31121383
ENSG00000226324	RP11-115M14.1	grey	0.33666671
ENSG00000267088	RP11-115N12.1	grey	0.10857424
ENSG00000267585	RP11-115P21.1	brown	-0.3633616
ENSG00000253383	RP11-116B19.2	grey	0.38637407
ENSG00000258342	RP11-116N8.1	yellow	0.46565544
ENSG00000257826	RP11-116N8.4	grey	-0.0922143
ENSG00000267193	RP11-116O18.3	greenyellow	-0.4067968
ENSG00000253536	RP11-1174L13.2	grey	0.2270852
ENSG00000257169	RP11-117N2.2	grey	0.45200059
ENSG00000259174	RP11-1180F24.1	grey	0.3789827
ENSG00000256427	RP11-118B22.4	grey	-0.0097459
ENSG00000266100	RP11-118E18.2	grey	0.20010266
ENSG00000270124	RP11-118F19.1	grey	-0.0938709
ENSG00000265689	RP11-118G23.1	grey	-0.117203
ENSG00000264643	RP11-118G23.2	grey	0.10429642
ENSG00000251556	RP11-118M9.3	cyan	-0.4558981
ENSG00000242686	RP11-1191J2.2	grey	0.0132126
ENSG00000262339	RP11-1197K16.2	grey	0.44540268
ENSG00000229539	RP11-119B16.2	grey	0.16883412
ENSG00000251637	RP11-119D9.1	grey	0.08775838
ENSG00000226381	RP11-119F19.2	grey	0.38950612
ENSG00000260400	RP11-119F7.5	grey	0.0359374
ENSG00000241002	RP11-119H12.1	grey	0.17024791
ENSG00000234841	RP11-119H12.4	grey	0.13943246
ENSG00000251126	RP11-119H12.6	grey	0.34438146
ENSG00000247134	RP11-11N9.4	grey	0.11350689
ENSG00000250243	RP11-120A1.1	turquoise	-0.4817572
ENSG00000225472	RP11-120J1.1	turquoise	0.68271523
ENSG00000267868	RP11-120K24.3	grey	0.04878979
ENSG00000269356	RP11-120K24.4	grey	0.21677788
ENSG00000267009	RP11-120M18.2	grey	0.16312846
ENSG00000235204	RP11-121A14.2	grey	0.42626944

ENSG00000259959	RP11-121C2.2	grey	0.34176915
ENSG00000257467	RP11-121G22.3	grey	-0.0031128
ENSG00000259280	RP11-122D10.1	grey	0.03617396
ENSG00000235477	RP11-122G18.5	grey	0.33628373
ENSG00000270235	RP11-122K13.14	brown	0.56585987
ENSG00000231057	RP11-122M14.1	grey	-0.0914932
ENSG00000254923	RP11-1236K1.8	grey	0.18942751
ENSG00000182625	RP11-123C21.1	blue	-0.4681804
ENSG00000270986	RP11-123C21.2	grey	0.38308476
ENSG00000225078	RP11-123N4.4	grey	0.16367349
ENSG00000257083	RP11-123O10.3	grey	0.36338023
ENSG00000256248	RP11-123O10.4	grey	0.32028952
ENSG00000239627	RP11-124A7.1	yellow	0.49053072
ENSG00000250012	RP11-124N2.1	grey	0.33555977
ENSG00000251460	RP11-1258F18.1	grey	0.17345203
ENSG00000236404	RP11-125B21.2	red	-0.7174665
ENSG00000237976	RP11-126K1.6	grey	0.16940897
ENSG00000234432	RP11-1275H24.1	grey	0.07827725
ENSG00000247679	RP11-1277A3.1	grey	0.09389438
ENSG00000246596	RP11-1277A3.2	grey	0.10163612
ENSG00000253350	RP11-127H5.1	grey	0.15936126
ENSG00000228261	RP11-127L20.3	grey	-0.0785814
ENSG00000260641	RP11-1299A16.3	yellow	0.42599387
ENSG00000238099	RP11-12A2.3	turquoise	0.35708774
ENSG00000236233	RP11-12A20.7	grey	0.01541166
ENSG00000231967	RP11-12D24.6	greenyellow	-0.4080735
ENSG00000228127	RP11-12L8.1	grey	0.07282382
ENSG00000271143	RP11-12L8.2	grey	0.27913948
ENSG00000250131	RP11-130F10.1	grey	0.23224344
ENSG00000269976	RP11-130L8.2	grey	0.29147716
ENSG00000248750	RP11-131K17.1	grey	0.21793191
ENSG00000265494	RP11-131K5.2	grey	0.10404472
ENSG00000223653	RP11-131L23.1	grey	0.33210062
ENSG00000248996	RP11-1334A24.6	grey	0.18945478
ENSG00000249690	RP11-1336O20.2	grey	0.23447412
ENSG00000233464	RP11-133I21.2	grey	0.23123969
ENSG00000257964	RP11-133N21.10	grey	0.11987507
ENSG00000235481	RP11-133O22.6	grey	0.24843161
ENSG00000251417	RP11-1348G14.4	grey	0.0491113
ENSG00000260796	RP11-1348G14.5	grey	0.34355603
ENSG00000224536	RP11-134G8.7	grey	0.29176972
ENSG00000224818	RP11-134G8.8	grey	0.20672304
ENSG00000212695	RP11-134K1.2	yellow	0.54556165
ENSG00000235389	RP11-134K1.3	grey	0.38717759
ENSG00000231507	RP11-134P9.3	grey	-0.0844846
ENSG00000223842	RP11-135J2.3	grey	0.12967722
ENSG00000228063	RP11-135J2.4	grey	0.230124
ENSG00000250892	RP11-1365D11.1	grey	0.10412453

ENSG00000258225	RP11-136F16.1	grey	0.31325638
ENSG00000257220	RP11-136F16.2	grey	0.27689976
ENSG00000267667	RP11-136H19.1	grey	0.38170001
ENSG00000244945	RP11-1379J22.2	grey	0.30963502
ENSG00000221817	RP11-137L10.6	yellow	0.69236216
ENSG00000264246	RP11-138C24.2	grey	0.24216475
ENSG00000270755	RP11-138E2.1	grey	0.28806745
ENSG00000268873	RP11-138H11.1	turquoise	0.37251611
ENSG00000228704	RP11-138M12.1	grey	0.21599476
ENSG00000250397	RP11-1391J7.1	grey	0.13888016
ENSG00000259688	RP11-139F4.2	grey	0.41950548
ENSG00000225973	RP11-139H15.1	grey	0.06000275
ENSG00000269908	RP11-1406H17.1	grey	0.09165845
ENSG00000174093	RP11-1407O15.2	grey	-0.1939903
ENSG00000235010	RP11-140A10.3	grey	-0.241105
ENSG00000254701	RP11-1415C14.4	grey	0.13805804
ENSG00000270019	RP11-141B14.1	grey	0.42387478
ENSG00000227911	RP11-141M1.1	grey	0.23937951
ENSG00000235450	RP11-142A5.1	grey	0.15829788
ENSG00000260975	RP11-142G1.3	grey	0.02753
ENSG00000259360	RP11-142J21.1	grey	0.19252492
ENSG00000229751	RP11-142M10.2	turquoise	-0.3995447
ENSG00000266371	RP11-142O6.1	grey	0.36081735
ENSG00000266053	RP11-143J12.2	grey	0.43185321
ENSG00000260248	RP11-143K11.1	purple	0.61110908
ENSG00000232830	RP11-144C15.1	grey	0.16142838
ENSG00000241526	RP11-144C9.1	grey	0.44090322
ENSG00000257545	RP11-144F15.1	grey	0.21465048
ENSG00000248510	RP11-145G20.1	brown	0.4560456
ENSG00000269728	RP11-145M9.4	cyan	0.41861116
ENSG00000254491	RP11-145O15.2	yellow	0.52616757
ENSG00000255069	RP11-145O15.3	blue	-0.410495
ENSG00000270523	RP11-145P16.3	brown	-0.4090658
ENSG00000240240	RP11-146D12.2	grey	0.21647456
ENSG00000261840	RP11-146F11.1	grey	0.14517733
ENSG00000232176	RP11-146N23.1	grey	0.26397433
ENSG00000265100	RP11-147L13.2	turquoise	0.44035114
ENSG00000267708	RP11-147L13.7	grey	0.17939385
ENSG00000267731	RP11-147L13.8	grey	-0.0489855
ENSG00000242428	RP11-147N17.1	grey	-0.0496542
ENSG00000232110	RP11-149I23.3	green	0.73911241
ENSG00000263167	RP11-149I9.2	grey	0.17635449
ENSG00000253248	RP11-149P24.1	grey	0.26173597
ENSG00000251203	RP11-14I17.1	grey	0.15882169
ENSG00000254362	RP11-14I17.3	grey	0.4839207
ENSG00000232527	RP11-14N7.2	blue	0.44615624
ENSG00000259585	RP11-150L8.4	grey	0.13188636
ENSG00000228053	RP11-151F5.2	grey	0.39492091

ENSG00000237963	RP11-151G12.2	grey	0.21780545
ENSG00000260661	RP11-152L20.3	grey	0.28046625
ENSG00000227540	RP11-152N13.5	grey	0.10930246
ENSG00000257576	RP11-153M3.1	grey	0.29725047
ENSG00000232295	RP11-154D6.1	brown	-0.4822137
ENSG00000248629	RP11-154F14.2	grey	0.09165262
ENSG00000270562	RP11-154H23.3	grey	0.22363807
ENSG00000240758	RP11-155G14.6	pink	0.71481543
ENSG00000259623	RP11-156E6.1	grey	0.08866326
ENSG00000254054	RP11-156K13.3	cyan	0.40177099
ENSG00000262879	RP11-156P1.3	grey	0.04707045
ENSG00000270207	RP11-157E16.1	grey	0.15785103
ENSG00000255836	RP11-157G21.2	grey	0.09317832
ENSG00000217557	RP11-157L10.1	grey	0.35844949
ENSG00000268573	RP11-158H5.7	yellow	0.39606004
ENSG00000253106	RP11-158K1.3	grey	0.46515728
ENSG00000259407	RP11-158M2.3	grey	0.05143916
ENSG00000259416	RP11-158M2.5	grey	0.18720775
ENSG00000264112	RP11-159D12.2	grey	-0.1636743
ENSG00000229729	RP11-159G9.5	cyan	-0.4404849
ENSG00000246528	RP11-159H10.3	grey	0.13253767
ENSG00000254862	RP11-159H22.2	grey	0.25653991
ENSG00000258558	RP11-159L20.2	grey	0.30277678
ENSG00000237993	RP11-159M11.2	yellow	0.45080692
ENSG00000270179	RP11-159N11.4	grey	0.22781269
ENSG00000267191	RP11-15A1.2	grey	0.41231043
ENSG00000267058	RP11-15A1.3	brown	0.54328043
ENSG00000264269	RP11-15F12.1	grey	0.40856018
ENSG00000269837	RP11-15H20.5	blue	0.58403274
ENSG00000213971	RP11-15H20.6	grey	0.19341173
ENSG00000236252	RP11-15J10.8	grey	0.16653044
ENSG00000261533	RP11-15N24.4	grey	0.07123539
ENSG00000255311	RP11-160H12.2	yellow	0.52636617
ENSG00000254629	RP11-160H12.3	yellow	0.43166906
ENSG00000263470	RP11-160O5.1	grey	0.19825105
ENSG00000257954	RP11-161H23.10	green	0.33155781
ENSG00000187229	RP11-161I2.1	grey	0.14341802
ENSG00000260807	RP11-161M6.2	grey	-0.2393242
ENSG00000264278	RP11-162A12.2	grey	0.20768138
ENSG00000227586	RP11-162A23.5	grey	0.271653
ENSG00000254687	RP11-162D9.3	grey	0.50279728
ENSG00000236514	RP11-162G10.5	grey	0.20621353
ENSG00000236591	RP11-162J8.3	grey	0.4329508
ENSG00000239486	RP11-163E9.1	grey	0.18063047
ENSG00000239969	RP11-163E9.2	grey	0.28868579
ENSG00000251603	RP11-164P12.4	grey	0.20174506
ENSG00000227603	RP11-165J3.6	grey	0.23170709
ENSG00000234719	RP11-166B2.1	grey	0.181959

ENSG00000261216	RP11-166B2.5	grey	0.28928138
ENSG00000255248	RP11-166D19.1	grey	-0.1192693
ENSG00000230358	RP11-166O4.1	grey	0.06446823
ENSG00000228019	RP11-166O4.4	grey	0.35506554
ENSG00000235475	RP11-166O4.5	grey	0.28951515
ENSG00000263004	RP11-166P13.3	grey	0.01170203
ENSG00000263089	RP11-166P13.4	grey	0.05585777
ENSG00000254972	RP11-167J8.3	grey	0.37965243
ENSG00000267834	RP11-167N5.5	grey	0.43857874
ENSG00000226163	RP11-167P22.3	grey	0.41870086
ENSG00000251050	RP11-168A11.4	grey	-0.0024335
ENSG00000249599	RP11-168E14.1	grey	0.17141261
ENSG00000237073	RP11-168K11.2	blue	-0.3275536
ENSG00000236744	RP11-168O22.1	grey	0.03600038
ENSG00000227467	RP11-169D4.1	black	0.4701214
ENSG00000261173	RP11-169E6.1	grey	0.3749073
ENSG00000265883	RP11-169I9.2	black	0.52123129
ENSG00000233954	RP11-169K16.7	grey	0.17216538
ENSG00000179743	RP11-169K16.9	grey	-0.05644
ENSG00000270531	RP11-169K17.2	grey	0.29328836
ENSG00000270965	RP11-169K19.1	grey	0.26125166
ENSG00000264853	RP11-16C1.2	grey	0.15271616
ENSG00000259448	RP11-16E12.1	yellow	0.38767987
ENSG00000261544	RP11-16E23.3	grey	0.37264049
ENSG00000259924	RP11-16E23.4	cyan	-0.4261802
ENSG00000261191	RP11-16L14.2	grey	0.36560558
ENSG00000224644	RP11-16L21.7	grey	0.41004722
ENSG00000260572	RP11-16N11.2	brown	-0.3967796
ENSG00000253875	RP11-16P20.3	grey	0.33304174
ENSG00000261428	RP11-16P6.1	grey	0.1003829
ENSG00000228657	RP11-170J3.2	grey	0.24428508
ENSG00000197476	RP11-170L3.7	grey	0.40936804
ENSG00000240775	RP11-170N16.2	grey	0.44262637
ENSG00000264196	RP11-171G2.1	grey	0.35768402
ENSG00000267784	RP11-171I2.1	grey	0.22067643
ENSG00000271401	RP11-171I2.3	grey	0.13604802
ENSG00000254060	RP11-172E10.1	grey	0.42998211
ENSG00000230058	RP11-172E9.2	grey	0.41419909
ENSG00000267560	RP11-173A16.1	grey	0.23698379
ENSG00000266900	RP11-173A16.2	grey	0.17186217
ENSG00000232059	RP11-173A6.2	grey	0.30300493
ENSG00000228444	RP11-173B14.4	grey	0.11104927
ENSG00000259090	RP11-173D9.5	grey	0.20177726
ENSG00000265683	RP11-173M1.5	grey	0.20676344
ENSG00000264469	RP11-173M1.8	grey	0.3270969
ENSG00000250327	RP11-173M11.2	grey	0.18293326
ENSG00000255946	RP11-173P15.7	grey	0.23826989
ENSG00000214772	RP11-174G6.1	grey	0.47238204

ENSG00000261324	RP11-174G6.5	grey	0.19122972
ENSG00000237578	RP11-174M13.1	salmon	-0.4704332
ENSG00000256137	RP11-174M13.2	grey	0.1920414
ENSG00000236439	RP11-175B9.3	grey	0.23490554
ENSG00000231025	RP11-175O19.4	grey	0.15059109
ENSG00000257489	RP11-175P13.3	grey	0.21019464
ENSG00000228470	RP11-176D17.3	grey	0.3678609
ENSG00000264015	RP11-176N18.2	grey	0.35399056
ENSG00000233519	RP11-177F11.1	grey	0.31882983
ENSG00000267526	RP11-178C3.6	grey	0.16757489
ENSG00000265094	RP11-178F10.1	grey	0.41167227
ENSG00000267957	RP11-178G16.4	yellow	-0.5406517
ENSG00000269772	RP11-178G16.5	grey	0.06897282
ENSG00000261592	RP11-178L8.3	grey	0.43071615
ENSG00000270006	RP11-178L8.7	grey	0.34587343
ENSG00000269901	RP11-178L8.9	grey	0.29649775
ENSG00000224261	RP11-179G5.1	grey	0.21085677
ENSG00000224714	RP11-179K3.2	grey	0.42603706
ENSG00000250039	RP11-17E2.2	greenyellow	-0.4760043
ENSG00000270776	RP11-17G11.1	grey	0.15104067
ENSG00000256746	RP11-17G12.3	grey	0.30349474
ENSG00000265356	RP11-17M24.1	grey	0.18056517
ENSG00000263435	RP11-17M24.2	grey	-0.1582325
ENSG00000270257	RP11-17P16.2	grey	0.31799671
ENSG00000225497	RP11-180I22.2	grey	0.30819008
ENSG00000257004	RP11-180M15.3	grey	0.39541763
ENSG00000271215	RP11-180M15.5	grey	0.33214615
ENSG00000226088	RP11-180O5.2	grey	0.32549878
ENSG00000263931	RP11-180P8.1	grey	0.32999197
ENSG00000265262	RP11-180P8.2	grey	0.4029825
ENSG00000266411	RP11-180P8.3	grey	0.1469194
ENSG00000270324	RP11-180P8.4	grey	0.1943214
ENSG00000254370	RP11-181B11.1	grey	0.06295565
ENSG00000237919	RP11-181B18.1	grey	0.17874892
ENSG00000271590	RP11-181E10.3	grey	0.34808908
ENSG00000182873	RP11-181G12.2	grey	0.0764128
ENSG00000251687	RP11-181K12.2	grey	0.20567698
ENSG00000234784	RP11-182I10.1	grey	0.34964239
ENSG00000259244	RP11-182J1.12	grey	0.35127047
ENSG00000233674	RP11-184I16.3	grey	0.12587008
ENSG00000259963	RP11-185J20.2	grey	0.24890272
ENSG00000228302	RP11-186N15.3	grey	0.10713092
ENSG00000232116	RP11-187C18.2	grey	-0.0607814
ENSG00000259124	RP11-187O7.3	grey	0.25618595
ENSG00000248816	RP11-18D7.3	grey	0.22617817
ENSG00000242622	RP11-18H7.1	grey	0.16745255
ENSG00000269609	RP11-18I14.10	grey	-0.2351993
ENSG00000258178	RP11-18J9.3	red	0.56050135

ENSG00000242880	RP11-190P13.2	grey	0.27306941
ENSG00000258472	RP11-192H23.4	brown	0.6125846
ENSG00000264608	RP11-192H23.8	blue	0.55227892
ENSG00000259381	RP11-192M23.1	grey	0.33306575
ENSG00000267222	RP11-194N12.2	grey	0.50929205
ENSG00000155282	RP11-195B21.3	grey	0.13309994
ENSG00000236021	RP11-195C7.1	grey	0.39005702
ENSG00000254561	RP11-196E1.3	grey	-0.1550865
ENSG00000260911	RP11-196G11.2	grey	0.04527963
ENSG00000262766	RP11-196G11.4	grey	0.21901524
ENSG00000264207	RP11-196G18.23	grey	0.13042712
ENSG00000255886	RP11-196H14.2	grey	0.24190787
ENSG00000255817	RP11-196H14.4	black	0.46120759
ENSG00000242290	RP11-197K3.1	turquoise	0.4634845
ENSG00000223812	RP11-197K6.1	green	-0.5599683
ENSG00000225213	RP11-197M22.2	grey	0.33303146
ENSG00000203321	RP11-197P3.4	grey	0.43519072
ENSG00000229587	RP11-197P3.5	grey	0.42447658
ENSG00000215284	RP11-198M15.1	grey	0.28732843
ENSG00000242429	RP11-19G24.2	grey	0.3109451
ENSG00000225551	RP11-19J3.5	grey	0.3152171
ENSG00000260141	RP11-19N8.4	grey	0.06397183
ENSG00000228625	RP11-1B20.1	grey	0.3554317
ENSG00000253477	RP11-1C8.4	blue	0.54605635
ENSG00000261670	RP11-1C8.5	blue	0.44557945
ENSG00000254330	RP11-1D12.1	grey	0.16287465
ENSG00000253208	RP11-1G11.2	grey	0.09130121
ENSG00000255476	RP11-1H15.2	grey	0.1055217
ENSG00000249170	RP11-1J11.1	grey	0.32001077
ENSG00000243939	RP11-200A13.1	grey	0.18082508
ENSG00000232768	RP11-201O14.2	grey	0.45425938
ENSG00000229458	RP11-202D18.2	red	0.44016057
ENSG00000226803	RP11-203B9.4	grey	0.45413842
ENSG00000231691	RP11-203F10.5	grey	0.17074558
ENSG00000219133	RP11-203F10.6	turquoise	-0.5095321
ENSG00000255506	RP11-203F8.1	grey	0.32605602
ENSG00000227218	RP11-203J24.8	grey	0.24533559
ENSG00000235625	RP11-203P2.2	turquoise	0.51238108
ENSG00000237606	RP11-203P23.2	grey	0.42115017
ENSG00000231754	RP11-204E9.1	blue	0.40103441
ENSG00000204837	RP11-204M4.2	grey	0.22066503
ENSG00000228327	RP11-206L10.2	grey	0.12876778
ENSG00000235373	RP11-206L10.3	grey	0.11531074
ENSG00000229905	RP11-206L10.4	grey	0.37595333
ENSG00000230092	RP11-206L10.8	grey	-0.0866099
ENSG00000237491	RP11-206L10.9	grey	-0.0411856
ENSG00000225408	RP11-207C16.4	grey	0.18126097
ENSG00000204894	RP11-208G20.2	grey	0.51090607

ENSG00000270405	RP11-208G20.3	grey	0.04111388
ENSG00000214210	RP11-208P4.1	grey	0.29106573
ENSG00000266126	RP11-209D14.4	grey	0.19338666
ENSG00000265908	RP11-20B24.6	grey	0.01745468
ENSG00000264304	RP11-20B24.7	grey	0.23590094
ENSG00000256004	RP11-20D14.4	grey	0.29766324
ENSG00000249790	RP11-20D14.6	grey	0.18363445
ENSG00000257526	RP11-20E24.1	grey	0.32183103
ENSG00000251652	RP11-20I20.2	grey	0.22793779
ENSG00000261613	RP11-20I23.13	grey	-0.0839979
ENSG00000261140	RP11-20I23.6	magenta	-0.4325487
ENSG00000260436	RP11-20I23.7	grey	0.39369091
ENSG00000269937	RP11-20I23.8	grey	-0.0595949
ENSG00000263924	RP11-210K20.2	grey	0.35792596
ENSG00000270879	RP11-210K20.4	cyan	-0.4305556
ENSG00000258010	RP11-210M15.1	greenyellow	-0.4444402
ENSG00000259495	RP11-210M15.2	grey	-0.1441732
ENSG00000257896	RP11-210N13.1	grey	-0.0507954
ENSG00000236636	RP11-211A18.1	grey	0.4109536
ENSG00000228729	RP11-211A18.2	grey	0.39471132
ENSG00000253956	RP11-211F2.1	grey	0.25219742
ENSG00000256533	RP11-212D19.5	grey	0.28804829
ENSG00000249848	RP11-212F11.1	grey	0.48180311
ENSG00000213280	RP11-212P7.1	grey	0.37256738
ENSG00000230303	RP11-213G2.2	grey	0.26898881
ENSG00000165121	RP11-213G2.3	lightgreen	0.75483829
ENSG00000270095	RP11-214K3.18	turquoise	0.43257783
ENSG00000270061	RP11-214K3.19	brown	-0.3579528
ENSG00000269938	RP11-214K3.20	grey	0.22783322
ENSG00000269997	RP11-214K3.21	grey	0.06722953
ENSG00000270048	RP11-214K3.22	grey	0.23227202
ENSG00000270130	RP11-214K3.23	grey	0.21385537
ENSG00000269923	RP11-214K3.24	grey	0.24939685
ENSG00000256827	RP11-214K3.5	grey	0.31700422
ENSG00000234017	RP11-214N15.5	grey	0.35272336
ENSG00000241651	RP11-214O14.1	grey	0.44795462
ENSG00000261340	RP11-215H22.1	blue	0.4380453
ENSG00000248559	RP11-215P8.3	grey	0.32841166
ENSG00000228395	RP11-216B9.6	grey	0.1116794
ENSG00000237846	RP11-216M21.1	grey	-0.2028798
ENSG00000233207	RP11-216M21.7	grey	0.05107414
ENSG00000223599	RP11-216N14.7	grey	0.13206543
ENSG00000236082	RP11-217L21.1	grey	0.27989533
ENSG00000203396	RP11-217O12.1	grey	0.24311375
ENSG00000270001	RP11-218C14.8	turquoise	0.61015737
ENSG00000248725	RP11-218C23.1	turquoise	0.3945313
ENSG00000225393	RP11-218L14.4	grey	0.0563194
ENSG00000177406	RP11-218M22.1	yellow	0.65630331

ENSG00000245534	RP11-219B17.1	grey	0.44563554
ENSG00000254208	RP11-219B4.3	grey	0.31819217
ENSG00000256824	RP11-21A7A.2	grey	0.22778597
ENSG00000256341	RP11-21A7A.3	grey	0.14934474
ENSG00000256481	RP11-21A7A.4	grey	0.0211554
ENSG00000261744	RP11-21B21.4	grey	0.23425307
ENSG00000264714	RP11-21G15.1	grey	-0.2115406
ENSG00000261653	RP11-21L1.1	grey	0.30662649
ENSG00000260635	RP11-21M24.3	grey	0.2106891
ENSG00000270575	RP11-21M7.2	grey	0.35049779
ENSG00000264458	RP11-220C2.1	yellow	0.45244362
ENSG00000233137	RP11-220I1.1	turquoise	0.31409915
ENSG00000228292	RP11-220I1.4	grey	0.30804303
ENSG00000227527	RP11-223A3.1	grey	0.24750534
ENSG00000249806	RP11-223C24.1	grey	0.49533342
ENSG00000261829	RP11-223I10.1	grey	0.18887852
ENSG00000232480	RP11-224O19.2	grey	-0.0139434
ENSG00000260766	RP11-226L15.5	grey	-0.0286132
ENSG00000249216	RP11-227F19.5	yellow	0.43834351
ENSG00000264373	RP11-227G15.6	grey	0.43740243
ENSG00000264083	RP11-227G15.9	grey	0.15709887
ENSG00000250889	RP11-229C3.2	grey	0.12930641
ENSG00000213590	RP11-229P13.2	grey	0.25205367
ENSG00000235117	RP11-229P13.20	grey	-0.1501214
ENSG00000264050	RP11-22N12.2	yellow	0.66009894
ENSG00000261766	RP11-22P6.2	grey	0.3297209
ENSG00000235545	RP11-230B22.1	turquoise	0.50267892
ENSG00000271551	RP11-230C9.2	grey	0.19681102
ENSG00000270487	RP11-230C9.3	grey	0.27318001
ENSG00000271265	RP11-230C9.4	grey	0.31371158
ENSG00000237336	RP11-230L22.4	grey	0.23959099
ENSG00000254634	RP11-231C14.3	grey	0.4856173
ENSG00000248184	RP11-231C18.1	grey	0.06552731
ENSG00000257279	RP11-231I16.1	black	0.71707853
ENSG00000248773	RP11-231L11.3	grey	0.3923127
ENSG00000237971	RP11-231N9.1	grey	0.26088351
ENSG00000236155	RP11-231P20.2	grey	0.16593124
ENSG00000258478	RP11-232C2.1	grey	0.38650689
ENSG00000258874	RP11-232C2.2	grey	0.35424414
ENSG00000242299	RP11-234A1.1	grey	0.19690038
ENSG00000255474	RP11-234B24.2	grey	0.34585338
ENSG00000232406	RP11-234K24.3	grey	0.16407497
ENSG00000261916	RP11-235E17.4	grey	0.22127008
ENSG00000262248	RP11-235E17.5	grey	0.3402161
ENSG00000262903	RP11-235E17.6	grey	0.18311612
ENSG00000248423	RP11-235P11.1	grey	0.33138148
ENSG00000248332	RP11-236J17.5	grey	-0.0746152
ENSG00000235224	RP11-236P24.1	grey	0.29658707

ENSG00000234019	RP11-236P24.3	grey	0.2041899
ENSG00000253122	RP11-238I10.1	grey	0.294052
ENSG00000259833	RP11-23E19.1	grey	0.29991307
ENSG00000259798	RP11-23E19.2	grey	0.24792881
ENSG00000254480	RP11-23F23.2	grey	0.1715871
ENSG00000228153	RP11-23I7.1	grey	-0.0367312
ENSG00000254876	RP11-23J9.5	grey	0.06986346
ENSG00000260618	RP11-23N2.4	yellow	0.39097111
ENSG00000244630	RP11-241J12.1	grey	0.35460953
ENSG00000267002	RP11-242D8.1	grey	0.08880935
ENSG00000267595	RP11-242D8.2	grey	0.24500383
ENSG00000234675	RP11-242F11.2	black	0.56554659
ENSG00000258637	RP11-242P2.1	grey	0.37239199
ENSG00000259106	RP11-242P2.2	grey	0.29914691
ENSG00000261617	RP11-243A14.1	yellow	0.62134446
ENSG00000236559	RP11-243J16.7	grey	0.29587111
ENSG00000241014	RP11-244H3.1	grey	0.03789569
ENSG00000231521	RP11-244N9.4	grey	0.30091072
ENSG00000261534	RP11-244O19.1	black	0.88996664
ENSG00000231563	RP11-245P10.4	grey	0.20006752
ENSG00000248540	RP11-247C2.2	magenta	0.47283398
ENSG00000229169	RP11-247I13.6	grey	0.30331273
ENSG00000217576	RP11-248G5.8	yellow	0.44070638
ENSG00000269906	RP11-248J18.2	lightgreen	0.5890333
ENSG00000270062	RP11-248J18.3	grey	0.09463036
ENSG00000218872	RP11-249L21.2	grey	0.45483812
ENSG00000228834	RP11-249L21.4	grey	0.11357915
ENSG00000270909	RP11-24B13.2	grey	0.00293866
ENSG00000270101	RP11-24B19.3	grey	0.05608227
ENSG00000269965	RP11-24B19.4	grey	0.14826024
ENSG00000233967	RP11-250B2.3	grey	0.17775189
ENSG00000235310	RP11-250H24.4	grey	-0.0150097
ENSG00000214331	RP11-252A24.2	grey	-0.0061529
ENSG00000260539	RP11-252A24.7	grey	0.22062921
ENSG00000229721	RP11-252I14.1	grey	0.38705339
ENSG00000253908	RP11-252I14.2	grey	0.38049332
ENSG00000259999	RP11-252K23.1	grey	0.41590203
ENSG00000261783	RP11-252K23.2	grey	0.30092913
ENSG00000232158	RP11-252O2.2	black	0.67098693
ENSG00000238140	RP11-253A20.1	grey	0.35138349
ENSG00000255670	RP11-253I19.3	brown	-0.5494605
ENSG00000271370	RP11-253I19.4	grey	0.33004409
ENSG00000234925	RP11-254B13.3	blue	-0.4310862
ENSG00000234040	RP11-254B13.4	grey	0.43900077
ENSG00000260077	RP11-254F7.2	grey	0.08573574
ENSG00000260743	RP11-255C15.3	grey	0.2754023
ENSG00000233836	RP11-255H23.2	grey	0.15497317
ENSG00000260834	RP11-256I9.2	grey	-0.0059476

ENSG00000227329	RP11-258C19.4	turquoise	0.44458175
ENSG00000270189	RP11-258C19.7	grey	-0.0779261
ENSG00000226578	RP11-258F22.1	grey	0.21341271
ENSG00000260137	RP11-258F22.2	grey	0.31224344
ENSG00000258844	RP11-259K15.2	grey	-0.0697435
ENSG00000248994	RP11-259O2.1	grey	0.1044214
ENSG00000233340	RP11-25C19.3	grey	0.38159812
ENSG00000232739	RP11-25G10.2	grey	0.28280481
ENSG00000167912	RP11-25K19.1	turquoise	0.73868577
ENSG00000214837	RP11-261C10.3	grey	0.02287521
ENSG00000227230	RP11-261C10.5	grey	0.1317401
ENSG00000253326	RP11-261C10.7	grey	0.10095997
ENSG00000225471	RP11-262D11.2	brown	0.59323409
ENSG00000238113	RP11-262H14.1	grey	-0.2415191
ENSG00000170161	RP11-262H14.4	brown	-0.6342352
ENSG00000229422	RP11-262H14.5	grey	0.32299858
ENSG00000224222	RP11-262I2.2	grey	0.06011193
ENSG00000257325	RP11-263E1.1	yellow	0.55931184
ENSG00000231064	RP11-263K19.4	turquoise	0.51627932
ENSG00000257875	RP11-263K4.1	grey	0.01590152
ENSG00000267207	RP11-264B14.1	grey	0.2487096
ENSG00000229105	RP11-264C15.2	grey	0.35729645
ENSG00000236199	RP11-264I13.2	grey	0.15237419
ENSG00000243574	RP11-264K23.1	grey	0.10227959
ENSG00000260733	RP11-264L1.4	blue	-0.3979681
ENSG00000260490	RP11-265N6.3	grey	0.20724492
ENSG00000229044	RP11-266K22.2	grey	0.44655395
ENSG00000244050	RP11-266K4.1	grey	0.13478186
ENSG00000215241	RP11-266K4.9	grey	-0.0972818
ENSG00000260034	RP11-266L9.2	grey	0.20716019
ENSG00000262700	RP11-266L9.3	green	0.56111527
ENSG00000262587	RP11-266L9.4	grey	0.30421075
ENSG00000262155	RP11-266L9.5	yellow	0.48570837
ENSG00000263326	RP11-266L9.6	grey	0.19309626
ENSG00000227115	RP11-267C16.1	yellow	0.74001502
ENSG00000253175	RP11-267M23.6	grey	0.30418598
ENSG00000232995	RP11-267N12.3	green	0.57877869
ENSG00000243303	RP11-268I9.1	grey	0.37681725
ENSG00000116883	RP11-268J15.5	blue	0.5306404
ENSG00000250166	RP11-268P4.5	grey	-0.016329
ENSG00000236031	RP11-269F20.1	grey	0.00288479
ENSG00000263501	RP11-269G24.2	grey	0.50335539
ENSG00000249328	RP11-26J3.1	grey	0.12131716
ENSG00000253551	RP11-26M5.2	yellow	0.64848826
ENSG00000254314	RP11-26M5.3	yellow	0.73221657
ENSG00000261198	RP11-26O3.1	grey	0.31926009
ENSG00000242488	RP11-270M14.1	grey	0.41754144
ENSG00000259103	RP11-270M14.4	grey	0.23306733

ENSG00000239280	RP11-271C24.3	grey	0.41198508
ENSG00000265798	RP11-271K11.5	cyan	0.48127086
ENSG00000259528	RP11-272D12.2	grey	0.45992857
ENSG00000269998	RP11-272L13.3	darkorange	0.57921454
ENSG00000270911	RP11-272L13.4	darkred	-0.4488856
ENSG00000234740	RP11-272P10.2	grey	0.43488184
ENSG00000247317	RP11-273G15.2	blue	0.49355289
ENSG00000242588	RP11-274B21.1	yellow	0.65934965
ENSG00000271553	RP11-274B21.10	grey	0.21969155
ENSG00000243302	RP11-274B21.2	salmon	-0.5652858
ENSG00000243679	RP11-274B21.3	grey	0.14839623
ENSG00000230715	RP11-274B21.4	grey	0.19591367
ENSG00000241493	RP11-274B21.5	grey	0.51905719
ENSG00000270810	RP11-274B21.8	grey	0.32165592
ENSG00000271344	RP11-274B21.9	grey	0.0615877
ENSG00000240912	RP11-274J15.2	grey	0.04206113
ENSG00000256571	RP11-274J7.2	grey	0.16478756
ENSG00000256399	RP11-274J7.3	yellow	0.44096161
ENSG00000232027	RP11-275F13.3	grey	0.31529558
ENSG00000241231	RP11-275H4.1	turquoise	0.55917726
ENSG00000259598	RP11-275I4.1	grey	0.19835824
ENSG00000227711	RP11-275O4.3	grey	0.44121249
ENSG00000261293	RP11-276H1.2	grey	0.32806945
ENSG00000259445	RP11-276M12.1	grey	0.18402742
ENSG00000203325	RP11-277A4.4	grey	0.43683563
ENSG00000220739	RP11-277I20.3	grey	0.16064188
ENSG00000268544	RP11-277L2.4	grey	0.24611489
ENSG00000269614	RP11-277L2.5	grey	0.21825126
ENSG00000245648	RP11-277P12.20	yellow	0.39241506
ENSG00000260671	RP11-278A23.2	grey	0.12117627
ENSG00000257511	RP11-278C7.1	grey	0.2799109
ENSG00000226828	RP11-278H7.1	turquoise	-0.4349863
ENSG00000245750	RP11-279F6.1	grey	-0.1272639
ENSG00000249679	RP11-279O9.4	grey	0.26431422
ENSG00000214654	RP11-27I1.4	grey	0.20069929
ENSG00000270917	RP11-27I1.6	grey	0.29852501
ENSG00000259390	RP11-27M9.1	grey	0.18320089
ENSG00000231901	RP11-281A20.1	grey	0.23332151
ENSG00000228512	RP11-281A20.2	turquoise	0.372455
ENSG00000261330	RP11-281J9.1	grey	0.30354741
ENSG00000213328	RP11-281O15.2	grey	0.31641594
ENSG00000254035	RP11-281O15.4	grey	0.31892066
ENSG00000253284	RP11-282K24.3	turquoise	-0.4562411
ENSG00000235423	RP11-282O18.3	grey	0.32737991
ENSG00000256894	RP11-283G6.3	grey	0.14943803
ENSG00000256234	RP11-283G6.4	grey	0.16482672
ENSG00000255671	RP11-283I3.1	grey	0.19597794
ENSG00000256577	RP11-283I3.2	cyan	-0.4623402

ENSG00000261799	RP11-283I3.6	blue	-0.5377261
ENSG00000250326	RP11-284M14.1	brown	-0.4506467
ENSG00000259834	RP11-284N8.3	purple	0.78004968
ENSG00000242861	RP11-285F7.2	yellow	0.50609918
ENSG00000223462	RP11-285G1.9	grey	0.12408083
ENSG00000245293	RP11-286E11.1	green	0.84612844
ENSG00000249604	RP11-286E11.2	grey	0.38588411
ENSG00000265316	RP11-286N3.1	grey	0.28239472
ENSG00000258843	RP11-286O18.1	grey	0.47316999
ENSG00000248803	RP11-287J9.1	turquoise	0.54822237
ENSG00000213073	RP11-288H12.3	grey	0.36431298
ENSG00000270949	RP11-288H12.4	grey	0.22030519
ENSG00000237938	RP11-288I21.1	grey	0.31036844
ENSG00000271500	RP11-288K12.1	pink	0.43570472
ENSG00000271639	RP11-289A15.1	grey	0.26694037
ENSG00000267701	RP11-28F1.2	turquoise	0.60032487
ENSG00000267940	RP11-290F24.6	lightcyan	-0.7434452
ENSG00000249096	RP11-290F5.1	grey	0.29035704
ENSG00000257941	RP11-290L1.4	grey	0.34514138
ENSG00000271454	RP11-290L7.5	brown	0.33465629
ENSG00000236872	RP11-291L19.1	grey	0.47764882
ENSG00000215142	RP11-292B8.1	grey	0.23736681
ENSG00000270549	RP11-293K19.1	grey	0.11961529
ENSG00000259319	RP11-293M10.6	black	0.42687614
ENSG00000233461	RP11-295G20.2	grey	0.15624703
ENSG00000229739	RP11-295K2.3	grey	0.378477
ENSG00000261291	RP11-295M3.2	grey	0.16002379
ENSG00000239665	RP11-295P9.3	grey	0.29781729
ENSG00000225112	RP11-295P9.6	grey	0.3853362
ENSG00000247228	RP11-296I10.3	turquoise	0.51051434
ENSG00000261556	RP11-296I10.6	grey	0.18415937
ENSG00000232751	RP11-296O14.1	grey	0.41709507
ENSG00000231615	RP11-296O14.2	lightgreen	0.5378683
ENSG00000203739	RP11-296O14.3	grey	0.03212338
ENSG00000270049	RP11-297D21.4	grey	0.29975157
ENSG00000260432	RP11-297M9.2	red	-0.6793411
ENSG00000260788	RP11-298D21.1	yellow	0.74928785
ENSG00000237734	RP11-298E2.2	grey	0.27445878
ENSG00000257285	RP11-298I3.1	grey	0.38025863
ENSG00000226899	RP11-298J20.3	grey	0.34052248
ENSG00000269387	RP11-298J23.8	grey	0.10648367
ENSG00000259172	RP11-299G20.2	yellow	0.59410831
ENSG00000259764	RP11-299G20.3	yellow	0.4317649
ENSG00000242531	RP11-299J3.6	grey	0.45272372
ENSG00000258605	RP11-299L17.4	grey	0.25583613
ENSG00000269942	RP11-29B2.5	grey	0.26565042
ENSG00000261553	RP11-29G8.3	turquoise	-0.5562248
ENSG00000223503	RP11-29H23.6	grey	0.30369113

ENSG00000271267	RP11-29H23.7	grey	0.39154596
ENSG00000269946	RP11-2B6.3	grey	0.22392167
ENSG00000270953	RP11-2E11.9	grey	-0.1307751
ENSG00000259417	RP11-2E17.1	turquoise	0.62742882
ENSG00000218596	RP11-2J18.1	blue	-0.4838685
ENSG00000248529	RP11-2O17.2	yellow	0.62434341
ENSG00000233110	RP11-301L8.2	grey	0.15729866
ENSG00000227700	RP11-301M17.1	grey	0.18940353
ENSG00000239617	RP11-302B13.1	green	-0.5852232
ENSG00000260213	RP11-303E16.3	brown	-0.5413206
ENSG00000261141	RP11-303E16.5	grey	0.38001919
ENSG00000255122	RP11-303G3.6	grey	0.04492652
ENSG00000255253	RP11-303G3.9	grey	0.2945336
ENSG00000204584	RP11-304F15.3	grey	0.21845724
ENSG00000262837	RP11-304F15.7	grey	0.21829409
ENSG00000260260	RP11-304L19.5	greenyellow	0.63074281
ENSG00000230287	RP11-305E17.4	grey	0.40769959
ENSG00000224616	RP11-305E17.6	turquoise	0.37600565
ENSG00000259994	RP11-305E6.4	grey	0.00907831
ENSG00000229694	RP11-305L7.6	grey	0.33450913
ENSG00000241905	RP11-305O4.1	grey	0.20588601
ENSG00000227267	RP11-305O4.2	grey	0.52117564
ENSG00000224348	RP11-306K13.1	yellow	-0.3355929
ENSG00000223774	RP11-307B6.3	grey	0.18968384
ENSG00000232063	RP11-307E17.8	grey	-0.0372451
ENSG00000233367	RP11-307L3.4	grey	0.45602264
ENSG00000227681	RP11-307P5.1	grey	0.33952704
ENSG00000226249	RP11-307P5.2	grey	0.54561767
ENSG00000234062	RP11-308D16.4	turquoise	-0.4755245
ENSG00000228741	RP11-309I15.1	grey	0.11214756
ENSG00000239815	RP11-309L24.4	grey	0.28201253
ENSG00000224593	RP11-30B1.1	grey	0.23988998
ENSG00000259402	RP11-30K9.4	grey	0.3633326
ENSG00000245975	RP11-30K9.6	grey	0.20484689
ENSG00000253667	RP11-30L15.4	grey	0.19578627
ENSG00000266696	RP11-30L3.2	grey	0.30513045
ENSG00000231829	RP11-310E22.5	grey	0.03318206
ENSG00000257456	RP11-310I24.1	grey	0.20737817
ENSG00000268659	RP11-310J24.3	grey	-0.1158619
ENSG00000270893	RP11-311B14.1	grey	0.24350861
ENSG00000232186	RP11-311P8.2	grey	0.04807725
ENSG00000227950	RP11-312B8.2	turquoise	0.4281927
ENSG00000229874	RP11-312O7.2	grey	0.15674975
ENSG00000230427	RP11-313A24.1	grey	0.23182989
ENSG00000253726	RP11-313C15.1	grey	0.08962011
ENSG00000215146	RP11-313J2.1	grey	-0.0679281
ENSG00000259296	RP11-313P18.1	grey	0.44967685
ENSG00000259241	RP11-313P18.2	grey	0.32225242

ENSG00000230939	RP11-314C16.1	grey	0.1732633
ENSG00000229728	RP11-314N13.3	grey	0.26540841
ENSG00000260657	RP11-315D16.4	grey	0.27203819
ENSG00000258162	RP11-315E17.1	brown	-0.3693782
ENSG00000233906	RP11-315I14.2	grey	0.45968937
ENSG00000255337	RP11-315O6.1	brown	-0.3776933
ENSG00000259606	RP11-316E14.6	grey	-0.0189224
ENSG00000228728	RP11-316K19.3	grey	0.3572926
ENSG00000227492	RP11-316M21.6	grey	0.44768434
ENSG00000236308	RP11-316M21.7	grey	0.40839464
ENSG00000259589	RP11-317G6.1	grey	0.33043516
ENSG00000253549	RP11-317J10.2	grey	0.39568477
ENSG00000254733	RP11-317J19.1	green	0.4319221
ENSG00000257307	RP11-317N8.4	grey	0.31467354
ENSG00000258938	RP11-317N8.5	salmon	-0.4903647
ENSG00000261185	RP11-317P15.5	blue	0.6921876
ENSG00000255159	RP11-318C2.1	grey	0.24419956
ENSG00000230777	RP11-318C24.1	grey	0.36970263
ENSG00000227740	RP11-318C24.2	grey	0.22626666
ENSG00000233379	RP11-318G21.4	greenyellow	-0.508182
ENSG00000270092	RP11-318K12.3	turquoise	0.43998842
ENSG00000261252	RP11-318L16.6	grey	0.02370228
ENSG00000247081	RP11-318M2.2	pink	0.77375337
ENSG00000248932	RP11-319G6.1	grey	0.06377979
ENSG00000248790	RP11-319G6.3	grey	0.24401796
ENSG00000260816	RP11-319G9.3	brown	-0.3700214
ENSG00000238198	RP11-31F15.2	brown	-0.6356627
ENSG00000226403	RP11-31F19.1	yellow	0.63710171
ENSG00000265544	RP11-31I22.1	grey	0.17429559
ENSG00000265115	RP11-31I22.2	grey	0.28530119
ENSG00000265125	RP11-31I22.3	grey	-0.0371295
ENSG00000265697	RP11-31I22.4	grey	0.23101231
ENSG00000260878	RP11-320H14.1	turquoise	0.59547352
ENSG00000254783	RP11-320L11.2	grey	0.2530636
ENSG00000258580	RP11-320M16.2	grey	0.34063438
ENSG00000257613	RP11-320P7.1	grey	0.2229324
ENSG00000257139	RP11-320P7.2	yellow	0.39600677
ENSG00000257543	RP11-321F8.4	grey	0.27525033
ENSG00000237414	RP11-321L2.2	greenyellow	-0.5493534
ENSG00000259397	RP11-323I15.2	grey	-0.07138
ENSG00000259665	RP11-323I15.3	grey	0.02963203
ENSG00000259336	RP11-323I15.5	grey	-0.1699036
ENSG00000235618	RP11-324H6.5	cyan	-0.4386003
ENSG00000233200	RP11-324I22.2	grey	0.23463374
ENSG00000271335	RP11-324I22.4	grey	0.04206837
ENSG00000270599	RP11-324O2.6	grey	0.30179403
ENSG00000225715	RP11-325E14.5	grey	0.23771525
ENSG00000259545	RP11-325E5.4	grey	0.31024823

ENSG00000261114	RP11-325K4.2	grey	0.41838525
ENSG00000259258	RP11-325L12.3	brown	0.31694215
ENSG00000270218	RP11-325L12.5	grey	0.15629461
ENSG00000246323	RP11-325L7.1	grey	0.16145053
ENSG00000260212	RP11-325O24.5	grey	0.17321656
ENSG00000251661	RP11-326C3.11	grey	0.14534268
ENSG00000254910	RP11-326C3.7	grey	0.24622819
ENSG00000254031	RP11-326E22.1	grey	0.24531527
ENSG00000271646	RP11-326I11.3	grey	0.11600711
ENSG00000271538	RP11-326I11.4	grey	0.08502875
ENSG00000270426	RP11-326I11.5	blue	-0.351527
ENSG00000253168	RP11-326L2.1	grey	0.24567513
ENSG00000237587	RP11-327I22.4	grey	0.36209353
ENSG00000231518	RP11-327L3.3	grey	0.29521238
ENSG00000251171	RP11-327O17.2	greenyellow	-0.5036341
ENSG00000231856	RP11-327P2.5	grey	0.20199151
ENSG00000257376	RP11-328C8.2	grey	0.1952063
ENSG00000224500	RP11-328K22.1	grey	0.37085941
ENSG00000260281	RP11-329J18.2	grey	0.23395861
ENSG00000260744	RP11-329J18.3	grey	0.27889199
ENSG00000260012	RP11-329J18.4	grey	-0.0679457
ENSG00000237161	RP11-32B5.1	grey	0.0624002
ENSG00000253554	RP11-32K4.1	grey	0.39151335
ENSG00000225505	RP11-330C7.3	grey	0.3601027
ENSG00000239465	RP11-330L19.2	grey	0.27054426
ENSG00000269907	RP11-330M2.4	grey	0.28419035
ENSG00000270367	RP11-331G2.6	grey	0.354179
ENSG00000214748	RP11-332E4.1	grey	0.11675107
ENSG00000267280	RP11-332H18.4	grey	0.11192602
ENSG00000267131	RP11-332H18.5	grey	0.23597833
ENSG00000237827	RP11-332O19.2	turquoise	0.40451852
ENSG00000261879	RP11-333E1.1	grey	0.2209614
ENSG00000263164	RP11-333E1.2	grey	-0.0056641
ENSG00000250568	RP11-333E13.2	grey	0.10096705
ENSG00000260977	RP11-333I13.1	turquoise	-0.4911888
ENSG00000267798	RP11-333J10.3	grey	0.22392353
ENSG00000224671	RP11-335O13.8	grey	0.1571355
ENSG00000232466	RP11-337A23.5	grey	0.42006217
ENSG00000237188	RP11-337C18.8	salmon	-0.6273308
ENSG00000256085	RP11-337L12.1	grey	0.34327704
ENSG00000263477	RP11-338L22.2	grey	0.20293806
ENSG00000270171	RP11-338N10.1	grey	-0.0928427
ENSG00000270035	RP11-338N10.2	grey	0.05282403
ENSG00000269978	RP11-338N10.3	grey	0.20562165
ENSG00000228510	RP11-339B21.8	grey	0.23744376
ENSG00000245958	RP11-33B1.1	grey	-0.0606891
ENSG00000248280	RP11-33B1.2	grey	0.16917438
ENSG00000250950	RP11-33B1.3	grey	0.35150055

ENSG00000260091	RP11-33B1.4	green	0.42926103
ENSG00000254432	RP11-33I11.2	yellow	0.40869489
ENSG00000258380	RP11-33N16.2	grey	0.47682409
ENSG00000270632	RP11-340C20.3	grey	0.42919405
ENSG00000261827	RP11-340F14.5	yellow	0.51506731
ENSG00000232433	RP11-341A11.2	grey	0.23766631
ENSG00000213755	RP11-342F17.1	grey	0.30593507
ENSG00000270696	RP11-342K6.1	turquoise	0.62714911
ENSG00000270462	RP11-342K6.3	grey	0.26794932
ENSG00000261970	RP11-342L8.2	grey	-0.0179626
ENSG00000259202	RP11-342M21.2	black	0.51580451
ENSG00000224114	RP11-343H5.4	grey	0.40383968
ENSG00000231429	RP11-343N15.2	grey	0.11243449
ENSG00000269996	RP11-343N15.5	grey	-0.1714121
ENSG00000225299	RP11-344A5.1	grey	0.22070853
ENSG00000263904	RP11-344B2.3	grey	0.33999736
ENSG00000266369	RP11-344E13.4	grey	0.0644722
ENSG00000231606	RP11-344F13.1	magenta	-0.3820072
ENSG00000234041	RP11-345I18.1	grey	0.52695289
ENSG00000225711	RP11-345I18.4	grey	0.249352
ENSG00000229659	RP11-345K20.2	grey	0.22313652
ENSG00000269737	RP11-345P4.7	grey	0.13118448
ENSG00000217041	RP11-346C16.1	grey	0.35904637
ENSG00000213150	RP11-346C16.2	yellow	0.45153933
ENSG00000253878	RP11-347C18.3	grey	0.28637641
ENSG00000226530	RP11-348F1.2	grey	0.28005783
ENSG00000229151	RP11-348F1.3	grey	0.12128695
ENSG00000270589	RP11-348N5.7	yellow	0.59131551
ENSG00000257621	RP11-349A22.5	grey	-0.2255688
ENSG00000259232	RP11-349G13.1	grey	0.36335938
ENSG00000224937	RP11-34H11.6	grey	0.28130695
ENSG00000241860	RP11-34P13.13	grey	0.05590699
ENSG00000257742	RP11-350F4.2	grey	-0.2246546
ENSG00000258007	RP11-350G24.1	grey	0.3560436
ENSG00000258033	RP11-350G24.2	yellow	0.64050311
ENSG00000228264	RP11-350G8.7	grey	0.2216645
ENSG00000219433	RP11-350J20.4	grey	0.3785684
ENSG00000231760	RP11-350J20.5	grey	0.33748045
ENSG00000255556	RP11-351I21.6	grey	-0.2346915
ENSG00000254423	RP11-351I21.7	grey	-0.2111945
ENSG00000259649	RP11-351M8.1	grey	0.0782601
ENSG00000224412	RP11-351O1.3	grey	0.57837017
ENSG00000270421	RP11-351O1.4	grey	0.44639798
ENSG00000260773	RP11-352G18.2	grey	0.43367625
ENSG00000261597	RP11-353B9.1	brown	0.39674295
ENSG00000223916	RP11-353H3.1	grey	0.21420439
ENSG00000234091	RP11-353M9.1	grey	0.04477319
ENSG00000229801	RP11-353N4.1	brown	0.47562681

ENSG00000269501	RP11-353N4.6	brown	-0.4136995
ENSG00000231104	RP11-354M20.3	grey	0.30798287
ENSG00000265739	RP11-354P11.3	grey	0.1882908
ENSG00000266987	RP11-354P11.8	grey	0.26907213
ENSG00000261696	RP11-354P17.15	greenyellow	-0.4910757
ENSG00000270820	RP11-355B11.2	turquoise	0.58829566
ENSG00000258903	RP11-355I22.2	blue	0.64689927
ENSG00000258842	RP11-355I22.5	grey	0.44247718
ENSG00000259796	RP11-355I22.7	blue	0.65504639
ENSG00000261094	RP11-355O1.11	brown	0.42928766
ENSG00000213216	RP11-355O1.7	grey	0.33486812
ENSG00000260507	RP11-356C4.3	grey	0.33031113
ENSG00000250303	RP11-356J5.12	yellow	-0.4739237
ENSG00000214264	RP11-356J5.5	grey	0.02641292
ENSG00000235437	RP11-357C3.3	grey	0.16303689
ENSG00000248840	RP11-357G3.2	grey	0.31476354
ENSG00000255618	RP11-357K6.1	blue	0.4034771
ENSG00000261049	RP11-357N13.1	grey	0.41886774
ENSG00000260194	RP11-357N13.2	grey	0.23813849
ENSG00000261630	RP11-357N13.3	grey	0.30614391
ENSG00000267658	RP11-358B23.1	grey	0.17016483
ENSG00000226390	RP11-358D14.2	grey	0.34868045
ENSG00000250132	RP11-359B12.2	grey	0.07449039
ENSG00000254061	RP11-359B20.1	grey	0.37575151
ENSG00000270607	RP11-359E10.1	turquoise	-0.5557025
ENSG00000236722	RP11-359I18.1	grey	0.26224792
ENSG00000256973	RP11-359J14.2	grey	0.02573352
ENSG00000257956	RP11-359M6.3	grey	0.23131773
ENSG00000259110	RP11-359N5.1	grey	0.00930254
ENSG00000270403	RP11-35P15.1	grey	0.253081
ENSG00000225591	RP11-360D2.2	grey	0.27046776
ENSG00000249207	RP11-360F5.1	grey	0.36543848
ENSG00000249685	RP11-360F5.3	grey	0.4617458
ENSG00000259238	RP11-361D15.2	grey	0.13963527
ENSG00000225135	RP11-361F15.2	grey	0.03718353
ENSG00000269984	RP11-362K14.5	brown	0.48975021
ENSG00000254027	RP11-363E6.3	grey	0.13847605
ENSG00000260912	RP11-363E7.4	tan	0.72007303
ENSG00000258957	RP11-363J20.1	grey	0.15734487
ENSG00000258520	RP11-363J20.2	grey	0.26886819
ENSG00000230707	RP11-364B14.3	grey	0.05755063
ENSG00000256031	RP11-364C11.3	grey	0.49168824
ENSG00000259515	RP11-365N19.2	grey	0.28986463
ENSG00000248632	RP11-366M4.11	grey	0.1895287
ENSG00000236941	RP11-366M4.8	grey	0.25989866
ENSG00000228280	RP11-367B6.2	grey	0.13228254
ENSG00000232142	RP11-367H5.8	grey	0.53573404
ENSG00000232648	RP11-367N14.2	grey	0.30356959

ENSG00000257680	RP11-367O10.1	grey	0.23242455
ENSG00000242268	RP11-368I23.2	purple	0.5981345
ENSG00000258611	RP11-368J22.2	grey	0.14046916
ENSG00000260658	RP11-368L12.1	grey	0.30903151
ENSG00000230796	RP11-368M16.9	grey	0.27036248
ENSG00000258993	RP11-368P15.2	grey	0.38772099
ENSG00000258985	RP11-368P15.3	grey	0.02884153
ENSG00000226876	RP11-36N20.1	grey	0.26761598
ENSG00000231787	RP11-370B6.1	grey	0.32181688
ENSG00000232184	RP11-370K11.1	blue	-0.3890318
ENSG00000241570	RP11-372E1.6	brown	0.47688833
ENSG00000241592	RP11-372E1.7	grey	0.25100011
ENSG00000270681	RP11-372K14.2	turquoise	0.4109308
ENSG00000239739	RP11-373E16.4	yellow	0.51838795
ENSG00000250877	RP11-373J21.1	grey	0.21706467
ENSG00000267520	RP11-373L24.1	blue	-0.2908417
ENSG00000247199	RP11-373N22.3	yellow	0.4570901
ENSG00000237321	RP11-374A22.1	grey	-0.069554
ENSG00000223379	RP11-374M1.4	grey	0.254206
ENSG00000235659	RP11-374M1.5	grey	0.33922789
ENSG00000233099	RP11-375A5.1	grey	0.01559379
ENSG00000267230	RP11-376M2.2	grey	0.28268304
ENSG00000261338	RP11-378A13.1	brown	0.35635139
ENSG00000261402	RP11-378I6.1	black	0.70936245
ENSG00000225265	RP11-378J18.3	grey	0.21086313
ENSG00000229399	RP11-378J18.6	grey	0.2903318
ENSG00000239804	RP11-379B18.1	grey	0.10745741
ENSG00000241346	RP11-379B18.3	grey	0.46297604
ENSG00000241288	RP11-379B18.5	turquoise	-0.4774235
ENSG00000270615	RP11-379C1.1	darkorange	0.48965142
ENSG00000243675	RP11-379F4.1	grey	0.54779069
ENSG00000240207	RP11-379F4.4	grey	-0.0858189
ENSG00000271122	RP11-379H18.1	brown	-0.5602602
ENSG00000261086	RP11-379H8.1	grey	0.21166461
ENSG00000233993	RP11-379J5.5	grey	0.10836306
ENSG00000259175	RP11-379K22.2	black	0.44687693
ENSG00000270919	RP11-379K22.3	grey	0.17186378
ENSG00000267746	RP11-379L18.1	red	0.51883762
ENSG00000251136	RP11-37B2.1	grey	-0.1519407
ENSG00000261136	RP11-37C7.3	grey	0.16974563
ENSG00000230633	RP11-37E23.5	turquoise	-0.3007514
ENSG00000254824	RP11-37O16.8	grey	0.27931304
ENSG00000224745	RP11-380G5.2	brown	-0.479271
ENSG00000213613	RP11-380G5.3	grey	0.33210652
ENSG00000270028	RP11-380L11.4	brown	0.40769861
ENSG00000269365	RP11-380M21.4	grey	0.37767504
ENSG00000251292	RP11-380P13.2	greenyellow	-0.505221
ENSG00000250159	RP11-381K20.2	yellow	0.55799474

ENSG00000270697	RP11-381K20.4	grey	0.35498881
ENSG00000270237	RP11-381K20.5	grey	0.09525864
ENSG00000182021	RP11-381O7.3	grey	0.11594582
ENSG00000235355	RP11-381O7.4	grey	0.28322592
ENSG00000234320	RP11-381O7.6	grey	0.31876978
ENSG00000259308	RP11-382A20.1	grey	0.30935975
ENSG00000260579	RP11-382A20.2	royalblue	-0.4469392
ENSG00000260608	RP11-382A20.7	brown	-0.3540707
ENSG00000227962	RP11-382D8.3	grey	0.27994457
ENSG00000254272	RP11-382J24.2	grey	0.32709578
ENSG00000225527	RP11-383B4.4	grey	0.24264205
ENSG00000224023	RP11-383C5.4	grey	0.13627515
ENSG00000267146	RP11-383D22.1	grey	0.4571208
ENSG00000213041	RP11-383G10.3	grey	0.28710943
ENSG00000244327	RP11-383G6.3	grey	0.03328603
ENSG00000237586	RP11-383J24.2	grey	0.24839886
ENSG00000271483	RP11-384A12.1	grey	0.43260197
ENSG00000229832	RP11-384C4.2	grey	0.36201762
ENSG00000225338	RP11-384C4.3	grey	0.40246201
ENSG00000230063	RP11-384C4.6	grey	0.33206089
ENSG00000239268	RP11-384F7.2	red	0.56390115
ENSG00000225892	RP11-384K6.2	grey	0.0629471
ENSG00000260404	RP11-384K6.6	grey	-0.0395498
ENSG00000260252	RP11-384M15.3	grey	0.46008348
ENSG00000267034	RP11-384O8.1	blue	0.50169381
ENSG00000260947	RP11-384P7.7	turquoise	0.40006739
ENSG00000266538	RP11-385D13.3	blue	0.31693212
ENSG00000230325	RP11-385F5.4	grey	0.19366316
ENSG00000257913	RP11-386G11.5	grey	0.05481251
ENSG00000253976	RP11-386G21.1	grey	0.27517798
ENSG00000253857	RP11-386G21.2	blue	0.3956305
ENSG00000229052	RP11-386I23.1	grey	-0.1151873
ENSG00000264968	RP11-387H17.4	grey	0.17512589
ENSG00000265799	RP11-387H17.6	grey	0.21288714
ENSG00000262410	RP11-388C12.8	grey	0.30790146
ENSG00000225181	RP11-388K2.1	grey	0.13507897
ENSG00000232682	RP11-388P9.2	yellow	0.63524564
ENSG00000244061	RP11-389C8.1	grey	0.1950108
ENSG00000261269	RP11-389C8.2	tan	0.76003241
ENSG00000261292	RP11-389G6.3	grey	0.16668175
ENSG00000236066	RP11-389O22.1	turquoise	-0.3630353
ENSG00000261229	RP11-38G5.4	grey	0.29337335
ENSG00000231187	RP11-38L15.3	blue	0.43624133
ENSG00000241732	RP11-38P22.2	black	-0.5490523
ENSG00000261632	RP11-390D11.1	grey	0.43871698
ENSG00000236924	RP11-390F4.6	grey	0.28159076
ENSG00000230581	RP11-390F4.8	grey	0.42596255
ENSG00000255520	RP11-390K5.3	grey	0.42711987

ENSG00000257191	RP11-390N6.1	grey	0.35972065
ENSG00000216802	RP11-390P2.2	turquoise	0.47162676
ENSG00000225177	RP11-390P2.4	turquoise	0.39723176
ENSG00000260229	RP11-391L3.5	grey	0.19014651
ENSG00000229789	RP11-391M20.1	grey	0.27555105
ENSG00000250989	RP11-392E22.5	grey	0.18077521
ENSG00000267747	RP11-392O1.4	grey	0.36420634
ENSG00000228536	RP11-392O17.1	grey	0.35449379
ENSG00000271711	RP11-392O18.2	green	0.3520003
ENSG00000218313	RP11-393I2.2	grey	0.15243525
ENSG00000230546	RP11-393I23.3	grey	0.31053071
ENSG00000235038	RP11-393I23.4	grey	0.0680716
ENSG00000270811	RP11-393K12.4	grey	0.49968301
ENSG00000270109	RP11-393M11.2	grey	0.25657359
ENSG00000260336	RP11-395B7.7	green	0.58348467
ENSG00000254615	RP11-395G23.3	grey	-0.1964279
ENSG00000253476	RP11-395I14.2	grey	0.268837
ENSG00000260296	RP11-395I6.3	grey	0.09128871
ENSG00000235695	RP11-395P16.1	grey	0.29152734
ENSG00000234771	RP11-395P17.3	grey	0.02558227
ENSG00000225518	RP11-396C23.2	grey	0.19608963
ENSG00000233369	RP11-396K3.1	brown	-0.3204578
ENSG00000267284	RP11-397A16.1	grey	0.38451826
ENSG00000227466	RP11-397C12.1	yellow	0.65091335
ENSG00000235410	RP11-397C18.2	blue	0.49032283
ENSG00000229206	RP11-397O4.1	grey	0.43046681
ENSG00000227417	RP11-397P13.6	grey	0.26022027
ENSG00000223505	RP11-397P13.7	grey	0.3813076
ENSG00000254258	RP11-398H6.1	grey	0.11400794
ENSG00000229852	RP11-398K22.12	grey	0.0862012
ENSG00000250746	RP11-39C10.1	grey	0.20482506
ENSG00000224083	RP11-39K24.4	grey	0.32921368
ENSG00000259481	RP11-39M21.1	grey	0.10291804
ENSG00000259575	RP11-39M21.2	grey	0.39146988
ENSG00000237764	RP11-3B12.2	grey	0.10832451
ENSG00000205898	RP11-3B12.4	grey	0.45305292
ENSG00000224899	RP11-3B12.5	grey	0.35329153
ENSG00000259287	RP11-3D4.2	yellow	0.3504814
ENSG00000259408	RP11-3D4.3	grey	0.46838918
ENSG00000254575	RP11-3G21.1	grey	0.27210493
ENSG00000213839	RP11-3J10.4	grey	0.24499443
ENSG00000232454	RP11-3J10.7	grey	0.2493826
ENSG00000241157	RP11-3K24.1	grey	0.43169881
ENSG00000234851	RP11-3P17.3	grey	0.20928404
ENSG00000269888	RP11-3P17.5	grey	0.10146589
ENSG00000251291	RP11-400D2.3	grey	0.19483426
ENSG00000267632	RP11-400F19.18	grey	0.32213824
ENSG00000237807	RP11-400K9.4	yellowgreen	0.54168895

ENSG00000239300	RP11-400L8.2	grey	0.19543201
ENSG00000236848	RP11-401L13.5	grey	0.42070838
ENSG00000270723	RP11-401N16.1	grey	0.34292824
ENSG00000261685	RP11-401P9.4	salmon	-0.5706045
ENSG00000253539	RP11-402L5.1	grey	0.56406838
ENSG00000254051	RP11-403D15.1	turquoise	0.45578206
ENSG00000255206	RP11-403D15.2	grey	0.30941812
ENSG00000224972	RP11-403H13.1	grey	0.5378163
ENSG00000235999	RP11-403I13.8	grey	-0.0515351
ENSG00000240590	RP11-403P13.1	grey	0.13178242
ENSG00000260650	RP11-403P17.2	grey	0.06534289
ENSG00000244062	RP11-404G16.2	yellow	0.73083763
ENSG00000249513	RP11-404I7.1	grey	0.13581884
ENSG00000176320	RP11-404O13.5	grey	0.29902326
ENSG00000255910	RP11-405A12.2	grey	0.34259918
ENSG00000259113	RP11-406H23.2	grey	0.07020805
ENSG00000258039	RP11-406H4.1	grey	0.21997061
ENSG00000232939	RP11-406O23.2	yellow	0.58554861
ENSG00000260038	RP11-407G23.4	grey	0.36983268
ENSG00000254579	RP11-407P18.1	blue	0.43709201
ENSG00000229029	RP11-408A13.1	grey	0.40720587
ENSG00000259554	RP11-408J6.1	grey	0.19166819
ENSG00000230684	RP11-409K20.6	grey	0.30105858
ENSG00000231132	RP11-40C11.2	black	-0.519434
ENSG00000219928	RP11-40C6.2	grey	0.29572999
ENSG00000233067	RP11-40F8.2	grey	0.34976718
ENSG00000259915	RP11-410E4.1	blue	-0.4406915
ENSG00000253180	RP11-410L14.1	grey	0.09044466
ENSG00000253948	RP11-410L14.2	green	-0.5173263
ENSG00000233293	RP11-410N8.3	grey	0.25662398
ENSG00000267150	RP11-411B10.2	grey	0.28261401
ENSG00000267756	RP11-411B10.4	grey	0.027286
ENSG00000260329	RP11-412D9.4	grey	0.16008258
ENSG00000230735	RP11-413E1.4	blue	-0.318313
ENSG00000243422	RP11-413E6.1	grey	0.1221309
ENSG00000227512	RP11-413M3.4	grey	0.12719444
ENSG00000249753	RP11-415I12.3	grey	0.40823907
ENSG00000225313	RP11-415J8.3	grey	0.23414252
ENSG00000233246	RP11-415J8.5	yellow	0.30707532
ENSG00000257246	RP11-416A17.6	grey	0.09687082
ENSG00000260328	RP11-416I2.1	magenta	0.72213413
ENSG00000226431	RP11-416N2.3	grey	0.28803017
ENSG00000230506	RP11-416N4.4	grey	0.38367246
ENSG00000250116	RP11-417F21.1	yellow	0.44895844
ENSG00000256955	RP11-417L19.2	grey	0.19626461
ENSG00000262140	RP11-417N10.3	grey	0.30774499
ENSG00000225761	RP11-417O11.5	grey	0.19786591
ENSG00000231365	RP11-418J17.1	grey	0.09516487

ENSG00000270154	RP11-419I17.1	grey	-0.1069403
ENSG00000266258	RP11-41O4.1	grey	0.14080223
ENSG00000218521	RP11-420A21.1	grey	0.33504493
ENSG00000251432	RP11-420A23.1	grey	0.01408791
ENSG00000263220	RP11-420A6.2	grey	0.34366766
ENSG00000253335	RP11-420B22.1	black	-0.4926501
ENSG00000230438	RP11-420G6.4	grey	-0.0161868
ENSG00000226438	RP11-420K8.1	grey	0.36964637
ENSG00000260411	RP11-420N3.2	blue	0.53260018
ENSG00000264895	RP11-421E14.2	grey	-0.239348
ENSG00000229509	RP11-421E17.4	grey	0.41075091
ENSG00000247903	RP11-421F16.3	grey	-0.0253124
ENSG00000233184	RP11-421L21.3	grey	0.05559936
ENSG00000248050	RP11-422N16.3	grey	0.02044466
ENSG00000260987	RP11-423G4.7	blue	0.66592712
ENSG00000170089	RP11-423H2.1	grey	-0.3579076
ENSG00000230255	RP11-423O2.1	grey	0.21833787
ENSG00000236055	RP11-423O2.2	grey	0.10208068
ENSG00000234978	RP11-423O2.5	grey	0.14339894
ENSG00000259182	RP11-424I19.2	grey	0.32768729
ENSG00000256564	RP11-424M22.3	blue	-0.3618919
ENSG00000196951	RP11-425I13.3	grey	-0.1142067
ENSG00000244313	RP11-425L10.1	grey	0.12900849
ENSG00000267762	RP11-426J5.2	grey	0.35807613
ENSG00000231046	RP11-428F8.2	grey	0.27095825
ENSG00000248627	RP11-428L21.1	grey	0.30784486
ENSG00000249239	RP11-428L21.2	grey	0.29587101
ENSG00000256654	RP11-429A20.4	grey60	0.4726259
ENSG00000261478	RP11-429B14.4	grey	-0.1839914
ENSG00000259398	RP11-430B1.1	cyan	-0.4227319
ENSG00000259577	RP11-430B1.2	grey	-0.0261531
ENSG00000240710	RP11-430C7.4	grey	0.08850812
ENSG00000240219	RP11-430C7.5	grey	0.05958506
ENSG00000254427	RP11-430H10.1	grey	0.06877879
ENSG00000226457	RP11-430L17.1	grey	0.39473783
ENSG00000213529	RP11-432F4.2	grey	0.27657568
ENSG00000238058	RP11-432J22.2	grey	0.23429696
ENSG00000258702	RP11-433J8.1	blue	0.41396689
ENSG00000263177	RP11-433P17.2	grey	-0.029629
ENSG00000260837	RP11-434B12.1	yellow	-0.5219689
ENSG00000247157	RP11-434C1.1	blue	0.58938318
ENSG00000256237	RP11-434C1.2	grey	0.26486166
ENSG00000270863	RP11-434C1.3	turquoise	-0.4915365
ENSG00000249894	RP11-434D9.2	grey	0.38466974
ENSG00000242569	RP11-435B5.3	grey	0.07283247
ENSG00000185044	RP11-435B5.4	grey	0.03341621
ENSG00000238261	RP11-435B5.5	grey	0.04259306
ENSG00000238085	RP11-435F13.2	grey	0.11806647

ENSG00000271659	RP11-435O5.4	grey	0.21535645
ENSG00000250206	RP11-436H11.2	brown	-0.3821941
ENSG00000250530	RP11-436H11.3	grey	0.11560241
ENSG00000255776	RP11-436I9.3	grey	0.34035379
ENSG00000231252	RP11-436K8.1	grey	-0.0085458
ENSG00000242888	RP11-436M15.1	grey	0.42556441
ENSG00000249417	RP11-438D8.2	grey	0.34594506
ENSG00000257231	RP11-438N16.2	grey	0.21097999
ENSG00000234998	RP11-439A17.10	grey	0.3501273
ENSG00000253764	RP11-439C15.4	grey	0.09389962
ENSG00000225300	RP11-439E19.1	grey	0.39283481
ENSG00000260855	RP11-439E19.10	grey	-0.3016436
ENSG00000227953	RP11-439E19.3	grey	-0.2390407
ENSG00000224460	RP11-439L18.2	grey	0.31963364
ENSG00000229017	RP11-439L18.3	grey	-0.0836573
ENSG00000249112	RP11-43D2.2	grey	0.50369152
ENSG00000258111	RP11-43D4.2	grey	0.26252334
ENSG00000188002	RP11-43F13.1	turquoise	0.5473101
ENSG00000249592	RP11-440L14.1	grey	0.19042637
ENSG00000244249	RP11-441M10.1	grey	0.34005974
ENSG00000224934	RP11-441O15.3	grey	0.31295156
ENSG00000236467	RP11-443A13.5	blue	0.29079227
ENSG00000237162	RP11-443F16.1	cyan	-0.536739
ENSG00000236556	RP11-443O13.3	grey	0.1160932
ENSG00000227554	RP11-444D13.1	grey	0.2918088
ENSG00000255864	RP11-444D3.1	grey	0.17243721
ENSG00000132832	RP11-445H22.3	blue	0.48947311
ENSG00000244558	RP11-445H22.4	grey	0.05459958
ENSG00000227215	RP11-445L13__B	grey	0.26078106
ENSG00000224799	RP11-445O16.3	grey	0.10499523
ENSG00000248138	RP11-446J8.1	grey	0.1916335
ENSG00000249779	RP11-447H19.3	grey	0.43778832
ENSG00000261490	RP11-448G15.3	grey	0.07881116
ENSG00000224901	RP11-448G4.4	grey	0.41744557
ENSG00000260448	RP11-449H11.1	grey	0.34523085
ENSG00000270409	RP11-44D5.1	grey	0.32917855
ENSG00000261804	RP11-44F14.2	brown	-0.5648982
ENSG00000248165	RP11-44F21.2	grey	0.25497813
ENSG00000261267	RP11-44I10.3	grey	0.38782722
ENSG00000184414	RP11-44M6.3	greenyellow	-0.4739591
ENSG00000253372	RP11-44N11.1	grey	0.30057241
ENSG00000257556	RP11-44N21.1	blue	0.33054159
ENSG00000240045	RP11-451G4.2	black	0.79294707
ENSG00000203876	RP11-451M19.3	pink	-0.4768974
ENSG00000234042	RP11-452D2.2	grey	0.17175531
ENSG00000228106	RP11-452F19.3	grey	-0.0135828
ENSG00000254649	RP11-452H21.2	grey	0.35876726
ENSG00000260267	RP11-452L6.5	grey	0.24273185

ENSG00000260625	RP11-452L6.7	grey	0.23563235
ENSG00000270228	RP11-453E17.3	brown	-0.3924949
ENSG00000270292	RP11-453E17.4	grey	0.15637303
ENSG00000250869	RP11-453N18.1	grey	0.24675527
ENSG00000261056	RP11-454F8.2	grey	0.03701185
ENSG00000259318	RP11-454L9.2	grey	0.40175787
ENSG00000249937	RP11-454P21.1	grey	0.13817388
ENSG00000265015	RP11-454P7.3	grey	-0.0583963
ENSG00000250616	RP11-455F5.3	grey	0.06907198
ENSG00000255928	RP11-456I15.2	blue	0.28526755
ENSG00000229808	RP11-456P18.2	grey	0.37316933
ENSG00000261584	RP11-457M11.5	turquoise	-0.4490006
ENSG00000233396	RP11-458D21.1	grey	0.21336347
ENSG00000232546	RP11-458F8.1	grey	0.31450292
ENSG00000230295	RP11-458F8.2	grey	0.25221929
ENSG00000260927	RP11-459F6.3	grey	0.23368654
ENSG00000237869	RP11-459O16.1	grey	0.05590785
ENSG00000235149	RP11-45I20.1	grey	0.41513732
ENSG00000263624	RP11-45M22.3	turquoise	-0.4813841
ENSG00000266498	RP11-45M22.5	grey	0.27320876
ENSG00000259775	RP11-45P15.4	grey	-0.2574034
ENSG00000255227	RP11-460B17.2	grey	0.09466496
ENSG00000254530	RP11-460B17.3	grey	0.25124953
ENSG00000235893	RP11-460E7.8	grey	0.30422772
ENSG00000271225	RP11-460N11.3	grey	0.18950357
ENSG00000227113	RP11-460N20.4	grey	0.21861403
ENSG00000226002	RP11-460N20.5	grey	0.11192988
ENSG00000270948	RP11-460N20.7	grey	0.27259011
ENSG00000251340	RP11-461G12.2	grey	-0.0477478
ENSG00000270751	RP11-461L13.2	grey	0.33359347
ENSG00000246379	RP11-461O7.1	grey	0.33861846
ENSG00000259447	RP11-462P6.1	grey	0.38733106
ENSG00000254538	RP11-463D19.1	grey	0.38338869
ENSG00000259587	RP11-463I20.2	grey	0.02809084
ENSG00000259181	RP11-463I20.3	grey	-0.094637
ENSG00000264546	RP11-464D20.6	grey	0.48426475
ENSG00000265643	RP11-465I4.2	grey	0.26767451
ENSG00000264845	RP11-465I4.3	grey	0.37401151
ENSG00000256071	RP11-465L8.1	grey	0.23190542
ENSG00000231373	RP11-466A17.1	turquoise	0.45488583
ENSG00000265337	RP11-466A19.5	yellow	0.39677977
ENSG00000256280	RP11-466C23.5	brown	0.46966263
ENSG00000224358	RP11-466F5.8	grey	0.2400687
ENSG00000249755	RP11-466G12.2	grey	0.35800296
ENSG00000244398	RP11-466H18.1	grey	-0.0748814
ENSG00000251107	RP11-466P24.4	grey	0.37704443
ENSG00000250348	RP11-466P24.6	grey	0.26532663
ENSG00000237934	RP11-467D18.2	grey	0.13018136

ENSG00000259826	RP11-467D6.1	blue	0.31252512
ENSG00000213542	RP11-467H10.1	grey	0.34294021
ENSG00000259628	RP11-467H10.2	grey	0.22105669
ENSG00000253973	RP11-467K18.2	grey	0.0362174
ENSG00000259321	RP11-468E2.5	grey	0.1059312
ENSG00000225766	RP11-468E2.9	grey	0.07820693
ENSG00000260835	RP11-468I15.1	grey	0.3677068
ENSG00000260006	RP11-469M7.1	grey	0.20417745
ENSG00000251520	RP11-46A10.6	grey	0.36179083
ENSG00000270711	RP11-46A10.8	greenyellow	-0.4778602
ENSG00000259877	RP11-46C24.7	grey	0.2987142
ENSG00000238260	RP11-46F15.2	grey	0.28619764
ENSG00000250790	RP11-46H11.3	yellow	-0.366625
ENSG00000232342	RP11-46O21.2	grey	0.23888071
ENSG00000229312	RP11-470P21.2	grey	0.10902545
ENSG00000258424	RP11-471B22.2	grey	0.31788706
ENSG00000263648	RP11-471L13.3	greenyellow	-0.4964592
ENSG00000243721	RP11-472I20.1	grey	0.35913088
ENSG00000254512	RP11-472I20.2	turquoise	0.53480704
ENSG00000231441	RP11-472M19.2	grey	0.23130932
ENSG00000237797	RP11-472N13.3	grey	-0.0511367
ENSG00000257458	RP11-473C19.1	darkorange	0.46094454
ENSG00000248283	RP11-473D24.1	floralwhite	0.64670607
ENSG00000263244	RP11-473I1.10	grey	0.00921239
ENSG00000262944	RP11-473I1.9	turquoise	0.4880841
ENSG00000263072	RP11-473M20.14	green	-0.33118
ENSG00000261971	RP11-473M20.7	grey	-0.0536548
ENSG00000243402	RP11-473O4.1	grey	0.33170134
ENSG00000253720	RP11-473O4.3	grey	0.22396686
ENSG00000254263	RP11-473O4.4	grey	0.30079052
ENSG00000254317	RP11-473O4.5	grey	0.29554364
ENSG00000261369	RP11-474B12.1	grey	0.2440839
ENSG00000226318	RP11-474D14.2	grey	0.2358508
ENSG00000264270	RP11-474I11.7	grey	0.31340946
ENSG00000264829	RP11-474I11.8	grey	0.21549159
ENSG00000217488	RP11-474L11.5	grey	0.3051415
ENSG00000257752	RP11-474L23.3	grey	0.28873622
ENSG00000266127	RP11-474N24.2	grey	0.31336676
ENSG00000177736	RP11-474P12.3	cyan	-0.4025499
ENSG00000243384	RP11-475O23.2	grey	0.29491161
ENSG00000236408	RP11-476H24.1	grey	0.28778161
ENSG00000235381	RP11-477D19.2	grey	0.19483309
ENSG00000269887	RP11-477H21.2	turquoise	0.46351754
ENSG00000239593	RP11-477J21.6	grey	0.29588841
ENSG00000225118	RP11-477L16.2	grey	0.41387457
ENSG00000251148	RP11-478C1.7	grey	0.51315001
ENSG00000249077	RP11-478C1.8	grey	0.43212673
ENSG00000258210	RP11-478C19.2	grey	-0.0631686

ENSG00000250321	RP11-478C6.4	grey	0.28397971
ENSG00000251188	RP11-478C6.6	green	-0.5190009
ENSG00000229588	RP11-479J7.2	blue	0.42672008
ENSG00000248475	RP11-479O16.1	grey	0.34461334
ENSG00000232334	RP11-47G11.2	grey	0.33209698
ENSG00000258926	RP11-47I22.1	turquoise	0.50758898
ENSG00000260261	RP11-480A16.1	grey	0.11152239
ENSG00000235449	RP11-480I12.2	grey	0.38721011
ENSG00000219222	RP11-480N24.3	grey	0.30185365
ENSG00000220614	RP11-480N24.4	grey	0.32871453
ENSG00000254507	RP11-481A20.10	grey	0.27706514
ENSG00000255098	RP11-481A20.11	grey	0.31242563
ENSG00000261078	RP11-481J2.1	grey	0.31171441
ENSG00000260186	RP11-481J2.2	magenta	0.67040954
ENSG00000257711	RP11-482D24.2	black	0.5393663
ENSG00000257918	RP11-482D24.3	grey	0.18439518
ENSG00000260750	RP11-482M8.1	grey	0.26902095
ENSG00000269935	RP11-482M8.3	grey	0.01483704
ENSG00000262119	RP11-483C6.1	grey	0.03387161
ENSG00000229278	RP11-483F11.7	grey	0.00789496
ENSG00000234789	RP11-483H20.4	grey	0.16570384
ENSG00000255462	RP11-483L5.1	grey	0.25689878
ENSG00000254907	RP11-484D2.2	grey	0.45841544
ENSG00000254463	RP11-484D2.3	grey	0.2442099
ENSG00000254577	RP11-484D2.4	grey	0.4799773
ENSG00000267764	RP11-484L8.1	grey	0.03211125
ENSG00000266304	RP11-484N16.1	grey	0.35014423
ENSG00000261622	RP11-484P15.1	blue	0.3349408
ENSG00000230638	RP11-486B10.4	grey	0.05337079
ENSG00000213540	RP11-486G15.1	grey	0.31842205
ENSG00000271576	RP11-486G15.2	grey	0.05690452
ENSG00000247373	RP11-486O12.2	turquoise	0.43567543
ENSG00000257759	RP11-486O13.4	grey	0.04583942
ENSG00000236073	RP11-487E1.2	grey	0.4927222
ENSG00000232437	RP11-487I5.4	grey	0.33066785
ENSG00000258301	RP11-488C13.5	grey	0.05247433
ENSG00000259865	RP11-488L18.10	grey	0.03857204
ENSG00000234453	RP11-488L4.1	grey	0.20582729
ENSG00000259446	RP11-489D6.2	grey	0.11627964
ENSG00000253988	RP11-489O18.1	pink	-0.5488854
ENSG00000240898	RP11-48B14.1	grey	0.15464228
ENSG00000260317	RP11-48B3.4	grey	0.16268976
ENSG00000225376	RP11-490D19.6	grey	0.31397005
ENSG00000259946	RP11-490G2.2	grey	-0.129112
ENSG00000229447	RP11-490K7.4	grey	0.36847472
ENSG00000260025	RP11-490M8.1	yellow	-0.7324724
ENSG00000228686	RP11-492I21.1	grey	0.29910433
ENSG00000258510	RP11-493G17.4	grey	0.51855313

ENSG00000270178	RP11-494H4.3	grey	0.37142663
ENSG00000255933	RP11-495K9.5	green	0.40506479
ENSG00000255091	RP11-495O11.1	grey	0.29163695
ENSG00000241946	RP11-496B10.1	grey	0.35496519
ENSG00000239774	RP11-496B10.3	grey	0.2510206
ENSG00000249626	RP11-496H1.1	grey	0.33876915
ENSG00000205562	RP11-497E19.1	blue	0.47473205
ENSG00000250687	RP11-497H16.7	grey	0.33618432
ENSG00000269983	RP11-497H16.9	turquoise	0.50415966
ENSG00000262663	RP11-497H17.1	grey	0.05106105
ENSG00000263731	RP11-498C9.15	grey	0.35015465
ENSG00000261337	RP11-498D10.5	grey	0.08810331
ENSG00000263320	RP11-498D10.6	grey	0.1875141
ENSG00000269979	RP11-498E2.7	grey	0.41888763
ENSG00000270102	RP11-498E2.8	grey	0.18131086
ENSG00000235494	RP11-498P14.4	grey	0.18893093
ENSG00000203279	RP11-498P14.5	grey	0.09334398
ENSG00000248161	RP11-499E18.1	grey	-0.0659258
ENSG00000259692	RP11-499F3.2	grey	0.42657471
ENSG00000240291	RP11-499P20.2	grey	0.15043267
ENSG00000260023	RP11-49C24.1	grey	0.15493893
ENSG00000260552	RP11-49I11.1	grey	0.03891608
ENSG00000266957	RP11-49K24.4	grey	0.53833991
ENSG00000267800	RP11-49K24.5	grey	0.44344827
ENSG00000267724	RP11-49K24.8	grey	0.2908832
ENSG00000236095	RP11-49O14.2	grey	0.33305691
ENSG00000230815	RP11-49O14.3	grey	0.31171365
ENSG00000232259	RP11-4C20.3	grey	0.29647211
ENSG00000227076	RP11-4C20.4	grey	0.36072843
ENSG00000227071	RP11-4E23.2	grey	0.23033309
ENSG00000264630	RP11-4F22.2	pink	0.46618225
ENSG00000256306	RP11-4N23.1	blue	0.51228219
ENSG00000259953	RP11-4O1.2	brown	0.68970531
ENSG00000270782	RP11-500K19.2	grey	0.36878083
ENSG00000249409	RP11-501E14.1	grey	-0.0099353
ENSG00000248374	RP11-501M7.1	yellow	0.42782125
ENSG00000254165	RP11-503E24.2	grey	0.29685116
ENSG00000251229	RP11-503N18.5	grey	0.40982543
ENSG00000263982	RP11-504I13.3	grey	0.26503895
ENSG00000229930	RP11-504P24.5	grey	0.27617308
ENSG00000259376	RP11-505E24.3	grey	0.19539318
ENSG00000260018	RP11-505K9.1	yellow	-0.381267
ENSG00000251307	RP11-506H20.1	grey	0.16567841
ENSG00000271410	RP11-506H20.2	grey	0.29285858
ENSG00000244733	RP11-506M13.3	grey	0.17345146
ENSG00000251339	RP11-506N2.1	grey	0.20128062
ENSG00000233693	RP11-506O24.1	grey	0.29726393
ENSG00000258983	RP11-507K2.2	grey	0.32149992

ENSG00000258789	RP11-507K2.3	grey	0.09003802
ENSG00000230782	RP11-508N12.2	grey	0.1354828
ENSG00000257180	RP11-509E10.1	grey	-0.0399403
ENSG00000261452	RP11-509E16.1	black	0.72752081
ENSG00000237359	RP11-509J21.2	grey	0.29837048
ENSG00000235326	RP11-509J21.3	grey	0.14293021
ENSG00000226669	RP11-509J21.4	grey	0.24585479
ENSG00000253137	RP11-509P12.1	grey	0.21085227
ENSG00000254694	RP11-50B3.4	grey	0.21299808
ENSG00000259250	RP11-50C13.1	grey	0.33402548
ENSG00000261668	RP11-50D9.3	yellow	-0.5252281
ENSG00000224127	RP11-510C10.2	grey	0.14279978
ENSG00000258230	RP11-511H9.3	grey	0.35602044
ENSG00000232403	RP11-511I11.1	grey	0.31088114
ENSG00000225762	RP11-511I2.2	grey	0.33206357
ENSG00000256304	RP11-512M8.3	grey	0.25079289
ENSG00000225303	RP11-512N4.2	grey	0.40078574
ENSG00000226750	RP11-513D4.3	grey	0.36096235
ENSG00000254898	RP11-513D5.2	grey	0.13760146
ENSG00000255968	RP11-513G19.1	grey	0.14204753
ENSG00000225339	RP11-513I15.6	turquoise	0.56573848
ENSG00000239480	RP11-514P8.2	grey	0.17140191
ENSG00000262848	RP11-517A5.5	grey	0.47626327
ENSG00000261167	RP11-517B11.7	grey	0.16474495
ENSG00000261243	RP11-517C16.4	grey	0.00668065
ENSG00000250602	RP11-517I3.1	turquoise	0.66420622
ENSG00000258378	RP11-517O13.1	grey	0.37284904
ENSG00000259935	RP11-519C12.1	grey	0.26312929
ENSG00000259519	RP11-519G16.2	yellow	-0.3573453
ENSG00000259342	RP11-519G16.5	grey	0.28617229
ENSG00000263883	RP11-51F16.5	grey	0.03414474
ENSG00000224631	RP11-51O6.1	greenyellow	0.43269917
ENSG00000237645	RP11-521A24.1	grey	0.23436764
ENSG00000251602	RP11-521B24.3	grey	0.16608199
ENSG00000260368	RP11-521I2.3	grey	0.29350973
ENSG00000260163	RP11-521O16.2	blue	0.56609539
ENSG00000254473	RP11-522I20.3	turquoise	0.44942357
ENSG00000237300	RP11-522L3.3	grey	0.23875621
ENSG00000226352	RP11-523H24.3	grey	0.47603132
ENSG00000261295	RP11-524D16__A	grey	0.13574028
ENSG00000226493	RP11-524O24.2	grey	0.38588901
ENSG00000251076	RP11-526F3.1	grey	0.42049824
ENSG00000270127	RP11-526I2.5	grey	0.03357693
ENSG00000228539	RP11-526K17.2	grey	0.18512533
ENSG00000230166	RP11-526K21.2	grey	0.35290179
ENSG00000225554	RP11-527D7.1	grey	-0.1199679
ENSG00000231842	RP11-527F13.1	grey	0.088471
ENSG00000261643	RP11-529E10.6	grey60	0.5093688

ENSG00000244932	RP11-529F4.1	grey	-0.0910479
ENSG00000261372	RP11-529H20.6	grey	0.04291882
ENSG00000261777	RP11-529K1.2	green	0.42424086
ENSG00000255459	RP11-52B19.10	grey	0.26126122
ENSG00000223450	RP11-52I18.1	lightgreen	0.50760982
ENSG00000229502	RP11-52J3.2	grey	0.27494318
ENSG00000234361	RP11-52J3.3	grey	0.43600067
ENSG00000249580	RP11-52L11.6	grey	0.16947383
ENSG00000242836	RP11-52L11.7	grey	0.31260777
ENSG00000239632	RP11-52L11.9	grey	0.31440706
ENSG00000258044	RP11-530C5.2	grey	0.37499266
ENSG00000262503	RP11-530N7.2	grey	0.21221388
ENSG00000263316	RP11-530N7.3	grey	0.21045385
ENSG00000253636	RP11-531A24.5	grey	0.3381226
ENSG00000235145	RP11-533E19.3	grey	0.42987593
ENSG00000236896	RP11-535C21.3	grey	0.3642898
ENSG00000224848	RP11-535M15.1	grey	0.06328874
ENSG00000233820	RP11-535M15.2	grey	0.05820689
ENSG00000225279	RP11-536C5.2	greenyellow	-0.4334543
ENSG00000227741	RP11-536C5.7	grey	0.05127472
ENSG00000255021	RP11-536I6.2	grey	0.06726935
ENSG00000271580	RP11-536L3.4	grey	0.36014863
ENSG00000233144	RP11-537A6.9	grey	0.22344995
ENSG00000236489	RP11-537I16.2	grey	0.28580027
ENSG00000243314	RP11-538P18.1	grey	0.12848495
ENSG00000249019	RP11-539G18.1	grey	0.47050176
ENSG00000225302	RP11-539I5.1	grey	0.14785274
ENSG00000251580	RP11-539L10.3	yellow	-0.3785151
ENSG00000267529	RP11-53B2.1	black	0.66212956
ENSG00000267702	RP11-53B2.2	black	0.71119137
ENSG00000267136	RP11-53B2.3	black	0.67585396
ENSG00000267503	RP11-53B2.4	tan	0.60425947
ENSG00000267366	RP11-53B2.5	grey	0.33693773
ENSG00000263393	RP11-53I6.4	grey	0.3976812
ENSG00000251141	RP11-53O19.1	grey	-0.1527747
ENSG00000248779	RP11-53O19.2	grey	0.20563928
ENSG00000270015	RP11-540B6.6	yellow	0.40836164
ENSG00000259521	RP11-540O11.4	grey	0.31742054
ENSG00000259617	RP11-540O11.6	grey	0.34831976
ENSG00000231906	RP11-541M12.3	grey	0.28634777
ENSG00000260461	RP11-541N10.3	grey	-0.0199051
ENSG00000269667	RP11-542M13.2	grey	0.21142136
ENSG00000247970	RP11-543C4.1	grey	0.28466548
ENSG00000258672	RP11-543C4.3	greenyellow	-0.4900711
ENSG00000227081	RP11-543P15.1	greenyellow	0.51667856
ENSG00000236986	RP11-544A12.4	grey	0.41742809
ENSG00000246851	RP11-544A12.8	grey	0.19674251
ENSG00000223478	RP11-545E17.3	brown	0.44229928

ENSG00000240419	RP11-545E8.1	grey	0.31630739
ENSG00000241449	RP11-545G3.1	grey	0.4979945
ENSG00000258591	RP11-545M17.3	grey	0.1191588
ENSG00000259125	RP11-545N8.3	grey	0.34497049
ENSG00000261002	RP11-546B15.1	grey	0.14339021
ENSG00000260571	RP11-546B15.2	grey	0.29713594
ENSG00000270557	RP11-546J1.1	grey	0.16602981
ENSG00000237457	RP11-547I7.2	red	0.68241034
ENSG00000249244	RP11-548H18.2	grey	0.18498487
ENSG00000224653	RP11-548K12.10	grey	0.24263932
ENSG00000233562	RP11-548N1.1	grey	0.30997261
ENSG00000259985	RP11-549B18.1	grey	0.14190381
ENSG00000251023	RP11-549J18.1	grey	0.29831398
ENSG00000214067	RP11-549L6.2	grey	0.48321532
ENSG00000213433	RP11-54C4.1	grey	0.23459849
ENSG00000215237	RP11-54D18.2	grey	-0.0306177
ENSG00000251196	RP11-54F2.1	grey	0.38366251
ENSG00000225693	RP11-54K16.2	blue	-0.5369903
ENSG00000240809	RP11-550F7.1	grey60	0.59287092
ENSG00000215796	RP11-551G24.2	grey	0.10984769
ENSG00000256984	RP11-551L14.6	grey	0.13585596
ENSG00000260601	RP11-552C15.1	grey	0.06947368
ENSG00000228058	RP11-552D4.1	cyan	0.79828202
ENSG00000259588	RP11-552E10.1	grey	0.12103152
ENSG00000243960	RP11-552M11.4	grey	0.13513752
ENSG00000260948	RP11-552M11.8	grey	0.02076277
ENSG00000240751	RP11-553D4.2	grey	0.0367964
ENSG00000214280	RP11-553K23.2	grey	0.26863941
ENSG00000259591	RP11-554D20.1	grey	0.36569216
ENSG00000237461	RP11-554F20.1	grey	0.22524225
ENSG00000242768	RP11-556G22.1	blue	0.31888066
ENSG00000248242	RP11-556I14.2	grey	0.11953115
ENSG00000264513	RP11-556O9.2	brown	-0.4147833
ENSG00000264529	RP11-556O9.3	grey	0.18140977
ENSG00000232310	RP11-557H15.4	yellow	0.61285392
ENSG00000271264	RP11-558F24.6	grey	0.32846776
ENSG00000260495	RP11-55K13.1	grey	0.18725258
ENSG00000254712	RP11-560B16.5	grey	0.30903834
ENSG00000234418	RP11-560I19.1	grey	0.31736106
ENSG00000259017	RP11-561B11.6	grey	0.29703415
ENSG00000224525	RP11-561I11.3	grey	-0.0482151
ENSG00000223974	RP11-561N12.4	grey	0.3390599
ENSG00000235349	RP11-561N12.6	grey	0.44778879
ENSG00000226904	RP11-561O23.8	grey	-0.0877074
ENSG00000239455	RP11-561O4.1	grey	0.1072529
ENSG00000250027	RP11-563E2.2	grey	0.4481356
ENSG00000215244	RP11-563J2.2	turquoise	0.45637302
ENSG00000249752	RP11-563M4.1	grey	0.24093217

ENSG00000243116	RP11-564C24.1	grey	0.30223492
ENSG00000231345	RP11-564C4.6	grey	0.31219574
ENSG00000255624	RP11-564D11.3	grey	-0.0679419
ENSG00000261453	RP11-565N2.1	grey	0.29711323
ENSG00000259815	RP11-565N2.2	grey	0.23758711
ENSG00000227094	RP11-565P22.2	grey	0.27706995
ENSG00000260804	RP11-566E18.3	blue	0.40143512
ENSG00000259425	RP11-566K19.5	grey	-0.1833107
ENSG00000259344	RP11-566K19.6	yellow	-0.4969276
ENSG00000228818	RP11-567G24.1	grey	0.37041068
ENSG00000233735	RP11-567G24.3	grey	0.32093403
ENSG00000270585	RP11-568G11.4	grey	0.29166412
ENSG00000242193	RP11-568K15.1	lightcyan	0.52447317
ENSG00000226862	RP11-569A11.1	grey	-0.0050789
ENSG00000225620	RP11-569A11.2	grey	0.40643291
ENSG00000237212	RP11-569G13.2	grey	0.24772251
ENSG00000259556	RP11-56B16.2	brown	0.38317992
ENSG00000256070	RP11-56H16.1	grey	-0.1867901
ENSG00000234043	RP11-56M3.1	grey	0.1401623
ENSG00000203402	RP11-571E6.3	grey	0.3597102
ENSG00000227383	RP11-571F15.2	grey	0.40474594
ENSG00000270039	RP11-571M6.17	yellow	-0.4405147
ENSG00000257499	RP11-571M6.8	magenta	0.67135997
ENSG00000251536	RP11-572C21.1	grey	0.17443135
ENSG00000240861	RP11-572F4.1	grey	0.04122887
ENSG00000270195	RP11-572O17.1	grey	0.36476871
ENSG00000220842	RP11-572P18.1	brown	0.4301118
ENSG00000232233	RP11-573D15.2	tan	0.49999285
ENSG00000230951	RP11-573D15.6	grey	0.14163621
ENSG00000261671	RP11-573G6.6	grey	-0.1701467
ENSG00000255445	RP11-573M3.3	grey	0.18582175
ENSG00000269189	RP11-573N10.1	grey	0.40587161
ENSG00000231509	RP11-574F11.3	grey	0.30860373
ENSG00000228606	RP11-574F21.2	grey	0.51785974
ENSG00000229990	RP11-574K11.8	grey	-0.0149869
ENSG00000259855	RP11-574O16.1	grey	0.17266627
ENSG00000238246	RP11-575A19.2	grey	0.23945685
ENSG00000261600	RP11-575H3.1	grey	0.11325324
ENSG00000235298	RP11-575L7.8	blue	-0.3718503
ENSG00000250068	RP11-576C12.1	grey	0.37413332
ENSG00000267793	RP11-576C2.1	grey	0.01590921
ENSG00000250057	RP11-576N17.5	grey	0.23804001
ENSG00000254050	RP11-577N1.1	turquoise	0.33761233
ENSG00000261377	RP11-578F21.12	grey	-0.0214327
ENSG00000254398	RP11-578F21.2	grey	0.14595905
ENSG00000261480	RP11-578F21.6	grey	0.23145426
ENSG00000254352	RP11-578O24.2	grey	0.28177205
ENSG00000253762	RP11-579E24.2	grey	0.43670077

ENSG00000270480	RP11-57B24.1	grey	0.27232208
ENSG00000270842	RP11-57B24.2	yellow	0.38595665
ENSG00000224797	RP11-57C19.6	grey	0.2965192
ENSG00000231992	RP11-57H12.2	grey	0.37344021
ENSG00000226026	RP11-57H12.3	grey	0.17527771
ENSG00000228852	RP11-57H12.5	grey	0.43992973
ENSG00000225292	RP11-57H14.3	grey	0.28286792
ENSG00000260917	RP11-57H14.4	yellow	-0.3552741
ENSG00000265374	RP11-57J16.1	grey	0.11553243
ENSG00000216809	RP11-57K17.1	grey	0.23425977
ENSG00000260780	RP11-580I1.1	grey	0.11220661
ENSG00000261621	RP11-580I1.2	grey	-0.0487676
ENSG00000263766	RP11-580I16.2	grey	0.1454551
ENSG00000256625	RP11-582E3.4	grey	0.25921377
ENSG00000251034	RP11-582J16.4	grey	0.26456335
ENSG00000253200	RP11-582J16.5	grey	0.45993434
ENSG00000264057	RP11-583F2.1	grey	0.01269869
ENSG00000266598	RP11-583F2.5	grey	0.26692236
ENSG00000253488	RP11-583M2.2	cyan	-0.4041988
ENSG00000257497	RP11-585P4.5	grey	0.10111093
ENSG00000249896	RP11-586D19.1	grey	-0.0949182
ENSG00000240752	RP11-588D3.1	grey	0.40991211
ENSG00000258168	RP11-588H23.3	grey	0.07021923
ENSG00000260244	RP11-588K22.2	turquoise	-0.5142186
ENSG00000258419	RP11-588P7.1	grey	0.16809078
ENSG00000258662	RP11-588P7.2	grey	-0.0767631
ENSG00000249710	RP11-588P8.1	black	0.53429391
ENSG00000226676	RP11-589B3.6	grey	0.28134562
ENSG00000271075	RP11-589M4.4	grey	0.27465529
ENSG00000269899	RP11-589N15.2	grey	0.24210837
ENSG00000262089	RP11-589P10.5	blue	-0.464464
ENSG00000257159	RP11-58A17.3	grey	0.37144482
ENSG00000269975	RP11-58B17.2	blue	0.76177536
ENSG00000267039	RP11-58G13.1	grey	0.16577278
ENSG00000243175	RP11-58H15.1	yellow	0.60758598
ENSG00000255092	RP11-58K22.4	yellow	0.38566269
ENSG00000212664	RP11-592N21.1	grey	0.24752338
ENSG00000270901	RP11-592P9.1	grey	0.43514874
ENSG00000254266	RP11-594N15.2	grey	0.32998489
ENSG00000260398	RP11-594N15.3	grey	-0.2332248
ENSG00000251429	RP11-597D13.7	yellow	0.73182047
ENSG00000248429	RP11-597D13.9	turquoise	0.41006139
ENSG00000256694	RP11-598F7.5	grey	0.21298611
ENSG00000256540	RP11-598F7.6	grey	0.17937646
ENSG00000248249	RP11-598O12.1	turquoise	0.68451368
ENSG00000259732	RP11-59H7.3	blue	-0.3533341
ENSG00000255105	RP11-5A11.1	grey	0.28296534
ENSG00000254644	RP11-5A11.2	grey	0.24372105

ENSG00000248878	RP11-5N11.1	grey	0.43225203
ENSG00000248624	RP11-5N11.2	grey	0.1450712
ENSG00000249114	RP11-5N11.3	grey	0.2052586
ENSG00000248813	RP11-5N11.4	brown	-0.4701932
ENSG00000248378	RP11-5N11.5	grey	0.40846758
ENSG00000250164	RP11-5N11.6	grey	0.27852889
ENSG00000249688	RP11-5N11.7	grey	0.26580482
ENSG00000261072	RP11-5N19.3	grey	0.12127959
ENSG00000260285	RP11-600F24.7	turquoise	-0.44027
ENSG00000258317	RP11-603J24.5	grey	0.21488153
ENSG00000258012	RP11-603K19.1	grey	0.21322111
ENSG00000177803	RP11-606P2.1	grey	0.23602739
ENSG00000265542	RP11-60A24.3	grey	0.24181522
ENSG00000257815	RP11-611E13.2	grey	0.19811496
ENSG00000260054	RP11-611L7.1	grey	-0.0700592
ENSG00000256325	RP11-611O2.1	grey	0.43506716
ENSG00000256185	RP11-612B6.2	turquoise	0.60322348
ENSG00000235183	RP11-613C6.4	grey	0.36553818
ENSG00000254409	RP11-613D13.4	grey	0.31665956
ENSG00000260084	RP11-615I2.1	grey	0.26259365
ENSG00000250130	RP11-616K22.1	grey	0.18727684
ENSG00000231160	RP11-617D20.1	grey	-0.0033481
ENSG00000259291	RP11-617F23.1	grey	0.01920615
ENSG00000258375	RP11-617J18.1	grey	0.30310682
ENSG00000261635	RP11-618N24.1	grey	0.01787929
ENSG00000255362	RP11-619A14.3	grey	0.15008002
ENSG00000225652	RP11-619F23.2	grey	0.13971856
ENSG00000249678	RP11-619J20.1	grey	0.32367534
ENSG00000253244	RP11-619L12.3	grey	0.28416731
ENSG00000253447	RP11-619L12.4	grey60	0.41200316
ENSG00000267028	RP11-619L19.1	grey	0.38407018
ENSG00000267402	RP11-619L19.2	grey	0.23790183
ENSG00000261705	RP11-61A14.2	grey	0.38807914
ENSG00000235033	RP11-61I13.3	yellow	0.70331032
ENSG00000260805	RP11-61J19.4	grey	0.11284467
ENSG00000257953	RP11-620J15.1	blue	-0.5049289
ENSG00000257698	RP11-620J15.3	brown	0.51752047
ENSG00000226439	RP11-621K7.1	grey	0.38297716
ENSG00000204272	RP11-622K12.1	yellow	-0.5197598
ENSG00000253535	RP11-624C23.1	grey	0.10782797
ENSG00000259345	RP11-624L4.1	grey	0.09571608
ENSG00000237742	RP11-624M8.1	grey	0.16133774
ENSG00000235902	RP11-626E13.1	green	0.38957529
ENSG00000259255	RP11-627D16.1	brown	-0.3974681
ENSG00000259509	RP11-627D16.2	black	0.60026042
ENSG00000247416	RP11-629G13.1	grey	0.33704429
ENSG00000261220	RP11-629O1.2	grey	0.03400059
ENSG00000240265	RP11-62G11.1	yellow	0.466328

ENSG00000270966	RP11-62H7.3	grey	0.34207381
ENSG00000242261	RP11-62J1.3	grey	0.06096328
ENSG00000224658	RP11-631F7.1	grey	0.56506227
ENSG00000230205	RP11-631F7.2	grey	0.51697691
ENSG00000250461	RP11-631M6.2	grey	0.17127917
ENSG00000251682	RP11-631M6.3	grey	0.29984551
ENSG00000257354	RP11-631N16.2	grey	0.4164154
ENSG00000257995	RP11-632B21.1	purple	-0.4945682
ENSG00000230202	RP11-632C17__A	grey	0.218108
ENSG00000254912	RP11-632K20.2	grey	-0.0676205
ENSG00000223509	RP11-632K20.7	grey	0.14937495
ENSG00000267390	RP11-635N19.1	grey	0.0464333
ENSG00000267430	RP11-635N19.2	grey	0.08289178
ENSG00000267413	RP11-636O21.1	purple	0.41565931
ENSG00000258581	RP11-638I2.10	grey	0.35861906
ENSG00000258904	RP11-638I2.2	grey	0.19797182
ENSG00000261886	RP11-63A1.1	grey	0.32186811
ENSG00000255132	RP11-63C8.1	grey	0.03105836
ENSG00000249868	RP11-63E5.6	grey	0.14546593
ENSG00000217653	RP11-63K6.1	grey	0.23439637
ENSG00000260558	RP11-63M22.1	grey	0.26317042
ENSG00000248466	RP11-640B6.1	greenyellow	-0.4211544
ENSG00000236943	RP11-640M9.1	grey	0.25327003
ENSG00000225241	RP11-640M9.2	grey	-0.1696649
ENSG00000264497	RP11-640N20.5	grey	0.43544488
ENSG00000232407	RP11-641C17.2	yellow	0.4852923
ENSG00000213178	RP11-641D5.1	brown	0.40120767
ENSG00000254141	RP11-642D21.1	grey	0.40259298
ENSG00000253420	RP11-642D21.2	cyan	-0.4499349
ENSG00000258311	RP11-644F5.10	brown	-0.3934368
ENSG00000258056	RP11-644F5.11	turquoise	0.59085887
ENSG00000213587	RP11-646D13.1	grey	0.36939256
ENSG00000269921	RP11-646I6.5	turquoise	0.39174308
ENSG00000270147	RP11-646I6.6	grey	0.23924999
ENSG00000253116	RP11-648L3.2	grey	0.28084078
ENSG00000248216	RP11-648M2.2	grey	0.25112202
ENSG00000254843	RP11-648O15.1	grey	0.44952777
ENSG00000253582	RP11-649G15.2	grey	0.24252787
ENSG00000213144	RP11-64B16.2	grey	0.18476052
ENSG00000256609	RP11-64B16.4	grey	0.06100415
ENSG00000261374	RP11-64K12.10	blue	0.48731272
ENSG00000248152	RP11-650J17.1	grey	0.09309156
ENSG00000249373	RP11-650J17.2	grey	0.31797598
ENSG00000271624	RP11-651K21.1	blue	-0.3964074
ENSG00000242791	RP11-651P23.5	grey	0.0253513
ENSG00000259621	RP11-654A16.1	grey	0.21462927
ENSG00000238287	RP11-656D10.3	grey	0.2987455
ENSG00000240086	RP11-657O9.1	grey	-0.1051849

ENSG00000213409	RP11-658F2.3	grey	0.19833909
ENSG00000240915	RP11-659E9.2	grey	0.21958731
ENSG00000254698	RP11-659G9.3	brown	-0.5369612
ENSG00000262454	RP11-65J21.3	grey	0.19960152
ENSG00000233901	RP11-65J3.1	yellow	-0.349394
ENSG00000260931	RP11-65L3.1	grey	-0.1368324
ENSG00000270956	RP11-65L3.4	grey	0.18650117
ENSG00000254682	RP11-660L16.2	grey	-0.1130746
ENSG00000265010	RP11-661C3.2	grey	0.42850896
ENSG00000258437	RP11-661G16.1	grey	0.38957827
ENSG00000266708	RP11-661O13.1	grey	0.19646291
ENSG00000242958	RP11-662B19.1	grey	0.29135791
ENSG00000254251	RP11-662G23.1	yellow	0.46045363
ENSG00000248801	RP11-664D7.4	grey	-0.2581119
ENSG00000256287	RP11-664H17.1	grey	0.37445365
ENSG00000249460	RP11-665C14.2	grey	0.15419943
ENSG00000239686	RP11-665C16.1	grey	0.41317508
ENSG00000258455	RP11-665C16.5	grey	0.4496428
ENSG00000249252	RP11-665G4.1	yellow	0.48165134
ENSG00000261543	RP11-665J16.1	grey	-0.0767794
ENSG00000248787	RP11-666A20.4	grey	0.33689281
ENSG00000267543	RP11-666A8.7	grey	0.39195829
ENSG00000234428	RP11-666F17.1	brown	-0.4519671
ENSG00000263050	RP11-667K14.3	grey	0.31222005
ENSG00000262810	RP11-667K14.5	grey	0.32414858
ENSG00000262402	RP11-667K14.8	grey	0.37613386
ENSG00000256452	RP11-667M19.10	grey	0.4766967
ENSG00000262881	RP11-669E14.4	grey	0.22440093
ENSG00000262372	RP11-669E14.6	grey	0.37686467
ENSG00000232386	RP11-66B24.2	grey	0.11221301
ENSG00000223356	RP11-66D17.5	grey	0.20038236
ENSG00000262020	RP11-66H6.3	grey	0.30977825
ENSG00000260081	RP11-66N11.8	grey	-0.0392594
ENSG00000157306	RP11-66N24.4	grey	-0.1449518
ENSG00000262951	RP11-670E13.2	grey	0.12166225
ENSG00000264263	RP11-671P2.1	grey	0.37185637
ENSG00000259781	RP11-673C5.1	grey	0.37274848
ENSG00000248828	RP11-673E1.4	grey	0.21314505
ENSG00000254563	RP11-673F18.1	grey	0.37429222
ENSG00000253361	RP11-675F6.3	grey	0.25693219
ENSG00000254705	RP11-675M1.2	grey	0.32106388
ENSG00000263958	RP11-676J15.1	magenta	0.46336407
ENSG00000254433	RP11-677I18.3	grey	0.248034
ENSG00000255045	RP11-677M14.2	black	0.66826591
ENSG00000250073	RP11-677M14.3	grey	-0.0057744
ENSG00000254941	RP11-677M14.5	turquoise	0.46154396
ENSG00000254509	RP11-677M14.6	grey	0.07243101
ENSG00000245498	RP11-677M14.7	grey	0.12130465

ENSG00000255040	RP11-677N16.1	grey	0.21060234
ENSG00000267143	RP11-677O4.6	grey	0.32295057
ENSG00000268081	RP11-678G14.2	grey	0.19353829
ENSG00000264596	RP11-678G15.1	grey	0.34428139
ENSG00000266767	RP11-678G15.2	grey	0.41188241
ENSG00000243813	RP11-678L1.1	grey	-0.1080324
ENSG00000250938	RP11-679C8.2	grey	0.25821392
ENSG00000230894	RP11-67K19.3	grey	0.36134105
ENSG00000257605	RP11-680A11.5	grey	0.12460603
ENSG00000263906	RP11-680F20.11	grey	-0.0838357
ENSG00000255027	RP11-680F20.9	grey	0.05982646
ENSG00000260872	RP11-680G24.5	grey	0.02490729
ENSG00000213287	RP11-680L20.1	grey	0.39433943
ENSG00000254044	RP11-681L8.1	grey	0.47706056
ENSG00000254884	RP11-682B13.2	grey	0.27668609
ENSG00000254924	RP11-684B2.3	grey	0.31006037
ENSG00000246308	RP11-685M7.3	grey	0.06131227
ENSG00000231662	RP11-686D16.1	grey	0.07602414
ENSG00000260030	RP11-686F15.2	grey	0.19648811
ENSG00000238168	RP11-686G8.1	grey	0.2968676
ENSG00000258099	RP11-686G8.2	grey	0.29553771
ENSG00000267167	RP11-687F6.1	grey	0.09680197
ENSG00000267587	RP11-687F6.4	grey	0.256262
ENSG00000255317	RP11-688I9.2	grey	0.02153522
ENSG00000254938	RP11-688I9.4	grey	0.26327977
ENSG00000205959	RP11-689P11.2	grey	-0.0351956
ENSG00000265625	RP11-68I3.11	grey	0.28707011
ENSG00000263370	RP11-68I3.5	grey	0.3568974
ENSG00000246792	RP11-68L18.1	grey	0.13470961
ENSG00000260966	RP11-690D19.3	brown	-0.2350501
ENSG00000266753	RP11-690G19.3	grey	0.15957824
ENSG00000237641	RP11-690I21.1	turquoise	0.4197761
ENSG00000261096	RP11-690I21.2	grey	0.18956413
ENSG00000267393	RP11-691H4.3	black	0.52366252
ENSG00000267694	RP11-691H4.4	black	0.5524624
ENSG00000258765	RP11-692C24.2	grey	0.10230631
ENSG00000250220	RP11-692D12.1	grey	0.21282633
ENSG00000270441	RP11-694I15.7	grey	0.03582966
ENSG00000256751	RP11-695J4.2	grey	0.30203549
ENSG00000224700	RP11-697E14.2	grey	0.28658588
ENSG00000270322	RP11-697E2.10	grey	0.34085839
ENSG00000271100	RP11-697H9.5	grey	0.25973824
ENSG00000253712	RP11-697M17.2	grey	0.37160466
ENSG00000253330	RP11-697N18.3	grey	0.22984334
ENSG00000269924	RP11-697N18.4	grey	0.44150494
ENSG00000261355	RP11-698N11.4	grey	0.21028809
ENSG00000182109	RP11-69E11.4	grey	-0.2088447
ENSG00000236673	RP11-69I8.2	grey	0.17379518

ENSG00000238221	RP11-69L16.4	grey	0.35036577
ENSG00000240739	RP11-69M1.1	grey	0.41041978
ENSG00000255565	RP11-6B19.1	brown	-0.5018588
ENSG00000249955	RP11-6E9.4	grey	0.47708553
ENSG00000243916	RP11-6K23.1	grey	0.22152108
ENSG00000266821	RP11-6N17.2	grey	0.26389853
ENSG00000266601	RP11-6N17.3	grey	0.36556947
ENSG00000264920	RP11-6N17.4	grey	-0.0256956
ENSG00000261616	RP11-6O2.3	grey	0.17117063
ENSG00000255348	RP11-700F16.3	grey	0.38543306
ENSG00000266954	RP11-701H16.4	red	-0.4678041
ENSG00000271347	RP11-701H24.7	turquoise	0.4380525
ENSG00000251139	RP11-701P16.2	grey	0.29803694
ENSG00000254653	RP11-702F3.1	grey	0.31223541
ENSG00000259217	RP11-702M1.2	grey	0.25185151
ENSG00000250908	RP11-703C10.1	grey	0.18447948
ENSG00000266049	RP11-703M24.5	grey	0.40019918
ENSG00000257027	RP11-705C15.3	grey	-0.112282
ENSG00000205664	RP11-706O15.1	grey	-0.0698839
ENSG00000205035	RP11-707M1.1	grey	-0.012492
ENSG00000254487	RP11-707M1.7	grey	0.29197967
ENSG00000255268	RP11-707M1.9	black	0.55404638
ENSG00000259678	RP11-707P17.1	grey	0.46060798
ENSG00000259668	RP11-707P17.2	grey	0.22237979
ENSG00000255028	RP11-708B6.2	grey	0.21728635
ENSG00000262973	RP11-708H21.4	grey	0.43406011
ENSG00000260236	RP11-708J19.1	grey	0.2571889
ENSG00000227514	RP11-709P2.1	grey	0.03192187
ENSG00000230001	RP11-70J12.1	grey	0.2922657
ENSG00000265194	RP11-70L8.4	grey	0.34494122
ENSG00000271474	RP11-710C12.1	lightgreen	0.44816314
ENSG00000250761	RP11-711G10.1	turquoise	0.37079824
ENSG00000255790	RP11-711K1.7	grey	0.04457495
ENSG00000245552	RP11-712B9.2	grey	0.3043397
ENSG00000270689	RP11-712B9.4	grey	0.2418116
ENSG00000270578	RP11-712B9.5	grey	0.33125127
ENSG00000266965	RP11-712P20.2	red	-0.5333186
ENSG00000265579	RP11-713C5.1	magenta	0.7408429
ENSG00000244604	RP11-713H12.1	grey	0.41032424
ENSG00000270606	RP11-713H12.2	grey	0.23929731
ENSG00000204241	RP11-713P17.3	grey	-0.1388871
ENSG00000250410	RP11-714G18.1	grey	0.31227851
ENSG00000231362	RP11-715I4.1	grey	0.32778226
ENSG00000260095	RP11-715J22.3	grey	0.25402518
ENSG00000260293	RP11-715J22.6	grey	0.17786863
ENSG00000255015	RP11-716H6.1	grey	0.13704884
ENSG00000255219	RP11-716H6.2	grey	0.05118127
ENSG00000254441	RP11-718B12.5	grey	0.35907175

ENSG00000225727	RP11-71C5.2	grey	0.37767677
ENSG00000250934	RP11-71E19.1	grey	0.09159465
ENSG00000251448	RP11-71E19.2	grey	0.30561001
ENSG00000242199	RP11-71H17.1	cyan	-0.4706386
ENSG00000260391	RP11-71H17.7	blue	0.64286444
ENSG00000239922	RP11-71N10.1	yellow	0.37071822
ENSG00000265477	RP11-720L2.2	grey	0.20935317
ENSG00000264514	RP11-720L2.4	grey	0.32152239
ENSG00000259434	RP11-720L8.1	blue	0.3307486
ENSG00000253586	RP11-723D22.2	grey	0.41922524
ENSG00000253829	RP11-723D22.3	grey	0.38616012
ENSG00000261159	RP11-723O4.9	grey	0.18693575
ENSG00000251523	RP11-724M22.1	grey	0.32175178
ENSG00000255234	RP11-727A23.10	grey	0.04978688
ENSG00000254676	RP11-727A23.4	grey	0.42072321
ENSG00000247137	RP11-727A23.5	grey	-0.1738842
ENSG00000267811	RP11-727F15.11	grey	0.12905934
ENSG00000269176	RP11-727F15.12	grey	0.17872959
ENSG00000269463	RP11-727F15.13	grey	0.39844402
ENSG00000265043	RP11-728E14.3	grey	-0.0832797
ENSG00000224016	RP11-728K20.1	grey	0.21184854
ENSG00000260555	RP11-728K20.2	grey	-0.0624133
ENSG00000237631	RP11-72B4.2	grey	0.41203599
ENSG00000238079	RP11-72I2.1	grey	0.37498355
ENSG00000260735	RP11-72I8.1	grey	0.10682356
ENSG00000228330	RP11-730A19.5	grey	0.16908994
ENSG00000270822	RP11-730B22.1	grey	0.44149117
ENSG00000249012	RP11-731D1.1	grey	0.34994537
ENSG00000242431	RP11-731J8.1	grey	0.32121162
ENSG00000260918	RP11-731J8.2	red	0.78564451
ENSG00000238019	RP11-732M18.2	grey	0.37261886
ENSG00000236537	RP11-732M18.3	green	0.75323353
ENSG00000270344	RP11-734K2.4	grey	0.18970341
ENSG00000226409	RP11-735G4.1	grey	0.00448454
ENSG00000255471	RP11-736K20.5	grey	0.38578542
ENSG00000266397	RP11-737O24.1	grey	0.36573523
ENSG00000265907	RP11-737O24.2	grey	0.28085206
ENSG00000263606	RP11-737O24.3	grey	0.11669624
ENSG00000226330	RP11-739N20.2	grey	0.11974727
ENSG00000248206	RP11-739P1.2	grey	0.43490767
ENSG00000260978	RP11-73C9.1	grey	0.27103316
ENSG00000237088	RP11-73E6.2	grey	0.31656182
ENSG00000260526	RP11-73K9.2	grey	0.22535702
ENSG00000270591	RP11-73M18.11	grey	0.26851429
ENSG00000270108	RP11-73M18.6	grey	0.27251831
ENSG00000269958	RP11-73M18.8	blue	-0.4652645
ENSG00000269963	RP11-73M18.9	grey	0.01511744
ENSG00000230459	RP11-741G21.1	grey	0.36364347

ENSG00000270112	RP11-742D12.2	grey	0.09556482
ENSG00000227097	RP11-742N3.1	grey	-0.1623596
ENSG00000267354	RP11-749H17.2	grey	0.28485778
ENSG00000262050	RP11-74E22.3	grey	0.21507039
ENSG00000261963	RP11-74E22.4	grey	0.19940504
ENSG00000258035	RP11-74K11.2	grey	0.15796328
ENSG00000262902	RP11-750B16.1	grey	0.10738579
ENSG00000255197	RP11-750H9.5	cyan	0.55411792
ENSG00000270072	RP11-750H9.7	yellow	-0.5102357
ENSG00000226222	RP11-753E22.3	grey	0.3792896
ENSG00000255158	RP11-754B17.1	grey	0.17368343
ENSG00000239801	RP11-755B10.2	grey	0.21533571
ENSG00000255320	RP11-755F10.1	blue	-0.389391
ENSG00000254461	RP11-755F10.3	grey	0.30418031
ENSG00000243141	RP11-757G14.1	grey	0.3522866
ENSG00000253849	RP11-759A9.1	grey	0.29346395
ENSG00000254392	RP11-759A9.2	grey	0.14497011
ENSG00000224072	RP11-75A9.3	grey	0.10656688
ENSG00000225706	RP11-75C9.1	brown	-0.3922501
ENSG00000234021	RP11-75C9.2	grey	0.22851027
ENSG00000213443	RP11-75L1.2	grey	0.02265341
ENSG00000254343	RP11-760H22.2	grey	0.31334115
ENSG00000264707	RP11-760N9.1	brown	-0.3512546
ENSG00000235244	RP11-761E20.1	lightgreen	0.66612287
ENSG00000259322	RP11-762H8.1	grey	0.27223648
ENSG00000259562	RP11-762H8.2	grey	0.39444677
ENSG00000257509	RP11-762I7.4	grey	0.38081569
ENSG00000249049	RP11-763F8.1	grey	0.31002696
ENSG00000241400	RP11-764I5.1	grey	0.38107835
ENSG00000225411	RP11-764K9.1	grey	0.13721921
ENSG00000215548	RP11-764K9.4	grey	0.07118033
ENSG00000266501	RP11-766H1.1	grey	0.1977267
ENSG00000255693	RP11-766N7.3	grey	0.01880714
ENSG00000248995	RP11-766N7.4	grey	0.35689836
ENSG00000239835	RP11-767L7.1	blue	-0.294078
ENSG00000234329	RP11-767N6.2	grey	0.29056538
ENSG00000240393	RP11-768G7.1	grey	-0.0341369
ENSG00000241213	RP11-768G7.2	grey	0.12399342
ENSG00000242816	RP11-768G7.3	grey	0.03691405
ENSG00000250354	RP11-76G10.1	grey	0.44268993
ENSG00000255557	RP11-770G2.2	grey	0.21837812
ENSG00000255435	RP11-770J1.3	grey	0.07446042
ENSG00000255384	RP11-770J1.4	grey	0.26325196
ENSG00000269944	RP11-770J1.7	cyan	-0.4234232
ENSG00000239344	RP11-771F20.1	grey	0.30215469
ENSG00000225484	RP11-773D16.1	grey	0.20257358
ENSG00000266969	RP11-773H22.4	brown	-0.5381383
ENSG00000251615	RP11-774O3.3	grey	0.13837898

ENSG00000151963	RP11-775A3.1	grey	0.34119155
ENSG00000243508	RP11-775J23.2	grey	0.30963847
ENSG00000226476	RP11-776H12.1	grey	-0.1722361
ENSG00000255365	RP11-776K3.1	grey	0.40656083
ENSG00000270510	RP11-777F6.3	grey	0.36411702
ENSG00000256120	RP11-778H2.1	turquoise	0.48406523
ENSG00000250062	RP11-778J15.1	grey	0.29292467
ENSG00000234149	RP11-77G23.2	grey	0.43806914
ENSG00000260276	RP11-77H9.2	grey	0.03924808
ENSG00000261476	RP11-77K12.3	grey	0.20117833
ENSG00000262583	RP11-77K12.5	grey	0.18686164
ENSG00000232228	RP11-77O7.1	grey	0.44603214
ENSG00000249846	RP11-77P16.4	grey	0.07871795
ENSG00000227896	RP11-77P6.2	grey	0.25137959
ENSG00000258066	RP11-781A6.1	grey	0.30567995
ENSG00000232274	RP11-782C8.2	grey	0.06090054
ENSG00000232336	RP11-782C8.3	grey	0.05810941
ENSG00000237291	RP11-782C8.4	grey	-0.0013591
ENSG00000225278	RP11-782C8.5	grey	-0.0087499
ENSG00000256537	RP11-785H5.1	grey	0.45321362
ENSG00000256712	RP11-785H5.2	brown	-0.6576629
ENSG00000253711	RP11-787D18.1	grey	0.08238769
ENSG00000266149	RP11-789C17.3	grey	0.45316301
ENSG00000263970	RP11-789C17.5	grey	0.25771813
ENSG00000267455	RP11-78A19.2	grey	0.17871967
ENSG00000263551	RP11-78F17.1	grey	0.25415469
ENSG00000270091	RP11-78O7.2	grey	-0.0257737
ENSG00000236928	RP11-792A8.1	grey	0.38338706
ENSG00000271064	RP11-792A8.3	brown	-0.4341573
ENSG00000266846	RP11-793A3.2	yellow	0.34990261
ENSG00000270175	RP11-793H13.11	grey	0.20839797
ENSG00000256443	RP11-794G24.1	darkorange	0.59581449
ENSG00000267127	RP11-795F19.5	brown	0.4427777
ENSG00000226541	RP11-795J1.2	grey	0.33480898
ENSG00000258512	RP11-796G6.2	grey	0.15926315
ENSG00000260787	RP11-797A18.4	grey	0.23389593
ENSG00000269951	RP11-797A18.6	grey	0.06192208
ENSG00000234338	RP11-797H7.1	darkgrey	0.65871823
ENSG00000213640	RP11-797H7.3	grey	0.19216497
ENSG00000189316	RP11-797H7.5	grey	-0.0883752
ENSG00000271332	RP11-797J4.1	grey	0.13860084
ENSG00000131484	RP11-798G7.5	grey	0.14410153
ENSG00000266918	RP11-798G7.8	grey	0.01026591
ENSG00000253520	RP11-798K23.5	grey	0.22809547
ENSG00000205682	RP11-798L4.1	grey	0.23903314
ENSG00000248774	RP11-798M19.3	grey	0.46772544
ENSG00000264924	RP11-799B12.2	grey	0.36329429
ENSG00000265750	RP11-799B12.4	cyan	-0.4893655

ENSG00000234248	RP11-799O21.2	grey	0.36488758
ENSG00000236426	RP11-79M19.2	grey	0.11368392
ENSG00000237950	RP11-7O11.3	grey	0.17197582
ENSG00000267015	RP11-800A18.4	grey	0.22744312
ENSG00000254730	RP11-801G16.2	grey	0.24220861
ENSG00000244699	RP11-803B1.2	grey	0.14645764
ENSG00000270190	RP11-803D5.4	grey	0.26151627
ENSG00000249106	RP11-806K15.1	turquoise	0.54799061
ENSG00000253671	RP11-806O11.1	turquoise	0.52026544
ENSG00000225140	RP11-809C18.3	yellow	0.72060385
ENSG00000242357	RP11-809C18.5	yellow	0.52656807
ENSG00000258102	RP11-809C9.2	grey	0.12883212
ENSG00000227535	RP11-809N15.2	grey	0.13148954
ENSG00000256148	RP11-809N8.5	grey	0.27175081
ENSG00000255786	RP11-809N8.6	grey	0.33275901
ENSG00000253210	RP11-809O17.1	grey	0.15679651
ENSG00000240996	RP11-80H5.7	grey	0.32546835
ENSG00000241679	RP11-80H8.4	grey	0.2827135
ENSG00000242318	RP11-810D13.1	grey	0.22862614
ENSG00000270413	RP11-810O3.2	grey	0.08904657
ENSG00000270581	RP11-811P12.3	greenyellow	-0.4906453
ENSG00000257897	RP11-812D23.1	red	0.56649068
ENSG00000264885	RP11-815I9.4	grey	0.39775666
ENSG00000259276	RP11-815J21.3	grey	0.28740847
ENSG00000259367	RP11-815J21.4	grey	0.27139895
ENSG00000261098	RP11-819C21.1	brown	-0.5583863
ENSG00000213252	RP11-819M15.1	grey	0.38763095
ENSG00000213170	RP11-81B10.2	grey	0.25102876
ENSG00000241316	RP11-81N13.1	turquoise	0.42209244
ENSG00000256159	RP11-820K3.4	grey	0.44531072
ENSG00000255090	RP11-820L6.1	green	0.59621466
ENSG00000266466	RP11-822E23.2	grey	0.20517
ENSG00000266673	RP11-822E23.3	grey	0.38412194
ENSG00000248469	RP11-826N14.2	grey	-0.1125128
ENSG00000258968	RP11-829H16.5	grey	0.39485834
ENSG00000224945	RP11-82L18.2	grey	-0.0464849
ENSG00000231632	RP11-82L18.4	purple	0.54623434
ENSG00000231985	RP11-82L20.1	grey	-0.084971
ENSG00000263657	RP11-82O19.1	grey	0.13350601
ENSG00000265713	RP11-82O19.2	grey	0.30262205
ENSG00000258400	RP11-831F12.2	grey	0.17207444
ENSG00000255129	RP11-839D17.3	grey	0.20811466
ENSG00000235096	RP11-83A16.1	grey	0.38935805
ENSG00000248863	RP11-83A24.1	grey	0.10898696
ENSG00000214650	RP11-83B20.1	grey	0.20200362
ENSG00000231409	RP11-83J16.1	grey	0.29343444
ENSG00000249736	RP11-83M16.5	grey	0.44306442
ENSG00000259118	RP11-840I19.3	grey	0.08137505

ENSG00000251414	RP11-843P14.1	grey	0.36801779
ENSG00000250909	RP11-843P14.2	grey	0.38948469
ENSG00000251667	RP11-844P9.3	grey	0.2223382
ENSG00000267504	RP11-845C23.2	grey	0.42519397
ENSG00000257829	RP11-845M18.6	grey	-0.1229894
ENSG00000265743	RP11-848P1.3	cyan	0.51994168
ENSG00000266340	RP11-848P1.4	blue	-0.3872939
ENSG00000264148	RP11-848P1.7	grey	0.40895767
ENSG00000266865	RP11-848P1.9	grey	-0.0476826
ENSG00000265749	RP11-849F2.5	grey	0.22030136
ENSG00000263809	RP11-849F2.7	grey	0.23676907
ENSG00000269928	RP11-849F2.8	grey	0.17705272
ENSG00000269947	RP11-849F2.9	grey	0.25109673
ENSG00000254469	RP11-849H4.2	grey	0.16838331
ENSG00000263146	RP11-849I19.1	turquoise	0.68038664
ENSG00000248692	RP11-84A1.3	grey	0.25732933
ENSG00000203620	RP11-84A19.2	grey	0.26050539
ENSG00000271359	RP11-84C13.1	grey	0.18976371
ENSG00000270720	RP11-84C13.2	grey	0.21626502
ENSG00000254148	RP11-84E24.2	grey	0.28227031
ENSG00000257557	RP11-84G21.1	grey	0.15781571
ENSG00000260986	RP11-854K16.3	grey	0.35302481
ENSG00000266717	RP11-855A2.3	grey	0.2885375
ENSG00000214283	RP11-85F14.1	grey	0.1999267
ENSG00000239213	RP11-85F14.5	grey	-0.016593
ENSG00000258704	RP11-85K15.2	grey	0.47015449
ENSG00000244128	RP11-85M11.2	grey	-0.1992546
ENSG00000234921	RP11-85O21.5	grey	0.21471139
ENSG00000267108	RP11-861E21.1	grey	0.3511758
ENSG00000267199	RP11-861E21.2	grey	0.15752436
ENSG00000258490	RP11-862P13.1	grey	0.1561045
ENSG00000233170	RP11-863K10.4	grey	0.10598568
ENSG00000258136	RP11-864J10.4	grey	0.22479838
ENSG00000227615	RP11-864N7.2	grey	-0.2702032
ENSG00000254337	RP11-865I6.2	grey	0.49066182
ENSG00000245156	RP11-867G23.3	grey	0.42231961
ENSG00000255468	RP11-867G23.8	grey	0.21368039
ENSG00000269290	RP11-869B15.1	yellow	-0.5017292
ENSG00000266513	RP11-874J12.3	grey	0.23731852
ENSG00000266401	RP11-874J12.4	grey	-0.0810661
ENSG00000263013	RP11-876N24.5	green	0.35389201
ENSG00000267568	RP11-87G24.3	grey	0.12963082
ENSG00000267521	RP11-87G24.6	grey	0.47128326
ENSG00000233961	RP11-87H9.2	grey	0.00113124
ENSG00000231242	RP11-87H9.3	grey	-0.1076619
ENSG00000234360	RP11-87N24.1	grey	0.60452784
ENSG00000232035	RP11-87N24.3	grey	0.17763676
ENSG00000257052	RP11-881M11.2	grey	0.17506744

ENSG00000256150	RP11-885B4.1	grey	0.26746082
ENSG00000255669	RP11-885B4.2	grey	0.33751056
ENSG00000186076	RP11-887P2.3	cyan	-0.4565129
ENSG00000258274	RP11-887P2.5	yellow	0.45213522
ENSG00000258303	RP11-887P2.6	pink	0.48030685
ENSG00000218227	RP11-889L3.1	grey	-0.0334004
ENSG00000270059	RP11-88H12.2	grey	-0.0425035
ENSG00000231437	RP11-88H9.2	grey	-0.127298
ENSG00000233178	RP11-88I18.2	red	-0.5083802
ENSG00000266413	RP11-88L24.4	grey	-0.1083418
ENSG00000255455	RP11-890B15.3	grey	-0.0141015
ENSG00000246451	RP11-894P9.1	grey	0.1718808
ENSG00000261810	RP11-895K13.2	black	0.47132551
ENSG00000259658	RP11-89K11.1	grey	0.18960683
ENSG00000259439	RP11-89K21.1	magenta	0.49202402
ENSG00000215184	RP11-8L18.2	turquoise	0.64926559
ENSG00000247287	RP11-902B17.1	magenta	-0.4774817
ENSG00000271417	RP11-909M7.3	blue	0.51540292
ENSG00000256101	RP11-90D4.3	grey	0.0876077
ENSG00000258204	RP11-90E9.1	grey	0.20950295
ENSG00000230229	RP11-90J7.4	grey	0.09775816
ENSG00000235121	RP11-90L20.2	yellow	0.36898577
ENSG00000255115	RP11-91P24.3	grey	0.28121324
ENSG00000254691	RP11-91P24.5	grey	0.15865653
ENSG00000255449	RP11-91P24.6	grey	0.29976995
ENSG00000254459	RP11-91P24.7	grey	0.16630722
ENSG00000260122	RP11-923I11.3	turquoise	-0.4990358
ENSG00000260473	RP11-923I11.4	grey	0.196525
ENSG00000261586	RP11-923I11.6	grey	-0.0666933
ENSG00000265218	RP11-927P21.1	grey	0.10004312
ENSG00000266155	RP11-927P21.6	grey	0.11444698
ENSG00000264673	RP11-92B11.3	grey	0.05907503
ENSG00000260088	RP11-92G12.3	grey	0.13920373
ENSG00000254205	RP11-92K15.1	grey	0.27961409
ENSG00000259483	RP11-930O11.2	grey	0.45334293
ENSG00000261747	RP11-932O9.3	black	0.51664205
ENSG00000270016	RP11-932O9.8	black	0.45754124
ENSG00000269930	RP11-932O9.9	grey	-0.2842755
ENSG00000250608	RP11-933H2.4	grey	0.12429494
ENSG00000234942	RP11-93H12.2	grey	0.26929458
ENSG00000270002	RP11-93H12.4	grey	0.09173902
ENSG00000218475	RP11-93K7.1	grey	0.20136977
ENSG00000226448	RP11-93L14.1	yellow	0.5813383
ENSG00000250624	RP11-93M12.1	grey	0.14970621
ENSG00000236869	RP11-944L7.4	grey	0.38781344
ENSG00000254241	RP11-946L20.1	yellow	0.69626801
ENSG00000253877	RP11-946L20.2	yellow	0.79620778
ENSG00000258646	RP11-950C14.3	grey	0.29891579

ENSG00000248885	RP11-950K24.2	grey	0.13193646
ENSG00000257507	RP11-956E11.1	grey	0.48544013
ENSG00000269973	RP11-95D17.1	blue	0.44165507
ENSG00000260171	RP11-95H11.1	grey	0.05703954
ENSG00000262516	RP11-95J11.1	grey	0.24014738
ENSG00000267651	RP11-95O2.1	grey	0.22451132
ENSG00000267707	RP11-95O2.5	grey	0.23650357
ENSG00000230024	RP11-95P13.1	grey	0.23672321
ENSG00000257126	RP11-966I7.1	magenta	0.37312178
ENSG00000257056	RP11-966I7.2	yellow	0.49245486
ENSG00000257748	RP11-966I7.3	grey	0.27445962
ENSG00000247934	RP11-967K21.1	grey	0.29515671
ENSG00000257596	RP11-968A15.2	grey	0.16839677
ENSG00000256139	RP11-968O1.5	grey	0.18516058
ENSG00000237280	RP11-96C23.9	grey	0.17644618
ENSG00000260891	RP11-96D1.5	grey	0.18866422
ENSG00000259072	RP11-96D24.1	yellow	0.48767339
ENSG00000261502	RP11-96H17.1	grey	0.25419869
ENSG00000269986	RP11-96H17.3	grey	0.27104617
ENSG00000257261	RP11-96H19.1	grey	0.05427652
ENSG00000232811	RP11-96K19.2	grey	0.05038798
ENSG00000261654	RP11-96K19.4	grey	0.32116506
ENSG00000267249	RP11-973H7.3	grey	0.16585279
ENSG00000259116	RP11-973N13.4	blue	-0.435642
ENSG00000257740	RP11-977G19.12	grey	0.15485979
ENSG00000258199	RP11-977G19.5	grey	0.10649951
ENSG00000249487	RP11-97O12.2	grey	-0.0498644
ENSG00000261451	RP11-981G7.1	grey	0.08053179
ENSG00000253641	RP11-981G7.2	grey	0.19549016
ENSG00000253678	RP11-981G7.3	grey	0.25355716
ENSG00000258302	RP11-981P6.1	grey	0.15707695
ENSG00000256050	RP11-982M15.6	grey	-0.073779
ENSG00000258736	RP11-982M15.7	grey	0.14931059
ENSG00000257475	RP11-983P16.2	grey	0.52180815
ENSG00000257337	RP11-983P16.4	green	0.73902271
ENSG00000261200	RP11-989E6.10	grey	-0.0311783
ENSG00000256299	RP11-989F5.3	grey	0.1471031
ENSG00000227045	RP11-98D18.1	grey	0.19275543
ENSG00000269621	RP11-98D18.15	grey	0.11568673
ENSG00000269489	RP11-98D18.17	grey	-0.016957
ENSG00000203288	RP11-98D18.9	grey	0.02313589
ENSG00000248978	RP11-98H4.1	grey	0.19314107
ENSG00000254212	RP11-98H4.4	grey	0.22156109
ENSG00000228506	RP11-98I9.4	grey	0.22573735
ENSG00000198237	RP11-98J23.2	grey	0.04219065
ENSG00000257176	RP11-996F15.2	grey	0.14428605
ENSG00000259969	RP11-999E24.3	turquoise	0.52602209
ENSG00000253495	RP11-99I9.2	yellow	0.47336767

ENSG00000250241	RP11-9G1.3	grey	-0.195689
ENSG00000230898	RP11-9L18.3	grey	0.28291188
ENSG00000265298	RP13-104F24.3	grey	0.25321008
ENSG00000244730	RP13-1056D16.2	grey	0.30892703
ENSG00000259277	RP13-126C7.1	grey	0.41015522
ENSG00000223343	RP13-131K19.2	grey	0.08279371
ENSG00000227844	RP13-143G15.3	blue	-0.3851305
ENSG00000237596	RP13-143G15.4	black	0.52619572
ENSG00000231181	RP13-15M17.1	grey	0.5125807
ENSG00000260011	RP13-20L14.1	grey	0.12574774
ENSG00000228487	RP13-225O21.2	grey	0.21125591
ENSG00000229369	RP13-225O21.5	grey	0.52320223
ENSG00000230491	RP13-228J13.10	grey	0.45795278
ENSG00000265399	RP13-270P17.2	yellow	0.44366779
ENSG00000255237	RP13-317D12.3	grey	0.22642156
ENSG00000203397	RP13-36G14.3	grey	0.04659992
ENSG00000226465	RP13-401N8.1	grey	0.19477022
ENSG00000251244	RP13-487K5.1	grey	0.24452732
ENSG00000249453	RP13-497K6.1	grey	0.34011561
ENSG00000261496	RP13-514E23.1	darkred	0.35258438
ENSG00000260563	RP13-516M14.1	grey	0.02983771
ENSG00000246448	RP13-578N3.3	grey	0.06232945
ENSG00000253716	RP13-582O9.5	grey	0.02811477
ENSG00000240888	RP13-635I23.3	grey	0.29789334
ENSG00000262652	RP13-638C3.2	grey	0.36504865
ENSG00000264569	RP13-650J16.1	grey	0.11233393
ENSG00000254757	RP13-726E6.1	grey	0.16590874
ENSG00000255367	RP13-726E6.2	grey	0.22799175
ENSG00000263531	RP13-753N3.1	grey	0.27248736
ENSG00000225460	RP13-93L13.1	yellow	0.4687778
ENSG00000231799	RP13-93L13.2	yellow	0.53990183
ENSG00000256742	RP13-941N14.1	grey	0.19402604
ENSG00000102218	RP2	turquoise	0.40102534
ENSG00000230322	RP3-323N1.2	grey	0.09578576
ENSG00000227954	RP3-323P13.2	grey	-0.0033867
ENSG00000226310	RP3-323P24.3	grey	0.14892063
ENSG00000101898	RP3-324O17.4	grey	0.27467121
ENSG00000234263	RP3-325F22.3	grey	0.38786621
ENSG00000230597	RP3-331H24.4	grey	0.35551739
ENSG00000196634	RP3-337D23.3	grey	0.48832897
ENSG00000219470	RP3-337H4.6	grey	0.25180622
ENSG00000233848	RP3-348I23.2	grey	0.3996714
ENSG00000216316	RP3-354N19.3	grey	0.16984911
ENSG00000271644	RP3-365I19.2	grey	0.33425962
ENSG00000271430	RP3-368A4.5	grey	-0.1879689
ENSG00000271533	RP3-368A4.6	green	0.50586012
ENSG00000255608	RP3-377H17.2	grey	0.15992699
ENSG00000233064	RP3-380B8.4	grey	0.46617309

ENSG00000234084	RP3-388E23.2	grey	0.0235346
ENSG00000261251	RP3-388M5.9	grey	0.30004125
ENSG00000226153	RP3-389A20.5	grey	0.15769901
ENSG00000218631	RP3-395C13.1	grey	0.15912822
ENSG00000226193	RP3-398G3.5	grey	0.42540105
ENSG00000228624	RP3-399L15.3	yellow	0.61174811
ENSG00000228839	RP3-400N23.6	grey	0.14927134
ENSG00000220694	RP3-403A15.1	grey	0.22676552
ENSG00000236324	RP3-403L10.3	grey	0.19924729
ENSG00000257298	RP3-405J10.3	grey	0.48890245
ENSG00000220660	RP3-406A7.3	grey	0.35125246
ENSG00000216613	RP3-406A7.5	yellow	0.60669873
ENSG00000260418	RP3-406A7.7	blue	-0.3863249
ENSG00000258603	RP3-414A15.10	grey	0.22181058
ENSG00000258695	RP3-414A15.2	grey	0.15736833
ENSG00000220506	RP3-415N12.1	grey	0.31588857
ENSG00000224384	RP3-417O22.3	red	-0.4812889
ENSG00000226684	RP3-418C23.2	grey	0.14633254
ENSG00000270031	RP3-426I6.6	grey	0.39928674
ENSG00000244627	RP3-449O17.1	blue	-0.4491637
ENSG00000218730	RP3-453I5.2	grey	0.37371774
ENSG00000257877	RP3-462E2.3	grey	0.25583019
ENSG00000270018	RP3-462E2.5	grey	-0.0431427
ENSG00000270638	RP3-466P17.1	grey	0.18761524
ENSG00000236266	RP3-467L1.4	grey	0.24514303
ENSG00000269925	RP3-467L1.6	grey	0.34881397
ENSG00000260000	RP3-467N11.1	grey	-0.067938
ENSG00000233183	RP3-468B3.2	grey	0.12029288
ENSG00000270890	RP3-468K18.6	grey	0.29195714
ENSG00000225891	RP3-476K8.3	grey	0.22270102
ENSG00000268340	RP3-477O4.16	grey	0.16787372
ENSG00000235008	RP3-497J21.1	grey	-0.1493671
ENSG00000196564	RP3-499B10.3	grey	0.30565375
ENSG00000228274	RP3-508I15.9	grey	0.20697006
ENSG00000247925	RP3-510L9.1	greenyellow	-0.4614331
ENSG00000236166	RP3-523C21.2	grey	0.19117473
ENSG00000271218	RP3-523E19.2	grey	0.4852409
ENSG00000220920	RP3-525L6.2	grey	0.4932899
ENSG00000230910	RP3-525N10.2	grey	-0.4877226
ENSG00000227541	RP3-525N14.2	grey	0.18391394
ENSG00000232043	RP4-530I15.9	grey	0.1851263
ENSG00000228634	RP4-534N18.2	grey	0.45203432
ENSG00000235189	RP4-537K23.4	yellow	0.59960017
ENSG00000227290	RP4-544H6.2	grey	0.28618297
ENSG00000261101	RP4-545K15.5	grey	-0.1303054
ENSG00000261786	RP4-555D20.2	floralwhite	0.62132465
ENSG00000260464	RP4-561L24.3	grey	0.1392108
ENSG00000203650	RP4-562J12.2	grey	0.15462366

ENSG00000230498	RP4-564M11.2	purple	0.50055288
ENSG00000227733	RP4-565E6.1	turquoise	-0.3533511
ENSG00000231119	RP4-569M23.2	grey	0.02650368
ENSG00000225938	RP4-575N6.4	turquoise	0.49613251
ENSG00000229291	RP4-580N22.2	grey	0.27624629
ENSG00000240449	RP4-584D14.5	grey	0.22987778
ENSG00000239377	RP4-584D14.6	brown	-0.4815839
ENSG00000232564	RP4-591N18.2	magenta	-0.4510229
ENSG00000260938	RP4-593C16.3	lightgreen	-0.5397963
ENSG00000223787	RP4-593M8.1	grey	0.36591505
ENSG00000264443	RP4-594I10.3	brown	-0.4927672
ENSG00000237640	RP4-604G5.1	grey	0.12058629
ENSG00000267734	RP4-604K5.3	cyan	-0.406079
ENSG00000225490	RP4-610C12.3	grey	0.01682898
ENSG00000261737	RP4-612B15.3	magenta	-0.4163681
ENSG00000261582	RP4-614O4.11	cyan	-0.4684691
ENSG00000235088	RP4-620E11.4	grey	0.41090139
ENSG00000230953	RP4-631H13.6	grey	-0.0316903
ENSG00000238061	RP4-635A23.4	grey	0.21506306
ENSG00000230337	RP4-635E18.6	grey	0.00369611
ENSG00000235501	RP4-639F20.1	grey	0.08411191
ENSG00000266993	RP4-657D16.3	grey	0.22558796
ENSG00000270728	RP4-657E11.10	turquoise	0.46224303
ENSG00000271554	RP4-665N4.8	grey	0.40712009
ENSG00000231703	RP4-669H2.1	grey	0.0077757
ENSG00000237094	RP4-669L17.10	grey	0.00294246
ENSG00000232710	RP4-669P10.16	grey	0.1947532
ENSG00000227370	RP4-669P10.19	grey	0.1395632
ENSG00000232528	RP4-673D20.3	turquoise	0.48039369
ENSG00000234282	RP4-673D20.4	grey	0.25431771
ENSG00000271461	RP4-673D20.6	turquoise	0.35005698
ENSG00000228988	RP4-677H15.4	grey	0.2676323
ENSG00000181227	RP4-682C21.2	grey	-0.0917015
ENSG00000255542	RP4-683L5.1	turquoise	0.55146267
ENSG00000231622	RP4-700A9.1	grey	0.3131737
ENSG00000234225	RP4-704D21.2	grey	0.36393288
ENSG00000234593	RP4-704D23.1	grey	0.40217965
ENSG00000255480	RP4-710M3.2	grey	0.32386476
ENSG00000229635	RP4-713B5.2	grey	0.1824153
ENSG00000261254	RP4-714D9.5	grey	0.17031028
ENSG00000229567	RP4-717I23.2	grey	0.47687001
ENSG00000223745	RP4-717I23.3	grey	-0.2888895
ENSG00000238194	RP4-719C8.1	grey	0.35281194
ENSG00000228204	RP4-724E13.2	yellow	0.64683504
ENSG00000236274	RP4-728D4.3	grey	0.42281305
ENSG00000232450	RP4-730K3.3	grey	0.36330904
ENSG00000241720	RP4-735C1.4	grey	0.07224546
ENSG00000233423	RP4-736H5.3	grey	0.09440434

ENSG00000269896	RP4-740C4.6	grey	0.14051246
ENSG00000236526	RP4-742J24.2	yellow	0.72549938
ENSG00000258545	RP4-755D9.1	grey	0.08420468
ENSG00000235513	RP4-756G23.5	yellow	0.42798765
ENSG00000226824	RP4-756H11.3	grey	0.09997966
ENSG00000224870	RP4-758J18.2	brown	-0.5938783
ENSG00000231081	RP4-760C5.3	grey	-3.78E-05
ENSG00000269892	RP4-761J14.10	grey	0.23730294
ENSG00000270050	RP4-769N13.7	grey	0.36466834
ENSG00000258634	RP4-773N10.4	turquoise	0.41725822
ENSG00000232712	RP4-777D9.2	grey	0.31642248
ENSG00000263683	RP4-777O23.1	grey	0.20119047
ENSG00000264520	RP4-777O23.2	grey	0.39522953
ENSG00000213361	RP4-778K6.1	grey	0.48013437
ENSG00000242154	RP4-778K6.3	grey	0.34123502
ENSG00000254539	RP4-791M13.3	grey	0.05663648
ENSG00000255148	RP4-791M13.4	grey	0.16343373
ENSG00000254913	RP4-791M13.5	grey	0.51017903
ENSG00000232453	RP4-794H19.1	yellow	0.43703569
ENSG00000235292	RP4-796I8.1	grey	0.35707052
ENSG00000231295	RP4-797C5.2	grey	0.27801165
ENSG00000261135	RP4-798A10.7	grey	0.20866076
ENSG00000236244	RP4-799P18.3	grey	0.11627515
ENSG00000231272	RP4-799P18.4	grey	0.28024803
ENSG00000232166	RP4-799P18.5	grey	0.38936027
ENSG00000228407	RP4-800M22.1	grey	0.41383285
ENSG00000223429	RP4-800M22.2	grey	0.27882208
ENSG00000270742	RP4-802A10.1	turquoise	0.79866494
ENSG00000237481	RP4-803J11.2	grey	0.33021024
ENSG00000226308	RP4-813D12.3	grey	0.01341045
ENSG00000236018	RP4-814D15.1	grey	0.31623637
ENSG00000225224	RP4-814D15.2	grey	0.44237848
ENSG00000258325	RP4-816N1.6	grey	0.33817079
ENSG00000248503	RP5-1000K24.2	grey	0.38972023
ENSG00000241054	RP5-1002M8.4	grey	0.19523876
ENSG00000231999	RP5-1007M22.2	darkred	-0.6852584
ENSG00000259065	RP5-1021I20.1	grey	0.14812915
ENSG00000258891	RP5-1021I20.5	grey	0.28350955
ENSG00000270140	RP5-1021I20.6	grey	0.19483356
ENSG00000233578	RP5-1022P6.3	grey	0.3294674
ENSG00000226754	RP5-1024G6.5	grey	0.02406425
ENSG00000179447	RP5-1027G4.3	grey	0.06623706
ENSG00000235526	RP5-1028L10.2	magenta	-0.355708
ENSG00000262905	RP5-1029F21.2	grey	0.1312882
ENSG00000224093	RP5-1033H22.2	yellow	0.53280688
ENSG00000222044	RP5-1039K5.16	yellow	0.4809256
ENSG00000229999	RP5-1042K10.10	grey	0.10582275
ENSG00000227413	RP5-1042K10.12	grey	0.39299677

ENSG00000229294	RP5-1044H5.1	yellow	0.68554624
ENSG00000218732	RP5-1046G13.2	grey	0.2968877
ENSG00000262678	RP5-1050D4.4	grey	0.16106993
ENSG00000261326	RP5-1057J7.6	grey	-0.1924445
ENSG00000236908	RP5-1063M23.2	turquoise	-0.4258305
ENSG00000251484	RP5-1065J22.4	greenyellow	-0.4818673
ENSG00000226920	RP5-1068B5.3	grey	0.4550469
ENSG00000241913	RP5-1073F15.1	grey	0.38503381
ENSG00000235527	RP5-1073O3.7	grey	0.46950221
ENSG00000260257	RP5-1085F17.3	grey	0.24332113
ENSG00000260536	RP5-1085F17.4	grey	0.34958066
ENSG00000270605	RP5-1092A3.4	grey	0.18146244
ENSG00000235036	RP5-1099D15.1	grey	0.20305971
ENSG00000227211	RP5-1100E15.4	grey	0.09628876
ENSG00000225377	RP5-1103G7.4	grey	0.15848708
ENSG00000227188	RP5-1104E15.6	grey	0.09533728
ENSG00000232022	RP5-1109J22.1	grey	0.03124748
ENSG00000230177	RP5-1112D6.4	brown	0.47480924
ENSG00000232912	RP5-1115A15.1	grey	0.14711567
ENSG00000270282	RP5-1115A15.2	grey	0.30035375
ENSG00000237686	RP5-1120P11.1	blue	0.3921558
ENSG00000228386	RP5-1125A11.4	grey	0.34131275
ENSG00000234185	RP5-1132H15.1	grey	0.44524789
ENSG00000230392	RP5-1139I1.1	grey	0.34278762
ENSG00000224629	RP5-1142J19.2	grey	0.45010095
ENSG00000256020	RP5-1154L15.2	yellow	0.50879636
ENSG00000231566	RP5-1158E12.3	grey	0.03797513
ENSG00000258930	RP5-1163L11.2	grey	0.13240677
ENSG00000258966	RP5-1163L11.3	grey	0.28340262
ENSG00000261778	RP5-1173P7.1	grey	0.34747121
ENSG00000229822	RP5-1174J21.2	grey	0.22785949
ENSG00000225417	RP5-1178H5.2	grey	0.16266218
ENSG00000236772	RP5-1184F4.5	grey	0.01274789
ENSG00000184068	RP5-821D11.7	grey	0.39342439
ENSG00000259723	RP5-823G15.5	grey	0.28435707
ENSG00000228692	RP5-826L7.1	grey	0.26742295
ENSG00000235605	RP5-827C21.1	black	0.48893771
ENSG00000236358	RP5-827C21.2	grey	0.0329869
ENSG00000231663	RP5-827C21.4	grey	0.06853204
ENSG00000237853	RP5-833A20.1	grey	0.30372964
ENSG00000236676	RP5-837I24.4	grey	0.14853371
ENSG00000224113	RP5-837J1.2	grey	0.04563246
ENSG00000204990	RP5-842K16.1	grey	0.37753622
ENSG00000261629	RP5-842K16.2	grey	0.50551472
ENSG00000213703	RP5-849H19.2	grey	0.34643578
ENSG00000225767	RP5-850O15.3	grey	0.21279762
ENSG00000231768	RP5-855F14.1	grey	0.164637
ENSG00000198744	RP5-857K21.11	grey	0.19287086

ENSG00000230021	RP5-857K21.4	grey	0.23959029
ENSG00000228438	RP5-859I17.2	grey	0.37135425
ENSG00000228436	RP5-864K19.4	grey	0.38529082
ENSG00000239291	RP5-867C24.1	grey	0.33155705
ENSG00000262265	RP5-867C24.4	grey	0.28936683
ENSG00000228604	RP5-872K7.7	grey	0.34109231
ENSG00000197062	RP5-874C20.3	brown	-0.5931305
ENSG00000270326	RP5-874C20.6	grey	0.45592309
ENSG00000250282	RP5-875H18.4	grey	0.35815114
ENSG00000260793	RP5-882C2.2	grey	-0.0580253
ENSG00000226499	RP5-882O7.1	grey	0.39451807
ENSG00000224592	RP5-884C9.2	grey	0.03748499
ENSG00000223589	RP5-884C9.3	grey	0.21828383
ENSG00000228084	RP5-884G6.2	grey	0.00677527
ENSG00000236810	RP5-886K2.3	grey	0.29224338
ENSG00000263412	RP5-890E16.2	grey	0.21443235
ENSG00000240731	RP5-890O3.9	grey	0.09358771
ENSG00000244701	RP5-894A10.2	purple	-0.6495752
ENSG00000270157	RP5-894A10.6	grey	0.21762197
ENSG00000255306	RP5-901A4.1	grey	0.05866966
ENSG00000230415	RP5-902P8.10	grey	0.190658
ENSG00000261624	RP5-914M6.1	grey	0.38661881
ENSG00000259456	RP5-914P20.5	brown	-0.3857819
ENSG00000237478	RP5-926E3.1	grey	0.16143281
ENSG00000255966	RP5-940J5.3	grey	0.20214483
ENSG00000247853	RP5-940J5.6	grey	0.18109656
ENSG00000249028	RP5-951N9.1	grey	0.28059868
ENSG00000227006	RP5-956O18.2	grey	0.26310647
ENSG00000231977	RP5-963E22.4	grey	0.09516871
ENSG00000271137	RP5-966M1.4	grey	0.29918208
ENSG00000243696	RP5-966M1.6	grey	0.06493575
ENSG00000230839	RP5-968J1.1	grey	0.03951606
ENSG00000237832	RP5-974N19.1	grey	0.02241149
ENSG00000229591	RP5-981O7.2	grey	0.28872439
ENSG00000259209	RP5-991G20.2	grey	0.47507354
ENSG00000259901	RP5-991G20.4	grey	0.18313132
ENSG00000234917	RP5-994D16.3	yellow	-0.3952918
ENSG00000228452	RP5-994D16.9	grey	0.11395169
ENSG00000230163	RP5-997D16.2	grey	0.30640052
ENSG00000231010	RP6-109B7.2	yellow	0.48137365
ENSG00000241990	RP6-109B7.3	grey	-0.1693129
ENSG00000235159	RP6-109B7.4	blue	-0.5157399
ENSG00000236064	RP6-191P20.4	grey	-0.1420044
ENSG00000260118	RP6-201G10.2	grey	0.07535902
ENSG00000226637	RP6-206I17.3	grey	0.29455745
ENSG00000232183	RP6-27P15.2	turquoise	-0.4006754
ENSG00000259153	RP6-65G23.3	yellow	-0.4814805
ENSG00000269945	RP6-91H8.5	grey	-0.0999459

ENSG00000270069	RP6-99M1.2	grey	-0.0354595
ENSG00000269902	RP6-99M1.3	grey	0.15952833
ENSG00000164610	RP9	grey	-0.0724673
ENSG00000205763	RP9P	grey	-0.0551569
ENSG00000132383	RPA1	salmon	0.61465638
ENSG00000258392	RPA2P1	grey	0.36911162
ENSG00000106399	RPA3	salmon	0.62473652
ENSG00000219545	RPA3-AS1	grey	0.24898762
ENSG00000129197	RPAIN	green	-0.7266721
ENSG00000103932	RPAP1	grey	0.15334855
ENSG00000005175	RPAP3	grey	-0.2640982
ENSG00000197713	RPE	brown	-0.6074648
ENSG00000117133	RPF1	brown	0.50811407
ENSG00000156313	RPGR	pink	0.51702505
ENSG00000103494	RPGRIP1L	blue	0.69125841
ENSG00000089169	RPH3A	purple	0.84106067
ENSG00000153574	RPIA	grey	-0.0592051
ENSG00000147403	RPL10	greenyellow	0.58841622
ENSG00000198755	RPL10A	greenyellow	0.75516853
ENSG00000226360	RPL10AP6	grey	0.12236717
ENSG00000230734	RPL10P3	grey	-0.0327267
ENSG00000142676	RPL11	greenyellow	0.66258196
ENSG00000197958	RPL12	greenyellow	0.63581432
ENSG00000232493	RPL12P11	grey	0.23294053
ENSG00000230993	RPL12P15	grey	0.39394374
ENSG00000233189	RPL12P29	grey	0.52049168
ENSG00000167526	RPL13	greenyellow	0.84723647
ENSG00000142541	RPL13A	greenyellow	0.91724463
ENSG00000231301	RPL13AP	grey	0.27782896
ENSG00000231322	RPL13AP17	grey	0.42952608
ENSG00000244053	RPL13AP2	grey	0.31844789
ENSG00000136149	RPL13AP25	greenyellow	0.59644292
ENSG00000236552	RPL13AP5	greenyellow	0.66061332
ENSG00000234118	RPL13AP6	grey	0.28380057
ENSG00000215030	RPL13P12	grey	-0.0296141
ENSG00000240370	RPL13P5	grey	0.3550764
ENSG00000213176	RPL13P6	grey	0.42390139
ENSG00000188846	RPL14	greenyellow	0.78978331
ENSG00000139239	RPL14P1	grey	0.21496948
ENSG00000174748	RPL15	greenyellow	0.59919386
ENSG00000212802	RPL15P3	grey	0.37975114
ENSG00000265681	RPL17	grey	0.08717337
ENSG00000241261	RPL17P19	grey	0.20569534
ENSG00000228331	RPL17P43	turquoise	-0.4037362
ENSG00000213700	RPL17P50	grey	0.33312657
ENSG00000063177	RPL18	greenyellow	0.78310826
ENSG00000105640	RPL18A	greenyellow	0.7490436
ENSG00000225043	RPL18AP2	grey	0.28093972

ENSG00000213442	RPL18AP3	greenyellow	0.56920471
ENSG00000232439	RPL18AP7	brown	-0.5303836
ENSG00000214359	RPL18P10	blue	-0.4432051
ENSG00000108298	RPL19	greenyellow	0.7256968
ENSG00000224415	RPL19P12	grey	0.34190938
ENSG00000122026	RPL21	greenyellow	0.82251601
ENSG00000244582	RPL21P120	grey	0.24550239
ENSG00000240531	RPL21P123	grey	-0.0430862
ENSG00000235482	RPL21P135	grey	0.32383886
ENSG00000220749	RPL21P28	brown	0.27861571
ENSG00000224019	RPL21P32	grey	0.17656679
ENSG00000231688	RPL21P43	grey	0.27983624
ENSG00000229585	RPL21P44	grey	0.27502563
ENSG00000241829	RPL21P54	grey	-0.0306399
ENSG00000213860	RPL21P75	brown	0.35964851
ENSG00000180662	RPL21P8	blue	-0.4208104
ENSG00000116251	RPL22	greenyellow	0.84527406
ENSG00000163584	RPL22L1	grey	0.05380239
ENSG00000125691	RPL23	brown	0.62321256
ENSG00000198242	RPL23A	greenyellow	0.86495946
ENSG00000237887	RPL23AP32	grey	-0.0421932
ENSG00000232568	RPL23AP35	grey	0.28962034
ENSG00000223508	RPL23AP53	grey	0.4608663
ENSG00000240970	RPL23AP64	grey	0.34078474
ENSG00000240356	RPL23AP7	grey	0.06273732
ENSG00000242748	RPL23AP81	grey	0.40907963
ENSG00000184319	RPL23AP82	grey	-0.1293244
ENSG00000176054	RPL23P2	grey	0.13327194
ENSG00000114391	RPL24	brown	0.68302699
ENSG00000161970	RPL26	greenyellow	0.83901431
ENSG00000037241	RPL26L1	grey	-0.1106619
ENSG00000244229	RPL26P35	grey	0.23870639
ENSG00000131469	RPL27	brown	0.6632871
ENSG00000166441	RPL27A	brown	0.65739564
ENSG00000108107	RPL28	greenyellow	0.60820576
ENSG00000162244	RPL29	greenyellow	0.63223265
ENSG00000241112	RPL29P14	grey	0.43838327
ENSG00000100316	RPL3	grey	-0.4647352
ENSG00000156482	RPL30	greenyellow	0.7614874
ENSG00000229014	RPL30P13	grey	0.39270442
ENSG00000243256	RPL30P14	grey	0.20562708
ENSG00000071082	RPL31	greenyellow	0.78395505
ENSG00000227207	RPL31P12	grey	0.11218112
ENSG00000232430	RPL31P15	grey	0.23009759
ENSG00000230837	RPL31P2	grey	0.41131209
ENSG00000229756	RPL31P20	grey	0.13084551
ENSG00000219355	RPL31P52	grey	0.45460112
ENSG00000229865	RPL31P54	grey	0.12119597

ENSG00000243297	RPL31P61	grey	0.22019269
ENSG00000144713	RPL32	greenyellow	0.85466112
ENSG00000241941	RPL32P26	grey	0.54496551
ENSG00000251474	RPL32P3	red	-0.5620148
ENSG00000109475	RPL34	brown	0.71378965
ENSG00000240534	RPL34P17	yellow	0.57192586
ENSG00000231333	RPL34P6	grey	0.34906228
ENSG00000136942	RPL35	greenyellow	0.82949708
ENSG00000182899	RPL35A	greenyellow	0.91455825
ENSG00000233460	RPL35AP31	black	0.54132759
ENSG00000220583	RPL35P2	grey	0.42402896
ENSG00000219902	RPL35P3	brown	-0.392136
ENSG00000244018	RPL35P6	grey	0.07464966
ENSG00000130255	RPL36	greenyellow	0.69067709
ENSG00000241343	RPL36A	greenyellow	0.73001627
ENSG00000165502	RPL36AL	cyan	0.45101394
ENSG00000235369	RPL36AP15	grey	0.32517868
ENSG00000235619	RPL36AP33	grey	0.26293015
ENSG00000242087	RPL36AP41	grey	0.14585893
ENSG00000213180	RPL36AP48	grey	0.41987605
ENSG00000224664	RPL36AP53	grey	0.34884346
ENSG00000235656	RPL36P18	grey	0.45002993
ENSG00000232540	RPL36P19	grey	0.33477242
ENSG00000145592	RPL37	grey	-0.3107862
ENSG00000197756	RPL37A	brown	0.65611311
ENSG00000239559	RPL37P2	grey	0.07239235
ENSG00000172809	RPL38	greenyellow	0.87171419
ENSG00000198918	RPL39	brown	0.60963663
ENSG00000163923	RPL39L	lightcyan	-0.6094463
ENSG00000236860	RPL39P29	grey	0.29997944
ENSG00000226580	RPL39P40	grey	0.08591233
ENSG00000232573	RPL3P4	lightcyan	-0.5168231
ENSG00000231546	RPL3P5	grey	0.04306846
ENSG00000213891	RPL3P6	grey	0.14312218
ENSG00000174444	RPL4	greenyellow	0.68192658
ENSG00000229117	RPL41	greenyellow	0.78511484
ENSG00000256338	RPL41P2	grey	0.15298762
ENSG00000229638	RPL4P4	grey	0.23288168
ENSG00000230071	RPL4P6	grey	0.28339736
ENSG00000122406	RPL5	salmon	0.47247541
ENSG00000241061	RPL5P1	grey	0.20681596
ENSG00000242882	RPL5P11	grey	0.29097185
ENSG00000243914	RPL5P14	turquoise	-0.3605352
ENSG00000220744	RPL5P18	grey	-0.0451368
ENSG00000240395	RPL5P23	grey	0.42264534
ENSG00000244052	RPL5P24	grey	0.16610132
ENSG00000225251	RPL5P25	grey	0.2964089
ENSG00000228195	RPL5P27	grey	0.28905411

ENSG00000242276	RPL5P3	grey	0.41168401
ENSG00000243431	RPL5P30	grey	0.20869046
ENSG00000229994	RPL5P4	grey	0.0027311
ENSG00000089009	RPL6	greenyellow	0.80557207
ENSG00000227056	RPL6P2	grey	0.25912611
ENSG00000235552	RPL6P27	greenyellow	0.60133836
ENSG00000147604	RPL7	greenyellow	0.80745398
ENSG00000148303	RPL7A	greenyellow	0.71315997
ENSG00000240522	RPL7AP10	grey	0.22447925
ENSG00000242071	RPL7AP6	grey	0.15508388
ENSG00000213152	RPL7AP60	grey	0.216066
ENSG00000231981	RPL7L1P12	grey	0.27986857
ENSG00000231490	RPL7L1P2	grey	0.32292797
ENSG00000235090	RPL7L1P3	grey	0.08758383
ENSG00000214485	RPL7P1	grey	0.16550028
ENSG00000234619	RPL7P11	grey	0.37368633
ENSG00000232994	RPL7P14	grey	0.37951725
ENSG00000240622	RPL7P15	grey	0.45585201
ENSG00000214062	RPL7P17	grey	0.08301409
ENSG00000243806	RPL7P18	turquoise	-0.5367166
ENSG00000216636	RPL7P25	grey	0.37840167
ENSG00000184612	RPL7P26	grey	0.16203437
ENSG00000214457	RPL7P29	grey	0.37294296
ENSG00000231006	RPL7P32	grey	0.43321041
ENSG00000243020	RPL7P39	grey	0.29827834
ENSG00000229917	RPL7P46	grey	0.21359444
ENSG00000242931	RPL7P49	grey	0.23693758
ENSG00000228599	RPL7P52	brown	-0.5566616
ENSG00000224401	RPL7P57	grey	0.19463926
ENSG00000224315	RPL7P7	grey	0.28571144
ENSG00000137970	RPL7P9	grey	0.12619978
ENSG00000161016	RPL8	greenyellow	0.69293967
ENSG00000163682	RPL9	greenyellow	0.71116719
ENSG00000267355	RPL9P29	grey	0.26504853
ENSG00000240074	RPL9P30	grey	0.11517152
ENSG00000242100	RPL9P32	grey	0.35355263
ENSG00000238103	RPL9P7	grey	0.10688073
ENSG00000089157	RPLP0	greenyellow	0.77001649
ENSG00000137818	RPLP1	greenyellow	0.85410959
ENSG00000177600	RPLP2	greenyellow	0.642508
ENSG00000118705	RPN2	grey	0.02592299
ENSG00000241370	RPP21	brown	0.60444081
ENSG00000178718	RPP25	blue	0.31147661
ENSG00000164967	RPP25L	turquoise	0.36036562
ENSG00000152464	RPP38	grey	0.14089383
ENSG00000124787	RPP40	grey	0.17212634
ENSG00000259001	RPPH1	turquoise	-0.4960742
ENSG00000141425	RPRD1A	red	0.71358324

ENSG00000101413	RPRD1B	blue	-0.4924381
ENSG00000163125	RPRD2	darkorange	0.53892415
ENSG00000179673	RPRML	magenta	0.53437846
ENSG00000124614	RPS10	greenyellow	0.55422835
ENSG00000213950	RPS10P2	grey	0.32459053
ENSG00000223396	RPS10P7	grey	0.38001814
ENSG00000142534	RPS11	lightcyan	0.57550668
ENSG00000232888	RPS11P5	grey	0.24432561
ENSG00000243024	RPS11P6	blue	-0.3632161
ENSG00000112306	RPS12	greenyellow	0.77206598
ENSG00000225193	RPS12P26	grey	0.25935923
ENSG00000240435	RPS12P27	grey	0.1576804
ENSG00000226216	RPS12P5	grey	0.43909311
ENSG00000110700	RPS13	greenyellow	0.62764341
ENSG00000164587	RPS14	greenyellow	0.86994551
ENSG00000226396	RPS14P3	grey	0.37485674
ENSG00000115268	RPS15	greenyellow	0.54718938
ENSG00000134419	RPS15A	greenyellow	0.76826685
ENSG00000214535	RPS15AP1	grey	0.37808247
ENSG00000225447	RPS15AP10	grey	0.06036823
ENSG00000239483	RPS15AP16	grey	0.49897353
ENSG00000242979	RPS15AP18	grey	0.35028928
ENSG00000214903	RPS15AP3	grey	0.25675288
ENSG00000241808	RPS15AP34	grey	0.36775979
ENSG00000233072	RPS15AP6	grey	0.46044232
ENSG00000229806	RPS15P5	grey	0.44600053
ENSG00000105193	RPS16	greenyellow	0.69143142
ENSG00000224706	RPS17P13	grey	0.25504246
ENSG00000231500	RPS18	greenyellow	0.55477622
ENSG00000220848	RPS18P9	grey	0.35437588
ENSG00000105372	RPS19	greenyellow	0.78584367
ENSG00000140988	RPS2	greenyellow	0.58605737
ENSG00000008988	RPS20	greenyellow	0.84741549
ENSG00000227523	RPS20P15	grey	0.48925446
ENSG00000242085	RPS20P33	grey	0.11020112
ENSG00000241907	RPS20P4	grey	0.37126794
ENSG00000171858	RPS21	greenyellow	0.66373036
ENSG00000186468	RPS23	greenyellow	0.48880547
ENSG00000251374	RPS23P5	grey	0.38752159
ENSG00000230629	RPS23P8	grey	0.01417319
ENSG00000138326	RPS24	brown	0.68312129
ENSG00000118181	RPS25	greenyellow	0.68929678
ENSG00000197728	RPS26	brown	0.48921967
ENSG00000242829	RPS26P21	grey	0.19581963
ENSG00000224602	RPS26P5	grey	0.34581485
ENSG00000226339	RPS26P56	grey	0.28767421
ENSG00000177954	RPS27	greenyellow	0.6135108
ENSG00000143947	RPS27A	greenyellow	0.86708964

ENSG00000185088	RPS27L	salmon	0.61633137
ENSG00000225907	RPS27P16	grey	0.3640532
ENSG00000233927	RPS28	greenyellow	0.5248414
ENSG00000213741	RPS29	brown	0.50160962
ENSG00000240873	RPS29P22	grey	0.17100634
ENSG00000232818	RPS2P32	grey	0.02119384
ENSG00000244060	RPS2P41	grey	0.36759919
ENSG00000243609	RPS2P44	grey	0.20575578
ENSG00000189343	RPS2P46	grey	0.15786215
ENSG00000240342	RPS2P5	grey	0.07088141
ENSG00000149273	RPS3	greenyellow	0.84984111
ENSG00000145425	RPS3A	greenyellow	0.80208777
ENSG00000242229	RPS3AP14	grey	0.21965024
ENSG00000240083	RPS3AP22	grey	0.21131425
ENSG00000214389	RPS3AP26	salmon	0.59621898
ENSG00000237818	RPS3AP29	grey	0.16950611
ENSG00000242607	RPS3AP34	yellow	-0.5248655
ENSG00000244244	RPS3AP42	grey	0.25909869
ENSG00000242661	RPS3AP43	grey	0.27414562
ENSG00000205871	RPS3AP47	greenyellow	0.5113843
ENSG00000234797	RPS3AP6	salmon	0.64936691
ENSG00000230935	RPS3P1	grey	0.44593425
ENSG00000231307	RPS3P2	grey	0.09330677
ENSG00000198034	RPS4X	greenyellow	0.81491331
ENSG00000243663	RPS4XP14	yellow	0.52330782
ENSG00000244097	RPS4XP17	grey	0.30087179
ENSG00000244073	RPS4XP6	grey	0.16943743
ENSG00000129824	RPS4Y1	grey	-0.1792613
ENSG00000083845	RPS5	greenyellow	0.48779095
ENSG00000137154	RPS6	greenyellow	0.63552633
ENSG00000117676	RPS6KA1	grey	-0.0895403
ENSG00000071242	RPS6KA2	red	-0.5346965
ENSG00000232082	RPS6KA2-IT1	grey	0.01250049
ENSG00000177189	RPS6KA3	grey	-0.1290654
ENSG00000162302	RPS6KA4	blue	0.38887628
ENSG00000100784	RPS6KA5	magenta	0.64899936
ENSG00000072133	RPS6KA6	blue	0.4179257
ENSG00000108443	RPS6KB1	green	0.62962453
ENSG00000175634	RPS6KB2	grey	0.15077435
ENSG00000136643	RPS6KC1	grey	0.25084314
ENSG00000198208	RPS6KL1	turquoise	-0.4794603
ENSG00000171863	RPS7	greenyellow	0.75585476
ENSG00000183405	RPS7P1	grey	0.04805864
ENSG00000213326	RPS7P11	grey	0.18524727
ENSG00000240167	RPS7P7	grey	0.12961749
ENSG00000142937	RPS8	greenyellow	0.77174756
ENSG00000231660	RPS8P6	grey	0.28409887
ENSG00000170889	RPS9	greenyellow	0.80897268

ENSG00000168028	RPSA	brown	0.58203818
ENSG00000233984	RPSAP14	grey	0.23833135
ENSG00000216471	RPSAP43	yellow	0.40060708
ENSG00000205246	RPSAP58	turquoise	-0.5434248
ENSG00000214629	RPSAP6	cyan	-0.4543986
ENSG00000141564	RPTOR	grey	0.35511652
ENSG00000007376	RPUSD1	grey	0.1788728
ENSG00000166133	RPUSD2	grey	-0.0190836
ENSG00000156990	RPUSD3	blue	0.67544632
ENSG00000144580	RQCD1	brown	0.56030807
ENSG00000155876	RRAGA	darkred	0.62270287
ENSG00000083750	RRAGB	black	-0.5594487
ENSG00000116954	RRAGC	grey	0.32416673
ENSG00000025039	RRAGD	grey	-0.1828896
ENSG00000126458	RRAS	blue	-0.6876929
ENSG00000133818	RRAS2	grey	0.11307545
ENSG00000125844	RRBP1	yellow	0.7388791
ENSG00000124782	RREB1	turquoise	0.45618431
ENSG00000167325	RRM1	grey	-0.0838838
ENSG00000048392	RRM2B	grey	0.05089451
ENSG00000085721	RRN3	grey	0.06972072
ENSG00000248124	RRN3P1	brown	0.47643119
ENSG00000103472	RRN3P2	grey	0.15576435
ENSG00000257122	RRN3P3	turquoise	-0.6263043
ENSG00000143303	RRNAD1	grey	0.21961833
ENSG00000160214	RRP1	black	0.37861814
ENSG00000052749	RRP12	turquoise	-0.6319855
ENSG00000067533	RRP15	grey	0.03620007
ENSG00000160208	RRP1B	turquoise	-0.4766179
ENSG00000189306	RRP7A	grey	-0.0307536
ENSG00000182841	RRP7B	red	-0.6043465
ENSG00000132275	RRP8	green	0.72311178
ENSG00000114767	RRP9	grey	0.1161627
ENSG00000102104	RS1	turquoise	-0.5739952
ENSG00000136444	RSAD1	black	0.58182107
ENSG00000134321	RSAD2	grey	-0.0503425
ENSG00000081019	RSBN1	grey	0.0372486
ENSG00000187257	RSBN1L	brown	-0.4587367
ENSG00000214293	RSBN1L-AS1	grey	0.07987798
ENSG00000048649	RSF1	grey	-0.0688552
ENSG00000254985	RSF1-IT2	grey	0.28844383
ENSG00000132881	RSG1	grey	0.13821569
ENSG00000171490	RSL1D1	grey	-0.1375751
ENSG00000137876	RSL24D1	salmon	0.56525768
ENSG00000237273	RSL24D1P8	grey	0.07567766
ENSG00000160188	RSPH1	brown	0.61092331
ENSG00000130363	RSPH3	blue	0.47440747
ENSG00000101282	RSPO4	grey	0.01935646

ENSG00000159579	RSPRY1	grey	0.13962957
ENSG00000174891	RSRC1	grey60	0.44877945
ENSG00000111011	RSRC2	grey	0.07922649
ENSG00000148484	RSU1	red	0.69503212
ENSG00000213659	RSU1P3	grey	0.14983177
ENSG00000137996	RTCA	turquoise	-0.4499264
ENSG00000100218	RTDR1	blue	0.40445041
ENSG00000137815	RTF1	blue	0.65985307
ENSG00000114993	RTKN	yellow	0.81684189
ENSG00000139970	RTN1	blue	0.78020341
ENSG00000125744	RTN2	blue	0.4997665
ENSG00000133318	RTN3	blue	0.82758873
ENSG00000115310	RTN4	lightgreen	0.63880496
ENSG00000130347	RTN4IP1	brown	0.48940407
ENSG00000040608	RTN4R	grey	0.14932612
ENSG00000136514	RTP4	grey	0.31216488
ENSG00000176225	RTTN	turquoise	-0.4611678
ENSG00000176783	RUFY1	grey	0.14048492
ENSG00000204130	RUFY2	darkred	-0.5370372
ENSG00000018189	RUFY3	turquoise	-0.5228051
ENSG00000198863	RUNDC1	brown	0.46030356
ENSG00000108309	RUNDC3A	blue	0.83017669
ENSG00000105784	RUNDC3B	turquoise	-0.5040531
ENSG00000159216	RUNX1	royalblue	0.75741101
ENSG00000124813	RUNX2	cyan	0.600377
ENSG00000160753	RUSC1	yellow	-0.7067041
ENSG00000225855	RUSC1-AS1	grey	0.15684752
ENSG00000198853	RUSC2	blue	0.82183215
ENSG00000175792	RUVBL1	green	-0.687192
ENSG00000183207	RUVBL2	blue	0.69766988
ENSG00000111832	RWDD1	green	0.50126004
ENSG00000013392	RWDD2A	brown	0.3270893
ENSG00000156253	RWDD2B	pink	-0.5573275
ENSG00000122481	RWDD3	grey	0.0274588
ENSG00000182552	RWDD4	mediumpurp	0.61365601
ENSG00000186350	RXRA	blue	-0.6215323
ENSG00000204231	RXRB	grey	-0.0849867
ENSG00000143171	RXRG	magenta	0.69083539
ENSG00000163602	RYBP	darkred	-0.6591327
ENSG00000163785	RYK	brown	-0.7352507
ENSG00000198626	RYR2	magenta	0.86512124
ENSG00000198838	RYR3	black	0.83033974
ENSG00000160678	S100A1	turquoise	0.73042233
ENSG00000197747	S100A10	grey	-0.2524743
ENSG00000163191	S100A11	royalblue	0.83106195
ENSG00000189171	S100A13	turquoise	0.33255341
ENSG00000188643	S100A16	turquoise	0.68298668
ENSG00000197956	S100A6	grey	-0.2245187

ENSG00000160307	S100B	turquoise	0.54277302
ENSG00000116497	S100PBP	red	-0.6548037
ENSG00000170989	S1PR1	turquoise	0.90749501
ENSG00000213694	S1PR3	pink	0.61342135
ENSG00000180739	S1PR5	yellow	0.83115925
ENSG00000166788	SAAL1	grey	0.06915654
ENSG00000168061	SAC3D1	brown	0.51919583
ENSG00000151835	SACS	grey60	0.72106726
ENSG00000142230	SAE1	brown	0.57867039
ENSG00000160633	SAFB	turquoise	-0.5876811
ENSG00000130254	SAFB2	darkgrey	0.61552799
ENSG00000103449	SALL1	turquoise	0.32128385
ENSG00000165821	SALL2	yellowgreen	0.82362076
ENSG00000256463	SALL3	red	0.60409599
ENSG00000141858	SAMD1	brown	-0.7455922
ENSG00000130590	SAMD10	grey	-0.1422729
ENSG00000177570	SAMD12	turquoise	-0.7018147
ENSG00000167100	SAMD14	grey	0.0013467
ENSG00000100583	SAMD15	blue	0.32476273
ENSG00000020577	SAMD4A	turquoise	0.47240603
ENSG00000179134	SAMD4B	brown	-0.5826825
ENSG00000203727	SAMD5	grey60	0.60965052
ENSG00000156671	SAMD8	turquoise	0.78122587
ENSG00000205413	SAMD9	grey	-0.008968
ENSG00000177409	SAMD9L	lightcyan	0.45589565
ENSG00000101347	SAMHD1	royalblue	0.69440029
ENSG00000100347	SAMM50	brown	0.61919601
ENSG00000155307	SAMSN1	royalblue	0.70363891
ENSG00000136715	SAP130	lightcyan	0.74507096
ENSG00000150459	SAP18	darkgrey	0.69858916
ENSG00000164105	SAP30	turquoise	0.5205739
ENSG00000161526	SAP30BP	green	-0.5801196
ENSG00000164576	SAP30L	grey	0.255106
ENSG00000079332	SAR1A	brown	-0.5754845
ENSG00000152700	SAR1B	turquoise	0.74112562
ENSG00000004139	SARM1	grey	0.16346844
ENSG00000205323	SARNP	grey	0.32405475
ENSG00000031698	SARS	brown	0.66658729
ENSG00000104835	SARS2	grey	0.04227028
ENSG00000175467	SART1	grey	-0.1291422
ENSG00000075856	SART3	salmon	0.62671483
ENSG00000111961	SASH1	turquoise	0.68693252
ENSG00000122122	SASH3	royalblue	0.84859414
ENSG00000156876	SASS6	brown	-0.4333411
ENSG00000141504	SAT2	brown	0.67638424
ENSG00000182568	SATB1	purple	0.6930338
ENSG00000184788	SATL1	grey	0.09874462
ENSG00000151748	SAV1	lightgreen	0.69044727

ENSG00000112167	SAYSD1	grey	0.11056206
ENSG00000126524	SBDS	grey	-0.0831629
ENSG00000225648	SBDSP1	brown	-0.7320834
ENSG00000100241	SBF1	grey	-0.164998
ENSG00000133812	SBF2	grey	0.12555356
ENSG00000246273	SBF2-AS1	turquoise	-0.4213858
ENSG00000188322	SBK1	yellow	-0.54789
ENSG00000139697	SBNO1	grey	0.19387077
ENSG00000064932	SBNO2	pink	0.7587349
ENSG00000269490	SBP1	grey	0.27211092
ENSG00000164764	SBSPON	grey	0.06080314
ENSG00000230741	SC22CB-1D7.1	grey	0.35432449
ENSG00000109929	SC5D	turquoise	0.40812824
ENSG00000126461	SCAF1	grey	-0.1071678
ENSG00000139218	SCAF11	blue	-0.6530101
ENSG00000156304	SCAF4	grey	-0.0862278
ENSG00000213079	SCAF8	brown	-0.3929207
ENSG00000173611	SCAI	blue	0.65448685
ENSG00000140497	SCAMP2	turquoise	0.66867477
ENSG00000116521	SCAMP3	grey	0.14367405
ENSG00000198794	SCAMP5	blue	0.77724343
ENSG00000171222	SCAND1	brown	0.80016063
ENSG00000176700	SCAND2P	grey	0.09554207
ENSG00000232040	SCAND3	blue	0.45430006
ENSG00000232795	SCAND3P1	grey	0.12141144
ENSG00000114650	SCAP	grey	0.12934885
ENSG00000140386	SCAPER	grey	0.0035006
ENSG00000168077	SCARA3	turquoise	0.52716179
ENSG00000073060	SCARB1	black	0.89544796
ENSG00000138760	SCARB2	turquoise	0.39659803
ENSG00000074660	SCARF1	tan	0.66695798
ENSG00000252947	SCARNA1	grey	0.27522195
ENSG00000239002	SCARNA10	turquoise	-0.3128905
ENSG00000252481	SCARNA13	grey	0.18281854
ENSG00000252218	SCARNA15	grey	0.38747727
ENSG00000251992	SCARNA17	grey	0.16902609
ENSG00000252139	SCARNA18	grey	0.26433071
ENSG00000252577	SCARNA20	grey	0.45096791
ENSG00000252778	SCARNA20	grey	0.40473272
ENSG00000252906	SCARNA3	grey	0.37057173
ENSG00000252010	SCARNA5	black	0.47412625
ENSG00000238741	SCARNA7	greenyellow	-0.4235861
ENSG00000254911	SCARNA9	brown	-0.5014393
ENSG00000143653	SCCPDH	grey	-0.1718702
ENSG00000099194	SCD	green	0.68111537
ENSG00000145284	SCD5	turquoise	0.64931342
ENSG00000092108	SCFD1	blue	0.54987377
ENSG00000184178	SCFD2	grey	0.17054749

ENSG00000171951	SCG2	blue	0.80730596
ENSG00000104112	SCG3	turquoise	0.37063405
ENSG00000166922	SCG5	blue	0.87650955
ENSG00000268751	SCGB1B2P	grey	0.10949926
ENSG00000205209	SCGB2B2	grey	0.20260579
ENSG00000164265	SCGB3A2	grey	0.0070849
ENSG00000151967	SCHIP1	yellow	0.7284453
ENSG00000006747	SCIN	turquoise	-0.472108
ENSG00000132330	SCLY	grey	0.26961311
ENSG00000010803	SCMH1	grey	0.21979731
ENSG00000047634	SCML1	magenta	-0.4928141
ENSG00000102098	SCML2	grey	0.42852162
ENSG00000271105	SCML2P2	grey	0.22298765
ENSG00000144285	SCN1A	blue	0.61104185
ENSG00000136531	SCN2A	blue	0.74005416
ENSG00000149575	SCN2B	turquoise	-0.7379686
ENSG00000153253	SCN3A	blue	0.71297442
ENSG00000166257	SCN3B	blue	0.72814945
ENSG00000177098	SCN4B	magenta	0.74157343
ENSG00000136546	SCN7A	yellow	-0.5460608
ENSG00000196876	SCN8A	blue	0.74930859
ENSG00000169432	SCN9A	purple	0.85100397
ENSG00000163156	SCNM1	brown	0.4202859
ENSG00000162572	SCNN1D	turquoise	-0.4812878
ENSG00000133028	SCO1	darkred	0.39669462
ENSG00000153130	SCOC	grey	-0.270141
ENSG00000116171	SCP2	turquoise	0.76718689
ENSG00000121064	SCPEP1	grey	-0.4223064
ENSG00000164106	SCRG1	turquoise	0.57839351
ENSG00000180900	SCRIB	grey	0.00631561
ENSG00000136193	SCRN1	turquoise	0.73112458
ENSG00000141295	SCRN2	grey	-0.0722251
ENSG00000144306	SCRN3	grey	0.02691897
ENSG00000170616	SCRT1	grey	0.11619506
ENSG00000175356	SCUBE2	grey	0.0006445
ENSG00000146197	SCUBE3	grey	-0.0398393
ENSG00000142186	SCYL1	brown	0.64270268
ENSG00000136021	SCYL2	grey	-0.1890865
ENSG00000000457	SCYL3	grey	0.03861395
ENSG00000198301	SDAD1	turquoise	-0.4452488
ENSG00000228451	SDAD1P1	grey	0.14063137
ENSG00000169439	SDC2	turquoise	0.54482013
ENSG00000124145	SDC4	turquoise	0.75142032
ENSG00000137575	SDCBP	grey	-0.1842596
ENSG00000125775	SDCBP2	grey	0.05092229
ENSG00000234684	SDCBP2-AS1	grey	0.16859162
ENSG00000226705	SDCBPP1	grey	0.19905929
ENSG00000165689	SDCCAG3	grey	0.12298986

ENSG00000054282	SDCCAG8	darkgrey	0.52159709
ENSG00000143751	SDE2	turquoise	0.53969178
ENSG00000078808	SDF4	grey	0.2230963
ENSG00000073578	SDHA	green	-0.6039611
ENSG00000205138	SDHAF1	brown	0.45672664
ENSG00000167985	SDHAF2	turquoise	-0.2261326
ENSG00000185485	SDHAP1	grey	-0.0470063
ENSG00000185986	SDHAP3	grey	0.14713169
ENSG00000117118	SDHB	brown	0.6773318
ENSG00000214549	SDHCP2	grey	0.37678068
ENSG00000255035	SDHCP4	grey	0.31376563
ENSG00000204370	SDHD	turquoise	0.82430098
ENSG00000069188	SDK2	grey	0.0925382
ENSG00000168497	SDPR	tan	0.80448692
ENSG00000100445	SDR39U1	grey	0.17608351
ENSG00000135094	SDS	turquoise	0.59293778
ENSG00000139410	SDSL	turquoise	0.36254251
ENSG00000140612	SEC11A	blue	-0.3615102
ENSG00000166562	SEC11C	grey	-0.2746989
ENSG00000157020	SEC13	salmon	0.8508878
ENSG00000129657	SEC14L1	blue	-0.6313304
ENSG00000213693	SEC14L1P1	grey	0.10195103
ENSG00000100003	SEC14L2	turquoise	0.46415449
ENSG00000103184	SEC14L5	yellow	0.68081153
ENSG00000214491	SEC14L6	grey	0.2263299
ENSG00000148396	SEC16A	brown	0.46051797
ENSG00000121542	SEC22A	grey	0.16230913
ENSG00000223380	SEC22B	brown	-0.7925189
ENSG00000093183	SEC22C	turquoise	0.56020384
ENSG00000100934	SEC23A	grey	-0.0585066
ENSG00000101310	SEC23B	grey	0.18068654
ENSG00000107651	SEC23IP	turquoise	-0.4024575
ENSG00000113615	SEC24A	grey	0.28924783
ENSG00000138802	SEC24B	grey	0.18752713
ENSG00000247950	SEC24B-AS1	grey	-0.0360796
ENSG00000176986	SEC24C	turquoise	-0.3035654
ENSG00000150961	SEC24D	pink	0.62086799
ENSG00000138674	SEC31A	lightgreen	-0.7272579
ENSG00000058262	SEC61A1	grey	0.34034786
ENSG00000065665	SEC61A2	blue	0.54064795
ENSG00000106803	SEC61B	grey	-0.0937416
ENSG00000132432	SEC61G	greenyellow	0.51270304
ENSG00000008952	SEC62	darkgrey	0.6179937
ENSG00000240373	SEC62-AS1	grey	0.44613353
ENSG00000025796	SEC63	darkgrey	0.44027628
ENSG00000187742	SECISBP2	lightcyan	-0.6929006
ENSG00000138593	SECISBP2L	yellow	0.76603463
ENSG00000085415	SEH1L	grey	-0.0433805

ENSG00000071537	SEL1L	grey	-0.1110476
ENSG00000091490	SEL1L3	turquoise	0.36772762
ENSG00000143416	SELENBP1	turquoise	0.76094733
ENSG00000113811	SELK	grey	0.08861391
ENSG00000188404	SELL	pink	0.56384288
ENSG00000198832	SELM	grey	-0.101245
ENSG00000110876	SELPLG	cyan	0.84402036
ENSG00000198843	SELT	turquoise	0.44644957
ENSG00000075213	SEMA3A	purple	0.64151212
ENSG00000012171	SEMA3B	yellow	0.83175266
ENSG00000232352	SEMA3B-AS1	yellow	0.42868267
ENSG00000075223	SEMA3C	grey	0.00851288
ENSG00000170381	SEMA3E	grey	-0.0462229
ENSG00000010319	SEMA3G	tan	0.57689764
ENSG00000196189	SEMA4A	yellowgreen	0.72899335
ENSG00000185033	SEMA4B	turquoise	0.59418312
ENSG00000168758	SEMA4C	yellow	0.78956211
ENSG00000187764	SEMA4D	yellow	0.80030201
ENSG00000135622	SEMA4F	blue	0.25438938
ENSG00000092421	SEMA6A	yellow	0.60459186
ENSG00000167680	SEMA6B	darkorange	0.7514482
ENSG00000137872	SEMA6D	yellow	0.56417557
ENSG00000138623	SEMA7A	black	0.77923735
ENSG00000254703	SENCR	tan	0.63020713
ENSG00000163904	SENP2	turquoise	-0.3780455
ENSG00000161956	SENP3	grey	0.07197026
ENSG00000119231	SENP5	mediumpurp	-0.5676462
ENSG00000112701	SENP6	brown	-0.5068612
ENSG00000138468	SENP7	lightgreen	0.5936517
ENSG00000166192	SENP8	grey	0.32147433
ENSG00000086475	SEPHS1	turquoise	0.66274285
ENSG00000182722	SEPHS1P1	grey	0.21392534
ENSG00000213938	SEPHS1P6	grey	0.04405132
ENSG00000179918	SEPHS2	grey	0.26372926
ENSG00000250722	SEPP1	yellow	0.77330198
ENSG00000109618	SEPSECS	turquoise	0.46474379
ENSG00000214765	SEPT7P2	grey	0.14708349
ENSG00000178980	SEPW1	brown	0.44781154
ENSG00000122335	SERAC1	brown	0.36282222
ENSG00000142864	SERBP1	turquoise	0.44008719
ENSG00000242142	SERBP1P3	grey	0.17396343
ENSG00000249565	SERBP1P5	grey	0.39485095
ENSG00000140264	SERF2	grey	0.00807871
ENSG00000129158	SERGEF	turquoise	-0.6649747
ENSG00000172250	SERHL	grey	0.22226741
ENSG00000183569	SERHL2	grey	0.11066486
ENSG00000111897	SERINC1	grey	0.16011042
ENSG00000132824	SERINC3	brown	-0.604911

ENSG00000164300	SERINC5	yellow	0.68733603
ENSG00000120742	SERP1	turquoise	0.64894083
ENSG00000151778	SERP2	brown	0.56467379
ENSG00000196136	SERPINA3	pink	0.80081862
ENSG00000124570	SERPINB6	blue	-0.6075952
ENSG00000166401	SERPINB8	pink	0.56254466
ENSG00000170542	SERPINB9	pink	0.58901348
ENSG00000099937	SERPIND1	grey	-0.0292727
ENSG00000135919	SERPINE2	turquoise	0.72931787
ENSG00000253309	SERPINE3	grey	0.15270719
ENSG00000132386	SERPINF1	grey	-0.0108554
ENSG00000149131	SERPING1	pink	0.62905112
ENSG00000163536	SERPINI1	blue	0.75298415
ENSG00000197019	SERTAD1	pink	0.62711144
ENSG00000179833	SERTAD2	blue	-0.8248386
ENSG00000167565	SERTAD3	grey	0.36785756
ENSG00000082497	SERTAD4	magenta	0.62526368
ENSG00000203706	SERTAD4-AS1	grey	-0.2636358
ENSG00000080546	SESN1	grey	0.02356442
ENSG00000130766	SESN2	grey	0.35916347
ENSG00000149212	SESN3	red	0.70333439
ENSG00000187231	SESTD1	brown	-0.6037871
ENSG00000119335	SET	darkgrey	0.60166725
ENSG00000099381	SETD1A	salmon	-0.6023669
ENSG00000139718	SETD1B	grey	0.13244813
ENSG00000181555	SETD2	green	0.79155361
ENSG00000183576	SETD3	lightgreen	0.55578196
ENSG00000185917	SETD4	grey	-0.080523
ENSG00000168137	SETD5	grey	-0.0139286
ENSG00000206573	SETD5-AS1	grey	0.07804097
ENSG00000103037	SETD6	lightcyan	0.46962254
ENSG00000183955	SETD8	brown	-0.6099396
ENSG00000155542	SETD9	grey	0.09457094
ENSG00000143379	SETDB1	turquoise	-0.3609991
ENSG00000136169	SETDB2	grey	0.10732346
ENSG00000170364	SETMAR	grey	0.04536219
ENSG00000231905	SETP10	grey	0.17568571
ENSG00000239570	SETP11	grey	0.07896724
ENSG00000233998	SETP5	grey	0.44549688
ENSG00000107290	SETX	blue	-0.5877629
ENSG00000063015	SEZ6	blue	0.7989263
ENSG00000100095	SEZ6L	blue	0.50497488
ENSG00000174938	SEZ6L2	blue	0.89035322
ENSG00000168066	SF1	grey	-0.2412855
ENSG00000099995	SF3A1	grey	0.0894607
ENSG00000104897	SF3A2	turquoise	-0.4854219
ENSG00000183431	SF3A3	brown	0.78678854
ENSG00000230568	SF3A3P1	grey	0.18102266

ENSG00000254449	SF3A3P2	grey	0.15413996
ENSG00000115524	SF3B1	lightgreen	0.59090681
ENSG00000115128	SF3B14	grey	-0.0704874
ENSG00000087365	SF3B2	salmon	0.83337229
ENSG00000189091	SF3B3	salmon	0.52341087
ENSG00000143368	SF3B4	grey	0.10708338
ENSG00000169976	SF3B5	yellow	-0.5196607
ENSG00000198089	SFI1	turquoise	-0.6072454
ENSG00000163935	SFMBT1	grey	0.05107744
ENSG00000198879	SFMBT2	blue	-0.4116514
ENSG00000116560	SFPQ	lightgreen	0.57830979
ENSG00000156384	SFR1	yellow	0.59743992
ENSG00000104332	SFRP1	yellow	0.58354942
ENSG00000145423	SFRP2	grey	-0.3437035
ENSG00000106483	SFRP4	grey	-0.1988472
ENSG00000198818	SFT2D1	grey	-0.2537793
ENSG00000213064	SFT2D2	grey60	-0.6011731
ENSG00000225383	SFTA1P	magenta	0.41732981
ENSG00000133661	SFTPD	grey	-0.0281436
ENSG00000164466	SFXN1	grey	0.0797089
ENSG00000156398	SFXN2	grey	-0.1496682
ENSG00000107819	SFXN3	grey	-0.0367978
ENSG00000183605	SFXN4	grey	-0.0117774
ENSG00000144040	SFXN5	turquoise	0.59021019
ENSG00000163069	SGCB	turquoise	0.78215372
ENSG00000170624	SGCD	grey	-0.1927562
ENSG00000127990	SGCE	turquoise	0.36747047
ENSG00000102683	SGCG	mediumpurp	-0.5894326
ENSG00000185053	SGCZ	grey	0.14084701
ENSG00000118473	SGIP1	blue	0.68759236
ENSG00000118515	SGK1	yellow	0.65364762
ENSG00000101049	SGK2	yellow	0.70814574
ENSG00000182319	SGK223	black	0.50886964
ENSG00000104205	SGK3	turquoise	0.37623144
ENSG00000167524	SGK494	grey	-0.0318346
ENSG00000198964	SGMS1	yellow	0.69056637
ENSG00000164023	SGMS2	green	0.86424932
ENSG00000163535	SGOL2	grey	0.22089796
ENSG00000166224	SGPL1	turquoise	0.78569872
ENSG00000126821	SGPP1	grey	-0.1435008
ENSG00000163082	SGPP2	grey	0.36193057
ENSG00000181523	SGSH	black	-0.59081
ENSG00000167037	SGSM1	purple	0.81844184
ENSG00000141258	SGSM2	blue	0.74845033
ENSG00000100359	SGSM3	brown	0.682223
ENSG00000104969	SGTA	grey	0.13580859
ENSG00000197860	SGTB	blue	0.58040087
ENSG00000178188	SH2B1	turquoise	-0.6120144

ENSG00000111252	SH2B3	blue	-0.4721326
ENSG00000095370	SH2D3C	tan	0.57782482
ENSG00000104611	SH2D4A	turquoise	0.38315337
ENSG00000189410	SH2D5	blue	0.54998645
ENSG00000185437	SH3BGR	grey	-0.0409875
ENSG00000131171	SH3BGRL	lightcyan	0.89525575
ENSG00000198478	SH3BGRL2	yellow	0.25752821
ENSG00000087266	SH3BP2	turquoise	0.73390045
ENSG00000130147	SH3BP4	blue	-0.8040335
ENSG00000131370	SH3BP5	turquoise	-0.4455978
ENSG00000224660	SH3BP5-AS1	grey	0.22874217
ENSG00000175137	SH3BP5L	turquoise	-0.5223895
ENSG00000109686	SH3D19	green	0.73115747
ENSG00000214193	SH3D21	grey	-0.0960257
ENSG00000141985	SH3GL1	grey	-0.0223869
ENSG00000266777	SH3GL1P1	grey	0.02844057
ENSG00000264943	SH3GL1P2	grey	0.13423207
ENSG00000267352	SH3GL1P3	grey	0.1812944
ENSG00000107295	SH3GL2	blue	0.71249383
ENSG00000140600	SH3GL3	yellow	0.68728114
ENSG00000097033	SH3GLB1	turquoise	0.82033737
ENSG00000148341	SH3GLB2	darkmagenta	0.61571802
ENSG00000147010	SH3KBP1	blue	0.7582549
ENSG00000107957	SH3PXD2A	green	0.80690886
ENSG00000174705	SH3PXD2B	blue	-0.7048623
ENSG00000154447	SH3RF1	turquoise	0.34907749
ENSG00000156463	SH3RF2	grey	-0.1676865
ENSG00000172985	SH3RF3	grey	-0.1602385
ENSG00000169247	SH3TC2	yellow	0.87512015
ENSG00000035115	SH3YL1	grey	-0.2323763
ENSG00000161681	SHANK1	purple	0.81782628
ENSG00000162105	SHANK2	blue	0.76019023
ENSG00000226627	SHANK2-AS1	grey	0.06677991
ENSG00000236262	SHANK2-AS2	grey	0.29067948
ENSG00000251322	SHANK3	darkorange	0.58548249
ENSG00000179526	SHARPIN	magenta	-0.530633
ENSG00000107338	SHB	grey	-0.1118447
ENSG00000160691	SHC1	pink	0.85777409
ENSG00000129946	SHC2	grey	-0.1396998
ENSG00000148082	SHC3	purple	0.69560616
ENSG00000185634	SHC4	yellow	0.70949254
ENSG00000169291	SHE	grey	0.21654907
ENSG00000138606	SHF	grey	-0.1961952
ENSG00000127922	SHFM1	brown	0.60417738
ENSG00000180730	SHISA2	grey	0.06397801
ENSG00000198892	SHISA4	lightcyan	0.69679561
ENSG00000164054	SHISA5	pink	0.67228284
ENSG00000188803	SHISA6	yellow	-0.3367039

ENSG00000187902	SHISA7	darkorange	0.67704192
ENSG00000237515	SHISA9	blue	0.68034522
ENSG00000176974	SHMT1	blue	-0.4394914
ENSG00000182199	SHMT2	turquoise	0.4251819
ENSG00000108061	SHOC2	brown	-0.4079341
ENSG00000197417	SHPK	turquoise	0.39392696
ENSG00000146414	SHPRH	yellow	0.53017809
ENSG00000144736	SHQ1	grey	0.33121274
ENSG00000164403	SHROOM1	yellow	0.55244214
ENSG00000146950	SHROOM2	grey	-0.1393671
ENSG00000138771	SHROOM3	grey	0.20517643
ENSG00000158352	SHROOM4	yellow	0.87429989
ENSG00000110013	SIAE	grey	-0.0253295
ENSG00000196470	SIAH1	grey	-0.0831784
ENSG00000181788	SIAH2	grey	0.09727755
ENSG00000244265	SIAH2-AS1	blue	-0.3126059
ENSG00000215475	SIAH3	grey	-0.0726581
ENSG00000072858	SIDT1	yellow	-0.3972333
ENSG00000149577	SIDT2	grey	0.04007615
ENSG00000185187	SIGIRR	grey	-0.0162054
ENSG00000142512	SIGLEC10	cyan	0.74187757
ENSG00000105366	SIGLEC8	cyan	0.7536876
ENSG00000147955	SIGMAR1	lightcyan	0.69228765
ENSG00000170145	SIK2	brown	0.28924422
ENSG00000160584	SIK3	floralwhite	0.8285019
ENSG00000231865	SIK3-IT1	yellow	0.7300151
ENSG00000052723	SIKE1	lightcyan	0.79925411
ENSG00000120725	SIL1	grey60	0.60181162
ENSG00000159263	SIM2	grey	0.29103858
ENSG00000170085	SIMC1	grey	-0.1987115
ENSG00000127511	SIN3B	blue	0.55581268
ENSG00000213445	SIPA1	royalblue	0.72149115
ENSG00000197555	SIPA1L1	magenta	0.79607129
ENSG00000116991	SIPA1L2	magenta	-0.4159647
ENSG00000105738	SIPA1L3	grey	0.18146889
ENSG00000198053	SIRPA	red	0.64241835
ENSG00000223750	SIRPB3P	greenyellow	-0.5354927
ENSG00000096717	SIRT1	lightgreen	0.77291962
ENSG00000068903	SIRT2	yellow	0.7013719
ENSG00000142082	SIRT3	grey	0.09104178
ENSG00000089163	SIRT4	grey	0.28802607
ENSG00000124523	SIRT5	grey	0.07868993
ENSG00000077463	SIRT6	grey	0.02181983
ENSG00000187531	SIRT7	grey	0.17181478
ENSG00000184990	SIVA1	green	-0.580504
ENSG00000138083	SIX3	magenta	0.68132416
ENSG00000236502	SIX3-AS1	grey	-0.2273073
ENSG00000100625	SIX4	grey	0.45230321

ENSG00000154839	SKA1	blue	0.27137739
ENSG00000182628	SKA2	brown	-0.8374112
ENSG00000005020	SKAP2	grey	-0.222565
ENSG00000157933	SKI	brown	-0.5688589
ENSG00000180592	SKIDA1	grey	0.11077814
ENSG00000136603	SKIL	brown	-0.6075498
ENSG00000204351	SKIV2L	brown	0.62492109
ENSG00000113558	SKP1	greenyellow	0.43157285
ENSG00000145604	SKP2	turquoise	0.63921702
ENSG00000155926	SLA	royalblue	0.77991193
ENSG00000139737	SLAIN1	yellow	0.83391419
ENSG00000109171	SLAIN2	lightcyan	0.74222076
ENSG00000163950	SLBP	yellow	-0.5546133
ENSG00000126903	SLC10A3	grey	0.49419916
ENSG00000145248	SLC10A4	grey	0.07971602
ENSG00000253598	SLC10A5	grey	-0.0785534
ENSG00000120519	SLC10A7	grey	0.03578436
ENSG00000110911	SLC11A2	grey	0.11801117
ENSG00000064651	SLC12A2	yellow	0.75134469
ENSG00000124067	SLC12A4	turquoise	0.37863501
ENSG00000124140	SLC12A5	blue	0.74361432
ENSG00000140199	SLC12A6	turquoise	-0.5300543
ENSG00000221955	SLC12A8	blue	0.55450456
ENSG00000146828	SLC12A9	grey	-0.1522205
ENSG00000158296	SLC13A3	grey	-0.1044735
ENSG00000164707	SLC13A4	grey	0.33730268
ENSG00000141469	SLC14A1	blue	-0.5455099
ENSG00000163406	SLC15A2	turquoise	0.69026784
ENSG00000110446	SLC15A3	cyan	0.42675047
ENSG00000139370	SLC15A4	grey	0.27229657
ENSG00000155380	SLC16A1	blue	-0.5992379
ENSG00000226419	SLC16A1-AS1	yellow	0.55978885
ENSG00000112394	SLC16A10	greenyellow	0.51464393
ENSG00000163053	SLC16A14	grey	0.00605309
ENSG00000147100	SLC16A2	red	0.61979509
ENSG00000168679	SLC16A4	tan	0.62532766
ENSG00000108932	SLC16A6	tan	0.54460691
ENSG00000118596	SLC16A7	grey	-0.0662975
ENSG00000100156	SLC16A8	grey	-0.0864005
ENSG00000165449	SLC16A9	turquoise	0.76492082
ENSG00000187714	SLC18A3	grey	0.12626922
ENSG00000146409	SLC18B1	turquoise	0.73530803
ENSG00000173638	SLC19A1	grey	0.32950398
ENSG00000117479	SLC19A2	grey	0.4838667
ENSG00000106688	SLC1A1	blue	0.31727039
ENSG00000110436	SLC1A2	turquoise	0.71842662
ENSG00000079215	SLC1A3	turquoise	0.65427687
ENSG00000115902	SLC1A4	turquoise	0.60435345

ENSG00000105143	SLC1A6	blue	0.65736753
ENSG00000144136	SLC20A1	grey	0.28923322
ENSG00000168575	SLC20A2	turquoise	0.40774854
ENSG00000163393	SLC22A15	yellow	0.72827288
ENSG00000092096	SLC22A17	blue	0.75123413
ENSG00000146477	SLC22A3	grey	0.03782657
ENSG00000197208	SLC22A4	grey	0.13703444
ENSG00000197375	SLC22A5	grey	0.2130174
ENSG00000197901	SLC22A6	yellowgreen	0.57433224
ENSG00000089057	SLC23A2	red	0.53087007
ENSG00000074621	SLC24A1	green	0.47087425
ENSG00000155886	SLC24A2	red	-0.5787158
ENSG00000185052	SLC24A3	blue	0.51949956
ENSG00000140090	SLC24A4	grey	-0.2496965
ENSG00000188467	SLC24A5	grey	0.05438979
ENSG00000100075	SLC25A1	turquoise	0.4263312
ENSG00000108528	SLC25A11	yellow	-0.3808836
ENSG00000115840	SLC25A12	blue	0.6368389
ENSG00000004864	SLC25A13	yellow	0.71744737
ENSG00000102078	SLC25A14	brown	0.81597179
ENSG00000102743	SLC25A15	grey	0.26006164
ENSG00000251299	SLC25A15P3	grey	0.23737531
ENSG00000249288	SLC25A15P5	grey	0.39618575
ENSG00000122912	SLC25A16	grey	0.44755092
ENSG00000100372	SLC25A17	blue	0.42268482
ENSG00000182902	SLC25A18	turquoise	0.76162096
ENSG00000125454	SLC25A19	grey	0.37632875
ENSG00000178537	SLC25A20	turquoise	0.68361436
ENSG00000258708	SLC25A21-AS1	grey	0.27885827
ENSG00000177542	SLC25A22	purple	0.78372415
ENSG00000085491	SLC25A24	grey	0.22654445
ENSG00000148339	SLC25A25	grey	0.4205671
ENSG00000144741	SLC25A26	grey	0.11177716
ENSG00000153291	SLC25A27	grey	0.09781802
ENSG00000155287	SLC25A28	blue	-0.5882516
ENSG00000197119	SLC25A29	purple	0.54303067
ENSG00000075415	SLC25A3	green	-0.7246016
ENSG00000174032	SLC25A30	grey	-0.1952494
ENSG00000164933	SLC25A32	grey	0.19933123
ENSG00000171612	SLC25A33	turquoise	0.64715887
ENSG00000162461	SLC25A34	grey	0.31928821
ENSG00000125434	SLC25A35	grey	0.11157999
ENSG00000114120	SLC25A36	lightcyan	0.65648914
ENSG00000267781	SLC25A36P1	grey	0.20997878
ENSG00000147454	SLC25A37	yellow	0.59266562
ENSG00000144659	SLC25A38	brown	0.57889841
ENSG00000013306	SLC25A39	blue	-0.375031
ENSG00000226148	SLC25A39P1	grey	0.35421373

ENSG00000151729	SLC25A4	grey	-0.2676106
ENSG00000075303	SLC25A40	blue	0.48020038
ENSG00000181240	SLC25A41	grey	0.07688729
ENSG00000181035	SLC25A42	green	-0.4591605
ENSG00000077713	SLC25A43	turquoise	0.47506265
ENSG00000160785	SLC25A44	grey	0.25754216
ENSG00000162241	SLC25A45	grey	0.03410243
ENSG00000164209	SLC25A46	grey	-0.1173381
ENSG00000145832	SLC25A48	turquoise	0.43964098
ENSG00000005022	SLC25A5	turquoise	0.37118948
ENSG00000224281	SLC25A5-AS1	grey	0.07923052
ENSG00000122696	SLC25A51	grey	0.11013887
ENSG00000176274	SLC25A53	turquoise	0.58082613
ENSG00000215347	SLC25A5P1	grey	0.21346211
ENSG00000169100	SLC25A6	turquoise	-0.4763935
ENSG00000181045	SLC26A11	black	-0.671141
ENSG00000155850	SLC26A2	blue	-0.6021862
ENSG00000233705	SLC26A4-AS1	purple	0.64662563
ENSG00000225697	SLC26A6	grey	0.01971865
ENSG00000112053	SLC26A8	blue	0.65589213
ENSG00000130304	SLC27A1	grey	0.31128963
ENSG00000143554	SLC27A3	grey	0.00357682
ENSG00000167114	SLC27A4	blue	0.65557633
ENSG00000083807	SLC27A5	brown	0.42121
ENSG00000137860	SLC28A2	grey	0.02739437
ENSG00000112759	SLC29A1	greenyellow	0.47601475
ENSG00000174669	SLC29A2	grey	-0.0094539
ENSG00000198246	SLC29A3	blue	-0.3507803
ENSG00000117394	SLC2A1	pink	0.58242373
ENSG00000227533	SLC2A1-AS1	turquoise	-0.4858563
ENSG00000197496	SLC2A10	turquoise	0.79221582
ENSG00000133460	SLC2A11	yellow	-0.5335294
ENSG00000146411	SLC2A12	turquoise	0.55583194
ENSG00000151229	SLC2A13	purple	0.68077068
ENSG00000185031	SLC2A3P2	grey	0.06544461
ENSG00000254088	SLC2A3P4	grey	0.31674271
ENSG00000181856	SLC2A4	turquoise	0.59353488
ENSG00000125520	SLC2A4RG	grey	0.11991333
ENSG00000142583	SLC2A5	royalblue	0.76961248
ENSG00000160326	SLC2A6	grey	0.43993811
ENSG00000136856	SLC2A8	grey	0.12552681
ENSG00000170385	SLC30A1	grey	0.12835278
ENSG00000196660	SLC30A10	yellowgreen	0.6828822
ENSG00000104154	SLC30A4	black	0.80917416
ENSG00000145740	SLC30A5	red	0.63044856
ENSG00000152683	SLC30A6	turquoise	0.60106315
ENSG00000162695	SLC30A7	turquoise	0.40317416
ENSG00000014824	SLC30A9	brown	0.31804012

ENSG00000136868	SLC31A1	pink	0.5499576
ENSG00000136867	SLC31A2	yellow	0.78091246
ENSG00000101438	SLC32A1	magenta	0.61936662
ENSG00000169359	SLC33A1	grey	0.18262358
ENSG00000164414	SLC35A1	grey	-0.1275198
ENSG00000102100	SLC35A2	grey	0.10346513
ENSG00000176087	SLC35A4	turquoise	0.41536732
ENSG00000138459	SLC35A5	turquoise	0.46612324
ENSG00000121073	SLC35B1	green	-0.6838964
ENSG00000157593	SLC35B2	turquoise	0.62630502
ENSG00000124786	SLC35B3	blue	-0.4104398
ENSG00000205060	SLC35B4	green	-0.5997991
ENSG00000181830	SLC35C1	turquoise	0.37143179
ENSG00000080189	SLC35C2	turquoise	0.34201662
ENSG00000116704	SLC35D1	grey	0.27845664
ENSG00000130958	SLC35D2	yellow	0.70329752
ENSG00000182747	SLC35D3	grey	0.01457899
ENSG00000127526	SLC35E1	yellow	-0.5142039
ENSG00000238286	SLC35E1P1	grey	0.29557383
ENSG00000215790	SLC35E2	grey	-0.0260197
ENSG00000189339	SLC35E2B	grey	-0.2393299
ENSG00000175782	SLC35E3	grey	0.24385283
ENSG00000100036	SLC35E4	turquoise	-0.3835548
ENSG00000183780	SLC35F3	magenta	0.51565961
ENSG00000151812	SLC35F4	turquoise	-0.3824994
ENSG00000115084	SLC35F5	yellow	0.47087654
ENSG00000213699	SLC35F6	turquoise	0.44783023
ENSG00000176273	SLC35G1	grey	0.33089158
ENSG00000168917	SLC35G2	grey	0.12934608
ENSG00000123643	SLC36A1	grey	0.10980357
ENSG00000180773	SLC36A4	grey	-0.1879274
ENSG00000160190	SLC37A1	yellow	-0.3831383
ENSG00000157800	SLC37A3	brown	0.45398157
ENSG00000137700	SLC37A4	grey	0.08024587
ENSG00000111371	SLC38A1	blue	0.63548206
ENSG00000157637	SLC38A10	lightcyan	-0.5700346
ENSG00000169507	SLC38A11	black	0.61632352
ENSG00000134294	SLC38A2	yellow	0.55034617
ENSG00000188338	SLC38A3	turquoise	0.54048127
ENSG00000017483	SLC38A5	tan	0.64455636
ENSG00000139974	SLC38A6	grey	-0.3357377
ENSG00000177058	SLC38A9	grey	0.38043632
ENSG00000143570	SLC39A1	pink	0.73116282
ENSG00000196950	SLC39A10	blue	0.30121613
ENSG00000148482	SLC39A12	turquoise	0.42159793
ENSG00000165915	SLC39A13	grey	-0.01791
ENSG00000104635	SLC39A14	pink	0.87222184
ENSG00000141873	SLC39A3	brown	0.33249618

ENSG00000141424	SLC39A6	brown	-0.3553114
ENSG00000112473	SLC39A7	grey	0.09310823
ENSG00000138821	SLC39A8	black	0.69174272
ENSG00000029364	SLC39A9	grey	0.18724718
ENSG00000138079	SLC3A1	grey	0.40910555
ENSG00000168003	SLC3A2	turquoise	0.59825045
ENSG00000138449	SLC40A1	turquoise	0.64544222
ENSG00000133065	SLC41A1	turquoise	0.82573386
ENSG00000136052	SLC41A2	blue	0.39803364
ENSG00000167703	SLC43A2	grey	0.27301473
ENSG00000134802	SLC43A3	turquoise	0.48910193
ENSG00000070214	SLC44A1	yellow	0.86484323
ENSG00000129353	SLC44A2	turquoise	0.79576927
ENSG00000143036	SLC44A3	turquoise	0.66443924
ENSG00000162426	SLC45A1	blue	0.61299978
ENSG00000158715	SLC45A3	yellow	0.80594245
ENSG00000022567	SLC45A4	grey	0.09770829
ENSG00000076351	SLC46A1	grey	0.31044453
ENSG00000139508	SLC46A3	turquoise	0.53825735
ENSG00000142494	SLC47A1	grey	0.28267839
ENSG00000211584	SLC48A1	yellow	0.65538401
ENSG00000144290	SLC4A10	blue	0.87338581
ENSG00000088836	SLC4A11	grey	0.08578941
ENSG00000163798	SLC4A1AP	turquoise	-0.4551685
ENSG00000164889	SLC4A2	turquoise	0.46991469
ENSG00000114923	SLC4A3	purple	0.74338196
ENSG00000080493	SLC4A4	turquoise	0.69737033
ENSG00000033867	SLC4A7	turquoise	-0.5227265
ENSG00000050438	SLC4A8	grey	0.22515745
ENSG00000169241	SLC50A1	blue	-0.2950446
ENSG00000185803	SLC52A2	grey	0.31395833
ENSG00000158865	SLC5A11	yellow	0.73575363
ENSG00000148942	SLC5A12	red	-0.3979904
ENSG00000198743	SLC5A3	grey	-0.0595532
ENSG00000100191	SLC5A4	grey	0.15492537
ENSG00000138074	SLC5A6	tan	0.72572276
ENSG00000115665	SLC5A7	grey	-0.0363544
ENSG00000157103	SLC6A1	yellowgreen	0.66688279
ENSG00000232287	SLC6A1-AS1	grey	0.05474194
ENSG00000111181	SLC6A12	grey	-0.0254198
ENSG00000010379	SLC6A13	grey	-0.0084495
ENSG00000072041	SLC6A15	turquoise	-0.3891929
ENSG00000197106	SLC6A17	blue	0.79315277
ENSG00000131389	SLC6A6	black	0.71590302
ENSG00000130821	SLC6A8	yellow	0.71029893
ENSG00000139514	SLC7A1	tan	0.83622191
ENSG00000130876	SLC7A10	turquoise	0.59506884
ENSG00000151012	SLC7A11	turquoise	0.85207373

ENSG00000013293	SLC7A14	grey	-0.2256574
ENSG00000003989	SLC7A2	turquoise	0.43814109
ENSG00000099960	SLC7A4	blue	0.4565883
ENSG00000103257	SLC7A5	tan	0.8238991
ENSG00000103064	SLC7A6	grey	0.1685156
ENSG00000103061	SLC7A6OS	grey	0.09836905
ENSG00000155465	SLC7A7	royalblue	0.55511795
ENSG00000092068	SLC7A8	grey60	0.68356166
ENSG00000183023	SLC8A1	purple	0.86068144
ENSG00000227028	SLC8A1-AS1	purple	0.79382102
ENSG00000118160	SLC8A2	blue	0.63252063
ENSG00000100678	SLC8A3	blue	0.73567076
ENSG00000089060	SLC8B1	grey	0.19700487
ENSG00000090020	SLC9A1	black	0.78594113
ENSG00000115616	SLC9A2	grey	0.17709546
ENSG00000109062	SLC9A3R1	blue	-0.6713965
ENSG00000065054	SLC9A3R2	yellow	0.42731704
ENSG00000135740	SLC9A5	turquoise	-0.4909684
ENSG00000198689	SLC9A6	blue	0.64037985
ENSG00000065923	SLC9A7	turquoise	-0.6725597
ENSG00000227825	SLC9A7P1	grey	0.36175955
ENSG00000197818	SLC9A8	grey	0.04315056
ENSG00000240012	SLC9A9-AS1	blue	-0.3710876
ENSG00000164037	SLC9B1	grey	0.23915036
ENSG00000164038	SLC9B2	grey	0.0678392
ENSG00000084453	SLCO1A2	green	0.90267382
ENSG00000139155	SLCO1C1	turquoise	0.68568364
ENSG00000137491	SLCO2B1	cyan	0.67225319
ENSG00000176463	SLCO3A1	yellow	0.79080332
ENSG00000101187	SLCO4A1	pink	0.84048281
ENSG00000137571	SLCO5A1	grey	-0.033195
ENSG00000172716	SLFN11	grey	0.04952023
ENSG00000166750	SLFN5	grey	-0.0380554
ENSG00000119705	SLIRP	greenyellow	0.61333526
ENSG00000145147	SLIT2	turquoise	-0.7148655
ENSG00000248228	SLIT2-IT1	grey	0.25258653
ENSG00000184347	SLIT3	turquoise	-0.4550633
ENSG00000178235	SLITRK1	darkorange	0.47912968
ENSG00000185985	SLITRK2	turquoise	0.72132811
ENSG00000121871	SLITRK3	blue	0.58425517
ENSG00000184564	SLITRK6	grey	-0.0855704
ENSG00000065613	SLK	magenta	0.69184141
ENSG00000141391	SLMO1	grey	0.10844823
ENSG00000101166	SLMO2	turquoise	0.80153436
ENSG00000137776	SLTM	darkgrey	0.77167259
ENSG00000164609	SLU7	turquoise	-0.5597562
ENSG00000188827	SLX4	grey	0.09124695
ENSG00000149346	SLX4IP	grey	-0.015395

ENSG00000170365	SMAD1	grey	-0.259595
ENSG00000250902	SMAD1-AS1	grey	0.32283084
ENSG00000250582	SMAD1-AS2	grey	0.36079829
ENSG00000175387	SMAD2	brown	-0.6174962
ENSG00000166949	SMAD3	black	0.7119187
ENSG00000141646	SMAD4	turquoise	0.59506031
ENSG00000113658	SMAD5	turquoise	0.83623633
ENSG00000137834	SMAD6	tan	0.67510809
ENSG00000101665	SMAD7	black	0.62076487
ENSG00000120693	SMAD9	grey	0.01189688
ENSG00000236711	SMAD9-AS1	grey	0.25230797
ENSG00000112305	SMAP1	blue	0.64972463
ENSG00000084070	SMAP2	turquoise	-0.7662977
ENSG00000102038	SMARCA1	turquoise	-0.32465
ENSG00000080503	SMARCA2	turquoise	0.40216604
ENSG00000127616	SMARCA4	turquoise	-0.5525252
ENSG00000153147	SMARCA5	lightgreen	0.82316556
ENSG00000138375	SMARCAL1	turquoise	-0.5260834
ENSG00000099956	SMARCB1	brown	0.38813492
ENSG00000173473	SMARCC1	green	0.85797719
ENSG00000139613	SMARCC2	grey	-0.1090871
ENSG00000066117	SMARCD1	turquoise	-0.3865514
ENSG00000108604	SMARCD2	grey	0.07195743
ENSG00000082014	SMARCD3	purple	0.70839707
ENSG00000073584	SMARCE1	grey	-0.0272636
ENSG00000225215	SMARCE1P1	grey	0.34191174
ENSG00000230793	SMARCE1P5	grey	0.18449503
ENSG00000072501	SMC1A	darkgrey	0.74104663
ENSG00000136824	SMC2	grey	-0.1255688
ENSG00000108055	SMC3	lightgreen	0.68535476
ENSG00000113810	SMC4	yellow	0.49156293
ENSG00000198887	SMC5	pink	0.44296293
ENSG00000268364	SMC5-AS1	grey	0.28296359
ENSG00000163029	SMC6	lightgreen	0.54354781
ENSG00000101596	SMCHD1	blue	-0.771057
ENSG00000166002	SMCO4	grey	-0.1119808
ENSG00000176994	SMCR8	grey	-0.0785462
ENSG00000183172	SMDT1	greenyellow	0.5977433
ENSG00000100796	SMEK1	blue	-0.452556
ENSG00000138041	SMEK2	turquoise	0.64980857
ENSG00000157106	SMG1	grey	-0.0005006
ENSG00000237296	SMG1P1	grey	0.20808628
ENSG00000198952	SMG5	turquoise	-0.4310896
ENSG00000070366	SMG6	grey	0.30037657
ENSG00000116698	SMG7	grey	-0.166144
ENSG00000167447	SMG8	grey	0.24685958
ENSG00000184785	SMIM10	turquoise	0.33660155
ENSG00000163866	SMIM12	green	-0.5410426

ENSG00000163683	SMIM14	bisque4	0.83966201
ENSG00000188725	SMIM15	blue	-0.4474541
ENSG00000268182	SMIM17	grey	-0.0782682
ENSG00000253457	SMIM18	grey	0.07193445
ENSG00000227258	SMIM2-AS1	grey	0.25442597
ENSG00000250317	SMIM20	turquoise	0.33479801
ENSG00000168273	SMIM4	grey	-0.0557153
ENSG00000259120	SMIM6	yellow	0.49107141
ENSG00000214046	SMIM7	grey	0.03836192
ENSG00000111850	SMIM8	brown	-0.4305853
ENSG00000240204	SMKR1	brown	0.57263999
ENSG00000256162	SMLR1	black	0.58706383
ENSG00000172062	SMN1	grey	0.06967042
ENSG00000205571	SMN2	grey	0.25716824
ENSG00000119953	SMNDC1	lightgreen	0.63536101
ENSG00000128602	SMO	turquoise	0.57507111
ENSG00000112562	SMOC2	grey	-0.1938205
ENSG00000088826	SMOX	turquoise	0.6895655
ENSG00000166311	SMPD1	yellow	0.29406556
ENSG00000135587	SMPD2	grey	-0.2067762
ENSG00000103056	SMPD3	black	0.81665973
ENSG00000136699	SMPD4	turquoise	-0.4987724
ENSG00000172594	SMPDL3A	grey	0.02355173
ENSG00000102172	SMS	grey	-0.0470375
ENSG00000183963	SMTN	blue	-0.5279037
ENSG00000122692	SMU1	brown	-0.3611691
ENSG00000123415	SMUG1	brown	0.39727475
ENSG00000198742	SMURF1	darkmagenta	0.69687612
ENSG00000108854	SMURF2	brown	-0.7326113
ENSG00000143499	SMYD2	grey	-0.1856487
ENSG00000185420	SMYD3	brown	0.78156897
ENSG00000230184	SMYD3-IT1	grey	0.28859905
ENSG00000186532	SMYD4	grey	0.18951126
ENSG00000135632	SMYD5	blue	0.35846224
ENSG00000124216	SNAI1	grey	0.11082724
ENSG00000237194	SNAI1P1	grey	0.18563988
ENSG00000185669	SNAI3	grey	-0.1598679
ENSG00000260630	SNAI3-AS1	grey	-0.2183215
ENSG00000092531	SNAP23	blue	-0.712407
ENSG00000132639	SNAP25	blue	0.70844905
ENSG00000227906	SNAP25-AS1	blue	0.59568632
ENSG00000099940	SNAP29	turquoise	0.39967544
ENSG00000143740	SNAP47	brown	0.65893793
ENSG00000065609	SNAP91	blue	0.85212739
ENSG00000023608	SNAPC1	grey	0.05729809
ENSG00000104976	SNAPC2	blue	0.41143059
ENSG00000165684	SNAPC4	blue	0.74741384
ENSG00000143553	SNAPIN	grey	0.1322513

ENSG00000145335	SNCA	turquoise	-0.4438363
ENSG00000074317	SNCB	blue	0.5642949
ENSG00000173267	SNCG	magenta	-0.4469561
ENSG00000197157	SND1	turquoise	-0.4952944
ENSG00000162804	SNED1	blue	-0.7072298
ENSG00000159210	SNF8	salmon	0.5281823
ENSG00000247092	SNHG10	grey	-0.0431089
ENSG00000174365	SNHG11	grey	0.17012567
ENSG00000197989	SNHG12	grey	-0.2064471
ENSG00000224078	SNHG14	bisque4	0.68945082
ENSG00000232956	SNHG15	brown	0.55789314
ENSG00000163597	SNHG16	grey	-0.0087904
ENSG00000196756	SNHG17	grey	0.03926141
ENSG00000250786	SNHG18	blue	-0.4621508
ENSG00000242125	SNHG3	grey	0.11683308
ENSG00000203875	SNHG5	grey	-0.0815062
ENSG00000245910	SNHG6	grey	-0.5025554
ENSG00000233016	SNHG7	grey	0.04575761
ENSG00000269893	SNHG8	brown	0.60917588
ENSG00000255198	SNHG9	greenyellow	0.31696005
ENSG00000163877	SNIP1	grey	-0.060248
ENSG00000184602	SNN	blue	-0.6722934
ENSG00000252405	snoMe28S-Am26	grey	0.5541697
ENSG00000251959	snoR442	grey	0.35531498
ENSG00000207407	SNORA1	grey	0.34178425
ENSG00000221164	SNORA11	grey	0.58114769
ENSG00000221245	SNORA11	grey	0.40095387
ENSG00000221716	SNORA11	grey	0.33250912
ENSG00000221102	SNORA11B	grey	0.46211479
ENSG00000212342	SNORA12	grey	0.60432221
ENSG00000212464	SNORA12	grey	0.55622577
ENSG00000212175	SNORA12	turquoise	0.46065729
ENSG00000201643	SNORA14A	grey	0.4961422
ENSG00000207181	SNORA14B	grey	0.21631248
ENSG00000206785	SNORA15	grey	0.45796399
ENSG00000207062	SNORA15	grey	0.46747439
ENSG00000212293	SNORA16	grey	0.17606982
ENSG00000201544	SNORA16B	grey	0.40369662
ENSG00000212461	SNORA17	grey	0.51965397
ENSG00000206592	SNORA18	grey	0.47814851
ENSG00000202343	SNORA2	grey	0.32403908
ENSG00000199571	SNORA22	grey	0.41914294
ENSG00000206603	SNORA22	grey	0.48758742
ENSG00000206634	SNORA22	grey	0.37920738
ENSG00000207344	SNORA22	grey	0.47332987
ENSG00000200075	SNORA25	grey	0.24392415
ENSG00000201957	SNORA25	grey	0.43230291
ENSG00000252290	SNORA25	grey	0.51662469

ENSG00000252993	SNORA25	grey	0.43554447
ENSG00000212588	SNORA26	grey	0.22896454
ENSG00000221639	SNORA3	grey	0.22875247
ENSG00000206755	SNORA30	grey	0.24935996
ENSG00000252291	SNORA31	grey	0.36191489
ENSG00000252349	SNORA31	grey	0.32888847
ENSG00000253013	SNORA31	grey	0.24375138
ENSG00000253041	SNORA31	grey	0.36314607
ENSG00000253051	SNORA31	grey	0.33498298
ENSG00000253059	SNORA31	grey	0.50429271
ENSG00000201384	SNORA32	grey	0.51329981
ENSG00000200534	SNORA33	grey	0.2783308
ENSG00000252045	SNORA33	grey	0.53072824
ENSG00000208839	SNORA35	grey	0.11700242
ENSG00000222370	SNORA36B	grey	0.16578666
ENSG00000207233	SNORA37	grey	0.48144364
ENSG00000208308	SNORA40	grey	0.46059041
ENSG00000253047	SNORA40	grey	0.38430158
ENSG00000212579	SNORA40	pink	0.51804295
ENSG00000207217	SNORA42	grey	0.31282025
ENSG00000212607	SNORA45	grey	0.35028205
ENSG00000212371	SNORA46	grey	0.51599314
ENSG00000238961	SNORA47	grey	0.37137107
ENSG00000212391	SNORA48	brown	-0.4052005
ENSG00000212181	SNORA48	grey	0.56494531
ENSG00000212458	SNORA48	grey	0.19940391
ENSG00000252774	SNORA48	grey	0.57145643
ENSG00000206952	SNORA50	grey	0.21371648
ENSG00000200113	SNORA51	blue	-0.6304525
ENSG00000201516	SNORA51	grey	0.62470365
ENSG00000201863	SNORA51	grey	0.4787735
ENSG00000207008	SNORA54	grey	0.50099437
ENSG00000212529	SNORA57	grey	0.51664609
ENSG00000212567	SNORA57	grey	0.34817484
ENSG00000202374	SNORA62	grey	0.45306448
ENSG00000201448	SNORA63	grey	0.41083073
ENSG00000252448	SNORA63	grey	0.1144315
ENSG00000202517	SNORA64	grey	0.42283591
ENSG00000201302	SNORA65	grey	0.33783521
ENSG00000207523	SNORA66	grey	0.20013093
ENSG00000251795	SNORA66	grey	0.16429417
ENSG00000201619	SNORA67	grey	0.2107998
ENSG00000212338	SNORA67	grey	0.35773258
ENSG00000252473	SNORA67	grey	0.3290648
ENSG00000206913	SNORA7	grey	0.25778428
ENSG00000252657	SNORA70	grey	0.34447526
ENSG00000253042	SNORA70	lightgreen	0.51698471
ENSG00000206937	SNORA70B	grey	0.38127318

ENSG00000207221	SNORA70E	grey	0.47364134
ENSG00000206650	SNORA70G	greenyellow	-0.4631021
ENSG00000235408	SNORA71B	grey	0.37964151
ENSG00000201944	SNORA72	grey	0.17306648
ENSG00000199977	SNORA73	grey	0.43127851
ENSG00000200087	SNORA73B	grey	0.41849272
ENSG00000252213	SNORA74	grey	0.51481817
ENSG00000252904	SNORA76	grey	0.47273449
ENSG00000266402	SNORA76	grey	0.29883669
ENSG00000221643	SNORA77	grey	0.16862429
ENSG00000222489	SNORA79	grey	0.13810163
ENSG00000200792	SNORA80	grey	0.42917703
ENSG00000239183	SNORA84	grey	0.41229842
ENSG00000252192	SNORA9	grey	0.42354776
ENSG00000221500	SNORD100	grey	0.62488307
ENSG00000206754	SNORD101	grey	0.42101108
ENSG00000239014	SNORD108	grey	0.07229374
ENSG00000270246	SNORD109A	grey	0.31303228
ENSG00000238317	SNORD11	grey	0.54340986
ENSG00000238966	SNORD112	grey	0.51903841
ENSG00000251769	SNORD112	grey	0.27509692
ENSG00000251949	SNORD112	grey	0.24533079
ENSG00000252009	SNORD112	grey	0.31243336
ENSG00000252354	SNORD112	grey	0.25775817
ENSG00000252559	SNORD112	grey	0.4569384
ENSG00000252873	SNORD112	grey	0.33838127
ENSG00000252946	SNORD112	grey	0.32651998
ENSG00000201036	SNORD113	grey	0.10314477
ENSG00000201710	SNORD113	grey	0.03480648
ENSG00000222095	SNORD113	grey	0.15583521
ENSG00000222185	SNORD113	grey	0.39546886
ENSG00000202191	SNORD113-1	grey	0.07873479
ENSG00000212384	SNORD113-2	grey	0.18670276
ENSG00000201700	SNORD113-3	yellow	-0.3936591
ENSG00000201672	SNORD113-4	grey	0.45640147
ENSG00000200632	SNORD113-7	grey	0.43670629
ENSG00000200367	SNORD113-8	grey	0.48080254
ENSG00000201950	SNORD113-9	grey	0.48923458
ENSG00000199575	SNORD114-1	grey	0.33913724
ENSG00000200279	SNORD114-10	grey	0.25888138
ENSG00000200608	SNORD114-11	grey	0.45501418
ENSG00000202270	SNORD114-12	grey	0.53467871
ENSG00000201247	SNORD114-13	grey	0.52260818
ENSG00000199593	SNORD114-14	grey	0.47489608
ENSG00000201557	SNORD114-15	grey	0.45028374
ENSG00000199914	SNORD114-16	grey	0.48397525
ENSG00000201569	SNORD114-17	grey	0.20548846
ENSG00000202142	SNORD114-18	grey	0.232429

ENSG00000199942	SNORD114-19	grey	0.4416901
ENSG00000200823	SNORD114-2	grey	0.21590155
ENSG00000202048	SNORD114-20	grey	0.3926102
ENSG00000200406	SNORD114-23	grey	0.4158403
ENSG00000201899	SNORD114-24	grey	0.50665878
ENSG00000200612	SNORD114-25	grey	0.42858272
ENSG00000200413	SNORD114-26	grey	0.37643215
ENSG00000200636	SNORD114-27	grey	0.44943084
ENSG00000200480	SNORD114-28	grey	0.64827993
ENSG00000201689	SNORD114-29	grey	0.38729613
ENSG00000201839	SNORD114-3	grey	0.38096084
ENSG00000201318	SNORD114-30	grey	0.30498765
ENSG00000200089	SNORD114-31	grey	0.32662461
ENSG00000200832	SNORD114-4	grey	0.4541391
ENSG00000201263	SNORD114-6	grey	0.00841869
ENSG00000199390	SNORD114-7	grey	0.17446536
ENSG00000212428	SNORD115	grey	0.2210299
ENSG00000212380	SNORD115-45	grey	0.07995594
ENSG00000212528	SNORD115-47	grey	0.4684719
ENSG00000252985	SNORD116	grey	0.23547828
ENSG00000207063	SNORD116-1	grey	0.54346895
ENSG00000207197	SNORD116-12	grey	0.48199941
ENSG00000207137	SNORD116-13	grey	0.48694328
ENSG00000206621	SNORD116-14	grey	0.2828956
ENSG00000207263	SNORD116-16	grey	0.39775218
ENSG00000206688	SNORD116-18	grey	0.46215649
ENSG00000207460	SNORD116-19	grey	0.27309723
ENSG00000207001	SNORD116-2	grey	0.46168879
ENSG00000261069	SNORD116-20	grey	0.18402596
ENSG00000207279	SNORD116-24	grey	0.41055227
ENSG00000252326	SNORD116-25	grey	0.47380928
ENSG00000251815	SNORD116-26	grey	0.36980577
ENSG00000251896	SNORD116-27	grey	0.3733738
ENSG00000207245	SNORD116-29	grey	0.22566831
ENSG00000252277	SNORD116-30	grey	0.23599901
ENSG00000207442	SNORD116-6	grey	0.5220981
ENSG00000207133	SNORD116-7	grey	0.4138537
ENSG00000207093	SNORD116-8	grey	0.39949838
ENSG00000206727	SNORD116-9	grey	0.4493984
ENSG00000201785	SNORD117	grey	0.41889831
ENSG00000238886	SNORD121A	grey	0.46259689
ENSG00000238793	SNORD124	grey	0.44506748
ENSG00000222365	SNORD12B	grey	0.44879124
ENSG00000201330	SNORD32B	grey	0.31911158
ENSG00000252022	SNORD33	grey	0.45801836
ENSG00000200706	SNORD45	grey	0.49314261
ENSG00000251830	SNORD46	grey	0.20640407
ENSG00000265706	SNORD53_SNOR	grey	0.42391944

ENSG00000200753	SNORD56	grey	0.27538215
ENSG00000207444	SNORD56B	grey	0.34324229
ENSG00000206989	SNORD63	grey	0.38858576
ENSG00000222937	SNORD63	grey	0.53796097
ENSG00000251987	SNORD63	grey	0.27230191
ENSG00000212161	SNORD64	grey	0.3394734
ENSG00000212135	SNORD67	grey	0.48965537
ENSG00000212452	SNORD69	grey	0.41852099
ENSG00000207297	SNORD7	grey	0.4308372
ENSG00000212534	SNORD70	grey	0.44734649
ENSG00000223224	SNORD71	grey	0.28528661
ENSG00000201264	SNORD73	grey	0.28899616
ENSG00000200999	SNORD74	grey	0.55222869
ENSG00000201025	SNORD74	grey	0.22225821
ENSG00000201666	SNORD74	grey	0.37980671
ENSG00000221300	SNORD75	grey	0.50565949
ENSG00000251878	SNORD79	grey	0.34608367
ENSG00000200785	SNORD8	grey	0.33151922
ENSG00000202023	SNORD81	grey	0.34731318
ENSG00000264994	SNORD92	grey	0.55095495
ENSG00000208772	SNORD94	grey	0.33889384
ENSG00000221539	SNORD99	grey	0.31251202
ENSG00000238832	snoU109	grey	0.33443937
ENSG00000238693	snoU13	black	0.47818814
ENSG00000238834	snoU13	green	0.45027309
ENSG00000238301	snoU13	grey	0.27769843
ENSG00000238311	snoU13	grey	0.2186546
ENSG00000238314	snoU13	grey	0.48853852
ENSG00000238319	snoU13	grey	0.40118118
ENSG00000238322	snoU13	grey	0.40567501
ENSG00000238329	snoU13	grey	0.23212444
ENSG00000238339	snoU13	grey	0.55908651
ENSG00000238343	snoU13	grey	0.37558955
ENSG00000238351	snoU13	grey	0.56294027
ENSG00000238368	snoU13	grey	0.50360164
ENSG00000238369	snoU13	grey	0.3898449
ENSG00000238384	snoU13	grey	0.381325
ENSG00000238403	snoU13	grey	0.36403986
ENSG00000238422	snoU13	grey	0.52157643
ENSG00000238425	snoU13	grey	0.50987183
ENSG00000238450	snoU13	grey	0.42952084
ENSG00000238462	snoU13	grey	0.42086256
ENSG00000238502	snoU13	grey	0.36711267
ENSG00000238528	snoU13	grey	0.47919525
ENSG00000238533	snoU13	grey	0.43610577
ENSG00000238541	snoU13	grey	0.5553934
ENSG00000238564	snoU13	grey	0.45530069
ENSG00000238566	snoU13	grey	0.26279791

ENSG00000238567	snoU13	grey	0.46770411
ENSG00000238596	snoU13	grey	0.33760923
ENSG00000238612	snoU13	grey	0.58531961
ENSG00000238615	snoU13	grey	0.43269925
ENSG00000238624	snoU13	grey	0.38832281
ENSG00000238631	snoU13	grey	0.54600814
ENSG00000238683	snoU13	grey	0.50669898
ENSG00000238717	snoU13	grey	0.44841111
ENSG00000238718	snoU13	grey	0.46785222
ENSG00000238722	snoU13	grey	0.4371658
ENSG00000238732	snoU13	grey	0.52965386
ENSG00000238736	snoU13	grey	0.52075609
ENSG00000238756	snoU13	grey	0.32928363
ENSG00000238763	snoU13	grey	0.32587237
ENSG00000238781	snoU13	grey	0.54264955
ENSG00000238790	snoU13	grey	0.20872024
ENSG00000238806	snoU13	grey	0.51074775
ENSG00000238815	snoU13	grey	0.62623174
ENSG00000238841	snoU13	grey	0.50070013
ENSG00000238858	snoU13	grey	0.39876009
ENSG00000238864	snoU13	grey	0.24800267
ENSG00000238868	snoU13	grey	0.44495008
ENSG00000238878	snoU13	grey	0.48811911
ENSG00000238888	snoU13	grey	0.32273085
ENSG00000238896	snoU13	grey	0.28572065
ENSG00000238902	snoU13	grey	0.34339687
ENSG00000238906	snoU13	grey	0.33934658
ENSG00000238918	snoU13	grey	0.33444623
ENSG00000238922	snoU13	grey	0.39604849
ENSG00000238939	snoU13	grey	0.57196425
ENSG00000238940	snoU13	grey	0.58875429
ENSG00000238947	snoU13	grey	0.20147528
ENSG00000238948	snoU13	grey	0.40226308
ENSG00000238974	snoU13	grey	0.38903332
ENSG00000238975	snoU13	grey	0.40693958
ENSG00000238982	snoU13	grey	0.43211421
ENSG00000238988	snoU13	grey	0.35556373
ENSG00000239011	snoU13	grey	0.48408167
ENSG00000239024	snoU13	grey	0.50833362
ENSG00000239033	snoU13	grey	0.4281667
ENSG00000239034	snoU13	grey	0.60341935
ENSG00000239035	snoU13	grey	0.37550378
ENSG00000239041	snoU13	grey	0.5162546
ENSG00000239045	snoU13	grey	0.51357864
ENSG00000239052	snoU13	grey	0.42577238
ENSG00000239054	snoU13	grey	0.28111646
ENSG00000239063	snoU13	grey	0.55285238
ENSG00000239077	snoU13	grey	0.42366251

ENSG00000239084	snoU13	grey	0.48614189
ENSG00000239089	snoU13	grey	0.3936714
ENSG00000239091	snoU13	grey	0.44201292
ENSG00000239100	snoU13	grey	0.29362307
ENSG00000239103	snoU13	grey	0.49429759
ENSG00000239121	snoU13	grey	0.54936418
ENSG00000239128	snoU13	grey	0.40403941
ENSG00000239132	snoU13	grey	0.39806488
ENSG00000239133	snoU13	grey	0.512765
ENSG00000239136	snoU13	grey	0.31915073
ENSG00000239140	snoU13	grey	0.49825047
ENSG00000239161	snoU13	grey	0.38854475
ENSG00000239170	snoU13	grey	0.48559106
ENSG00000251737	snoU13	grey	0.43184239
ENSG00000251860	snoU13	grey	0.3503581
ENSG00000252058	snoU13	grey	0.48768007
ENSG00000252071	snoU13	grey	0.08216617
ENSG00000252265	snoU13	grey	0.45114804
ENSG00000252295	snoU13	grey	0.22501192
ENSG00000252447	snoU13	grey	0.36117006
ENSG00000252525	snoU13	grey	0.31827829
ENSG00000252609	snoU13	grey	0.40357314
ENSG00000238465	snoU13	red	0.4820186
ENSG00000238309	snoU13	turquoise	0.4537702
ENSG00000251847	snoZ13_snr52	grey	0.34122096
ENSG00000251721	snoZ5	grey	0.5045875
ENSG00000101298	SNPH	grey	0.19403594
ENSG00000163788	SNRK	grey	0.29798855
ENSG00000234617	SNRK-AS1	grey	0.23574449
ENSG00000144028	SNRNP200	grey	0.0699327
ENSG00000124380	SNRNP27	lightcyan	0.60111599
ENSG00000184209	SNRNP35	grey	-0.1448179
ENSG00000060688	SNRNP40	grey	-0.0905894
ENSG00000077312	SNRPA	grey	-0.0968698
ENSG00000131876	SNRPA1	turquoise	-0.5531299
ENSG00000125835	SNRPB	grey	0.18040648
ENSG00000124562	SNRPC	grey	-0.2031995
ENSG00000167088	SNRPD1	brown	0.37405218
ENSG00000125743	SNRPD2	brown	0.77669361
ENSG00000100028	SNRPD3	red	0.44014211
ENSG00000182004	SNRPE	salmon	0.68243646
ENSG00000214124	SNRPEP9	grey	0.28993546
ENSG00000139343	SNRPF	brown	0.54134265
ENSG00000143977	SNRPG	mediumpurp	0.64231713
ENSG00000236577	SNRPGP14	grey	0.33763474
ENSG00000224543	SNRPGP15	grey	0.18106601
ENSG00000257979	SNRPGP18	grey	0.41389819
ENSG00000239919	SNRPGP3	grey	0.22749829

ENSG00000267203	SNRPGP4	grey	0.4239561
ENSG00000228551	SNRPGP9	grey	0.24105381
ENSG00000128739	SNRPN	grey	0.10082831
ENSG00000101400	SNTA1	turquoise	0.74539014
ENSG00000172164	SNTB1	turquoise	0.67263861
ENSG00000168807	SNTB2	grey	0.14467171
ENSG00000169371	SNUPN	brown	0.40947686
ENSG00000214265	SNURF	grey	0.0737817
ENSG00000100603	SNW1	red	-0.5962378
ENSG00000028528	SNX1	brown	-0.6363892
ENSG00000086300	SNX10	blue	0.67978575
ENSG00000002919	SNX11	grey	0.15930901
ENSG00000147164	SNX12	darkgrey	0.50632821
ENSG00000071189	SNX13	grey	0.17826052
ENSG00000135317	SNX14	grey	-0.0515657
ENSG00000110025	SNX15	grey	-0.1493208
ENSG00000178996	SNX18	brown	-0.6449004
ENSG00000235571	SNX18P14	grey	0.3254287
ENSG00000225015	SNX18P15	grey	0.21331658
ENSG00000225345	SNX18P3	floralwhite	0.47820276
ENSG00000234416	SNX18P4	grey	0.23657103
ENSG00000224185	SNX18P9	grey	0.05088201
ENSG00000120451	SNX19	grey	0.17638284
ENSG00000205302	SNX2	salmon	0.52309967
ENSG00000124104	SNX21	grey	0.1425334
ENSG00000157734	SNX22	turquoise	0.21233597
ENSG00000064652	SNX24	turquoise	0.46418059
ENSG00000109762	SNX25	turquoise	-0.4132034
ENSG00000143376	SNX27	grey	-0.1073366
ENSG00000048471	SNX29	turquoise	0.46842459
ENSG00000198106	SNX29P2	grey	0.165814
ENSG00000148158	SNX30	yellow	0.6382519
ENSG00000172803	SNX32	blue	0.70477428
ENSG00000173548	SNX33	turquoise	0.715536
ENSG00000089006	SNX5	turquoise	0.7042232
ENSG00000129515	SNX6	green	0.87415242
ENSG00000106266	SNX8	grey	-0.11013
ENSG00000130340	SNX9	yellow	0.53398035
ENSG00000057252	SOAT1	royalblue	0.4705865
ENSG00000112320	SOBP	grey	-0.2301159
ENSG00000120833	SOCS2	blue	-0.5368855
ENSG00000180008	SOCS4	brown	-0.7548326
ENSG00000171150	SOCS5	brown	-0.7776251
ENSG00000174111	SOCS7	grey	0.19271125
ENSG00000142168	SOD1	salmon	0.73168318
ENSG00000112096	SOD2	pink	0.65797455
ENSG00000109610	SOD3	turquoise	0.39254512
ENSG00000149639	SOGA1	turquoise	0.58077759

ENSG00000168502	SOGA2	grey	0.07599673
ENSG00000159140	SON	lightcyan	-0.6583161
ENSG00000095637	SORBS1	red	-0.3808478
ENSG00000154556	SORBS2	darkorange	0.5368833
ENSG00000120896	SORBS3	brown	-0.4544205
ENSG00000184985	SORCS2	grey	0.10863603
ENSG00000156395	SORCS3	blue	0.7399461
ENSG00000226387	SORCS3-AS1	grey	0.24498375
ENSG00000140263	SORD	blue	0.34129937
ENSG00000137642	SORL1	turquoise	0.46401339
ENSG00000134243	SORT1	yellow	0.79283722
ENSG00000115904	SOS1	grey	-0.0931034
ENSG00000229692	SOS1-IT1	grey	0.28068466
ENSG00000100485	SOS2	yellow	0.74168621
ENSG00000198944	SOWAHA	magenta	0.7247856
ENSG00000198142	SOWAHC	turquoise	0.33746496
ENSG00000182968	SOX1	yellowgreen	0.59805913
ENSG00000100146	SOX10	yellow	0.72030905
ENSG00000176887	SOX11	grey	-0.2762457
ENSG00000177732	SOX12	grey	-0.076771
ENSG00000143842	SOX13	turquoise	0.58377382
ENSG00000129194	SOX15	grey	0.14474817
ENSG00000164736	SOX17	tan	0.67744187
ENSG00000203883	SOX18	tan	0.58035316
ENSG00000181449	SOX2	turquoise	0.63094461
ENSG00000242808	SOX2-OT	yellow	0.72106869
ENSG00000125285	SOX21	turquoise	0.76593971
ENSG00000227640	SOX21-AS1	turquoise	0.70362623
ENSG00000134532	SOX5	turquoise	0.74711571
ENSG00000110693	SOX6	yellowgreen	0.78377876
ENSG00000171056	SOX7	tan	0.77613232
ENSG00000005513	SOX8	yellow	0.67784328
ENSG00000125398	SOX9	turquoise	0.87818951
ENSG00000234899	SOX9-AS1	black	-0.6041716
ENSG00000185591	SP1	blue	-0.8380672
ENSG00000067066	SP100	royalblue	0.71804788
ENSG00000135899	SP110	royalblue	0.70347363
ENSG00000185404	SP140L	grey	0.16363105
ENSG00000167182	SP2	brown	-0.7004723
ENSG00000172845	SP3	brown	-0.7068974
ENSG00000105866	SP4	grey	-0.0171593
ENSG00000217236	SP9	magenta	0.58863345
ENSG00000236797	SPA17P1	grey	0.2717819
ENSG00000182310	SPACA6P	darkgrey	0.48915917
ENSG00000104450	SPAG1	grey	-0.0734799
ENSG00000144451	SPAG16	turquoise	0.39483269
ENSG00000061656	SPAG4	grey	0.32161448
ENSG00000076382	SPAG5	grey	0.30475864

ENSG00000091640	SPAG7	brown	0.69688124
ENSG00000008294	SPAG9	brown	-0.5886485
ENSG00000113140	SPARC	turquoise	0.51638963
ENSG00000152583	SPARCL1	turquoise	0.49997366
ENSG00000122432	SPATA1	grey	0.0791588
ENSG00000182957	SPATA13	yellow	0.62341238
ENSG00000227213	SPATA13-AS1	grey	0.40655423
ENSG00000158480	SPATA2	grey	0.20327328
ENSG00000006282	SPATA20	grey	0.18759788
ENSG00000170469	SPATA24	grey	0.19434133
ENSG00000149634	SPATA25	grey	0.19546009
ENSG00000158792	SPATA2L	grey	0.16070293
ENSG00000167523	SPATA33	grey	-0.032013
ENSG00000145375	SPATA5	darkgrey	0.70226839
ENSG00000171763	SPATA5L1	lightcyan	-0.6492203
ENSG00000132122	SPATA6	blue	-0.5654139
ENSG00000106686	SPATA6L	brown	0.4689159
ENSG00000042317	SPATA7	grey	-0.0936566
ENSG00000160284	SPATC1L	grey	0.15736645
ENSG00000123352	SPATS2	blue	0.66623619
ENSG00000196141	SPATS2L	black	-0.6267711
ENSG00000114902	SPCS1	turquoise	0.3720442
ENSG00000118363	SPCS2	mediumpurp	0.68187866
ENSG00000129128	SPCS3	grey	-0.1139964
ENSG00000040275	SPDL1	green	0.84921848
ENSG00000163806	SPDYA	grey	0.29599442
ENSG00000136206	SPDYE1	grey	-0.0880461
ENSG00000205238	SPDYE2	grey	0.17190085
ENSG00000214300	SPDYE3	grey	-0.2393588
ENSG00000166667	SPDYE6	grey	0.15298395
ENSG00000128487	SPECC1	green	0.84198267
ENSG00000152582	SPEF2	purple	0.85218913
ENSG00000072195	SPEG	grey60	0.57502509
ENSG00000258484	SPESP1	grey	0.212985
ENSG00000104133	SPG11	blue	-0.514943
ENSG00000133104	SPG20	green	0.64705879
ENSG00000120664	SPG20OS	yellow	0.63911008
ENSG00000090487	SPG21	grey	-0.1409654
ENSG00000197912	SPG7	brown	0.68038433
ENSG00000063176	SPHK2	grey	0.40014055
ENSG00000153820	SPHKAP	grey	0.09199884
ENSG00000066336	SPI1	grey	-0.3259119
ENSG00000163611	SPICE1	turquoise	-0.3354998
ENSG00000164808	SPIDR	grey	-0.0115696
ENSG00000106723	SPIN1	brown	-0.2815093
ENSG00000147059	SPIN2A	grey	-0.03264
ENSG00000186787	SPIN2B	greenyellow	0.51851575
ENSG00000204271	SPIN3	grey	0.01406573

ENSG00000186767	SPIN4	grey	-0.2892018
ENSG00000214510	SPINK13	yellow	0.53272027
ENSG00000229453	SPINK8	grey	0.51310565
ENSG00000134278	SPIRE1	turquoise	0.63640556
ENSG00000204991	SPIRE2	yellow	-0.6381304
ENSG00000183018	SPNS2	floralwhite	0.62051324
ENSG00000152377	SPOCK1	grey	0.16061376
ENSG00000107742	SPOCK2	brown	0.56135429
ENSG00000196104	SPOCK3	red	-0.7787263
ENSG00000152268	SPON1	turquoise	0.68423497
ENSG00000159674	SPON2	grey	0.1058461
ENSG00000121067	SPOP	turquoise	0.45143798
ENSG00000144228	SPOPL	green	0.82771344
ENSG00000072080	SPP2	grey	0.06402202
ENSG00000138600	SPPL2A	brown	-0.4681535
ENSG00000005206	SPPL2B	grey	-0.210989
ENSG00000157837	SPPL3	brown	0.63206182
ENSG00000116096	SPR	grey	-0.1874645
ENSG00000166068	SPRED1	turquoise	0.6427309
ENSG00000198369	SPRED2	red	0.53092668
ENSG00000188766	SPRED3	grey	0.05211965
ENSG00000010072	SPRTN	turquoise	0.44219899
ENSG00000164056	SPRY1	turquoise	0.48486221
ENSG00000136158	SPRY2	turquoise	0.60636872
ENSG00000168939	SPRY3	yellow	-0.3676317
ENSG00000187678	SPRY4	lightcyan	0.57731002
ENSG00000167778	SPRYD3	turquoise	-0.5284752
ENSG00000176422	SPRYD4	grey	0.18039604
ENSG00000123178	SPRYD7	purple	0.68192293
ENSG00000171621	SPSB1	yellow	0.72264217
ENSG00000111671	SPSB2	grey	0.27726789
ENSG00000197694	SPTAN1	brown	0.69653749
ENSG00000070182	SPTB	blue	0.61400566
ENSG00000115306	SPTBN1	darkmagenta	-0.5697817
ENSG00000173898	SPTBN2	blue	0.71485826
ENSG00000160460	SPTBN4	turquoise	-0.5538043
ENSG00000090054	SPTLC1	grey	0.21642335
ENSG00000230397	SPTLC1P1	grey	0.24622528
ENSG00000172296	SPTLC3	grey	0.08328181
ENSG00000165389	SPTSSA	blue	-0.7223901
ENSG00000196542	SPTSSB	blue	0.36257842
ENSG00000104549	SQLE	turquoise	-0.6020982
ENSG00000137767	SQRDL	pink	0.76388668
ENSG00000161011	SQSTM1	blue	-0.6216492
ENSG00000213523	SRA1	brown	0.54702805
ENSG00000068784	SRBD1	grey	0.26403726
ENSG00000017373	SRCIN1	yellow	0.61419641
ENSG00000145545	SRD5A1	purple	0.61849146

ENSG00000128039	SRD5A3	grey	-0.0769851
ENSG00000249700	SRD5A3-AS1	grey	0.33174526
ENSG00000198911	SREBF2	turquoise	-0.4708913
ENSG00000153914	SREK1	darkgrey	0.6196866
ENSG00000153006	SREK1IP1	green	0.55809906
ENSG00000112658	SRF	grey	-0.2205465
ENSG00000151304	SRFBP1	lightgreen	0.39608313
ENSG00000196935	SRGAP1	black	0.73576548
ENSG00000163486	SRGAP2	cyan	0.62142318
ENSG00000196369	SRGAP2B	grey	-0.3730715
ENSG00000171943	SRGAP2C	grey	-0.0968376
ENSG00000196220	SRGAP3	red	0.71693221
ENSG00000228723	SRGAP3-AS2	grey	0.28374421
ENSG00000227929	SRGAP3-AS3	grey	0.17904563
ENSG00000122862	SRGN	pink	0.79607514
ENSG00000271386	SRGNP1	grey	0.00132808
ENSG00000075142	SRI	turquoise	0.55322273
ENSG00000116649	SRM	grey	0.31467501
ENSG00000258485	SRMP2	grey	0.30712946
ENSG00000140319	SRP14	grey	0.01724193
ENSG00000248508	SRP14-AS1	green	0.56570777
ENSG00000153037	SRP19	blue	0.37828331
ENSG00000167881	SRP68	brown	0.54454898
ENSG00000237911	SRP68P3	grey	0.29090386
ENSG00000174780	SRP72	green	-0.6669855
ENSG00000188451	SRP72P2	grey	0.29853438
ENSG00000143742	SRP9	turquoise	0.85532802
ENSG00000180581	SRP9P1	grey	0.14874558
ENSG00000096063	SRPK1	black	0.74029735
ENSG00000135250	SRPK2	grey	0.21278696
ENSG00000182934	SRPR	darkgrey	-0.5328219
ENSG00000101955	SRPX	turquoise	0.43663349
ENSG00000102359	SRPX2	grey	-0.0538768
ENSG00000167720	SRR	turquoise	0.39948142
ENSG00000100104	SRRD	grey	0.08041579
ENSG00000177679	SRRM3	red	-0.5645984
ENSG00000087087	SRRT	grey	-0.2345771
ENSG00000136450	SRSF1	lightgreen	0.85336792
ENSG00000188529	SRSF10	grey	0.06392388
ENSG00000154548	SRSF12	yellow	-0.6312293
ENSG00000161547	SRSF2	mediumpurp	0.505835
ENSG00000112081	SRSF3	turquoise	0.61383713
ENSG00000116350	SRSF4	darkgrey	0.70222934
ENSG00000100650	SRSF5	brown	-0.36678
ENSG00000115875	SRSF7	grey	-0.0200097
ENSG00000180771	SRSF8	grey	-0.0011512
ENSG00000111786	SRSF9	grey	-0.1425325
ENSG00000141380	SS18	turquoise	0.83681611

ENSG00000008324	SS18L2	salmon	0.49083402
ENSG00000266201	SS18L2P2	yellow	0.45297714
ENSG00000138385	SSB	mediumpurp	0.68212371
ENSG00000106028	SSBP1	brown	0.63103672
ENSG00000145687	SSBP2	grey	-0.3349266
ENSG00000157216	SSBP3	blue	0.57656211
ENSG00000130511	SSBP4	turquoise	-0.4732236
ENSG00000179954	SSC5D	greenyellow	-0.5248673
ENSG00000138434	SSFA2	darkgrey	0.69378235
ENSG00000084112	SSH1	yellow	-0.4818907
ENSG00000141298	SSH2	royalblue	0.6724981
ENSG00000172830	SSH3	yellow	0.65450301
ENSG00000176101	SSNA1	grey	0.19864975
ENSG00000123096	SSPN	turquoise	0.74998192
ENSG00000163479	SSR2	brown	0.50531779
ENSG00000180879	SSR4	brown	0.49931336
ENSG00000149136	SSRP1	lightcyan	-0.7210455
ENSG00000173465	SSSCA1	grey	0.14047622
ENSG00000260233	SSSCA1-AS1	grey	0.05619637
ENSG00000157005	SST	darkmagenta	0.56511369
ENSG00000180616	SSTR2	purple	0.56627155
ENSG00000160075	SSU72	brown	0.74199747
ENSG00000217330	SSXP10	grey	0.33509254
ENSG00000100380	ST13	turquoise	0.42326532
ENSG00000243759	ST13P15	darkred	-0.5707156
ENSG00000147488	ST18	yellow	0.88973759
ENSG00000180953	ST20	grey	-0.0411618
ENSG00000008513	ST3GAL1	pink	0.58412654
ENSG00000157350	ST3GAL2	grey	0.18825637
ENSG00000126091	ST3GAL3	brown	0.66877924
ENSG00000110080	ST3GAL4	yellow	0.53852604
ENSG00000115525	ST3GAL5	brown	0.72912714
ENSG00000064225	ST3GAL6	black	0.65707561
ENSG00000239445	ST3GAL6-AS1	grey	0.29628596
ENSG00000166444	ST5	red	0.74159488
ENSG00000073849	ST6GAL1	cyan	0.77042183
ENSG00000144057	ST6GAL2	turquoise	0.43674992
ENSG00000238250	ST6GAL2-IT1	grey	0.2949445
ENSG00000070526	ST6GALNAC1	tan	0.72243836
ENSG00000184005	ST6GALNAC3	darkred	-0.6916403
ENSG00000136840	ST6GALNAC4	turquoise	0.68329244
ENSG00000233469	ST6GALNAC4P1	grey	0.26330792
ENSG00000117069	ST6GALNAC5	purple	0.66221008
ENSG00000160408	ST6GALNAC6	floralwhite	-0.6048478
ENSG00000004866	ST7	yellow	-0.5238627
ENSG00000226367	ST7-AS2	grey	0.31444486
ENSG00000214188	ST7-OT4	turquoise	0.6069576
ENSG00000007341	ST7L	grey	-0.1368003

ENSG00000111728	ST8SIA1	black	-0.5559651
ENSG00000177511	ST8SIA3	blue	0.6242617
ENSG00000113532	ST8SIA4	black	0.44572569
ENSG00000101638	ST8SIA5	grey	-0.1575432
ENSG00000148488	ST8SIA6	tan	0.51737403
ENSG00000010327	STAB1	royalblue	0.76999813
ENSG00000141750	STAC2	darkmagenta	0.64913865
ENSG00000118007	STAG1	blue	-0.5678509
ENSG00000101972	STAG2	blue	-0.7080587
ENSG00000066923	STAG3	red	-0.5124864
ENSG00000205583	STAG3L1	grey	0.24930697
ENSG00000160828	STAG3L2	grey	0.27587538
ENSG00000174353	STAG3L3	grey	0.13245944
ENSG00000106610	STAG3L4	grey	0.06836923
ENSG00000242294	STAG3L5P	grey	0.26463111
ENSG00000136738	STAM	grey	0.11094124
ENSG00000115145	STAM2	turquoise	0.7038464
ENSG00000124356	STAMBP	yellow	0.46476542
ENSG00000138134	STAMBPL1	brown	0.63303123
ENSG00000178078	STAP2	grey	-0.0381777
ENSG00000147465	STAR	purple	0.68327337
ENSG00000214530	STARD10	black	0.50044733
ENSG00000133121	STARD13	yellow	0.62936496
ENSG00000236581	STARD13-AS	yellow	0.71806045
ENSG00000230300	STARD13-IT1	grey	0.41586314
ENSG00000010270	STARD3NL	grey	-0.0008993
ENSG00000164211	STARD4	grey	0.16293052
ENSG00000246859	STARD4-AS1	grey	0.17783061
ENSG00000084090	STARD7	turquoise	0.6176575
ENSG00000204685	STARD7-AS1	grey	0.31095693
ENSG00000130052	STARD8	tan	0.8084224
ENSG00000159433	STARD9	yellow	0.65009732
ENSG00000115415	STAT1	grey	0.04898334
ENSG00000170581	STAT2	turquoise	-0.4064699
ENSG00000168610	STAT3	pink	0.89457805
ENSG00000138378	STAT4	purple	0.81578988
ENSG00000173757	STAT5B	blue	-0.7660581
ENSG00000166888	STAT6	grey	-0.2930609
ENSG00000124214	STAU1	grey	0.05702427
ENSG00000105889	STEAP1B	yellow	0.45825427
ENSG00000157214	STEAP2	grey	-0.004141
ENSG00000256762	STH	yellow	0.48133218
ENSG00000123473	STIL	grey	0.33396597
ENSG00000167323	STIM1	brown	0.50389539
ENSG00000109689	STIM2	turquoise	-0.6005755
ENSG00000072786	STK10	grey	-0.0148535
ENSG00000118046	STK11	brown	0.64476063
ENSG00000144589	STK11IP	brown	0.6767648

ENSG00000115661	STK16	grey	0.01599103
ENSG00000164543	STK17A	grey	-0.2361855
ENSG00000081320	STK17B	turquoise	0.70600032
ENSG00000204344	STK19	grey	0.08378079
ENSG00000115694	STK25	blue	0.54116449
ENSG00000104375	STK3	grey	0.30700994
ENSG00000196335	STK31	black	0.72881012
ENSG00000169302	STK32A	grey	0.02850426
ENSG00000165752	STK32C	turquoise	-0.6465681
ENSG00000130413	STK33	turquoise	0.53574706
ENSG00000125834	STK35	grey	0.1743305
ENSG00000163482	STK36	grey	-0.0342074
ENSG00000112079	STK38	yellow	0.52402759
ENSG00000211455	STK38L	turquoise	0.47295492
ENSG00000198648	STK39	turquoise	-0.3903832
ENSG00000101109	STK4	grey	-0.0445306
ENSG00000196182	STK40	pink	0.49954024
ENSG00000117632	STMN1	turquoise	-0.4666159
ENSG00000104435	STMN2	grey60	0.6352655
ENSG00000197457	STMN3	blue	0.52840416
ENSG00000015592	STMN4	turquoise	-0.4085859
ENSG00000148175	STOM	turquoise	0.7864448
ENSG00000067221	STOML1	brown	0.69631268
ENSG00000165283	STOML2	darkred	0.70919401
ENSG00000140022	STON2	turquoise	0.7092411
ENSG00000165730	STOX1	turquoise	0.47665735
ENSG00000173320	STOX2	turquoise	-0.4752728
ENSG00000001460	STPG1	blue	0.53893981
ENSG00000169689	STRA13	grey	0.06852969
ENSG00000266173	STRADA	red	-0.6725693
ENSG00000082146	STRADB	lightcyan	0.70652421
ENSG00000242866	STRC	grey	0.12854069
ENSG00000128578	STRIP2	grey60	0.53076939
ENSG00000115808	STRN	red	-0.8372017
ENSG00000196792	STRN3	grey	0.10860343
ENSG00000090372	STRN4	turquoise	-0.5127312
ENSG00000101846	STS	blue	0.54145948
ENSG00000134910	STT3A	turquoise	0.39428305
ENSG00000163527	STT3B	grey	-0.1629465
ENSG00000104915	STX10	grey	0.12828476
ENSG00000117758	STX12	turquoise	0.5430156
ENSG00000124222	STX16	blue	-0.3890323
ENSG00000136874	STX17	green	0.77122483
ENSG00000168818	STX18	grey	-0.2103328
ENSG00000247708	STX18-AS1	grey	-0.0467654
ENSG00000248221	STX18-IT1	grey	0.25669863
ENSG00000099365	STX1B	blue	0.76991242
ENSG00000111450	STX2	turquoise	0.46288736

ENSG00000166900	STX3	grey	0.23107522
ENSG00000103496	STX4	grey	-0.19674
ENSG00000162236	STX5	turquoise	-0.5751916
ENSG00000135823	STX6	lightcyan	-0.5413294
ENSG00000079950	STX7	lightcyan	0.68146493
ENSG00000170310	STX8	brown	0.76392616
ENSG00000136854	STXBP1	blue	0.84855446
ENSG00000116266	STXBP3	turquoise	0.74026251
ENSG00000166263	STXBP4	grey	0.16938532
ENSG00000164506	STXBP5	turquoise	-0.4827402
ENSG00000233452	STXBP5-AS1	grey60	0.59996078
ENSG00000168952	STXBP6	grey	0.05699923
ENSG00000060140	STYK1	brown	0.65822639
ENSG00000198252	STYX	lightcyan	0.63731948
ENSG00000127952	STYXL1	brown	0.4550011
ENSG00000227203	SUB1P1	grey	0.29109659
ENSG00000136143	SUCLA2	grey	-0.0073407
ENSG00000227848	SUCLA2-AS1	grey	0.31837458
ENSG00000229593	SUCLA2P3	blue	-0.3797471
ENSG00000163541	SUCLG1	salmon	0.7521758
ENSG00000172340	SUCLG2	turquoise	0.81375088
ENSG00000094975	SUCO	grey	-0.1117348
ENSG00000111707	SUDS3	blue	0.37692521
ENSG00000107882	SUFU	turquoise	0.51084862
ENSG00000175600	SUGCT	grey	0.37714232
ENSG00000105705	SUGP1	brown	0.76500726
ENSG00000064607	SUGP2	grey	-0.3034019
ENSG00000213842	SUGT1P2	grey	0.15434704
ENSG00000239827	SUGT1P3	grey	0.14396966
ENSG00000196502	SULT1A1	yellow	-0.3754115
ENSG00000197165	SULT1A2	grey	0.00033034
ENSG00000198075	SULT1C4	turquoise	0.72835192
ENSG00000130540	SULT4A1	blue	0.81602902
ENSG00000144455	SUMF1	grey	0.0104992
ENSG00000116030	SUMO1	turquoise	0.39199974
ENSG00000235082	SUMO1P3	grey	0.21376794
ENSG00000188612	SUMO2	mediumpurp	0.72108369
ENSG00000271096	SUMO2P14	grey	0.09816779
ENSG00000228976	SUMO2P8	grey	0.3814409
ENSG00000184900	SUMO3	greenyellow	0.59666587
ENSG00000177688	SUMO4	grey	0.32148043
ENSG00000164828	SUN1	grey	-0.1660612
ENSG00000100242	SUN2	yellow	0.85529533
ENSG00000139531	SUOX	turquoise	0.48625468
ENSG00000092201	SUPT16H	turquoise	-0.3855213
ENSG00000102710	SUPT20H	blue	-0.284184
ENSG00000196284	SUPT3H	grey	0.13001424
ENSG00000213246	SUPT4H1	grey	-0.0444482

ENSG00000196235	SUPT5H	grey	-0.0088656
ENSG00000109111	SUPT6H	grey	0.12966612
ENSG00000119760	SUPT7L	turquoise	-0.4973623
ENSG00000156502	SUPV3L1	grey	0.01240198
ENSG00000148290	SURF1	grey	-0.0982922
ENSG00000148291	SURF2	grey	-0.2129371
ENSG00000148248	SURF4	yellow	0.46501238
ENSG00000148296	SURF6	darkgrey	0.66229174
ENSG00000106868	SUSD1	blue	0.80085645
ENSG00000157303	SUSD3	cyan	0.74351265
ENSG00000143502	SUSD4	blue	0.85727975
ENSG00000173705	SUSD5	lightcyan	0.65561097
ENSG00000101945	SUV39H1	grey	0.19781929
ENSG00000152455	SUV39H2	grey	0.09997692
ENSG00000110066	SUV420H1	blue	-0.5934357
ENSG00000264538	SUZ12P	turquoise	-0.3649933
ENSG00000159164	SV2A	blue	0.91345432
ENSG00000185518	SV2B	brown	0.40188264
ENSG00000165124	SVEP1	yellow	0.70719462
ENSG00000197321	SVIL	grey	0.17488266
ENSG00000198168	SVIP	grey	-0.259089
ENSG00000166111	SVOP	turquoise	-0.7391094
ENSG00000133789	SWAP70	blue	-0.5926227
ENSG00000175854	SWI5	brown	0.4871163
ENSG00000173928	SWSAP1	grey	0.34766593
ENSG00000116668	SWT1	green	0.45194627
ENSG00000169895	SYAP1	grey	0.26033383
ENSG00000147642	SYBU	grey	-0.0541486
ENSG00000171772	SYCE1	grey	0.05947313
ENSG00000196074	SYCP2	brown	-0.5564586
ENSG00000153157	SYCP2L	grey	0.25289818
ENSG00000139351	SYCP3	grey	0.15405308
ENSG00000105137	SYDE1	turquoise	0.49247411
ENSG00000097096	SYDE2	grey	0.01416053
ENSG00000117614	SYF2	grey	-0.0992181
ENSG00000233148	SYF2P2	grey	0.31864595
ENSG00000165025	SYK	cyan	0.90022253
ENSG00000125755	SYMPK	turquoise	-0.7030735
ENSG00000008056	SYN1	blue	0.82643563
ENSG00000157152	SYN2	purple	0.81942024
ENSG00000185666	SYN3	grey	-0.0508774
ENSG00000162520	SYNC	turquoise	0.72054295
ENSG00000135316	SYNCRIP	brown	-0.7158796
ENSG00000101463	SYNDIG1	black	0.75616904
ENSG00000183379	SYNDIG1L	grey	0.00330085
ENSG00000131018	SYNE1	grey	-0.0075384
ENSG00000054654	SYNE2	tan	0.73998355
ENSG00000176438	SYNE3	yellow	0.55243124

ENSG00000197283	SYNGAP1	blue	0.79412441
ENSG00000100321	SYNGR1	purple	0.81041548
ENSG00000108639	SYNGR2	yellow	0.73368604
ENSG00000127561	SYNGR3	blue	0.83663937
ENSG00000159082	SYNJ1	blue	0.91537706
ENSG00000078269	SYNJ2	yellow	0.83268753
ENSG00000233496	SYNJ2-IT1	grey	0.18463773
ENSG00000213463	SYNJ2BP	brown	-0.8182593
ENSG00000182253	SYNM	grey	0.005241
ENSG00000172403	SYNPO2	yellowgreen	0.61991571
ENSG00000163630	SYNPR	purple	0.84561129
ENSG00000241359	SYNPR-AS1	grey	0.26957137
ENSG00000006114	SYNRG	green	-0.5927222
ENSG00000102003	SYP	blue	0.74271007
ENSG00000008282	SYPL1	turquoise	0.66839611
ENSG00000143028	SYPL2	turquoise	0.47934212
ENSG00000204070	SYS1	grey	0.37393986
ENSG00000254806	SYS1-DBNDD2	yellow	0.61538262
ENSG00000067715	SYT1	blue	0.8030892
ENSG00000132718	SYT11	grey	0.13626158
ENSG00000173227	SYT12	grey	-0.1389425
ENSG00000019505	SYT13	blue	0.62042353
ENSG00000143469	SYT14	salmon	-0.4451797
ENSG00000204176	SYT15	turquoise	-0.4755994
ENSG00000139973	SYT16	turquoise	-0.6317819
ENSG00000103528	SYT17	red	0.6088297
ENSG00000143858	SYT2	grey60	0.39091795
ENSG00000213023	SYT3	blue	0.62927443
ENSG00000132872	SYT4	blue	0.72523193
ENSG00000129990	SYT5	blue	0.73664368
ENSG00000134207	SYT6	turquoise	-0.402719
ENSG00000011347	SYT7	blue	0.61213782
ENSG00000170743	SYT9	darkmagenta	-0.6328337
ENSG00000137501	SYTL2	grey	-0.1160889
ENSG00000102362	SYTL4	turquoise	0.3747189
ENSG00000147041	SYTL5	magenta	0.76911949
ENSG00000162298	SYVN1	yellow	0.30986092
ENSG00000198198	SZT2	grey	-0.1238451
ENSG00000055208	TAB2	grey	-0.2036642
ENSG00000157625	TAB3	grey	0.22138031
ENSG00000006128	TAC1	magenta	0.85298047
ENSG00000166863	TAC3	magenta	0.41143937
ENSG00000176358	TAC4	grey	0.16459021
ENSG00000147526	TACC1	blue	-0.7854005
ENSG00000138162	TACC2	brown	0.82261962
ENSG00000013810	TACC3	grey	0.11595949
ENSG00000136463	TACO1	darkred	0.62998698
ENSG00000115353	TACR1	grey	-0.0560982

ENSG00000075073	TACR2	grey	0.02807082
ENSG00000152382	TADA1	grey	-0.3029208
ENSG00000108264	TADA2A	blue	0.47836296
ENSG00000173011	TADA2B	blue	-0.4342439
ENSG00000147133	TAF1	darkgrey	0.68481624
ENSG00000166337	TAF10	grey	-0.0784083
ENSG00000064995	TAF11	brown	0.57789762
ENSG00000120656	TAF12	grey	0.14999807
ENSG00000197780	TAF13	brown	-0.660018
ENSG00000172660	TAF15	darkgrey	0.68528066
ENSG00000143498	TAF1A	grey	0.14235797
ENSG00000115750	TAF1B	grey	-0.2367154
ENSG00000103168	TAF1C	grey	-0.190424
ENSG00000166012	TAF1D	greenyellow	0.58470506
ENSG00000064313	TAF2	grey	0.31698752
ENSG00000165632	TAF3	green	0.73748139
ENSG00000130699	TAF4	grey	-0.0247269
ENSG00000141384	TAF4B	turquoise	-0.602396
ENSG00000148835	TAF5	yellow	0.34439806
ENSG00000135801	TAF5L	grey	0.18590993
ENSG00000106290	TAF6	grey	0.26066493
ENSG00000162227	TAF6L	grey	0.02671986
ENSG00000102387	TAF7L	brown	0.47419635
ENSG00000137413	TAF8	grey	0.23431038
ENSG00000085231	TAF9	yellow	-0.6619339
ENSG00000187325	TAF9B	turquoise	0.43292242
ENSG00000213871	TAF9BP1	grey	0.38794018
ENSG00000267692	TAF9P3	grey	0.38532984
ENSG00000164691	TAGAP	cyan	0.46733883
ENSG00000149591	TAGLN	grey	-0.0722894
ENSG00000158710	TAGLN2	pink	0.71321546
ENSG00000253676	TAGLN2P1	grey	0.44750325
ENSG00000144834	TAGLN3	blue	0.69602338
ENSG00000162367	TAL1	cyan	0.60646857
ENSG00000177156	TALDO1	brown	0.44419708
ENSG00000144559	TAMM41	turquoise	-0.4667452
ENSG00000170921	TANC2	blue	0.69434976
ENSG00000183597	TANGO2	magenta	-0.5003496
ENSG00000136560	TANK	grey	-0.2067318
ENSG00000160551	TAOK1	lightgreen	0.64097467
ENSG00000149930	TAOK2	turquoise	-0.4785116
ENSG00000135090	TAOK3	blue	0.37449065
ENSG00000168394	TAP1	pink	0.8213991
ENSG00000204267	TAP2	blue	-0.5784251
ENSG00000231925	TAPBP	grey	0.24522928
ENSG00000139192	TAPBPL	grey	0.10320032
ENSG00000204261	TAPSAR1	blue	-0.4321448
ENSG00000169762	TAPT1	turquoise	0.75467394

ENSG00000263327	TAPT1-AS1	grey	0.0553507
ENSG00000059588	TARBP1	blue	0.56177517
ENSG00000139546	TARBP2	grey	0.05407007
ENSG00000120948	TARDBP	brown	-0.6204605
ENSG00000113407	TARS	lightcyan	-0.502445
ENSG00000143374	TARS2	blue	0.45222962
ENSG00000185418	TARSL2	grey	0.00691596
ENSG00000256682	TAS2R12	grey	0.35850088
ENSG00000212128	TAS2R13	grey	0.17360438
ENSG00000256981	TAS2R18	yellow	0.65717325
ENSG00000255837	TAS2R20	grey	0.34097198
ENSG00000127362	TAS2R3	grey	0.31609647
ENSG00000256188	TAS2R30	lightgreen	0.53990422
ENSG00000256436	TAS2R31	grey	0.18245706
ENSG00000236398	TAS2R39	yellow	0.59277233
ENSG00000127364	TAS2R4	grey	0.39911661
ENSG00000221937	TAS2R40	yellow	0.62115229
ENSG00000127366	TAS2R5	grey	0.17507595
ENSG00000212126	TAS2R50	grey	0.19539107
ENSG00000270923	TAS2R6	grey	0.32792462
ENSG00000256019	TAS2R63P	grey	0.21114348
ENSG00000089123	TASP1	blue	0.56719537
ENSG00000147687	TATDN1	grey	-0.2031781
ENSG00000234369	TATDN1P1	grey	0.38771059
ENSG00000157014	TATDN2	turquoise	-0.4900879
ENSG00000218226	TATDN2P2	grey	0.2003298
ENSG00000203705	TATDN3	grey	-0.0577362
ENSG00000106052	TAX1BP1	red	0.53193912
ENSG00000213977	TAX1BP3	grey	-0.0823097
ENSG00000065882	TBC1D1	grey	0.14581146
ENSG00000099992	TBC1D10A	greenyellow	-0.4888081
ENSG00000169221	TBC1D10B	grey	-0.0726481
ENSG00000175463	TBC1D10C	grey	-0.0720627
ENSG00000108239	TBC1D12	darkred	-0.7770105
ENSG00000107021	TBC1D13	turquoise	-0.4726359
ENSG00000132405	TBC1D14	grey	-0.2166811
ENSG00000121749	TBC1D15	blue	-0.3796762
ENSG00000167291	TBC1D16	grey	0.28096821
ENSG00000104946	TBC1D17	grey	-0.2111444
ENSG00000109680	TBC1D19	grey	0.21750246
ENSG00000095383	TBC1D2	yellow	0.53507601
ENSG00000125875	TBC1D20	turquoise	0.77186372
ENSG00000054611	TBC1D22A	turquoise	-0.5632146
ENSG00000065491	TBC1D22B	turquoise	-0.5841729
ENSG00000036054	TBC1D23	turquoise	0.51417077
ENSG00000162065	TBC1D24	red	0.58416221
ENSG00000068354	TBC1D25	blue	0.56463262
ENSG00000167202	TBC1D2B	grey	-0.0012535

ENSG00000111490	TBC1D30	grey60	0.71326016
ENSG00000156787	TBC1D31	grey	0.1931636
ENSG00000146350	TBC1D32	grey	-0.1349489
ENSG00000136111	TBC1D4	tan	0.54111689
ENSG00000131374	TBC1D5	grey	-0.0118154
ENSG00000145979	TBC1D7	brown	0.54536608
ENSG00000204634	TBC1D8	grey	0.19219944
ENSG00000109436	TBC1D9	blue	0.68156471
ENSG00000197226	TBC1D9B	grey	0.12814268
ENSG00000171530	TBCA	green	-0.5074195
ENSG00000235829	TBCAP2	lightcyan	0.5427369
ENSG00000105254	TBCB	black	-0.4971944
ENSG00000124659	TBCC	brown	0.54777629
ENSG00000113838	TBCCD1	blue	-0.4839931
ENSG00000141556	TBCD	brown	0.75773149
ENSG00000116957	TBCE	grey	0.29222621
ENSG00000154114	TBCEL	lightgreen	0.74975245
ENSG00000145348	TBCK	grey	-0.0840455
ENSG00000101849	TBL1X	turquoise	0.55526393
ENSG00000177565	TBL1XR1	grey	0.14441346
ENSG00000231310	TBL1XR1-AS1	grey	0.20321641
ENSG00000106638	TBL2	grey	0.03717749
ENSG00000183751	TBL3	green	-0.7249219
ENSG00000112592	TBP	grey	0.02791121
ENSG00000028839	TBPL1	grey	0.09864748
ENSG00000154144	TBRG1	red	-0.6446333
ENSG00000136270	TBRG4	brown	0.60236443
ENSG00000112837	TBX18	grey	0.08651315
ENSG00000059377	TBXAS1	cyan	0.85948002
ENSG00000179152	TCAIM	turquoise	0.49076516
ENSG00000173991	TCAP	grey	0.00264337
ENSG00000187735	TCEA1	yellow	0.59038257
ENSG00000230409	TCEA1P2	grey	0.15467231
ENSG00000236184	TCEA1P4	grey	0.16711579
ENSG00000204219	TCEA3	black	-0.5877931
ENSG00000172465	TCEAL1	mediumpurp	0.66524949
ENSG00000196507	TCEAL3	grey	-0.1026591
ENSG00000133142	TCEAL4	brown	0.42063149
ENSG00000204065	TCEAL5	blue	0.64179984
ENSG00000204071	TCEAL6	black	-0.5756484
ENSG00000180964	TCEAL8	turquoise	0.72388863
ENSG00000176896	TCEANC	grey	0.17552153
ENSG00000116205	TCEANC2	green	0.75598659
ENSG00000154582	TCEB1	brown	0.63135357
ENSG00000241975	TCEB1P19	grey	0.14573913
ENSG00000255006	TCEB1P22	grey	0.18854293
ENSG00000103363	TCEB2	brown	0.4256324
ENSG00000234152	TCEB2P1	grey	0.45158129

ENSG00000011007	TCEB3	grey	0.23825084
ENSG00000113649	TCERG1	blue	0.50576705
ENSG00000140262	TCF12	yellow	0.67451672
ENSG00000100207	TCF20	darkorange	0.51228557
ENSG00000141002	TCF25	turquoise	-0.8047216
ENSG00000071564	TCF3	blue	-0.5717676
ENSG00000196628	TCF4	turquoise	0.71172343
ENSG00000081059	TCF7	brown	-0.7075313
ENSG00000152284	TCF7L1	turquoise	0.46408112
ENSG00000231134	TCF7L1-IT1	grey	0.28647433
ENSG00000148737	TCF7L2	red	0.69601505
ENSG00000101190	TCFL5	green	0.72658767
ENSG00000139437	TCHP	blue	-0.4053817
ENSG00000110719	TCIRG1	royalblue	0.6299933
ENSG00000185339	TCN2	tan	0.68330952
ENSG00000070814	TCOF1	turquoise	-0.4472692
ENSG00000242220	TCP10L	grey	0.26941162
ENSG00000176148	TCP11L1	darkorange	0.54833283
ENSG00000166046	TCP11L2	yellow	0.73260445
ENSG00000145022	TCTA	brown	-0.5617394
ENSG00000146221	TCTE1	purple	0.53958477
ENSG00000213123	TCTEX1D2	brown	0.66575112
ENSG00000204852	TCTN1	grey	-0.1846426
ENSG00000168778	TCTN2	yellow	-0.4442311
ENSG00000119977	TCTN3	grey	0.36758063
ENSG00000139372	TDG	grey	0.19822736
ENSG00000241438	TDGF1P6	brown	-0.3757561
ENSG00000042088	TDP1	grey	-0.019609
ENSG00000111802	TDP2	grey	-0.1506974
ENSG00000173809	TDRD12	lightcyan	-0.6350301
ENSG00000083544	TDRD3	red	-0.6184698
ENSG00000162782	TDRD5	turquoise	-0.428626
ENSG00000180113	TDRD6	grey	-0.0020502
ENSG00000196116	TDRD7	blue	-0.3790855
ENSG00000156414	TDRD9	salmon	0.53579951
ENSG00000182134	TDRKH	turquoise	-0.4112481
ENSG00000180190	TDRP	brown	-0.3588281
ENSG00000187079	TEAD1	turquoise	0.92194065
ENSG00000074219	TEAD2	tan	0.51381553
ENSG00000007866	TEAD3	pink	0.54956262
ENSG00000205356	TECPR1	brown	0.71467831
ENSG00000196663	TECPR2	yellow	0.50964485
ENSG00000099797	TECR	turquoise	0.3633881
ENSG00000235043	TECRP1	grey	0.31353596
ENSG00000109927	TECTA	black	0.7625342
ENSG00000167074	TEF	grey	-0.269249
ENSG00000172171	TEFM	grey	-0.0176333
ENSG00000120156	TEK	tan	0.63166261

ENSG00000188681	TEKT4P2	grey	0.06706445
ENSG00000100726	TELO2	darkred	0.52198757
ENSG00000111077	TENC1	green	0.74609656
ENSG00000145934	TENM2	purple	0.82423099
ENSG00000218336	TENM3	purple	0.83591171
ENSG00000129566	TEP1	darkorange	-0.5194608
ENSG00000147601	TERF1	grey	-0.0562802
ENSG00000132604	TERF2	blue	0.47140225
ENSG00000088992	TESC	magenta	0.77525802
ENSG00000107140	TESK1	grey	0.21638294
ENSG00000070759	TESK2	yellow	0.64787557
ENSG00000135426	TESPA1	black	0.83042461
ENSG00000138336	TET1	turquoise	0.38824192
ENSG00000168769	TET2	lightcyan	0.42075256
ENSG00000251586	TET2-AS1	grey	0.37108747
ENSG00000187605	TET3	grey	0.05634037
ENSG00000136891	TEX10	grey	-0.0991228
ENSG00000170925	TEX13B	grey	0.36283534
ENSG00000133863	TEX15	black	0.5610797
ENSG00000136478	TEX2	brown	-0.669524
ENSG00000144043	TEX261	turquoise	0.61946461
ENSG00000164081	TEX264	green	-0.4481791
ENSG00000153495	TEX29	grey	0.15793469
ENSG00000151287	TEX30	grey	0.26611677
ENSG00000091513	TF	yellow	0.73242387
ENSG00000108064	TFAM	turquoise	0.66762737
ENSG00000116819	TFAP2E	grey	-0.0144441
ENSG00000029639	TFB1M	grey	0.13980448
ENSG00000162851	TFB2M	grey	0.27363882
ENSG00000135457	TFCP2	green	0.54751291
ENSG00000198176	TFDP1	turquoise	0.73143158
ENSG00000068323	TFE3	grey	0.1224294
ENSG00000112561	TFEB	yellow	0.83001383
ENSG00000114354	TFG	grey	0.11987885
ENSG00000100109	TFIP11	green	-0.5357737
ENSG00000242337	TFP1	grey	0.13387887
ENSG00000003436	TFPI	brown	-0.3204891
ENSG00000105619	TFPT	yellow	-0.2995009
ENSG00000088451	TGDS	turquoise	0.36599494
ENSG00000163235	TGFA	yellow	0.75473592
ENSG00000105329	TGFB1	royalblue	0.76035311
ENSG00000092969	TGFB2	turquoise	0.84648323
ENSG00000119699	TGFB3	green	0.85969514
ENSG00000106799	TGFBR1	royalblue	0.63052891
ENSG00000163513	TGFBR2	tan	0.81168088
ENSG00000069702	TGFBR3	turquoise	0.7008847
ENSG00000260001	TGFBR3L	brown	0.38661548
ENSG00000135966	TGFBRAP1	brown	0.33820306

ENSG00000177426	TGIF1	grey	0.13580653
ENSG00000118707	TGIF2	brown	-0.4206358
ENSG00000198959	TGM2	tan	0.83455412
ENSG00000152291	TGOLN2	turquoise	0.37915867
ENSG00000137574	TGS1	green	0.52134607
ENSG00000115970	THADA	grey	0.20858598
ENSG00000131931	THAP1	turquoise	0.46010757
ENSG00000129028	THAP10	grey	0.1414487
ENSG00000168286	THAP11	grey	-0.3095845
ENSG00000041988	THAP3	green	-0.3575242
ENSG00000176946	THAP4	grey	0.21943746
ENSG00000177683	THAP5	turquoise	0.51416978
ENSG00000224324	THAP5P1	grey	0.15246472
ENSG00000174796	THAP6	turquoise	0.40446787
ENSG00000184436	THAP7	grey	0.17965502
ENSG00000161277	THAP8	grey	0.11014489
ENSG00000168152	THAP9	yellow	0.49028369
ENSG00000251022	THAP9-AS1	grey	-0.0002974
ENSG00000178726	THBD	pink	0.6728474
ENSG00000137801	THBS1	pink	0.54334743
ENSG00000186340	THBS2	yellow	0.60040321
ENSG00000113296	THBS4	grey	0.00165439
ENSG00000159445	THEM4	grey	-0.0036501
ENSG00000196407	THEM5	green	0.43736683
ENSG00000130193	THEM6	grey	0.04803316
ENSG00000130775	THEMIS2	darkred	-0.5835766
ENSG00000113272	THG1L	grey	0.39726476
ENSG00000185875	THNSL1	yellowgreen	0.62274183
ENSG00000144115	THNSL2	grey	0.0582108
ENSG00000051596	THOC3	grey	-0.161989
ENSG00000100296	THOC5	turquoise	-0.46898
ENSG00000131652	THOC6	brown	0.39937071
ENSG00000163634	THOC7	blue	0.32363151
ENSG00000240549	THOC7-AS1	grey	0.15936676
ENSG00000172009	THOP1	blue	0.51781032
ENSG00000126351	THRA	brown	0.48101436
ENSG00000151090	THRB	grey	-0.2043617
ENSG00000224822	THRB-IT1	grey	0.23910809
ENSG00000136114	THSD1	turquoise	0.46691919
ENSG00000187720	THSD4	grey	0.47855728
ENSG00000259431	THTPA	turquoise	0.44574192
ENSG00000066654	THUMPD1	grey	-0.0775911
ENSG00000138050	THUMPD2	brown	0.36280217
ENSG00000134077	THUMPD3	grey	0.05471928
ENSG00000154096	THY1	blue	0.78916961
ENSG00000151500	THYN1	turquoise	-0.7106308
ENSG00000116001	TIA1	grey	-0.3619638
ENSG00000221995	TIAF1	yellow	0.55747184

ENSG00000151923	TIAL1	lightcyan	-0.5046979
ENSG00000156299	TIAM1	grey	-0.1030235
ENSG00000146426	TIAM2	turquoise	-0.4387243
ENSG00000127666	TICAM1	grey	0.22235376
ENSG00000066056	TIE1	tan	0.78724493
ENSG00000145365	TIFA	pink	0.64456899
ENSG00000221944	TIGD1	grey	-0.1265635
ENSG00000180346	TIGD2	grey	0.01810846
ENSG00000173825	TIGD3	grey	0.02017354
ENSG00000169989	TIGD4	grey	-0.0203466
ENSG00000179886	TIGD5	cyan	-0.4312482
ENSG00000164296	TIGD6	grey	-0.0336813
ENSG00000140993	TIGD7	grey	-0.006359
ENSG00000111602	TIMELESS	tan	0.5591578
ENSG00000134809	TIMM10	brown	0.708554
ENSG00000132286	TIMM10B	brown	-0.7770465
ENSG00000134375	TIMM17A	darkred	0.61847847
ENSG00000126768	TIMM17B	brown	0.59825604
ENSG00000075336	TIMM21	turquoise	0.37547384
ENSG00000177370	TIMM22	lightgreen	-0.6292558
ENSG00000138297	TIMM23	yellow	-0.6999441
ENSG00000105197	TIMM50	yellow	-0.699168
ENSG00000126953	TIMM8A	grey	-0.0811836
ENSG00000150779	TIMM8B	red	0.62360077
ENSG00000100575	TIMM9	green	-0.6245045
ENSG00000236900	TIMM9P1	grey	0.42437108
ENSG00000232608	TIMM9P2	grey	0.33724687
ENSG00000113845	TIMMDC1	blue	-0.3994789
ENSG00000102265	TIMP1	pink	0.72277249
ENSG00000035862	TIMP2	grey60	-0.4112194
ENSG00000100234	TIMP3	turquoise	0.81720921
ENSG00000092330	TINF2	blue	-0.3957478
ENSG00000163659	TIPARP	pink	0.67029777
ENSG00000075131	TIPIN	grey	0.17435653
ENSG00000143155	TIPRL	blue	0.34252435
ENSG00000150455	TIRAP	grey	0.05556639
ENSG00000137221	TJAP1	yellow	0.7971246
ENSG00000104067	TJP1	yellow	0.68875029
ENSG00000119139	TJP2	yellow	0.72708586
ENSG00000167900	TK1	grey	-0.1070803
ENSG00000166548	TK2	grey	0.07661533
ENSG00000163931	TKT	grey	0.23384201
ENSG00000160606	TLCD1	turquoise	0.59364243
ENSG00000140950	TLDC1	grey	0.15410963
ENSG00000196781	TLE1	grey	-0.0895745
ENSG00000065717	TLE2	grey	-0.0973139
ENSG00000140332	TLE3	red	0.41336519
ENSG00000106829	TLE4	darkmagenta	0.813494

ENSG00000198586	TLK1	yellow	-0.6420557
ENSG00000146872	TLK2	darkgrey	0.66654909
ENSG00000226049	TLK2P1	grey	-0.1813909
ENSG00000095587	TLL2	grey	-0.0862871
ENSG00000137076	TLN1	blue	-0.7526925
ENSG00000171914	TLN2	brown	0.66469464
ENSG00000174125	TLR1	royalblue	0.82836311
ENSG00000137462	TLR2	royalblue	0.88022118
ENSG00000164342	TLR3	cyan	0.5502969
ENSG00000136869	TLR4	turquoise	0.74586827
ENSG00000187554	TLR5	royalblue	0.72114158
ENSG00000174130	TLR6	cyan	0.69417301
ENSG00000162604	TM2D1	mediumpurp	0.51599402
ENSG00000169490	TM2D2	yellow	-0.4721968
ENSG00000184277	TM2D3	lightcyan	-0.5512728
ENSG00000169908	TM4SF1	tan	0.63882524
ENSG00000163762	TM4SF18	tan	0.81710206
ENSG00000136404	TM6SF1	brown	-0.3645442
ENSG00000149809	TM7SF2	darkgrey	-0.6393626
ENSG00000064115	TM7SF3	brown	-0.3508275
ENSG00000125304	TM9SF2	lightgreen	-0.5791642
ENSG00000077147	TM9SF3	mediumpurp	0.82745214
ENSG00000101337	TM9SF4	brown	0.42559982
ENSG00000198498	TMA16	grey	-0.0475992
ENSG00000232112	TMA7	greenyellow	0.67276975
ENSG00000135926	TMBIM1	pink	0.85829301
ENSG00000155957	TMBIM4	grey	-0.1441641
ENSG00000139644	TMBIM6	turquoise	0.71093056
ENSG00000141524	TMC6	yellow	0.62173269
ENSG00000170537	TMC7	yellow	0.78509046
ENSG00000172765	TMCC1	grey	0.21122762
ENSG00000271270	TMCC1-AS1	blue	0.37348922
ENSG00000133069	TMCC2	grey	0.00632836
ENSG00000057704	TMCC3	yellow	0.85503753
ENSG00000143183	TMCO1	turquoise	0.76743915
ENSG00000150403	TMCO3	brown	-0.5895406
ENSG00000162542	TMCO4	grey	-0.1129956
ENSG00000113119	TMCO6	grey	-0.0112298
ENSG00000170348	TMED10	turquoise	0.63990217
ENSG00000215367	TMED11P	grey	0.14246854
ENSG00000086598	TMED2	grey	-0.0728357
ENSG00000166557	TMED3	green	-0.5797759
ENSG00000158604	TMED4	turquoise	0.45776165
ENSG00000117500	TMED5	turquoise	0.56633519
ENSG00000134970	TMED7	grey60	-0.6669128
ENSG00000100580	TMED8	turquoise	0.70683788
ENSG00000184840	TMED9	grey	-0.1026548
ENSG00000144339	TMEFF2	red	0.44057797

ENSG00000091947	TMEM101	salmon	0.59484475
ENSG00000109066	TMEM104	grey	0.29467465
ENSG00000184988	TMEM106A	cyan	0.83795632
ENSG00000106460	TMEM106B	lightcyan	0.72183934
ENSG00000179029	TMEM107	grey	0.04418543
ENSG00000110108	TMEM109	tan	0.5534798
ENSG00000178307	TMEM11	grey	0.10632186
ENSG00000126062	TMEM115	grey	0.37759587
ENSG00000198270	TMEM116	grey	0.08693554
ENSG00000139173	TMEM117	grey	0.02194012
ENSG00000183160	TMEM119	cyan	0.83543056
ENSG00000189077	TMEM120A	green	-0.4821995
ENSG00000188735	TMEM120B	turquoise	-0.5853046
ENSG00000184986	TMEM121	brown	0.60398075
ENSG00000152558	TMEM123	yellow	0.53667893
ENSG00000179178	TMEM125	yellow	0.70384284
ENSG00000171202	TMEM126A	grey	0.0870848
ENSG00000171204	TMEM126B	red	0.58676357
ENSG00000135956	TMEM127	grey	0.28294577
ENSG00000132406	TMEM128	grey	0.02888703
ENSG00000168936	TMEM129	grey	0.00903158
ENSG00000166448	TMEM130	blue	0.88241587
ENSG00000075568	TMEM131	blue	0.70845659
ENSG00000006118	TMEM132A	turquoise	0.38747851
ENSG00000139364	TMEM132B	darkred	0.52998897
ENSG00000181234	TMEM132C	turquoise	0.70095591
ENSG00000151952	TMEM132D	magenta	0.57613297
ENSG00000170647	TMEM133	turquoise	0.64020403
ENSG00000172663	TMEM134	grey	-0.1999644
ENSG00000166575	TMEM135	turquoise	0.42137756
ENSG00000181264	TMEM136	brown	-0.719385
ENSG00000149483	TMEM138	grey	0.0322655
ENSG00000146859	TMEM140	yellow	0.57347472
ENSG00000244187	TMEM141	yellow	-0.4236774
ENSG00000161558	TMEM143	grey	0.0357876
ENSG00000164124	TMEM144	yellow	0.90206133
ENSG00000167619	TMEM145	black	0.45987051
ENSG00000105677	TMEM147	brown	0.55986828
ENSG00000096092	TMEM14A	greenyellow	0.36554979
ENSG00000137210	TMEM14B	yellow	-0.4291003
ENSG00000111843	TMEM14C	turquoise	0.71389901
ENSG00000221962	TMEM14E	blue	-0.4789659
ENSG00000168890	TMEM150A	blue	-0.4661658
ENSG00000249242	TMEM150C	turquoise	0.48365492
ENSG00000179292	TMEM151A	floralwhite	0.83488789
ENSG00000178233	TMEM151B	yellow	-0.5510413
ENSG00000121895	TMEM156	grey	-0.1912685
ENSG00000249992	TMEM158	grey	-0.283221

ENSG00000011638	TMEM159	purple	0.66027519
ENSG00000130748	TMEM160	grey	0.01473926
ENSG00000064545	TMEM161A	grey	0.20184568
ENSG00000164180	TMEM161B	blue	-0.3684719
ENSG00000247828	TMEM161B-AS1	magenta	0.40059027
ENSG00000152128	TMEM163	yellow	-0.6153603
ENSG00000157600	TMEM164	red	0.58001652
ENSG00000134851	TMEM165	yellow	0.82520898
ENSG00000174695	TMEM167A	grey60	-0.6286316
ENSG00000215717	TMEM167B	grey	0.089098
ENSG00000146802	TMEM168	grey	-0.0750884
ENSG00000163449	TMEM169	grey	0.1955141
ENSG00000166822	TMEM170A	turquoise	0.83829709
ENSG00000205269	TMEM170B	turquoise	0.78815105
ENSG00000184584	TMEM173	grey	-0.1132663
ENSG00000127419	TMEM175	blue	0.3880043
ENSG00000002933	TMEM176A	turquoise	0.63599175
ENSG00000106565	TMEM176B	turquoise	0.66470382
ENSG00000144120	TMEM177	grey	0.06630154
ENSG00000152154	TMEM178A	grey	-0.3644698
ENSG00000258986	TMEM179	blue	0.70573623
ENSG00000185475	TMEM179B	yellow	0.60474598
ENSG00000151353	TMEM18	brown	0.64620155
ENSG00000138111	TMEM180	grey	0.09392629
ENSG00000146433	TMEM181	turquoise	-0.5548709
ENSG00000163444	TMEM183A	blue	0.57990888
ENSG00000227609	TMEM183AP1	grey	0.49167778
ENSG00000198792	TMEM184B	grey	0.19101303
ENSG00000164168	TMEM184C	grey	0.27236293
ENSG00000155984	TMEM185A	green	-0.613897
ENSG00000226479	TMEM185B	turquoise	0.60568291
ENSG00000184857	TMEM186	salmon	0.54553592
ENSG00000177854	TMEM187	grey	0.1159201
ENSG00000240849	TMEM189	floralwhite	0.57385672
ENSG00000139291	TMEM19	grey	0.05267631
ENSG00000206140	TMEM191C	yellow	-0.3274328
ENSG00000170088	TMEM192	turquoise	0.39613654
ENSG00000166881	TMEM194A	grey	-0.1279871
ENSG00000189362	TMEM194B	turquoise	0.37971714
ENSG00000173452	TMEM196	darkmagenta	0.74546656
ENSG00000188760	TMEM198	grey	-0.014041
ENSG00000182796	TMEM198B	yellow	0.51208623
ENSG00000244045	TMEM199	grey	0.28714917
ENSG00000135048	TMEM2	tan	0.79238195
ENSG00000253304	TMEM200B	blue	-0.4527474
ENSG00000206432	TMEM200C	turquoise	0.44992818
ENSG00000188807	TMEM201	grey	0.15903033
ENSG00000187713	TMEM203	darkred	0.48973455

ENSG00000131634	TMEM204	tan	0.77504608
ENSG00000105518	TMEM205	salmon	0.55112892
ENSG00000065600	TMEM206	yellow	0.77830135
ENSG00000168701	TMEM208	yellow	-0.5297432
ENSG00000146842	TMEM209	grey	-0.0815591
ENSG00000206069	TMEM211	blue	0.29986468
ENSG00000119777	TMEM214	grey	0.12416765
ENSG00000187049	TMEM216	grey	0.13988063
ENSG00000150433	TMEM218	grey	-0.0988298
ENSG00000149932	TMEM219	brown	0.53557435
ENSG00000187824	TMEM220	turquoise	0.47131743
ENSG00000186501	TMEM222	grey	-0.0047726
ENSG00000168569	TMEM223	grey	0.02931505
ENSG00000234224	TMEM229A	turquoise	0.58639631
ENSG00000198133	TMEM229B	grey	0.12975543
ENSG00000089063	TMEM230	lightcyan	0.68506368
ENSG00000160055	TMEM234	brown	0.45290944
ENSG00000204278	TMEM235	yellow	0.68796668
ENSG00000155755	TMEM237	black	-0.4864902
ENSG00000134490	TMEM241	blue	0.39160749
ENSG00000215712	TMEM242	yellow	-0.3889185
ENSG00000135185	TMEM243	grey	-0.0617097
ENSG00000106771	TMEM245	grey	0.14180989
ENSG00000165152	TMEM246	blue	0.74273305
ENSG00000106609	TMEM248	salmon	0.74960664
ENSG00000149582	TMEM25	blue	0.764325
ENSG00000153485	TMEM251	grey	0.0049207
ENSG00000133678	TMEM254	grey	-0.0152011
ENSG00000230091	TMEM254-AS1	grey	0.1349985
ENSG00000205544	TMEM256	grey	-0.2321905
ENSG00000221870	TMEM257	grey	-0.1013703
ENSG00000134825	TMEM258	turquoise	-0.6295857
ENSG00000182087	TMEM259	salmon	0.56196632
ENSG00000070269	TMEM260	turquoise	-0.431786
ENSG00000137038	TMEM261	greenyellow	0.64060247
ENSG00000270814	TMEM261P1	grey	-0.025703
ENSG00000109133	TMEM33	lightcyan	0.56681426
ENSG00000126950	TMEM35	purple	0.76617392
ENSG00000072954	TMEM38A	grey	0.10218154
ENSG00000095209	TMEM38B	grey60	-0.5468605
ENSG00000176142	TMEM39A	blue	-0.4956247
ENSG00000121775	TMEM39B	grey	-0.15805
ENSG00000163900	TMEM41A	lightgreen	-0.6693639
ENSG00000169964	TMEM42	green	-0.3293823
ENSG00000170876	TMEM43	blue	-0.6023917
ENSG00000145014	TMEM44	black	0.50554104
ENSG00000231770	TMEM44-AS1	grey	0.18996703
ENSG00000147027	TMEM47	turquoise	0.82557558

ENSG00000118600	TMEM5	grey	0.05851051
ENSG00000183726	TMEM50A	grey	-0.15016
ENSG00000142188	TMEM50B	magenta	-0.5504978
ENSG00000171729	TMEM51	turquoise	0.51455978
ENSG00000165685	TMEM52B	red	-0.46291
ENSG00000126106	TMEM53	grey	0.02584232
ENSG00000121900	TMEM54	blue	-0.5332534
ENSG00000165782	TMEM55B	blue	0.37552406
ENSG00000152078	TMEM56	turquoise	0.61541839
ENSG00000204178	TMEM57	turquoise	-0.4655746
ENSG00000116209	TMEM59	turquoise	0.38616714
ENSG00000105696	TMEM59L	blue	0.71677364
ENSG00000135211	TMEM60	grey	-0.1387614
ENSG00000137842	TMEM62	blue	0.3268148
ENSG00000196187	TMEM63A	yellow	0.71566208
ENSG00000137216	TMEM63B	blue	0.67563094
ENSG00000165548	TMEM63C	blue	0.534742
ENSG00000180694	TMEM64	grey	-0.2099577
ENSG00000164983	TMEM65	lightcyan	0.69623378
ENSG00000133872	TMEM66	red	0.65799969
ENSG00000167904	TMEM68	grey	0.24225376
ENSG00000159596	TMEM69	turquoise	0.44605334
ENSG00000175606	TMEM70	brown	0.55906241
ENSG00000164841	TMEM74	brown	-0.4489178
ENSG00000125895	TMEM74B	turquoise	-0.4313336
ENSG00000163472	TMEM79	grey	0.21080767
ENSG00000177042	TMEM80	grey	-0.0208613
ENSG00000151117	TMEM86A	grey	-0.1812055
ENSG00000180089	TMEM86B	grey	0.00673227
ENSG00000103978	TMEM87A	grey	0.07265347
ENSG00000153214	TMEM87B	grey	0.16690869
ENSG00000167874	TMEM88	tan	0.4891697
ENSG00000129925	TMEM8A	grey	0.15300925
ENSG00000137103	TMEM8B	grey	0.07785875
ENSG00000116857	TMEM9	red	0.67215996
ENSG00000142046	TMEM91	blue	0.62656383
ENSG00000109084	TMEM97	grey	0.10335144
ENSG00000006042	TMEM98	yellow	0.76901856
ENSG00000167920	TMEM99	grey	0.17619797
ENSG00000254860	TMEM9B-AS1	grey	0.13638601
ENSG00000144747	TMF1	grey	0.32395099
ENSG00000185973	TMLHE	grey	0.02480291
ENSG00000224533	TMLHE-AS1	grey	0.18938129
ENSG00000136842	TMOD1	magenta	0.74949195
ENSG00000128872	TMOD2	turquoise	0.67144077
ENSG00000138594	TMOD3	blue	-0.4289009
ENSG00000120802	TMPO	grey	0.36299545
ENSG00000188167	TMPPE	turquoise	0.54156461

ENSG00000232398	TMPRSS11CP	grey	0.32637302
ENSG00000153802	TMPRSS11D	grey	0.21545845
ENSG00000229009	TMPRSS11GP	grey	0.29210903
ENSG00000166682	TMPRSS5	grey	0.03710519
ENSG00000187045	TMPRSS6	black	0.5357748
ENSG00000034510	TMSB10	brown	0.65197201
ENSG00000228499	TMSB10P1	grey	0.46483309
ENSG00000205542	TMSB4X	greenyellow	0.77177405
ENSG00000236876	TMSB4XP1	grey	0.49285729
ENSG00000223437	TMSB4XP3	grey	0.2333281
ENSG00000223551	TMSB4XP4	greenyellow	0.52352758
ENSG00000187653	TMSB4XP8	greenyellow	0.59570027
ENSG00000154620	TMSB4Y	grey	-0.0658143
ENSG00000133687	TMTC1	brown	0.52485113
ENSG00000179104	TMTC2	yellow	0.91015572
ENSG00000139324	TMTC3	turquoise	0.61618637
ENSG00000125247	TMTC4	yellow	0.80365133
ENSG00000164897	TMUB1	grey	0.18059224
ENSG00000168591	TMUB2	brown	0.4706161
ENSG00000139921	TMX1	turquoise	0.75247213
ENSG00000213593	TMX2	grey	0.28459023
ENSG00000166479	TMX3	grey	-0.2592303
ENSG00000041982	TNC	grey	-0.0861523
ENSG00000109079	TNFAIP1	pink	0.66151171
ENSG00000185215	TNFAIP2	grey	0.03948229
ENSG00000118503	TNFAIP3	pink	0.56616168
ENSG00000123610	TNFAIP6	yellow	0.53824252
ENSG00000163154	TNFAIP8L2	royalblue	0.66521473
ENSG00000183578	TNFAIP8L3	black	-0.6911139
ENSG00000120889	TNFRSF10B	pink	0.70857134
ENSG00000127863	TNFRSF19	grey	-0.0379462
ENSG00000067182	TNFRSF1A	pink	0.88510168
ENSG00000028137	TNFRSF1B	royalblue	0.7874796
ENSG00000146072	TNFRSF21	grey	-0.194858
ENSG00000215788	TNFRSF25	grey	-0.0932685
ENSG00000121858	TNFSF10	tan	0.39372776
ENSG00000239697	TNFSF12	brown	-0.3161098
ENSG00000161955	TNFSF13	turquoise	0.64685194
ENSG00000102524	TNFSF13B	royalblue	0.45583997
ENSG00000117586	TNFSF4	grey	0.00640509
ENSG00000125657	TNFSF9	grey	0.18993896
ENSG00000154310	TNIK	turquoise	0.46367674
ENSG00000145901	TNIP1	grey	0.04437812
ENSG00000168884	TNIP2	pink	0.80369815
ENSG00000174292	TNK1	grey	-0.3576704
ENSG00000061938	TNK2	blue	0.58437976
ENSG00000173273	TNKS	magenta	-0.5159978
ENSG00000149115	TNKS1BP1	grey	0.12374956

ENSG00000107854	TNKS2	grey	0.07715834
ENSG00000101470	TNNC2	turquoise	-0.4925961
ENSG00000105048	TNNT1	grey	-0.1073564
ENSG00000118245	TNP1	grey	0.2132175
ENSG00000083312	TNPO1	turquoise	0.70284634
ENSG00000225423	TNPO1P1	grey	0.20295985
ENSG00000105576	TNPO2	red	0.36937124
ENSG00000064419	TNPO3	mediumpurp	-0.6175587
ENSG00000182095	TNRC18	grey	-0.0979766
ENSG00000090905	TNRC6A	grey	0.35331555
ENSG00000100354	TNRC6B	turquoise	0.43676839
ENSG00000078687	TNRC6C	yellow	0.50779077
ENSG00000204282	TNRC6C-AS1	yellow	0.48486649
ENSG00000079308	TNS1	green	0.79881939
ENSG00000136205	TNS3	turquoise	0.64924607
ENSG00000168477	TNXB	grey	0.14864991
ENSG00000141232	TOB1	turquoise	0.61109433
ENSG00000229980	TOB1-AS1	grey	-0.0994005
ENSG00000183864	TOB2	blue	-0.8263893
ENSG00000132773	TOE1	grey	-0.005435
ENSG00000078902	TOLLIP	yellow	-0.5972312
ENSG00000255153	TOLLIP-AS1	grey	0.11023433
ENSG00000100284	TOM1	blue	0.55180853
ENSG00000141198	TOM1L1	black	-0.4667074
ENSG00000175662	TOM1L2	brown	0.33326116
ENSG00000173726	TOMM20	turquoise	-0.6175521
ENSG00000100216	TOMM22	turquoise	0.44492695
ENSG00000025772	TOMM34	blue	0.70684589
ENSG00000130204	TOMM40	turquoise	-0.4041972
ENSG00000158882	TOMM40L	grey	0.20403328
ENSG00000175768	TOMM5	turquoise	-0.5447113
ENSG00000196683	TOMM7	brown	0.69889256
ENSG00000198900	TOP1	blue	-0.4503599
ENSG00000184428	TOP1MT	grey	-0.1076209
ENSG00000177302	TOP3A	turquoise	-0.5243983
ENSG00000100038	TOP3B	grey	-0.2037699
ENSG00000163781	TOPBP1	brown	0.37497931
ENSG00000197579	TOPORS	lightgreen	0.85071323
ENSG00000235453	TOPORS-AS1	greenyellow	0.43276049
ENSG00000136827	TOR1A	green	-0.3554346
ENSG00000143337	TOR1AIP1	turquoise	0.58478118
ENSG00000136816	TOR1B	grey	0.2576428
ENSG00000160404	TOR2A	grey	0.14008309
ENSG00000186283	TOR3A	grey	0.02473701
ENSG00000198846	TOX	red	0.74145266
ENSG00000124191	TOX2	grey	-0.0047409
ENSG00000103460	TOX3	purple	0.62303943
ENSG00000092203	TOX4	grey	0.10329074

ENSG00000141510	TP53	turquoise	0.56249518
ENSG00000067369	TP53BP1	turquoise	-0.6525068
ENSG00000143514	TP53BP2	turquoise	0.83525663
ENSG00000175274	TP53I11	yellow	-0.4190116
ENSG00000167543	TP53I13	grey	0.06477954
ENSG00000115129	TP53I3	magenta	-0.4839406
ENSG00000164938	TP53INP1	brown	-0.7304667
ENSG00000078804	TP53INP2	green	0.91041399
ENSG00000172315	TP53RK	brown	-0.6791312
ENSG00000182165	TP53TG1	yellow	-0.3787135
ENSG00000124251	TP53TG5	yellow	0.73089649
ENSG00000227372	TP73-AS1	blue	-0.4724042
ENSG00000146242	TPBG	black	0.68513225
ENSG00000261594	TPBGL	grey	-0.0535988
ENSG00000186815	TPCN1	blue	-0.4340949
ENSG00000162341	TPCN2	grey	-0.085919
ENSG00000076554	TPD52	turquoise	-0.3804464
ENSG00000111907	TPD52L1	purple	-0.3747036
ENSG00000101150	TPD52L2	grey	0.03454229
ENSG00000141933	TPGS1	grey	0.24285432
ENSG00000134779	TPGS2	grey	0.03019526
ENSG00000129167	TPH1	blue	0.49841919
ENSG00000111669	TPI1	yellow	-0.6682335
ENSG00000196511	TPK1	grey	0.30845152
ENSG00000140416	TPM1	grey	-0.0647536
ENSG00000198467	TPM2	grey	-0.3621013
ENSG00000143549	TPM3	brown	0.46397777
ENSG00000213050	TPM3P1	turquoise	0.35767388
ENSG00000187536	TPM3P7	grey	0.22626936
ENSG00000241015	TPM3P9	grey	0.16006983
ENSG00000167460	TPM4	yellow	0.61746761
ENSG00000166340	TPP1	turquoise	0.70007859
ENSG00000134900	TPP2	grey	0.15829302
ENSG00000171368	TPPP	yellow	0.61974562
ENSG00000159713	TPPP3	grey	0.01695642
ENSG00000047410	TPR	lightgreen	0.74713166
ENSG00000163870	TPRA1	grey	0.21867138
ENSG00000144034	TPRKB	grey	0.22760565
ENSG00000176058	TPRN	yellow	0.57308394
ENSG00000169902	TPST1	pink	0.67531076
ENSG00000128294	TPST2	grey	0.19627222
ENSG00000133112	TPT1	greenyellow	0.80892241
ENSG00000170919	TPT1-AS1	grey	-0.0893565
ENSG00000180221	TPT1P10	grey	0.22124237
ENSG00000214108	TPT1P5	grey	0.46453254
ENSG00000253771	TPTE2P1	grey	0.01064512
ENSG00000168852	TPTE2P5	grey	0.135227
ENSG00000100181	TPTEP1	yellow	0.41160179

ENSG00000088325	TPX2	grey	0.04910992
ENSG00000164548	TRA2A	lightgreen	0.70779212
ENSG00000170638	TRABD	grey	0.10784053
ENSG00000229164	TRAC	blue	0.33712232
ENSG00000102871	TRADD	yellow	0.33200997
ENSG00000056558	TRAF1	yellow	0.70797707
ENSG00000127191	TRAF2	turquoise	-0.3147323
ENSG00000131323	TRAF3	turquoise	0.44603849
ENSG00000204104	TRAF3IP1	darkgrey	0.50285359
ENSG00000056972	TRAF3IP2	pink	0.58008595
ENSG00000231889	TRAF3IP2-AS1	turquoise	0.37362258
ENSG00000009790	TRAF3IP3	cyan	0.52100227
ENSG00000076604	TRAF4	yellow	0.54670643
ENSG00000082512	TRAF5	grey	0.09868959
ENSG00000175104	TRAF6	grey	0.16868178
ENSG00000131653	TRAF7	grey	0.15775103
ENSG00000135148	TRAFD1	grey	-0.1902132
ENSG00000183763	TRAIP	green	-0.514108
ENSG00000182606	TRAK1	grey	0.15994437
ENSG00000115993	TRAK2	green	0.76803719
ENSG00000067167	TRAM1	blue	-0.7051218
ENSG00000174599	TRAM1L1	grey	-0.0293672
ENSG00000065308	TRAM2	tan	0.61699352
ENSG00000225791	TRAM2-AS1	lightcyan	0.69440579
ENSG00000168016	TRANK1	blue	0.59045734
ENSG00000126602	TRAP1	brown	0.62442036
ENSG00000170043	TRAPPC1	grey	0.04808081
ENSG00000160218	TRAPPC10	yellow	0.49747283
ENSG00000168538	TRAPPC11	turquoise	-0.5303991
ENSG00000171853	TRAPPC12	brown	0.61938012
ENSG00000225234	TRAPPC12-AS1	grey	0.0484572
ENSG00000113597	TRAPPC13	brown	0.59771832
ENSG00000196459	TRAPPC2	blue	0.46597557
ENSG00000167515	TRAPPC2L	blue	0.44368922
ENSG00000256060	TRAPPC2P1	grey	0.25953845
ENSG00000054116	TRAPPC3	brown	0.76621877
ENSG00000196655	TRAPPC4	yellow	-0.6367353
ENSG00000181029	TRAPPC5	brown	0.36488665
ENSG00000182400	TRAPPC6B	grey	-0.472811
ENSG00000153339	TRAPPC8	grey	0.15674848
ENSG00000167632	TRAPPC9	green	-0.4284048
ENSG00000256379	TRAV8-5	grey	0.32376264
ENSG00000211772	TRBC2	magenta	-0.3935593
ENSG00000231165	TRBV26OR9-2	grey	0.17683199
ENSG00000239992	TRBVA	grey	0.31668874
ENSG00000241911	TRBVB	grey	0.34505017
ENSG00000107614	TRDMT1	black	0.559675
ENSG00000095970	TREM2	cyan	0.88442417

ENSG00000124496	TRERF1	magenta	0.48945992
ENSG00000213689	TREX1	grey	0.18811139
ENSG00000227191	TRGC2	grey	0.23203753
ENSG00000211688	TRGJP2	grey	0.42736199
ENSG00000072657	TRHDE	grey	0.01197201
ENSG00000170855	TRIAP1	brown	0.43430857
ENSG00000173334	TRIB1	turquoise	0.51268581
ENSG00000071575	TRIB2	turquoise	0.53786303
ENSG00000154370	TRIM11	grey	-0.2378793
ENSG00000204977	TRIM13	brown	-0.6111668
ENSG00000106785	TRIM14	turquoise	0.50043765
ENSG00000221926	TRIM16	grey	0.07781229
ENSG00000108448	TRIM16L	grey	0.1169518
ENSG00000162931	TRIM17	red	-0.5603979
ENSG00000132109	TRIM21	pink	0.57143003
ENSG00000132274	TRIM22	black	0.49215763
ENSG00000113595	TRIM23	grey	0.03832033
ENSG00000122779	TRIM24	grey	-0.0858437
ENSG00000121060	TRIM25	blue	-0.4882525
ENSG00000234127	TRIM26	lightcyan	0.59620941
ENSG00000236475	TRIM26BP	grey	0.20646299
ENSG00000204713	TRIM27	brown	0.4255175
ENSG00000130726	TRIM28	grey	0.0745021
ENSG00000110171	TRIM3	grey	-0.0848305
ENSG00000119401	TRIM32	grey	0.02187587
ENSG00000197323	TRIM33	turquoise	0.40975735
ENSG00000104228	TRIM35	grey	0.02101791
ENSG00000152503	TRIM36	blue	0.61014268
ENSG00000108395	TRIM37	blue	0.76230133
ENSG00000112343	TRIM38	tan	0.59796573
ENSG00000204599	TRIM39	turquoise	0.36384702
ENSG00000146833	TRIM4	grey	-0.0916725
ENSG00000146063	TRIM41	lightgreen	0.65240762
ENSG00000166326	TRIM44	lightgreen	0.58693613
ENSG00000134253	TRIM45	black	-0.4780017
ENSG00000163462	TRIM46	blue	0.75581744
ENSG00000132481	TRIM47	turquoise	0.56820211
ENSG00000132256	TRIM5	pink	0.65503083
ENSG00000219061	TRIM51FP	grey	0.25186056
ENSG00000183718	TRIM52	grey	0.04524004
ENSG00000169871	TRIM56	green	0.69122367
ENSG00000162722	TRIM58	grey	0.10956887
ENSG00000213186	TRIM59	yellow	0.76141231
ENSG00000227986	TRIM60P18	grey	0.26330109
ENSG00000183439	TRIM61	grey	0.12883245
ENSG00000116525	TRIM62	grey	0.1142407
ENSG00000141569	TRIM65	grey	0.13358402
ENSG00000166436	TRIM66	purple	0.70929555

ENSG00000119283	TRIM67	grey	0.06693663
ENSG00000167333	TRIM68	grey	-0.0548771
ENSG00000185880	TRIM69	grey	0.1962887
ENSG00000178809	TRIM73	grey	0.325834
ENSG00000171206	TRIM8	blue	-0.5066147
ENSG00000100505	TRIM9	darkmagenta	-0.5123025
ENSG00000038382	TRIO	turquoise	-0.5316181
ENSG00000125733	TRIP10	pink	0.70007684
ENSG00000100815	TRIP11	blue	-0.4608181
ENSG00000153827	TRIP12	blue	-0.6344651
ENSG00000103671	TRIP4	turquoise	-0.4452781
ENSG00000087077	TRIP6	turquoise	0.69111931
ENSG00000205133	TRIQK	grey	0.06550769
ENSG00000043514	TRIT1	grey	0.08722206
ENSG00000104907	TRMT1	turquoise	-0.4532723
ENSG00000145331	TRMT10A	grey	0.4022048
ENSG00000165275	TRMT10B	green	0.38679219
ENSG00000174173	TRMT10C	turquoise	0.45708441
ENSG00000173113	TRMT112	greenyellow	0.67187074
ENSG00000183665	TRMT12	grey	0.22954466
ENSG00000122435	TRMT13	yellow	0.67811386
ENSG00000121486	TRMT1L	grey	-0.0153547
ENSG00000099899	TRMT2A	grey	0.2513806
ENSG00000188917	TRMT2B	grey	0.03014513
ENSG00000155275	TRMT44	yellowgreen	-0.6697601
ENSG00000126814	TRMT5	darkred	0.537673
ENSG00000089195	TRMT6	turquoise	-0.5152775
ENSG00000166166	TRMT61A	black	0.51765253
ENSG00000171103	TRMT61B	greenyellow	-0.4477267
ENSG00000100416	TRMU	brown	0.57298144
ENSG00000180098	TRNAU1AP	brown	0.48224205
ENSG00000253368	TRNP1	blue	0.7019024
ENSG00000072756	TRNT1	grey	-0.058042
ENSG00000067445	TRO	blue	0.81691595
ENSG00000116747	TROVE2	turquoise	0.85481594
ENSG00000138741	TRPC3	greenyellow	-0.5967917
ENSG00000133107	TRPC4	grey	0.1661257
ENSG00000100991	TRPC4AP	turquoise	-0.4964891
ENSG00000072315	TRPC5	grey	0.13605869
ENSG00000142185	TRPM2	blue	0.43992601
ENSG00000083067	TRPM3	turquoise	0.5479273
ENSG00000130529	TRPM4	grey	0.01313388
ENSG00000119121	TRPM6	yellow	0.75448898
ENSG00000092439	TRPM7	black	0.48444182
ENSG00000149743	TRPT1	grey	-0.127299
ENSG00000167723	TRPV3	blue	-0.4688214
ENSG00000165125	TRPV6	red	-0.5830879
ENSG00000196367	TRRAP	grey	0.13767115

ENSG00000165832	TRUB1	blue	0.42930692
ENSG00000167112	TRUB2	grey	0.15040765
ENSG00000165699	TSC1	grey	0.00120319
ENSG00000103197	TSC2	grey	-0.0196198
ENSG00000102804	TSC22D1	pink	0.47541695
ENSG00000196428	TSC22D2	grey	0.08702369
ENSG00000157514	TSC22D3	blue	-0.5045996
ENSG00000166925	TSC22D4	yellow	0.59908014
ENSG00000198860	TSEN15	grey	-0.1572476
ENSG00000230604	TSEN15P2	grey	0.45989173
ENSG00000154743	TSEN2	turquoise	-0.5919314
ENSG00000170892	TSEN34	grey	0.07713242
ENSG00000182173	TSEN54	grey	0.13387815
ENSG00000123297	TSFM	grey	-0.0849986
ENSG00000074319	TSG101	lightgreen	-0.6384174
ENSG00000135951	TSGA10	grey	0.09967474
ENSG00000165409	TSHR	grey	0.02240058
ENSG00000179981	TSHZ1	turquoise	0.38472262
ENSG00000182463	TSHZ2	grey	-0.1561965
ENSG00000121297	TSHZ3	magenta	-0.413669
ENSG00000211460	TSN	turquoise	0.58262501
ENSG00000171045	TSNARE1	grey	-0.1589424
ENSG00000116918	TSNAX	darkred	0.50426867
ENSG00000102904	TSNAXIP1	brown	0.71496822
ENSG00000106025	TSPAN12	red	0.59863066
ENSG00000106537	TSPAN13	grey60	0.65393046
ENSG00000108219	TSPAN14	magenta	-0.5851387
ENSG00000099282	TSPAN15	yellow	0.79415737
ENSG00000048140	TSPAN17	grey	-0.0825307
ENSG00000134198	TSPAN2	grey	0.14179206
ENSG00000140391	TSPAN3	mediumpurp	0.63072945
ENSG00000135452	TSPAN31	grey	0.23231322
ENSG00000158457	TSPAN33	blue	-0.4063768
ENSG00000168785	TSPAN5	lightcyan	-0.577239
ENSG00000000003	TSPAN6	turquoise	0.52117704
ENSG00000156298	TSPAN7	darkred	0.72246226
ENSG00000011105	TSPAN9	grey	0.02558414
ENSG00000100300	TSPO	blue	-0.5419788
ENSG00000235217	TSPY26P	grey	-0.0357234
ENSG00000189241	TSPYL1	brown	0.75680513
ENSG00000184205	TSPYL2	blue	0.32587873
ENSG00000187189	TSPYL4	blue	0.7190615
ENSG00000180543	TSPYL5	blue	0.68170135
ENSG00000178021	TSPYL6	turquoise	-0.4372886
ENSG00000167721	TSR1	grey	0.24473816
ENSG00000158526	TSR2	darkgrey	0.70334566
ENSG00000007520	TSR3	grey	0.05677439
ENSG00000032389	TSSC1	green	-0.6948574

ENSG00000224885	TSSC1-IT1	grey	0.13374271
ENSG00000184281	TSSC4	salmon	0.6661156
ENSG00000162526	TSSK3	grey	0.06494304
ENSG00000139908	TSSK4	yellow	0.40156554
ENSG00000104522	TSTA3	yellow	-0.5128666
ENSG00000215845	TSTD1	greenyellow	0.66565857
ENSG00000136925	TSTD2	red	-0.5728237
ENSG00000228439	TSTD3	cyan	-0.4755698
ENSG00000146216	TTBK1	darkorange	0.75044336
ENSG00000128881	TTBK2	grey	0.33623277
ENSG00000113312	TTC1	grey	0.00729349
ENSG00000149292	TTC12	grey	-0.0116548
ENSG00000143643	TTC13	turquoise	-0.4048175
ENSG00000011295	TTC19	brown	0.50631623
ENSG00000168026	TTC21A	turquoise	-0.5888452
ENSG00000123607	TTC21B	turquoise	-0.3872695
ENSG00000006555	TTC22	grey	-0.1239996
ENSG00000103852	TTC23	grey	0.17976268
ENSG00000205838	TTC23L	yellow	0.49327342
ENSG00000105948	TTC26	grey	0.03672515
ENSG00000018699	TTC27	yellowgreen	-0.6208573
ENSG00000100154	TTC28	grey60	-0.5154647
ENSG00000235954	TTC28-AS1	brown	0.33456712
ENSG00000182670	TTC3	turquoise	-0.5856097
ENSG00000228677	TTC3-AS1	lightcyan	-0.4658452
ENSG00000197557	TTC30A	turquoise	0.41477034
ENSG00000196659	TTC30B	grey	0.22727071
ENSG00000115282	TTC31	grey	0.01928867
ENSG00000183891	TTC32	yellow	0.41609398
ENSG00000113638	TTC33	grey	0.02535481
ENSG00000215912	TTC34	grey	-0.0934462
ENSG00000172425	TTC36	grey	0.2237409
ENSG00000198677	TTC37	grey	-0.0921751
ENSG00000075234	TTC38	grey	0.09141206
ENSG00000085831	TTC39A	blue	0.5358592
ENSG00000155158	TTC39B	grey	0.27039584
ENSG00000168234	TTC39C	yellow	-0.6280615
ENSG00000215105	TTC3P1	blue	0.35630205
ENSG00000136319	TTC5	red	0.51641259
ENSG00000068724	TTC7A	red	-0.5750129
ENSG00000165914	TTC7B	darkorange	0.62515677
ENSG00000165533	TTC8	blue	0.63664885
ENSG00000133985	TTC9	purple	0.81850327
ENSG00000174521	TTC9B	grey	-0.2672879
ENSG00000162222	TTC9C	brown	0.59336796
ENSG00000125482	TTF1	grey	0.11478147
ENSG00000116830	TTF2	grey	0.10812961
ENSG00000101407	TTI1	grey	-0.0364772

ENSG00000129696	TTI2	grey	0.22627822
ENSG00000114999	TTL	grey	0.1942731
ENSG00000100271	TTLL1	grey	0.05264078
ENSG00000175764	TTLL11	red	-0.826135
ENSG00000237548	TTLL11-IT1	grey	0.34714733
ENSG00000100304	TTLL12	grey	0.08754302
ENSG00000214021	TTLL3	brown	0.51133967
ENSG00000135912	TTLL4	blue	-0.4060201
ENSG00000119685	TTLL5	grey	-0.0370854
ENSG00000137941	TTLL7	yellow	0.74864795
ENSG00000233061	TTLL7-IT1	yellow	0.6546588
ENSG00000131044	TTLL9	grey	-0.2275166
ENSG00000155657	TTN	grey	-0.0259759
ENSG00000237298	TTN-AS1	grey	0.38182543
ENSG00000137561	TTPA	turquoise	0.63469349
ENSG00000124120	TTPAL	grey	0.27421066
ENSG00000229236	TTTY10	grey	-0.0673131
ENSG00000176728	TTTY14	grey	-0.160488
ENSG00000233864	TTTY15	grey	0.00604498
ENSG00000167614	TTYH1	turquoise	0.5724423
ENSG00000141540	TTYH2	yellow	0.93460759
ENSG00000136295	TTYH3	purple	0.39794098
ENSG00000166402	TUB	yellowgreen	0.70406462
ENSG00000167552	TUBA1A	darkmagenta	0.6064023
ENSG00000123416	TUBA1B	grey	0.0852434
ENSG00000167553	TUBA1C	brown	-0.2905524
ENSG00000075886	TUBA3D	grey	0.01618745
ENSG00000127824	TUBA4A	blue	0.54543619
ENSG00000183785	TUBA8	grey	0.12659353
ENSG00000196230	TUBB	yellow	-0.5011299
ENSG00000137285	TUBB2B	turquoise	0.67748577
ENSG00000104833	TUBB4A	floralwhite	0.52628061
ENSG00000188229	TUBB4B	red	0.65650738
ENSG00000176014	TUBB6	pink	0.69530791
ENSG00000108423	TUBD1	grey	0.28939555
ENSG00000074935	TUBE1	grey	0.05781166
ENSG00000131462	TUBG1	grey	0.20799157
ENSG00000237972	TUBG1P	grey	0.12973274
ENSG00000037042	TUBG2	blue	0.65187183
ENSG00000130640	TUBGCP2	brown	0.6086948
ENSG00000126216	TUBGCP3	red	-0.5631244
ENSG00000137822	TUBGCP4	lightgreen	-0.6117599
ENSG00000153575	TUBGCP5	pink	-0.4807723
ENSG00000128159	TUBGCP6	brown	0.50238352
ENSG00000143367	TUFT1	grey	0.17891322
ENSG00000253352	TUG1	turquoise	0.33782201
ENSG00000078246	TULP3	brown	-0.6007369
ENSG00000130338	TULP4	green	0.86549701

ENSG00000198680	TUSC1	grey	-0.0026029
ENSG00000114383	TUSC2	grey	-0.2424252
ENSG00000104723	TUSC3	turquoise	-0.6377598
ENSG00000243197	TUSC7	grey	0.08498933
ENSG00000149016	TUT1	grey	0.24916977
ENSG00000166676	TVP23A	yellow	-0.5142447
ENSG00000171928	TVP23B	grey	0.23576357
ENSG00000175106	TVP23C	grey	-0.0641598
ENSG00000262470	TVP23CP2	grey	0.22114166
ENSG00000151239	TWF1	turquoise	0.57067799
ENSG00000178082	TWF1P1	yellow	0.70922201
ENSG00000247596	TWF2	turquoise	-0.5521136
ENSG00000105849	TWISTNB	grey	-0.1126289
ENSG00000128791	TWSG1	turquoise	0.72644056
ENSG00000084652	TXLNA	pink	0.55251418
ENSG00000086712	TXLNG	turquoise	-0.4963006
ENSG00000131002	TXLNG2P	grey	-0.1664513
ENSG00000100348	TXN2	grey	0.27722386
ENSG00000153066	TXNDC11	turquoise	-0.3811525
ENSG00000117862	TXNDC12	lightgreen	-0.5718558
ENSG00000087301	TXNDC16	grey	0.07137439
ENSG00000129235	TXNDC17	grey	-0.0548027
ENSG00000115514	TXNDC9	grey	-0.0477814
ENSG00000091164	TXNL1	brown	-0.4810565
ENSG00000141759	TXNL4A	brown	0.84602751
ENSG00000140830	TXNL4B	blue	-0.5280735
ENSG00000234036	TXNP6	grey	0.22945487
ENSG00000198431	TXNRD1	grey	0.2681159
ENSG00000184470	TXNRD2	green	-0.5399808
ENSG00000197763	TXNRD3	grey	0.25339114
ENSG00000105397	TYK2	grey	-0.118496
ENSG00000176890	TYMS	yellow	0.5643714
ENSG00000092445	TYRO3	darkmagenta	0.54918951
ENSG00000011600	TYROBP	cyan	0.86783108
ENSG00000156521	TYSND1	grey	-0.0213199
ENSG00000198874	TYW1	grey	-0.0392277
ENSG00000254184	TYW1B	grey	0.19352568
ENSG00000162623	TYW3	grey	0.07546785
ENSG00000162971	TYW5	grey	0.25118627
ENSG00000160201	U2AF1	grey	-0.0939451
ENSG00000161265	U2AF1L4	grey	-0.0192312
ENSG00000063244	U2AF2	grey	-0.1060617
ENSG00000199856	U3	grey	0.51225309
ENSG00000200693	U3	grey	0.29860645
ENSG00000212211	U3	grey	0.35215441
ENSG00000252792	U3	grey	0.53803402
ENSG00000252981	U3	grey	0.48399936
ENSG00000265369	U3	turquoise	-0.4714381

ENSG00000228481	U51244.2	grey	0.25997965
ENSG00000232725	U52111.14	grey	0.27037319
ENSG00000213600	U73169.1	grey	0.25901384
ENSG00000238963	U8	grey	0.4504479
ENSG00000259886	U82695.10	darkred	0.56076531
ENSG00000137831	UACA	green	0.71971946
ENSG00000117143	UAP1	grey	0.20844279
ENSG00000197355	UAP1L1	grey	0.0847865
ENSG00000130985	UBA1	grey	0.29918449
ENSG00000126261	UBA2	grey	0.29347255
ENSG00000144744	UBA3	turquoise	0.64146415
ENSG00000081307	UBA5	grey	-0.17849
ENSG00000221983	UBA52	greenyellow	0.78433405
ENSG00000240793	UBA52P8	grey	0.17516655
ENSG00000033178	UBA6	yellow	0.36013
ENSG00000182179	UBA7	cyan	0.61943995
ENSG00000130560	UBAC1	grey	-0.0371236
ENSG00000134882	UBAC2	grey	0.11307589
ENSG00000153443	UBALD1	grey	0.0492984
ENSG00000185262	UBALD2	blue	-0.7081439
ENSG00000165006	UBAP1	grey	0.26725567
ENSG00000246922	UBAP1L	turquoise	-0.4881812
ENSG00000137073	UBAP2	lightcyan	0.71539396
ENSG00000143569	UBAP2L	darkorange	0.54139119
ENSG00000154127	UBASH3B	grey	-0.14079
ENSG00000170315	UBB	green	-0.7280033
ENSG00000150991	UBC	brown	0.41839338
ENSG00000077721	UBE2A	grey	0.05662139
ENSG00000119048	UBE2B	grey	-0.0266802
ENSG00000258648	UBE2CP1	grey	0.37122558
ENSG00000072401	UBE2D1	grey	0.03670999
ENSG00000131508	UBE2D2	grey	-0.2946438
ENSG00000078967	UBE2D4	lightcyan	0.63300119
ENSG00000170142	UBE2E1	brown	-0.7580209
ENSG00000182247	UBE2E2	blue	0.54598436
ENSG00000170035	UBE2E3	red	0.66308128
ENSG00000184182	UBE2F	grey	0.13500782
ENSG00000261199	UBE2FP2	grey	0.40582626
ENSG00000233337	UBE2FP3	grey	0.33542642
ENSG00000132388	UBE2G1	grey	-0.0974625
ENSG00000184787	UBE2G2	grey	-0.1184878
ENSG00000186591	UBE2H	turquoise	0.66095831
ENSG00000103275	UBE2I	grey	-0.3441287
ENSG00000198833	UBE2J1	brown	-0.7002313
ENSG00000185651	UBE2L3	grey	0.05289255
ENSG00000156587	UBE2L6	turquoise	0.67959563
ENSG00000130725	UBE2M	grey	-0.0946401
ENSG00000177889	UBE2N	red	0.64441119

ENSG00000175931	UBE2O	blue	0.71530269
ENSG00000160714	UBE2Q1	turquoise	-0.4442003
ENSG00000140367	UBE2Q2	brown	-0.7286626
ENSG00000259511	UBE2Q2L	grey	0.17732088
ENSG00000189136	UBE2Q2P1	grey	0.06774218
ENSG00000237550	UBE2Q2P6	grey	0.03010256
ENSG00000107341	UBE2R2	magenta	0.46216739
ENSG00000108106	UBE2S	grey	0.03630425
ENSG00000077152	UBE2T	brown	0.76565365
ENSG00000244687	UBE2V1	grey	0.06996845
ENSG00000226632	UBE2V1P1	grey	0.01125068
ENSG00000104343	UBE2W	blue	-0.2702846
ENSG00000159202	UBE2Z	brown	0.42036148
ENSG00000114062	UBE3A	salmon	-0.5412882
ENSG00000151148	UBE3B	brown	0.30564627
ENSG00000009335	UBE3C	brown	0.6528429
ENSG00000118420	UBE3D	turquoise	-0.5115031
ENSG00000110344	UBE4A	grey	0.13105683
ENSG00000130939	UBE4B	red	-0.655867
ENSG00000103353	UBFD1	yellow	-0.6282994
ENSG00000120942	UBIAD1	grey	0.12403689
ENSG00000122042	UBL3	red	-0.4934474
ENSG00000102178	UBL4A	grey	0.05901803
ENSG00000198258	UBL5	brown	0.22478897
ENSG00000164332	UBLCP1	darkgrey	-0.6055079
ENSG00000118900	UBN1	turquoise	-0.3875598
ENSG00000157741	UBN2	greenyellow	0.41492829
ENSG00000185019	UBOX5	turquoise	-0.4378628
ENSG00000153560	UBP1	turquoise	-0.4088064
ENSG00000135018	UBQLN1	blue	0.52578784
ENSG00000188021	UBQLN2	blue	0.57654174
ENSG00000160803	UBQLN4	red	-0.4832193
ENSG00000144357	UBR3	turquoise	-0.6976534
ENSG00000127481	UBR4	green	-0.6891794
ENSG00000104517	UBR5	grey	0.17855469
ENSG00000012963	UBR7	salmon	0.57694014
ENSG00000165886	UBTD1	magenta	-0.437024
ENSG00000168246	UBTD2	grey	-0.0722856
ENSG00000108312	UBTF	grey	-0.1184639
ENSG00000162191	UBXN1	darkgrey	0.48237469
ENSG00000225986	UBXN10-AS1	grey	0.18282188
ENSG00000158062	UBXN11	blue	0.67402339
ENSG00000173960	UBXN2A	brown	-0.3989119
ENSG00000215114	UBXN2B	grey	-0.1933764
ENSG00000144224	UBXN4	brown	-0.8498363
ENSG00000167671	UBXN6	brown	0.76346322
ENSG00000163960	UBXN7	turquoise	0.48127214
ENSG00000104691	UBXN8	green	-0.4565289

ENSG00000154277	UCHL1	blue	0.89057121
ENSG00000118939	UCHL3	lightcyan	-0.5956572
ENSG00000116750	UCHL5	grey	0.06865847
ENSG00000130717	UCK1	grey	0.31917337
ENSG00000143179	UCK2	turquoise	-0.4400955
ENSG00000198276	UCKL1	grey	-0.0626997
ENSG00000163794	UCN	grey	0.08128438
ENSG00000175567	UCP2	cyan	0.57735832
ENSG00000175564	UCP3	grey	0.08336606
ENSG00000151116	UEVLD	turquoise	0.70359562
ENSG00000143222	UFC1	brown	0.62896871
ENSG00000070010	UFD1L	grey	0.0239741
ENSG00000014123	UFL1	blue	-0.5671583
ENSG00000233797	UFL1-AS1	grey	0.39425028
ENSG00000120686	UFM1	brown	-0.7209251
ENSG00000109775	UFSP2	grey	0.0574972
ENSG00000148154	UGCG	grey	-0.1206805
ENSG00000109814	UGDH	turquoise	0.51806222
ENSG00000102595	UGGT2	grey	0.06637678
ENSG00000169764	UGP2	yellow	-0.5426827
ENSG00000174607	UGT8	yellow	0.83481733
ENSG00000152332	UHMK1	red	0.55992979
ENSG00000065060	UHRF1BP1	grey	0.33142627
ENSG00000111647	UHRF1BP1L	blue	0.59686851
ENSG00000147854	UHRF2	grey	0.07615954
ENSG00000087206	UIMC1	blue	-0.3287479
ENSG00000083290	ULK2	grey	-0.1774632
ENSG00000140474	ULK3	black	0.51455564
ENSG00000168038	ULK4	grey	-0.0782607
ENSG00000114491	UMPS	grey	0.09022438
ENSG00000109103	UNC119	red	0.50281086
ENSG00000175970	UNC119B	turquoise	0.6851709
ENSG00000130477	UNC13A	blue	0.85715922
ENSG00000198722	UNC13B	lightcyan	-0.6783376
ENSG00000137766	UNC13C	grey	-0.0158065
ENSG00000140553	UNC45A	brown	0.59400154
ENSG00000115446	UNC50	red	0.50440549
ENSG00000113763	UNC5A	darkorange	0.539745
ENSG00000107731	UNC5B	yellow	0.76178562
ENSG00000182168	UNC5C	grey	0.0489705
ENSG00000156687	UNC5D	brown	0.53807347
ENSG00000133958	UNC79	turquoise	-0.6041937
ENSG00000144406	UNC80	blue	0.86804869
ENSG00000110057	UNC93B1	cyan	0.41253906
ENSG00000076248	UNG	turquoise	0.8239302
ENSG00000132478	UNK	grey	-0.1520883
ENSG00000059145	UNKL	blue	-0.3995598
ENSG00000240520	UOX	grey	0.07688511

ENSG00000005007	UPF1	grey	0.07134234
ENSG00000151461	UPF2	darkgrey	0.6141868
ENSG00000169062	UPF3A	grey	-0.3658842
ENSG00000234709	UPF3AP3	grey	0.22036488
ENSG00000125351	UPF3B	grey	-0.1039474
ENSG00000114638	UPK1B	turquoise	-0.4153258
ENSG00000110375	UPK2	yellow	-0.4101123
ENSG00000243566	UPK3B	grey	-0.0234833
ENSG00000183696	UPP1	grey60	0.35902982
ENSG00000007001	UPP2	blue	0.45326779
ENSG00000237327	UPP2-IT1	grey	0.22166464
ENSG00000094841	UPRT	grey	-0.0835153
ENSG00000101019	UQCC1	green	-0.7033074
ENSG00000137288	UQCC2	yellow	-0.6080405
ENSG00000184076	UQCR10	yellow	-0.576192
ENSG00000156467	UQCRB	brown	0.72132882
ENSG00000010256	UQCRC1	green	-0.6058261
ENSG00000140740	UQCRC2	green	-0.7943562
ENSG00000169021	UQCRFS1	brown	0.58265999
ENSG00000173660	UQCRH	brown	0.79540386
ENSG00000164405	UQCRQ	brown	0.79368701
ENSG00000142207	URB1	brown	0.56467864
ENSG00000135763	URB2	turquoise	0.44522856
ENSG00000106608	URGCP	brown	0.78115267
ENSG00000105176	URI1	green	0.82524725
ENSG00000167118	URM1	yellow	0.62446507
ENSG00000126088	UROD	brown	0.55232805
ENSG00000188690	UROS	blue	0.65192031
ENSG00000103005	USB1	grey	0.02273228
ENSG00000053501	USE1	magenta	0.40030375
ENSG00000158773	USF1	grey	0.05468508
ENSG00000105698	USF2	brown	-0.4995093
ENSG00000006611	USH1C	purple	-0.6249954
ENSG00000173915	USMG5	brown	0.71907066
ENSG00000231549	USMG5P1	grey	0.11745534
ENSG00000138768	USO1	grey	0.0827484
ENSG00000162607	USP1	turquoise	0.72872472
ENSG00000103194	USP10	green	-0.5513427
ENSG00000102226	USP11	blue	0.83609634
ENSG00000152484	USP12	lightgreen	0.77232957
ENSG00000232162	USP12-AS1	grey	0.36312587
ENSG00000230641	USP12-AS2	grey	0.25563705
ENSG00000058056	USP13	grey	0.09273092
ENSG00000101557	USP14	turquoise	-0.5880511
ENSG00000135655	USP15	turquoise	-0.4478635
ENSG00000156256	USP16	lightgreen	0.69711159
ENSG00000232399	USP17L13	turquoise	-0.5717784
ENSG00000223443	USP17L2	grey	0.25637208

ENSG00000226430	USP17L7	grey	0.16197179
ENSG00000184979	USP18	grey	0.05438992
ENSG00000172046	USP19	grey	0.02061692
ENSG00000036672	USP2	grey	-0.0073855
ENSG00000136878	USP20	blue	0.5351828
ENSG00000143258	USP21	grey	0.08015325
ENSG00000124422	USP22	yellow	-0.4173386
ENSG00000162402	USP24	red	0.48728833
ENSG00000155313	USP25	turquoise	-0.497482
ENSG00000242013	USP27X	blue	0.64601067
ENSG00000048028	USP28	grey	0.15086459
ENSG00000140455	USP3	blue	-0.6534645
ENSG00000259248	USP3-AS1	grey	0.3620783
ENSG00000135093	USP30	grey	0.01365834
ENSG00000103404	USP31	yellow	0.44121009
ENSG00000170832	USP32	red	-0.6527102
ENSG00000188933	USP32P1	grey	0.05112198
ENSG00000233327	USP32P2	grey	-0.0125228
ENSG00000189423	USP32P3	yellow	0.56339536
ENSG00000077254	USP33	grey	0.12252307
ENSG00000115464	USP34	darkorange	-0.5259834
ENSG00000118369	USP35	grey	0.13374818
ENSG00000055483	USP36	grey	0.22391843
ENSG00000135913	USP37	lightcyan	-0.5953001
ENSG00000170185	USP38	turquoise	0.41889986
ENSG00000168883	USP39	lightcyan	-0.6751843
ENSG00000114316	USP4	grey	0.1761436
ENSG00000085982	USP40	yellow	0.48982226
ENSG00000106346	USP42	turquoise	-0.5431173
ENSG00000123552	USP45	grey	0.11657707
ENSG00000109189	USP46	turquoise	0.74556834
ENSG00000248866	USP46-AS1	turquoise	0.35957334
ENSG00000170242	USP47	grey	-0.0915052
ENSG00000090686	USP48	lightcyan	-0.6184649
ENSG00000164663	USP49	grey	0.05298767
ENSG00000111667	USP5	blue	0.73069856
ENSG00000247746	USP51	grey	0.0406535
ENSG00000145390	USP53	turquoise	0.50667366
ENSG00000166348	USP54	yellow	0.92985098
ENSG00000129204	USP6	turquoise	0.30599299
ENSG00000148429	USP6NL	grey	0.13888132
ENSG00000187555	USP7	salmon	0.5811853
ENSG00000138592	USP8	grey	0.08004067
ENSG00000124486	USP9X	grey	0.31463096
ENSG00000114374	USP9Y	grey	-0.0867236
ENSG00000132952	USPL1	grey	0.34562562
ENSG00000111962	UST	blue	0.42287384
ENSG00000183520	UTP11L	grey	-0.1788063

ENSG00000156697	UTP14A	grey	0.03393412
ENSG00000253797	UTP14C	grey	0.29163834
ENSG00000164338	UTP15	grey	0.312384
ENSG00000011260	UTP18	grey	-0.0975039
ENSG00000147679	UTP23	grey	0.07406085
ENSG00000132467	UTP3	grey	0.10085742
ENSG00000108651	UTP6	lightcyan	-0.7030336
ENSG00000152818	UTRN	grey	-0.3115794
ENSG00000188958	UTS2B	grey	0.14018712
ENSG00000183878	UTY	grey	-0.0934717
ENSG00000198382	UVRAG	yellow	0.64390397
ENSG00000163945	UVSSA	blue	-0.3708442
ENSG00000115652	UXS1	turquoise	-0.3204042
ENSG00000126756	UXT	grey	-0.317715
ENSG00000103043	VAC14	blue	0.44496746
ENSG00000139190	VAMP1	grey	-0.2039376
ENSG00000220205	VAMP2	blue	0.37301026
ENSG00000049245	VAMP3	yellow	0.71715425
ENSG00000117533	VAMP4	grey	-0.080422
ENSG00000168899	VAMP5	pink	0.64997751
ENSG00000124333	VAMP7	lightgreen	0.75114277
ENSG00000118640	VAMP8	royalblue	0.74627864
ENSG00000173218	VANGL1	red	0.66207548
ENSG00000162738	VANGL2	turquoise	0.81578478
ENSG00000101558	VAPA	mediumpurp	0.58437681
ENSG00000124164	VAPB	brown	-0.2772512
ENSG00000204394	VARS	brown	0.73737237
ENSG00000137411	VARS2	grey	0.07660246
ENSG00000071246	VASH1	lightgreen	0.54756451
ENSG00000143494	VASH2	magenta	-0.5223047
ENSG00000168140	VASN	pink	0.7167944
ENSG00000125753	VASP	pink	0.62846288
ENSG00000108828	VAT1	grey	-0.2394708
ENSG00000171724	VAT1L	blue	0.70649254
ENSG00000252283	Vault	grey	0.2256537
ENSG00000252328	Vault	grey	0.20334687
ENSG00000141968	VAV1	cyan	0.65357507
ENSG00000160293	VAV2	grey	0.04424401
ENSG00000134215	VAV3	grey	0.13761261
ENSG00000155959	VBP1	turquoise	0.59509813
ENSG00000162692	VCAM1	grey	0.08677142
ENSG00000035403	VCL	grey	0.21070632
ENSG00000165280	VCP	brown	0.71089156
ENSG00000100483	VCPKMT	salmon	0.63800547
ENSG00000241847	VDAC1P8	grey	0.22331831
ENSG00000165637	VDAC2	green	-0.5772789
ENSG00000078668	VDAC3	brown	0.80164745
ENSG00000112715	VEGFA	pink	0.39532148

ENSG00000173511	VEGFB	turquoise	0.63141986
ENSG00000150630	VEGFC	grey	0.37887223
ENSG00000136451	VEZF1	yellow	0.70101358
ENSG00000028203	VEZT	grey	0.12640457
ENSG00000128564	VGF	blue	0.5325267
ENSG00000144560	VGLL4	blue	-0.5710502
ENSG00000136059	VILL	turquoise	0.30289045
ENSG00000026025	VIM	turquoise	0.52413652
ENSG00000131871	VIMP	grey	-0.038127
ENSG00000151445	VIPAS39	brown	0.49162659
ENSG00000114812	VIPR1	purple	0.76929477
ENSG00000232354	VIPR1-AS1	grey	-0.2157911
ENSG00000167397	VKORC1	grey	0.07888854
ENSG00000147852	VLDLR	red	-0.8302431
ENSG00000160131	VMA21	lightcyan	0.79351354
ENSG00000187650	VMAC	grey	0.22408774
ENSG00000233121	VN1R20P	grey	0.19797322
ENSG00000228038	VN1R51P	grey	0.08776436
ENSG00000268272	VN1R78P	grey	0.34096993
ENSG00000268357	VN1R81P	grey	0.18082978
ENSG00000268995	VN1R82P	turquoise	0.3977305
ENSG00000269345	VN1R85P	grey	0.36657304
ENSG00000154978	VOPP1	red	0.6636313
ENSG00000145041	VPRBP	turquoise	-0.3639539
ENSG00000160695	VPS11	grey	-0.0242353
ENSG00000197969	VPS13A	grey60	0.49627608
ENSG00000132549	VPS13B	grey	0.17022085
ENSG00000129003	VPS13C	grey	-0.268472
ENSG00000048707	VPS13D	darkred	0.60400136
ENSG00000215305	VPS16	brown	0.55426686
ENSG00000104142	VPS18	brown	0.69040158
ENSG00000131475	VPS25	darkred	0.64401047
ENSG00000122958	VPS26A	green	0.6619756
ENSG00000151502	VPS26B	brown	0.73422025
ENSG00000224895	VPS26BP1	blue	-0.3787884
ENSG00000160948	VPS28	red	0.57142471
ENSG00000111237	VPS29	green	-0.6020408
ENSG00000139719	VPS33A	salmon	0.59395587
ENSG00000184056	VPS33B	brown	0.7142887
ENSG00000069329	VPS35	blue	0.74992411
ENSG00000136100	VPS36	grey	0.04545614
ENSG00000155975	VPS37A	green	0.56018879
ENSG00000139722	VPS37B	grey	0.0479144
ENSG00000176428	VPS37D	yellow	-0.3411919
ENSG00000166887	VPS39	brown	0.60986477
ENSG00000136631	VPS45	darkred	0.75701771
ENSG00000132612	VPS4A	grey	0.02610017
ENSG00000119541	VPS4B	turquoise	0.53767342

ENSG00000149823	VPS51	brown	0.74218192
ENSG00000223501	VPS52	grey	0.09866396
ENSG00000141252	VPS53	blue	0.62831059
ENSG00000163159	VPS72	yellow	-0.5237719
ENSG00000156931	VPS8	grey	0.33987739
ENSG00000075399	VPS9D1	blue	0.49030333
ENSG00000261373	VPS9D1-AS1	grey	-0.0323369
ENSG00000100749	VRK1	lightgreen	0.73836498
ENSG00000028116	VRK2	yellow	0.82738007
ENSG00000105053	VRK3	lightgreen	-0.5805574
ENSG00000101842	VSIG1	grey	0.10570884
ENSG00000176834	VSIG10	brown	-0.4928502
ENSG00000186806	VSIG10L	yellow	-0.4449285
ENSG00000019102	VSIG2	black	0.79138817
ENSG00000155659	VSIG4	royalblue	0.82710567
ENSG00000163032	VSNL1	purple	0.70337103
ENSG00000132821	VSTM2L	blue	0.41887311
ENSG00000165633	VSTM4	turquoise	0.39095422
ENSG00000100987	VSX1	grey	0.12328095
ENSG00000009844	VTA1	blue	0.55596092
ENSG00000151532	VTI1A	blue	0.48552841
ENSG00000100568	VTI1B	grey	0.03114973
ENSG00000179403	VWA1	yellow	0.65438305
ENSG00000110002	VWA5A	brown	0.56617718
ENSG00000145198	VWA5B2	grey	0.10793522
ENSG00000204396	VWA7	blue	0.4049364
ENSG00000102763	VWA8	blue	0.43567178
ENSG00000250289	VWA8P1	grey	0.12510935
ENSG00000138614	VWA9	grey	0.08791321
ENSG00000167992	VWCE	blue	0.46227652
ENSG00000146530	VWDE	blue	0.64569419
ENSG00000110799	VWF	tan	0.86832694
ENSG00000095787	WAC	grey	0.11409907
ENSG00000062650	WAPAL	brown	-0.695201
ENSG00000116874	WARS2	grey	0.03448217
ENSG00000224238	WARS2-IT1	grey	0.2503737
ENSG00000015285	WAS	royalblue	0.66791414
ENSG00000112290	WASF1	blue	0.81188136
ENSG00000158195	WASF2	blue	-0.7720345
ENSG00000132970	WASF3	turquoise	0.75788799
ENSG00000146556	WASH2P	grey	-0.1920172
ENSG00000185596	WASH3P	grey	-0.2131729
ENSG00000225373	WASH5P	grey	0.08733168
ENSG00000182484	WASH6P	grey	-0.0984357
ENSG00000227232	WASH7P	grey	0.11766852
ENSG00000239779	WBP1	grey	-0.0791385
ENSG00000166272	WBP1L	blue	-0.4584346
ENSG00000132471	WBP2	darkgrey	-0.5577419

ENSG00000183066	WBP2NL	grey	0.19849925
ENSG00000120688	WBP4	yellow	0.47362049
ENSG00000185222	WBP5	turquoise	0.52008529
ENSG00000174374	WBSCR16	grey	0.29488033
ENSG00000185274	WBSCR17	blue	0.69424927
ENSG00000071462	WBSCR22	salmon	0.72250148
ENSG00000085449	WDFY1	grey	0.22487514
ENSG00000139668	WDFY2	yellow	0.54045241
ENSG00000163625	WDFY3	blue	-0.5749485
ENSG00000251260	WDFY3-AS1	grey	0.32849217
ENSG00000180769	WDFY3-AS2	turquoise	0.78334587
ENSG00000198554	WDHD1	red	-0.4226147
ENSG00000071127	WDR1	turquoise	0.56174744
ENSG00000120008	WDR11	grey	-0.19015
ENSG00000227165	WDR11-AS1	grey	0.02090849
ENSG00000138442	WDR12	salmon	0.56835385
ENSG00000101940	WDR13	turquoise	-0.6140059
ENSG00000150627	WDR17	grey	0.00029981
ENSG00000065268	WDR18	grey	0.14101912
ENSG00000140153	WDR20	yellow	0.51255008
ENSG00000127580	WDR24	brown	0.49638249
ENSG00000176473	WDR25	brown	0.69441765
ENSG00000162923	WDR26	turquoise	0.65689185
ENSG00000184465	WDR27	grey	-0.040531
ENSG00000065183	WDR3	brown	0.53151019
ENSG00000148225	WDR31	yellow	-0.4849974
ENSG00000136709	WDR33	grey	0.12039224
ENSG00000119333	WDR34	yellow	-0.3853348
ENSG00000118965	WDR35	turquoise	0.3940457
ENSG00000134987	WDR36	grey	-0.0867953
ENSG00000047056	WDR37	yellow	-0.286836
ENSG00000160193	WDR4	grey	0.03068806
ENSG00000164253	WDR41	black	0.67416573
ENSG00000163811	WDR43	lightgreen	0.53853647
ENSG00000196998	WDR45	green	-0.3443555
ENSG00000141580	WDR45B	turquoise	0.67450664
ENSG00000227057	WDR46	brown	0.76727481
ENSG00000085433	WDR47	turquoise	-0.6277938
ENSG00000196363	WDR5	grey	0.00439764
ENSG00000185798	WDR53	grey	0.0856217
ENSG00000005448	WDR54	brown	0.84437518
ENSG00000120314	WDR55	grey	0.26062089
ENSG00000103091	WDR59	grey	-0.0808418
ENSG00000196981	WDR5B	grey	0.00730468
ENSG00000178252	WDR6	grey	-0.1131945
ENSG00000126870	WDR60	darkgrey	0.50338643
ENSG00000140395	WDR61	salmon	0.75825642
ENSG00000243710	WDR65	blue	0.64821094

ENSG00000158023	WDR66	brown	0.64580536
ENSG00000091157	WDR7	green	-0.8158235
ENSG00000082068	WDR70	turquoise	-0.4961401
ENSG00000177082	WDR73	grey	0.11187331
ENSG00000133316	WDR74	brown	0.56556557
ENSG00000115368	WDR75	grey	-0.0693674
ENSG00000092470	WDR76	grey	0.27759195
ENSG00000116455	WDR77	grey	0.01623409
ENSG00000167716	WDR81	grey	0.26387933
ENSG00000164091	WDR82	brown	0.64056081
ENSG00000123154	WDR83	brown	0.3959121
ENSG00000105583	WDR83OS	brown	0.49215511
ENSG00000187260	WDR86	grey	0.16154115
ENSG00000243836	WDR86-AS1	grey	0.22180354
ENSG00000166359	WDR88	grey	0.36015966
ENSG00000140006	WDR89	green	0.66283928
ENSG00000105875	WDR91	grey	0.16396293
ENSG00000243667	WDR92	grey	-0.037292
ENSG00000196151	WDSUB1	grey	-0.1429153
ENSG00000142784	WDTC1	grey	0.1920291
ENSG00000156795	WDYHV1	green	-0.3813004
ENSG00000101443	WFDC2	black	0.82034006
ENSG00000109501	WFS1	turquoise	0.3966421
ENSG00000156232	WHAMM	grey	0.17316562
ENSG00000248334	WHAMMP2	grey	0.19892304
ENSG00000187667	WHAMMP3	magenta	-0.4332595
ENSG00000109685	WHSC1	black	-0.5208642
ENSG00000147548	WHSC1L1	grey	-0.0645743
ENSG00000215861	WI2-1896O14.1	grey	0.33298072
ENSG00000270301	WI2-2334D6.1	grey	0.17816357
ENSG00000232637	WI2-3658N16.1	grey	0.28275057
ENSG00000170473	WIBG	grey	-0.0564155
ENSG00000156076	WIF1	turquoise	0.60548655
ENSG00000115935	WIPF1	yellow	0.78930246
ENSG00000171475	WIPF2	turquoise	0.75717343
ENSG00000070540	WIPI1	purple	-0.55763
ENSG00000157954	WIPI2	salmon	0.6157692
ENSG00000011451	WIZ	grey	0.27114302
ENSG00000116729	WLS	turquoise	0.63576046
ENSG00000060237	WNK1	green	0.92884395
ENSG00000165238	WNK2	blue	0.73923477
ENSG00000196632	WNK3	grey	-0.0949786
ENSG00000125084	WNT1	grey	0.0616213
ENSG00000169884	WNT10B	grey	-0.3247066
ENSG00000111186	WNT5B	grey	0.2459078
ENSG00000115596	WNT6	grey60	0.53173529
ENSG00000154764	WNT7A	grey	-0.0980516
ENSG00000188064	WNT7B	turquoise	0.40834117

ENSG00000116213	WRAP73	grey	0.02354795
ENSG00000182093	WRB	grey	-0.0255409
ENSG00000165392	WRN	brown	-0.487833
ENSG00000124535	WRNIP1	greenyellow	0.38176123
ENSG00000109046	WSB1	darkred	-0.5885993
ENSG00000176871	WSB2	brown	-0.7144234
ENSG00000179314	WSCD1	grey	-0.0740725
ENSG00000075035	WSCD2	magenta	0.54779556
ENSG00000146457	WTAP	pink	0.54426901
ENSG00000113645	WWC1	turquoise	0.54610279
ENSG00000151718	WWC2	grey	-0.0241786
ENSG00000047644	WWC3	green	0.85093759
ENSG00000186153	WWOX	grey	0.24849213
ENSG00000123124	WWP1	red	-0.6619314
ENSG00000198373	WWP2	grey	0.03976945
ENSG00000018408	WWTR1	brown	-0.5675668
ENSG00000241985	WWTR1-IT1	grey	0.38176084
ENSG00000076924	XAB2	salmon	0.47342549
ENSG00000132530	XAF1	grey	0.1063
ENSG00000100219	XBP1	pink	0.5951133
ENSG00000249947	XBP1P1	grey	0.46041913
ENSG00000047597	XK	blue	0.60795363
ENSG00000206579	XKR4	blue	0.66631
ENSG00000171044	XKR6	grey	-0.0982175
ENSG00000260903	XKR7	grey	-0.0218019
ENSG00000158156	XKR8	grey	0.04214619
ENSG00000136936	XPA	grey	-0.0872147
ENSG00000154767	XPC	grey	-0.0701716
ENSG00000108039	XPNPEP1	salmon	0.67152051
ENSG00000196236	XPNPEP3	turquoise	0.55095894
ENSG00000132953	XPO4	turquoise	0.41103337
ENSG00000124571	XPO5	brown	0.74071693
ENSG00000169180	XPO6	brown	0.5604503
ENSG00000130227	XPO7	turquoise	-0.498814
ENSG00000184575	XPOT	turquoise	0.39048155
ENSG00000143324	XPR1	yellow	-0.7004556
ENSG00000073050	XRCC1	turquoise	-0.563177
ENSG00000126215	XRCC3	grey	0.12994793
ENSG00000152422	XRCC4	grey	-0.1338614
ENSG00000079246	XRCC5	grey	-0.0061288
ENSG00000196419	XRCC6	brown	0.77882885
ENSG00000166896	XRCC6BP1	grey	0.21424014
ENSG00000114127	XRN1	blue	-0.4146188
ENSG00000088930	XRN2	blue	-0.6351353
ENSG00000166435	XRRA1	grey	0.12979811
ENSG00000232694	XX-CR54.3	grey	0.06789091
ENSG00000161132	XXbac-B444P24.1	grey	0.22877389
ENSG00000235617	XXbac-B476C20.1	grey	0.25540275

ENSG00000234913	XXbac-B476C20.1	grey	0.2866532
ENSG00000235445	XXbac-B476C20.1	grey	0.26032294
ENSG00000215493	XXbac-B562F10.1	grey	0.10386679
ENSG00000181404	XXyac-YRM2039.:	grey	0.0109902
ENSG00000226445	XXyac-YX65C7_A.	grey	0.3309264
ENSG00000173950	XXYLT1	grey	-0.063613
ENSG00000093217	XYLB	purple	0.46319965
ENSG00000015532	XYLT2	grey	0.31194288
ENSG00000200298	Y_RNA	black	-0.479862
ENSG00000199200	Y_RNA	grey	0.47195843
ENSG00000199204	Y_RNA	grey	0.50873516
ENSG00000199273	Y_RNA	grey	0.34979613
ENSG00000199331	Y_RNA	grey	0.25737503
ENSG00000199357	Y_RNA	grey	0.45524857
ENSG00000199366	Y_RNA	grey	0.41822364
ENSG00000199459	Y_RNA	grey	0.42320581
ENSG00000199471	Y_RNA	grey	0.48881993
ENSG00000199476	Y_RNA	grey	0.32089576
ENSG00000199530	Y_RNA	grey	0.45860592
ENSG00000199550	Y_RNA	grey	0.46223849
ENSG00000199567	Y_RNA	grey	0.21872144
ENSG00000199630	Y_RNA	grey	0.46290012
ENSG00000199667	Y_RNA	grey	0.42189652
ENSG00000199711	Y_RNA	grey	0.57821295
ENSG00000199781	Y_RNA	grey	0.44211648
ENSG00000199890	Y_RNA	grey	0.21288066
ENSG00000199899	Y_RNA	grey	0.23581427
ENSG00000199933	Y_RNA	grey	0.3190085
ENSG00000199938	Y_RNA	grey	0.46596382
ENSG00000199980	Y_RNA	grey	0.47459136
ENSG00000200008	Y_RNA	grey	0.41542426
ENSG00000200090	Y_RNA	grey	0.53549703
ENSG00000200127	Y_RNA	grey	0.40869837
ENSG00000200141	Y_RNA	grey	0.4666613
ENSG00000200164	Y_RNA	grey	0.25438656
ENSG00000200170	Y_RNA	grey	0.42150676
ENSG00000200241	Y_RNA	grey	0.66525155
ENSG00000200309	Y_RNA	grey	0.46298022
ENSG00000200351	Y_RNA	grey	0.42057941
ENSG00000200361	Y_RNA	grey	0.16123464
ENSG00000200397	Y_RNA	grey	0.33978042
ENSG00000200419	Y_RNA	grey	0.46976871
ENSG00000200428	Y_RNA	grey	0.37301825
ENSG00000200502	Y_RNA	grey	0.49478083
ENSG00000200591	Y_RNA	grey	0.47510442
ENSG00000200610	Y_RNA	grey	0.36516523
ENSG00000200630	Y_RNA	grey	0.40070271
ENSG00000200688	Y_RNA	grey	0.43550834

ENSG00000200742	Y_RNA	grey	0.46940877
ENSG00000200769	Y_RNA	grey	0.49572639
ENSG00000200834	Y_RNA	grey	0.30043205
ENSG00000200842	Y_RNA	grey	0.44191837
ENSG00000200855	Y_RNA	grey	0.48226248
ENSG00000200953	Y_RNA	grey	0.33441504
ENSG00000201012	Y_RNA	grey	0.40762014
ENSG00000201013	Y_RNA	grey	0.40152268
ENSG00000201034	Y_RNA	grey	0.60109387
ENSG00000201047	Y_RNA	grey	0.42657748
ENSG00000201071	Y_RNA	grey	0.59266179
ENSG00000201121	Y_RNA	grey	0.49218203
ENSG00000201217	Y_RNA	grey	0.30487793
ENSG00000201228	Y_RNA	grey	0.3431339
ENSG00000201309	Y_RNA	grey	0.43851427
ENSG00000201343	Y_RNA	grey	0.36481302
ENSG00000201354	Y_RNA	grey	0.57874906
ENSG00000201371	Y_RNA	grey	0.43052401
ENSG00000201501	Y_RNA	grey	0.26160671
ENSG00000201509	Y_RNA	grey	0.54047734
ENSG00000201563	Y_RNA	grey	0.55248102
ENSG00000201566	Y_RNA	grey	0.43245365
ENSG00000201573	Y_RNA	grey	0.62530052
ENSG00000201678	Y_RNA	grey	0.20832833
ENSG00000201724	Y_RNA	grey	0.51956066
ENSG00000201820	Y_RNA	grey	0.48374815
ENSG00000201850	Y_RNA	grey	0.43136817
ENSG00000201892	Y_RNA	grey	0.30478768
ENSG00000201900	Y_RNA	grey	0.43391046
ENSG00000201916	Y_RNA	grey	0.50552684
ENSG00000202008	Y_RNA	grey	0.47416768
ENSG00000202041	Y_RNA	grey	0.32108663
ENSG00000202079	Y_RNA	grey	0.25886449
ENSG00000202141	Y_RNA	grey	0.4702455
ENSG00000202169	Y_RNA	grey	0.27447592
ENSG00000202190	Y_RNA	grey	0.57904233
ENSG00000202222	Y_RNA	grey	0.57103833
ENSG00000202297	Y_RNA	grey	0.53053943
ENSG00000202318	Y_RNA	grey	0.44452769
ENSG00000202382	Y_RNA	grey	0.28479927
ENSG00000202412	Y_RNA	grey	0.47201253
ENSG00000202417	Y_RNA	grey	0.45473582
ENSG00000202461	Y_RNA	grey	0.56140529
ENSG00000202470	Y_RNA	grey	0.50166288
ENSG00000202487	Y_RNA	grey	0.47325079
ENSG00000202514	Y_RNA	grey	0.35401445
ENSG00000202533	Y_RNA	grey	0.41660457
ENSG00000206582	Y_RNA	grey	0.40700952

ENSG00000206645	Y_RNA	grey	0.46410018
ENSG00000206668	Y_RNA	grey	0.45470247
ENSG00000206676	Y_RNA	grey	0.16683434
ENSG00000206682	Y_RNA	grey	0.52746414
ENSG00000206728	Y_RNA	grey	0.34181962
ENSG00000206738	Y_RNA	grey	0.28339166
ENSG00000206739	Y_RNA	grey	0.20879039
ENSG00000206751	Y_RNA	grey	0.33142665
ENSG00000206756	Y_RNA	grey	0.47109951
ENSG00000206768	Y_RNA	grey	0.52129105
ENSG00000206781	Y_RNA	grey	0.34995949
ENSG00000206795	Y_RNA	grey	0.39318642
ENSG00000206806	Y_RNA	grey	0.45461183
ENSG00000206847	Y_RNA	grey	0.38627127
ENSG00000207021	Y_RNA	grey	0.41082012
ENSG00000207032	Y_RNA	grey	0.60927723
ENSG00000207034	Y_RNA	grey	0.31914044
ENSG00000207036	Y_RNA	grey	0.46321658
ENSG00000207086	Y_RNA	grey	0.58143396
ENSG00000207117	Y_RNA	grey	0.42284473
ENSG00000207139	Y_RNA	grey	0.55941856
ENSG00000207146	Y_RNA	grey	0.39090707
ENSG00000207155	Y_RNA	grey	0.45574958
ENSG00000207212	Y_RNA	grey	0.53963332
ENSG00000207223	Y_RNA	grey	0.44453157
ENSG00000207258	Y_RNA	grey	0.42577921
ENSG00000207281	Y_RNA	grey	0.38506614
ENSG00000207326	Y_RNA	grey	0.37470287
ENSG00000207342	Y_RNA	grey	0.29076275
ENSG00000207368	Y_RNA	grey	0.42671043
ENSG00000207379	Y_RNA	grey	0.37570465
ENSG00000207383	Y_RNA	grey	0.48993111
ENSG00000207390	Y_RNA	grey	0.29069459
ENSG00000207401	Y_RNA	grey	0.22831627
ENSG00000207425	Y_RNA	grey	0.28939383
ENSG00000207438	Y_RNA	grey	0.18732033
ENSG00000207484	Y_RNA	grey	0.47408651
ENSG00000207497	Y_RNA	grey	0.47665485
ENSG00000207525	Y_RNA	grey	0.32654646
ENSG00000212205	Y_RNA	grey	0.34121356
ENSG00000212306	Y_RNA	grey	0.44173656
ENSG00000212418	Y_RNA	grey	0.50589432
ENSG00000212582	Y_RNA	grey	0.24569811
ENSG00000222395	Y_RNA	grey	0.37562318
ENSG00000222430	Y_RNA	grey	0.37933153
ENSG00000222503	Y_RNA	grey	0.32253681
ENSG00000222506	Y_RNA	grey	0.4182204
ENSG00000222511	Y_RNA	grey	0.38317957

ENSG00000223188	Y_RNA	grey	0.57630251
ENSG00000223249	Y_RNA	grey	0.34608481
ENSG00000238366	Y_RNA	grey	0.28651039
ENSG00000238516	Y_RNA	grey	0.43159712
ENSG00000238585	Y_RNA	grey	0.36021772
ENSG00000238713	Y_RNA	grey	0.55289756
ENSG00000238813	Y_RNA	grey	0.32754769
ENSG00000251867	Y_RNA	grey	0.00799016
ENSG00000252034	Y_RNA	grey	0.32909614
ENSG00000252085	Y_RNA	grey	0.12154921
ENSG00000252106	Y_RNA	grey	0.50996979
ENSG00000252210	Y_RNA	grey	0.42215731
ENSG00000252317	Y_RNA	grey	0.32987162
ENSG00000252420	Y_RNA	grey	0.45878778
ENSG00000252652	Y_RNA	grey	0.47261306
ENSG00000252742	Y_RNA	grey	0.32638337
ENSG00000252759	Y_RNA	grey	0.63205777
ENSG00000252822	Y_RNA	grey	0.4266753
ENSG00000252874	Y_RNA	grey	0.42202102
ENSG00000252965	Y_RNA	grey	0.3160065
ENSG00000252975	Y_RNA	grey	0.22650481
ENSG00000207370	Y_RNA	turquoise	0.53171108
ENSG00000239040	Y_RNA	yellow	0.60363934
ENSG00000241127	YAE1D1	grey	-0.0030837
ENSG00000015153	YAF2	red	0.64574566
ENSG00000137693	YAP1	turquoise	0.8023279
ENSG00000134684	YARS	grey60	0.84271405
ENSG00000139131	YARS2	green	-0.4269043
ENSG00000182362	YBEY	grey	0.04084776
ENSG00000065978	YBX1	blue	-0.7469215
ENSG00000240002	YBX1P3	grey	0.24367384
ENSG00000226048	YBX1P8	grey	0.33460574
ENSG00000060138	YBX3	pink	0.91472208
ENSG00000161179	YDJC	mediumpurp	-0.4833715
ENSG00000163872	YEATS2	turquoise	-0.6890183
ENSG00000233885	YEATS2-AS1	grey	0.27300363
ENSG00000127337	YEATS4	grey	-0.1769837
ENSG00000176105	YES1	brown	-0.8836505
ENSG00000174851	YIF1A	grey	0.04000416
ENSG00000167645	YIF1B	darkgrey	-0.6384234
ENSG00000058799	YIPF1	pink	0.5638467
ENSG00000130733	YIPF2	grey	0.27833464
ENSG00000119820	YIPF4	grey	-0.034285
ENSG00000145817	YIPF5	grey	0.1523106
ENSG00000181704	YIPF6	turquoise	0.81582876
ENSG00000250067	YJEFN3	magenta	0.65548921
ENSG00000106636	YKT6	turquoise	-0.3626905
ENSG00000119596	YLPM1	turquoise	-0.668461

ENSG00000136758	YME1L1	green	-0.6301241
ENSG00000180667	YOD1	grey	0.26507116
ENSG00000100027	YPEL1	yellow	0.47832821
ENSG00000175155	YPEL2	darkred	-0.6436315
ENSG00000090238	YPEL3	grey	-0.2360565
ENSG00000166793	YPEL4	turquoise	-0.51302
ENSG00000119801	YPEL5	grey	-0.1750466
ENSG00000083896	YTHDC1	lightgreen	0.71793991
ENSG00000149658	YTHDF1	pink	0.74278551
ENSG00000198492	YTHDF2	grey	-0.0755865
ENSG00000185728	YTHDF3	grey	0.08575747
ENSG00000270673	YTHDF3-AS1	grey	0.18121194
ENSG00000166913	YWHAB	brown	0.90561687
ENSG00000108953	YWHAE	grey	-0.2980763
ENSG00000170027	YWHAG	purple	0.66383586
ENSG00000128245	YWHAH	blue	0.78666817
ENSG00000134308	YWHAQ	grey	0.03815198
ENSG00000249986	YWHAQP6	grey	0.42602458
ENSG00000164924	YWHAZ	blue	0.47103941
ENSG00000100811	YY1	grey	-0.0583592
ENSG00000163374	YY1AP1	grey	-0.1280651
ENSG00000230661	YY1P1	grey	0.11276349
ENSG00000221392	Z73964.1	grey	0.33657723
ENSG00000197047	Z73979.1	grey	0.18792703
ENSG00000228061	Z83001.1	grey	0.28931323
ENSG00000229891	Z83851.1	grey	-0.1710083
ENSG00000264878	Z85986.1	grey	0.48971933
ENSG00000265106	Z93241.1	grey	0.46936117
ENSG00000221743	Z95152.1	grey	0.37307135
ENSG00000207643	Z95704.1	grey	0.35528939
ENSG00000269605	Z97053.1	grey	0.43160447
ENSG00000269048	Z98049.1	grey	0.00259261
ENSG00000196674	Z98881.1	grey	0.41850927
ENSG00000257053	Z98941.1	brown	-0.5194031
ENSG00000180011	ZADH2	brown	-0.662625
ENSG00000169064	ZBBX	blue	0.6368944
ENSG00000214717	ZBED1	turquoise	0.48943577
ENSG00000132846	ZBED3	yellow	0.65770731
ENSG00000250802	ZBED3-AS1	yellow	0.48185038
ENSG00000100426	ZBED4	brown	-0.5916516
ENSG00000236287	ZBED5	grey	-0.1325833
ENSG00000247271	ZBED5-AS1	grey	-0.0593768
ENSG00000257315	ZBED6	green	0.67958247
ENSG00000205189	ZBTB10	turquoise	0.59437966
ENSG00000066422	ZBTB11	turquoise	-0.5621157
ENSG00000198081	ZBTB14	brown	-0.7716786
ENSG00000109906	ZBTB16	pink	0.66081406
ENSG00000116809	ZBTB17	red	-0.56118

ENSG00000179456	ZBTB18	grey	0.07577058
ENSG00000181472	ZBTB2	grey	0.10585314
ENSG00000181722	ZBTB20	turquoise	0.63805447
ENSG00000241560	ZBTB20-AS1	grey	0.26065885
ENSG00000241295	ZBTB20-AS2	grey	0.43322212
ENSG00000239946	ZBTB20-AS3	grey	0.28342899
ENSG00000242767	ZBTB20-AS4	brown	-0.3579194
ENSG00000236104	ZBTB22	grey	-0.0138052
ENSG00000112365	ZBTB24	grey	-0.1991629
ENSG00000089775	ZBTB25	brown	-0.5471019
ENSG00000171448	ZBTB26	brown	-0.7030584
ENSG00000185670	ZBTB3	grey	0.39797588
ENSG00000177485	ZBTB33	turquoise	0.47367539
ENSG00000177125	ZBTB34	turquoise	0.80770343
ENSG00000185278	ZBTB37	brown	-0.6532484
ENSG00000177311	ZBTB38	blue	-0.339941
ENSG00000166860	ZBTB39	turquoise	0.66747848
ENSG00000174282	ZBTB4	darkgrey	0.59863092
ENSG00000237200	ZBTB40-IT1	grey	0.2124464
ENSG00000177888	ZBTB41	grey	0.28175991
ENSG00000169155	ZBTB43	turquoise	0.55236147
ENSG00000196323	ZBTB44	brown	-0.6623604
ENSG00000130584	ZBTB46	grey	0.07096406
ENSG00000114853	ZBTB47	green	0.72556453
ENSG00000204859	ZBTB48	grey	0.10467288
ENSG00000168826	ZBTB49	grey	0.03798212
ENSG00000168795	ZBTB5	blue	-0.6729556
ENSG00000186130	ZBTB6	turquoise	0.59520694
ENSG00000160685	ZBTB7B	pink	0.54954695
ENSG00000184828	ZBTB7C	grey	0.29002471
ENSG00000176261	ZBTB8OS	turquoise	-0.3977633
ENSG00000213588	ZBTB9	turquoise	0.57783962
ENSG00000104427	ZC2HC1A	yellow	-0.4710729
ENSG00000119703	ZC2HC1C	grey	0.02486543
ENSG00000135482	ZC3H10	grey	0.28858182
ENSG00000058673	ZC3H11A	grey	0.0405986
ENSG00000102053	ZC3H12B	turquoise	-0.6056981
ENSG00000149289	ZC3H12C	turquoise	0.45154998
ENSG00000123200	ZC3H13	darkgrey	0.81100298
ENSG00000100722	ZC3H14	grey	-0.1371863
ENSG00000065548	ZC3H15	turquoise	-0.4421771
ENSG00000158545	ZC3H18	grey	-0.0048254
ENSG00000014164	ZC3H3	grey	0.33870223
ENSG00000130749	ZC3H4	lightgreen	0.580416
ENSG00000188177	ZC3H6	darkgrey	0.57150727
ENSG00000122299	ZC3H7A	grey	0.05725878
ENSG00000100403	ZC3H7B	salmon	-0.5393173
ENSG00000144161	ZC3H8	grey	0.00296623

ENSG00000105939	ZC3HAV1	turquoise	0.63895669
ENSG00000146858	ZC3HAV1L	grey	0.11449918
ENSG00000091732	ZC3HC1	brown	0.47105063
ENSG00000126970	ZC4H2	blue	0.46411423
ENSG00000155329	ZCCHC10	grey	0.15651043
ENSG00000134744	ZCCHC11	grey	-0.0090027
ENSG00000174460	ZCCHC12	purple	0.84002934
ENSG00000140948	ZCCHC14	turquoise	0.39086667
ENSG00000121766	ZCCHC17	brown	0.39170877
ENSG00000165424	ZCCHC24	green	0.95317339
ENSG00000168228	ZCCHC4	grey	0.36379392
ENSG00000147905	ZCCHC7	lightgreen	0.504927
ENSG00000033030	ZCCHC8	grey	0.27442191
ENSG00000131732	ZCCHC9	grey	-0.0271471
ENSG00000139168	ZCRB1	bisque4	0.74873035
ENSG00000078487	ZCWPW1	turquoise	-0.4620054
ENSG00000206559	ZCWPW2	grey	0.2363522
ENSG00000159714	ZDHHC1	grey	0.14104398
ENSG00000188818	ZDHHC11	grey	-0.0825942
ENSG00000206077	ZDHHC11B	yellow	0.32117281
ENSG00000160446	ZDHHC12	grey	0.02746354
ENSG00000177054	ZDHHC13	grey	0.28708877
ENSG00000175048	ZDHHC14	grey	0.0045401
ENSG00000102383	ZDHHC15	blue	0.49002876
ENSG00000171307	ZDHHC16	grey	0.16944511
ENSG00000186908	ZDHHC17	grey	-0.1637097
ENSG00000204160	ZDHHC18	grey	0.03292567
ENSG00000104219	ZDHHC2	turquoise	0.79723027
ENSG00000180776	ZDHHC20	darkred	-0.7703846
ENSG00000236953	ZDHHC20-IT1	grey	0.26044563
ENSG00000255460	ZDHHC20P3	grey	0.03887131
ENSG00000175893	ZDHHC21	lightcyan	0.78088053
ENSG00000184307	ZDHHC23	brown	0.58113686
ENSG00000174165	ZDHHC24	grey	0.01016168
ENSG00000163812	ZDHHC3	pink	0.7153368
ENSG00000136247	ZDHHC4	grey	-0.0598492
ENSG00000156599	ZDHHC5	grey	0.32792094
ENSG00000153786	ZDHHC7	grey	0.19596705
ENSG00000099904	ZDHHC8	grey	0.02364967
ENSG00000133519	ZDHHC8P1	grey	-0.1340114
ENSG00000188706	ZDHHC9	yellow	0.81293589
ENSG00000148516	ZEB1	grey	-0.2613065
ENSG00000237036	ZEB1-AS1	turquoise	0.39368606
ENSG00000169554	ZEB2	green	0.94891836
ENSG00000160445	ZER1	darkorange	0.62779136
ENSG00000104231	ZFAND1	grey	-0.2008583
ENSG00000178381	ZFAND2A	brown	0.55550436
ENSG00000158552	ZFAND2B	brown	0.70733657

ENSG00000156639	ZFAND3	blue	-0.6452655
ENSG00000172671	ZFAND4	grey	0.35955005
ENSG00000107372	ZFAND5	grey	-0.0897741
ENSG00000086666	ZFAND6	grey	-0.2318853
ENSG00000177410	ZFAS1	grey	-0.4834947
ENSG00000066827	ZFAT	grey	0.33515204
ENSG00000133858	ZFC3H1	red	-0.6530903
ENSG00000136367	ZFHX2	grey	-0.1295239
ENSG00000140836	ZFHX3	magenta	-0.5033835
ENSG00000091656	ZFHX4	grey	-0.0154712
ENSG00000253661	ZFHX4-AS1	grey	0.20446013
ENSG00000184517	ZFP1	grey	0.01015914
ENSG00000198939	ZFP2	brown	-0.499475
ENSG00000196867	ZFP28	grey	-0.0589657
ENSG00000180787	ZFP3	turquoise	0.59389775
ENSG00000120784	ZFP30	turquoise	0.37200289
ENSG00000128016	ZFP36	pink	0.73731129
ENSG00000185650	ZFP36L1	turquoise	0.51327366
ENSG00000152518	ZFP36L2	turquoise	0.46125532
ENSG00000136866	ZFP37	grey	0.05030226
ENSG00000181638	ZFP41	grey	0.22350611
ENSG00000204644	ZFP57	yellow	0.70494369
ENSG00000196670	ZFP62	brown	-0.8297124
ENSG00000020256	ZFP64	grey	0.21534278
ENSG00000187815	ZFP69	magenta	0.43044042
ENSG00000187801	ZFP69B	magenta	0.56347255
ENSG00000181007	ZFP82	grey	0.33918134
ENSG00000184939	ZFP90	grey	0.04911187
ENSG00000186660	ZFP91	brown	-0.887586
ENSG00000162300	ZFPL1	grey	0.21043529
ENSG00000169946	ZFPM2	magenta	-0.5232639
ENSG00000056097	ZFR	grey	0.12105377
ENSG00000105278	ZFR2	purple	0.79639702
ENSG00000005889	ZFX	turquoise	0.52324546
ENSG00000067646	ZFY	grey	-0.0449973
ENSG00000165861	ZFYVE1	grey	0.29061147
ENSG00000039319	ZFYVE16	yellow	0.71480492
ENSG00000166140	ZFYVE19	grey	-0.1485748
ENSG00000100711	ZFYVE21	turquoise	0.50072309
ENSG00000072121	ZFYVE26	grey	0.36119333
ENSG00000155256	ZFYVE27	brown	0.67910997
ENSG00000159733	ZFYVE28	turquoise	-0.5145976
ENSG00000157077	ZFYVE9	grey	0.12879553
ENSG00000220201	ZGLP1	turquoise	-0.3476714
ENSG00000165156	ZHX1	turquoise	0.7130359
ENSG00000178764	ZHX2	brown	-0.7422252
ENSG00000174306	ZHX3	turquoise	0.51770552
ENSG00000152977	ZIC1	darkmagenta	-0.6073472

ENSG00000043355	ZIC2	grey	0.17775244
ENSG00000174963	ZIC4	darkmagenta	-0.5542609
ENSG00000171649	ZIK1	grey	0.0567604
ENSG00000269699	ZIM2	grey	0.18593963
ENSG00000106261	ZKSCAN1	yellow	0.56108314
ENSG00000189298	ZKSCAN3	grey	0.1494455
ENSG00000187626	ZKSCAN4	grey	0.15703163
ENSG00000196652	ZKSCAN5	brown	-0.5941398
ENSG00000196345	ZKSCAN7	grey	0.05574034
ENSG00000198315	ZKSCAN8	lightgreen	0.7728536
ENSG00000166432	ZMAT1	lightgreen	0.68172998
ENSG00000172667	ZMAT3	mediumpurp	0.7163673
ENSG00000165061	ZMAT4	blue	0.66880219
ENSG00000100319	ZMAT5	turquoise	0.47886687
ENSG00000108175	ZMIZ1	turquoise	0.66334259
ENSG00000224596	ZMIZ1-AS1	grey	-0.2433189
ENSG00000122515	ZMIZ2	darkred	-0.3729342
ENSG00000084073	ZMPSTE24	turquoise	0.80536975
ENSG00000197056	ZMYM1	turquoise	0.5680543
ENSG00000147130	ZMYM3	blue	0.67102114
ENSG00000146463	ZMYM4	grey	-0.0435438
ENSG00000227409	ZMYM4-AS1	cyan	-0.4527735
ENSG00000132950	ZMYM5	green	0.64529844
ENSG00000163867	ZMYM6	grey	-0.034995
ENSG00000015171	ZMYND11	bisque4	0.85901761
ENSG00000066185	ZMYND12	grey	0.02125707
ENSG00000165724	ZMYND19	grey	0.0408368
ENSG00000258917	ZMYND19P1	grey	0.25040739
ENSG00000101040	ZMYND8	turquoise	-0.4441083
ENSG00000256223	ZNF10	pink	-0.5501837
ENSG00000197020	ZNF100	grey	0.09220486
ENSG00000234473	ZNF101P2	grey	0.1473647
ENSG00000103994	ZNF106	grey	0.03687368
ENSG00000196247	ZNF107	turquoise	0.55072377
ENSG00000062370	ZNF112	brown	-0.7600677
ENSG00000152926	ZNF117	lightcyan	0.58462743
ENSG00000164631	ZNF12	brown	-0.6634709
ENSG00000197961	ZNF121	grey	0.2103475
ENSG00000196418	ZNF124	brown	-0.4134545
ENSG00000172262	ZNF131	brown	-0.3206909
ENSG00000131849	ZNF132	grey	-0.0529471
ENSG00000125846	ZNF133	brown	0.64754574
ENSG00000213762	ZNF134	turquoise	0.42079715
ENSG00000176293	ZNF135	brown	-0.5140613
ENSG00000123870	ZNF137P	grey	0.36554664
ENSG00000197008	ZNF138	salmon	-0.667234
ENSG00000105708	ZNF14	grey	0.05485129
ENSG00000131127	ZNF141	grey	-0.1169464

ENSG00000166478	ZNF143	brown	-0.4596209
ENSG00000167635	ZNF146	lightgreen	0.84347091
ENSG00000163848	ZNF148	grey	-0.1287502
ENSG00000204920	ZNF155	greenyellow	-0.4829755
ENSG00000147117	ZNF157	grey	0.2266802
ENSG00000170631	ZNF16	grey	0.14984235
ENSG00000170949	ZNF160	grey	0.20189851
ENSG00000197279	ZNF165	brown	0.43249449
ENSG00000175787	ZNF169	grey	0.08498786
ENSG00000186272	ZNF17	grey	0.0638492
ENSG00000103343	ZNF174	brown	0.49725237
ENSG00000105497	ZNF175	brown	-0.5058059
ENSG00000154957	ZNF18	grey	0.00108813
ENSG00000167384	ZNF180	grey	0.05670114
ENSG00000197841	ZNF181	blue	-0.5737463
ENSG00000147118	ZNF182	grey	0.23675389
ENSG00000096654	ZNF184	grey	-0.1418934
ENSG00000147394	ZNF185	grey	-0.13983
ENSG00000136870	ZNF189	yellow	0.62830013
ENSG00000157429	ZNF19	grey	0.21031324
ENSG00000226314	ZNF192P1	grey	0.12895259
ENSG00000005801	ZNF195	grey	-0.1540607
ENSG00000186448	ZNF197	brown	-0.6853894
ENSG00000233509	ZNF197-AS1	grey	0.47818984
ENSG00000163067	ZNF2	grey	-0.0659668
ENSG00000010539	ZNF200	turquoise	0.36784408
ENSG00000166261	ZNF202	grey	0.13813601
ENSG00000204789	ZNF204P	purple	0.79553627
ENSG00000122386	ZNF205	grey	0.17638421
ENSG00000010244	ZNF207	lightgreen	-0.6216859
ENSG00000160321	ZNF208	grey	-0.0209934
ENSG00000121417	ZNF211	grey	0.00071926
ENSG00000170260	ZNF212	grey	0.19574302
ENSG00000085644	ZNF213	grey	-0.0229059
ENSG00000149050	ZNF214	grey	0.00867523
ENSG00000171940	ZNF217	yellow	0.46636342
ENSG00000165804	ZNF219	grey	0.10622176
ENSG00000159905	ZNF221	grey	0.21996909
ENSG00000159885	ZNF222	grey	0.07860993
ENSG00000178386	ZNF223	grey	0.31318619
ENSG00000267680	ZNF224	grey	0.36756996
ENSG00000167380	ZNF226	grey	-0.1645912
ENSG00000131115	ZNF227	grey	0.16342587
ENSG00000167383	ZNF229	darkred	0.54558241
ENSG00000159882	ZNF230	lightcyan	-0.529327
ENSG00000167840	ZNF232	grey	0.02513128
ENSG00000159915	ZNF233	yellow	-0.4537918
ENSG00000263002	ZNF234	turquoise	0.35144358

ENSG00000159917	ZNF235	grey	0.12550492
ENSG00000130856	ZNF236	salmon	-0.5767015
ENSG00000196793	ZNF239	grey	0.05388686
ENSG00000172466	ZNF24	turquoise	0.75619291
ENSG00000198105	ZNF248	grey	0.16182183
ENSG00000175395	ZNF25	blue	0.68967387
ENSG00000196150	ZNF250	grey	0.25542761
ENSG00000198169	ZNF251	turquoise	-0.5015529
ENSG00000196922	ZNF252P	turquoise	0.5553557
ENSG00000256771	ZNF253	black	0.5579616
ENSG00000213096	ZNF254	turquoise	0.58297889
ENSG00000152454	ZNF256	grey	0.07059008
ENSG00000109917	ZNF259	turquoise	-0.594944
ENSG00000198393	ZNF26	grey	0.22846681
ENSG00000254004	ZNF260	turquoise	0.74617862
ENSG00000006194	ZNF263	grey	0.22242305
ENSG00000083844	ZNF264	green	0.59310246
ENSG00000174652	ZNF266	grey	0.1539573
ENSG00000185947	ZNF267	blue	-0.4462968
ENSG00000090612	ZNF268	turquoise	0.39113122
ENSG00000257267	ZNF271	grey	0.13599105
ENSG00000198039	ZNF273	grey	-0.118216
ENSG00000171606	ZNF274	grey	0.12640424
ENSG00000063587	ZNF275	turquoise	0.79928044
ENSG00000158805	ZNF276	grey	0.15533455
ENSG00000198839	ZNF277	grey	0.03168766
ENSG00000198538	ZNF28	turquoise	0.72849293
ENSG00000198477	ZNF280B	grey	-0.1384824
ENSG00000056277	ZNF280C	black	-0.4743662
ENSG00000137871	ZNF280D	grey	-0.0952475
ENSG00000170265	ZNF282	grey	0.03869758
ENSG00000167637	ZNF283	grey	0.33191631
ENSG00000186026	ZNF284	grey	0.34354551
ENSG00000267508	ZNF285	brown	0.56073366
ENSG00000187607	ZNF286A	grey	0.45970127
ENSG00000249459	ZNF286B	grey	0.26555083
ENSG00000141040	ZNF287	greenyellow	-0.4622529
ENSG00000188994	ZNF292	brown	-0.5381967
ENSG00000170684	ZNF296	grey	0.24382948
ENSG00000108452	ZNF29P	grey	0.52678734
ENSG00000166526	ZNF3	grey	0.0221238
ENSG00000168661	ZNF30	grey	-0.0231937
ENSG00000145908	ZNF300	lightgreen	0.49156986
ENSG00000131845	ZNF304	grey	0.0312043
ENSG00000130803	ZNF317	grey	0.22221109
ENSG00000171467	ZNF318	grey	-0.1690876
ENSG00000166188	ZNF319	grey	0.16866412
ENSG00000230565	ZNF32-AS2	grey	0.36991559

ENSG00000182986	ZNF320	grey	0.08803797
ENSG00000181315	ZNF322	grey	0.3299247
ENSG00000083812	ZNF324	grey	0.07499391
ENSG00000249471	ZNF324B	grey	0.02069459
ENSG00000162664	ZNF326	green	0.81280566
ENSG00000181894	ZNF329	grey	0.15354759
ENSG00000109445	ZNF330	grey	0.17199976
ENSG00000160961	ZNF333	blue	-0.5001236
ENSG00000198185	ZNF334	grey	-0.1484104
ENSG00000198026	ZNF335	turquoise	-0.4896651
ENSG00000130684	ZNF337	grey	0.28330106
ENSG00000225192	ZNF33BP1	grey	0.11438159
ENSG00000196378	ZNF34	brown	-0.5581276
ENSG00000131061	ZNF341	turquoise	-0.5724061
ENSG00000088876	ZNF343	grey	0.12679065
ENSG00000251247	ZNF345	grey	0.25418803
ENSG00000113761	ZNF346	grey	-0.1023996
ENSG00000251666	ZNF346-IT1	grey	0.27719044
ENSG00000197937	ZNF347	grey	0.26319996
ENSG00000169981	ZNF35	blue	-0.4652559
ENSG00000256683	ZNF350	grey	-0.032016
ENSG00000169131	ZNF354A	brown	-0.6235576
ENSG00000178338	ZNF354B	green	0.71970445
ENSG00000177932	ZNF354C	blue	-0.5850466
ENSG00000198816	ZNF358	grey	0.2783138
ENSG00000160094	ZNF362	salmon	-0.7281946
ENSG00000138311	ZNF365	turquoise	-0.60201
ENSG00000178175	ZNF366	tan	0.80854185
ENSG00000165244	ZNF367	grey60	-0.380285
ENSG00000075407	ZNF37A	brown	-0.7362736
ENSG00000234420	ZNF37BP	grey	0.15904585
ENSG00000161298	ZNF382	grey	-0.1173947
ENSG00000188283	ZNF383	grey	0.03427752
ENSG00000126746	ZNF384	grey	-0.0158126
ENSG00000161642	ZNF385A	lightcyan	0.69249355
ENSG00000144331	ZNF385B	black	0.72580287
ENSG00000151789	ZNF385D	grey	-0.1201068
ENSG00000124613	ZNF391	purple	0.61912217
ENSG00000160908	ZNF394	grey	0.48494083
ENSG00000186918	ZNF395	turquoise	0.53109769
ENSG00000186496	ZNF396	grey	0.19940157
ENSG00000186812	ZNF397	yellow	0.60173477
ENSG00000197024	ZNF398	grey	-0.1483731
ENSG00000176222	ZNF404	grey	0.35857203
ENSG00000215421	ZNF407	grey	0.27941547
ENSG00000175213	ZNF408	grey	0.25158754
ENSG00000147124	ZNF41	grey	0.01205773
ENSG00000119725	ZNF410	grey	0.44841741

ENSG00000133250	ZNF414	grey	-0.0416282
ENSG00000170954	ZNF415	grey	0.05568722
ENSG00000083817	ZNF416	grey	-0.0811153
ENSG00000173480	ZNF417	grey	0.44307186
ENSG00000196724	ZNF418	lightgreen	0.52341832
ENSG00000105136	ZNF419	grey	-0.026768
ENSG00000102935	ZNF423	red	0.83226986
ENSG00000204947	ZNF425	red	0.57226745
ENSG00000130818	ZNF426	blue	-0.3704995
ENSG00000131116	ZNF428	mediumpurp	0.60536489
ENSG00000197013	ZNF429	grey	0.19054198
ENSG00000198521	ZNF43	turquoise	0.44669353
ENSG00000118620	ZNF430	grey	-0.0202625
ENSG00000196705	ZNF431	grey	-0.1518789
ENSG00000256087	ZNF432	grey	0.27143899
ENSG00000197647	ZNF433	grey	0.14689424
ENSG00000125945	ZNF436	grey	0.10340975
ENSG00000183621	ZNF438	grey	-0.0014485
ENSG00000171291	ZNF439	grey	0.06112818
ENSG00000197857	ZNF44	grey	0.16534765
ENSG00000197044	ZNF441	grey	0.02732245
ENSG00000198342	ZNF442	grey	0.37690041
ENSG00000180855	ZNF443	grey	0.16275657
ENSG00000167685	ZNF444	brown	0.63567653
ENSG00000185219	ZNF445	brown	-0.5737415
ENSG00000083838	ZNF446	grey	-0.0863732
ENSG00000124459	ZNF45	grey	0.3359495
ENSG00000178187	ZNF454	grey	0.18235621
ENSG00000197714	ZNF460	green	0.54449308
ENSG00000197808	ZNF461	grey	0.13880052
ENSG00000148143	ZNF462	blue	0.43112727
ENSG00000181444	ZNF467	black	0.5133934
ENSG00000204604	ZNF468	grey	0.14652575
ENSG00000225614	ZNF469	grey	0.04162379
ENSG00000197016	ZNF470	grey	0.21126509
ENSG00000196263	ZNF471	yellow	0.6416869
ENSG00000180035	ZNF48	brown	0.36256118
ENSG00000198464	ZNF480	turquoise	0.67496416
ENSG00000127081	ZNF484	grey	0.05530762
ENSG00000198298	ZNF485	grey	-0.0705586
ENSG00000256229	ZNF486	grey	0.25531242
ENSG00000243660	ZNF487	grey	-0.0921448
ENSG00000165388	ZNF488	yellow	0.65210964
ENSG00000188033	ZNF490	grey	-0.0833704
ENSG00000177599	ZNF491	grey	0.16130558
ENSG00000162714	ZNF496	grey	0.15284057
ENSG00000174586	ZNF497	grey	0.05429829
ENSG00000103199	ZNF500	grey	0.0873967

ENSG00000186446	ZNF501	brown	-0.5130833
ENSG00000196653	ZNF502	turquoise	0.37696652
ENSG00000165655	ZNF503	grey	-0.0831569
ENSG00000237149	ZNF503-AS2	grey	-0.0484099
ENSG00000081665	ZNF506	grey	0.12717841
ENSG00000168813	ZNF507	grey	0.12954754
ENSG00000081386	ZNF510	grey	-0.1150707
ENSG00000198546	ZNF511	grey	-0.1120808
ENSG00000243943	ZNF512	grey	0.03912558
ENSG00000196700	ZNF512B	grey	0.04607644
ENSG00000163795	ZNF513	turquoise	0.4902088
ENSG00000144026	ZNF514	grey	0.14516847
ENSG00000101493	ZNF516	turquoise	0.78697486
ENSG00000178163	ZNF518B	turquoise	0.62043926
ENSG00000175322	ZNF519	grey	0.12427532
ENSG00000198795	ZNF521	red	0.6168467
ENSG00000171443	ZNF524	grey	-0.0995889
ENSG00000203326	ZNF525	brown	-0.4465513
ENSG00000167625	ZNF526	grey	0.22851647
ENSG00000189164	ZNF527	turquoise	0.39047587
ENSG00000167555	ZNF528	grey	0.06168327
ENSG00000186020	ZNF529	lightcyan	0.72372725
ENSG00000183647	ZNF530	grey	0.02633011
ENSG00000074657	ZNF532	green	0.4553372
ENSG00000198633	ZNF534	grey	0.25260719
ENSG00000198597	ZNF536	yellow	0.85057954
ENSG00000171817	ZNF540	blue	0.52708309
ENSG00000178229	ZNF543	grey	0.16677483
ENSG00000198131	ZNF544	yellow	-0.5178386
ENSG00000187187	ZNF546	grey	0.27634419
ENSG00000152433	ZNF547	grey	0.20061587
ENSG00000188785	ZNF548	brown	-0.6256275
ENSG00000121406	ZNF549	yellow	-0.4985916
ENSG00000251369	ZNF550	turquoise	0.32428101
ENSG00000204519	ZNF551	grey	0.17561916
ENSG00000178935	ZNF552	grey	0.35450634
ENSG00000172006	ZNF554	grey	-0.132407
ENSG00000186300	ZNF555	turquoise	0.43456409
ENSG00000130544	ZNF557	grey	0.27486716
ENSG00000167785	ZNF558	blue	-0.5388314
ENSG00000188321	ZNF559	grey	-0.1166566
ENSG00000171469	ZNF561	grey	0.24214346
ENSG00000171466	ZNF562	green	0.43340227
ENSG00000188868	ZNF563	grey	0.25764709
ENSG00000249709	ZNF564	grey	0.40722864
ENSG00000196357	ZNF565	green	0.46391131
ENSG00000186017	ZNF566	turquoise	0.3018919
ENSG00000189042	ZNF567	grey	0.17010267

ENSG00000198453	ZNF568	grey	0.08811877
ENSG00000196437	ZNF569	grey	-0.1008416
ENSG00000171970	ZNF57	grey	0.25137225
ENSG00000171827	ZNF570	grey	0.08592906
ENSG00000180479	ZNF571	grey	0.00305301
ENSG00000267470	ZNF571-AS1	red	-0.6490141
ENSG00000180938	ZNF572	turquoise	0.50568191
ENSG00000189144	ZNF573	grey	0.16614497
ENSG00000105732	ZNF574	grey	-0.0314341
ENSG00000176472	ZNF575	grey	-0.1527335
ENSG00000124444	ZNF576	grey	0.18355526
ENSG00000161551	ZNF577	grey	0.15746064
ENSG00000258405	ZNF578	grey	0.11434959
ENSG00000213015	ZNF580	grey	-0.2733764
ENSG00000171425	ZNF581	grey	0.09208886
ENSG00000018869	ZNF582	grey	0.11388478
ENSG00000267454	ZNF582-AS1	grey	0.04280607
ENSG00000198440	ZNF583	grey	0.17511253
ENSG00000171574	ZNF584	grey	0.01529826
ENSG00000196967	ZNF585A	grey	0.10763804
ENSG00000245680	ZNF585B	red	0.53179201
ENSG00000083828	ZNF586	grey	0.15163039
ENSG00000269343	ZNF587B	grey	0.03531581
ENSG00000164048	ZNF589	grey	0.06472126
ENSG00000166716	ZNF592	brown	-0.4172186
ENSG00000142684	ZNF593	grey	0.27940987
ENSG00000180626	ZNF594	grey	-0.0220382
ENSG00000197701	ZNF595	grey	0.15562731
ENSG00000172748	ZNF596	grey	0.23102188
ENSG00000167981	ZNF597	turquoise	0.48711304
ENSG00000167962	ZNF598	brown	0.33526185
ENSG00000153896	ZNF599	grey	0.14689245
ENSG00000196458	ZNF605	grey	0.07468117
ENSG00000166704	ZNF606	brown	-0.5256306
ENSG00000198182	ZNF607	grey	0.392569
ENSG00000168916	ZNF608	salmon	-0.534199
ENSG00000180357	ZNF609	green	0.75046191
ENSG00000167554	ZNF610	grey	0.03573746
ENSG00000213020	ZNF611	blue	0.37293725
ENSG00000176024	ZNF613	grey	0.20104265
ENSG00000142556	ZNF614	grey	0.1300591
ENSG00000197619	ZNF615	grey	0.15679685
ENSG00000204611	ZNF616	grey	0.13127104
ENSG00000157657	ZNF618	grey	-0.0100527
ENSG00000177873	ZNF619	green	0.51574223
ENSG00000177842	ZNF620	grey	0.10863082
ENSG00000172888	ZNF621	brown	-0.8840329
ENSG00000173545	ZNF622	grey	0.20657093

ENSG00000183309	ZNF623	brown	-0.7383599
ENSG00000198551	ZNF627	grey	0.16275629
ENSG00000102870	ZNF629	salmon	-0.6340771
ENSG00000221994	ZNF630	grey	-0.0006023
ENSG00000238068	ZNF630-AS1	grey	0.21674159
ENSG00000075292	ZNF638	lightgreen	0.79193477
ENSG00000121864	ZNF639	brown	-0.4956223
ENSG00000167528	ZNF641	turquoise	0.74706626
ENSG00000167395	ZNF646	grey	0.42775953
ENSG00000198093	ZNF649	grey	-0.1397664
ENSG00000198740	ZNF652	green	0.80870995
ENSG00000235278	ZNF652P1	grey	0.42363613
ENSG00000161914	ZNF653	grey	0.26024604
ENSG00000197343	ZNF655	grey	0.00772869
ENSG00000196409	ZNF658	grey	0.04101467
ENSG00000160229	ZNF66	grey	0.1998767
ENSG00000144792	ZNF660	grey	0.18175304
ENSG00000182983	ZNF662	turquoise	0.62517107
ENSG00000215452	ZNF663P	green	0.53357282
ENSG00000179195	ZNF664	brown	-0.8774762
ENSG00000197497	ZNF665	grey	0.08359904
ENSG00000198046	ZNF667	blue	0.63386465
ENSG00000166770	ZNF667-AS1	greenyellow	0.43940143
ENSG00000167394	ZNF668	grey	0.20109908
ENSG00000188295	ZNF669	grey	0.37652986
ENSG00000083814	ZNF671	grey	0.00018935
ENSG00000171161	ZNF672	grey	0.1735557
ENSG00000251192	ZNF674	grey	0.09493172
ENSG00000230844	ZNF674-AS1	grey	0.24023582
ENSG00000197372	ZNF675	grey	-0.0109918
ENSG00000196109	ZNF676	grey	0.33865848
ENSG00000197928	ZNF677	turquoise	0.48098072
ENSG00000181450	ZNF678	brown	-0.7664495
ENSG00000173041	ZNF680	turquoise	0.74231954
ENSG00000196172	ZNF681	lightcyan	0.63514964
ENSG00000197124	ZNF682	black	0.54167294
ENSG00000117010	ZNF684	grey	-0.1134722
ENSG00000143373	ZNF687	grey	0.39260532
ENSG00000229809	ZNF688	brown	0.53089848
ENSG00000156853	ZNF689	grey	-0.0379258
ENSG00000198429	ZNF69	salmon	-0.4958953
ENSG00000164011	ZNF691	grey	-0.0568749
ENSG00000185730	ZNF696	grey	0.12113541
ENSG00000143067	ZNF697	grey	-0.2440359
ENSG00000196110	ZNF699	grey	0.26034549
ENSG00000147789	ZNF7	grey	0.16461854
ENSG00000187792	ZNF70	brown	-0.743703
ENSG00000196757	ZNF700	blue	-0.4588133

ENSG00000167562	ZNF701	brown	-0.3777144
ENSG00000242779	ZNF702P	grey	0.02283075
ENSG00000183779	ZNF703	turquoise	0.80630245
ENSG00000164684	ZNF704	brown	-0.466331
ENSG00000120963	ZNF706	greenyellow	0.46423285
ENSG00000182141	ZNF708	yellow	0.60563407
ENSG00000197951	ZNF71	brown	-0.5673447
ENSG00000140548	ZNF710	brown	-0.5226309
ENSG00000178665	ZNF713	grey	-0.1396208
ENSG00000160352	ZNF714	grey	0.02833257
ENSG00000227124	ZNF717	grey	-0.0442489
ENSG00000250312	ZNF718	grey	0.26042896
ENSG00000197302	ZNF720	turquoise	0.54087576
ENSG00000182903	ZNF721	grey	-0.0627241
ENSG00000196081	ZNF724P	turquoise	0.49831829
ENSG00000269067	ZNF728	grey	0.03285369
ENSG00000186777	ZNF732	grey	-0.0311871
ENSG00000234444	ZNF736	turquoise	0.36057264
ENSG00000237440	ZNF737	grey	-0.051209
ENSG00000172687	ZNF738	grey	0.20756889
ENSG00000185252	ZNF74	grey	0.16310067
ENSG00000139651	ZNF740	grey	0.01200211
ENSG00000181220	ZNF746	turquoise	0.52522707
ENSG00000169955	ZNF747	grey	0.20371623
ENSG00000186230	ZNF749	grey	0.18106542
ENSG00000162086	ZNF75A	blue	0.63300527
ENSG00000186376	ZNF75D	grey	0.16206008
ENSG00000065029	ZNF76	turquoise	-0.452429
ENSG00000160336	ZNF761	grey	0.13158566
ENSG00000169951	ZNF764	grey	-0.0003565
ENSG00000196417	ZNF765	grey	0.24428625
ENSG00000196214	ZNF766	grey	0.27290803
ENSG00000133624	ZNF767	red	-0.5924014
ENSG00000169957	ZNF768	darkgrey	0.67599052
ENSG00000175691	ZNF77	grey	0.14455769
ENSG00000198146	ZNF770	turquoise	0.36405984
ENSG00000179965	ZNF771	brown	0.51014986
ENSG00000197128	ZNF772	turquoise	0.67226632
ENSG00000152439	ZNF773	grey	0.18269738
ENSG00000196391	ZNF774	black	0.59194685
ENSG00000196456	ZNF775	grey	-0.0020441
ENSG00000152443	ZNF776	grey	-0.0558336
ENSG00000196453	ZNF777	grey	0.05496072
ENSG00000170100	ZNF778	grey	0.15973586
ENSG00000197782	ZNF780A	grey	0.08564328
ENSG00000128000	ZNF780B	salmon	-0.5460983
ENSG00000196381	ZNF781	grey	0.03858876
ENSG00000204946	ZNF783	grey	0.21654875

ENSG00000179922	ZNF784	grey	0.14294425
ENSG00000197362	ZNF786	brown	0.44955956
ENSG00000142409	ZNF787	grey	0.28444816
ENSG00000198556	ZNF789	grey	-0.0141299
ENSG00000196152	ZNF79	grey	0.04752789
ENSG00000197863	ZNF790	turquoise	0.45338844
ENSG00000173875	ZNF791	grey	0.03733783
ENSG00000180884	ZNF792	grey	0.0160422
ENSG00000196466	ZNF799	grey	0.28289875
ENSG00000083842	ZNF8	turquoise	0.43965569
ENSG00000170396	ZNF804A	turquoise	-0.4202069
ENSG00000204524	ZNF805	blue	-0.5442454
ENSG00000198482	ZNF808	turquoise	0.02545808
ENSG00000197779	ZNF81	turquoise	0.58697045
ENSG00000198346	ZNF813	grey	0.2356647
ENSG00000204514	ZNF814	green	-0.4851034
ENSG00000235944	ZNF815P	grey	-0.1705823
ENSG00000269001	ZNF818P	grey	0.34787232
ENSG00000102984	ZNF821	brown	0.51351956
ENSG00000197933	ZNF823	grey	0.26552307
ENSG00000231205	ZNF826P	grey	-0.0475624
ENSG00000151612	ZNF827	grey	-0.0078563
ENSG00000167766	ZNF83	black	-0.5647697
ENSG00000198783	ZNF830	grey	0.05363079
ENSG00000124203	ZNF831	purple	0.65188577
ENSG00000127903	ZNF835	grey	0.09961708
ENSG00000196267	ZNF836	grey	0.01385641
ENSG00000022976	ZNF839	blue	0.40119617
ENSG00000217896	ZNF839P1	grey	0.18050499
ENSG00000197608	ZNF841	grey	0.14482699
ENSG00000176723	ZNF843	grey	0.29794442
ENSG00000223547	ZNF844	grey	0.05299255
ENSG00000213799	ZNF845	grey	0.20730318
ENSG00000196605	ZNF846	green	0.5985683
ENSG00000105750	ZNF85	grey	0.19993621
ENSG00000267041	ZNF850	greenyellow	-0.4409062
ENSG00000178917	ZNF852	grey	0.03896939
ENSG00000236609	ZNF853	brown	-0.6329209
ENSG00000106479	ZNF862	grey	-0.0408283
ENSG00000261221	ZNF865	grey	0.12033488
ENSG00000234284	ZNF879	grey	0.08520196
ENSG00000221923	ZNF880	grey	0.11680811
ENSG00000228623	ZNF883	grey	-0.0234962
ENSG00000214029	ZNF891	brown	-0.6300698
ENSG00000167232	ZNF91	grey	-0.1321667
ENSG00000146757	ZNF92	turquoise	0.45191063
ENSG00000184635	ZNF93	black	0.64493512
ENSG00000124201	ZNFX1	blue	-0.6910911

ENSG00000106400	ZNHIT1	brown	0.69825999
ENSG00000174276	ZNHIT2	grey	0.05649051
ENSG00000108278	ZNHIT3	yellow	-0.7647496
ENSG00000117174	ZNHIT6	darkgrey	0.71071153
ENSG00000066379	ZNRD1	brown	0.63747807
ENSG00000204623	ZNRD1-AS1	grey	0.087676
ENSG00000180233	ZNRF2	grey	0.03436921
ENSG00000225264	ZNRF2P2	green	0.49808486
ENSG00000183579	ZNRF3	turquoise	0.72869137
ENSG00000177993	ZNRF3-AS1	grey	0.34339835
ENSG00000235786	ZNRF3-IT1	grey	0.2225946
ENSG00000188372	ZP3	grey	-0.090308
ENSG00000019995	ZRANB1	turquoise	0.43714409
ENSG00000132485	ZRANB2	grey	-0.4126044
ENSG00000229956	ZRANB2-AS2	grey	-0.104755
ENSG00000121988	ZRANB3	grey	0.12664052
ENSG00000169249	ZRSR2	darkgrey	0.58532373
ENSG00000152467	ZSCAN1	grey	0.02102296
ENSG00000158691	ZSCAN12	grey	0.00091324
ENSG00000219891	ZSCAN12P1	grey	0.14600425
ENSG00000269293	ZSCAN16-AS1	grey	-0.1756248
ENSG00000176371	ZSCAN2	grey	-0.0799261
ENSG00000121903	ZSCAN20	greenyellow	-0.4394632
ENSG00000166529	ZSCAN21	brown	-0.6012886
ENSG00000182318	ZSCAN22	blue	-0.3578637
ENSG00000187987	ZSCAN23	grey	0.3590407
ENSG00000197037	ZSCAN25	grey	0.20910395
ENSG00000140265	ZSCAN29	green	0.74592786
ENSG00000186814	ZSCAN30	yellow	0.54133915
ENSG00000235109	ZSCAN31	grey	0.18683256
ENSG00000140987	ZSCAN32	green	0.70294349
ENSG00000131848	ZSCAN5A	blue	0.37252256
ENSG00000267908	ZSCAN5D	grey	0.07102511
ENSG00000137185	ZSCAN9	grey	0.34597734
ENSG00000168612	ZSWIM1	grey	0.16281368
ENSG00000132801	ZSWIM3	grey	0.07211705
ENSG00000132003	ZSWIM4	grey	0.06898025
ENSG00000162415	ZSWIM5	grey	-0.1625597
ENSG00000214941	ZSWIM7	grey	-0.1563357
ENSG00000214655	ZSWIM8	yellow	0.31404862
ENSG00000153975	ZUFSP	grey	0.14724087
ENSG00000086827	ZW10	grey	0.09210854
ENSG00000174442	ZWILCH	brown	0.5006109
ENSG00000198455	ZXDB	grey	-0.1688923
ENSG00000070476	ZXDC	grey	-0.1636247
ENSG00000162378	ZYG11B	yellow	-0.6886015
ENSG00000159840	ZYX	pink	0.56210149
ENSG00000074755	ZZEF1	grey	0.1217942

ENSG00000036549	ZZZ3	lightgreen	0.57058433
